# Characterization of the gut bacterial and viral microbiota in latent autoimmune diabetes in adults

**DOI:** 10.1038/s41598-024-58985-w

**Published:** 2024-04-09

**Authors:** Casper S. Poulsen, Dan Hesse, Gabriel R. Fernandes, Tue H. Hansen, Timo Kern, Allan Linneberg, Lore Van Espen, Torben Jørgensen, Trine Nielsen, Amra C. Alibegovic, Jelle Matthijnssens, Oluf Pedersen, Henrik Vestergaard, Torben Hansen, Mette K. Andersen

**Affiliations:** 1 The Novo Nordisk Foundation Center for Basic Metabolic Research, Faculty of Health and Medical Sciences, University of Copenhagen, Copenhagen, Denmark; 2 Steno Diabetes Center Copenhagen, Gentofte, Denmark; 3 Novo Nordisk A/S, Soeborg, Denmark; 4 Biosystems Informatics, Institute René Rachou-Fiocruz Minas, Belo Horizonte, Brazil; 5 Clinical Microbiomics A/S, Copenhagen, Denmark; 6 Center for Clinical Research and Prevention, Bispebjerg and Frederiksberg Hospital, The Capital Region, Copenhagen, Denmark; 7 Department of Clinical Medicine, Faculty of Health and Medical Sciences, University of Copenhagen, Copenhagen, Denmark; 8 Department of Microbiology, Immunology & Transplantation, Rega Institute, Laboratory of Clinical & Epidemiological Virology, KU Leuven, Leuven, Belgium; 9 Department of Public Health, Faculty of Health and Medical Sciences, University of Copenhagen, Copenhagen, Denmark; 10 Center for Clinical Metabolic Research, Department of Medicine, Gentofte University Hospital, Copenhagen, Denmark; 11 Department of Medicine, Bornholms Hospital, Rønne, Denmark

**Keywords:** Type 1 diabetes, Diabetes, Type 1 diabetes, Type 2 diabetes, Microbiology, Bacteria, Virology

## Abstract

Latent autoimmune diabetes in adults (LADA) is a heterogeneous disease characterized by autoantibodies against insulin producing pancreatic beta cells and initial lack of need for insulin treatment. The aim of the present study was to investigate if individuals with LADA have an altered gut microbiota relative to non-diabetic control subjects, individuals with type 1 diabetes (T1D), and individuals with type 2 diabetes (T2D). Bacterial community profiling was performed with primers targeting the variable region 4 of the 16S rRNA gene and sequenced. Amplicon sequence variants (ASVs) were generated with DADA2 and annotated to the SILVA database. The gut virome was sequenced, using a viral particle enrichment and metagenomics approach, assembled, and quantified to describe the composition of the viral community. Comparison of the bacterial alpha- and beta-diversity measures revealed that the gut bacteriome of individuals with LADA resembled that of individuals with T2D. Yet, specific genera were found to differ in abundance in individuals with LADA compared with T1D and T2D, indicating that LADA has unique taxonomical features. The virome composition reflected the stability of the most dominant order *Caudovirales* and the families *Siphoviridae*, *Podoviridae*, and *Inoviridae*, and the dominant family *Microviridae*. Further studies are needed to confirm these findings.

## Introduction

Diabetes is a heterogeneous disease, characterized by elevated levels of blood glucose. Diabetes might be viewed as a spectrum, ranging from severely insulin deficient type 1 diabetes (T1D), with onset primarily in early childhood, to insulin resistant age-related type 2 diabetes (T2D), with onset primarily in middle-aged individuals and in late adulthood. The spectrum also encompasses latent autoimmune diabetes in adults (LADA), which is rather common, accounting for up to 10% of individuals initially diagnosed with T2D^1^. Phenotypically, LADA is placed between T1D and T2D, with respect to age-at diagnosis, measures of adiposity, circulating lipid levels, blood pressure, and progression to insulin dependence^1–6^. Also genetically, LADA seems to be an intermediate between T1D and T2D^5,7–11^. However, the contribution from T1D -associated genomic variants in the predisposition to LADA seems to be stronger than the contribution from T2D-associated variants, likely in part due to a considerable difference in effect sizes associated with T1D- and T2D-associated variants, respectively. The overlaps between diabetic subtypes can make the classification of adult diabetes challenging. Hence, improved understanding of the pathophysiology of LADA, and identification of biomarkers that could help distinguish LADA from T1D and T2D could be of clinical value. One strategy to shed light on these issues might be characterization of the human gut microbiome.

The human gut microbiome composition is shaped by multiple factors including host genetics, age, diet, environment, lifestyle, and external factors such as the use of medication, particularly antibiotics and metformin^12,13^. During the last two decades, the role of the gut microbiota in health and disease, particularly the bacteriome, has gained increasing attention. The gut microbiome has multiple functions for host metabolism, immunity, and brain biology^14–16^. Hence, growing evidence, from both animal and human studies, suggests that compositional, taxonomical, and functional changes in the gut bacteriome are linked to both T1D and T2D^17–19^. Gut-bacteriome abnormalities have been shown to affect the immune system and the gut permeability, suggested to trigger the autoimmune reaction leading to T1D^20,21^, and to disrupt the energy-generation and expenditure balance, which influence the risk of T2D^22^. Fang et al. ^23^ compared the gut bacterial microbiome between individuals with LADA, T1D, and T2D. This comparison indicated that these diabetic subtypes might differ in gut bacterial composition. Furthermore, Hu et al. ^24^ used metagenomics to compare the gut microbiome between individuals with adult-onset T1D and T2D including DNA viruses in the analyses and also found signatures differentiating the two groups. The intestinal virome plays an essential role in modulating the gut bacteriome since its core is composed of bacteriophages. The balance between bacteria and virus populations is critical for a healthy state of the overall gut microbiome^25^. Finding associations between the viral community and diseases is, however, a challenge due to the high variability across individuals. Yet, recent studies have shed some light on the role of the virome both in T1D and T2D^26,27^.

The aim of this study was to explore the intestinal bacterial and viral microbiome of individuals with LADA and compare findings with microbiome features of individuals with T1D and T2D as well as with non-diabetic control subjects.

## Results

The individuals with LADA were intermediary between T1D and T2D with respect to adiposity-related traits and measures of glucose homeostasis (Table 1).

**Table 1:** Overview of study population and quality of data parameters.

	Controls	Type 1 diabetes	LADA	Type 2 diabetes
Sex				
Male	44	17	37	44
Female	26	13	23	26
Age (year)	61 ± 10	52 ± 7	64 ± 10	61 ± 10
BMI (kg/m^2^)	27 ± 4	27 ± 5	29 ± 5	31 ± 6
Metformin treatment				
Yes	0	0	48	47
No	70	30	12	23
Waist circumference (cm)	93 ± 11	98 ± 13	103 ± 14	107 ± 14
Hip circumference (cm)	101 ± 8	106 ± 8	107 ± 10	108 ± 10
Waist/hip ratio	0.92 ± 0.08	0.92 ± 0.08	0.96 ± 0.09	1.00 ± 0.08
HbA1c (mmol/l)	38.5 ± 3.9	73.3 ± 8.8	63.1 ± 13.1	51.6 ± 13.9
Fasting plasma glucose (mmol/l)	5.8 ± 0.5	9.7 ± 4.2	8.8 ± 3.0	8.5 ± 2.7
Bacteriome sequencing depth (Mill. annotated Reads)	0.136 ± 0.026	0.150 ± 0.028	0.128 ± 0.024	0.129 ± 0.023
Viral sequencing depth (Mill. mapped Reads)	4.295 ± 2.747	3.932 ± 2.831	5.936 ± 2.718	5.803 ± 3.187
Cell count (Bill. cells/g)	20.4 ± 6.8	18.7 ± 5.7	18.9 ± 5.5	19.6 ± 6.1

Numerical values except counts are provided as means ± st. dev.

### Bacterial alpha and beta diversity

**Figure 1:**
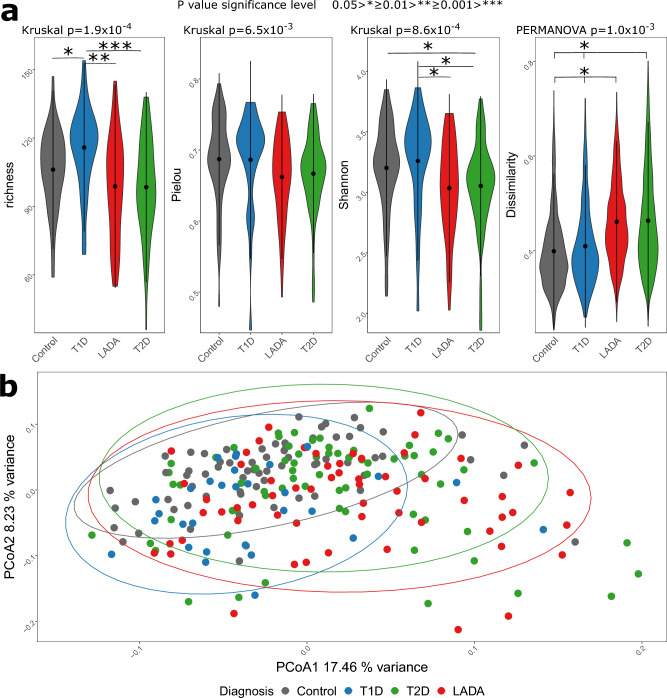
Exploration of differences between diagnostic groups investigating alpha and beta-diversity parameters (**a**), and by PCoA (**b**). Alpha-diversity was visualized with violin plots and included richness, Pielou’s evenness, and Shannon diversity. A non-parametric overall comparison of diagnostic groups was performed using the Kruskal–Wallis test. If significant, a follow-up pairwise comparison of groups was performed with the Mann–Whitney test and p-values were Bonferroni corrected. Bray–Curtis dissimilarity calculated from Hellinger transformed total sum scaled data was used as beta-diversity measure and the violin plot represented the within group variation. PERMANOVA was used to compare all diagnostic groups and follow-up pairwise comparison of diagnostic groups where p-values were Bonferroni corrected. PCoA were used to visualize dissimilarities of all samples. Variance explained by the first two axes were included in their labels. Violin plots of beta-diversity represented within diagnostic group dissimilarity.

In analyses of the bacterial gut microbiota alpha-diversity and beta-diversity metrics were compared between the diagnostic groups to investigate if individuals with LADA had a distinct compositional profile. For all alpha-diversity measures, richness (p = 1.9 × 10^−4^), Pielous evenness (p = 6.5 × 10^−3^), and Shannon diversity (p = 8.6 × 10^−4^), a significant difference was detected across groups using the Kruskal–Wallis test. Individuals with LADA resembled individuals with T2D across all alpha-diversity measures, and the difference was most pronounced between T1D and the remaining groups, mostly driven by differences in richness (Fig. 1a). Comparison of beta-diversity revealed a significant difference in bacterial composition between diagnostic groups, based on differences in centroid position (PERMANOVA p = 1.0 × 10^−3^) and dispersion (betadisper p = 4.5 × 10^−4^) (Fig. 1a). A visual evaluation based on the beta-diversity in the PCoA plot revealed that individuals with T2D and LADA clustered, and differed the most compared with T1D (Fig. 1b). This observation was also evident from the pairwise PERMANOVA, where T2D and LADA were the only groups that did not differ significantly from each other (Fig. 1a). To control for the possible confounding of metformin treatment, we ran additional analyses removing metformin treated individuals with LADA (n = 48) and T2D (n = 47). In these analyses, with a much reduced sample size, individuals with LADA and T2D resembled controls both with respect to alpha-diversity and beta-diversity (Supplementary Fig. 1a,b).

### Bacterial abundance

**Figure 2:**
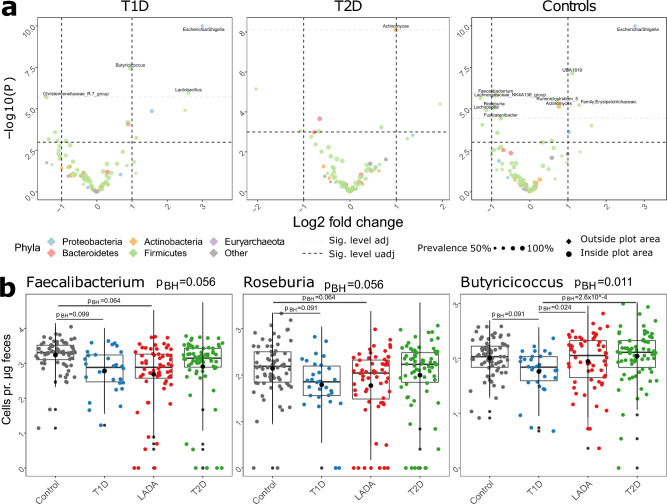
Differences in specific genera observed between diagnostic groups, Volcano plot showing results from the differential abundance analysis (**a**), and boxplots of selected genera (**b**). Differential abundance tests were performed with cell counts as normalization factors in DESeq2 performing both likelihood ratio test, comparing all diagnostic groups, and a Wald test to make pairwise comparisons between diagnostic groups. p-values in the Wald test were Benjamini–Hochberg corrected.

**Figure 3:**
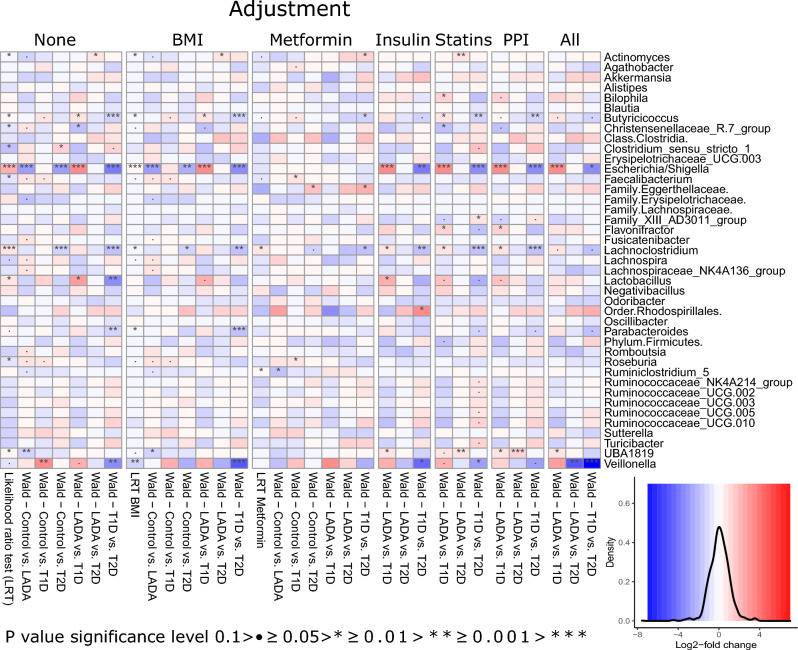
Heatmap of DESeq test results comparing across all groups using the likelihood ratio test implementation (LRT) and comparing diagnostic groups pairwise using Wald test. Analysis included adjustment for BMI, use of anti-diabetic medication (insulin, statins, PPI), and all of the mentioned covariates. Adjustment for metformin was analogous to the overall analysis scheme by removing samples from individuals treated with metformin. The first specified diagnostic group is the reference for the DESeq test in the Wald test. A density plot of the effect sizes (log2fold change) is included with the same color key used in the heatmap. Genera with the lowest p-value in the LRT without adjustment were included, but Log2fold changes and p-values from all genera comparison are provided in Supplementary Table 1. The heatmap was generated with the R package pheatmap with layout adjustments performed in inkscape.

The abundance of a number of bacterial genera was significantly different when comparing individuals with LADA with the three remaining diagnostic groups (Fig. 2a). The absolute abundance of one genus differed significantly between individuals with LADA and T2D (*Actinomyces*: p_BH_ = 0.025). Ten genera differed significantly between individuals with LADA and the control group, where *Escherichia* was the most significant (*Escherichia*/*Shigella*: p_BH_ = 1.6 × 10^−5^). Four genera differed when comparing individuals with LADA and T1D (*Escherichia*/*Shigella*: p_BH_ = 3.2 × 10^−6^, *Butyricicoccus*: p_BH_ = 0.024, *Lactobacillus*: p_BH_ = 0.068, *Christensenellaceae*_R.7_group: p_BH_ = 0.068) (Fig. 2a,b, Supplementary Fig. 2). Hence, supporting the observation of individulas with LADA being more similar to T2D than T1D. When analyzing short-chain fatty acid producers, including *Faecalibacterium*, *Clostridium*, *Roseburia*, *Anaerostipes*, *Bifidobacterium*, *Butyricicoccus*, and *Akkermansia,* we found that two of these genera were less abundant in individuals with LADA compared with non-diabetic control subjects (*Roseburia*: p_BH_ = 0.064, *Faecalibacterium*: p_BH_ = 0.064) (Supplementary File 1). *Faecalibacterium* was the most abundant genus in control subjects, but not in any of the diabetes groups (Fig. 2b, Supplementary Fig. 3). With respect to other butyrate producers, the abundance of *Butyricicoccus* was lower in individuals with T1D relative to both LADA (p_BH_ = 0.024) and T2D (p_BH_ = 2.6 × 10^−4^). All of the above-mentioned genera were significantly different in the likelihood ratio test (Fig. 3, Supplementary Table 1).

When controlling for metformin treatment, by removing individuals with LADA and T2D treated with metformin, the number of significantly differentially abundant genera was limited to Ruminiclostridium_5 (p_BH_ = 0.018) when comparing individuals with LADA with the control group (Supplementary Fig. 2a, Supplementary Table 1). Comparing the differential abundance of genera after correcting for BMI and anti-diabetic medication (insulin, statins, and proton pump inhibitors (PPI)), revealed in general consistent signatures. Of note, removing individuals treated with metformin, the differences observed for *Escherichia/Shigella* and UBA1819 disappeared (Fig. 3, Supplementary Table 1).

### Prediction of bacterial function

The functional prediction of the bacterial gut microbiota revealed similar patterns in PCoA analyses across groups. However, relatively large differences were observed for the evenness estimates between the diagnostic groups resulting in a higher Shannon diversity in individuals with T2D and LADA relative to control subjects and individuals with T1D. In general, a higher dissimilarity within individual groups of individuals with diabetes was observed relative to control subjects (Supplementary Fig. 4a,b, Supplementary File 2).

Compared with control subjects, 12 KEGG orthologs were significantly more abundant in individuals with LADA. Ten of these orthologs were also significantly more abundant in individuals with LADA compared with T1D. These more abundant orthologs were mainly involved in xenobiotics biodegradation and metabolism and lipid metabolism. Other highlighted pathways included two involved in metabolism of terpenoids, where the putative abundance of the function geraniol degradation was both significantly higher in individuals with LADA and T2D relative to T1D and control subjects. Individuals with LADA and T2D also had higher abundance of carotenoid biosynthesis when compared with control subjects. Furthermore, abundance of pathways involved in retinol metabolism was significantly higher in individuals with LADA relative to control subjects. No KEGG orthologs differed significantly between individuals with LADA and T2D (Supplementary Fig. 5a,b, Supplementary File 2).

### Viral abundance

**Figure 4:**
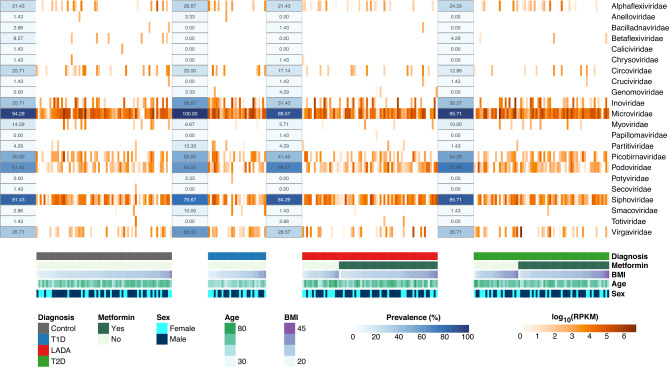
Heatmap of virome abundance and prevalence. The abundance of 22 taxonomic families is represented in log10(RPKM) and grouped by diagnosis. The prevalence represents the percentage of individuals in one group where the virus could be identified. The sex and usage of metformin are observed as qualitative information, while BMI (kg/m^2^) and age (years) are pictured as a quantitative colormap. The heatmap was generated with the R package pheatmap with layout adjustments performed in illustrator.

Our analyses of the viral gut microbiota showed that the most represented families were *Microviridae* (455 sequences), *Siphoviridae* (325), *Picobirnaviridae* (263), *Podoviridae* (199), and *Inoviridae* (41). The most represented families were also the most prevalent in the different diagnostic groups (Fig. 4). There was no association between the presence of a viral family and the diagnostic group of study participants (Supplementary Table 2). Considering the mean abundance of each viral family, the most abundant were *Microviridae* (101748 RPKM), *Inoviridae* (21822 RPKM), *Virgaviridae* (13683 RPKM), *Siphoviridae* (10598 RPKM), and *Podoviridae* (3715 RPKM). Comparing the quantification ranking between different groups (Wilcoxon rank test), we found no difference between diagnostic groups (Supplementary Table 3). The differential abundance analysis revealed *Podoviridae* as more abundant in individuals with T2D when compared with control subjects (*Podoviridae*: p_BH_ = 0.023) (Supplementary Table 4). However, considering outliers with extremely high counts, the difference observed might be false positive (Supplementary Fig. 6).

## Discussion

We examined the composition, taxonomy, and predicted functional profile of the bacterial gut microbiota, using 16S rRNA gene amplicon sequencing, and the viral gut microbiota, using metagenomics, in individuals with LADA compared with non-diabetic control subjects, and individuals with T1D or T2D. Overall, individuals with LADA were characterized by distinct features in their bacterial gut microbiota resembling those, which have been reported previously^23^. However, our results also indicated that the bacterial gut microbiota of individuals with LADA was most similar to individuals with T2D, which is in contrast to a previous study showing that the microbiota of individuals with LADA was more similar to individuals with T1D^23^.

By performing taxonomic and functional analysis of alpha and beta-diversity measures, we showed differences across diagnostic groups, but also revealed that individuals with LADA were most similar to individuals with T2D. Based on bacterial beta-diversity Hu et al. ^24^, using metagenomics, similarly found adult-onset T1D to be more similar, but still significantly different compared to T2D, relative to healthy cotrols. With respect to the abundance of bacterial genera, we found that *Actinomyces* was significantly more abundant in LADA compared with T2D and represented the only genus that was significantly different in the comparison of LADA and T2D. This signature was also observed after correcting for BMI, and anti-diabetic medication. Higher abundance of *Actinomyces* in feces has been associated with gestational diabetes and inflammation of the gut^28,29^. The link to inflammation provides a speculative explanation of the similarity of the abundance of *Actinomyces* in LADA and T1D. Despite previous observations that an increase in the abundance of *Actinomyces* is a marker of obesity and altered bacterial gut microbiota^28,30^, we did not observe a difference between individuals with T2D and control subjects.

On the bacterial genera level, the abundance of key short-chain fatty acid producers, including *Faecalibacterium* and *Roseburia*, have been reported to be relatively depleted in T1D and T2D ^19,24,31,32^. Here, we found that both genera were less abundant in individuals with LADA compared with the control group, which is in line with previous observations^23^. However, in contrast to previous observations we observed similar abundance of these short-chain fatty acid producers in LADA and T1D. This was also observed after correcting for BMI or removing individuals treated with metformin. These findings could indicate that lower abundance of these genera is a general change of the gut bacterial microbiota characterizing individuals with diabetes. Moreover, another key butyrate producer, *Clostridium*, was also observed to have lower abundance among individuals with T2D compared with control subjects, which is in line with previous observations in individuals with pre-diabetes and T2D^33,34^. The abundance of *Clostridium* did not differ between individuals with LADA and control subjects. Comparing individuals with LADA with non-diabetic control subjects, we observed a previously described metformin signature^12^, as *Escherichia/Shigella* and UBA1819 were significantly more abundant among individuals with LADA, but only in the analysis where we included the metformin treated individuals.

Variation in functional potential of the bacterial gut microbiome might provide mechanistic insight into the differentiation of the diabetes entities. In Hu et al. ^24^ *Eubacterium rectale* represented a key short-chain acid producer depleted in adult-onset T1D. However, in this study the genus was observed in few samples and did not meet inclusion criteria for differential abundance analyses, highlighting, that despite not being a key microbiome member in the present study the functional profile is conserved. The differences in community members might represent cohort differences, but could also be due to methodology and differences in the diabetes phenotypes compared in the studies. In the present study functional insights were based on predictions from the 16S rRNA gene sequencing data. The abundance of a number of KEGG orthologs involved in lipid metabolism and xenobiotics biodegradation and metabolism, differed comparing individuals with LADA with T1D or control subjects, whereas no differences in abundance of KEGG orthologs were observed between LADA and T2D. Moreover, the abundance of the functional pathway geraniol degradation was significantly higher in LADA compared with T1D and control subjects, which is in line with a previous study showing that Geraniol has antimicrobial properties and is associated with improved glucose homeostasis in diabetes induced in rats^35^. The pathways carotenoid biosynthesis and retinol metabolism were significantly higher in LADA compared with control subjects, which might be explained by the insulin sensitizing effects of carotenoids and retinoids^36,37^. No significant differences in functional pathways were observed between individuals with LADA and T2D, which might indicate that some of the underlying pathways linked to the gut bacteria are similar in LADA and T2D. These findings related to functional differences in the bacterial gut microbiota of LADA, T1D, and T2D should be investigated further in studies applying metagenomics sequencing in a larger sample size with well matched diagnostic groups. Also, the interplay between the host and the gut bacterial potential to modify or synthesize these bioactive compounds have not been explored.

Taxonomic and functional investigations into the microbiome have been associated with the development of T1D, T2D, and maturity-onset diabetes of the young. Nevertheless, findings have been somewhat inconsistent due to differences in methodology and heterogeneous populations^19,32,38,39^. Metformin treatment, a common first-line medication prescribed to individuals diagnosed with LADA and T2D, is a confounding variable in gut microbiota studies^12,40,41^. Attempting to rule out a possible impact of metformin, we repeated all analyses after removal of individuals treated with metformin. This subgroup analysis indicated that LADA and T2D individuals resembled controls, when comparing alpha and beta-diversity measures of their gut microbiota. However, removal of such a large group of samples consequently resulted in a loss of statistical power, and therefore, might introduce type 2 errors. The inclusion of metformin treated individuals was based on a pragmatic approach, as exclusion of metformin treated T2D individuals would challenge patient recruitment immensely and potentially also lead to selection bias. Besides metformin treatment, it would also have been relevant to take other possible confounders into account, including dietary habits. However, even though the diagnostic groups were collected as part of different studies, we consider our study sample to be relatively homogeneous in terms of lifestyle, environment, and ethnicity, as all individuals were recruited from the same part of the country and all individuals were of Danish ancestry. Statistical power might also be an issue in the comparison to the smaller group of individuals with T1D, which possibly explains the relatively few differentially abundant bacterial genera observed in the comparison with individuals with LADA, where we tentatively expected to find greater differences based on the comparisons of alpha and beta-diversity measures. In the study, bacterial-cell counting was performed, which was used to obtain absolute abundances that benefit quantitative analysis.

The non-redundant virome dataset permitted us to explore the gut environment and health and disease status associations. Although we were able to classify a fraction of the viruses into families (13.7%), most of the phages could not be classified, due to the current incompleteness of phage reference databases. The human virome is largely unknown and description of the virome in association with diabetes is sparse and lacking in the context of LADA^24,42^. The human gut virome may be considered an individual signature^25^. The most prevalent families in our study including *Microviridae*, *Siphoviridae*, and *Podoviridae* have been reported as prevalent and abundant in other studies including a range of different cohorts^25,43,44^. In particular, the family Podoviridae, has been related to systemic lupus erythematosus^45^, but has not been associated with T2D^46,47^. Even though we did not find significant associations across the diagnostic groups, this study recognizes the relevance of assessing the microbial environment in a broader context by assessing both the bacterial and viral community. Using metagenomics possibly including metabolomics has the potential to improve the functional understanding of the mechanism underlying the link between microbiota and diabetes subtypes^23,24^.

In conclusion, the present study characterized both the bacterial and viral gut microbiota across diabetic subtypes. We showed that the bacterial gut microbiota of individuals with LADA was most similar to that of individuals with T2D, but there were also great similarities with individuals with T1D. We identified unique taxonomical features in individuals with LADA, and also the signature of lower abundance of short-chain fatty acid producing bacteria, which is shared across all the diabetic subtypes. We did not observe clear virome signatures associating with the diagnostic groups. Our findings may contribute to the understanding of the gut microbiota-related pathophysiology underlying LADA, but we did not identify clear gut microbial biomarkers that can distinguish LADA from T1D or T2D. To gain further information about the potential role of the gut microbiota in LADA pathophysiology it would be relevant in future studies to assess a larger group of individuals with LADA, and at the same time include additional phenotypic information to facilitate analysis of the potential effect of disease duration, anti-diabetic treatment, dietary habits, as well as type and level of autoantibodies.

## Methods

### Study participants

The study comprised 60 individuals diagnosed with LADA, 70 individuals diagnosed withT2D, 30 individuals diagnosed with T1D, and 70 non-diabetic control subjects (Table 1). Individuals with LADA were recruited from Steno Diabetes Center Copenhagen. LADA was defined by; age at onset above 30 years, presence of glutamic acid decarboxylase autoantibodies (GADA), and preserved beta-cell function indicated by clinical diagnosis, treatment without insulin during the first year after diagnosis, or fasting C-peptide above 300 pmol/l.

To match inividuals with LADA on age and sex, 70 individuals with T2D were selected from the MicrobDiab study sample^48^, where all individuals had been diagnosed with diabetes within five years of the clinical visit, and were white Europeans between 35 and 75 years of age. Similarly, 70 matched non-diabetic control subjects were selected from the DanFunD cohort^49^. From the MetaHIT study we obtained 30 eligible individuals with T1D^12,50^. In all cohorts participants treated with antibiotics within three weeks before sample collection and examination were excluded. All included individuals were collected from the greater Copenhagen area and were all of Danish ancestry.

Collection and handling of fecal samples were performed according to the same protocol across all studies. The samples were collected at home using a standard stool collection kit. The protocol emphasized antiseptic handling, using sterile containers, and freezing locally at − 20 °C before transferring in cooling bags to a central facility where samples was stored at − 80 °C.

### DNA extraction and estimation of bacterial cell load

DNA was extracted from 200 mg of feces using the NucleoSpinSoil kit (Macherey–Nagel, Germany), following the manufacturer’s instructions. Quality control of DNA included measurements of yield, purity, and degree of fragmentation using a Qubit 2.0 fluorometer, a NanoDrop2000 spectrometer, and agarose gel electrophoresis, respectively. Bacterial community profiling was performed with primers (515F/806R) targeting the hypervariable region 4 of the 16S rRNA gene, and sequencing on the Illumina HiSeq platform generating a total of 48,794,462 (MEAN = 203,310 SD = 40,317 Minimum = 86,379) 150 bp paired-end reads. Reads were trimmed, filtered, de-replicated, and merged leaving 32,073,812 (MEAN = 133,641 SD = 25,466 Minimum = 61,161) reads for further processing by generating amplicon sequence variants (ASVs) (n_ASVs_ = 6455), and taxonomically annotation to the SILVA database using DADA2 (v1.6.0) in R (v3.4.1)^51^. Cell counts were obtained by fluorescence-activated Cell Sorting (FACS) and used to obtain absolute numbers of cells, which were used in the differential abundance analyses as described below^52^. This enabled the quantitative differential abundance analyses setup relative to a classical compositional approach with concurrent loss of power. It was not possible to obtain a cell count from one sample, which was therefore set as the mean of all samples, because the cell counts obtained were not significantly different from each other in the diagnostic groups (p = 0.62) (Table 1).

### Analyses of the bacterial gut microbiota

Alpha-diversity metrics included richness, Pielou’s evenness index, and Shannon diversity using the diversity function in the vegan package. Differences in alpha-diversity were compared with a Kruskal–Wallis test and a pairwise follow-up Bonferroni corrected Mann–Whitney test. Beta-diversity calculations were based on Bray–Curtis dissimilarities calculated from Hellinger transformed count data. These were used as input to perform PERMANOVA, heatmap dendrograms, and principal coordinate analysis (PCoA). PERMANOVA was performed using the adonis2 implementation in the vegan package as well as a Bonferroni corrected pairwise PERMANOVA between the diagnostic groups. A heatmap of taxonomic abundances was generated for the 30 most abundant genera standardized to zero mean and unit variance. Sample-based dendrograms were generated from the Bray–Curtis dissimilarity matrix using complete-linkage clustering. The genera-based dendrograms were generated using Pearson product-moment correlation coefficients with complete-linkage clustering. PCoA and canonical correspondence analysis (CCA) plots were generated using the capscale function in vegan unconstrained and constrained, respectively.

Differential abundance tests were performed with cell counts as normalization factors in DESeq2 performing both likelihood ratio test (LRT) comparing all diagnostic groups, and a Wald test to make pairwise comparisons of diagnostic groups^53^. Only genera present in 50 or more percent of the samples were included. These genera accounted for the vast majority of the reads (MEAN = 88.9% SD = 11.1). The test results reported in the text is with correction for BMI, but the different test results (effect size: log2fold change) were visualized in a heatmap also accounting for the covariates: Insulin, statins, and PPI. However, information was not available for the control group for these covariates. The comparison of LADA with the other diagnostic groups was visualized as volcano plots and selected significant genera as boxplots. Correction of multiple testing were performed using the Benjamini–Hochberg method (BH) with the default significance level p-value < 0.1. In the rest of the analyses a p-value < 0.05 was considered statistically significant.

Metformin was only used as treatment by some of the T2D (n = 47) and LADA (n = 48) individuals and was therefore not represented in the other groups. To account for effects of metformin on gut microbiota, the analyses were performed omitting data from individuals treated with metformin. Test results provided in text is here without the correction for BMI (Supplementary File 1). Metagenomic function of the DADA2 annotated 16S rRNA gene data was predicted using piphillin and the KEGG database^54,55^. A custom script was used to generate R compatible KEGG brite hierarchies from the online KEGG orthology resource, available at https://github.com/Hansen-Group/KEGGjsonR^56^. Orthologs classified as human diseases and organismal systems were removed from the downstream analyses. The statistical analyses applied to the taxonomical data described above were performed analogously to investigate differences in functional orthologs (Supplementary File 2).

Statistical analyses of the bacterial gut microbiota were performed in R (v4.1.2) and package specifications and function calls are available in supplementary Files 1 and File 2. Visualization of data was performed with ggplot2 3.3.5, pheatmap 1.0.12 and VennDiagram 1.7.1 with final modifications in Inkscape and illustrator, however, all original plots are available in Supplementary Files 1 and File 2. The count tables of ASVs were aggregated to different taxonomic levels but data presented were at genus level. Analyses were performed including: (1) all individuals, and (2) after exclusion of LADA and T2D individuals treated with metformin (Supplementary File 1).

### Processing and analyses of the viral gut microbiota

Separate aliquots of the stool samples were sent for processing according to the NetoVIR protocol, used to enrich viral-like particles and to prepare samples for sequencing^57^. The virome data was characterized as described in Van Espen et al. ^58^. In short, raw reads were trimmed to remove low-quality bases and sequencing adapters using Trimmomatic^59^, reads mapping human reference genome and contaminome using Bowtie2 ^60^ were removed and the remaining reads were per-sample de novo assembled using metaSPAdes ^61^. The obtained scaffolds longer than 1kb were clustered at 95% identity over 80% sequence length to generate a non-redundant reference set, containing 150,743 scaffolds ranging from 1000 bp long to 301 ,716 bp. The filtered reads were mapped to the non-redundant reference database using BWA ^58,62^. The number of mapped reads to each contig was used to create an abundance table for statistical analysis. Viral scaffolds (n = 21,678) were identified using a scoring system based on homology to reference viruses at the amino acid and/or genome level, genome structure, the presence of virus-specific genes, and VirSorter category^63,64^. Eukaryotic viruses were taxonomically classified based on homology to classified reference viruses, while phages (prokaryotic viruses) were classified using vConTACT2^65^.

The statistical analysis was performed in the R (v4.0.5). The absolute counts were summarized into viral family levels for differential abundance analysis using DESeq2^53^. Adjusted p-values < 0.1 were considered as significant for discussion. The counts were normalized, calculating the number of reads per kilobase of contig per million reads mapped (RPKM) and used for heatmap visualization. The ranked abundance difference between groups was assessed using the Wilcoxon rank test. The association between viral prevalence and the diagnosis was measured using Fisher's Exact test. All the p-values were adjusted using the FDR method, and p-values < 0.05 were considered significant.

### Ethics declarations

All individuals gave written informed consent before participation and studies were approved by the Ethical Committees of the Capital Region of Denmark (MicrobDiab: H-3-2013-102; DanFund: H-3-2011-081 and H-3-2012-0015; MetaHIT: HC-2008-017; LADA: H-16046195). Studies were conducted in accordance with the principles of the Declaration of Helsinki.

### Supplementary Information

Supplementary Information.

Supplementary Table 1.

Supplementary Table 2.

Supplementary Table 3.

Supplementary Table 4.

## Data Availability

The datasets generated and analysed during the current study are available under accession number PRJEB68363 (https://www.ebi.ac.uk/ena/browser/view/PRJEB68363).
