# Supplementary material for: Characterization of the gut bacterial and viral microbiota in latent autoimmune diabetes in adults
Source: Sci Rep. 2024 Apr 9;14:8315. doi: 10.1038/s41598-024-58985-w (PMC11003976; doi:10.1038/s41598-024-58985-w)
Supplement: Supplementary file 1 — Supplementary Information. [file 41598_2024_58985_MOESM1_ESM.pdf]

Characterization of the gut bacterial and viral microbiota in latent autoimmune diabetes in adults

Casper S. Poulsen[1], Dan Hesse[2,3], Gabriel R. Fernandes[4], Tue H. Hansen[1], Timo Kern[1,5], Allan Linneberg[6,7], Lore Van Espen[8], Torben Jørgensen[9,10], Trine Nielsen[1], Amra C. Alibegovic[2,3], Jelle Matthijnssens[8], Oluf Pedersen[1,11], Henrik Vestergaard[1,2,12], Torben Hansen[1]*, and Mette K. Andersen[1]*

Affiliation

[1] The Novo Nordisk Foundation Center for Basic Metabolic Research, Faculty of Health and Medical Sciences, University of Copenhagen, Copenhagen, Denmark

[2] Steno Diabetes Center Copenhagen, Gentofte, Denmark

[3] Novo Nordisk A/S, Soeborg, Denmark

[4] Biosystems Informatics, Institute René Rachou – Fiocruz Minas, Belo Horizonte, Brazil

[5] Clinical Microbiomics A/S, Copenhagen, Denmark

[6] Center for Clinical Research and Prevention, Bispebjerg and Frederiksberg Hospital, The Capital Region, Copenhagen, Denmark

[7] Department of Clinical Medicine, Faculty of Health and Medical Sciences, University of Copenhagen, Copenhagen, Denmark

[8] KU Leuven, Department of Microbiology, Immunology & Transplantation, Rega Institute, Laboratory of Clinical & Epidemiological Virology, Leuven, Belgium

[9] Center for Clinical Research and Prevention, Bispebjerg and Frederiksberg Hospital, The Capital Region, Copenhagen, Denmark

[10] Department of Public Health, Faculty of Health and Medical Sciences, University of Copenhagen, Copenhagen, Denmark

[11] Center for Clinical Metabolic Research, Department of Medicine, Gentofte University Hospital, Copenhagen, Denmark

[12] Department of Medicine, Bornholms Hospital, Rønne, Denmark

Correspondence

Torben Hansen & Mette K. Andersen

torben.hansen@sund.ku.dk & metteandersen@sund.ku.dk

Supplementary figure 1: Exploration of differences between diagnostic groups investigating alpha and beta-diversity parameters (a), and by PCoA (b), after removal of individuals treated with metformin. Alpha-diversity was visualized with violin plots and included richness, Pielou's evenness, and Shannon diversity. A non-parametric overall comparison of diagnostic groups was performed using the Kruskal-Wallis test. If significant, a follow-up pairwise comparison of groups was performed with the Mann-Whitney test and p-values were Bonferroni corrected. Bray-Curtis dissimilarity calculated from Hellinger transformed total sum scaled data was used as beta-diversity measure and the violin plot represented the within group variation. PERMANOVA was used to compare all diagnostic groups and follow-up pairwise comparison of diagnostic groups were p-values were Bonferroni corrected. PCoA were used to visualize dissimilarities of all samples. Variance explained by the first two axes were included in their labels. Violin plots of beta-diversity represented within diagnostic group dissimilarity.

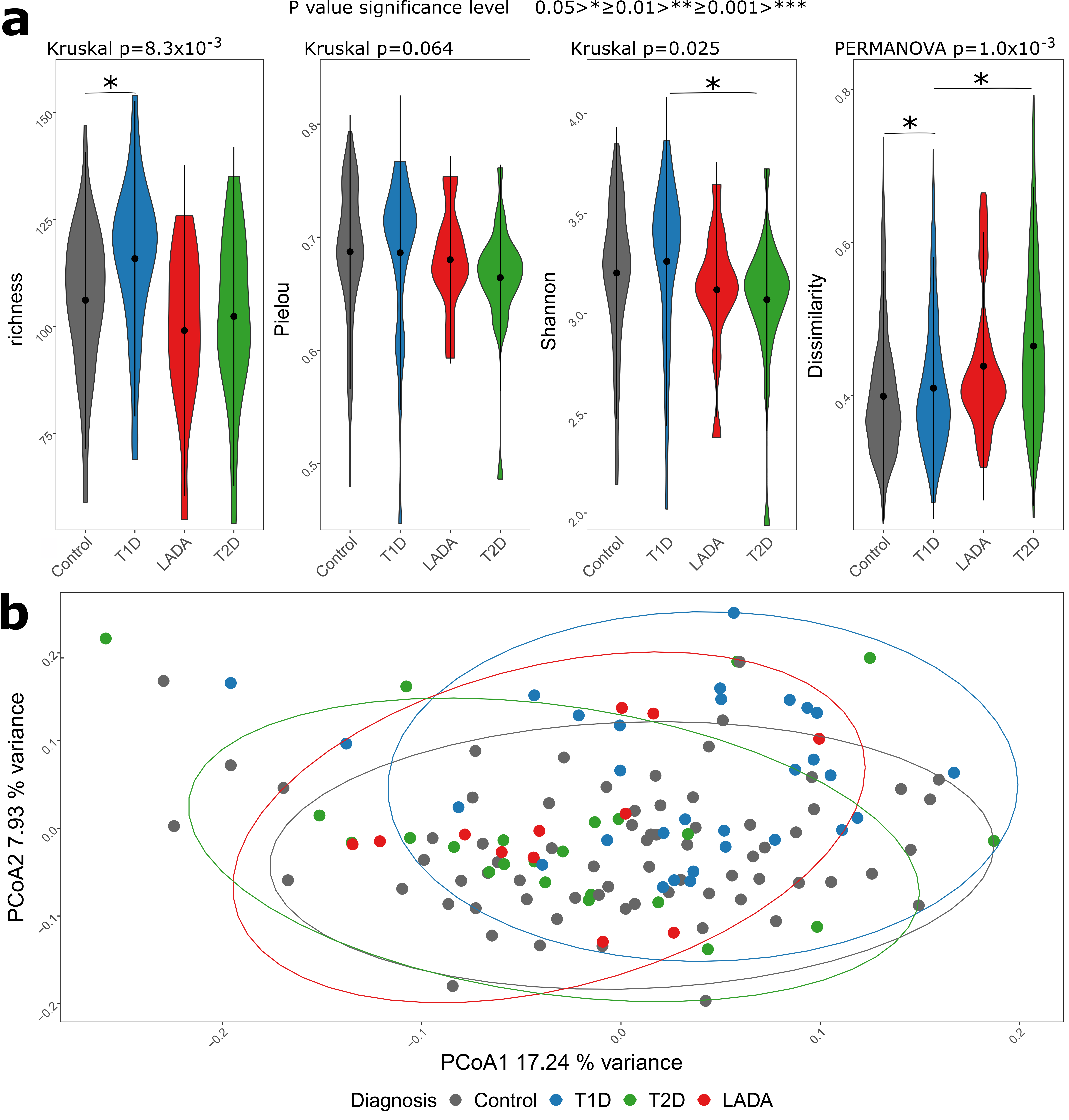

a
P value significance level 0.05>*≥0.01>**≥0.001>***
Kruskal p=8.3x10^-3
Kruskal p=0.064
Kruskal p=0.025
PERMANOVA p=1.0x10^-3
richness
Pielou
Shannon
Dissimilarity
Control
T1D
LADA
T2D
b
PCoA2 7.93 % variance
PCoA1 17.24 % variance
Diagnosis
Control
T1D
T2D
LADA

Supplementary figure 2: Differences in specific genera observed between diagnostic groups, Volcano plot showing results from the differential abundance analysis (a), and boxplots of selected genera (b), after removal of individuals treated with metformin. Differential abundance tests were performed with cell counts as normalization factors in DESeq2 performing both likelihood ratio test, comparing all diagnostic groups, and a Wald test to make pairwise comparisons between diagnostic groups. P-values in the Wald test are Benjamini-Hochberg corrected.

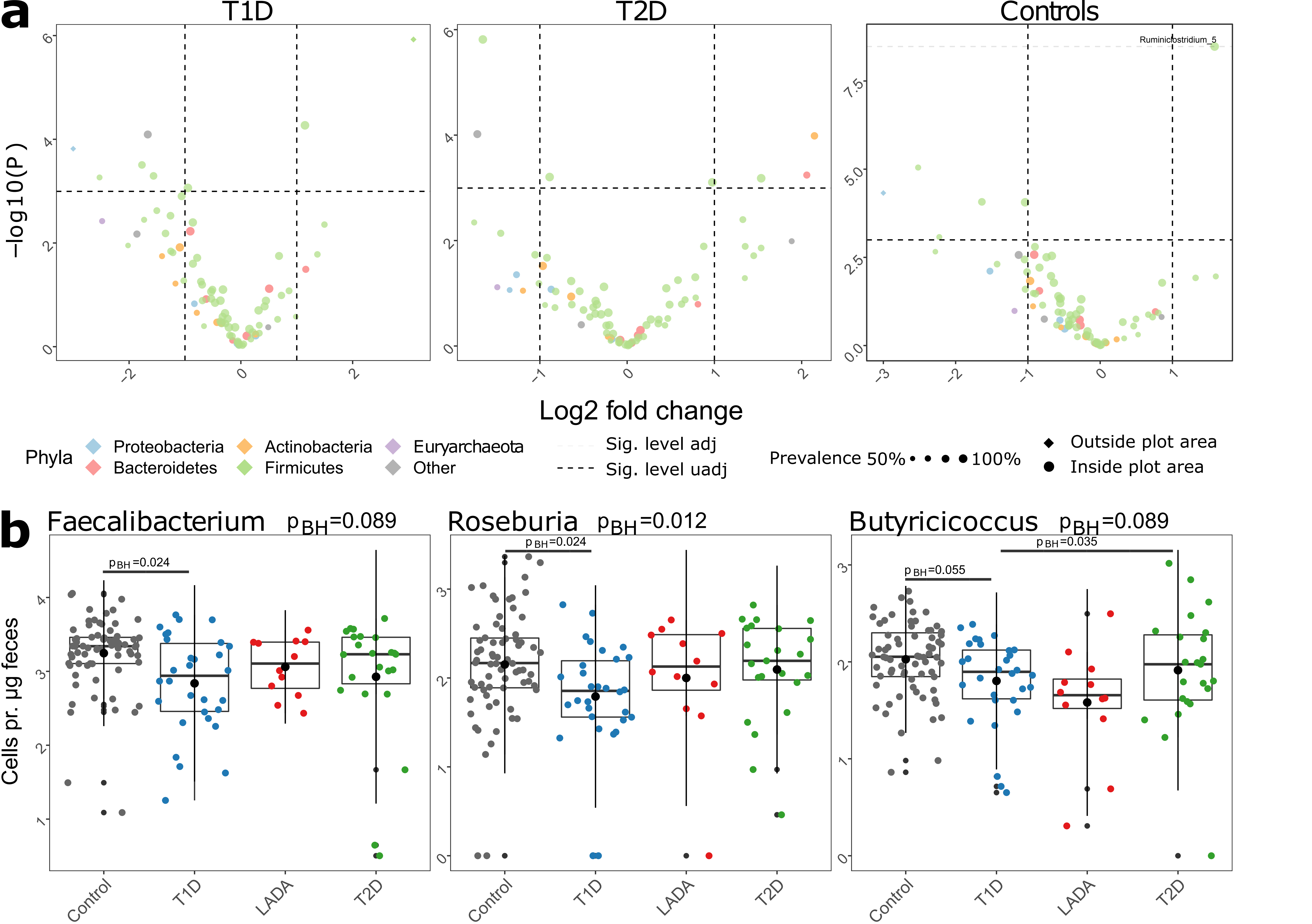
a
T1D
T2D
Controls
Ruminiclostridium_5
−log10(P)
Log2 fold change
Phyla
Proteobacteria
Bacteroidetes
Actinobacteria
Firmicutes
Euryarchaeota
Other
Sig. level adj
Sig. level uadj
Prevalence 50% 100%
Outside plot area
Inside plot area
b
Faecalibacterium p_{BH}=0.089
Roseburia p_{BH}=0.012
Butyricicoccus p_{BH}=0.089
p_{BH}=0.024
p_{BH}=0.024
p_{BH}=0.055
p_{BH}=0.035
Cells pr. µg feces
Control
T1D
LADA
T2D

Supplementary figure 3: Rank abundance curves of genera showing their abundance distribution in all samples and the different diagnostic groups (Controls, type 1 diabetes, type 2 diabetes and LADA).

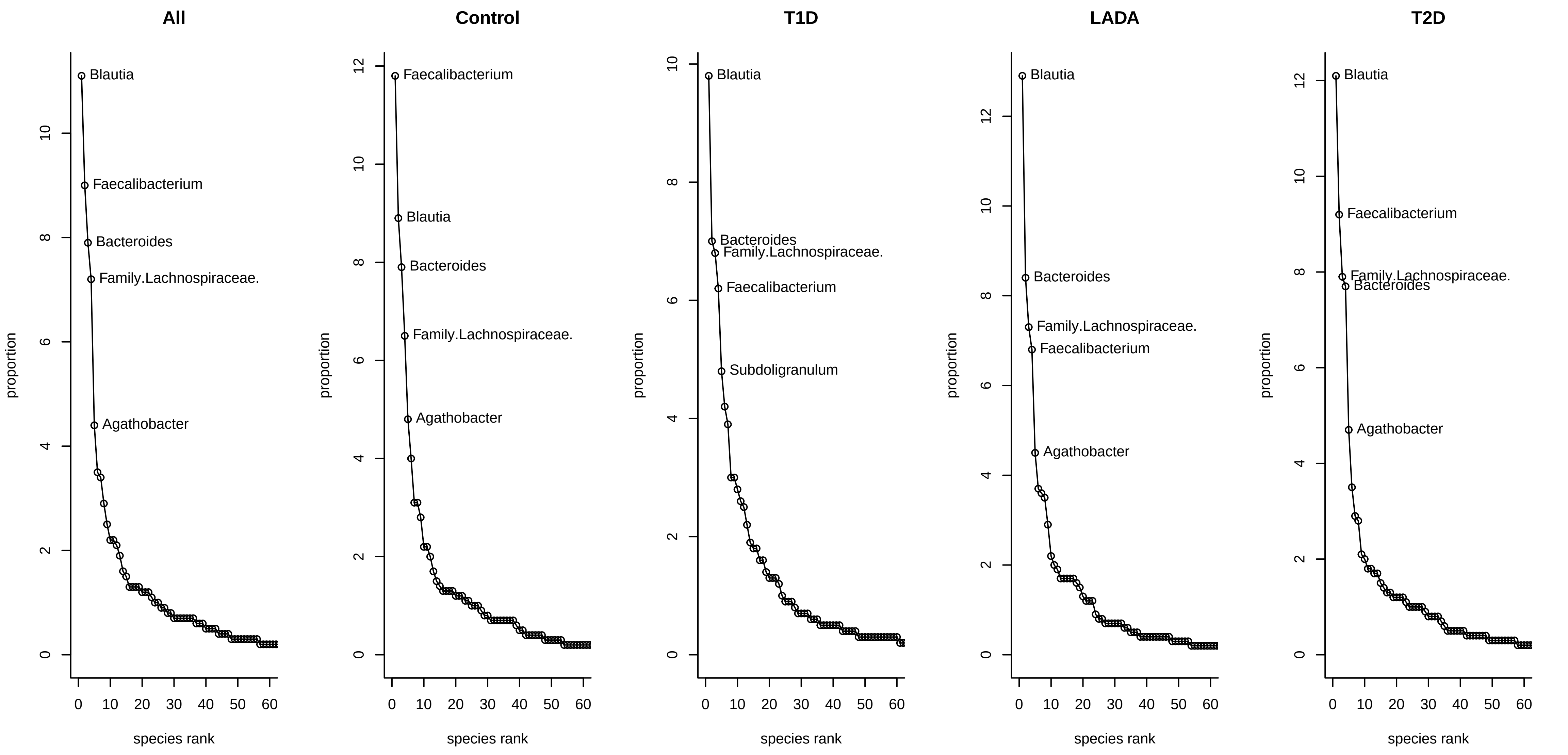
All
Control
T1D
LADA
T2D
proportion
species rank
Blautia
Faecalibacterium
Bacteroides
Family.Lachnospiraceae.
Agathobacter
Subdoligranulum
0
2
4
6
8
10
12
0
10
20
30
40
50
60

Supplementary figure 4: Exploration of functional differences between diagnostic groups investigating alpha and beta-diversity parameters (a), and by PCoA (b). Alpha-diversity was visualized with violin plots and included richness, Pielou's evenness, and Shannon diversity. A non-parametric overall comparison of diagnostic groups was performed using the Kruskal-Wallis test. If significant, a follow-up pairwise comparison of groups was performed with the Mann-Whitney test and p-values were Bonferroni corrected. Bray-Curtis dissimilarity calculated from Hellinger transformed total sum scaled data was used as beta-diversity measure and the violin plot represented the within group variation. PERMANOVA was used to compare all diagnostic groups and follow-up pairwise comparison of diagnostic groups were p-values were Bonferroni corrected. PCoA were used to visualize dissimilarities of all samples. Variance explained by the first two axes were included in their labels. Violin plots of beta-diversity represented within diagnostic group dissimilarity.

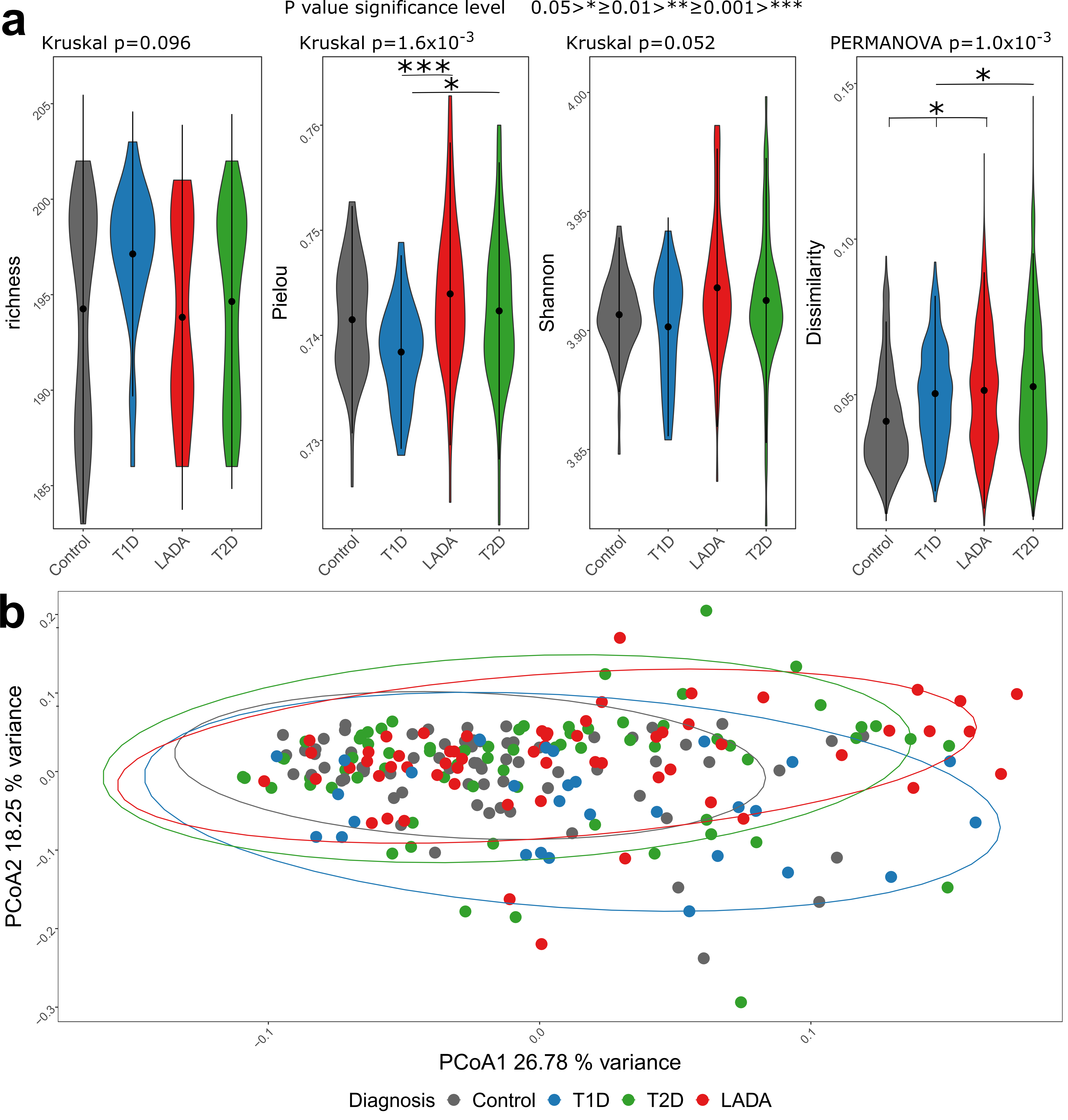

P value significance level 0.05>*≥0.01>**≥0.001>***
a
Kruskal p=0.096
Kruskal p=1.6x10-3
Kruskal p=0.052
PERMANOVA p=1.0x10-3
richness
Pielou
Shannon
Dissimilarity
Control
T1D
LADA
T2D
b
PCoA2 18.25 % variance
PCoA1 26.78 % variance
Diagnosis
Control
T1D
T2D
LADA

Supplementary figure 5: Differences in functional orthologues observed between diagnostic groups, Volcano plot showing results from the differential abundance analysis (a), and boxplots of selected orthologues (b). Differential abundance tests were performed with cell counts as normalization factors in DESeq2 performing both likelihood ratio test, comparing all diagnostic groups, and a Wald test to make pairwise comparisons between diagnostic groups. P-values in the Wald test are Benjamini-Hochberg corrected.

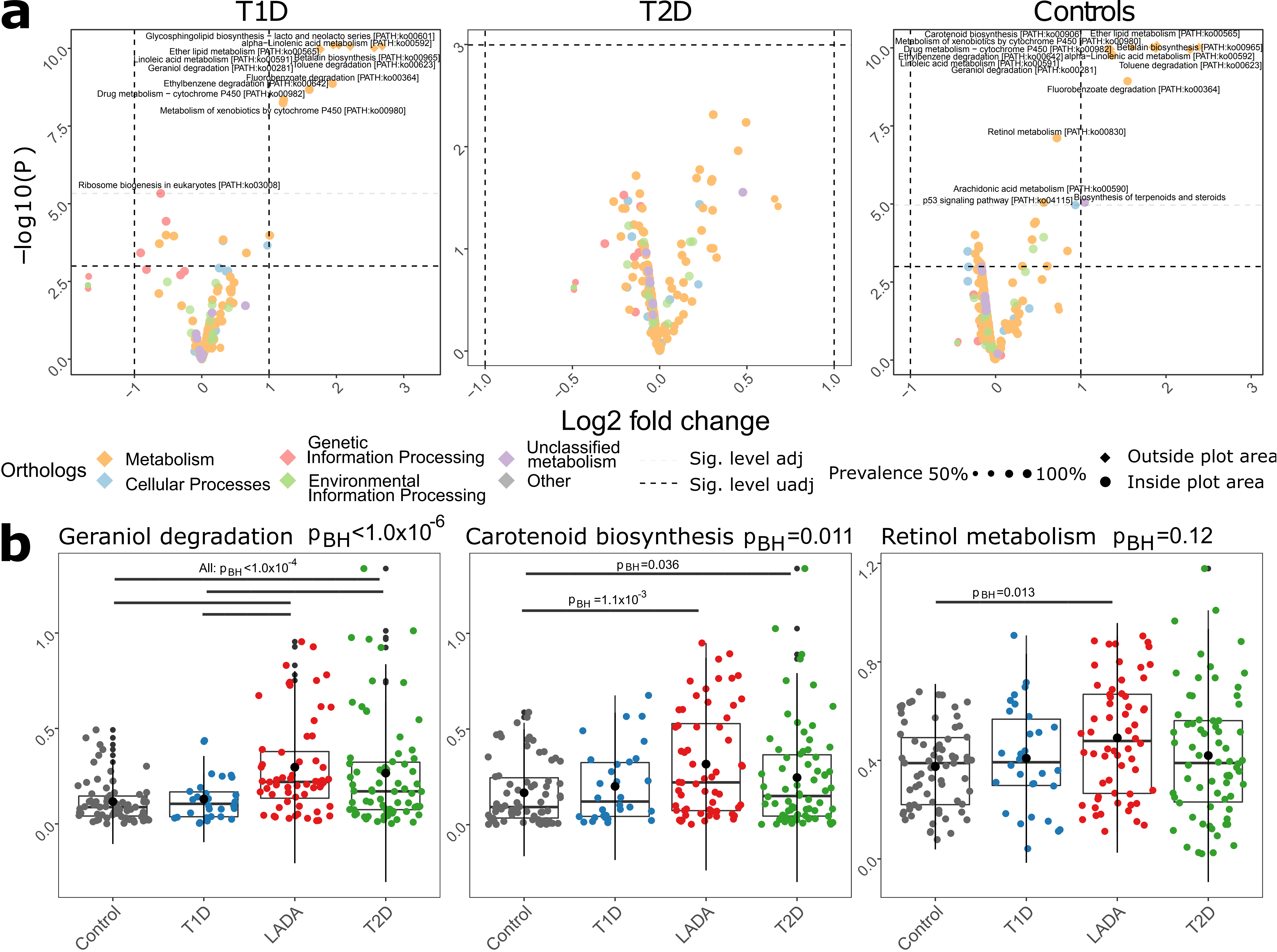
a
T1D
T2D
Controls
Glycosphingolipid biosynthesis – lacto and neolacto series [PATH:ko00601]
alpha−Linolenic acid metabolism [PATH:ko00592]
Ether lipid metabolism [PATH:ko00565]
Linoleic acid metabolism [PATH:ko00591]
Betalain biosynthesis [PATH:ko00965]
Geraniol degradation [PATH:ko00281]
Toluene degradation [PATH:ko00623]
Fluorobenzoate degradation [PATH:ko00364]
Ethylbenzene degradation [PATH:ko00642]
Drug metabolism − cytochrome P450 [PATH:ko00982]
Metabolism of xenobiotics by cytochrome P450 [PATH:ko00980]
Ribosome biogenesis in eukaryotes [PATH:ko03008]
Carotenoid biosynthesis [PATH:ko00906]
Retinol metabolism [PATH:ko00830]
Arachidonic acid metabolism [PATH:ko00590]
p53 signaling pathway [PATH:ko04115]
Biosynthesis of terpenoids and steroids
−log10(P)
Log2 fold change
Orthologs
Metabolism
Cellular Processes
Genetic Information Processing
Environmental Information Processing
Unclassified metabolism
Other
Sig. level adj
Sig. level uadj
Prevalence 50% 100%
Outside plot area
Inside plot area
b
Geraniol degradation $p_{BH}<1.0x10^{-6}$
All: $p_{BH}<1.0x10^{-4}$
Carotenoid biosynthesis $p_{BH}=0.011$
$p_{BH}=0.036$
$p_{BH}=1.1x10^{-3}$
Retinol metabolism $p_{BH}=0.12$
$p_{BH}=0.013$
Control
T1D
LADA
T2D

Supplementary figure 6: Differences in the Podoviridae family across individuals with distinct diagnosis. The boxplot pictures the difference, identified using DESeq2 Wald test, between T2D and Control individuals.

Podoviridae

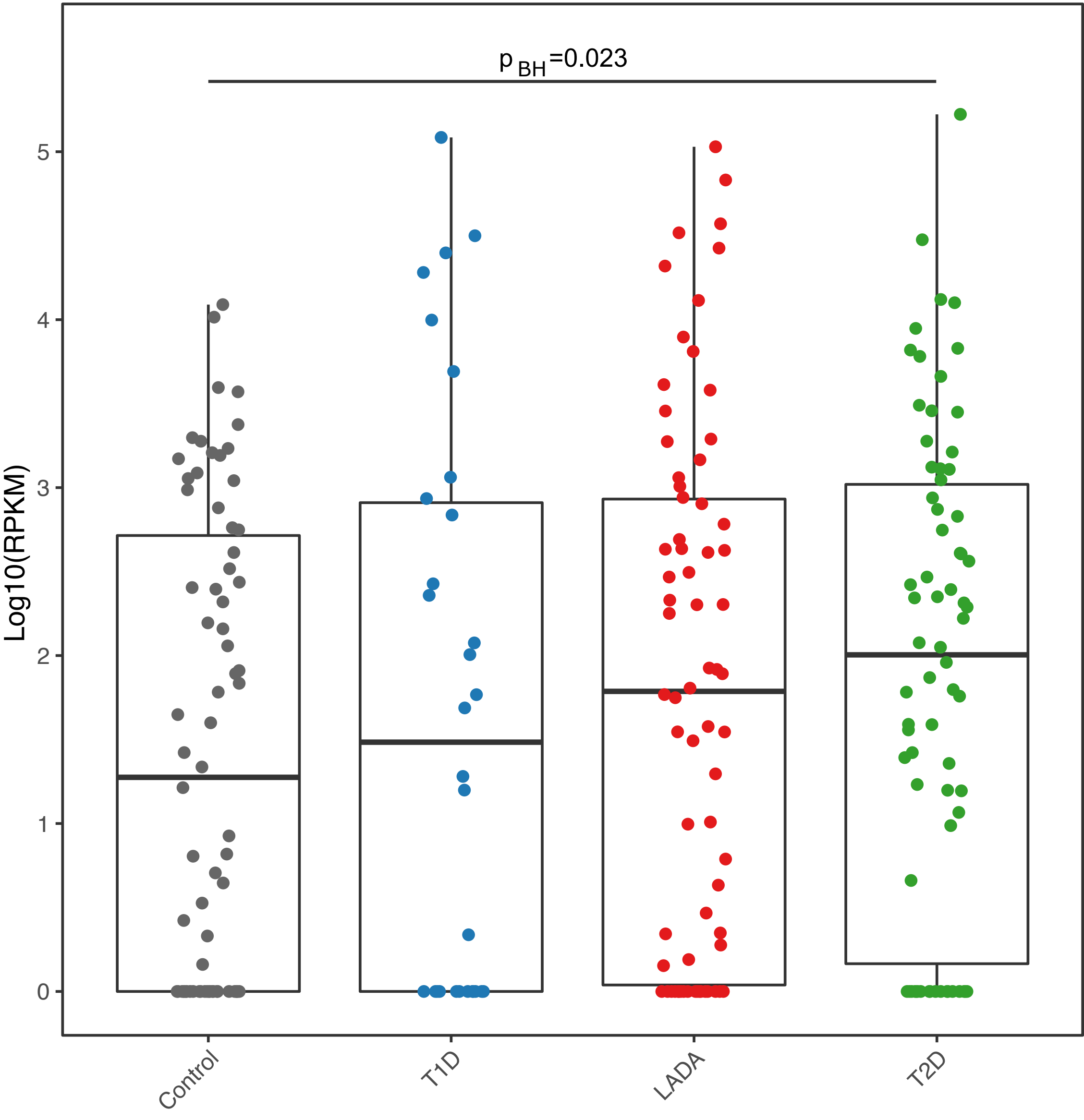

Supplementary file 1: R analysis. Supporting information include additional data summary, PCoA dimensions 1-3 also subsetted to diagnostic groups in pairs, heatmaps, CCA, venn diagrams comparing significant entities between the other groups and violin plots of all genera that had a significant result obtained from the DESeq (Wald) test.

Microbiome analysis LADA study

Casper Sahl Poulsen

februar 24, 2022

Contents

Introduction . . . 2
- Samples . . . 2
- Metadata . . . 2
- Packages . . . 2
- Overview . . . 3
- Used colors throughout script . . . 7

Analysis . . . 9
- Summary metadata, quality of data and ecology . . . 9
- Summary metadata, quality of data and ecology Metformin removed . . . 22
- Alpha diversity . . . 36
- Alpha diversity remove metformin . . . 40
- Violin plots Dissimilarities . . . 43
- Violin plots Dissimilarities remove metformin . . . 51
- PCoA . . . 59
- PCoA remove metformin . . . 75
- Create figure 1 . . . 92
- PERMANOVA . . . 93
- PERMANOVA remove metformin . . . 99
- PERMANOVA MED subset . . . 102
- Pairwise PERMANOVA . . . 106
- Pairwise PERMANOVA remove metformin . . . 109
- Heatmap . . . 110
- CCA . . . 112
- DESeq2 LRT . . . 120
- DESeq2 LRT remove metformin . . . 129
- DESeq Wald . . . 138

DESeq Wald Remove metformin . . . 181
Create figure 2 . . . 217
DESeq heatmap . . . 218
Additional . . . 246
Session information . . . 246
This document was processed on: . . . 247

Introduction

Analysis of microbiome data (16S rRNA gene amplicon profiling) from the latent autoimmune diabetes in adults (LADA) study.

Samples

70 LADA
30 T1D
70 T2D
70 Non-diabetic controls

Metadata

Metadata: is the original metadata provided
MetadataEkstra: is subsequent provided metadata used only in the summary chunks. Have it as separate object, because square bracket selectors are used throughout the script.
MetadataMed: Selected covariates included not part of MetadataEkstra. Extracted from different metadata files from the original cohorts (see also "StenoClean__LADA__Medication__CSP__220105.Rmd).

Packages

```
#install.packages(c("dplyr", "knitr", "ggplot2", "vegan", "gridExtra", "zCompositions",
#                    "compositions", "reshape2", "tidyr", "digest", "mime",
#                    "exactRankTests", "nlme", "plotly", "BiodiversityR", "pheatmap",
#                    "RColorBrewer", "htmlTable", "curl", "stringr", "seqinr", "cowplot",
#                    "VennDiagram", "hablar", "ggnewscale"))
library(tibble)
library(dplyr)
library(knitr)
library(ggplot2)
library(vegan)
library(gridExtra)
library(zCompositions)
library(compositions)
library(reshape2)
library(tidyr)
library(digest)
library(mime)
library(exactRankTests)
```

```r
library(nlme)
library(plotly)
library(htmlTable)
library(curl)
library(BiodiversityR)
library(pheatmap)
library(RColorBrewer)
library(stringr)
library(seqinr)
library(cowplot)
library(VennDiagram)
library(hablar)
library(ggnewscale)

#Bioconductor packages
#if (!requireNamespace("BiocManager", quietly = TRUE))
#    install.packages("BiocManager")
#BiocManager::install("mixOmics")
library("mixOmics")

#BiocManager::install("BiocParallel")
library("BiocParallel")

#BiocManager::install("ALDEx2")
library("ALDEx2")

#BiocManager::install("SIAMCAT")
library("SIAMCAT")

#BiocManager::install("DESeq2")
library("DESeq2")

# old way devtools::install_bitbucket("biobakery/maaslin2@default", ref="tip")
#BiocManager::install("Maaslin2")
library("Maaslin2")
```

Overview

Have to run "Reading in data" and "Pre-processing" from script "MicroLADA_200220.Rmd" to generate files

```r
Metadata <- read.delim(file="P:/CBMR/LADA/Text/Analysis/Metadata_200318.txt",
                       check.names=FALSE,
                       stringsAsFactors = FALSE,
                       strip.white=TRUE,
                       dec=",")
#Specify column types
Metadata <- Metadata %>% convert(chr(Metformin),
                                 num(age, BMI))
#head(Metadata)
#confidential but metadata as colnames and samples as rownames
```

```r
Taxonomy <- read.delim(file="P:/CBMR/LADA/Text/Analysis/Taxonomy_200318.txt",
                       check.names=FALSE,
                       stringsAsFactors = FALSE,
                       strip.white=TRUE,
                       dec=",")
#head(Taxonomy[,1:6])
#confidential but samples as colnames and features/genera as rownames

Feature <- read.delim(file="P:/CBMR/LADA/Text/Analysis/Feature_200318.txt",
                      check.names=FALSE,
                      stringsAsFactors = FALSE,
                      strip.white=TRUE,
                      dec=",")
head(Feature)
```

```
##   Kingdom        Phylum           Class                  Order
## 1 Archaea Euryarchaeota Methanobacteria      Methanobacteriales
## 2 Archaea Euryarchaeota Methanobacteria      Methanobacteriales
## 3 Archaea Euryarchaeota Methanobacteria      Methanobacteriales
## 4 Archaea Euryarchaeota  Thermoplasmata Methanomassiliicoccales
## 5 Archaea Euryarchaeota  Thermoplasmata Methanomassiliicoccales
## 6 Archaea Euryarchaeota  Thermoplasmata Methanomassiliicoccales
##                    Family                           Genus
## 1      Methanobacteriaceae              Methanobrevibacter
## 2      Methanobacteriaceae                  Methanosphaera
## 3      Methanobacteriaceae     Family.Methanobacteriaceae.
## 4 Methanomassiliicoccaceae           Methanomassiliicoccus
## 5  Methanomethylophilaceae Candidatus_Methanomethylophilus
## 6  Methanomethylophilaceae Family.Methanomethylophilaceae.
```

```r
if (setequal(colnames(Taxonomy), Metadata$MicrobiomeID)==FALSE) {
  stop("Metadata and Taxonomy out of sync")
}

#Create syntactically valid variable names
Metadata$MicrobiomeID <- paste("L", Metadata$MicrobiomeID, sep="")
colnames(Taxonomy) <- Metadata$MicrobiomeID

#Order Diagnosis
Metadata$Diagnosis<-ordered(Metadata$Diagnosis,
                            levels=c("Control", "T1D", "LADA", "T2D"))

##Removing age below 30 at diagnosis of LADA
age<-c("LLADA079", "LLADA085", "LLADA082", "LLADA063")
#Ageinfo<-Metadata[match(age, Metadata$MicrobiomeID), ]
Metadata<-Metadata[-match(age, Metadata$MicrobiomeID), ]
##Applying subsetting to OTU tables
Taxonomy<-dplyr::select(Taxonomy, one_of(as.character(Metadata$MicrobiomeID)))

##Removing LADA that were not tested positive for GAD
GAD<-c("LLADA090", "LLADA076", "LLADA095", "LLADA070", "LLADA080")
#GADinfo<-Metadata[match(GAD, Metadata$MicrobiomeID), ]
Metadata<-Metadata[-match(GAD, Metadata$MicrobiomeID), ]
```

```r
##Removing LADA with insufficient metadata available (lacking C-peptide measurement)
Ins<-c("LLADA005")
#Insinfo<-Metadata[match(Ins, Metadata$MicrobiomeID), ]
Metadata<-Metadata[-match(Ins, Metadata$MicrobiomeID), ]

##Applying subsetting to OTU tables
Taxonomy<-dplyr::select(Taxonomy, one_of(as.character(Metadata$MicrobiomeID)))

##Remove orgs that are not present after subsetting.
Taxonomy <- Taxonomy[rowSums(Taxonomy)>0,]

length(Taxonomy)
```

```
## [1] 230
```

```r
#Filtering at the beginning of script
TaxonomyDA<-Taxonomy[length(Taxonomy)-rowSums(Taxonomy == 0) >= length(Taxonomy)/2,]

mean(colSums(TaxonomyDA)/colSums(Taxonomy))
```

```
## [1] 0.8889192
```

```r
sd(colSums(TaxonomyDA)/colSums(Taxonomy))
```

```
## [1] 0.1107038
```

```r
range(colSums(TaxonomyDA)/colSums(Taxonomy))
```

```
## [1] 0.2321024 0.9973209
```

```r
hist(colSums(TaxonomyDA)/colSums(Taxonomy))
```

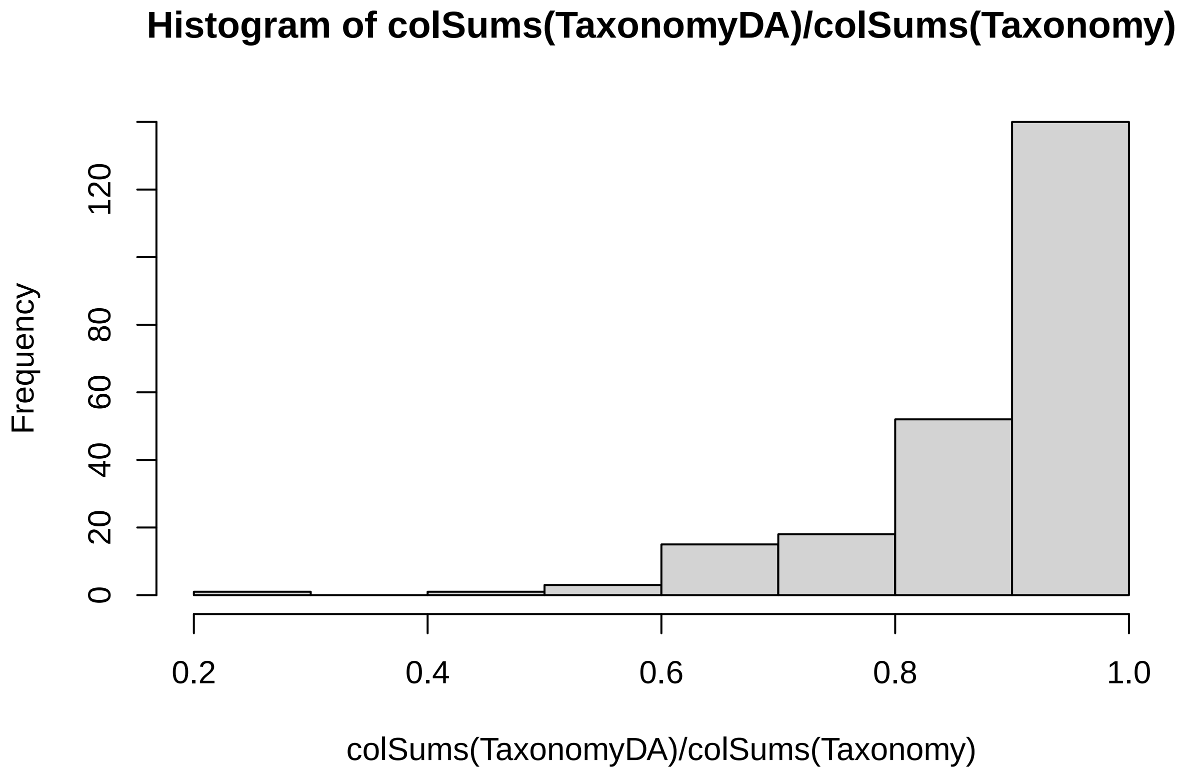


```
##Compared sample where many reads are removed due to 50% filtering
#samplebef<-select(Taxonomy, one_of("L606044"))
#samplefilter<-select(TaxonomyDA, one_of("L606044"))

##Used to provide medication overview
#write.table(Metadata, file="MetadataMedMerge.txt",
#             quote = F, row.names = F, sep="\t")

##See "StenoClean_LADA_Medication_CSP_220105.Rmd" for addition of medication
  #Do not have information on medicine of controls
MetadataMed <- read.delim(file="../Metadata_Medication.txt",
                       check.names=FALSE,
                       stringsAsFactors = FALSE,
                       strip.white=TRUE)

#Encoding BMI
Metadata$BMIord <- ifelse(Metadata$BMI<18.5, "Under",
              ifelse(Metadata$BMI>=18.5 & Metadata$BMI<25, "Normal",
              ifelse(Metadata$BMI>=25 & Metadata$BMI<30, "Over",
              ifelse(Metadata$BMI>=30 & Metadata$BMI<35, "Ob1",
              ifelse(Metadata$BMI>=35 & Metadata$BMI<40, "Ob2",
              ifelse(Metadata$BMI>=40, "Ob3",
                "Other"))))))
MetadataMed$BMIord <- ifelse(MetadataMed$BMI<18.5, "Under",
              ifelse(MetadataMed$BMI>=18.5 & MetadataMed$BMI<25, "Normal",
              ifelse(MetadataMed$BMI>=25 & MetadataMed$BMI<30, "Over",
```

```
             ifelse(MetadataMed$BMI>=30 & MetadataMed$BMI<35, "Ob1",
             ifelse(MetadataMed$BMI>=35 & MetadataMed$BMI<40, "Ob2",
             ifelse(MetadataMed$BMI>=40, "Ob3",
                "Other"))))))
table(Metadata$BMIord) #Based on this could make 3 classes
```

```
##
## Normal    Ob1    Ob2    Ob3   Over  Under
##     54     45     22      6    102      1
```

```
#table(MetadataMed$BMIord)
Metadata$BMIclass <- ifelse(Metadata$BMI<25, "Class1",
             ifelse(Metadata$BMI>=25 & Metadata$BMI<30, "Class2",
             ifelse(Metadata$BMI>=30, "Class3",
                "Other")))
MetadataMed$BMIclass <- ifelse(MetadataMed$BMI<25, "Class1",
             ifelse(MetadataMed$BMI>=25 & MetadataMed$BMI<30, "Class2",
             ifelse(MetadataMed$BMI>=30, "Class3",
                "Other")))
table(Metadata$BMIclass)
```

```
##
## Class1 Class2 Class3
##     55    102     73
```

```
Metadata$BMIordclass <- factor(ifelse(Metadata$BMI<25, 1,
             ifelse(Metadata$BMI>=25 & Metadata$BMI<30, 2,
             ifelse(Metadata$BMI>=30, 3,
                "Other"))))
MetadataMed$BMIordclass <- factor(ifelse(MetadataMed$BMI<25, 1,
             ifelse(MetadataMed$BMI>=25 & MetadataMed$BMI<30, 2,
             ifelse(MetadataMed$BMI>=30, 3,
                "Other"))))

#Quartiles
Metadata$BMIq <- as.numeric(cut_number(Metadata$BMI,4))
MetadataMed$BMIq <- as.numeric(cut_number(MetadataMed$BMI,4))
#table(Metadata$BMIq)
```

Used colors throughout script

```
#install.packages("viridis")
#install.packages("colorBlindness")
#install.packages("RColorBrewer")
library(viridis)
library(colorBlindness)
library(RColorBrewer)
display.brewer.pal(n=8, "Dark2")
display.brewer.pal(n=9, "Set1")
display.brewer.pal(n=12, "Paired")
```

```r
colorBlindness::displayAllColors(viridis::viridis(10))
colorBlindness::displayAllColors(rainbow(10))
colorBlindness::displayAllColors(brewer.pal(n=12, "Paired")) #Should be color blind friendly
colorBlindness::displayAllColors(brewer.pal(n=12, "Set1"))
colorBlindness::displayAllColors(brewer.pal(n=12, "Dark2")) #Should be color blind friendly
colorBlindness::displayAllColors(brewer.pal(n=12, "Set2"))

#From paired but adding Grey from Dark2. Using dark version for
brewer.pal(n=12, "Paired")
brewer.pal(n=12, "Dark2")
brewer.pal(n=12, "Set2")

##From Paired
#"#A6CEE3" Proteobacteria
#"#1F78B4" T1D
#"#B2DF8A" Firmicutes
#"#33A02C" T2D
#"#FB9A99" Bacteroidetes
#"#E31A1C" LADA
#"#FDBF6F" Actinobacteria
#"#FF7F00"
#"#CAB2D6" Euryarchaeota
#"#6A3D9A"
#"#FFFF99"
#"#B15928"

#From Dark2
#"#666666" Controls dark grey

#From Set2
#"#B3B3B3" Other light grey

colorBlindness::displayAllColors(c("#666666", "#1F78B4", "#E31A1C", "#33A02C",
                                   "#A6CEE3", "#B2DF8A", "#FB9A99", "#FDBF6F",
                                   "#CAB2D6", "#B3B3B3"))
```

Controls "#0000FF" Blue new "#666666" dark grey
T1D "#FF0000" Red new "#1F78B4" dark blue
T2D "#228B22" Green new "#33A02C" dark green LADA "#FFD700" Yellow new "#E31A1C" red

Proteobacteria "#3366CC" lightblue new "#005F6A" Petrol new "#A6CEE3" light blue
Bacteroidetes "#DC3912" Redorange new "#9C58A1" Purple new "#FB9A99" pink
Actinobacteria "#FF9900" Yelloworange new "#EF9F26" Orange new "#ADD8E6" light blue new "#00FFFF" Aqua new "#FDBF6F" orange Firmicutes "#109618" Green new "#97D0A7" Seafoam new "#B2DF8A" light green Euryarchaeota "#DD4477" Pink new "#CAB2D6" light purple
Other "#B82E2E" Red new "#4C0013" Bordeaux new "#B3B3B3" light grey

Colors used from color brewer paired. The dark colors for diagnostic groups and light for the phyla for instance in cca and vulcano plots. To represent controls a dark grey was used, a light grey was used for other phyla. LADA is deliberately red to draw the readers attention.

Analysis

Summary metadata, quality of data and ecology

Used to create table 1

```
rm(list=setdiff(ls(), c("Metadata", "MetadataMed", "Feature", "TaxonomyDA", "Taxonomy")))

##Summary metadata and quality of data
#Adding cell count to infer absolute cell numbers
# old path path<-paste("Q:/",
#             "Projects/",
#             "LADA/",
#             "LADA_Sandra_Evelina/",
#             "LADA_JKV/",
#             "LADA_R_AfterFlow_Analysis_FinalCounts/",
#             "LADA_FinalCounts/",
#             "2019-09-03_CountAnalysis.rds", sep="")

path <- paste("J:/",
              "CBMR/",
              "SUN-CBMR-Hansen-Group/",
              "Projects/",
              "LADA/",
              "LADA_Sandra_Evelina/",
              "LADA_JKV/",
              "LADA_R_AfterFlow_Analysis_FinalCounts/",
              "LADA_FinalCounts/",
              "2019-09-03_CountAnalysis.rds", sep="")

TotalCounts<-readRDS(path)
TotalCountsProcess <-
  data.frame(MicrobiomeID=TotalCounts$DF_Save_w$ID,
             CellNorm=TotalCounts$DF_Save_w$Cells_per_g_feces_FR_corrected_mean)
TotalCountsProcess$MicrobiomeID <- paste("L", TotalCountsProcess$MicrobiomeID,
                                         sep="")

covariates<-merge(Metadata, TotalCountsProcess, by="MicrobiomeID")

#One value is NaN in sample L602059 assigns value as average
covariates$CellNorm[is.nan(covariates$CellNorm)]<-
  mean(na.omit(covariates$CellNorm))

covariates<-merge(covariates, data.frame(seq_depth=colSums(Taxonomy),
                                         MicrobiomeID=names(Taxonomy)),
                  by="MicrobiomeID")
print("Sex: male=1, female=2")
```

```
## [1] "Sex: male=1, female=2"
```

```
table(Metadata$sex, Metadata$Diagnosis)
```

```
##
```

```
##     Control T1D LADA T2D
##   1      44  17   37  44
##   2      26  13   23  26
```

```r
print("Metformin: No treatment=0, treatment=1")
```

```
## [1] "Metformin: No treatment=0, treatment=1"
```

```r
table(Metadata$Metformin, Metadata$Diagnosis)
```

```
##
##     Control T1D LADA T2D
##   0      70  30   12  23
##   1       0   0   48  47
```

```r
print("Age, BMI and seq_depth")
```

```
## [1] "Age, BMI and seq_depth"
```

```r
aggregate(covariates[, c(2:3, 9:10)], list(covariates$Diagnosis), mean)
```

```
##   Group.1      age      BMI BMIord BMIclass
## 1 Control 61.44243 26.67011     NA       NA
## 2     T1D 51.73196 26.66011     NA       NA
## 3    LADA 63.76249 28.92226     NA       NA
## 4     T2D 61.42814 31.27686     NA       NA
```

```r
aggregate(covariates[, c(2:3, 9:10)], list(covariates$Diagnosis), sd)
```

```
##   Group.1       age      BMI BMIord BMIclass
## 1 Control 10.189143 3.722805     NA       NA
## 2     T1D  6.961407 4.555865     NA       NA
## 3    LADA 10.183290 4.866525     NA       NA
## 4     T2D  9.956564 5.512931     NA       NA
```

```r
##Look into metadata if patient received metformin or not
print("Sex: male=1, female=2. Metformin: No treatment=0, treatment=1")
```

```
## [1] "Sex: male=1, female=2. Metformin: No treatment=0, treatment=1"
```

```r
table(Metadata$sex, Metadata$Metformin)
```

```
##
##      0  1
##   1 78 64
##   2 57 31
```

```r
print("Age, BMI and seq_depth")
```

```
## [1] "Age, BMI and seq_depth"
```

```r
aggregate(covariates[, c(2:3, 9:10)], list(covariates$Metformin), mean)
```

```
##   Group.1      age      BMI BMIord BMIclass
## 1       0 60.12622 27.62709     NA       NA
## 2       1 61.70115 30.12390     NA       NA
```

```r
aggregate(covariates[, c(2:3, 9:10)], list(covariates$Metformin), sd)
```

```
##   Group.1      age      BMI BMIord BMIclass
## 1       0 10.57613 4.706784     NA       NA
## 2       1 10.02674 5.276407     NA       NA
```

```r
##Testing covariates
#Kruskal test
kruskal.test(BMI ~ Diagnosis, data=covariates)
```

```
##
##  Kruskal-Wallis rank sum test
##
## data:  BMI by Diagnosis
## Kruskal-Wallis chi-squared = 37.354, df = 3, p-value = 3.872e-08
```

```r
kruskal.test(age ~ Diagnosis, data=covariates)
```

```
##
##  Kruskal-Wallis rank sum test
##
## data:  age by Diagnosis
## Kruskal-Wallis chi-squared = 31.529, df = 3, p-value = 6.576e-07
```

```r
kruskal.test(CellNorm ~ Diagnosis, data=covariates)
```

```
##
##  Kruskal-Wallis rank sum test
##
## data:  CellNorm by Diagnosis
## Kruskal-Wallis chi-squared = 1.7778, df = 3, p-value = 0.6198
```

```r
kruskal.test(seq_depth ~ Diagnosis, data=covariates)
```

```
##
##  Kruskal-Wallis rank sum test
##
## data:  seq_depth by Diagnosis
## Kruskal-Wallis chi-squared = 14.806, df = 3, p-value = 0.00199
```

```r
#If significant Mann-Whitney pairwise post-test
pairwise.wilcox.test(covariates$BMI, covariates$Diagnosis,
                     p.adjust.method="bonferroni")
```

```
##
##  Pairwise comparisons using Wilcoxon rank sum test with continuity correction
##
## data:  covariates$BMI and covariates$Diagnosis
##
##      Control T1D     LADA
## T1D  1.00000 -       -
## LADA 0.03204 0.15124 -
## T2D  1e-07   0.00024 0.04139
##
## P value adjustment method: bonferroni
```

```r
pairwise.wilcox.test(covariates$age, covariates$Diagnosis,
                     p.adjust.method="bonferroni")
```

```
##
##  Pairwise comparisons using Wilcoxon rank sum test with continuity correction
##
## data:  covariates$age and covariates$Diagnosis
##
##      Control T1D     LADA
## T1D  1.1e-05 -       -
## LADA 1       8.7e-07 -
## T2D  1       1.8e-05 1
##
## P value adjustment method: bonferroni
```

```r
pairwise.wilcox.test(covariates$seq_depth, covariates$Diagnosis,
                     p.adjust.method="bonferroni")
```

```
##
##  Pairwise comparisons using Wilcoxon rank sum test with continuity correction
##
## data:  covariates$seq_depth and covariates$Diagnosis
##
##      Control T1D    LADA
## T1D  0.0514  -      -
## LADA 0.7095  0.0038 -
## T2D  1.0000  0.0054 1.0000
##
## P value adjustment method: bonferroni
```

```r
##Plot rarefaction curves can decrease step for final plotting
#set.seed(1)
#rarecurve(t(Taxonomy), step=100, xlab="Annotated reads",
#          ylab="Genera", label=FALSE)
```

```
#Create rarefaction curves with colors according to categories
#Adding colors to the rarecurves according to experiment, Controls=#666666 (blue),
#T1D=#1F78B4 (red), T2D=#33A02C (forestgreen), LADA=#E31A1C (gold)
rare<-data.frame(t(Taxonomy))
rare<-add_rownames(rare, "MicrobiomeID")
rare<-merge(rare, Metadata, by="MicrobiomeID")
rare$colors<-ifelse(grepl("0", rare$group), "#666666",
                    ifelse(grepl("1", rare$group), "#1F78B4",
                           ifelse(grepl("2", rare$group), "#33A02C",
                                  ifelse(grepl("4", rare$group),
                                         "#E31A1C", "pink"))))
#table(rare$colors)
rare$line<-ifelse(grepl("0", rare$Metformin), "solid",
                  ifelse(grepl("1", rare$Metformin), "dotted", "longdash"))
#table(rare$line)

#Check order
if (setequal(colnames(Taxonomy), rare$MicrobiomeID)==FALSE) {
  stop("Taxonomy and Metadata is not corresponding with each other")
}

set.seed(1)
rarecurve(t(Taxonomy), step=500, xlab="Annotated reads", ylab="Genera",
          col=rare$colors,
          lty=rare$line, label=FALSE) #piphillin pathways 100000, else 100
```

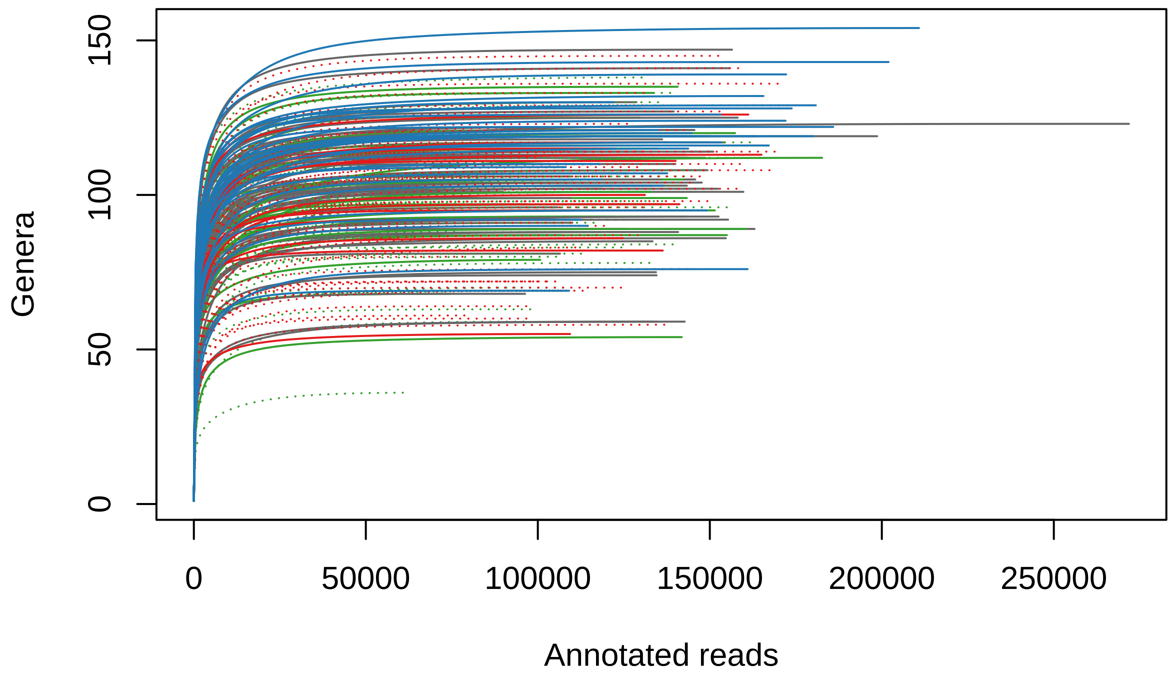

```r
pdf(paste("MicroLADA_Rarecurve.pdf", sep=""), height=6, width=8)
rarecurve(t(Taxonomy), step=500, xlab="Annotated reads", ylab="Genera",
          col=rare$colors,
          lty=rare$line, label=FALSE)
dev.off()
```

```
## pdf
##   2
```

```r
##Rank abundance curves
#Total sum scaling (Use relative abundances)
Taxonomy2<-sweep(Taxonomy, 2, colSums(Taxonomy), FUN="/")

##RAC all and diagnostic groupings
pdf(paste("MicroLADA_RAC", ".pdf", sep=""), width=12, height=6)
par(mfrow=c(1,5))
Subset <- c("All", "Control", "T1D", "LADA", "T2D")

for (i in Subset) {
  #Subsetting Metadata according to
  if (i=="All") {
    Metadata2 <- Metadata
  } else if (i=="Control") {
    Metadata2<-filter(Metadata, Diagnosis == "Control")
  } else if (i=="T1D") {
    Metadata2<-filter(Metadata, Diagnosis == "T1D")
  } else if (i=="LADA") {
    Metadata2<-filter(Metadata, Diagnosis == "LADA")
  } else if (i=="T2D") {
    Metadata2<-filter(Metadata, Diagnosis == "T2D")
  } else {
    print("Subset defined not valid")
  }

  #Applying subsetting to OTU tables, have already TSS
  Taxonomy3<-dplyr::select(Taxonomy2, one_of(as.character(Metadata2$MicrobiomeID)))
  #Setting up ranks
  RankAbun.1 <- rankabundance(t(Taxonomy3))
  #Create RACplot
  rankabunplot(RankAbun.1, scale='proportion', addit=FALSE, specnames=c(1,2,3,4,5),
               xlim=c(0,60), main=i)
}
dev.off()
```

```
## pdf
##   2
```

```r
#Include in pdf
Subset <- c("All", "Control", "T1D", "LADA", "T2D")
for (i in Subset) {
  #Subsetting Metadata according to
  if (i=="All") {
```

```
    Metadata2 <- Metadata
  } else if (i=="Control") {
    Metadata2<-filter(Metadata, Diagnosis == "Control")
  } else if (i=="T1D") {
    Metadata2<-filter(Metadata, Diagnosis == "T1D")
  } else if (i=="LADA") {
    Metadata2<-filter(Metadata, Diagnosis == "LADA")
  } else if (i=="T2D") {
    Metadata2<-filter(Metadata, Diagnosis == "T2D")
  } else {
    print("Subset defined not valid")
  }

  #Applying subsetting to OTU tables, have already TSS
  Taxonomy3<-dplyr::select(Taxonomy2, one_of(as.character(Metadata2$MicrobiomeID)))
  #Setting up ranks
  RankAbun.1 <- rankabundance(t(Taxonomy3))
  #Create RACplot
  rankabunplot(RankAbun.1, scale='proportion', addit=FALSE, specnames=c(1,2,3,4,5),
               xlim=c(0,60), main=i)
}
```

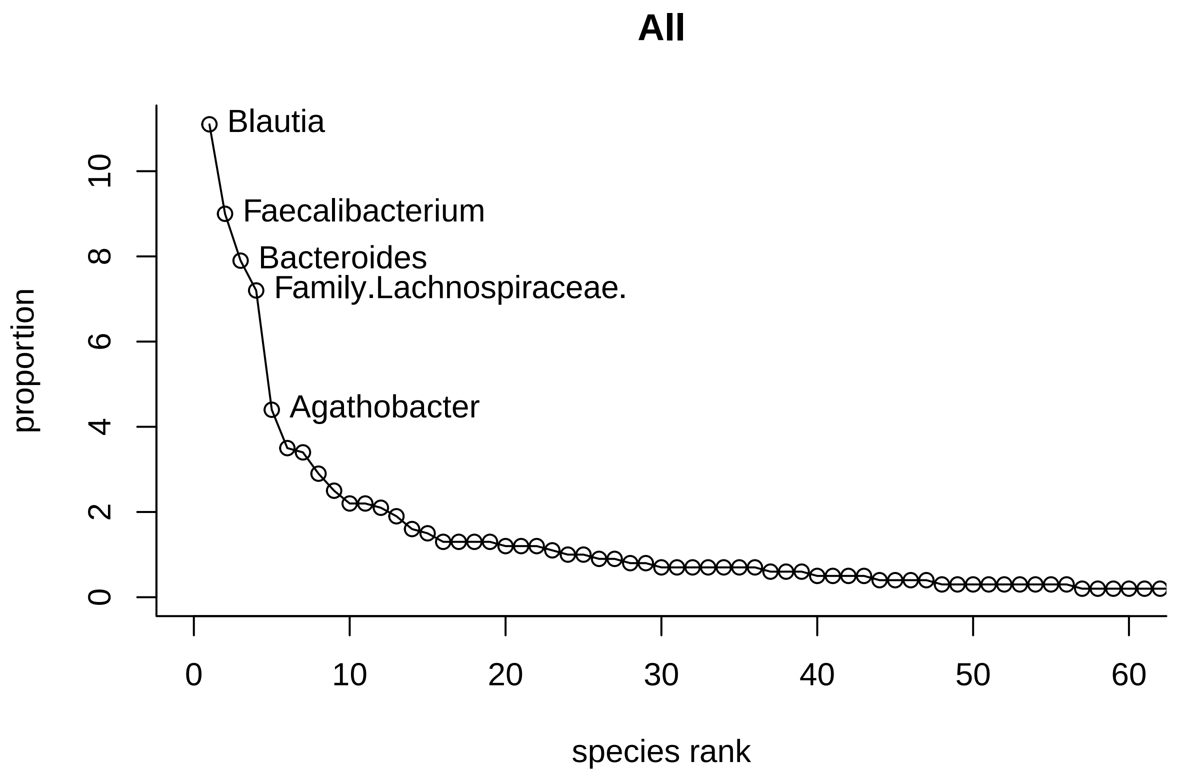

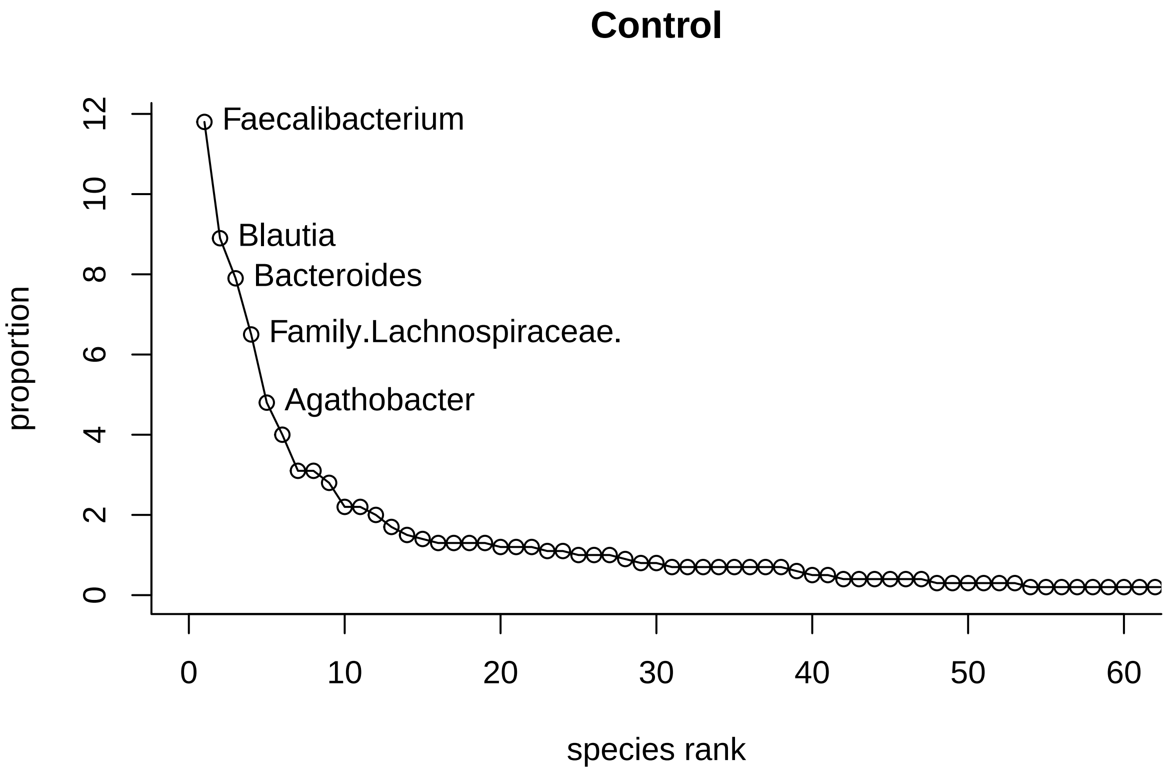
Control
Faecalibacterium
Blautia
Bacteroides
Family.Lachnospiraceae.
Agathobacter
proportion
0
2
4
6
8
10
12
0
10
20
30
40
50
60
species rank

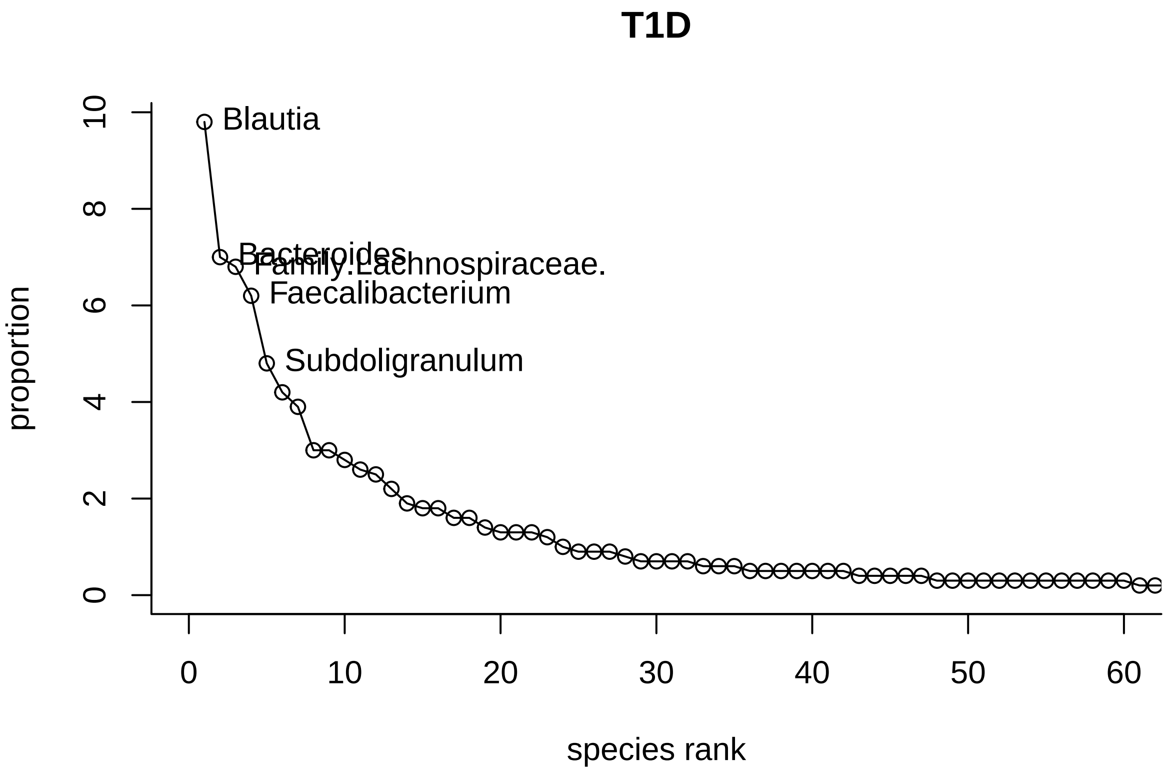
T1D
Blautia
Bacteroides
Family.Lachnospiraceae.
Faecalibacterium
Subdoligranulum
proportion
0
2
4
6
8
10
0
10
20
30
40
50
60
species rank

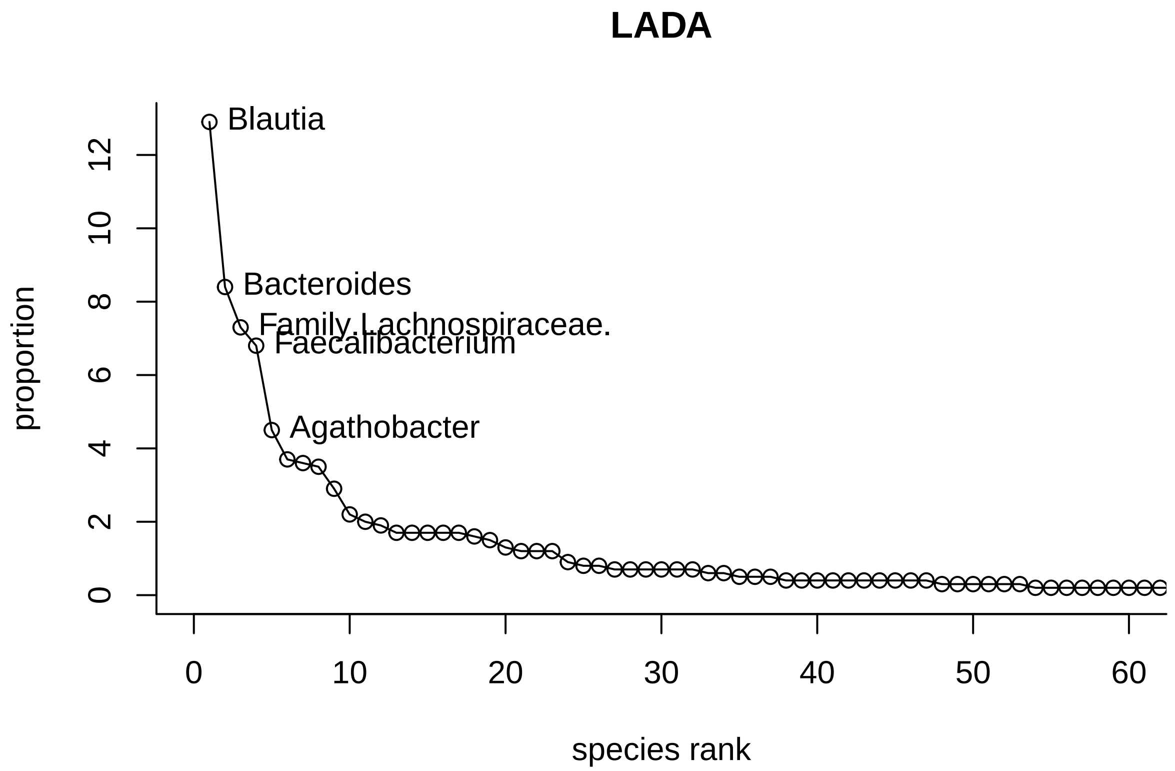

LADA
Blautia
Bacteroides
Family.Lachnospiraceae.
Faecalibacterium
Agathobacter
proportion
0
2
4
6
8
10
12
0
10
20
30
40
50
60
species rank

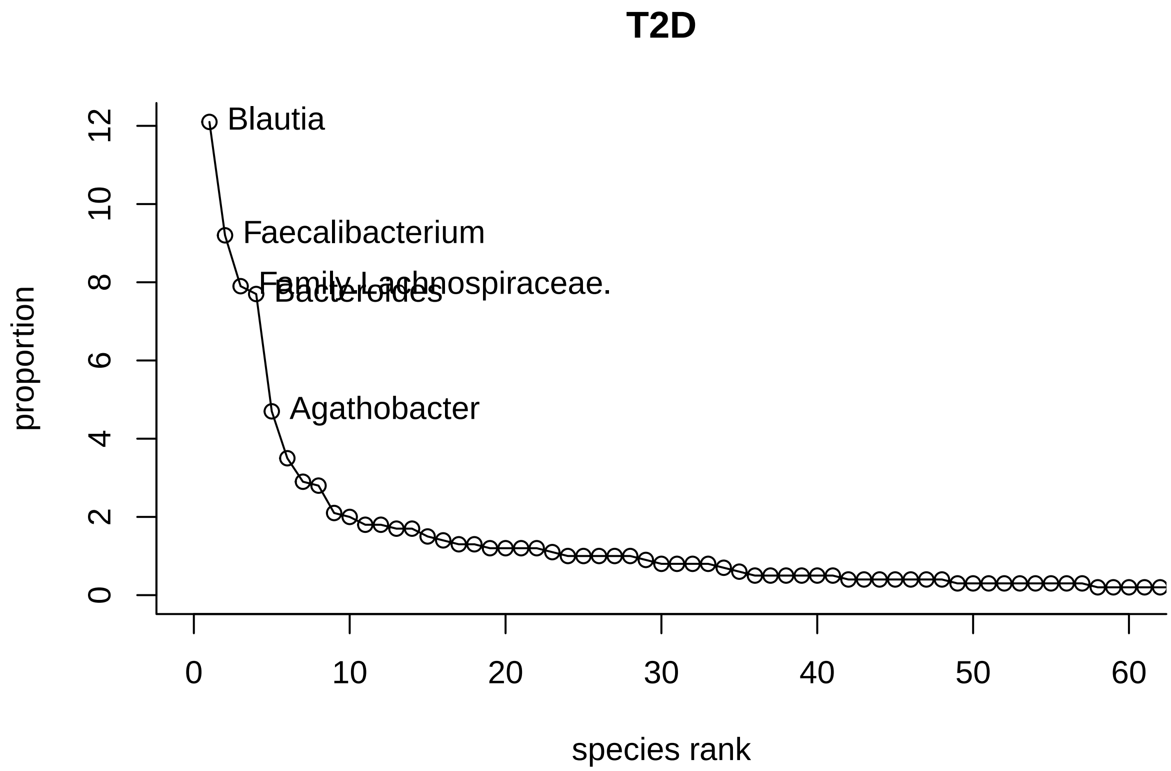


```
#Additional metadata
MetadataEkstra <- read.csv(file="P:/CBMR/LADA/Text/Analysis/LADA_microbiome_extra_metadata.txt",
                           check.names=FALSE,
                           stringsAsFactors = FALSE,
                           strip.white=TRUE,
                           dec=".")

MetadataEkstra$MicrobiomeID <- paste("L", MetadataEkstra$MicrobiomeID,
                                     sep="")

#Merge with previous metadata
covariates <- merge(covariates, MetadataEkstra[,c(1,12:length(MetadataEkstra))])

#Aggregate diagnosis mean and sd
aggregate(covariates[, c(2:3, 12:length(covariates))], list(covariates$Diagnosis), mean,
          na.rm=TRUE)
```

```
##   Group.1      age      BMI     BMIq    CellNorm seq_depth     waist      hip
## 1 Control 61.44243 26.67011 2.042857 20384275367  135669.1  92.89714 101.4143
## 2     T1D 51.73196 26.66011 2.066667 18731782275  150154.5  98.30000 106.1833
## 3    LADA 63.76249 28.92226 2.583333 18940404238  127868.0 103.21667 107.3333
## 4     T2D 61.42814 31.27686 3.071429 19624620327  129389.5 107.45588 107.6324
##         whr      sbp      dbp hba1c_pct hba1c_mmol_mol      glu     chol
## 1 0.9160348 135.7643 81.03571  5.674286       38.47143 5.765714 5.444286
## 2 0.9241084 137.3500 75.98333  8.853333       73.26073 9.740000 4.860000
```

```
## 3 0.9602380 131.6278 77.80556  7.920580       63.06667 8.803333 4.201667
## 4 0.9978302 136.1159 79.89130  6.867143       51.55365 8.477941 4.651429
##        hdl      ldl     trig
## 1 1.369286 3.470000 1.344857
## 2 1.406333 2.703448 1.364333
## 3 1.331833 2.057627 1.729167
## 4 1.210429 2.537879 1.954857
```

```
aggregate(covariates[, c(2:3, 12:length(covariates))], list(covariates$Diagnosis), sd,
          na.rm=TRUE)
```

```
##   Group.1       age      BMI      BMIq   CellNorm seq_depth    waist      hip
## 1 Control 10.189143 3.722805 0.9696214 6781846681  25597.96 10.80459 7.671692
## 2     T1D  6.961407 4.555865 1.2015316 5712095609  27690.10 12.71206 7.790891
## 3    LADA 10.183290 4.866525 1.1393079 5544046390  23620.93 14.49148 9.553891
## 4     T2D  9.956564 5.512931 0.9528208 6146706989  23218.59 13.53319 9.787144
##          whr      sbp       dbp hba1c_pct hba1c_mmol_mol       glu     chol
## 1 0.08380676 18.33301 10.241052 0.3561817       3.907338 0.4845095 1.126574
## 2 0.08055011 21.20026 10.346150 0.8076075       8.826342 4.2322734 1.324725
## 3 0.09391664 17.78699  7.853962 1.1954893      13.065502 2.9649916 1.019054
## 4 0.08120027 16.47573 10.378098 1.2696823      13.876358 2.7289408 1.343017
##         hdl       ldl      trig
## 1 0.3772260 1.0489954 0.8167996
## 2 0.4137422 0.7248068 0.9030855
## 3 0.3909939 0.7788593 1.0163315
## 4 0.3592038 1.0221794 1.2446846
```

```
#Aggregate sex mean and sd
aggregate(covariates[, c(2:3, 12:length(covariates))], list(covariates$sex), mean,
          na.rm=TRUE)
```

```
##   Group.1      age      BMI     BMIq    CellNorm seq_depth     waist      hip
## 1       1 59.92969 28.55643 2.464789 20272037367  132059.5 103.06549 103.8380
## 2       2 62.14355 28.82288 2.556818 18413308125  136117.8  96.70349 108.1221
##         whr      sbp      dbp hba1c_pct hba1c_mmol_mol      glu     chol
## 1 0.9906106 135.4178 80.06338  7.048703       53.54204 7.912857 4.645775
## 2 0.8912291 134.3027 77.74521  7.020671       53.18873 7.871591 5.055682
##        hdl      ldl     trig
## 1 1.228028 2.644928 1.692183
## 2 1.457955 2.851163 1.538295
```

```
aggregate(covariates[, c(2:3, 12:length(covariates))], list(covariates$sex), sd,
          na.rm=TRUE)
```

```
##   Group.1      age      BMI     BMIq   CellNorm seq_depth    waist       hip
## 1       1 10.32654 4.689904 1.108714 6154527421  25160.23 12.78662  7.423021
## 2       2 10.32517 5.701434 1.153286 5972255901  26073.67 15.37737 11.148710
##          whr      sbp      dbp hba1c_pct hba1c_mmol_mol      glu     chol
## 1 0.07681929 16.90142  9.60506  1.469174       16.05979 2.926826 1.198499
## 2 0.07928849 19.87744 10.05909  1.503488       16.46194 3.134126 1.375973
##         hdl      ldl      trig
## 1 0.3401986 1.056925 1.1107317
## 2 0.4118005 1.116164 0.9472746
```

```r
#Test differences
df<-data.frame()
for (i in c(2:3, 12:length(covariates))) {
  design<-paste(colnames(covariates[i]), "~ Diagnosis")
  #Run kruskal-wallis test
  kwobject<-kruskal.test(formula(design), data=covariates)
  #print(kwobject)
  #Run follow up mann-whitney
  mwobject<-pairwise.wilcox.test(covariates[,i], covariates[,8],
                  p.adjust.method="bonferroni")
  #print(mwobject)
  #Bind all test
  df<-rbind(df, data.frame(kwobject$data.name, kwobject$statistic, kwobject$p.value,
                        mwobject$p.value[1,1], mwobject$p.value[2,1], mwobject$p.value[3,1],
                        mwobject$p.value[2,2], mwobject$p.value[3,2], mwobject$p.value[3,3]))

}

colnames(df)<-c("Design", "chistat", "kw p-value (p)", "mw p control vs T1D",
                "mw p control vs LADA", "mw p control vs T2D", "mw p T1D vs LADA",
                "mw p-value T1D vs T2D","mw p-value LADA vs T2D")
kable(df[, 1:5], row.names=FALSE)
```

Design	chistat	kw p-value (p)	mw p control vs T1D	mw p control vs LADA
age by Diagnosis	31.529338	0.0000007	0.0000109	1.0000000
BMI by Diagnosis	37.354166	0.0000000	1.0000000	0.0320433
BMIq by Diagnosis	34.423317	0.0000002	1.0000000	0.0397857
CellNorm by Diagnosis	1.777821	0.6197731	1.0000000	1.0000000
seq_depth by Diagnosis	14.805968	0.0019902	0.0514030	0.7094970
waist by Diagnosis	41.517971	0.0000000	0.5295941	0.0001757
hip by Diagnosis	22.464307	0.0000522	0.0206165	0.0015338
whr by Diagnosis	29.216784	0.0000020	1.0000000	0.0531195
sbp by Diagnosis	3.275907	0.3510101	1.0000000	0.8702673
dbp by Diagnosis	7.564246	0.0559305	0.1759817	0.1800562
hba1c_pct by Diagnosis	158.089616	0.0000000	0.0000000	0.0000000
hba1c_mmol_mol by Diagnosis	157.723084	0.0000000	0.0000000	0.0000000
glu by Diagnosis	95.326163	0.0000000	0.0000018	0.0000000
chol by Diagnosis	40.847549	0.0000000	0.0137855	0.0000000
hdl by Diagnosis	10.868167	0.0124605	1.0000000	1.0000000
ldl by Diagnosis	57.755456	0.0000000	0.0011958	0.0000000
trig by Diagnosis	20.304425	0.0001468	1.0000000	0.0502674

```r
kable(df[, c(1, 6:9)], row.names=FALSE)
```

Design	mw p control vs T2D	mw p T1D vs LADA	mw p-value T1D vs T2D	mw p-value LADA vs T2D
age by Diagnosis	1.0000000	0.0000009	0.0000177	1.0000000
BMI by Diagnosis	0.0000001	0.1512350	0.0002367	0.0413942
BMIq by Diagnosis	0.0000001	0.2511605	0.0008957	0.0857981
CellNorm by Diagnosis	1.0000000	1.0000000	1.0000000	1.0000000

Design	mw p control vs T2D	mw p T1D vs LADA	mw p-value T1D vs T2D	mw p-value LADA vs T2D
seq_depth by Diagnosis	1.0000000	0.0037655	0.0053844	1.0000000
waist by Diagnosis	0.0000000	0.5796713	0.0157666	0.9439178
hip by Diagnosis	0.0001607	1.0000000	1.0000000	1.0000000
whr by Diagnosis	0.0000026	0.4417814	0.0009814	0.2648907
sbp by Diagnosis	1.0000000	1.0000000	1.0000000	0.6594205
dbp by Diagnosis	1.0000000	1.0000000	0.4748683	1.0000000
hba1c_pct by Diagnosis	0.0000000	0.0000212	0.0000000	0.0000000
hba1c_mmol_mol by Diagnosis	0.0000000	0.0000212	0.0000000	0.0000000
glu by Diagnosis	0.0000000	0.9620485	0.3009942	1.0000000
chol by Diagnosis	0.0003009	0.0396162	1.0000000	0.7831524
hdl by Diagnosis	0.0212935	1.0000000	0.0887728	0.2690728
ldl by Diagnosis	0.0000060	0.0032265	0.6392474	0.1175232
trig by Diagnosis	0.0005226	0.1705718	0.0098608	1.0000000

```
#Diagnostic criteria
T1D<-filter(covariates, Diagnosis=="T1D")
range(T1D$hba1c_pct)
```

```
## [1]  7.6 11.0
```

```
Controls<-filter(covariates, Diagnosis=="Control")
range(Controls$glu)
```

```
## [1] 4.2 6.8
```

```
range(Controls$hba1c_pct)
```

```
## [1] 4.8 6.2
```

Summary metadata, quality of data and ecology Metformin removed

```
rm(list=setdiff(ls(), c("Metadata", "MetadataMed", "Feature", "TaxonomyDA", "Taxonomy")))
##Removing treated with metformin
#Metadata$Metformin <- ifelse(is.na(Metadata$Metformin), "Unknown", Metadata$Metformin)
Metadata2<-filter(Metadata, !Metformin == 1)
##Applying subsetting to OTU tables
Taxonomy2<-dplyr::select(Taxonomy, one_of(as.character(Metadata2$MicrobiomeID)))
##Remove orgs that are not present after subsetting.
Taxonomy2 <- Taxonomy2[rowSums(Taxonomy2)>0,]


##Summary metadata and quality of data
#Adding cell count to infer absolute cell numbers
# old path path<-paste("Q:/",
#            "Projects/",
#            "LADA/",
```

```r
#             "LADA_Sandra_Evelina/",
#             "LADA_JKV/",
#             "LADA_R_AfterFlow_Analysis_FinalCounts/",
#             "LADA_FinalCounts/",
#             "2019-09-03_CountAnalysis.rds", sep="")

path <- paste("J:/",
              "CBMR/",
              "SUN-CBMR-Hansen-Group/",
              "Projects/",
              "LADA/",
              "LADA_Sandra_Evelina/",
              "LADA_JKV/",
              "LADA_R_AfterFlow_Analysis_FinalCounts/",
              "LADA_FinalCounts/",
              "2019-09-03_CountAnalysis.rds", sep="")

TotalCounts<-readRDS(path)
TotalCountsProcess <-
  data.frame(MicrobiomeID=TotalCounts$DF_Save_w$ID,
             CellNorm=TotalCounts$DF_Save_w$Cells_per_g_feces_FR_corrected_mean)
TotalCountsProcess$MicrobiomeID <- paste("L", TotalCountsProcess$MicrobiomeID,
                                         sep="")
covariates<-merge(Metadata2, TotalCountsProcess, by="MicrobiomeID")

#One value is NaN in sample L602059 assigns value as average
covariates$CellNorm[is.nan(covariates$CellNorm)]<-
  mean(na.omit(covariates$CellNorm))

covariates<-merge(covariates, data.frame(seq_depth=colSums(Taxonomy2),
                                         MicrobiomeID=names(Taxonomy2)),
                  by="MicrobiomeID")
print("Sex: male=1, female=2")
```

```
## [1] "Sex: male=1, female=2"
```

```r
table(Metadata2$sex, Metadata2$Diagnosis)
```

```
##
##     Control T1D LADA T2D
##   1      44  17    5  12
##   2      26  13    7  11
```

```r
print("Metformin: No treatment=0, treatment=1")
```

```
## [1] "Metformin: No treatment=0, treatment=1"
```

```r
table(Metadata2$Metformin, Metadata2$Diagnosis)
```

```
##
##     Control T1D LADA T2D
##   0      70  30   12  23
```

```r
print("Age, BMI and seq_depth")
```

```
## [1] "Age, BMI and seq_depth"
```

```r
aggregate(covariates[, c(2:3, 9:10)], list(covariates$Diagnosis), mean)
```

```
##   Group.1      age      BMI BMIord BMIclass
## 1 Control 61.44243 26.67011     NA       NA
## 2     T1D 51.73196 26.66011     NA       NA
## 3    LADA 66.61670 28.20713     NA       NA
## 4     T2D 63.68304 31.49826     NA       NA
```

```r
aggregate(covariates[, c(2:3, 9:10)], list(covariates$Diagnosis), sd)
```

```
##   Group.1       age      BMI BMIord BMIclass
## 1 Control 10.189143 3.722805     NA       NA
## 2     T1D  6.961407 4.555865     NA       NA
## 3    LADA 12.720776 5.022780     NA       NA
## 4     T2D  8.549262 5.608501     NA       NA
```

```r
##Look into metadata if patient received metformin or not
print("Sex: male=1, female=2. Metformin: No treatment=0, treatment=1")
```

```
## [1] "Sex: male=1, female=2. Metformin: No treatment=0, treatment=1"
```

```r
table(Metadata2$sex, Metadata2$Metformin)
```

```
##    
##       0
##   1 78
##   2 57
```

```r
print("Age, BMI and seq_depth")
```

```
## [1] "Age, BMI and seq_depth"
```

```r
aggregate(covariates[, c(2:3, 9:10)], list(covariates$Metformin), mean)
```

```
##   Group.1      age      BMI BMIord BMIclass
## 1       0 60.12622 27.62709     NA       NA
```

```r
aggregate(covariates[, c(2:3, 9:10)], list(covariates$Metformin), sd)
```

```
##   Group.1      age      BMI BMIord BMIclass
## 1       0 10.57613 4.706784     NA       NA
```

```r
##Testing covariates
#Kruskal test
kruskal.test(BMI ~ Diagnosis, data=covariates)
```

```
##
##  Kruskal-Wallis rank sum test
##
## data:  BMI by Diagnosis
## Kruskal-Wallis chi-squared = 16.525, df = 3, p-value = 0.0008847
```

```r
kruskal.test(age ~ Diagnosis, data=covariates)
```

```
##
##  Kruskal-Wallis rank sum test
##
## data:  age by Diagnosis
## Kruskal-Wallis chi-squared = 30.881, df = 3, p-value = 9.006e-07
```

```r
kruskal.test(CellNorm ~ Diagnosis, data=covariates)
```

```
##
##  Kruskal-Wallis rank sum test
##
## data:  CellNorm by Diagnosis
## Kruskal-Wallis chi-squared = 9.1305, df = 3, p-value = 0.02761
```

```r
kruskal.test(seq_depth ~ Diagnosis, data=covariates)
```

```
##
##  Kruskal-Wallis rank sum test
##
## data:  seq_depth by Diagnosis
## Kruskal-Wallis chi-squared = 7.8999, df = 3, p-value = 0.04813
```

```r
#If significant Mann-Whitney pairwise post-test
pairwise.wilcox.test(covariates$BMI, covariates$Diagnosis,
                     p.adjust.method="bonferroni")
```

```
##
##  Pairwise comparisons using Wilcoxon rank sum test with continuity correction
##
## data:  covariates$BMI and covariates$Diagnosis
##
##      Control T1D     LADA
## T1D  1.00000 -       -
## LADA 1.00000 1.00000 -
## T2D  0.00059 0.00504 0.68860
##
## P value adjustment method: bonferroni
```

```r
pairwise.wilcox.test(covariates$age, covariates$Diagnosis,
                     p.adjust.method="bonferroni")
```

```
##
##  Pairwise comparisons using Wilcoxon rank sum test with continuity correction
##
## data:  covariates$age and covariates$Diagnosis
##
##      Control T1D     LADA
## T1D  1.1e-05 -       -
## LADA 0.79832 0.00057 -
## T2D  1.00000 7.8e-06 1.00000
##
## P value adjustment method: bonferroni
```

```r
pairwise.wilcox.test(covariates$seq_depth, covariates$Diagnosis,
                     p.adjust.method="bonferroni")
```

```
##
##  Pairwise comparisons using Wilcoxon rank sum test with continuity correction
##
## data:  covariates$seq_depth and covariates$Diagnosis
##
##      Control T1D   LADA
## T1D  0.051   -     -
## LADA 1.000   0.407 -
## T2D  1.000   0.293 1.000
##
## P value adjustment method: bonferroni
```

```r
##Plot rarefaction curves can decrease step for final plotting
#set.seed(1)
#rarecurve(t(Taxonomy), step=100, xlab="Annotated reads",
#          ylab="Genera", label=FALSE)

#Create rarefaction curves with colors according to categories
#Adding colors to the rarecurves according to experiment, Controls=#666666 (blue),
#T1D=#1F78B4 (red), T2D=#33A02C (forestgreen), LADA=#E31A1C (gold)
rare<-data.frame(t(Taxonomy2))
rare<-add_rownames(rare, "MicrobiomeID")
rare<-merge(rare, Metadata2, by="MicrobiomeID")
rare$colors<-ifelse(grepl("0", rare$group), "#666666",
                    ifelse(grepl("1", rare$group), "#1F78B4",
                           ifelse(grepl("2", rare$group), "#33A02C",
                                  ifelse(grepl("4", rare$group),
                                         "#E31A1C", "pink"))))
#table(rare$colors)
rare$line<-ifelse(grepl("0", rare$Metformin), "solid",
                  ifelse(grepl("1", rare$Metformin), "dotted", "longdash"))
#table(rare$line)

#Check order
```

```r
if (setequal(colnames(Taxonomy2), rare$MicrobiomeID)==FALSE) {
  stop("Taxonomy and Metadata is not corresponding with each other")
}

set.seed(1)
rarecurve(t(Taxonomy2), step=500, xlab="Annotated reads", ylab="Genera",
          col=rare$colors,
          lty=rare$line, label=FALSE) #piphillin pathways 100000, else 100
```

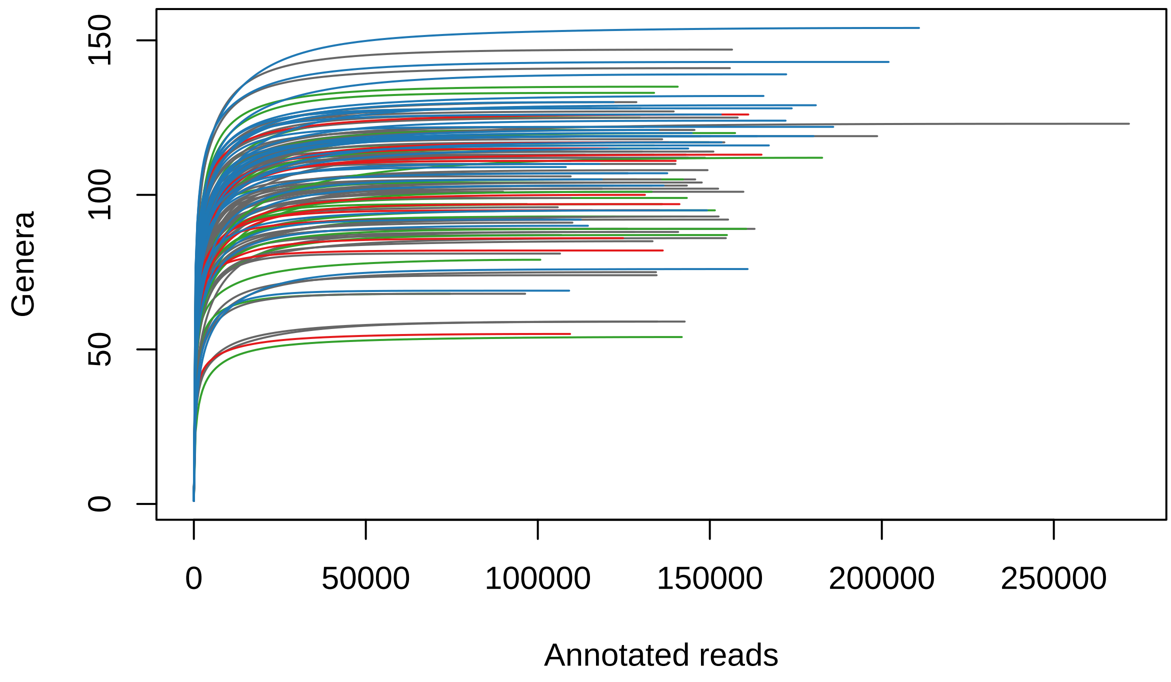


```r
pdf(paste("MicroLADA_Rarecurve_MetRem.pdf", sep=""), height=6, width=8)
rarecurve(t(Taxonomy2), step=500, xlab="Annotated reads", ylab="Genera",
          col=rare$colors,
          lty=rare$line, label=FALSE)
dev.off()
```

```
## pdf
##   2
```

```r
##Rank abundance curves
#Total sum scaling (Use relative abundances)
Taxonomy2<-sweep(Taxonomy2, 2, colSums(Taxonomy2), FUN="/")

##RAC all and diagnostic groupings
pdf(paste("MicroLADA_RAC_MetRem", ".pdf", sep=""), width=12, height=6)
```

```r
par(mfrow=c(1,5))
Subset <- c("All", "Control", "T1D", "LADA", "T2D")

for (i in Subset) {
  #Subsetting Metadata according to
  if (i=="All") {
    Metadata3 <- Metadata2
  } else if (i=="Control") {
    Metadata3<-filter(Metadata2, Diagnosis == "Control")
  } else if (i=="T1D") {
    Metadata3<-filter(Metadata2, Diagnosis == "T1D")
  } else if (i=="LADA") {
    Metadata3<-filter(Metadata2, Diagnosis == "LADA")
  } else if (i=="T2D") {
    Metadata3<-filter(Metadata2, Diagnosis == "T2D")
  } else {
    print("Subset defined not valid")
  }

  #Applying subsetting to OTU tables, have already TSS
  Taxonomy3<-dplyr::select(Taxonomy2, one_of(as.character(Metadata3$MicrobiomeID)))
  #Setting up ranks
  RankAbun.1 <- rankabundance(t(Taxonomy3))
  #Create RACplot
  rankabunplot(RankAbun.1, scale='proportion', addit=FALSE, specnames=c(1,2,3,4,5),
             xlim=c(0,60), main=i)
}
dev.off()
```

```
## pdf
##   2
```

```r
#Include in pdf
Subset <- c("All", "Control", "T1D", "LADA", "T2D")
for (i in Subset) {
  #Subsetting Metadata according to
  if (i=="All") {
    Metadata3 <- Metadata2
  } else if (i=="Control") {
    Metadata3<-filter(Metadata2, Diagnosis == "Control")
  } else if (i=="T1D") {
    Metadata3<-filter(Metadata2, Diagnosis == "T1D")
  } else if (i=="LADA") {
    Metadata3<-filter(Metadata2, Diagnosis == "LADA")
  } else if (i=="T2D") {
    Metadata3<-filter(Metadata2, Diagnosis == "T2D")
  } else {
    print("Subset defined not valid")
  }

  #Applying subsetting to OTU tables, have already TSS
  Taxonomy3<-dplyr::select(Taxonomy2, one_of(as.character(Metadata3$MicrobiomeID)))
  #Setting up ranks
```

```
  RankAbun.1 <- rankabundance(t(Taxonomy3))
  #Create RACplot
  rankabunplot(RankAbun.1, scale='proportion', addit=FALSE, specnames=c(1,2,3,4,5),
               xlim=c(0,60), main=i)
}
```

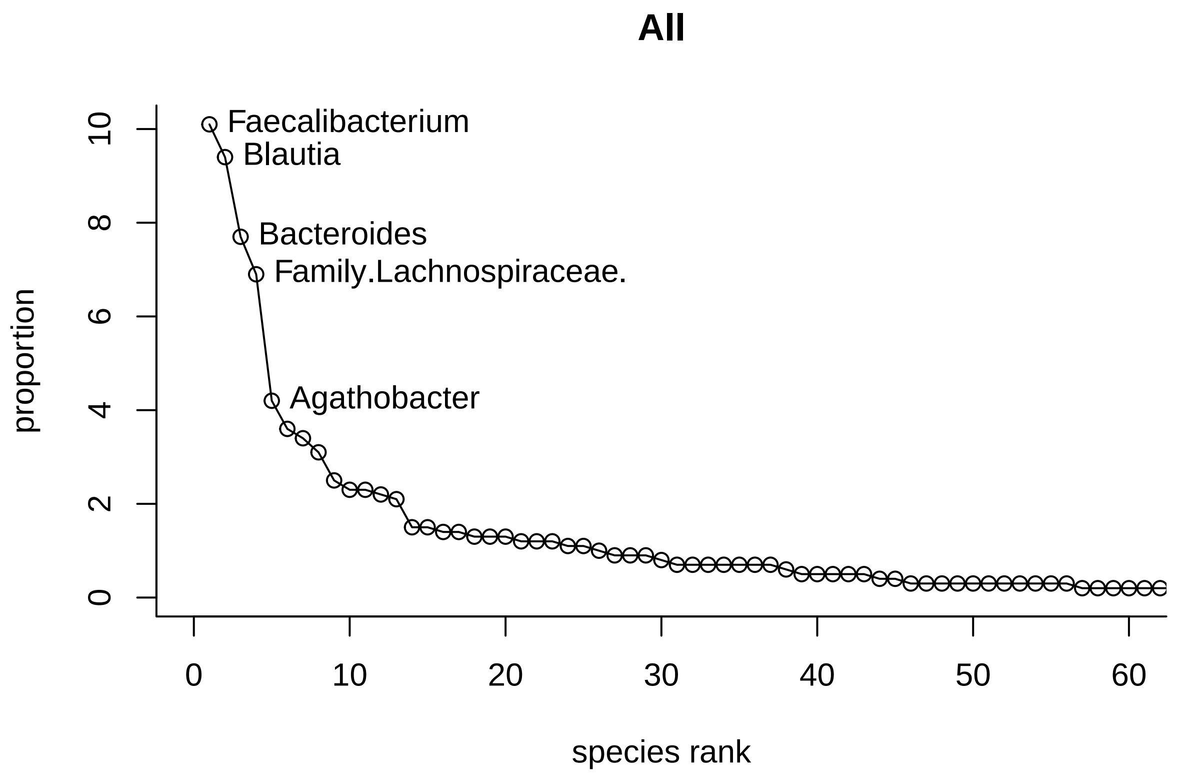

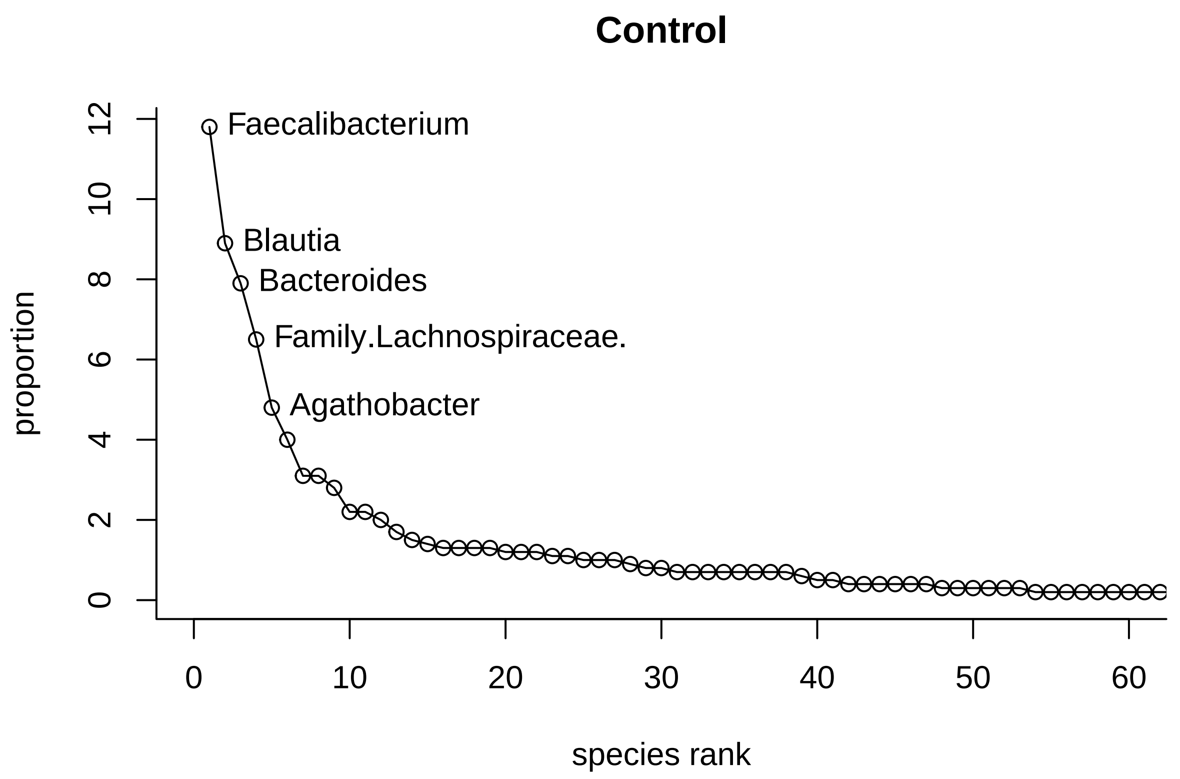
Control
proportion
species rank
Faecalibacterium
Blautia
Bacteroides
Family.Lachnospiraceae.
Agathobacter
0
2
4
6
8
10
12
0
10
20
30
40
50
60

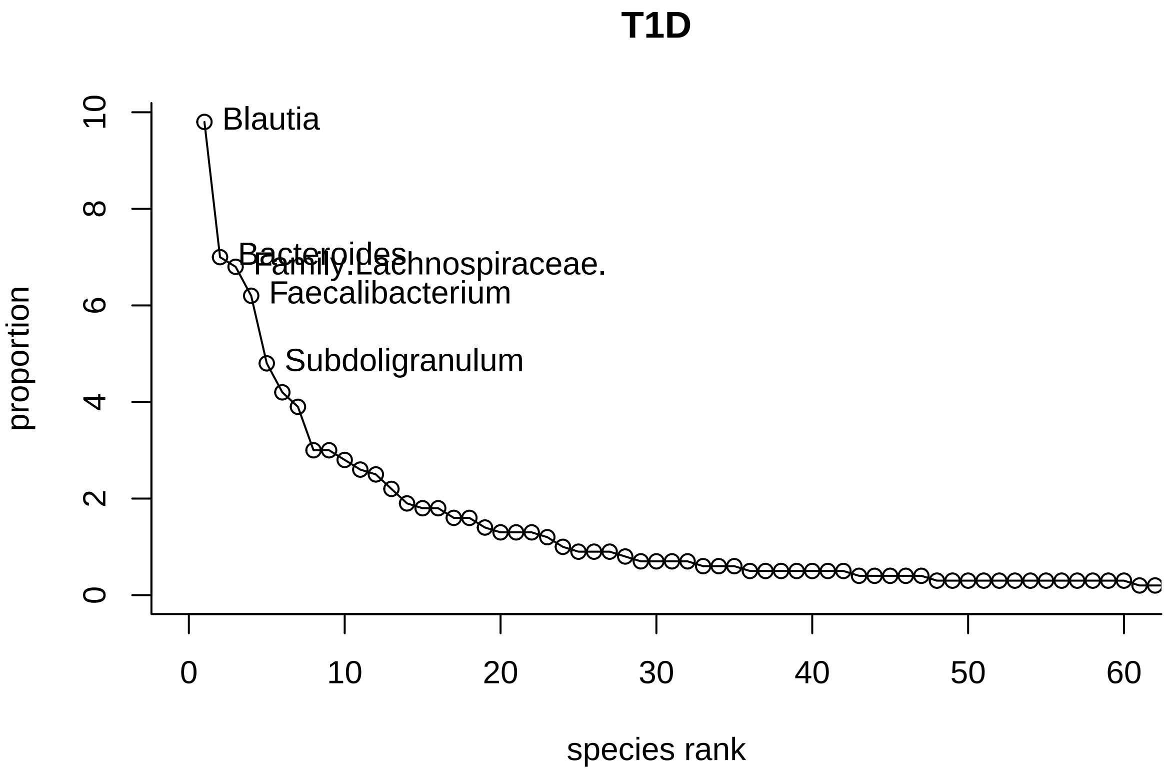
T1D
Blautia
Bacteroides
Family.Lachnospiraceae.
Faecalibacterium
Subdoligranulum
proportion
0
2
4
6
8
10
0
10
20
30
40
50
60
species rank

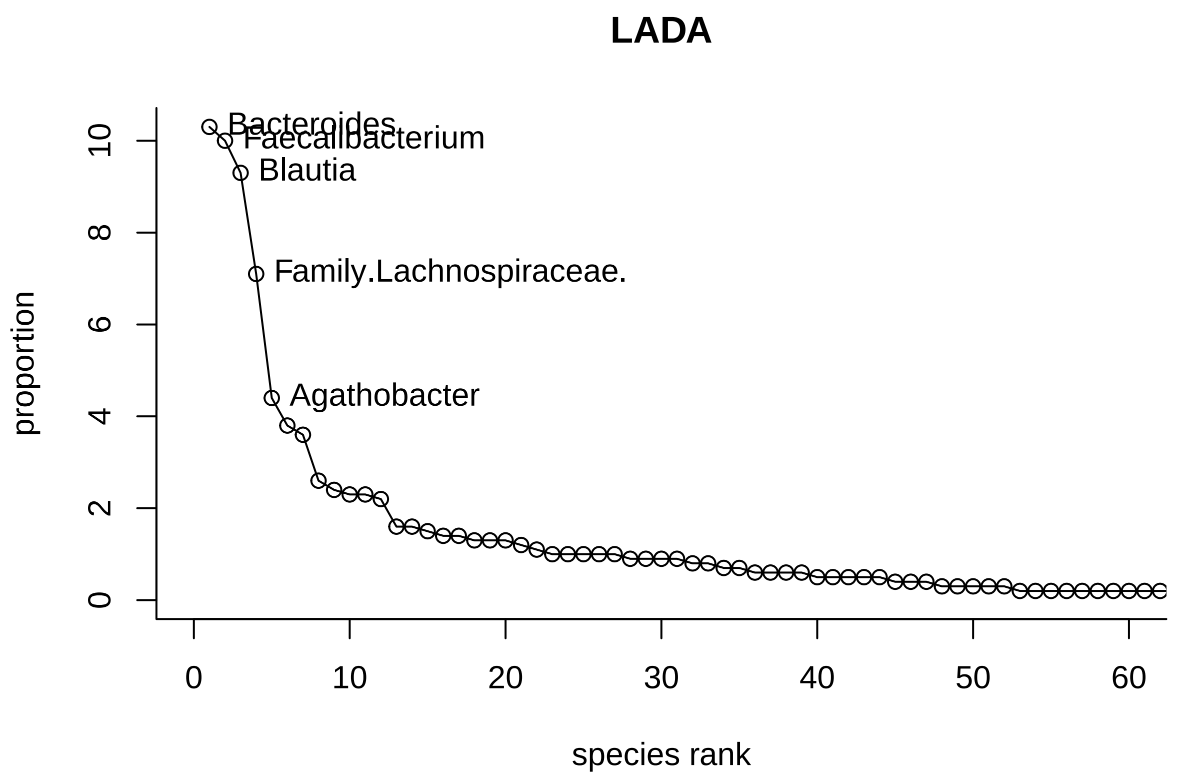

LADA
proportion
species rank
Bacteroides
Faecalibacterium
Blautia
Family.Lachnospiraceae.
Agathobacter
0
2
4
6
8
10
0
10
20
30
40
50
60

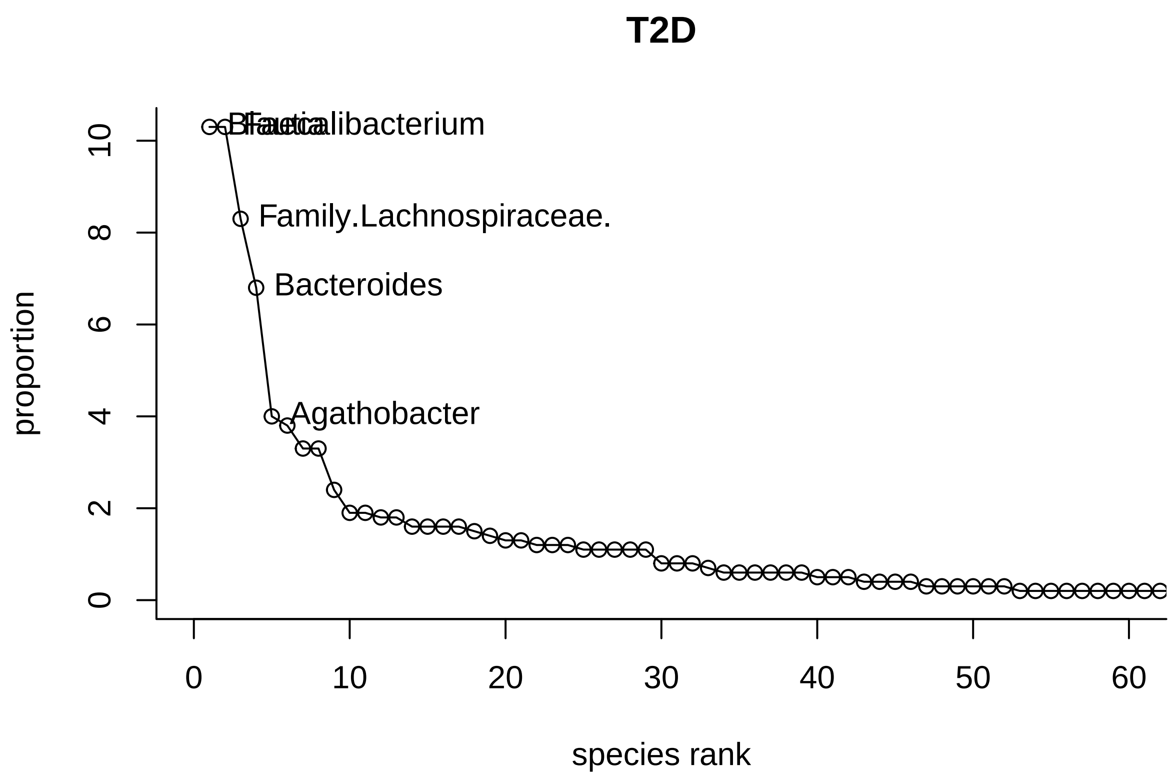


```r
#Additional metadata
MetadataEkstra <- read.csv(file="P:/CBMR/LADA/Text/Analysis/LADA_microbiome_extra_metadata.txt",
                        check.names=FALSE,
                        stringsAsFactors = FALSE,
                        strip.white=TRUE,
                        dec=".")

MetadataEkstra$MicrobiomeID <- paste("L", MetadataEkstra$MicrobiomeID,
                                     sep="")
#Remove metformin
MetadataEkstra$Metformin <- ifelse(is.na(MetadataEkstra$Metformin), "Unknown", MetadataEkstra$Metformin)
MetadataEkstra<-filter(MetadataEkstra, !Metformin == 1)

#Merge with previous metadata
covariates <- merge(covariates, MetadataEkstra[,c(1,12:length(MetadataEkstra))])

#Aggregate diagnosis mean and sd
aggregate(covariates[, c(2:3, 12:length(covariates))], list(covariates$Diagnosis), mean,
          na.rm=TRUE)
```

```
##   Group.1      age      BMI     BMIq    CellNorm seq_depth     waist      hip
## 1 Control 61.44243 26.67011 2.042857 20384275367  135669.1  92.89714 101.4143
## 2     T1D 51.73196 26.66011 2.066667 18731782275  150154.5  98.30000 106.1833
## 3    LADA 66.61670 28.20713 2.416667 15590817082  134002.0  97.41667 105.7500
## 4     T2D 63.68304 31.49826 3.130435 16982538343  134876.8 109.14286 109.8571
```

```
##         whr      sbp      dbp hba1c_pct hba1c_mmol_mol       glu     chol
## 1 0.9160348 135.7643 81.03571  5.674286       38.47143  5.765714 5.444286
## 2 0.9241084 137.3500 75.98333  8.853333       73.26073  9.740000 4.860000
## 3 0.9171111 135.3056 75.30556  8.646477       71.00000 10.050000 4.258333
## 4 0.9917660 137.9318 80.43182  6.882609       51.72268  7.795652 5.113043
##        hdl      ldl     trig
## 1 1.369286 3.470000 1.344857
## 2 1.406333 2.703448 1.364333
## 3 1.441667 2.358333 1.203333
## 4 1.306957 3.040909 1.770870
```

```r
aggregate(covariates[, c(2:3, 12:length(covariates))], list(covariates$Diagnosis), sd,
          na.rm=TRUE)
```

```
##   Group.1       age      BMI      BMIq   CellNorm seq_depth    waist      hip
## 1 Control 10.189143 3.722805 0.9696214 6781846681  25597.96 10.80459 7.671692
## 2     T1D  6.961407 4.555865 1.2015316 5712095609  27690.10 12.71206 7.790891
## 3    LADA 12.720776 5.022780 1.2401124 4604942130  17894.55 16.89787 9.196096
## 4     T2D  8.549262 5.608501 1.0137396 4888235680  22974.66 15.22592 9.323549
##          whr      sbp       dbp hba1c_pct hba1c_mmol_mol       glu      chol
## 1 0.08380676 18.33301 10.241052 0.3561817       3.907338 0.4845095 1.1265744
## 2 0.08055011 21.20026 10.346150 0.8076075       8.826342 4.2322734 1.3247251
## 3 0.11090078 27.12316  7.624334 1.4023922      15.326744 3.5783440 0.8743396
## 4 0.09003109 15.77317  9.861295 1.2730251      13.912891 2.7111685 1.3548916
##         hdl       ldl      trig
## 1 0.3772260 1.0489954 0.8167996
## 2 0.4137422 0.7248068 0.9030855
## 3 0.4074942 0.8328029 0.5037375
## 4 0.3891300 1.1537348 1.0565162
```

```r
#Aggregate sex mean and sd
aggregate(covariates[, c(2:3, 12:length(covariates))], list(covariates$sex), mean,
          na.rm=TRUE)
```

```
##   Group.1      age      BMI     BMIq    CellNorm seq_depth     waist      hip
## 1       1 58.85014 27.72057 2.243590 19682450132  135967.7 100.02949 102.8718
## 2       2 61.87243 27.49916 2.298246 18093154746  142213.7  92.91818 106.1182
##         whr      sbp      dbp hba1c_pct hba1c_mmol_mol      glu     chol
## 1 0.9703762 136.8226 81.09615  6.773460       50.53729 7.182051 5.023077
## 2 0.8725230 135.8929 76.77976  6.956629       52.46553 7.640351 5.329825
##        hdl      ldl     trig
## 1 1.260128 3.109091 1.481923
## 2 1.528246 3.162500 1.309649
```

```r
aggregate(covariates[, c(2:3, 12:length(covariates))], list(covariates$sex), sd,
          na.rm=TRUE)
```

```
##   Group.1      age      BMI     BMIq   CellNorm seq_depth    waist       hip
## 1       1 10.64268 4.322535 1.118741 6322346025  24849.49 12.35226  7.233327
## 2       2 10.32174 5.224095 1.133341 6104790238  26366.18 14.57140 10.077002
##          whr      sbp       dbp hba1c_pct hba1c_mmol_mol      glu     chol
## 1 0.07398661 17.32918  9.446039  1.624815       17.76417 2.929995 1.109689
```

```
## 2 0.07895551 21.95524 10.706984  1.561148       17.10433 3.298477 1.385507
##         hdl      ldl      trig
## 1 0.3359943 1.054088 0.8860859
## 2 0.4024883 1.064905 0.8395066
```

```
#Test differences
df<-data.frame()
for (i in c(2:3, 12:length(covariates))) {
  design<-paste(colnames(covariates[i]), "~ Diagnosis")
  #Run kruskal-wallis test
  kwobject<-kruskal.test(formula(design), data=covariates)
  #print(kwobject)
  #Run follow up mann-whitney
  mwobject<-pairwise.wilcox.test(covariates[,i], covariates[,8],
                    p.adjust.method="bonferroni")
  #print(mwobject)
  #Bind all test
  df<-rbind(df, data.frame(kwobject$data.name, kwobject$statistic, kwobject$p.value,
                          mwobject$p.value[1,1], mwobject$p.value[2,1], mwobject$p.value[3,1],
                          mwobject$p.value[2,2], mwobject$p.value[3,2], mwobject$p.value[3,3]))

}

colnames(df)<-c("Design", "chistat", "kw p-value (p)", "mw p control vs T1D",
                "mw p control vs LADA", "mw p control vs T2D", "mw p T1D vs LADA",
                "mw p-value T1D vs T2D","mw p-value LADA vs T2D")
kable(df[, 1:5], row.names=FALSE)
```

Design	chistat	kw p-value (p)	mw p control vs T1D	mw p control vs LADA
age by Diagnosis	30.8807870	0.0000009	0.0000109	0.7983155
BMI by Diagnosis	16.5253521	0.0008847	1.0000000	1.0000000
BMIq by Diagnosis	16.6786850	0.0008228	1.0000000	1.0000000
CellNorm by Diagnosis	9.1304607	0.0276057	1.0000000	0.0929905
seq_depth by Diagnosis	7.8999374	0.0481256	0.0514030	1.0000000
waist by Diagnosis	17.8198774	0.0004791	0.5295941	1.0000000
hip by Diagnosis	18.6119098	0.0003289	0.0206165	0.7844861
whr by Diagnosis	9.3836893	0.0246015	1.0000000	1.0000000
sbp by Diagnosis	0.5651568	0.9043605	1.0000000	1.0000000
dbp by Diagnosis	7.4063630	0.0600138	0.1759817	0.2760244
hba1c_pct by Diagnosis	98.4356210	0.0000000	0.0000000	0.0000002
hba1c_mmol_mol by Diagnosis	98.3646295	0.0000000	0.0000000	0.0000002
glu by Diagnosis	52.7694420	0.0000000	0.0000018	0.0000338
chol by Diagnosis	17.6586322	0.0005172	0.0137855	0.0012148
hdl by Diagnosis	1.5100365	0.6799559	1.0000000	1.0000000
ldl by Diagnosis	18.9521978	0.0002797	0.0011958	0.0060282
trig by Diagnosis	5.8578350	0.1187355	1.0000000	1.0000000

```
kable(df[, c(1, 6:9)], row.names=FALSE)
```

Design	mw p control vs T2D	mw p T1D vs LADA	mw p-value T1D vs T2D	mw p-value LADA vs T2D
age by Diagnosis	1.0000000	0.0005720	0.0000078	1.0000000
BMI by Diagnosis	0.0005874	1.0000000	0.0050354	0.6886029
BMIq by Diagnosis	0.0003140	1.0000000	0.0121791	0.5623014
CellNorm by Diagnosis	0.1808303	0.6998477	1.0000000	1.0000000
seq_depth by Diagnosis	1.0000000	0.4065423	0.2928497	1.0000000
waist by Diagnosis	0.0001944	1.0000000	0.1050003	0.7471481
hip by Diagnosis	0.0011702	1.0000000	1.0000000	1.0000000
whr by Diagnosis	0.0164348	1.0000000	0.0927908	0.5107970
sbp by Diagnosis	1.0000000	1.0000000	1.0000000	1.0000000
dbp by Diagnosis	1.0000000	1.0000000	0.7308104	1.0000000
hba1c_pct by Diagnosis	0.0000000	1.0000000	0.0000376	0.0046811
hba1c_mmol_mol by Diagnosis	0.0000000	1.0000000	0.0000376	0.0046811
glu by Diagnosis	0.0000006	1.0000000	0.1712058	0.3220620
chol by Diagnosis	1.0000000	0.2505788	1.0000000	0.8339288
hdl by Diagnosis	1.0000000	1.0000000	1.0000000	1.0000000
ldl by Diagnosis	1.0000000	1.0000000	1.0000000	0.6979424
trig by Diagnosis	0.1357372	1.0000000	0.3643004	0.4408450

```
#Diagnostic criteria
T1D<-filter(covariates, Diagnosis=="T1D")
range(T1D$hba1c_pct)
```

```
## [1]  7.6 11.0
```

```
Controls<-filter(covariates, Diagnosis=="Control")
range(Controls$glu)
```

```
## [1] 4.2 6.8
```

```
range(Controls$hba1c_pct)
```

```
## [1] 4.8 6.2
```

Alpha diversity

Part of figure 1

```
rm(list=setdiff(ls(), c("Metadata", "MetadataMed", "Feature", "TaxonomyDA", "Taxonomy")))

#Calculate alpha diversity stats
#Use vegan to calculate various diversity and richness indices for each sample
set.seed(1)
diversityCalc <- data.frame(Shannon=diversity(t(Taxonomy), index="shannon"),
                            Simpson=diversity(t(Taxonomy), index="simpson"),
                            invSimpson=diversity(t(Taxonomy), index="invsimpson"),
                            fisher=fisher.alpha(t(Taxonomy)),
                            richness=specnumber(t(Taxonomy)),
```

```r
                              rarefy_min_count=rarefy(t(Taxonomy),
                                                         sample=min(rowSums(t(Taxonomy)))),
                              chao1=estimateR(t(Taxonomy))["S.chao1",],
                              chao1SE=estimateR(t(Taxonomy))["se.chao1",],
                              ShannonRar=diversity(rrarefy(data.frame(t(Taxonomy)),
                                                              min(rowSums(t(Taxonomy)))),
                                                      index="shannon"),
                              SimpsonRar=diversity(rrarefy(data.frame(t(Taxonomy)),
                                                              min(rowSums(t(Taxonomy)))),
                                                      index="simpson"),
                              invSimpsonRar=diversity(rrarefy(data.frame(t(Taxonomy)),
                                                                 min(rowSums(t(Taxonomy)))),
                                                         index="invsimpson"),
                              Pielou=diversity(t(Taxonomy))/log(specnumber(t(Taxonomy))))
#Merge with metadata.
diversityCalc<-add_rownames(diversityCalc, "MicrobiomeID")
Metadata2 <- merge(Metadata, diversityCalc, by="MicrobiomeID")

#Create a list to hold the plot objects.
Fig1List <- list()
#Create vector to loop
AlphaDiv<-c("fisher", "Shannon", "Simpson", "invSimpson", "richness", "chao1",
            "ShannonRar", "SimpsonRar", "invSimpsonRar", "Pielou")
#Plot
for (i in AlphaDiv) {
#Create plot name
pltName <- paste('Alpha', i, sep = '')
#create boxplots
Fig1List[[ pltName ]]<-
ggplot(Metadata2, aes_string(x="Diagnosis", y=i, group="Diagnosis")) +
    geom_violin(aes(fill=Diagnosis, trim=FALSE)) +
    stat_summary(fun.data="mean_sdl",
                 mult=1, #mean plus minus a constant (mult=1) times the st.dev
                 geom="pointrange",
                 width=0.2 ) +
    #stat_summary(fun.y = mean, geom = "point") +
    #facet_grid(. ~ Metformin, scales="fixed") + #Scales can also be "free"
    #ggtitle(paste("Genus", i, sep=" ")) +
    #xlab("Diagnosis") +
    ylab(paste(i)) +
    scale_fill_manual(values=c(Control = "#666666", T1D = "#1F78B4",
                               T2D = "#33A02C", LADA = "#E31A1C")) +
    theme_bw() +
    theme(legend.position="none", panel.grid.major = element_blank(),
          panel.grid.minor = element_blank(), axis.title=element_text(size=20),
          axis.title.x = element_blank(),
          axis.text.x = element_text(angle = 45, hjust = 1, size=16),
          axis.text.y = element_text(angle = 45, hjust = 1, size=12))
}


#Have the plots stored in lists
lay <- rbind(c(1,2,3))
```

```r
pdf(paste("MicroLADA_Alpha", ".pdf", sep=""), width=12, height=6)
grid.arrange(Fig1List$Alpharichness,
             Fig1List$AlphaPielou, Fig1List$AlphaShannon, layout_matrix = lay)
dev.off()
```

```
## pdf
##   2
```

```r
grid.arrange(Fig1List$Alpharichness,
             Fig1List$AlphaPielou, Fig1List$AlphaShannon, layout_matrix = lay)
```

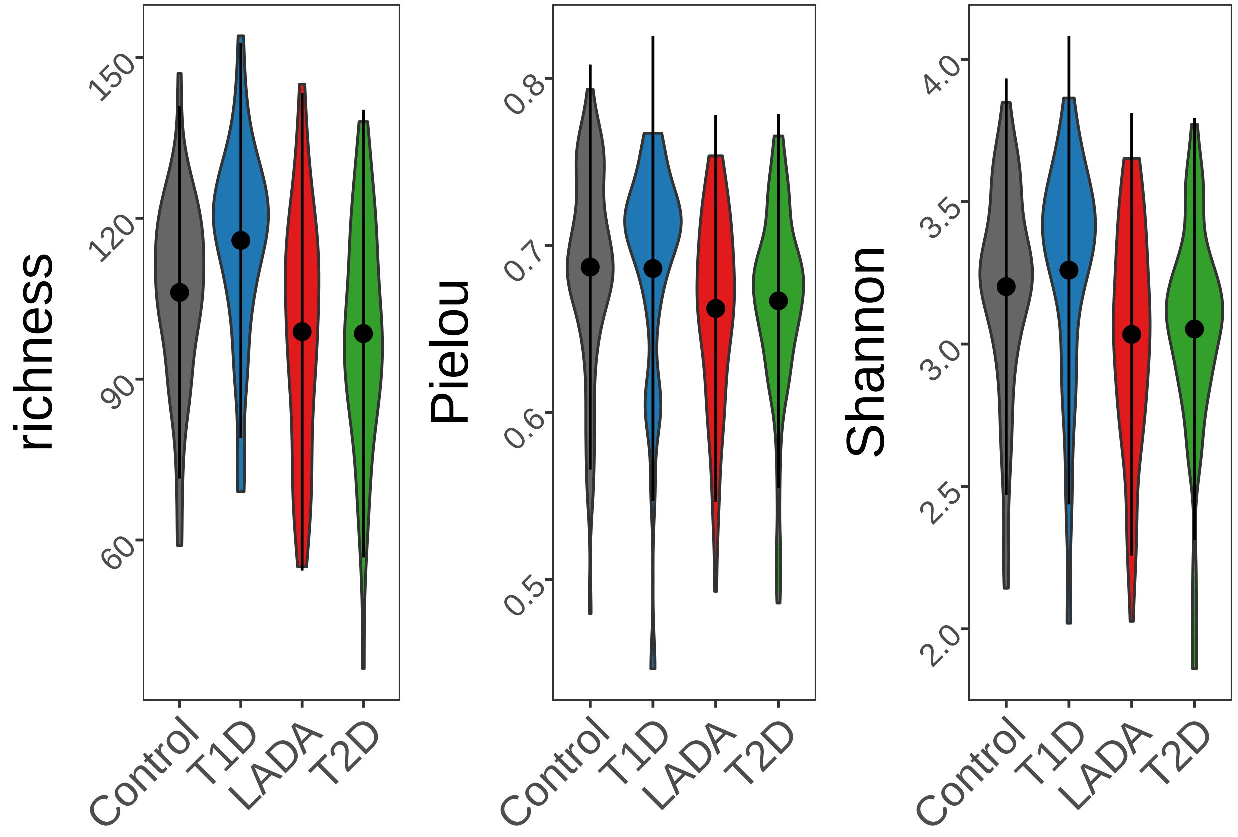


```r
#Kruskal test
kruskal.test(richness ~ Diagnosis, data=Metadata2)
```

```
##
##  Kruskal-Wallis rank sum test
##
## data:  richness by Diagnosis
## Kruskal-Wallis chi-squared = 19.779, df = 3, p-value = 0.0001886
```

```r
kruskal.test(Pielou ~ Diagnosis, data=Metadata2)
```

```
##
```

```
##  Kruskal-Wallis rank sum test
##
## data:  Pielou by Diagnosis
## Kruskal-Wallis chi-squared = 12.261, df = 3, p-value = 0.006541
```

```
kruskal.test(Shannon ~ Diagnosis, data=Metadata2)
```

```
##
##  Kruskal-Wallis rank sum test
##
## data:  Shannon by Diagnosis
## Kruskal-Wallis chi-squared = 16.588, df = 3, p-value = 0.000859
```

```
#If significant Mann-Whitney pairwise post-test
pairwise.wilcox.test(Metadata2$richness, Metadata2$Diagnosis,
                     p.adjust.method="bonferroni")
```

```
##
##  Pairwise comparisons using Wilcoxon rank sum test with continuity correction
##
## data:  Metadata2$richness and Metadata2$Diagnosis
##
##      Control T1D    LADA
## T1D  0.0345  -      -
## LADA 0.3275  0.0024 -
## T2D  0.1161  0.0010 1.0000
##
## P value adjustment method: bonferroni
```

```
pairwise.wilcox.test(Metadata2$Pielou, Metadata2$Diagnosis,
                     p.adjust.method="bonferroni")
```

```
##
##  Pairwise comparisons using Wilcoxon rank sum test with continuity correction
##
## data:  Metadata2$Pielou and Metadata2$Diagnosis
##
##      Control T1D   LADA
## T1D  1.000   -     -
## LADA 0.065   0.085 -
## T2D  0.091   0.127 1.000
##
## P value adjustment method: bonferroni
```

```
pairwise.wilcox.test(Metadata2$Shannon, Metadata2$Diagnosis,
                     p.adjust.method="bonferroni")
```

```
##
##  Pairwise comparisons using Wilcoxon rank sum test with continuity correction
##
## data:  Metadata2$Shannon and Metadata2$Diagnosis
```

```
##
##      Control T1D   LADA
## T1D  1.000   -     -
## LADA 0.066   0.030 -
## T2D  0.019   0.018 1.000
##
## P value adjustment method: bonferroni
```

Alpha diversity remove metformin

```
rm(list=setdiff(ls(), c("Metadata", "MetadataMed", "Feature", "TaxonomyDA", "Taxonomy"
                        , "Fig1List")))
##Removing treated with metformin
#Metadata$Metformin <- ifelse(is.na(Metadata$Metformin), "Unknown", Metadata$Metformin)
Metadata2<-filter(Metadata, !Metformin == 1)
##Applying subsetting to OTU tables
Taxonomy2<-dplyr::select(Taxonomy, one_of(as.character(Metadata2$MicrobiomeID)))
##Remove orgs that are not present after subsetting.
Taxonomy2 <- Taxonomy2[rowSums(Taxonomy2)>0,]


#Calculate alpha diversity stats
#Use vegan to calculate various diversity and richness indices for each sample
set.seed(1)
diversityCalc <- data.frame(Shannon=diversity(t(Taxonomy2), index="shannon"),
                            Simpson=diversity(t(Taxonomy2), index="simpson"),
                            invSimpson=diversity(t(Taxonomy2), index="invsimpson"),
                            fisher=fisher.alpha(t(Taxonomy2)),
                            richness=specnumber(t(Taxonomy2)),
                            rarefy_min_count=rarefy(t(Taxonomy2),
                                                    sample=min(rowSums(t(Taxonomy2)))),
                            chao1=estimateR(t(Taxonomy2))["S.chao1",],
                            chao1SE=estimateR(t(Taxonomy2))["se.chao1",],
                            ShannonRar=diversity(rrarefy(data.frame(t(Taxonomy2)),
                                                         min(rowSums(t(Taxonomy2)))),
                                                 index="shannon"),
                            SimpsonRar=diversity(rrarefy(data.frame(t(Taxonomy2)),
                                                         min(rowSums(t(Taxonomy2)))),
                                                 index="simpson"),
                            invSimpsonRar=diversity(rrarefy(data.frame(t(Taxonomy2)),
                                                            min(rowSums(t(Taxonomy2)))),
                                                    index="invsimpson"),
                            Pielou=diversity(t(Taxonomy2))/log(specnumber(t(Taxonomy2))))
#Merge with metadata.
diversityCalc<-add_rownames(diversityCalc, "MicrobiomeID")
Metadata2 <- merge(Metadata2, diversityCalc, by="MicrobiomeID")

#Create a list to hold the plot objects.
Fig1ListRemMet <- list()
#Create vector to loop
AlphaDiv<-c("fisher", "Shannon", "Simpson", "invSimpson", "richness", "chao1",
            "ShannonRar", "SimpsonRar", "invSimpsonRar", "Pielou")
```

```r
#Plot
for (i in AlphaDiv) {
#Create plot name
pltName <- paste('Alpha', i, sep = '')
#create boxplots
Fig1ListRemMet[[ pltName ]]<-
ggplot(Metadata2, aes_string(x="Diagnosis", y=i, group="Diagnosis")) +
    geom_violin(aes(fill=Diagnosis, trim=FALSE)) +
    stat_summary(fun.data="mean_sdl",
                 mult=1, #mean plus minus a constant (mult=1) times the st.dev
                 geom="pointrange",
                 width=0.2 ) +
    #stat_summary(fun.y = mean, geom = "point") +
    #facet_grid(. ~ Metformin, scales="fixed") + #Scales can also be "free"
    #ggtitle(paste("Genus", i, sep=" ")) +
    #xlab("Diagnosis") +
    ylab(paste(i)) +
    scale_fill_manual(values=c(Control = "#666666", T1D = "#1F78B4",
                               T2D = "#33A02C", LADA = "#E31A1C")) +
    theme_bw() +
    theme(legend.position="none", panel.grid.major = element_blank(),
          panel.grid.minor = element_blank(), axis.title=element_text(size=20),
          axis.title.x = element_blank(),
          axis.text.x = element_text(angle = 45, hjust = 1, size=16),
          axis.text.y = element_text(angle = 45, hjust = 1, size=12))
}


#Have the plots stored in lists
lay <- rbind(c(1,2,3))
pdf(paste("MicroLADA_Alpha_RemMet", ".pdf", sep=""), width=12, height=6)
grid.arrange(Fig1ListRemMet$Alpharichness,
             Fig1ListRemMet$AlphaPielou,
             Fig1ListRemMet$AlphaShannon, layout_matrix = lay)
dev.off()
```

```
## pdf
##   2
```

```r
grid.arrange(Fig1ListRemMet$Alpharichness,
             Fig1ListRemMet$AlphaPielou,
             Fig1ListRemMet$AlphaShannon, layout_matrix = lay)
```

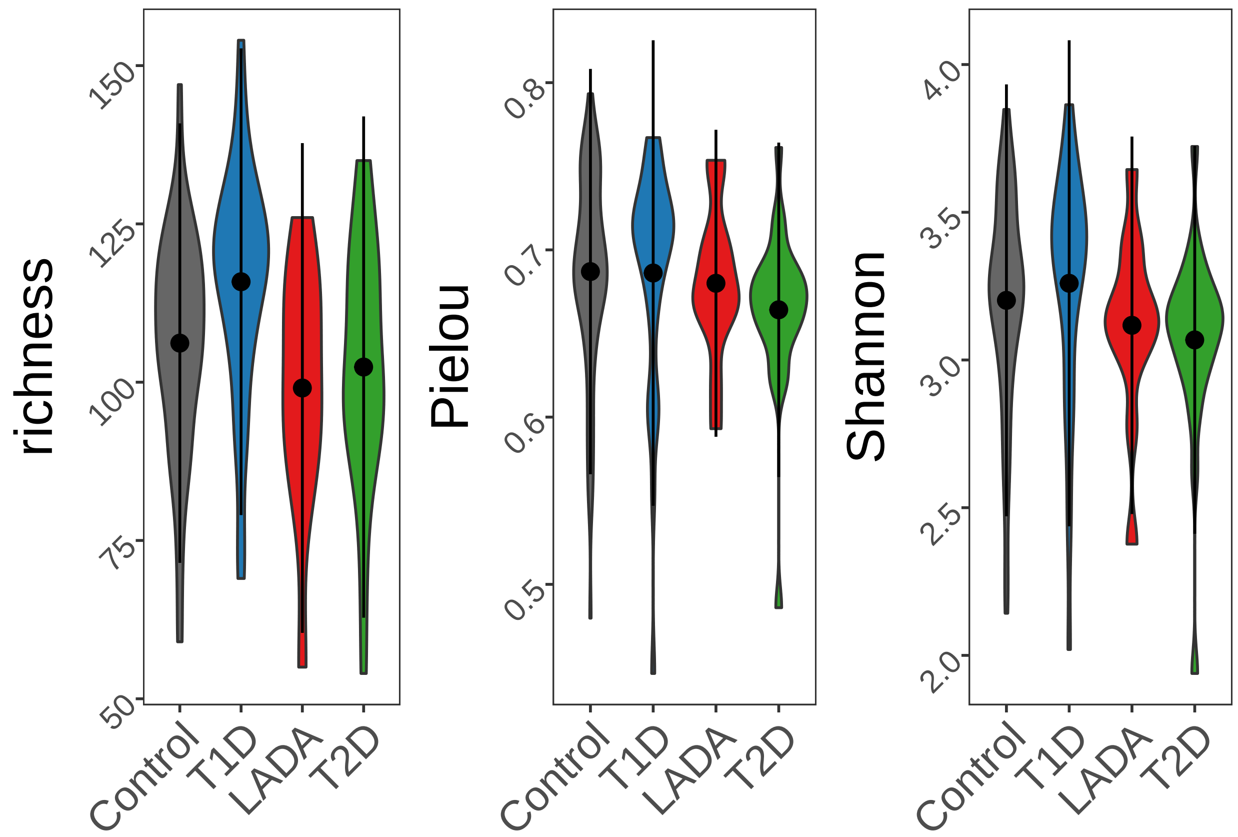


```
#Kruskal test
kruskal.test(richness ~ Diagnosis, data=Metadata2)
```

```
##
##  Kruskal-Wallis rank sum test
##
## data:  richness by Diagnosis
## Kruskal-Wallis chi-squared = 11.736, df = 3, p-value = 0.008344
```

```
kruskal.test(Pielou ~ Diagnosis, data=Metadata2)
```

```
##
##  Kruskal-Wallis rank sum test
##
## data:  Pielou by Diagnosis
## Kruskal-Wallis chi-squared = 7.2492, df = 3, p-value = 0.06437
```

```
kruskal.test(Shannon ~ Diagnosis, data=Metadata2)
```

```
##
##  Kruskal-Wallis rank sum test
##
## data:  Shannon by Diagnosis
## Kruskal-Wallis chi-squared = 9.3625, df = 3, p-value = 0.02484
```

```r
#If significant Mann-Whitney pairwise post-test
pairwise.wilcox.test(Metadata2$richness, Metadata2$Diagnosis,
                     p.adjust.method="bonferroni")
```

```
##
##  Pairwise comparisons using Wilcoxon rank sum test with continuity correction
##
## data:  Metadata2$richness and Metadata2$Diagnosis
##
##      Control T1D   LADA
## T1D  0.034   -     -
## LADA 1.000   0.057 -
## T2D  1.000   0.079 1.000
##
## P value adjustment method: bonferroni
```

```r
pairwise.wilcox.test(Metadata2$Pielou, Metadata2$Diagnosis,
                     p.adjust.method="bonferroni")
```

```
##
##  Pairwise comparisons using Wilcoxon rank sum test with continuity correction
##
## data:  Metadata2$Pielou and Metadata2$Diagnosis
##
##      Control T1D   LADA
## T1D  1.000   -     -
## LADA 1.000   1.000 -
## T2D  0.147   0.094 1.000
##
## P value adjustment method: bonferroni
```

```r
pairwise.wilcox.test(Metadata2$Shannon, Metadata2$Diagnosis,
                     p.adjust.method="bonferroni")
```

```
##
##  Pairwise comparisons using Wilcoxon rank sum test with continuity correction
##
## data:  Metadata2$Shannon and Metadata2$Diagnosis
##
##      Control T1D   LADA
## T1D  1.000   -     -
## LADA 1.000   0.433 -
## T2D  0.169   0.037 1.000
##
## P value adjustment method: bonferroni
```

Violin plots Dissimilarities

Part of figure 1

```r
rm(list=setdiff(ls(), c("Metadata", "MetadataMed", "Feature", "TaxonomyDA", "Taxonomy",
                        "Fig1List", "Fig1ListRemMet")))

##Create long format of dissimilarities
#Multi dimensional scaling
distmatrix <- vegdist(decostand(t(Taxonomy), method="hellinger"), method="bray")

#Contains all values except the ones equal to 0
meltPwBC<-subset(melt(as.matrix(distmatrix)))
rm(distmatrix) #Have comparisons of both 1vs2 and 2vs1 ..., but are removed during the
               #following filtration.
               #The same applies to comparisons of the same sample with itself


LADA_V <- subset(Metadata, Diagnosis=="LADA") %>% dplyr::select(one_of("MicrobiomeID")) %>%
  pull(MicrobiomeID) #%>% droplevels()
T1D_V <- subset(Metadata, Diagnosis=="T1D") %>% dplyr::select(one_of("MicrobiomeID")) %>%
  pull(MicrobiomeID) #%>% droplevels()
T2D_V <- subset(Metadata, Diagnosis=="T2D") %>% dplyr::select(one_of("MicrobiomeID")) %>%
  pull(MicrobiomeID) #%>% droplevels()
Control_V <- subset(Metadata, Diagnosis=="Control") %>% dplyr::select(one_of("MicrobiomeID")) %>%
  pull(MicrobiomeID) #%>% droplevels()

meltPwBC$Compare <- ifelse (meltPwBC$Var1  %in%  LADA_V & meltPwBC$Var2  %in%  T1D_V,
                            "LADA vs T1D",
                    ifelse (meltPwBC$Var1  %in%  LADA_V & meltPwBC$Var2  %in%  T2D_V,
                            "LADA vs T2D",
                    ifelse (meltPwBC$Var1  %in%  LADA_V & meltPwBC$Var2  %in%  Control_V,
                            "LADA vs Control",
                    ifelse (meltPwBC$Var1  %in%  T1D_V & meltPwBC$Var2  %in%  T2D_V,
                            "T1D vs T2D",
                    ifelse (meltPwBC$Var1  %in%  T1D_V & meltPwBC$Var2  %in%  Control_V,
                            "T1D vs Control",
                    ifelse (meltPwBC$Var1  %in%  T2D_V & meltPwBC$Var2  %in%  Control_V,
                            "T2D vs Control", "hmmmmmm"))))))
##Control validation
#table((meltPwBC$Var1  %in%  LADA_V) & (meltPwBC$Var2  %in%  T1D_V))
#table(meltPwBC$Compare)

meltPwBC<-subset(meltPwBC, meltPwBC$Compare!="hmmmmmm")

#Defining the order, can change if needed
meltPwBC$Compare<-factor(meltPwBC$Compare,
                         levels= c("LADA vs T1D", "LADA vs T2D", "LADA vs Control",
                                   "T1D vs T2D", "T1D vs Control", "T2D vs Control"))

violinDissi<-ggplot(meltPwBC, aes(x=Compare, y=value)) +
  geom_violin(trim=FALSE, fill="purple", colour="purple") +
  stat_summary(fun.data="mean_sdl", mult=1, #mean ± a constant (mult=1) times the st.dev
               geom="pointrange", width=0.2 ) +
  #geom_dotplot(binaxis='y', stackdir='center', dotsize=0.01) +
  labs(x="", y="Dissimilarity") +
  theme_bw() +
```

```
  theme(legend.position="none", panel.grid.major = element_blank(),
        panel.grid.minor = element_blank())

violinDissi
```

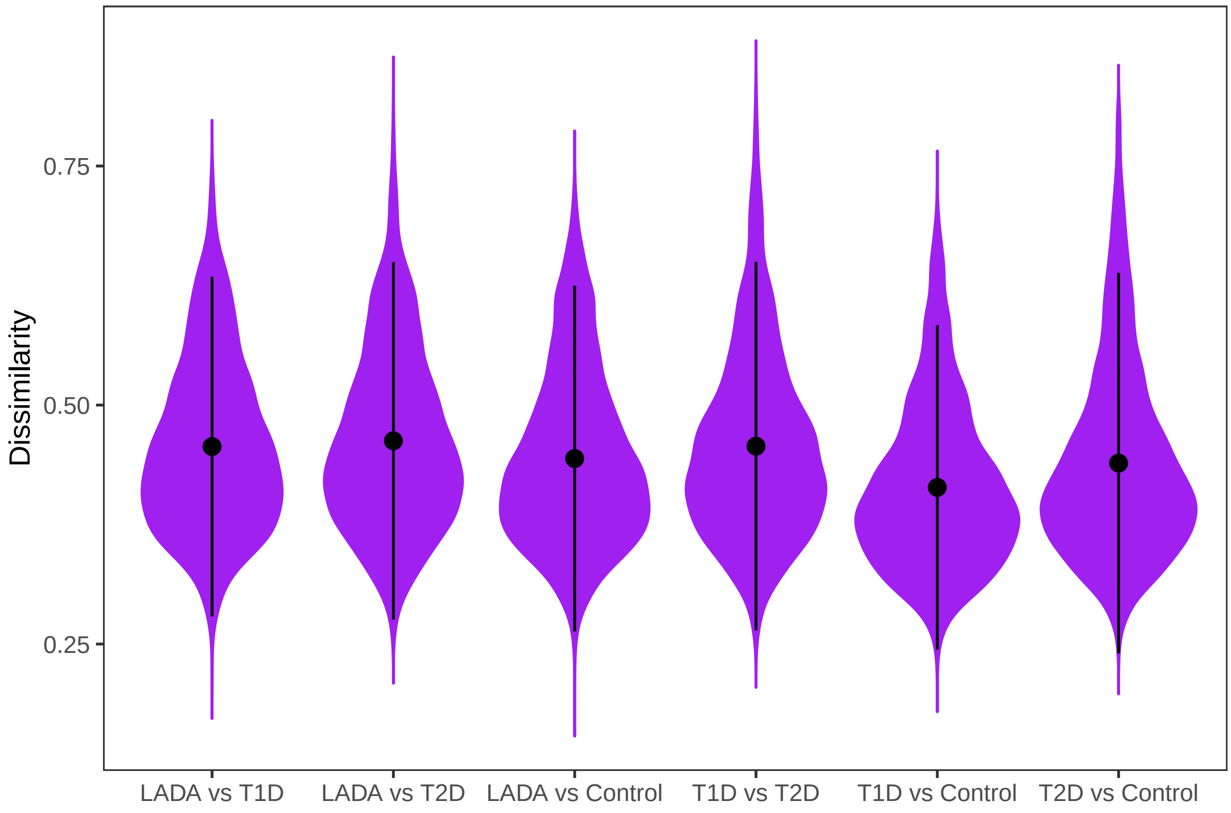


```
pdf(paste("MicroLADA_ViolinDissi", ".pdf", sep=""), width=12, height=6)
violinDissi
dev.off()
```

```
## pdf
##   2
```

```
#Kruskal test
kruskal.test(value ~ Compare, data=meltPwBC)
```

```
##
##  Kruskal-Wallis rank sum test
##
## data:  value by Compare
## Kruskal-Wallis chi-squared = 540.07, df = 5, p-value < 2.2e-16
```

```
#If significant Mann-Whitney pairwise post-test
pairwise.wilcox.test(meltPwBC$value, meltPwBC$Compare, p.adjust.method="bonferroni")
```

```
##
##  Pairwise comparisons using Wilcoxon rank sum test with continuity correction
##
## data:  meltPwBC$value and meltPwBC$Compare
##
##                  LADA vs T1D LADA vs T2D LADA vs Control T1D vs T2D
## LADA vs T2D      0.620       -           -               -
## LADA vs Control  7.2e-07     < 2e-16     -               -
## T1D vs T2D       1.000       0.058       1.5e-05         -
## T1D vs Control   < 2e-16     < 2e-16     < 2e-16         < 2e-16
## T2D vs Control   < 2e-16     < 2e-16     6.1e-05         < 2e-16
##                  T1D vs Control
## LADA vs T2D      -
## LADA vs Control  -
## T1D vs T2D       -
## T1D vs Control   -
## T2D vs Control   < 2e-16
##
## P value adjustment method: bonferroni
```

```
######################################################################
##Create long format of dissimilarities
#Multi dimensional scaling
distmatrix <- vegdist(decostand(t(Taxonomy), method="hellinger"), method="bray")



#Contains all values except the ones equal to 0
meltPwBC<-subset(melt(as.matrix(distmatrix)), value!=0)
#meltPwBC<-subset(melt(as.matrix(distmatrix)))
rm(distmatrix) #Have comparisons of both 1vs2 and 2vs1 ..., but are removed during the
               #following filtration.
               #The same applies to comparisons of the same sample with itself


LADA_V <- subset(Metadata, Diagnosis=="LADA") %>% dplyr::select(one_of("MicrobiomeID")) %>%
  pull(MicrobiomeID) #%>% droplevels()
T1D_V <- subset(Metadata, Diagnosis=="T1D") %>% dplyr::select(one_of("MicrobiomeID")) %>%
  pull(MicrobiomeID) #%>% droplevels()
T2D_V <- subset(Metadata, Diagnosis=="T2D") %>% dplyr::select(one_of("MicrobiomeID")) %>%
  pull(MicrobiomeID) #%>% droplevels()
Control_V <- subset(Metadata, Diagnosis=="Control") %>% dplyr::select(one_of("MicrobiomeID")) %>%
  pull(MicrobiomeID) #%>% droplevels()

meltPwBC$Compare <- ifelse (meltPwBC$Var1  %in%  LADA_V & meltPwBC$Var2  %in%
                              LADA_V, "LADA",
                    ifelse (meltPwBC$Var1  %in%  T1D_V & meltPwBC$Var2  %in%
                              T1D_V, "T1D",
                    ifelse (meltPwBC$Var1  %in%  T2D_V & meltPwBC$Var2  %in%
                              T2D_V, "T2D",
                    ifelse (meltPwBC$Var1  %in%  Control_V & meltPwBC$Var2  %in%
                              Control_V, "Control",
                    ifelse (meltPwBC$Var1  %in%  LADA_V & meltPwBC$Var2  %in%
                              T1D_V, "LADA vs T1D",
```

```
                    ifelse (meltPwBC$Var1  %in%  LADA_V & meltPwBC$Var2  %in%
                              T2D_V, "LADA vs T2D",
                    ifelse (meltPwBC$Var1  %in%  LADA_V & meltPwBC$Var2  %in%
                              Control_V, "LADA vs Control",
                    ifelse (meltPwBC$Var1  %in%  T1D_V & meltPwBC$Var2  %in%
                              T2D_V, "T1D vs T2D",
                    ifelse (meltPwBC$Var1  %in%  T1D_V & meltPwBC$Var2  %in%
                              Control_V, "T1D vs Control",
                    ifelse (meltPwBC$Var1  %in%  T2D_V & meltPwBC$Var2  %in%
                              Control_V, "T2D vs Control", "hmmmmmm"))))))))))
##Control validation
#table((meltPwBC$Var1  %in%  LADA_V) & (meltPwBC$Var2  %in%  T1D_V))
#table(meltPwBC$Compare)

meltPwBC<-subset(meltPwBC, meltPwBC$Compare!="hmmmmmm")

#Defining the order, can change if needed
meltPwBC$Compare<-factor(meltPwBC$Compare, levels= c("Control", "T1D",
                                                     "T2D", "LADA",
                                                     "LADA vs T1D", "LADA vs T2D",
                                                     "LADA vs Control", "T1D vs T2D",
                                                     "T1D vs Control", "T2D vs Control"))

violinDissi<-ggplot(meltPwBC, aes(x=Compare, y=value)) +
  geom_violin(trim=FALSE, fill="purple", colour="purple") +
  stat_summary(fun.data="mean_sdl", mult=1, #mean ± a constant (mult=1) times the st.dev
               geom="pointrange", width=0.2 ) +
  #geom_dotplot(binaxis='y', stackdir='center', dotsize=0.01) +
  labs(x="", y="Dissimilarity") +
  theme_bw() +
  theme(legend.position="none", panel.grid.major = element_blank(),
        panel.grid.minor = element_blank(),
        axis.text.x = element_text(angle = 45, hjust = 1))

violinDissi
```

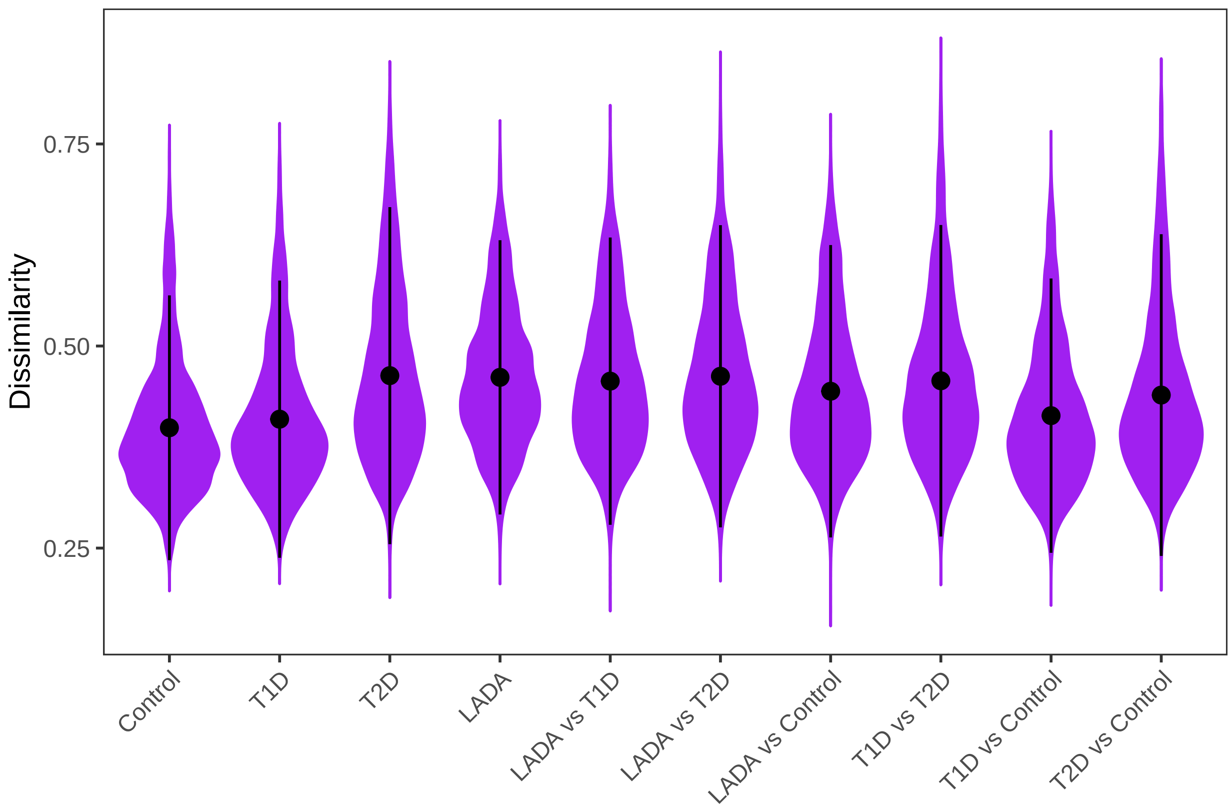


```
pdf(paste("MicroLADA_ViolinDissiAllComp", ".pdf", sep=""), width=12, height=6)
violinDissi
dev.off()
```

```
## pdf
##   2
```

```
#Kruskal test
kruskal.test(value ~ Compare, data=meltPwBC)
```

```
##
##  Kruskal-Wallis rank sum test
##
## data:  value by Compare
## Kruskal-Wallis chi-squared = 2259.9, df = 9, p-value < 2.2e-16
```

```
#If significant Mann-Whitney pairwise post-test
pairwise.wilcox.test(meltPwBC$value, meltPwBC$Compare, p.adjust.method="bonferroni")
```

```
##
##  Pairwise comparisons using Wilcoxon rank sum test with continuity correction
##
## data:  meltPwBC$value and meltPwBC$Compare
##
```

```
##                  Control T1D     T2D     LADA    LADA vs T1D LADA vs T2D
## T1D              0.03991 -       -       -       -           -
## T2D              < 2e-16 < 2e-16 -       -       -           -
## LADA             < 2e-16 < 2e-16 1.00000 -       -           -
## LADA vs T1D      < 2e-16 < 2e-16 1.00000 0.47498 -           -
## LADA vs T2D      < 2e-16 < 2e-16 1.00000 1.00000 1.00000     -
## LADA vs Control  < 2e-16 < 2e-16 1.1e-12 < 2e-16 2.2e-06     < 2e-16
## T1D vs T2D       < 2e-16 < 2e-16 1.00000 0.02330 1.00000     0.17316
## T1D vs Control   1.8e-11 1.00000 < 2e-16 < 2e-16 < 2e-16     < 2e-16
## T2D vs Control   < 2e-16 9.2e-16 < 2e-16 < 2e-16 < 2e-16     < 2e-16
##                  LADA vs Control T1D vs T2D T1D vs Control
## T1D              -               -          -
## T2D              -               -          -
## LADA             -               -          -
## LADA vs T1D      -               -          -
## LADA vs T2D      -               -          -
## LADA vs Control  -               -          -
## T1D vs T2D       4.4e-05         -          -
## T1D vs Control   < 2e-16         < 2e-16    -
## T2D vs Control   0.00018         3.8e-16    < 2e-16
##
## P value adjustment method: bonferroni
```

```r
#####################################################################
meltPwBC<-filter(meltPwBC,
                 Compare == "Control" | Compare == "T1D" |
                   Compare == "T2D" | Compare == "LADA")

meltPwBC$Compare<-ordered(meltPwBC$Compare,
                            levels=c("Control", "T1D", "LADA", "T2D"))

violinDissi<-ggplot(meltPwBC, aes(x=Compare, y=value)) +
    geom_violin(aes(fill=Compare, trim=FALSE)) +
    stat_summary(fun.data="mean_sdl", mult=1, #mean ± a constant (mult=1) times the st.dev
                 geom="pointrange", width=0.2 ) +
    labs(y="Dissimilarity") +
    scale_fill_manual(values=c(Control = "#666666", T1D = "#1F78B4",
                               T2D = "#33A02C", LADA = "#E31A1C")) +
    theme_bw() +
    theme(legend.position="none", panel.grid.major = element_blank(),
          panel.grid.minor = element_blank(), axis.title=element_text(size=20),
          axis.title.x = element_blank(),
          axis.text.x = element_text(angle = 45, hjust = 1, size=16),
          axis.text.y = element_text(angle = 45, hjust = 1, size=12))


violinDissi
```

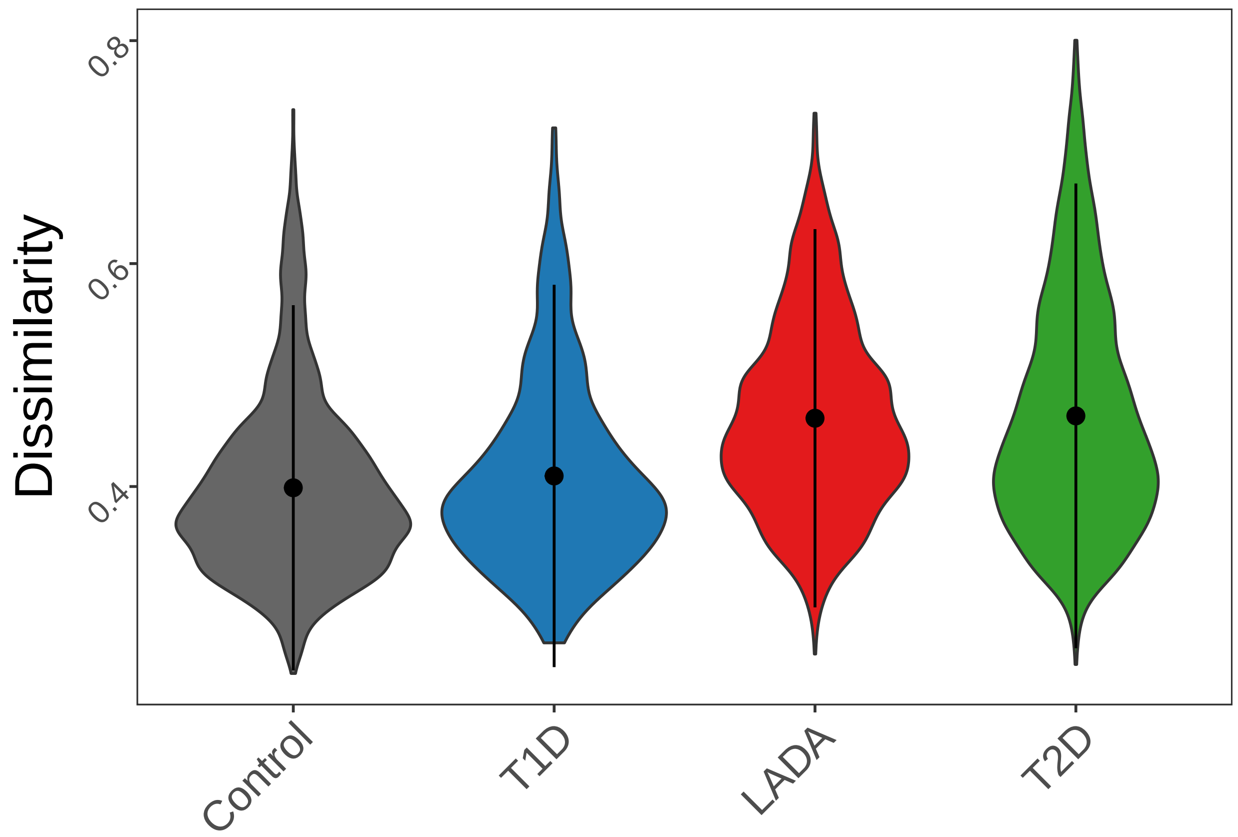


```
pdf(paste("MicroLADA_ViolinDissiCompWithin", ".pdf", sep=""), width=12, height=6)
violinDissi
dev.off()
```

```
## pdf
##   2
```

```
#Save plot i previous list
Fig1List[[ "DissiBrayHel" ]] <- violinDissi

#Kruskal test
kruskal.test(value ~ Compare, data=meltPwBC)
```

```
##
##  Kruskal-Wallis rank sum test
##
## data:  value by Compare
## Kruskal-Wallis chi-squared = 1601.5, df = 3, p-value < 2.2e-16
```

```
#If significant Mann-Whitney pairwise post-test
pairwise.wilcox.test(meltPwBC$value, meltPwBC$Compare, p.adjust.method="bonferroni")
```

```
##
##  Pairwise comparisons using Wilcoxon rank sum test with continuity correction
```

```
##
## data:  meltPwBC$value and meltPwBC$Compare
##
##      Control T1D    LADA
## T1D  0.0053  -      -
## LADA <2e-16  <2e-16 -
## T2D  <2e-16  <2e-16 0.1564
##
## P value adjustment method: bonferroni
```

Violin plots Dissimilarities remove metformin

```
rm(list=setdiff(ls(), c("Metadata", "MetadataMed", "Feature", "TaxonomyDA", "Taxonomy",
                        "Fig1List", "Fig1ListRemMet")))
##Removing treated with metformin
#Metadata$Metformin <- ifelse(is.na(Metadata$Metformin), "Unknown", Metadata$Metformin)
Metadata2<-filter(Metadata, !Metformin == 1)
##Applying subsetting to OTU tables
Taxonomy2<-dplyr::select(Taxonomy, one_of(as.character(Metadata2$MicrobiomeID)))
##Remove orgs that are not present after subsetting.
Taxonomy2 <- Taxonomy2[rowSums(Taxonomy2)>0,]




##Create long format of dissimilarities
#Multi dimensional scaling
distmatrix <- vegdist(decostand(t(Taxonomy2), method="hellinger"), method="bray")

#Contains all values except the ones equal to 0
meltPwBC<-subset(melt(as.matrix(distmatrix)))
rm(distmatrix) #Have comparisons of both 1vs2 and 2vs1 ..., but are removed during the
               #following filtration.
               #The same applies to comparisons of the same sample with itself


LADA_V <- subset(Metadata2, Diagnosis=="LADA") %>% dplyr::select(one_of("MicrobiomeID")) %>%
  pull(MicrobiomeID) #%>% droplevels()
T1D_V <- subset(Metadata2, Diagnosis=="T1D") %>% dplyr::select(one_of("MicrobiomeID")) %>%
  pull(MicrobiomeID) #%>% droplevels()
T2D_V <- subset(Metadata2, Diagnosis=="T2D") %>% dplyr::select(one_of("MicrobiomeID")) %>%
  pull(MicrobiomeID) #%>% droplevels()
Control_V <- subset(Metadata2, Diagnosis=="Control") %>% dplyr::select(one_of("MicrobiomeID")) %>%
  pull(MicrobiomeID) #%>% droplevels()

meltPwBC$Compare <- ifelse (meltPwBC$Var1  %in%  LADA_V & meltPwBC$Var2  %in%  T1D_V,
                            "LADA vs T1D",
                    ifelse (meltPwBC$Var1  %in%  LADA_V & meltPwBC$Var2  %in%  T2D_V,
                            "LADA vs T2D",
                    ifelse (meltPwBC$Var1  %in%  LADA_V & meltPwBC$Var2  %in%  Control_V,
                            "LADA vs Control",
                    ifelse (meltPwBC$Var1  %in%  T1D_V & meltPwBC$Var2  %in%  T2D_V,
                            "T1D vs T2D",
```

```r
                    ifelse (meltPwBC$Var1  %in%  T1D_V & meltPwBC$Var2  %in%  Control_V,
                            "T1D vs Control",
                    ifelse (meltPwBC$Var1  %in%  T2D_V & meltPwBC$Var2  %in%  Control_V,
                            "T2D vs Control", "hmmmmmm"))))))
##Control validation
#table((meltPwBC$Var1  %in%  LADA_V) & (meltPwBC$Var2  %in%  T1D_V))
#table(meltPwBC$Compare)

meltPwBC<-subset(meltPwBC, meltPwBC$Compare!="hmmmmmm")

#Defining the order, can change if needed
meltPwBC$Compare<-factor(meltPwBC$Compare,
                         levels= c("LADA vs T1D", "LADA vs T2D", "LADA vs Control",
                                   "T1D vs T2D", "T1D vs Control", "T2D vs Control"))

violinDissi<-ggplot(meltPwBC, aes(x=Compare, y=value)) +
  geom_violin(trim=FALSE, fill="purple", colour="purple") +
  stat_summary(fun.data="mean_sdl", mult=1, #mean ± a constant (mult=1) times the st.dev
               geom="pointrange", width=0.2 ) +
  #geom_dotplot(binaxis='y', stackdir='center', dotsize=0.01) +
  labs(x="", y="Dissimilarity") +
  theme_bw() +
  theme(legend.position="none", panel.grid.major = element_blank(),
        panel.grid.minor = element_blank())

violinDissi
```

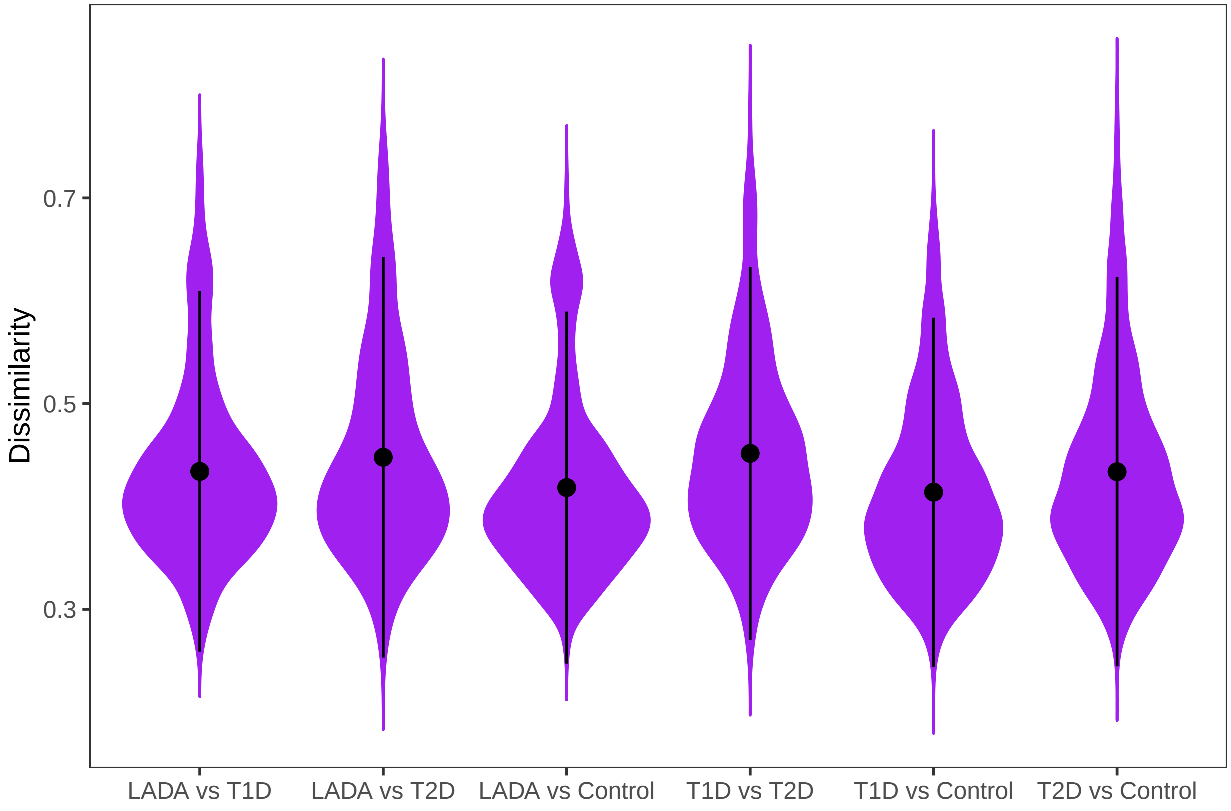


```
pdf(paste("MicroLADA_ViolinDissiRemMet", ".pdf", sep=""), width=12, height=6)
violinDissi
dev.off()
```

```
## pdf
##   2
```

```
#Kruskal test
kruskal.test(value ~ Compare, data=meltPwBC)
```

```
##
##  Kruskal-Wallis rank sum test
##
## data:  value by Compare
## Kruskal-Wallis chi-squared = 141.97, df = 5, p-value < 2.2e-16
```

```
#If significant Mann-Whitney pairwise post-test
pairwise.wilcox.test(meltPwBC$value, meltPwBC$Compare, p.adjust.method="bonferroni")
```

```
##
##  Pairwise comparisons using Wilcoxon rank sum test with continuity correction
##
## data:  meltPwBC$value and meltPwBC$Compare
##
```

```
##                  LADA vs T1D LADA vs T2D LADA vs Control T1D vs T2D
## LADA vs T2D     1.00000     -           -               -
## LADA vs Control 0.00475     3.9e-05     -               -
## T1D vs T2D      0.00693     1.00000     5.8e-16         -
## T1D vs Control  0.00018     4.7e-07     1.00000         < 2e-16
## T2D vs Control  1.00000     0.43232     0.00046         3.4e-06
##                 T1D vs Control
## LADA vs T2D     -
## LADA vs Control -
## T1D vs T2D      -
## T1D vs Control  -
## T2D vs Control  4.9e-09
##
## P value adjustment method: bonferroni
```

```
########################################################################
##Create long format of dissimilarities
#Multi dimensional scaling
distmatrix <- vegdist(decostand(t(Taxonomy2), method="hellinger"), method="bray")



#Contains all values except the ones equal to 0
meltPwBC<-subset(melt(as.matrix(distmatrix)), value!=0)
#meltPwBC<-subset(melt(as.matrix(distmatrix)))
rm(distmatrix) #Have comparisons of both 1vs2 and 2vs1 ..., but are removed during the
               #following filtration.
               #The same applies to comparisons of the same sample with itself


LADA_V <- subset(Metadata2, Diagnosis=="LADA") %>% dplyr::select(one_of("MicrobiomeID")) %>%
  pull(MicrobiomeID) #%>% droplevels()
T1D_V <- subset(Metadata2, Diagnosis=="T1D") %>% dplyr::select(one_of("MicrobiomeID")) %>%
  pull(MicrobiomeID) #%>% droplevels()
T2D_V <- subset(Metadata2, Diagnosis=="T2D") %>% dplyr::select(one_of("MicrobiomeID")) %>%
  pull(MicrobiomeID) #%>% droplevels()
Control_V <- subset(Metadata2, Diagnosis=="Control") %>% dplyr::select(one_of("MicrobiomeID")) %>%
  pull(MicrobiomeID) #%>% droplevels()

meltPwBC$Compare <- ifelse (meltPwBC$Var1  %in%  LADA_V & meltPwBC$Var2  %in%
                              LADA_V, "LADA",
                    ifelse (meltPwBC$Var1  %in%  T1D_V & meltPwBC$Var2  %in%
                              T1D_V, "T1D",
                    ifelse (meltPwBC$Var1  %in%  T2D_V & meltPwBC$Var2  %in%
                              T2D_V, "T2D",
                    ifelse (meltPwBC$Var1  %in%  Control_V & meltPwBC$Var2  %in%
                              Control_V, "Control",
                    ifelse (meltPwBC$Var1  %in%  LADA_V & meltPwBC$Var2  %in%
                              T1D_V, "LADA vs T1D",
                    ifelse (meltPwBC$Var1  %in%  LADA_V & meltPwBC$Var2  %in%
                              T2D_V, "LADA vs T2D",
                    ifelse (meltPwBC$Var1  %in%  LADA_V & meltPwBC$Var2  %in%
                              Control_V, "LADA vs Control",
                    ifelse (meltPwBC$Var1  %in%  T1D_V & meltPwBC$Var2  %in%
```

```r
                              T2D_V, "T1D vs T2D",
                    ifelse (meltPwBC$Var1  %in%  T1D_V & meltPwBC$Var2  %in%
                              Control_V, "T1D vs Control",
                    ifelse (meltPwBC$Var1  %in%  T2D_V & meltPwBC$Var2  %in%
                              Control_V, "T2D vs Control", "hmmmmmm"))))))))))
##Control validation
#table((meltPwBC$Var1  %in%  LADA_V) & (meltPwBC$Var2  %in%  T1D_V))
#table(meltPwBC$Compare)

meltPwBC<-subset(meltPwBC, meltPwBC$Compare!="hmmmmmm")

#Defining the order, can change if needed
meltPwBC$Compare<-factor(meltPwBC$Compare, levels= c("Control", "T1D",
                                                     "T2D", "LADA",
                                                     "LADA vs T1D", "LADA vs T2D",
                                                     "LADA vs Control", "T1D vs T2D",
                                                     "T1D vs Control", "T2D vs Control"))

violinDissi<-ggplot(meltPwBC, aes(x=Compare, y=value)) +
  geom_violin(trim=FALSE, fill="purple", colour="purple") +
  stat_summary(fun.data="mean_sdl", mult=1, #mean ± a constant (mult=1) times the st.dev
               geom="pointrange", width=0.2 ) +
  #geom_dotplot(binaxis='y', stackdir='center', dotsize=0.01) +
  labs(x="", y="Dissimilarity") +
  theme_bw() +
  theme(legend.position="none", panel.grid.major = element_blank(),
        panel.grid.minor = element_blank(),
        axis.text.x = element_text(angle = 45, hjust = 1))

violinDissi
```

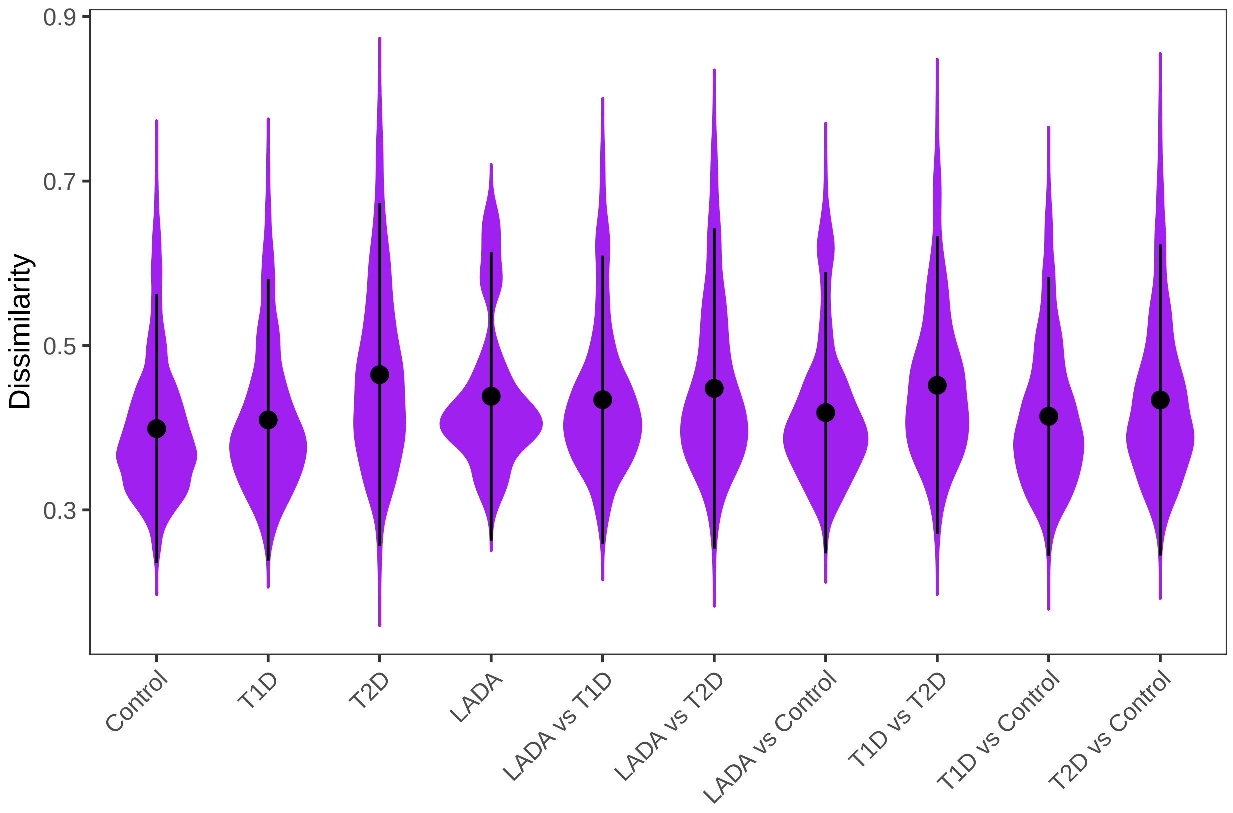


```
pdf(paste("MicroLADA_ViolinDissiAllCompRemMet", ".pdf", sep=""), width=12, height=6)
violinDissi
dev.off()
```

```
## pdf
##   2
```

```
#Kruskal test
kruskal.test(value ~ Compare, data=meltPwBC)
```

```
##
##  Kruskal-Wallis rank sum test
##
## data:  value by Compare
## Kruskal-Wallis chi-squared = 585.56, df = 9, p-value < 2.2e-16
```

```
#If significant Mann-Whitney pairwise post-test
pairwise.wilcox.test(meltPwBC$value, meltPwBC$Compare, p.adjust.method="bonferroni")
```

```
##
##  Pairwise comparisons using Wilcoxon rank sum test with continuity correction
##
## data:  meltPwBC$value and meltPwBC$Compare
##
```

```
##                  Control T1D     T2D     LADA    LADA vs T1D LADA vs T2D
## T1D              0.03991 -       -       -       -           -
## T2D              < 2e-16 < 2e-16 -       -       -           -
## LADA             8.1e-07 0.00267 0.20726 -       -           -
## LADA vs T1D      4.9e-15 7.8e-06 0.00039 1.00000 -           -
## LADA vs T2D      < 2e-16 4.6e-08 0.84965 1.00000 1.00000     -
## LADA vs Control  2.1e-09 0.57580 < 2e-16 0.19224 0.01425     0.00012
## T1D vs T2D       < 2e-16 < 2e-16 1.00000 1.00000 0.02078     1.00000
## T1D vs Control   1.8e-11 1.00000 < 2e-16 0.05540 0.00053     1.4e-06
## T2D vs Control   < 2e-16 2.3e-09 2.8e-08 1.00000 1.00000     1.00000
##                  LADA vs Control T1D vs T2D T1D vs Control
## T1D              -               -          -
## T2D              -               -          -
## LADA             -               -          -
## LADA vs T1D      -               -          -
## LADA vs T2D      -               -          -
## LADA vs Control  -               -          -
## T1D vs T2D       1.7e-15         -          -
## T1D vs Control   1.00000         < 2e-16    -
## T2D vs Control   0.00137         1.0e-05    1.5e-08
##
## P value adjustment method: bonferroni
```

```r
#####################################################################
meltPwBC<-filter(meltPwBC,
                 Compare == "Control" | Compare == "T1D" |
                   Compare == "T2D" | Compare == "LADA")

meltPwBC$Compare<-ordered(meltPwBC$Compare,
                            levels=c("Control", "T1D", "LADA", "T2D"))

violinDissi<-ggplot(meltPwBC, aes(x=Compare, y=value)) +
    geom_violin(aes(fill=Compare, trim=FALSE)) +
    stat_summary(fun.data="mean_sdl", mult=1, #mean ± a constant (mult=1) times the st.dev
                 geom="pointrange", width=0.2 ) +
    labs(y="Dissimilarity") +
    scale_fill_manual(values=c(Control = "#666666", T1D = "#1F78B4",
                               T2D = "#33A02C", LADA = "#E31A1C")) +
    theme_bw() +
    theme(legend.position="none", panel.grid.major = element_blank(),
          panel.grid.minor = element_blank(), axis.title=element_text(size=20),
          axis.title.x = element_blank(),
          axis.text.x = element_text(angle = 45, hjust = 1, size=16),
          axis.text.y = element_text(angle = 45, hjust = 1, size=12))


violinDissi
```

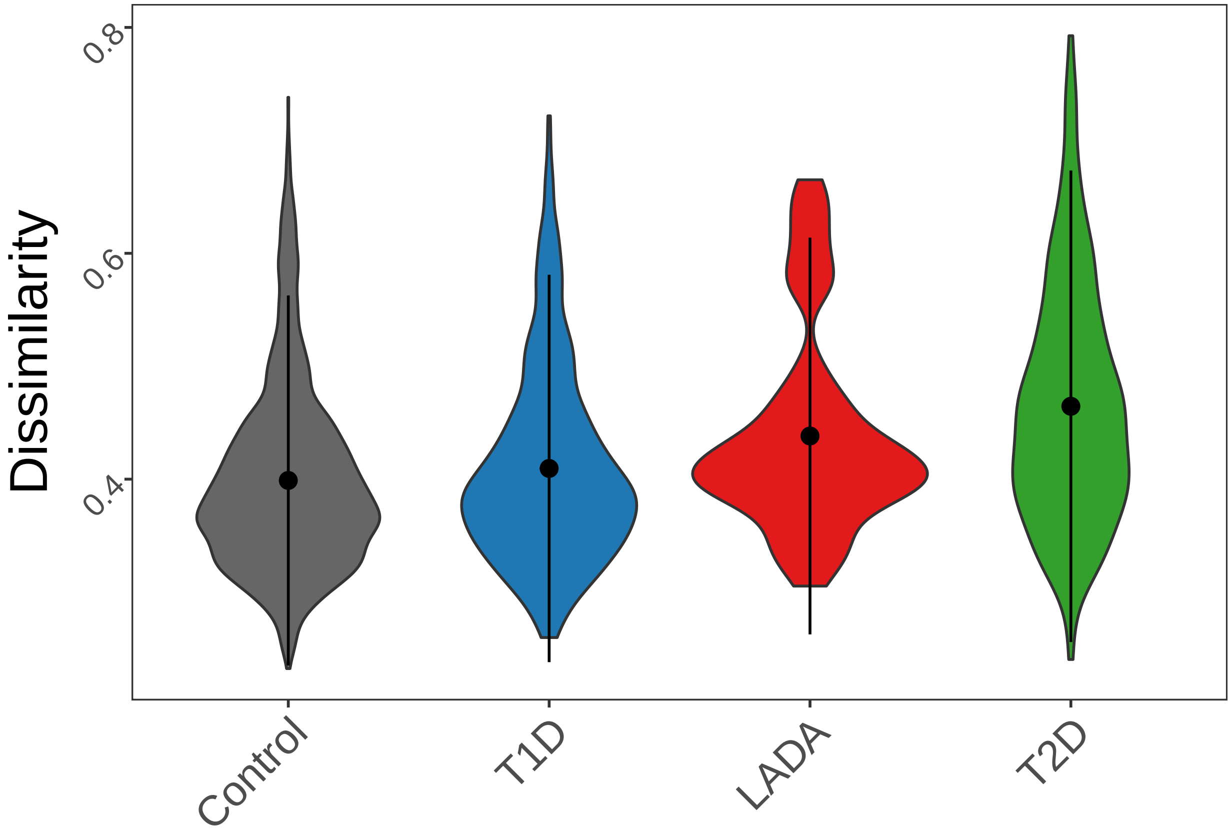


```
pdf(paste("MicroLADA_ViolinDissiCompWithinRemMet", ".pdf", sep=""), width=12, height=6)
violinDissi
dev.off()
```

```
## pdf
##   2
```

```
#Save plot in previous list
Fig1ListRemMet[[ "DissiBrayHel" ]] <- violinDissi

#Kruskal test
kruskal.test(value ~ Compare, data=meltPwBC)
```

```
##
##  Kruskal-Wallis rank sum test
##
## data:  value by Compare
## Kruskal-Wallis chi-squared = 238.56, df = 3, p-value < 2.2e-16
```

```
#If significant Mann-Whitney pairwise post-test
pairwise.wilcox.test(meltPwBC$value, meltPwBC$Compare, p.adjust.method="bonferroni")
```

```
##
##  Pairwise comparisons using Wilcoxon rank sum test with continuity correction
```

```
##
## data:  meltPwBC$value and meltPwBC$Compare
##
##      Control T1D     LADA
## T1D  0.00532 -       -
## LADA 1.1e-07 0.00036 -
## T2D  < 2e-16 < 2e-16 0.02763
##
## P value adjustment method: bonferroni
```

PCoA

Part of figure 1

```
rm(list=setdiff(ls(), c("Metadata", "MetadataMed", "Feature", "TaxonomyDA", "Taxonomy",
                        "Fig1List", "Fig1ListRemMet")))

Subset <- c("LADAControl", "LADAT1D", "LADAT2D", "All")
#i <- c("All")
#i <- c("LADAControl")
for (i in Subset) {

#Subsetting Metadata according to
if (i=="All") {
  print(i)
  Metadata2 <- Metadata
} else if (i=="LADAControl") {
  print(i)
  Metadata2<-filter(Metadata, Diagnosis == "LADA" | Diagnosis == "Control")
} else if (i=="LADAT1D") {
  print(i)
  Metadata2<-filter(Metadata, Diagnosis == "LADA" | Diagnosis == "T1D")
} else if (i=="LADAT2D") {
  print(i)
  Metadata2<-filter(Metadata, Diagnosis == "LADA" | Diagnosis == "T2D")
} else {
  print("Subset defined not valid")
}

#Apply subsetting to Taxonomy table
Taxonomy2<-dplyr::select(Taxonomy, one_of(as.character(Metadata2$MicrobiomeID)))


#Hellinger transformation
Taxonomy2 <- data.frame(t(decostand(t(Taxonomy2), method="hellinger")))
#Maks TSS
Taxonomy2<-sweep(Taxonomy2, 2, colSums(Taxonomy2), FUN="/")
#Dissimilarity
distmatrix <- vegdist(t(Taxonomy2), method="bray")
#Multi dimensional scaling with capscale
PCoAcsObject<-capscale(distmatrix~1)
```

```r
##Add eig to plot axes. with cmdscale there are negative values not with capscale
eig <- PCoAcsObject$CA$eig
# Calculate the variation explained by PCoA1, 2, 3 and 4
# and use it to generate axis labels
eig_1_2 <- eig[1:4] / sum(eig) * 100 #Vector with variance explained
#  by the first 4 axes
eig_1 <- paste("PCoA1", round(eig_1_2[1], digits = 2), "% variance")
eig_2 <- paste("PCoA2", round(eig_1_2[2], digits = 2), "% variance")
eig_3 <- paste("PCoA3", round(eig_1_2[3], digits = 2), "% variance")
eig_4 <- paste("PCoA4", round(eig_1_2[4], digits = 2), "% variance")

##Pull out coordinates for plotting from the ca object
#Structuring to add to Metadata2
PCoACA<-PCoAcsObject$CA #The ca object contains the actual ordination results:
#u ((Weighted) orthonormal site scores),
#v ((Weighted) orthonormal species scores) all na in mine (unconstrained),
#Xbar (The standardized data matrix after previous stages of analysis),
#and imaginary.u.eig ???.
#Info http://cc.oulu.fi/~jarioksa/softhelp/vegan/html/cca.object.html
PCoA<-as.data.frame(PCoACA$u)
#Change colnames. Now add dis and trans info to names
colnames(PCoA) <- c("MDS1BrayHel","MDS2BrayHel", "MDS3BrayHel","MDS4BrayHel")
#Add row names to df
PCoA$MicrobiomeID <- row.names(PCoA)
#Remove leading X
PCoA$MicrobiomeID <- gsub("X", "", PCoA$MicrobiomeID)
#Merge according to Sample
Metadata2<-merge(Metadata2, PCoA, by="MicrobiomeID")


#PCoA MDS1 and MDS2 pdf
pdf(paste("MicroLADA_PCoA", i, ".pdf", sep=""), width=9, height=6)
print(ggplot(Metadata2) +
  geom_point(aes(x=MDS1BrayHel, y=MDS2BrayHel, color = Diagnosis,
                 group = Diagnosis), size=3) +
  stat_ellipse(aes(MDS1BrayHel, y=MDS2BrayHel, color = Diagnosis,
                  group = Diagnosis)) +
  scale_color_manual(values=c(Control = "#666666", T1D = "#1F78B4",
                             T2D = "#33A02C", LADA = "#E31A1C")) +
  ggtitle(paste("PCoA", i, sep=" ")) +
  labs(colour="Diagnosis", x = eig_1, y = eig_2) +
  theme_bw() +
  theme(panel.grid.major = element_blank(), panel.grid.minor = element_blank(),
        axis.title=element_text(size=12), legend.position="bottom"))
dev.off()

#PCoA MDS1 and MDS2 plotly
print(ggplotly(ggplot(Metadata2[, c(6,8,13,14)]) +
  geom_point(aes(x=MDS1BrayHel, y=MDS2BrayHel, color = Diagnosis,
                 group = Dg_LADA), size=3) +
  stat_ellipse(aes(MDS1BrayHel, y=MDS2BrayHel, color = Diagnosis,
                  group = Diagnosis)) +
  scale_color_manual(values=c(Control = "#666666", T1D = "#1F78B4",
```

```
                              T2D = "#33A02C", LADA = "#E31A1C")) +
  ggtitle(paste("PCoA", i, sep=" ")) +
  labs(colour="Diagnosis", x = eig_1, y = eig_2) +
  theme_bw() +
  theme(panel.grid.major = element_blank(), panel.grid.minor = element_blank(),
        axis.title=element_text(size=12), legend.position="bottom")))

#PCoA MDS1 and MDS2
print(ggplot(Metadata2) +
  geom_point(aes(x=MDS1BrayHel, y=MDS2BrayHel, color = Diagnosis,
                 group = Diagnosis), size=3) +
  stat_ellipse(aes(MDS1BrayHel, y=MDS2BrayHel, color = Diagnosis,
                   group = Diagnosis)) +
  scale_color_manual(values=c(Control = "#666666", T1D = "#1F78B4",
                              T2D = "#33A02C", LADA = "#E31A1C")) +
  ggtitle(paste("PCoA", i, sep=" ")) +
  labs(colour="Diagnosis", x = eig_1, y = eig_2) +
  theme_bw() +
  theme(panel.grid.major = element_blank(), panel.grid.minor = element_blank(),
        axis.title=element_text(size=12), legend.position="bottom"))

#PCoA MDS1 and MDS3
print(ggplot(Metadata2) +
  geom_point(aes(x=MDS1BrayHel, y=MDS3BrayHel, color = Diagnosis,
                 group = Diagnosis), size=3) +
  stat_ellipse(aes(MDS1BrayHel, y=MDS3BrayHel, color = Diagnosis,
                   group = Diagnosis)) +
  scale_color_manual(values=c(Control = "#666666", T1D = "#1F78B4",
                              T2D = "#33A02C", LADA = "#E31A1C")) +
  ggtitle(paste("PCoA", i, sep=" ")) +
  labs(colour="Diagnosis", x = eig_1, y = eig_3) +
  theme_bw() +
  theme(panel.grid.major = element_blank(), panel.grid.minor = element_blank(),
        axis.title=element_text(size=12), legend.position="bottom"))

#PCoA MDS2 and MDS3
print(ggplot(Metadata2) +
  geom_point(aes(x=MDS2BrayHel, y=MDS3BrayHel, color = Diagnosis,
                 group = Diagnosis), size=3) +
  stat_ellipse(aes(MDS2BrayHel, y=MDS3BrayHel, color = Diagnosis,
                   group = Diagnosis)) +
  scale_color_manual(values=c(Control = "#666666", T1D = "#1F78B4",
                              T2D = "#33A02C", LADA = "#E31A1C")) +
  ggtitle(paste("PCoA", i, sep=" ")) +
  labs(colour="Diagnosis", x = eig_2, y = eig_3) +
  theme_bw() +
  theme(panel.grid.major = element_blank(), panel.grid.minor = element_blank(),
        axis.title=element_text(size=12), legend.position="bottom"))

#Add a categorical indicator of group in Metadata
Metadata2$Metformine<-as.factor(ifelse(grepl("1", Metadata2$Metformin), "Yes",
                                       ifelse(grepl("0", Metadata2$Metformin),
                                              "No", "Unknown")))
```

```r
#Order Treatment metformine for plotting
Metadata2$Metformine <- factor(Metadata2$Metformine, levels=c("Yes", "No", "Unknown"))
#Create PCoA for figure 1
if (i=="All") {
  PCoAall<-ggplot(Metadata2) +
  geom_point(aes(MDS1BrayHel, MDS2BrayHel, color = Diagnosis, #shape = Metformine,
                 group = Diagnosis), size=5) +
  stat_ellipse(aes(MDS1BrayHel, MDS2BrayHel, color = Diagnosis, group = Diagnosis)) +
  #stat_ellipse(aes(MDS1BrayHel, MDS2BrayHel, group = Metformine,
  #                 linetype=factor(Metadata2$Metformine)), alpha = 0.7) +
  scale_color_manual(values=c(Control = "#666666", T1D = "#1F78B4",
                              T2D = "#33A02C", LADA = "#E31A1C")) +
  scale_shape_manual(values=c(3, 16)) +
  scale_linetype_manual(values=c("longdash", "dotted"), guide=FALSE) +
  #ggtitle(paste("PCoA", "LADA & T2D Metformin", sep=" ")) +
  labs(colour="Diagnosis", x = eig_1, y = eig_2) + #, shape="Metformin treatment"
  theme_bw() +
  theme(panel.grid.major = element_blank(), panel.grid.minor = element_blank(),
        axis.title=element_text(size=22), legend.position="bottom",
        legend.title=element_text(size=20), legend.text=element_text(size=20),
        axis.text.x = element_text(angle = 45, hjust = 1, size=12),
        axis.text.y = element_text(angle = 45, hjust = 1, size=12))

  print(PCoAall)
  pdf("MicroLADA_PCoAAllMet.pdf", width=9, height=6)
  print(PCoAall)
  dev.off()
  #Save plot in previous list
  Fig1List[[ "PCoAall" ]] <- PCoAall
  #return(PCoAall)
}

##Stressplot
##Extract ordination distances and merge with observed dissimilarity
#stress<-stressplot(PCoAcsObject)
#df <- melt(as.matrix(stress))
#names(df)<-c("rowOrd", "colOrd", "OrdDist")
#df<-filter(df, OrdDist>0)
#df2 <- melt(as.matrix(distmatrix))
#names(df2)<-c("rowObs", "colObs", "ObsDism")
#df2<-filter(df2, ObsDism>0)
#df<-unite(df, mergecol, c(rowOrd, colOrd), remove=FALSE)
#df2<-unite(df2, mergecol, c(rowObs, colObs), remove=FALSE)
#ggstress<-merge(df, df2, by="mergecol")

##create stressplot
#print(ggplot(ggstress) +
#  geom_point(aes(ObsDism, OrdDist)) +
#  ggtitle(paste("Stressplot", i, sep=" ")) +
#  labs(x = "Observed dissimilarity", y = "Ordination distance") +
#  theme_bw() +
#  theme(panel.grid.major = element_blank(), panel.grid.minor = element_blank(),
#        axis.title=element_text(size=12)))
```

```
##Screeplot
#screeplot<-data.frame(PCoAcsObject$CA$eig)
#colnames(screeplot)<-c("eig")
#screeplot$eig <- screeplot$eig[1:length(screeplot$eig)] /
#  sum(screeplot$eig) * 100
#screeplot<-add_rownames(screeplot, "MDS")
#screeplot$MDS <- factor(screeplot$MDS,
#                         levels=c(sprintf("MDS%d", 1:length(screeplot$eig))))

##create screeplot
#print(ggplot(screeplot, aes(x=MDS, y=eig)) +
#  geom_bar(stat="identity") +
#  labs(x ="MDS", y ="eig (%)") +
#  ggtitle(paste("Screeplot", i, sep=" ")) +
#  theme_bw() +
#  theme(panel.grid.major = element_blank(), panel.grid.minor = element_blank(),
#        axis.title=element_text(size=12), axis.text.x=element_blank(),
#        axis.ticks.x=element_blank()))

}
```

```
## [1] "LADAControl"
```

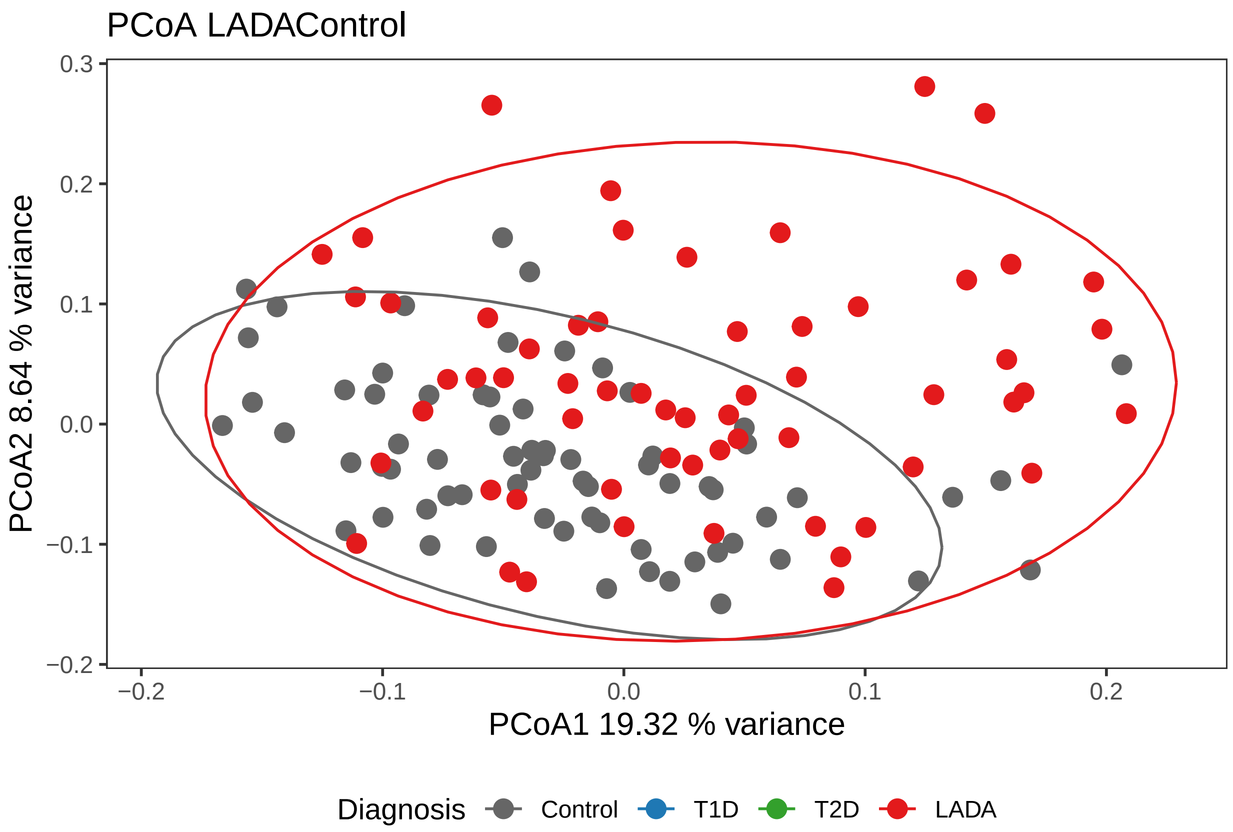

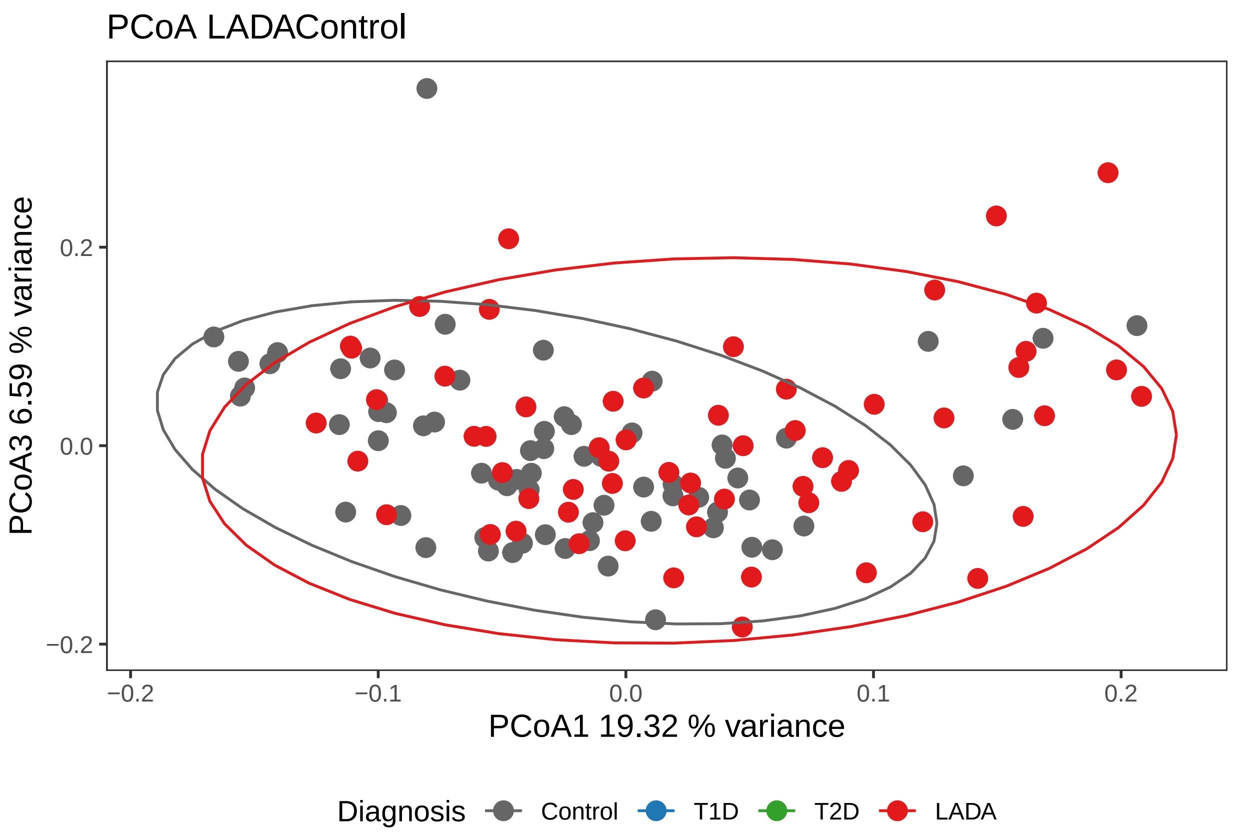
PCoA LADAControl
PCoA3 6.59 % variance
0.2
0.0
−0.2
−0.2
−0.1
0.0
0.1
0.2
PCoA1 19.32 % variance
Diagnosis
Control
T1D
T2D
LADA

```
## [1] "LADAT1D"
```

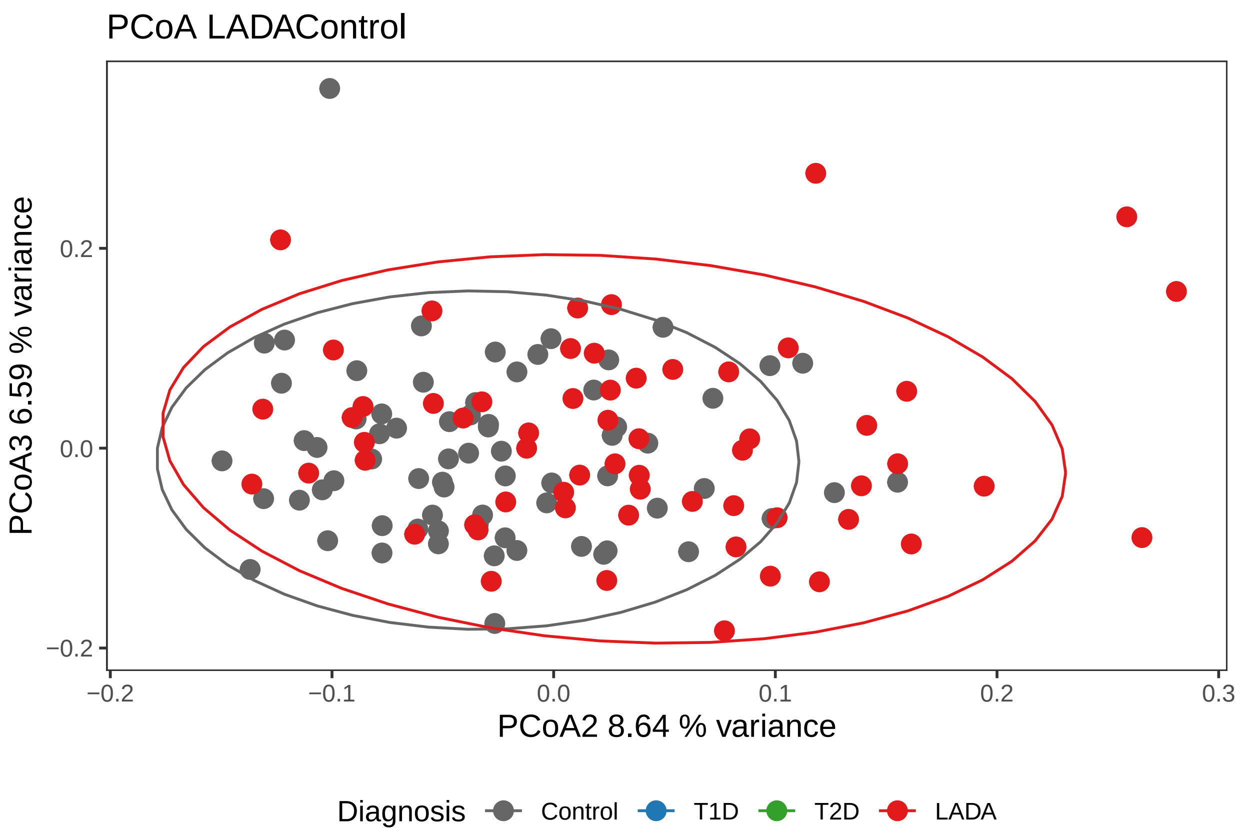
PCoA LADAControl
PCoA3 6.59 % variance
0.2
0.0
−0.2
−0.2
−0.1
0.0
0.1
0.2
0.3
PCoA2 8.64 % variance
Diagnosis
Control
T1D
T2D
LADA

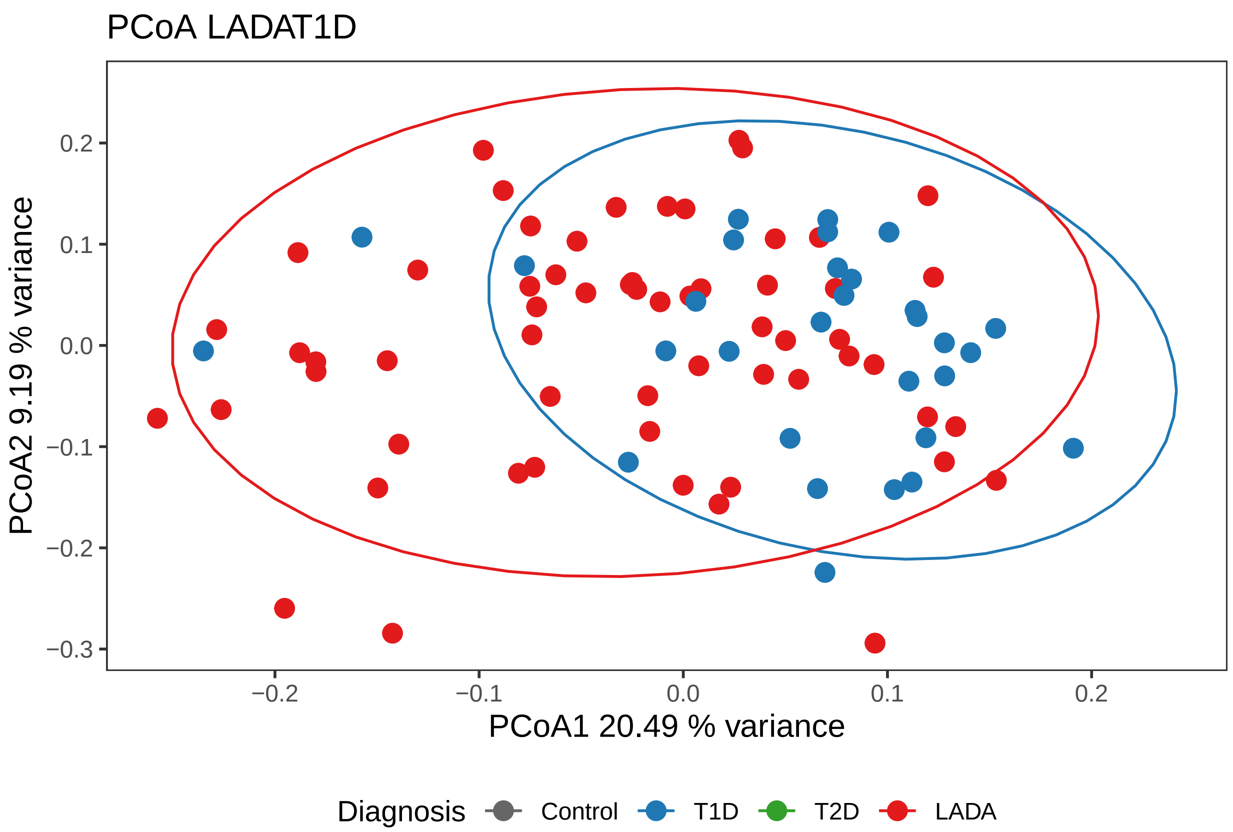
PCoA LADAT1D
PCoA2 9.19 % variance
0.2
0.1
0.0
−0.1
−0.2
−0.3
−0.2
−0.1
0.0
0.1
0.2
PCoA1 20.49 % variance
Diagnosis
Control
T1D
T2D
LADA

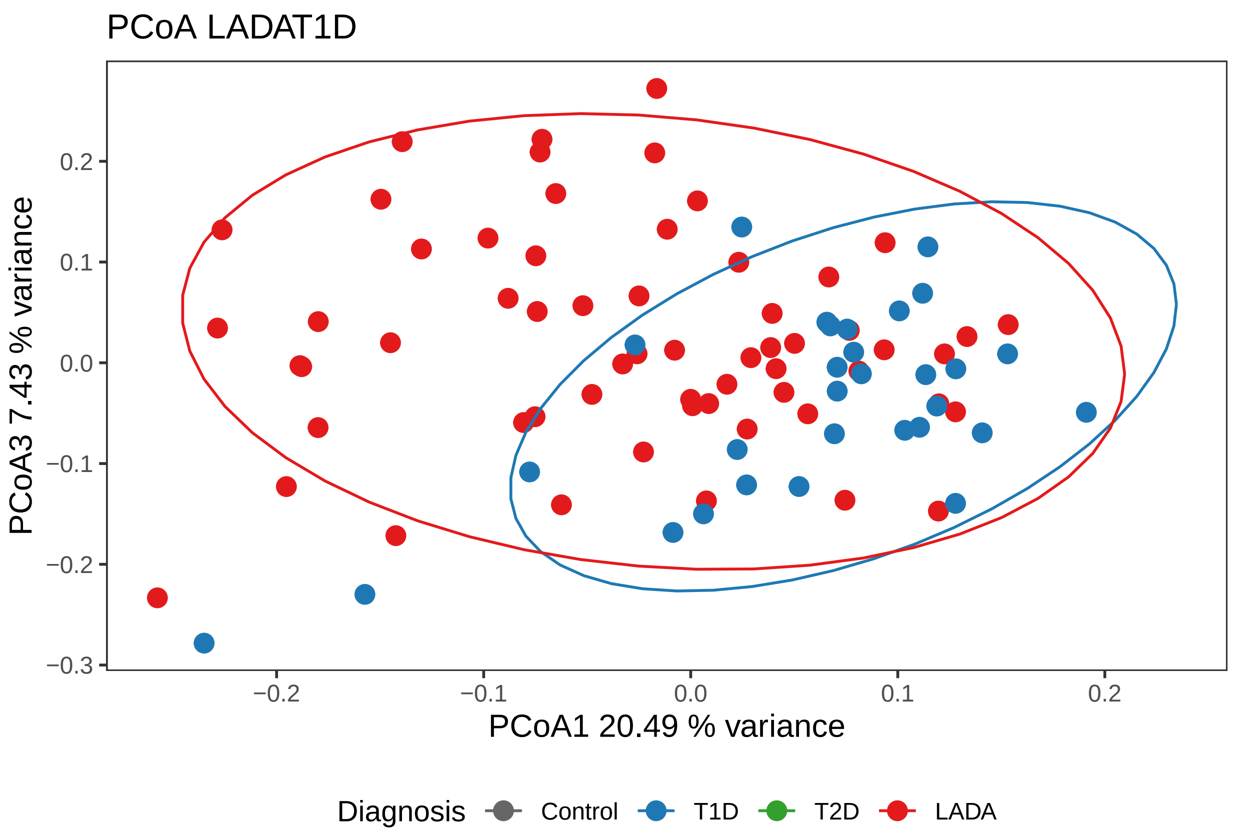

PCoA LADAT1D
PCoA3 7.43 % variance
0.2
0.1
0.0
−0.1
−0.2
−0.3
−0.2
−0.1
0.0
0.1
0.2
PCoA1 20.49 % variance
Diagnosis
Control
T1D
T2D
LADA


```
## [1] "LADAT2D"
```

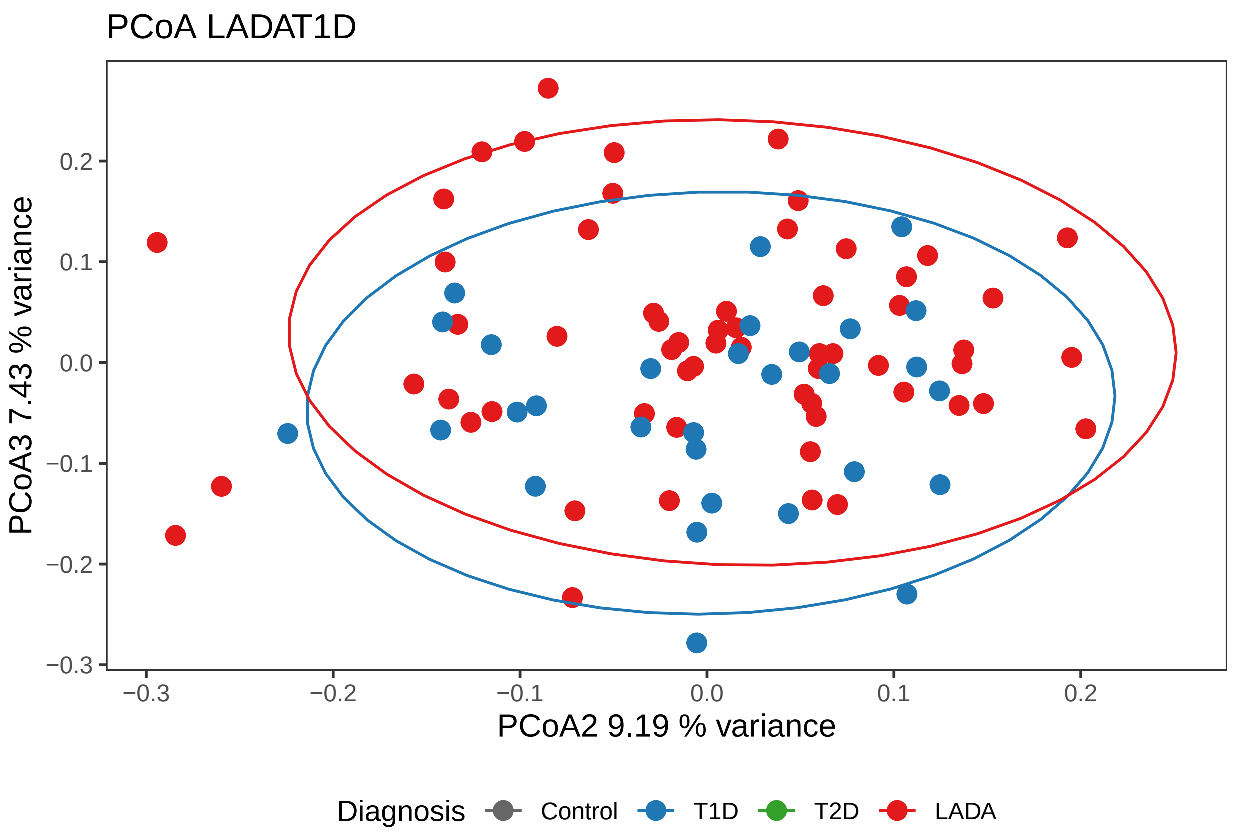
PCoA LADAT1D
PCoA3 7.43 % variance
PCoA2 9.19 % variance
0.2
0.1
0.0
−0.1
−0.2
−0.3
−0.3
−0.2
−0.1
0.0
0.1
0.2
Diagnosis
Control
T1D
T2D
LADA

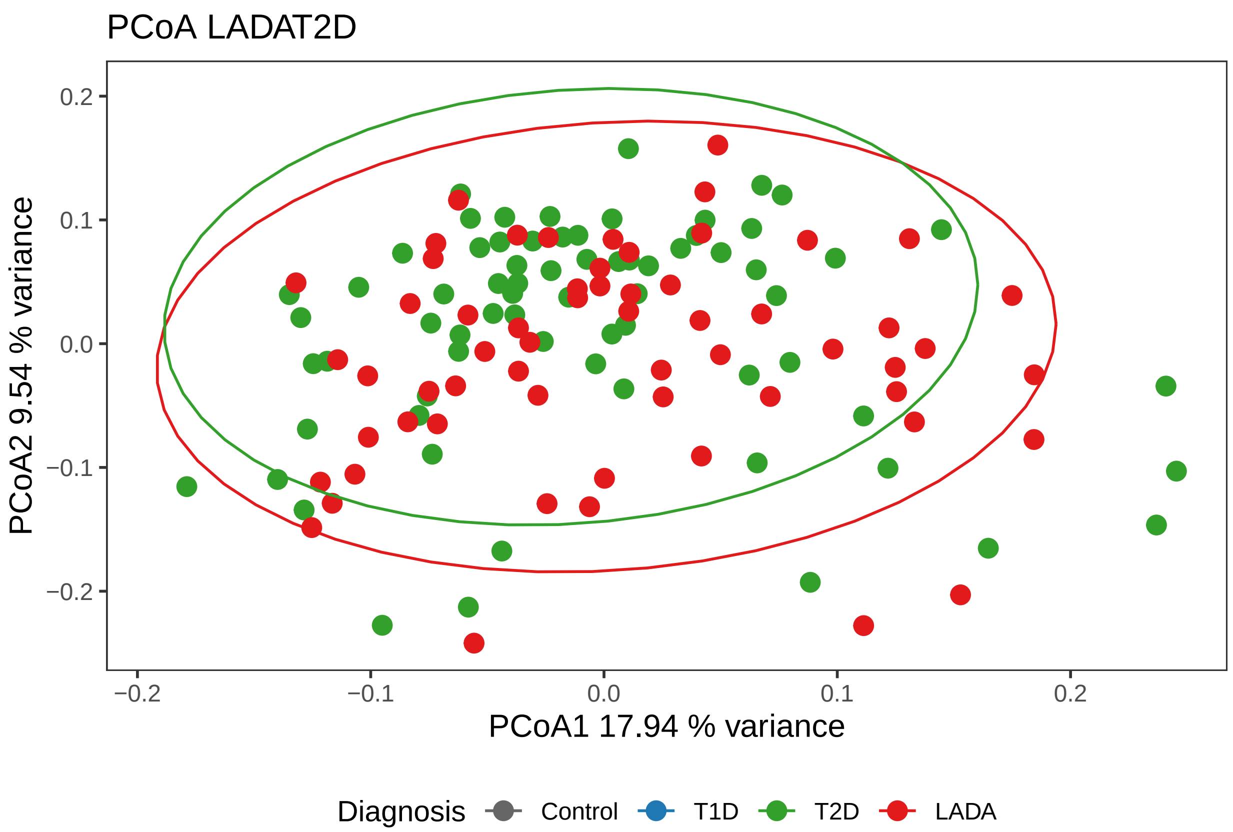
PCoA LADAT2D
0.2
0.1
0.0
−0.1
−0.2
PCoA2 9.54 % variance
−0.2
−0.1
0.0
0.1
0.2
PCoA1 17.94 % variance
Diagnosis
Control
T1D
T2D
LADA

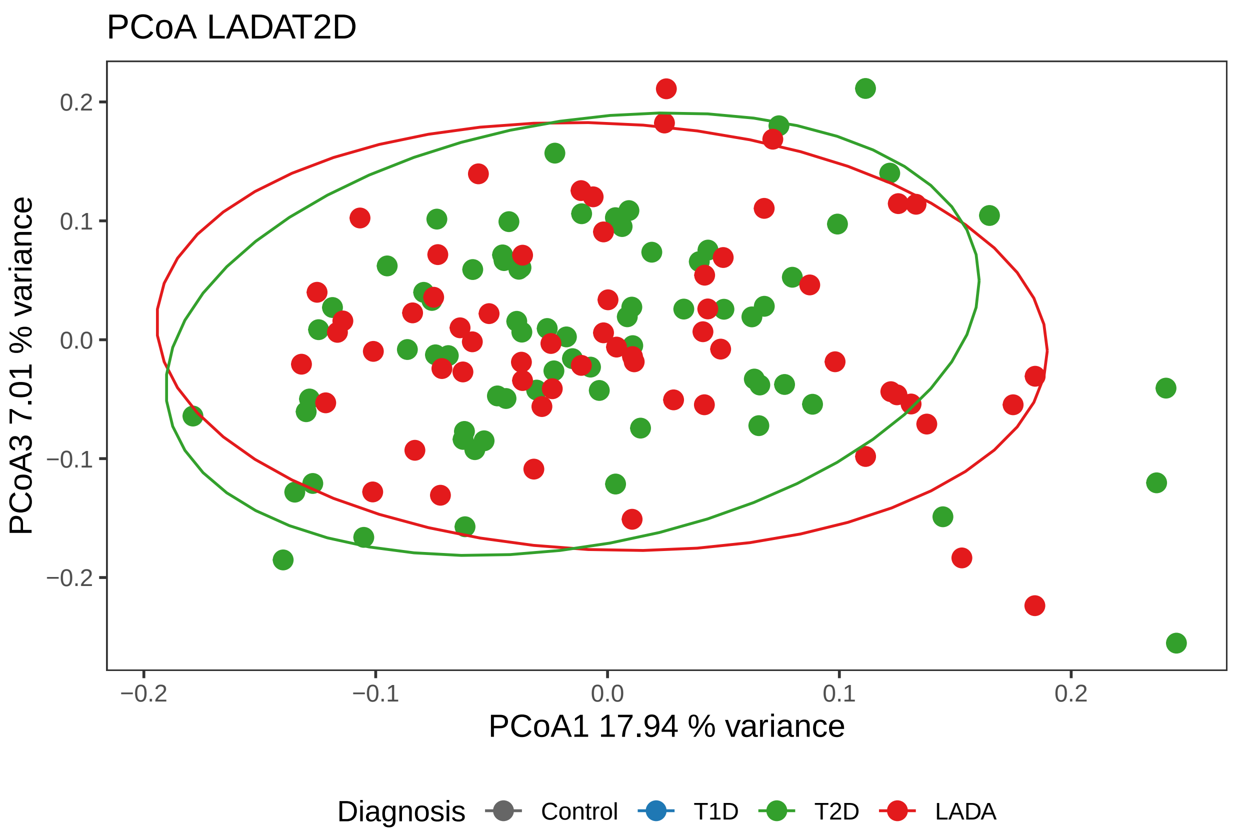
PCoA LADAT2D
PCoA3 7.01 % variance
PCoA1 17.94 % variance
0.2
0.1
0.0
−0.1
−0.2
−0.2
−0.1
0.0
0.1
0.2
Diagnosis
Control
T1D
T2D
LADA

```
## [1] "All"
```

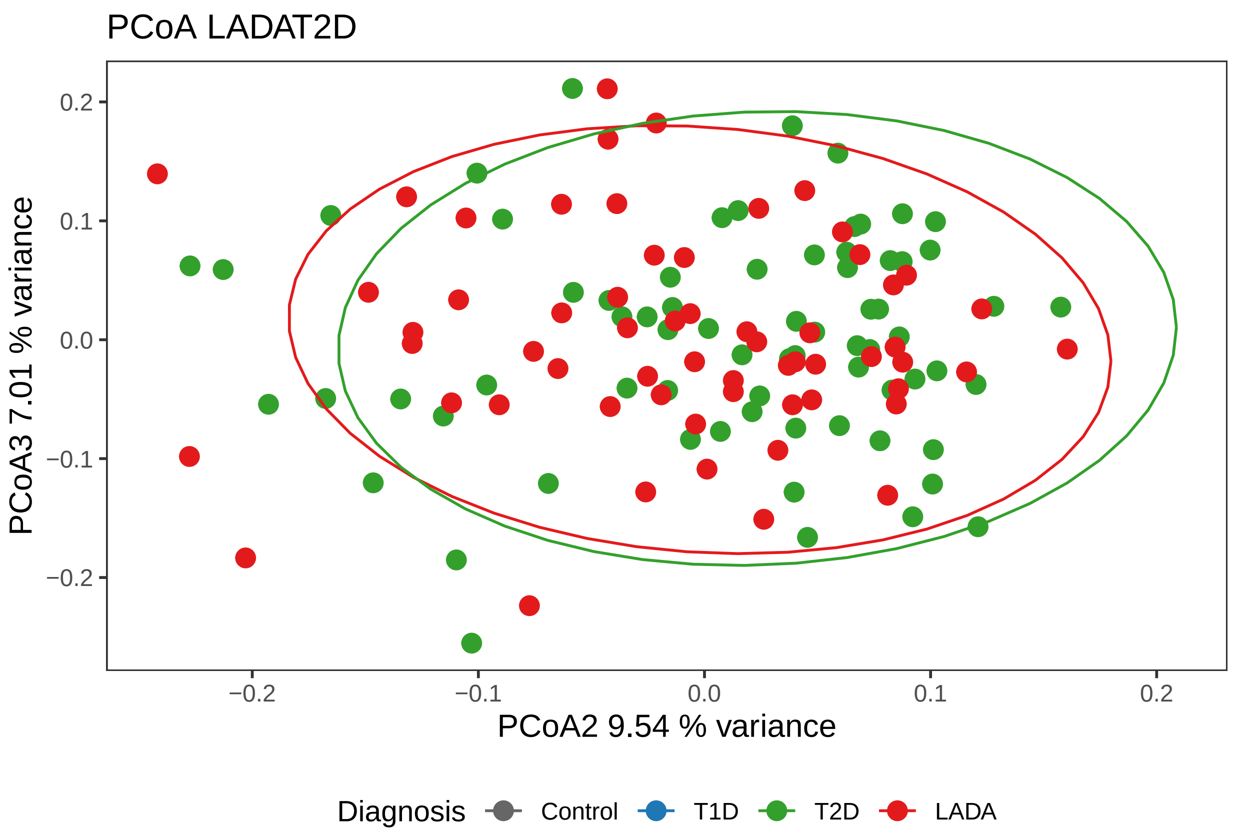
PCoA LADAT2D
PCoA3 7.01 % variance
PCoA2 9.54 % variance
0.2
0.1
0.0
−0.1
−0.2
−0.2
−0.1
0.0
0.1
0.2
Diagnosis
Control
T1D
T2D
LADA

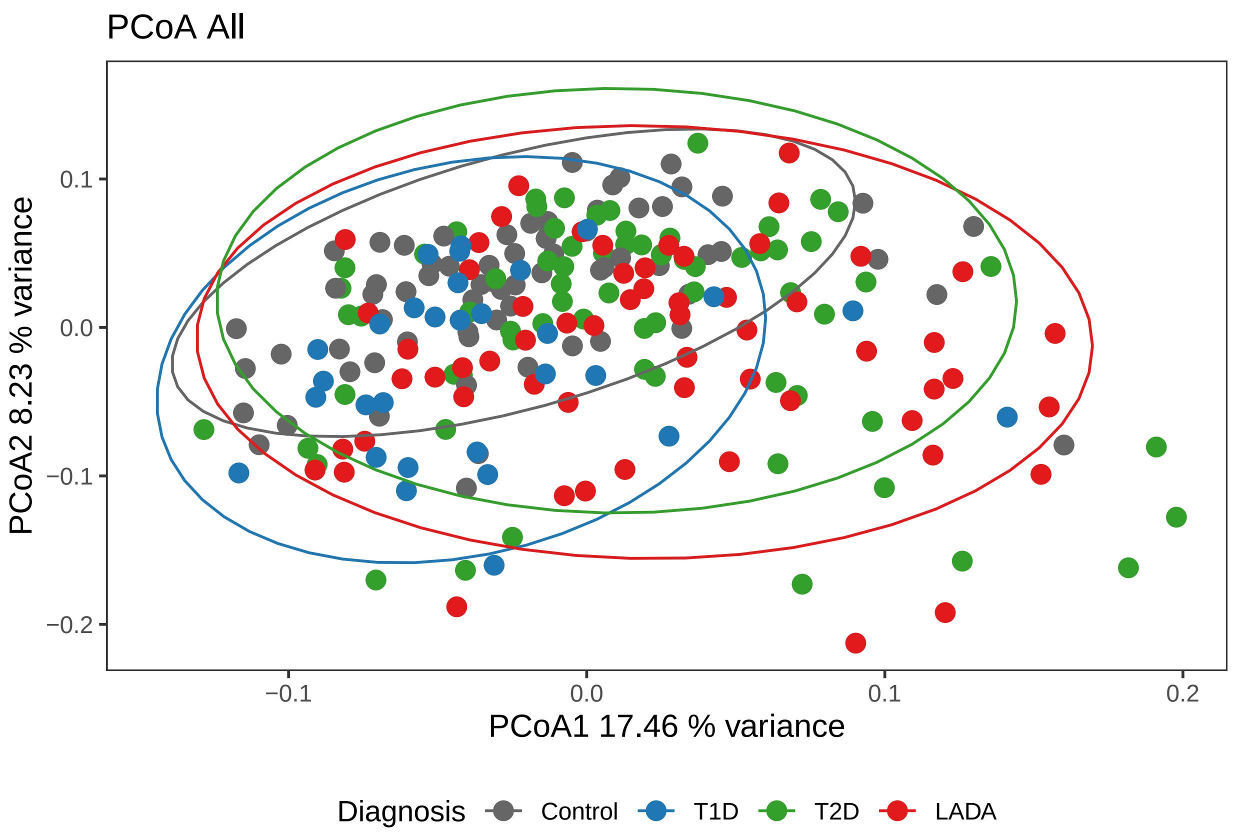
PCoA All
PCoA2 8.23 % variance
0.1
0.0
−0.1
−0.2
−0.1
0.0
0.1
0.2
PCoA1 17.46 % variance
Diagnosis
Control
T1D
T2D
LADA

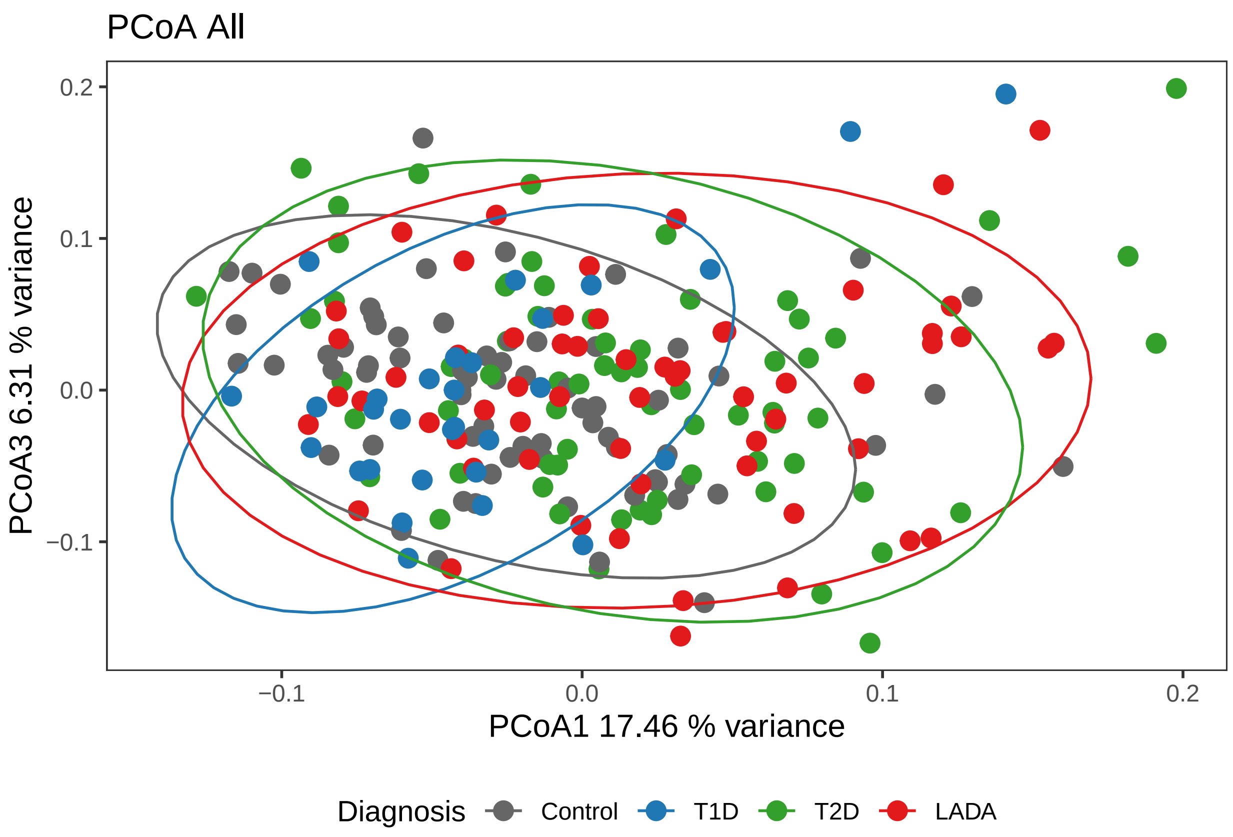

PCoA All
0.2
0.1
0.0
−0.1
PCoA3 6.31 % variance
−0.1
0.0
0.1
0.2
PCoA1 17.46 % variance
Diagnosis
Control
T1D
T2D
LADA

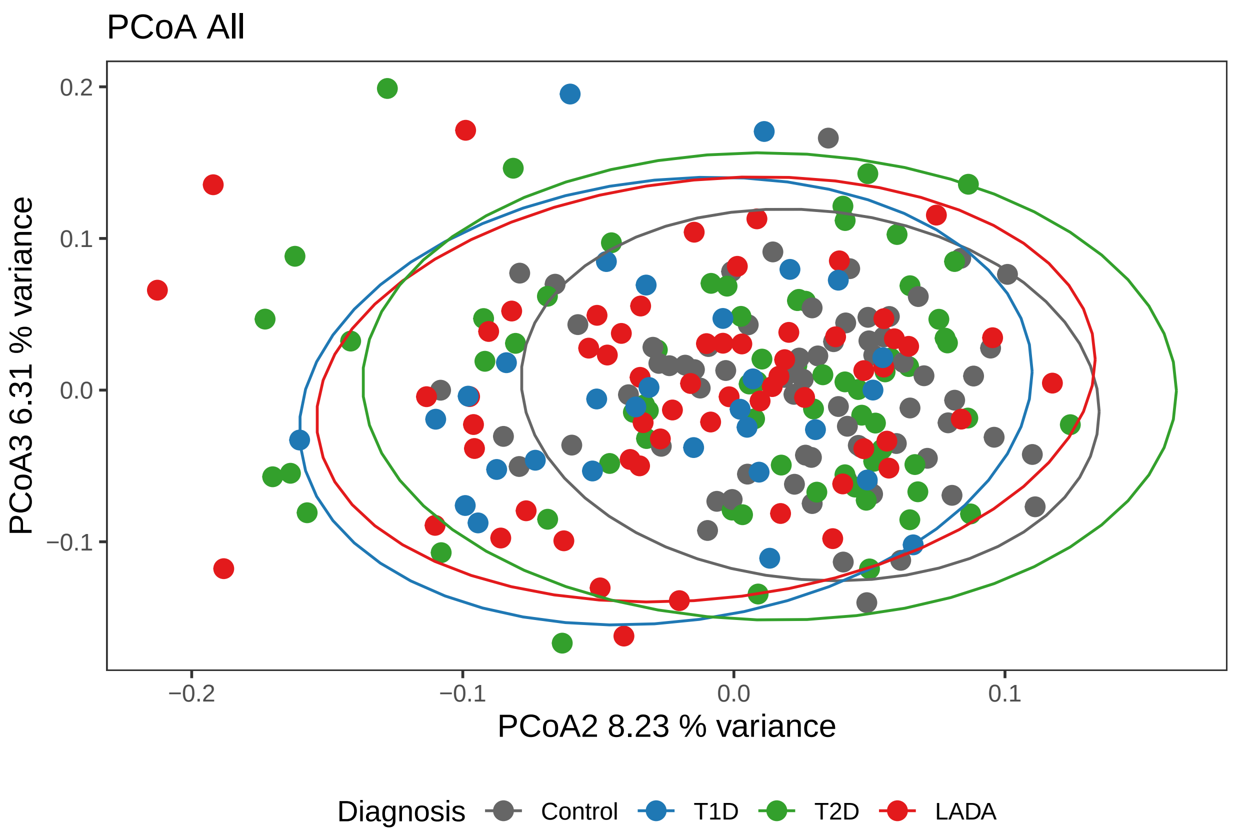
PCoA All
PCoA3 6.31 % variance
PCoA2 8.23 % variance
0.2
0.1
0.0
−0.1
−0.2
−0.1
0.0
0.1
Diagnosis
Control
T1D
T2D
LADA

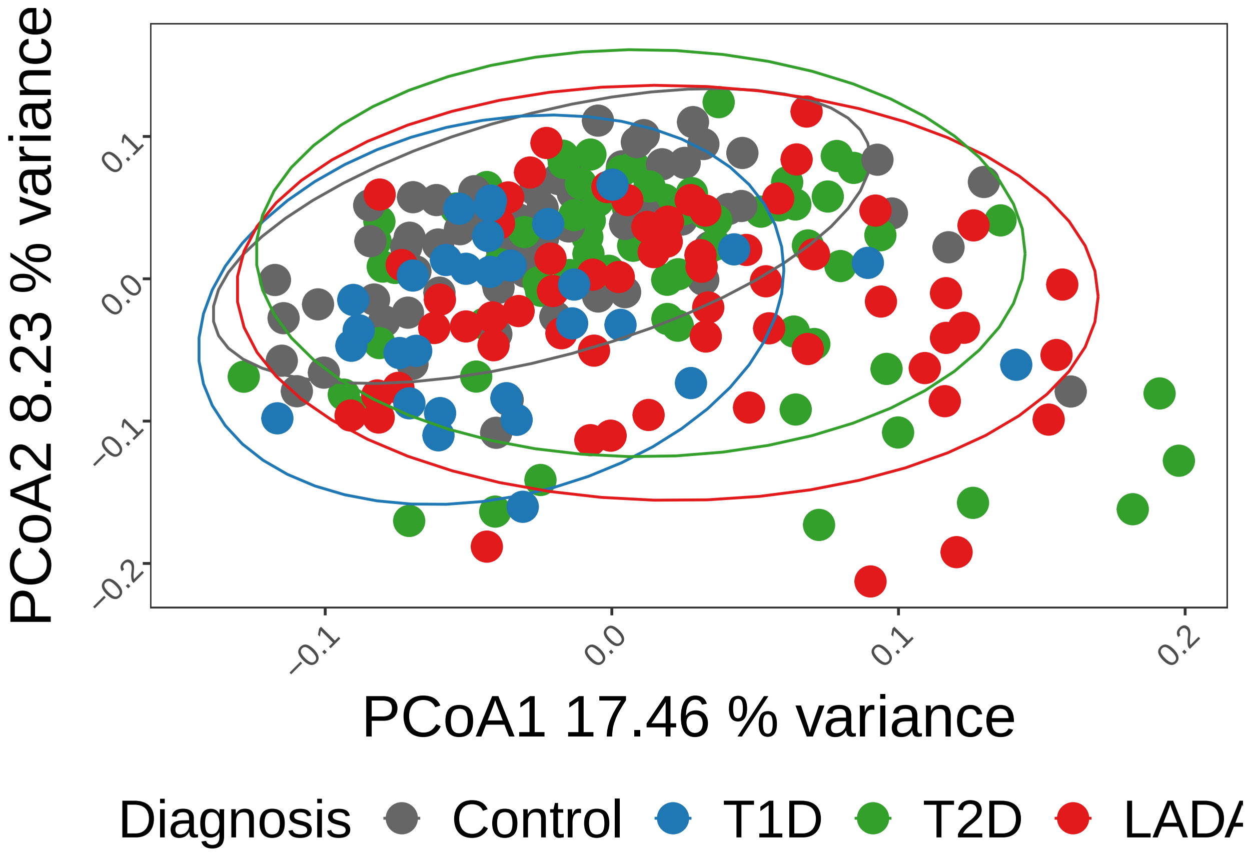

PCoA remove metformin

```
rm(list=setdiff(ls(), c("Metadata", "MetadataMed", "Feature", "TaxonomyDA", "Taxonomy",
                        "Fig1List", "Fig1ListRemMet")))
##Removing treated with metformin
#Metadata$Metformin <- ifelse(is.na(Metadata$Metformin), "Unknown", Metadata$Metformin)
Metadata2<-filter(Metadata, !Metformin == 1)
##Applying subsetting to OTU tables
Taxonomy2<-dplyr::select(Taxonomy, one_of(as.character(Metadata2$MicrobiomeID)))
##Remove orgs that are not present after subsetting.
Taxonomy2 <- Taxonomy2[rowSums(Taxonomy2)>0,]

Subset <- c("LADAControl", "LADAT1D", "LADAT2D", "All")
#i <- c("All")
#i <- c("LADAControl")
for (i in Subset) {

#Subsetting Metadata according to
if (i=="All") {
  print(i)
  Metadata3 <- Metadata2
} else if (i=="LADAControl") {
  print(i)
  Metadata3<-filter(Metadata2, Diagnosis == "LADA" | Diagnosis == "Control")
```

```r
} else if (i=="LADAT1D") {
  print(i)
  Metadata3<-filter(Metadata2, Diagnosis == "LADA" | Diagnosis == "T1D")
} else if (i=="LADAT2D") {
  print(i)
  Metadata3<-filter(Metadata2, Diagnosis == "LADA" | Diagnosis == "T2D")
} else {
  print("Subset defined not valid")
}

#Apply subsetting to Taxonomy table
Taxonomy3<-dplyr::select(Taxonomy2, one_of(as.character(Metadata3$MicrobiomeID)))


#Hellinger transformation
Taxonomy3 <- data.frame(t(decostand(t(Taxonomy3), method="hellinger")))
#Maks TSS
Taxonomy3<-sweep(Taxonomy3, 2, colSums(Taxonomy3), FUN="/")
#Dissimilarity
distmatrix <- vegdist(t(Taxonomy3), method="bray")
#Multi dimensional scaling with capscale
PCoAcsObject<-capscale(distmatrix~1)


##Add eig to plot axes. with cmdscale there are negative values not with capscale
eig <- PCoAcsObject$CA$eig
# Calculate the variation explained by PCoA1, 2, 3 and 4
# and use it to generate axis labels
eig_1_2 <- eig[1:4] / sum(eig) * 100 #Vector with variance explained
#  by the first 4 axes
eig_1 <- paste("PCoA1", round(eig_1_2[1], digits = 2), "% variance")
eig_2 <- paste("PCoA2", round(eig_1_2[2], digits = 2), "% variance")
eig_3 <- paste("PCoA3", round(eig_1_2[3], digits = 2), "% variance")
eig_4 <- paste("PCoA4", round(eig_1_2[4], digits = 2), "% variance")

##Pull out coordinates for plotting from the ca object
#Structuring to add to Metadata2
PCoACA<-PCoAcsObject$CA #The ca object contains the actual ordination results:
#u ((Weighted) orthonormal site scores),
#v ((Weighted) orthonormal species scores) all na in mine (unconstrained),
#Xbar (The standardized data matrix after previous stages of analysis),
#and imaginary.u.eig ???.
#Info http://cc.oulu.fi/~jarioksa/softhelp/vegan/html/cca.object.html
PCoA<-as.data.frame(PCoACA$u)
#Change colnames. Now add dis and trans info to names
colnames(PCoA) <- c("MDS1BrayHel","MDS2BrayHel", "MDS3BrayHel","MDS4BrayHel")
#Add row names to df
PCoA$MicrobiomeID <- row.names(PCoA)
#Remove leading X
PCoA$MicrobiomeID <- gsub("X", "", PCoA$MicrobiomeID)
#Merge according to Sample
Metadata4<-merge(Metadata3, PCoA, by="MicrobiomeID")
```

```r
#PCoA MDS1 and MDS2 pdf
pdf(paste("MicroLADA_PCoARemMet", i, ".pdf", sep=""), width=9, height=6)
print(ggplot(Metadata4) +
  geom_point(aes(x=MDS1BrayHel, y=MDS2BrayHel, color = Diagnosis,
                 group = Diagnosis), size=3) +
  stat_ellipse(aes(MDS1BrayHel, y=MDS2BrayHel, color = Diagnosis,
                   group = Diagnosis)) +
  scale_color_manual(values=c(Control = "#666666", T1D = "#1F78B4",
                              T2D = "#33A02C", LADA = "#E31A1C")) +
  ggtitle(paste("PCoA", i, sep=" ")) +
  labs(colour="Diagnosis", x = eig_1, y = eig_2) +
  theme_bw() +
  theme(panel.grid.major = element_blank(), panel.grid.minor = element_blank(),
        axis.title=element_text(size=12), legend.position="bottom"))
dev.off()

#PCoA MDS1 and MDS2 plotly
print(ggplotly(ggplot(Metadata4[, c(6,8,13,14)]) +
  geom_point(aes(x=MDS1BrayHel, y=MDS2BrayHel, color = Diagnosis,
                 group = Dg_LADA), size=3) +
  stat_ellipse(aes(MDS1BrayHel, y=MDS2BrayHel, color = Diagnosis,
                   group = Diagnosis)) +
  scale_color_manual(values=c(Control = "#666666", T1D = "#1F78B4",
                              T2D = "#33A02C", LADA = "#E31A1C")) +
  ggtitle(paste("PCoA", i, sep=" ")) +
  labs(colour="Diagnosis", x = eig_1, y = eig_2) +
  theme_bw() +
  theme(panel.grid.major = element_blank(), panel.grid.minor = element_blank(),
        axis.title=element_text(size=12), legend.position="bottom")))

#PCoA MDS1 and MDS2
print(ggplot(Metadata4) +
  geom_point(aes(x=MDS1BrayHel, y=MDS2BrayHel, color = Diagnosis,
                 group = Diagnosis), size=3) +
  stat_ellipse(aes(MDS1BrayHel, y=MDS2BrayHel, color = Diagnosis,
                   group = Diagnosis)) +
  scale_color_manual(values=c(Control = "#666666", T1D = "#1F78B4",
                              T2D = "#33A02C", LADA = "#E31A1C")) +
  ggtitle(paste("PCoA", i, sep=" ")) +
  labs(colour="Diagnosis", x = eig_1, y = eig_2) +
  theme_bw() +
  theme(panel.grid.major = element_blank(), panel.grid.minor = element_blank(),
        axis.title=element_text(size=12), legend.position="bottom"))

#PCoA MDS1 and MDS3
print(ggplot(Metadata4) +
  geom_point(aes(x=MDS1BrayHel, y=MDS3BrayHel, color = Diagnosis,
                 group = Diagnosis), size=3) +
  stat_ellipse(aes(MDS1BrayHel, y=MDS3BrayHel, color = Diagnosis,
                   group = Diagnosis)) +
  scale_color_manual(values=c(Control = "#666666", T1D = "#1F78B4",
                              T2D = "#33A02C", LADA = "#E31A1C")) +
  ggtitle(paste("PCoA", i, sep=" ")) +
```

```r
  labs(colour="Diagnosis", x = eig_1, y = eig_3) +
  theme_bw() +
  theme(panel.grid.major = element_blank(), panel.grid.minor = element_blank(),
        axis.title=element_text(size=12), legend.position="bottom"))

#PCoA MDS2 and MDS3
print(ggplot(Metadata4) +
  geom_point(aes(x=MDS2BrayHel, y=MDS3BrayHel, color = Diagnosis,
                 group = Diagnosis), size=3) +
  stat_ellipse(aes(MDS2BrayHel, y=MDS3BrayHel, color = Diagnosis,
                   group = Diagnosis)) +
  scale_color_manual(values=c(Control = "#666666", T1D = "#1F78B4",
                              T2D = "#33A02C", LADA = "#E31A1C")) +
  ggtitle(paste("PCoA", i, sep=" ")) +
  labs(colour="Diagnosis", x = eig_2, y = eig_3) +
  theme_bw() +
  theme(panel.grid.major = element_blank(), panel.grid.minor = element_blank(),
        axis.title=element_text(size=12), legend.position="bottom"))

#Add a categorical indicator of group in Metadata
Metadata4$Metformine<-as.factor(ifelse(grepl("1", Metadata4$Metformin), "Yes",
                                       ifelse(grepl("0", Metadata4$Metformin),
                                              "No", "Unknown")))
#Order Treatment metformine for plotting
Metadata4$Metformine <- factor(Metadata4$Metformine, levels=c("Yes", "No", "Unknown"))
#Create PCoA for figure 1
if (i=="All") {
  PCoAall<-ggplot(Metadata4) +
  geom_point(aes(MDS1BrayHel, MDS2BrayHel, color = Diagnosis, #shape = Metformine,
                 group = Diagnosis), size=5) +
  stat_ellipse(aes(MDS1BrayHel, MDS2BrayHel, color = Diagnosis, group = Diagnosis)) +
  #stat_ellipse(aes(MDS1BrayHel, MDS2BrayHel, group = Metformine,
  #                 linetype=factor(Metadata4$Metformine)), alpha = 0.7) +
  scale_color_manual(values=c(Control = "#666666", T1D = "#1F78B4",
                              T2D = "#33A02C", LADA = "#E31A1C")) +
  scale_shape_manual(values=c(3, 16)) +
  scale_linetype_manual(values=c("longdash", "dotted"), guide=FALSE) +
  #ggtitle(paste("PCoA", "LADA & T2D Metformin", sep=" ")) +
  labs(colour="Diagnosis", x = eig_1, y = eig_2) + #, shape="Metformin treatment"
  theme_bw() +
  theme(panel.grid.major = element_blank(), panel.grid.minor = element_blank(),
        axis.title=element_text(size=22), legend.position="bottom",
        legend.title=element_text(size=20), legend.text=element_text(size=20),
        axis.text.x = element_text(angle = 45, hjust = 1, size=12),
        axis.text.y = element_text(angle = 45, hjust = 1, size=12))

  print(PCoAall)
  pdf("MicroLADA_PCoAAllMetRemMet.pdf", width=9, height=6)
  print(PCoAall)
  dev.off()
  #Save plot in previous list
  Fig1ListRemMet[[ "PCoAall" ]] <- PCoAall
  #return(PCoAall)
```

```
}

##Stressplot
##Extract ordination distances and merge with observed dissimilarity
#stress<-stressplot(PCoAcsObject)
#df <- melt(as.matrix(stress))
#names(df)<-c("rowOrd", "colOrd", "OrdDist")
#df<-filter(df, OrdDist>0)
#df2 <- melt(as.matrix(distmatrix))
#names(df2)<-c("rowObs", "colObs", "ObsDism")
#df2<-filter(df2, ObsDism>0)
#df<-unite(df, mergecol, c(rowOrd, colOrd), remove=FALSE)
#df2<-unite(df2, mergecol, c(rowObs, colObs), remove=FALSE)
#ggstress<-merge(df, df2, by="mergecol")

##create stressplot
#print(ggplot(ggstress) +
#  geom_point(aes(ObsDism, OrdDist)) +
#  ggtitle(paste("Stressplot", i, sep=" ")) +
#  labs(x = "Observed dissimilarity", y = "Ordination distance") +
#  theme_bw() +
#  theme(panel.grid.major = element_blank(), panel.grid.minor = element_blank(),
#        axis.title=element_text(size=12)))


##Screeplot
#screeplot<-data.frame(PCoAcsObject$CA$eig)
#colnames(screeplot)<-c("eig")
#screeplot$eig <- screeplot$eig[1:length(screeplot$eig)] /
#  sum(screeplot$eig) * 100
#screeplot<-add_rownames(screeplot, "MDS")
#screeplot$MDS <- factor(screeplot$MDS,
#                         levels=c(sprintf("MDS%d", 1:length(screeplot$eig))))

##create screeplot
#print(ggplot(screeplot, aes(x=MDS, y=eig)) +
#  geom_bar(stat="identity") +
#  labs(x ="MDS", y ="eig (%)") +
#  ggtitle(paste("Screeplot", i, sep=" ")) +
#  theme_bw() +
#  theme(panel.grid.major = element_blank(), panel.grid.minor = element_blank(),
#        axis.title=element_text(size=12), axis.text.x=element_blank(),
#        axis.ticks.x=element_blank()))

}
```

```
## [1] "LADAControl"
```

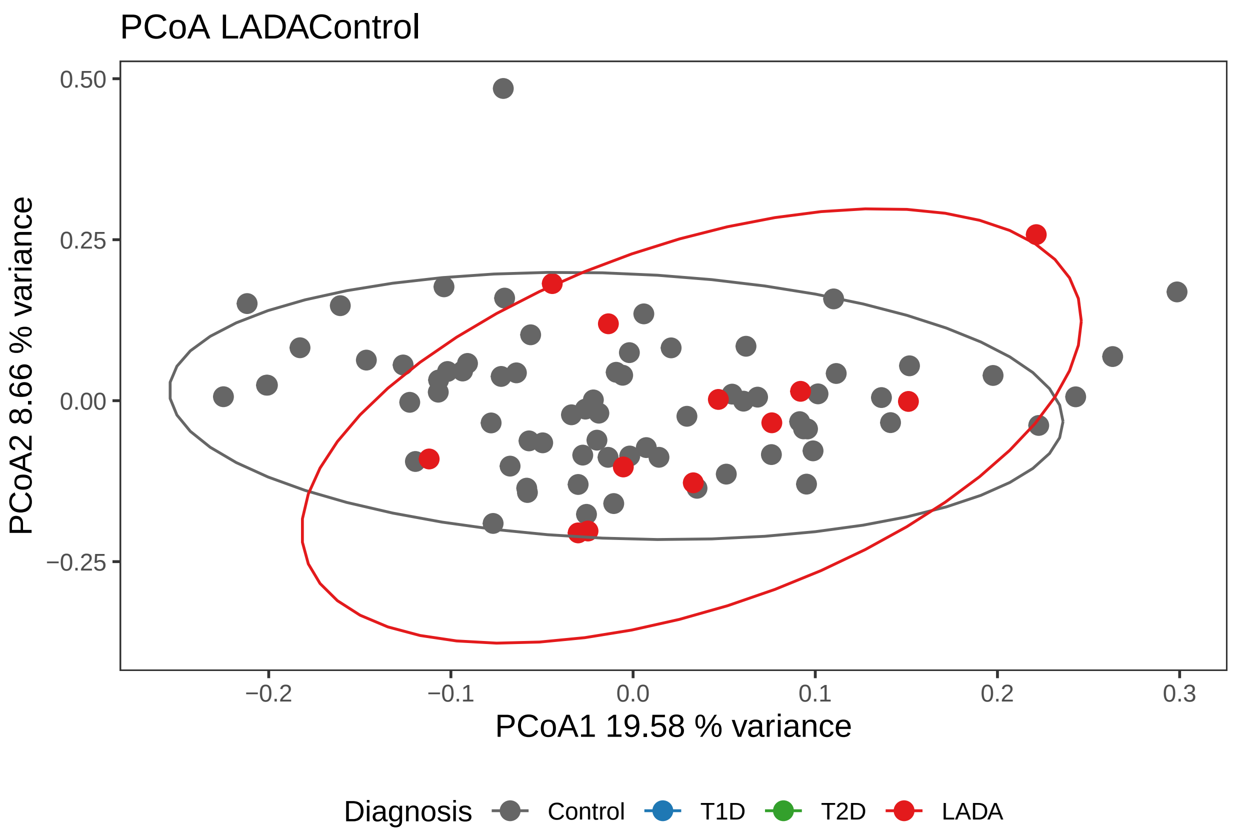
PCoA LADAControl
0.50
0.25
0.00
−0.25
PCoA2 8.66 % variance
−0.2
−0.1
0.0
0.1
0.2
0.3
PCoA1 19.58 % variance
Diagnosis
Control
T1D
T2D
LADA

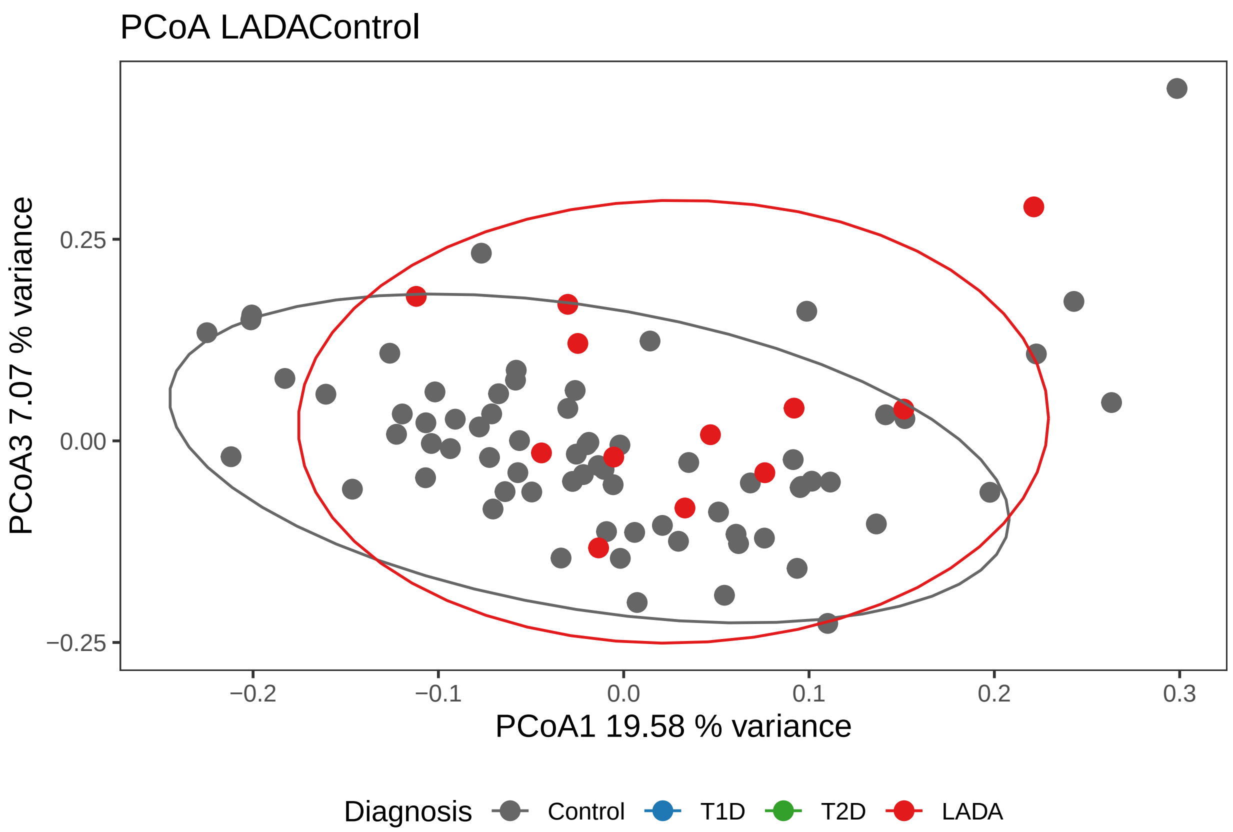

PCoA LADAControl
PCoA3 7.07 % variance
0.25
0.00
−0.25
−0.2
−0.1
0.0
0.1
0.2
0.3
PCoA1 19.58 % variance
Diagnosis
Control
T1D
T2D
LADA


```
## [1] "LADAT1D"
```

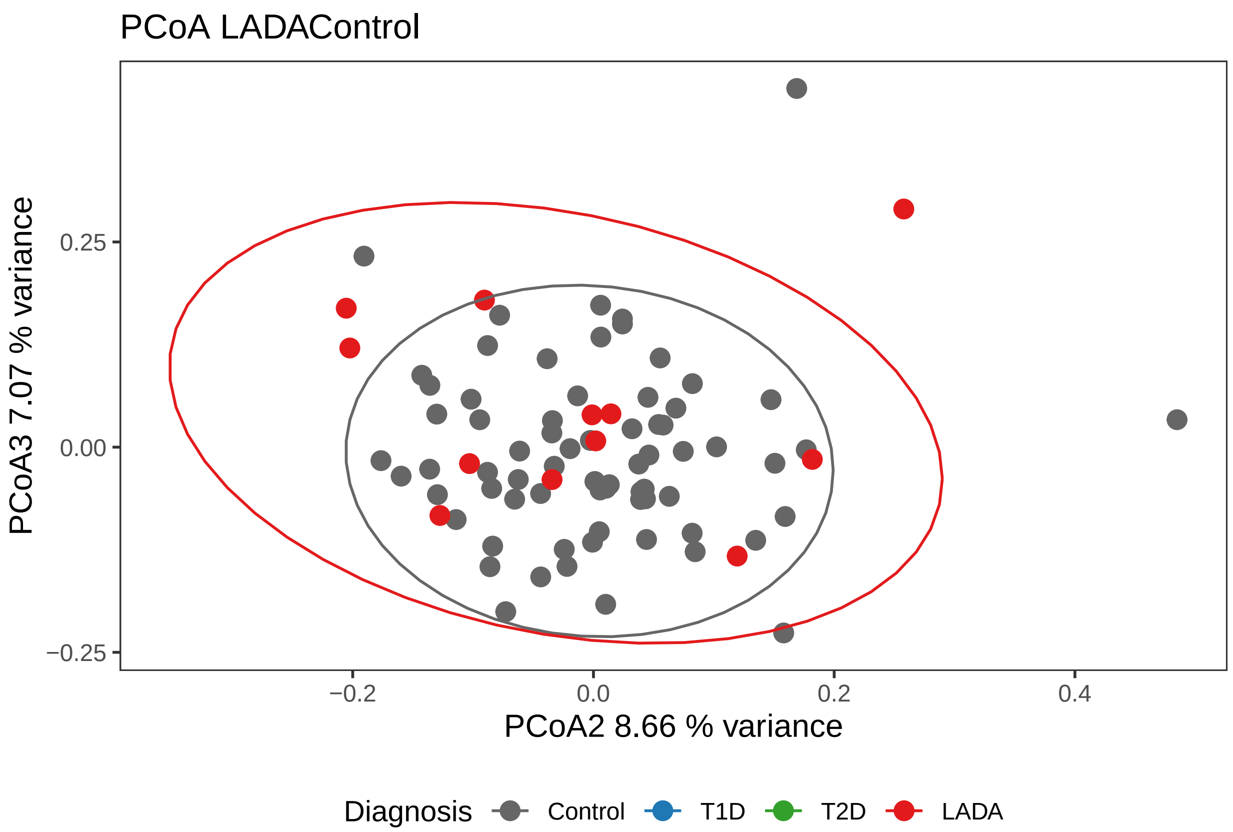
PCoA LADAControl
PCoA3 7.07 % variance
0.25
0.00
−0.25
−0.2
0.0
0.2
0.4
PCoA2 8.66 % variance
Diagnosis
Control
T1D
T2D
LADA

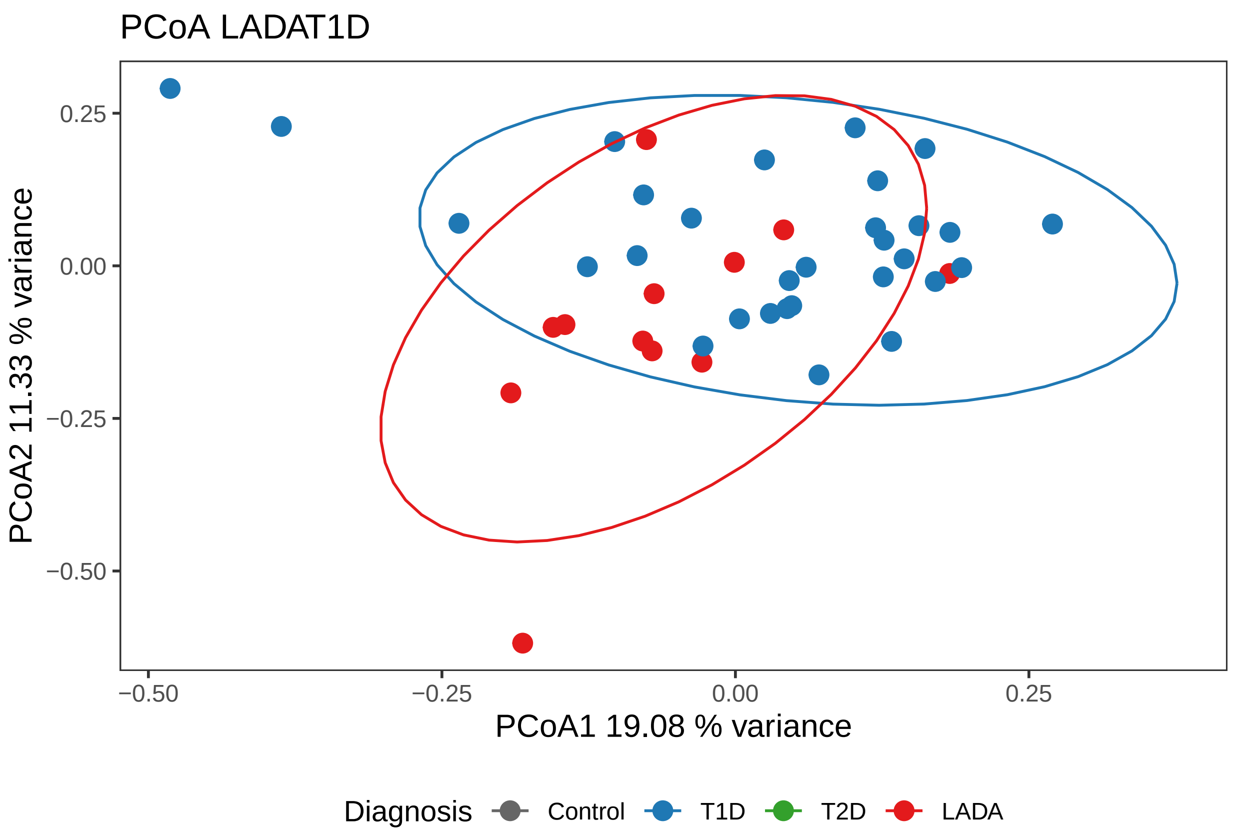
PCoA LADAT1D
PCoA2 11.33 % variance
0.25
0.00
−0.25
−0.50
−0.50
−0.25
0.00
0.25
PCoA1 19.08 % variance
Diagnosis
Control
T1D
T2D
LADA

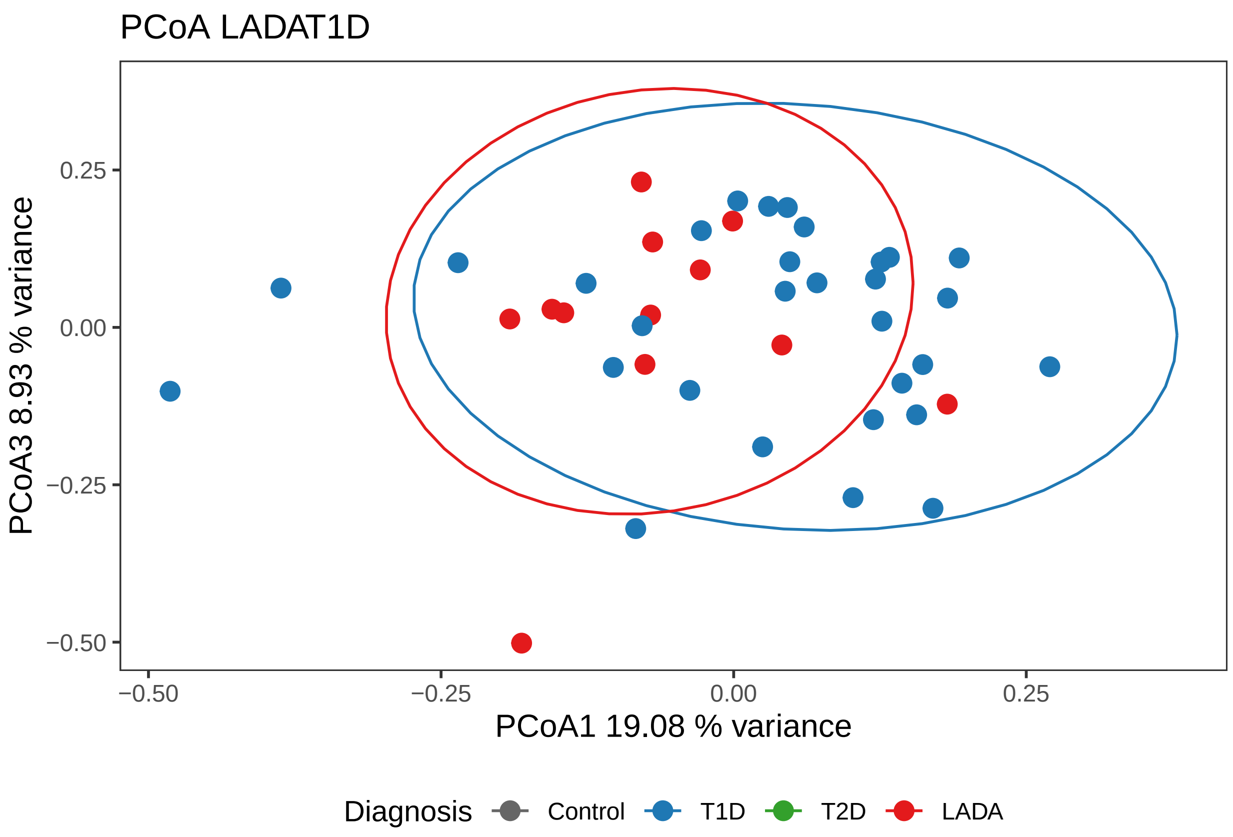
PCoA LADAT1D
PCoA3 8.93 % variance
PCoA1 19.08 % variance
0.25
0.00
−0.25
−0.50
−0.50
−0.25
0.00
0.25
Diagnosis
Control
T1D
T2D
LADA

```
## [1] "LADAT2D"
```

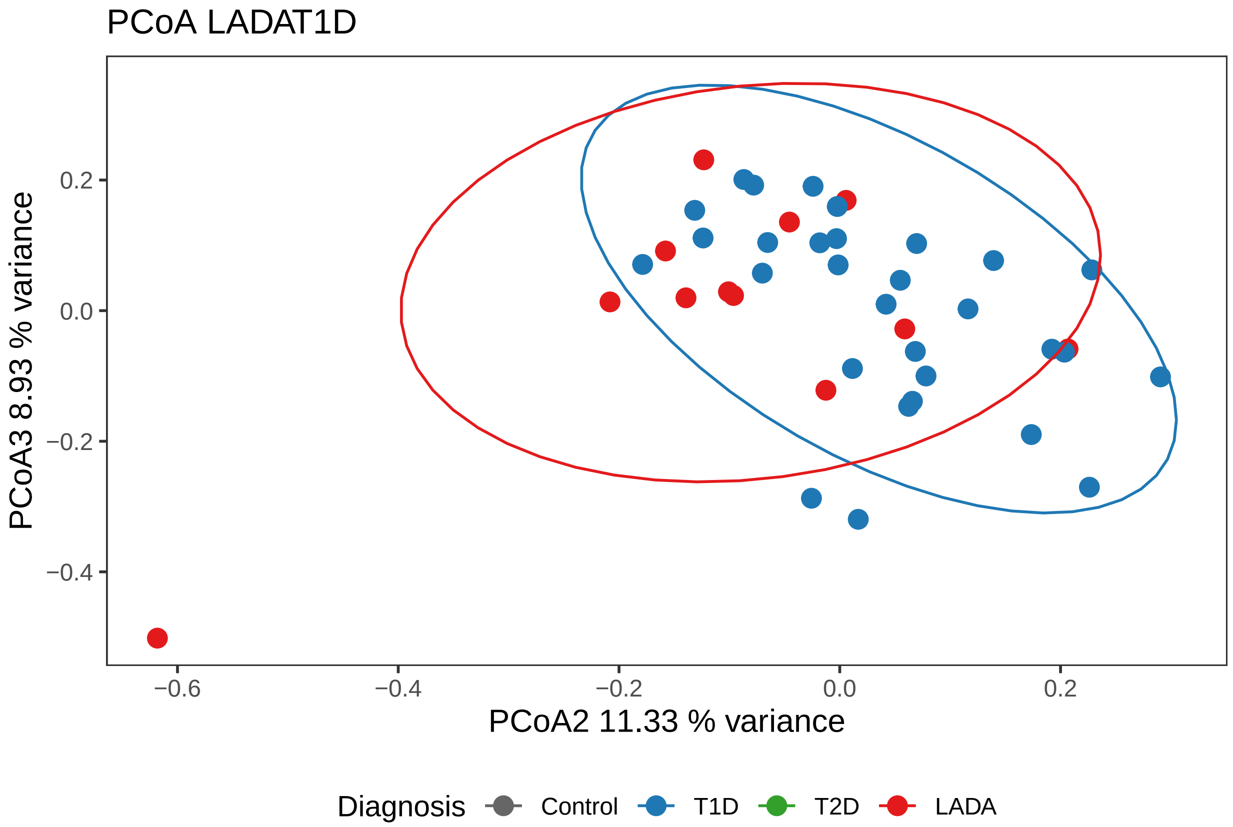
PCoA LADAT1D
PCoA3 8.93 % variance
0.2
0.0
−0.2
−0.4
−0.6
−0.4
−0.2
0.0
0.2
PCoA2 11.33 % variance
Diagnosis
Control
T1D
T2D
LADA

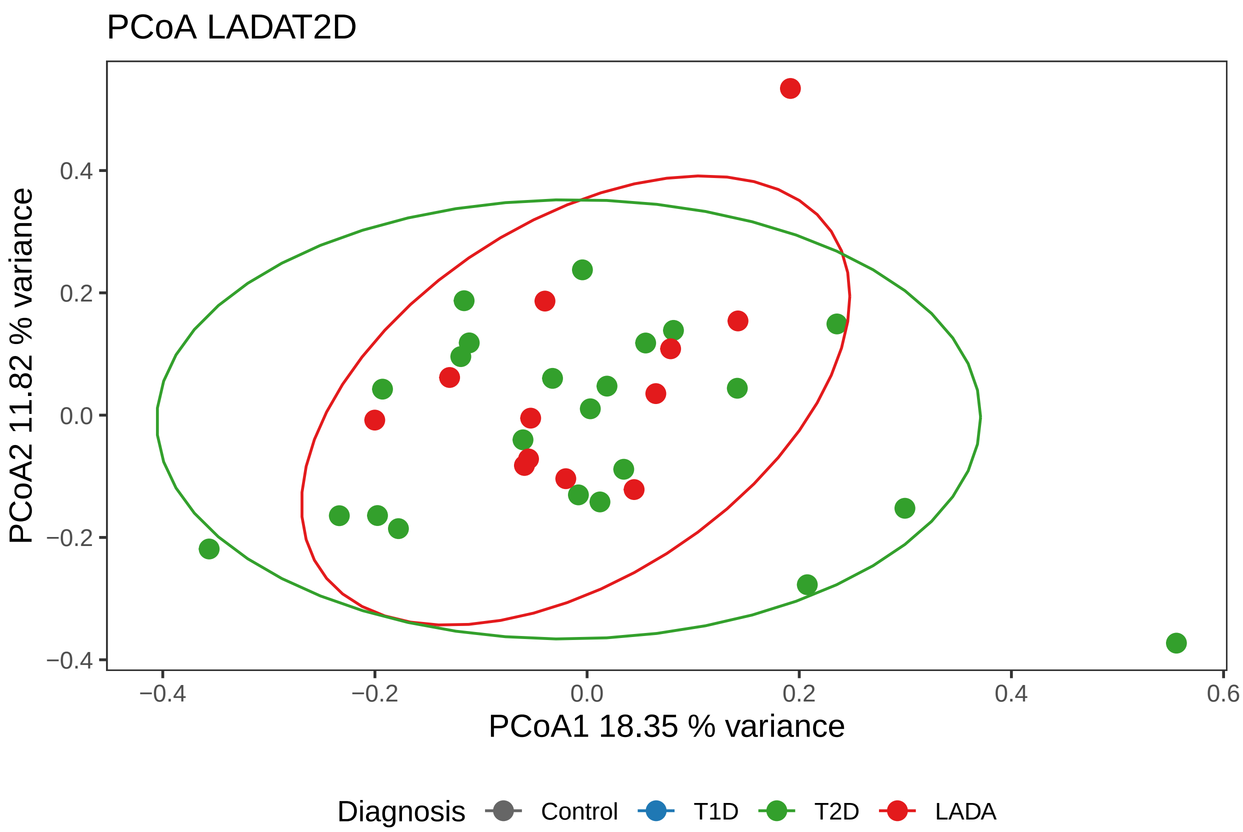
PCoA LADAT2D
PCoA2 11.82 % variance
0.4
0.2
0.0
−0.2
−0.4
−0.4
−0.2
0.0
0.2
0.4
0.6
PCoA1 18.35 % variance
Diagnosis
Control
T1D
T2D
LADA

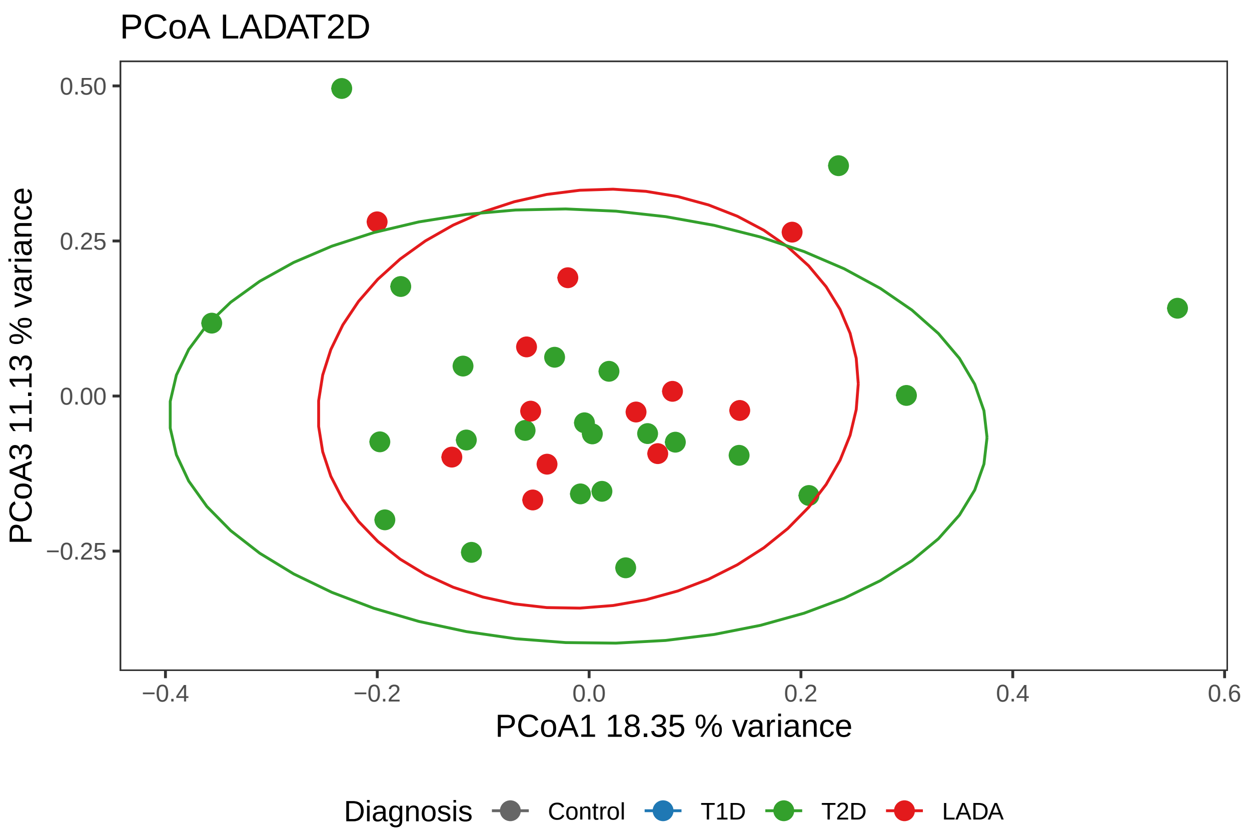
PCoA LADAT2D
0.50
0.25
0.00
−0.25
PCoA3 11.13 % variance
−0.4
−0.2
0.0
0.2
0.4
0.6
PCoA1 18.35 % variance
Diagnosis
Control
T1D
T2D
LADA

```
## [1] "All"
```

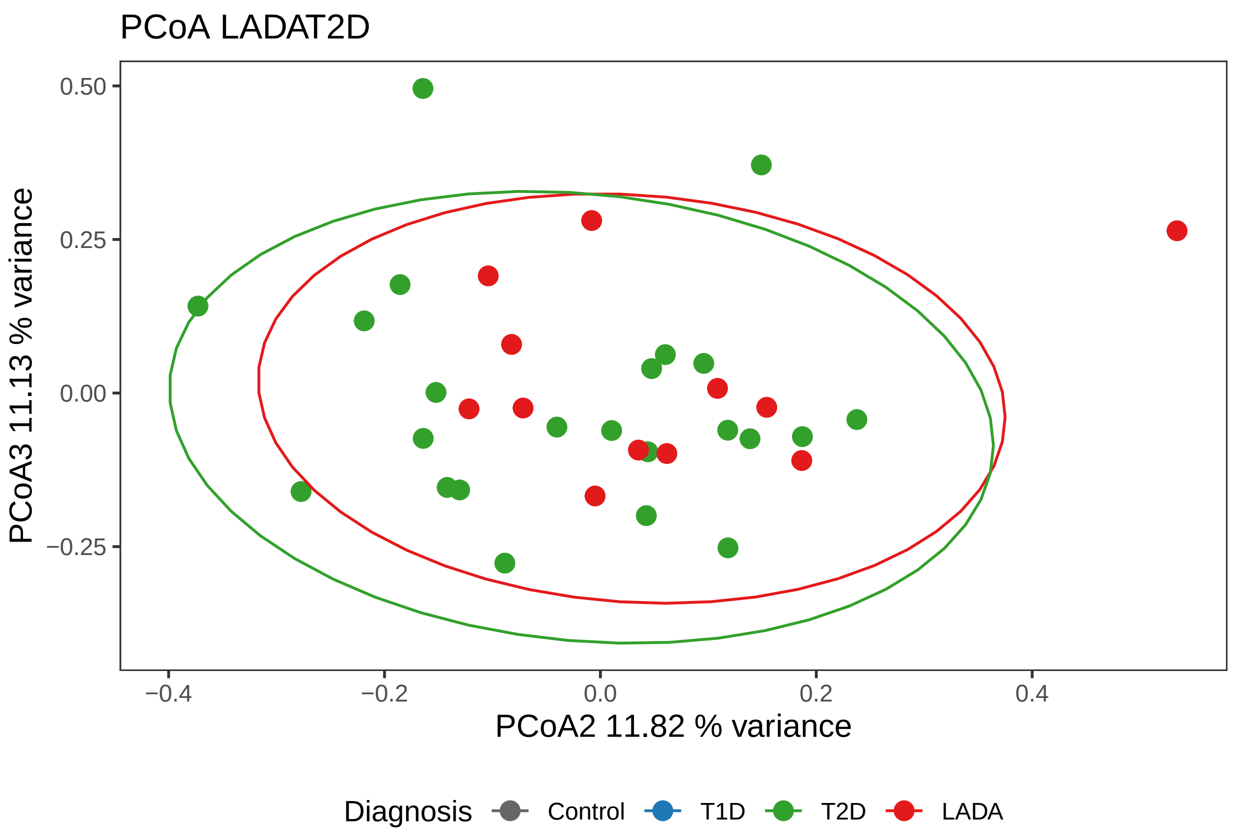
PCoA LADAT2D
0.50
0.25
0.00
−0.25
PCoA3 11.13 % variance
−0.4
−0.2
0.0
0.2
0.4
PCoA2 11.82 % variance
Diagnosis
Control
T1D
T2D
LADA

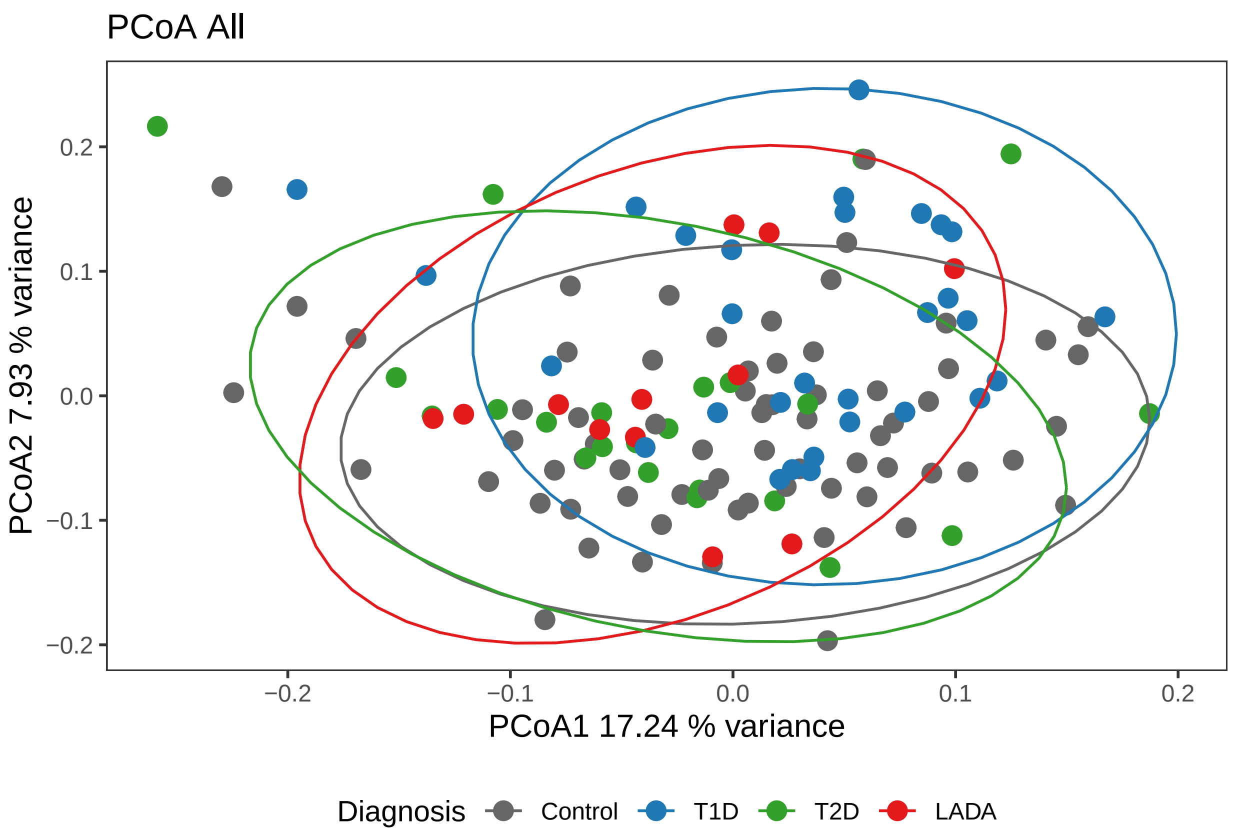
PCoA All
PCoA2 7.93 % variance
0.2
0.1
0.0
−0.1
−0.2
−0.2
−0.1
0.0
0.1
0.2
PCoA1 17.24 % variance
Diagnosis
Control
T1D
T2D
LADA

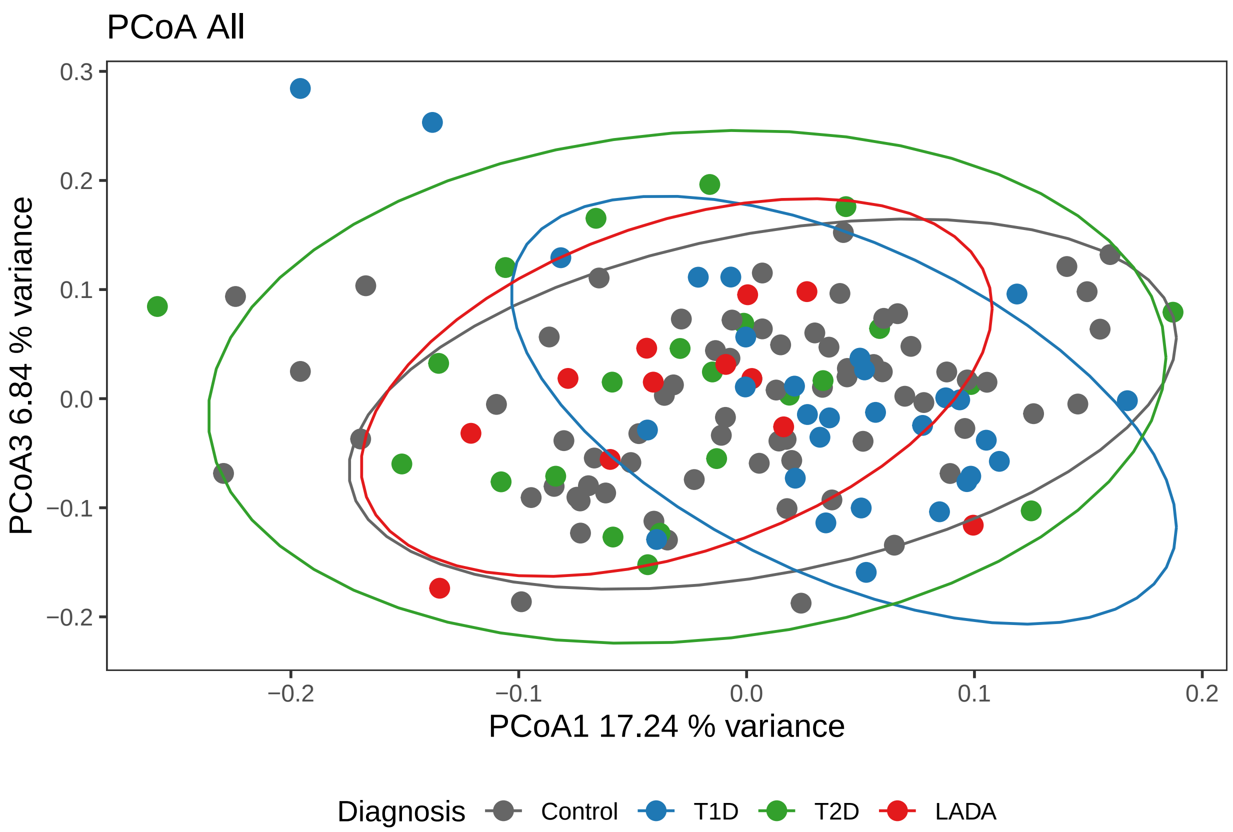
PCoA All
PCoA3 6.84 % variance
PCoA1 17.24 % variance
0.3
0.2
0.1
0.0
−0.1
−0.2
−0.2
−0.1
0.0
0.1
0.2
Diagnosis
Control
T1D
T2D
LADA

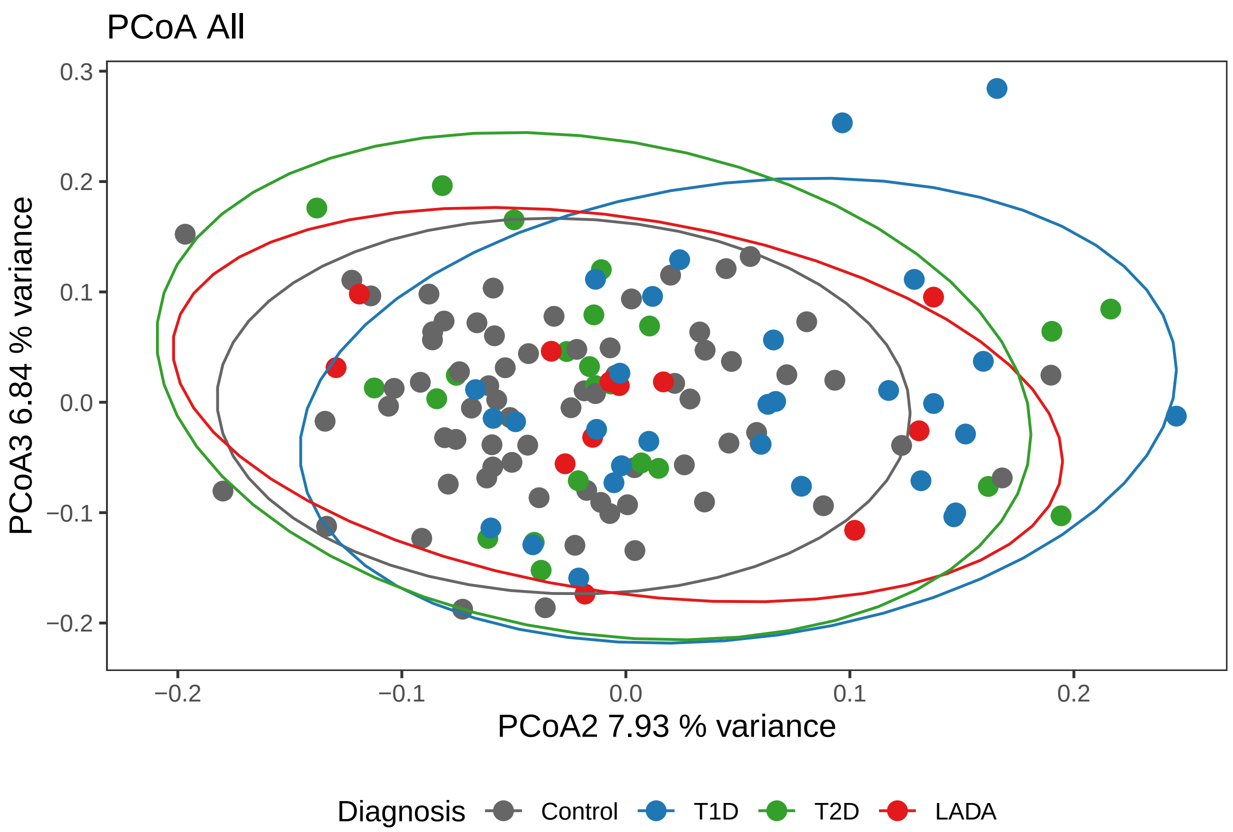
PCoA All
PCoA3 6.84 % variance
0.3
0.2
0.1
0.0
−0.1
−0.2
−0.2
−0.1
0.0
0.1
0.2
PCoA2 7.93 % variance
Diagnosis
Control
T1D
T2D
LADA

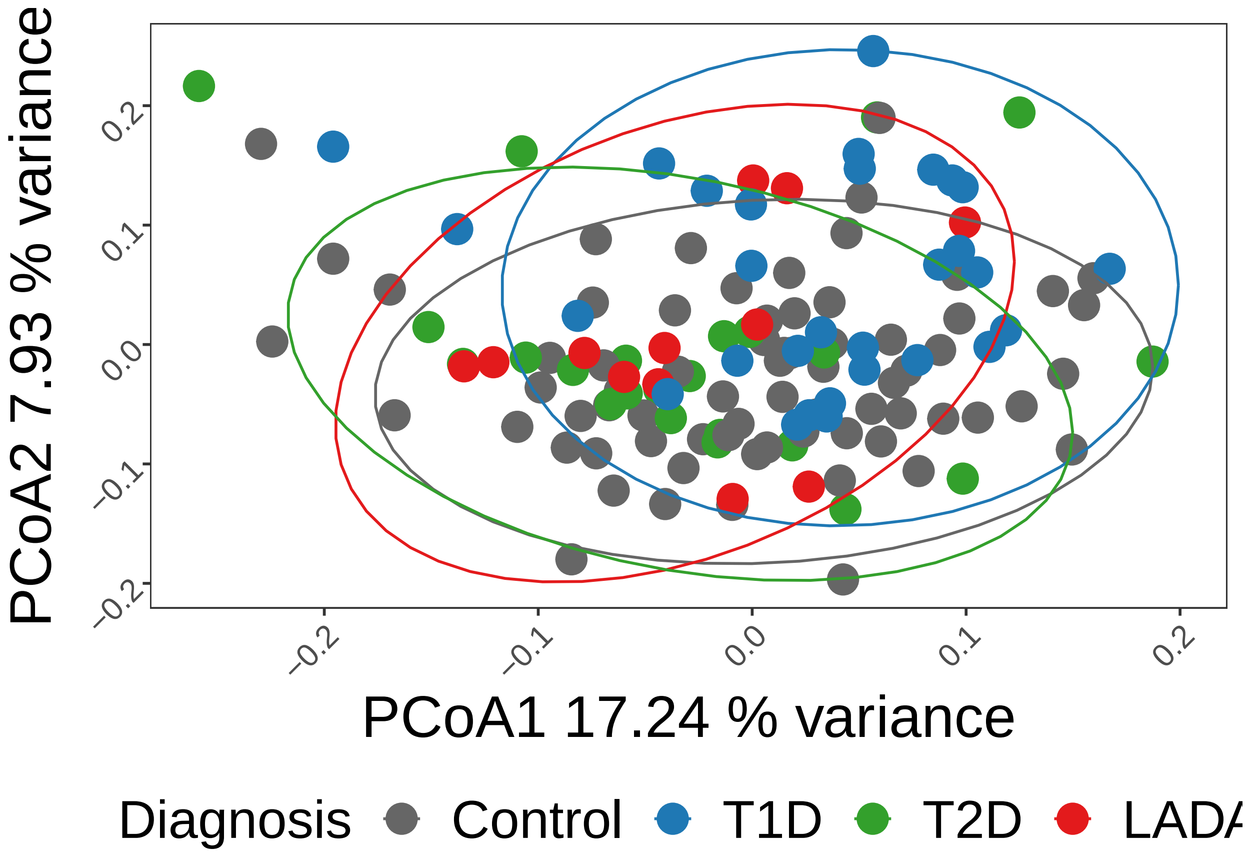

Create figure 1

Investigating grouping diagnosis. Which group does LADA resemble the most and which are different form each other.

```
#Have the plots stored in list
lay <- rbind(c(1,2,3,4),
             c(1,2,3,4),
             c(5,5,5,5),
             c(5,5,5,5))


pdf(paste("MicroLADA_Figure1.pdf", sep=""), width=15, height=15)
grid.arrange(Fig1List$Alpharichness,
             Fig1List$AlphaPielou,
             Fig1List$AlphaShannon,
             Fig1List$DissiBrayHel,
             Fig1List$PCoAall, layout_matrix = lay)
dev.off()
```

```
## pdf
##   2
```

```r
pdf(paste("MicroLADA_Figure1RemMet.pdf", sep=""), width=15, height=15)
grid.arrange(Fig1ListRemMet$Alpharichness,
             Fig1ListRemMet$AlphaPielou,
             Fig1ListRemMet$AlphaShannon,
             Fig1ListRemMet$DissiBrayHel,
             Fig1ListRemMet$PCoAall, layout_matrix = lay)
dev.off()
```

```
## pdf
##   2
```

PERMANOVA

Permutational Multivariate ANOVA based on dissimilarities (Bray-Curtis, Hellinger transformed data) using vegan::adonis
Added to figure 1 using inkscape.

```r
rm(list=setdiff(ls(), c("Metadata", "MetadataMed", "Feature", "TaxonomyDA", "Taxonomy")))

#Hellinger transformation
Taxonomy2 <- data.frame(t(decostand(t(Taxonomy), method="hellinger")))
#Maks TSS
Taxonomy2<-sweep(Taxonomy2, 2, colSums(Taxonomy2), FUN="/")

#Dissimilarity
distmatrix <- vegdist(t(Taxonomy2), method="bray")

#adonis can handle both continous and factor predictors
set.seed(1)
adonisObject<-adonis2(distmatrix ~ Diagnosis, Metadata, by="terms",
                      perm=999) #, perm=999 can increase to get exact p-values
adonisObject #If significant then difference between groups
```

```
## Permutation test for adonis under reduced model
## Terms added sequentially (first to last)
## Permutation: free
## Number of permutations: 999
##
## adonis2(formula = distmatrix ~ Diagnosis, data = Metadata, permutations = 999, by = "terms")
##            Df SumOfSqs      R2      F Pr(>F)
## Diagnosis   3   1.1356 0.04669 3.6899  0.001 ***
## Residual  226  23.1839 0.95331
## Total     229  24.3195 1.00000
## ---
## Signif. codes:  0 '***' 0.001 '**' 0.01 '*' 0.05 '.' 0.1 ' ' 1
```

```r
#With Metformin
set.seed(1)
adonisObject<-adonis2(distmatrix ~ Diagnosis + Metformin, Metadata, by="terms",
                      perm=999) #, perm=999 can increase to get exact p-values
adonisObject #If significant then difference between groups
```

```
## Permutation test for adonis under reduced model
## Terms added sequentially (first to last)
## Permutation: free
## Number of permutations: 999
##
## adonis2(formula = distmatrix ~ Diagnosis + Metformin, data = Metadata, permutations = 999, by = "ter
##            Df SumOfSqs      R2      F Pr(>F)
## Diagnosis   3   1.1356 0.04669 3.7086  0.001 ***
## Metformin   1   0.2187 0.00899 2.1428  0.016 *
## Residual  225  22.9652 0.94431
## Total     229  24.3195 1.00000
## ---
## Signif. codes:  0 '***' 0.001 '**' 0.01 '*' 0.05 '.' 0.1 ' ' 1
```

```
set.seed(1)
adonisObject<-adonis2(distmatrix ~ Diagnosis * Metformin, Metadata, by="terms",
                      perm=999) #, perm=999 can increase to get exact p-values
adonisObject #If significant then difference between groups
```

```
## Permutation test for adonis under reduced model
## Terms added sequentially (first to last)
## Permutation: free
## Number of permutations: 999
##
## adonis2(formula = distmatrix ~ Diagnosis * Metformin, data = Metadata, permutations = 999, by = "ter
##                      Df SumOfSqs      R2      F Pr(>F)
## Diagnosis             3   1.1356 0.04669 3.7067  0.001 ***
## Metformin             1   0.2187 0.00899 2.1417  0.016 *
## Diagnosis:Metformin   1   0.0907 0.00373 0.8880  0.566
## Residual            224  22.8745 0.94058
## Total               229  24.3195 1.00000
## ---
## Signif. codes:  0 '***' 0.001 '**' 0.01 '*' 0.05 '.' 0.1 ' ' 1
```

```
#With Metformin first in the model formula
set.seed(1)
adonisObject<-adonis2(distmatrix ~ Metformin + Diagnosis, Metadata, by="terms",
                      perm=999) #, perm=999 can increase to get exact p-values
adonisObject #If significant then difference between groups
```

```
## Permutation test for adonis under reduced model
## Terms added sequentially (first to last)
## Permutation: free
## Number of permutations: 999
##
## adonis2(formula = distmatrix ~ Metformin + Diagnosis, data = Metadata, permutations = 999, by = "ter
##            Df SumOfSqs      R2      F Pr(>F)
## Metformin   1   0.8017 0.03297 7.8551  0.001 ***
## Diagnosis   3   0.5525 0.02272 1.8045  0.008 **
## Residual  225  22.9652 0.94431
## Total     229  24.3195 1.00000
## ---
## Signif. codes:  0 '***' 0.001 '**' 0.01 '*' 0.05 '.' 0.1 ' ' 1
```

```
#Actual test reported in figure 1
#With by = margin does not matter the order
set.seed(1)
adonisObject<-adonis2(distmatrix ~ Metformin + Diagnosis, Metadata, by="margin",
                      perm=999) #, perm=999 can increase to get exact p-values
adonisObject #If significant then difference between groups
```

```
## Permutation test for adonis under reduced model
## Marginal effects of terms
## Permutation: free
## Number of permutations: 999
##
## adonis2(formula = distmatrix ~ Metformin + Diagnosis, data = Metadata, permutations = 999, by = "marg
##            Df SumOfSqs      R2      F Pr(>F)
## Metformin   1   0.2187 0.00899 2.1428  0.016 *
## Diagnosis   3   0.5525 0.02272 1.8045  0.008 **
## Residual  225  22.9652 0.94431
## Total     229  24.3195 1.00000
## ---
## Signif. codes:  0 '***' 0.001 '**' 0.01 '*' 0.05 '.' 0.1 ' ' 1
```

```
#Include BMI
set.seed(1)
adonisObject<-adonis2(distmatrix ~ Metformin + Diagnosis + BMI, Metadata, by="margin",
                      perm=999) #, perm=999 can increase to get exact p-values
adonisObject #If significant then difference between groups
```

```
## Permutation test for adonis under reduced model
## Marginal effects of terms
## Permutation: free
## Number of permutations: 999
##
## adonis2(formula = distmatrix ~ Metformin + Diagnosis + BMI, data = Metadata, permutations = 999, by =
##            Df SumOfSqs      R2      F Pr(>F)
## Metformin   1   0.2173 0.00894 2.1314  0.016 *
## Diagnosis   3   0.5207 0.02141 1.7023  0.011 *
## BMI         1   0.1249 0.00514 1.2252  0.208
## Residual  224  22.8403 0.93918
## Total     229  24.3195 1.00000
## ---
## Signif. codes:  0 '***' 0.001 '**' 0.01 '*' 0.05 '.' 0.1 ' ' 1
```

```
set.seed(1)
adonisObject<-adonis2(distmatrix ~ Metformin + Diagnosis + BMIord, Metadata, by="margin",
                      perm=999) #, perm=999 can increase to get exact p-values
adonisObject #If significant then difference between groups
```

```
## Permutation test for adonis under reduced model
## Marginal effects of terms
## Permutation: free
## Number of permutations: 999
##
```

```
## adonis2(formula = distmatrix ~ Metformin + Diagnosis + BMIord, data = Metadata, permutations = 999, 
##            Df SumOfSqs      R2      F Pr(>F)
## Metformin   1   0.2115 0.00870 2.0647  0.018 *
## Diagnosis   3   0.5096 0.02095 1.6584  0.010 **
## BMIord      5   0.4329 0.01780 0.8454  0.816
## Residual  220  22.5323 0.92651
## Total     229  24.3195 1.00000
## ---
## Signif. codes:  0 '***' 0.001 '**' 0.01 '*' 0.05 '.' 0.1 ' ' 1
```

```r
set.seed(1)
adonisObject<-adonis2(distmatrix ~ Metformin + Diagnosis + BMIclass, Metadata, by="margin",
                      perm=999) #, perm=999 can increase to get exact p-values
adonisObject #If significant then difference between groups
```

```
## Permutation test for adonis under reduced model
## Marginal effects of terms
## Permutation: free
## Number of permutations: 999
##
## adonis2(formula = distmatrix ~ Metformin + Diagnosis + BMIclass, data = Metadata, permutations = 999
##            Df SumOfSqs      R2      F Pr(>F)
## Metformin   1   0.2145 0.00882 2.1015  0.017 *
## Diagnosis   3   0.5260 0.02163 1.7174  0.009 **
## BMIclass    2   0.1987 0.00817 0.9730  0.496
## Residual  223  22.7666 0.93614
## Total     229  24.3195 1.00000
## ---
## Signif. codes:  0 '***' 0.001 '**' 0.01 '*' 0.05 '.' 0.1 ' ' 1
```

```r
#set.seed(1)
#adonisObject<-adonis2(distmatrix ~ Metformin + Diagnosis + BMIordclass, Metadata, by="margin",
#                      perm=999) #, perm=999 can increase to get exact p-values
#adonisObject #If significant then difference between groups same ass BMIclass

set.seed(1)
adonisObject<-adonis2(distmatrix ~ Metformin + Diagnosis + BMIq, Metadata, by="margin",
                      perm=999) #, perm=999 can increase to get exact p-values
adonisObject #If significant then difference between groups
```

```
## Permutation test for adonis under reduced model
## Marginal effects of terms
## Permutation: free
## Number of permutations: 999
##
## adonis2(formula = distmatrix ~ Metformin + Diagnosis + BMIq, data = Metadata, permutations = 999, by
##            Df SumOfSqs      R2      F Pr(>F)
## Metformin   1   0.2174 0.00894 2.1309  0.016 *
## Diagnosis   3   0.5321 0.02188 1.7384  0.009 **
## BMIq        1   0.1129 0.00464 1.1068  0.294
## Residual  224  22.8523 0.93967
## Total     229  24.3195 1.00000
## ---
## Signif. codes:  0 '***' 0.001 '**' 0.01 '*' 0.05 '.' 0.1 ' ' 1
```

```r
set.seed(1)
adonisObject<-adonis2(distmatrix ~ Metformin * Diagnosis * BMIq, Metadata, by="margin",
                      perm=999) #, perm=999 can increase to get exact p-values
adonisObject #If significant then difference between groups
```

```
## Permutation test for adonis under reduced model
## Marginal effects of terms
## Permutation: free
## Number of permutations: 999
##
## adonis2(formula = distmatrix ~ Metformin * Diagnosis * BMIq, data = Metadata, permutations = 999, by
##                           Df SumOfSqs      R2      F Pr(>F)
## Metformin:Diagnosis:BMIq   1   0.0744 0.00306 0.7278  0.775
## Residual                 218  22.2849 0.91634
## Total                    229  24.3195 1.00000
```

```r
set.seed(1)
adonisObject<-adonis2(distmatrix ~ Diagnosis + BMIq, Metadata, by="margin",
                      perm=999) #, perm=999 can increase to get exact p-values
adonisObject #If significant then difference between groups
```

```
## Permutation test for adonis under reduced model
## Marginal effects of terms
## Permutation: free
## Number of permutations: 999
##
## adonis2(formula = distmatrix ~ Diagnosis + BMIq, data = Metadata, permutations = 999, by = "margin")
##            Df SumOfSqs      R2      F Pr(>F)
## Diagnosis   3   0.9694 0.03986 3.1514  0.001 ***
## BMIq        1   0.1142 0.00470 1.1141  0.288
## Residual  225  23.0697 0.94861
## Total     229  24.3195 1.00000
## ---
## Signif. codes:  0 '***' 0.001 '**' 0.01 '*' 0.05 '.' 0.1 ' ' 1
```

```r
## Evaluating the model assumptions
TestModel <- with(Metadata, betadisper(distmatrix, Diagnosis)) #Can not run
  #betadisper with multiple independant variables
#TestModel
#plot(TestModel)
plot(TestModel, label=FALSE)
```

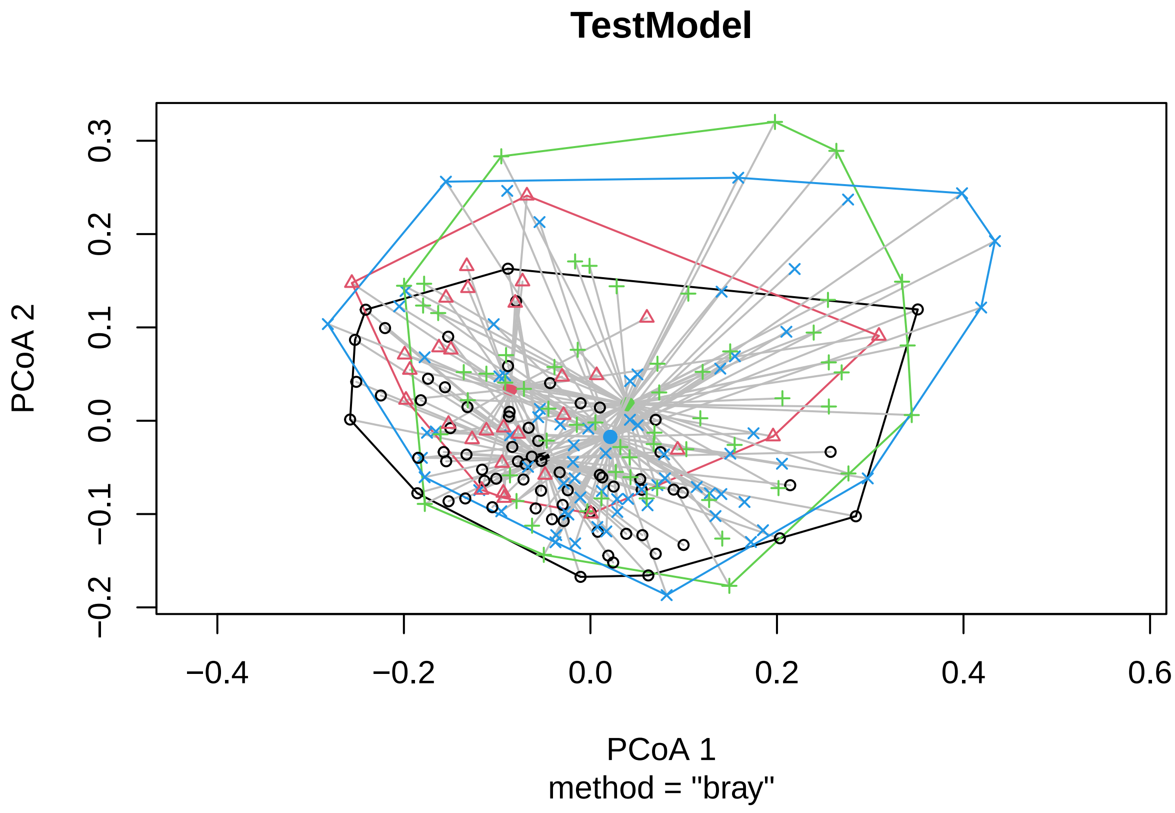


```
boxplot(TestModel)
```

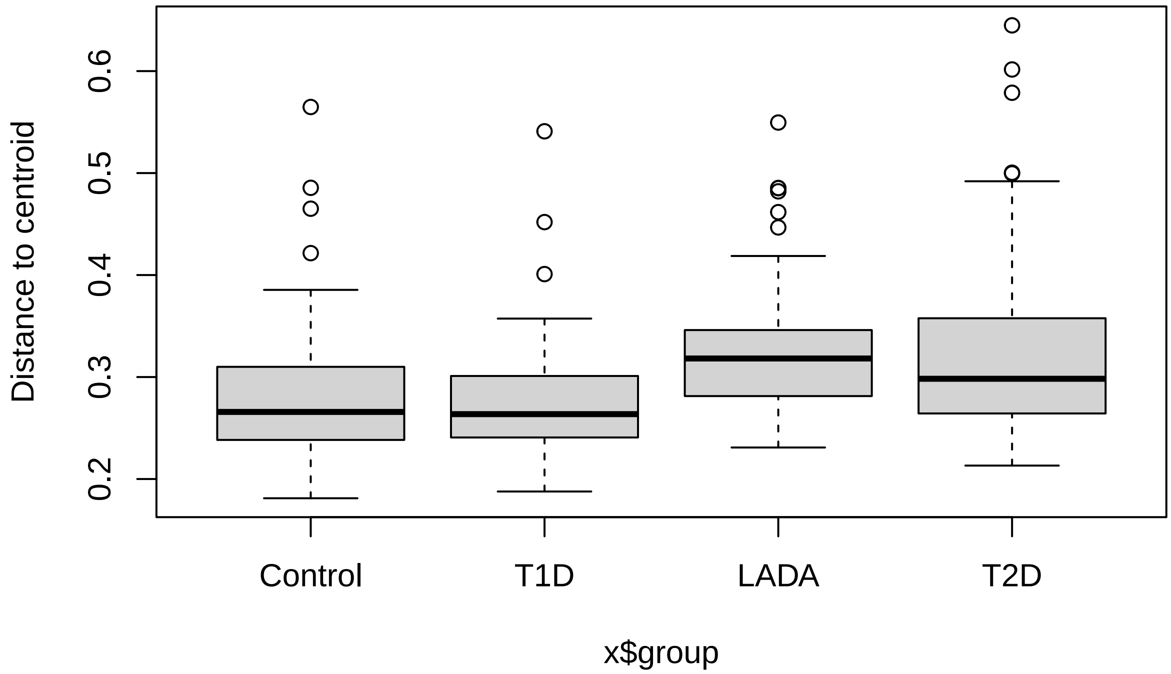


```r
anova(TestModel) #p>0.05 -> Assumption met
```

```
## Analysis of Variance Table
##
## Response: Distances
##            Df  Sum Sq  Mean Sq F value    Pr(>F)
## Groups      3 0.11234 0.037446  6.2162 0.0004486 ***
## Residuals 226 1.36141 0.006024
## ---
## Signif. codes:  0 '***' 0.001 '**' 0.01 '*' 0.05 '.' 0.1 ' ' 1
```

```r
#permutest(TestModel)

table(Metadata$Metformin, Metadata$Diagnosis)
```

```
##
##     Control T1D LADA T2D
##   0      70  30   12  23
##   1       0   0   48  47
```

PERMANOVA remove metformin

```r
rm(list=setdiff(ls(), c("Metadata", "MetadataMed", "Feature", "TaxonomyDA", "Taxonomy")))

##Removing treated with metformin
#Metadata$Metformin <- ifelse(is.na(Metadata$Metformin), "Unknown", Metadata$Metformin)
Metadata2<-filter(Metadata, !Metformin == 1)
##Applying subsetting to OTU tables
Taxonomy2<-dplyr::select(Taxonomy, one_of(as.character(Metadata2$MicrobiomeID)))
##Remove orgs that are not present after subsetting.
Taxonomy2 <- Taxonomy2[rowSums(Taxonomy2)>0,]


#Hellinger transformation
Taxonomy2 <- data.frame(t(decostand(t(Taxonomy2), method="hellinger")))
#Maks TSS
Taxonomy2<-sweep(Taxonomy2, 2, colSums(Taxonomy2), FUN="/")

#Dissimilarity
distmatrix <- vegdist(t(Taxonomy2), method="bray")

set.seed(1)
adonisObject<-adonis2(distmatrix ~ Diagnosis, Metadata2, by="margin",
                      perm=999) #, perm=999 can increase to get exact p-values
adonisObject #If significant then difference between groups
```

```
## Permutation test for adonis under NA model
## Marginal effects of terms
## Permutation: free
## Number of permutations: 999
##
## adonis2(formula = distmatrix ~ Diagnosis, data = Metadata2, permutations = 999, by = "margin")
##            Df SumOfSqs      R2      F Pr(>F)
## Diagnosis   3   0.5289 0.04185 1.9072  0.001 ***
## Residual  131  12.1109 0.95815
## Total     134  12.6399 1.00000
## ---
## Signif. codes:  0 '***' 0.001 '**' 0.01 '*' 0.05 '.' 0.1 ' ' 1
```

```r
set.seed(1)
adonisObject<-adonis2(distmatrix ~ Diagnosis + BMIq, Metadata2, by="margin",
                      perm=999) #, perm=999 can increase to get exact p-values
adonisObject #If significant then difference between groups
```

```
## Permutation test for adonis under reduced model
## Marginal effects of terms
## Permutation: free
## Number of permutations: 999
##
## adonis2(formula = distmatrix ~ Diagnosis + BMIq, data = Metadata2, permutations = 999, by = "margin")
##            Df SumOfSqs      R2      F Pr(>F)
## Diagnosis   3   0.4900 0.03876 1.7652  0.004 **
## BMIq        1   0.0827 0.00654 0.8935  0.539
## Residual  130  12.0282 0.95161
```

```
## Total      134  12.6399 1.00000
## ---
## Signif. codes:  0 '***' 0.001 '**' 0.01 '*' 0.05 '.' 0.1 ' ' 1
```

```r
## Evaluating the model assumptions
TestModel <- with(Metadata2, betadisper(distmatrix, Diagnosis)) #Can not run
  #betadisper with multiple independant variables
#TestModel
#plot(TestModel)
plot(TestModel, label=FALSE)
```

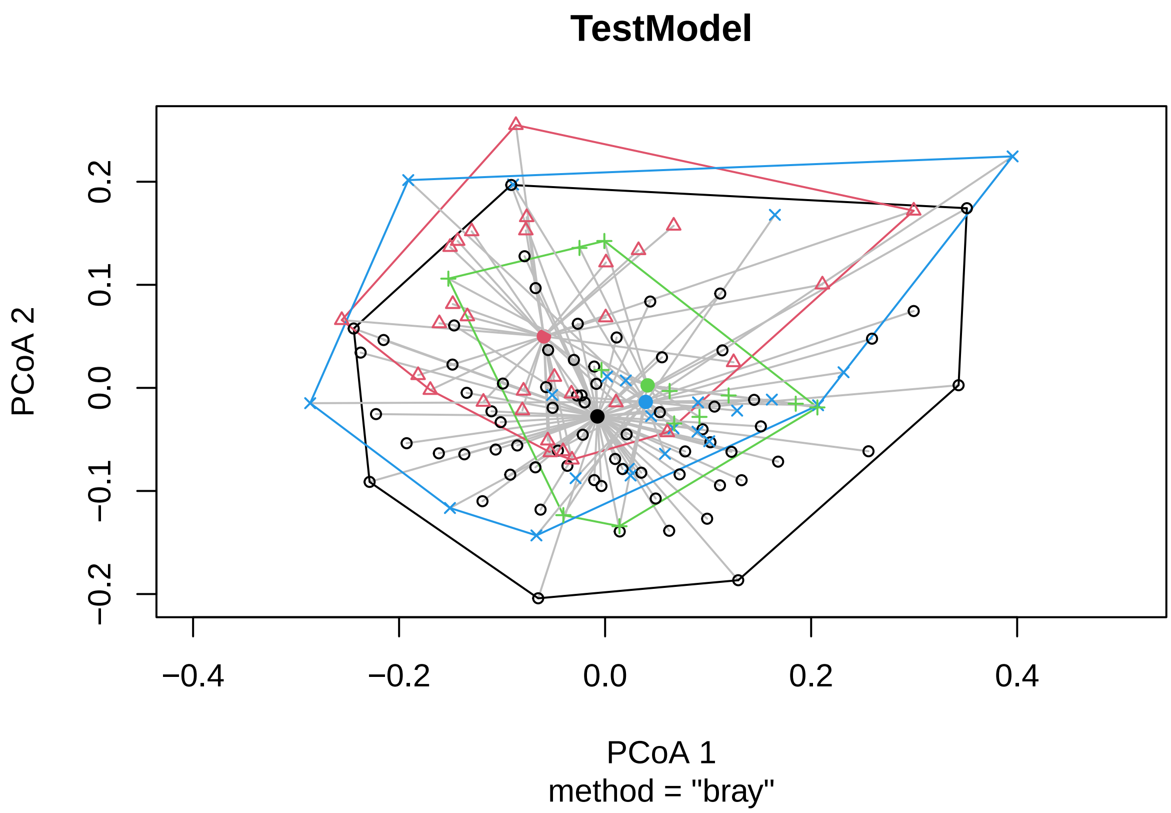


```r
boxplot(TestModel)
```

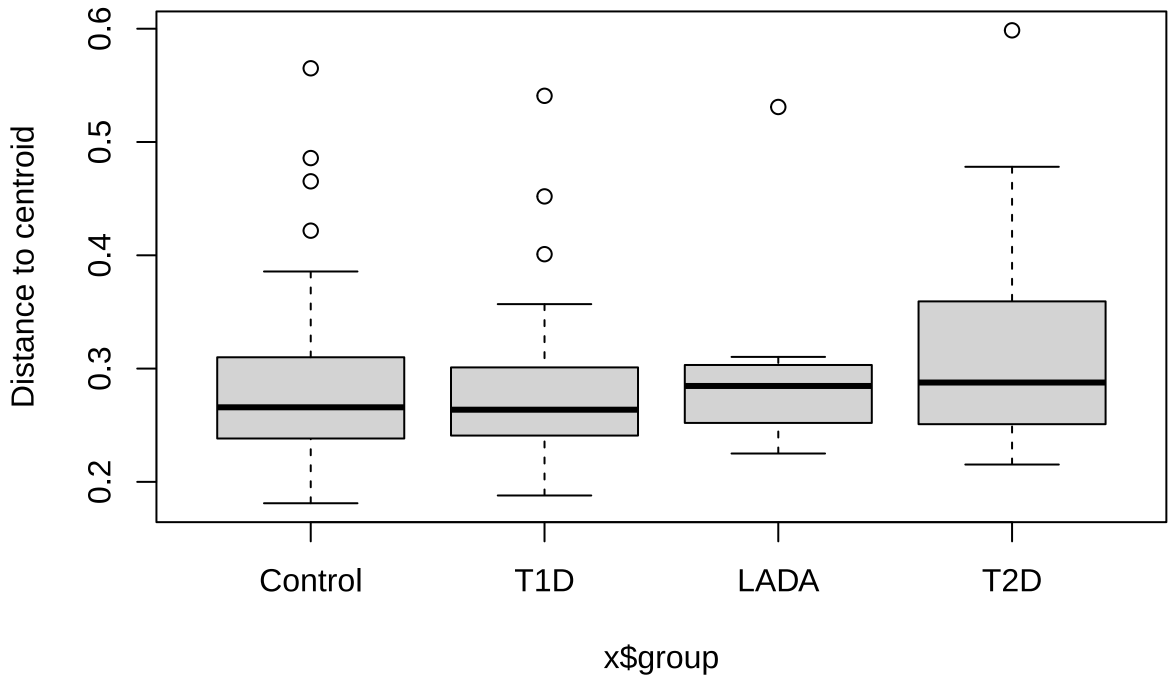


```
anova(TestModel) #p>0.05 -> Assumption met
```

```
## Analysis of Variance Table
##
## Response: Distances
##            Df  Sum Sq   Mean Sq F value Pr(>F)
## Groups      3 0.02961 0.0098715    1.74  0.162
## Residuals 131 0.74318 0.0056732
```

```
#permutest(TestModel)

table(Metadata2$Metformin, Metadata2$Diagnosis)
```

```
##
##     Control T1D LADA T2D
##   0      70  30   12  23
```

PERMANOVA MED subset

```
rm(list=setdiff(ls(), c("Metadata", "MetadataMed", "Feature", "TaxonomyDA", "Taxonomy")))

##Metadatamed
```

```r
##Applying subsetting to OTU tables
Taxonomy2<-dplyr::select(Taxonomy, one_of(as.character(MetadataMed$MicrobiomeID)))

#Hellinger transformation
Taxonomy2 <- data.frame(t(decostand(t(Taxonomy2), method="hellinger")))
#Maks TSS
Taxonomy2<-sweep(Taxonomy2, 2, colSums(Taxonomy2), FUN="/")

#Dissimilarity
distmatrix <- vegdist(t(Taxonomy2), method="bray")

#With by = margin does not matter the order
set.seed(1)
adonisObject<-adonis2(distmatrix ~ Diagnosis + BMIq, MetadataMed, by="margin",
                      perm=999) #, perm=999 can increase to get exact p-values
adonisObject #If significant then difference between groups
```

```
## Permutation test for adonis under reduced model
## Marginal effects of terms
## Permutation: free
## Number of permutations: 999
##
## adonis2(formula = distmatrix ~ Diagnosis + BMIq, data = MetadataMed, permutations = 999, by = "margi
##            Df SumOfSqs      R2      F Pr(>F)
## Diagnosis   2   0.6507 0.03616 2.9590  0.001 ***
## BMIq        1   0.1426 0.00793 1.2969  0.174
## Residual  156  17.1534 0.95328
## Total     159  17.9940 1.00000
## ---
## Signif. codes:  0 '***' 0.001 '**' 0.01 '*' 0.05 '.' 0.1 ' ' 1
```

```r
set.seed(1)
adonisObject<-adonis2(distmatrix ~ Metformin + Diagnosis, MetadataMed, by="margin",
                      perm=999) #, perm=999 can increase to get exact p-values
adonisObject #If significant then difference between groups
```

```
## Permutation test for adonis under reduced model
## Marginal effects of terms
## Permutation: free
## Number of permutations: 999
##
## adonis2(formula = distmatrix ~ Metformin + Diagnosis, data = MetadataMed, permutations = 999, by = "
##            Df SumOfSqs      R2      F Pr(>F)
## Metformin   1   0.2187 0.01215 1.9979  0.020 *
## Diagnosis   2   0.4030 0.02240 1.8406  0.011 *
## Residual  156  17.0773 0.94905
## Total     159  17.9940 1.00000
## ---
## Signif. codes:  0 '***' 0.001 '**' 0.01 '*' 0.05 '.' 0.1 ' ' 1
```

```r
set.seed(1)
adonisObject<-adonis2(distmatrix ~ Diagnosis + Metformin + BMIq , MetadataMed, by="margin",
```

```r
                         perm=999) #, perm=999 can increase to get exact p-values
adonisObject #If significant then difference between groups
```

```
## Permutation test for adonis under reduced model
## Marginal effects of terms
## Permutation: free
## Number of permutations: 999
##
## adonis2(formula = distmatrix ~ Diagnosis + Metformin + BMIq, data = MetadataMed, permutations = 999,
##            Df SumOfSqs      R2      F Pr(>F)
## Diagnosis   2   0.4018 0.02233 1.8390  0.011 *
## Metformin   1   0.2218 0.01233 2.0304  0.018 *
## BMIq        1   0.1457 0.00810 1.3337  0.154
## Residual  155  16.9316 0.94096
## Total     159  17.9940 1.00000
## ---
## Signif. codes:  0 '***' 0.001 '**' 0.01 '*' 0.05 '.' 0.1 ' ' 1
```

```r
set.seed(1)
adonisObject<-adonis2(distmatrix ~ Diagnosis + Metformin + BMIq + med_insulin + med_statins + med_proton
                         perm=999) #, perm=999 can increase to get exact p-values
adonisObject #If significant then difference between groups
```

```
## Permutation test for adonis under reduced model
## Marginal effects of terms
## Permutation: free
## Number of permutations: 999
##
## adonis2(formula = distmatrix ~ Diagnosis + Metformin + BMIq + med_insulin + med_statins + med_protonp
##                           Df SumOfSqs      R2      F Pr(>F)
## Diagnosis                  2   0.3261 0.01812 1.4917  0.052 .
## Metformin                  1   0.1940 0.01078 1.7742  0.038 *
## BMIq                       1   0.1436 0.00798 1.3136  0.160
## med_insulin                1   0.0934 0.00519 0.8545  0.607
## med_statins                1   0.1179 0.00655 1.0784  0.308
## med_protonpump_inhibitor   1   0.0980 0.00545 0.8964  0.553
## Residual                 152  16.6159 0.92341
## Total                    159  17.9940 1.00000
## ---
## Signif. codes:  0 '***' 0.001 '**' 0.01 '*' 0.05 '.' 0.1 ' ' 1
```

```r
## Evaluating the model assumptions
TestModel <- with(MetadataMed, betadisper(distmatrix, Diagnosis)) #Can not run
  #betadisper with multiple independant variables
#TestModel
#plot(TestModel)
plot(TestModel, label=FALSE)
```

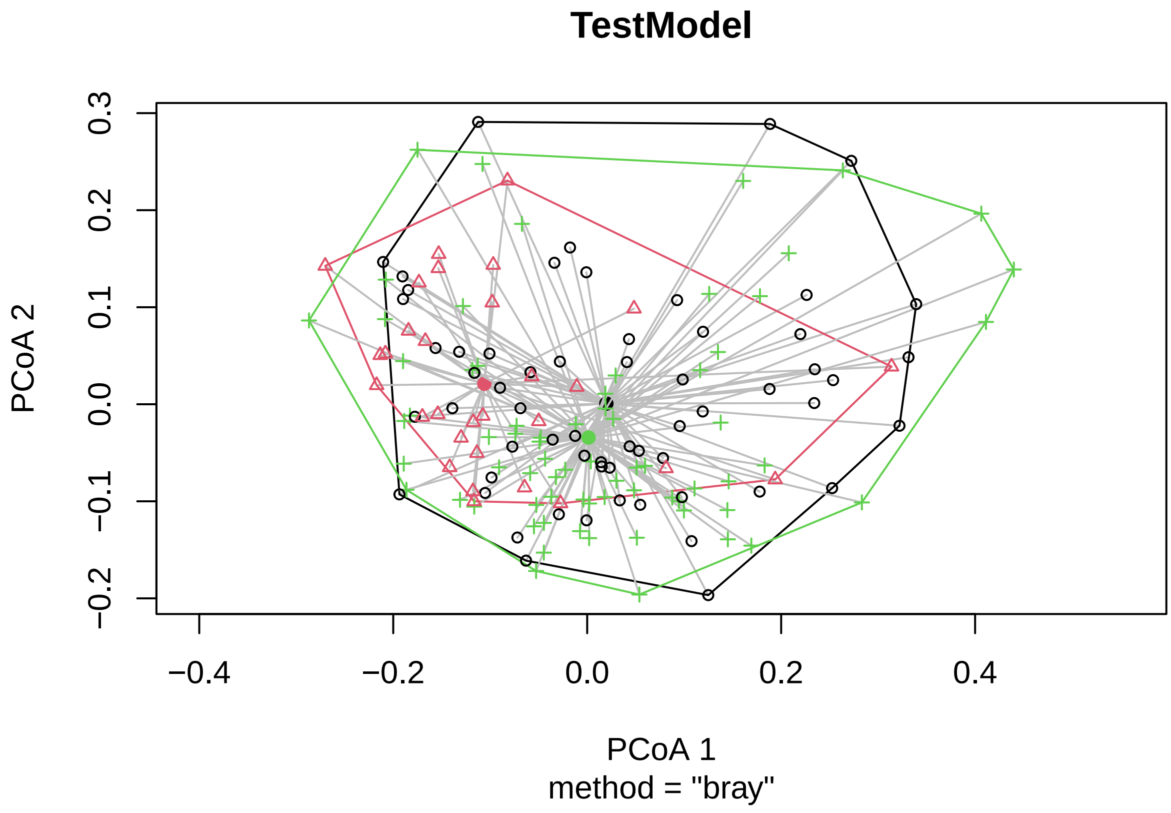


```
boxplot(TestModel)
```

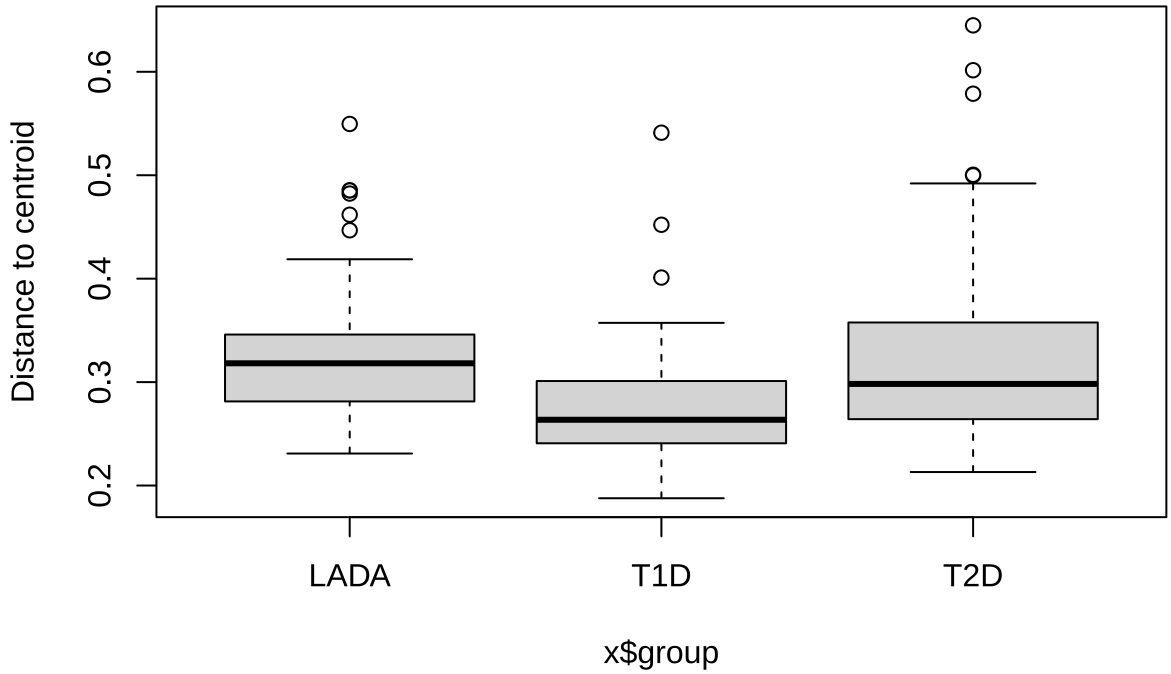


```r
anova(TestModel) #p>0.05 -> Assumption met
```

```
## Analysis of Variance Table
##
## Response: Distances
##            Df  Sum Sq   Mean Sq F value Pr(>F)
## Groups      2 0.04655 0.0232754  3.5285 0.0317 *
## Residuals 157 1.03563 0.0065964
## ---
## Signif. codes:  0 '***' 0.001 '**' 0.01 '*' 0.05 '.' 0.1 ' ' 1
```

```r
#permutest(TestModel)
```

Pairwise PERMANOVA

Pairwise adonis, found this function from Pedro Martinez Arbizu on researchgate (https://www.researchgate.net/post/How_can_I_do_PerMANOVA_pairwise_contrasts_in_R) He also made implementation of adonis2 https://github.com/pmartinezarbizu/pairwiseAdonis

```r
rm(list=setdiff(ls(), c("Metadata", "MetadataMed", "Feature", "TaxonomyDA", "Taxonomy")))

##Initial normalization/transformation the function runs method from adonis passed from
##vegdist
#Hellinger transformation
```

```r
Taxonomy2 <- data.frame(t(decostand(t(Taxonomy), method="hellinger")))
#Maks TSS
Taxonomy2<-sweep(Taxonomy2, 2, colSums(Taxonomy2), FUN="/")

##Testing function
#x <- t(Taxonomy2)
#factors <- as.character(Metadata$Diagnosis)
#sim.method <- 'bray'
#p.adjust.m <- 'bonferroni'
#elem<-1

pairwise.adonis <- function(x, factors, sim.method = 'bray', p.adjust.m ='bonferroni')
#x = the community table
#factors = a column or vector with all factors to be tested pairwise
#sim.method = similarity function, one of the functions available in vegdist(); default is
#'bray' for bray-curtis.
#p.adjust.m = the p.value correction method, one of the methods supported by p.adjust();
#default is 'bonferroni'
{
#library(vegan)
co = combn(unique(factors),2)
pairs = c()
F.Model =c()
R2 = c()
p.value = c()
for(elem in 1:ncol(co)){
ad = adonis(x[factors %in% c(co[1,elem],co[2,elem]),] ~
              factors[factors %in% c(co[1,elem],co[2,elem])] ,
            method =sim.method); #Extracting a matrix only containing samples of a pair
pairs = c(pairs,paste(co[1,elem],'vs',co[2,elem]));
F.Model =c(F.Model,ad$aov.tab[1,4]);
R2 = c(R2,ad$aov.tab[1,5]);
p.value = c(p.value,ad$aov.tab[1,6])
}
p.adjusted = p.adjust(p.value,method=p.adjust.m) #adjusting after all comparisons
pairw.res = data.frame(pairs,F.Model,R2,p.value,p.adjusted)
return(pairw.res)
}

pairwise.adonis(t(Taxonomy2), as.character(Metadata$Diagnosis))
```

```
##              pairs   F.Model          R2 p.value p.adjusted
## 1  T2D vs Control 3.7595007 0.026520273   0.001      0.006
## 2     T2D vs LADA 0.9943077 0.007708152   0.399      1.000
## 3      T2D vs T1D 4.6878365 0.045651332   0.001      0.006
## 4 Control vs LADA 5.4738620 0.041010741   0.001      0.006
## 5  Control vs T1D 3.0663286 0.030339764   0.002      0.012
## 6     LADA vs T1D 4.8712429 0.052451574   0.001      0.006
```

```r
pairw.res<-pairwise.adonis(t(Taxonomy2), as.character(Metadata$Diagnosis))


##Implementation of adonis2 can not get to work
```

```r
##My implementation of adonis2 for my data when running with diagnosis

##Testing function
x <- t(Taxonomy2)
factors <- as.character(Metadata$Diagnosis)
factors2 <- as.character(Metadata$Metformin)
sim.method <- 'bray'
p.adjust.m <- 'bonferroni'
#elem<-1
#elem<-5

#pairwise.adonis.multiplevar <- function(x, factors, factors2, sim.method = 'bray',
#p.adjust.m ='bonferroni')
#x = the community table
#factors = a column or vector with all factors to be tested pairwise
#sim.method = similarity function, one of the functions available in vegdist(); default is
#'bray' for bray-curtis
#p.adjust.m = the p.value correction method, one of the methods supported by p.adjust();
#default is 'bonferroni'
#{
#library(vegan)
co = combn(unique(factors),2)
pairs = c()
F.Model =c()
R2 = c()
p.value = c()
F.Model.2 =c()
R2.2 = c()
p.value.2 = c()
#for(elem in 1:ncol(co)){
for(elem in c(1:4,6)){
ad = adonis2(x[factors %in% c(co[1,elem],co[2,elem]),] ~
               factors[factors %in% c(co[1,elem],co[2,elem])] +
               factors2[factors %in% c(co[1,elem],co[2,elem])],
             method =sim.method, by="margin"); #Extracting a matrix only containing
                                               #samples of a pair
pairs = c(pairs,paste(co[1,elem],'vs',co[2,elem]));
F.Model =c(F.Model,ad$F[1]);
R2 = c(R2,ad$R2[1]);
p.value = c(p.value,ad$`Pr(>F)`[1])
F.Model.2 =c(F.Model.2,ad$F[2])
R2.2 = c(R2.2,ad$R2[2])
p.value.2 = c(p.value.2,ad$`Pr(>F)`[2])
}
p.adjusted = p.adjust(p.value,method=p.adjust.m)
p.adjusted.2 = p.adjust(p.value.2,method=p.adjust.m)
pairw.res.2 = data.frame(pairs, F.Model, R2, p.value, p.adjusted,
                         F.Model.2, R2.2, p.value.2, p.adjusted.2)
#return(pairw.res)
#}

#pairwise.adonis.multiplevar(t(Taxonomy2), factors = as.character(Metadata$Diagnosis),
#                             factors2 = as.character(Metadata$Metformin),
```

```
#                             sim.method = 'bray', p.adjust.m ='bonferroni')

pairw.res.2
```

```
##              pairs  F.Model          R2 p.value p.adjusted F.Model.2        R2.2
## 1  T2D vs Control 1.255319 0.008855077   0.194      0.970 1.0030931 0.007075862
## 2     T2D vs LADA 0.884964 0.006811912   0.568      1.000 1.9127888 0.014723479
## 3      T2D vs T1D 2.428035 0.023660398   0.009      0.045 0.9354444 0.009115598
## 4 Control vs LADA 1.068822 0.007937252   0.329      1.000 2.1365222 0.015866169
## 5     LADA vs T1D 1.835182 0.019537745   0.038      0.190 2.0032817 0.021327376
##   p.value.2 p.adjusted.2
## 1     0.406        1.000
## 2     0.023        0.115
## 3     0.517        1.000
## 4     0.010        0.050
## 5     0.023        0.115
```

```
write.table(pairw.res, file="adonis_pairwisediagnosis.txt",
            quote = F, row.names = F, sep="\t")
write.table(pairw.res.2, file="adonis2_pairwisediagnosiswvarmet.txt",
            quote = F, row.names = F, sep="\t")
```

Pairwise PERMANOVA remove metformin

```
rm(list=setdiff(ls(), c("Metadata", "MetadataMed", "Feature", "TaxonomyDA", "Taxonomy")))

##Removing treated with metformin
#Metadata$Metformin <- ifelse(is.na(Metadata$Metformin), "Unknown", Metadata$Metformin)
Metadata2<-filter(Metadata, !Metformin == 1)
##Applying subsetting to OTU tables
Taxonomy2<-dplyr::select(Taxonomy, one_of(as.character(Metadata2$MicrobiomeID)))
##Remove orgs that are not present after subsetting.
Taxonomy2 <- Taxonomy2[rowSums(Taxonomy2)>0,]

##Initial normalization/transformation the function runs method from adonis passed from
##vegdist
#Hellinger transformation
Taxonomy2 <- data.frame(t(decostand(t(Taxonomy2), method="hellinger")))
#Maks TSS
Taxonomy2<-sweep(Taxonomy2, 2, colSums(Taxonomy2), FUN="/")

##Testing function
#x <- t(Taxonomy2)
#factors <- as.character(Metadata$Diagnosis)
#sim.method <- 'bray'
#p.adjust.m <- 'bonferroni'
#elem<-1

pairwise.adonis <- function(x, factors, sim.method = 'bray', p.adjust.m ='bonferroni')
#x = the community table
#factors = a column or vector with all factors to be tested pairwise
```

```
#sim.method = similarity function, one of the functions available in vegdist(); default is
#'bray' for bray-curtis.
#p.adjust.m = the p.value correction method, one of the methods supported by p.adjust();
#default is 'bonferroni'
{
#library(vegan)
co = combn(unique(factors),2)
pairs = c()
F.Model =c()
R2 = c()
p.value = c()
for(elem in 1:ncol(co)){
ad = adonis(x[factors %in% c(co[1,elem],co[2,elem]),] ~
              factors[factors %in% c(co[1,elem],co[2,elem])] ,
            method =sim.method); #Extracting a matrix only containing samples of a pair
pairs = c(pairs,paste(co[1,elem],'vs',co[2,elem]));
F.Model =c(F.Model,ad$aov.tab[1,4]);
R2 = c(R2,ad$aov.tab[1,5]);
p.value = c(p.value,ad$aov.tab[1,6])
}
p.adjusted = p.adjust(p.value,method=p.adjust.m) #adjusting after all comparisons
pairw.res = data.frame(pairs,F.Model,R2,p.value,p.adjusted)
return(pairw.res)
}

pairwise.adonis(t(Taxonomy2), as.character(Metadata2$Diagnosis))
```

```
##               pairs  F.Model         R2 p.value p.adjusted
## 1  Control vs T2D 1.365293 0.01478146   0.117      0.702
## 2 Control vs LADA 1.191066 0.01466992   0.222      1.000
## 3  Control vs T1D 3.066329 0.03033976   0.001      0.006
## 4     T2D vs LADA 0.698268 0.02072118   0.861      1.000
## 5      T2D vs T1D 2.619818 0.04885914   0.003      0.018
## 6     LADA vs T1D 2.083516 0.04950908   0.013      0.078
```

```
pairw.res<-pairwise.adonis(t(Taxonomy2), as.character(Metadata2$Diagnosis))


##Implementation of adonis2 can not get to work
##Made my implementation of adonis2 for my data when running with diagnosis and
  #in this case the other factor metformin so removed when removing samples with
  #patients treated with metformin


write.table(pairw.res, file="adonis_pairwisediagnosisremovedmetsamples.txt",
            quote = F, row.names = F, sep="\t")
```

Heatmap

```
rm(list=setdiff(ls(), c("Metadata", "MetadataMed", "Feature", "TaxonomyDA", "Taxonomy")))
```

```r
#Add a categorical indicator of sex in Metadata also used in rest of script
Metadata$Sex<-as.factor(ifelse(grepl("1", Metadata$sex), "Male",
                               ifelse(grepl("2", Metadata$sex), "Female",
                                      "hmmmmm")))

#The organisms clustering
OrgCluster<-"correlation"

#How many organisms to include in heatmap
Orgs<-25 #Write a number 20-50 seems appropiate for readability

HeatmapExplainers<-c("Sex", "Metformin", "Diagnosis")

#Make list of annotation colors
annotation_colorsNew = list(Diagnosis = c(Control = "#666666", T1D = "#1F78B4",
                                          LADA = "#E31A1C", T2D = "#33A02C"),
                            Metformin = c("0" = "white", "1" = "black"),
                            Sex = c(Male = "black", Female = "white"))


#Order genera, based on rowsums
TaxHeatmap <- Taxonomy[order(rowSums(Taxonomy), decreasing = T),]

#Impose a maximum number of plotted genera
TaxHeatmap <- TaxHeatmap[1:min(c(nrow(TaxHeatmap), Orgs)),]


##Then I standardized the orgs into zero mean and unit variance
TaxHeatmap <- data.frame(t(decostand(t(TaxHeatmap), method="standardize")))
#Can also use scale in pheatmap, but not exactly sure what scaling that is being performed


#Make dataframe with Metadata for heatmap annotation
colannodf <- data.frame(Metadata[, HeatmapExplainers], row.names = Metadata$MicrobiomeID)

#Calculate sample-distance matrix
#Note, that this is done on the full set, not just the shown. Makes sense eventhough not
#show in heatmap they can be in the clustering calculations, this also means samples can
#look more similar in the heatmap but not cluster as closely. Also calculates on the not
#log transformed data.
#filtering of the Counttable depending on rowSums.
Tax2 <- Taxonomy[rowSums(Taxonomy)>0,] #Removing all rows that only contains zeroes
#Tax2 <- Tax2[rowSums(Tax2)>(5*ncol(Tax2)),] #Removing all rows(Species)  that is below an
#average count of 5.
# replace 0 values with an estimate using simple multiplicative replacement
#Tax2 <- t(cmultRepl(t(Tax2), method="CZM", label=0))
#Maks TSS
Tax2<-sweep(Tax2, 2, colSums(Tax2), FUN="/")
#Calculate sample-distance matrix
#Note, that this is done on the full set, not just the shown. Makes sense eventhough not
#show in heatmap they can be in the clustering calculations, this also means samples can
#look more similar in the heatmap but not cluster as closely. Also calculates on the not
#log transformed data.
```

```
#distmatrix_Species <- vegdist(ilr(t(Tax2)), method="euclidean") #Previously
distmatrix_Species <- vegdist(decostand(t(Tax2), method="hellinger"), method="bray")

#Draw the heatmap
pdf(paste("MicroLADA_Heatmap", ".pdf", sep=""), width=15, height=5)
pheatmap(TaxHeatmap,
         #color = colorRampPalette(rev(brewer.pal(n = 7, name = "Blues")))(100),
         color = colorRampPalette(rev(brewer.pal(n = 7, name ="RdYlBu")))(100),
         margins=c(8,8),
         treeheight_row = 70,
         treeheight_col = 70,
         scale="none",
         clustering_distance_cols = distmatrix_Species,
         clustering_distance_rows = OrgCluster,
         annotation_col = colannodf,
         cutree_cols = 2,
         show_colnames = FALSE,
         #cellwidth=5,
         #cellheight=4,
         fontsize=10,
         annotation_colors = annotation_colorsNew[1:7],
         annotation_legend = TRUE)
dev.off()
```

```
## pdf
##   3
```

```
#Does not output plot to knitr
```

CCA

```
rm(list=setdiff(ls(), c("Metadata", "MetadataMed", "Feature", "TaxonomyDA", "Taxonomy", "Fig2List")))

#Create a list to hold the plot objects.
Fig2List <- list()

#Norm of Tax. Should be taken into account. The chi square distance that is the basis of
#cca makes raw counts appropriate.
#See https://sites.google.com/site/mb3gustame/reference/dissimilarity

#Filtering of the Counttable depending on rowSums. Prevents overplotting
Taxonomy2 <- Taxonomy[rowSums(Taxonomy)>(50*length(Taxonomy)),]
#Taxonomy2<-Taxonomy

##Total sum scaling (Use relative abundances)
Taxonomy2<-sweep(Taxonomy2, 2, colSums(Taxonomy2), FUN="/")


##Perform cca
ccaord <- cca(t(Taxonomy2) ~ Diagnosis + Condition(Metformin), Metadata)
ccaord
```

```
## Call: cca(formula = t(Taxonomy2) ~ Diagnosis + Condition(Metformin),
## data = Metadata)
##
##                 Inertia Proportion Rank
## Total           3.28993    1.00000
## Conditional     0.04234    0.01287    1
## Constrained     0.05203    0.01581    3
## Unconstrained 3.19557    0.97132  122
## Inertia is scaled Chi-square
##
## Eigenvalues for constrained axes:
##     CCA1     CCA2     CCA3
## 0.026008 0.013717 0.012301
##
## Eigenvalues for unconstrained axes:
##     CA1     CA2     CA3     CA4     CA5     CA6     CA7     CA8
## 0.23924 0.17501 0.16455 0.16302 0.13492 0.12763 0.11811 0.11654
## (Showing 8 of 122 unconstrained eigenvalues)
```

```
#summary(ccaord)
plot(ccaord)
```

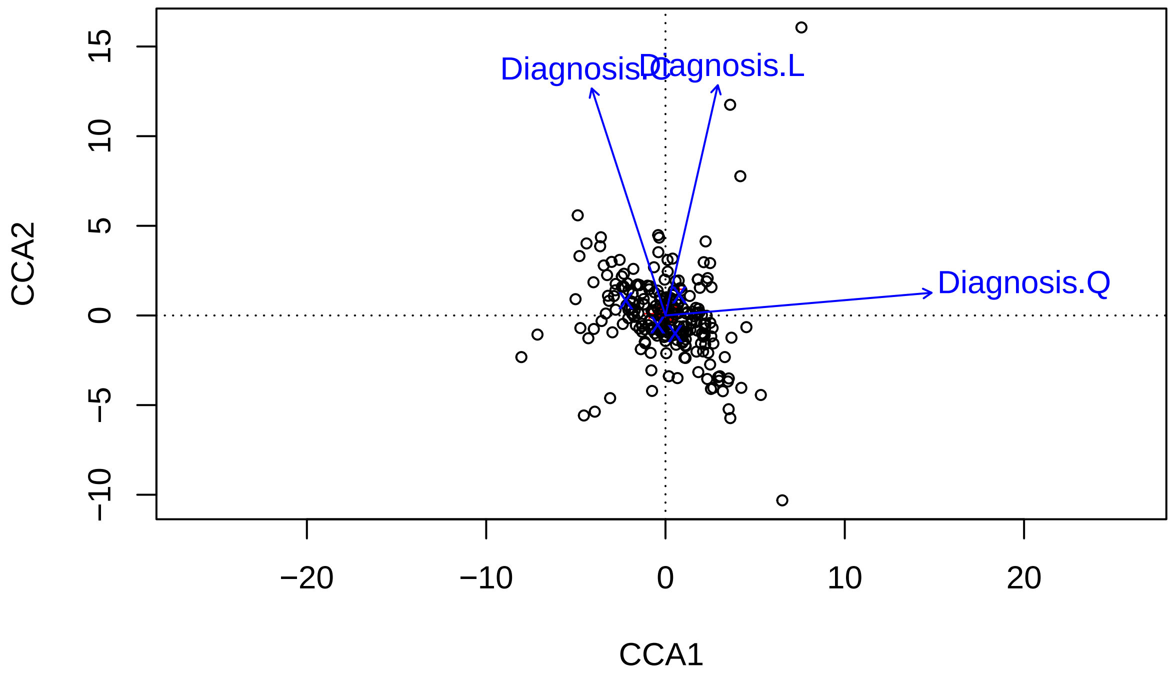


```
anova(ccaord) #Only Diagnosis
```

```
## Permutation test for cca under reduced model
```

```
## Permutation: free
## Number of permutations: 999
##
## Model: cca(formula = t(Taxonomy2) ~ Diagnosis + Condition(Metformin), data = Metadata)
##           Df ChiSquare      F Pr(>F)
## Model      3    0.0520 1.2211   0.05 *
## Residual 225    3.1956
## ---
## Signif. codes:  0 '***' 0.001 '**' 0.01 '*' 0.05 '.' 0.1 ' ' 1
```

```
##Other model
#ccaord2 <- cca(t(Taxonomy2)~Diagnosis+Metformin, Metadata)
#ccaord2
#summary(ccaord2)
#plot(ccaord2)
#anova(ccaord2, by="margin", permutations=199)

##ggvegan implementation of visualization
#devtools::install_github("gavinsimpson/ggvegan")
#library(ggvegan)
#autoplot(ccaord)

#Custom modification refer to cca object description
#https://www.rdocumentation.org/packages/vegan/versions/2.4-2/topics/cca.object


##My own implementation of cca visualization
#Extract scores from cca object
scores <- vegan::scores(ccaord, display=c("sp", "wa", "cn", "bp"), choices=c(1,2,3))

#Extract site information and add to metadata
sites <- data.frame(scores$sites)
sites$MicrobiomeID <- rownames(sites)
Metadata2 <- merge(Metadata, sites, by="MicrobiomeID")

#Extract species information and add to feature information
species <- data.frame(scores$species)
species$Genus <- rownames(species)
Feature2 <- merge(Feature, species, by="Genus")


#Create column org grouping for colouring
Feature2$Phyla <- ifelse(Feature2$Phylum=="Firmicutes", "Firmicutes",
          ifelse(Feature2$Phylum=="Proteobacteria", "Proteobacteria",
          ifelse(Feature2$Phylum=="Actinobacteria", "Actinobacteria",
          ifelse(Feature2$Phylum=="Bacteroidetes", "Bacteroidetes",
          ifelse(Feature2$Phylum=="Euryarchaeota", "Euryarchaeota",
                 "Other")))))
#Order Phyla for plotting
Feature2$Phyla <- factor(Feature2$Phyla, levels=c("Proteobacteria", "Bacteroidetes",
                                                  "Actinobacteria", "Firmicutes",
                                                  "Euryarchaeota", "Other"))
```

```r
#Extract information on inertia
inertia <- ccaord$CCA$tot.chi/ccaord$tot.chi
subheader <- paste("Inertia constrained by the explanatory variables",
                   round(inertia, digits=2))
##Extract information on eig
#eig1 <- (cca$CCA$eig[1]/(sum(cca$CCA$eig)+sum(cca$CA$eig)))*100
#eig_1 <- paste("CCA1", round(eig1, digits=2), "% of total variance")
#eig2 <- (cca$CCA$eig[2]/(sum(cca$CCA$eig)+sum(cca$CA$eig)))*100
#eig_2 <- paste("CCA2", round(eig2, digits=2), "% of total variance")

#Add a categorical indicator of group in Metadata
Metadata2$Metformin<-as.factor(ifelse(grepl("1", Metadata2$Metformin), "Yes",
                                      ifelse(grepl("0", Metadata2$Metformin), "No",
                                             "Unknown")))
#Order Treatment metformine for plotting
Metadata2$Metformin <- factor(Metadata2$Metformin, levels=c("Yes", "No", "Unknown"))

arrows<-data.frame(scores$centroids)
arrows$xstart<-0
arrows$ystart<-0
arrows$naming<-row.names(arrows)
arrows$naming<-str_remove(arrows$naming, "Diagnosis")

#Add text specific organisms to plot
textgenus<-Feature2 %>% dplyr::select(one_of("Genus", "CCA1", "CCA2")) %>%
  filter(Genus=="Faecalibacterium" |
           Genus=="Actinomyces" |
           Genus=="Butyricicoccus" |
           Genus=="Lachnoclostridium")


p1<-ggplot() +
  geom_point(data=Metadata2[which(Metadata2$Diagnosis=='Control'), ],
             aes(x=CCA1, y=CCA2, shape=Metformin, group=Diagnosis),
             color="#666666", size=1.5, alpha=0.5) +
  geom_point(data=Metadata2[which(Metadata2$Diagnosis=='T1D'), ],
             aes(x=CCA1, y=CCA2, shape=Metformin, group=Diagnosis),
             color="#1F78B4", size=1.5, alpha=0.5) +
  geom_point(data=Metadata2[which(Metadata2$Diagnosis=='T2D'), ],
             aes(x=CCA1, y=CCA2, shape=Metformin, group=Diagnosis),
             color="#33A02C", size=1.5, alpha=0.5) +
  geom_point(data=Metadata2[which(Metadata2$Diagnosis=='LADA'), ],
             aes(x=CCA1, y=CCA2, shape=Metformin, group=Diagnosis),
             color="#E31A1C", size=1.5, alpha=0.5) +
  scale_shape_manual(values=c(3, 16)) +
  geom_point(data=Feature2[which(Feature2$Phyla=="Proteobacteria"), ],
             aes(x=CCA1, y=CCA2, group=Genus), color="#A6CEE3", shape=18, size=0.7) +
  geom_point(data=Feature2[which(Feature2$Phyla=="Bacteroidetes"), ],
             aes(x=CCA1, y=CCA2, group=Genus), color="#FB9A99", shape=18, size=0.7) +
  geom_point(data=Feature2[which(Feature2$Phyla=="Actinobacteria"), ],
             aes(x=CCA1, y=CCA2, group=Genus), color="#FDBF6F", shape=18, size=0.7) +
  geom_point(data=Feature2[which(Feature2$Phyla=="Firmicutes"), ],
             aes(x=CCA1, y=CCA2, group=Genus),
```

```r
             color="#B2DF8A", shape=18, size=0.7, alpha=0.75) +
  geom_point(data=Feature2[which(Feature2$Phyla=="Euryarchaeota"), ],
             aes(x=CCA1, y=CCA2, group=Genus), color="#CAB2D6", shape=18, size=0.7) +
  geom_point(data=Feature2[which(Feature2$Phyla=="Other"), ],
             aes(x=CCA1, y=CCA2, group=Genus), color="#B3B3B3", shape=18, size=0.7) +
  geom_segment(data=arrows, aes(x=xstart, y=ystart, xend=CCA1, yend=CCA2),
               arrow=arrow(length = unit(0.01, "npc"))) +
  geom_text(data=arrows, aes(x = CCA1, y = CCA2, label = naming),
            hjust = 0, nudge_x = 0.05, size=6) +
  theme_bw() +
  theme(panel.grid.major = element_blank(),
        panel.grid.minor = element_blank(),
        axis.title=element_text(size=22),
        legend.position="none",
        legend.title=element_text(size=20),
        legend.text=element_text(size=20),
        axis.text.x = element_text(angle = 45, hjust = 1, size=12),
        axis.text.y = element_text(angle = 45, hjust = 1, size=12)) +
  scale_y_reverse()
pdf("MicroLADA_CCA.pdf", width=9, height=6)
p1
dev.off()
```

```
## pdf
##   2
```

```r
#Save to list
Fig2List[[ "CCAalls" ]] <- p1

#Created plotly version only to be able to look into specific genera plotted
ggplotly(p1)
```

```r
ggplotly(p1+xlim(-1.5, 2.5)+ylim(-1.5,2))
```

```r
#Overlay plots with cowplot
#p2<-p1+xlim(-1.5, 2.5)+ylim(-1.5,2)
p2<-ggplot() +
#  geom_point(data=Metadata2[which(Metadata2$Diagnosis=='Control'), ],
#             aes(x=CCA1, y=CCA2, shape=Metformin, group=Diagnosis),
#             color="#666666", size=1.5, alpha=0.5) +
#  geom_point(data=Metadata2[which(Metadata2$Diagnosis=='T1D'), ],
#             aes(x=CCA1, y=CCA2, shape=Metformin, group=Diagnosis),
#             color="#1F78B4", size=1.5, alpha=0.5) +
#  geom_point(data=Metadata2[which(Metadata2$Diagnosis=='T2D'), ],
#             aes(x=CCA1, y=CCA2, shape=Metformin, group=Diagnosis),
#             color="#33A02C", size=1.5, alpha=0.5) +
#  geom_point(data=Metadata2[which(Metadata2$Diagnosis=='LADA'), ],
#             aes(x=CCA1, y=CCA2, shape=Metformin, group=Diagnosis),
#             color="#E31A1C", size=1.5, alpha=0.5) +
  scale_shape_manual(values=c(3, 16)) +
  geom_point(data=Feature2[which(Feature2$Phyla=="Proteobacteria"), ],
             aes(x=CCA1, y=CCA2, group=Genus), color="#A6CEE3", shape=18, size=3) +
```

```
  geom_point(data=Feature2[which(Feature2$Phyla=="Bacteroidetes"), ],
             aes(x=CCA1, y=CCA2, group=Genus), color="#FB9A99", shape=18, size=3) +
  geom_point(data=Feature2[which(Feature2$Phyla=="Actinobacteria"), ],
             aes(x=CCA1, y=CCA2, group=Genus), color="#FDBF6F", shape=18, size=3) +
  geom_point(data=Feature2[which(Feature2$Phyla=="Firmicutes"), ],
             aes(x=CCA1, y=CCA2, group=Genus),
             color="#B2DF8A", shape=18, size=3, alpha=0.75) +
  geom_point(data=Feature2[which(Feature2$Phyla=="Euryarchaeota"), ],
             aes(x=CCA1, y=CCA2, group=Genus), color="#CAB2D6", shape=18, size=3) +
  geom_point(data=Feature2[which(Feature2$Phyla=="Other"), ],
             aes(x=CCA1, y=CCA2, group=Genus), color="#B3B3B3", shape=18, size=3) +
  geom_segment(data=arrows, aes(x=xstart, y=ystart, xend=CCA1, yend=CCA2),
               arrow=arrow(length = unit(0.01, "npc"))) +
  geom_text(data=arrows, aes(x = CCA1, y = CCA2, label = naming),
            hjust = 0, nudge_x = 0.05, size=10) +
  geom_text(data=textgenus, aes(x = CCA1, y = CCA2, label = Genus),
            hjust = 0, nudge_x = 0.05, size=8) +
  theme_bw() +
  theme(panel.grid.major = element_blank(),
        panel.grid.minor = element_blank(),
        axis.title=element_text(size=30),
        legend.position="none",
        legend.title=element_text(size=20),
        legend.text=element_text(size=20),
        axis.text.x = element_text(angle = 45, hjust = 1, size=22),
        axis.text.y = element_text(angle = 45, hjust = 1, size=22)) +
  scale_y_reverse() +
  xlim(-2.5, 2.5) +
  ylim(-1.5,2)


p3<-p1 + theme(axis.line=element_blank(),
               axis.text.x=element_blank(),
               axis.text.y=element_blank(),
               axis.ticks=element_blank(),
               axis.title.x=element_blank(),
               axis.title.y=element_blank(),
               legend.position="none",
               panel.background=element_blank(),
               #panel.border=element_blank(),
               panel.grid.major=element_blank(),
               panel.grid.minor=element_blank(),
               plot.background=element_blank())

ggdraw(p2) + draw_plot(p3, x = 0.30, y = -0.22, scale = 0.40)
```

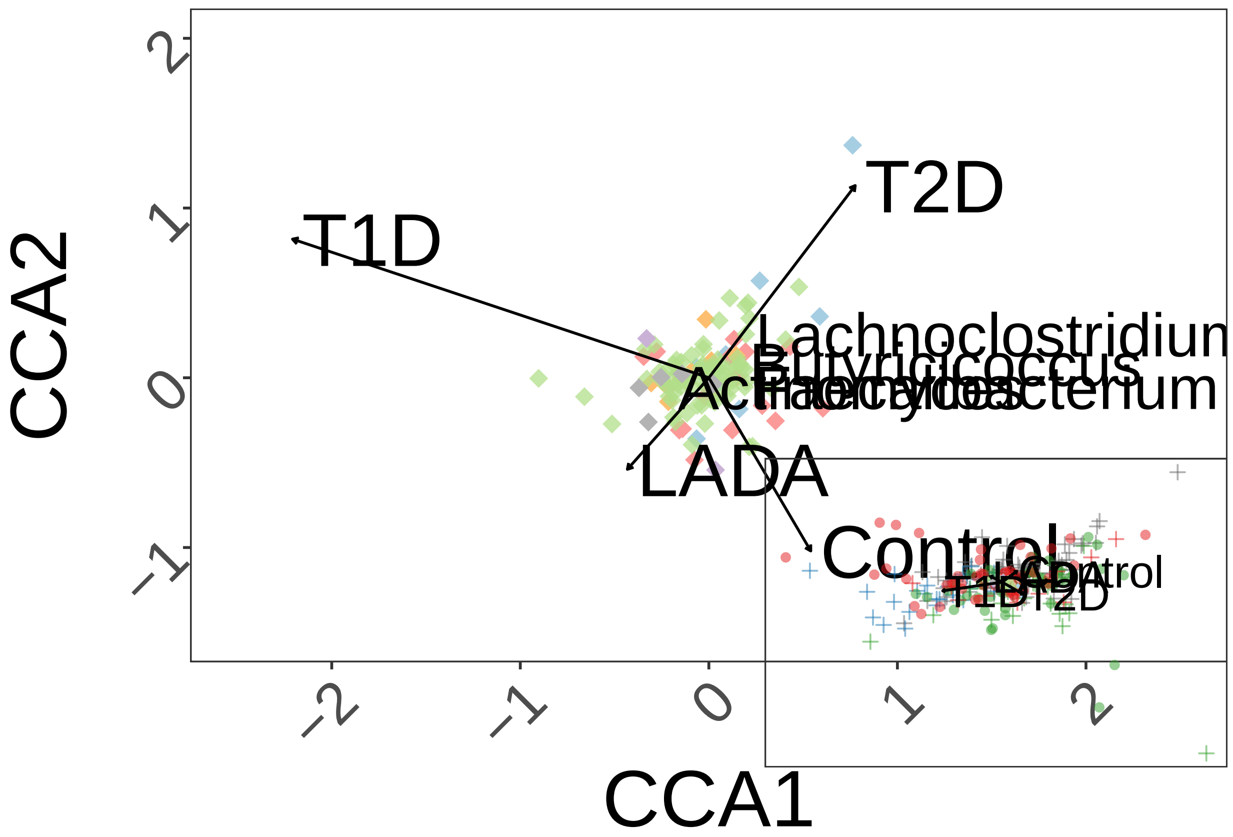


```
p4<-ggdraw(p2) + draw_plot(p3, x = 0.30, y = -0.18, scale = 0.40) #-22 below axis
#Save to list
Fig2List[[ "CCAoverlay" ]] <- p4
pdf("MicroLADA_CCAoverlay.pdf", width=9, height=6)
p4
dev.off()
```

```
## pdf
##   2
```

```
##Extract legends
#Extract legends phyla
Orgcol<-c("Proteobacteria" = "#A6CEE3",
          "Bacteroidetes" = "#FB9A99",
          "Actinobacteria" = "#FDBF6F",
          "Firmicutes" = "#B2DF8A",
          "Euryarchaeota" = "#CAB2D6",
          "Other" = "#B3B3B3")
#Plot with only legend colors for phyla
legend<-ggplot() +
  geom_point(data=Feature2, aes(x=CCA1, y=CCA2, group=Genus, color=Phyla),
             shape=18, size=6) +
  scale_color_manual(values=Orgcol) +
  theme_bw() +
  theme(legend.title=element_text(size=16),
```

```
        legend.text=element_text(size=14),
        legend.position="bottom")
#Store legends
legendplot<-get_legend(legend)
pdf("LegendCCAphyla_bottom.pdf", width=24, height=12)
grid.arrange(legendplot)
dev.off()
```

```
## pdf
##   2
```

```
#Save to list
Fig2List[[ "legendphylabottom" ]] <- legendplot

#Plot with only legend colors for phyla
legend<-ggplot() +
  geom_point(data=Feature2, aes(x=CCA1, y=CCA2, group=Genus, color=Phyla),
             shape=18, size=6) +
  scale_color_manual(values=Orgcol) +
  theme_bw() +
  theme(legend.title=element_text(size=16),
        legend.text=element_text(size=14),
        legend.position="right")
#Store legends
legendplot<-get_legend(legend)
pdf("LegendCCAphyla_right.pdf", width=24, height=12)
grid.arrange(legendplot)
dev.off()
```

```
## pdf
##   2
```

```
#Save to list
Fig2List[[ "legendphylaright" ]] <- legendplot

#Extract legends diagnosis
legend<-ggplot() +
  geom_point(data=Metadata2,
             aes(x=CCA1, y=CCA2, shape=Metformin, color=Diagnosis, group=Diagnosis),
             size=6, alpha=0.5) +
  scale_shape_manual(values=c(3, 16)) +
  scale_color_manual(values=c(Control = "#666666", T1D = "#1F78B4",
                              T2D = "#33A02C", LADA = "#E31A1C")) +
  theme_bw() +
  theme(legend.title=element_text(size=16),
        legend.text=element_text(size=14),
        legend.position="bottom")
#Store legends
legendplot<-get_legend(legend)
pdf("LegendCCAsamples_bottom.pdf", width=24, height=12)
grid.arrange(legendplot)
dev.off()
```

```
## pdf
##   2
```

```
#Save to list
Fig2List[[ "legendsamplesbottom" ]] <- legendplot

#Extract legends diagnosis
legend<-ggplot() +
  geom_point(data=Metadata2,
             aes(x=CCA1, y=CCA2, shape=Metformin, color=Diagnosis, group=Diagnosis),
             size=6, alpha=0.5) +
  scale_shape_manual(values=c(3, 16)) +
  scale_color_manual(values=c(Control = "#666666", T1D = "#1F78B4",
                              T2D = "#33A02C", LADA = "#E31A1C")) +
  theme_bw() +
  theme(legend.title=element_text(size=16),
        legend.text=element_text(size=14),
        legend.position="right")

#Store legends
legendplot<-get_legend(legend)
pdf("LegendCCAsamples_right.pdf", width=24, height=12)
grid.arrange(legendplot)
dev.off()
```

```
## pdf
##   2
```

```
#Save to list
Fig2List[[ "legendsamplesright" ]] <- legendplot
```

DESeq2 LRT

Analysis of deviance ANODEV
Size factors as column sums (Relative abundance), Total count standardized relative abundance as column sums

```
rm(list=setdiff(ls(), c("Metadata", "MetadataMed", "Feature", "TaxonomyDA", "Taxonomy", "Fig2List")))

#Can also be run with metformin as covariate, but decided not to due to treatment follow
#diagnosis.
#Better to compare with analysis run with individuals receiving metformin removed.

#Reassign names
Metadata2<-Metadata
Taxonomy2<-TaxonomyDA

#The factor can not be ordered for DESeq to run
Metadata2$Diagnosis<-factor(Metadata2$Diagnosis, ordered=FALSE)


#Create design formula
```

```r
#design <- formula(paste("~ ", "Diagnosis", "+",  "Metformin"))
design <- formula(paste("~ ", "Diagnosis", "+",  "BMIq"))
#design <- formula(paste("~ ", "Diagnosis"))
#Create DESeq2 object with matrix and metadata
dds <- DESeqDataSetFromMatrix(countData = Taxonomy2,
                              colData = Metadata2,
                              design = design)


##Assess and calculate size factors using the different methods
SF<-data.frame(Ratio=sizeFactors(estimateSizeFactors(dds, type="ratio")),
               Poscounts=sizeFactors(estimateSizeFactors(dds, type="poscounts")),
               Iterate=sizeFactors(estimateSizeFactors(dds, type="poscounts")),
               #"iterate" takes a lot of time changed to "poscounts" but kept due to the
               #following code
               Relative=colSums(Taxonomy2)/mean(colSums(Taxonomy2))
               )
SF<-add_rownames(SF, var="MicrobiomeID")
#Adding total abundances
# old path path<-paste("Q:/",
#             "Projects/",
#             "LADA/",
#             "LADA_Sandra_Evelina/",
#             "LADA_JKV/",
#             "LADA_R_AfterFlow_Analysis_FinalCounts/",
#             "LADA_FinalCounts/",
#             "2019-09-03_CountAnalysis.rds", sep="")

path <- paste("J:/",
              "CBMR/",
              "SUN-CBMR-Hansen-Group/",
              "Projects/",
              "LADA/",
              "LADA_Sandra_Evelina/",
              "LADA_JKV/",
              "LADA_R_AfterFlow_Analysis_FinalCounts/",
              "LADA_FinalCounts/",
              "2019-09-03_CountAnalysis.rds", sep="")


TotalCounts<-readRDS(path)
TotalCountsProcess <-
  data.frame(MicrobiomeID=TotalCounts$DF_Save_w$ID,
             CellNorm=TotalCounts$DF_Save_w$Cells_per_g_feces_FR_corrected_mean)
TotalCountsProcess$MicrobiomeID <- paste("L", TotalCountsProcess$MicrobiomeID,
                                         sep="")
SF2<-merge(SF, TotalCountsProcess, by="MicrobiomeID")

#One value is NaN in sample 602059 assigns value as average
SF2$CellNorm[is.nan(SF2$CellNorm)]<-mean(na.omit(SF2$CellNorm))

SF2$CellNormStand<-SF2$CellNorm/mean(SF2$CellNorm) #Watch out for NaN values can assign
                                                   #average, but wants the error
```

```r
SF3<-merge(Metadata2, SF2, by="MicrobiomeID")
#DESeq divides with sizeFactor (High cell count should have low sizeFactor providing
#larger counts in a sample)
#Still have to consider seq data is relative
SF3$NormRelCell<-SF3$Relative/SF3$CellNormStand
#Overview of normalization factors
aggregate(SF3[, 14:20], list(SF3$Diagnosis), mean) #Have to run heatmap chunk adding sex
```

```
##   Group.1    Ratio Poscounts   Iterate  Relative    CellNorm CellNormStand
## 1 Control 1.021277 1.0816841 1.0816841 1.0024242 20384275367     1.0420944
## 2     T1D 1.189194 1.3530621 1.3530621 1.1533999 18731782275     0.9576149
## 3    LADA 1.154124 1.0508265 1.0508265 0.9635197 18940404238     0.9682802
## 4     T2D 1.102417 0.9754019 0.9754019 0.9631018 19624620327     1.0032590
##   NormRelCell
## 1    1.078577
## 2    1.333993
## 3    1.069666
## 4    1.076697
```

```r
                                                               #to metadata
aggregate(SF3[, 14:20], list(SF3$Diagnosis), sd)
```

```
##   Group.1     Ratio Poscounts   Iterate  Relative   CellNorm CellNormStand
## 1 Control 0.5077220 0.3258182 0.3258182 0.2396082 6781846681     0.3467047
## 2     T1D 0.3607959 0.4887817 0.4887817 0.1896984 5712095609     0.2920164
## 3    LADA 0.5115578 0.4411272 0.4411272 0.2136868 5544046390     0.2834253
## 4     T2D 0.4568660 0.3704665 0.3704665 0.2075670 6146706989     0.3142348
##   NormRelCell
## 1   0.5133752
## 2   0.5220606
## 3   0.3592809
## 4   0.4836133
```

```r
pairs(SF3[, c(14:17,19,20)], col=SF3$Diagnosis)
```

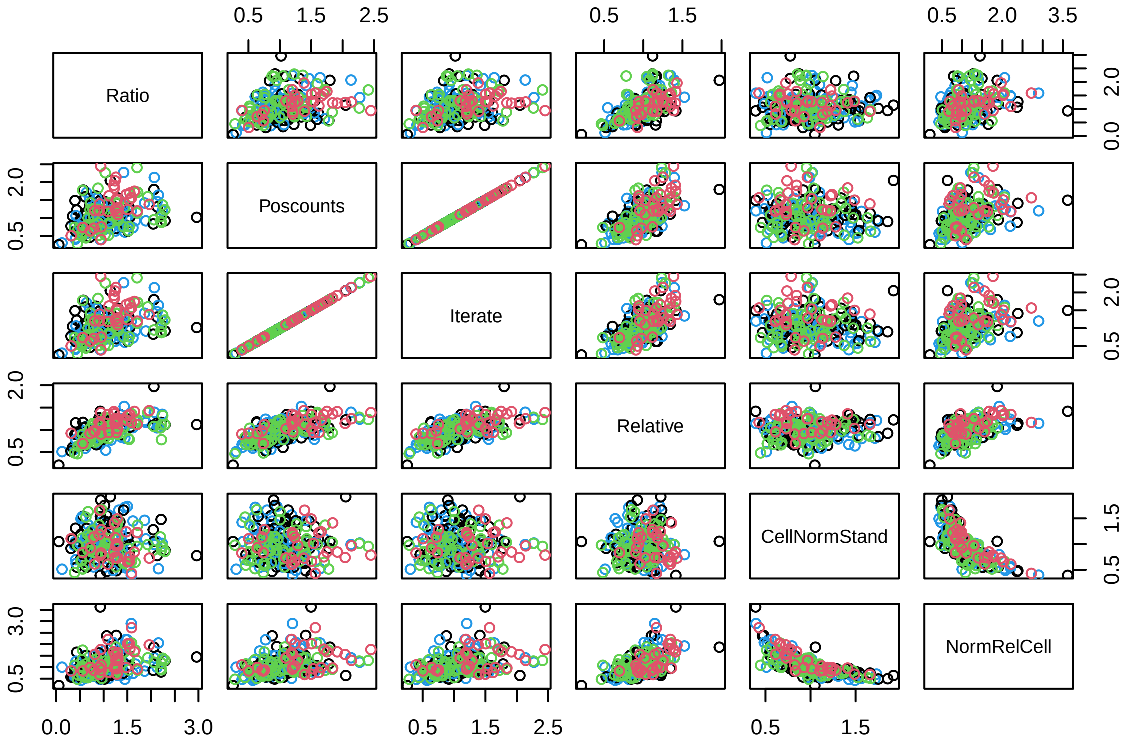


```
pairs(SF3[, c(15,17,19,20)], col=SF3$Diagnosis)
```

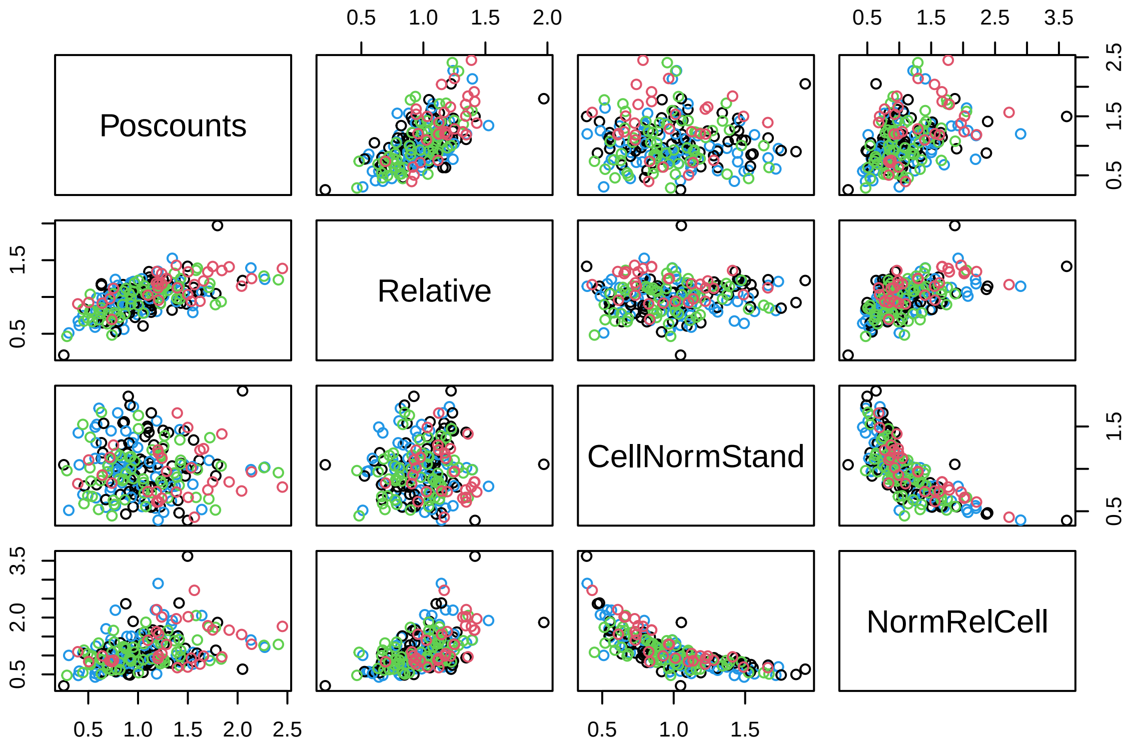


```
#I do want CellNormStand and NormRelCell to be negatively correlated due to DESeq divide
#with sizeFactor. It is also good that CellNormStand seems uncorrelated to the other
#normalization factors.


##Select size factors calculated above for normalization
dds@colData@listData$sizeFactor <- SF3[,20]
#hist(dds@colData@listData$sizeFactor)
#colnames(SF3[13])
normname<-colnames(SF3[20])

##See vignette for note on factor levels
#Filtering of the Counttable. Bare minimum is performed later. Also to reduce memory of
#the dds
dds2 <- dds[ rowSums(counts(dds))>50*length(Taxonomy2),] #Previously 0 instead of 1

#Estimate dispersions and fit the GLM
#dds2 <- DESeq(dds2, test="LRT", reduced = ~Metformin)
dds2 <- DESeq(dds2, test="LRT", reduced = ~1)

res<-results(dds2)
summary(res)
```

```
##
## out of 82 with nonzero total read count
```

```
## adjusted p-value < 0.1
## LFC > 0 (up)       : 8, 9.8%
## LFC < 0 (down)     : 7, 8.5%
## outliers [1]       : 0, 0%
## low counts [2]     : 0, 0%
## (mean count < 49)
## [1] see 'cooksCutoff' argument of ?results
## [2] see 'independentFiltering' argument of ?results
```

```
resultsNames(dds2)
```

```
## [1] "Intercept"                 "Diagnosis_T1D_vs_Control"
## [3] "Diagnosis_LADA_vs_Control" "Diagnosis_T2D_vs_Control"
## [5] "BMIq"
```

```
#MA plots from inbuild DESeq function
plotMA(dds2, alpha=0.1)
```

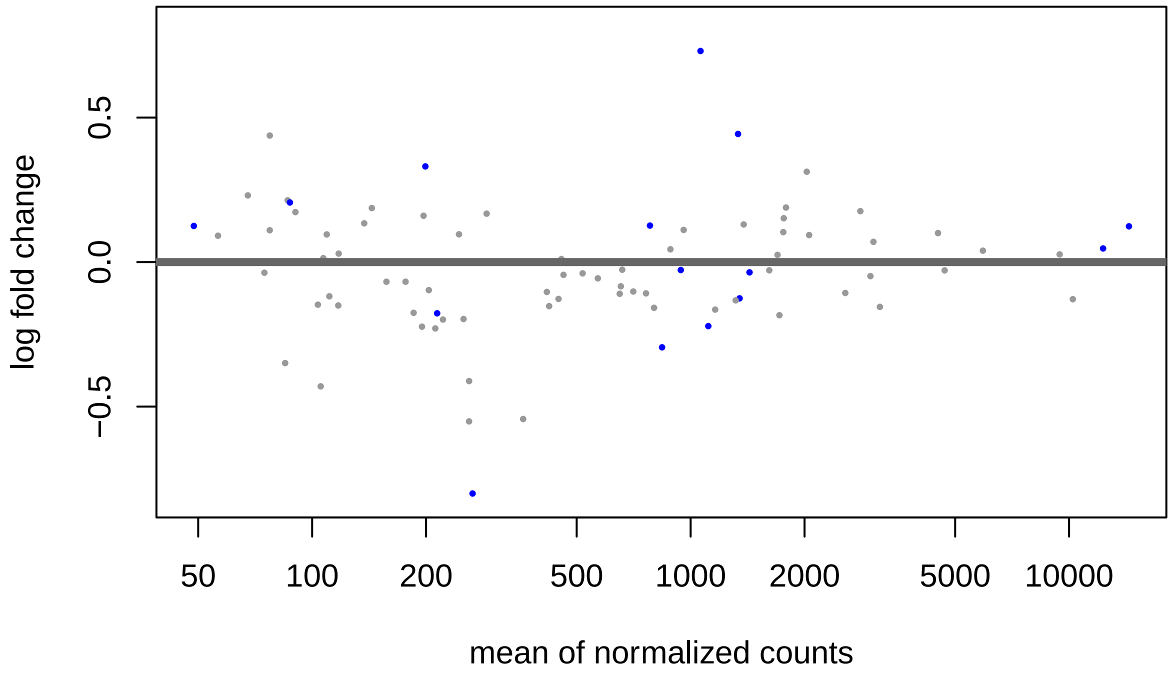


```
plotMA(res, ylim=c(-2,2))
```

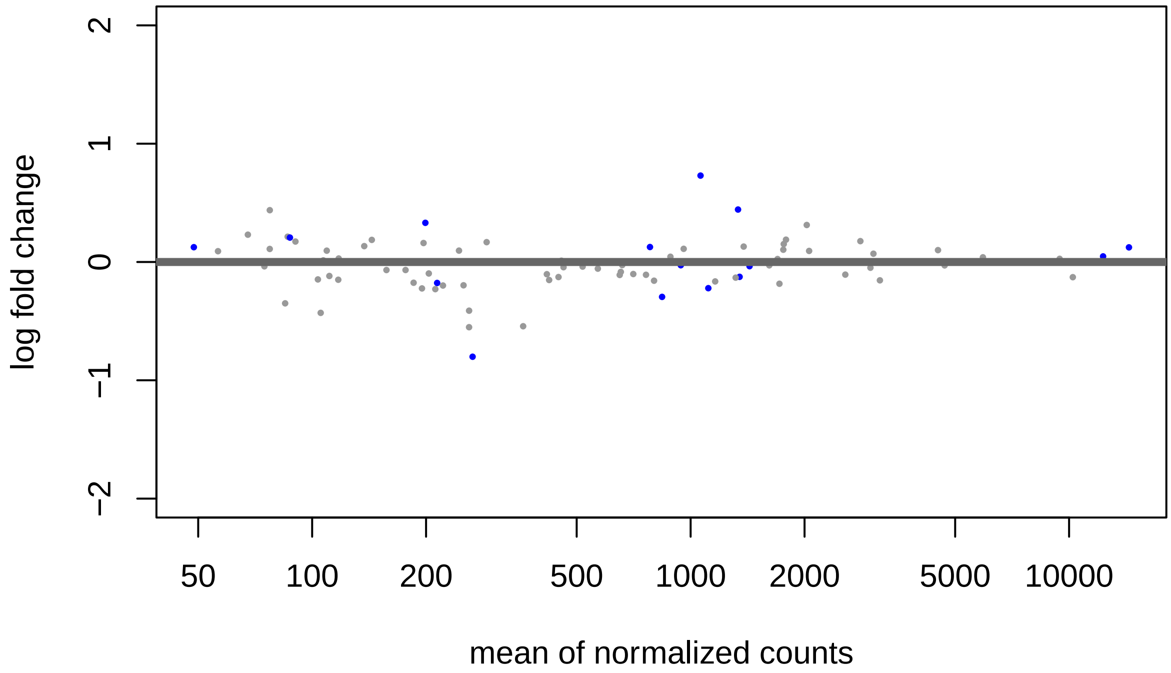


```
#Unshrunken/shrunken
#res<-results(dds2, addMLE=TRUE)
#resLFC <- DESeq2::lfcShrink(dds2, coef=2)
#resLFC
#DESeq2::plotMA(resLFC, ylim=c(-2,2))

#Make histogram
#pdf(paste("HistogramDESeq", ".pdf", sep=""), height=6, width=12)
hist(res$pvalue[res$baseMean > 1], breaks = 0:20/20,
     col = "grey50", border = "white")
```

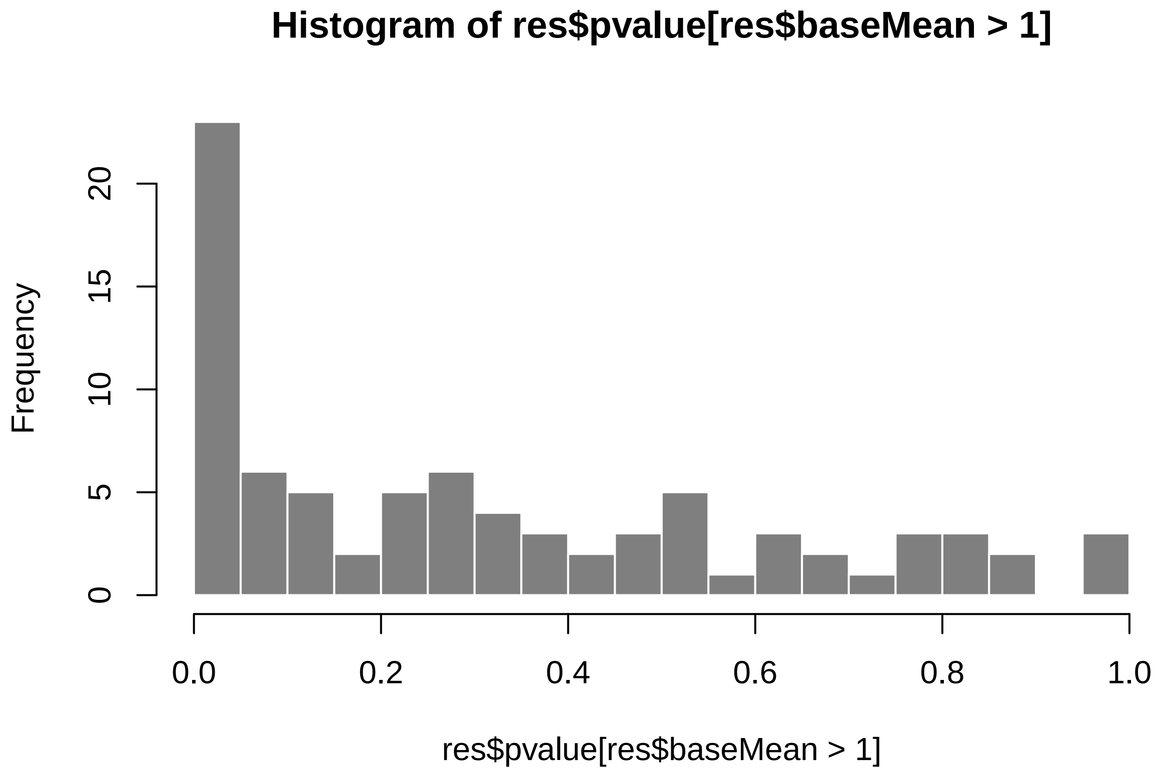


```
#dev.off()

#Show dispersion plot with shrinkage
#pdf(paste("DispersionplotDESeq", ".pdf", sep=""), height=9, width=12)
plotDispEsts(dds2)
```

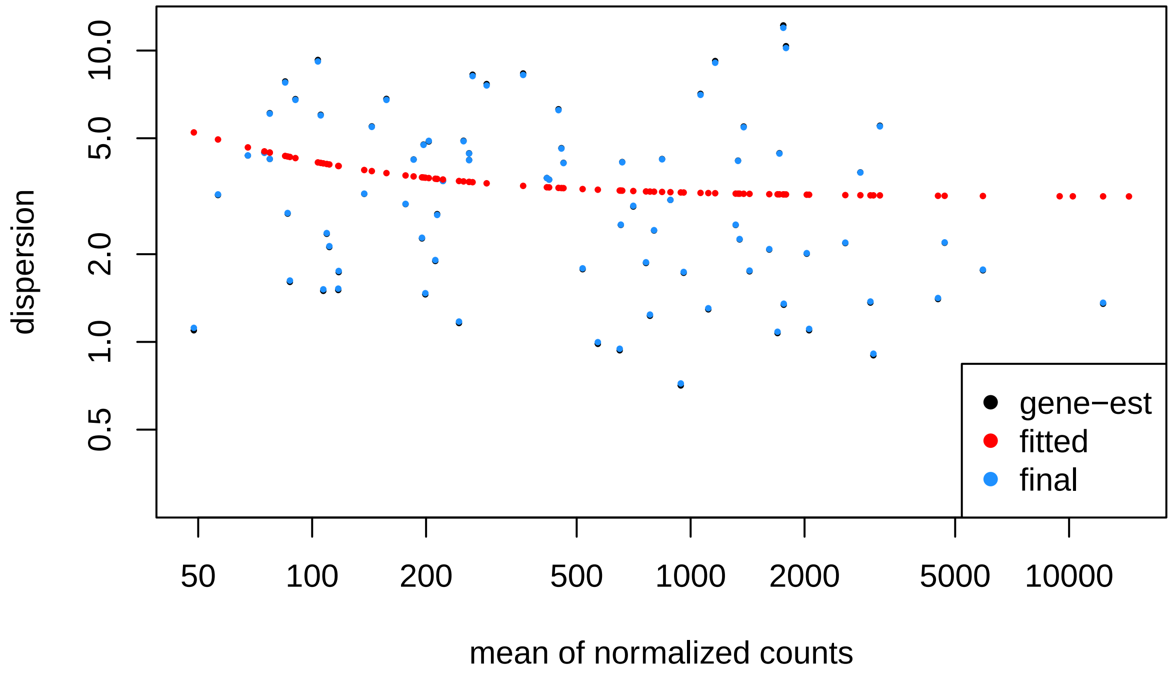


```
#dev.off()

##Plot PCA
#dds2_rlog<-rlogTransformation(dds2)
##pdf(paste("PCADESeq", ".pdf", sep=""), height=9, width=9)
#plotPCA(dds2_rlog, intgroup=c("Diagnosis"))+coord_fixed()
##dev.off()

#Data structuring
resSig=subset(res, pvalue<0.05) #Could also have selected other value or padj
df <- data.frame(res)
dfSig <- data.frame(resSig)

#Save table.
write.table(dfSig, file="DESeq_SigLRT.txt", sep="\t", dec=",", row.names = T)
print("Significant LRT")
```

```
## [1] "Significant LRT"
```

```
kable(dfSig)
```

	baseMean	log2FoldChange	lfcSE	stat	pvalue	padj
Actinomyces	48.68949	0.1250123	0.0981755	18.603766	0.0009401	0.0138739

	baseMean	log2FoldChange	lfcSE	stat	pvalue	padj
Parabacteroides	1113.74127	-0.2214684	0.1051180	15.688121	0.0034675	0.0315930
Lactobacillus	1062.10266	0.7303690	0.2442038	20.885143	0.0003337	0.0068411
Christensenellaceae_R.7_group	1347.24525	-0.1254238	0.1381507	14.046646	0.0071477	0.0555450
Clostridium_sensu_stricto_1	840.65653	-0.2949778	0.1893029	17.128070	0.0018253	0.0187091
Blautia	14393.56810	0.1237883	0.0611240	13.582127	0.0087554	0.0598285
Fusicatenibacter	2986.10151	-0.0485782	0.1079209	9.529692	0.0491407	0.1751973
Lachnoclostridium	780.88361	0.1263943	0.1025334	28.296170	0.0000109	0.0004454
Lachnospira	213.93756	-0.1772069	0.1520756	12.762801	0.0124949	0.0711619
Roseburia	1431.19573	-0.0354981	0.1219584	12.962207	0.0114619	0.0711619
Butyricicoccus	941.98315	-0.0274939	0.0781318	18.433469	0.0010152	0.0138739
Faecalibacterium	12297.06909	0.0471612	0.1073988	12.667973	0.0130174	0.0711619
Negativibacillus	77.28674	0.4378604	0.1898986	10.756939	0.0294359	0.1340967
Oscillibacter	199.11901	0.3310706	0.1117231	13.951594	0.0074512	0.0555450
Ruminiclostridium_5	649.61339	-0.1096863	0.0896150	9.767440	0.0445327	0.1659857
Ruminiclostridium_6	259.81035	-0.5511814	0.1937340	11.562436	0.0209198	0.1072137
Ruminococcaceae_UCG.010	259.84142	-0.4116403	0.1887162	10.108381	0.0386411	0.1584284
UBA1819	87.35346	0.2062960	0.1176827	17.505838	0.0015410	0.0180512
Family.Erysipelotrichaceae.	56.39729	0.0912006	0.1652792	10.360298	0.0347767	0.1500891
Veillonella	265.43054	-0.8007728	0.2632080	25.894313	0.0000332	0.0009084
Order.Rhodospirillales.	84.86068	-0.3494951	0.2566468	11.156592	0.0248594	0.1199099
Bilophila	137.28563	0.1341460	0.1654708	9.857536	0.0428971	0.1659857
Escherichia/Shigella	1334.40881	0.4433030	0.1881418	51.089174	0.0000000	0.0000000

DESeq2 LRT remove metformin

Analysis of deviance ANODEV
Size factors as column sums (Relative abundance), Total count standardized relative abundance as column sums

```
rm(list=setdiff(ls(), c("Metadata", "MetadataMed", "Feature", "TaxonomyDA", "Taxonomy",
                        "Fig2List")))
#Reassign names
#Metadata2<-Metadata
Taxonomy2<-TaxonomyDA

##Removing treated with metformin
#Metadata$Metformin <- ifelse(is.na(Metadata$Metformin), "Unknown", Metadata$Metformin)
Metadata2<-filter(Metadata, !Metformin == 1)
##Applying subsetting to OTU tables
Taxonomy2<-dplyr::select(Taxonomy2, one_of(as.character(Metadata2$MicrobiomeID)))
##Remove orgs that are not present after subsetting.
Taxonomy2 <- Taxonomy2[rowSums(Taxonomy2)>0,]


#Can also be run with metformin as covariate, but decided not to due to treatment follow
#diagnosis.
#Better to compare with analysis run with individuals receiving metformin removed.
#As performed here


#The factor can not be ordered for DESeq to run
```

```r
Metadata2$Diagnosis<-factor(Metadata2$Diagnosis, ordered=FALSE)


#Create design formula
#design <- formula(paste("~ ", "Diagnosis", "+",  "Metformin"))
design <- formula(paste("~ ", "Diagnosis", "+",  "BMIq"))
#design <- formula(paste("~ ", "Diagnosis"))
#Create DESeq2 object with matrix and metadata
dds <- DESeqDataSetFromMatrix(countData = Taxonomy2,
                              colData = Metadata2,
                              design = design)


##Assess and calculate size factors using the different methods
SF<-data.frame(Ratio=sizeFactors(estimateSizeFactors(dds, type="ratio")),
               Poscounts=sizeFactors(estimateSizeFactors(dds, type="poscounts")),
               Iterate=sizeFactors(estimateSizeFactors(dds, type="poscounts")),
               #"iterate" takes a lot of time changed to "poscounts" but kept due to the
               #following code
               Relative=colSums(Taxonomy2)/mean(colSums(Taxonomy2))
               )
SF<-add_rownames(SF, var="MicrobiomeID")
#Adding total abundances
# old path path<-paste("Q:/",
#             "Projects/",
#             "LADA/",
#             "LADA_Sandra_Evelina/",
#             "LADA_JKV/",
#             "LADA_R_AfterFlow_Analysis_FinalCounts/",
#             "LADA_FinalCounts/",
#             "2019-09-03_CountAnalysis.rds", sep="")

path <- paste("J:/",
              "CBMR/",
              "SUN-CBMR-Hansen-Group/",
              "Projects/",
              "LADA/",
              "LADA_Sandra_Evelina/",
              "LADA_JKV/",
              "LADA_R_AfterFlow_Analysis_FinalCounts/",
              "LADA_FinalCounts/",
              "2019-09-03_CountAnalysis.rds", sep="")


TotalCounts<-readRDS(path)
TotalCountsProcess <-
  data.frame(MicrobiomeID=TotalCounts$DF_Save_w$ID,
             CellNorm=TotalCounts$DF_Save_w$Cells_per_g_feces_FR_corrected_mean)
TotalCountsProcess$MicrobiomeID <- paste("L", TotalCountsProcess$MicrobiomeID,
                                         sep="")
SF2<-merge(SF, TotalCountsProcess, by="MicrobiomeID")

#One value is NaN in sample 602059 assigns value as average
```

```r
SF2$CellNorm[is.nan(SF2$CellNorm)]<-mean(na.omit(SF2$CellNorm))

SF2$CellNormStand<-SF2$CellNorm/mean(SF2$CellNorm) #Watch out for NaN values can assign
                                                   #average, but wants the error
SF3<-merge(Metadata2, SF2, by="MicrobiomeID")
#DESeq divides with sizeFactor (High cell count should have low sizeFactor providing
#larger counts in a sample)
#Still have to consider seq data is relative
SF3$NormRelCell<-SF3$Relative/SF3$CellNormStand
#Overview of normalization factors
aggregate(SF3[, 14:20], list(SF3$Diagnosis), mean) #Have to run heatmap chunk adding sex
```

```
##   Group.1    Ratio Poscounts  Iterate  Relative    CellNorm CellNormStand
## 1 Control 1.093106  1.002662 1.002662 0.9644647 20384275367     1.0722125
## 2     T1D 1.232576  1.261833 1.261833 1.1097233 18731782275     0.9852914
## 3    LADA 1.203409  1.213468 1.213468 1.0061418 15590817082     0.8200767
## 4     T2D 1.079416  0.942112 0.942112 0.9618292 16982538343     0.8932812
##   NormRelCell
## 1    1.008584
## 2    1.247425
## 3    1.292202
## 4    1.183913
```

```r
                                                   #to metadata
aggregate(SF3[, 14:20], list(SF3$Diagnosis), sd)
```

```
##   Group.1     Ratio Poscounts   Iterate  Relative   CellNorm CellNormStand
## 1 Control 0.4622606 0.2998114 0.2998114 0.2305348 6781846681     0.3567250
## 2     T1D 0.4965321 0.4582512 0.4582512 0.1825149 5712095609     0.3004561
## 3    LADA 0.5245937 0.4814438 0.4814438 0.1937037 4604942130     0.2422199
## 4     T2D 0.5152121 0.3336165 0.3336165 0.2220192 4888235680     0.2571211
##   NormRelCell
## 1   0.4800604
## 2   0.4881822
## 3   0.3379476
## 4   0.4797532
```

```r
pairs(SF3[, c(14:17,19,20)], col=SF3$Diagnosis)
```

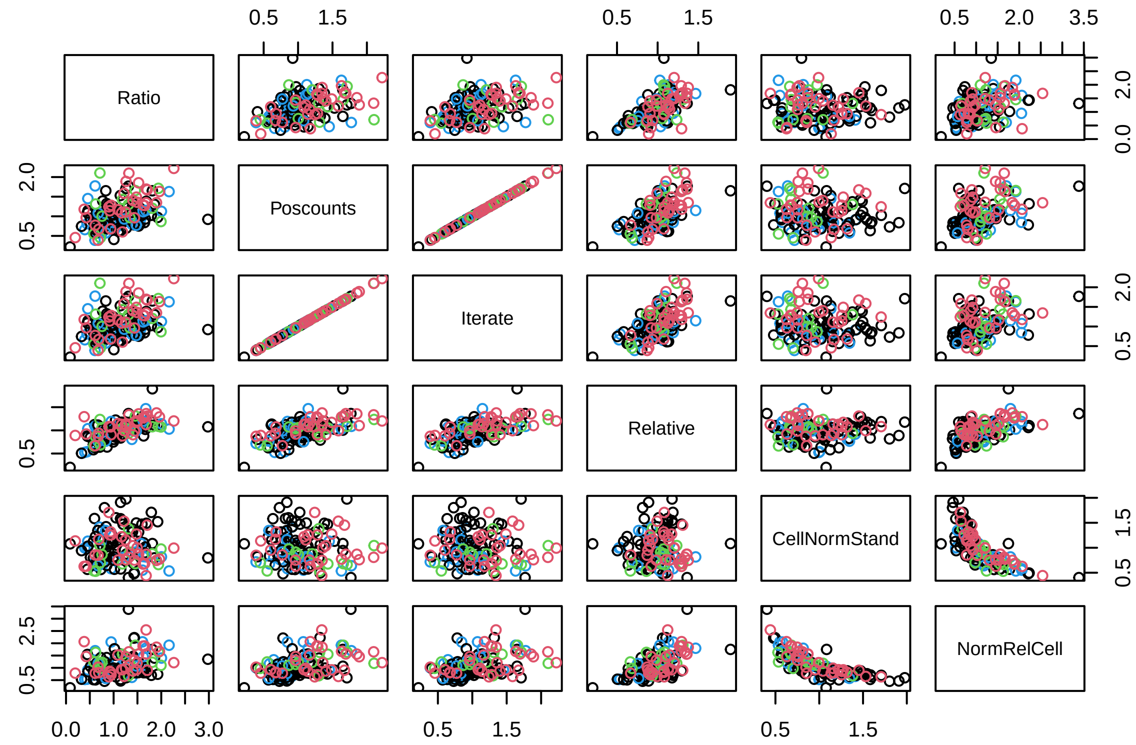


```r
pairs(SF3[, c(15,17,19,20)], col=SF3$Diagnosis)
```

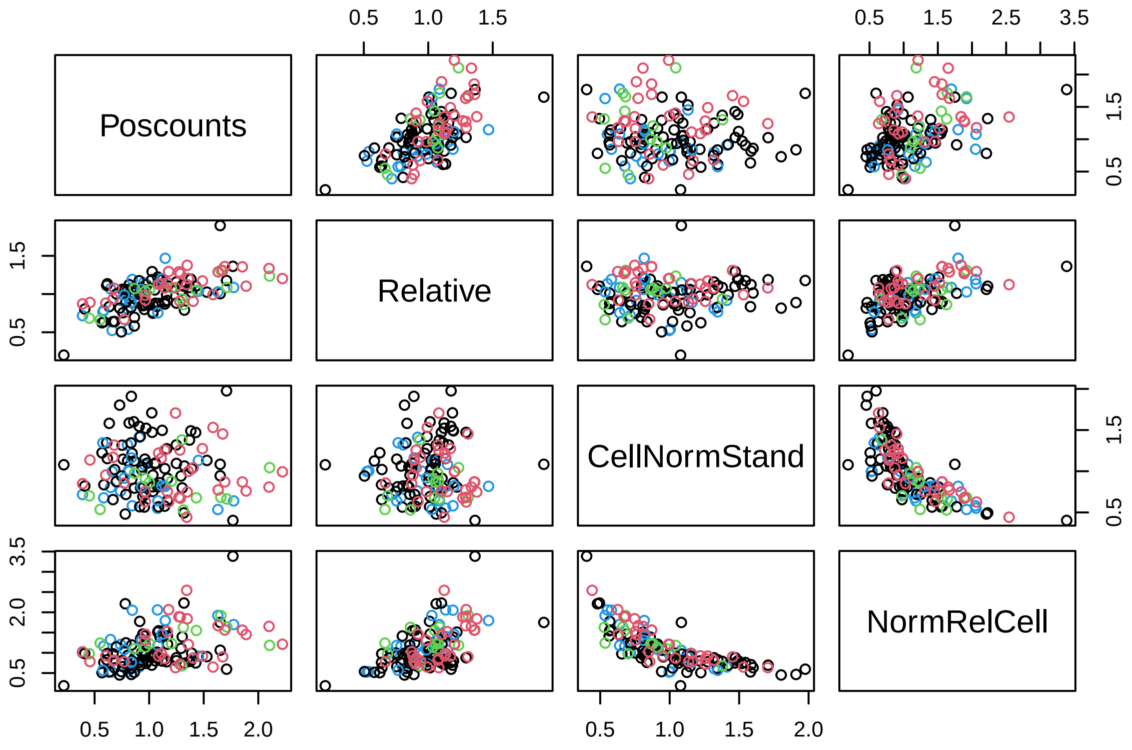


```
#I do want CellNormStand and NormRelCell to be negatively correlated due to DESeq divide
#with sizeFactor. It is also good that CellNormStand seems uncorrelated to the other
#normalization factors.


##Select size factors calculated above for normalization
dds@colData@listData$sizeFactor <- SF3[,20]
#hist(dds@colData@listData$sizeFactor)
#colnames(SF3[13])
normname<-colnames(SF3[20])

##See vignette for note on factor levels
#Filtering of the Counttable. Bare minimum is performed later. Also to reduce memory of
#the dds
dds2 <- dds[ rowSums(counts(dds))>50*length(Taxonomy2),] #Previously 0 instead of 1

#Estimate dispersions and fit the GLM
#dds2 <- DESeq(dds2, test="LRT", reduced = ~Metformin)
dds2 <- DESeq(dds2, test="LRT", reduced = ~1)

res<-results(dds2)
summary(res)
```

```
##
## out of 81 with nonzero total read count
```

```
## adjusted p-value < 0.1
## LFC > 0 (up)       : 3, 3.7%
## LFC < 0 (down)     : 3, 3.7%
## outliers [1]       : 0, 0%
## low counts [2]     : 0, 0%
## (mean count < 48)
## [1] see 'cooksCutoff' argument of ?results
## [2] see 'independentFiltering' argument of ?results
```

```
resultsNames(dds2)
```

```
## [1] "Intercept"                 "Diagnosis_T1D_vs_Control"
## [3] "Diagnosis_LADA_vs_Control" "Diagnosis_T2D_vs_Control"
## [5] "BMIq"
```

```
#MA plots from inbuild DESeq function
plotMA(dds2, alpha=0.1)
```

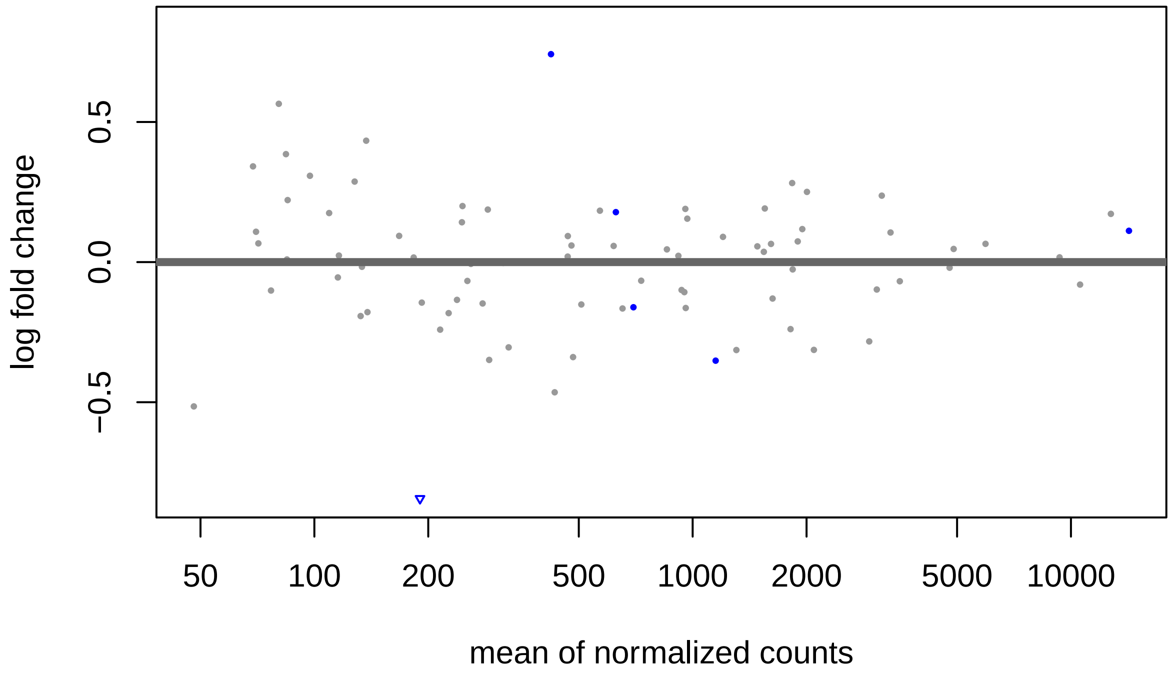


```
plotMA(res, ylim=c(-2,2))
```

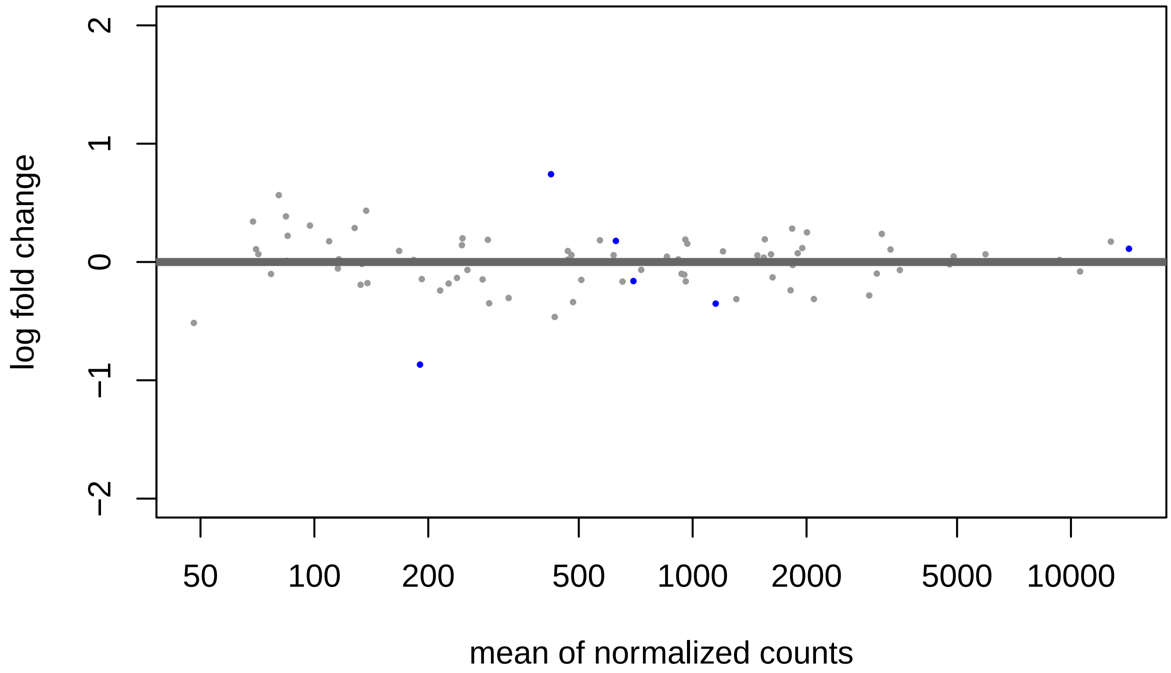


```r
#Unshrunken/shrunken
#res<-results(dds2, addMLE=TRUE)
#resLFC <- DESeq2::lfcShrink(dds2, coef=2)
#resLFC
#DESeq2::plotMA(resLFC, ylim=c(-2,2))

#Make histogram
#pdf(paste("HistogramDESeq", ".pdf", sep=""), height=6, width=12)
hist(res$pvalue[res$baseMean > 1], breaks = 0:20/20,
     col = "grey50", border = "white")
```

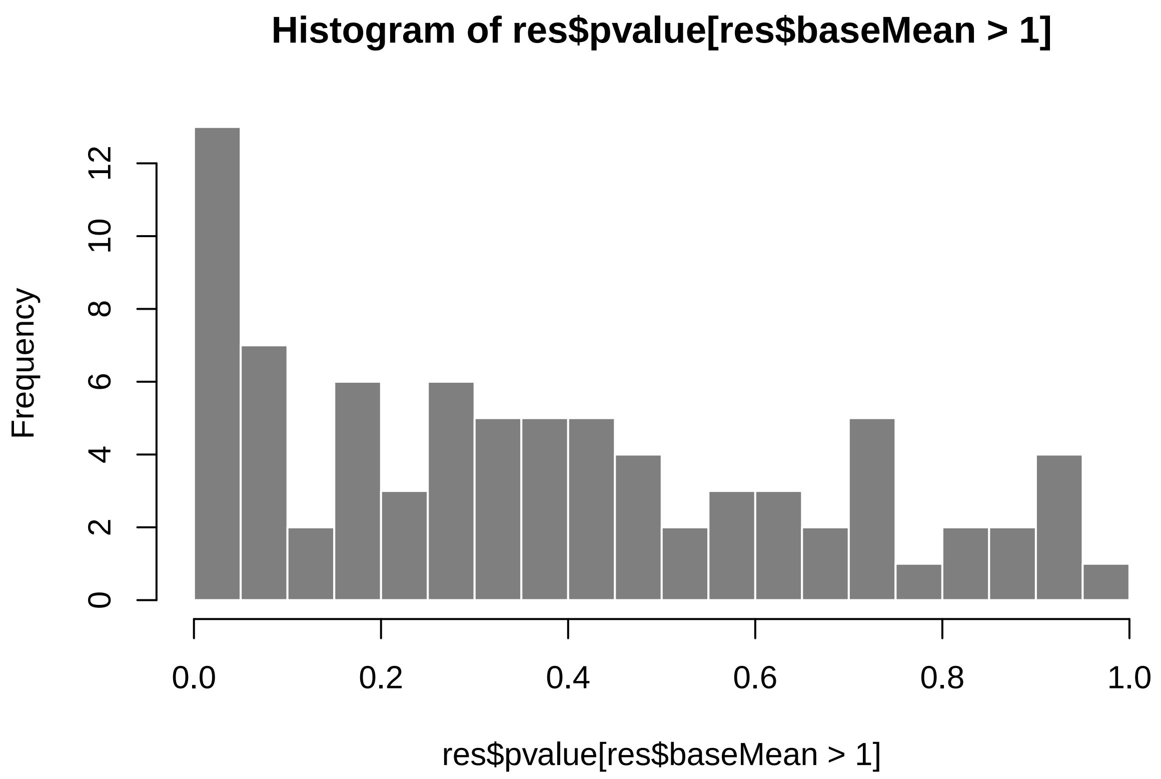


```
#dev.off()

#Show dispersion plot with shrinkage
#pdf(paste("DispersionplotDESeq", ".pdf", sep=""), height=9, width=12)
plotDispEsts(dds2)
```

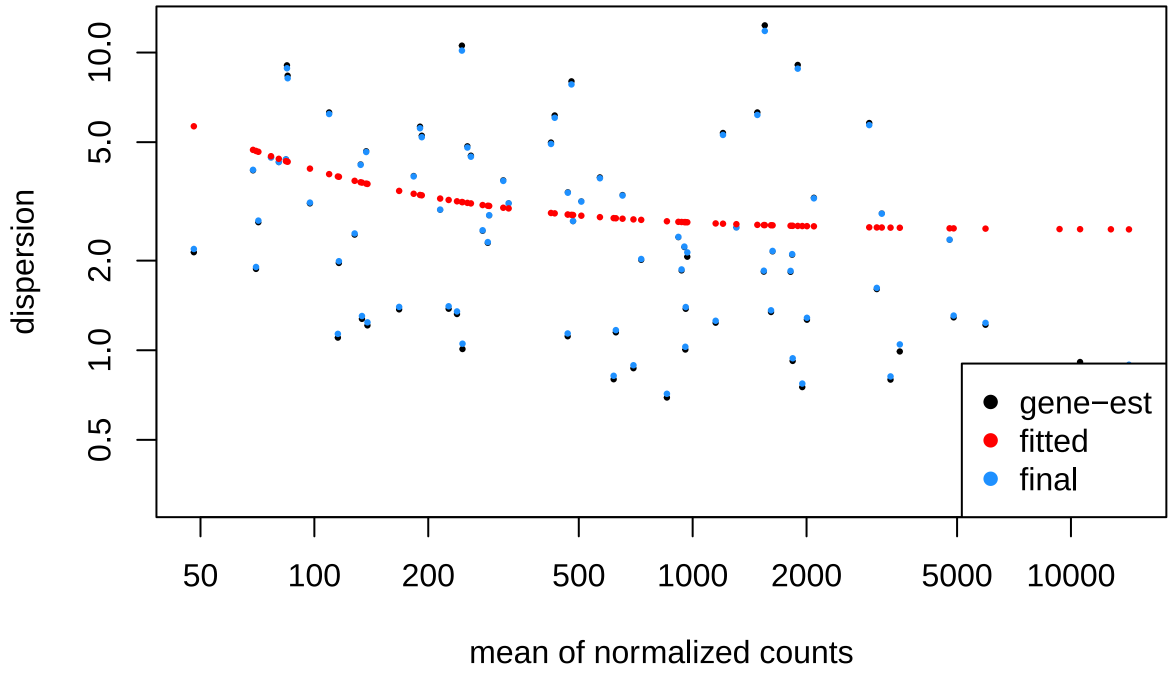


```
#dev.off()

##Plot PCA
#dds2_rlog<-rlogTransformation(dds2)
##pdf(paste("PCADESeq", ".pdf", sep=""), height=9, width=9)
#plotPCA(dds2_rlog, intgroup=c("Diagnosis"))+coord_fixed()
##dev.off()

#Data structuring
resSig=subset(res, pvalue<0.05) #Could also have selected other value or padj
df <- data.frame(res)
dfSig <- data.frame(resSig)

#Save table.
write.table(dfSig, file="DESeq_SigLRTRemMet.txt", sep="\t", dec=",", row.names = T)
print("Significant LRT")
```

```
## [1] "Significant LRT"
```

```
kable(dfSig)
```

	baseMean	log2FoldChange	lfcSE	stat	pvalue	padj
Family.Eggerthellaceae.	507.82344	-0.1512598	0.2120578	11.66578	0.0200176	0.1474022

	baseMean	log2FoldChange	lfcSE	stat	pvalue	padj
Alistipes	2005.08084	0.2504997	0.1352641	10.15503	0.0378957	0.2361191
Parabacteroides	1150.20068	-0.3518096	0.1337562	21.15970	0.0002944	0.0198638
Streptococcus	1626.63392	-0.1300053	0.1751785	10.16914	0.0376729	0.2361191
GCA.900066575	48.01925	-0.5149756	0.1775909	11.87897	0.0182743	0.1474022
Lachnoclostridium	626.64815	0.1782757	0.1290445	17.73651	0.0013893	0.0225072
Roseburia	1611.25968	0.0648992	0.1392521	11.78944	0.0189877	0.1474022
Butyricicoccus	854.99157	0.0453647	0.1008986	13.38095	0.0095569	0.1105869
Faecalibacterium	14234.24963	0.1116576	0.1127932	14.14663	0.0068415	0.0923601
Ruminiclostridium_5	697.42166	-0.1612049	0.1126770	20.03971	0.0004905	0.0198638
Ruminiclostridium_6	289.80533	-0.3490619	0.2012804	12.13863	0.0163496	0.1474022
Veillonella	190.21664	-0.8673058	0.2820774	17.86563	0.0013110	0.0225072
Escherichia/Shigella	422.24727	0.7420216	0.2649870	18.22389	0.0011157	0.0225072

DESeq Wald

All pairwise Differential abundance analysis comparison (DESeq and visualized venn)

```
rm(list=setdiff(ls(), c("Metadata", "MetadataMed", "Feature", "TaxonomyDA", "Taxonomy", "Fig2List")))

#Reassign names
Metadata2<-Metadata
#Order Diagnosis
Metadata2$Diagnosis<-ordered(Metadata2$Diagnosis,
                            levels=c("Control", "T1D", "T2D", "LADA"))
#Included filtering
Taxonomy2<-TaxonomyDA
#Taxonomy2<-Taxonomy[length(Taxonomy)-rowSums(Taxonomy == 0) >= 30,]


#The factor can not be ordered for DESeq to run
Metadata2$Diagnosis<-factor(Metadata2$Diagnosis, ordered=FALSE)

#Create design formula
#design <- formula(paste("~ ", "Diagnosis"))
design <- formula(paste("~ ", "Diagnosis", "+",  "BMIq"))
#design <- formula(paste("~ ", "Diagnosis", "+ Metformin"))
#Create DESeq2 object with matrix and metadata
dds <- DESeqDataSetFromMatrix(countData = Taxonomy2,
                              colData = Metadata2,
                              design = design)


##Assess and calculate size factors using the different methods
SF<-data.frame(Ratio=sizeFactors(estimateSizeFactors(dds, type="ratio")),
               Poscounts=sizeFactors(estimateSizeFactors(dds, type="poscounts")),
               #"iterate" takes a lot of time changed to "poscounts" but kept due to the
               #following code
               Iterate=sizeFactors(estimateSizeFactors(dds, type="poscounts")),
               Relative=colSums(Taxonomy2)/mean(colSums(Taxonomy2))
               )
SF<-add_rownames(SF, var="MicrobiomeID")
```

```
#Adding total abundances
# old path path<-paste("Q:/",
#             "Projects/",
#             "LADA/",
#             "LADA_Sandra_Evelina/",
#             "LADA_JKV/",
#             "LADA_R_AfterFlow_Analysis_FinalCounts/",
#             "LADA_FinalCounts/",
#             "2019-09-03_CountAnalysis.rds", sep="")

path <- paste("J:/",
              "CBMR/",
              "SUN-CBMR-Hansen-Group/",
              "Projects/",
              "LADA/",
              "LADA_Sandra_Evelina/",
              "LADA_JKV/",
              "LADA_R_AfterFlow_Analysis_FinalCounts/",
              "LADA_FinalCounts/",
              "2019-09-03_CountAnalysis.rds", sep="")

TotalCounts<-readRDS(path)
TotalCountsProcess <-
  data.frame(MicrobiomeID=TotalCounts$DF_Save_w$ID,
             CellNorm=TotalCounts$DF_Save_w$Cells_per_g_feces_FR_corrected_mean)
TotalCountsProcess$MicrobiomeID <- paste("L", TotalCountsProcess$MicrobiomeID,
                                         sep="")
SF2<-merge(SF, TotalCountsProcess, by="MicrobiomeID")

#One value is NaN in sample 602059 assigns value as average
SF2$CellNorm[is.nan(SF2$CellNorm)]<-mean(na.omit(SF2$CellNorm))

SF2$CellNormStand<-SF2$CellNorm/mean(SF2$CellNorm) #Watch out for NaN values can assign
                                                   #average, but wants the error
SF3<-merge(Metadata2, SF2, by="MicrobiomeID")
#DESeq divides with sizeFactor (High cell count should have low sizeFactor providing
#larger counts in a sample)
#Still have to consider seq data is relative
SF3$NormRelCell<-SF3$Relative/SF3$CellNormStand
#Overview of normalization factors
aggregate(SF3[, 14:20], list(SF3$Diagnosis), mean) #Have to run heatmap chunk adding sex
```

Group.1 Ratio Poscounts Iterate Relative CellNorm CellNormStand 1 Control 1.021277 1.0816841 1.0816841 1.0024242 20384275367 1.0420944 2 T1D 1.189194 1.3530621 1.3530621 1.1533999 18731782275 0.9576149 3 T2D 1.102417 0.9754019 0.9754019 0.9631018 19624620327 1.0032590 4 LADA 1.154124 1.0508265 1.0508265 0.9635197 18940404238 0.9682802 NormRelCell 1 1.078577 2 1.333993 3 1.076697 4 1.069666

```
                                                   #to metadata
aggregate(SF3[, 14:20], list(SF3$Diagnosis), sd)
```

Group.1 Ratio Poscounts Iterate Relative CellNorm CellNormStand 1 Control 0.5077220 0.3258182 0.3258182 0.2396082 6781846681 0.3467047 2 T1D 0.3607959 0.4887817 0.4887817 0.1896984 5712095609 0.2920164 3 T2D 0.4568660 0.3704665 0.3704665 0.2075670 6146706989 0.3142348 4 LADA 0.5115578

0.4411272 0.4411272 0.2136868 5544046390 0.2834253 NormRelCell 1 0.5133752 2 0.5220606 3 0.4836133 4 0.3592809

```
pairs(SF3[, c(14:17,19,20)], col=SF3$Diagnosis)
```

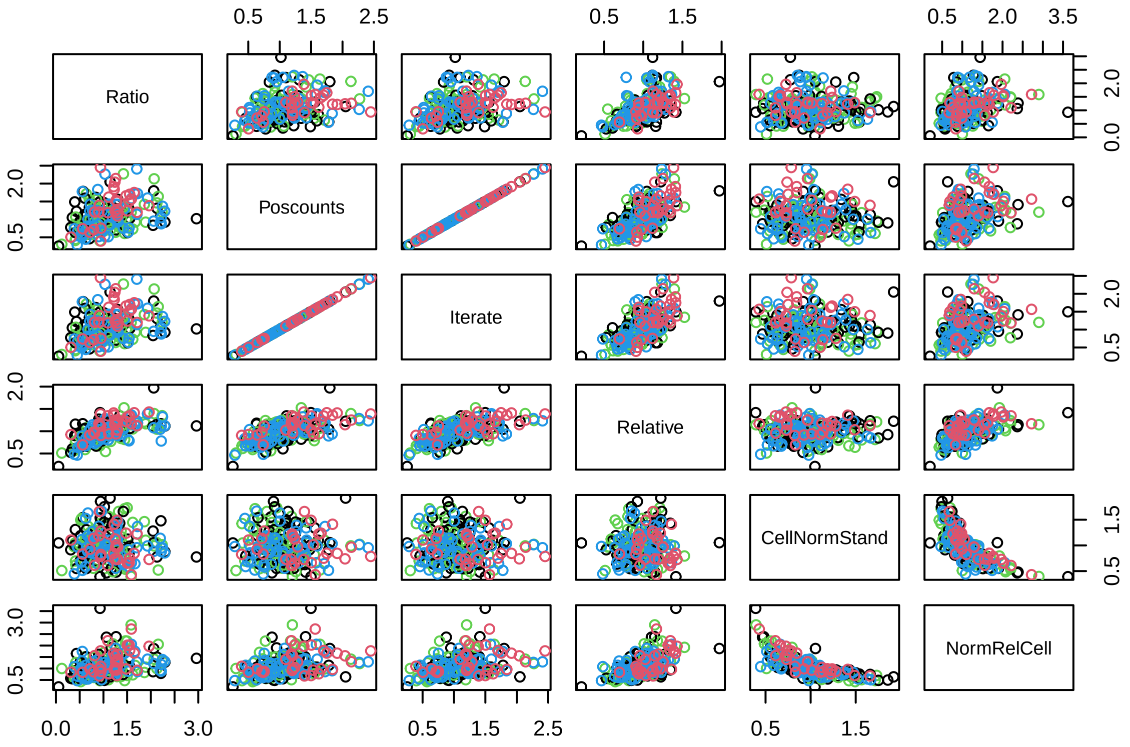


```
pairs(SF3[, c(15,17,19,20)], col=SF3$Diagnosis)
```

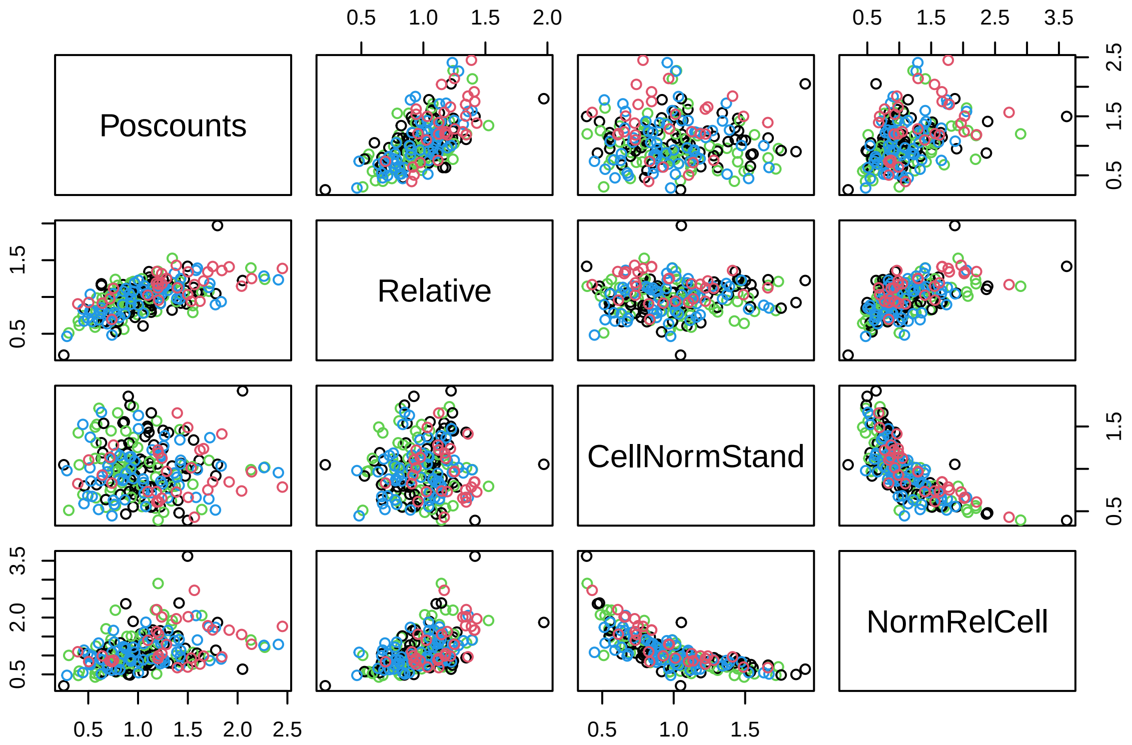


```
#I do want CellNormStand and NormRelCell to be negatively correlated due to DESeq divide
#with sizeFactor.
#It is also good that CellNormStand seems uncorrelated to the other normalization factors.

##Select size factors calculated above for normalization
dds@colData@listData$sizeFactor <- SF3[,20]

##See vignette for note on factor levels
#Filtering of the Counttable. Bare minimum is performed later. Also to reduce memory of

#the dds
dds <- dds[ rowSums(counts(dds))>(50*length(Taxonomy2)),] #Previously 0 instead of 1

#Estimate dispersions and fit the GLM
dds <- DESeq(dds)

res<-results(dds)
summary(res)
```

out of 82 with nonzero total read count adjusted p-value < 0.1 LFC > 0 (up) : 2, 2.4% LFC < 0 (down) : 2, 2.4% outliers [1] : 0, 0% low counts [2] : 0, 0% (mean count < 49) [1] see 'cooksCutoff' argument of ?results [2] see 'independentFiltering' argument of ?results

```
resultsNames(dds)
```

[1] "Intercept" "Diagnosis_T1D_vs_Control" [3] "Diagnosis_T2D_vs_Control" "Diagnosis_LADA_vs_Control"
[5] "BMIq"

```
#Threshold for FDR-adjusted p-values
padj_threshold <- 0.1

if (is.factor(colData(dds)[,"Diagnosis"]) ==  TRUE) {

#Find all two-wise combinations of levels in chosen test factor
test <- combn(levels(colData(dds)[,"Diagnosis"]), 2)
test[,3]<-c("LADA", "Control") #Want LADA first
test[,5]<-c("LADA", "T1D") #Want LADA first
test[,6]<-c("LADA", "T2D") #Want LADA first
foo = test[1,]
poo = test[2,]

#Do DESeq2 analysis on all combinations of levels and save as list
result <- vector("list",length(foo))
for (i in 1:length(foo) ) {
  for (j in 1:length(poo)) {
    result[i] = results(dds, contrast = c("Diagnosis" ,foo[i], poo[i]))
  }
}

#Produce output tables with significant taxa from each comparison
res_list <- vector("list",length(foo))
for (i in 1:length(result) ) {
  res_stat <- data.frame(result[i])
  res_stat <- res_stat[is.na(res_stat$pvalue) == FALSE,]
  stat_sig <- na.omit(res_stat[res_stat$padj < padj_threshold,])
  res_stat$gene <- rownames(res_stat)
  res_stat$g1 <- test[1,i]
  res_stat$g2 <- test[2,i]
  res_stat$compare <- paste(test[1,i], "vs", test[2,i])
  res_list[i] <- list(res_stat)
}

res_stat <- do.call('rbind', res_list)
rownames(res_stat) <- 1:nrow(res_stat)
res_stat <- res_stat[with(res_stat, order(padj, pvalue, abs(log2FoldChange))),]
stat_sig <- na.omit(res_stat[res_stat$padj < padj_threshold,])
}

if (is.numeric(colData(dds)[,"Diagnosis"]) ==  TRUE) {

res_stat <- data.frame((results(dds)))
res_stat$maxCooks <- apply(assays(dds)[["cooks"]], 1, max)
res_stat <- res_stat[is.na(res_stat$pvalue) == FALSE,]
res_stat <- res_stat[with(res_stat, order(padj, pvalue, abs(log2FoldChange))), ]
res_stat$gene <- rownames(res_stat)
max_cooks <- quantile(na.omit(res_stat$maxCooks), cooks_quantile_cutoff)
stat_sig <-
  na.omit(res_stat[res_stat$padj < padj_threshold & res_stat$maxCooks < max_cooks,])
}
```

```r
#All comparisons
write.table(res_stat, file="DESeqRes_Orgs.txt", quote = F, row.names = F, sep="\t")

#Significant taxa in table
if (nrow(stat_sig) > 0) {
kable(stat_sig, row.names = F)
write.table(stat_sig, file="DESeqRes_SigOrgs.txt", quote = F, row.names = F, sep="\t")
} else {
  print("No significant taxa were found.")
}
print("Significant Wald")
```

[1] "Significant Wald"

```r
kable(stat_sig)
```

	baseMean	log2FoldChange	lfcSE	stat	pvalue	padj	gene	g1	g2	compare
407	1334.40881	3.6657267	0.6670490	5.495438	0.0000000	0.0000032	Escherichia/Shigella	LADA	T1D	LADA vs T1D
243	1334.40881	2.7557712	0.5289800	5.209594	0.0000002	0.0000155	Escherichia/Shigella	LADA	Control	LADA vs Control
319	265.43054	-4.5396043	0.9396150	-4.831345	0.0000014	0.0001112	Veillonella	T1D	T2D	T1D vs T2D
325	1334.40881	-3.1124702	0.6711965	-4.637197	0.0000035	0.0001448	Escherichia/Shigella	T1D	T2D	T1D vs T2D
289	941.98315	-1.2372614	0.2787381	-4.438796	0.0000090	0.0002473	Butyricicoccus	T1D	T2D	T1D vs T2D
261	1113.74127	-1.5492262	0.3749662	-4.131643	0.0000360	0.0007384	Parabacteroides	T1D	T2D	T1D vs T2D
161	1334.40881	-2.2025147	0.5349798	-4.117005	0.0000384	0.0031474	Escherichia/Shigella	Control	T2D	Control vs T2D
277	780.88361	-1.3452813	0.3657440	-3.678205	0.0002349	0.0038521	Lachnoclostridium	T1D	T2D	T1D vs T2D
113	780.88361	-1.0372730	0.2915573	-3.557699	0.0003741	0.0153388	Lachnoclostridium	Control	T2D	Control vs T2D
371	941.98315	0.9528609	0.2770232	3.439643	0.0005825	0.0238817	Butyricicoccus	LADA	T1D	LADA vs T1D
412	48.68949	0.9942958	0.2751489	3.613665	0.0003019	0.0247557	Actinomyces	LADA	T2D	LADA vs T2D
227	87.35346	1.1110681	0.3310424	3.356271	0.0007900	0.0323904	UBA1819	LADA	Control	LADA vs Control
208	12297.06909	-0.8962497	0.3019667	-2.968041	0.0029970	0.0641923	Faecalibacterium	LADA	Control	LADA vs Control
199	705.55318	-1.2942909	0.4429952	-2.921681	0.0034815	0.0641923	Lachnospiraceae_NK4A136_group	LADA	Control	LADA vs Control
213	649.61339	0.7179311	0.2519946	2.848995	0.0043858	0.0641923	Ruminiclostridium_5	LADA	Control	LADA vs Control
235	56.39729	1.2954165	0.4651192	2.785128	0.0053507	0.0641923	Family.Erysipelotrichaceae	LADA	Control	LADA vs Control
166	48.68949	0.7612893	0.2760578	2.757717	0.0058207	0.0641923	Actinomyces	LADA	Control	LADA vs Control

	baseMean	log2FoldChange	lfcSE	stat	pvalue	padj	gene	g1	g2	compare
203	1431.19573	-0.9374024	0.3429058	-2.733702	0.0062627	0.0641923	Roseburia	LADA	Control	LADA vs Control
196	213.93756	-1.1475016	0.4276126	-2.683507	0.0072854	0.0663784	Lachnospira	LADA	Control	LADA vs Control
344	1062.10266	2.6072896	0.8657027	3.011761	0.0025974	0.0676607	Lactobacillus	LADA	T1D	LADA vs T1D
346	1347.24525	-1.4388295	0.4896864	-2.938267	0.0033005	0.0676607	Christensenellaceae_R.7_group	LADA	T1D	LADA vs T1D
43	941.98315	0.8272636	0.2674796	3.092810	0.0019827	0.0905858	Butyricicoccus	Control	T1D	Control vs T1D
39	1431.19573	1.2775012	0.4174109	3.060536	0.0022094	0.0905858	Roseburia	Control	T1D	Control vs T1D
194	2986.10151	-0.7635978	0.3034354	-2.516509	0.0118524	0.0971897	Fusicatenibacter	LADA	Control	LADA vs Control
44	12297.06901	1.0694890	0.3675676	2.909639	0.0036185	0.0989047	Faecalibacterium	Control	T1D	Control vs T1D

```r
kable(stat_sig[,c(5:7,10)])
```

	pvalue	padj	gene	compare
407	0.0000000	0.0000032	Escherichia/Shigella	LADA vs T1D
243	0.0000002	0.0000155	Escherichia/Shigella	LADA vs Control
319	0.0000014	0.0001112	Veillonella	T1D vs T2D
325	0.0000035	0.0001448	Escherichia/Shigella	T1D vs T2D
289	0.0000090	0.0002473	Butyricicoccus	T1D vs T2D
261	0.0000360	0.0007384	Parabacteroides	T1D vs T2D
161	0.0000384	0.0031474	Escherichia/Shigella	Control vs T2D
277	0.0002349	0.0038521	Lachnoclostridium	T1D vs T2D
113	0.0003741	0.0153388	Lachnoclostridium	Control vs T2D
371	0.0005825	0.0238817	Butyricicoccus	LADA vs T1D
412	0.0003019	0.0247557	Actinomyces	LADA vs T2D
227	0.0007900	0.0323904	UBA1819	LADA vs Control
208	0.0029970	0.0641923	Faecalibacterium	LADA vs Control
199	0.0034815	0.0641923	Lachnospiraceae_NK4A136_group	LADA vs Control
213	0.0043858	0.0641923	Ruminiclostridium_5	LADA vs Control
235	0.0053507	0.0641923	Family.Erysipelotrichaceae.	LADA vs Control
166	0.0058207	0.0641923	Actinomyces	LADA vs Control
203	0.0062627	0.0641923	Roseburia	LADA vs Control
196	0.0072854	0.0663784	Lachnospira	LADA vs Control
344	0.0025974	0.0676607	Lactobacillus	LADA vs T1D
346	0.0033005	0.0676607	Christensenellaceae_R.7_group	LADA vs T1D
43	0.0019827	0.0905858	Butyricicoccus	Control vs T1D
39	0.0022094	0.0905858	Roseburia	Control vs T1D
194	0.0118524	0.0971897	Fusicatenibacter	LADA vs Control
44	0.0036185	0.0989047	Faecalibacterium	Control vs T1D

```r
##Venn diagram comparing LADA to other groups
sig_LADA <- subset(stat_sig, g1=="LADA")
sig_LADA_T1D <- subset(sig_LADA, g2=="T1D") %>% dplyr::select(one_of("gene")) %>%
```

```
  pull(gene)
sig_LADA_T2D <- subset(sig_LADA, g2=="T2D") %>% dplyr::select(one_of("gene")) %>%
  pull(gene)
sig_LADA_Control <- subset(sig_LADA, g2=="Control") %>% dplyr::select(one_of("gene")) %>%
  pull(gene)

#Colors
myCol <- c( "#1F78B4", "#33A02C", "#666666")
#
temp <- venn.diagram(list(T1D = sig_LADA_T1D,
                          T2D = sig_LADA_T2D,
                          Control = sig_LADA_Control), filename = NULL,

                     # Circles
                     lwd = 2,
                     lty = 'blank',
                     fill = myCol,

                     # Numbers
                     cex = 3,
                     fontface = "bold",
                     fontfamily = "sans",

                     # Set names
                     cat.cex = 3,
                     cat.fontface = "bold",
                     cat.default.pos = "outer",
                     cat.pos = c(0, 0, 0),
                     cat.dist = c(0.085, 0.085, 0.024),
                     cat.fontfamily = "sans",
                     rotation = 1)

grid.arrange(gTree(children=temp))
```

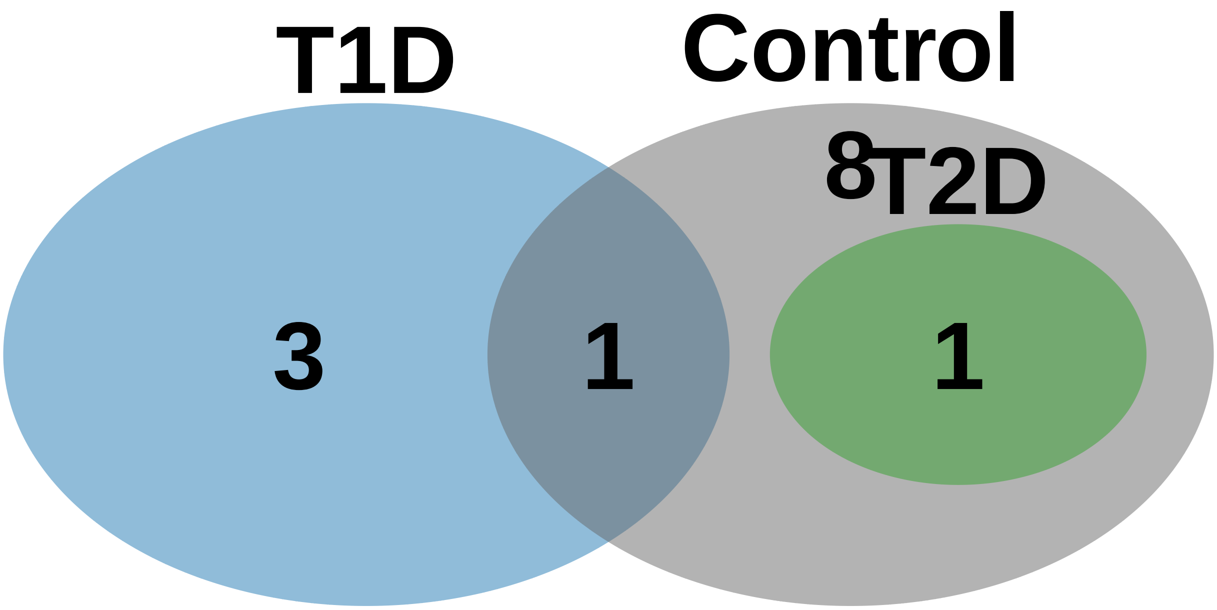


```
Fig2List[[ "VennLADA" ]] <-
  gTree(children=temp, top="N differential abundant relative to LADA")

#Genera LADA that are significantly different from all other groups
intersect(intersect(sig_LADA_T1D,sig_LADA_T2D),sig_LADA_Control)
```

character(0)

```
##Venn diagram comparing T1D to other groups
sig_T1D <- subset(stat_sig, g1=="T1D" | g2=="T1D")
sig_T1D_LADA <- subset(sig_T1D, g1=="LADA") %>% dplyr::select(one_of("gene")) %>%
  pull(gene)
sig_T1D_T2D <- subset(sig_T1D, g2=="T2D") %>% dplyr::select(one_of("gene")) %>%
  pull(gene)
sig_T1D_Control <- subset(sig_T1D, g1=="Control") %>% dplyr::select(one_of("gene")) %>%
  pull(gene)
#Colors
myCol <- c( "#E31A1C", "#33A02C", "#666666")
#
temp <- venn.diagram(list(LADA = sig_T1D_LADA,
                          T2D = sig_T1D_T2D,
                          Control = sig_T1D_Control), filename = NULL,

                     # Circles
                     lwd = 2,
```

```
                      lty = 'blank',
                      fill = myCol,

                      # Numbers
                      cex = 2,
                      fontface = "bold",
                      fontfamily = "sans",

                      # Set names
                      cat.cex = 2,
                      cat.fontface = "bold",
                      cat.default.pos = "outer",
                      cat.pos = c(0, 0, 0),
                      cat.dist = c(0.055, 0.055, 0.024),
                      cat.fontfamily = "sans",
                      rotation = 1)

grid.arrange(gTree(children=temp))
```

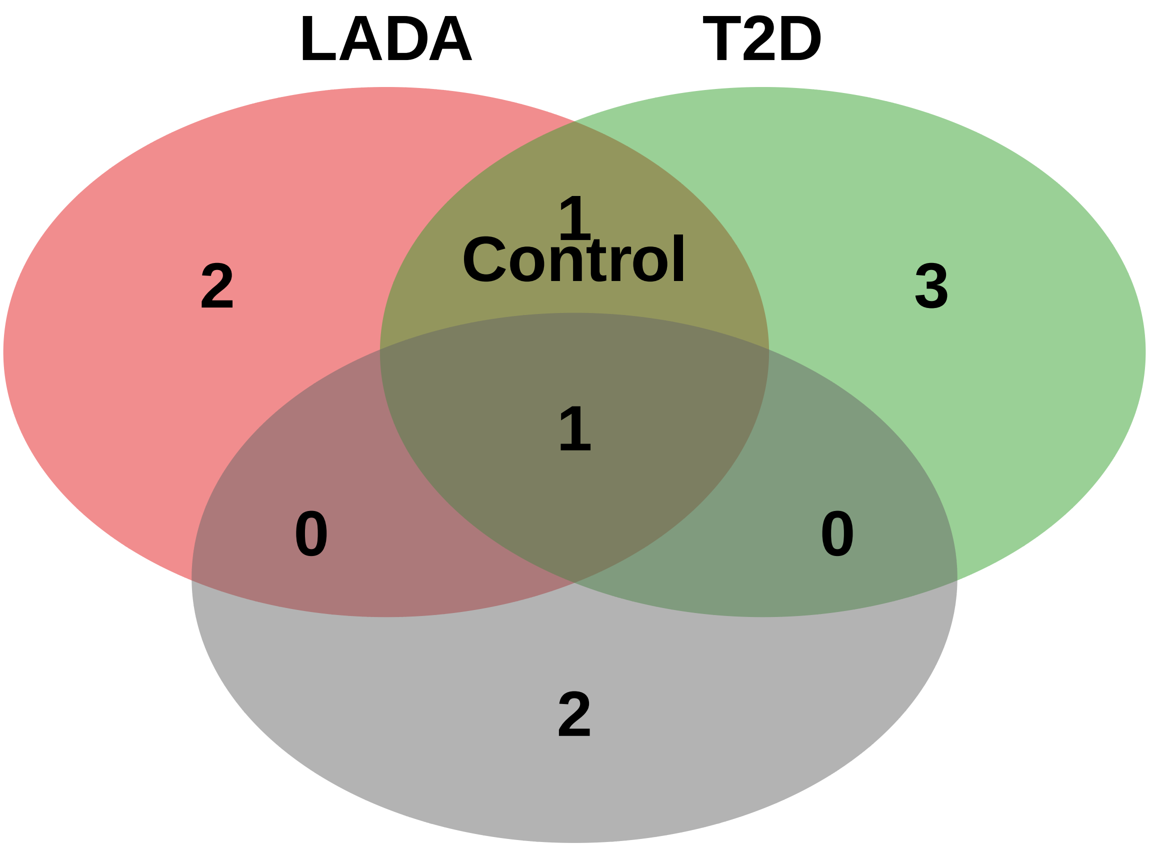


```
Fig2List[[ "VennT1D" ]] <-
  gTree(children=temp, top="N differential abundant relative to T1D")

#Genera T1D that are significantly different from all other groups
intersect(intersect(sig_T1D_LADA,sig_T1D_T2D),sig_T1D_Control)
```

[1] "Butyricicoccus"

```
##Venn diagram comparing T2D to other groups
sig_T2D <- subset(stat_sig, g1=="T2D" | g2=="T2D")
sig_T2D_LADA <- subset(sig_T2D, g1=="LADA") %>% dplyr::select(one_of("gene")) %>%
  pull(gene)
sig_T2D_T1D <- subset(sig_T2D, g1=="T1D") %>% dplyr::select(one_of("gene")) %>%
  pull(gene)
sig_T2D_Control <- subset(sig_T2D, g1=="Control") %>% dplyr::select(one_of("gene")) %>%
  pull(gene)
#Colors
myCol <- c( "#E31A1C", "#1F78B4", "#666666")
#
temp <- venn.diagram(list(LADA = sig_T2D_LADA,
                          T1D = sig_T2D_T1D,
                          Control = sig_T2D_Control), filename = NULL,

                     # Circles
                     lwd = 2,
                     lty = 'blank',
                     fill = myCol,

                     # Numbers
                     cex = 2,
                     fontface = "bold",
                     fontfamily = "sans",

                     # Set names
                     cat.cex = 2,
                     cat.fontface = "bold",
                     cat.default.pos = "outer",
                     cat.pos = c(0, 0, 0),
                     cat.dist = c(0.055, 0.055, 0.024),
                     cat.fontfamily = "sans",
                     rotation = 1)

grid.arrange(gTree(children=temp))
```

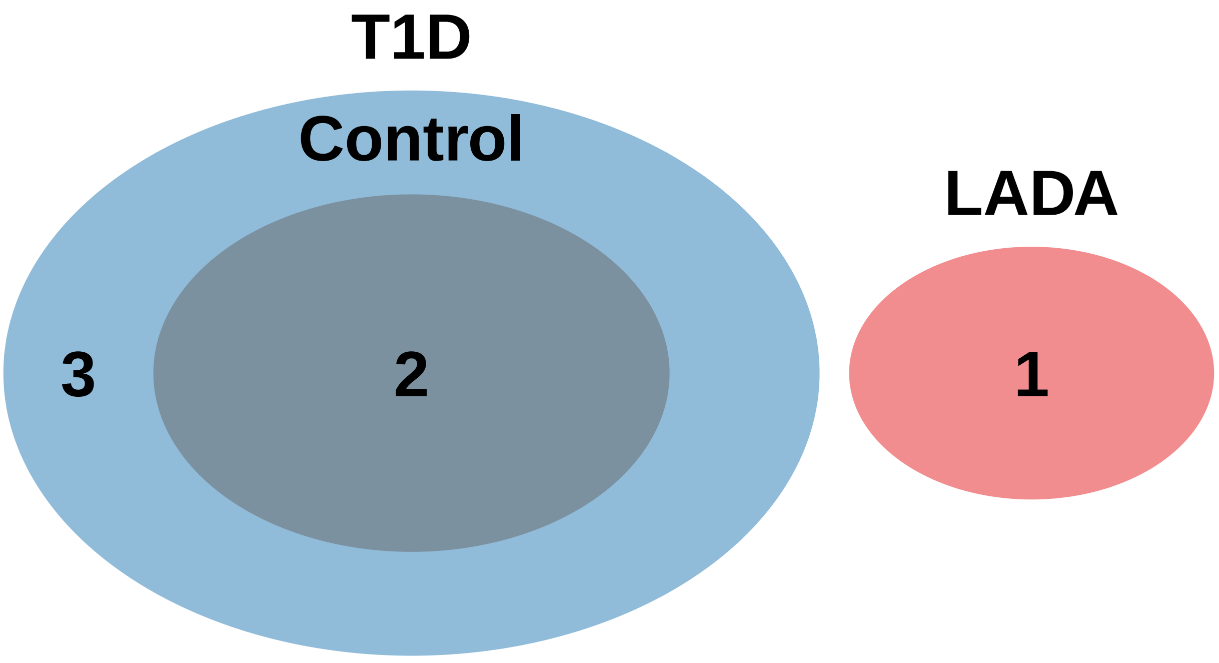


```
Fig2List[[ "VennT2D" ]] <-
  gTree(children=temp, top="N differential abundant relative to T2D")

#Genera T2D that are significantly different from all other groups
intersect(intersect(sig_T2D_LADA,sig_T2D_T1D),sig_T2D_Control)
```

character(0)

```
##Venn diagram comparing controls to other groups
sig_Control <- subset(stat_sig, g1=="Control" | g2=="Control")
sig_Control_LADA <- subset(sig_Control, g1=="LADA") %>% dplyr::select(one_of("gene")) %>%
  pull(gene)
sig_Control_T1D <- subset(sig_Control, g2=="T1D") %>% dplyr::select(one_of("gene")) %>%
  pull(gene)
sig_Control_T2D <- subset(sig_Control, g2=="T2D") %>% dplyr::select(one_of("gene")) %>%
  pull(gene)
#Colors
myCol <- c( "#E31A1C", "#1F78B4", "#33A02C")
#
temp <- venn.diagram(list(LADA = sig_Control_LADA,
                          T1D = sig_Control_T1D,
                          T2D = sig_Control_T2D), filename = NULL,

                     # Circles
                     lwd = 2,
```

```
                     lty = 'blank',
                     fill = myCol,

                     # Numbers
                     cex = 3,
                     fontface = "bold",
                     fontfamily = "sans",

                     # Set names
                     cat.cex = 3,
                     cat.fontface = "bold",
                     cat.default.pos = "outer",
                     cat.pos = c(0, 0, 0),
                     cat.dist = c(0.055, 0.055, 0.024),
                     cat.fontfamily = "sans",
                     rotation = 1)

grid.arrange(gTree(children=temp))
```

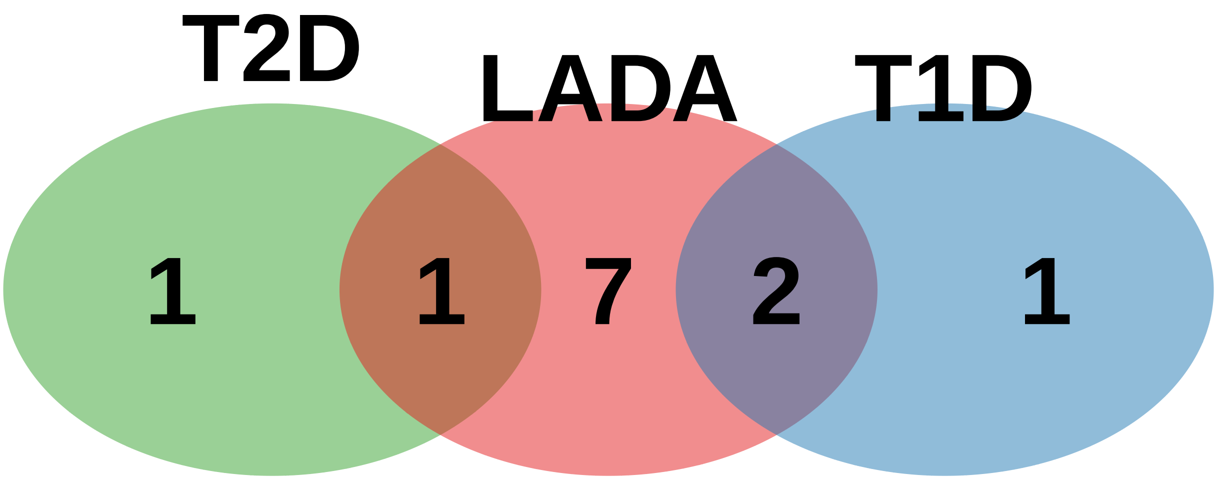


```
Fig2List[[ "VennControls" ]] <-
  gTree(children=temp, top="N differential abundant relative to controls")

#Genera Controls that are significantly different from all other groups
intersect(intersect(sig_T2D_LADA,sig_T2D_T1D),sig_T2D_Control)
```

character(0)

```
##Create boxplots / violin plots
#Normalize to get cells pr. gram feces
if (setequal(colnames(Taxonomy2), SF3$MicrobiomeID)==FALSE) {
  stop("Metadata and Taxonomy out of sync")
}

#Total sum scaling (Use relative abundances)
Taxonomy3<-sweep(Taxonomy2, 2, colSums(Taxonomy), FUN="/")
#Obtain values as cells pr. gram feces
Taxonomy3<-sweep(Taxonomy3, 2, SF3$CellNorm, FUN="*")
#Make to micro gram
Taxonomy3<-Taxonomy3/10^6


##Select organisms
SelOrgs<-unique(stat_sig$gene)
##Add SCFA producers see Venegas et al. 2019
SelOrgs<-unique(c(SelOrgs,
                  "Faecalibacterium", #prausnitzii
#                  "Clostridium", #leptum present as sensu stricto 1
#                  "Eubacterium", #rectale or hallii, here linosum
                  "Roseburia",  #
                  "Anaerostipes",
                  "Bifidobacterium",
                  "Butyricicoccus",
                  "Akkermansia"))

tTaxSelect<-dplyr::select(as.data.frame(t(Taxonomy3)), one_of(c(SelOrgs)))
#Reducing the number because duplicates are removed
tTaxSelect<-add_rownames(tTaxSelect, "MicrobiomeID")
Plotting<-merge(SF3, tTaxSelect, by="MicrobiomeID")

##Might have issues with special characters, so this chunk might be needed
SelOrgs<-str_replace(SelOrgs, " ","_")
SelOrgs<-str_replace(SelOrgs, "/","_")
SelOrgs<-gsub("\\[|\\]", "", SelOrgs)
SelOrgs<-str_replace(SelOrgs, ":","_")
colnames(Plotting) <- str_replace(colnames(Plotting)," ","_")
colnames(Plotting) <- str_replace(colnames(Plotting),"/","_")
colnames(Plotting) <- gsub("\\[|\\]", "", colnames(Plotting))
colnames(Plotting) <- str_replace(colnames(Plotting),":","_")

#Plot logs on y axis
Plotting2 <- bind_cols(Plotting[,1:16], log10(Plotting[,17:ncol(Plotting)]+1))
#See end of chunk for boxplots removed the violinplots, because of redundancy


##Create vulcano plots
#Cut offs Benjamini-Hochberg method to add to vulcano plot
#Don't know how to get exact so it is a cut-off corresponding to the BH method
BHAll<-aggregate(stat_sig[, 5:6], list(stat_sig$compare), max)
row.names(BHAll)<-BHAll$Group.1
```

```
BHLadaCon <- BHAll[which(rownames(BHAll)=="LADA vs Control"),
                   which(colnames(BHAll)=="pvalue")]
BHLadaT1D <- BHAll[which(rownames(BHAll)=="LADA vs T1D"),
                   which(colnames(BHAll)=="pvalue")]
BHLadaT2D <- BHAll[which(rownames(BHAll)=="LADA vs T2D"),
                   which(colnames(BHAll)=="pvalue")]


#Vulcano plot
res_stat$minuslog10<--log(res_stat$pvalue)
#
#range(res_stat$minuslog10)
#range(res_stat$log2FoldChange)

names(res_stat)[names(res_stat) == 'gene'] <- 'Genus'
Feature2<-merge(res_stat, Feature, by="Genus")

#Create column org grouping for colouring
Feature2$Phyla <- ifelse(Feature2$Phylum=="Firmicutes", "Firmicutes",
          ifelse(Feature2$Phylum=="Proteobacteria", "Proteobacteria",
          ifelse(Feature2$Phylum=="Actinobacteria", "Actinobacteria",
          ifelse(Feature2$Phylum=="Bacteroidetes", "Bacteroidetes",
          ifelse(Feature2$Phylum=="Euryarchaeota", "Euryarchaeota",
                 "Other")))))
#Order Phyla for plotting
Feature2$Phyla <- factor(Feature2$Phyla, levels=c("Proteobacteria", "Bacteroidetes",
                                                  "Actinobacteria", "Firmicutes",
                                                  "Euryarchaeota", "Other"))


##Summary significant orgs
#aggregate(Plotting[, 17:ncol(Plotting)], list(Plotting$Diagnosis), mean)
#aggregate(Plotting[, 17:ncol(Plotting)], list(Plotting$Diagnosis), sd)
zeroes <- function(x){
  sum(x == 0)
}
#aggregate(Plotting[, 17:ncol(Plotting)], list(Plotting$Diagnosis), zeroes)
zerocounts <- aggregate(Plotting[, 17:ncol(Plotting)], list(Plotting$Diagnosis), zeroes)
#bind_cols(zerocounts[,1], zerocounts[,2:ncol(zerocounts)]/c(70,30,70,70)*100)


#Add prevalence to plotting
prevalence<-data.frame((240-apply(Taxonomy, 1, zeroes))/240*100)
colnames(prevalence) <- c("Prevalence")
prevalence<-add_rownames(prevalence, var = "Genus")
Feature2 <- merge(Feature2, prevalence, by="Genus")


#Define boundaries
#Always run first without these lines to get indication of very low and high
#log2FoldChange and pvalue
Featurein <- filter(Feature2, -3<log2FoldChange & log2FoldChange<3 & 10>minuslog10)
Featureout <- filter(Feature2, -3>log2FoldChange | 3<log2FoldChange | 10<minuslog10)
```

```r
Featureout$log2FoldChange[Featureout$log2FoldChange > 3] <- 3
Featureout$log2FoldChange[Featureout$log2FoldChange < (-3)] <- -3
Featureout$minuslog10[Featureout$minuslog10 > 10] <- 10


#Add text to vulcano plots
p1<-ggplot() +
  geom_point(data=Featurein[which(Featurein$Phyla=="Proteobacteria" &
                                    Featurein$compare=="LADA vs T1D"), ],
             aes(x=log2FoldChange, y=minuslog10, group=Genus), color="#A6CEE3", shape=16,
             size=Featurein[which(Featurein$Phyla=="Proteobacteria" &
                                    Featurein$compare=="LADA vs T1D"), ]
             [,ncol(Featurein)]/40+0.5) +
  #did not add jitter to the first on purpose. Sizes are scaled from percent
  #to the range 0.5-3
  geom_point(data=Featureout[which(Featureout$Phyla=="Proteobacteria" &
                                     Featureout$compare=="LADA vs T1D"), ],
             aes(x=log2FoldChange, y=minuslog10, group=Genus), color="#A6CEE3", shape=18,
             size=Featureout[which(Featureout$Phyla=="Proteobacteria" &
                                     Featureout$compare=="LADA vs T1D"), ]
             [,ncol(Featureout)]/40+0.5) +
  geom_point(data=Featurein[which(Featurein$Phyla=="Bacteroidetes" &
                                    Featurein$compare=="LADA vs T1D"), ],
             aes(x=log2FoldChange, y=minuslog10, group=Genus), color="#FB9A99", shape=16,
             size=Featurein[which(Featurein$Phyla=="Bacteroidetes" &
                                    Featurein$compare=="LADA vs T1D"), ]
             [,ncol(Featurein)]/40+0.5) +
  geom_jitter(data=Featureout[which(Featureout$Phyla=="Bacteroidetes" &
                                      Featureout$compare=="LADA vs T1D"), ],
             aes(x=log2FoldChange, y=minuslog10, group=Genus), color="#FB9A99", shape=18,
             size=Featureout[which(Featureout$Phyla=="Bacteroidetes" &
                                     Featureout$compare=="LADA vs T1D"), ]
             [,ncol(Featureout)]/40+0.5, width = 0.1, height = 0.1) +
  geom_point(data=Featurein[which(Featurein$Phyla=="Actinobacteria" &
                                    Featurein$compare=="LADA vs T1D"), ],
             aes(x=log2FoldChange, y=minuslog10, group=Genus), color="#FDBF6F", shape=16,
             size=Featurein[which(Featurein$Phyla=="Actinobacteria" &
                                    Featurein$compare=="LADA vs T1D"), ]
             [,ncol(Featurein)]/40+0.5) +
  geom_jitter(data=Featureout[which(Featureout$Phyla=="Actinobacteria" &
                                      Featureout$compare=="LADA vs T1D"), ],
             aes(x=log2FoldChange, y=minuslog10, group=Genus), color="#FDBF6F", shape=18,
             size=Featureout[which(Featureout$Phyla=="Actinobacteria" &
                                     Featureout$compare=="LADA vs T1D"), ]
             [,ncol(Featureout)]/40+0.5, width = 0.1, height = 0.1) +
  geom_point(data=Featurein[which(Featurein$Phyla=="Firmicutes" &
                                    Featurein$compare=="LADA vs T1D"), ],
             aes(x=log2FoldChange, y=minuslog10, group=Genus),
             color="#B2DF8A", shape=16, alpha=0.75,
             size=Featurein[which(Featurein$Phyla=="Firmicutes" &
                                    Featurein$compare=="LADA vs T1D"), ]
             [,ncol(Featurein)]/40+0.5) +
  geom_jitter(data=Featureout[which(Featureout$Phyla=="Firmicutes" &
```

```
                                       Featureout$compare=="LADA vs T1D"), ],
             aes(x=log2FoldChange, y=minuslog10, group=Genus),
             color="#B2DF8A", shape=18,
             size=Featureout[which(Featureout$Phyla=="Firmicutes" &
                                      Featureout$compare=="LADA vs T1D"), ]
             [,ncol(Featureout)]/40+0.5, width = 0.1, height = 0.1) +
  geom_point(data=Featurein[which(Featurein$Phyla=="Euryarchaeota" &
                                     Featurein$compare=="LADA vs T1D"), ],
             aes(x=log2FoldChange, y=minuslog10, group=Genus), color="#CAB2D6", shape=16,
             size=Featurein[which(Featurein$Phyla=="Euryarchaeota" &
                                     Featurein$compare=="LADA vs T1D"), ]
             [,ncol(Featurein)]/40+0.5) +
  geom_jitter(data=Featureout[which(Featureout$Phyla=="Euryarchaeota" &
                                       Featureout$compare=="LADA vs T1D"), ],
             aes(x=log2FoldChange, y=minuslog10, group=Genus), color="#CAB2D6", shape=18,
             size=Featureout[which(Featureout$Phyla=="Euryarchaeota" &
                                      Featureout$compare=="LADA vs T1D"), ]
             [,ncol(Featureout)]/40+0.5, width = 0.1, height = 0.1) +
  geom_point(data=Featurein[which(Featurein$Phyla=="Other" &
                                     Featurein$compare=="LADA vs T1D"), ],
             aes(x=log2FoldChange, y=minuslog10, group=Genus), color="#B3B3B3", shape=16,
             size=Featurein[which(Featurein$Phyla=="Other" &
                                     Featurein$compare=="LADA vs T1D"), ]
             [,ncol(Featurein)]/40+0.5) +
  geom_jitter(data=Featureout[which(Featureout$Phyla=="Other" &
                                       Featureout$compare=="LADA vs T1D"), ],
             aes(x=log2FoldChange, y=minuslog10, group=Genus), color="#B3B3B3", shape=18,
             size=Featureout[which(Featureout$Phyla=="Other" &
                                      Featureout$compare=="LADA vs T1D"), ]
             [,ncol(Featureout)]/40+0.5, width = 0.1, height = 0.1) +
  geom_text(data=Featurein[which(Featurein$padj<padj_threshold &
                                    Featurein$compare=="LADA vs T1D"), ],
             aes(x=log2FoldChange, y=minuslog10, group=Genus, label=Genus),
            nudge_y = 0.2, size=2.5) +
  geom_text(data=Featureout[which(Featureout$padj<padj_threshold &
                                     Featureout$compare=="LADA vs T1D"), ],
             aes(x=log2FoldChange, y=minuslog10, group=Genus, label=Genus),
            nudge_x=0.6, nudge_y = -0.2, size=2.5) +
#  geom_text(data=Featureout[which(Featureout$padj<padj_threshold &
#                                   Featureout$compare=="LADA vs T1D" &
#                                   Featureout$Genus=="Rikenellaceae_RC9_gut_group"), ],
#              aes(x=log2FoldChange, y=minuslog10, group=Genus, label=Genus),
#             nudge_x=1.22, nudge_y = 0) +
  geom_hline(yintercept = -log(0.05), linetype="dashed") +
  geom_hline(yintercept = -log(BHLadaT1D), linetype="dashed", alpha=0.1) +
  geom_vline(xintercept = -1, linetype="dashed") +
  geom_vline(xintercept = 1, linetype="dashed") +
  theme_bw() +
  ggtitle("T1D") +
  xlab("Log2 fold change") +
  ylab("-log10(P)") +
  theme(panel.grid.major = element_blank(),
        panel.grid.minor = element_blank(),
```

```
        axis.title=element_text(size=22),
        legend.position="bottom",
        legend.title=element_text(size=20),
        legend.text=element_text(size=20),
        axis.text.x = element_text(angle = 45, hjust = 1, size=12),
        axis.text.y = element_text(angle = 45, hjust = 1, size=12),
        plot.title = element_text(size = 22, hjust=0.5))
#ggplotly(p1)
p1
```

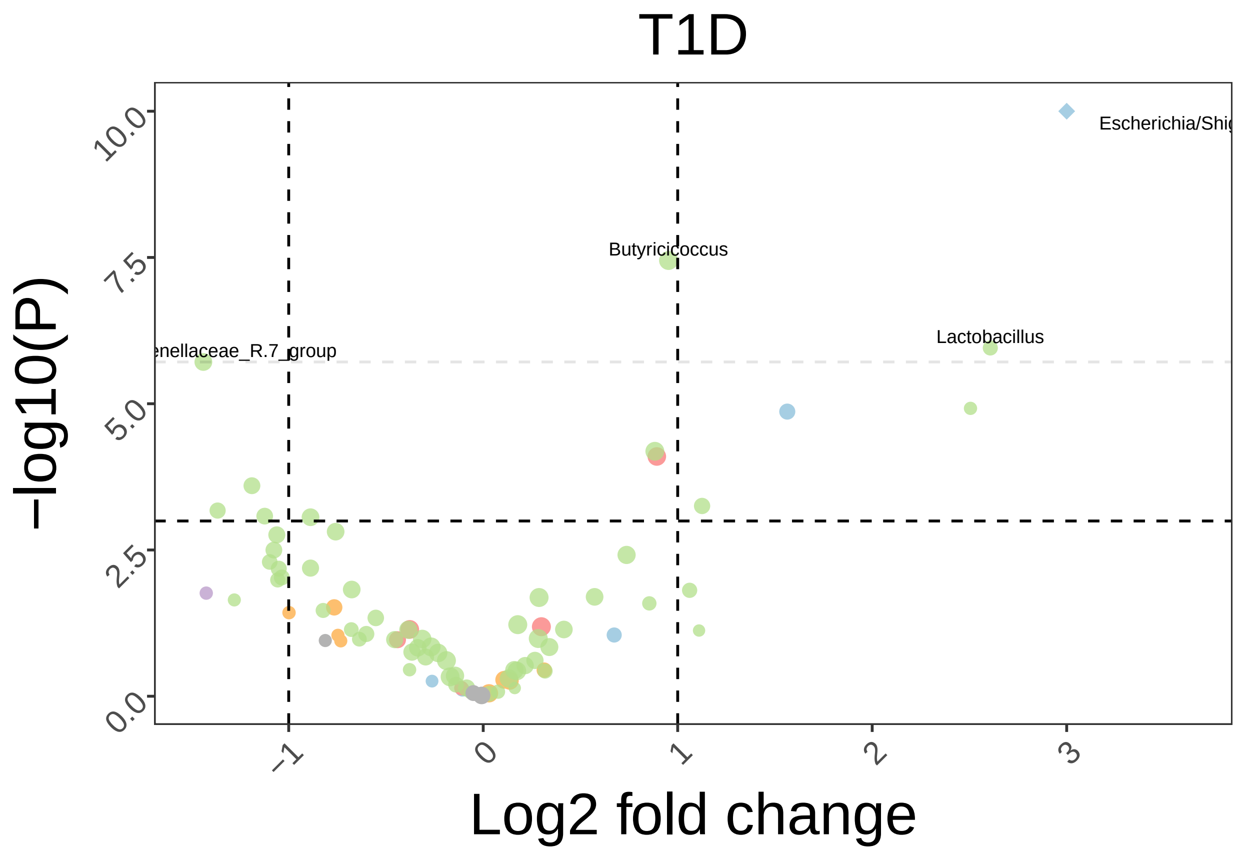


```
Fig2List[["vulcLadaT1D"]] <- p1


p2<-ggplot() +
  geom_point(data=Featurein[which(Featurein$Phyla=="Proteobacteria" &
                                    Featurein$compare=="LADA vs T2D"), ],
             aes(x=log2FoldChange, y=minuslog10, group=Genus), color="#A6CEE3", shape=16,
             size=Featurein[which(Featurein$Phyla=="Proteobacteria" &
                                    Featurein$compare=="LADA vs T2D"), ]
             [,ncol(Featurein)]/40+0.5) +
  #did not add jitter to the first on purpose. Sizes are scaled from percent
  #to the range 0.5-3
  geom_point(data=Featureout[which(Featureout$Phyla=="Proteobacteria" &
                                     Featureout$compare=="LADA vs T2D"), ],
             aes(x=log2FoldChange, y=minuslog10, group=Genus), color="#A6CEE3", shape=18,
```

```
            size=Featureout[which(Featureout$Phyla=="Proteobacteria" &
                                    Featureout$compare=="LADA vs T2D"), ]
            [,ncol(Featureout)]/40+0.5) +
  geom_point(data=Featurein[which(Featurein$Phyla=="Bacteroidetes" &
                                   Featurein$compare=="LADA vs T2D"), ],
            aes(x=log2FoldChange, y=minuslog10, group=Genus), color="#FB9A99", shape=16,
            size=Featurein[which(Featurein$Phyla=="Bacteroidetes" &
                                   Featurein$compare=="LADA vs T2D"), ]
            [,ncol(Featurein)]/40+0.5) +
  geom_jitter(data=Featureout[which(Featureout$Phyla=="Bacteroidetes" &
                                     Featureout$compare=="LADA vs T2D"), ],
            aes(x=log2FoldChange, y=minuslog10, group=Genus), color="#FB9A99", shape=18,
            size=Featureout[which(Featureout$Phyla=="Bacteroidetes" &
                                    Featureout$compare=="LADA vs T2D"), ]
            [,ncol(Featureout)]/40+0.5,
            width = 0.1, height = 0.1) +
  geom_point(data=Featurein[which(Featurein$Phyla=="Actinobacteria" &
                                   Featurein$compare=="LADA vs T2D"), ],
            aes(x=log2FoldChange, y=minuslog10, group=Genus), color="#FDBF6F", shape=16,
            size=Featurein[which(Featurein$Phyla=="Actinobacteria" &
                                   Featurein$compare=="LADA vs T2D"), ]
            [,ncol(Featurein)]/40+0.5) +
  geom_jitter(data=Featureout[which(Featureout$Phyla=="Actinobacteria" &
                                     Featureout$compare=="LADA vs T2D"), ],
            aes(x=log2FoldChange, y=minuslog10, group=Genus), color="#FDBF6F", shape=18,
            size=Featureout[which(Featureout$Phyla=="Actinobacteria" &
                                    Featureout$compare=="LADA vs T2D"), ]
            [,ncol(Featureout)]/40+0.5, width = 0.1, height = 0.1) +
  geom_point(data=Featurein[which(Featurein$Phyla=="Firmicutes" &
                                   Featurein$compare=="LADA vs T2D"), ],
            aes(x=log2FoldChange, y=minuslog10, group=Genus),
            color="#B2DF8A", shape=16, alpha=0.75,
            size=Featurein[which(Featurein$Phyla=="Firmicutes" &
                                   Featurein$compare=="LADA vs T2D"), ]
            [,ncol(Featurein)]/40+0.5) +
  geom_jitter(data=Featureout[which(Featureout$Phyla=="Firmicutes" &
                                     Featureout$compare=="LADA vs T2D"), ],
            aes(x=log2FoldChange, y=minuslog10, group=Genus),
            color="#B2DF8A", shape=18,
            size=Featureout[which(Featureout$Phyla=="Firmicutes" &
                                    Featureout$compare=="LADA vs T2D"), ]
            [,ncol(Featureout)]/40+0.5, width = 0.1, height = 0.1) +
  geom_point(data=Featurein[which(Featurein$Phyla=="Euryarchaeota" &
                                   Featurein$compare=="LADA vs T2D"), ],
            aes(x=log2FoldChange, y=minuslog10, group=Genus), color="#CAB2D6", shape=16,
            size=Featurein[which(Featurein$Phyla=="Euryarchaeota" &
                                   Featurein$compare=="LADA vs T2D"), ]
            [,ncol(Featurein)]/40+0.5) +
  geom_jitter(data=Featureout[which(Featureout$Phyla=="Euryarchaeota" &
                                     Featureout$compare=="LADA vs T2D"), ],
            aes(x=log2FoldChange, y=minuslog10, group=Genus), color="#CAB2D6", shape=18,
            size=Featureout[which(Featureout$Phyla=="Euryarchaeota" &
                                    Featureout$compare=="LADA vs T2D"), ]
```

```
             [,ncol(Featureout)]/40+0.5, width = 0.1, height = 0.1) +
  geom_point(data=Featurein[which(Featurein$Phyla=="Other" &
                                  Featurein$compare=="LADA vs T2D"), ],
             aes(x=log2FoldChange, y=minuslog10, group=Genus), color="#B3B3B3", shape=16,
             size=Featurein[which(Featurein$Phyla=="Other" &
                                  Featurein$compare=="LADA vs T2D"), ]
             [,ncol(Featurein)]/40+0.5) +
  geom_jitter(data=Featureout[which(Featureout$Phyla=="Other" &
                                    Featureout$compare=="LADA vs T2D"), ],
             aes(x=log2FoldChange, y=minuslog10, group=Genus), color="#B3B3B3", shape=18,
             size=Featureout[which(Featureout$Phyla=="Other" &
                                   Featureout$compare=="LADA vs T2D"), ]
             [,ncol(Featureout)]/40+0.5, width = 0.1, height = 0.1) +
  geom_text(data=Featurein[which(Featurein$padj<padj_threshold &
                                 Featurein$compare=="LADA vs T2D"), ],
             aes(x=log2FoldChange, y=minuslog10, group=Genus, label=Genus),
            nudge_y = 0.2, size=2.5) +
  geom_text(data=Featureout[which(Featureout$padj<padj_threshold &
                                  Featureout$compare=="LADA vs T2D"), ],
             aes(x=log2FoldChange, y=minuslog10, group=Genus, label=Genus),
            nudge_x=0.6, nudge_y = -0.2, size=2.5) +
#  geom_text(data=Featureout[which(Featureout$padj<padj_threshold &
#                                  Featureout$compare=="LADA vs T1D" &
#                                  Featureout$Genus=="Rikenellaceae_RC9_gut_group"), ],
#              aes(x=log2FoldChange, y=minuslog10, group=Genus, label=Genus),
#             nudge_x=1.22, nudge_y = 0) +
  geom_hline(yintercept = -log(0.05), linetype="dashed") +
  geom_hline(yintercept = -log(BHLadaT2D), linetype="dashed", alpha=0.1) +
  geom_vline(xintercept = -1, linetype="dashed") +
  geom_vline(xintercept = 1, linetype="dashed") +
  theme_bw() +
  ggtitle("T2D") +
  xlab("Log2 fold change") +
  ylab("-log10(P)") +
  theme(panel.grid.major = element_blank(),
        panel.grid.minor = element_blank(),
        axis.title=element_text(size=22),
        legend.position="bottom",
        legend.title=element_text(size=20),
        legend.text=element_text(size=20),
        axis.text.x = element_text(angle = 45, hjust = 1, size=12),
        axis.text.y = element_text(angle = 45, hjust = 1, size=12),
        plot.title = element_text(size = 22, hjust=0.5))
#ggplotly(p2)
p2
```

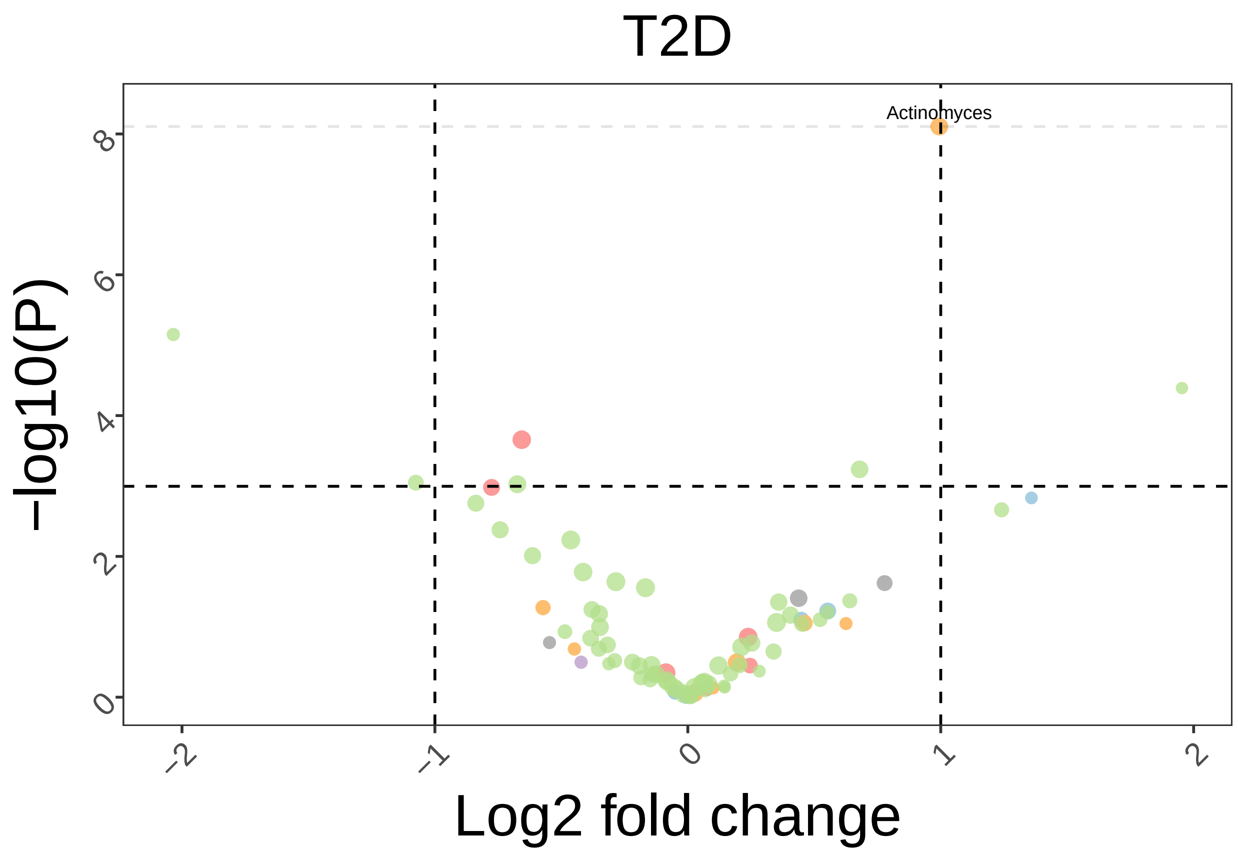


```r
Fig2List[["vulcLadaT2D"]] <- p2


p3<-ggplot() +
  geom_point(data=Featurein[which(Featurein$Phyla=="Proteobacteria" &
                                   Featurein$compare=="LADA vs Control"), ],
             aes(x=log2FoldChange, y=minuslog10, group=Genus), color="#A6CEE3", shape=16,
             size=Featurein[which(Featurein$Phyla=="Proteobacteria" &
                                   Featurein$compare=="LADA vs Control"), ]
             [,ncol(Featurein)]/40+0.5) +
  #did not add jitter to the first on purpose. Sizes are scaled from percent to the range 0.5-3
  geom_point(data=Featureout[which(Featureout$Phyla=="Proteobacteria" &
                                    Featureout$compare=="LADA vs Control"), ],
             aes(x=log2FoldChange, y=minuslog10, group=Genus), color="#A6CEE3", shape=18,
             size=Featureout[which(Featureout$Phyla=="Proteobacteria" &
                                    Featureout$compare=="LADA vs Control"), ]
             [,ncol(Featureout)]/40+0.5) +
  geom_point(data=Featurein[which(Featurein$Phyla=="Bacteroidetes" &
                                   Featurein$compare=="LADA vs Control"), ],
             aes(x=log2FoldChange, y=minuslog10, group=Genus), color="#FB9A99", shape=16,
             size=Featurein[which(Featurein$Phyla=="Bacteroidetes" &
                                   Featurein$compare=="LADA vs Control"), ]
             [,ncol(Featurein)]/40+0.5) +
  geom_jitter(data=Featureout[which(Featureout$Phyla=="Bacteroidetes" &
                                     Featureout$compare=="LADA vs Control"), ],
             aes(x=log2FoldChange, y=minuslog10, group=Genus), color="#FB9A99", shape=18,
```

```
            size=Featureout[which(Featureout$Phyla=="Bacteroidetes" &
                                   Featureout$compare=="LADA vs Control"), ]
            [,ncol(Featureout)]/40+0.5, width = 0.1, height = 0.1) +
  geom_point(data=Featurein[which(Featurein$Phyla=="Actinobacteria" &
                                  Featurein$compare=="LADA vs Control"), ],
            aes(x=log2FoldChange, y=minuslog10, group=Genus), color="#FDBF6F", shape=16,
            size=Featurein[which(Featurein$Phyla=="Actinobacteria" &
                                  Featurein$compare=="LADA vs Control"), ]
            [,ncol(Featurein)]/40+0.5) +
  geom_jitter(data=Featureout[which(Featureout$Phyla=="Actinobacteria" &
                                    Featureout$compare=="LADA vs Control"), ],
            aes(x=log2FoldChange, y=minuslog10, group=Genus), color="#FDBF6F", shape=18,
            size=Featureout[which(Featureout$Phyla=="Actinobacteria" &
                                   Featureout$compare=="LADA vs Control"), ]
            [,ncol(Featureout)]/40+0.5, width = 0.1, height = 0.1) +
  geom_point(data=Featurein[which(Featurein$Phyla=="Firmicutes" &
                                  Featurein$compare=="LADA vs Control"), ],
            aes(x=log2FoldChange, y=minuslog10, group=Genus),
            color="#B2DF8A", shape=16, alpha=0.75,
            size=Featurein[which(Featurein$Phyla=="Firmicutes" &
                                  Featurein$compare=="LADA vs Control"), ]
            [,ncol(Featurein)]/40+0.5) +
  geom_jitter(data=Featureout[which(Featureout$Phyla=="Firmicutes" &
                                    Featureout$compare=="LADA vs Control"), ],
            aes(x=log2FoldChange, y=minuslog10, group=Genus),
            color="#B2DF8A", shape=18,
            size=Featureout[which(Featureout$Phyla=="Firmicutes" &
                                   Featureout$compare=="LADA vs Control"), ]
            [,ncol(Featureout)]/40+0.5, width = 0.1, height = 0.1) +
  geom_point(data=Featurein[which(Featurein$Phyla=="Euryarchaeota" &
                                  Featurein$compare=="LADA vs Control"), ],
            aes(x=log2FoldChange, y=minuslog10, group=Genus), color="#CAB2D6", shape=16,
            size=Featurein[which(Featurein$Phyla=="Euryarchaeota" &
                                  Featurein$compare=="LADA vs Control"), ]
            [,ncol(Featurein)]/40+0.5) +
  geom_jitter(data=Featureout[which(Featureout$Phyla=="Euryarchaeota" &
                                    Featureout$compare=="LADA vs Control"), ],
            aes(x=log2FoldChange, y=minuslog10, group=Genus), color="#CAB2D6", shape=18,
            size=Featureout[which(Featureout$Phyla=="Euryarchaeota" &
                                   Featureout$compare=="LADA vs Control"), ]
            [,ncol(Featureout)]/40+0.5, width = 0.1, height = 0.1) +
  geom_point(data=Featurein[which(Featurein$Phyla=="Other" &
                                  Featurein$compare=="LADA vs Control"), ],
            aes(x=log2FoldChange, y=minuslog10, group=Genus), color="#B3B3B3", shape=16,
            size=Featurein[which(Featurein$Phyla=="Other" &
                                  Featurein$compare=="LADA vs Control"), ]
            [,ncol(Featurein)]/40+0.5) +
  geom_jitter(data=Featureout[which(Featureout$Phyla=="Other" &
                                    Featureout$compare=="LADA vs Control"), ],
            aes(x=log2FoldChange, y=minuslog10, group=Genus), color="#B3B3B3", shape=18,
            size=Featureout[which(Featureout$Phyla=="Other" &
                                   Featureout$compare=="LADA vs Control"), ]
            [,ncol(Featureout)]/40+0.5, width = 0.1, height = 0.1) +
```

```
  geom_text(data=Featurein[which(Featurein$padj<padj_threshold &
                                  Featurein$compare=="LADA vs Control"), ],
            aes(x=log2FoldChange, y=minuslog10, group=Genus, label=Genus),
           nudge_y = 0.2, size=2.5) +
  geom_text(data=Featureout[which(Featureout$padj<padj_threshold &
                                   Featureout$compare=="LADA vs Control"), ],
            aes(x=log2FoldChange, y=minuslog10, group=Genus, label=Genus),
           nudge_x=0.6, nudge_y = -0.2, size=2.5) +
#  geom_text(data=Featureout[which(Featureout$padj<padj_threshold &
#                                   Featureout$compare=="LADA vs T1D" &
#                                   Featureout$Genus=="Rikenellaceae_RC9_gut_group"), ],
#             aes(x=log2FoldChange, y=minuslog10, group=Genus, label=Genus),
#            nudge_x=1.22, nudge_y = 0) +
  geom_hline(yintercept = -log(0.05), linetype="dashed") +
  geom_hline(yintercept = -log(BHLadaCon), linetype="dashed", alpha=0.1) +
  geom_vline(xintercept = -1, linetype="dashed") +
  geom_vline(xintercept = 1, linetype="dashed") +
  theme_bw() +
  ggtitle("Controls") +
  xlab("Log2 fold change") +
  ylab("-log10(P)") +
  theme(panel.grid.major = element_blank(),
        panel.grid.minor = element_blank(),
        axis.title=element_text(size=22),
        legend.position="bottom",
        legend.title=element_text(size=20),
        legend.text=element_text(size=20),
        axis.text.x = element_text(angle = 45, hjust = 1, size=12),
        axis.text.y = element_text(angle = 45, hjust = 1, size=12),
        plot.title = element_text(size = 22, hjust=0.5))
#ggplotly(p3)
p3
```

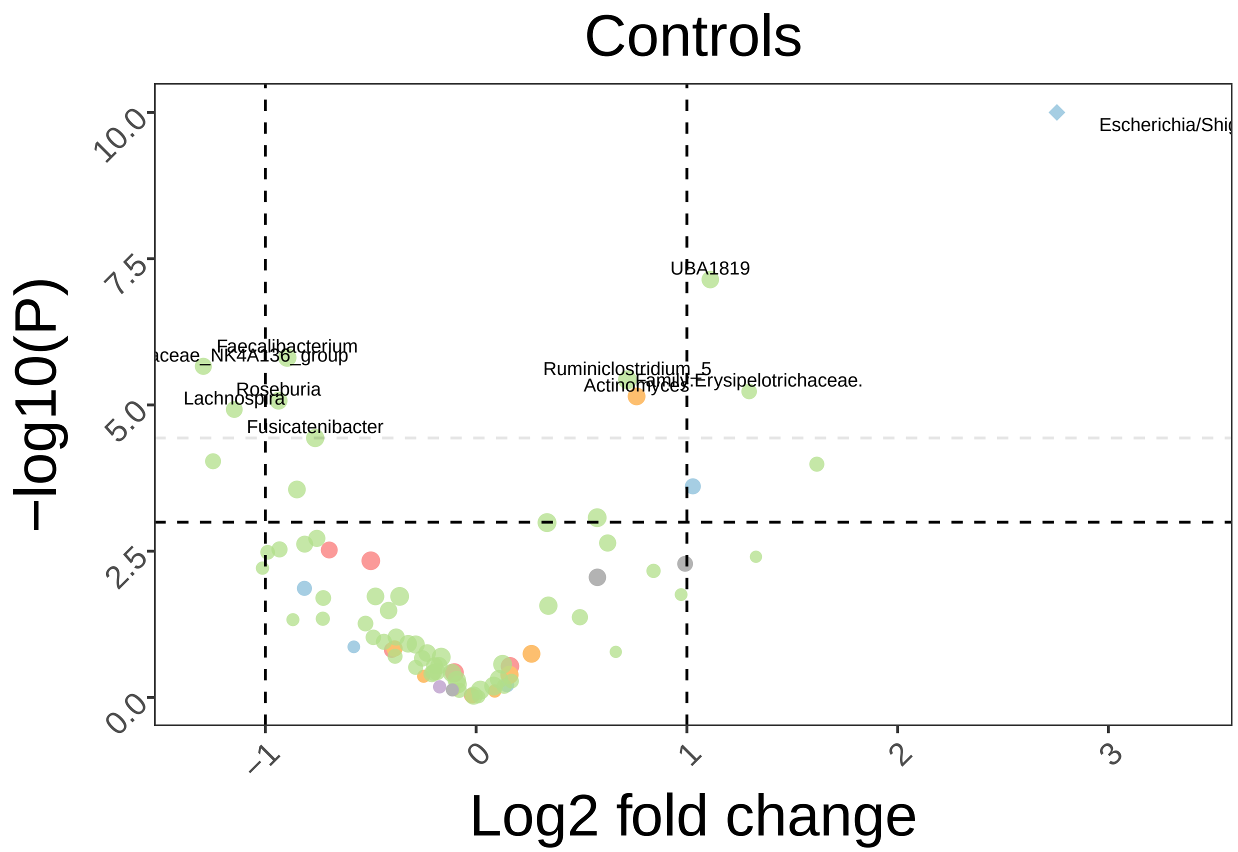


```
Fig2List[["vulcLadaControl"]] <- p3

lay <- rbind(c(1,2,3))
pdf(paste("MicroLADA_Vulcano.pdf", sep=""), width=15, height=5)
grid.arrange(p1,p2,p3,layout_matrix = lay)
dev.off()
```

pdf 2

```
##Boxplot with stats intertwined
#Stats have to be in the end of the chunk don't ask me why
#Have to use the same naming in res_stat to output tables
res_stat$Genus<-str_replace_all(res_stat$Genus, " ","_")
res_stat$Genus<-str_replace_all(res_stat$Genus, "/","_")
res_stat$Genus<-gsub("\\[|\\]", "", res_stat$Genus)
res_stat$Genus<-str_replace_all(res_stat$Genus, ":","_")
res_stat$Genus<-str_replace_all(res_stat$Genus, "-","_")

#Order Diagnosis
Plotting2$Diagnosis<-ordered(Plotting2$Diagnosis,
                             levels=c("Control", "T1D", "LADA", "T2D"))

for (i in SelOrgs) {
Boxplot <-
ggplot(Plotting2, aes_string(x="Diagnosis", y=i, group="Diagnosis")) +
```

```
    geom_boxplot() +
#    geom_boxplot(aes(fill=Diagnosis, trim=FALSE)) +
#    geom_jitter() +
    geom_jitter(aes(color=Diagnosis), size=2) +
    stat_summary(fun.data="mean_sdl",
                 mult=1, #mean plus minus a constant (mult=1) times the st.dev
                 geom="pointrange",
                 width=0.2 ) +
    #stat_summary(fun.y = mean, geom = "point") +
    #facet_grid(. ~ Metformin, scales="fixed") + #Scales can also be "free"
    ggtitle(i) +
    #xlab("Diagnosis") +
    ylab("Cells pr. g feces") +
    scale_fill_manual(values=c(Control = "#666666", T1D = "#1F78B4",
                                T2D = "#33A02C", LADA = "#E31A1C")) +
    scale_color_manual(values=c(Control = "#666666", T1D = "#1F78B4",
                                T2D = "#33A02C", LADA = "#E31A1C")) +
    theme_bw() +
    theme(legend.position="none",
          title =element_text(size=18, face='bold'),
          panel.grid.major = element_blank(),
          panel.grid.minor = element_blank(),
          axis.title=element_text(size=16),
          axis.title.x = element_blank(),
          axis.text.x = element_text(angle = 45, hjust = 1, size=14),
          axis.text.y = element_text(angle = 45, hjust = 1, size=12))
Fig2List[[i]] <- Boxplot
#print(kable(stat_sig[which(stat_sig$gene==i), c(5,6,7,10)]))
cat("\n")
tabling<-res_stat[which(res_stat$Genus==i), c(5,6,7,10)]
print(kable(tabling))
#print(tabling)
cat("\n")
print(Boxplot)
}
```

	pvalue	padj	Genus	compare
407	0.0000000	0.0000032	Escherichia_Shigella	LADA vs T1D
243	0.0000002	0.0000155	Escherichia_Shigella	LADA vs Control
325	0.0000035	0.0001448	Escherichia_Shigella	T1D vs T2D
161	0.0000384	0.0031474	Escherichia_Shigella	Control vs T2D
79	0.1577557	0.6671050	Escherichia_Shigella	Control vs T1D
489	0.2938808	0.8784397	Escherichia_Shigella	LADA vs T2D

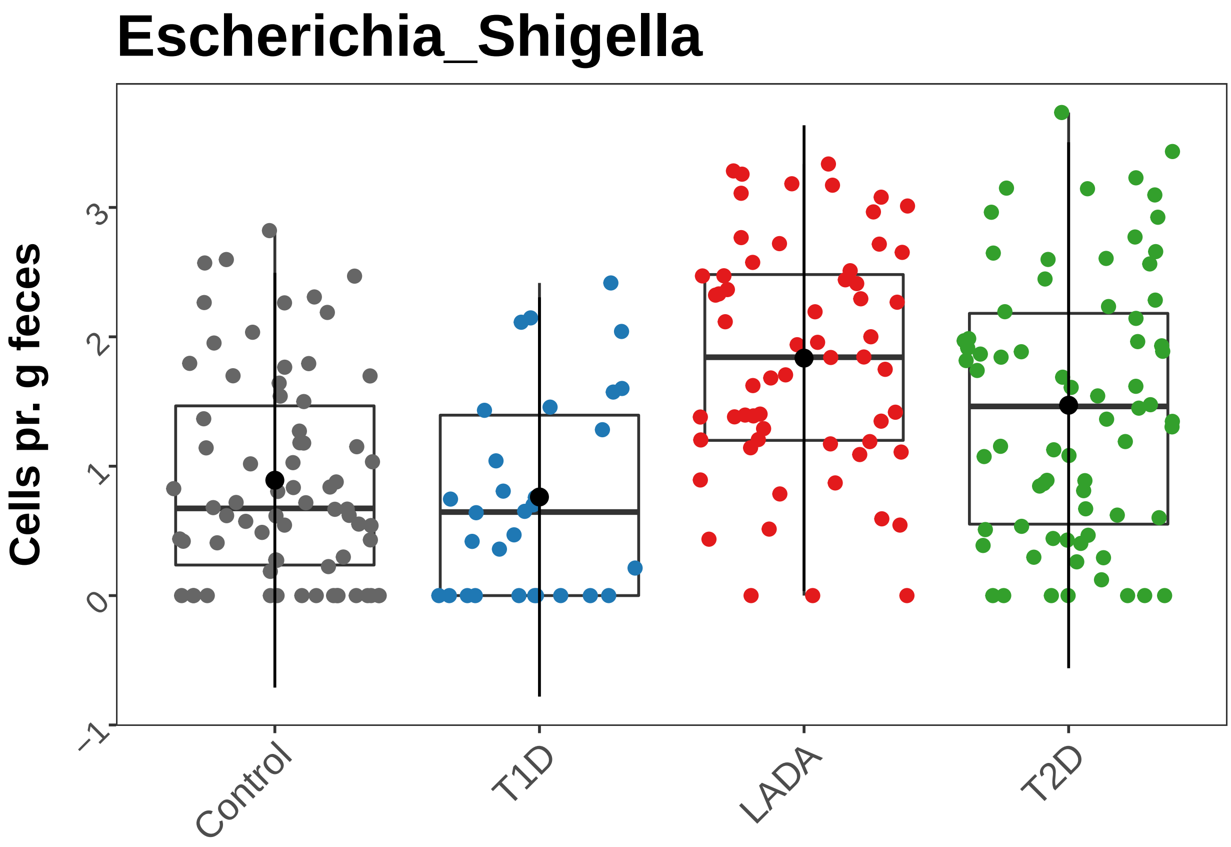

	pvalue	padj	Genus	compare
319	0.0000014	0.0001112	Veillonella	T1D vs T2D
401	0.0072926	0.1053626	Veillonella	LADA vs T1D
73	0.0056782	0.1164036	Veillonella	Control vs T1D
155	0.0062284	0.1282578	Veillonella	Control vs T2D
483	0.0057891	0.2373534	Veillonella	LADA vs T2D
237	0.9868731	0.9868731	Veillonella	LADA vs Control

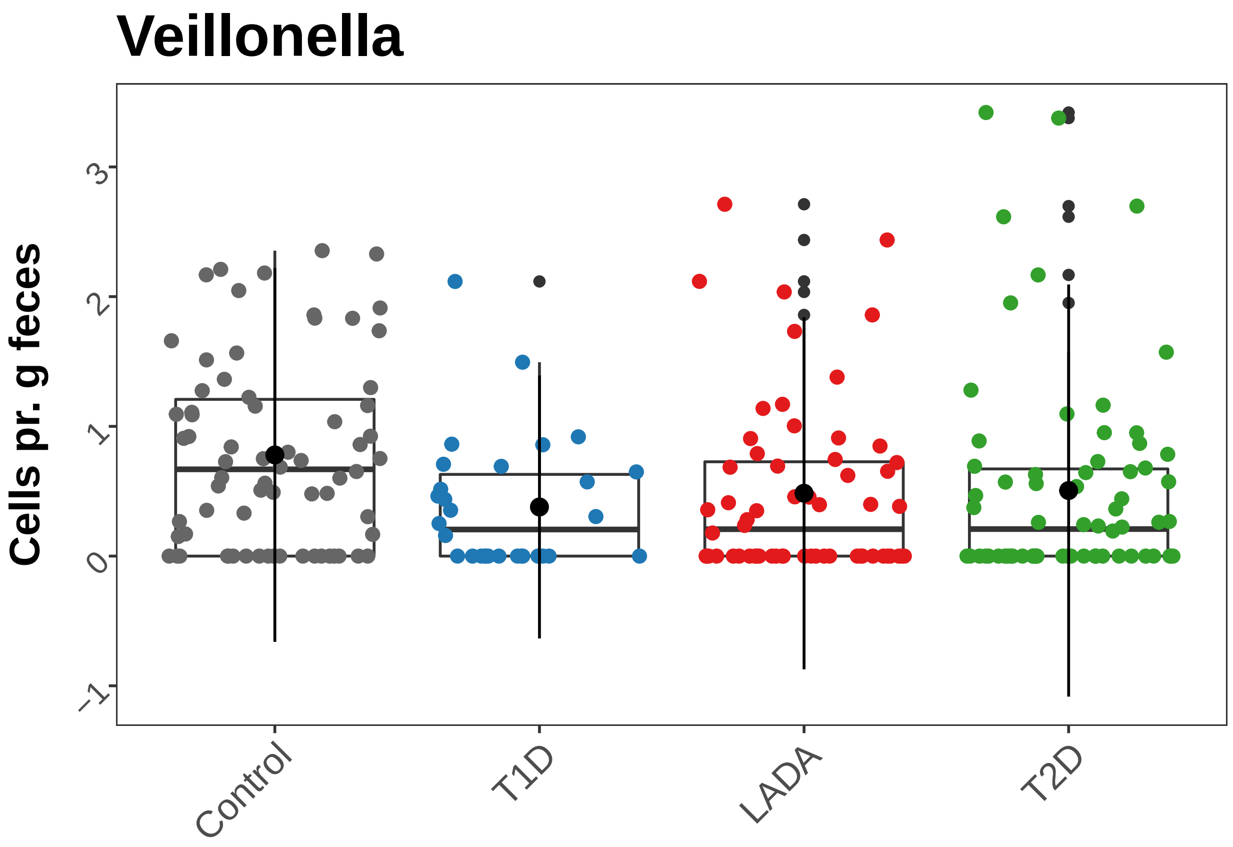

	pvalue	padj	Genus	compare
289	0.0000090	0.0002473	Butyricicoccus	T1D vs T2D
371	0.0005825	0.0238817	Butyricicoccus	LADA vs T1D
43	0.0019827	0.0905858	Butyricicoccus	Control vs T1D
125	0.0649629	0.4097658	Butyricicoccus	Control vs T2D
207	0.5675075	0.8442104	Butyricicoccus	LADA vs Control
453	0.1938785	0.8784397	Butyricicoccus	LADA vs T2D

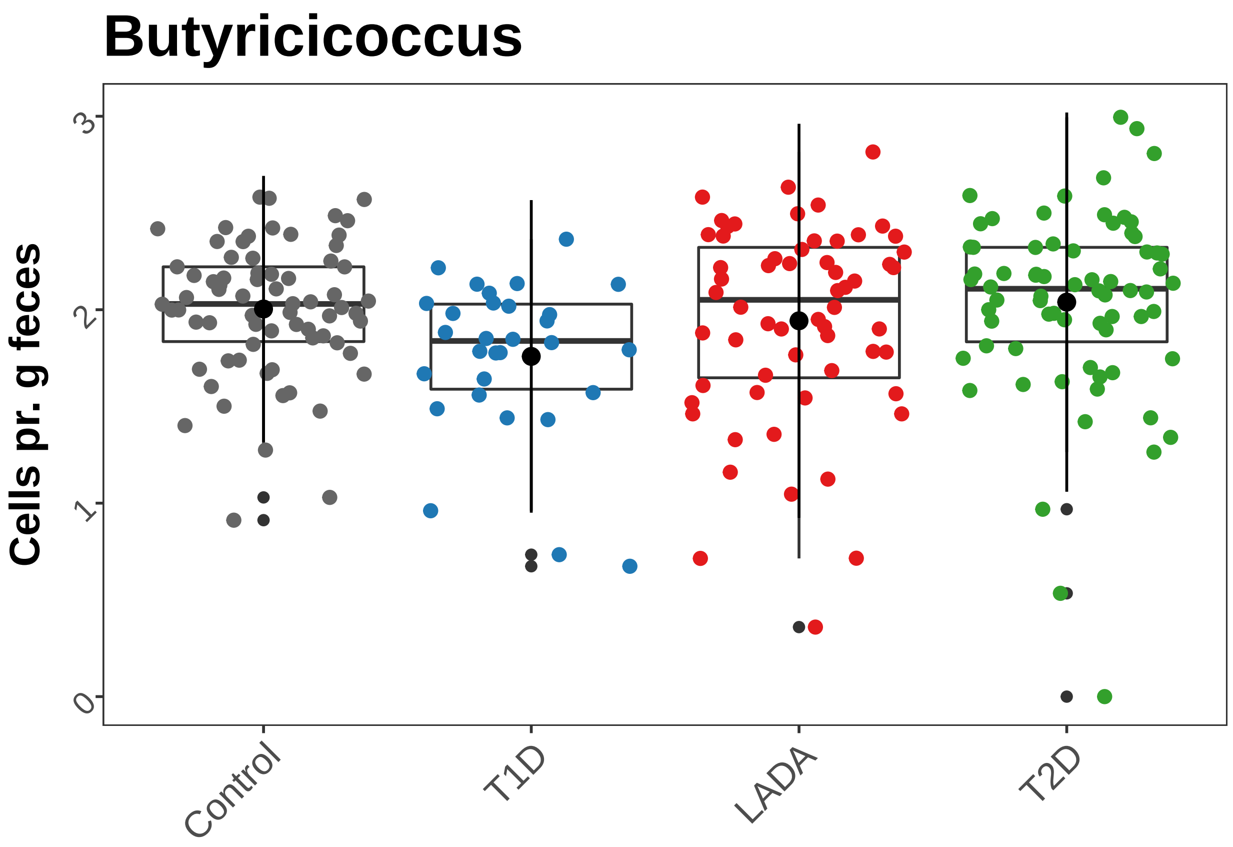

	pvalue	padj	Genus	compare
261	0.0000360	0.0007384	Parabacteroides	T1D vs T2D
97	0.0062565	0.1282578	Parabacteroides	Control vs T2D
343	0.0166021	0.1701718	Parabacteroides	LADA vs T1D
15	0.0418953	0.4294270	Parabacteroides	Control vs T1D
425	0.0257991	0.5204546	Parabacteroides	LADA vs T2D
179	0.5868292	0.8442104	Parabacteroides	LADA vs Control

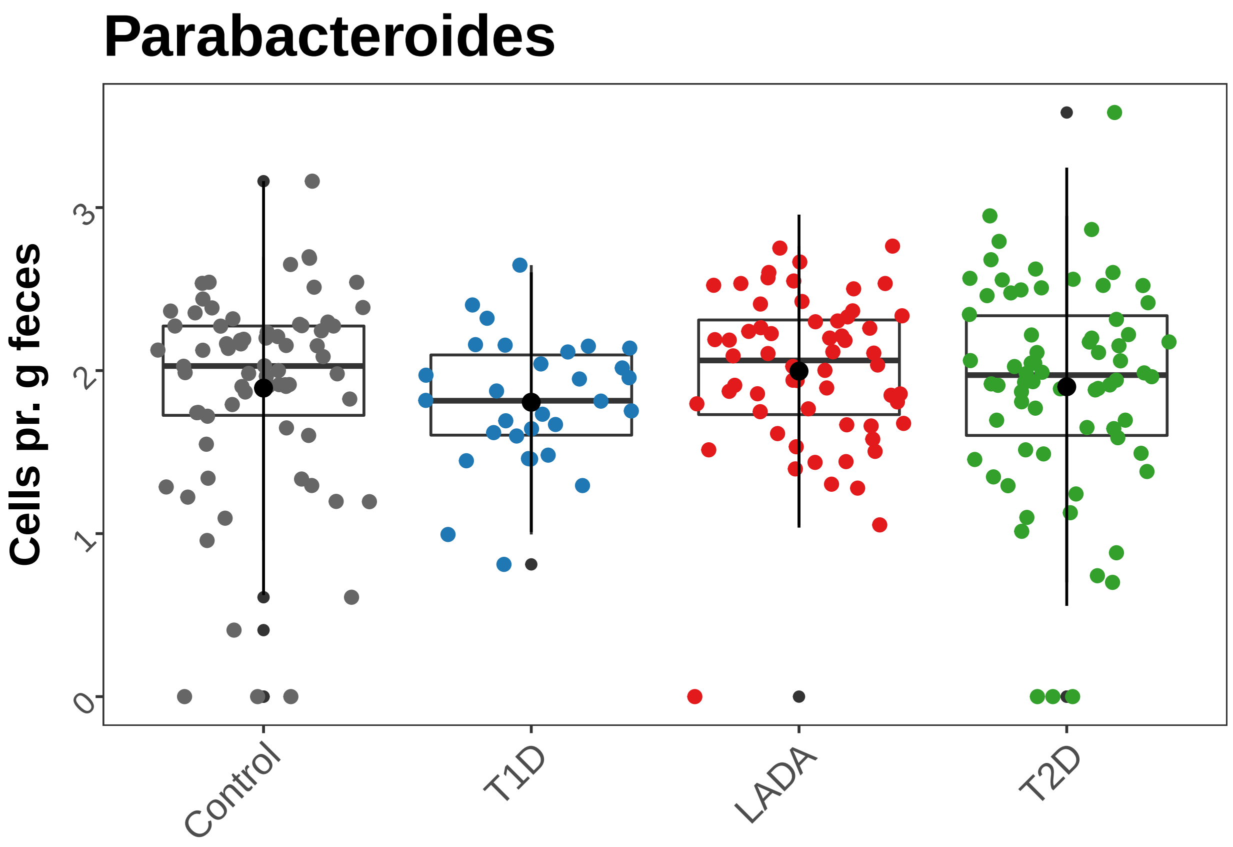

	pvalue	padj	Genus	compare
277	0.0002349	0.0038521	Lachnoclostridium	T1D vs T2D
113	0.0003741	0.0153388	Lachnoclostridium	Control vs T2D
359	0.0151897	0.1701718	Lachnoclostridium	LADA vs T1D
195	0.0462996	0.2531043	Lachnoclostridium	LADA vs Control
441	0.1071864	0.6760990	Lachnoclostridium	LADA vs T2D
31	0.3802075	0.8204478	Lachnoclostridium	Control vs T1D

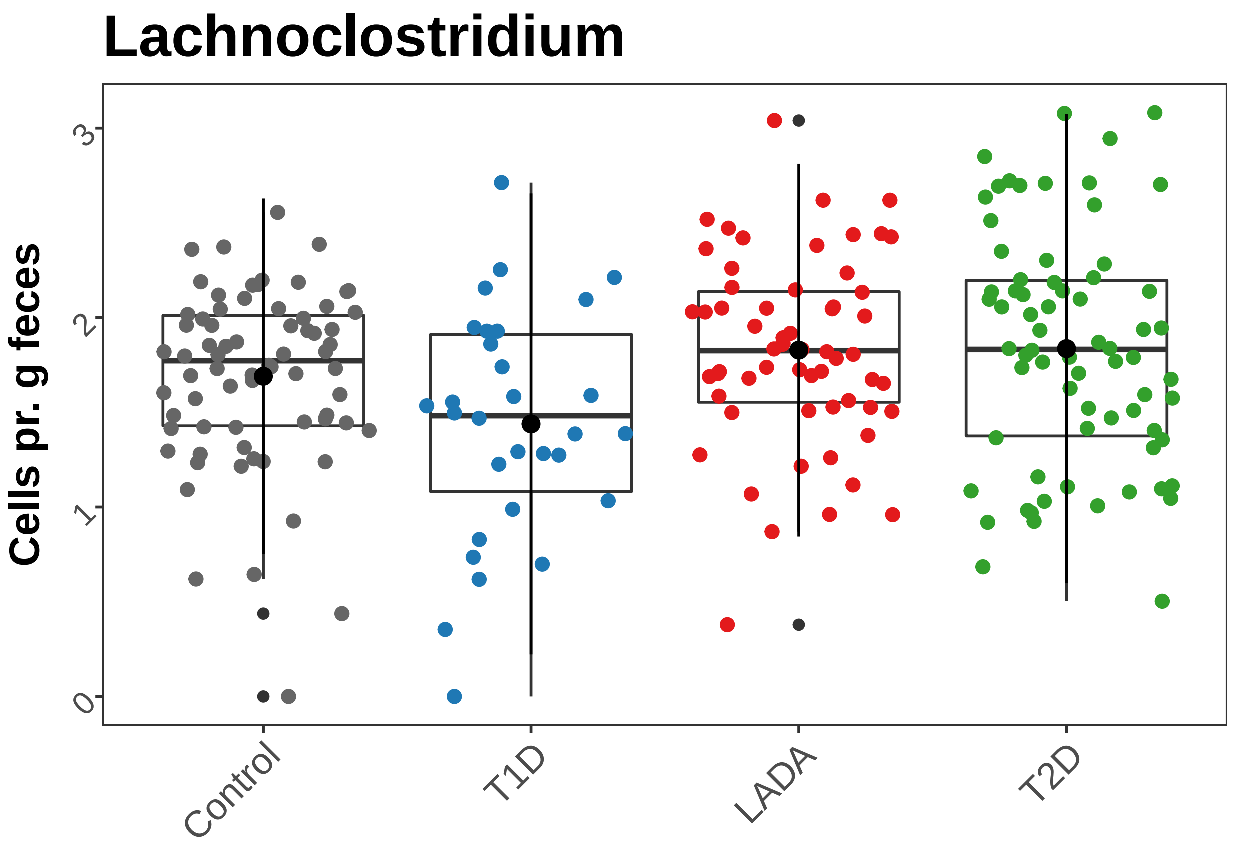

	pvalue	padj	Genus	compare
412	0.0003019	0.0247557	Actinomyces	LADA vs T2D
166	0.0058207	0.0641923	Actinomyces	LADA vs Control
248	0.0113021	0.1356968	Actinomyces	T1D vs T2D
2	0.0516306	0.4704122	Actinomyces	Control vs T1D
84	0.4052829	0.7912666	Actinomyces	Control vs T2D
330	0.7551490	0.9048022	Actinomyces	LADA vs T1D

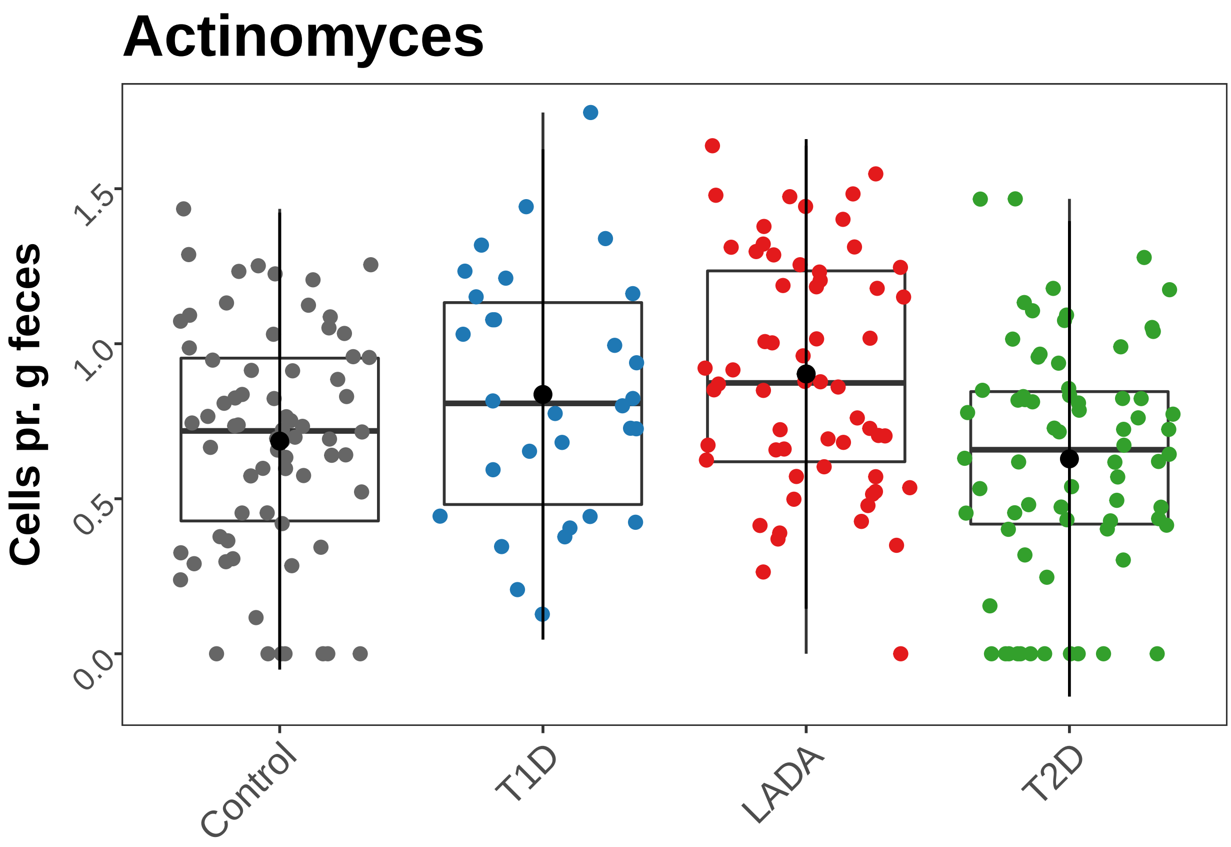

	pvalue	padj	Genus	compare
227	0.0007900	0.0323904	UBA1819	LADA vs Control
473	0.0393120	0.5204546	UBA1819	LADA vs T2D
145	0.1972930	0.6220744	UBA1819	Control vs T2D
391	0.3196032	0.6366683	UBA1819	LADA vs T1D
63	0.0839280	0.6421130	UBA1819	Control vs T1D
309	0.5286115	0.7740383	UBA1819	T1D vs T2D

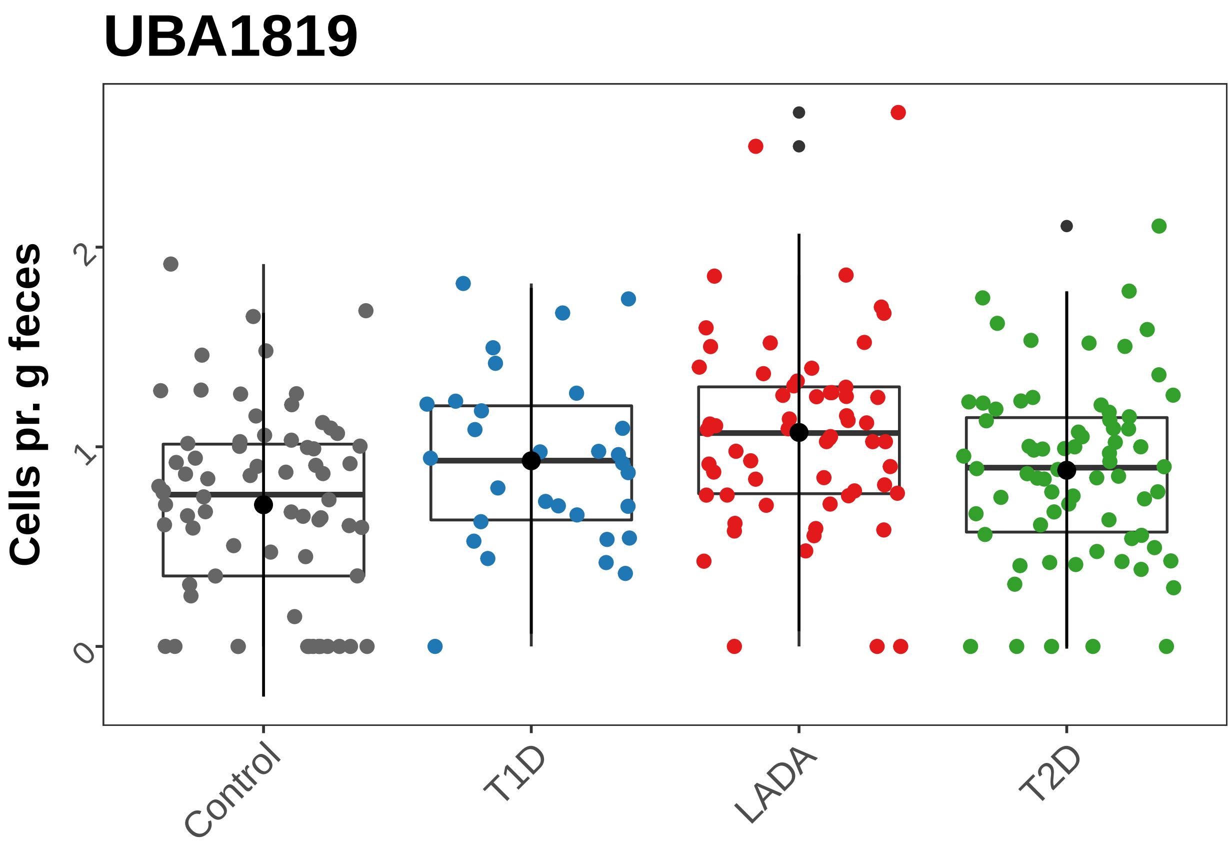

	pvalue	padj	Genus	compare
208	0.0029970	0.0641923	Faecalibacterium	LADA vs Control
44	0.0036185	0.0989047	Faecalibacterium	Control vs T1D
290	0.1253968	0.3685716	Faecalibacterium	T1D vs T2D
126	0.1141709	0.4749576	Faecalibacterium	Control vs T2D
372	0.6490728	0.8323675	Faecalibacterium	LADA vs T1D
454	0.1690955	0.8784397	Faecalibacterium	LADA vs T2D

Faecalibacterium

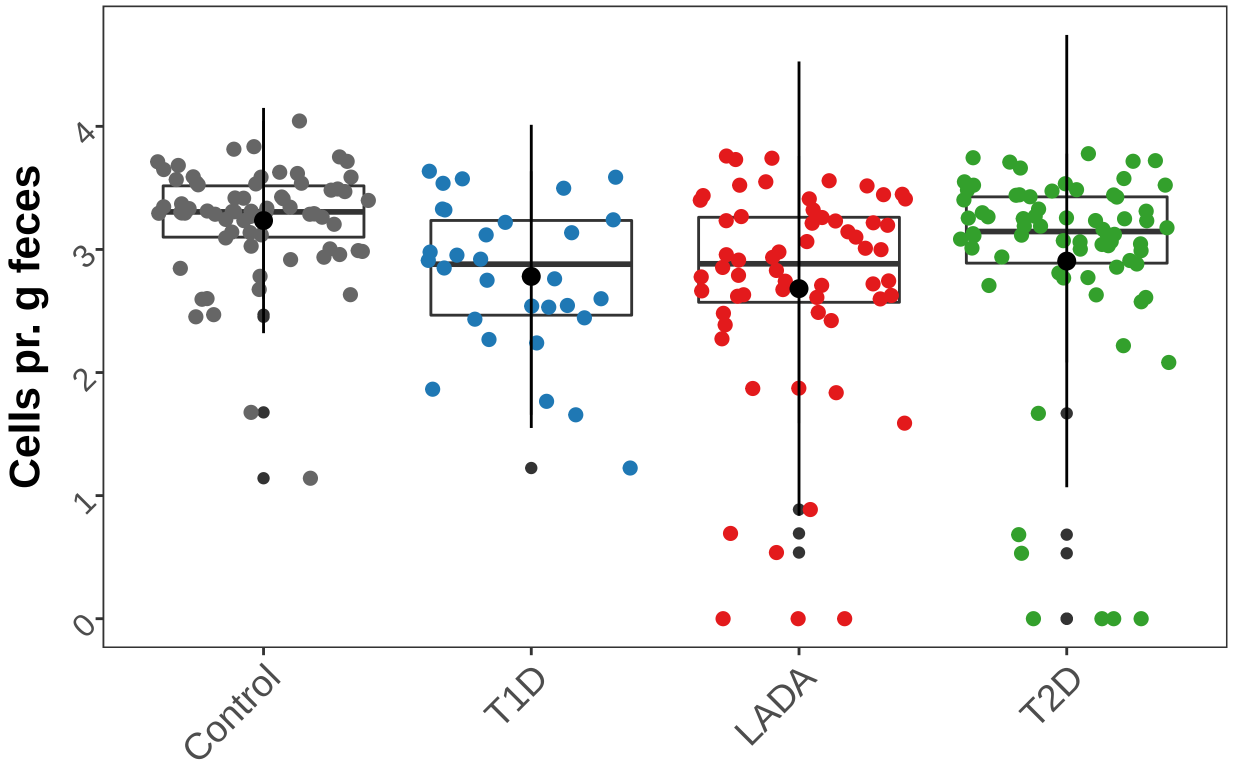

	pvalue	padj	Genus	compare
199	0.0034815	0.0641923	Lachnospiraceae_NK4A136_group	LADA vs Control
363	0.1118797	0.4631237	Lachnospiraceae_NK4A136_group	LADA vs T1D
117	0.2177415	0.6220744	Lachnospiraceae_NK4A136_group	Control vs T2D
445	0.0927767	0.6339739	Lachnospiraceae_NK4A136_group	LADA vs T2D
35	0.4509587	0.8217470	Lachnospiraceae_NK4A136_group	Control vs T1D
281	0.7953727	0.9280418	Lachnospiraceae_NK4A136_group	T1D vs T2D

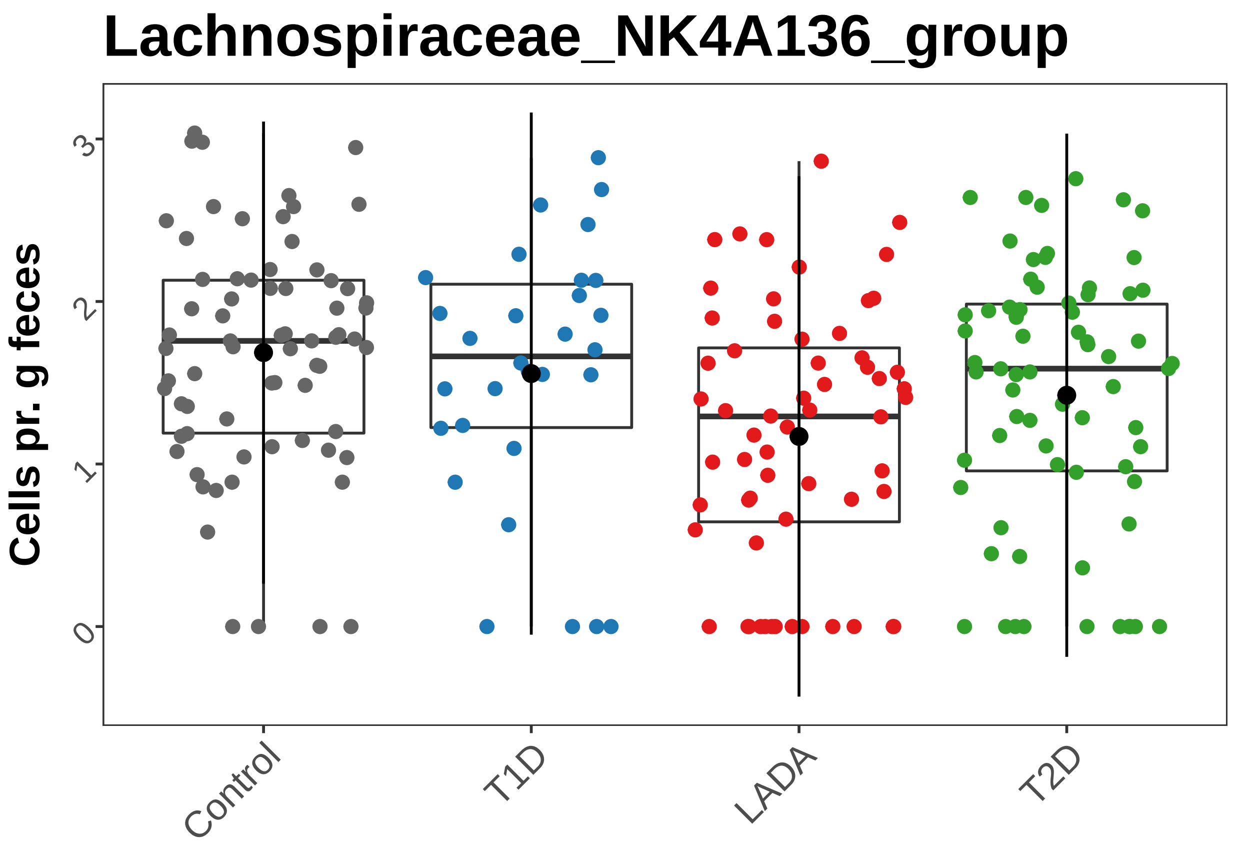

	pvalue	padj	Genus	compare
213	0.0043858	0.0641923	Ruminiclostridium_5	LADA vs Control
131	0.0094080	0.1285754	Ruminiclostridium_5	Control vs T2D
377	0.3727788	0.6366683	Ruminiclostridium_5	LADA vs T1D
49	0.1562554	0.6671050	Ruminiclostridium_5	Control vs T1D
295	0.4775574	0.7388625	Ruminiclostridium_5	T1D vs T2D
459	0.8232229	0.9803916	Ruminiclostridium_5	LADA vs T2D

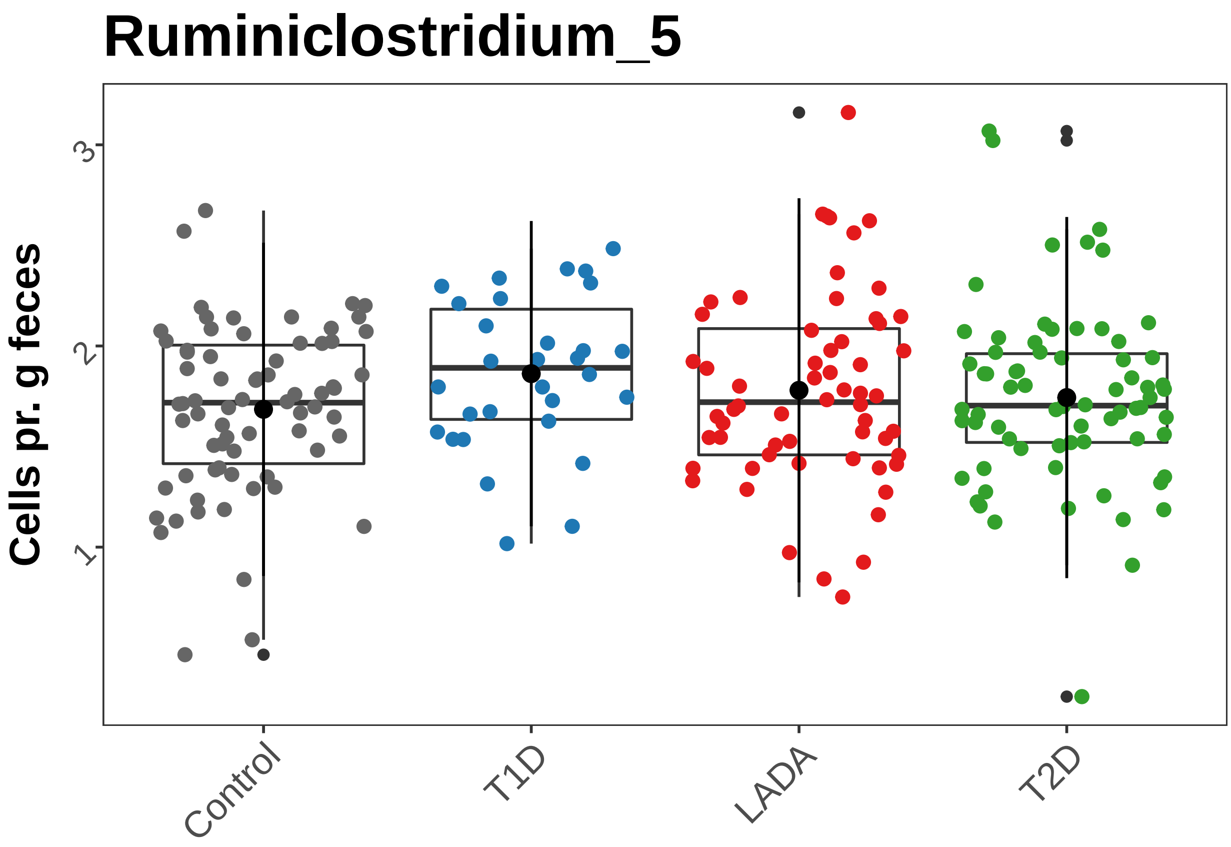

	pvalue	padj	Genus	compare
235	0.0053507	0.0641923	Family.Erysipelotrichaceae.	LADA vs Control
153	0.0166478	0.1650890	Family.Erysipelotrichaceae.	Control vs T2D
71	0.0254208	0.3474170	Family.Erysipelotrichaceae.	Control vs T1D
317	0.8148660	0.9280418	Family.Erysipelotrichaceae.	T1D vs T2D
481	0.7149626	0.9803916	Family.Erysipelotrichaceae.	LADA vs T2D
399	0.9576265	0.9815672	Family.Erysipelotrichaceae.	LADA vs T1D

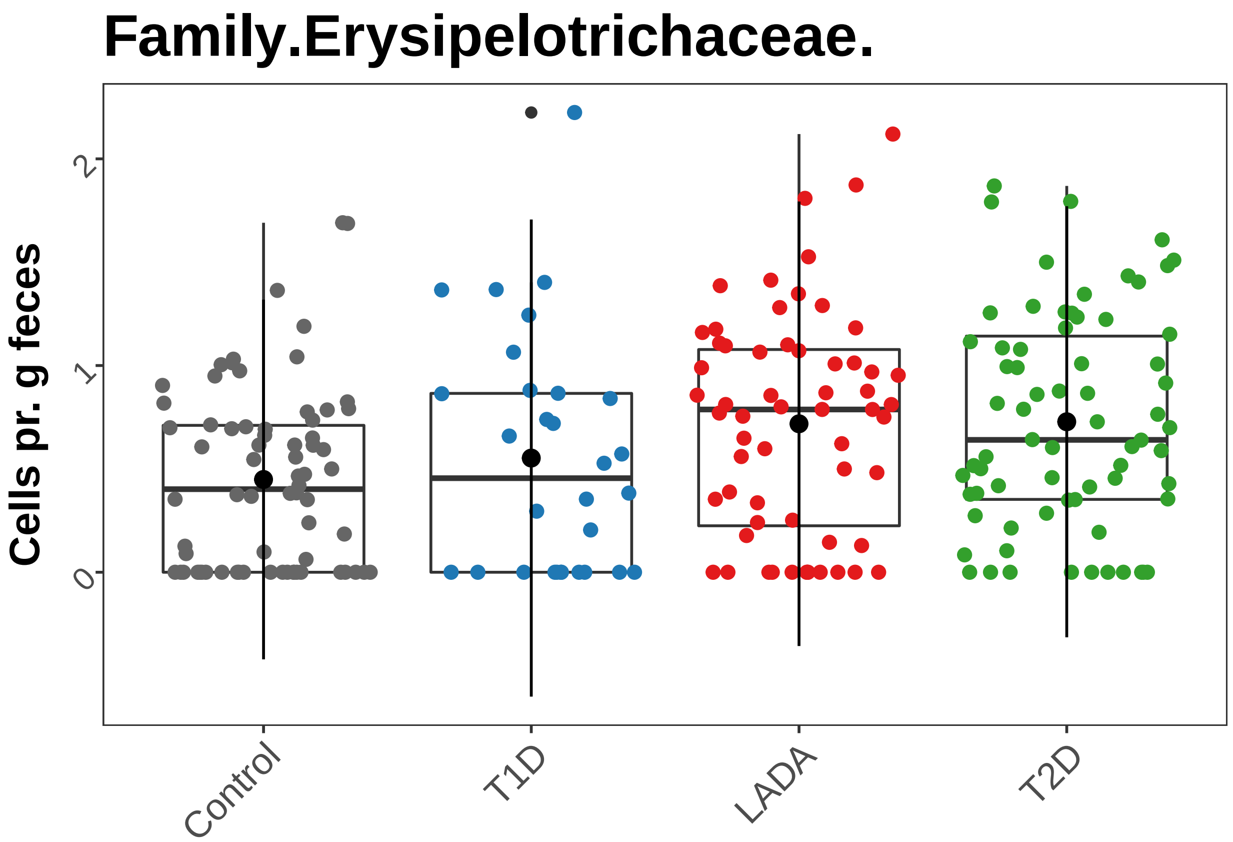

	pvalue	padj	Genus	compare
203	0.0062627	0.0641923	Roseburia	LADA vs Control
39	0.0022094	0.0905858	Roseburia	Control vs T1D
285	0.0197282	0.1797459	Roseburia	T1D vs T2D
449	0.0485321	0.5204546	Roseburia	LADA vs T2D
367	0.4314917	0.6760736	Roseburia	LADA vs T1D
121	0.4477485	0.7962182	Roseburia	Control vs T2D

Roseburia

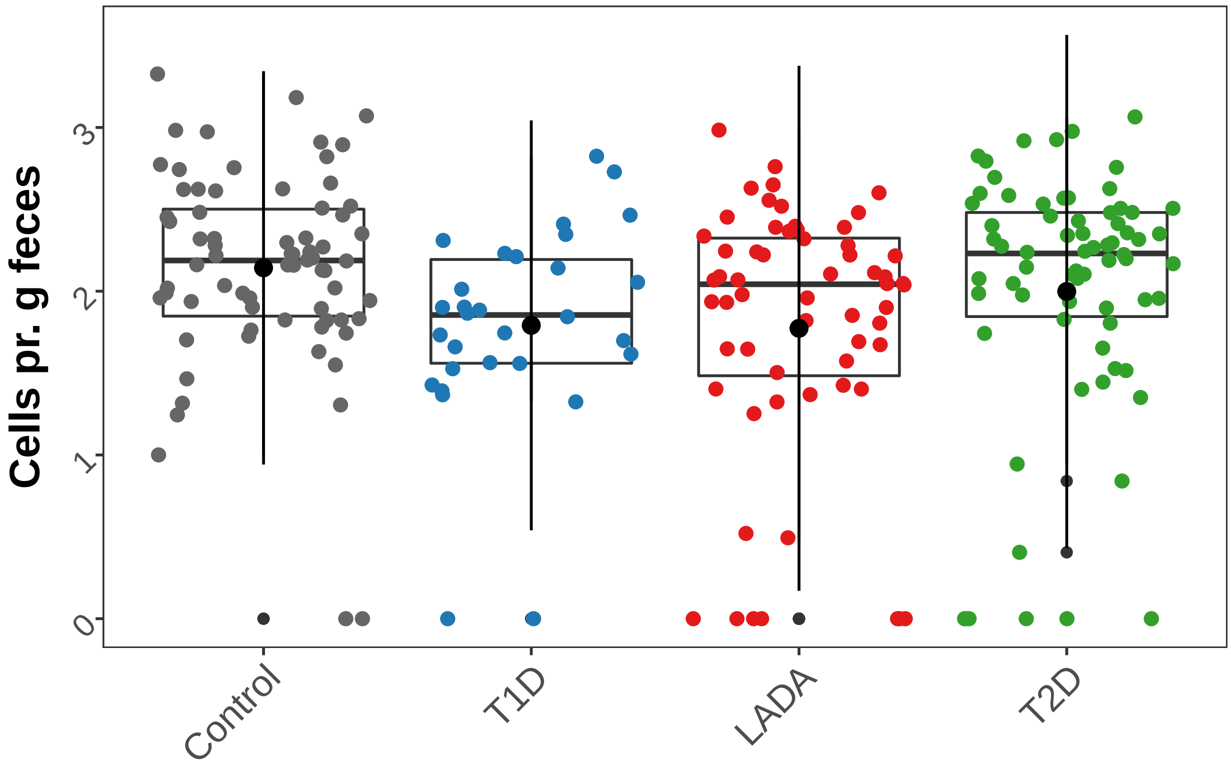

	pvalue	padj	Genus	compare
196	0.0072854	0.0663784	Lachnospira	LADA vs Control
360	0.0273593	0.2492734	Lachnospira	LADA vs T1D
114	0.0317613	0.2604427	Lachnospira	Control vs T2D
278	0.0735948	0.3650550	Lachnospira	T1D vs T2D
32	0.9361923	0.9615651	Lachnospira	Control vs T1D
442	0.6075759	0.9724127	Lachnospira	LADA vs T2D

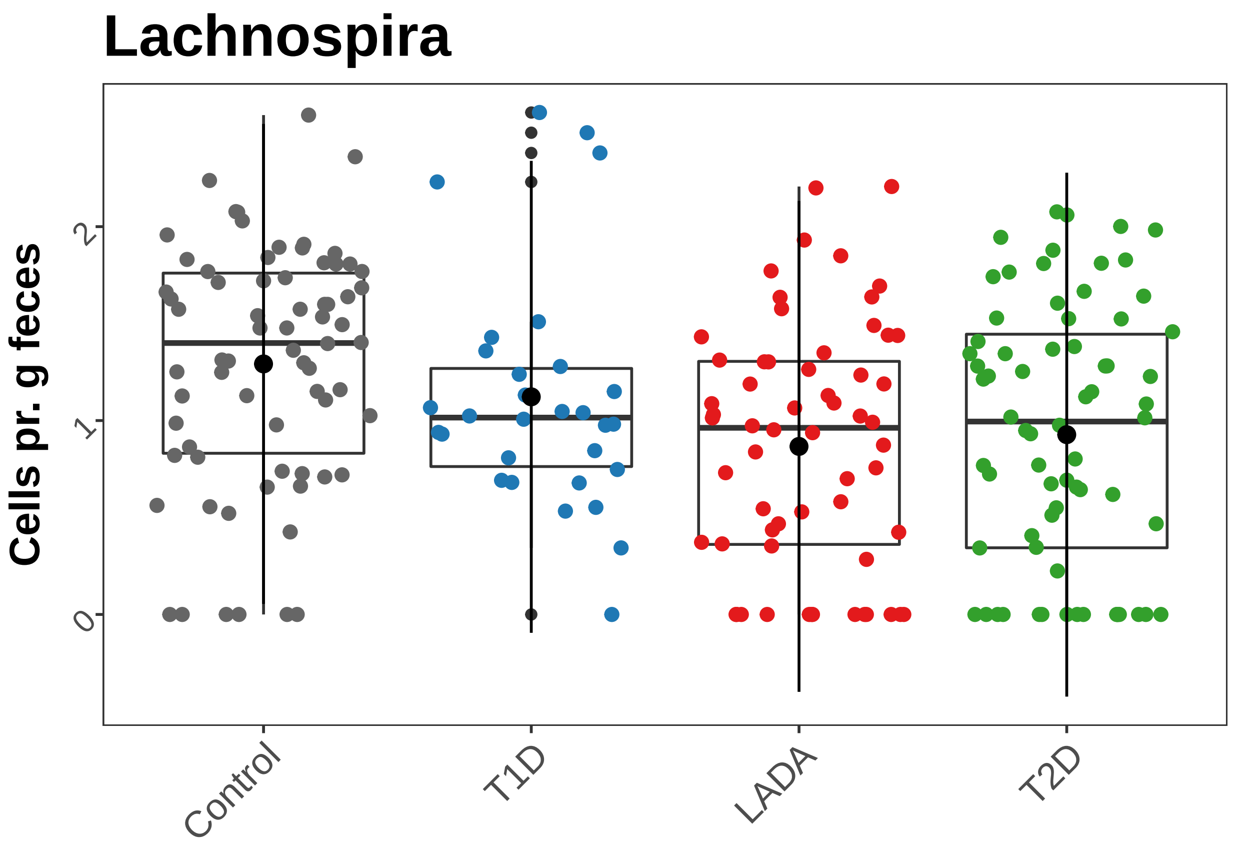

	pvalue	padj	Genus	compare
344	0.0025974	0.0676607	Lactobacillus	LADA vs T1D
180	0.0185493	0.1267534	Lactobacillus	LADA vs Control
262	0.1166548	0.3685716	Lactobacillus	T1D vs T2D
426	0.0698171	0.5204546	Lactobacillus	LADA vs T2D
16	0.2359274	0.6671050	Lactobacillus	Control vs T1D
98	0.5881729	0.7962182	Lactobacillus	Control vs T2D

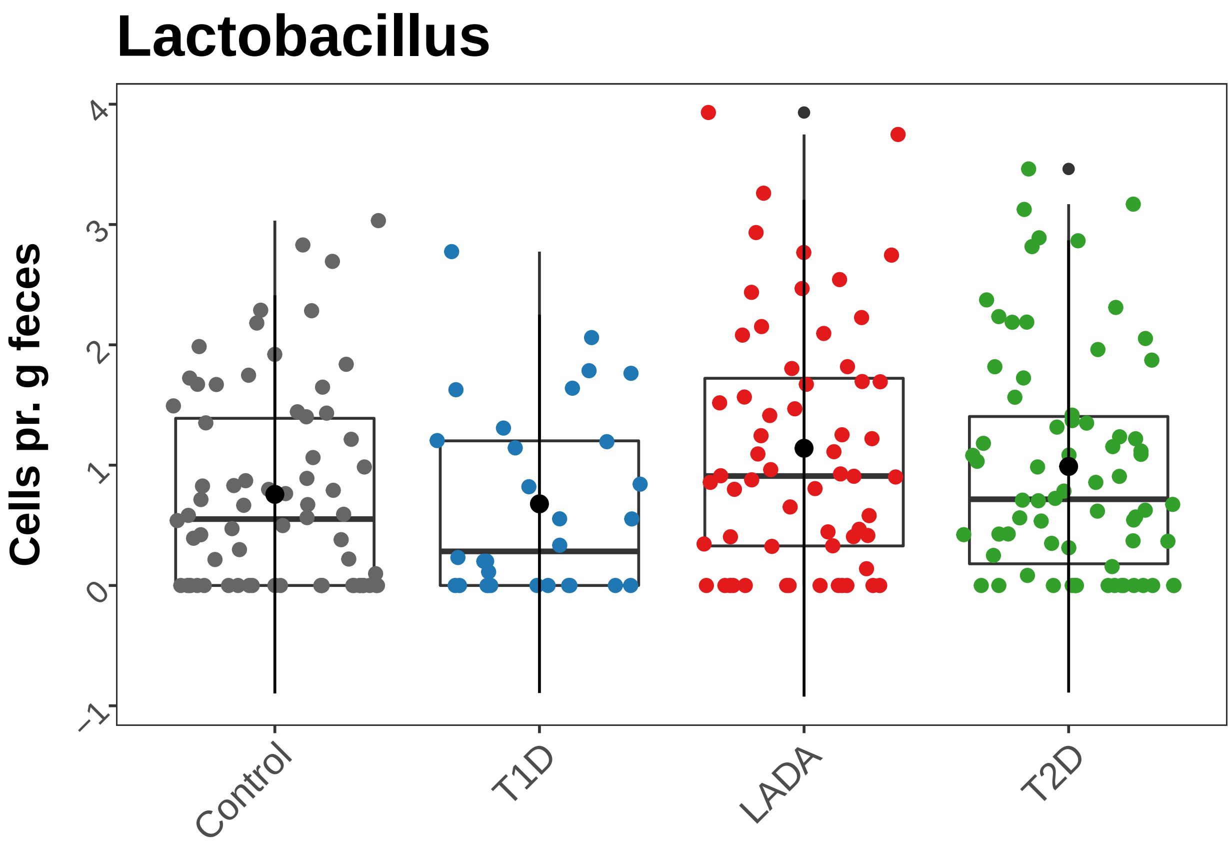

	pvalue	padj	Genus	compare
346	0.0033005	0.0676607	Christensenellaceae_R.7_group	LADA vs T1D
182	0.0285825	0.1674119	Christensenellaceae_R.7_group	LADA vs Control
264	0.0267350	0.2192269	Christensenellaceae_R.7_group	T1D vs T2D
100	0.2002863	0.6220744	Christensenellaceae_R.7_group	Control vs T2D
18	0.2132451	0.6671050	Christensenellaceae_R.7_group	Control vs T1D
428	0.3697127	0.8916601	Christensenellaceae_R.7_group	LADA vs T2D

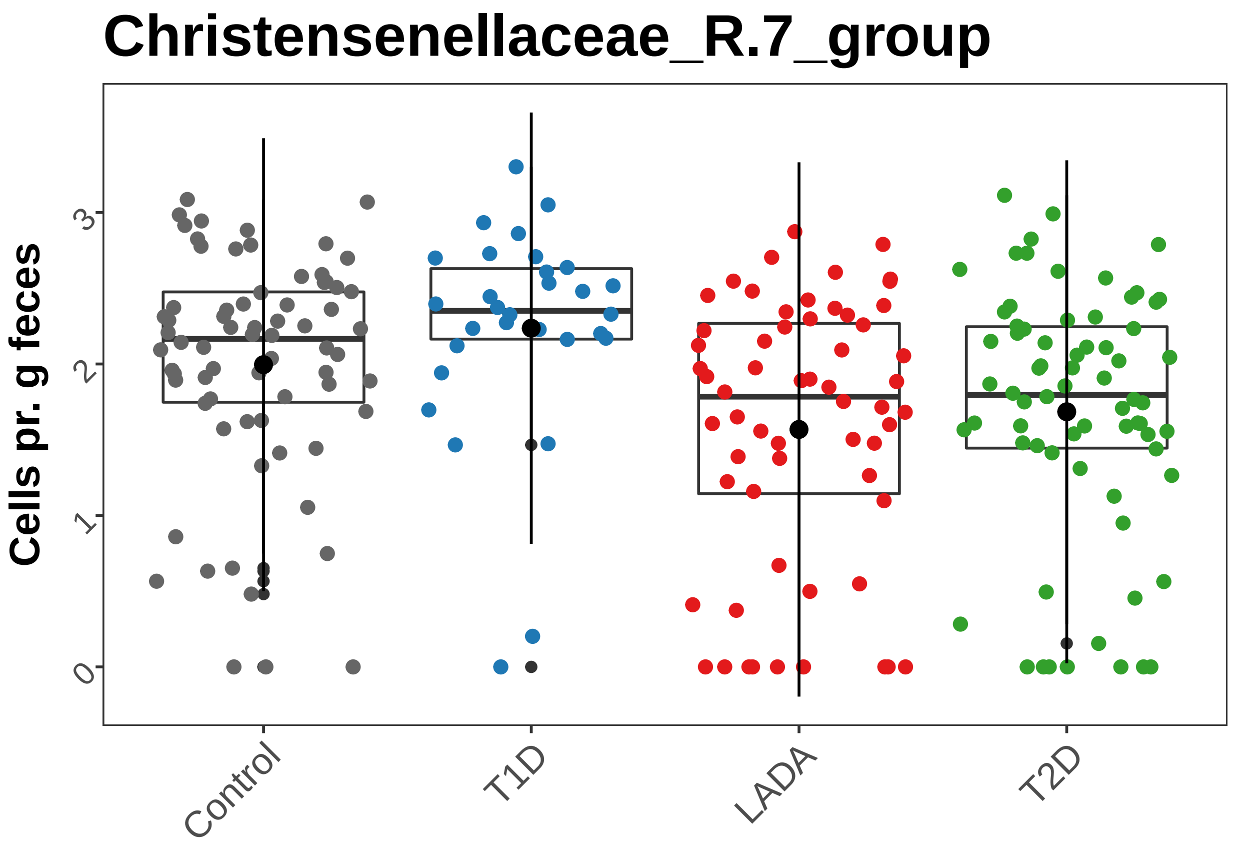

	pvalue	padj	Genus	compare
194	0.0118524	0.0971897	Fusicatenibacter	LADA vs Control
112	0.0433784	0.3233661	Fusicatenibacter	Control vs T2D
30	0.0939678	0.6421130	Fusicatenibacter	Control vs T1D
358	0.7046549	0.8889492	Fusicatenibacter	LADA vs T1D
440	0.6346494	0.9724127	Fusicatenibacter	LADA vs T2D
276	0.9972992	0.9972992	Fusicatenibacter	T1D vs T2D

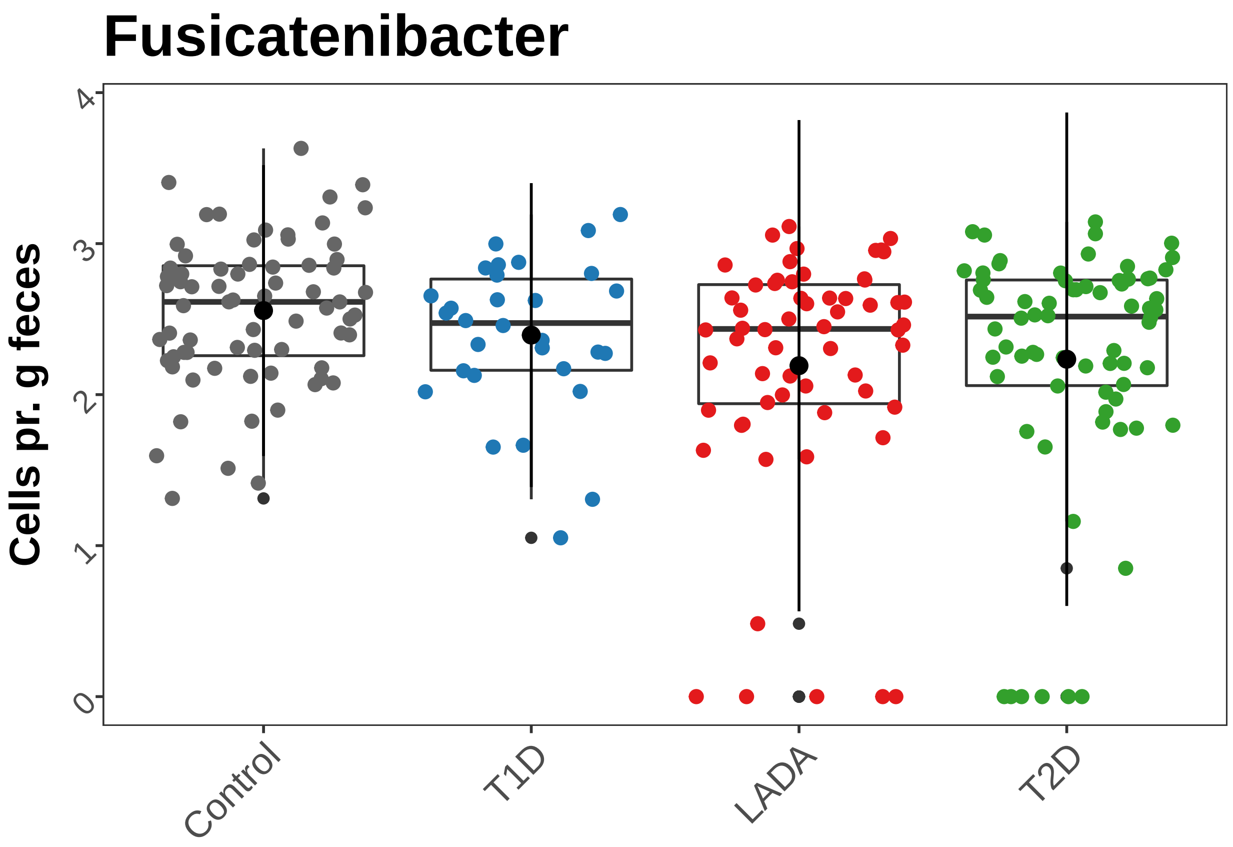

	pvalue	padj	Genus	compare
187	0.1777630	0.4541213	Anaerostipes	LADA vs Control
105	0.1158433	0.4749576	Anaerostipes	Control vs T2D
269	0.3294686	0.5806610	Anaerostipes	T1D vs T2D
351	0.4302210	0.6760736	Anaerostipes	LADA vs T1D
23	0.7716586	0.8754881	Anaerostipes	Control vs T1D
433	0.8076673	0.9803916	Anaerostipes	LADA vs T2D

Anaerostipes

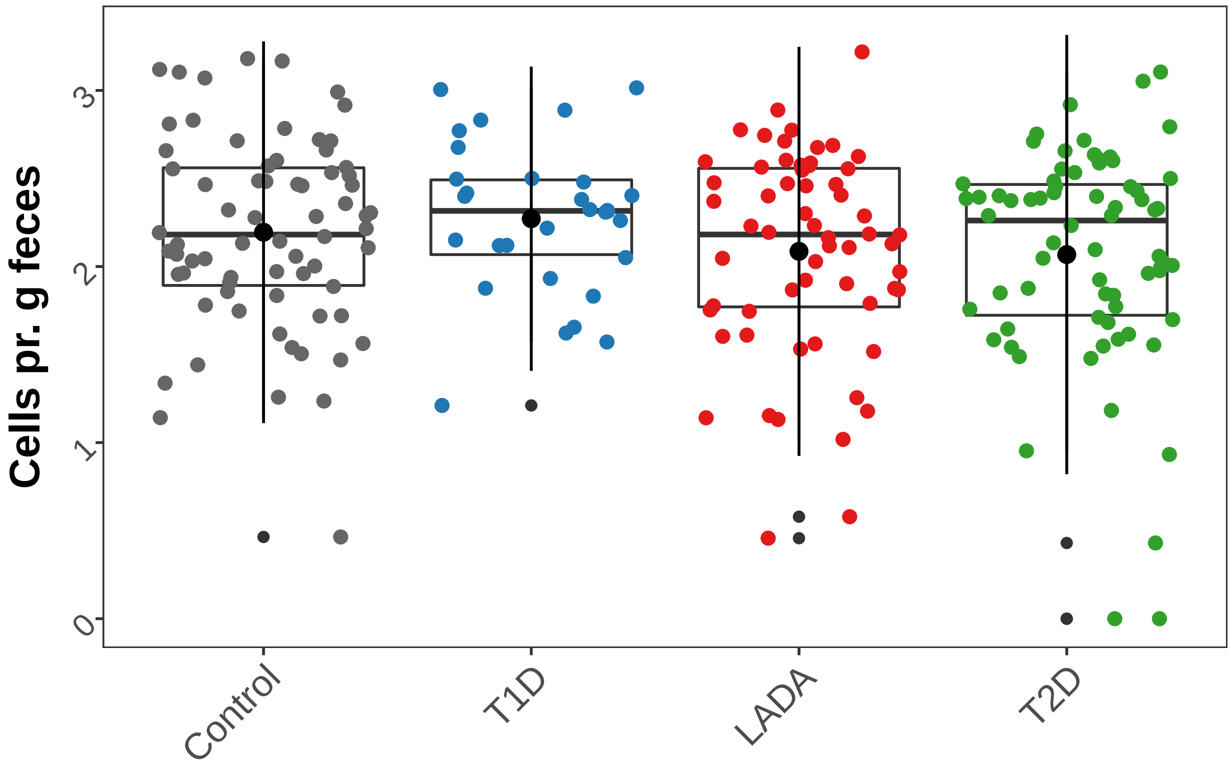

	pvalue	padj	Genus	compare
167	0.6798262	0.8710273	Bifidobacterium	LADA vs Control
3	0.7830703	0.8754881	Bifidobacterium	Control vs T1D
249	0.7349926	0.9131726	Bifidobacterium	T1D vs T2D
85	0.9257307	0.9608850	Bifidobacterium	Control vs T2D
413	0.6109544	0.9724127	Bifidobacterium	LADA vs T2D
331	0.9509381	0.9815672	Bifidobacterium	LADA vs T1D

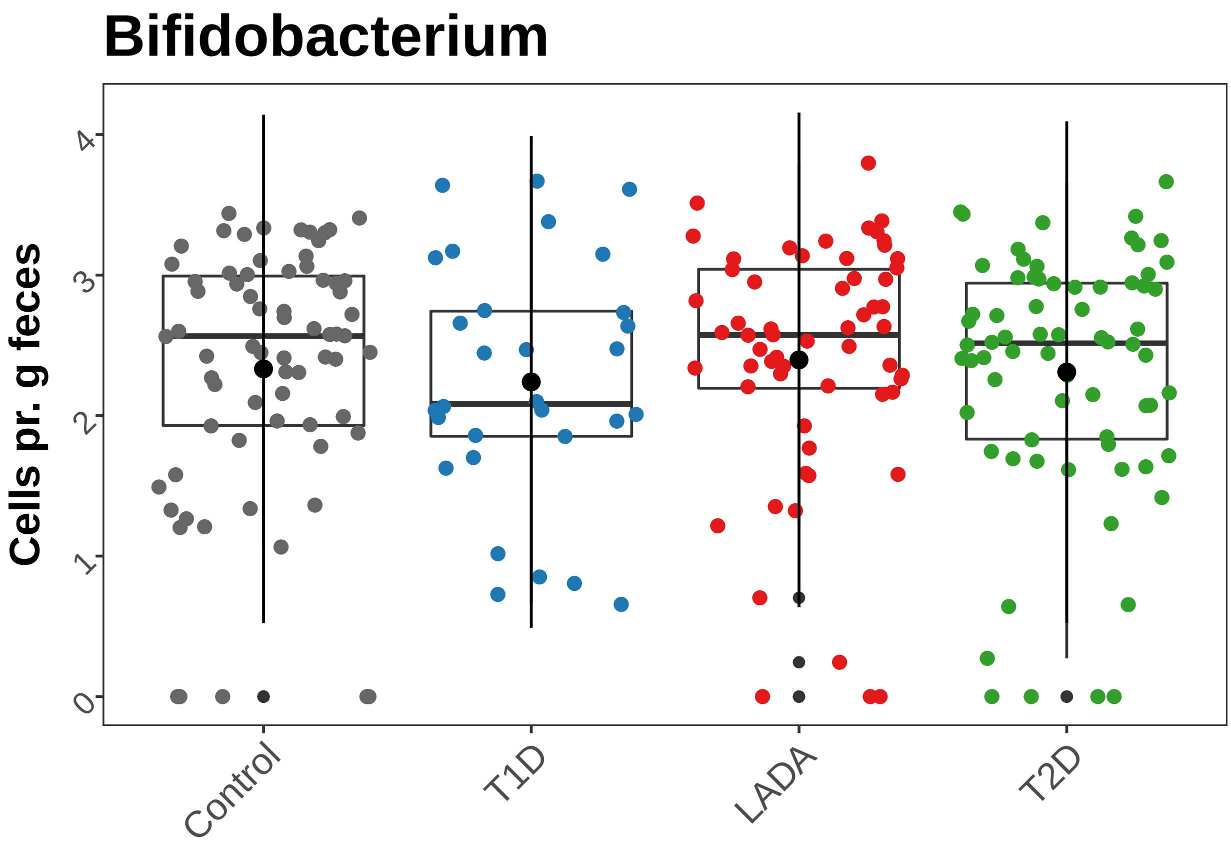

	pvalue	padj	Genus	compare
245	0.1018599	0.3341004	Akkermansia	LADA vs Control
327	0.2812873	0.5517125	Akkermansia	T1D vs T2D
81	0.1578675	0.6671050	Akkermansia	Control vs T1D
163	0.7276007	0.8709821	Akkermansia	Control vs T2D
491	0.1977348	0.8784397	Akkermansia	LADA vs T2D
409	0.9473993	0.9815672	Akkermansia	LADA vs T1D

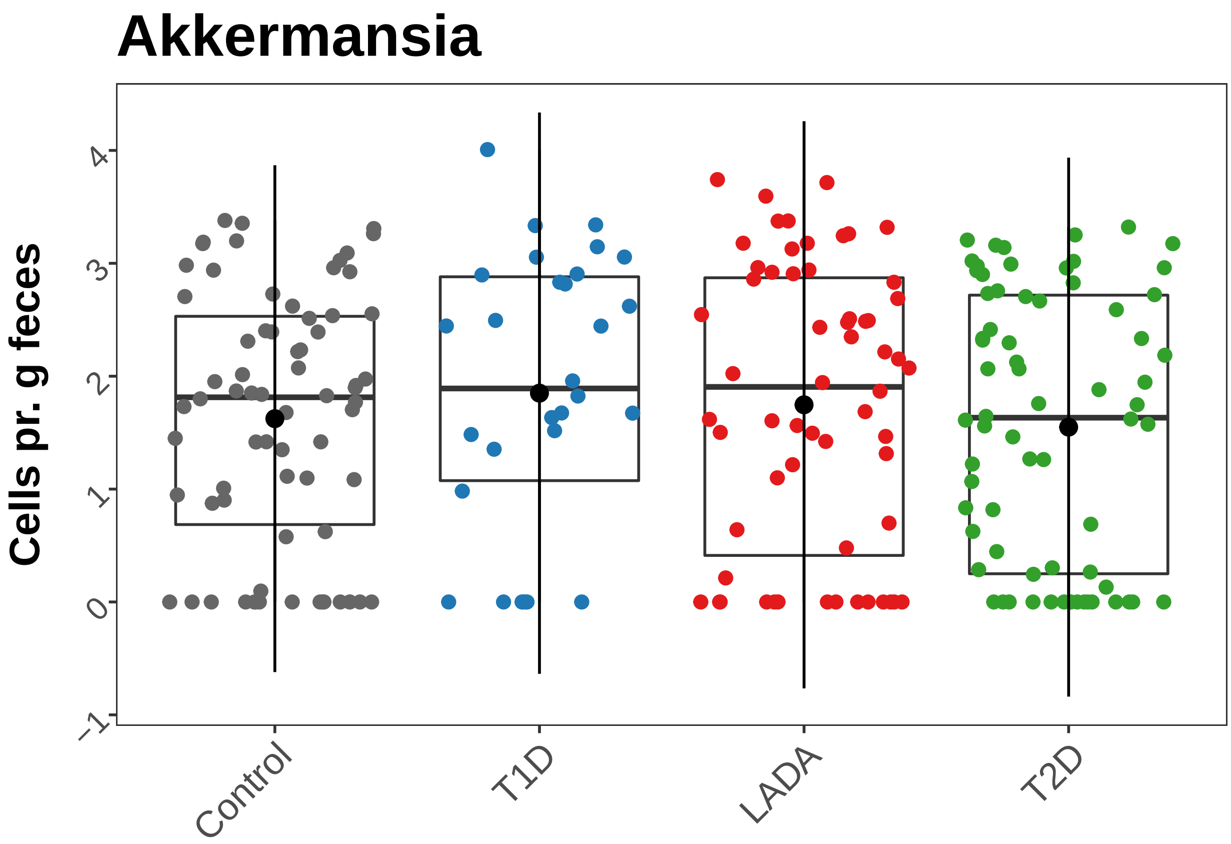


```
lay <- rbind(c(1,2,3))
pdf(paste("MicroLADA_GeneraDA.pdf", sep=""), width=15, height=5)
grid.arrange(Fig2List$Faecalibacterium,
             Fig2List$Roseburia,
             Fig2List$Butyricicoccus,layout_matrix = lay)
dev.off()
```

pdf 2

```
#ggplot(Plotting2, aes(x=BMI, y=Actinomyces)) +
#  geom_point()
```

DESeq Wald Remove metformin

All pairwise Differential abundance analysis comparison (DESeq and visualized venn)

```
rm(list=setdiff(ls(), c("Metadata", "MetadataMed", "Feature", "TaxonomyDA", "Taxonomy", "Fig2List")))
#Create a list to hold the plot objects.
Fig2ListRemMet <- list()

#Reassign names
#Metadata2<-Metadata
Taxonomy2<-TaxonomyDA
```

```
##Removing treated with metformin
#Metadata$Metformin <- ifelse(is.na(Metadata$Metformin), "Unknown", Metadata$Metformin)
Metadata2<-filter(Metadata, !Metformin == 1)
##Applying subsetting to OTU tables
Taxonomy2<-dplyr::select(Taxonomy2, one_of(as.character(Metadata2$MicrobiomeID)))
##Remove orgs that are not present after subsetting.
Taxonomy2 <- Taxonomy2[rowSums(Taxonomy2)>0,]



#Order Diagnosis
Metadata2$Diagnosis<-ordered(Metadata2$Diagnosis,
                             levels=c("Control", "T1D", "T2D", "LADA"))
#The factor can not be ordered for DESeq to run
Metadata2$Diagnosis<-factor(Metadata2$Diagnosis, ordered=FALSE)


#Create design formula
design <- formula(paste("~ ", "Diagnosis")) #To be consistent the analysis with metformin
  #removed is always without correcting for BMI
#design <- formula(paste("~ ", "Diagnosis", "+",  "BMIq"))
#design <- formula(paste("~ ", "Diagnosis", "+ Metformin"))
#Create DESeq2 object with matrix and metadata
dds <- DESeqDataSetFromMatrix(countData = Taxonomy2,
                              colData = Metadata2,
                              design = design)


##Assess and calculate size factors using the different methods
SF<-data.frame(Ratio=sizeFactors(estimateSizeFactors(dds, type="ratio")),
               Poscounts=sizeFactors(estimateSizeFactors(dds, type="poscounts")),
               #"iterate" takes a lot of time changed to "poscounts" but kept due to the
               #following code
               Iterate=sizeFactors(estimateSizeFactors(dds, type="poscounts")),
               Relative=colSums(Taxonomy2)/mean(colSums(Taxonomy2))
               )
SF<-add_rownames(SF, var="MicrobiomeID")
#Adding total abundances
# old path path<-paste("Q:/",
#             "Projects/",
#             "LADA/",
#             "LADA_Sandra_Evelina/",
#             "LADA_JKV/",
#             "LADA_R_AfterFlow_Analysis_FinalCounts/",
#             "LADA_FinalCounts/",
#             "2019-09-03_CountAnalysis.rds", sep="")

path <- paste("J:/",
              "CBMR/",
              "SUN-CBMR-Hansen-Group/",
              "Projects/",
              "LADA/",
              "LADA_Sandra_Evelina/",
```

```
              "LADA_JKV/",
              "LADA_R_AfterFlow_Analysis_FinalCounts/",
              "LADA_FinalCounts/",
              "2019-09-03_CountAnalysis.rds", sep="")

TotalCounts<-readRDS(path)
TotalCountsProcess <-
  data.frame(MicrobiomeID=TotalCounts$DF_Save_w$ID,
             CellNorm=TotalCounts$DF_Save_w$Cells_per_g_feces_FR_corrected_mean)
TotalCountsProcess$MicrobiomeID <- paste("L", TotalCountsProcess$MicrobiomeID,
                                         sep="")
SF2<-merge(SF, TotalCountsProcess, by="MicrobiomeID")

#One value is NaN in sample 602059 assigns value as average
SF2$CellNorm[is.nan(SF2$CellNorm)]<-mean(na.omit(SF2$CellNorm))

SF2$CellNormStand<-SF2$CellNorm/mean(SF2$CellNorm) #Watch out for NaN values can assign
                                                   #average, but wants the error
SF3<-merge(Metadata2, SF2, by="MicrobiomeID")
#DESeq divides with sizeFactor (High cell count should have low sizeFactor providing
#larger counts in a sample)
#Still have to consider seq data is relative
SF3$NormRelCell<-SF3$Relative/SF3$CellNormStand
#Overview of normalization factors
aggregate(SF3[, 14:20], list(SF3$Diagnosis), mean) #Have to run heatmap chunk adding sex
```

Group.1 Ratio Poscounts Iterate Relative CellNorm CellNormStand 1 Control 1.093106 1.002662 1.002662 0.9644647 20384275367 1.0722125 2 T1D 1.232576 1.261833 1.261833 1.1097233 18731782275 0.9852914 3 T2D 1.079416 0.942112 0.942112 0.9618292 16982538343 0.8932812 4 LADA 1.203409 1.213468 1.213468 1.0061418 15590817082 0.8200767 NormRelCell 1 1.008584 2 1.247425 3 1.183913 4 1.292202

```
                                                   #to metadata
aggregate(SF3[, 14:20], list(SF3$Diagnosis), sd)
```

Group.1 Ratio Poscounts Iterate Relative CellNorm CellNormStand 1 Control 0.4622606 0.2998114 0.2998114 0.2305348 6781846681 0.3567250 2 T1D 0.4965321 0.4582512 0.4582512 0.1825149 5712095609 0.3004561 3 T2D 0.5152121 0.3336165 0.3336165 0.2220192 4888235680 0.2571211 4 LADA 0.5245937 0.4814438 0.4814438 0.1937037 4604942130 0.2422199 NormRelCell 1 0.4800604 2 0.4881822 3 0.4797532 4 0.3379476

```
pairs(SF3[, c(14:17,19,20)], col=SF3$Diagnosis)
```

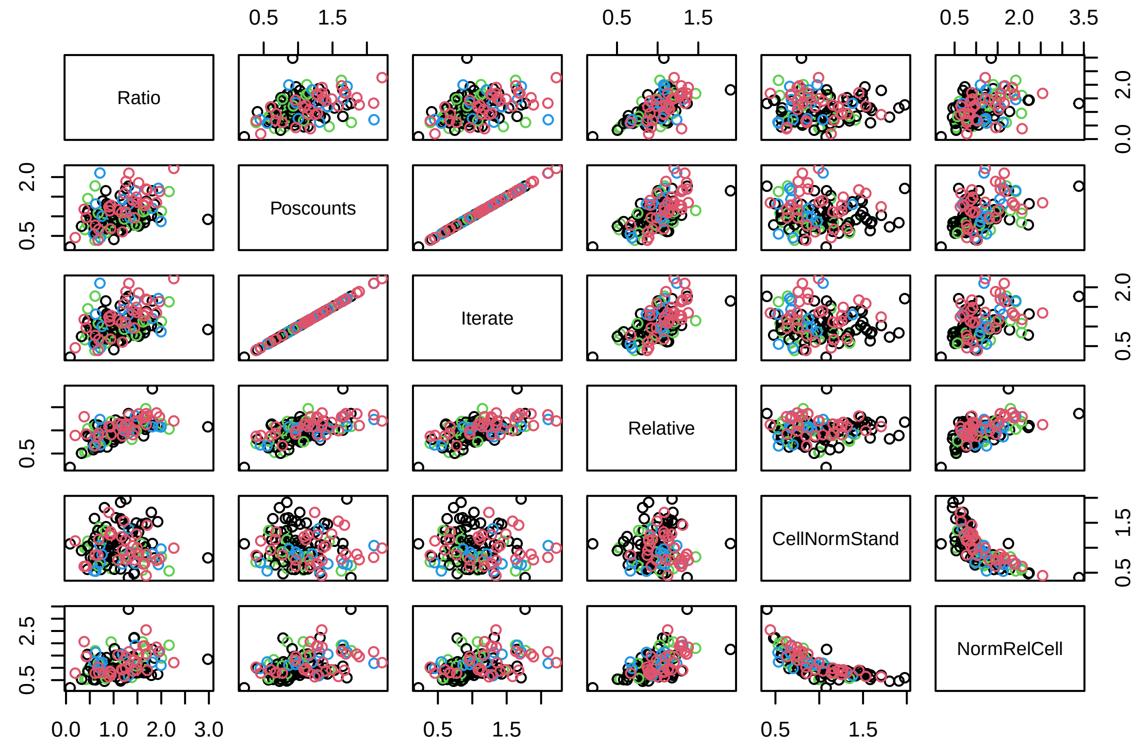


```
pairs(SF3[, c(15,17,19,20)], col=SF3$Diagnosis)
```

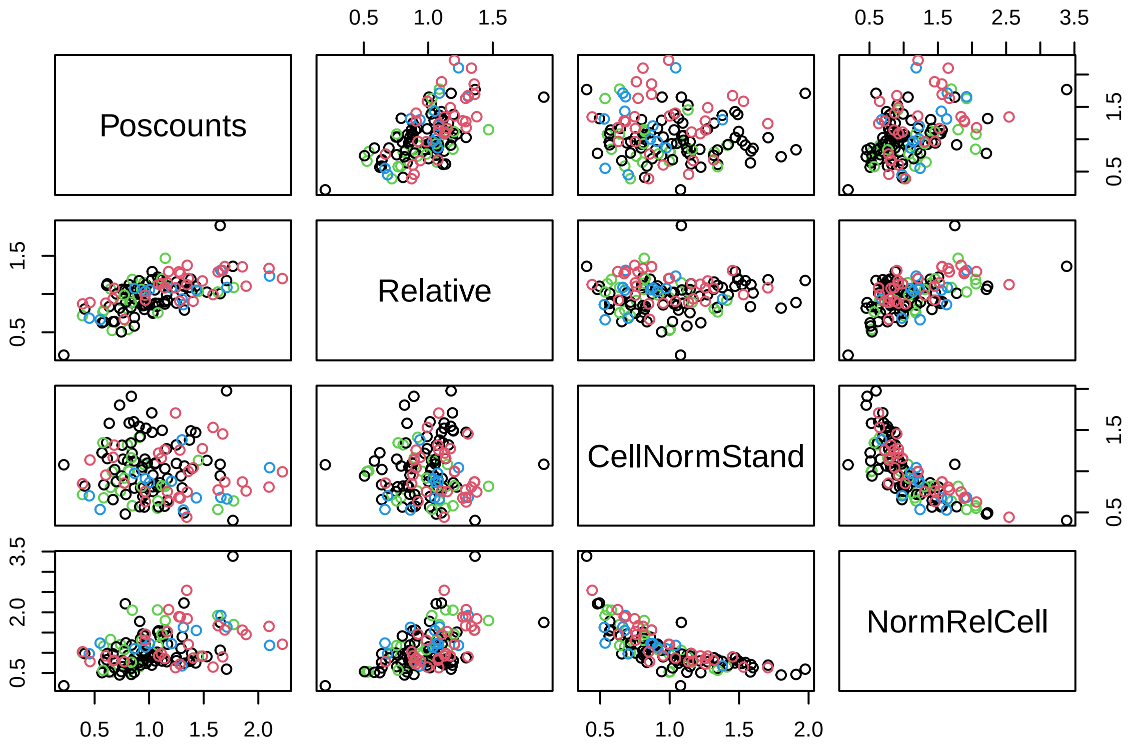


```
#I do want CellNormStand and NormRelCell to be negatively correlated due to DESeq divide
#with sizeFactor.
#It is also good that CellNormStand seems uncorrelated to the other normalization factors.

##Select size factors calculated above for normalization
dds@colData@listData$sizeFactor <- SF3[,20]

##See vignette for note on factor levels
#Filtering of the Counttable. Bare minimum is performed later. Also to reduce memory of

#the dds
dds <- dds[ rowSums(counts(dds))>(50*length(Taxonomy2)),] #Previously 0 instead of 1

#Estimate dispersions and fit the GLM
dds <- DESeq(dds)

res<-results(dds)
summary(res)
```

out of 81 with nonzero total read count adjusted p-value < 0.1 LFC > 0 (up) : 1, 1.2% LFC < 0 (down) : 0, 0% outliers [1] : 0, 0% low counts [2] : 0, 0% (mean count < 44) [1] see 'cooksCutoff' argument of ?results [2] see 'independentFiltering' argument of ?results

```
resultsNames(dds)
```

[1] "Intercept" "Diagnosis_T1D_vs_Control" [3] "Diagnosis_T2D_vs_Control" "Diagnosis_LADA_vs_Control"

```r
#Threshold for FDR-adjusted p-values
padj_threshold <- 0.1

if (is.factor(colData(dds)[,"Diagnosis"]) ==  TRUE) {

#Find all two-wise combinations of levels in chosen test factor
test <- combn(levels(colData(dds)[,"Diagnosis"]), 2)
test[,3]<-c("LADA", "Control") #Want LADA first
test[,5]<-c("LADA", "T1D") #Want LADA first
test[,6]<-c("LADA", "T2D") #Want LADA first
foo = test[1,]
poo = test[2,]

#Do DESeq2 analysis on all combinations of levels and save as list
result <- vector("list",length(foo))
for (i in 1:length(foo) ) {
  for (j in 1:length(poo)) {
    result[i] = results(dds, contrast = c("Diagnosis" ,foo[i], poo[i]))
  }
}

#Produce output tables with significant taxa from each comparison
res_list <- vector("list",length(foo))
for (i in 1:length(result) ) {
  res_stat <- data.frame(result[i])
  res_stat <- res_stat[is.na(res_stat$pvalue) == FALSE,]
  stat_sig <- na.omit(res_stat[res_stat$padj < padj_threshold,])
  res_stat$gene <- rownames(res_stat)
  res_stat$g1 <- test[1,i]
  res_stat$g2 <- test[2,i]
  res_stat$compare <- paste(test[1,i], "vs", test[2,i])
  res_list[i] <- list(res_stat)
}

res_stat <- do.call('rbind', res_list)
rownames(res_stat) <- 1:nrow(res_stat)
res_stat <- res_stat[with(res_stat, order(padj, pvalue, abs(log2FoldChange))),]
stat_sig <- na.omit(res_stat[res_stat$padj < padj_threshold,])
}

if (is.numeric(colData(dds)[,"Diagnosis"]) ==  TRUE) {

res_stat <- data.frame((results(dds)))
res_stat$maxCooks <- apply(assays(dds)[["cooks"]], 1, max)
res_stat <- res_stat[is.na(res_stat$pvalue) == FALSE,]
res_stat <- res_stat[with(res_stat, order(padj, pvalue, abs(log2FoldChange))), ]
res_stat$gene <- rownames(res_stat)
max_cooks <- quantile(na.omit(res_stat$maxCooks), cooks_quantile_cutoff)
stat_sig <-
  na.omit(res_stat[res_stat$padj < padj_threshold & res_stat$maxCooks < max_cooks,])
}

#All comparisons
```

```r
write.table(res_stat, file="DESeqRes_OrgsRemMet.txt", quote = F, row.names = F, sep="\t")

#Significant taxa in table
if (nrow(stat_sig) > 0) {
kable(stat_sig, row.names = F)
write.table(stat_sig, file="DESeqRes_SigOrgsRemMet.txt", quote = F, row.names = F, sep="\t")
} else {
  print("No significant taxa were found.")
}
print("Significant Wald")
```

[1] "Significant Wald"

```r
kable(stat_sig)
```

	baseMean	log2FoldChange	lfcSE	stat	pvalue	padj	gene	g1	g2	compare
211	697.4217	1.5832131	0.4268749	3.708845	0.0002082	0.0168647	Ruminiclostridium_5	LADA	Control	LADA vs Control
44	14234.2496	1.0587494	0.2977371	3.555987	0.0003766	0.0229456	Faecalibacterium	Control	T1D	Control vs T1D
39	1611.2597	1.2643592	0.3667852	3.447138	0.0005666	0.0229456	Roseburia	Control	T1D	Control vs T1D
251	507.8234	2.5736292	0.7098165	3.625767	0.0002881	0.0233365	Family.Eggerthellaceae.	T1D	T2D	T1D vs T2D
89	507.8234	2.1949066	0.6156984	3.564905	0.0003640	0.0294830	Family.Eggerthellaceae.	Control	T2D	Control vs T2D
274	626.6481	-1.4400079	0.4333450	-3.323005	0.0008905	0.0360666	Lachnoclostridium	T1D	T2D	T1D vs T2D
259	1626.6339	1.8809095	0.5865600	3.206679	0.0013428	0.0362547	Streptococcus	T1D	T2D	T1D vs T2D
286	854.9916	-1.0420055	0.3375294	-3.087155	0.0020208	0.0409217	Butyricicoccus	T1D	T2D	T1D vs T2D
43	854.9916	0.8229626	0.2658188	3.095954	0.0019618	0.0529689	Butyricicoccus	Control	T1D	Control vs T1D
293	289.8053	1.9703268	0.6798502	2.898178	0.0037534	0.0608047	Ruminiclostridium_6	T1D	T2D	T1D vs T2D
97	1626.6339	1.6090377	0.5086961	3.163063	0.0015612	0.0632280	Streptococcus	Control	T2D	Control vs T2D
21	5945.1388	1.0283974	0.3493361	2.943862	0.0032414	0.0656392	Agathobacter	Control	T1D	Control vs T1D
112	626.6481	-1.1067154	0.3757587	-2.945283	0.0032266	0.0871181	Lachnoclostridium	Control	T2D	Control vs T2D

```r
kable(stat_sig[,c(5:7,10)])
```

	pvalue	padj	gene	compare
211	0.0002082	0.0168647	Ruminiclostridium_5	LADA vs Control
44	0.0003766	0.0229456	Faecalibacterium	Control vs T1D
39	0.0005666	0.0229456	Roseburia	Control vs T1D

	pvalue	padj	gene	compare
251	0.0002881	0.0233365	Family.Eggerthellaceae.	T1D vs T2D
89	0.0003640	0.0294830	Family.Eggerthellaceae.	Control vs T2D
274	0.0008905	0.0360666	Lachnoclostridium	T1D vs T2D
259	0.0013428	0.0362547	Streptococcus	T1D vs T2D
286	0.0020208	0.0409217	Butyricicoccus	T1D vs T2D
43	0.0019618	0.0529689	Butyricicoccus	Control vs T1D
293	0.0037534	0.0608047	Ruminiclostridium_6	T1D vs T2D
97	0.0015612	0.0632280	Streptococcus	Control vs T2D
21	0.0032414	0.0656392	Agathobacter	Control vs T1D
112	0.0032266	0.0871181	Lachnoclostridium	Control vs T2D

```
##Venn diagram comparing LADA to other groups
sig_LADA <- subset(stat_sig, g1=="LADA")
sig_LADA_T1D <- subset(sig_LADA, g2=="T1D") %>% dplyr::select(one_of("gene")) %>%
  pull(gene)
sig_LADA_T2D <- subset(sig_LADA, g2=="T2D") %>% dplyr::select(one_of("gene")) %>%
  pull(gene)
sig_LADA_Control <- subset(sig_LADA, g2=="Control") %>% dplyr::select(one_of("gene")) %>%
  pull(gene)

#Colors
myCol <- c( "#1F78B4", "#33A02C", "#666666")
#
temp <- venn.diagram(list(T1D = sig_LADA_T1D,
                          T2D = sig_LADA_T2D,
                          Control = sig_LADA_Control), filename = NULL,

                     # Circles
                     lwd = 2,
                     lty = 'blank',
                     fill = myCol,

                     # Numbers
                     cex = 3,
                     fontface = "bold",
                     fontfamily = "sans",

                     # Set names
                     cat.cex = 3,
                     cat.fontface = "bold",
                     cat.default.pos = "outer",
                     cat.pos = c(0, 0, 0),
                     cat.dist = c(0.085, 0.085, 0.024),
                     cat.fontfamily = "sans",
                     rotation = 1)

grid.arrange(gTree(children=temp))
```

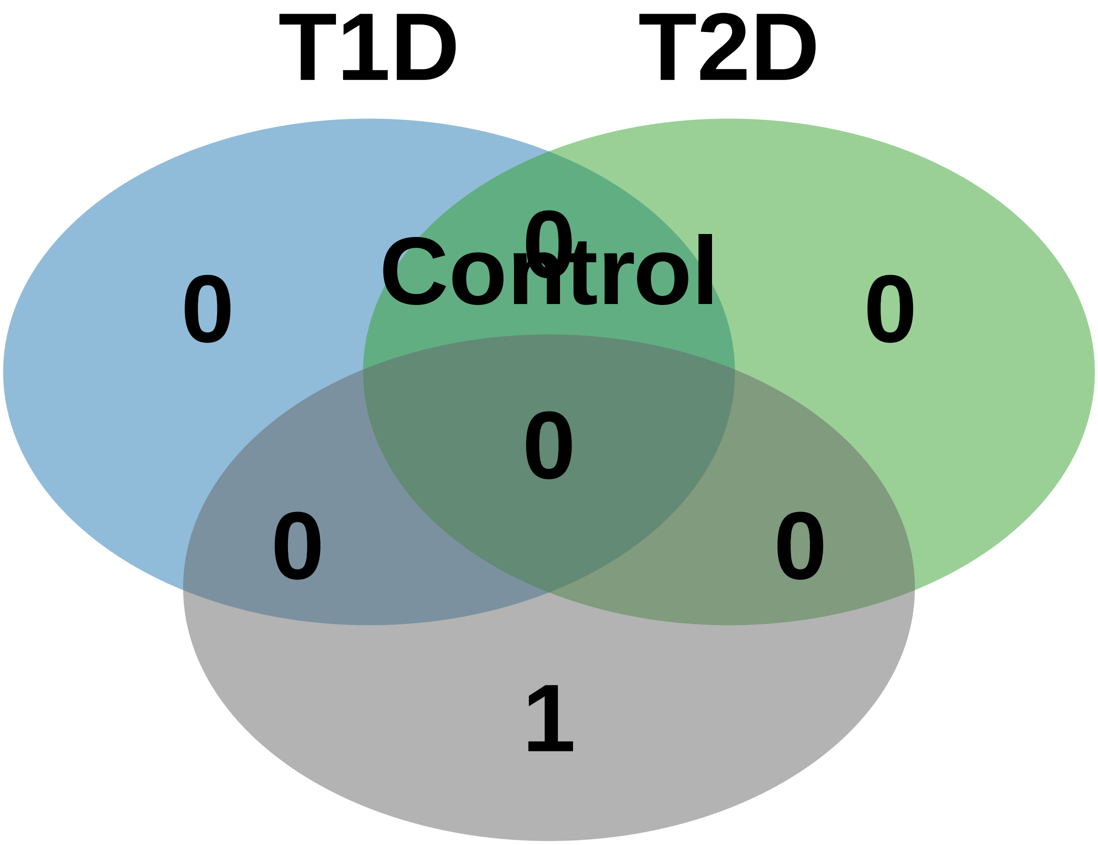


```
Fig2ListRemMet[[ "VennLADA" ]] <-
  gTree(children=temp, top="N differential abundant relative to LADA")

#Genera LADA that are significantly different from all other groups
intersect(intersect(sig_LADA_T1D,sig_LADA_T2D),sig_LADA_Control)
```

character(0)

```
##Venn diagram comparing T1D to other groups
sig_T1D <- subset(stat_sig, g1=="T1D" | g2=="T1D")
sig_T1D_LADA <- subset(sig_T1D, g1=="LADA") %>% dplyr::select(one_of("gene")) %>%
  pull(gene)
sig_T1D_T2D <- subset(sig_T1D, g2=="T2D") %>% dplyr::select(one_of("gene")) %>%
  pull(gene)
sig_T1D_Control <- subset(sig_T1D, g1=="Control") %>% dplyr::select(one_of("gene")) %>%
  pull(gene)
#Colors
myCol <- c( "#E31A1C", "#33A02C", "#666666")
#
temp <- venn.diagram(list(LADA = sig_T1D_LADA,
                          T2D = sig_T1D_T2D,
                          Control = sig_T1D_Control), filename = NULL,

                     # Circles
                     lwd = 2,
```

```
                      lty = 'blank',
                      fill = myCol,

                      # Numbers
                      cex = 2,
                      fontface = "bold",
                      fontfamily = "sans",

                      # Set names
                      cat.cex = 2,
                      cat.fontface = "bold",
                      cat.default.pos = "outer",
                      cat.pos = c(0, 0, 0),
                      cat.dist = c(0.055, 0.055, 0.024),
                      cat.fontfamily = "sans",
                      rotation = 1)

grid.arrange(gTree(children=temp))
```

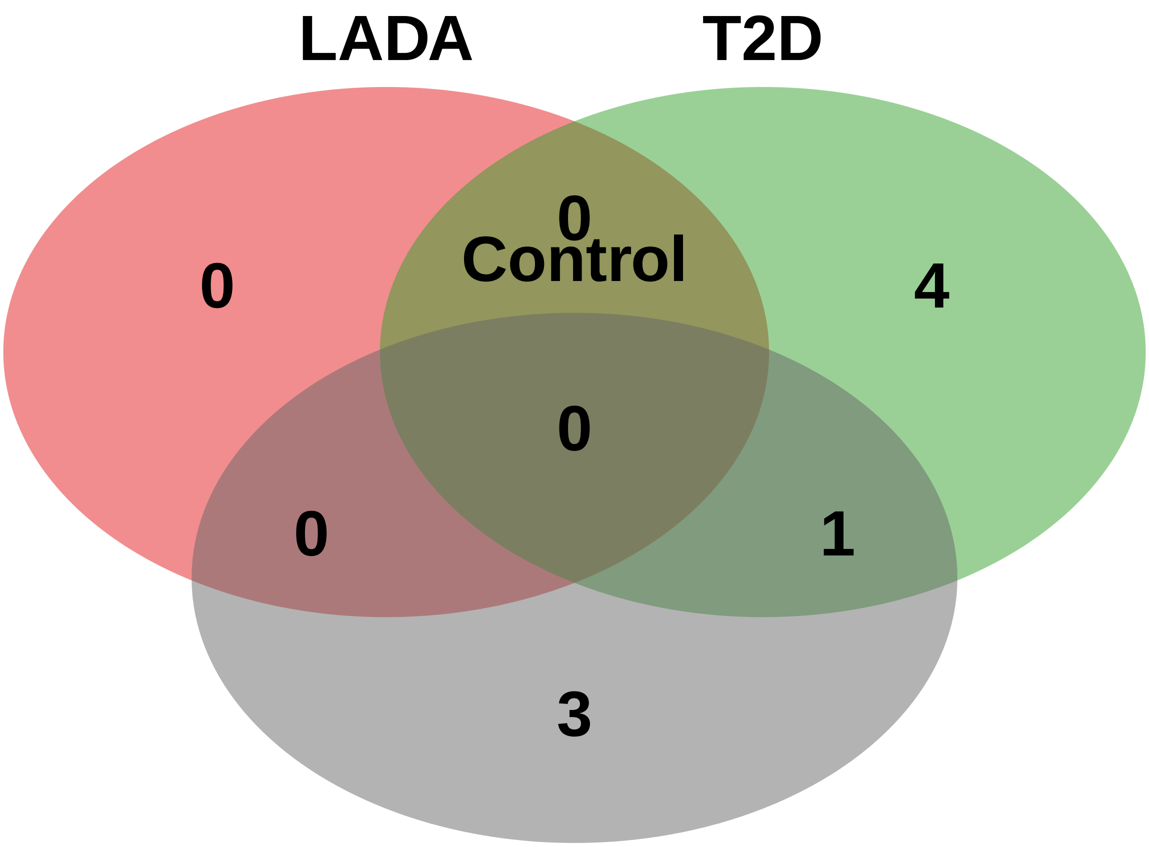


```
Fig2ListRemMet[[ "VennT1D" ]] <-
  gTree(children=temp, top="N differential abundant relative to T1D")

#Genera T1D that are significantly different from all other groups
intersect(intersect(sig_T1D_LADA,sig_T1D_T2D),sig_T1D_Control)
```

character(0)

```
##Venn diagram comparing T2D to other groups
sig_T2D <- subset(stat_sig, g1=="T2D" | g2=="T2D")
sig_T2D_LADA <- subset(sig_T2D, g1=="LADA") %>% dplyr::select(one_of("gene")) %>%
  pull(gene)
sig_T2D_T1D <- subset(sig_T2D, g1=="T1D") %>% dplyr::select(one_of("gene")) %>%
  pull(gene)
sig_T2D_Control <- subset(sig_T2D, g1=="Control") %>% dplyr::select(one_of("gene")) %>%
  pull(gene)
#Colors
myCol <- c( "#E31A1C", "#1F78B4", "#666666")
#
temp <- venn.diagram(list(LADA = sig_T2D_LADA,
                          T1D = sig_T2D_T1D,
                          Control = sig_T2D_Control), filename = NULL,

                     # Circles
                     lwd = 2,
                     lty = 'blank',
                     fill = myCol,

                     # Numbers
                     cex = 2,
                     fontface = "bold",
                     fontfamily = "sans",

                     # Set names
                     cat.cex = 2,
                     cat.fontface = "bold",
                     cat.default.pos = "outer",
                     cat.pos = c(0, 0, 0),
                     cat.dist = c(0.055, 0.055, 0.024),
                     cat.fontfamily = "sans",
                     rotation = 1)

grid.arrange(gTree(children=temp))
```

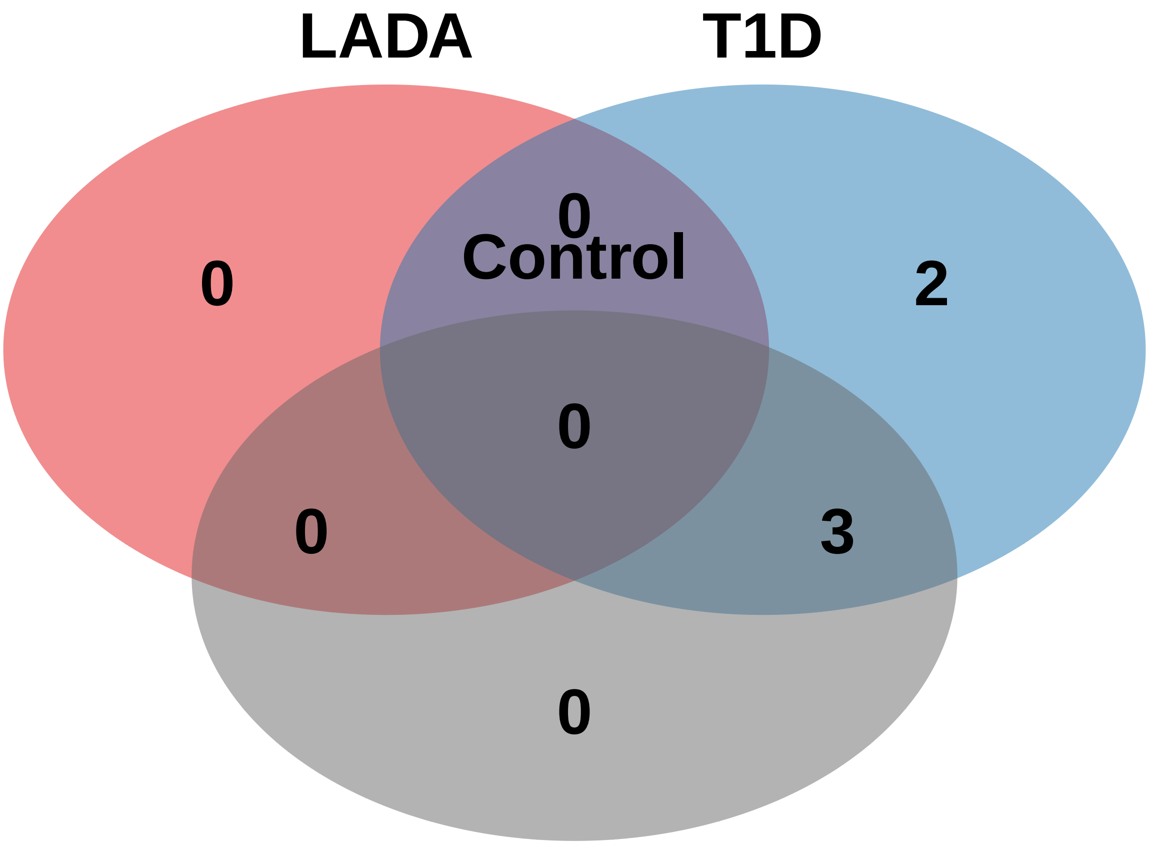


```
Fig2ListRemMet[[ "VennT2D" ]] <-
  gTree(children=temp, top="N differential abundant relative to T2D")

#Genera T2D that are significantly different from all other groups
intersect(intersect(sig_T2D_LADA,sig_T2D_T1D),sig_T2D_Control)
```

character(0)

```
##Venn diagram comparing controls to other groups
sig_Control <- subset(stat_sig, g1=="Control" | g2=="Control")
sig_Control_LADA <- subset(sig_Control, g1=="LADA") %>% dplyr::select(one_of("gene")) %>%
  pull(gene)
sig_Control_T1D <- subset(sig_Control, g2=="T1D") %>% dplyr::select(one_of("gene")) %>%
  pull(gene)
sig_Control_T2D <- subset(sig_Control, g2=="T2D") %>% dplyr::select(one_of("gene")) %>%
  pull(gene)
#Colors
myCol <- c( "#E31A1C", "#1F78B4", "#33A02C")
#
temp <- venn.diagram(list(LADA = sig_Control_LADA,
                          T1D = sig_Control_T1D,
                          T2D = sig_Control_T2D), filename = NULL,

                     # Circles
                     lwd = 2,
```

```
                     lty = 'blank',
                     fill = myCol,

                     # Numbers
                     cex = 3,
                     fontface = "bold",
                     fontfamily = "sans",

                     # Set names
                     cat.cex = 3,
                     cat.fontface = "bold",
                     cat.default.pos = "outer",
                     cat.pos = c(0, 0, 0),
                     cat.dist = c(0.055, 0.055, 0.024),
                     cat.fontfamily = "sans",
                     rotation = 1)

grid.arrange(gTree(children=temp))
```

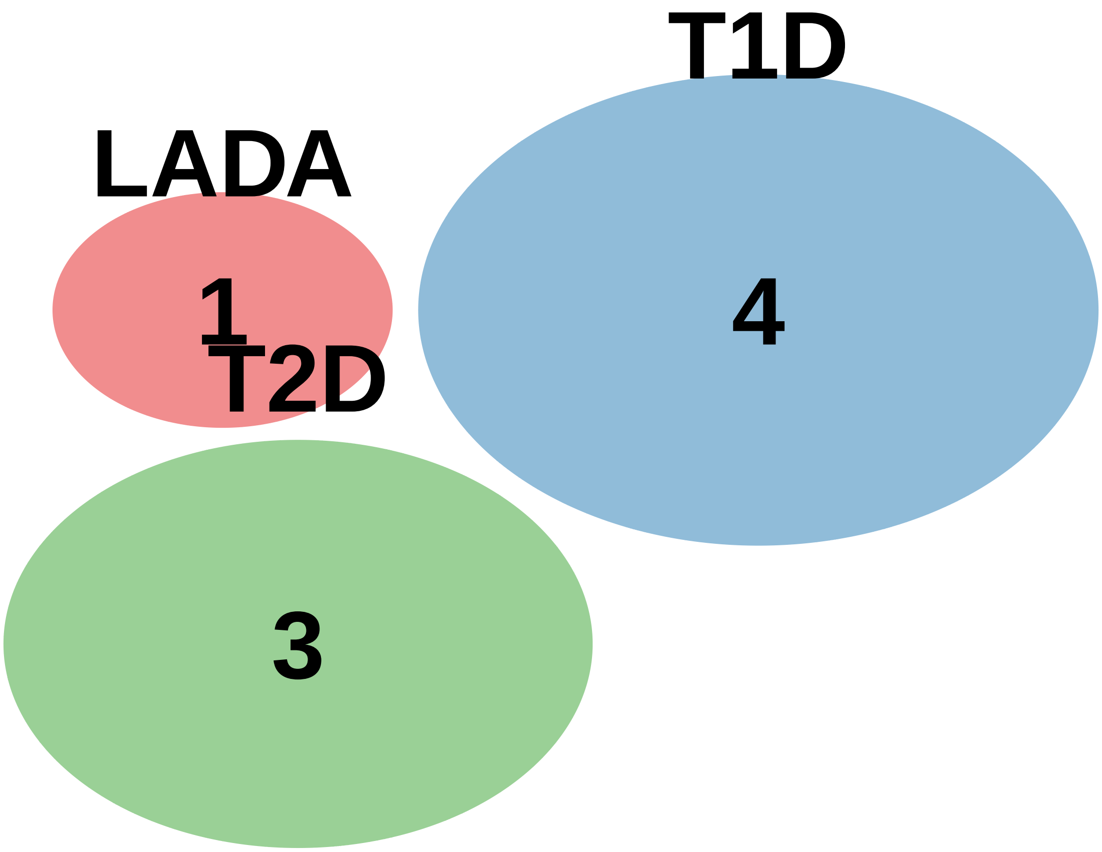


```
Fig2ListRemMet[[ "VennControls" ]] <-
  gTree(children=temp, top="N differential abundant relative to controls")

#Genera Controls that are significantly different from all other groups
intersect(intersect(sig_T2D_LADA,sig_T2D_T1D),sig_T2D_Control)
```

character(0)

```
##Create boxplots / violin plots
#Normalize to get cells pr. gram feces
if (setequal(colnames(Taxonomy2), SF3$MicrobiomeID)==FALSE) {
  stop("Metadata and Taxonomy out of sync")
}

#Total sum scaling (Use relative abundances)
Taxonomy3<-sweep(Taxonomy2, 2, colSums(Taxonomy), FUN="/")
#Obtain values as cells pr. gram feces
Taxonomy3<-sweep(Taxonomy3, 2, SF3$CellNorm, FUN="*")
#Make to micro gram
Taxonomy3<-Taxonomy3/10^6


##Select organisms
SelOrgs<-unique(stat_sig$gene)
##Add SCFA producers see Venegas et al. 2019
SelOrgs<-unique(c(SelOrgs,
                  "Faecalibacterium", #prausnitzii
#                  "Clostridium", #leptum present as sensu stricto 1
#                  "Eubacterium", #rectale or hallii, here linosum
                  "Roseburia",  #
                  "Anaerostipes",
                  "Bifidobacterium",
                  "Butyricicoccus",
                  "Akkermansia"))

tTaxSelect<-dplyr::select(as.data.frame(t(Taxonomy3)), one_of(c(SelOrgs)))
#Reducing the number because duplicates are removed
tTaxSelect<-add_rownames(tTaxSelect, "MicrobiomeID")
Plotting<-merge(SF3, tTaxSelect, by="MicrobiomeID")

##Might have issues with special characters, so this chunk might be needed
SelOrgs<-str_replace(SelOrgs, " ","_")
SelOrgs<-str_replace(SelOrgs, "/","_")
SelOrgs<-gsub("\\[|\\]", "", SelOrgs)
SelOrgs<-str_replace(SelOrgs, ":","_")
colnames(Plotting) <- str_replace(colnames(Plotting)," ","_")
colnames(Plotting) <- str_replace(colnames(Plotting),"/","_")
colnames(Plotting) <- gsub("\\[|\\]", "", colnames(Plotting))
colnames(Plotting) <- str_replace(colnames(Plotting),":","_")

#Plot logs on y axis
Plotting2 <- bind_cols(Plotting[,1:16], log10(Plotting[,17:ncol(Plotting)]+1))
#See end of chunk for boxplots removed the violinplots, because of redundancy


##Create vulcano plots
#Cut offs Benjamini-Hochberg method to add to vulcano plot
#Don't know how to get exact so it is a cut-off corresponding to the BH method
BHAll<-aggregate(stat_sig[, 5:6], list(stat_sig$compare), max)
row.names(BHAll)<-BHAll$Group.1
```

```
BHLadaCon <- BHAll[which(rownames(BHAll)=="LADA vs Control"),
                   which(colnames(BHAll)=="pvalue")]
BHLadaT1D <- BHAll[which(rownames(BHAll)=="LADA vs T1D"),
                   which(colnames(BHAll)=="pvalue")]
BHLadaT2D <- BHAll[which(rownames(BHAll)=="LADA vs T2D"),
                   which(colnames(BHAll)=="pvalue")]


#Vulcano plot
res_stat$minuslog10<--log(res_stat$pvalue)
#
#range(res_stat$minuslog10)
#range(res_stat$log2FoldChange)

names(res_stat)[names(res_stat) == 'gene'] <- 'Genus'
Feature2<-merge(res_stat, Feature, by="Genus")

#Create column org grouping for colouring
Feature2$Phyla <- ifelse(Feature2$Phylum=="Firmicutes", "Firmicutes",
          ifelse(Feature2$Phylum=="Proteobacteria", "Proteobacteria",
          ifelse(Feature2$Phylum=="Actinobacteria", "Actinobacteria",
          ifelse(Feature2$Phylum=="Bacteroidetes", "Bacteroidetes",
          ifelse(Feature2$Phylum=="Euryarchaeota", "Euryarchaeota",
                 "Other")))))
#Order Phyla for plotting
Feature2$Phyla <- factor(Feature2$Phyla, levels=c("Proteobacteria", "Bacteroidetes",
                                                  "Actinobacteria", "Firmicutes",
                                                  "Euryarchaeota", "Other"))


##Summary significant orgs
#aggregate(Plotting[, 17:ncol(Plotting)], list(Plotting$Diagnosis), mean)
#aggregate(Plotting[, 17:ncol(Plotting)], list(Plotting$Diagnosis), sd)
zeroes <- function(x){
  sum(x == 0)
}
#aggregate(Plotting[, 17:ncol(Plotting)], list(Plotting$Diagnosis), zeroes)
zerocounts <- aggregate(Plotting[, 17:ncol(Plotting)], list(Plotting$Diagnosis), zeroes)
#bind_cols(zerocounts[,1], zerocounts[,2:ncol(zerocounts)]/c(70,30,70,70)*100)


#Add prevalence to plotting
prevalence<-data.frame((240-apply(Taxonomy, 1, zeroes))/240*100)
colnames(prevalence) <- c("Prevalence")
prevalence<-add_rownames(prevalence, var = "Genus")
Feature2 <- merge(Feature2, prevalence, by="Genus")


#Define boundaries
#Always run first without these lines to get indication of very low and high
#log2FoldChange and pvalue
Featurein <- filter(Feature2, -3<log2FoldChange & log2FoldChange<3 & 10>minuslog10)
Featureout <- filter(Feature2, -3>log2FoldChange | 3<log2FoldChange | 10<minuslog10)
```

```r
Featureout$log2FoldChange[Featureout$log2FoldChange > 3] <- 3
Featureout$log2FoldChange[Featureout$log2FoldChange < (-3)] <- -3
Featureout$minuslog10[Featureout$minuslog10 > 10] <- 10


#Add text to vulcano plots
p1<-ggplot() +
  geom_point(data=Featurein[which(Featurein$Phyla=="Proteobacteria" &
                                    Featurein$compare=="LADA vs T1D"), ],
             aes(x=log2FoldChange, y=minuslog10, group=Genus), color="#A6CEE3", shape=16,
             size=Featurein[which(Featurein$Phyla=="Proteobacteria" &
                                    Featurein$compare=="LADA vs T1D"), ]
             [,ncol(Featurein)]/40+0.5) +
  #did not add jitter to the first on purpose. Sizes are scaled from percent
  #to the range 0.5-3
  geom_point(data=Featureout[which(Featureout$Phyla=="Proteobacteria" &
                                     Featureout$compare=="LADA vs T1D"), ],
             aes(x=log2FoldChange, y=minuslog10, group=Genus), color="#A6CEE3", shape=18,
             size=Featureout[which(Featureout$Phyla=="Proteobacteria" &
                                     Featureout$compare=="LADA vs T1D"), ]
             [,ncol(Featureout)]/40+0.5) +
  geom_point(data=Featurein[which(Featurein$Phyla=="Bacteroidetes" &
                                    Featurein$compare=="LADA vs T1D"), ],
             aes(x=log2FoldChange, y=minuslog10, group=Genus), color="#FB9A99", shape=16,
             size=Featurein[which(Featurein$Phyla=="Bacteroidetes" &
                                    Featurein$compare=="LADA vs T1D"), ]
             [,ncol(Featurein)]/40+0.5) +
  geom_jitter(data=Featureout[which(Featureout$Phyla=="Bacteroidetes" &
                                      Featureout$compare=="LADA vs T1D"), ],
             aes(x=log2FoldChange, y=minuslog10, group=Genus), color="#FB9A99", shape=18,
             size=Featureout[which(Featureout$Phyla=="Bacteroidetes" &
                                     Featureout$compare=="LADA vs T1D"), ]
             [,ncol(Featureout)]/40+0.5, width = 0.1, height = 0.1) +
  geom_point(data=Featurein[which(Featurein$Phyla=="Actinobacteria" &
                                    Featurein$compare=="LADA vs T1D"), ],
             aes(x=log2FoldChange, y=minuslog10, group=Genus), color="#FDBF6F", shape=16,
             size=Featurein[which(Featurein$Phyla=="Actinobacteria" &
                                    Featurein$compare=="LADA vs T1D"), ]
             [,ncol(Featurein)]/40+0.5) +
  geom_jitter(data=Featureout[which(Featureout$Phyla=="Actinobacteria" &
                                      Featureout$compare=="LADA vs T1D"), ],
             aes(x=log2FoldChange, y=minuslog10, group=Genus), color="#FDBF6F", shape=18,
             size=Featureout[which(Featureout$Phyla=="Actinobacteria" &
                                     Featureout$compare=="LADA vs T1D"), ]
             [,ncol(Featureout)]/40+0.5, width = 0.1, height = 0.1) +
  geom_point(data=Featurein[which(Featurein$Phyla=="Firmicutes" &
                                    Featurein$compare=="LADA vs T1D"), ],
             aes(x=log2FoldChange, y=minuslog10, group=Genus),
             color="#B2DF8A", shape=16, alpha=0.75,
             size=Featurein[which(Featurein$Phyla=="Firmicutes" &
                                    Featurein$compare=="LADA vs T1D"), ]
             [,ncol(Featurein)]/40+0.5) +
  geom_jitter(data=Featureout[which(Featureout$Phyla=="Firmicutes" &
```

```
                                        Featureout$compare=="LADA vs T1D"), ],
            aes(x=log2FoldChange, y=minuslog10, group=Genus),
            color="#B2DF8A", shape=18,
            size=Featureout[which(Featureout$Phyla=="Firmicutes" &
                                       Featureout$compare=="LADA vs T1D"), ]
            [,ncol(Featureout)]/40+0.5, width = 0.1, height = 0.1) +
  geom_point(data=Featurein[which(Featurein$Phyla=="Euryarchaeota" &
                                      Featurein$compare=="LADA vs T1D"), ],
            aes(x=log2FoldChange, y=minuslog10, group=Genus), color="#CAB2D6", shape=16,
            size=Featurein[which(Featurein$Phyla=="Euryarchaeota" &
                                      Featurein$compare=="LADA vs T1D"), ]
            [,ncol(Featurein)]/40+0.5) +
  geom_jitter(data=Featureout[which(Featureout$Phyla=="Euryarchaeota" &
                                        Featureout$compare=="LADA vs T1D"), ],
            aes(x=log2FoldChange, y=minuslog10, group=Genus), color="#CAB2D6", shape=18,
            size=Featureout[which(Featureout$Phyla=="Euryarchaeota" &
                                       Featureout$compare=="LADA vs T1D"), ]
            [,ncol(Featureout)]/40+0.5, width = 0.1, height = 0.1) +
  geom_point(data=Featurein[which(Featurein$Phyla=="Other" &
                                      Featurein$compare=="LADA vs T1D"), ],
            aes(x=log2FoldChange, y=minuslog10, group=Genus), color="#B3B3B3", shape=16,
            size=Featurein[which(Featurein$Phyla=="Other" &
                                      Featurein$compare=="LADA vs T1D"), ]
            [,ncol(Featurein)]/40+0.5) +
  geom_jitter(data=Featureout[which(Featureout$Phyla=="Other" &
                                        Featureout$compare=="LADA vs T1D"), ],
            aes(x=log2FoldChange, y=minuslog10, group=Genus), color="#B3B3B3", shape=18,
            size=Featureout[which(Featureout$Phyla=="Other" &
                                       Featureout$compare=="LADA vs T1D"), ]
            [,ncol(Featureout)]/40+0.5, width = 0.1, height = 0.1) +
  geom_text(data=Featurein[which(Featurein$padj<padj_threshold &
                                     Featurein$compare=="LADA vs T1D"), ],
            aes(x=log2FoldChange, y=minuslog10, group=Genus, label=Genus),
           nudge_y = 0.2, size=2.5) +
  geom_text(data=Featureout[which(Featureout$padj<padj_threshold &
                                      Featureout$compare=="LADA vs T1D"), ],
            aes(x=log2FoldChange, y=minuslog10, group=Genus, label=Genus),
           nudge_x=0.6, nudge_y = -0.2, size=2.5) +
#  geom_text(data=Featureout[which(Featureout$padj<padj_threshold &
#                                     Featureout$compare=="LADA vs T1D" &
#                                     Featureout$Genus=="Rikenellaceae_RC9_gut_group"), ],
#             aes(x=log2FoldChange, y=minuslog10, group=Genus, label=Genus),
#            nudge_x=1.22, nudge_y = 0) +
  geom_hline(yintercept = -log(0.05), linetype="dashed") +
  geom_hline(yintercept = -log(BHLadaT1D), linetype="dashed", alpha=0.1) +
  geom_vline(xintercept = -1, linetype="dashed") +
  geom_vline(xintercept = 1, linetype="dashed") +
  theme_bw() +
  ggtitle("T1D") +
  xlab("Log2 fold change") +
  ylab("-log10(P)") +
  theme(panel.grid.major = element_blank(),
        panel.grid.minor = element_blank(),
```

```
        axis.title=element_text(size=22),
        legend.position="bottom",
        legend.title=element_text(size=20),
        legend.text=element_text(size=20),
        axis.text.x = element_text(angle = 45, hjust = 1, size=12),
        axis.text.y = element_text(angle = 45, hjust = 1, size=12),
        plot.title = element_text(size = 22, hjust=0.5))
#ggplotly(p1)
p1
```

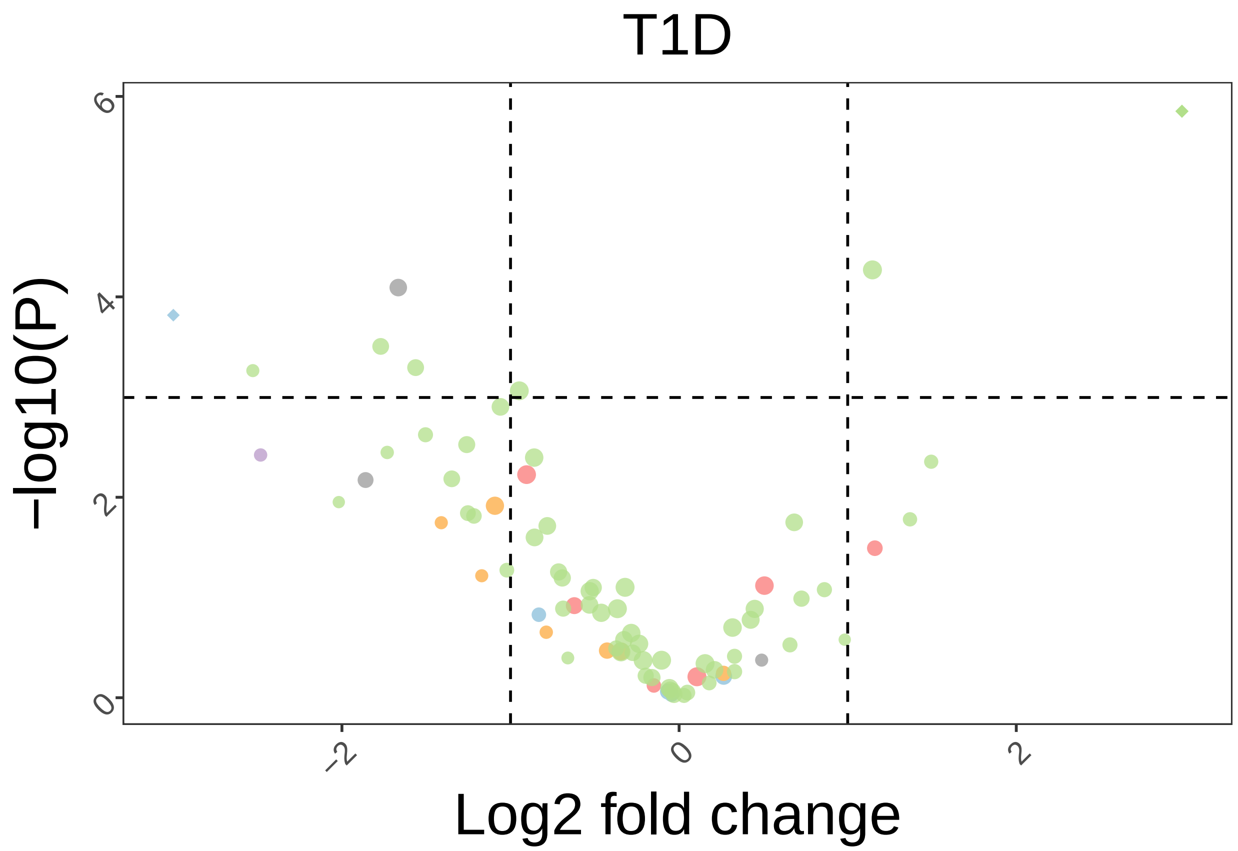


```
Fig2ListRemMet[["vulcLadaT1D"]] <- p1


p2<-ggplot() +
  geom_point(data=Featurein[which(Featurein$Phyla=="Proteobacteria" &
                                  Featurein$compare=="LADA vs T2D"), ],
             aes(x=log2FoldChange, y=minuslog10, group=Genus), color="#A6CEE3", shape=16,
             size=Featurein[which(Featurein$Phyla=="Proteobacteria" &
                                  Featurein$compare=="LADA vs T2D"), ]
             [,ncol(Featurein)]/40+0.5) +
  #did not add jitter to the first on purpose. Sizes are scaled from percent
  #to the range 0.5-3
  geom_point(data=Featureout[which(Featureout$Phyla=="Proteobacteria" &
                                   Featureout$compare=="LADA vs T2D"), ],
             aes(x=log2FoldChange, y=minuslog10, group=Genus), color="#A6CEE3", shape=18,
```

```
           size=Featureout[which(Featureout$Phyla=="Proteobacteria" &
                                    Featureout$compare=="LADA vs T2D"), ]
           [,ncol(Featureout)]/40+0.5) +
geom_point(data=Featurein[which(Featurein$Phyla=="Bacteroidetes" &
                                   Featurein$compare=="LADA vs T2D"), ],
           aes(x=log2FoldChange, y=minuslog10, group=Genus), color="#FB9A99", shape=16,
           size=Featurein[which(Featurein$Phyla=="Bacteroidetes" &
                                   Featurein$compare=="LADA vs T2D"), ]
           [,ncol(Featurein)]/40+0.5) +
geom_jitter(data=Featureout[which(Featureout$Phyla=="Bacteroidetes" &
                                     Featureout$compare=="LADA vs T2D"), ],
           aes(x=log2FoldChange, y=minuslog10, group=Genus), color="#FB9A99", shape=18,
           size=Featureout[which(Featureout$Phyla=="Bacteroidetes" &
                                    Featureout$compare=="LADA vs T2D"), ]
           [,ncol(Featureout)]/40+0.5,
           width = 0.1, height = 0.1) +
geom_point(data=Featurein[which(Featurein$Phyla=="Actinobacteria" &
                                   Featurein$compare=="LADA vs T2D"), ],
           aes(x=log2FoldChange, y=minuslog10, group=Genus), color="#FDBF6F", shape=16,
           size=Featurein[which(Featurein$Phyla=="Actinobacteria" &
                                   Featurein$compare=="LADA vs T2D"), ]
           [,ncol(Featurein)]/40+0.5) +
geom_jitter(data=Featureout[which(Featureout$Phyla=="Actinobacteria" &
                                     Featureout$compare=="LADA vs T2D"), ],
           aes(x=log2FoldChange, y=minuslog10, group=Genus), color="#FDBF6F", shape=18,
           size=Featureout[which(Featureout$Phyla=="Actinobacteria" &
                                    Featureout$compare=="LADA vs T2D"), ]
           [,ncol(Featureout)]/40+0.5, width = 0.1, height = 0.1) +
geom_point(data=Featurein[which(Featurein$Phyla=="Firmicutes" &
                                   Featurein$compare=="LADA vs T2D"), ],
           aes(x=log2FoldChange, y=minuslog10, group=Genus),
           color="#B2DF8A", shape=16, alpha=0.75,
           size=Featurein[which(Featurein$Phyla=="Firmicutes" &
                                   Featurein$compare=="LADA vs T2D"), ]
           [,ncol(Featurein)]/40+0.5) +
geom_jitter(data=Featureout[which(Featureout$Phyla=="Firmicutes" &
                                     Featureout$compare=="LADA vs T2D"), ],
           aes(x=log2FoldChange, y=minuslog10, group=Genus),
           color="#B2DF8A", shape=18,
           size=Featureout[which(Featureout$Phyla=="Firmicutes" &
                                    Featureout$compare=="LADA vs T2D"), ]
           [,ncol(Featureout)]/40+0.5, width = 0.1, height = 0.1) +
geom_point(data=Featurein[which(Featurein$Phyla=="Euryarchaeota" &
                                   Featurein$compare=="LADA vs T2D"), ],
           aes(x=log2FoldChange, y=minuslog10, group=Genus), color="#CAB2D6", shape=16,
           size=Featurein[which(Featurein$Phyla=="Euryarchaeota" &
                                   Featurein$compare=="LADA vs T2D"), ]
           [,ncol(Featurein)]/40+0.5) +
geom_jitter(data=Featureout[which(Featureout$Phyla=="Euryarchaeota" &
                                     Featureout$compare=="LADA vs T2D"), ],
           aes(x=log2FoldChange, y=minuslog10, group=Genus), color="#CAB2D6", shape=18,
           size=Featureout[which(Featureout$Phyla=="Euryarchaeota" &
                                    Featureout$compare=="LADA vs T2D"), ]
```

```
             [,ncol(Featureout)]/40+0.5, width = 0.1, height = 0.1) +
  geom_point(data=Featurein[which(Featurein$Phyla=="Other" &
                                  Featurein$compare=="LADA vs T2D"), ],
             aes(x=log2FoldChange, y=minuslog10, group=Genus), color="#B3B3B3", shape=16,
             size=Featurein[which(Featurein$Phyla=="Other" &
                                  Featurein$compare=="LADA vs T2D"), ]
             [,ncol(Featurein)]/40+0.5) +
  geom_jitter(data=Featureout[which(Featureout$Phyla=="Other" &
                                    Featureout$compare=="LADA vs T2D"), ],
             aes(x=log2FoldChange, y=minuslog10, group=Genus), color="#B3B3B3", shape=18,
             size=Featureout[which(Featureout$Phyla=="Other" &
                                   Featureout$compare=="LADA vs T2D"), ]
             [,ncol(Featureout)]/40+0.5, width = 0.1, height = 0.1) +
  geom_text(data=Featurein[which(Featurein$padj<padj_threshold &
                                 Featurein$compare=="LADA vs T2D"), ],
             aes(x=log2FoldChange, y=minuslog10, group=Genus, label=Genus),
            nudge_y = 0.2, size=2.5) +
  geom_text(data=Featureout[which(Featureout$padj<padj_threshold &
                                  Featureout$compare=="LADA vs T2D"), ],
             aes(x=log2FoldChange, y=minuslog10, group=Genus, label=Genus),
            nudge_x=0.6, nudge_y = -0.2, size=2.5) +
#  geom_text(data=Featureout[which(Featureout$padj<padj_threshold &
#                                  Featureout$compare=="LADA vs T1D" &
#                                  Featureout$Genus=="Rikenellaceae_RC9_gut_group"), ],
#              aes(x=log2FoldChange, y=minuslog10, group=Genus, label=Genus),
#             nudge_x=1.22, nudge_y = 0) +
  geom_hline(yintercept = -log(0.05), linetype="dashed") +
  geom_hline(yintercept = -log(BHLadaT2D), linetype="dashed", alpha=0.1) +
  geom_vline(xintercept = -1, linetype="dashed") +
  geom_vline(xintercept = 1, linetype="dashed") +
  theme_bw() +
  ggtitle("T2D") +
  xlab("Log2 fold change") +
  ylab("-log10(P)") +
  theme(panel.grid.major = element_blank(),
        panel.grid.minor = element_blank(),
        axis.title=element_text(size=22),
        legend.position="bottom",
        legend.title=element_text(size=20),
        legend.text=element_text(size=20),
        axis.text.x = element_text(angle = 45, hjust = 1, size=12),
        axis.text.y = element_text(angle = 45, hjust = 1, size=12),
        plot.title = element_text(size = 22, hjust=0.5))
#ggplotly(p2)
p2
```

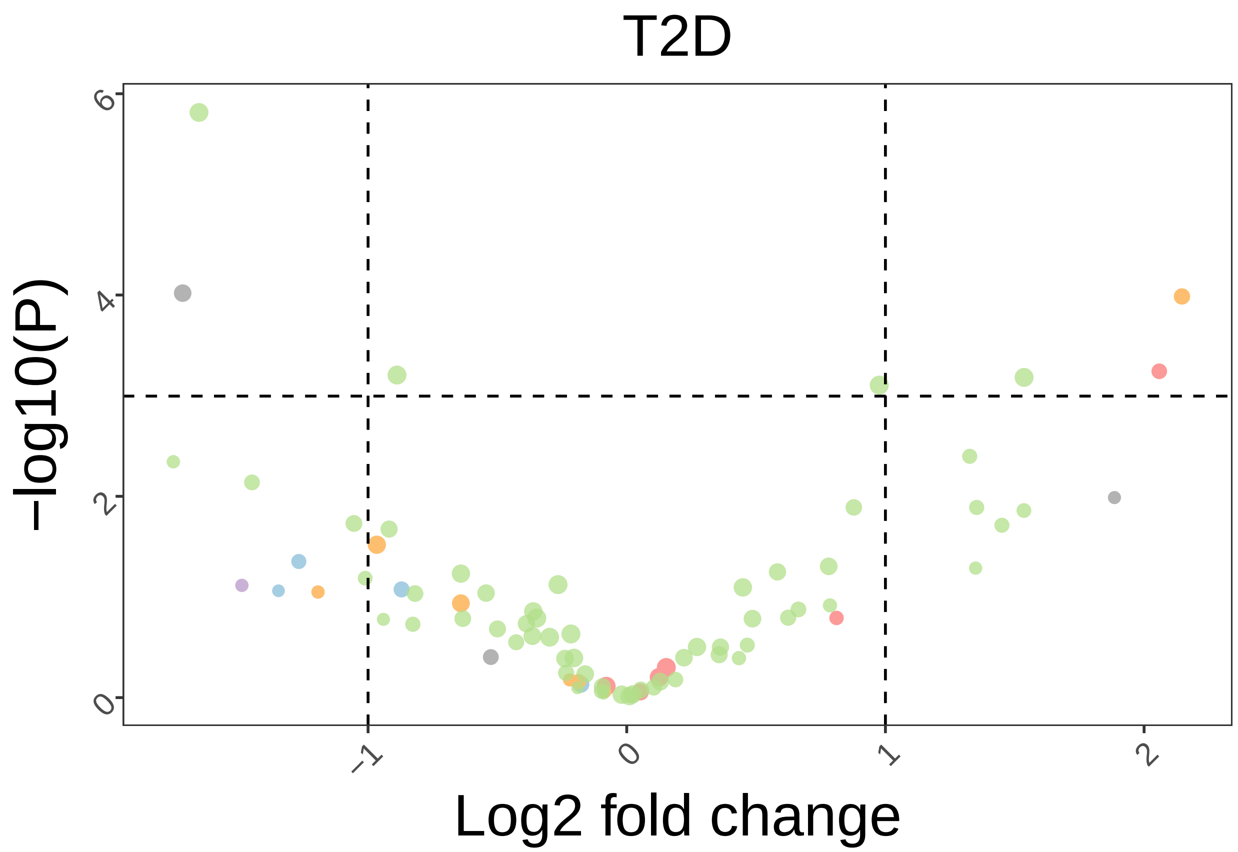


```
Fig2ListRemMet[["vulcLadaT2D"]] <- p2


p3<-ggplot() +
  geom_point(data=Featurein[which(Featurein$Phyla=="Proteobacteria" &
                                  Featurein$compare=="LADA vs Control"), ],
             aes(x=log2FoldChange, y=minuslog10, group=Genus), color="#A6CEE3", shape=16,
             size=Featurein[which(Featurein$Phyla=="Proteobacteria" &
                                  Featurein$compare=="LADA vs Control"), ]
             [,ncol(Featurein)]/40+0.5) +
  #did not add jitter to the first on purpose. Sizes are scaled from percent to the range 0.5-3
  geom_point(data=Featureout[which(Featureout$Phyla=="Proteobacteria" &
                                   Featureout$compare=="LADA vs Control"), ],
             aes(x=log2FoldChange, y=minuslog10, group=Genus), color="#A6CEE3", shape=18,
             size=Featureout[which(Featureout$Phyla=="Proteobacteria" &
                                   Featureout$compare=="LADA vs Control"), ]
             [,ncol(Featureout)]/40+0.5) +
  geom_point(data=Featurein[which(Featurein$Phyla=="Bacteroidetes" &
                                  Featurein$compare=="LADA vs Control"), ],
             aes(x=log2FoldChange, y=minuslog10, group=Genus), color="#FB9A99", shape=16,
             size=Featurein[which(Featurein$Phyla=="Bacteroidetes" &
                                  Featurein$compare=="LADA vs Control"), ]
             [,ncol(Featurein)]/40+0.5) +
  geom_jitter(data=Featureout[which(Featureout$Phyla=="Bacteroidetes" &
                                    Featureout$compare=="LADA vs Control"), ],
             aes(x=log2FoldChange, y=minuslog10, group=Genus), color="#FB9A99", shape=18,
```

```
           size=Featureout[which(Featureout$Phyla=="Bacteroidetes" &
                                   Featureout$compare=="LADA vs Control"), ]
           [,ncol(Featureout)]/40+0.5, width = 0.1, height = 0.1) +
geom_point(data=Featurein[which(Featurein$Phyla=="Actinobacteria" &
                                  Featurein$compare=="LADA vs Control"), ],
           aes(x=log2FoldChange, y=minuslog10, group=Genus), color="#FDBF6F", shape=16,
           size=Featurein[which(Featurein$Phyla=="Actinobacteria" &
                                  Featurein$compare=="LADA vs Control"), ]
           [,ncol(Featurein)]/40+0.5) +
geom_jitter(data=Featureout[which(Featureout$Phyla=="Actinobacteria" &
                                    Featureout$compare=="LADA vs Control"), ],
           aes(x=log2FoldChange, y=minuslog10, group=Genus), color="#FDBF6F", shape=18,
           size=Featureout[which(Featureout$Phyla=="Actinobacteria" &
                                   Featureout$compare=="LADA vs Control"), ]
           [,ncol(Featureout)]/40+0.5, width = 0.1, height = 0.1) +
geom_point(data=Featurein[which(Featurein$Phyla=="Firmicutes" &
                                  Featurein$compare=="LADA vs Control"), ],
           aes(x=log2FoldChange, y=minuslog10, group=Genus),
           color="#B2DF8A", shape=16, alpha=0.75,
           size=Featurein[which(Featurein$Phyla=="Firmicutes" &
                                  Featurein$compare=="LADA vs Control"), ]
           [,ncol(Featurein)]/40+0.5) +
geom_jitter(data=Featureout[which(Featureout$Phyla=="Firmicutes" &
                                    Featureout$compare=="LADA vs Control"), ],
           aes(x=log2FoldChange, y=minuslog10, group=Genus),
           color="#B2DF8A", shape=18,
           size=Featureout[which(Featureout$Phyla=="Firmicutes" &
                                   Featureout$compare=="LADA vs Control"), ]
           [,ncol(Featureout)]/40+0.5, width = 0.1, height = 0.1) +
geom_point(data=Featurein[which(Featurein$Phyla=="Euryarchaeota" &
                                  Featurein$compare=="LADA vs Control"), ],
           aes(x=log2FoldChange, y=minuslog10, group=Genus), color="#CAB2D6", shape=16,
           size=Featurein[which(Featurein$Phyla=="Euryarchaeota" &
                                  Featurein$compare=="LADA vs Control"), ]
           [,ncol(Featurein)]/40+0.5) +
geom_jitter(data=Featureout[which(Featureout$Phyla=="Euryarchaeota" &
                                    Featureout$compare=="LADA vs Control"), ],
           aes(x=log2FoldChange, y=minuslog10, group=Genus), color="#CAB2D6", shape=18,
           size=Featureout[which(Featureout$Phyla=="Euryarchaeota" &
                                   Featureout$compare=="LADA vs Control"), ]
           [,ncol(Featureout)]/40+0.5, width = 0.1, height = 0.1) +
geom_point(data=Featurein[which(Featurein$Phyla=="Other" &
                                  Featurein$compare=="LADA vs Control"), ],
           aes(x=log2FoldChange, y=minuslog10, group=Genus), color="#B3B3B3", shape=16,
           size=Featurein[which(Featurein$Phyla=="Other" &
                                  Featurein$compare=="LADA vs Control"), ]
           [,ncol(Featurein)]/40+0.5) +
geom_jitter(data=Featureout[which(Featureout$Phyla=="Other" &
                                    Featureout$compare=="LADA vs Control"), ],
           aes(x=log2FoldChange, y=minuslog10, group=Genus), color="#B3B3B3", shape=18,
           size=Featureout[which(Featureout$Phyla=="Other" &
                                   Featureout$compare=="LADA vs Control"), ]
           [,ncol(Featureout)]/40+0.5, width = 0.1, height = 0.1) +
```

```r
  geom_text(data=Featurein[which(Featurein$padj<padj_threshold &
                                  Featurein$compare=="LADA vs Control"), ],
            aes(x=log2FoldChange, y=minuslog10, group=Genus, label=Genus),
           nudge_y = 0.2, size=2.5) +
  geom_text(data=Featureout[which(Featureout$padj<padj_threshold &
                                   Featureout$compare=="LADA vs Control"), ],
            aes(x=log2FoldChange, y=minuslog10, group=Genus, label=Genus),
           nudge_x=0.6, nudge_y = -0.2, size=2.5) +
#  geom_text(data=Featureout[which(Featureout$padj<padj_threshold &
#                                   Featureout$compare=="LADA vs T1D" &
#                                   Featureout$Genus=="Rikenellaceae_RC9_gut_group"), ],
#             aes(x=log2FoldChange, y=minuslog10, group=Genus, label=Genus),
#            nudge_x=1.22, nudge_y = 0) +
  geom_hline(yintercept = -log(0.05), linetype="dashed") +
  geom_hline(yintercept = -log(BHLadaCon), linetype="dashed", alpha=0.1) +
  geom_vline(xintercept = -1, linetype="dashed") +
  geom_vline(xintercept = 1, linetype="dashed") +
  theme_bw() +
  ggtitle("Controls") +
  xlab("Log2 fold change") +
  ylab("-log10(P)") +
  theme(panel.grid.major = element_blank(),
        panel.grid.minor = element_blank(),
        axis.title=element_text(size=22),
        legend.position="bottom",
        legend.title=element_text(size=20),
        legend.text=element_text(size=20),
        axis.text.x = element_text(angle = 45, hjust = 1, size=12),
        axis.text.y = element_text(angle = 45, hjust = 1, size=12),
        plot.title = element_text(size = 22, hjust=0.5))
#ggplotly(p3)
p3
```

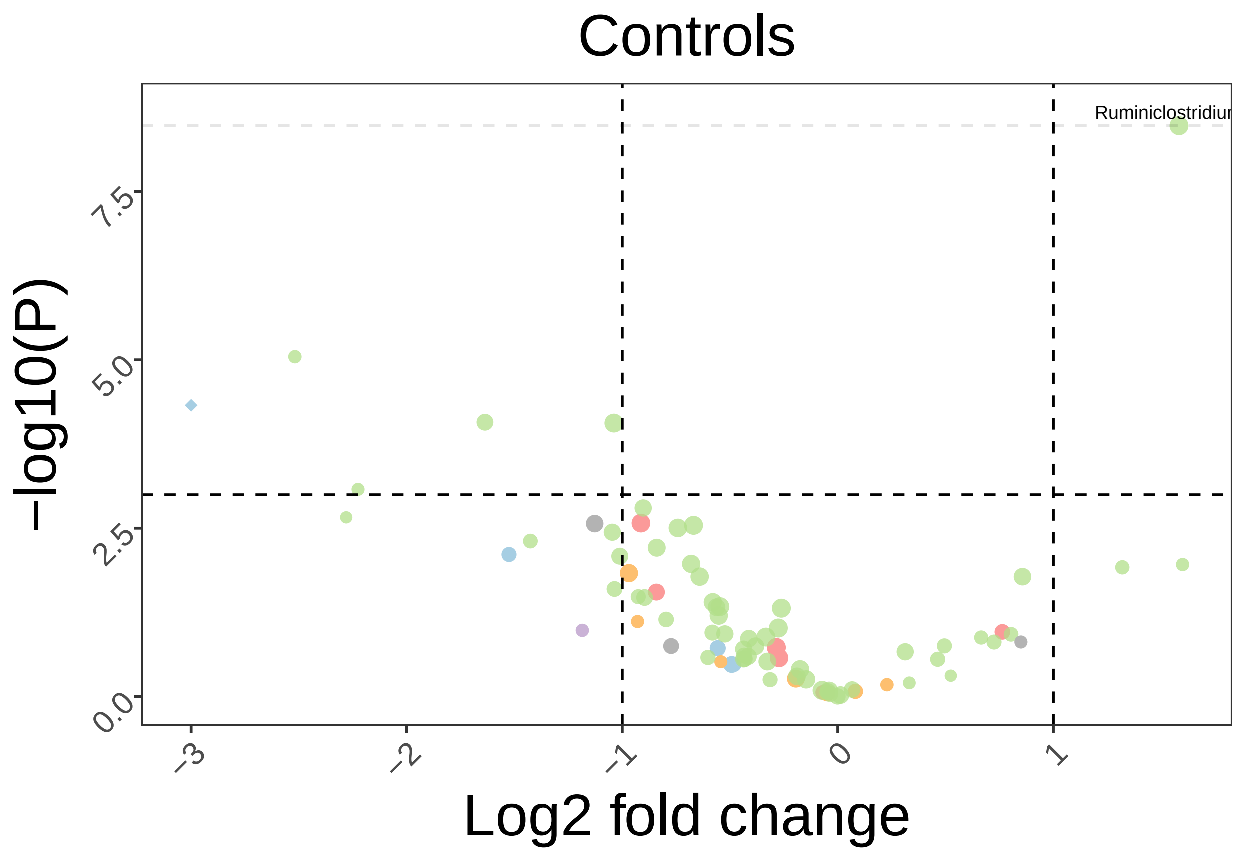


```
Fig2ListRemMet[["vulcLadaControl"]] <- p3

lay <- rbind(c(1,2,3))
pdf(paste("MicroLADA_VulcanoRemMet.pdf", sep=""), width=15, height=5)
grid.arrange(p1,p2,p3,layout_matrix = lay)
dev.off()
```

pdf 2

```
##Boxplot with stats intertwined
#Stats have to be in the end of the chunk don't ask me why
#Have to use the same naming in res_stat to output tables
res_stat$Genus<-str_replace_all(res_stat$Genus, " ","_")
res_stat$Genus<-str_replace_all(res_stat$Genus, "/","_")
res_stat$Genus<-gsub("\\[|\\]", "", res_stat$Genus)
res_stat$Genus<-str_replace_all(res_stat$Genus, ":","_")
res_stat$Genus<-str_replace_all(res_stat$Genus, "-","_")

#Order Diagnosis
Plotting2$Diagnosis<-ordered(Plotting2$Diagnosis,
                             levels=c("Control", "T1D", "LADA", "T2D"))

for (i in SelOrgs) {
Boxplot <-
ggplot(Plotting2, aes_string(x="Diagnosis", y=i, group="Diagnosis")) +
```

```
    geom_boxplot() +
#    geom_boxplot(aes(fill=Diagnosis, trim=FALSE)) +
#    geom_jitter() +
    geom_jitter(aes(color=Diagnosis), size=2) +
    stat_summary(fun.data="mean_sdl",
                 mult=1, #mean plus minus a constant (mult=1) times the st.dev
                 geom="pointrange",
                 width=0.2 ) +
    #stat_summary(fun.y = mean, geom = "point") +
    #facet_grid(. ~ Metformin, scales="fixed") + #Scales can also be "free"
    ggtitle(i) +
    #xlab("Diagnosis") +
    ylab("Cells pr. g feces") +
    scale_fill_manual(values=c(Control = "#666666", T1D = "#1F78B4",
                               T2D = "#33A02C", LADA = "#E31A1C")) +
    scale_color_manual(values=c(Control = "#666666", T1D = "#1F78B4",
                               T2D = "#33A02C", LADA = "#E31A1C")) +
    theme_bw() +
    theme(legend.position="none",
          title =element_text(size=18, face='bold'),
          panel.grid.major = element_blank(),
          panel.grid.minor = element_blank(),
          axis.title=element_text(size=16),
          axis.title.x = element_blank(),
          axis.text.x = element_text(angle = 45, hjust = 1, size=14),
          axis.text.y = element_text(angle = 45, hjust = 1, size=12))
Fig2ListRemMet[[i]] <- Boxplot
#print(kable(stat_sig[which(stat_sig$gene==i), c(5,6,7,10)]))
cat("\n")
tabling<-res_stat[which(res_stat$Genus==i), c(5,6,7,10)]
print(kable(tabling))
#print(tabling)
cat("\n")
print(Boxplot)
}
```

	pvalue	padj	Genus	compare
211	0.0002082	0.0168647	Ruminiclostridium_5	LADA vs Control
130	0.0645651	0.3639375	Ruminiclostridium_5	Control vs T2D
373	0.0139925	0.4424407	Ruminiclostridium_5	LADA vs T1D
454	0.0448406	0.5188697	Ruminiclostridium_5	LADA vs T2D
49	0.1434284	0.7164741	Ruminiclostridium_5	Control vs T1D
292	0.6521545	0.8253830	Ruminiclostridium_5	T1D vs T2D

Ruminiclostridium_5

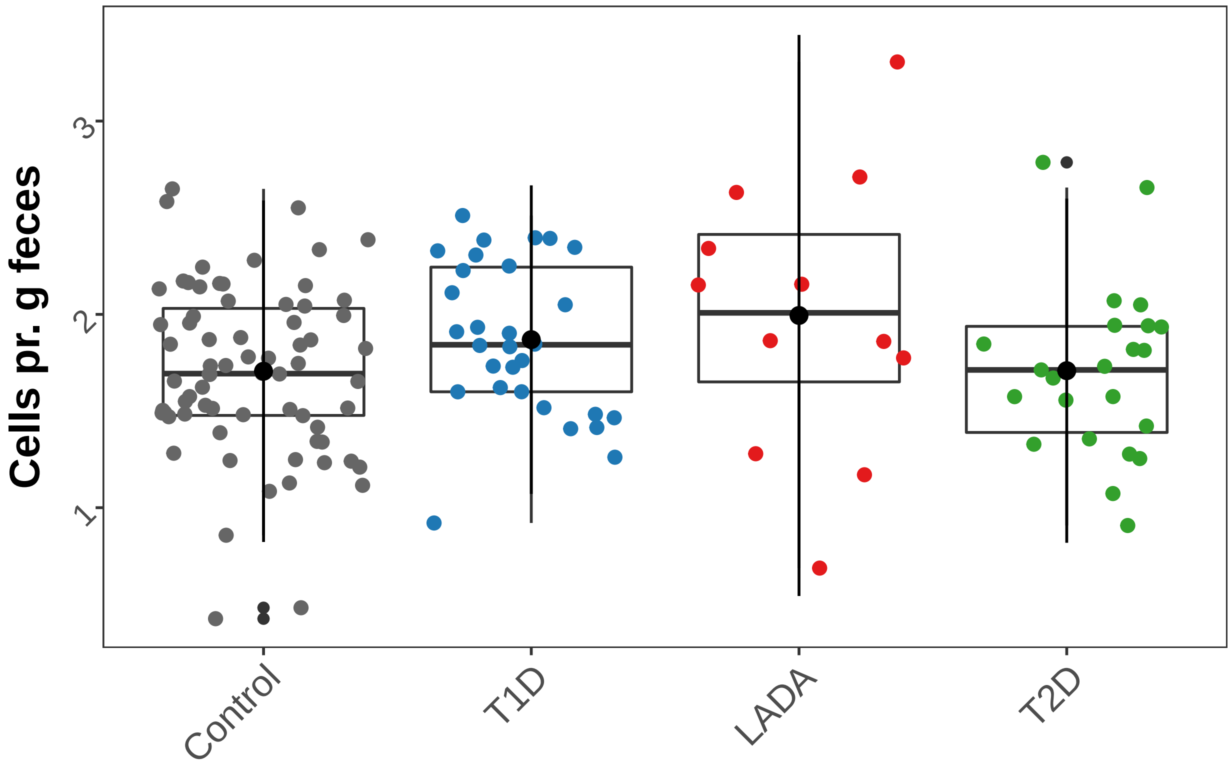

	pvalue	padj	Genus	compare
44	0.0003766	0.0229456	Faecalibacterium	Control vs T1D
125	0.1010235	0.4306792	Faecalibacterium	Control vs T2D
287	0.1682765	0.4396901	Faecalibacterium	T1D vs T2D
206	0.0817890	0.5431438	Faecalibacterium	LADA vs Control
368	0.4965938	0.8558319	Faecalibacterium	LADA vs T1D
449	0.6743478	0.9476449	Faecalibacterium	LADA vs T2D

Faecalibacterium

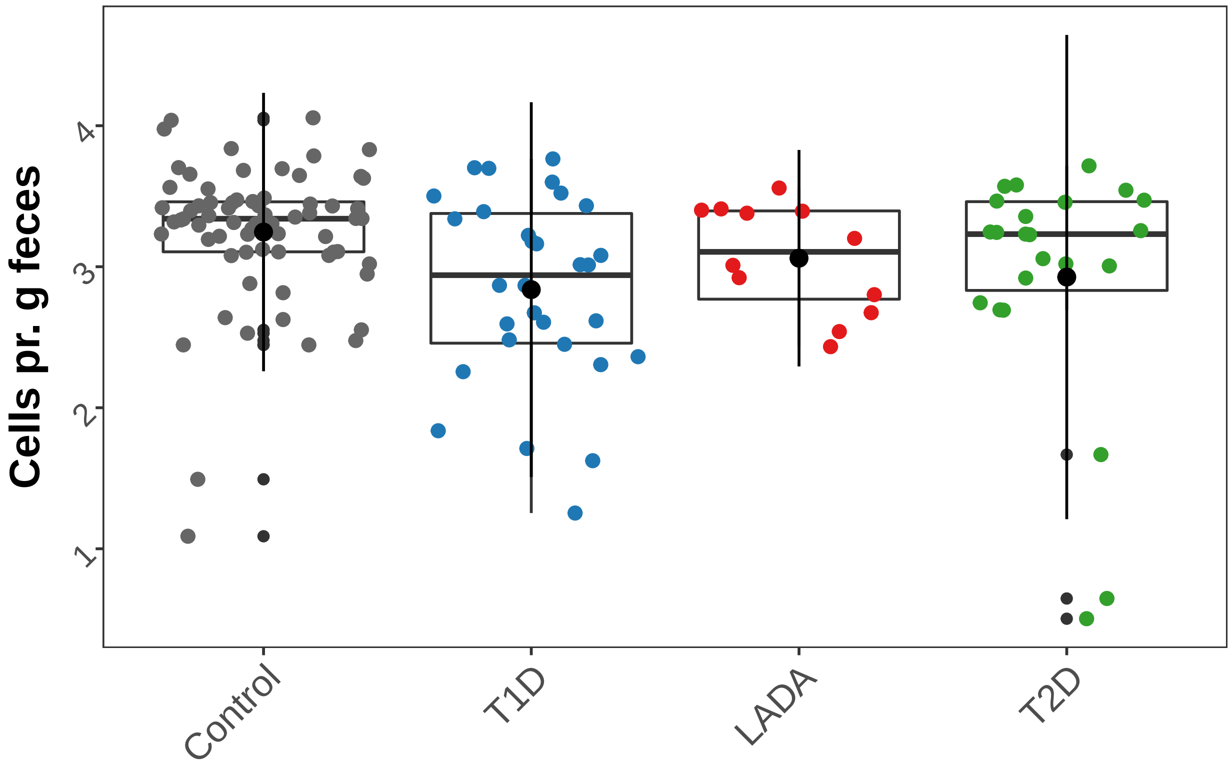

	pvalue	padj	Genus	compare
39	0.0005666	0.0229456	Roseburia	Control vs T1D
282	0.0903121	0.3670206	Roseburia	T1D vs T2D
120	0.2392542	0.5699879	Roseburia	Control vs T2D
201	0.1096977	0.5923676	Roseburia	LADA vs Control
363	0.4597352	0.8095337	Roseburia	LADA vs T1D
444	0.5424231	0.9258315	Roseburia	LADA vs T2D

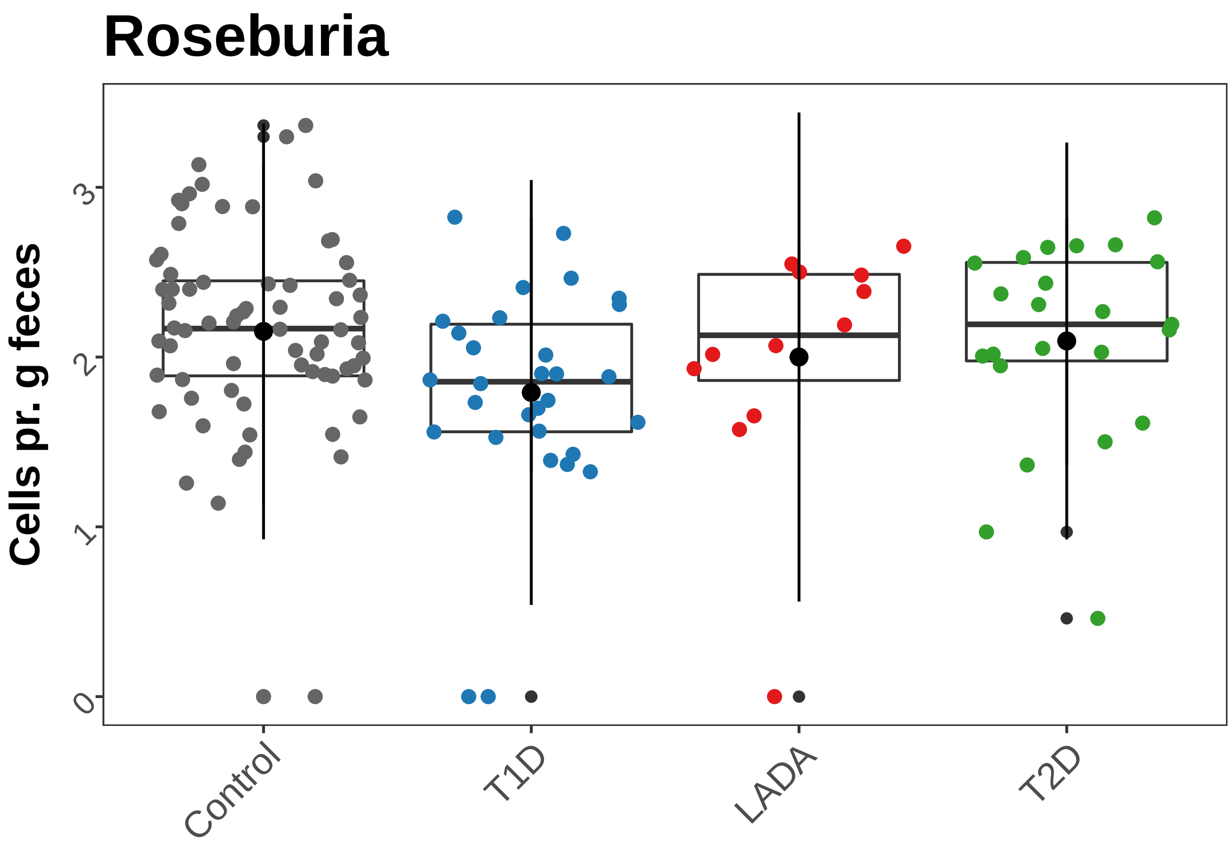

	pvalue	padj	Genus	compare
251	0.0002881	0.0233365	Family.Eggerthellaceae.	T1D vs T2D
89	0.0003640	0.0294830	Family.Eggerthellaceae.	Control vs T2D
413	0.0185763	0.5015598	Family.Eggerthellaceae.	LADA vs T2D
8	0.4977436	0.8157710	Family.Eggerthellaceae.	Control vs T1D
332	0.6250607	0.8863868	Family.Eggerthellaceae.	LADA vs T1D
170	0.9516212	0.9859781	Family.Eggerthellaceae.	LADA vs Control

Family.Eggerthellaceae.

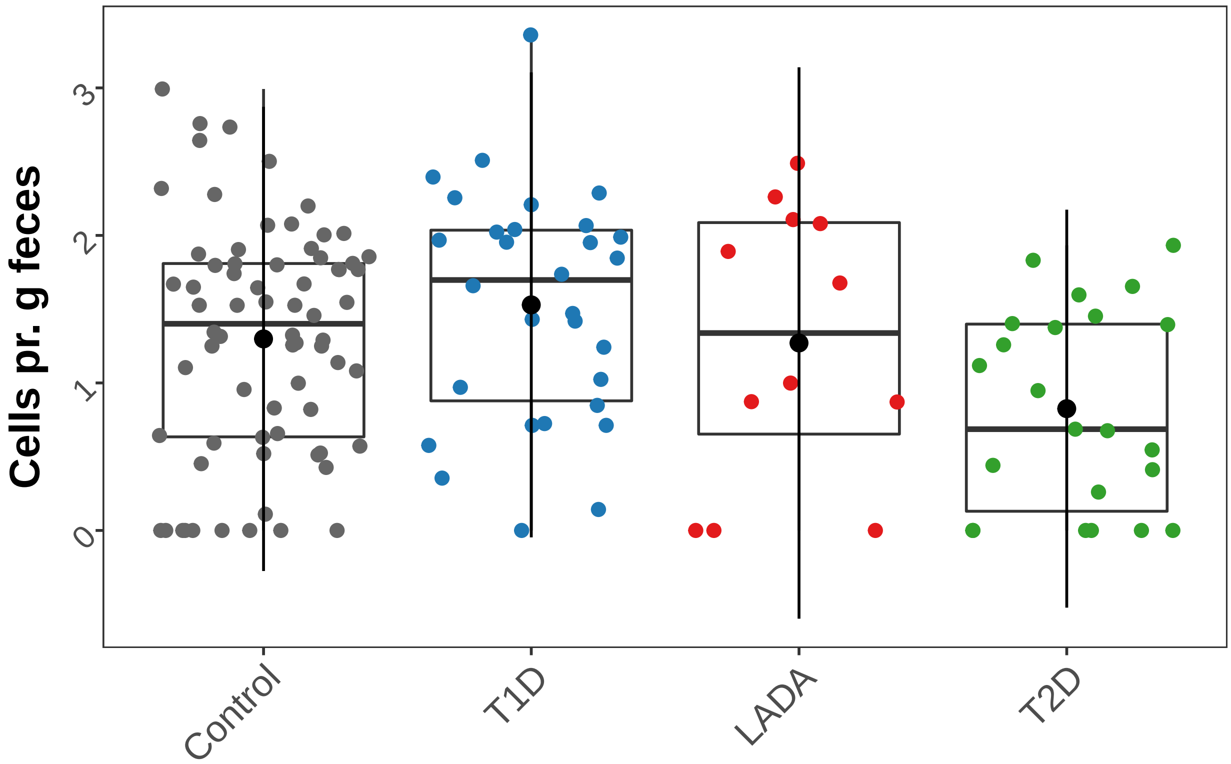

	pvalue	padj	Genus	compare
274	0.0008905	0.0360666	Lachnoclostridium	T1D vs T2D
112	0.0032266	0.0871181	Lachnoclostridium	Control vs T2D
436	0.0029853	0.2418090	Lachnoclostridium	LADA vs T2D
193	0.2631768	0.7036590	Lachnoclostridium	LADA vs Control
31	0.3288067	0.7565178	Lachnoclostridium	Control vs T1D
355	0.6894119	0.8863868	Lachnoclostridium	LADA vs T1D

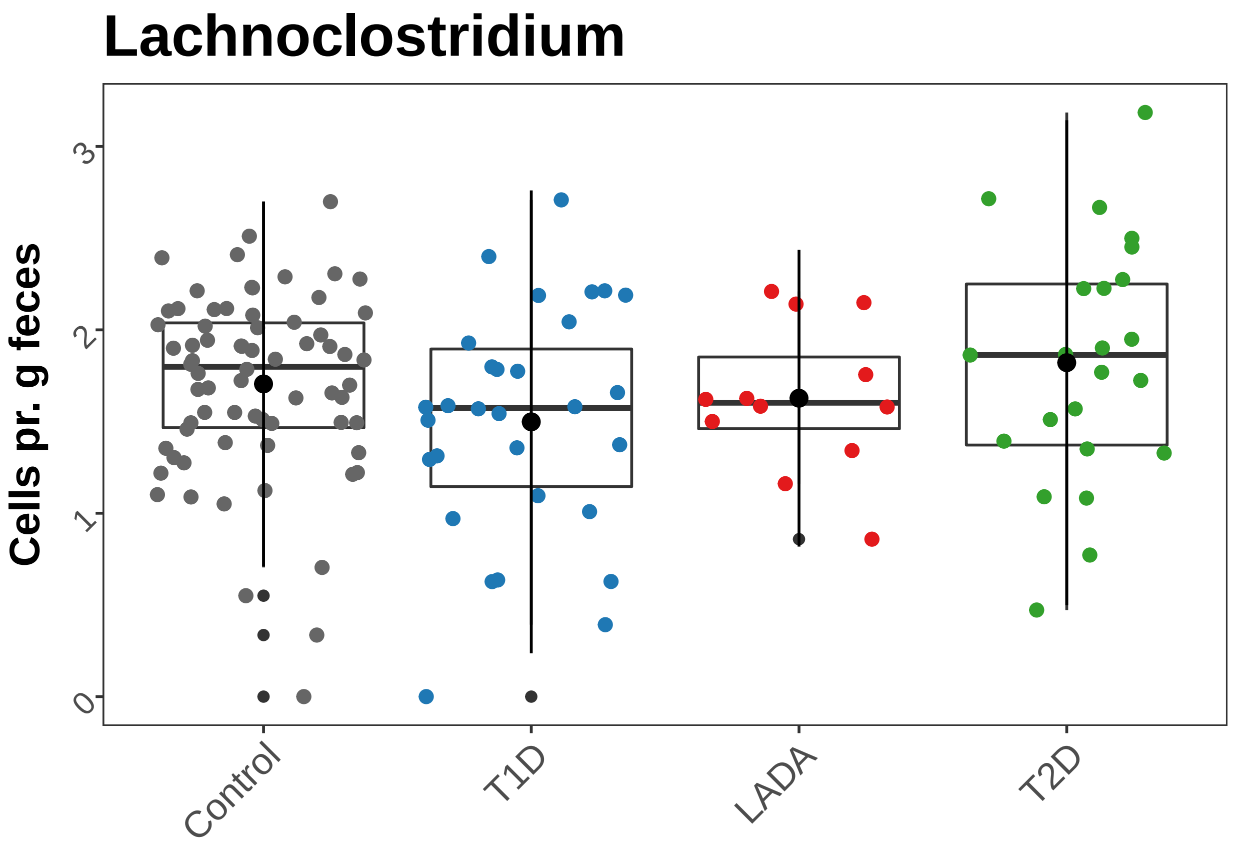

	pvalue	padj	Genus	compare
259	0.0013428	0.0362547	Streptococcus	T1D vs T2D
97	0.0015612	0.0632280	Streptococcus	Control vs T2D
421	0.0415266	0.5188697	Streptococcus	LADA vs T2D
16	0.5560186	0.8497642	Streptococcus	Control vs T1D
340	0.6332724	0.8863868	Streptococcus	LADA vs T1D
178	0.9121030	0.9859781	Streptococcus	LADA vs Control

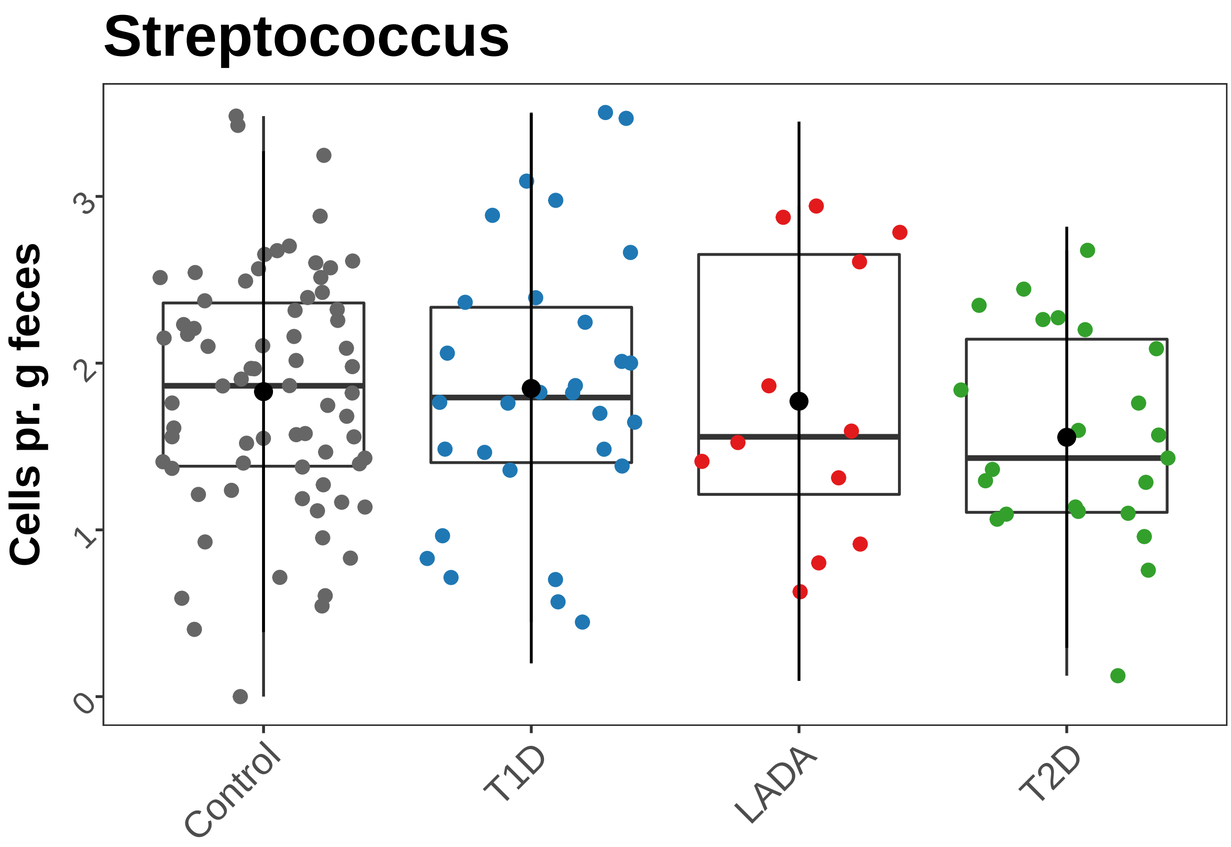

	pvalue	padj	Genus	compare
286	0.0020208	0.0409217	Butyricicoccus	T1D vs T2D
43	0.0019618	0.0529689	Butyricicoccus	Control vs T1D
448	0.0405686	0.5188697	Butyricicoccus	LADA vs T2D
205	0.0787013	0.5431438	Butyricicoccus	LADA vs Control
124	0.4541684	0.7827157	Butyricicoccus	Control vs T2D
367	0.7114996	0.9004917	Butyricicoccus	LADA vs T1D

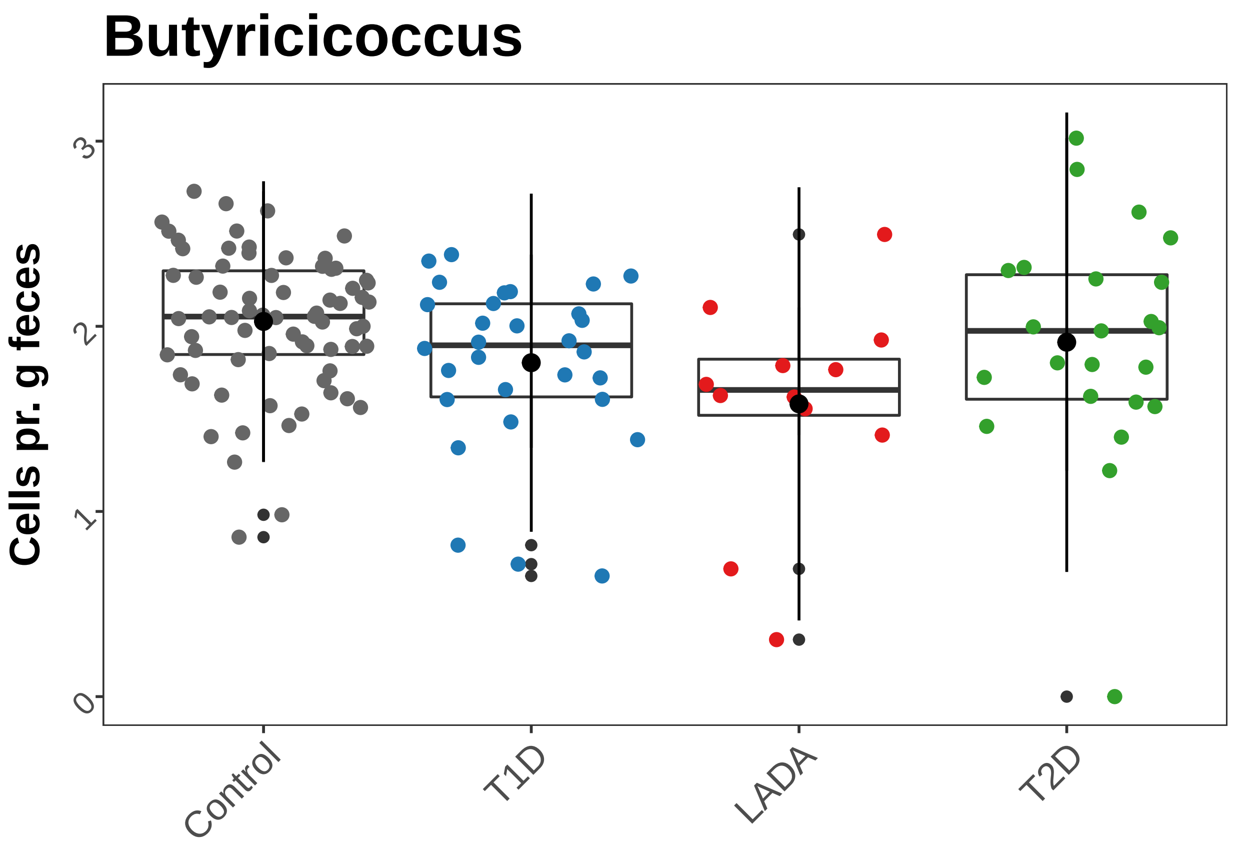

	pvalue	padj	Genus	compare
293	0.0037534	0.0608047	Ruminiclostridium_6	T1D vs T2D
131	0.0182794	0.2467725	Ruminiclostridium_6	Control vs T2D
374	0.0725195	0.5124954	Ruminiclostridium_6	LADA vs T1D
212	0.2270654	0.6895088	Ruminiclostridium_6	LADA vs Control
50	0.2794684	0.7452221	Ruminiclostridium_6	Control vs T1D
455	0.5937090	0.9430646	Ruminiclostridium_6	LADA vs T2D

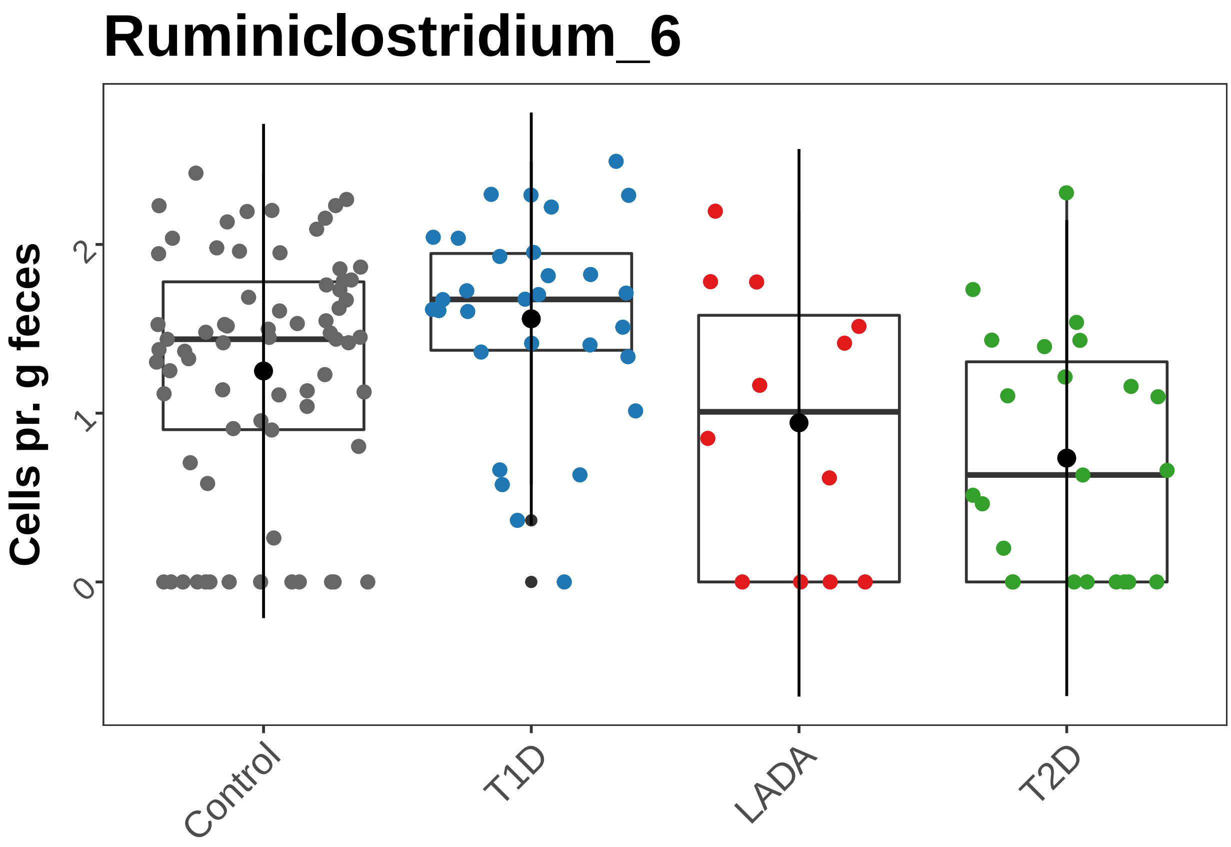

	pvalue	padj	Genus	compare
21	0.0032414	0.0656392	Agathobacter	Control vs T1D
102	0.1166599	0.4324500	Agathobacter	Control vs T2D
264	0.3383977	0.5710462	Agathobacter	T1D vs T2D
183	0.2462135	0.7036590	Agathobacter	LADA vs Control
345	0.4121693	0.7764118	Agathobacter	LADA vs T1D
426	0.9668870	0.9907084	Agathobacter	LADA vs T2D

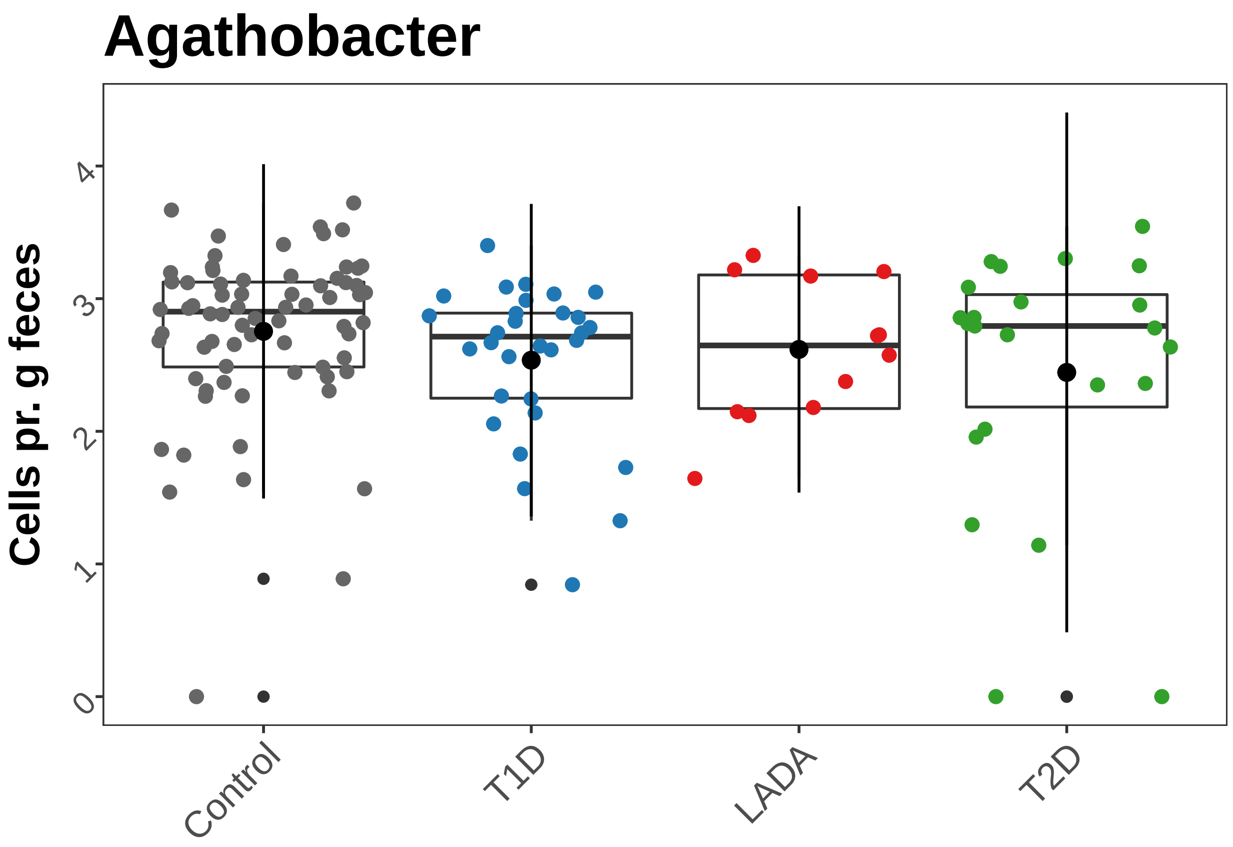

	pvalue	padj	Genus	compare
184	0.0172346	0.2792008	Anaerostipes	LADA vs Control
103	0.0272824	0.3156961	Anaerostipes	Control vs T2D
265	0.0929974	0.3670206	Anaerostipes	T1D vs T2D
346	0.0467761	0.4736077	Anaerostipes	LADA vs T1D
22	0.7657369	0.8888340	Anaerostipes	Control vs T1D
427	0.5486409	0.9258315	Anaerostipes	LADA vs T2D

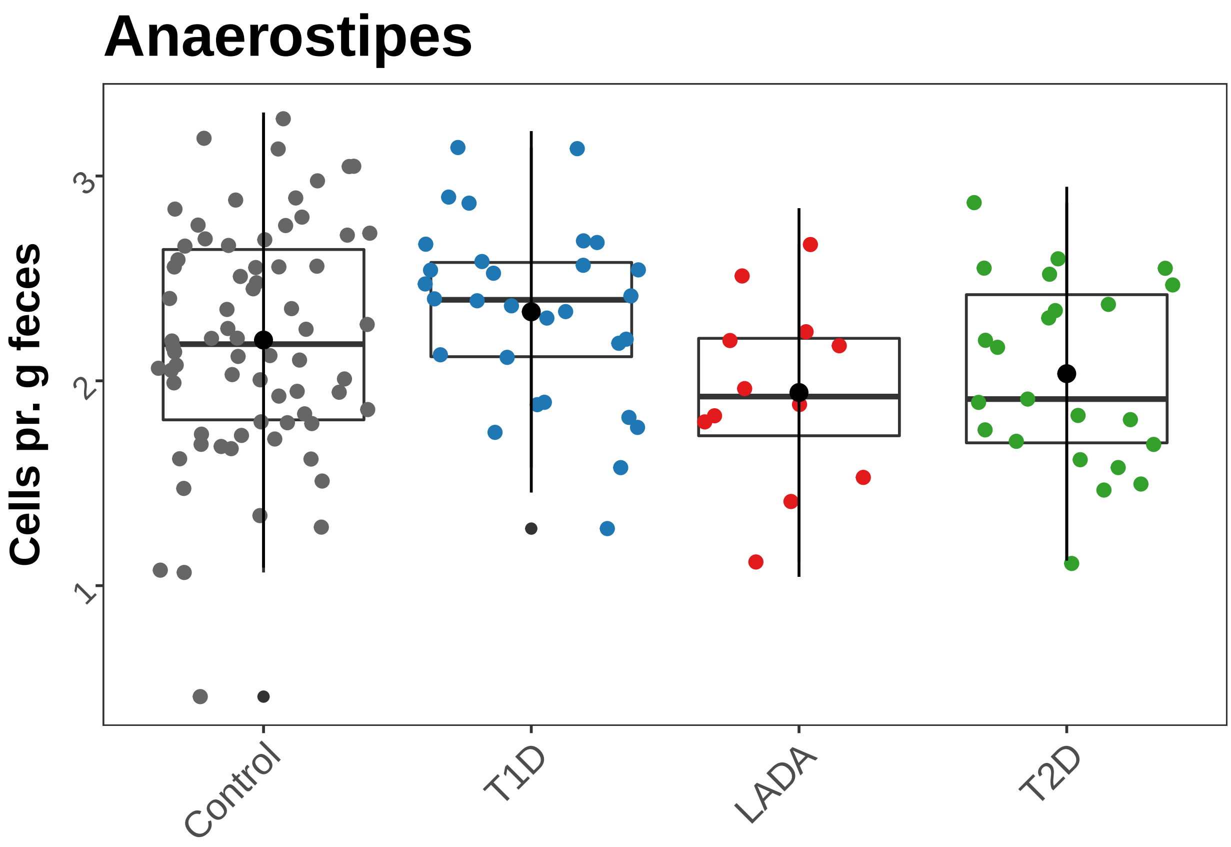

	pvalue	padj	Genus	compare
326	0.1472882	0.5612109	Bifidobacterium	LADA vs T1D
164	0.1600587	0.5939870	Bifidobacterium	LADA vs Control
407	0.2189681	0.8863523	Bifidobacterium	LADA vs T2D
2	0.7973036	0.9018955	Bifidobacterium	Control vs T1D
245	0.8363869	0.9155046	Bifidobacterium	T1D vs T2D
83	0.9960518	0.9960518	Bifidobacterium	Control vs T2D

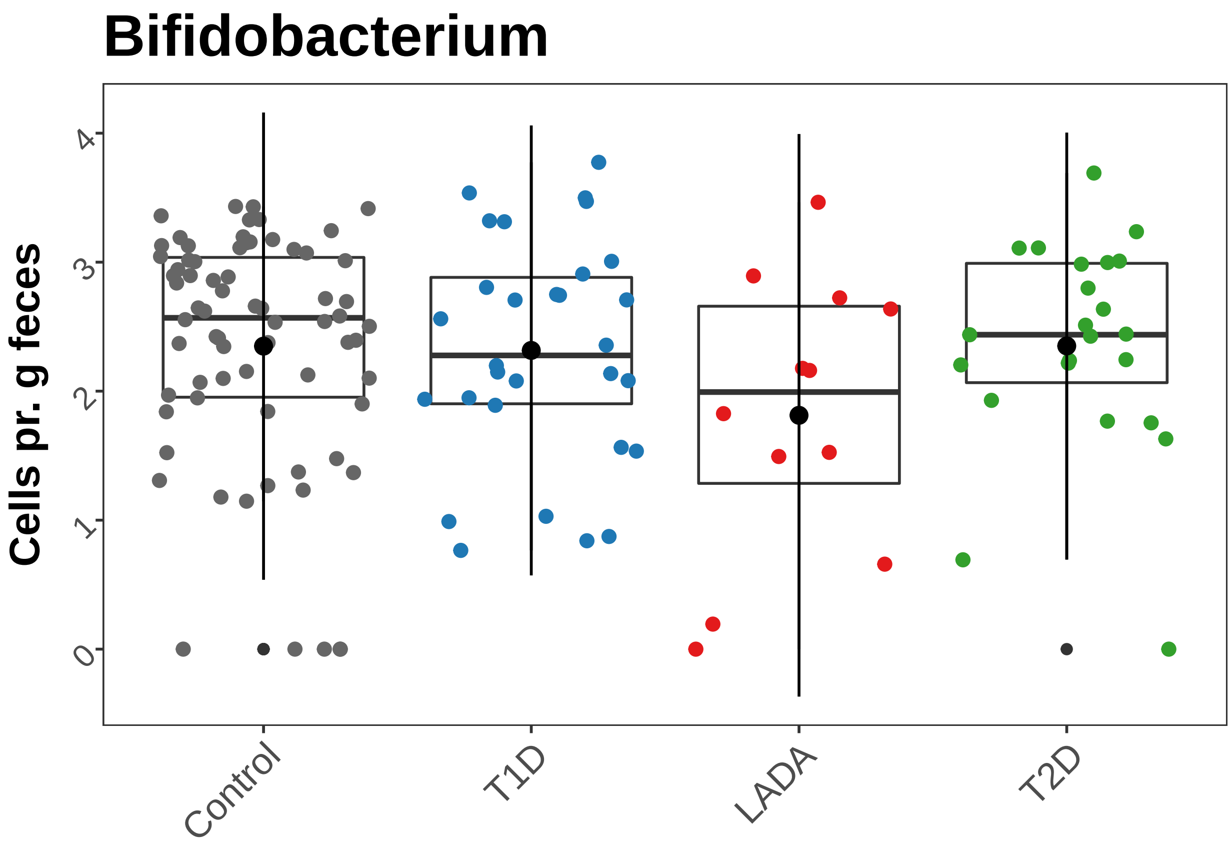

	pvalue	padj	Genus	compare
323	0.1621756	0.4378741	Akkermansia	T1D vs T2D
404	0.1139397	0.5127287	Akkermansia	LADA vs T1D
80	0.1482708	0.7164741	Akkermansia	Control vs T1D
242	0.4725144	0.7861343	Akkermansia	LADA vs Control
161	0.7647309	0.8675353	Akkermansia	Control vs T2D
485	0.6684168	0.9476449	Akkermansia	LADA vs T2D

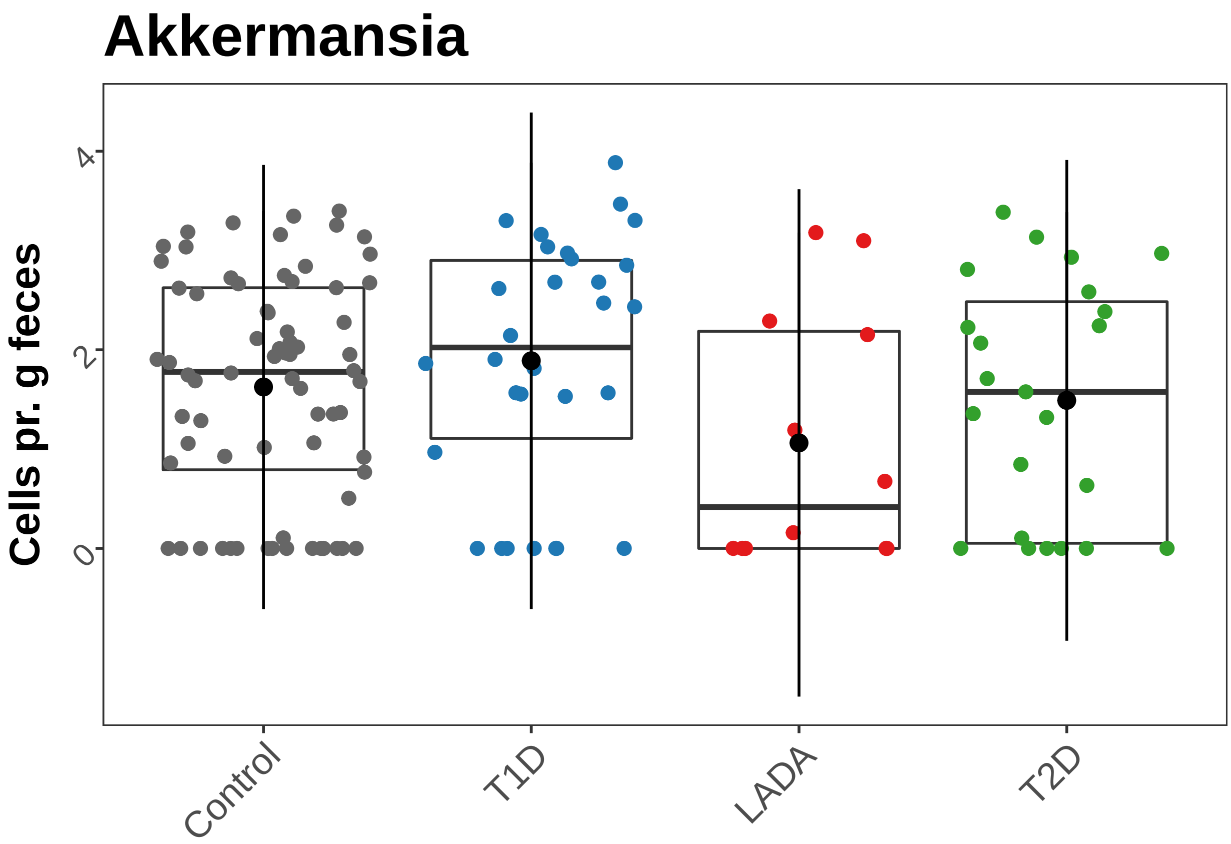


```
lay <- rbind(c(1,2,3))
pdf(paste("MicroLADA_GeneraDARemMet.pdf", sep=""), width=15, height=5)
grid.arrange(Fig2ListRemMet$Faecalibacterium,
             Fig2ListRemMet$Roseburia,
             Fig2ListRemMet$Butyricicoccus,layout_matrix = lay)
dev.off()
```

pdf 2

```
#ggplot(Plotting2, aes(x=BMI, y=Actinomyces)) +
#   geom_point()
```

Create figure 2

Investigating grouping diagnosis. Which group does LADA resemble the most and which are different from each other.
LADA is the reference in the vulcano plots.

```
#Have the plots stored in list
lay <- rbind(c(1,2,3),
             c(4,5,6))


pdf(paste("MicroLADA_Figure2.pdf", sep=""), width=15, height=10)
```

```
grid.arrange(Fig2List$vulcLadaT1D,
             Fig2List$vulcLadaT2D,
             Fig2List$vulcLadaControl,
             Fig2List$Faecalibacterium,
             Fig2List$Roseburia,
             Fig2List$Butyricicoccus,layout_matrix = lay)
dev.off()
```

```
## pdf
##   2
```

```
pdf(paste("MicroLADA_Figure2RemMet.pdf", sep=""), width=15, height=10)
grid.arrange(Fig2ListRemMet$vulcLadaT1D,
             Fig2ListRemMet$vulcLadaT2D,
             Fig2ListRemMet$vulcLadaControl,
             Fig2ListRemMet$Faecalibacterium,
             Fig2ListRemMet$Roseburia,
             Fig2ListRemMet$Butyricicoccus,layout_matrix = lay)
dev.off()
```

```
## pdf
##   2
```

DESeq heatmap

Figure 3
Many models to be included so all validation plots are removed. Includes both LRT and Wald
Structured to be compatible with creating af heatmap. Includes a dataframe of log2-fold changes a dataframe of padj and afterwards creating a dataframe containing symbols for significance levels. Density plot based on the log2-fold changes.
The first group is the reference for the DESeq tests depicted in the heatmaps

```
rm(list=setdiff(ls(), c("Metadata", "MetadataMed", "Feature", "TaxonomyDA", "Taxonomy")))

#Reassign names
Metadata2<-Metadata
Taxonomy2<-TaxonomyDA

#The factor can not be ordered for DESeq to run
Metadata2$Diagnosis<-factor(Metadata2$Diagnosis, ordered=FALSE)
##Other
#Metadata2$BMIq<-factor(Metadata2$BMIq)


##LRT none
#Create design formula
design <- formula(paste("~ ", "Diagnosis"))
#Create DESeq2 object with matrix and metadata
dds <- DESeqDataSetFromMatrix(countData = Taxonomy2,
                              colData = Metadata2,
                              design = design)
```

```r
##Assess and calculate size factors using the different methods
SF<-data.frame(Ratio=sizeFactors(estimateSizeFactors(dds, type="ratio")),
               Poscounts=sizeFactors(estimateSizeFactors(dds, type="poscounts")),
               Iterate=sizeFactors(estimateSizeFactors(dds, type="poscounts")),
               #"iterate" takes a lot of time changed to "poscounts" but kept due to the
               #following code
               Relative=colSums(Taxonomy2)/mean(colSums(Taxonomy2))
               )
SF<-add_rownames(SF, var="MicrobiomeID")
#Adding total abundances
path <- paste("J:/",
              "CBMR/",
              "SUN-CBMR-Hansen-Group/",
              "Projects/",
              "LADA/",
              "LADA_Sandra_Evelina/",
              "LADA_JKV/",
              "LADA_R_AfterFlow_Analysis_FinalCounts/",
              "LADA_FinalCounts/",
              "2019-09-03_CountAnalysis.rds", sep="")
TotalCounts<-readRDS(path)
TotalCountsProcess <-
  data.frame(MicrobiomeID=TotalCounts$DF_Save_w$ID,
             CellNorm=TotalCounts$DF_Save_w$Cells_per_g_feces_FR_corrected_mean)
TotalCountsProcess$MicrobiomeID <- paste("L", TotalCountsProcess$MicrobiomeID,
                                         sep="")
SF2<-merge(SF, TotalCountsProcess, by="MicrobiomeID")

#One value is NaN in sample 602059 assigns value as average
SF2$CellNorm[is.nan(SF2$CellNorm)]<-mean(na.omit(SF2$CellNorm))
SF2$CellNormStand<-SF2$CellNorm/mean(SF2$CellNorm) #Watch out for NaN values can assign
                                                   #average, but wants the error
SF3<-merge(Metadata2, SF2, by="MicrobiomeID")
#DESeq divides with sizeFactor (High cell count should have low sizeFactor providing
  #larger counts in a sample)
#Still have to consider seq data is relative
SF3$NormRelCell<-SF3$Relative/SF3$CellNormStand

##Select size factors calculated above for normalization
dds@colData@listData$sizeFactor <- SF3[,20]
#hist(dds@colData@listData$sizeFactor)
#colnames(SF3[13])
normname<-colnames(SF3[20])

##See vignette for note on factor levels
#Filtering of the Counttable. Bare minimum is performed later. Also to reduce memory of
#the dds
dds2 <- dds[ rowSums(counts(dds))>50*length(Taxonomy2),] #Previously 0 instead of 1

#Estimate dispersions and fit the GLM
dds2 <- DESeq(dds2, test="LRT", reduced = ~1)

res<-results(dds2)
```

```r
summary(res)
```

```
##
## out of 82 with nonzero total read count
## adjusted p-value < 0.1
## LFC > 0 (up)       : 6, 7.3%
## LFC < 0 (down)     : 7, 8.5%
## outliers [1]       : 0, 0%
## low counts [2]     : 0, 0%
## (mean count < 42)
## [1] see 'cooksCutoff' argument of ?results
## [2] see 'independentFiltering' argument of ?results
```

```r
resultsNames(dds2)
```

```
## [1] "Intercept"                 "Diagnosis_T1D_vs_Control"
## [3] "Diagnosis_LADA_vs_Control" "Diagnosis_T2D_vs_Control"
```

```r
#Data structuring
df <- data.frame(res)
df <- tibble::rownames_to_column(df, "Genera")
#After this it is a select and change naming, then merge
df_log2 <- df %>%  select(one_of("Genera", "log2FoldChange")) %>%
  dplyr::rename("LRT none"="log2FoldChange")

df_padj <- df %>% select(one_of("Genera", "padj")) %>%
  dplyr::rename("LRT none"="padj")









##Wald none
#Create design formula
design <- formula(paste("~ ", "Diagnosis"))
#Create DESeq2 object with matrix and metadata
dds <- DESeqDataSetFromMatrix(countData = Taxonomy2,
                              colData = Metadata2,
                              design = design)

##Select size factors calculated above for normalization
dds@colData@listData$sizeFactor <- SF3[,20]

##See vignette for note on factor levels
#Filtering of the Counttable. Bare minimum is performed later. Also to reduce memory of

#the dds
dds <- dds[ rowSums(counts(dds))>(50*length(Taxonomy2)),] #Previously 0 instead of 1
```

```r
#Estimate dispersions and fit the GLM
dds <- DESeq(dds)

res<-results(dds)
summary(res)
```

```
##
## out of 82 with nonzero total read count
## adjusted p-value < 0.1
## LFC > 0 (up)       : 2, 2.4%
## LFC < 0 (down)     : 1, 1.2%
## outliers [1]       : 0, 0%
## low counts [2]     : 0, 0%
## (mean count < 42)
## [1] see 'cooksCutoff' argument of ?results
## [2] see 'independentFiltering' argument of ?results
```

```r
resultsNames(dds)
```

```
## [1] "Intercept"                 "Diagnosis_T1D_vs_Control"
## [3] "Diagnosis_LADA_vs_Control" "Diagnosis_T2D_vs_Control"
```

```r
#Threshold for FDR-adjusted p-values
padj_threshold <- 0.1

if (is.factor(colData(dds)[,"Diagnosis"]) ==  TRUE) {

#Find all two-wise combinations of levels in chosen test factor
test <- combn(levels(colData(dds)[,"Diagnosis"]), 2)
test[,4]<-c("LADA", "T1D") #Want LADA first
foo = test[1,]
poo = test[2,]

#Do DESeq2 analysis on all combinations of levels and save as list
result <- vector("list",length(foo))
for (i in 1:length(foo) ) {
  for (j in 1:length(poo)) {
    result[i] = results(dds, contrast = c("Diagnosis" ,foo[i], poo[i]))
  }
}

#Produce output tables with significant taxa from each comparison
res_list <- vector("list",length(foo))
for (i in 1:length(result) ) {
  res_stat <- data.frame(result[i])
  res_stat <- res_stat[is.na(res_stat$pvalue) == FALSE,]
  stat_sig <- na.omit(res_stat[res_stat$padj < padj_threshold,])
  res_stat$gene <- rownames(res_stat)
  res_stat$g1 <- test[1,i]
  res_stat$g2 <- test[2,i]
  res_stat$compare <- paste(test[1,i], "vs", test[2,i])
  res_list[i] <- list(res_stat)
```

```r
}

res_stat <- do.call('cbind', res_list)
#rownames(res_stat) <- 1:nrow(res_stat)
#res_stat <- res_stat[with(res_stat, order(padj, pvalue, abs(log2FoldChange))),]
#stat_sig <- na.omit(res_stat[res_stat$padj < padj_threshold,])
}

((res_stat[,7]==res_stat[,17])==(res_stat[,27]==res_stat[,37]))==
  ((res_stat[,47]==res_stat[,57]))
```

```
##  [1] TRUE TRUE TRUE TRUE TRUE TRUE TRUE TRUE TRUE TRUE TRUE TRUE TRUE TRUE TRUE
## [16] TRUE TRUE TRUE TRUE TRUE TRUE TRUE TRUE TRUE TRUE TRUE TRUE TRUE TRUE TRUE
## [31] TRUE TRUE TRUE TRUE TRUE TRUE TRUE TRUE TRUE TRUE TRUE TRUE TRUE TRUE TRUE
## [46] TRUE TRUE TRUE TRUE TRUE TRUE TRUE TRUE TRUE TRUE TRUE TRUE TRUE TRUE TRUE
## [61] TRUE TRUE TRUE TRUE TRUE TRUE TRUE TRUE TRUE TRUE TRUE TRUE TRUE TRUE TRUE
## [76] TRUE TRUE TRUE TRUE TRUE TRUE TRUE
```

```r
#testordernone<-res_stat[2,c(10,20,30,40,50,60)]
df_log2_merge <- res_stat[,c(7,12,2,22,32,52,42)]
colnames(df_log2_merge)<- c("Genera", "No adj - Control vs. LADA", "No adj - Control vs. T1D",
                            "No adj - Control vs. T2D", "No adj - LADA vs. T1D",
                            "No adj - LADA vs. T2D", "No adj - T1D vs. T2D")
df_log2 <- merge(df_log2, df_log2_merge, by="Genera")

df_padj_merge <- res_stat[,c(7,16,6,26,36,56,46)]
colnames(df_padj_merge)<- c("Genera", "No adj - Control vs. LADA", "No adj - Control vs. T1D",
                            "No adj - Control vs. T2D", "No adj - LADA vs. T1D",
                            "No adj - LADA vs. T2D", "No adj - T1D vs. T2D")
df_padj <- merge(df_padj, df_padj_merge, by="Genera")







##LRT BMIq
#Create design formula
design <- formula(paste("~ ", "Diagnosis", "+", "BMIq"))
#Create DESeq2 object with matrix and metadata
dds <- DESeqDataSetFromMatrix(countData = Taxonomy2,
                              colData = Metadata2,
                              design = design)


##Assess and calculate size factors using the different methods
SF<-data.frame(Ratio=sizeFactors(estimateSizeFactors(dds, type="ratio")),
               Poscounts=sizeFactors(estimateSizeFactors(dds, type="poscounts")),
               Iterate=sizeFactors(estimateSizeFactors(dds, type="poscounts")),
               #"iterate" takes a lot of time changed to "poscounts" but kept due to the
               #following code
               Relative=colSums(Taxonomy2)/mean(colSums(Taxonomy2))
```

```r
                )
SF<-add_rownames(SF, var="MicrobiomeID")
#Adding total abundances
TotalCounts<-readRDS(path)
TotalCountsProcess <-
  data.frame(MicrobiomeID=TotalCounts$DF_Save_w$ID,
             CellNorm=TotalCounts$DF_Save_w$Cells_per_g_feces_FR_corrected_mean)
TotalCountsProcess$MicrobiomeID <- paste("L", TotalCountsProcess$MicrobiomeID,
                                         sep="")
SF2<-merge(SF, TotalCountsProcess, by="MicrobiomeID")

#One value is NaN in sample 602059 assigns value as average
SF2$CellNorm[is.nan(SF2$CellNorm)]<-mean(na.omit(SF2$CellNorm))
SF2$CellNormStand<-SF2$CellNorm/mean(SF2$CellNorm) #Watch out for NaN values can assign
                                                   #average, but wants the error
SF3<-merge(Metadata2, SF2, by="MicrobiomeID")
#DESeq divides with sizeFactor (High cell count should have low sizeFactor providing
  #larger counts in a sample)
#Still have to consider seq data is relative
SF3$NormRelCell<-SF3$Relative/SF3$CellNormStand

##Select size factors calculated above for normalization
dds@colData@listData$sizeFactor <- SF3[,20]
#hist(dds@colData@listData$sizeFactor)
#colnames(SF3[13])
normname<-colnames(SF3[20])

##See vignette for note on factor levels
#Filtering of the Counttable. Bare minimum is performed later. Also to reduce memory of
#the dds
dds2 <- dds[ rowSums(counts(dds))>50*length(Taxonomy2),] #Previously 0 instead of 1

#Estimate dispersions and fit the GLM
dds2 <- DESeq(dds2, test="LRT", reduced = ~BMIq)

res<-results(dds2)
summary(res)
```

```
##
## out of 82 with nonzero total read count
## adjusted p-value < 0.1
## LFC > 0 (up)       : 5, 6.1%
## LFC < 0 (down)     : 6, 7.3%
## outliers [1]       : 0, 0%
## low counts [2]     : 0, 0%
## (mean count < 49)
## [1] see 'cooksCutoff' argument of ?results
## [2] see 'independentFiltering' argument of ?results
```

```r
resultsNames(dds2)
```

```
## [1] "Intercept"                 "Diagnosis_T1D_vs_Control"
## [3] "Diagnosis_LADA_vs_Control" "Diagnosis_T2D_vs_Control"
```

```
## [5] "BMIq"
```

```r
#Data structuring
df <- data.frame(res)
df <- tibble::rownames_to_column(df, "Genera")
#After this it is a select and change naming, then merge
df_log2_merge <- df %>%  select(one_of("Genera", "log2FoldChange")) %>%
  dplyr::rename("LRT BMI"="log2FoldChange")
df_log2 <- merge(df_log2, df_log2_merge, by="Genera")

df_padj_merge <- df %>% select(one_of("Genera", "padj")) %>%
  dplyr::rename("LRT BMI"="padj")
df_padj <- merge(df_padj, df_padj_merge, by="Genera")







##Wald BMI
#Create design formula
design <- formula(paste("~ ", "Diagnosis", "+", "BMIq"))
#Create DESeq2 object with matrix and metadata
dds <- DESeqDataSetFromMatrix(countData = Taxonomy2,
                              colData = Metadata2,
                              design = design)

##Select size factors calculated above for normalization
dds@colData@listData$sizeFactor <- SF3[,20]

##See vignette for note on factor levels
#Filtering of the Counttable. Bare minimum is performed later. Also to reduce memory of

#the dds
dds <- dds[ rowSums(counts(dds))>(50*length(Taxonomy2)),] #Previously 0 instead of 1

#Estimate dispersions and fit the GLM
dds <- DESeq(dds)

res<-results(dds)
summary(res)
```

```
##
## out of 82 with nonzero total read count
## adjusted p-value < 0.1
## LFC > 0 (up)       : 2, 2.4%
## LFC < 0 (down)     : 2, 2.4%
## outliers [1]       : 0, 0%
## low counts [2]     : 0, 0%
## (mean count < 49)
## [1] see 'cooksCutoff' argument of ?results
## [2] see 'independentFiltering' argument of ?results
```

```r
resultsNames(dds)
```

```
## [1] "Intercept"                 "Diagnosis_T1D_vs_Control"
## [3] "Diagnosis_LADA_vs_Control" "Diagnosis_T2D_vs_Control"
## [5] "BMIq"
```

```r
#Threshold for FDR-adjusted p-values
padj_threshold <- 0.1

if (is.factor(colData(dds)[,"Diagnosis"]) ==  TRUE) {

#Find all two-wise combinations of levels in chosen test factor
test <- combn(levels(colData(dds)[,"Diagnosis"]), 2)
test[,4]<-c("LADA", "T1D") #Want LADA first
foo = test[1,]
poo = test[2,]

#Do DESeq2 analysis on all combinations of levels and save as list
result <- vector("list",length(foo))
for (i in 1:length(foo) ) {
  for (j in 1:length(poo)) {
    result[i] = results(dds, contrast = c("Diagnosis" ,foo[i], poo[i]))
  }
}

#Produce output tables with significant taxa from each comparison
res_list <- vector("list",length(foo))
for (i in 1:length(result) ) {
  res_stat <- data.frame(result[i])
  res_stat <- res_stat[is.na(res_stat$pvalue) == FALSE,]
  stat_sig <- na.omit(res_stat[res_stat$padj < padj_threshold,])
  res_stat$gene <- rownames(res_stat)
  res_stat$g1 <- test[1,i]
  res_stat$g2 <- test[2,i]
  res_stat$compare <- paste(test[1,i], "vs", test[2,i])
  res_list[i] <- list(res_stat)
}

res_stat <- do.call('cbind', res_list)
#rownames(res_stat) <- 1:nrow(res_stat)
#res_stat <- res_stat[with(res_stat, order(padj, pvalue, abs(log2FoldChange))),]
#stat_sig <- na.omit(res_stat[res_stat$padj < padj_threshold,])
}

((res_stat[,7]==res_stat[,17])==(res_stat[,27]==res_stat[,37]))==
  ((res_stat[,47]==res_stat[,57]))
```

```
##  [1] TRUE TRUE TRUE TRUE TRUE TRUE TRUE TRUE TRUE TRUE TRUE TRUE TRUE TRUE TRUE
## [16] TRUE TRUE TRUE TRUE TRUE TRUE TRUE TRUE TRUE TRUE TRUE TRUE TRUE TRUE TRUE
## [31] TRUE TRUE TRUE TRUE TRUE TRUE TRUE TRUE TRUE TRUE TRUE TRUE TRUE TRUE TRUE
## [46] TRUE TRUE TRUE TRUE TRUE TRUE TRUE TRUE TRUE TRUE TRUE TRUE TRUE TRUE TRUE
## [61] TRUE TRUE TRUE TRUE TRUE TRUE TRUE TRUE TRUE TRUE TRUE TRUE TRUE TRUE TRUE
## [76] TRUE TRUE TRUE TRUE TRUE TRUE TRUE
```

```
#testorderBMI<-res_stat[2,c(10,20,30,40,50,60)]
#testordernone==testorderBMI
df_log2_merge <- res_stat[,c(7,12,2,22,32,52,42)]
colnames(df_log2_merge)<- c("Genera", "BMI - Control vs. LADA", "BMI - Control vs. T1D",
                            "BMI - Control vs. T2D", "BMI - LADA vs. T1D",
                            "BMI - LADA vs. T2D", "BMI - T1D vs. T2D")
df_log2 <- merge(df_log2, df_log2_merge, by="Genera")

df_padj_merge <- res_stat[,c(7,16,6,26,36,56,46)]
colnames(df_padj_merge)<- c("Genera", "BMI - Control vs. LADA", "BMI - Control vs. T1D",
                            "BMI - Control vs. T2D", "BMI - LADA vs. T1D",
                            "BMI - LADA vs. T2D", "BMI - T1D vs. T2D")
df_padj <- merge(df_padj, df_padj_merge, by="Genera")








##LRT metformin
#Instead of adding to formula removes them from datasets since it is only present in some
  #groups
##Removing treated with metformin
#Metadata$Metformin <- ifelse(is.na(Metadata$Metformin), "Unknown", Metadata$Metformin)
Metadata2<-filter(Metadata, !Metformin == 1)
table(Metadata2$Diagnosis)
```

```
##
## Control     T1D    LADA     T2D
##      70      30      12      23
```

```
##Applying subsetting to OTU tables
Taxonomy2<-dplyr::select(Taxonomy, one_of(as.character(Metadata2$MicrobiomeID)))
##Reassign names
#Metadata2<-Metadata
Taxonomy2<-Taxonomy2[which(row.names(Taxonomy) %in% row.names(TaxonomyDA)), ]

#The factor can not be ordered for DESeq to run
Metadata2$Diagnosis<-factor(Metadata2$Diagnosis, ordered=FALSE)

#Create design formula
design <- formula(paste("~ ", "Diagnosis"))
#design <- formula(paste("~ ", "Diagnosis", "+", "BMIq"))
#Create DESeq2 object with matrix and metadata
dds <- DESeqDataSetFromMatrix(countData = Taxonomy2,
                              colData = Metadata2,
                              design = design)


##Assess and calculate size factors using the different methods
SF<-data.frame(Ratio=sizeFactors(estimateSizeFactors(dds, type="ratio")),
               Poscounts=sizeFactors(estimateSizeFactors(dds, type="poscounts")),
```

```
               Iterate=sizeFactors(estimateSizeFactors(dds, type="poscounts")),
               #"iterate" takes a lot of time changed to "poscounts" but kept due to the
               #following code
               Relative=colSums(Taxonomy2)/mean(colSums(Taxonomy2))
               )
SF<-add_rownames(SF, var="MicrobiomeID")
#Adding total abundances
TotalCounts<-readRDS(path)
TotalCountsProcess <-
  data.frame(MicrobiomeID=TotalCounts$DF_Save_w$ID,
             CellNorm=TotalCounts$DF_Save_w$Cells_per_g_feces_FR_corrected_mean)
TotalCountsProcess$MicrobiomeID <- paste("L", TotalCountsProcess$MicrobiomeID,
                                         sep="")
SF2<-merge(SF, TotalCountsProcess, by="MicrobiomeID")

#One value is NaN in sample 602059 assigns value as average
SF2$CellNorm[is.nan(SF2$CellNorm)]<-mean(na.omit(SF2$CellNorm))
SF2$CellNormStand<-SF2$CellNorm/mean(SF2$CellNorm) #Watch out for NaN values can assign
                                                   #average, but wants the error
SF3<-merge(Metadata2, SF2, by="MicrobiomeID")
#DESeq divides with sizeFactor (High cell count should have low sizeFactor providing
  #larger counts in a sample)
#Still have to consider seq data is relative
SF3$NormRelCell<-SF3$Relative/SF3$CellNormStand

##Select size factors calculated above for normalization
dds@colData@listData$sizeFactor <- SF3[,20]
#hist(dds@colData@listData$sizeFactor)
#colnames(SF3[13])
normname<-colnames(SF3[20])

##See vignette for note on factor levels
#Filtering of the Counttable. Bare minimum is performed later. Also to reduce memory of
#the dds
dds2 <- dds[ rowSums(counts(dds))>40*length(Taxonomy2),] #Previously 0 instead of 1
  #Changed to 40 instead of 50 after subsetting
    #Otherwise Actinomyces and Family.Erysi... are removed

#Estimate dispersions and fit the GLM
dds2 <- DESeq(dds2, test="LRT", reduced = ~1)

res<-results(dds2)
summary(res)
```

```
##
## out of 84 with nonzero total read count
## adjusted p-value < 0.1
## LFC > 0 (up)       : 3, 3.6%
## LFC < 0 (down)     : 2, 2.4%
## outliers [1]       : 0, 0%
## low counts [2]     : 0, 0%
## (mean count < 41)
## [1] see 'cooksCutoff' argument of ?results
```

```
## [2] see 'independentFiltering' argument of ?results
```

```
resultsNames(dds2)
```

```
## [1] "Intercept"                  "Diagnosis_T1D_vs_Control"
## [3] "Diagnosis_LADA_vs_Control" "Diagnosis_T2D_vs_Control"
```

```
#Data structuring
df <- data.frame(res)
df <- tibble::rownames_to_column(df, "Genera")
#After this it is a select and change naming, then merge
df_log2_merge <- df %>%  select(one_of("Genera", "log2FoldChange")) %>%
  dplyr::rename("LRT Metformin"="log2FoldChange")
df_log2 <- merge(df_log2, df_log2_merge, by="Genera")

df_padj_merge <- df %>% select(one_of("Genera", "padj")) %>%
  dplyr::rename("LRT Metformin"="padj")
df_padj <- merge(df_padj, df_padj_merge, by="Genera")

#setdiff(df$Genera, df_log2$Genera)






##Wald Metformin
#Create design formula
design <- formula(paste("~ ", "Diagnosis"))
#design <- formula(paste("~ ", "Diagnosis", "+", "BMIq"))
#Create DESeq2 object with matrix and metadata
dds <- DESeqDataSetFromMatrix(countData = Taxonomy2,
                              colData = Metadata2,
                              design = design)
##Select size factors calculated above for normalization
dds@colData@listData$sizeFactor <- SF3[,20]

##See vignette for note on factor levels
#Filtering of the Counttable. Bare minimum is performed later. Also to reduce memory of

#the dds
dds <- dds[ rowSums(counts(dds))>(40*length(Taxonomy2)),] #Previously 0 instead of 1
  #Changed to 40 instead of 50 after subsetting
    #Otherwise Actinomyces and Family.Erysi... are removed

#Estimate dispersions and fit the GLM
dds <- DESeq(dds)

res<-results(dds)
summary(res)
```

```
##
## out of 84 with nonzero total read count
```

```
## adjusted p-value < 0.1
## LFC > 0 (up)       : 1, 1.2%
## LFC < 0 (down)     : 2, 2.4%
## outliers [1]       : 0, 0%
## low counts [2]     : 0, 0%
## (mean count < 41)
## [1] see 'cooksCutoff' argument of ?results
## [2] see 'independentFiltering' argument of ?results
```

```
resultsNames(dds)
```

```
## [1] "Intercept"                 "Diagnosis_T1D_vs_Control"
## [3] "Diagnosis_LADA_vs_Control" "Diagnosis_T2D_vs_Control"
```

```
#Threshold for FDR-adjusted p-values
padj_threshold <- 0.1

if (is.factor(colData(dds)[,"Diagnosis"]) ==  TRUE) {

#Find all two-wise combinations of levels in chosen test factor
test <- combn(levels(colData(dds)[,"Diagnosis"]), 2)
test[,4]<-c("LADA", "T1D") #Want LADA first
foo = test[1,]
poo = test[2,]

#Do DESeq2 analysis on all combinations of levels and save as list
result <- vector("list",length(foo))
for (i in 1:length(foo) ) {
  for (j in 1:length(poo)) {
    result[i] = results(dds, contrast = c("Diagnosis" ,foo[i], poo[i]))
  }
}

#Produce output tables with significant taxa from each comparison
res_list <- vector("list",length(foo))
for (i in 1:length(result) ) {
  res_stat <- data.frame(result[i])
  res_stat <- res_stat[is.na(res_stat$pvalue) == FALSE,]
  stat_sig <- na.omit(res_stat[res_stat$padj < padj_threshold,])
  res_stat$gene <- rownames(res_stat)
  res_stat$g1 <- test[1,i]
  res_stat$g2 <- test[2,i]
  res_stat$compare <- paste(test[1,i], "vs", test[2,i])
  res_list[i] <- list(res_stat)
}

res_stat <- do.call('cbind', res_list)
#rownames(res_stat) <- 1:nrow(res_stat)
#res_stat <- res_stat[with(res_stat, order(padj, pvalue, abs(log2FoldChange))),]
#stat_sig <- na.omit(res_stat[res_stat$padj < padj_threshold,])
}

((res_stat[,7]==res_stat[,17])==(res_stat[,27]==res_stat[,37]))==
  ((res_stat[,47]==res_stat[,57]))
```

```
##  [1] TRUE TRUE TRUE TRUE TRUE TRUE TRUE TRUE TRUE TRUE TRUE TRUE TRUE TRUE TRUE
## [16] TRUE TRUE TRUE TRUE TRUE TRUE TRUE TRUE TRUE TRUE TRUE TRUE TRUE TRUE TRUE
## [31] TRUE TRUE TRUE TRUE TRUE TRUE TRUE TRUE TRUE TRUE TRUE TRUE TRUE TRUE TRUE
## [46] TRUE TRUE TRUE TRUE TRUE TRUE TRUE TRUE TRUE TRUE TRUE TRUE TRUE TRUE TRUE
## [61] TRUE TRUE TRUE TRUE TRUE TRUE TRUE TRUE TRUE TRUE TRUE TRUE TRUE TRUE TRUE
## [76] TRUE TRUE TRUE TRUE TRUE TRUE TRUE TRUE TRUE
```

```
#testorderMetformin<-res_stat[2,c(10,20,30,40,50,60)]
#testordernone==testorderMetformin
df_log2_merge <- res_stat[,c(7,12,2,22,32,52,42)]
colnames(df_log2_merge)<- c("Genera", "Metformin - Control vs. LADA", "Metformin - Control vs. T1D",
                            "Metformin - Control vs. T2D", "Metformin - LADA vs. T1D",
                            "Metformin - LADA vs. T2D", "Metformin - T1D vs. T2D")
df_log2 <- merge(df_log2, df_log2_merge, by="Genera")

df_padj_merge <- res_stat[,c(7,16,6,26,36,56,46)]
colnames(df_padj_merge)<- c("Genera", "Metformin - Control vs. LADA", "Metformin - Control vs. T1D",
                            "Metformin - Control vs. T2D", "Metformin - LADA vs. T1D",
                            "Metformin - LADA vs. T2D", "Metformin - T1D vs. T2D")
df_padj <- merge(df_padj, df_padj_merge, by="Genera")
```

```
#Subsetting taxonomy when running with metadatamed
Metadata2 <- MetadataMed
#Metadata2$BMIq<-factor(Metadata2$BMIq)
Metadata2$med_insulin<-factor(Metadata2$med_insulin)
Metadata2$med_statins<-factor(Metadata2$med_statins)
Metadata2$med_protonpump_inhibitor<-factor(Metadata2$med_protonpump_inhibitor)
##Applying subsetting to OTU tables
Taxonomy2<-dplyr::select(Taxonomy, one_of(as.character(Metadata2$MicrobiomeID)))

##Wald insulin
#Create design formula
design <- formula(paste("~ ", "Diagnosis", "+", "med_insulin"))
#Create DESeq2 object with matrix and metadata
dds <- DESeqDataSetFromMatrix(countData = Taxonomy2,
                              colData = Metadata2,
                              design = design)
##Assess and calculate size factors using the different methods
SF<-data.frame(Ratio=sizeFactors(estimateSizeFactors(dds, type="ratio")),
               Poscounts=sizeFactors(estimateSizeFactors(dds, type="poscounts")),
               Iterate=sizeFactors(estimateSizeFactors(dds, type="poscounts")),
               #"iterate" takes a lot of time changed to "poscounts" but kept due to the
               #following code
               Relative=colSums(Taxonomy2)/mean(colSums(Taxonomy2))
               )
```

```
SF<-add_rownames(SF, var="MicrobiomeID")
#Adding total abundances
TotalCounts<-readRDS(path)
TotalCountsProcess <-
  data.frame(MicrobiomeID=TotalCounts$DF_Save_w$ID,
             CellNorm=TotalCounts$DF_Save_w$Cells_per_g_feces_FR_corrected_mean)
TotalCountsProcess$MicrobiomeID <- paste("L", TotalCountsProcess$MicrobiomeID,
                                         sep="")
SF2<-merge(SF, TotalCountsProcess, by="MicrobiomeID")

#One value is NaN in sample 602059 assigns value as average
SF2$CellNorm[is.nan(SF2$CellNorm)]<-mean(na.omit(SF2$CellNorm))
SF2$CellNormStand<-SF2$CellNorm/mean(SF2$CellNorm) #Watch out for NaN values can assign
                                                   #average, but wants the error
SF3<-merge(Metadata2, SF2, by="MicrobiomeID")
#DESeq divides with sizeFactor (High cell count should have low sizeFactor providing
  #larger counts in a sample)
#Still have to consider seq data is relative
SF3$NormRelCell<-SF3$Relative/SF3$CellNormStand
##Select size factors calculated above for normalization
dds@colData@listData$sizeFactor <- SF3[,20]

##See vignette for note on factor levels
#Filtering of the Counttable. Bare minimum is performed later. Also to reduce memory of

#the dds
dds <- dds[ rowSums(counts(dds))>(50*length(Taxonomy2)),] #Previously 0 instead of 1

#Estimate dispersions and fit the GLM
dds <- DESeq(dds)

res<-results(dds)
summary(res)
```

```
##
## out of 124 with nonzero total read count
## adjusted p-value < 0.1
## LFC > 0 (up)       : 9, 7.3%
## LFC < 0 (down)     : 2, 1.6%
## outliers [1]       : 0, 0%
## low counts [2]     : 0, 0%
## (mean count < 0)
## [1] see 'cooksCutoff' argument of ?results
## [2] see 'independentFiltering' argument of ?results
```

```
resultsNames(dds)
```

```
## [1] "Intercept"             "Diagnosis_T1D_vs_LADA" "Diagnosis_T2D_vs_LADA"
## [4] "med_insulin_1_vs_0"
```

```
#Threshold for FDR-adjusted p-values
padj_threshold <- 0.1
```

```r
if (is.factor(colData(dds)[,"Diagnosis"]) ==  TRUE) {

#Find all two-wise combinations of levels in chosen test factor
test <- combn(levels(colData(dds)[,"Diagnosis"]), 2)
foo = test[1,]
poo = test[2,]

#Do DESeq2 analysis on all combinations of levels and save as list
result <- vector("list",length(foo))
for (i in 1:length(foo) ) {
  for (j in 1:length(poo)) {
    result[i] = results(dds, contrast = c("Diagnosis" ,foo[i], poo[i]))
  }
}

#Produce output tables with significant taxa from each comparison
res_list <- vector("list",length(foo))
for (i in 1:length(result) ) {
  res_stat <- data.frame(result[i])
  res_stat <- res_stat[is.na(res_stat$pvalue) == FALSE,]
  stat_sig <- na.omit(res_stat[res_stat$padj < padj_threshold,])
  res_stat$gene <- rownames(res_stat)
  res_stat$g1 <- test[1,i]
  res_stat$g2 <- test[2,i]
  res_stat$compare <- paste(test[1,i], "vs", test[2,i])
  res_list[i] <- list(res_stat)
}

res_stat <- do.call('cbind', res_list)
#rownames(res_stat) <- 1:nrow(res_stat)
#res_stat <- res_stat[with(res_stat, order(padj, pvalue, abs(log2FoldChange))),]
#stat_sig <- na.omit(res_stat[res_stat$padj < padj_threshold,])
}

((res_stat[,7]==res_stat[,17])==(res_stat[,27]==res_stat[,7]))
```

```
##   [1] TRUE TRUE TRUE TRUE TRUE TRUE TRUE TRUE TRUE TRUE TRUE TRUE TRUE TRUE TRUE
##  [16] TRUE TRUE TRUE TRUE TRUE TRUE TRUE TRUE TRUE TRUE TRUE TRUE TRUE TRUE TRUE
##  [31] TRUE TRUE TRUE TRUE TRUE TRUE TRUE TRUE TRUE TRUE TRUE TRUE TRUE TRUE TRUE
##  [46] TRUE TRUE TRUE TRUE TRUE TRUE TRUE TRUE TRUE TRUE TRUE TRUE TRUE TRUE TRUE
##  [61] TRUE TRUE TRUE TRUE TRUE TRUE TRUE TRUE TRUE TRUE TRUE TRUE TRUE TRUE TRUE
##  [76] TRUE TRUE TRUE TRUE TRUE TRUE TRUE TRUE TRUE TRUE TRUE TRUE TRUE TRUE TRUE
##  [91] TRUE TRUE TRUE TRUE TRUE TRUE TRUE TRUE TRUE TRUE TRUE TRUE TRUE TRUE TRUE
## [106] TRUE TRUE TRUE TRUE TRUE TRUE TRUE TRUE TRUE TRUE TRUE TRUE TRUE TRUE TRUE
## [121] TRUE TRUE TRUE TRUE
```

```r
#res_stat[2,c(10,20,30)]
#testordernone==testorderBMI
df_log2_merge <- res_stat[,c(7,2,12,22)]
colnames(df_log2_merge)<- c("Genera", "Insulin - LADA vs. T1D",
                            "Insulin - LADA vs. T2D", "Insulin - T1D vs. T2D")
df_log2 <- merge(df_log2, df_log2_merge, by="Genera")
```

```r
df_padj_merge <- res_stat[,c(7,6,16,26)]
colnames(df_padj_merge)<- c("Genera", "Insulin - LADA vs. T1D",
                            "Insulin - LADA vs. T2D", "Insulin - T1D vs. T2D")
df_padj <- merge(df_padj, df_padj_merge, by="Genera")







##Wald statins
#Create design formula
design <- formula(paste("~ ", "Diagnosis", "+", "med_statins"))
#Create DESeq2 object with matrix and metadata
dds <- DESeqDataSetFromMatrix(countData = Taxonomy2,
                              colData = Metadata2,
                              design = design)

##Select size factors calculated above for normalization
dds@colData@listData$sizeFactor <- SF3[,20]

##See vignette for note on factor levels
#Filtering of the Counttable. Bare minimum is performed later. Also to reduce memory of

#the dds
dds <- dds[ rowSums(counts(dds))>(50*length(Taxonomy2)),] #Previously 0 instead of 1

#Estimate dispersions and fit the GLM
dds <- DESeq(dds)

res<-results(dds)
summary(res)
```

```
##
## out of 124 with nonzero total read count
## adjusted p-value < 0.1
## LFC > 0 (up)       : 2, 1.6%
## LFC < 0 (down)     : 2, 1.6%
## outliers [1]       : 0, 0%
## low counts [2]     : 0, 0%
## (mean count < 0)
## [1] see 'cooksCutoff' argument of ?results
## [2] see 'independentFiltering' argument of ?results
```

```r
resultsNames(dds)
```

```
## [1] "Intercept"             "Diagnosis_T1D_vs_LADA" "Diagnosis_T2D_vs_LADA"
## [4] "med_statins_1_vs_0"
```

```r
#Threshold for FDR-adjusted p-values
padj_threshold <- 0.1
```

```r
if (is.factor(colData(dds)[,"Diagnosis"]) ==  TRUE) {

#Find all two-wise combinations of levels in chosen test factor
test <- combn(levels(colData(dds)[,"Diagnosis"]), 2)
foo = test[1,]
poo = test[2,]

#Do DESeq2 analysis on all combinations of levels and save as list
result <- vector("list",length(foo))
for (i in 1:length(foo) ) {
  for (j in 1:length(poo)) {
    result[i] = results(dds, contrast = c("Diagnosis" ,foo[i], poo[i]))
  }
}

#Produce output tables with significant taxa from each comparison
res_list <- vector("list",length(foo))
for (i in 1:length(result) ) {
  res_stat <- data.frame(result[i])
  res_stat <- res_stat[is.na(res_stat$pvalue) == FALSE,]
  stat_sig <- na.omit(res_stat[res_stat$padj < padj_threshold,])
  res_stat$gene <- rownames(res_stat)
  res_stat$g1 <- test[1,i]
  res_stat$g2 <- test[2,i]
  res_stat$compare <- paste(test[1,i], "vs", test[2,i])
  res_list[i] <- list(res_stat)
}

res_stat <- do.call('cbind', res_list)
#rownames(res_stat) <- 1:nrow(res_stat)
#res_stat <- res_stat[with(res_stat, order(padj, pvalue, abs(log2FoldChange))),]
#stat_sig <- na.omit(res_stat[res_stat$padj < padj_threshold,])
}

((res_stat[,7]==res_stat[,17])==(res_stat[,27]==res_stat[,7]))
```

```
##   [1] TRUE TRUE TRUE TRUE TRUE TRUE TRUE TRUE TRUE TRUE TRUE TRUE TRUE TRUE TRUE
##  [16] TRUE TRUE TRUE TRUE TRUE TRUE TRUE TRUE TRUE TRUE TRUE TRUE TRUE TRUE TRUE
##  [31] TRUE TRUE TRUE TRUE TRUE TRUE TRUE TRUE TRUE TRUE TRUE TRUE TRUE TRUE TRUE
##  [46] TRUE TRUE TRUE TRUE TRUE TRUE TRUE TRUE TRUE TRUE TRUE TRUE TRUE TRUE TRUE
##  [61] TRUE TRUE TRUE TRUE TRUE TRUE TRUE TRUE TRUE TRUE TRUE TRUE TRUE TRUE TRUE
##  [76] TRUE TRUE TRUE TRUE TRUE TRUE TRUE TRUE TRUE TRUE TRUE TRUE TRUE TRUE TRUE
##  [91] TRUE TRUE TRUE TRUE TRUE TRUE TRUE TRUE TRUE TRUE TRUE TRUE TRUE TRUE TRUE
## [106] TRUE TRUE TRUE TRUE TRUE TRUE TRUE TRUE TRUE TRUE TRUE TRUE TRUE TRUE TRUE
## [121] TRUE TRUE TRUE TRUE
```

```r
#res_stat[2,c(10,20,30)]
#testordernone==testorderBMI
df_log2_merge <- res_stat[,c(7,2,12,22)]
colnames(df_log2_merge)<- c("Genera", "Statins - LADA vs. T1D",
                            "Statins - LADA vs. T2D", "Statins - T1D vs. T2D")
df_log2 <- merge(df_log2, df_log2_merge, by="Genera")
```

```
df_padj_merge <- res_stat[,c(7,6,16,26)]
colnames(df_padj_merge)<- c("Genera", "Statins - LADA vs. T1D",
                            "Statins - LADA vs. T2D", "Statins - T1D vs. T2D")
df_padj <- merge(df_padj, df_padj_merge, by="Genera")
```

```
##Wald proton pump inhibitors
#Create design formula
design <- formula(paste("~ ", "Diagnosis", "+", "med_protonpump_inhibitor"))
#Create DESeq2 object with matrix and metadata
dds <- DESeqDataSetFromMatrix(countData = Taxonomy2,
                              colData = Metadata2,
                              design = design)

##Select size factors calculated above for normalization
dds@colData@listData$sizeFactor <- SF3[,20]

##See vignette for note on factor levels
#Filtering of the Counttable. Bare minimum is performed later. Also to reduce memory of

#the dds
dds <- dds[ rowSums(counts(dds))>(50*length(Taxonomy2)),] #Previously 0 instead of 1

#Estimate dispersions and fit the GLM
dds <- DESeq(dds)

res<-results(dds)
summary(res)
```

```
##
## out of 124 with nonzero total read count
## adjusted p-value < 0.1
## LFC > 0 (up)       : 0, 0%
## LFC < 0 (down)     : 5, 4%
## outliers [1]       : 5, 4%
## low counts [2]     : 0, 0%
## (mean count < 0)
## [1] see 'cooksCutoff' argument of ?results
## [2] see 'independentFiltering' argument of ?results
```

```
resultsNames(dds)
```

```
## [1] "Intercept"                    "Diagnosis_T1D_vs_LADA"
## [3] "Diagnosis_T2D_vs_LADA"        "med_protonpump_inhibitor_1_vs_0"
```

```r
#Threshold for FDR-adjusted p-values
padj_threshold <- 0.1

if (is.factor(colData(dds)[,"Diagnosis"]) ==  TRUE) {

#Find all two-wise combinations of levels in chosen test factor
test <- combn(levels(colData(dds)[,"Diagnosis"]), 2)
foo = test[1,]
poo = test[2,]

#Do DESeq2 analysis on all combinations of levels and save as list
result <- vector("list",length(foo))
for (i in 1:length(foo) ) {
  for (j in 1:length(poo)) {
    result[i] = results(dds, contrast = c("Diagnosis" ,foo[i], poo[i]))
  }
}

#Produce output tables with significant taxa from each comparison
res_list <- vector("list",length(foo))
for (i in 1:length(result) ) {
  res_stat <- data.frame(result[i])
  res_stat <- res_stat[is.na(res_stat$pvalue) == FALSE,]
  stat_sig <- na.omit(res_stat[res_stat$padj < padj_threshold,])
  res_stat$gene <- rownames(res_stat)
  res_stat$g1 <- test[1,i]
  res_stat$g2 <- test[2,i]
  res_stat$compare <- paste(test[1,i], "vs", test[2,i])
  res_list[i] <- list(res_stat)
}

res_stat <- do.call('cbind', res_list)
#rownames(res_stat) <- 1:nrow(res_stat)
#res_stat <- res_stat[with(res_stat, order(padj, pvalue, abs(log2FoldChange))),]
#stat_sig <- na.omit(res_stat[res_stat$padj < padj_threshold,])
}

((res_stat[,7]==res_stat[,17])==(res_stat[,27]==res_stat[,7]))
```

```
##   [1] TRUE TRUE TRUE TRUE TRUE TRUE TRUE TRUE TRUE TRUE TRUE TRUE TRUE TRUE TRUE
##  [16] TRUE TRUE TRUE TRUE TRUE TRUE TRUE TRUE TRUE TRUE TRUE TRUE TRUE TRUE TRUE
##  [31] TRUE TRUE TRUE TRUE TRUE TRUE TRUE TRUE TRUE TRUE TRUE TRUE TRUE TRUE TRUE
##  [46] TRUE TRUE TRUE TRUE TRUE TRUE TRUE TRUE TRUE TRUE TRUE TRUE TRUE TRUE TRUE
##  [61] TRUE TRUE TRUE TRUE TRUE TRUE TRUE TRUE TRUE TRUE TRUE TRUE TRUE TRUE TRUE
##  [76] TRUE TRUE TRUE TRUE TRUE TRUE TRUE TRUE TRUE TRUE TRUE TRUE TRUE TRUE TRUE
##  [91] TRUE TRUE TRUE TRUE TRUE TRUE TRUE TRUE TRUE TRUE TRUE TRUE TRUE TRUE TRUE
## [106] TRUE TRUE TRUE TRUE TRUE TRUE TRUE TRUE TRUE TRUE TRUE TRUE TRUE TRUE
```

```r
#res_stat[2,c(10,20,30)]
#testordernone==testorderBMI
df_log2_merge <- res_stat[,c(7,2,12,22)]
colnames(df_log2_merge)<- c("Genera", "PPI - LADA vs. T1D",
                            "PPI - LADA vs. T2D", "PPI - T1D vs. T2D")
```

```
df_log2 <- merge(df_log2, df_log2_merge, by="Genera")

df_padj_merge <- res_stat[,c(7,6,16,26)]
colnames(df_padj_merge)<- c("Genera", "PPI - LADA vs. T1D",
                            "PPI - LADA vs. T2D", "PPI - T1D vs. T2D")
df_padj <- merge(df_padj, df_padj_merge, by="Genera")
```

```
##Wald all
#Create design formula
design <- formula(paste("~ ", "Diagnosis",
                        "+", "BMIq",
                        #"+", "Metformin",
                        "+", "med_insulin",
                        "+", "med_statins",
                        "+", "med_protonpump_inhibitor"))
#Create DESeq2 object with matrix and metadata
dds <- DESeqDataSetFromMatrix(countData = Taxonomy2,
                              colData = Metadata2,
                              design = design)

##Select size factors calculated above for normalization
dds@colData@listData$sizeFactor <- SF3[,20]

##See vignette for note on factor levels
#Filtering of the Counttable. Bare minimum is performed later. Also to reduce memory of

#the dds
dds <- dds[ rowSums(counts(dds))>(50*length(Taxonomy2)),] #Previously 0 instead of 1

#Estimate dispersions and fit the GLM
dds <- DESeq(dds)

res<-results(dds)
summary(res)
```

```
##
## out of 124 with nonzero total read count
## adjusted p-value < 0.1
## LFC > 0 (up)       : 1, 0.81%
## LFC < 0 (down)     : 0, 0%
## outliers [1]       : 13, 10%
## low counts [2]     : 0, 0%
## (mean count < 0)
## [1] see 'cooksCutoff' argument of ?results
## [2] see 'independentFiltering' argument of ?results
```

```
resultsNames(dds)
```

```
## [1] "Intercept"                         "Diagnosis_T1D_vs_LADA"
## [3] "Diagnosis_T2D_vs_LADA"             "BMIq"
## [5] "med_insulin_1_vs_0"                "med_statins_1_vs_0"
## [7] "med_protonpump_inhibitor_1_vs_0"
```

```
#Threshold for FDR-adjusted p-values
padj_threshold <- 0.1

if (is.factor(colData(dds)[,"Diagnosis"]) ==  TRUE) {

#Find all two-wise combinations of levels in chosen test factor
test <- combn(levels(colData(dds)[,"Diagnosis"]), 2)
foo = test[1,]
poo = test[2,]

#Do DESeq2 analysis on all combinations of levels and save as list
result <- vector("list",length(foo))
for (i in 1:length(foo) ) {
  for (j in 1:length(poo)) {
    result[i] = results(dds, contrast = c("Diagnosis" ,foo[i], poo[i]))
  }
}

#Produce output tables with significant taxa from each comparison
res_list <- vector("list",length(foo))
for (i in 1:length(result) ) {
  res_stat <- data.frame(result[i])
  res_stat <- res_stat[is.na(res_stat$pvalue) == FALSE,]
  stat_sig <- na.omit(res_stat[res_stat$padj < padj_threshold,])
  res_stat$gene <- rownames(res_stat)
  res_stat$g1 <- test[1,i]
  res_stat$g2 <- test[2,i]
  res_stat$compare <- paste(test[1,i], "vs", test[2,i])
  res_list[i] <- list(res_stat)
}

res_stat <- do.call('cbind', res_list)
#rownames(res_stat) <- 1:nrow(res_stat)
#res_stat <- res_stat[with(res_stat, order(padj, pvalue, abs(log2FoldChange))),]
#stat_sig <- na.omit(res_stat[res_stat$padj < padj_threshold,])
}

((res_stat[,7]==res_stat[,17])==(res_stat[,27]==res_stat[,7]))
```

```
##   [1] TRUE TRUE TRUE TRUE TRUE TRUE TRUE TRUE TRUE TRUE TRUE TRUE TRUE TRUE TRUE
##  [16] TRUE TRUE TRUE TRUE TRUE TRUE TRUE TRUE TRUE TRUE TRUE TRUE TRUE TRUE TRUE
##  [31] TRUE TRUE TRUE TRUE TRUE TRUE TRUE TRUE TRUE TRUE TRUE TRUE TRUE TRUE TRUE
##  [46] TRUE TRUE TRUE TRUE TRUE TRUE TRUE TRUE TRUE TRUE TRUE TRUE TRUE TRUE TRUE
##  [61] TRUE TRUE TRUE TRUE TRUE TRUE TRUE TRUE TRUE TRUE TRUE TRUE TRUE TRUE TRUE
##  [76] TRUE TRUE TRUE TRUE TRUE TRUE TRUE TRUE TRUE TRUE TRUE TRUE TRUE TRUE TRUE
##  [91] TRUE TRUE TRUE TRUE TRUE TRUE TRUE TRUE TRUE TRUE TRUE TRUE TRUE TRUE TRUE
```

```
## [106] TRUE TRUE TRUE TRUE TRUE TRUE
```

```r
#res_stat[2,c(10,20,30)]
#testordernone==testorderBMI
df_log2_merge <- res_stat[,c(7,2,12,22)]
colnames(df_log2_merge)<- c("Genera", "All - LADA vs. T1D",
                            "All - LADA vs. T2D", "All - T1D vs. T2D")
df_log2 <- merge(df_log2, df_log2_merge, by="Genera")

df_padj_merge <- res_stat[,c(7,6,16,26)]
colnames(df_padj_merge)<- c("Genera", "All - LADA vs. T1D",
                            "All - LADA vs. T2D", "All - T1D vs. T2D")
df_padj <- merge(df_padj, df_padj_merge, by="Genera")









#Filter or merge according to list of genera wanting to include.
#GeneraList<-c("Actinomyces", "Roseburia", "Bifidobacterium", "Odoribacter",
#              "Lactobacillus", "Faecalibacterium")
#Based on lowest padj from LRT none. #Below median of LRT none
GeneraList <- df_padj[df_padj$`LRT none` < median(df_padj$`LRT none`), 1]
df_log2_plot<-subset(df_log2, Genera %in% GeneraList)
df_padj_plot<-subset(df_padj, Genera %in% GeneraList)


#Change genera to rownames
row.names(df_log2_plot) <- df_log2_plot$Genera
df_log2_plot <- select(df_log2_plot, -one_of("Genera"))

row.names(df_padj_plot) <- df_padj_plot$Genera
df_padj_plot <- select(df_padj_plot, -one_of("Genera"))


#Make symbol according to significance level
df_padj_plotsym<-df_padj_plot #Gets a df with same dimensions
#overrides so can just go from lowest and up
for (i in 1:nrow(df_padj_plotsym)) {
  for (j in 1:ncol(df_padj_plotsym)) {
    ifelse(df_padj_plot[i,j]<0.001, df_padj_plotsym[i,j]<-"***",
           ifelse(df_padj_plot[i,j]<0.01, df_padj_plotsym[i,j]<-"**",
                  ifelse(df_padj_plot[i,j]<0.05, df_padj_plotsym[i,j]<-"*",
    ifelse(df_padj_plot[i,j]<0.1, df_padj_plotsym[i,j]<-"\U00B7",
           ifelse(df_padj_plot[i,j]<2, df_padj_plotsym[i,j]<-"", "hmmm"))))) #2 values range 0-1
      #knows it is less
  }
}


max(abs(df_log2_plot))
```

```
## [1] 7.55298
```

```r
range <- max(abs(df_log2_plot))

#Create heatmap
range(df_log2_plot)
```

```
## [1] -7.552980  3.665727
```

```r
pheatmap(df_log2_plot,
         display_numbers = df_padj_plotsym,
         gaps_col = c(7, 14, 21, 24, 27, 30),
         cluster_rows=FALSE,
         show_rownames=TRUE,
         cluster_cols=FALSE,
         show_colnames=TRUE,
         fontsize_number=20,
         legend=TRUE,
         breaks = seq(-range, range, length.out = 30), #Colors centered at 0
         color=colorRampPalette(c("blue", "white", "red"))(30))
```

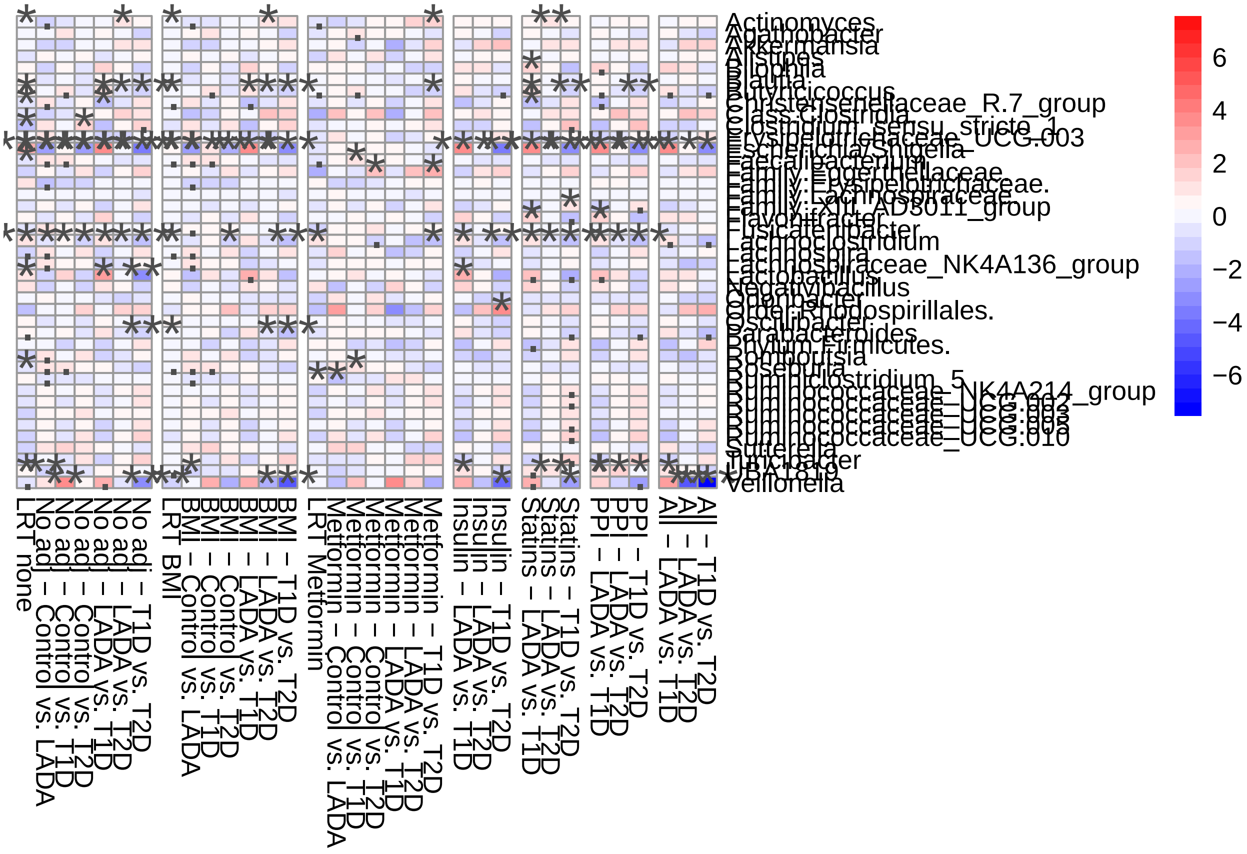

```r
#Draw the heatmap
dev.off()
```

```
## pdf
##   3
```

```
pdf(paste("MicroLADA_Figure3DESeqHeatmap1", ".pdf", sep=""), width=10, height=7)
pheatmap(df_log2_plot,
         display_numbers = df_padj_plotsym,
         gaps_col = c(7, 14, 21, 24, 27, 30),
         cluster_rows=FALSE,
         show_rownames=TRUE,
         cluster_cols=FALSE,
         show_colnames=TRUE,
         fontsize_number=10,
         legend=TRUE,
         breaks = seq(-range, range, length.out = 30),
         color=colorRampPalette(c("blue", "white", "red"))(30))

dev.off()
```

```
## pdf
##   3
```

```
#Make density plot
#In the end a solution with new_scale_fill and defining two background datasets
df_density<-data.frame(values=as.vector(as.matrix(df_log2_plot)))
xvalues <- c(-7, 7)
coloring<-c(colorRampPalette(c("red", "white", "red"))(30))
coloring2<-c(colorRampPalette(c("blue", "white", "blue"))(30))
background <- data.frame(lower = seq( xvalues[1], xvalues[2]-sum(abs(xvalues))/30,
                                      sum(abs(xvalues))/30 ),
                         upper = seq( xvalues[1]+sum(abs(xvalues))/30, xvalues[2],
                                      sum(abs(xvalues))/30 ),
                         col = coloring,
                         col2 = coloring2)

background1<-background[1:15,]
background2<-background[16:30,]

ggplot() +
    geom_rect(data = background1 ,
              mapping = aes(xmin = lower ,
                            xmax = upper ,
                            ymin = 0 ,
                            ymax = 0.7 ,
                            fill = col ) ,
              alpha = .9 ) +
    scale_fill_manual(values = alpha(background1$col2, 0.9))+
    new_scale_fill()+
    geom_rect(data = background2 ,
              mapping = aes(xmin = lower ,
                            xmax = upper ,
                            ymin = 0 ,
                            ymax = 0.7 ,
                            fill = col2 ) ,
```

```
             alpha = .9 ) +
    scale_fill_manual(values = alpha(background1$col, 0.9)) +
    theme_classic()+
    geom_density(data = df_density, aes(x=values), size=2) +
    theme_classic() +
    xlab("Log2-fold change") +
    ylab("Density") +
    theme(legend.position = "none")

pdf(paste("MicroLADA_Figure3DESeqHeatmap1density", ".pdf", sep=""), width=3, height=3)
ggplot() +
    geom_rect(data = background1 ,
              mapping = aes(xmin = lower ,
                            xmax = upper ,
                            ymin = 0 ,
                            ymax = 0.7 ,
                            fill = col ) ,
              alpha = .9 ) +
    scale_fill_manual(values = alpha(background1$col2, 0.9))+
    new_scale_fill()+
    geom_rect(data = background2 ,
              mapping = aes(xmin = lower ,
                            xmax = upper ,
                            ymin = 0 ,
                            ymax = 0.7 ,
                            fill = col2 ) ,
              alpha = .9 ) +
    scale_fill_manual(values = alpha(background1$col, 0.9)) +
    theme_classic()+
    geom_density(data = df_density, aes(x=values), size=1) +
    theme_classic() +
    xlab("Log2-fold change") +
    ylab("Density") +
    theme(legend.position = "none")
dev.off()
```

```
## pdf
##   3
```

```
##Here the other genera
#Filter or merge according to list of genera wanting to include.
#GeneraList<-c("Actinomyces", "Roseburia", "Bifidobacterium", "Odoribacter",
#               "Lactobacillus", "Faecalibacterium")
#Based on lowest padj from LRT none. #Below median of LRT none
GeneraList <- df_padj[df_padj$`LRT none` >= median(df_padj$`LRT none`), 1]
df_log2_plot<-subset(df_log2, Genera %in% GeneraList)
df_padj_plot<-subset(df_padj, Genera %in% GeneraList)


#Change genera to rownames
row.names(df_log2_plot) <- df_log2_plot$Genera
df_log2_plot <- select(df_log2_plot, -one_of("Genera"))
```

```r
row.names(df_padj_plot) <- df_padj_plot$Genera
df_padj_plot <- select(df_padj_plot, -one_of("Genera"))


#Make symbol according to significance level
df_padj_plotsym<-df_padj_plot #Gets a df with same dimensions
#overrides so can just go from lowest and up
for (i in 1:nrow(df_padj_plotsym)) {
  for (j in 1:ncol(df_padj_plotsym)) {
    ifelse(df_padj_plot[i,j]<0.001, df_padj_plotsym[i,j]<-"***",
           ifelse(df_padj_plot[i,j]<0.01, df_padj_plotsym[i,j]<-"**",
                  ifelse(df_padj_plot[i,j]<0.05, df_padj_plotsym[i,j]<-"*",
    ifelse(df_padj_plot[i,j]<0.1, df_padj_plotsym[i,j]<-"\U00B7",
           ifelse(df_padj_plot[i,j]<2, df_padj_plotsym[i,j]<-"", "hmmm"))))) #2 values range 0-1
      #knows it is less
  }
}


#max(abs(df_log2_plot))
#range <- max(abs(df_log2_plot)) #Would rather use the previous to make the heatmaps
  #directly comparable

#Create heatmap
range(df_log2_plot)
```

```
## [1] -2.913747  2.518626
```

```r
pheatmap(df_log2_plot,
         display_numbers = df_padj_plotsym,
         gaps_col = c(7, 14, 21, 24, 27, 30),
         cluster_rows=FALSE,
         show_rownames=TRUE,
         cluster_cols=FALSE,
         show_colnames=TRUE,
         fontsize_number=20,
         legend=TRUE,
         breaks = seq(-range, range, length.out = 30), #Colors centered at 0
         color=colorRampPalette(c("blue", "white", "red"))(30))


#Draw the heatmap
dev.off()
```

```
## null device
##           1
```

```r
pdf(paste("MicroLADA_Figure3DESeqHeatmap2", ".pdf", sep=""), width=10, height=7)
pheatmap(df_log2_plot,
         display_numbers = df_padj_plotsym,
         gaps_col = c(7, 14, 21, 24, 27, 30),
         cluster_rows=FALSE,
```

```
         show_rownames=TRUE,
         cluster_cols=FALSE,
         show_colnames=TRUE,
         fontsize_number=10,
         legend=TRUE,
         breaks = seq(-range, range, length.out = 30),
         color=colorRampPalette(c("blue", "white", "red"))(30))

dev.off()
```

```
## null device
##           1
```

```
#Make density plot
#In the end a solution with new_scale_fill and defining two background datasets
df_density<-data.frame(values=as.vector(as.matrix(df_log2_plot)))
xvalues <- c(-7, 7)
coloring<-c(colorRampPalette(c("red", "white", "red"))(30))
coloring2<-c(colorRampPalette(c("blue", "white", "blue"))(30))
background <- data.frame(lower = seq( xvalues[1], xvalues[2]-sum(abs(xvalues))/30,
                                      sum(abs(xvalues))/30 ),
                         upper = seq( xvalues[1]+sum(abs(xvalues))/30, xvalues[2],
                                      sum(abs(xvalues))/30 ),
                         col = coloring,
                         col2 = coloring2)

background1<-background[1:15,]
background2<-background[16:30,]

ggplot() +
    geom_rect(data = background1 ,
              mapping = aes(xmin = lower ,
                            xmax = upper ,
                            ymin = 0 ,
                            ymax = 0.75 ,
                            fill = col ) ,
              alpha = .9 ) +
    scale_fill_manual(values = alpha(background1$col2, 0.9))+
    new_scale_fill()+
    geom_rect(data = background2 ,
              mapping = aes(xmin = lower ,
                            xmax = upper ,
                            ymin = 0 ,
                            ymax = 0.75 ,
                            fill = col2 ) ,
              alpha = .9 ) +
    scale_fill_manual(values = alpha(background1$col, 0.9)) +
    theme_classic()+
    geom_density(data = df_density, aes(x=values), size=2) +
    theme_classic() +
    xlab("Log2-fold change") +
    ylab("Density") +
    theme(legend.position = "none")
```

```
pdf(paste("MicroLADA_Figure3DESeqHeatmap2density", ".pdf", sep=""), width=6, height=4)
ggplot() +
    geom_rect(data = background1 ,
              mapping = aes(xmin = lower ,
                            xmax = upper ,
                            ymin = 0 ,
                            ymax = 0.75 ,
                            fill = col ) ,
              alpha = .9 ) +
    scale_fill_manual(values = alpha(background1$col2, 0.9))+
    new_scale_fill()+
    geom_rect(data = background2 ,
              mapping = aes(xmin = lower ,
                            xmax = upper ,
                            ymin = 0 ,
                            ymax = 0.75 ,
                            fill = col2 ) ,
              alpha = .9 ) +
    scale_fill_manual(values = alpha(background1$col, 0.9)) +
    theme_classic()+
    geom_density(data = df_density, aes(x=values), size=2) +
    theme_classic() +
    xlab("Log2-fold change") +
    ylab("Density") +
    theme(legend.position = "none")
dev.off()
```

```
## pdf
##   2
```

```
#Make table log2fold changes and p values
df_log2 <- data.frame(df_log2[,-1], row.names = df_log2[,1])
df_padj <- data.frame(df_padj[,-1], row.names = df_padj[,1])

if((sum(colnames(df_log2)!=colnames(df_padj))==0)==FALSE) {stop("Columns does not match")}
if((sum(rownames(df_log2)!=rownames(df_padj))==0)==FALSE) {stop("Rows does not match")}

df_paste <- df_log2 #just to get dimensions, row- and columnnames

for (i in 1:nrow(df_log2)) {
  for (j in 1:ncol(df_log2)) {
    df_paste[i,j]<-paste(signif(df_log2[i,j], digits=4), " (",
                         signif(df_padj[i,j], digits=4) , ")")
  }
}
write.table(df_paste, file="DESeqHeatmapl2fpval_SupTab.txt", sep="\t", dec=",", row.names = T)
```

Additional

Session information

```
sessionInfo()
```

```
## R version 4.1.2 (2021-11-01)
## Platform: x86_64-w64-mingw32/x64 (64-bit)
## Running under: Windows 10 x64 (build 19044)
##
## Matrix products: default
##
## locale:
## [1] LC_COLLATE=Danish_Denmark.1252  LC_CTYPE=Danish_Denmark.1252
## [3] LC_MONETARY=Danish_Denmark.1252 LC_NUMERIC=C
## [5] LC_TIME=Danish_Denmark.1252
##
## attached base packages:
##  [1] stats4    grid      tcltk     stats     graphics  grDevices utils
##  [8] datasets  methods   base
##
## other attached packages:
##  [1] Maaslin2_1.8.0              DESeq2_1.34.0
##  [3] SummarizedExperiment_1.24.0 Biobase_2.54.0
##  [5] MatrixGenerics_1.6.0        matrixStats_0.61.0
##  [7] GenomicRanges_1.46.1        GenomeInfoDb_1.30.0
##  [9] IRanges_2.28.0              S4Vectors_0.32.3
## [11] BiocGenerics_0.40.0         SIAMCAT_1.14.0
## [13] phyloseq_1.38.0             mlr_2.19.0
## [15] ParamHelpers_1.14           ALDEx2_1.26.0
## [17] BiocParallel_1.28.3         mixOmics_6.18.1
## [19] ggnewscale_0.4.5            hablar_0.3.0
## [21] VennDiagram_1.7.1           futile.logger_1.4.3
## [23] cowplot_1.1.1               seqinr_4.2-8
## [25] stringr_1.4.0               RColorBrewer_1.1-2
## [27] pheatmap_1.0.12             BiodiversityR_2.14-1
## [29] curl_4.3.2                  htmlTable_2.4.0
## [31] plotly_4.10.0               nlme_3.1-153
## [33] exactRankTests_0.8-34       mime_0.12
## [35] digest_0.6.28               tidyr_1.1.4
## [37] reshape2_1.4.4              compositions_2.0-2
## [39] zCompositions_1.3.4         truncnorm_1.0-8
## [41] NADA_1.6-1.1                survival_3.2-13
## [43] MASS_7.3-54                 gridExtra_2.3
## [45] vegan_2.5-7                 lattice_0.20-45
## [47] permute_0.9-5               ggplot2_3.3.5
## [49] knitr_1.37                  dplyr_1.0.7
## [51] tibble_3.1.6
##
## loaded via a namespace (and not attached):
##   [1] bit64_4.0.5            DelayedArray_0.20.0    data.table_1.14.2
##   [4] rpart_4.1-15           KEGGREST_1.34.0        RCurl_1.98-1.5
##   [7] generics_0.1.1         lambda.r_1.2.4         RSQLite_2.2.9
```

```
##  [10] proxy_0.4-26           bit_4.0.4              bayesm_3.1-4
##  [13] PRROC_1.3.1            relimp_1.0-5           xfun_0.29
##  [16] hms_1.1.1              LiblineaR_2.10-12      evaluate_0.14
##  [19] DEoptimR_1.0-10        fansi_0.5.0            progress_1.2.2
##  [22] readxl_1.3.1           igraph_1.2.10          DBI_1.1.2
##  [25] geneplotter_1.72.0     htmlwidgets_1.5.4      tensorA_0.36.2
##  [28] rARPACK_0.11-0         purrr_0.3.4            ellipsis_0.3.2
##  [31] crosstalk_1.2.0        corrplot_0.92          RSpectra_0.16-0
##  [34] backports_1.4.1        insight_0.14.5         survey_4.1-1
##  [37] annotate_1.72.0        gridBase_0.4-7         vctrs_0.3.8
##  [40] abind_1.4-5            cachem_1.0.6           withr_2.4.3
##  [43] RcmdrMisc_2.7-2        robustbase_0.93-9      checkmate_2.0.0
##  [46] prettyunits_1.1.1      getopt_1.20.3          cluster_2.1.2
##  [49] ape_5.6                lazyeval_0.2.2         crayon_1.4.2
##  [52] ellipse_0.4.2          genefilter_1.76.0      labeling_0.4.2
##  [55] glmnet_4.1-3           pkgconfig_2.0.3        Rcmdr_2.7-2
##  [58] nnet_7.3-16            rlang_0.4.12           lifecycle_1.0.1
##  [61] sandwich_3.0-1         cellranger_1.1.0       Matrix_1.4-0
##  [64] carData_3.0-5          lpsymphony_1.22.0      Rhdf5lib_1.16.0
##  [67] boot_1.3-28            zoo_1.8-9              base64enc_0.1-3
##  [70] png_0.1-7              viridisLite_0.4.0      bitops_1.0-7
##  [73] rhdf5filters_1.6.0     pROC_1.18.0            Biostrings_2.62.0
##  [76] blob_1.2.2             shape_1.4.6            jpeg_0.1-9
##  [79] scales_1.1.1           memoise_2.0.1          magrittr_2.0.1
##  [82] plyr_1.8.6             zlibbioc_1.40.0        compiler_4.1.2
##  [85] lme4_1.1-27.1          ade4_1.7-18            XVector_0.34.0
##  [88] formatR_1.11           Formula_1.2-4          mgcv_1.8-38
##  [91] tidyselect_1.1.1       stringi_1.7.6          tcltk2_1.2-11
##  [94] forcats_0.5.1          highr_0.9              mitools_2.4
##  [97] yaml_2.2.1             locfit_1.5-9.4         latticeExtra_0.6-29
## [100] ggrepel_0.9.1          fastmatch_1.1-3        tools_4.1.2
## [103] parallel_4.1.2         rstudioapi_0.13        foreach_1.5.1
## [106] foreign_0.8-81         optparse_1.7.1         farver_2.1.0
## [109] RcppZiggurat_0.1.6     nortest_1.0-4          Rcpp_1.0.7
## [112] car_3.0-12             infotheo_1.2.0         httr_1.4.2
## [115] AnnotationDbi_1.56.2   effects_4.2-0          colorspace_2.0-2
## [118] XML_3.99-0.8           splines_4.1.2          multtest_2.50.0
## [121] xtable_1.8-4           jsonlite_1.7.2         nloptr_1.2.2.3
## [124] futile.options_1.0.1   BBmisc_1.11            corpcor_1.6.10
## [127] Rfast_2.0.4            R6_2.5.1               Hmisc_4.6-0
## [130] pillar_1.6.4           htmltools_0.5.2        glue_1.5.0
## [133] fastmap_1.1.0          minqa_1.2.4            class_7.3-19
## [136] beanplot_1.2           codetools_0.2-18       pcaPP_1.9-74
## [139] mvtnorm_1.1-3          utf8_1.2.2             biglm_0.9-2.1
## [142] parallelMap_1.5.1      rmarkdown_2.11         biomformat_1.22.0
## [145] munsell_0.5.0          e1071_1.7-9            rhdf5_2.38.0
## [148] GenomeInfoDbData_1.2.7 iterators_1.0.13       haven_2.4.3
## [151] gtable_0.3.0
```

This document was processed on:

```r
Sys.Date()
```

```
## [1] "2022-02-24"
```

Supplementary file 2: Functional R analysis. Supporting information include additional data summary, PCoA dimensions 1-3 also subsetted to diagnostic groups in pairs, heatmaps, CCA, venn diagrams comparing significant entities between the other groups and violin plots of all functional KEGG orthologs that had a significant result obtained from the DESeq (Wald) test.

Microbiome analysis LADA study – Functional analysis

Casper Sahl Poulsen

februar 24, 2022

Contents

Introduction . . . 2
- Samples . . . 2
- Metadata . . . 2
- Packages . . . 2
- Comments running piphillin . . . 3
- Reading in data and Overview . . . 4
- Used colors throughout script . . . 11

Analysis . . . 13
- Summary metadata, quality of data and ecology . . . 13
- Summary metadata, quality of data and ecology Metformin removed . . . 26
- Alpha diversity . . . 40
- Alpha diversity remove metformin . . . 44
- Violin plots Dissimilarities . . . 47
- Violin plots Dissimilarities remove metformin . . . 55
- PCoA . . . 63
- PCoA remove metformin . . . 79
- Create figure 1 . . . 96
- PERMANOVA . . . 97
- PERMANOVA remove metformin . . . 103
- PERMANOVA MED subset . . . 106
- Pairwise PERMANOVA . . . 110
- Pairwise PERMANOVA remove metformin . . . 113
- Heatmap . . . 114
- CCA . . . 116
- DESeq2 LRT . . . 124
- DESeq2 LRT remove metformin . . . 133

DESeq Wald 144
DESeq Wald Remove metformin 192
Create figure 2 373
DESeq heatmap 374
Additional 408
Session information 408
This document was processed on: 410

Introduction

Analysis of microbiome data (16S rRNA gene amplicon profiling) from the latent autoimmune diabetes in adults (LADA) study.

Samples

70 LADA
30 T1D
70 T2D
70 Non-diabetic controls

Metadata

Metadata: is the original metadata provided
MetadataEkstra: is subsequent provided metadata used only in the summary chunks. Have it as separate object, because square bracket selectors are used throughout the script.
MetadataMed: Selected covariates included not part of MetadataEkstra. Extracted from different metadata files from the original cohorts (see also "StenoClean_LADA_Medication_CSP_220105.Rmd).

Packages

```r
#install.packages(c("dplyr", "knitr", "ggplot2", "vegan", "gridExtra", "zCompositions",
#                   "compositions", "reshape2", "tidyr", "digest", "mime",
#                   "exactRankTests", "nlme", "plotly", "BiodiversityR", "pheatmap",
#                   "RColorBrewer", "htmlTable", "curl", "stringr", "seqinr", "cowplot",
#                   "VennDiagram", "hablar", "ggnewscale"))
library(tibble)
library(dplyr)
library(knitr)
library(ggplot2)
library(vegan)
library(gridExtra)
library(zCompositions)
library(compositions)
library(reshape2)
library(tidyr)
library(digest)
library(mime)
```

```
library(exactRankTests)
library(nlme)
library(plotly)
library(htmlTable)
library(curl)
library(BiodiversityR)
library(pheatmap)
library(RColorBrewer)
library(stringr)
library(seqinr)
library(cowplot)
library(VennDiagram)
library(hablar)
library(ggnewscale)

#Bioconductor packages
#if (!requireNamespace("BiocManager", quietly = TRUE))
#    install.packages("BiocManager")
#BiocManager::install("mixOmics")
library("mixOmics")

#BiocManager::install("BiocParallel")
library("BiocParallel")

#BiocManager::install("ALDEx2")
library("ALDEx2")

#BiocManager::install("SIAMCAT")
library("SIAMCAT")

#BiocManager::install("DESeq2")
library("DESeq2")

# old way devtools::install_bitbucket("biobakery/maaslin2@default", ref="tip")
#BiocManager::install("Maaslin2")
library("Maaslin2")
```

Comments running piphillin

```
##Running the online tool for piphillin had to create fasta and OTU csv
##Create fasta file of ref seqs
##otus <- paste(rep("otu", length(Taxonomy)), seq(length(Taxonomy)), sep="")
##sequences <- colnames(Taxonomy)
#write.fasta(sequences = as.list(colnames(Taxonomy)),
#            names = paste(rep("otu", length(Taxonomy)),
#                          seq(length(Taxonomy)), sep=""),
#            file.out="piphillinrepseq.fasta")

##Change names in taxonomy file to otus it does not matter that they are asv see two
##articles in mendeley on piphillin
#Taxonomy2<-Taxonomy
#names(Taxonomy2)<-paste(rep("otu", length(Taxonomy)), seq(length(Taxonomy)), sep="")
```

```
#Taxonomy3<-t(Taxonomy2)
#write.csv(Taxonomy3, file="P:/CBMR/LADA/piphillinOTU.csv")
##Added OTU to start of file manually

##Getting 3 tables from piphillin
#Object referred to as Taxonomy to make the script easily modifiable
##Not sure what this contains genome aap?
#load("ko_genome_contribution_table.Rdata")
#test<-genome_contribution[1:10,]
```

Reading in data and Overview

Have to run “Reading in data” and “Pre-processing” from script “MicroLADA_200220.Rmd” to generate files

```
Metadata <- read.delim(file="P:/CBMR/LADA/Text/Analysis/Metadata_200318.txt",
                       check.names=FALSE,
                       stringsAsFactors = FALSE,
                       strip.white=TRUE,
                       dec=",")
#Specify column types
Metadata <- Metadata %>% convert(chr(Metformin),
                                 num(age, BMI))
#head(Metadata)
#confidential but metadata as colnames and samples as rownames


##Pathways calls them taxonomy because I'm using the script from taxonomy analysis
Taxonomy <- read.table("P:/CBMR/LADA/ko_pathway_abund_table_unnorm.txt",
                       sep="", header=TRUE, check.names=FALSE)
#Change pathways into rownames
row.names(Taxonomy)<-Taxonomy$Pathway
Taxonomy <- select(Taxonomy, -one_of("Pathway"))

##Consider rounding all values up to mimic counts
Taxonomy<-ceiling(Taxonomy)

#head(Taxonomy[,1:6])
#confidential but samples as colnames and pathways as rownames

##Read KEGG pathways annotation corresponding to the feature table
#Feature from Tue
Feature <- read.table("P:/CBMR/LADA/Piphillin_KEGG_pathways_legend.csv",
                      sep=";", header=TRUE, check.names=FALSE)
colnames(Feature)<-c("ID", "Level1", "Level2", "Level3")
#table(Feature$Level1)
#Missing from my table
UnclassMetab <- filter(Feature, Feature$Level1 == "Unclassified metabolism")

#Directly from json KEGG BRITE using BriteJSONtoRdfGithub_200810 also
#available at github csapou
Feature <-
```

```
  read.delim(file="P:/CBMR/LADA/Text/Analysis/Functional/FeatureFunctional_200812.txt")
Feature <- data.frame(ID=Feature[,4], Feature[,1:3])
Feature <- rbind(Feature, UnclassMetab)
table(Feature$Level1)
```

```
##
##                    Brite Hierarchies                  Cellular Processes
##                                   52                                  33
## Environmental Information Processing       Genetic Information Processing
##                                   41                                  22
##                       Human Diseases                          Metabolism
##                                   92                                 168
##     Not Included in Pathway or Brite                  Organismal Systems
##                                   24                                  90
##               Unclassified metabolism
##                                   10
```

```
#Remove all without path (poorly characterized)
Feature <- filter(Feature, !Level1 == "Not Included in Pathway or Brite")

if (setequal(colnames(Taxonomy), Metadata$MicrobiomeID)==FALSE) {
  stop("Metadata and Taxonomy out of sync")
}


#Check for duplicated entries
nonuniq<-data.frame(table(row.names(Taxonomy)))
nonuniq[nonuniq$Freq > 1,]
```

```
## [1] Var1 Freq
## <0 rækker> (eller 0-længde row.names)
```

```
#Different only contained in first element so it is ok that not all pathways are found
#But it is not okay if we have pathways that is not in the feature table
setdiff(Feature$ID, rownames(Taxonomy))
```

```
##   [1] "ko00062"      "ko00073"      "ko00512"      "ko00532"      "ko00534"
##   [6] "ko00533"      "ko00563"      "ko00904"      "ko00905"      "ko01052"
##  [11] "ko00522"      "ko01059"      "ko01056"      "ko00253"      "ko00945"
##  [16] "ko00941"      "ko00942"      "ko00943"      "ko00403"      "ko01058"
##  [21] "ko00402"      "ko00331"      "ko00254"      "ko00998"      "ko00997"
##  [26] "ko00365"      "character(0)" "ko03040"      "ko04130"      "ko04120"
##  [31] "ko04014"      "ko04015"      "ko04010"      "ko04012"      "ko04310"
##  [36] "ko04330"      "ko04340"      "ko04341"      "ko04350"      "ko04390"
##  [41] "ko04391"      "ko04392"      "ko04370"      "ko04630"      "ko04064"
##  [46] "ko04668"      "ko04022"      "ko04075"      "ko04060"      "ko04061"
##  [51] "ko04512"      "ko04514"      "ko04136"      "ko04137"      "ko04139"
##  [56] "ko04111"      "ko04114"      "ko04218"      "ko04510"      "ko04520"
##  [61] "ko04540"      "ko04550"      "ko04810"      "ko04640"      "ko04610"
##  [66] "ko04611"      "ko04620"      "ko04624"      "ko04623"      "ko04625"
##  [71] "ko04650"      "ko04660"      "ko04658"      "ko04662"      "ko04664"
##  [76] "ko04666"      "ko04670"      "ko04672"      "ko04062"      "ko04911"
```

```
##  [81] "ko04923"      "ko04929"      "ko04912"      "ko04913"      "ko04926"
##  [86] "ko04935"      "ko04924"      "ko04925"      "ko04927"      "ko04261"
##  [91] "ko04270"      "ko04970"      "ko04971"      "ko04972"      "ko04975"
##  [96] "ko04977"      "ko04962"      "ko04960"      "ko04961"      "ko04966"
## [101] "ko04720"      "ko04730"      "ko04723"      "ko04721"      "ko04722"
## [106] "ko04744"      "ko04745"      "ko04740"      "ko04742"      "ko04750"
## [111] "ko04320"      "ko04360"      "ko04361"      "ko04380"      "ko04710"
## [116] "ko04713"      "ko04711"      "ko04712"      "ko05202"      "ko05235"
## [121] "ko05212"      "ko05226"      "ko05214"      "ko05216"      "ko05221"
## [126] "ko05220"      "ko05217"      "ko05218"      "ko05213"      "ko05224"
## [131] "ko05223"      "ko05310"      "ko05323"      "ko05320"      "ko05321"
## [136] "ko05330"      "ko05332"      "ko05017"      "ko05032"      "ko05033"
## [141] "ko05410"      "ko05412"      "ko05414"      "ko04950"      "ko04933"
## [146] "ko05130"      "ko05135"      "ko05144"      "ko05140"      "ko01522"
## [151] "[BR:ko01001" "[BR:ko01009" "[BR:ko01002" "[BR:ko01003" "[BR:ko01005"
## [156] "[BR:ko01011" "[BR:ko01004" "[BR:ko01008" "[BR:ko01006" "[BR:ko01007"
## [161] "[BR:ko00199" "[BR:ko00194" "[BR:ko03000" "[BR:ko03021" "[BR:ko03019"
## [166] "[BR:ko03041" "[BR:ko03011" "[BR:ko03009" "[BR:ko03016" "[BR:ko03012"
## [171] "[BR:ko03110" "[BR:ko04131" "[BR:ko04121" "[BR:ko03051" "[BR:ko03032"
## [176] "[BR:ko03036" "[BR:ko03400" "[BR:ko03029" "[BR:ko02000" "[BR:ko02044"
## [181] "[BR:ko02042" "[BR:ko02022" "[BR:ko02035" "[BR:ko04812" "[BR:ko04147"
## [186] "[BR:ko02048" "[BR:ko04030" "[BR:ko04050" "[BR:ko04054" "[BR:ko03310"
## [191] "[BR:ko04040" "[BR:ko04031" "[BR:ko04052" "[BR:ko04515" "[BR:ko04090"
## [196] "[BR:ko00535" "[BR:ko00536" "[BR:ko00537" "[BR:ko04091" "[BR:ko01504"
## [201] "[BR:ko03200" "[BR:ko03100"
```

```
#Had missing represented by unclassified metabolism added from Tue
setdiff(rownames(Taxonomy), Feature$ID)
```

```
## character(0)
```

```
#Merge feature and count table
#Call last feature Genus for intercompatibility
colnames(Feature)<-c("ID", "Level_1", "Level_2", "Genus")
row.names(Feature)<-Feature$ID
TaxEdit <- merge(Feature, Taxonomy, by="row.names")

##Percent lost from merging should be nothing when getting new feature table
#Are they in sync
colnames(TaxEdit)[6:length(TaxEdit)]==colnames(Taxonomy)
```

```
##   [1] TRUE TRUE TRUE TRUE TRUE TRUE TRUE TRUE TRUE TRUE TRUE TRUE TRUE TRUE TRUE
##  [16] TRUE TRUE TRUE TRUE TRUE TRUE TRUE TRUE TRUE TRUE TRUE TRUE TRUE TRUE TRUE
##  [31] TRUE TRUE TRUE TRUE TRUE TRUE TRUE TRUE TRUE TRUE TRUE TRUE TRUE TRUE TRUE
##  [46] TRUE TRUE TRUE TRUE TRUE TRUE TRUE TRUE TRUE TRUE TRUE TRUE TRUE TRUE TRUE
##  [61] TRUE TRUE TRUE TRUE TRUE TRUE TRUE TRUE TRUE TRUE TRUE TRUE TRUE TRUE TRUE
##  [76] TRUE TRUE TRUE TRUE TRUE TRUE TRUE TRUE TRUE TRUE TRUE TRUE TRUE TRUE TRUE
##  [91] TRUE TRUE TRUE TRUE TRUE TRUE TRUE TRUE TRUE TRUE TRUE TRUE TRUE TRUE TRUE
## [106] TRUE TRUE TRUE TRUE TRUE TRUE TRUE TRUE TRUE TRUE TRUE TRUE TRUE TRUE TRUE
## [121] TRUE TRUE TRUE TRUE TRUE TRUE TRUE TRUE TRUE TRUE TRUE TRUE TRUE TRUE TRUE
## [136] TRUE TRUE TRUE TRUE TRUE TRUE TRUE TRUE TRUE TRUE TRUE TRUE TRUE TRUE TRUE
## [151] TRUE TRUE TRUE TRUE TRUE TRUE TRUE TRUE TRUE TRUE TRUE TRUE TRUE TRUE TRUE
## [166] TRUE TRUE TRUE TRUE TRUE TRUE TRUE TRUE TRUE TRUE TRUE TRUE TRUE TRUE TRUE
```

```
## [181] TRUE TRUE TRUE TRUE TRUE TRUE TRUE TRUE TRUE TRUE TRUE TRUE TRUE TRUE TRUE
## [196] TRUE TRUE TRUE TRUE TRUE TRUE TRUE TRUE TRUE TRUE TRUE TRUE TRUE TRUE TRUE
## [211] TRUE TRUE TRUE TRUE TRUE TRUE TRUE TRUE TRUE TRUE TRUE TRUE TRUE TRUE TRUE
## [226] TRUE TRUE TRUE TRUE TRUE TRUE TRUE TRUE TRUE TRUE TRUE TRUE TRUE TRUE TRUE
```

```
#empty vector
vecs<-vector()
#Get all percentages
for (i in 1:length(Taxonomy)) {
  #print(sum(TaxEdit[,(i+5)])/sum(Taxonomy[,i]))
  vecs[i] <- sum(TaxEdit[,(i+5)])/sum(Taxonomy[,i])
}

range(vecs) #Nothings lost
```

```
## [1] 1 1
```

```
#Remove Human Diseases and Organismal Systems see recommendation by Tue in mail
TaxEdit <- filter(TaxEdit, !Level_1 == "Human Diseases" & !Level_1 == "Organismal Systems"
                  & !Level_1 == "Brite Hierarchies")


##Percent lost from filtering
#Are they in sync
colnames(TaxEdit)[6:length(TaxEdit)]==colnames(Taxonomy)
```

```
##   [1] TRUE TRUE TRUE TRUE TRUE TRUE TRUE TRUE TRUE TRUE TRUE TRUE TRUE TRUE TRUE
##  [16] TRUE TRUE TRUE TRUE TRUE TRUE TRUE TRUE TRUE TRUE TRUE TRUE TRUE TRUE TRUE
##  [31] TRUE TRUE TRUE TRUE TRUE TRUE TRUE TRUE TRUE TRUE TRUE TRUE TRUE TRUE TRUE
##  [46] TRUE TRUE TRUE TRUE TRUE TRUE TRUE TRUE TRUE TRUE TRUE TRUE TRUE TRUE TRUE
##  [61] TRUE TRUE TRUE TRUE TRUE TRUE TRUE TRUE TRUE TRUE TRUE TRUE TRUE TRUE TRUE
##  [76] TRUE TRUE TRUE TRUE TRUE TRUE TRUE TRUE TRUE TRUE TRUE TRUE TRUE TRUE TRUE
##  [91] TRUE TRUE TRUE TRUE TRUE TRUE TRUE TRUE TRUE TRUE TRUE TRUE TRUE TRUE TRUE
## [106] TRUE TRUE TRUE TRUE TRUE TRUE TRUE TRUE TRUE TRUE TRUE TRUE TRUE TRUE TRUE
## [121] TRUE TRUE TRUE TRUE TRUE TRUE TRUE TRUE TRUE TRUE TRUE TRUE TRUE TRUE TRUE
## [136] TRUE TRUE TRUE TRUE TRUE TRUE TRUE TRUE TRUE TRUE TRUE TRUE TRUE TRUE TRUE
## [151] TRUE TRUE TRUE TRUE TRUE TRUE TRUE TRUE TRUE TRUE TRUE TRUE TRUE TRUE TRUE
## [166] TRUE TRUE TRUE TRUE TRUE TRUE TRUE TRUE TRUE TRUE TRUE TRUE TRUE TRUE TRUE
## [181] TRUE TRUE TRUE TRUE TRUE TRUE TRUE TRUE TRUE TRUE TRUE TRUE TRUE TRUE TRUE
## [196] TRUE TRUE TRUE TRUE TRUE TRUE TRUE TRUE TRUE TRUE TRUE TRUE TRUE TRUE TRUE
## [211] TRUE TRUE TRUE TRUE TRUE TRUE TRUE TRUE TRUE TRUE TRUE TRUE TRUE TRUE TRUE
## [226] TRUE TRUE TRUE TRUE TRUE TRUE TRUE TRUE TRUE TRUE TRUE TRUE TRUE TRUE TRUE
```

```
#empty vector
vecs<-vector()
#Get all percentages
for (i in 1:length(Taxonomy)) {
  #print(sum(TaxEdit[,(i+5)])/sum(Taxonomy[,i]))
  vecs[i] <- sum(TaxEdit[,(i+5)])/sum(Taxonomy[,i])
}
range(vecs)
```

```
## [1] 0.9523525 0.9716654
```

```r
#Aggregate the rows that have exactly the same entries and sum counts
Tax2<-TaxEdit %>% group_by(Level_1, Level_2, Genus) %>%
  summarise_if(is.numeric, funs(sum))

#Check if there are multiple entries of the same, change according to aggregation
if (sum(table(Tax2$Genus)>1)!=0) {
  stop("Need unique entries for all entities")
}


#Make feature table
Feature<-Tax2[,1:3]


##Include for all selections on how to handle NA
#Add Class as row names and keep numeric columns
#rownames(Tax2) <- Tax2$Class
Taxonomy<-data.frame(Tax2[,4:ncol(Tax2)], row.names = Tax2$Genus,
                     check.names=FALSE) #Change when having other taxonomy tables

##Filtering for DA analysis not relevant much fewer zeroes
#TaxonomyDA<-Taxonomy[length(Taxonomy)-rowSums(Taxonomy == 0) >= 120,]


#Add a categorical indicator of group in Metadata
Metadata$Diagnosis<-as.factor(ifelse(grepl("0", Metadata$group), "Control",
                                     ifelse(grepl("1", Metadata$group), "T1D",
                                            ifelse(grepl("2", Metadata$group), "T2D",
                                                   ifelse(grepl("4", Metadata$group),
                                                          "LADA", "wrong")))))
#table(Metadata$Diagnosis)
#Change order
#levels(as.factor(Metadata2$Diagnosis))
Metadata$Diagnosis<-ordered(Metadata$Diagnosis,
                            levels=c("Control", "T1D", "T2D", "LADA"))

if (setequal(colnames(Taxonomy), Metadata$MicrobiomeID)==FALSE) {
  stop("Taxonomy and Metadata does not have the same entries")
}

Metadata <- Metadata[match(colnames(Taxonomy), Metadata$MicrobiomeID),]

##Add categorical variable to metadata with Metformin
Metadata$Metformin <- ifelse(is.na(Metadata$Metformin), "Unknown", Metadata$Metformin)
##Consider recoding unknown metformin treatment as no treatment
Metadata$Metformin <- ifelse(Metadata$Metformin=="Unknown", "0", Metadata$Metformin)




#Create syntactically valid variable names
Metadata$MicrobiomeID <- paste("L", Metadata$MicrobiomeID, sep="")
colnames(Taxonomy) <- Metadata$MicrobiomeID
```

```r
#Order Diagnosis
Metadata$Diagnosis<-ordered(Metadata$Diagnosis,
                            levels=c("Control", "T1D", "LADA", "T2D"))

##Removing age below 30 at diagnosis of LADA
age<-c("LLADA079", "LLADA085", "LLADA082", "LLADA063")
#Ageinfo<-Metadata[match(age, Metadata$MicrobiomeID), ]
Metadata<-Metadata[-match(age, Metadata$MicrobiomeID), ]
##Applying subsetting to OTU tables
Taxonomy<-dplyr::select(Taxonomy, one_of(as.character(Metadata$MicrobiomeID)))

##Removing LADA that were not tested positive for GAD
GAD<-c("LLADA090", "LLADA076", "LLADA095", "LLADA070", "LLADA080")
#GADinfo<-Metadata[match(GAD, Metadata$MicrobiomeID), ]
Metadata<-Metadata[-match(GAD, Metadata$MicrobiomeID), ]

##Removing LADA with insufficient metadata available (lacking C-peptide measurement)
Ins<-c("LLADA005")
#Insinfo<-Metadata[match(Ins, Metadata$MicrobiomeID), ]
Metadata<-Metadata[-match(Ins, Metadata$MicrobiomeID), ]

##Applying subsetting to OTU tables
Taxonomy<-dplyr::select(Taxonomy, one_of(as.character(Metadata$MicrobiomeID)))

##Remove orgs that are not present after subsetting.
Taxonomy <- Taxonomy[rowSums(Taxonomy)>0,]

length(Taxonomy)
```

```
## [1] 230
```

```r
#Filtering at the beginning of script
TaxonomyDA<-Taxonomy[length(Taxonomy)-rowSums(Taxonomy == 0) >= length(Taxonomy)/2,]

mean(colSums(TaxonomyDA)/colSums(Taxonomy))
```

```
## [1] 0.9999999
```

```r
sd(colSums(TaxonomyDA)/colSums(Taxonomy))
```

```
## [1] 1.090406e-06
```

```r
range(colSums(TaxonomyDA)/colSums(Taxonomy))
```

```
## [1] 0.9999855 1.0000000
```

```r
hist(colSums(TaxonomyDA)/colSums(Taxonomy))
```

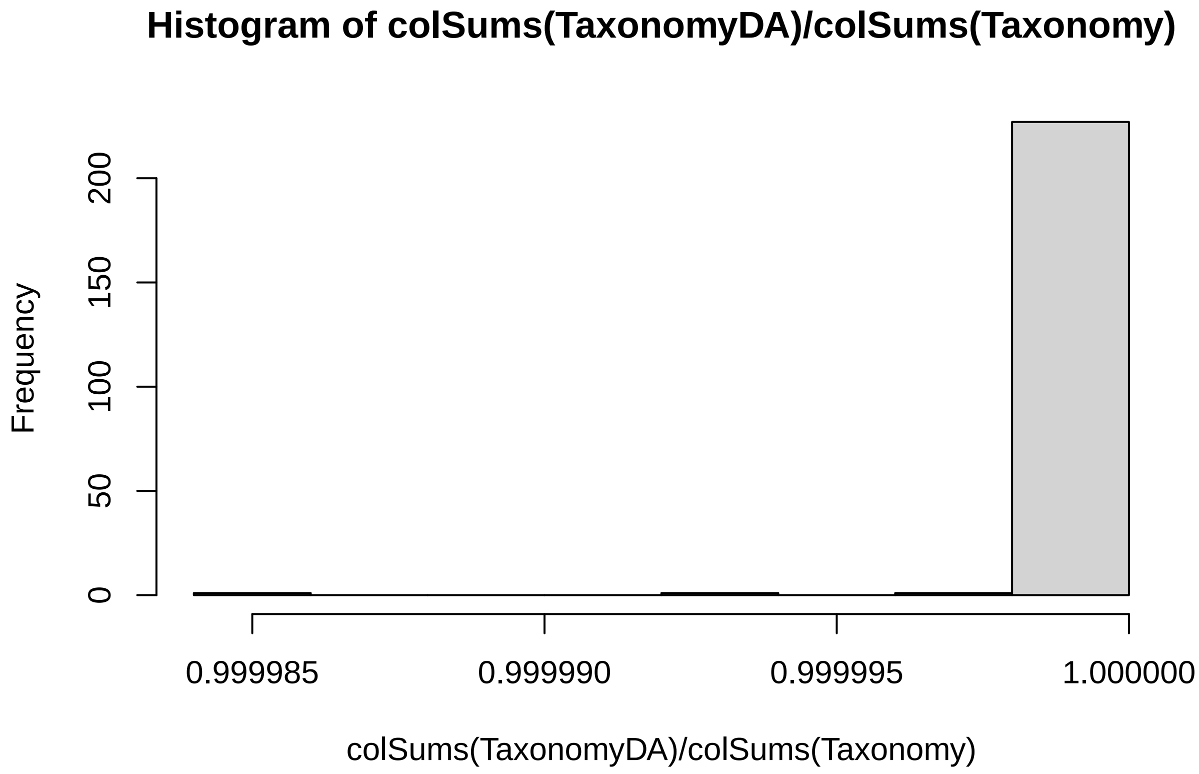


```
##Compared sample where many reads are removed due to 50% filtering
#samplebef<-select(Taxonomy, one_of("L606044"))
#samplefilter<-select(TaxonomyDA, one_of("L606044"))

##Used to provide medication overview
#write.table(Metadata, file="MetadataMedMerge.txt",
#             quote = F, row.names = F, sep="\t")

##See "StenoClean_LADA_Medication_CSP_220105.Rmd" for addition of medication
  #Do not have information on medicine of controls
MetadataMed <- read.delim(file="../Metadata_Medication.txt",
                       check.names=FALSE,
                       stringsAsFactors = FALSE,
                       strip.white=TRUE)

#Encoding BMI
Metadata$BMIord <- ifelse(Metadata$BMI<18.5, "Under",
              ifelse(Metadata$BMI>=18.5 & Metadata$BMI<25, "Normal",
              ifelse(Metadata$BMI>=25 & Metadata$BMI<30, "Over",
              ifelse(Metadata$BMI>=30 & Metadata$BMI<35, "Ob1",
              ifelse(Metadata$BMI>=35 & Metadata$BMI<40, "Ob2",
              ifelse(Metadata$BMI>=40, "Ob3",
                "Other"))))))
MetadataMed$BMIord <- ifelse(MetadataMed$BMI<18.5, "Under",
              ifelse(MetadataMed$BMI>=18.5 & MetadataMed$BMI<25, "Normal",
              ifelse(MetadataMed$BMI>=25 & MetadataMed$BMI<30, "Over",
```

```
              ifelse(MetadataMed$BMI>=30 & MetadataMed$BMI<35, "Ob1",
              ifelse(MetadataMed$BMI>=35 & MetadataMed$BMI<40, "Ob2",
              ifelse(MetadataMed$BMI>=40, "Ob3",
                 "Other"))))))
table(Metadata$BMIord) #Based on this could make 3 classes
```

```
##
## Normal    Ob1    Ob2    Ob3   Over  Under
##     54     45     22      6    102      1
```

```
#table(MetadataMed$BMIord)
Metadata$BMIclass <- ifelse(Metadata$BMI<25, "Class1",
              ifelse(Metadata$BMI>=25 & Metadata$BMI<30, "Class2",
              ifelse(Metadata$BMI>=30, "Class3",
                 "Other")))
MetadataMed$BMIclass <- ifelse(MetadataMed$BMI<25, "Class1",
              ifelse(MetadataMed$BMI>=25 & MetadataMed$BMI<30, "Class2",
              ifelse(MetadataMed$BMI>=30, "Class3",
                 "Other")))
table(Metadata$BMIclass)
```

```
##
## Class1 Class2 Class3
##     55    102     73
```

```
Metadata$BMIordclass <- factor(ifelse(Metadata$BMI<25, 1,
              ifelse(Metadata$BMI>=25 & Metadata$BMI<30, 2,
              ifelse(Metadata$BMI>=30, 3,
                 "Other"))))
MetadataMed$BMIordclass <- factor(ifelse(MetadataMed$BMI<25, 1,
              ifelse(MetadataMed$BMI>=25 & MetadataMed$BMI<30, 2,
              ifelse(MetadataMed$BMI>=30, 3,
                 "Other"))))

#Quartiles
Metadata$BMIq <- as.numeric(cut_number(Metadata$BMI,4))
MetadataMed$BMIq <- as.numeric(cut_number(MetadataMed$BMI,4))
#table(Metadata$BMIq)
```

Used colors throughout script

```
#install.packages("viridis")
#install.packages("colorBlindness")
#install.packages("RColorBrewer")
library(viridis)
library(colorBlindness)
library(RColorBrewer)
display.brewer.pal(n=8, "Dark2")
display.brewer.pal(n=9, "Set1")
display.brewer.pal(n=12, "Paired")
```

```r
colorBlindness::displayAllColors(viridis::viridis(10))
colorBlindness::displayAllColors(rainbow(10))
colorBlindness::displayAllColors(brewer.pal(n=12, "Paired")) #Should be color blind friendly
colorBlindness::displayAllColors(brewer.pal(n=12, "Set1"))
colorBlindness::displayAllColors(brewer.pal(n=12, "Dark2")) #Should be color blind friendly
colorBlindness::displayAllColors(brewer.pal(n=12, "Set2"))

#From paired but adding Grey from Dark2. Using dark version for
brewer.pal(n=12, "Paired")
brewer.pal(n=12, "Dark2")
brewer.pal(n=12, "Set2")

##From Paired
#"#A6CEE3" Proteobacteria in functional Cellular Processes
#"#1F78B4" T1D
#"#B2DF8A" Firmicutes in functional Environmental Information Processing
#"#33A02C" T2D
#"#FB9A99" Bacteroidetes in functional Genetic Information Processing
#"#E31A1C" LADA
#"#FDBF6F" Actinobacteria in functional Metabolism
#"#FF7F00"
#"#CAB2D6" Euryarchaeota in functional Unclassified metabolism
#"#6A3D9A"
#"#FFFF99"
#"#B15928"

#From Dark2
#"#666666" Controls dark grey

#From Set2
#"#B3B3B3" Other light grey in functional also Other

colorBlindness::displayAllColors(c("#666666", "#1F78B4", "#E31A1C", "#33A02C",
                                   "#A6CEE3", "#B2DF8A", "#FB9A99", "#FDBF6F",
                                   "#CAB2D6", "#B3B3B3"))
```

Controls "#0000FF" Blue new "#666666" dark grey
T1D "#FF0000" Red new "#1F78B4" dark blue
T2D "#228B22" Green new "#33A02C" dark green LADA "#FFD700" Yellow new "#E31A1C" red

Cellular Processes "#3366CC" lightblue new "#005F6A" Petrol new "#A6CEE3" light blue
Genetic Information Processing "#DC3912" Redorange new "#9C58A1" Purple new "#FB9A99" pink
Metabolism "#FF9900" Yelloworange new "#EF9F26" Orange new "#ADD8E6" light blue new "#00FFFF" Aqua new "#FDBF6F" orange Environmental Information Processing "#109618" Green new "#97D0A7" Seafoam new "#B2DF8A" light green Unclassified metabolism "#DD4477" Pink new "#CAB2D6" light purple
Other "#B82E2E" Red new "#4C0013" Bordeaux new "#B3B3B3" light grey

Colors used from color brewer paired. The dark colors for diagnostic groups and light for the Level_1 for instance in cca and vulcano plots. To represent controls a dark grey was used, a light grey was used for other Level_1. LADA is deliberately red to draw the readers attention.

Analysis

Summary metadata, quality of data and ecology

Used to create table 1

```
rm(list=setdiff(ls(), c("Metadata", "MetadataMed", "Feature", "TaxonomyDA", "Taxonomy")))

##Summary metadata and quality of data
#Adding cell count to infer absolute cell numbers
# old path path<-paste("Q:/",
#             "Projects/",
#             "LADA/",
#             "LADA_Sandra_Evelina/",
#             "LADA_JKV/",
#             "LADA_R_AfterFlow_Analysis_FinalCounts/",
#             "LADA_FinalCounts/",
#             "2019-09-03_CountAnalysis.rds", sep="")

path <- paste("J:/",
              "CBMR/",
              "SUN-CBMR-Hansen-Group/",
              "Projects/",
              "LADA/",
              "LADA_Sandra_Evelina/",
              "LADA_JKV/",
              "LADA_R_AfterFlow_Analysis_FinalCounts/",
              "LADA_FinalCounts/",
              "2019-09-03_CountAnalysis.rds", sep="")

TotalCounts<-readRDS(path)
TotalCountsProcess <-
  data.frame(MicrobiomeID=TotalCounts$DF_Save_w$ID,
             CellNorm=TotalCounts$DF_Save_w$Cells_per_g_feces_FR_corrected_mean)
TotalCountsProcess$MicrobiomeID <- paste("L", TotalCountsProcess$MicrobiomeID,
                                         sep="")

covariates<-merge(Metadata, TotalCountsProcess, by="MicrobiomeID")

#One value is NaN in sample L602059 assigns value as average
covariates$CellNorm[is.nan(covariates$CellNorm)]<-
  mean(na.omit(covariates$CellNorm))

covariates<-merge(covariates, data.frame(seq_depth=colSums(Taxonomy),
                                         MicrobiomeID=names(Taxonomy)),
                  by="MicrobiomeID")
print("Sex: male=1, female=2")
```

```
## [1] "Sex: male=1, female=2"
```

```
table(Metadata$sex, Metadata$Diagnosis)
```

```
##
```

```
##     Control T1D LADA T2D
##   1      44  17   37  44
##   2      26  13   23  26
```

```r
print("Metformin: No treatment=0, treatment=1")
```

```
## [1] "Metformin: No treatment=0, treatment=1"
```

```r
table(Metadata$Metformin, Metadata$Diagnosis)
```

```
##
##     Control T1D LADA T2D
##   0      70  30   12  23
##   1       0   0   48  47
```

```r
print("Age, BMI and seq_depth")
```

```
## [1] "Age, BMI and seq_depth"
```

```r
aggregate(covariates[, c(2:3, 9:10)], list(covariates$Diagnosis), mean)
```

```
##   Group.1      age      BMI BMIord BMIclass
## 1 Control 61.44243 26.67011     NA       NA
## 2     T1D 51.73196 26.66011     NA       NA
## 3    LADA 63.76249 28.92226     NA       NA
## 4     T2D 61.42814 31.27686     NA       NA
```

```r
aggregate(covariates[, c(2:3, 9:10)], list(covariates$Diagnosis), sd)
```

```
##   Group.1       age      BMI BMIord BMIclass
## 1 Control 10.189143 3.722805     NA       NA
## 2     T1D  6.961407 4.555865     NA       NA
## 3    LADA 10.183290 4.866525     NA       NA
## 4     T2D  9.956564 5.512931     NA       NA
```

```r
##Look into metadata if patient received metformin or not
print("Sex: male=1, female=2. Metformin: No treatment=0, treatment=1")
```

```
## [1] "Sex: male=1, female=2. Metformin: No treatment=0, treatment=1"
```

```r
table(Metadata$sex, Metadata$Metformin)
```

```
##
##      0  1
##   1 78 64
##   2 57 31
```

```r
print("Age, BMI and seq_depth")
```

```
## [1] "Age, BMI and seq_depth"
```

```r
aggregate(covariates[, c(2:3, 9:10)], list(covariates$Metformin), mean)
```

```
##   Group.1      age      BMI BMIord BMIclass
## 1       0 60.12622 27.62709     NA       NA
## 2       1 61.70115 30.12390     NA       NA
```

```r
aggregate(covariates[, c(2:3, 9:10)], list(covariates$Metformin), sd)
```

```
##   Group.1      age      BMI BMIord BMIclass
## 1       0 10.57613 4.706784     NA       NA
## 2       1 10.02674 5.276407     NA       NA
```

```r
##Testing covariates
#Kruskal test
kruskal.test(BMI ~ Diagnosis, data=covariates)
```

```
##
##  Kruskal-Wallis rank sum test
##
## data:  BMI by Diagnosis
## Kruskal-Wallis chi-squared = 37.354, df = 3, p-value = 3.872e-08
```

```r
kruskal.test(age ~ Diagnosis, data=covariates)
```

```
##
##  Kruskal-Wallis rank sum test
##
## data:  age by Diagnosis
## Kruskal-Wallis chi-squared = 31.529, df = 3, p-value = 6.576e-07
```

```r
kruskal.test(CellNorm ~ Diagnosis, data=covariates)
```

```
##
##  Kruskal-Wallis rank sum test
##
## data:  CellNorm by Diagnosis
## Kruskal-Wallis chi-squared = 1.7778, df = 3, p-value = 0.6198
```

```r
kruskal.test(seq_depth ~ Diagnosis, data=covariates)
```

```
##
##  Kruskal-Wallis rank sum test
##
## data:  seq_depth by Diagnosis
## Kruskal-Wallis chi-squared = 0.56799, df = 3, p-value = 0.9037
```

```r
#If significant Mann-Whitney pairwise post-test
pairwise.wilcox.test(covariates$BMI, covariates$Diagnosis,
                     p.adjust.method="bonferroni")
```

```
##
##  Pairwise comparisons using Wilcoxon rank sum test with continuity correction
##
## data:  covariates$BMI and covariates$Diagnosis
##
##      Control T1D     LADA
## T1D  1.00000 -       -
## LADA 0.03204 0.15124 -
## T2D  1e-07   0.00024 0.04139
##
## P value adjustment method: bonferroni
```

```r
pairwise.wilcox.test(covariates$age, covariates$Diagnosis,
                     p.adjust.method="bonferroni")
```

```
##
##  Pairwise comparisons using Wilcoxon rank sum test with continuity correction
##
## data:  covariates$age and covariates$Diagnosis
##
##      Control T1D     LADA
## T1D  1.1e-05 -       -
## LADA 1       8.7e-07 -
## T2D  1       1.8e-05 1
##
## P value adjustment method: bonferroni
```

```r
pairwise.wilcox.test(covariates$seq_depth, covariates$Diagnosis,
                     p.adjust.method="bonferroni")
```

```
##
##  Pairwise comparisons using Wilcoxon rank sum test with continuity correction
##
## data:  covariates$seq_depth and covariates$Diagnosis
##
##      Control T1D LADA
## T1D  1       -   -
## LADA 1       1   -
## T2D  1       1   1
##
## P value adjustment method: bonferroni
```

```r
##Plot rarefaction curves can decrease step for final plotting
#set.seed(1)
#rarecurve(t(Taxonomy), step=100, xlab="Annotated reads",
#          ylab="Genera", label=FALSE)
```

```
#Create rarefaction curves with colors according to categories
#Adding colors to the rarecurves according to experiment, Controls=#666666 (blue),
#T1D=#1F78B4 (red), T2D=#33A02C (forestgreen), LADA=#E31A1C (gold)
rare<-data.frame(t(Taxonomy))
rare<-add_rownames(rare, "MicrobiomeID")
rare<-merge(rare, Metadata, by="MicrobiomeID")
rare$colors<-ifelse(grepl("0", rare$group), "#666666",
                    ifelse(grepl("1", rare$group), "#1F78B4",
                           ifelse(grepl("2", rare$group), "#33A02C",
                                  ifelse(grepl("4", rare$group),
                                         "#E31A1C", "pink"))))
#table(rare$colors)
rare$line<-ifelse(grepl("0", rare$Metformin), "solid",
                  ifelse(grepl("1", rare$Metformin), "dotted", "longdash"))
#table(rare$line)

#Check order
if (setequal(colnames(Taxonomy), rare$MicrobiomeID)==FALSE) {
  stop("Taxonomy and Metadata is not corresponding with each other")
}

set.seed(1)
rarecurve(t(Taxonomy), step=100000, xlab="Annotated reads", ylab="Genera",
          col=rare$colors,
          lty=rare$line, label=FALSE) #piphillin pathways 100000, else 100
```

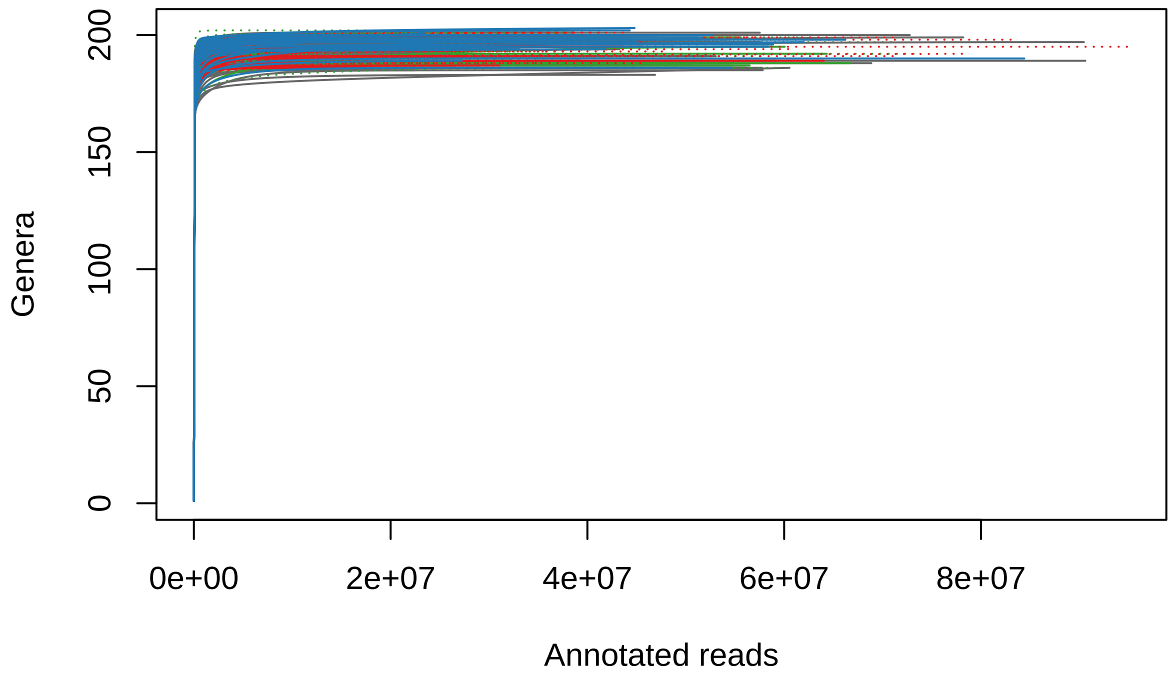

```r
pdf(paste("MicroLADA_Rarecurve_Func.pdf", sep=""), height=6, width=8)
rarecurve(t(Taxonomy), step=100000, xlab="Annotated reads", ylab="Genera",
          col=rare$colors,
          lty=rare$line, label=FALSE)
dev.off()
```

```
## pdf
##   2
```

```r
##Rank abundance curves
#Total sum scaling (Use relative abundances)
Taxonomy2<-sweep(Taxonomy, 2, colSums(Taxonomy), FUN="/")

##RAC all and diagnostic groupings
pdf(paste("MicroLADA_RAC_Func", ".pdf", sep=""), width=12, height=6)
par(mfrow=c(1,5))
Subset <- c("All", "Control", "T1D", "LADA", "T2D")

for (i in Subset) {
  #Subsetting Metadata according to
  if (i=="All") {
    Metadata2 <- Metadata
  } else if (i=="Control") {
    Metadata2<-filter(Metadata, Diagnosis == "Control")
  } else if (i=="T1D") {
    Metadata2<-filter(Metadata, Diagnosis == "T1D")
  } else if (i=="LADA") {
    Metadata2<-filter(Metadata, Diagnosis == "LADA")
  } else if (i=="T2D") {
    Metadata2<-filter(Metadata, Diagnosis == "T2D")
  } else {
    print("Subset defined not valid")
  }

  #Applying subsetting to OTU tables, have already TSS
  Taxonomy3<-dplyr::select(Taxonomy2, one_of(as.character(Metadata2$MicrobiomeID)))
  #Setting up ranks
  RankAbun.1 <- rankabundance(t(Taxonomy3))
  #Create RACplot
  rankabunplot(RankAbun.1, scale='proportion', addit=FALSE, specnames=c(1,2,3,4,5),
               xlim=c(0,60), main=i)
}
dev.off()
```

```
## pdf
##   2
```

```r
#Include in pdf
Subset <- c("All", "Control", "T1D", "LADA", "T2D")
for (i in Subset) {
  #Subsetting Metadata according to
  if (i=="All") {
```

```
    Metadata2 <- Metadata
  } else if (i=="Control") {
    Metadata2<-filter(Metadata, Diagnosis == "Control")
  } else if (i=="T1D") {
    Metadata2<-filter(Metadata, Diagnosis == "T1D")
  } else if (i=="LADA") {
    Metadata2<-filter(Metadata, Diagnosis == "LADA")
  } else if (i=="T2D") {
    Metadata2<-filter(Metadata, Diagnosis == "T2D")
  } else {
    print("Subset defined not valid")
  }

  #Applying subsetting to OTU tables, have already TSS
  Taxonomy3<-dplyr::select(Taxonomy2, one_of(as.character(Metadata2$MicrobiomeID)))
  #Setting up ranks
  RankAbun.1 <- rankabundance(t(Taxonomy3))
  #Create RACplot
  rankabunplot(RankAbun.1, scale='proportion', addit=FALSE, specnames=c(1,2,3,4,5),
               xlim=c(0,60), main=i)
}
```

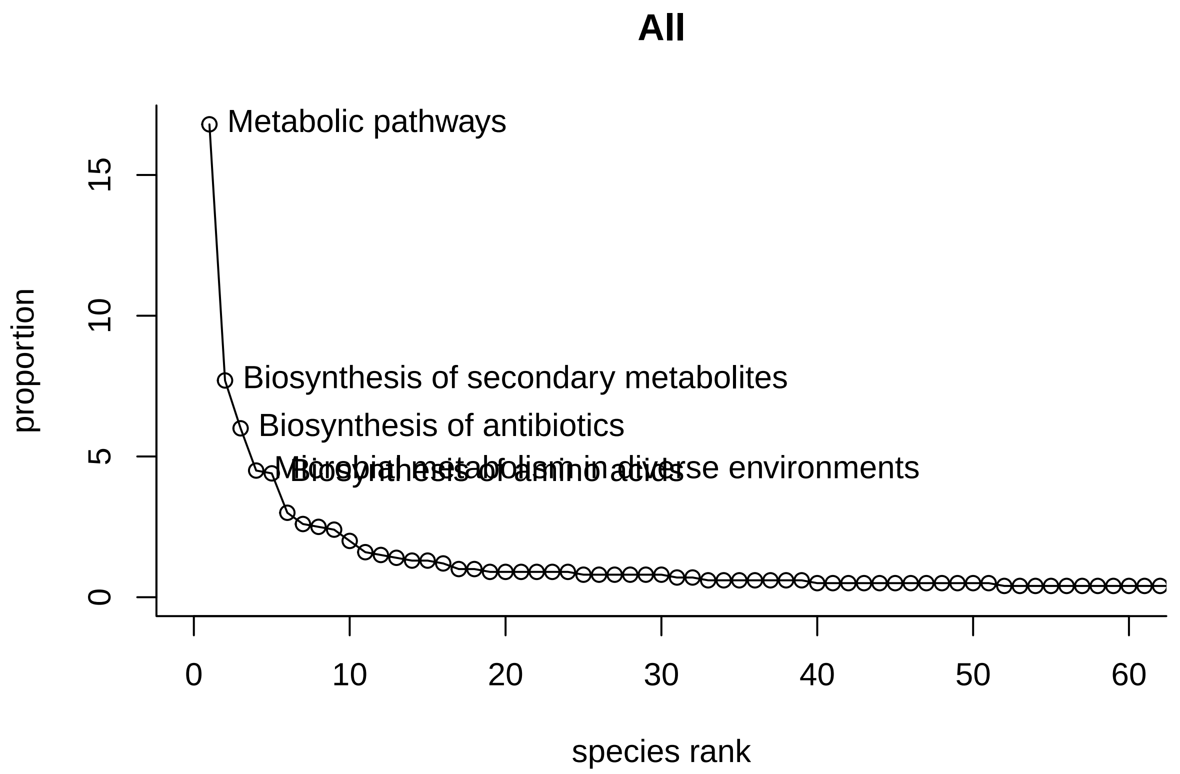

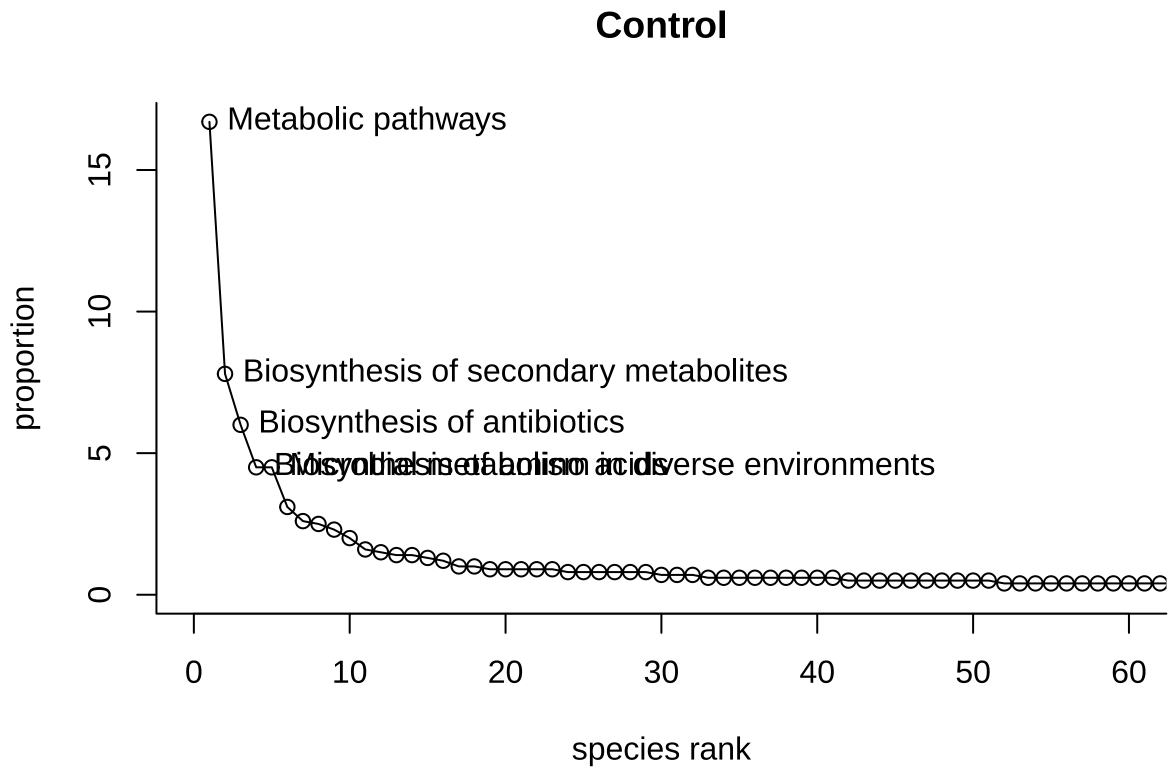
Control
Metabolic pathways
Biosynthesis of secondary metabolites
Biosynthesis of antibiotics
Biosynthesis of amino acids
Microbial metabolism in diverse environments
proportion
0
5
10
15
0
10
20
30
40
50
60
species rank

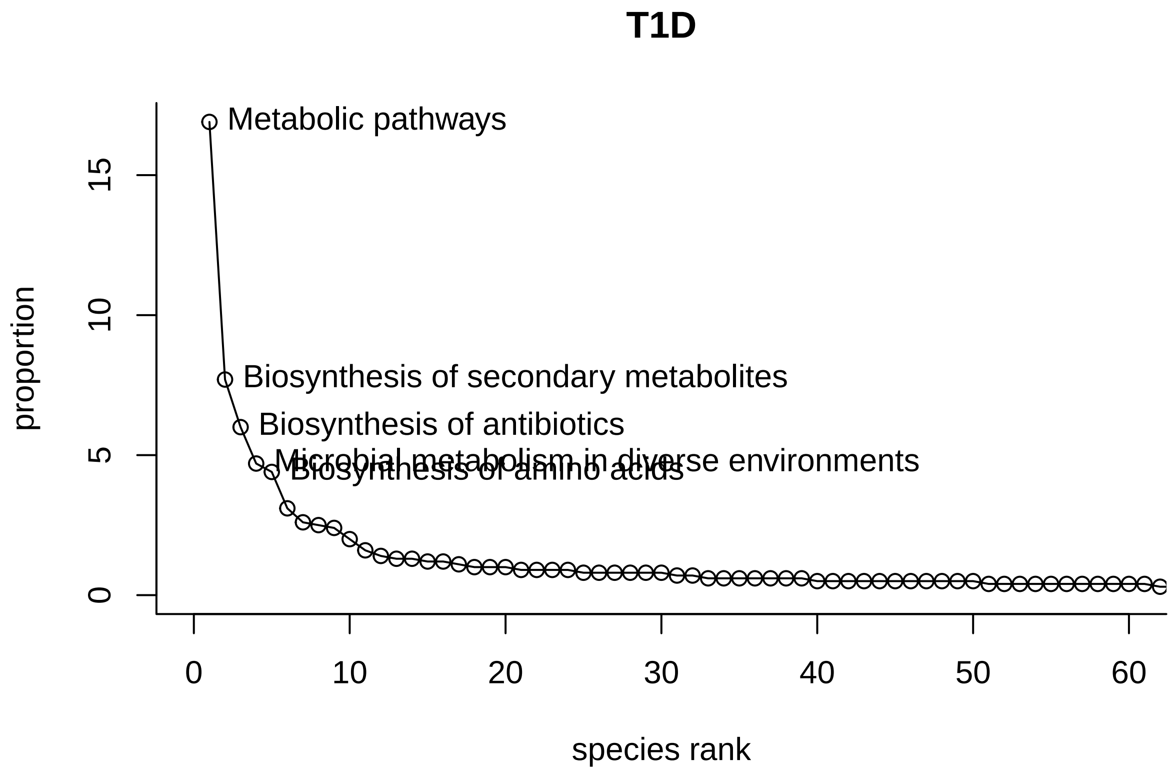
T1D
Metabolic pathways
Biosynthesis of secondary metabolites
Biosynthesis of antibiotics
Microbial metabolism in diverse environments
Biosynthesis of amino acids
proportion
0
5
10
15
0
10
20
30
40
50
60
species rank

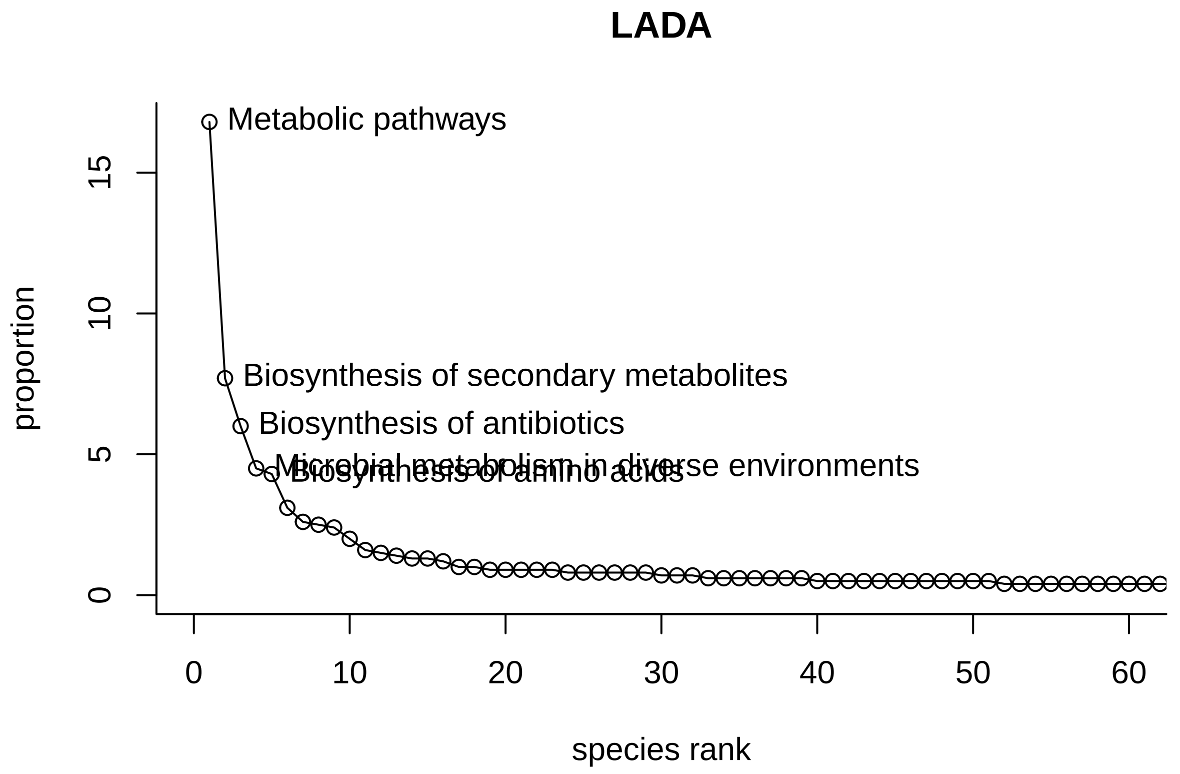

LADA
Metabolic pathways
Biosynthesis of secondary metabolites
Biosynthesis of antibiotics
Microbial metabolism in diverse environments
Biosynthesis of amino acids
proportion
0
5
10
15
0
10
20
30
40
50
60
species rank

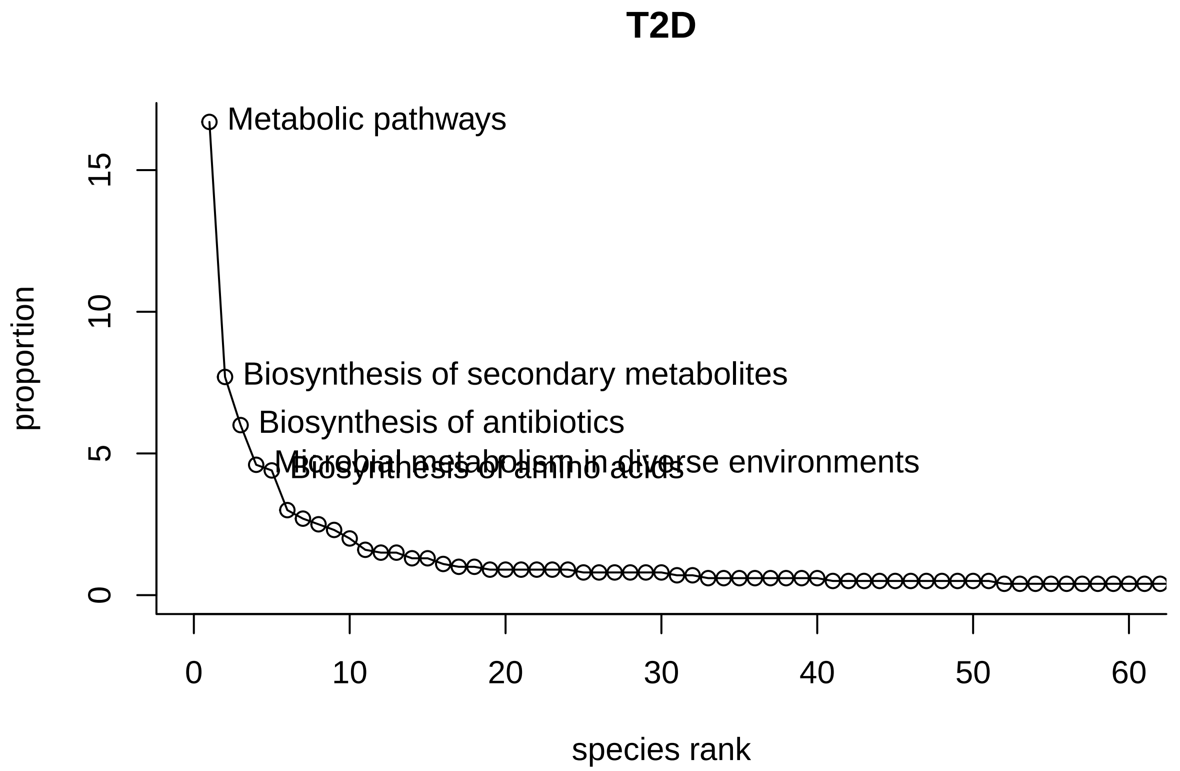


```r
#Additional metadata
MetadataEkstra <- read.csv(file="P:/CBMR/LADA/Text/Analysis/LADA_microbiome_extra_metadata.txt",
                          check.names=FALSE,
                          stringsAsFactors = FALSE,
                          strip.white=TRUE,
                          dec=".")

MetadataEkstra$MicrobiomeID <- paste("L", MetadataEkstra$MicrobiomeID,
                                     sep="")

#Merge with previous metadata
covariates <- merge(covariates, MetadataEkstra[,c(1,12:length(MetadataEkstra))])

#Aggregate diagnosis mean and sd
aggregate(covariates[, c(2:3, 12:length(covariates))], list(covariates$Diagnosis), mean,
          na.rm=TRUE)
```

```
##   Group.1      age      BMI     BMIq    CellNorm seq_depth     waist      hip
## 1 Control 61.44243 26.67011 2.042857 20384275367  40742480  92.89714 101.4143
## 2     T1D 51.73196 26.66011 2.066667 18731782275  42304822  98.30000 106.1833
## 3    LADA 63.76249 28.92226 2.583333 18940404238  41155438 103.21667 107.3333
## 4     T2D 61.42814 31.27686 3.071429 19624620327  41285784 107.45588 107.6324
##         whr      sbp      dbp hba1c_pct hba1c_mmol_mol      glu     chol
## 1 0.9160348 135.7643 81.03571  5.674286       38.47143 5.765714 5.444286
## 2 0.9241084 137.3500 75.98333  8.853333       73.26073 9.740000 4.860000
```

```
## 3 0.9602380 131.6278 77.80556  7.920580       63.06667 8.803333 4.201667
## 4 0.9978302 136.1159 79.89130  6.867143       51.55365 8.477941 4.651429
##        hdl      ldl     trig
## 1 1.369286 3.470000 1.344857
## 2 1.406333 2.703448 1.364333
## 3 1.331833 2.057627 1.729167
## 4 1.210429 2.537879 1.954857
```

```
aggregate(covariates[, c(2:3, 12:length(covariates))], list(covariates$Diagnosis), sd,
          na.rm=TRUE)
```

```
##   Group.1       age      BMI      BMIq   CellNorm seq_depth    waist      hip
## 1 Control 10.189143 3.722805 0.9696214 6781846681  17614052 10.80459 7.671692
## 2     T1D  6.961407 4.555865 1.2015316 5712095609  15783770 12.71206 7.790891
## 3    LADA 10.183290 4.866525 1.1393079 5544046390  16729647 14.49148 9.553891
## 4     T2D  9.956564 5.512931 0.9528208 6146706989  12799787 13.53319 9.787144
##          whr      sbp       dbp hba1c_pct hba1c_mmol_mol       glu     chol
## 1 0.08380676 18.33301 10.241052 0.3561817       3.907338 0.4845095 1.126574
## 2 0.08055011 21.20026 10.346150 0.8076075       8.826342 4.2322734 1.324725
## 3 0.09391664 17.78699  7.853962 1.1954893      13.065502 2.9649916 1.019054
## 4 0.08120027 16.47573 10.378098 1.2696823      13.876358 2.7289408 1.343017
##         hdl       ldl      trig
## 1 0.3772260 1.0489954 0.8167996
## 2 0.4137422 0.7248068 0.9030855
## 3 0.3909939 0.7788593 1.0163315
## 4 0.3592038 1.0221794 1.2446846
```

```
#Aggregate sex mean and sd
aggregate(covariates[, c(2:3, 12:length(covariates))], list(covariates$sex), mean,
          na.rm=TRUE)
```

```
##   Group.1      age      BMI     BMIq    CellNorm seq_depth     waist      hip
## 1       1 59.92969 28.55643 2.464789 20272037367  40593799 103.06549 103.8380
## 2       2 62.14355 28.82288 2.556818 18413308125  42228749  96.70349 108.1221
##         whr      sbp      dbp hba1c_pct hba1c_mmol_mol      glu     chol
## 1 0.9906106 135.4178 80.06338  7.048703       53.54204 7.912857 4.645775
## 2 0.8912291 134.3027 77.74521  7.020671       53.18873 7.871591 5.055682
##        hdl      ldl     trig
## 1 1.228028 2.644928 1.692183
## 2 1.457955 2.851163 1.538295
```

```
aggregate(covariates[, c(2:3, 12:length(covariates))], list(covariates$sex), sd,
          na.rm=TRUE)
```

```
##   Group.1      age      BMI     BMIq   CellNorm seq_depth    waist       hip
## 1       1 10.32654 4.689904 1.108714 6154527421  14646643 12.78662  7.423021
## 2       2 10.32517 5.701434 1.153286 5972255901  17323338 15.37737 11.148710
##          whr      sbp      dbp hba1c_pct hba1c_mmol_mol      glu     chol
## 1 0.07681929 16.90142  9.60506  1.469174       16.05979 2.926826 1.198499
## 2 0.07928849 19.87744 10.05909  1.503488       16.46194 3.134126 1.375973
##         hdl      ldl      trig
## 1 0.3401986 1.056925 1.1107317
## 2 0.4118005 1.116164 0.9472746
```

```r
#Test differences
df<-data.frame()
for (i in c(2:3, 12:length(covariates))) {
  design<-paste(colnames(covariates[i]), "~ Diagnosis")
  #Run kruskal-wallis test
  kwobject<-kruskal.test(formula(design), data=covariates)
  #print(kwobject)
  #Run follow up mann-whitney
  mwobject<-pairwise.wilcox.test(covariates[,i], covariates[,8],
                   p.adjust.method="bonferroni")
  #print(mwobject)
  #Bind all test
  df<-rbind(df, data.frame(kwobject$data.name, kwobject$statistic, kwobject$p.value,
                        mwobject$p.value[1,1], mwobject$p.value[2,1], mwobject$p.value[3,1],
                        mwobject$p.value[2,2], mwobject$p.value[3,2], mwobject$p.value[3,3]))

}

colnames(df)<-c("Design", "chistat", "kw p-value (p)", "mw p control vs T1D",
                "mw p control vs LADA", "mw p control vs T2D", "mw p T1D vs LADA",
                "mw p-value T1D vs T2D","mw p-value LADA vs T2D")
kable(df[, 1:5], row.names=FALSE)
```

Design	chistat	kw p-value (p)	mw p control vs T1D	mw p control vs LADA
age by Diagnosis	31.5293376	0.0000007	0.0000109	1.0000000
BMI by Diagnosis	37.3541663	0.0000000	1.0000000	0.0320433
BMIq by Diagnosis	34.4233168	0.0000002	1.0000000	0.0397857
CellNorm by Diagnosis	1.7778215	0.6197731	1.0000000	1.0000000
seq_depth by Diagnosis	0.5679933	0.9037189	1.0000000	1.0000000
waist by Diagnosis	41.5179712	0.0000000	0.5295941	0.0001757
hip by Diagnosis	22.4643067	0.0000522	0.0206165	0.0015338
whr by Diagnosis	29.2167844	0.0000020	1.0000000	0.0531195
sbp by Diagnosis	3.2759069	0.3510101	1.0000000	0.8702673
dbp by Diagnosis	7.5642457	0.0559305	0.1759817	0.1800562
hba1c_pct by Diagnosis	158.0896156	0.0000000	0.0000000	0.0000000
hba1c_mmol_mol by Diagnosis	157.7230843	0.0000000	0.0000000	0.0000000
glu by Diagnosis	95.3261632	0.0000000	0.0000018	0.0000000
chol by Diagnosis	40.8475487	0.0000000	0.0137855	0.0000000
hdl by Diagnosis	10.8681667	0.0124605	1.0000000	1.0000000
ldl by Diagnosis	57.7554562	0.0000000	0.0011958	0.0000000
trig by Diagnosis	20.3044253	0.0001468	1.0000000	0.0502674

```r
kable(df[, c(1, 6:9)], row.names=FALSE)
```

Design	mw p control vs T2D	mw p T1D vs LADA	mw p-value T1D vs T2D	mw p-value LADA vs T2D
age by Diagnosis	1.0000000	0.0000009	0.0000177	1.0000000
BMI by Diagnosis	0.0000001	0.1512350	0.0002367	0.0413942
BMIq by Diagnosis	0.0000001	0.2511605	0.0008957	0.0857981
CellNorm by Diagnosis	1.0000000	1.0000000	1.0000000	1.0000000

Design	mw p control vs T2D	mw p T1D vs LADA	mw p-value T1D vs T2D	mw p-value LADA vs T2D
seq_depth by Diagnosis	1.0000000	1.0000000	1.0000000	1.0000000
waist by Diagnosis	0.0000000	0.5796713	0.0157666	0.9439178
hip by Diagnosis	0.0001607	1.0000000	1.0000000	1.0000000
whr by Diagnosis	0.0000026	0.4417814	0.0009814	0.2648907
sbp by Diagnosis	1.0000000	1.0000000	1.0000000	0.6594205
dbp by Diagnosis	1.0000000	1.0000000	0.4748683	1.0000000
hba1c_pct by Diagnosis	0.0000000	0.0000212	0.0000000	0.0000000
hba1c_mmol_mol by Diagnosis	0.0000000	0.0000212	0.0000000	0.0000000
glu by Diagnosis	0.0000000	0.9620485	0.3009942	1.0000000
chol by Diagnosis	0.0003009	0.0396162	1.0000000	0.7831524
hdl by Diagnosis	0.0212935	1.0000000	0.0887728	0.2690728
ldl by Diagnosis	0.0000060	0.0032265	0.6392474	0.1175232
trig by Diagnosis	0.0005226	0.1705718	0.0098608	1.0000000

```
#Diagnostic criteria
T1D<-filter(covariates, Diagnosis=="T1D")
range(T1D$hba1c_pct)
```

```
## [1]  7.6 11.0
```

```
Controls<-filter(covariates, Diagnosis=="Control")
range(Controls$glu)
```

```
## [1] 4.2 6.8
```

```
range(Controls$hba1c_pct)
```

```
## [1] 4.8 6.2
```

Summary metadata, quality of data and ecology Metformin removed

```
rm(list=setdiff(ls(), c("Metadata", "MetadataMed", "Feature", "TaxonomyDA", "Taxonomy")))
##Removing treated with metformin
#Metadata$Metformin <- ifelse(is.na(Metadata$Metformin), "Unknown", Metadata$Metformin)
Metadata2<-filter(Metadata, !Metformin == 1)
##Applying subsetting to OTU tables
Taxonomy2<-dplyr::select(Taxonomy, one_of(as.character(Metadata2$MicrobiomeID)))
##Remove orgs that are not present after subsetting.
Taxonomy2 <- Taxonomy2[rowSums(Taxonomy2)>0,]


##Summary metadata and quality of data
#Adding cell count to infer absolute cell numbers
# old path path<-paste("Q:/",
#             "Projects/",
#             "LADA/",
```

```
#             "LADA_Sandra_Evelina/",
#             "LADA_JKV/",
#             "LADA_R_AfterFlow_Analysis_FinalCounts/",
#             "LADA_FinalCounts/",
#             "2019-09-03_CountAnalysis.rds", sep="")

path <- paste("J:/",
              "CBMR/",
              "SUN-CBMR-Hansen-Group/",
              "Projects/",
              "LADA/",
              "LADA_Sandra_Evelina/",
              "LADA_JKV/",
              "LADA_R_AfterFlow_Analysis_FinalCounts/",
              "LADA_FinalCounts/",
              "2019-09-03_CountAnalysis.rds", sep="")

TotalCounts<-readRDS(path)
TotalCountsProcess <-
  data.frame(MicrobiomeID=TotalCounts$DF_Save_w$ID,
             CellNorm=TotalCounts$DF_Save_w$Cells_per_g_feces_FR_corrected_mean)
TotalCountsProcess$MicrobiomeID <- paste("L", TotalCountsProcess$MicrobiomeID,
                                         sep="")
covariates<-merge(Metadata2, TotalCountsProcess, by="MicrobiomeID")

#One value is NaN in sample L602059 assigns value as average
covariates$CellNorm[is.nan(covariates$CellNorm)]<-
  mean(na.omit(covariates$CellNorm))

covariates<-merge(covariates, data.frame(seq_depth=colSums(Taxonomy2),
                                         MicrobiomeID=names(Taxonomy2)),
                  by="MicrobiomeID")
print("Sex: male=1, female=2")
```

```
## [1] "Sex: male=1, female=2"
```

```
table(Metadata2$sex, Metadata2$Diagnosis)
```

```
##
##     Control T1D LADA T2D
##   1      44  17    5  12
##   2      26  13    7  11
```

```
print("Metformin: No treatment=0, treatment=1")
```

```
## [1] "Metformin: No treatment=0, treatment=1"
```

```
table(Metadata2$Metformin, Metadata2$Diagnosis)
```

```
##
##     Control T1D LADA T2D
##   0      70  30   12  23
```

```r
print("Age, BMI and seq_depth")
```

```
## [1] "Age, BMI and seq_depth"
```

```r
aggregate(covariates[, c(2:3, 9:10)], list(covariates$Diagnosis), mean)
```

```
##   Group.1      age      BMI BMIord BMIclass
## 1 Control 61.44243 26.67011     NA       NA
## 2     T1D 51.73196 26.66011     NA       NA
## 3    LADA 66.61670 28.20713     NA       NA
## 4     T2D 63.68304 31.49826     NA       NA
```

```r
aggregate(covariates[, c(2:3, 9:10)], list(covariates$Diagnosis), sd)
```

```
##   Group.1       age      BMI BMIord BMIclass
## 1 Control 10.189143 3.722805     NA       NA
## 2     T1D  6.961407 4.555865     NA       NA
## 3    LADA 12.720776 5.022780     NA       NA
## 4     T2D  8.549262 5.608501     NA       NA
```

```r
##Look into metadata if patient received metformin or not
print("Sex: male=1, female=2. Metformin: No treatment=0, treatment=1")
```

```
## [1] "Sex: male=1, female=2. Metformin: No treatment=0, treatment=1"
```

```r
table(Metadata2$sex, Metadata2$Metformin)
```

```
##
##      0
##   1 78
##   2 57
```

```r
print("Age, BMI and seq_depth")
```

```
## [1] "Age, BMI and seq_depth"
```

```r
aggregate(covariates[, c(2:3, 9:10)], list(covariates$Metformin), mean)
```

```
##   Group.1      age      BMI BMIord BMIclass
## 1       0 60.12622 27.62709     NA       NA
```

```r
aggregate(covariates[, c(2:3, 9:10)], list(covariates$Metformin), sd)
```

```
##   Group.1      age      BMI BMIord BMIclass
## 1       0 10.57613 4.706784     NA       NA
```

```r
##Testing covariates
#Kruskal test
kruskal.test(BMI ~ Diagnosis, data=covariates)
```

```
##
##  Kruskal-Wallis rank sum test
##
## data:  BMI by Diagnosis
## Kruskal-Wallis chi-squared = 16.525, df = 3, p-value = 0.0008847
```

```r
kruskal.test(age ~ Diagnosis, data=covariates)
```

```
##
##  Kruskal-Wallis rank sum test
##
## data:  age by Diagnosis
## Kruskal-Wallis chi-squared = 30.881, df = 3, p-value = 9.006e-07
```

```r
kruskal.test(CellNorm ~ Diagnosis, data=covariates)
```

```
##
##  Kruskal-Wallis rank sum test
##
## data:  CellNorm by Diagnosis
## Kruskal-Wallis chi-squared = 9.1305, df = 3, p-value = 0.02761
```

```r
kruskal.test(seq_depth ~ Diagnosis, data=covariates)
```

```
##
##  Kruskal-Wallis rank sum test
##
## data:  seq_depth by Diagnosis
## Kruskal-Wallis chi-squared = 1.0785, df = 3, p-value = 0.7823
```

```r
#If significant Mann-Whitney pairwise post-test
pairwise.wilcox.test(covariates$BMI, covariates$Diagnosis,
                     p.adjust.method="bonferroni")
```

```
##
##  Pairwise comparisons using Wilcoxon rank sum test with continuity correction
##
## data:  covariates$BMI and covariates$Diagnosis
##
##      Control T1D     LADA
## T1D  1.00000 -       -
## LADA 1.00000 1.00000 -
## T2D  0.00059 0.00504 0.68860
##
## P value adjustment method: bonferroni
```

```r
pairwise.wilcox.test(covariates$age, covariates$Diagnosis,
                     p.adjust.method="bonferroni")
```

```
##
##  Pairwise comparisons using Wilcoxon rank sum test with continuity correction
##
## data:  covariates$age and covariates$Diagnosis
##
##      Control T1D     LADA
## T1D  1.1e-05 -       -
## LADA 0.79832 0.00057 -
## T2D  1.00000 7.8e-06 1.00000
##
## P value adjustment method: bonferroni
```

```r
pairwise.wilcox.test(covariates$seq_depth, covariates$Diagnosis,
                     p.adjust.method="bonferroni")
```

```
##
##  Pairwise comparisons using Wilcoxon rank sum test with continuity correction
##
## data:  covariates$seq_depth and covariates$Diagnosis
##
##      Control T1D LADA
## T1D  1       -   -
## LADA 1       1   -
## T2D  1       1   1
##
## P value adjustment method: bonferroni
```

```r
##Plot rarefaction curves can decrease step for final plotting
#set.seed(1)
#rarecurve(t(Taxonomy), step=100, xlab="Annotated reads",
#          ylab="Genera", label=FALSE)

#Create rarefaction curves with colors according to categories
#Adding colors to the rarecurves according to experiment, Controls=#666666 (blue),
#T1D=#1F78B4 (red), T2D=#33A02C (forestgreen), LADA=#E31A1C (gold)
rare<-data.frame(t(Taxonomy2))
rare<-add_rownames(rare, "MicrobiomeID")
rare<-merge(rare, Metadata2, by="MicrobiomeID")
rare$colors<-ifelse(grepl("0", rare$group), "#666666",
                    ifelse(grepl("1", rare$group), "#1F78B4",
                           ifelse(grepl("2", rare$group), "#33A02C",
                                  ifelse(grepl("4", rare$group),
                                         "#E31A1C", "pink"))))
#table(rare$colors)
rare$line<-ifelse(grepl("0", rare$Metformin), "solid",
                  ifelse(grepl("1", rare$Metformin), "dotted", "longdash"))
#table(rare$line)

#Check order
```

```r
if (setequal(colnames(Taxonomy2), rare$MicrobiomeID)==FALSE) {
  stop("Taxonomy and Metadata is not corresponding with each other")
}

set.seed(1)
rarecurve(t(Taxonomy2), step=100000, xlab="Annotated reads", ylab="Genera",
          col=rare$colors,
          lty=rare$line, label=FALSE) #piphillin pathways 100000, else 100
```

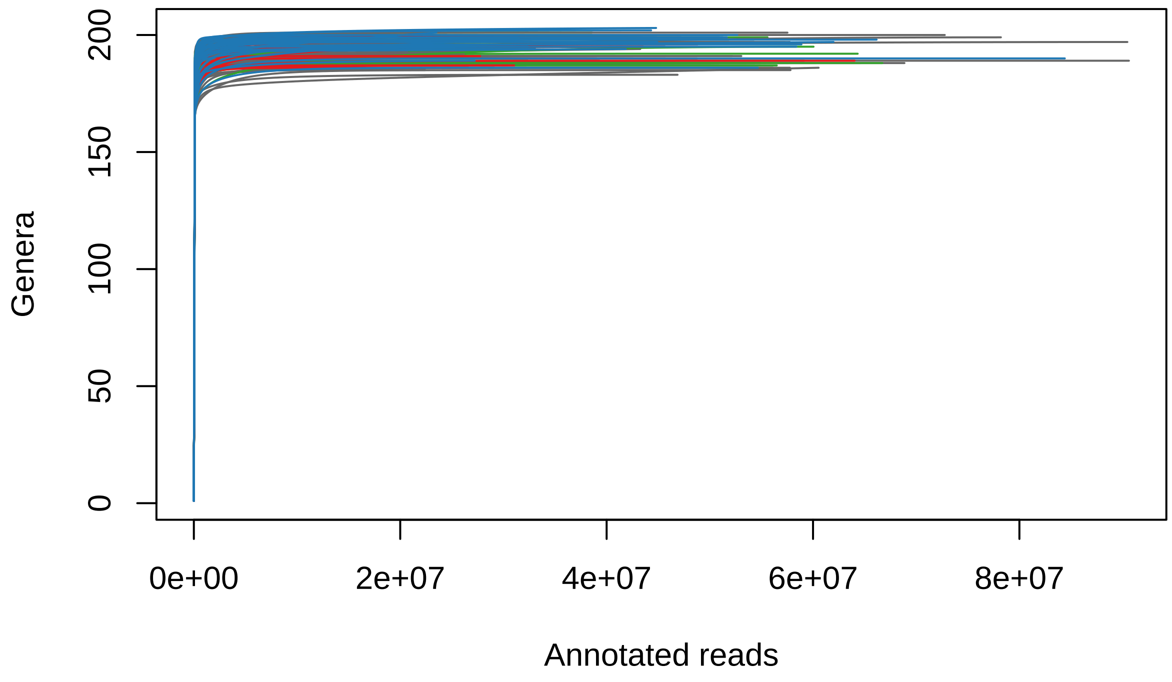


```r
pdf(paste("MicroLADA_Rarecurve_MetRem_Func.pdf", sep=""), height=6, width=8)
rarecurve(t(Taxonomy2), step=100000, xlab="Annotated reads", ylab="Genera",
          col=rare$colors,
          lty=rare$line, label=FALSE)
dev.off()
```

```
## pdf
##   2
```

```r
##Rank abundance curves
#Total sum scaling (Use relative abundances)
Taxonomy2<-sweep(Taxonomy2, 2, colSums(Taxonomy2), FUN="/")

##RAC all and diagnostic groupings
pdf(paste("MicroLADA_RAC_MetRem_Func", ".pdf", sep=""), width=12, height=6)
```

```
par(mfrow=c(1,5))
Subset <- c("All", "Control", "T1D", "LADA", "T2D")

for (i in Subset) {
  #Subsetting Metadata according to
  if (i=="All") {
    Metadata3 <- Metadata2
  } else if (i=="Control") {
    Metadata3<-filter(Metadata2, Diagnosis == "Control")
  } else if (i=="T1D") {
    Metadata3<-filter(Metadata2, Diagnosis == "T1D")
  } else if (i=="LADA") {
    Metadata3<-filter(Metadata2, Diagnosis == "LADA")
  } else if (i=="T2D") {
    Metadata3<-filter(Metadata2, Diagnosis == "T2D")
  } else {
    print("Subset defined not valid")
  }

  #Applying subsetting to OTU tables, have already TSS
  Taxonomy3<-dplyr::select(Taxonomy2, one_of(as.character(Metadata3$MicrobiomeID)))
  #Setting up ranks
  RankAbun.1 <- rankabundance(t(Taxonomy3))
  #Create RACplot
  rankabunplot(RankAbun.1, scale='proportion', addit=FALSE, specnames=c(1,2,3,4,5),
               xlim=c(0,60), main=i)
}
dev.off()
```

```
## pdf
##   2
```

```
#Include in pdf
Subset <- c("All", "Control", "T1D", "LADA", "T2D")
for (i in Subset) {
  #Subsetting Metadata according to
  if (i=="All") {
    Metadata3 <- Metadata2
  } else if (i=="Control") {
    Metadata3<-filter(Metadata2, Diagnosis == "Control")
  } else if (i=="T1D") {
    Metadata3<-filter(Metadata2, Diagnosis == "T1D")
  } else if (i=="LADA") {
    Metadata3<-filter(Metadata2, Diagnosis == "LADA")
  } else if (i=="T2D") {
    Metadata3<-filter(Metadata2, Diagnosis == "T2D")
  } else {
    print("Subset defined not valid")
  }

  #Applying subsetting to OTU tables, have already TSS
  Taxonomy3<-dplyr::select(Taxonomy2, one_of(as.character(Metadata3$MicrobiomeID)))
  #Setting up ranks
```

```
  RankAbun.1 <- rankabundance(t(Taxonomy3))
  #Create RACplot
  rankabunplot(RankAbun.1, scale='proportion', addit=FALSE, specnames=c(1,2,3,4,5),
               xlim=c(0,60), main=i)
}
```

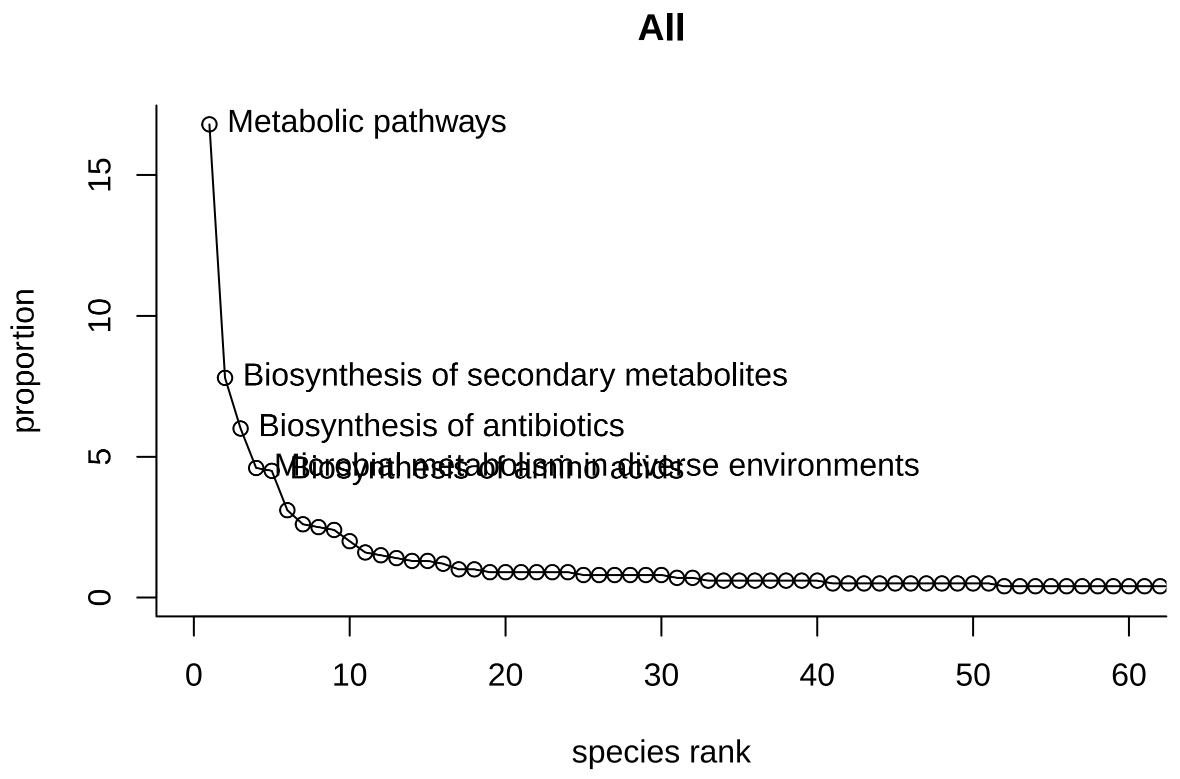

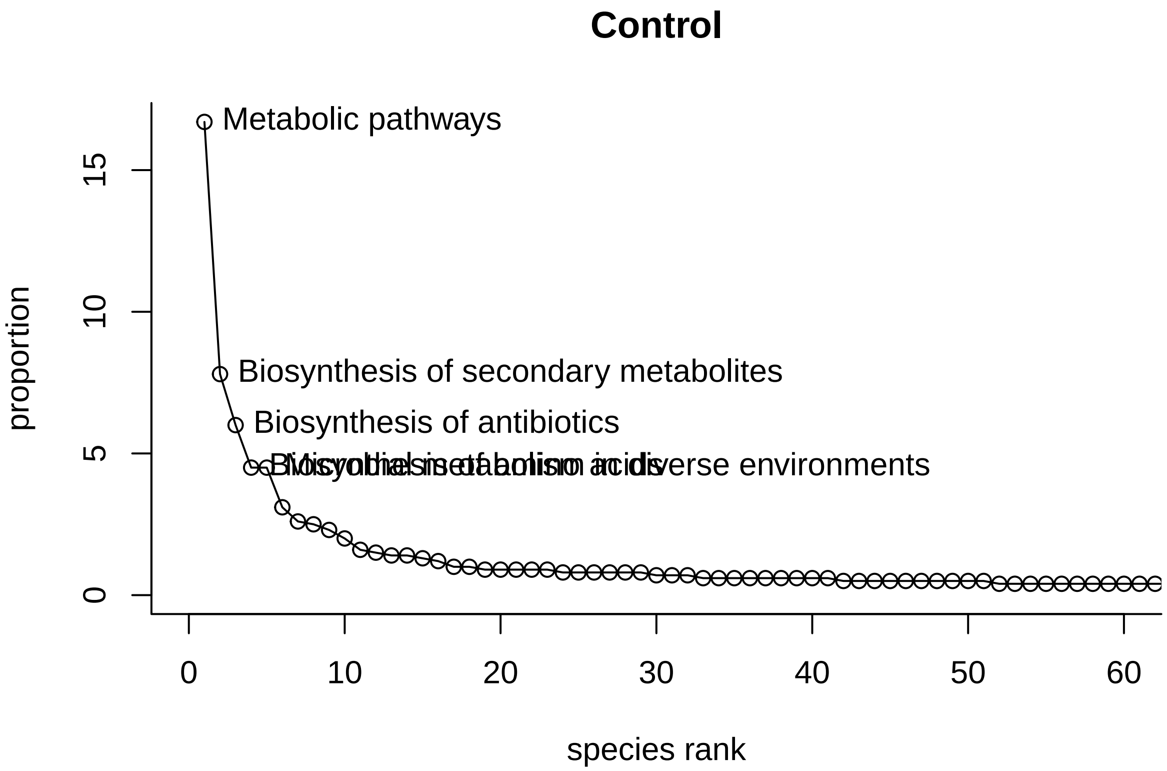
Control
Metabolic pathways
Biosynthesis of secondary metabolites
Biosynthesis of antibiotics
diverse environments
proportion
0
5
10
15
0
10
20
30
40
50
60
species rank

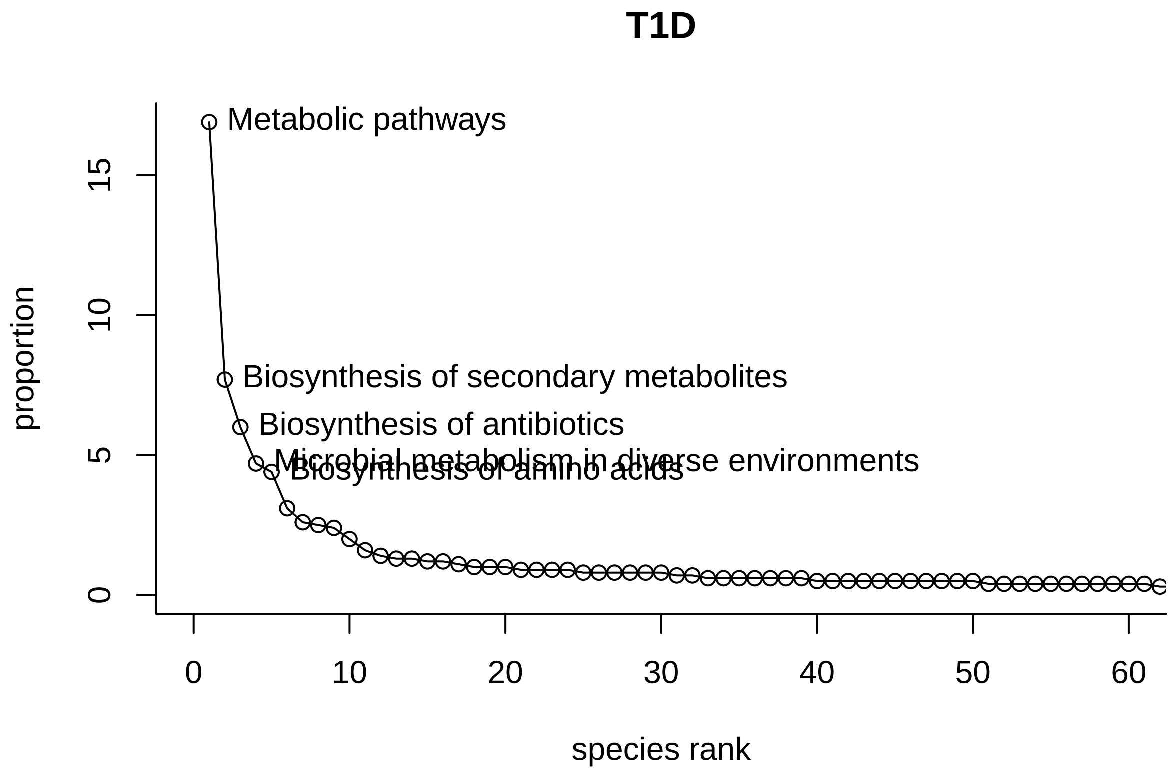
T1D
Metabolic pathways
Biosynthesis of secondary metabolites
Biosynthesis of antibiotics
Microbial metabolism in diverse environments
Biosynthesis of amino acids
proportion
species rank
0
5
10
15
0
10
20
30
40
50
60

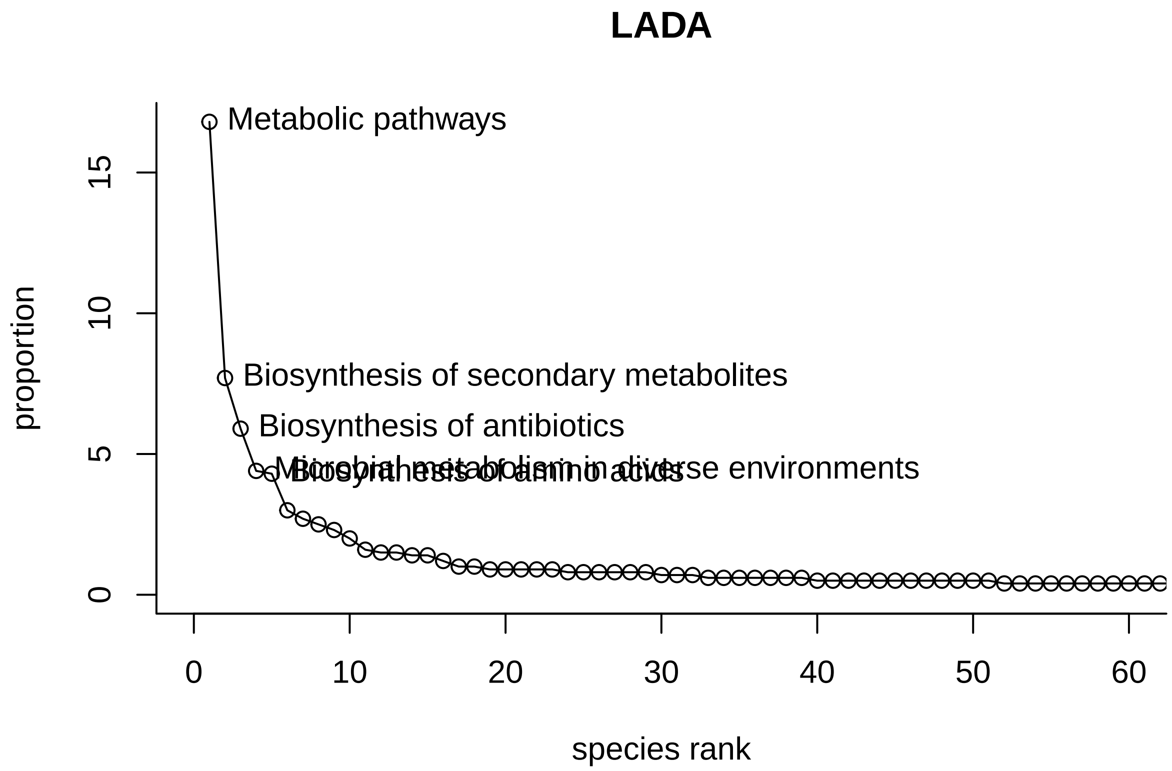
LADA
Metabolic pathways
Biosynthesis of secondary metabolites
Biosynthesis of antibiotics
Microbial metabolism in diverse environments
Biosynthesis of amino acids
proportion
0
5
10
15
0
10
20
30
40
50
60
species rank

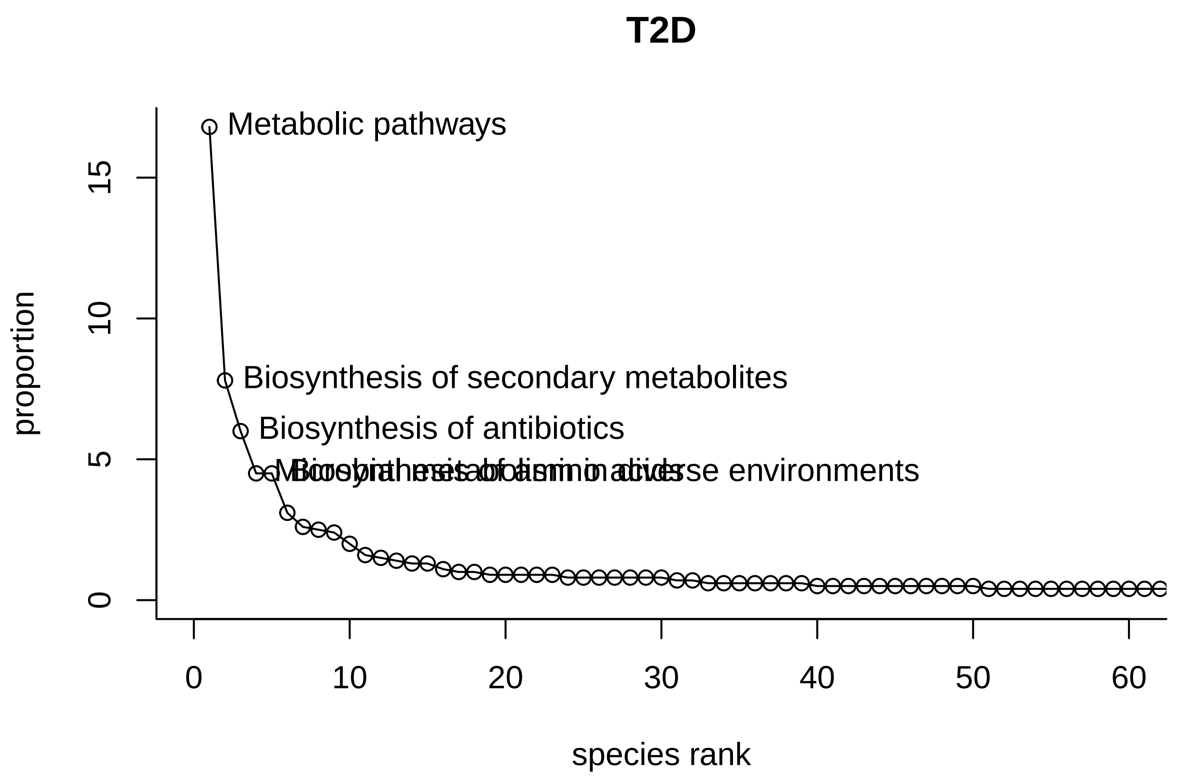


```
#Additional metadata
MetadataEkstra <- read.csv(file="P:/CBMR/LADA/Text/Analysis/LADA_microbiome_extra_metadata.txt",
                       check.names=FALSE,
                       stringsAsFactors = FALSE,
                       strip.white=TRUE,
                       dec=".")

MetadataEkstra$MicrobiomeID <- paste("L", MetadataEkstra$MicrobiomeID,
                                     sep="")
#Remove metformin
MetadataEkstra$Metformin <- ifelse(is.na(MetadataEkstra$Metformin), "Unknown", MetadataEkstra$Metformin)
MetadataEkstra<-filter(MetadataEkstra, !Metformin == 1)

#Merge with previous metadata
covariates <- merge(covariates, MetadataEkstra[,c(1,12:length(MetadataEkstra))])

#Aggregate diagnosis mean and sd
aggregate(covariates[, c(2:3, 12:length(covariates))], list(covariates$Diagnosis), mean,
          na.rm=TRUE)
```

```
##   Group.1      age      BMI     BMIq    CellNorm seq_depth     waist      hip
## 1 Control 61.44243 26.67011 2.042857 20384275367  40742480  92.89714 101.4143
## 2     T1D 51.73196 26.66011 2.066667 18731782275  42304822  98.30000 106.1833
## 3    LADA 66.61670 28.20713 2.416667 15590817082  37128727  97.41667 105.7500
## 4     T2D 63.68304 31.49826 3.130435 16982538343  39826225 109.14286 109.8571
```

```
##         whr      sbp      dbp hba1c_pct hba1c_mmol_mol       glu     chol
## 1 0.9160348 135.7643 81.03571  5.674286       38.47143  5.765714 5.444286
## 2 0.9241084 137.3500 75.98333  8.853333       73.26073  9.740000 4.860000
## 3 0.9171111 135.3056 75.30556  8.646477       71.00000 10.050000 4.258333
## 4 0.9917660 137.9318 80.43182  6.882609       51.72268  7.795652 5.113043
##        hdl      ldl     trig
## 1 1.369286 3.470000 1.344857
## 2 1.406333 2.703448 1.364333
## 3 1.441667 2.358333 1.203333
## 4 1.306957 3.040909 1.770870
```

```r
aggregate(covariates[, c(2:3, 12:length(covariates))], list(covariates$Diagnosis), sd,
          na.rm=TRUE)
```

```
##   Group.1       age      BMI      BMIq   CellNorm seq_depth    waist      hip
## 1 Control 10.189143 3.722805 0.9696214 6781846681  17614052 10.80459 7.671692
## 2     T1D  6.961407 4.555865 1.2015316 5712095609  15783770 12.71206 7.790891
## 3    LADA 12.720776 5.022780 1.2401124 4604942130  12472316 16.89787 9.196096
## 4     T2D  8.549262 5.608501 1.0137396 4888235680  14549344 15.22592 9.323549
##          whr      sbp       dbp hba1c_pct hba1c_mmol_mol       glu      chol
## 1 0.08380676 18.33301 10.241052 0.3561817       3.907338 0.4845095 1.1265744
## 2 0.08055011 21.20026 10.346150 0.8076075       8.826342 4.2322734 1.3247251
## 3 0.11090078 27.12316  7.624334 1.4023922      15.326744 3.5783440 0.8743396
## 4 0.09003109 15.77317  9.861295 1.2730251      13.912891 2.7111685 1.3548916
##         hdl       ldl      trig
## 1 0.3772260 1.0489954 0.8167996
## 2 0.4137422 0.7248068 0.9030855
## 3 0.4074942 0.8328029 0.5037375
## 4 0.3891300 1.1537348 1.0565162
```

```r
#Aggregate sex mean and sd
aggregate(covariates[, c(2:3, 12:length(covariates))], list(covariates$sex), mean,
          na.rm=TRUE)
```

```
##   Group.1      age      BMI     BMIq    CellNorm seq_depth     waist      hip
## 1       1 58.85014 27.72057 2.243590 19682450132  40385659 100.02949 102.8718
## 2       2 61.87243 27.49916 2.298246 18093154746  40922539  92.91818 106.1182
##         whr      sbp      dbp hba1c_pct hba1c_mmol_mol      glu     chol
## 1 0.9703762 136.8226 81.09615  6.773460       50.53729 7.182051 5.023077
## 2 0.8725230 135.8929 76.77976  6.956629       52.46553 7.640351 5.329825
##        hdl      ldl     trig
## 1 1.260128 3.109091 1.481923
## 2 1.528246 3.162500 1.309649
```

```r
aggregate(covariates[, c(2:3, 12:length(covariates))], list(covariates$sex), sd,
          na.rm=TRUE)
```

```
##   Group.1      age      BMI     BMIq   CellNorm seq_depth    waist       hip
## 1       1 10.64268 4.322535 1.118741 6322346025  15361771 12.35226  7.233327
## 2       2 10.32174 5.224095 1.133341 6104790238  17457682 14.57140 10.077002
##          whr      sbp      dbp hba1c_pct hba1c_mmol_mol      glu     chol
## 1 0.07398661 17.32918  9.446039  1.624815       17.76417 2.929995 1.109689
```

```
## 2 0.07895551 21.95524 10.706984  1.561148       17.10433 3.298477 1.385507
##         hdl       ldl      trig
## 1 0.3359943 1.054088 0.8860859
## 2 0.4024883 1.064905 0.8395066
```

```
#Test differences
df<-data.frame()
for (i in c(2:3, 12:length(covariates))) {
  design<-paste(colnames(covariates[i]), "~ Diagnosis")
  #Run kruskal-wallis test
  kwobject<-kruskal.test(formula(design), data=covariates)
  #print(kwobject)
  #Run follow up mann-whitney
  mwobject<-pairwise.wilcox.test(covariates[,i], covariates[,8],
                   p.adjust.method="bonferroni")
  #print(mwobject)
  #Bind all test
  df<-rbind(df, data.frame(kwobject$data.name, kwobject$statistic, kwobject$p.value,
                          mwobject$p.value[1,1], mwobject$p.value[2,1], mwobject$p.value[3,1],
                          mwobject$p.value[2,2], mwobject$p.value[3,2], mwobject$p.value[3,3]))

}

colnames(df)<-c("Design", "chistat", "kw p-value (p)", "mw p control vs T1D",
                "mw p control vs LADA", "mw p control vs T2D", "mw p T1D vs LADA",
                "mw p-value T1D vs T2D","mw p-value LADA vs T2D")
kable(df[, 1:5], row.names=FALSE)
```

Design	chistat	kw p-value (p)	mw p control vs T1D	mw p control vs LADA
age by Diagnosis	30.8807870	0.0000009	0.0000109	0.7983155
BMI by Diagnosis	16.5253521	0.0008847	1.0000000	1.0000000
BMIq by Diagnosis	16.6786850	0.0008228	1.0000000	1.0000000
CellNorm by Diagnosis	9.1304607	0.0276057	1.0000000	0.0929905
seq_depth by Diagnosis	1.0784511	0.7822784	1.0000000	1.0000000
waist by Diagnosis	17.8198774	0.0004791	0.5295941	1.0000000
hip by Diagnosis	18.6119098	0.0003289	0.0206165	0.7844861
whr by Diagnosis	9.3836893	0.0246015	1.0000000	1.0000000
sbp by Diagnosis	0.5651568	0.9043605	1.0000000	1.0000000
dbp by Diagnosis	7.4063630	0.0600138	0.1759817	0.2760244
hba1c_pct by Diagnosis	98.4356210	0.0000000	0.0000000	0.0000002
hba1c_mmol_mol by Diagnosis	98.3646295	0.0000000	0.0000000	0.0000002
glu by Diagnosis	52.7694420	0.0000000	0.0000018	0.0000338
chol by Diagnosis	17.6586322	0.0005172	0.0137855	0.0012148
hdl by Diagnosis	1.5100365	0.6799559	1.0000000	1.0000000
ldl by Diagnosis	18.9521978	0.0002797	0.0011958	0.0060282
trig by Diagnosis	5.8578350	0.1187355	1.0000000	1.0000000

```
kable(df[, c(1, 6:9)], row.names=FALSE)
```

Design	mw p control vs T2D	mw p T1D vs LADA	mw p-value T1D vs T2D	mw p-value LADA vs T2D
age by Diagnosis	1.0000000	0.0005720	0.0000078	1.0000000
BMI by Diagnosis	0.0005874	1.0000000	0.0050354	0.6886029
BMIq by Diagnosis	0.0003140	1.0000000	0.0121791	0.5623014
CellNorm by Diagnosis	0.1808303	0.6998477	1.0000000	1.0000000
seq_depth by Diagnosis	1.0000000	1.0000000	1.0000000	1.0000000
waist by Diagnosis	0.0001944	1.0000000	0.1050003	0.7471481
hip by Diagnosis	0.0011702	1.0000000	1.0000000	1.0000000
whr by Diagnosis	0.0164348	1.0000000	0.0927908	0.5107970
sbp by Diagnosis	1.0000000	1.0000000	1.0000000	1.0000000
dbp by Diagnosis	1.0000000	1.0000000	0.7308104	1.0000000
hba1c_pct by Diagnosis	0.0000000	1.0000000	0.0000376	0.0046811
hba1c_mmol_mol by Diagnosis	0.0000000	1.0000000	0.0000376	0.0046811
glu by Diagnosis	0.0000006	1.0000000	0.1712058	0.3220620
chol by Diagnosis	1.0000000	0.2505788	1.0000000	0.8339288
hdl by Diagnosis	1.0000000	1.0000000	1.0000000	1.0000000
ldl by Diagnosis	1.0000000	1.0000000	1.0000000	0.6979424
trig by Diagnosis	0.1357372	1.0000000	0.3643004	0.4408450

```
#Diagnostic criteria
T1D<-filter(covariates, Diagnosis=="T1D")
range(T1D$hba1c_pct)
```

```
## [1]  7.6 11.0
```

```
Controls<-filter(covariates, Diagnosis=="Control")
range(Controls$glu)
```

```
## [1] 4.2 6.8
```

```
range(Controls$hba1c_pct)
```

```
## [1] 4.8 6.2
```

Alpha diversity

Part of figure 1

```
rm(list=setdiff(ls(), c("Metadata", "MetadataMed", "Feature", "TaxonomyDA", "Taxonomy")))

#Calculate alpha diversity stats
#Use vegan to calculate various diversity and richness indices for each sample
set.seed(1)
diversityCalc <- data.frame(Shannon=diversity(t(Taxonomy), index="shannon"),
                            Simpson=diversity(t(Taxonomy), index="simpson"),
                            invSimpson=diversity(t(Taxonomy), index="invsimpson"),
                            fisher=fisher.alpha(t(Taxonomy)),
                            richness=specnumber(t(Taxonomy)),
```

```r
                             rarefy_min_count=rarefy(t(Taxonomy),
                                                     sample=min(rowSums(t(Taxonomy)))),
                             chao1=estimateR(t(Taxonomy))["S.chao1",],
                             chao1SE=estimateR(t(Taxonomy))["se.chao1",],
                             #ShannonRar=diversity(rrarefy(data.frame(t(Taxonomy)),
                             #                               min(rowSums(t(Taxonomy)))),
                             #                       index="shannon"),
                             #SimpsonRar=diversity(rrarefy(data.frame(t(Taxonomy)),
                             #                               min(rowSums(t(Taxonomy)))),
                             #                       index="simpson"),
                             #invSimpsonRar=diversity(rrarefy(data.frame(t(Taxonomy)),
                             #                                  min(rowSums(t(Taxonomy)))),
                             #                          index="invsimpson"),
                             Pielou=diversity(t(Taxonomy))/log(specnumber(t(Taxonomy))))
#Merge with metadata.
diversityCalc<-add_rownames(diversityCalc, "MicrobiomeID")
Metadata2 <- merge(Metadata, diversityCalc, by="MicrobiomeID")

#Create a list to hold the plot objects.
Fig1List <- list()
#Create vector to loop
#AlphaDiv<-c("fisher", "Shannon", "Simpson", "invSimpson", "richness", "chao1",
#             "ShannonRar", "SimpsonRar", "invSimpsonRar", "Pielou")
AlphaDiv<-c("fisher", "Shannon", "Simpson", "invSimpson", "richness", "chao1", "Pielou")
#Plot
for (i in AlphaDiv) {
#Create plot name
pltName <- paste('Alpha', i, sep = '')
#create boxplots
Fig1List[[ pltName ]]<-
ggplot(Metadata2, aes_string(x="Diagnosis", y=i, group="Diagnosis")) +
    geom_violin(aes(fill=Diagnosis, trim=FALSE)) +
    stat_summary(fun.data="mean_sdl",
                 mult=1, #mean plus minus a constant (mult=1) times the st.dev
                 geom="pointrange",
                 width=0.2 ) +
    #stat_summary(fun.y = mean, geom = "point") +
    #facet_grid(. ~ Metformin, scales="fixed") + #Scales can also be "free"
    #ggtitle(paste("Genus", i, sep=" ")) +
    #xlab("Diagnosis") +
    ylab(paste(i)) +
    scale_fill_manual(values=c(Control = "#666666", T1D = "#1F78B4",
                               T2D = "#33A02C", LADA = "#E31A1C")) +
    theme_bw() +
    theme(legend.position="none", panel.grid.major = element_blank(),
          panel.grid.minor = element_blank(), axis.title=element_text(size=20),
          axis.title.x = element_blank(),
          axis.text.x = element_text(angle = 45, hjust = 1, size=16),
          axis.text.y = element_text(angle = 45, hjust = 1, size=12))
}


#Have the plots stored in lists
```

```r
lay <- rbind(c(1,2,3))
pdf(paste("MicroLADA_Alpha_Func", ".pdf", sep=""), width=12, height=6)
grid.arrange(Fig1List$Alpharichness,
             Fig1List$AlphaPielou, Fig1List$AlphaShannon, layout_matrix = lay)
dev.off()
```

```
## pdf
##   2
```

```r
grid.arrange(Fig1List$Alpharichness,
             Fig1List$AlphaPielou, Fig1List$AlphaShannon, layout_matrix = lay)
```

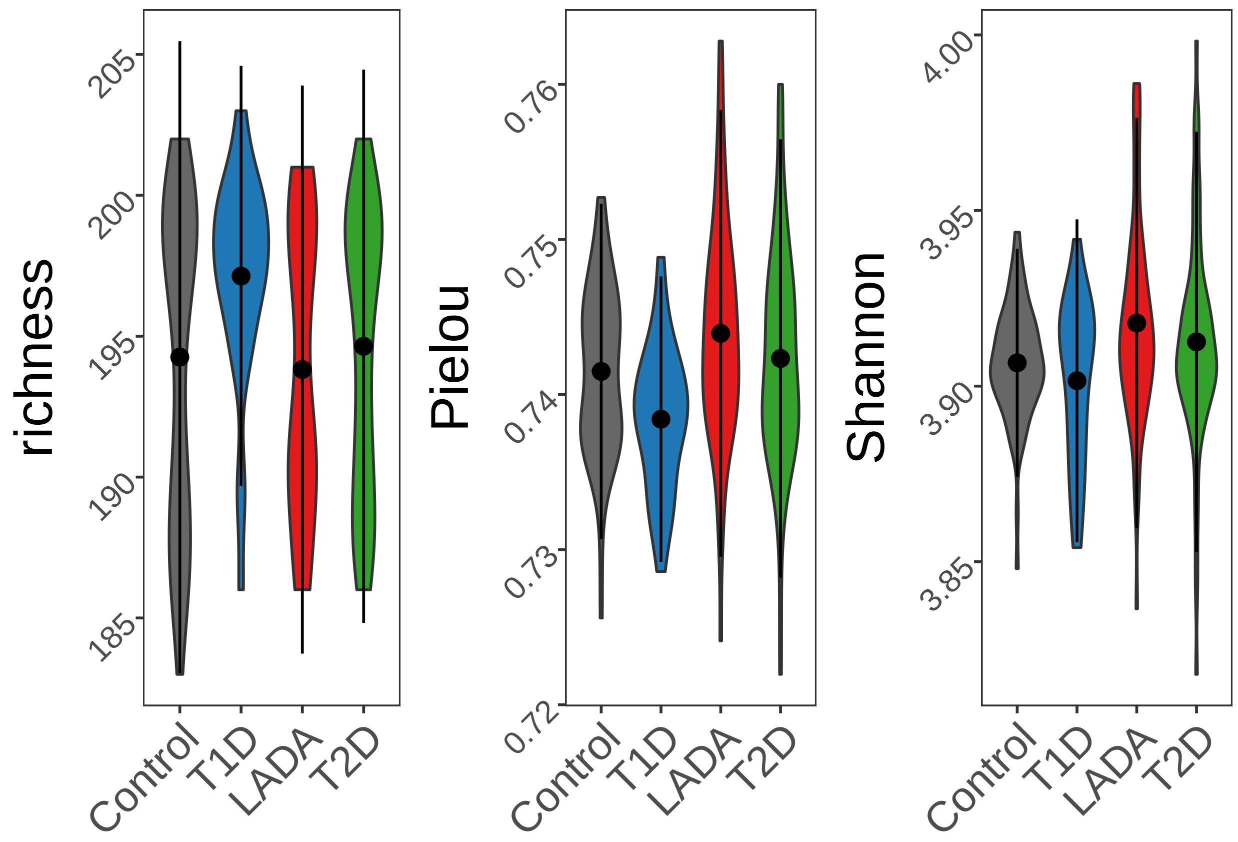


```r
#Kruskal test
kruskal.test(richness ~ Diagnosis, data=Metadata2)
```

```
##
##  Kruskal-Wallis rank sum test
##
## data:  richness by Diagnosis
## Kruskal-Wallis chi-squared = 6.3418, df = 3, p-value = 0.09611
```

```r
kruskal.test(Pielou ~ Diagnosis, data=Metadata2)
```

```
##
##  Kruskal-Wallis rank sum test
##
## data:  Pielou by Diagnosis
## Kruskal-Wallis chi-squared = 15.267, df = 3, p-value = 0.001602
```

```r
kruskal.test(Shannon ~ Diagnosis, data=Metadata2)
```

```
##
##  Kruskal-Wallis rank sum test
##
## data:  Shannon by Diagnosis
## Kruskal-Wallis chi-squared = 7.7326, df = 3, p-value = 0.05187
```

```r
#If significant Mann-Whitney pairwise post-test
pairwise.wilcox.test(Metadata2$richness, Metadata2$Diagnosis,
                     p.adjust.method="bonferroni")
```

```
##
##  Pairwise comparisons using Wilcoxon rank sum test with continuity correction
##
## data:  Metadata2$richness and Metadata2$Diagnosis
##
##      Control T1D   LADA
## T1D  0.366   -     -
## LADA 1.000   0.058 -
## T2D  1.000   0.250 1.000
##
## P value adjustment method: bonferroni
```

```r
pairwise.wilcox.test(Metadata2$Pielou, Metadata2$Diagnosis,
                     p.adjust.method="bonferroni")
```

```
##
##  Pairwise comparisons using Wilcoxon rank sum test with continuity correction
##
## data:  Metadata2$Pielou and Metadata2$Diagnosis
##
##      Control T1D     LADA
## T1D  0.06823 -       -
## LADA 0.24899 0.00085 -
## T2D  1.00000 0.04497 1.00000
##
## P value adjustment method: bonferroni
```

```r
pairwise.wilcox.test(Metadata2$Shannon, Metadata2$Diagnosis,
                     p.adjust.method="bonferroni")
```

```
##
##  Pairwise comparisons using Wilcoxon rank sum test with continuity correction
##
```

```
## data:  Metadata2$Shannon and Metadata2$Diagnosis
##
##      Control T1D   LADA
## T1D  1.000   -     -
## LADA 0.096   0.205 -
## T2D  1.000   0.964 1.000
##
## P value adjustment method: bonferroni
```

Alpha diversity remove metformin

```
rm(list=setdiff(ls(), c("Metadata", "MetadataMed", "Feature", "TaxonomyDA", "Taxonomy"
                        , "Fig1List")))
##Removing treated with metformin
#Metadata$Metformin <- ifelse(is.na(Metadata$Metformin), "Unknown", Metadata$Metformin)
Metadata2<-filter(Metadata, !Metformin == 1)
##Applying subsetting to OTU tables
Taxonomy2<-dplyr::select(Taxonomy, one_of(as.character(Metadata2$MicrobiomeID)))
##Remove orgs that are not present after subsetting.
Taxonomy2 <- Taxonomy2[rowSums(Taxonomy2)>0,]


#Calculate alpha diversity stats
#Use vegan to calculate various diversity and richness indices for each sample
set.seed(1)
diversityCalc <- data.frame(Shannon=diversity(t(Taxonomy2), index="shannon"),
                            Simpson=diversity(t(Taxonomy2), index="simpson"),
                            invSimpson=diversity(t(Taxonomy2), index="invsimpson"),
                            fisher=fisher.alpha(t(Taxonomy2)),
                            richness=specnumber(t(Taxonomy2)),
                            rarefy_min_count=rarefy(t(Taxonomy2),
                                                    sample=min(rowSums(t(Taxonomy2)))),
                            chao1=estimateR(t(Taxonomy2))["S.chao1",],
                            chao1SE=estimateR(t(Taxonomy2))["se.chao1",],
                            #ShannonRar=diversity(rrarefy(data.frame(t(Taxonomy2)),
                            #                              min(rowSums(t(Taxonomy2)))),
                            #                      index="shannon"),
                            #SimpsonRar=diversity(rrarefy(data.frame(t(Taxonomy2)),
                            #                              min(rowSums(t(Taxonomy2)))),
                            #                      index="simpson"),
                            #invSimpsonRar=diversity(rrarefy(data.frame(t(Taxonomy2)),
                            #                                 min(rowSums(t(Taxonomy2)))),
                            #                         index="invsimpson"),
                            Pielou=diversity(t(Taxonomy2))/log(specnumber(t(Taxonomy2))))
#Merge with metadata.
diversityCalc<-add_rownames(diversityCalc, "MicrobiomeID")
Metadata2 <- merge(Metadata2, diversityCalc, by="MicrobiomeID")

#Create a list to hold the plot objects.
Fig1ListRemMet <- list()
#Create vector to loop
#AlphaDiv<-c("fisher", "Shannon", "Simpson", "invSimpson", "richness", "chao1",
```

```r
#              "ShannonRar", "SimpsonRar", "invSimpsonRar", "Pielou")
AlphaDiv<-c("fisher", "Shannon", "Simpson", "invSimpson", "richness", "chao1", "Pielou")
#Plot
for (i in AlphaDiv) {
#Create plot name
pltName <- paste('Alpha', i, sep = '')
#create boxplots
Fig1ListRemMet[[ pltName ]]<-
ggplot(Metadata2, aes_string(x="Diagnosis", y=i, group="Diagnosis")) +
    geom_violin(aes(fill=Diagnosis, trim=FALSE)) +
    stat_summary(fun.data="mean_sdl",
                 mult=1, #mean plus minus a constant (mult=1) times the st.dev
                 geom="pointrange",
                 width=0.2 ) +
    #stat_summary(fun.y = mean, geom = "point") +
    #facet_grid(. ~ Metformin, scales="fixed") + #Scales can also be "free"
    #ggtitle(paste("Genus", i, sep=" ")) +
    #xlab("Diagnosis") +
    ylab(paste(i)) +
    scale_fill_manual(values=c(Control = "#666666", T1D = "#1F78B4",
                               T2D = "#33A02C", LADA = "#E31A1C")) +
    theme_bw() +
    theme(legend.position="none", panel.grid.major = element_blank(),
          panel.grid.minor = element_blank(), axis.title=element_text(size=20),
          axis.title.x = element_blank(),
          axis.text.x = element_text(angle = 45, hjust = 1, size=16),
          axis.text.y = element_text(angle = 45, hjust = 1, size=12))
}


#Have the plots stored in lists
lay <- rbind(c(1,2,3))
pdf(paste("MicroLADA_Alpha_RemMet_Func", ".pdf", sep=""), width=12, height=6)
grid.arrange(Fig1ListRemMet$Alpharichness,
             Fig1ListRemMet$AlphaPielou,
             Fig1ListRemMet$AlphaShannon, layout_matrix = lay)
dev.off()
```

```
## pdf
##   2
```

```r
grid.arrange(Fig1ListRemMet$Alpharichness,
             Fig1ListRemMet$AlphaPielou,
             Fig1ListRemMet$AlphaShannon, layout_matrix = lay)
```

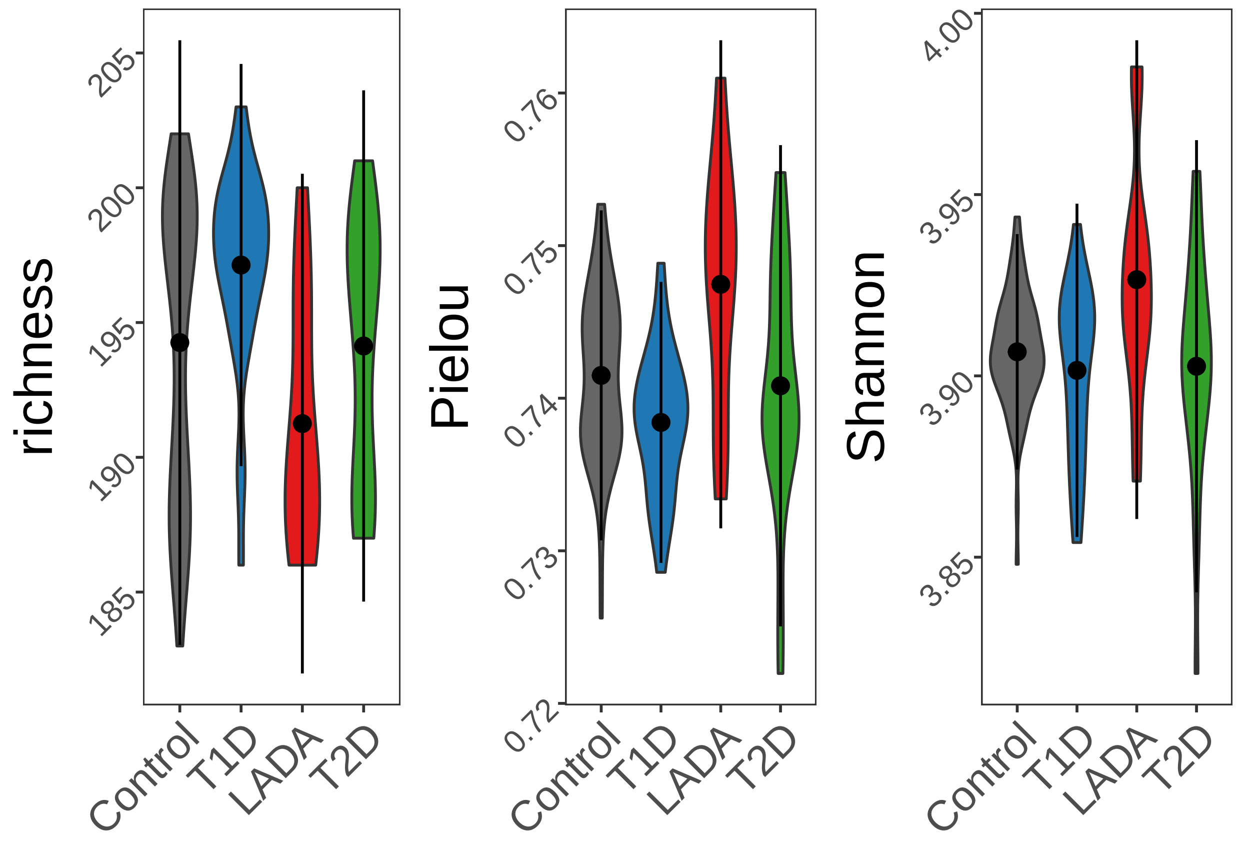


```
#Kruskal test
kruskal.test(richness ~ Diagnosis, data=Metadata2)
```

```
##
##  Kruskal-Wallis rank sum test
##
## data:  richness by Diagnosis
## Kruskal-Wallis chi-squared = 10.802, df = 3, p-value = 0.01285
```

```
kruskal.test(Pielou ~ Diagnosis, data=Metadata2)
```

```
##
##  Kruskal-Wallis rank sum test
##
## data:  Pielou by Diagnosis
## Kruskal-Wallis chi-squared = 13.649, df = 3, p-value = 0.003424
```

```
kruskal.test(Shannon ~ Diagnosis, data=Metadata2)
```

```
##
##  Kruskal-Wallis rank sum test
##
## data:  Shannon by Diagnosis
## Kruskal-Wallis chi-squared = 6.3561, df = 3, p-value = 0.09551
```

```
#If significant Mann-Whitney pairwise post-test
pairwise.wilcox.test(Metadata2$richness, Metadata2$Diagnosis,
                     p.adjust.method="bonferroni")
```

```
##
##  Pairwise comparisons using Wilcoxon rank sum test with continuity correction
##
## data:  Metadata2$richness and Metadata2$Diagnosis
##
##      Control T1D    LADA
## T1D  0.3663  -      -
## LADA 0.4764  0.0067 -
## T2D  1.0000  0.1381 0.5423
##
## P value adjustment method: bonferroni
```

```
pairwise.wilcox.test(Metadata2$Pielou, Metadata2$Diagnosis,
                     p.adjust.method="bonferroni")
```

```
##
##  Pairwise comparisons using Wilcoxon rank sum test with continuity correction
##
## data:  Metadata2$Pielou and Metadata2$Diagnosis
##
##      Control T1D    LADA
## T1D  0.0682  -      -
## LADA 0.0512  0.0061 -
## T2D  1.0000  1.0000 0.3171
##
## P value adjustment method: bonferroni
```

```
pairwise.wilcox.test(Metadata2$Shannon, Metadata2$Diagnosis,
                     p.adjust.method="bonferroni")
```

```
##
##  Pairwise comparisons using Wilcoxon rank sum test with continuity correction
##
## data:  Metadata2$Shannon and Metadata2$Diagnosis
##
##      Control T1D   LADA
## T1D  1.000   -     -
## LADA 0.096   0.177 -
## T2D  1.000   1.000 0.291
##
## P value adjustment method: bonferroni
```

Violin plots Dissimilarities

Part of figure 1

```r
rm(list=setdiff(ls(), c("Metadata", "MetadataMed", "Feature", "TaxonomyDA", "Taxonomy",
                        "Fig1List", "Fig1ListRemMet")))

##Create long format of dissimilarities
#Multi dimensional scaling
distmatrix <- vegdist(decostand(t(Taxonomy), method="hellinger"), method="bray")

#Contains all values except the ones equal to 0
meltPwBC<-subset(melt(as.matrix(distmatrix)))
rm(distmatrix) #Have comparisons of both 1vs2 and 2vs1 ..., but are removed during the
               #following filtration.
               #The same applies to comparisons of the same sample with itself


LADA_V <- subset(Metadata, Diagnosis=="LADA") %>% dplyr::select(one_of("MicrobiomeID")) %>%
  pull(MicrobiomeID) #%>% droplevels()
T1D_V <- subset(Metadata, Diagnosis=="T1D") %>% dplyr::select(one_of("MicrobiomeID")) %>%
  pull(MicrobiomeID) #%>% droplevels()
T2D_V <- subset(Metadata, Diagnosis=="T2D") %>% dplyr::select(one_of("MicrobiomeID")) %>%
  pull(MicrobiomeID) #%>% droplevels()
Control_V <- subset(Metadata, Diagnosis=="Control") %>% dplyr::select(one_of("MicrobiomeID")) %>%
  pull(MicrobiomeID) #%>% droplevels()

meltPwBC$Compare <- ifelse (meltPwBC$Var1  %in%  LADA_V & meltPwBC$Var2  %in%  T1D_V,
                            "LADA vs T1D",
                    ifelse (meltPwBC$Var1  %in%  LADA_V & meltPwBC$Var2  %in%  T2D_V,
                            "LADA vs T2D",
                    ifelse (meltPwBC$Var1  %in%  LADA_V & meltPwBC$Var2  %in%  Control_V,
                            "LADA vs Control",
                    ifelse (meltPwBC$Var1  %in%  T1D_V & meltPwBC$Var2  %in%  T2D_V,
                            "T1D vs T2D",
                    ifelse (meltPwBC$Var1  %in%  T1D_V & meltPwBC$Var2  %in%  Control_V,
                            "T1D vs Control",
                    ifelse (meltPwBC$Var1  %in%  T2D_V & meltPwBC$Var2  %in%  Control_V,
                            "T2D vs Control", "hmmmmmm"))))))
##Control validation
#table((meltPwBC$Var1  %in%  LADA_V) & (meltPwBC$Var2  %in%  T1D_V))
#table(meltPwBC$Compare)

meltPwBC<-subset(meltPwBC, meltPwBC$Compare!="hmmmmmm")

#Defining the order, can change if needed
meltPwBC$Compare<-factor(meltPwBC$Compare,
                         levels= c("LADA vs T1D", "LADA vs T2D", "LADA vs Control",
                                   "T1D vs T2D", "T1D vs Control", "T2D vs Control"))

violinDissi<-ggplot(meltPwBC, aes(x=Compare, y=value)) +
  geom_violin(trim=FALSE, fill="purple", colour="purple") +
  stat_summary(fun.data="mean_sdl", mult=1, #mean ± a constant (mult=1) times the st.dev
               geom="pointrange", width=0.2 ) +
  #geom_dotplot(binaxis='y', stackdir='center', dotsize=0.01) +
  labs(x="", y="Dissimilarity") +
  theme_bw() +
```

```r
  theme(legend.position="none", panel.grid.major = element_blank(),
        panel.grid.minor = element_blank())

violinDissi
```

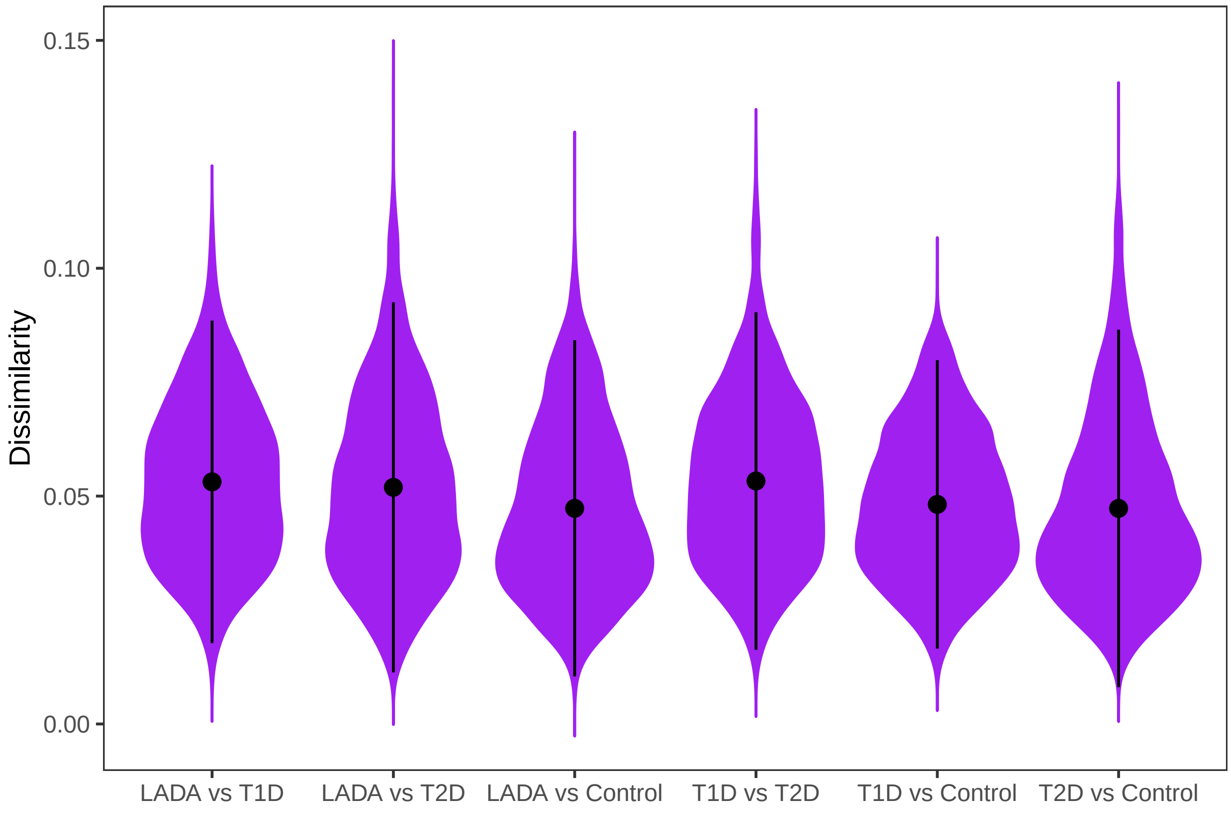


```r
pdf(paste("MicroLADA_ViolinDissi_Func", ".pdf", sep=""), width=12, height=6)
violinDissi
dev.off()
```

```
## pdf
##   2
```

```r
#Kruskal test
kruskal.test(value ~ Compare, data=meltPwBC)
```

```
##
##  Kruskal-Wallis rank sum test
##
## data:  value by Compare
## Kruskal-Wallis chi-squared = 382.99, df = 5, p-value < 2.2e-16
```

```r
#If significant Mann-Whitney pairwise post-test
pairwise.wilcox.test(meltPwBC$value, meltPwBC$Compare, p.adjust.method="bonferroni")
```

```
##
##  Pairwise comparisons using Wilcoxon rank sum test with continuity correction
##
## data:  meltPwBC$value and meltPwBC$Compare
##
##                 LADA vs T1D LADA vs T2D LADA vs Control T1D vs T2D
## LADA vs T2D     0.015       -           -               -
## LADA vs Control < 2e-16     < 2e-16     -               -
## T1D vs T2D      1.000       0.011       < 2e-16         -
## T1D vs Control  1.2e-15     1.7e-07     0.012           2.7e-16
## T2D vs Control  < 2e-16     < 2e-16     1.000           < 2e-16
##                 T1D vs Control
## LADA vs T2D     -
## LADA vs Control -
## T1D vs T2D      -
## T1D vs Control  -
## T2D vs Control  3.0e-05
##
## P value adjustment method: bonferroni
```

```
######################################################################
##Create long format of dissimilarities
#Multi dimensional scaling
distmatrix <- vegdist(decostand(t(Taxonomy), method="hellinger"), method="bray")



#Contains all values except the ones equal to 0
meltPwBC<-subset(melt(as.matrix(distmatrix)), value!=0)
#meltPwBC<-subset(melt(as.matrix(distmatrix)))
rm(distmatrix) #Have comparisons of both 1vs2 and 2vs1 ..., but are removed during the
               #following filtration.
               #The same applies to comparisons of the same sample with itself


LADA_V <- subset(Metadata, Diagnosis=="LADA") %>% dplyr::select(one_of("MicrobiomeID")) %>%
  pull(MicrobiomeID) #%>% droplevels()
T1D_V <- subset(Metadata, Diagnosis=="T1D") %>% dplyr::select(one_of("MicrobiomeID")) %>%
  pull(MicrobiomeID) #%>% droplevels()
T2D_V <- subset(Metadata, Diagnosis=="T2D") %>% dplyr::select(one_of("MicrobiomeID")) %>%
  pull(MicrobiomeID) #%>% droplevels()
Control_V <- subset(Metadata, Diagnosis=="Control") %>% dplyr::select(one_of("MicrobiomeID")) %>%
  pull(MicrobiomeID) #%>% droplevels()

meltPwBC$Compare <- ifelse (meltPwBC$Var1  %in%  LADA_V & meltPwBC$Var2  %in%
                              LADA_V, "LADA",
                    ifelse (meltPwBC$Var1  %in%  T1D_V & meltPwBC$Var2  %in%
                              T1D_V, "T1D",
                    ifelse (meltPwBC$Var1  %in%  T2D_V & meltPwBC$Var2  %in%
                              T2D_V, "T2D",
                    ifelse (meltPwBC$Var1  %in%  Control_V & meltPwBC$Var2  %in%
                              Control_V, "Control",
                    ifelse (meltPwBC$Var1  %in%  LADA_V & meltPwBC$Var2  %in%
                              T1D_V, "LADA vs T1D",
```

```
                    ifelse (meltPwBC$Var1  %in%  LADA_V & meltPwBC$Var2  %in%
                              T2D_V, "LADA vs T2D",
                    ifelse (meltPwBC$Var1  %in%  LADA_V & meltPwBC$Var2  %in%
                              Control_V, "LADA vs Control",
                    ifelse (meltPwBC$Var1  %in%  T1D_V & meltPwBC$Var2  %in%
                              T2D_V, "T1D vs T2D",
                    ifelse (meltPwBC$Var1  %in%  T1D_V & meltPwBC$Var2  %in%
                              Control_V, "T1D vs Control",
                    ifelse (meltPwBC$Var1  %in%  T2D_V & meltPwBC$Var2  %in%
                              Control_V, "T2D vs Control", "hmmmmmm"))))))))))
##Control validation
#table((meltPwBC$Var1  %in%  LADA_V) & (meltPwBC$Var2  %in%  T1D_V))
#table(meltPwBC$Compare)

meltPwBC<-subset(meltPwBC, meltPwBC$Compare!="hmmmmmm")

#Defining the order, can change if needed
meltPwBC$Compare<-factor(meltPwBC$Compare, levels= c("Control", "T1D",
                                                     "T2D", "LADA",
                                                     "LADA vs T1D", "LADA vs T2D",
                                                     "LADA vs Control", "T1D vs T2D",
                                                     "T1D vs Control", "T2D vs Control"))

violinDissi<-ggplot(meltPwBC, aes(x=Compare, y=value)) +
  geom_violin(trim=FALSE, fill="purple", colour="purple") +
  stat_summary(fun.data="mean_sdl", mult=1, #mean ± a constant (mult=1) times the st.dev
               geom="pointrange", width=0.2 ) +
  #geom_dotplot(binaxis='y', stackdir='center', dotsize=0.01) +
  labs(x="", y="Dissimilarity") +
  theme_bw() +
  theme(legend.position="none", panel.grid.major = element_blank(),
        panel.grid.minor = element_blank(),
        axis.text.x = element_text(angle = 45, hjust = 1))

violinDissi
```

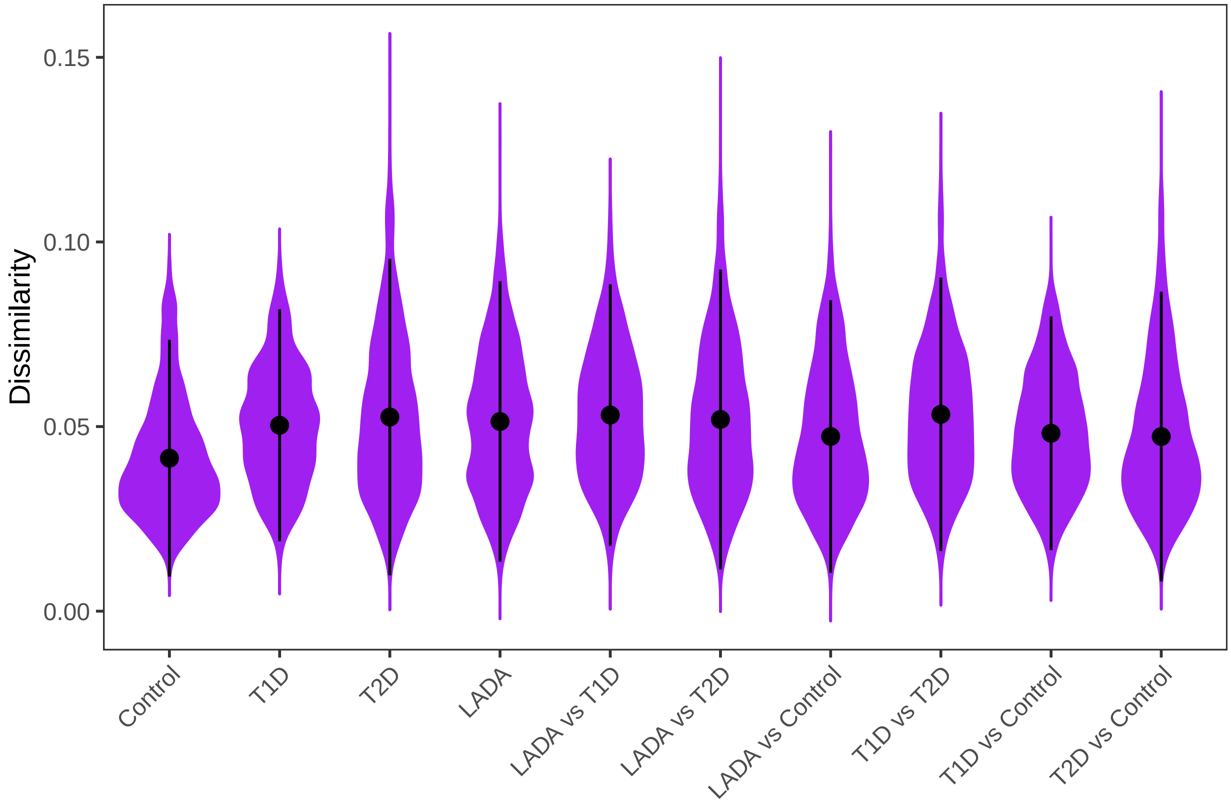


```
pdf(paste("MicroLADA_ViolinDissiAllComp_Func", ".pdf", sep=""), width=12, height=6)
violinDissi
dev.off()
```

```
## pdf
##   2
```

```
#Kruskal test
kruskal.test(value ~ Compare, data=meltPwBC)
```

```
##
##  Kruskal-Wallis rank sum test
##
## data:  value by Compare
## Kruskal-Wallis chi-squared = 1412.8, df = 9, p-value < 2.2e-16
```

```
#If significant Mann-Whitney pairwise post-test
pairwise.wilcox.test(meltPwBC$value, meltPwBC$Compare, p.adjust.method="bonferroni")
```

```
##
##  Pairwise comparisons using Wilcoxon rank sum test with continuity correction
##
## data:  meltPwBC$value and meltPwBC$Compare
##
```

```
##                 Control T1D     T2D     LADA    LADA vs T1D LADA vs T2D
## T1D             < 2e-16 -       -       -       -           -
## T2D             < 2e-16 1.000   -       -       -           -
## LADA            < 2e-16 1.000   1.000   -       -           -
## LADA vs T1D     < 2e-16 0.025   0.153   0.022   -           -
## LADA vs T2D     < 2e-16 1.000   1.000   1.000   0.046       -
## LADA vs Control < 2e-16 5.8e-07 < 2e-16 < 2e-16 < 2e-16     < 2e-16
## T1D vs T2D      < 2e-16 0.024   0.122   0.016   1.000       0.033
## T1D vs Control  < 2e-16 0.038   5.2e-08 1.7e-06 3.5e-15     5.1e-07
## T2D vs Control  < 2e-16 9.5e-10 < 2e-16 < 2e-16 < 2e-16     < 2e-16
##                 LADA vs Control T1D vs T2D T1D vs Control
## T1D             -               -          -
## T2D             -               -          -
## LADA            -               -          -
## LADA vs T1D     -               -          -
## LADA vs T2D     -               -          -
## LADA vs Control -               -          -
## T1D vs T2D      < 2e-16         -          -
## T1D vs Control  0.037           8.0e-16    -
## T2D vs Control  1.000           < 2e-16    9.1e-05
##
## P value adjustment method: bonferroni
```

```
######################################################################
meltPwBC<-filter(meltPwBC,
                 Compare == "Control" | Compare == "T1D" |
                   Compare == "T2D" | Compare == "LADA")

meltPwBC$Compare<-ordered(meltPwBC$Compare,
                            levels=c("Control", "T1D", "LADA", "T2D"))

violinDissi<-ggplot(meltPwBC, aes(x=Compare, y=value)) +
    geom_violin(aes(fill=Compare, trim=FALSE)) +
    stat_summary(fun.data="mean_sdl", mult=1, #mean ± a constant (mult=1) times the st.dev
                 geom="pointrange", width=0.2 ) +
    labs(y="Dissimilarity") +
    scale_fill_manual(values=c(Control = "#666666", T1D = "#1F78B4",
                              T2D = "#33A02C", LADA = "#E31A1C")) +
    theme_bw() +
    theme(legend.position="none", panel.grid.major = element_blank(),
          panel.grid.minor = element_blank(), axis.title=element_text(size=20),
          axis.title.x = element_blank(),
          axis.text.x = element_text(angle = 45, hjust = 1, size=16),
          axis.text.y = element_text(angle = 45, hjust = 1, size=12))


violinDissi
```

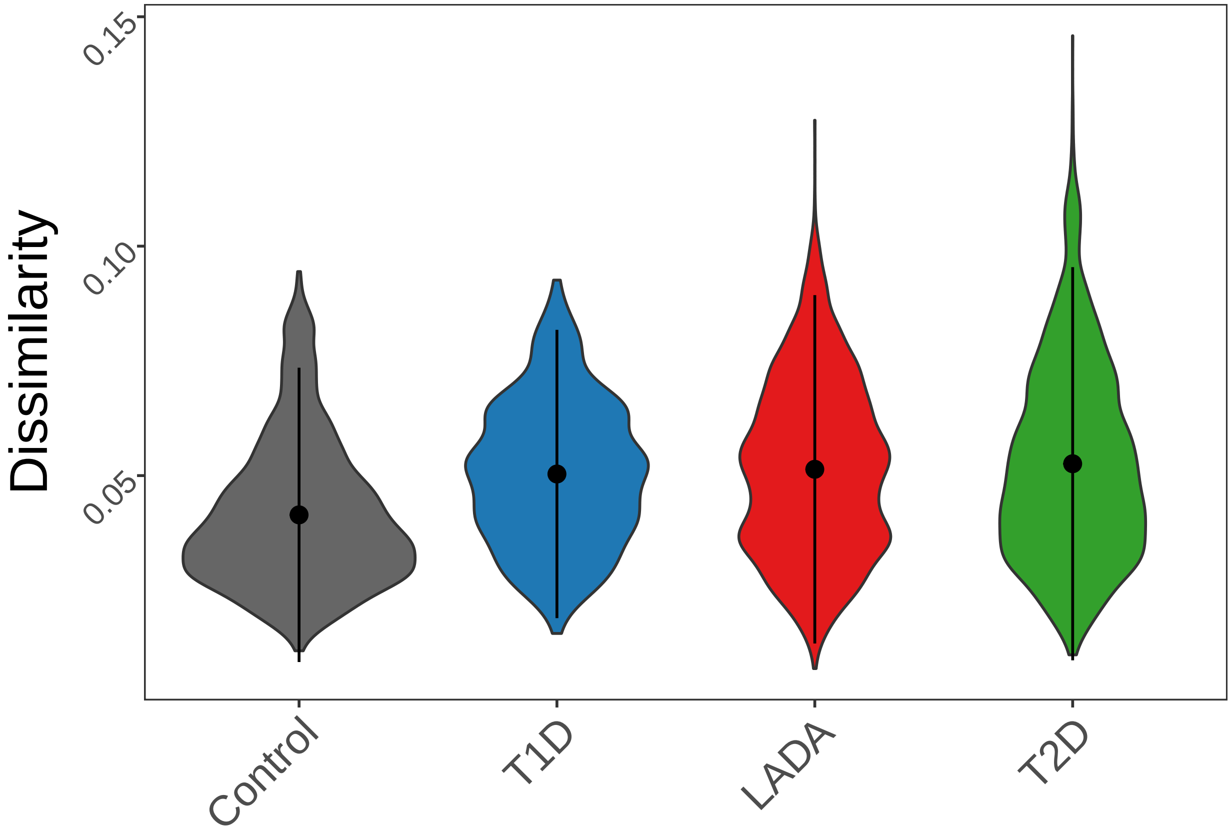


```
pdf(paste("MicroLADA_ViolinDissiCompWithin_Func", ".pdf", sep=""), width=12, height=6)
violinDissi
dev.off()
```

```
## pdf
##   2
```

```
#Save plot i previous list
Fig1List[[ "DissiBrayHel" ]] <- violinDissi

#Kruskal test
kruskal.test(value ~ Compare, data=meltPwBC)
```

```
##
##  Kruskal-Wallis rank sum test
##
## data:  value by Compare
## Kruskal-Wallis chi-squared = 956.99, df = 3, p-value < 2.2e-16
```

```
#If significant Mann-Whitney pairwise post-test
pairwise.wilcox.test(meltPwBC$value, meltPwBC$Compare, p.adjust.method="bonferroni")
```

```
##
##  Pairwise comparisons using Wilcoxon rank sum test with continuity correction
```

```
##
## data:  meltPwBC$value and meltPwBC$Compare
##
##      Control T1D LADA
## T1D  <2e-16  -   -
## LADA <2e-16  1   -
## T2D  <2e-16  1   1
##
## P value adjustment method: bonferroni
```

Violin plots Dissimilarities remove metformin

```
rm(list=setdiff(ls(), c("Metadata", "MetadataMed", "Feature", "TaxonomyDA", "Taxonomy",
                        "Fig1List", "Fig1ListRemMet")))
##Removing treated with metformin
#Metadata$Metformin <- ifelse(is.na(Metadata$Metformin), "Unknown", Metadata$Metformin)
Metadata2<-filter(Metadata, !Metformin == 1)
##Applying subsetting to OTU tables
Taxonomy2<-dplyr::select(Taxonomy, one_of(as.character(Metadata2$MicrobiomeID)))
##Remove orgs that are not present after subsetting.
Taxonomy2 <- Taxonomy2[rowSums(Taxonomy2)>0,]




##Create long format of dissimilarities
#Multi dimensional scaling
distmatrix <- vegdist(decostand(t(Taxonomy2), method="hellinger"), method="bray")

#Contains all values except the ones equal to 0
meltPwBC<-subset(melt(as.matrix(distmatrix)))
rm(distmatrix) #Have comparisons of both 1vs2 and 2vs1 ..., but are removed during the
               #following filtration.
               #The same applies to comparisons of the same sample with itself


LADA_V <- subset(Metadata2, Diagnosis=="LADA") %>% dplyr::select(one_of("MicrobiomeID")) %>%
  pull(MicrobiomeID) #%>% droplevels()
T1D_V <- subset(Metadata2, Diagnosis=="T1D") %>% dplyr::select(one_of("MicrobiomeID")) %>%
  pull(MicrobiomeID) #%>% droplevels()
T2D_V <- subset(Metadata2, Diagnosis=="T2D") %>% dplyr::select(one_of("MicrobiomeID")) %>%
  pull(MicrobiomeID) #%>% droplevels()
Control_V <- subset(Metadata2, Diagnosis=="Control") %>% dplyr::select(one_of("MicrobiomeID")) %>%
  pull(MicrobiomeID) #%>% droplevels()

meltPwBC$Compare <- ifelse (meltPwBC$Var1  %in%  LADA_V & meltPwBC$Var2  %in%  T1D_V,
                            "LADA vs T1D",
                    ifelse (meltPwBC$Var1  %in%  LADA_V & meltPwBC$Var2  %in%  T2D_V,
                            "LADA vs T2D",
                    ifelse (meltPwBC$Var1  %in%  LADA_V & meltPwBC$Var2  %in%  Control_V,
                            "LADA vs Control",
                    ifelse (meltPwBC$Var1  %in%  T1D_V & meltPwBC$Var2  %in%  T2D_V,
                            "T1D vs T2D",
```

```
                    ifelse (meltPwBC$Var1  %in%  T1D_V & meltPwBC$Var2  %in%  Control_V,
                            "T1D vs Control",
                    ifelse (meltPwBC$Var1  %in%  T2D_V & meltPwBC$Var2  %in%  Control_V,
                            "T2D vs Control", "hmmmmmm"))))))
##Control validation
#table((meltPwBC$Var1  %in%  LADA_V) & (meltPwBC$Var2  %in%  T1D_V))
#table(meltPwBC$Compare)

meltPwBC<-subset(meltPwBC, meltPwBC$Compare!="hmmmmmm")

#Defining the order, can change if needed
meltPwBC$Compare<-factor(meltPwBC$Compare,
                         levels= c("LADA vs T1D", "LADA vs T2D", "LADA vs Control",
                                   "T1D vs T2D", "T1D vs Control", "T2D vs Control"))

violinDissi<-ggplot(meltPwBC, aes(x=Compare, y=value)) +
  geom_violin(trim=FALSE, fill="purple", colour="purple") +
  stat_summary(fun.data="mean_sdl", mult=1, #mean ± a constant (mult=1) times the st.dev
               geom="pointrange", width=0.2 ) +
  #geom_dotplot(binaxis='y', stackdir='center', dotsize=0.01) +
  labs(x="", y="Dissimilarity") +
  theme_bw() +
  theme(legend.position="none", panel.grid.major = element_blank(),
        panel.grid.minor = element_blank())

violinDissi
```

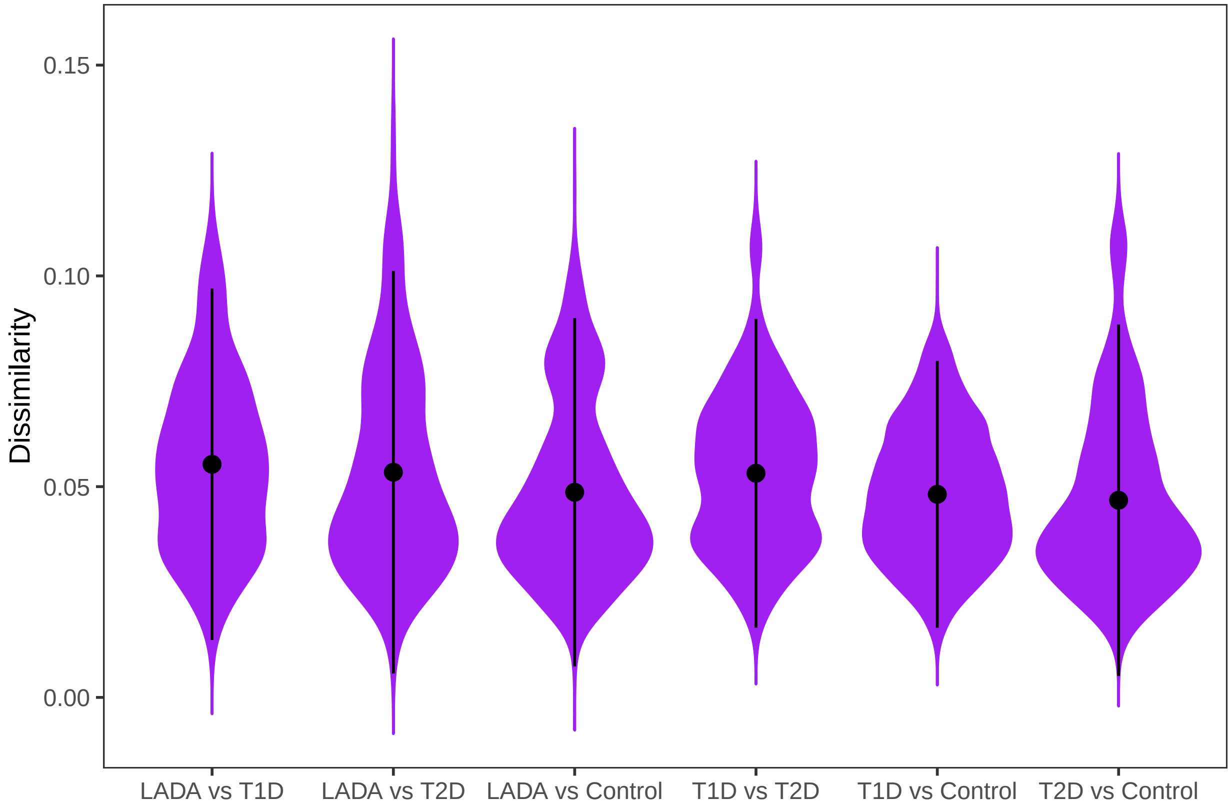


```
pdf(paste("MicroLADA_ViolinDissiRemMet_Func", ".pdf", sep=""), width=12, height=6)
violinDissi
dev.off()
```

```
## pdf
##   2
```

```
#Kruskal test
kruskal.test(value ~ Compare, data=meltPwBC)
```

```
##
##  Kruskal-Wallis rank sum test
##
## data:  value by Compare
## Kruskal-Wallis chi-squared = 127.43, df = 5, p-value < 2.2e-16
```

```
#If significant Mann-Whitney pairwise post-test
pairwise.wilcox.test(meltPwBC$value, meltPwBC$Compare, p.adjust.method="bonferroni")
```

```
##
##  Pairwise comparisons using Wilcoxon rank sum test with continuity correction
##
## data:  meltPwBC$value and meltPwBC$Compare
##
```

```
##                 LADA vs T1D LADA vs T2D LADA vs Control T1D vs T2D
## LADA vs T2D     1.00000     -           -               -
## LADA vs Control 1.0e-06     0.18745     -               -
## T1D vs T2D      1.00000     1.00000     4.3e-07         -
## T1D vs Control  2.9e-07     0.74973     0.93469         7.2e-08
## T2D vs Control  3.7e-13     0.00025     0.15655         < 2e-16
##                 T1D vs Control
## LADA vs T2D     -
## LADA vs Control -
## T1D vs T2D      -
## T1D vs Control  -
## T2D vs Control  1.3e-07
##
## P value adjustment method: bonferroni
```

```r
#####################################################################
##Create long format of dissimilarities
#Multi dimensional scaling
distmatrix <- vegdist(decostand(t(Taxonomy2), method="hellinger"), method="bray")



#Contains all values except the ones equal to 0
meltPwBC<-subset(melt(as.matrix(distmatrix)), value!=0)
#meltPwBC<-subset(melt(as.matrix(distmatrix)))
rm(distmatrix) #Have comparisons of both 1vs2 and 2vs1 ..., but are removed during the
               #following filtration.
               #The same applies to comparisons of the same sample with itself


LADA_V <- subset(Metadata2, Diagnosis=="LADA") %>% dplyr::select(one_of("MicrobiomeID")) %>%
  pull(MicrobiomeID) #%>% droplevels()
T1D_V <- subset(Metadata2, Diagnosis=="T1D") %>% dplyr::select(one_of("MicrobiomeID")) %>%
  pull(MicrobiomeID) #%>% droplevels()
T2D_V <- subset(Metadata2, Diagnosis=="T2D") %>% dplyr::select(one_of("MicrobiomeID")) %>%
  pull(MicrobiomeID) #%>% droplevels()
Control_V <- subset(Metadata2, Diagnosis=="Control") %>% dplyr::select(one_of("MicrobiomeID")) %>%
  pull(MicrobiomeID) #%>% droplevels()

meltPwBC$Compare <- ifelse (meltPwBC$Var1  %in%  LADA_V & meltPwBC$Var2  %in%
                              LADA_V, "LADA",
                    ifelse (meltPwBC$Var1  %in%  T1D_V & meltPwBC$Var2  %in%
                              T1D_V, "T1D",
                    ifelse (meltPwBC$Var1  %in%  T2D_V & meltPwBC$Var2  %in%
                              T2D_V, "T2D",
                    ifelse (meltPwBC$Var1  %in%  Control_V & meltPwBC$Var2  %in%
                              Control_V, "Control",
                    ifelse (meltPwBC$Var1  %in%  LADA_V & meltPwBC$Var2  %in%
                              T1D_V, "LADA vs T1D",
                    ifelse (meltPwBC$Var1  %in%  LADA_V & meltPwBC$Var2  %in%
                              T2D_V, "LADA vs T2D",
                    ifelse (meltPwBC$Var1  %in%  LADA_V & meltPwBC$Var2  %in%
                              Control_V, "LADA vs Control",
                    ifelse (meltPwBC$Var1  %in%  T1D_V & meltPwBC$Var2  %in%
```

```r
                              T2D_V, "T1D vs T2D",
                    ifelse (meltPwBC$Var1  %in%  T1D_V & meltPwBC$Var2  %in%
                              Control_V, "T1D vs Control",
                    ifelse (meltPwBC$Var1  %in%  T2D_V & meltPwBC$Var2  %in%
                              Control_V, "T2D vs Control", "hmmmmmm")))))))))
##Control validation
#table((meltPwBC$Var1  %in%  LADA_V) & (meltPwBC$Var2  %in%  T1D_V))
#table(meltPwBC$Compare)

meltPwBC<-subset(meltPwBC, meltPwBC$Compare!="hmmmmmm")

#Defining the order, can change if needed
meltPwBC$Compare<-factor(meltPwBC$Compare, levels= c("Control", "T1D",
                                                     "T2D", "LADA",
                                                     "LADA vs T1D", "LADA vs T2D",
                                                     "LADA vs Control", "T1D vs T2D",
                                                     "T1D vs Control", "T2D vs Control"))

violinDissi<-ggplot(meltPwBC, aes(x=Compare, y=value)) +
  geom_violin(trim=FALSE, fill="purple", colour="purple") +
  stat_summary(fun.data="mean_sdl", mult=1, #mean ± a constant (mult=1) times the st.dev
               geom="pointrange", width=0.2 ) +
  #geom_dotplot(binaxis='y', stackdir='center', dotsize=0.01) +
  labs(x="", y="Dissimilarity") +
  theme_bw() +
  theme(legend.position="none", panel.grid.major = element_blank(),
        panel.grid.minor = element_blank(),
        axis.text.x = element_text(angle = 45, hjust = 1))

violinDissi
```

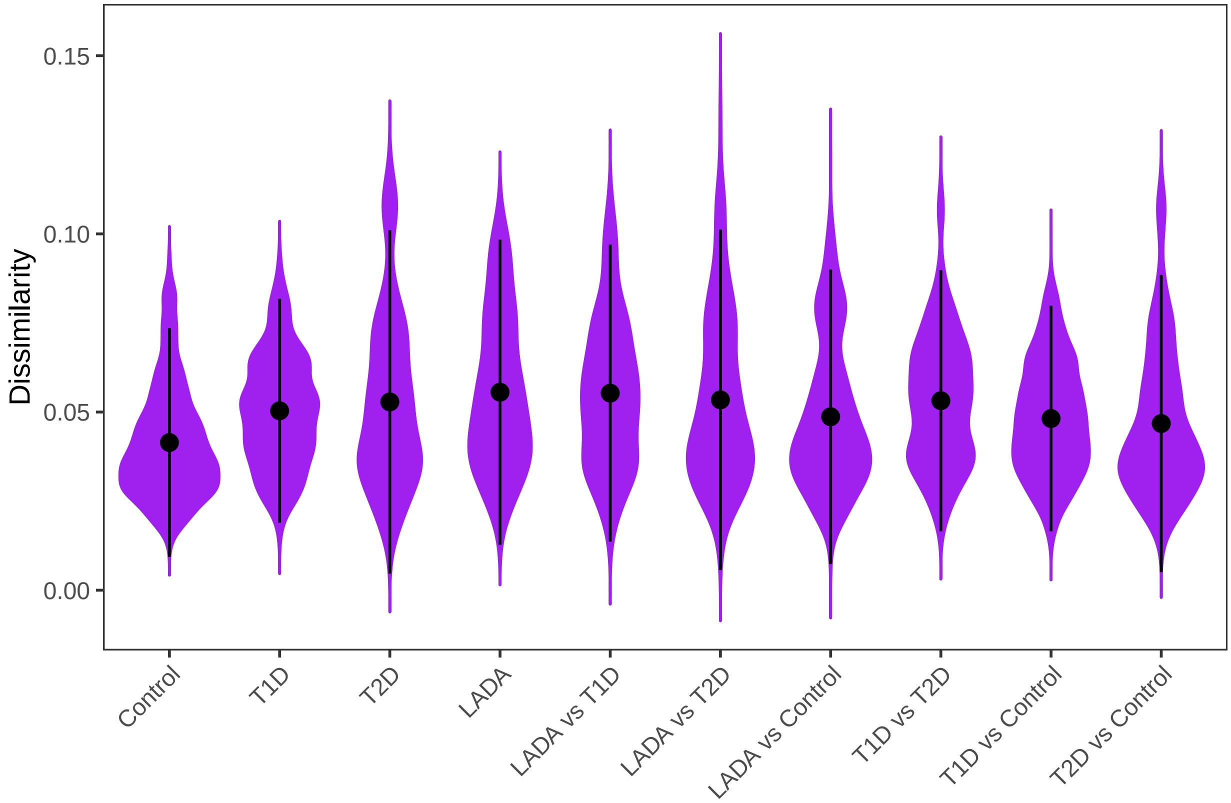


```r
pdf(paste("MicroLADA_ViolinDissiAllCompRemMet_Func", ".pdf", sep=""), width=12, height=6)
violinDissi
dev.off()
```

```
## pdf
##   2
```

```r
#Kruskal test
kruskal.test(value ~ Compare, data=meltPwBC)
```

```
##
##  Kruskal-Wallis rank sum test
##
## data:  value by Compare
## Kruskal-Wallis chi-squared = 735.77, df = 9, p-value < 2.2e-16
```

```r
#If significant Mann-Whitney pairwise post-test
pairwise.wilcox.test(meltPwBC$value, meltPwBC$Compare, p.adjust.method="bonferroni")
```

```
##
##  Pairwise comparisons using Wilcoxon rank sum test with continuity correction
##
## data:  meltPwBC$value and meltPwBC$Compare
##
```

```
##                  Control T1D     T2D     LADA    LADA vs T1D LADA vs T2D
## T1D              < 2e-16 -       -       -       -           -
## T2D              < 2e-16 1.00000 -       -       -           -
## LADA             5.3e-13 1.00000 1.00000 -       -           -
## LADA vs T1D      < 2e-16 0.04614 0.72305 1.00000 -           -
## LADA vs T2D      1.8e-14 1.00000 1.00000 1.00000 1.00000     -
## LADA vs Control  < 2e-16 0.00180 0.36234 0.01401 3.1e-06     0.56234
## T1D vs T2D       < 2e-16 0.32609 1.00000 1.00000 1.00000     1.00000
## T1D vs Control   < 2e-16 0.03779 1.00000 0.03832 8.6e-07     1.00000
## T2D vs Control   3.3e-13 9.3e-12 3.0e-05 4.9e-05 1.1e-12     0.00076
##                  LADA vs Control T1D vs T2D T1D vs Control
## T1D              -               -          -
## T2D              -               -          -
## LADA             -               -          -
## LADA vs T1D      -               -          -
## LADA vs T2D      -               -          -
## LADA vs Control  -               -          -
## T1D vs T2D       1.3e-06         -          -
## T1D vs Control   1.00000         2.1e-07    -
## T2D vs Control   0.46964         < 2e-16    3.8e-07
##
## P value adjustment method: bonferroni
```

```
#######################################################################
meltPwBC<-filter(meltPwBC,
                 Compare == "Control" | Compare == "T1D" |
                   Compare == "T2D" | Compare == "LADA")

meltPwBC$Compare<-ordered(meltPwBC$Compare,
                            levels=c("Control", "T1D", "LADA", "T2D"))

violinDissi<-ggplot(meltPwBC, aes(x=Compare, y=value)) +
    geom_violin(aes(fill=Compare, trim=FALSE)) +
    stat_summary(fun.data="mean_sdl", mult=1, #mean ± a constant (mult=1) times the st.dev
                 geom="pointrange", width=0.2 ) +
    labs(y="Dissimilarity") +
    scale_fill_manual(values=c(Control = "#666666", T1D = "#1F78B4",
                              T2D = "#33A02C", LADA = "#E31A1C")) +
    theme_bw() +
    theme(legend.position="none", panel.grid.major = element_blank(),
          panel.grid.minor = element_blank(), axis.title=element_text(size=20),
          axis.title.x = element_blank(),
          axis.text.x = element_text(angle = 45, hjust = 1, size=16),
          axis.text.y = element_text(angle = 45, hjust = 1, size=12))


violinDissi
```

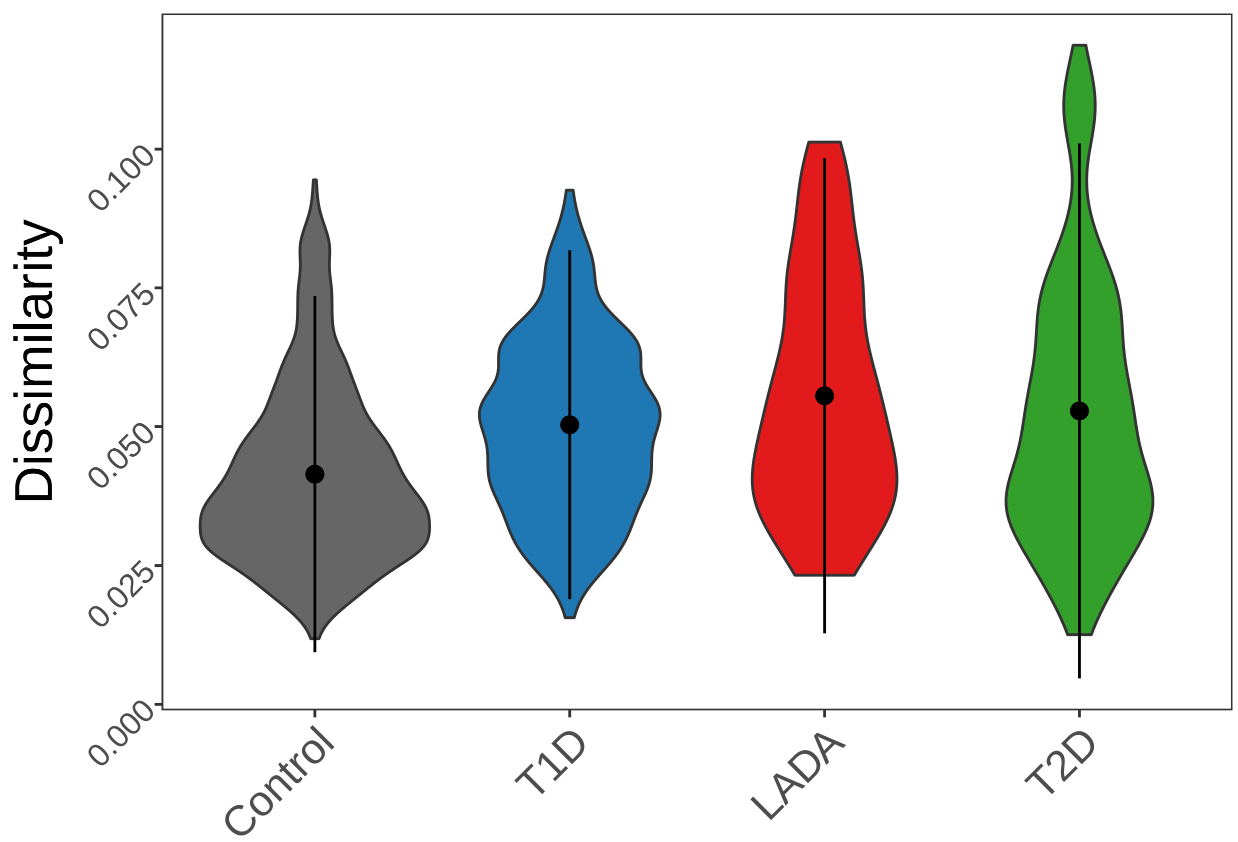


```
pdf(paste("MicroLADA_ViolinDissiCompWithinRemMet_Func", ".pdf", sep=""), width=12, height=6)
violinDissi
dev.off()
```

```
## pdf
##   2
```

```
#Save plot in previous list
Fig1ListRemMet[[ "DissiBrayHel" ]] <- violinDissi

#Kruskal test
kruskal.test(value ~ Compare, data=meltPwBC)
```

```
##
##  Kruskal-Wallis rank sum test
##
## data:  value by Compare
## Kruskal-Wallis chi-squared = 360.41, df = 3, p-value < 2.2e-16
```

```
#If significant Mann-Whitney pairwise post-test
pairwise.wilcox.test(meltPwBC$value, meltPwBC$Compare, p.adjust.method="bonferroni")
```

```
##
##  Pairwise comparisons using Wilcoxon rank sum test with continuity correction
```

```
##
## data:  meltPwBC$value and meltPwBC$Compare
##
##      Control T1D  LADA
## T1D  <2e-16  -    -
## LADA 7e-14   0.32 -
## T2D  <2e-16  1.00 0.61
##
## P value adjustment method: bonferroni
```

PCoA

Part of figure 1

```
rm(list=setdiff(ls(), c("Metadata", "MetadataMed", "Feature", "TaxonomyDA", "Taxonomy",
                        "Fig1List", "Fig1ListRemMet")))

Subset <- c("LADAControl", "LADAT1D", "LADAT2D", "All")
#i <- c("All")
#i <- c("LADAControl")
for (i in Subset) {

#Subsetting Metadata according to
if (i=="All") {
  print(i)
  Metadata2 <- Metadata
} else if (i=="LADAControl") {
  print(i)
  Metadata2<-filter(Metadata, Diagnosis == "LADA" | Diagnosis == "Control")
} else if (i=="LADAT1D") {
  print(i)
  Metadata2<-filter(Metadata, Diagnosis == "LADA" | Diagnosis == "T1D")
} else if (i=="LADAT2D") {
  print(i)
  Metadata2<-filter(Metadata, Diagnosis == "LADA" | Diagnosis == "T2D")
} else {
  print("Subset defined not valid")
}

#Apply subsetting to Taxonomy table
Taxonomy2<-dplyr::select(Taxonomy, one_of(as.character(Metadata2$MicrobiomeID)))


#Hellinger transformation
Taxonomy2 <- data.frame(t(decostand(t(Taxonomy2), method="hellinger")))
#Maks TSS
Taxonomy2<-sweep(Taxonomy2, 2, colSums(Taxonomy2), FUN="/")
#Dissimilarity
distmatrix <- vegdist(t(Taxonomy2), method="bray")
#Multi dimensional scaling with capscale
PCoAcsObject<-capscale(distmatrix~1)
```

```
##Add eig to plot axes. with cmdscale there are negative values not with capscale
eig <- PCoAcsObject$CA$eig
# Calculate the variation explained by PCoA1, 2, 3 and 4
# and use it to generate axis labels
eig_1_2 <- eig[1:4] / sum(eig) * 100 #Vector with variance explained
#  by the first 4 axes
eig_1 <- paste("PCoA1", round(eig_1_2[1], digits = 2), "% variance")
eig_2 <- paste("PCoA2", round(eig_1_2[2], digits = 2), "% variance")
eig_3 <- paste("PCoA3", round(eig_1_2[3], digits = 2), "% variance")
eig_4 <- paste("PCoA4", round(eig_1_2[4], digits = 2), "% variance")

##Pull out coordinates for plotting from the ca object
#Structuring to add to Metadata2
PCoACA<-PCoAcsObject$CA #The ca object contains the actual ordination results:
#u ((Weighted) orthonormal site scores),
#v ((Weighted) orthonormal species scores) all na in mine (unconstrained),
#Xbar (The standardized data matrix after previous stages of analysis),
#and imaginary.u.eig ???.
#Info http://cc.oulu.fi/~jarioksa/softhelp/vegan/html/cca.object.html
PCoA<-as.data.frame(PCoACA$u)
#Change colnames. Now add dis and trans info to names
colnames(PCoA) <- c("MDS1BrayHel","MDS2BrayHel", "MDS3BrayHel","MDS4BrayHel")
#Add row names to df
PCoA$MicrobiomeID <- row.names(PCoA)
#Remove leading X
PCoA$MicrobiomeID <- gsub("X", "", PCoA$MicrobiomeID)
#Merge according to Sample
Metadata2<-merge(Metadata2, PCoA, by="MicrobiomeID")


#PCoA MDS1 and MDS2 pdf
pdf(paste("MicroLADA_PCoA", i, "_Func.pdf", sep=""), width=9, height=6)
print(ggplot(Metadata2) +
  geom_point(aes(x=MDS1BrayHel, y=MDS2BrayHel, color = Diagnosis,
                 group = Diagnosis), size=3) +
  stat_ellipse(aes(MDS1BrayHel, y=MDS2BrayHel, color = Diagnosis,
                   group = Diagnosis)) +
  scale_color_manual(values=c(Control = "#666666", T1D = "#1F78B4",
                              T2D = "#33A02C", LADA = "#E31A1C")) +
  ggtitle(paste("PCoA", i, sep=" ")) +
  labs(colour="Diagnosis", x = eig_1, y = eig_2) +
  theme_bw() +
  theme(panel.grid.major = element_blank(), panel.grid.minor = element_blank(),
        axis.title=element_text(size=12), legend.position="bottom"))
dev.off()

#PCoA MDS1 and MDS2 plotly
print(ggplotly(ggplot(Metadata2[, c(6,8,13,14)]) +
  geom_point(aes(x=MDS1BrayHel, y=MDS2BrayHel, color = Diagnosis,
                 group = Dg_LADA), size=3) +
  stat_ellipse(aes(MDS1BrayHel, y=MDS2BrayHel, color = Diagnosis,
                   group = Diagnosis)) +
  scale_color_manual(values=c(Control = "#666666", T1D = "#1F78B4",
```

```r
                            T2D = "#33A02C", LADA = "#E31A1C")) +
  ggtitle(paste("PCoA", i, sep=" ")) +
  labs(colour="Diagnosis", x = eig_1, y = eig_2) +
  theme_bw() +
  theme(panel.grid.major = element_blank(), panel.grid.minor = element_blank(),
        axis.title=element_text(size=12), legend.position="bottom")))

#PCoA MDS1 and MDS2
print(ggplot(Metadata2) +
  geom_point(aes(x=MDS1BrayHel, y=MDS2BrayHel, color = Diagnosis,
                 group = Diagnosis), size=3) +
  stat_ellipse(aes(MDS1BrayHel, y=MDS2BrayHel, color = Diagnosis,
                   group = Diagnosis)) +
  scale_color_manual(values=c(Control = "#666666", T1D = "#1F78B4",
                            T2D = "#33A02C", LADA = "#E31A1C")) +
  ggtitle(paste("PCoA", i, sep=" ")) +
  labs(colour="Diagnosis", x = eig_1, y = eig_2) +
  theme_bw() +
  theme(panel.grid.major = element_blank(), panel.grid.minor = element_blank(),
        axis.title=element_text(size=12), legend.position="bottom"))

#PCoA MDS1 and MDS3
print(ggplot(Metadata2) +
  geom_point(aes(x=MDS1BrayHel, y=MDS3BrayHel, color = Diagnosis,
                 group = Diagnosis), size=3) +
  stat_ellipse(aes(MDS1BrayHel, y=MDS3BrayHel, color = Diagnosis,
                   group = Diagnosis)) +
  scale_color_manual(values=c(Control = "#666666", T1D = "#1F78B4",
                            T2D = "#33A02C", LADA = "#E31A1C")) +
  ggtitle(paste("PCoA", i, sep=" ")) +
  labs(colour="Diagnosis", x = eig_1, y = eig_3) +
  theme_bw() +
  theme(panel.grid.major = element_blank(), panel.grid.minor = element_blank(),
        axis.title=element_text(size=12), legend.position="bottom"))

#PCoA MDS2 and MDS3
print(ggplot(Metadata2) +
  geom_point(aes(x=MDS2BrayHel, y=MDS3BrayHel, color = Diagnosis,
                 group = Diagnosis), size=3) +
  stat_ellipse(aes(MDS2BrayHel, y=MDS3BrayHel, color = Diagnosis,
                   group = Diagnosis)) +
  scale_color_manual(values=c(Control = "#666666", T1D = "#1F78B4",
                            T2D = "#33A02C", LADA = "#E31A1C")) +
  ggtitle(paste("PCoA", i, sep=" ")) +
  labs(colour="Diagnosis", x = eig_2, y = eig_3) +
  theme_bw() +
  theme(panel.grid.major = element_blank(), panel.grid.minor = element_blank(),
        axis.title=element_text(size=12), legend.position="bottom"))

#Add a categorical indicator of group in Metadata
Metadata2$Metformine<-as.factor(ifelse(grepl("1", Metadata2$Metformin), "Yes",
                                     ifelse(grepl("0", Metadata2$Metformin),
                                            "No", "Unknown")))
```

```
#Order Treatment metformine for plotting
Metadata2$Metformine <- factor(Metadata2$Metformine, levels=c("Yes", "No", "Unknown"))
#Create PCoA for figure 1
if (i=="All") {
  PCoAall<-ggplot(Metadata2) +
  geom_point(aes(MDS1BrayHel, MDS2BrayHel, color = Diagnosis, #shape = Metformine,
                 group = Diagnosis), size=5) +
  stat_ellipse(aes(MDS1BrayHel, MDS2BrayHel, color = Diagnosis, group = Diagnosis)) +
  #stat_ellipse(aes(MDS1BrayHel, MDS2BrayHel, group = Metformine,
  #                 linetype=factor(Metadata2$Metformine)), alpha = 0.7) +
  scale_color_manual(values=c(Control = "#666666", T1D = "#1F78B4",
                              T2D = "#33A02C", LADA = "#E31A1C")) +
  scale_shape_manual(values=c(3, 16)) +
  scale_linetype_manual(values=c("longdash", "dotted"), guide=FALSE) +
  #ggtitle(paste("PCoA", "LADA & T2D Metformin", sep=" ")) +
  labs(colour="Diagnosis", x = eig_1, y = eig_2) + #, shape="Metformin treatment"
  theme_bw() +
  theme(panel.grid.major = element_blank(), panel.grid.minor = element_blank(),
        axis.title=element_text(size=22), legend.position="bottom",
        legend.title=element_text(size=20), legend.text=element_text(size=20),
        axis.text.x = element_text(angle = 45, hjust = 1, size=12),
        axis.text.y = element_text(angle = 45, hjust = 1, size=12))

  print(PCoAall)
  pdf("MicroLADA_PCoAAllMet_Func.pdf", width=9, height=6)
  print(PCoAall)
  dev.off()
  #Save plot in previous list
  Fig1List[[ "PCoAall" ]] <- PCoAall
  #return(PCoAall)
}

##Stressplot
##Extract ordination distances and merge with observed dissimilarity
#stress<-stressplot(PCoAcsObject)
#df <- melt(as.matrix(stress))
#names(df)<-c("rowOrd", "colOrd", "OrdDist")
#df<-filter(df, OrdDist>0)
#df2 <- melt(as.matrix(distmatrix))
#names(df2)<-c("rowObs", "colObs", "ObsDism")
#df2<-filter(df2, ObsDism>0)
#df<-unite(df, mergecol, c(rowOrd, colOrd), remove=FALSE)
#df2<-unite(df2, mergecol, c(rowObs, colObs), remove=FALSE)
#ggstress<-merge(df, df2, by="mergecol")

##create stressplot
#print(ggplot(ggstress) +
#  geom_point(aes(ObsDism, OrdDist)) +
#  ggtitle(paste("Stressplot", i, sep=" ")) +
#  labs(x = "Observed dissimilarity", y = "Ordination distance") +
#  theme_bw() +
#  theme(panel.grid.major = element_blank(), panel.grid.minor = element_blank(),
#        axis.title=element_text(size=12)))
```

```
##Screeplot
#screeplot<-data.frame(PCoAcsObject$CA$eig)
#colnames(screeplot)<-c("eig")
#screeplot$eig <- screeplot$eig[1:length(screeplot$eig)] /
#  sum(screeplot$eig) * 100
#screeplot<-add_rownames(screeplot, "MDS")
#screeplot$MDS <- factor(screeplot$MDS,
#                         levels=c(sprintf("MDS%d", 1:length(screeplot$eig))))

##create screeplot
#print(ggplot(screeplot, aes(x=MDS, y=eig)) +
#  geom_bar(stat="identity") +
#  labs(x ="MDS", y ="eig (%)") +
#  ggtitle(paste("Screeplot", i, sep=" ")) +
#  theme_bw() +
#  theme(panel.grid.major = element_blank(), panel.grid.minor = element_blank(),
#        axis.title=element_text(size=12), axis.text.x=element_blank(),
#        axis.ticks.x=element_blank()))

}
```

```
## [1] "LADAControl"
```

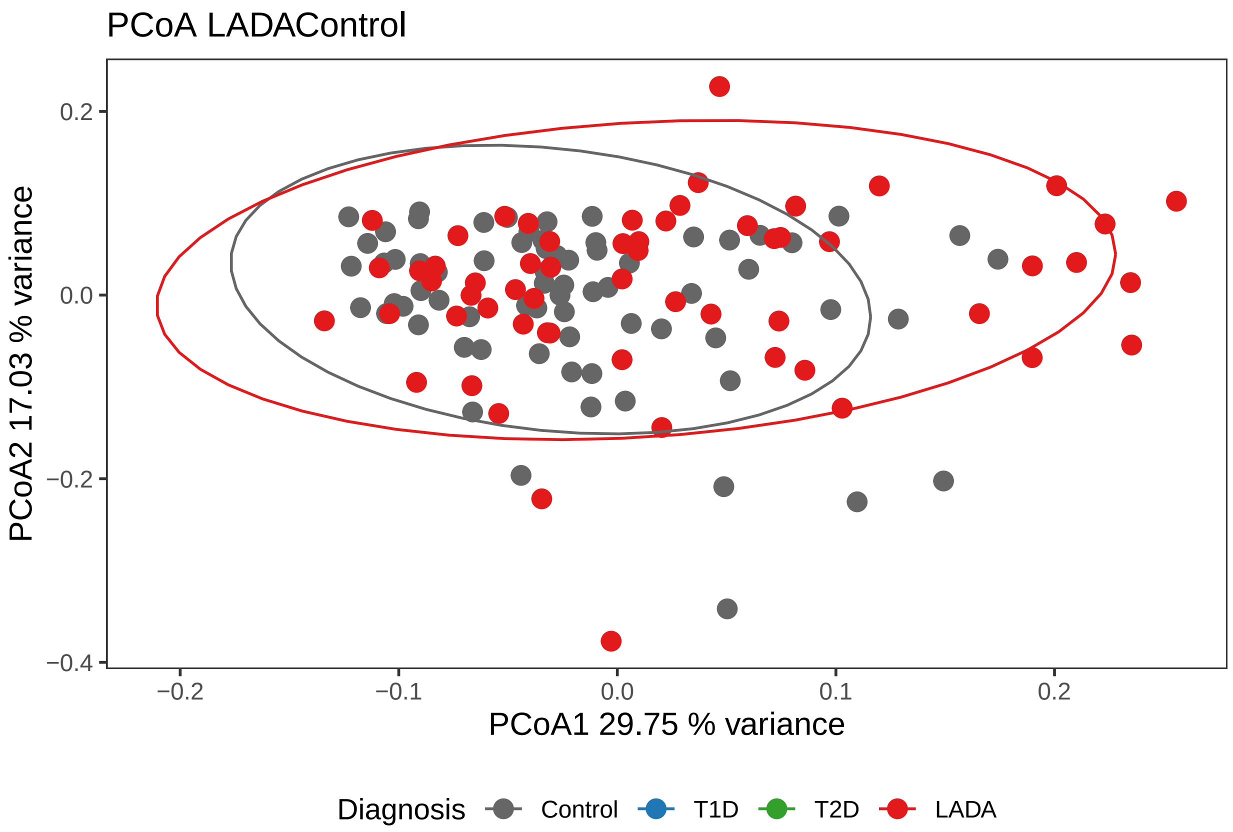

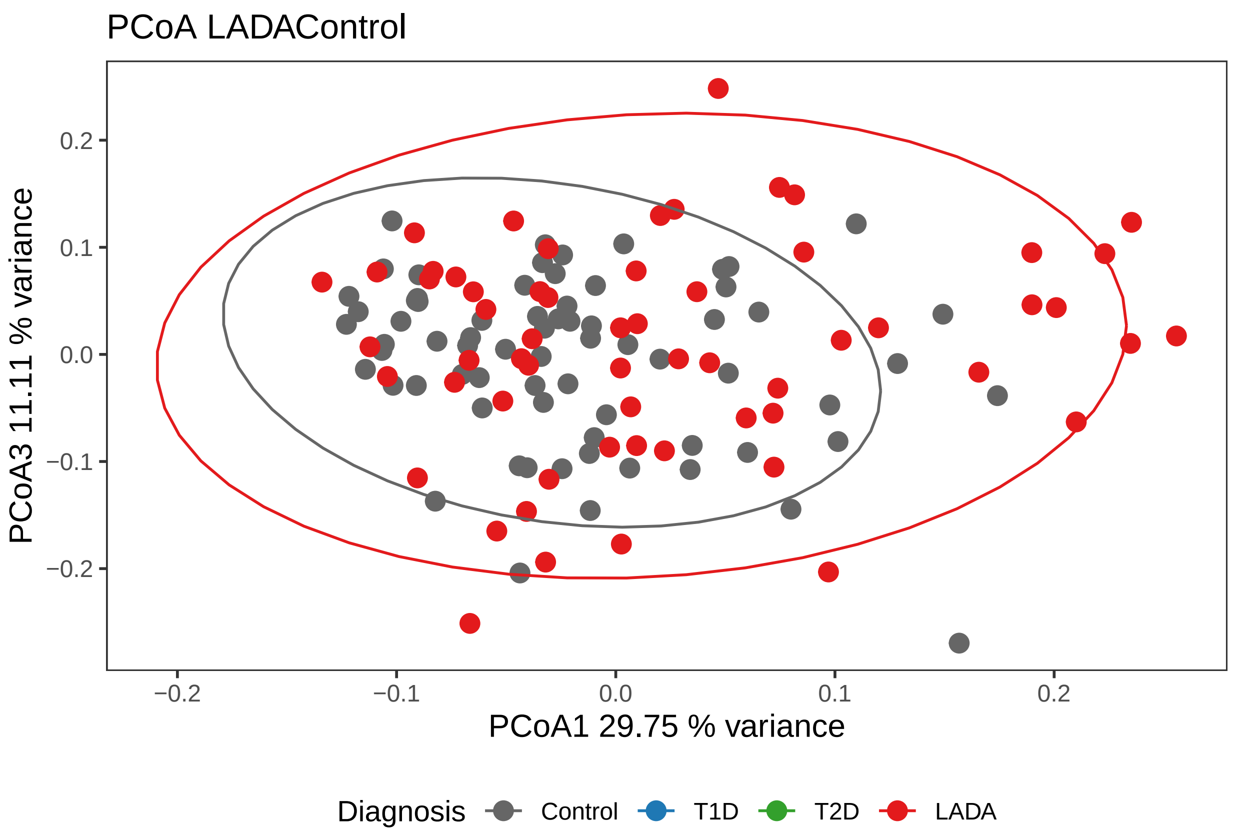
PCoA LADAControl
PCoA3 11.11 % variance
PCoA1 29.75 % variance
0.2
0.1
0.0
−0.1
−0.2
−0.2
−0.1
0.0
0.1
0.2
Diagnosis
Control
T1D
T2D
LADA

```
## [1] "LADAT1D"
```

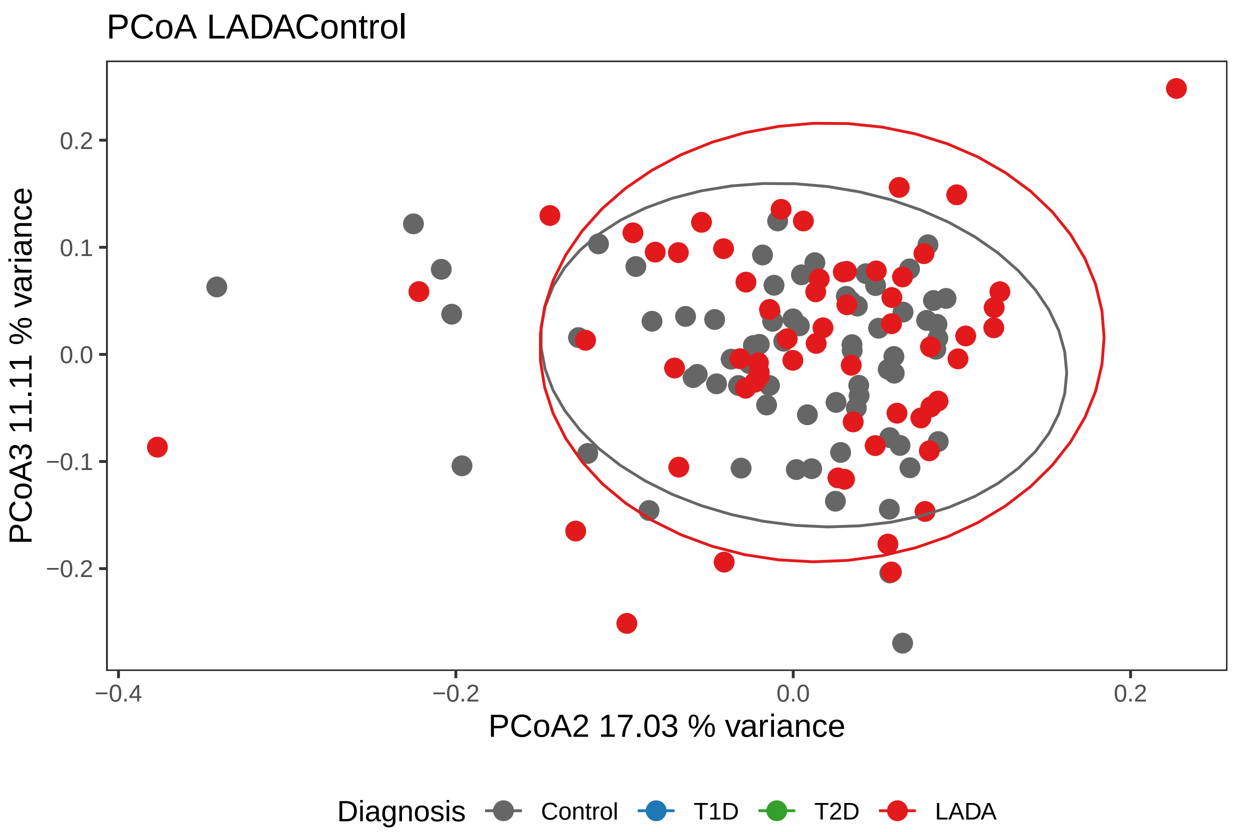
PCoA LADAControl
0.2
0.1
0.0
−0.1
−0.2
PCoA3 11.11 % variance
−0.4
−0.2
0.0
0.2
PCoA2 17.03 % variance
Diagnosis
Control
T1D
T2D
LADA

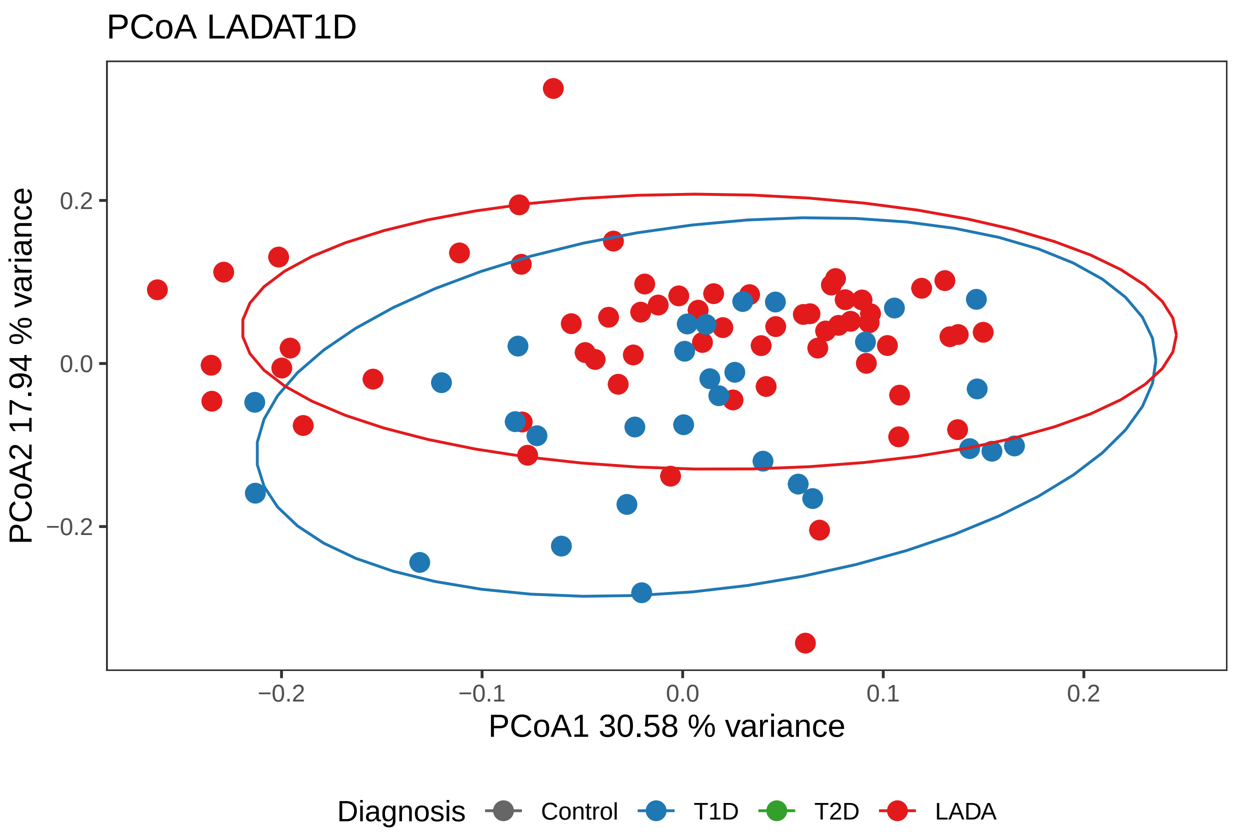
PCoA LADAT1D
PCoA2 17.94 % variance
0.2
0.0
−0.2
−0.2
−0.1
0.0
0.1
0.2
PCoA1 30.58 % variance
Diagnosis
Control
T1D
T2D
LADA

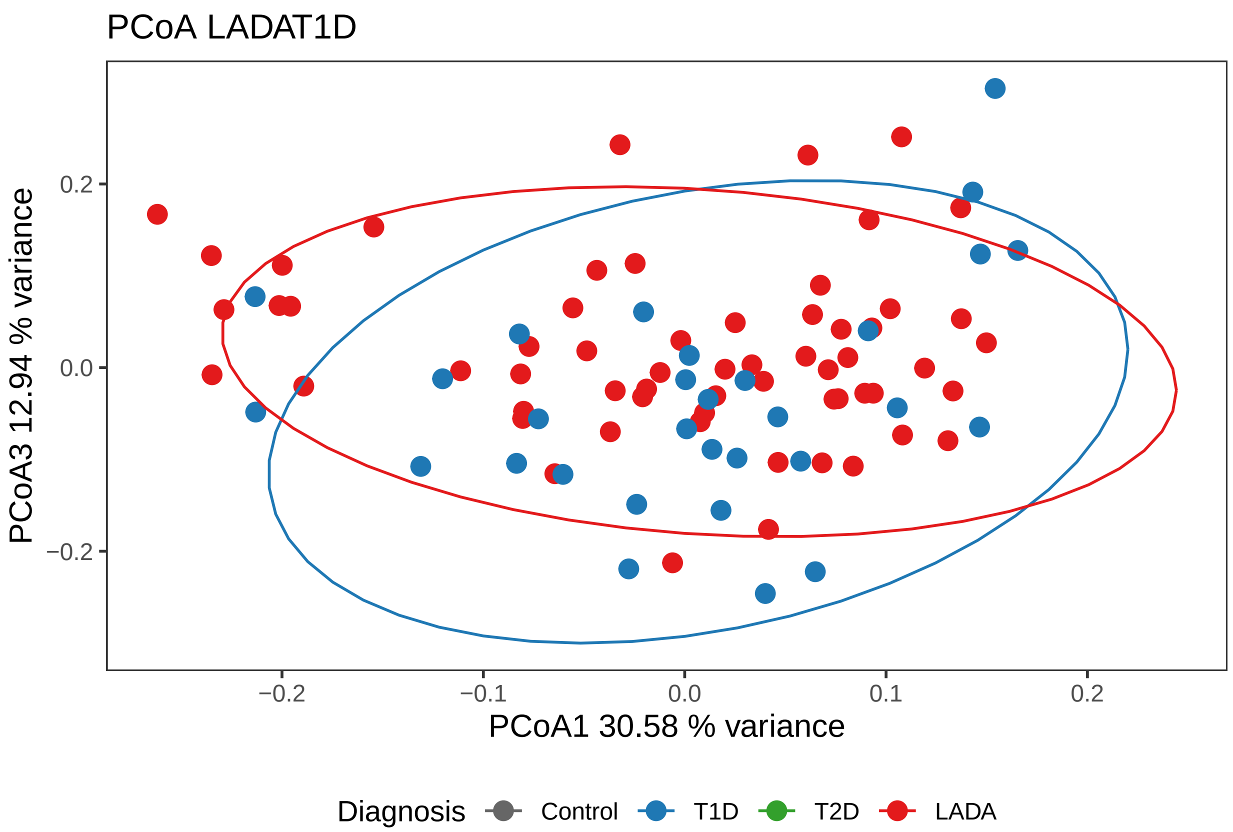
PCoA LADAT1D
PCoA3 12.94 % variance
0.2
0.0
−0.2
−0.2
−0.1
0.0
0.1
0.2
PCoA1 30.58 % variance
Diagnosis
Control
T1D
T2D
LADA

```
## [1] "LADAT2D"
```

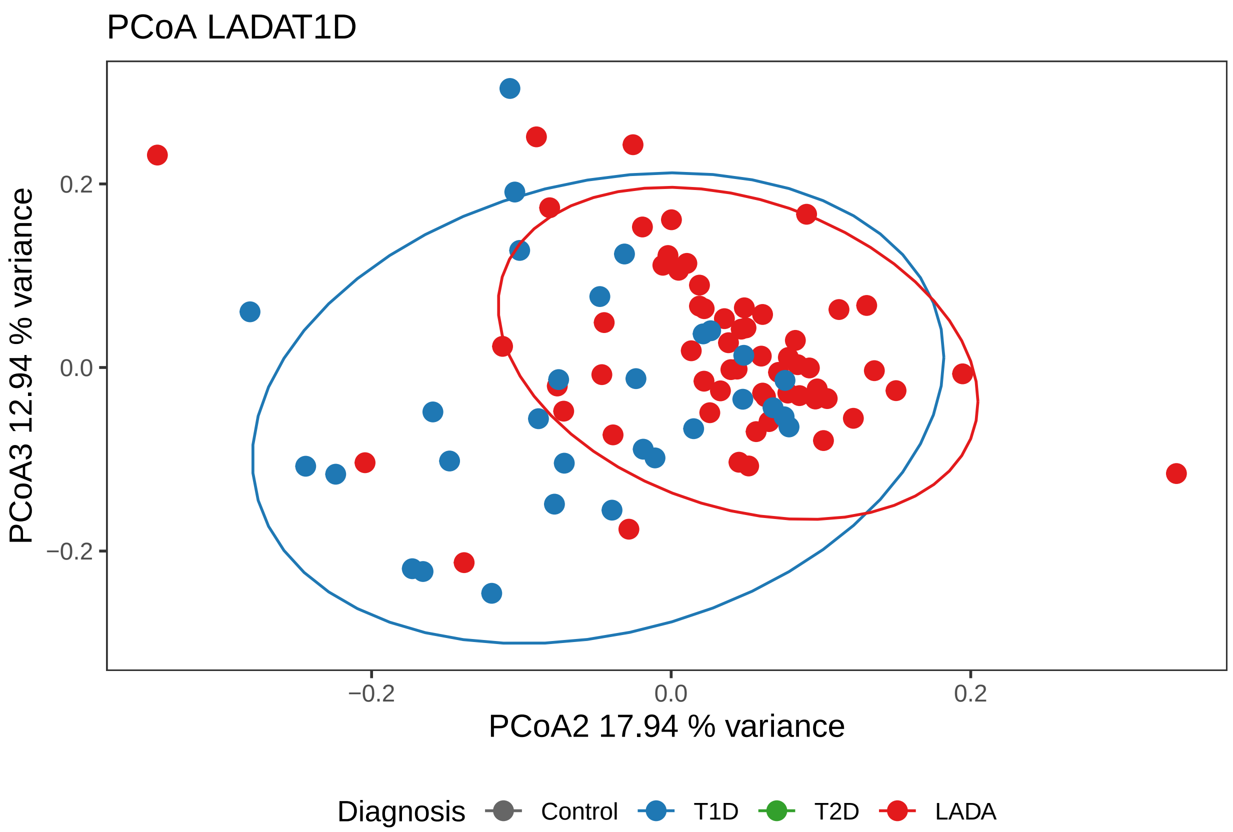
PCoA LADAT1D
0.2
0.0
−0.2
PCoA3 12.94 % variance
−0.2
0.0
0.2
PCoA2 17.94 % variance
Diagnosis
Control
T1D
T2D
LADA

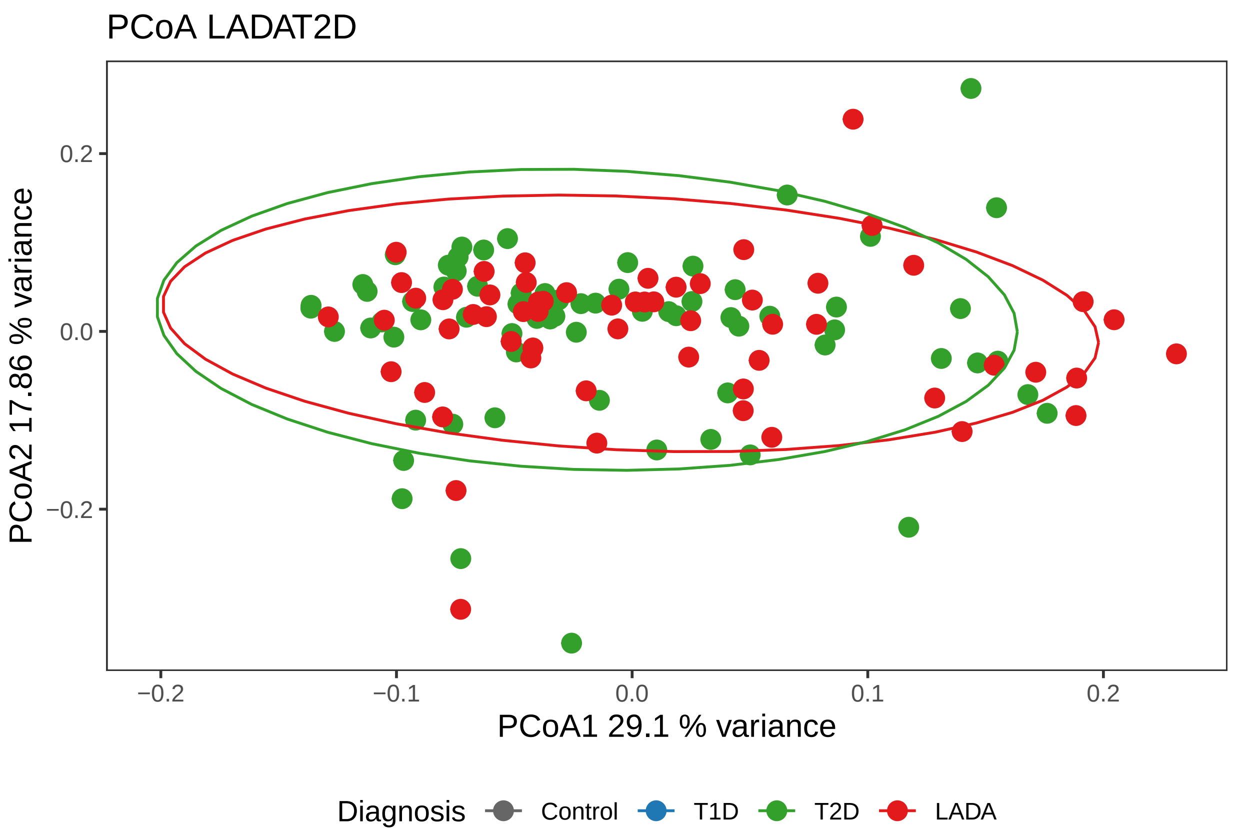
PCoA LADAT2D
PCoA2 17.86 % variance
PCoA1 29.1 % variance
0.2
0.0
−0.2
−0.2
−0.1
0.0
0.1
0.2
Diagnosis
Control
T1D
T2D
LADA

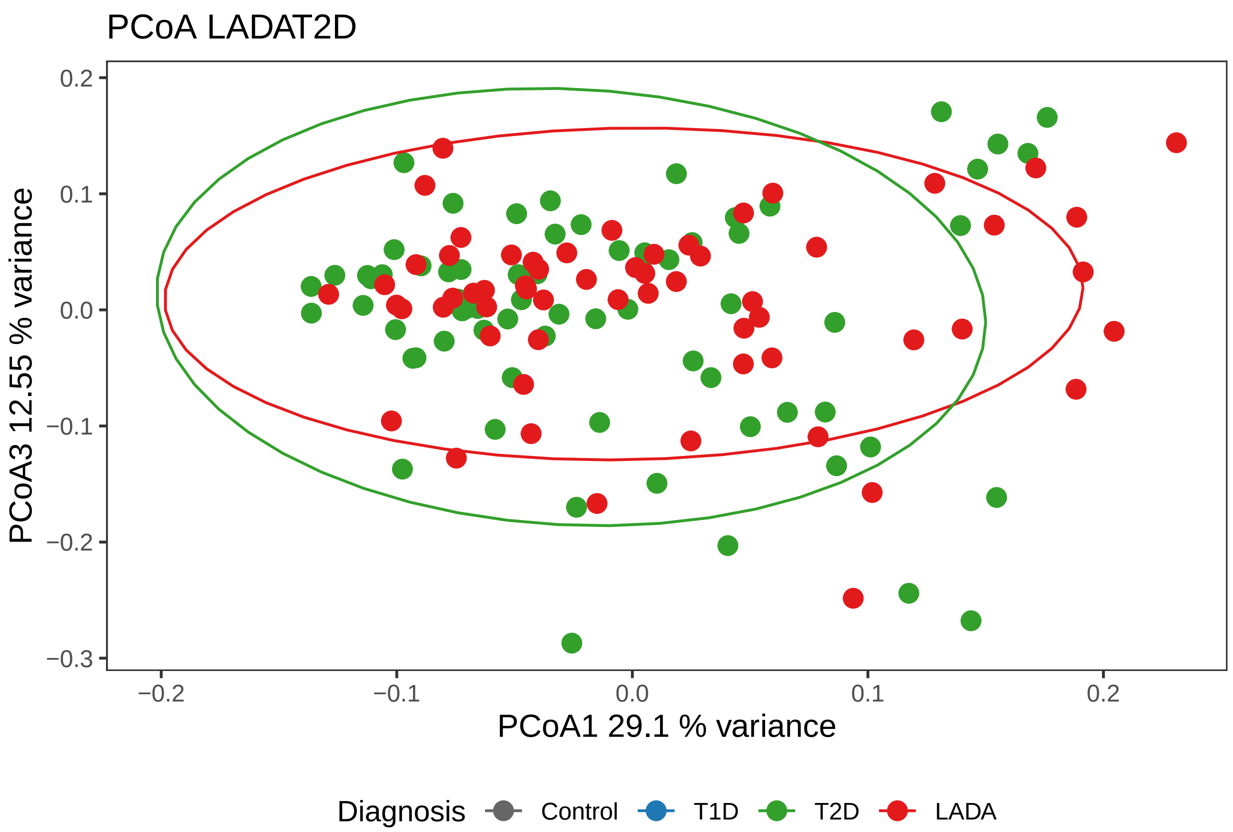

PCoA LADAT2D
PCoA3 12.55 % variance
PCoA1 29.1 % variance
0.2
0.1
0.0
−0.1
−0.2
−0.3
−0.2
−0.1
0.0
0.1
0.2
Diagnosis
Control
T1D
T2D
LADA


```
## [1] "All"
```

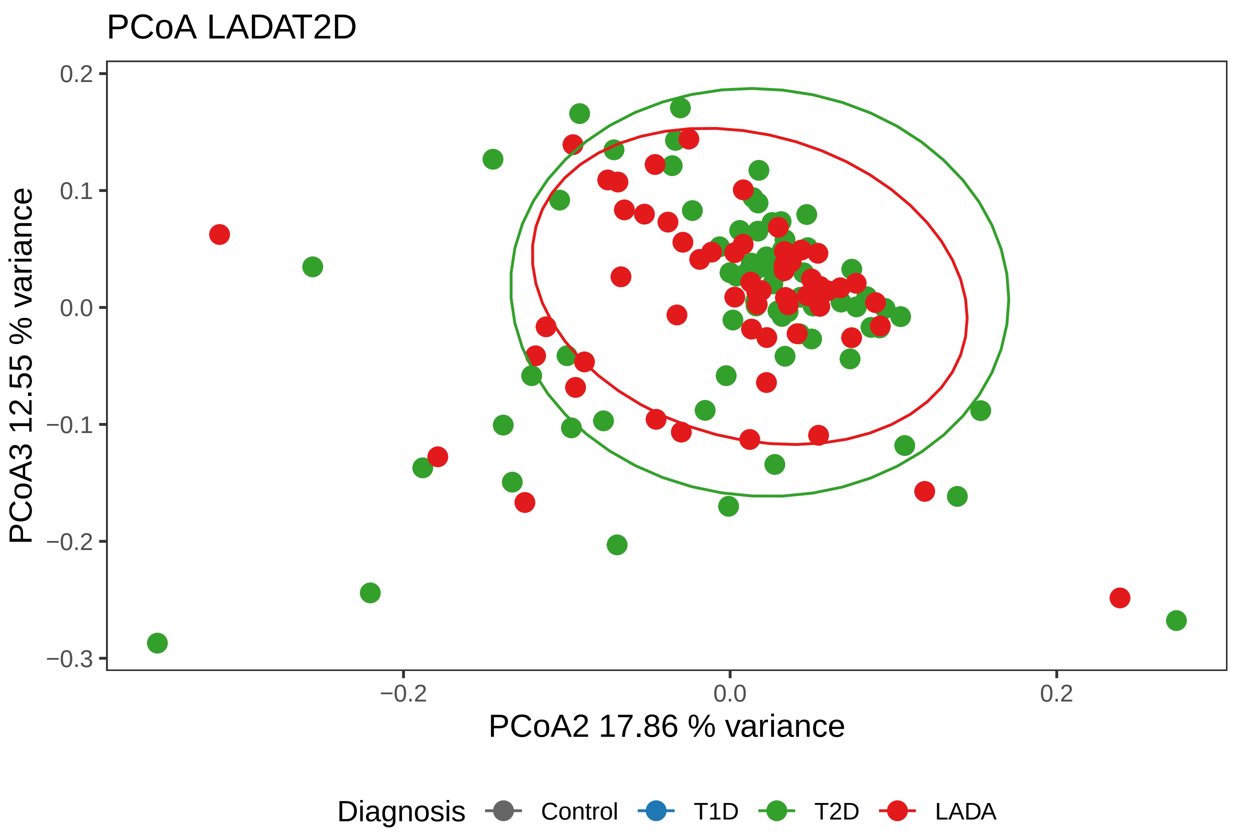

PCoA LADAT2D
0.2
0.1
0.0
−0.1
−0.2
−0.3
PCoA3 12.55 % variance
−0.2
0.0
0.2
PCoA2 17.86 % variance
Diagnosis
Control
T1D
T2D
LADA

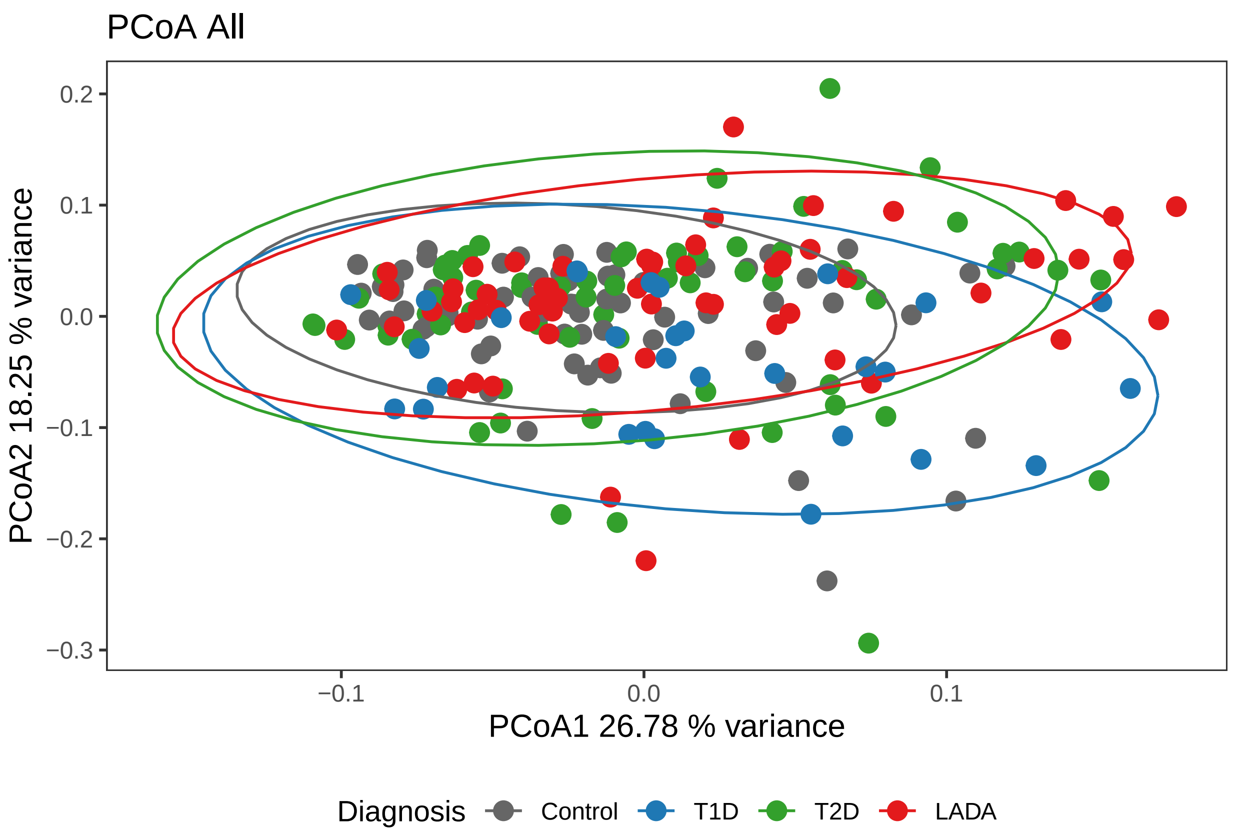

PCoA All
0.2
0.1
0.0
−0.1
−0.2
−0.3
PCoA2 18.25 % variance
−0.1
0.0
0.1
PCoA1 26.78 % variance
Diagnosis
Control
T1D
T2D
LADA

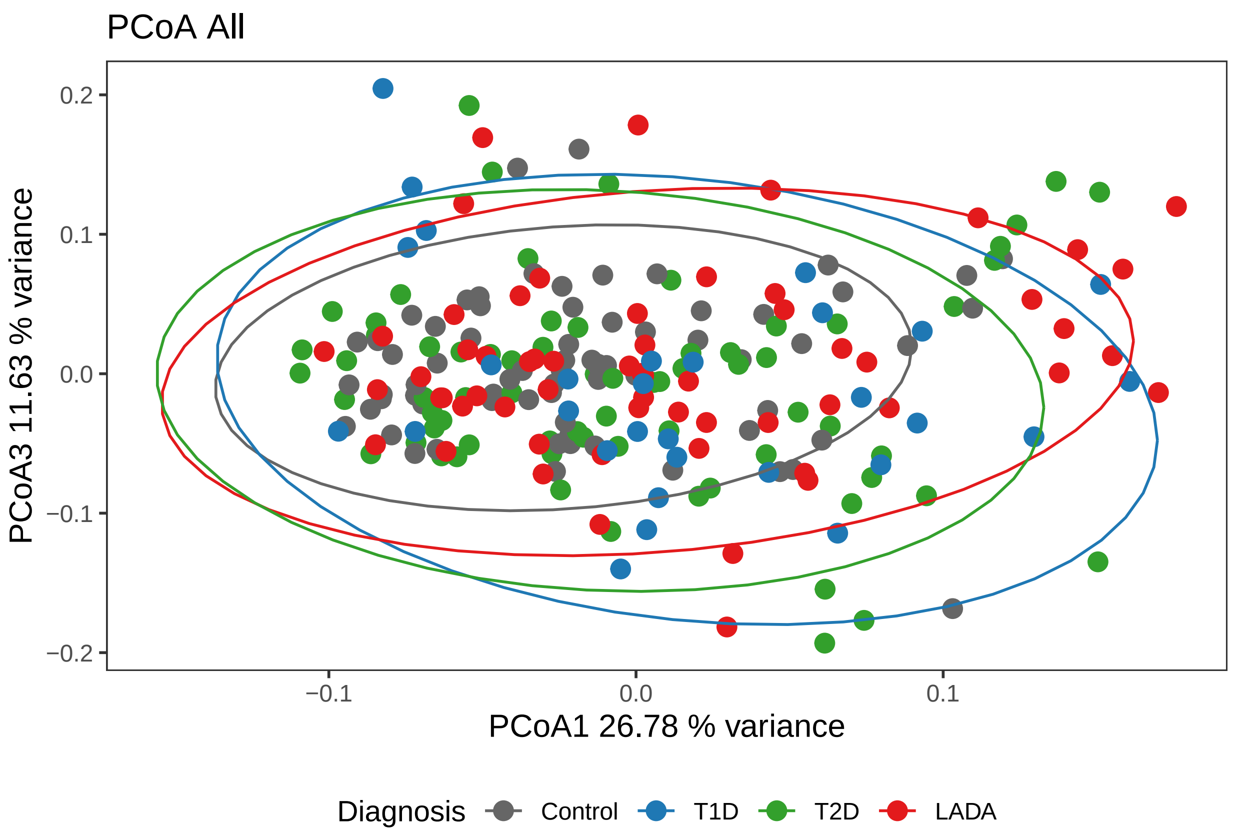
PCoA All
0.2
0.1
0.0
−0.1
−0.2
PCoA3 11.63 % variance
−0.1
0.0
0.1
PCoA1 26.78 % variance
Diagnosis
Control
T1D
T2D
LADA

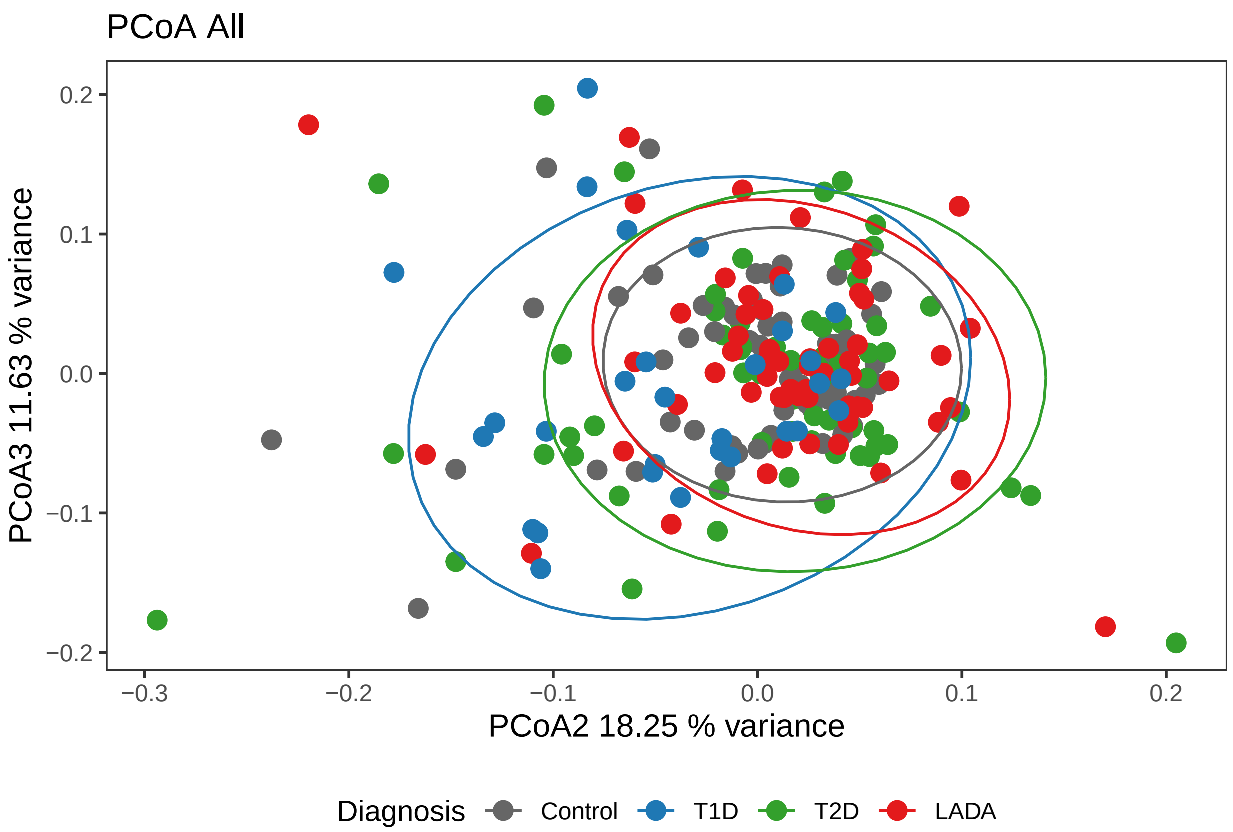
PCoA All
PCoA3 11.63 % variance
PCoA2 18.25 % variance
0.2
0.1
0.0
−0.1
−0.2
−0.3
Diagnosis
Control
T1D
T2D
LADA

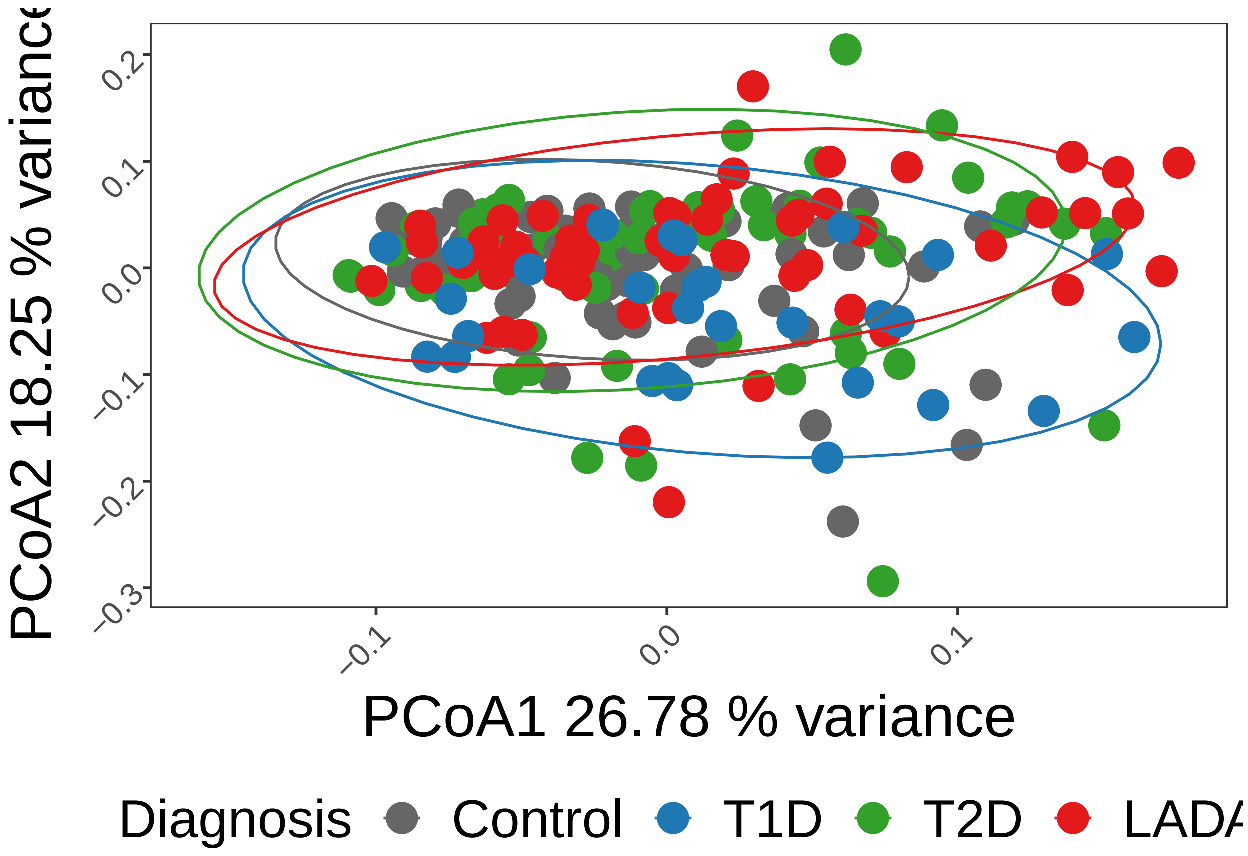

PCoA remove metformin

```
rm(list=setdiff(ls(), c("Metadata", "MetadataMed", "Feature", "TaxonomyDA", "Taxonomy",
                       "Fig1List", "Fig1ListRemMet")))
##Removing treated with metformin
#Metadata$Metformin <- ifelse(is.na(Metadata$Metformin), "Unknown", Metadata$Metformin)
Metadata2<-filter(Metadata, !Metformin == 1)
##Applying subsetting to OTU tables
Taxonomy2<-dplyr::select(Taxonomy, one_of(as.character(Metadata2$MicrobiomeID)))
##Remove orgs that are not present after subsetting.
Taxonomy2 <- Taxonomy2[rowSums(Taxonomy2)>0,]

Subset <- c("LADAControl", "LADAT1D", "LADAT2D", "All")
#i <- c("All")
#i <- c("LADAControl")
for (i in Subset) {

#Subsetting Metadata according to
if (i=="All") {
  print(i)
  Metadata3 <- Metadata2
} else if (i=="LADAControl") {
  print(i)
  Metadata3<-filter(Metadata2, Diagnosis == "LADA" | Diagnosis == "Control")
```

```
} else if (i=="LADAT1D") {
  print(i)
  Metadata3<-filter(Metadata2, Diagnosis == "LADA" | Diagnosis == "T1D")
} else if (i=="LADAT2D") {
  print(i)
  Metadata3<-filter(Metadata2, Diagnosis == "LADA" | Diagnosis == "T2D")
} else {
  print("Subset defined not valid")
}

#Apply subsetting to Taxonomy table
Taxonomy3<-dplyr::select(Taxonomy2, one_of(as.character(Metadata3$MicrobiomeID)))


#Hellinger transformation
Taxonomy3 <- data.frame(t(decostand(t(Taxonomy3), method="hellinger")))
#Maks TSS
Taxonomy3<-sweep(Taxonomy3, 2, colSums(Taxonomy3), FUN="/")
#Dissimilarity
distmatrix <- vegdist(t(Taxonomy3), method="bray")
#Multi dimensional scaling with capscale
PCoAcsObject<-capscale(distmatrix~1)


##Add eig to plot axes. with cmdscale there are negative values not with capscale
eig <- PCoAcsObject$CA$eig
# Calculate the variation explained by PCoA1, 2, 3 and 4
# and use it to generate axis labels
eig_1_2 <- eig[1:4] / sum(eig) * 100 #Vector with variance explained
#  by the first 4 axes
eig_1 <- paste("PCoA1", round(eig_1_2[1], digits = 2), "% variance")
eig_2 <- paste("PCoA2", round(eig_1_2[2], digits = 2), "% variance")
eig_3 <- paste("PCoA3", round(eig_1_2[3], digits = 2), "% variance")
eig_4 <- paste("PCoA4", round(eig_1_2[4], digits = 2), "% variance")

##Pull out coordinates for plotting from the ca object
#Structuring to add to Metadata2
PCoACA<-PCoAcsObject$CA #The ca object contains the actual ordination results:
#u ((Weighted) orthonormal site scores),
#v ((Weighted) orthonormal species scores) all na in mine (unconstrained),
#Xbar (The standardized data matrix after previous stages of analysis),
#and imaginary.u.eig ???.
#Info http://cc.oulu.fi/~jarioksa/softhelp/vegan/html/cca.object.html
PCoA<-as.data.frame(PCoACA$u)
#Change colnames. Now add dis and trans info to names
colnames(PCoA) <- c("MDS1BrayHel","MDS2BrayHel", "MDS3BrayHel","MDS4BrayHel")
#Add row names to df
PCoA$MicrobiomeID <- row.names(PCoA)
#Remove leading X
PCoA$MicrobiomeID <- gsub("X", "", PCoA$MicrobiomeID)
#Merge according to Sample
Metadata4<-merge(Metadata3, PCoA, by="MicrobiomeID")
```

```
#PCoA MDS1 and MDS2 pdf
pdf(paste("MicroLADA_PCoARemMet", i, "_Func.pdf", sep=""), width=9, height=6)
print(ggplot(Metadata4) +
  geom_point(aes(x=MDS1BrayHel, y=MDS2BrayHel, color = Diagnosis,
                 group = Diagnosis), size=3) +
  stat_ellipse(aes(MDS1BrayHel, y=MDS2BrayHel, color = Diagnosis,
                   group = Diagnosis)) +
  scale_color_manual(values=c(Control = "#666666", T1D = "#1F78B4",
                              T2D = "#33A02C", LADA = "#E31A1C")) +
  ggtitle(paste("PCoA", i, sep=" ")) +
  labs(colour="Diagnosis", x = eig_1, y = eig_2) +
  theme_bw() +
  theme(panel.grid.major = element_blank(), panel.grid.minor = element_blank(),
        axis.title=element_text(size=12), legend.position="bottom"))
dev.off()

#PCoA MDS1 and MDS2 plotly
print(ggplotly(ggplot(Metadata4[, c(6,8,13,14)]) +
  geom_point(aes(x=MDS1BrayHel, y=MDS2BrayHel, color = Diagnosis,
                 group = Dg_LADA), size=3) +
  stat_ellipse(aes(MDS1BrayHel, y=MDS2BrayHel, color = Diagnosis,
                   group = Diagnosis)) +
  scale_color_manual(values=c(Control = "#666666", T1D = "#1F78B4",
                              T2D = "#33A02C", LADA = "#E31A1C")) +
  ggtitle(paste("PCoA", i, sep=" ")) +
  labs(colour="Diagnosis", x = eig_1, y = eig_2) +
  theme_bw() +
  theme(panel.grid.major = element_blank(), panel.grid.minor = element_blank(),
        axis.title=element_text(size=12), legend.position="bottom")))

#PCoA MDS1 and MDS2
print(ggplot(Metadata4) +
  geom_point(aes(x=MDS1BrayHel, y=MDS2BrayHel, color = Diagnosis,
                 group = Diagnosis), size=3) +
  stat_ellipse(aes(MDS1BrayHel, y=MDS2BrayHel, color = Diagnosis,
                   group = Diagnosis)) +
  scale_color_manual(values=c(Control = "#666666", T1D = "#1F78B4",
                              T2D = "#33A02C", LADA = "#E31A1C")) +
  ggtitle(paste("PCoA", i, sep=" ")) +
  labs(colour="Diagnosis", x = eig_1, y = eig_2) +
  theme_bw() +
  theme(panel.grid.major = element_blank(), panel.grid.minor = element_blank(),
        axis.title=element_text(size=12), legend.position="bottom"))

#PCoA MDS1 and MDS3
print(ggplot(Metadata4) +
  geom_point(aes(x=MDS1BrayHel, y=MDS3BrayHel, color = Diagnosis,
                 group = Diagnosis), size=3) +
  stat_ellipse(aes(MDS1BrayHel, y=MDS3BrayHel, color = Diagnosis,
                   group = Diagnosis)) +
  scale_color_manual(values=c(Control = "#666666", T1D = "#1F78B4",
                              T2D = "#33A02C", LADA = "#E31A1C")) +
  ggtitle(paste("PCoA", i, sep=" ")) +
```

```r
  labs(colour="Diagnosis", x = eig_1, y = eig_3) +
  theme_bw() +
  theme(panel.grid.major = element_blank(), panel.grid.minor = element_blank(),
        axis.title=element_text(size=12), legend.position="bottom"))

#PCoA MDS2 and MDS3
print(ggplot(Metadata4) +
  geom_point(aes(x=MDS2BrayHel, y=MDS3BrayHel, color = Diagnosis,
                 group = Diagnosis), size=3) +
  stat_ellipse(aes(MDS2BrayHel, y=MDS3BrayHel, color = Diagnosis,
                   group = Diagnosis)) +
  scale_color_manual(values=c(Control = "#666666", T1D = "#1F78B4",
                              T2D = "#33A02C", LADA = "#E31A1C")) +
  ggtitle(paste("PCoA", i, sep=" ")) +
  labs(colour="Diagnosis", x = eig_2, y = eig_3) +
  theme_bw() +
  theme(panel.grid.major = element_blank(), panel.grid.minor = element_blank(),
        axis.title=element_text(size=12), legend.position="bottom"))

#Add a categorical indicator of group in Metadata
Metadata4$Metformine<-as.factor(ifelse(grepl("1", Metadata4$Metformin), "Yes",
                                ifelse(grepl("0", Metadata4$Metformin),
                                       "No", "Unknown")))
#Order Treatment metformine for plotting
Metadata4$Metformine <- factor(Metadata4$Metformine, levels=c("Yes", "No", "Unknown"))
#Create PCoA for figure 1
if (i=="All") {
  PCoAall<-ggplot(Metadata4) +
  geom_point(aes(MDS1BrayHel, MDS2BrayHel, color = Diagnosis, #shape = Metformine,
                 group = Diagnosis), size=5) +
  stat_ellipse(aes(MDS1BrayHel, MDS2BrayHel, color = Diagnosis, group = Diagnosis)) +
  #stat_ellipse(aes(MDS1BrayHel, MDS2BrayHel, group = Metformine,
  #                 linetype=factor(Metadata4$Metformine)), alpha = 0.7) +
  scale_color_manual(values=c(Control = "#666666", T1D = "#1F78B4",
                              T2D = "#33A02C", LADA = "#E31A1C")) +
  scale_shape_manual(values=c(3, 16)) +
  scale_linetype_manual(values=c("longdash", "dotted"), guide=FALSE) +
  #ggtitle(paste("PCoA", "LADA & T2D Metformin", sep=" ")) +
  labs(colour="Diagnosis", x = eig_1, y = eig_2) + #, shape="Metformin treatment"
  theme_bw() +
  theme(panel.grid.major = element_blank(), panel.grid.minor = element_blank(),
        axis.title=element_text(size=22), legend.position="bottom",
        legend.title=element_text(size=20), legend.text=element_text(size=20),
        axis.text.x = element_text(angle = 45, hjust = 1, size=12),
        axis.text.y = element_text(angle = 45, hjust = 1, size=12))

  print(PCoAall)
  pdf("MicroLADA_PCoAAllMetRemMet_Func.pdf", width=9, height=6)
  print(PCoAall)
  dev.off()
  #Save plot in previous list
  Fig1ListRemMet[[ "PCoAall" ]] <- PCoAall
  #return(PCoAall)
```

```
}

##Stressplot
##Extract ordination distances and merge with observed dissimilarity
#stress<-stressplot(PCoAcsObject)
#df <- melt(as.matrix(stress))
#names(df)<-c("rowOrd", "colOrd", "OrdDist")
#df<-filter(df, OrdDist>0)
#df2 <- melt(as.matrix(distmatrix))
#names(df2)<-c("rowObs", "colObs", "ObsDism")
#df2<-filter(df2, ObsDism>0)
#df<-unite(df, mergecol, c(rowOrd, colOrd), remove=FALSE)
#df2<-unite(df2, mergecol, c(rowObs, colObs), remove=FALSE)
#ggstress<-merge(df, df2, by="mergecol")

##create stressplot
#print(ggplot(ggstress) +
#  geom_point(aes(ObsDism, OrdDist)) +
#  ggtitle(paste("Stressplot", i, sep=" ")) +
#  labs(x = "Observed dissimilarity", y = "Ordination distance") +
#  theme_bw() +
#  theme(panel.grid.major = element_blank(), panel.grid.minor = element_blank(),
#        axis.title=element_text(size=12)))


##Screeplot
#screeplot<-data.frame(PCoAcsObject$CA$eig)
#colnames(screeplot)<-c("eig")
#screeplot$eig <- screeplot$eig[1:length(screeplot$eig)] /
#  sum(screeplot$eig) * 100
#screeplot<-add_rownames(screeplot, "MDS")
#screeplot$MDS <- factor(screeplot$MDS,
#                        levels=c(sprintf("MDS%d", 1:length(screeplot$eig))))

##create screeplot
#print(ggplot(screeplot, aes(x=MDS, y=eig)) +
#  geom_bar(stat="identity") +
#  labs(x ="MDS", y ="eig (%)") +
#  ggtitle(paste("Screeplot", i, sep=" ")) +
#  theme_bw() +
#  theme(panel.grid.major = element_blank(), panel.grid.minor = element_blank(),
#        axis.title=element_text(size=12), axis.text.x=element_blank(),
#        axis.ticks.x=element_blank()))

}
```

```
## [1] "LADAControl"
```

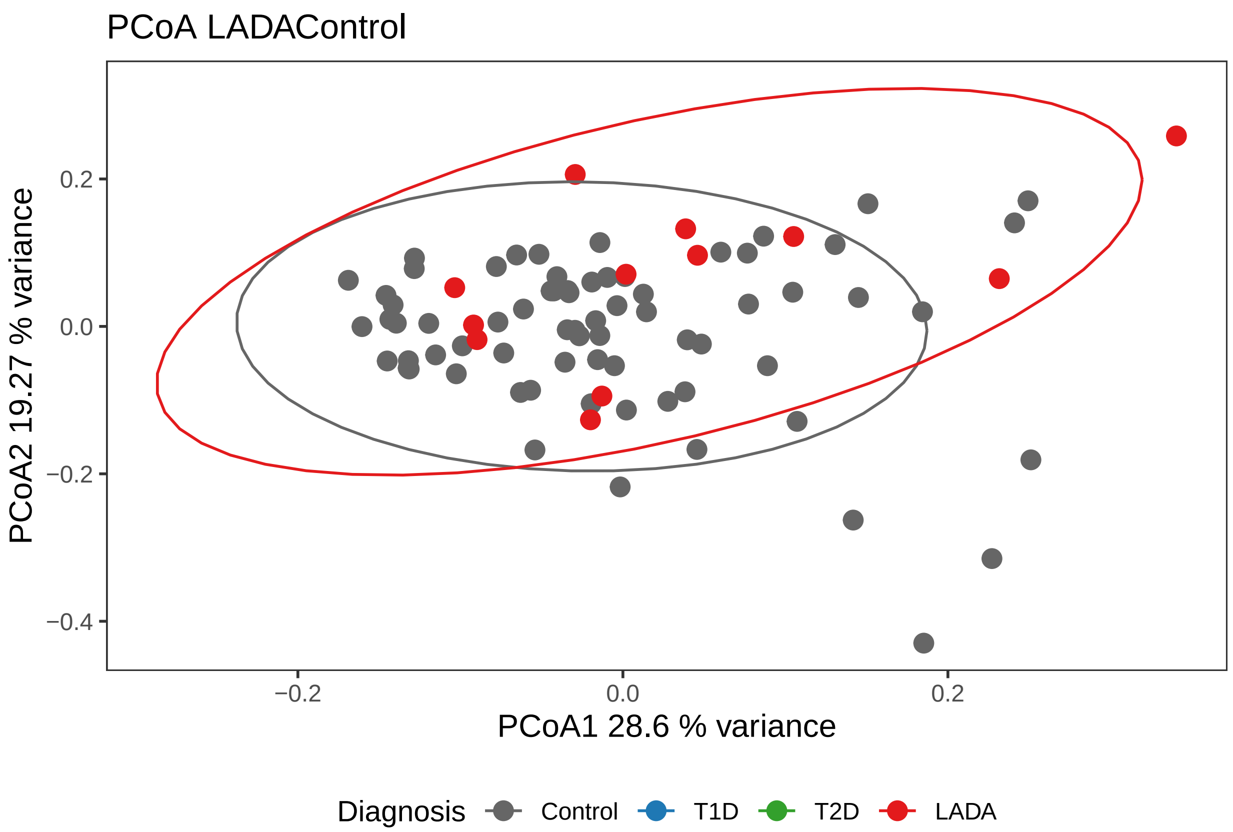

PCoA LADAControl
0.2
0.0
−0.2
−0.4
PCoA2 19.27 % variance
−0.2
0.0
0.2
PCoA1 28.6 % variance
Diagnosis
Control
T1D
T2D
LADA

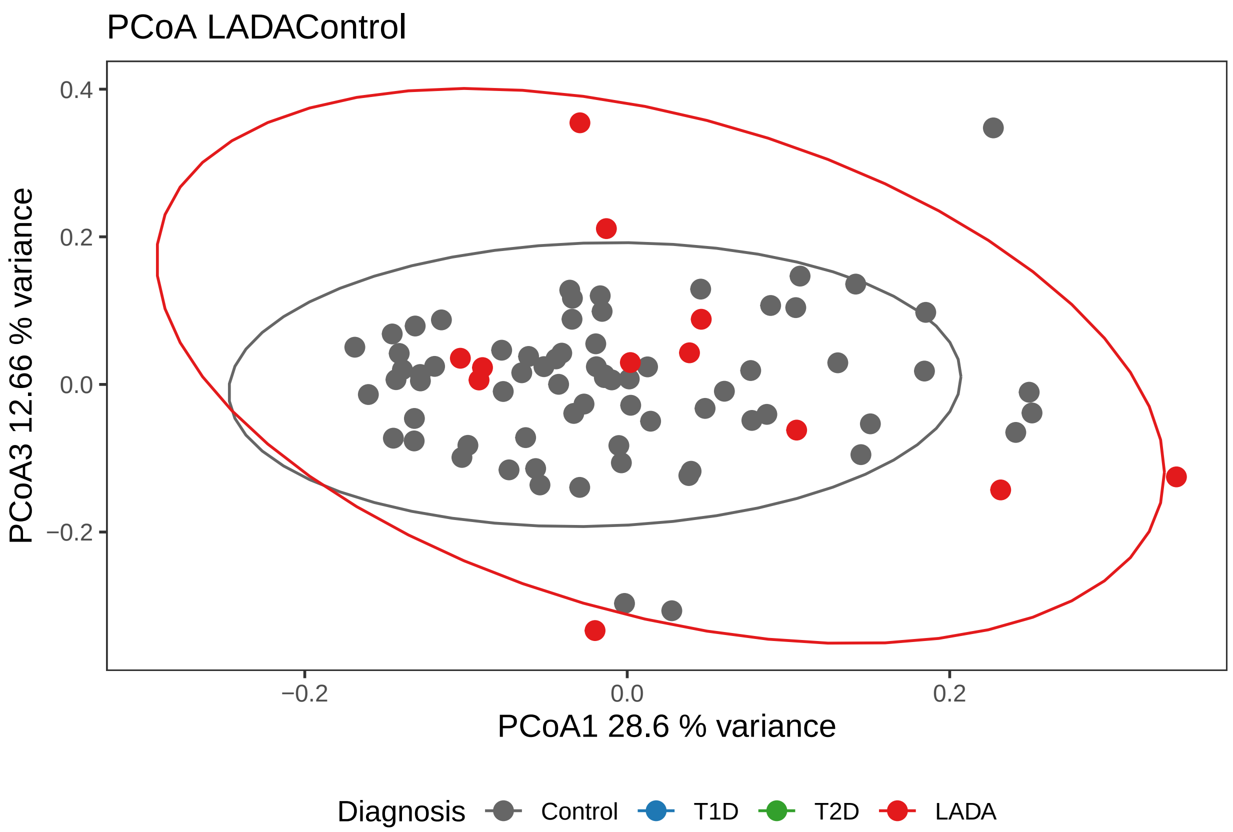
PCoA LADAControl
0.4
0.2
0.0
−0.2
PCoA3 12.66 % variance
−0.2
0.0
0.2
PCoA1 28.6 % variance
Diagnosis
Control
T1D
T2D
LADA

```
## [1] "LADAT1D"
```

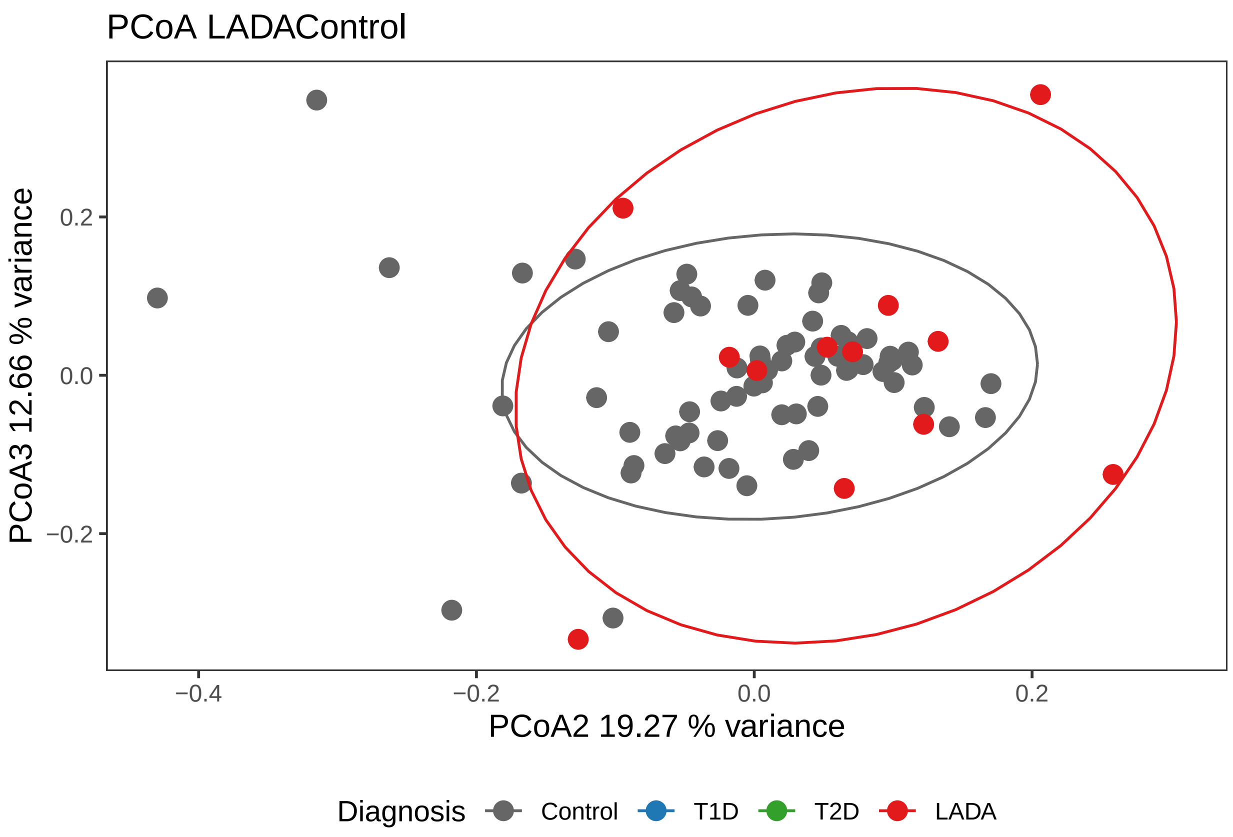
PCoA LADAControl
PCoA3 12.66 % variance
0.2
0.0
−0.2
−0.4
−0.2
0.0
0.2
PCoA2 19.27 % variance
Diagnosis
Control
T1D
T2D
LADA

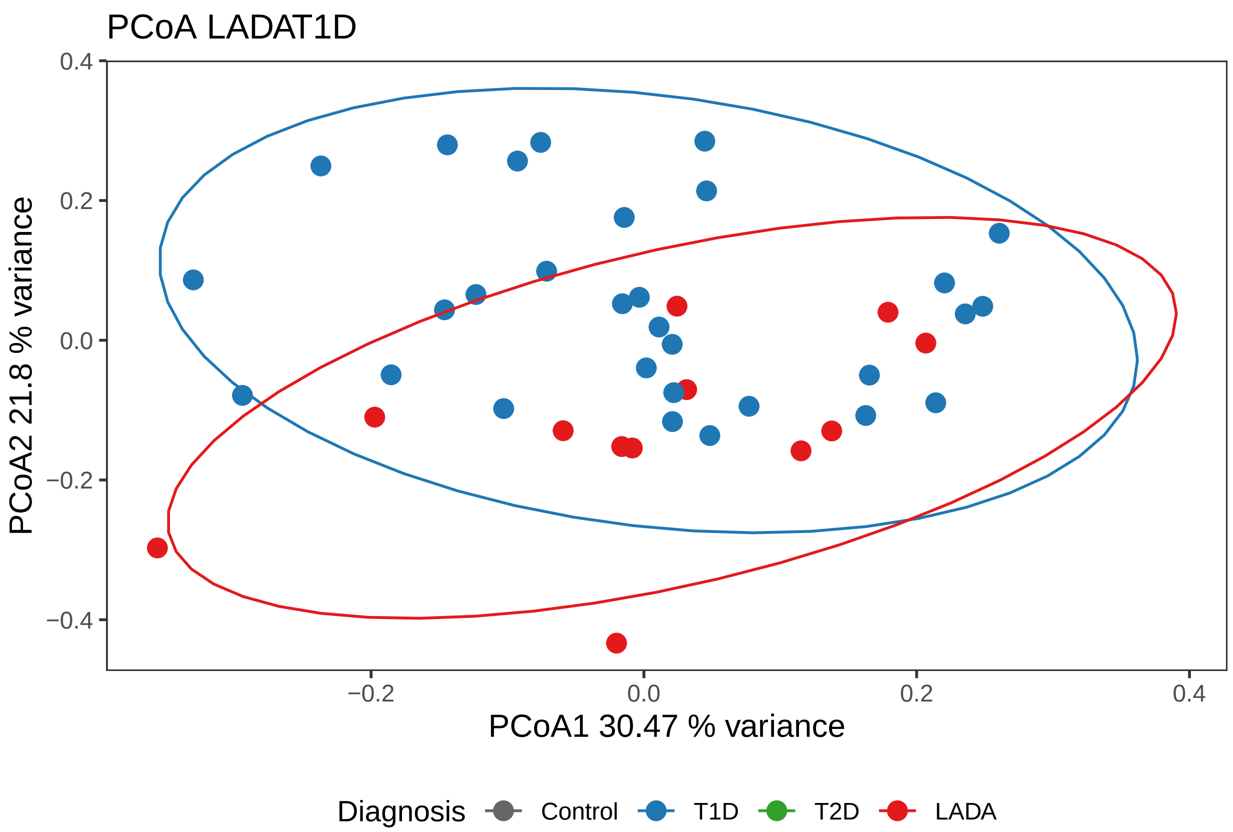

PCoA LADAT1D
0.4
0.2
0.0
−0.2
−0.4
PCoA2 21.8 % variance
−0.2
0.0
0.2
0.4
PCoA1 30.47 % variance
Diagnosis
Control
T1D
T2D
LADA

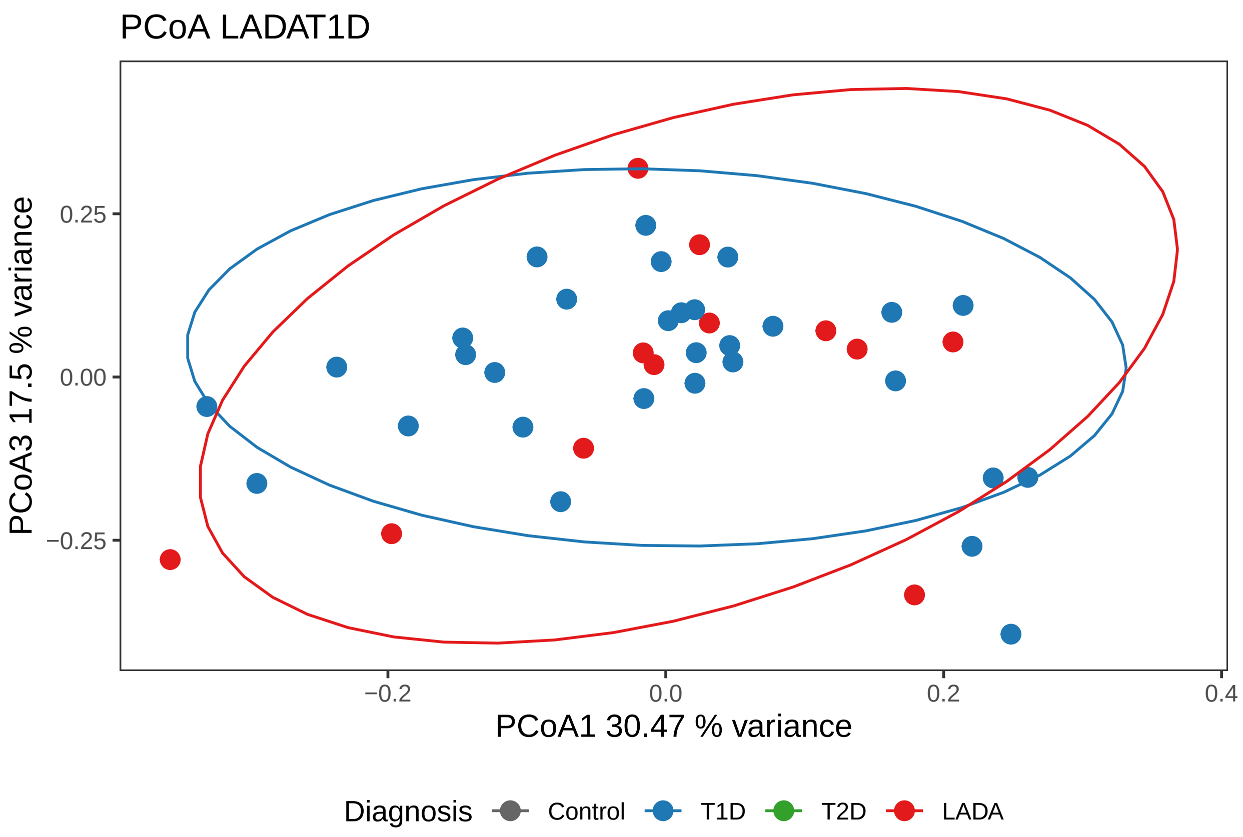
PCoA LADAT1D
PCoA3 17.5 % variance
0.25
0.00
−0.25
−0.2
0.0
0.2
0.4
PCoA1 30.47 % variance
Diagnosis
Control
T1D
T2D
LADA

```
## [1] "LADAT2D"
```

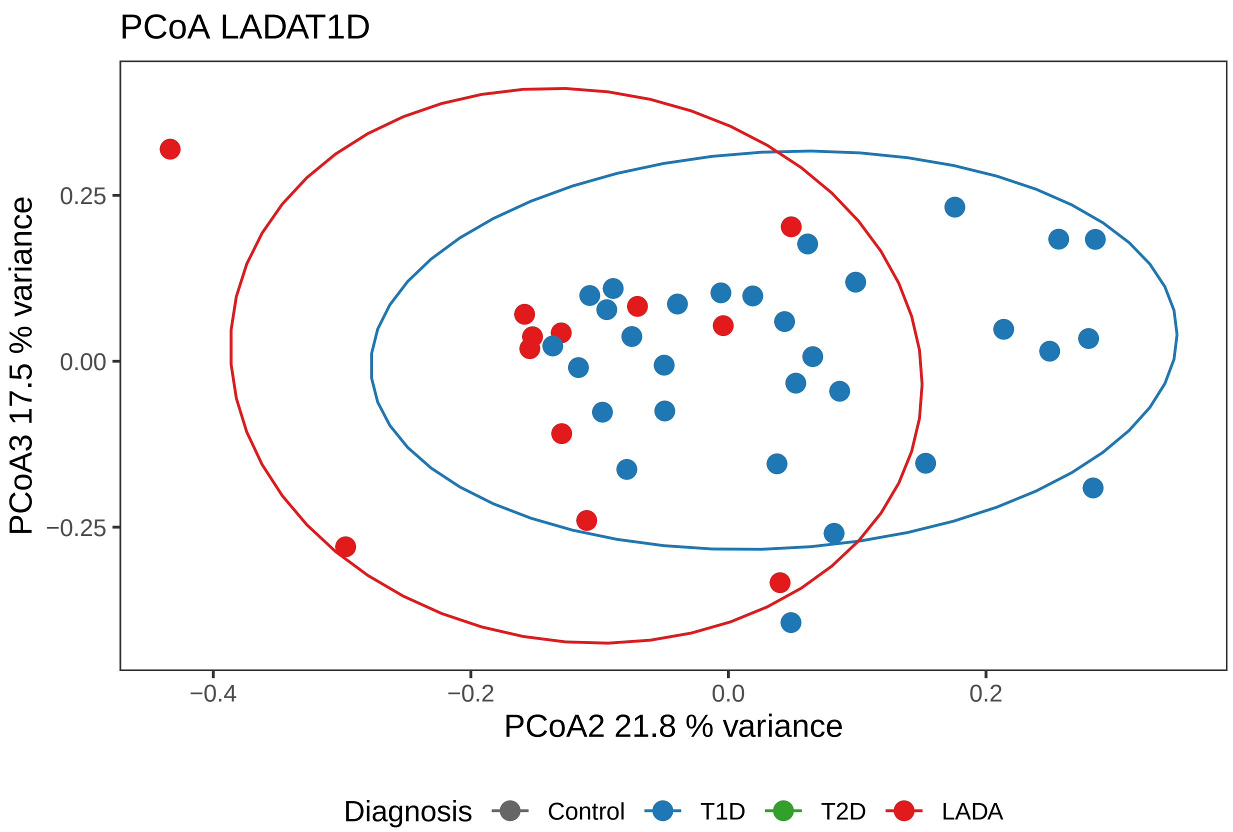

PCoA LADAT1D
PCoA3 17.5 % variance
0.25
0.00
−0.25
−0.4
−0.2
0.0
0.2
PCoA2 21.8 % variance
Diagnosis
Control
T1D
T2D
LADA

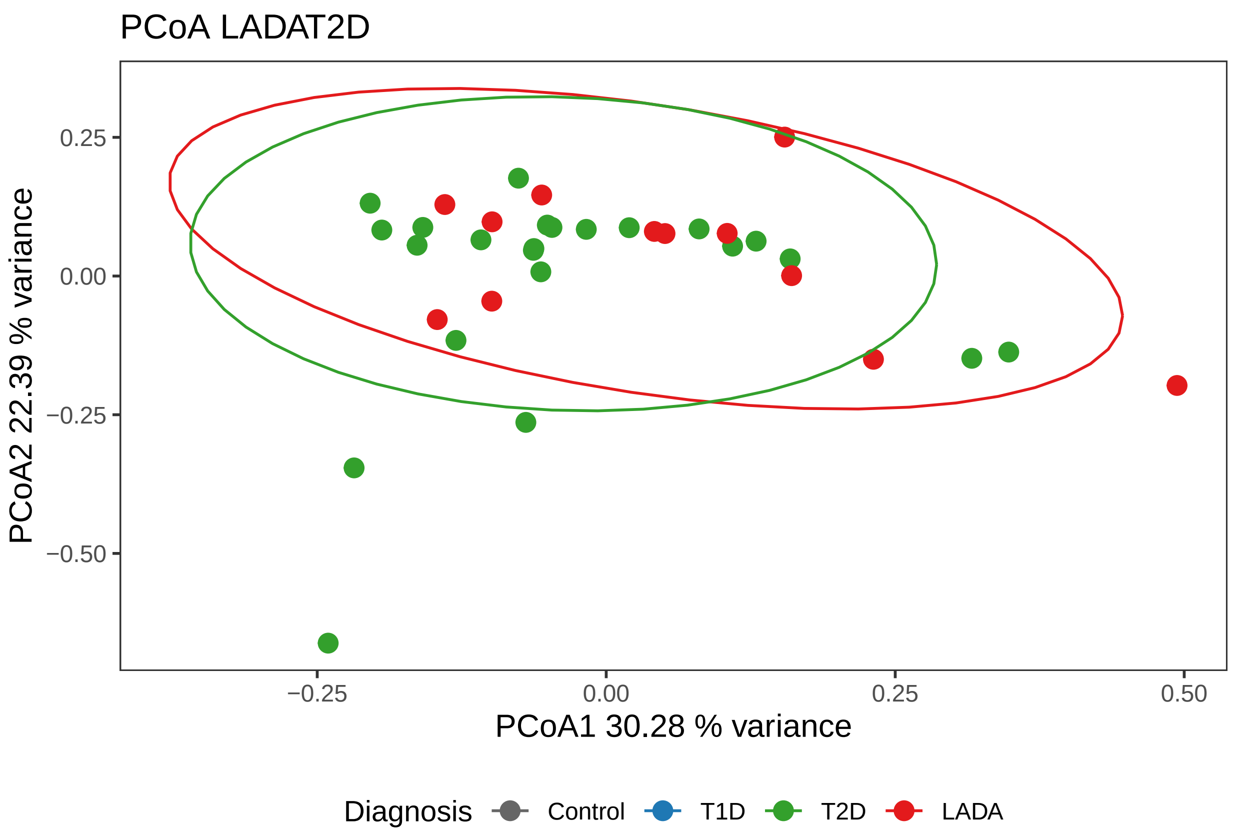
PCoA LADAT2D
PCoA2 22.39 % variance
0.25
0.00
−0.25
−0.50
−0.25
0.00
0.25
0.50
PCoA1 30.28 % variance
Diagnosis
Control
T1D
T2D
LADA

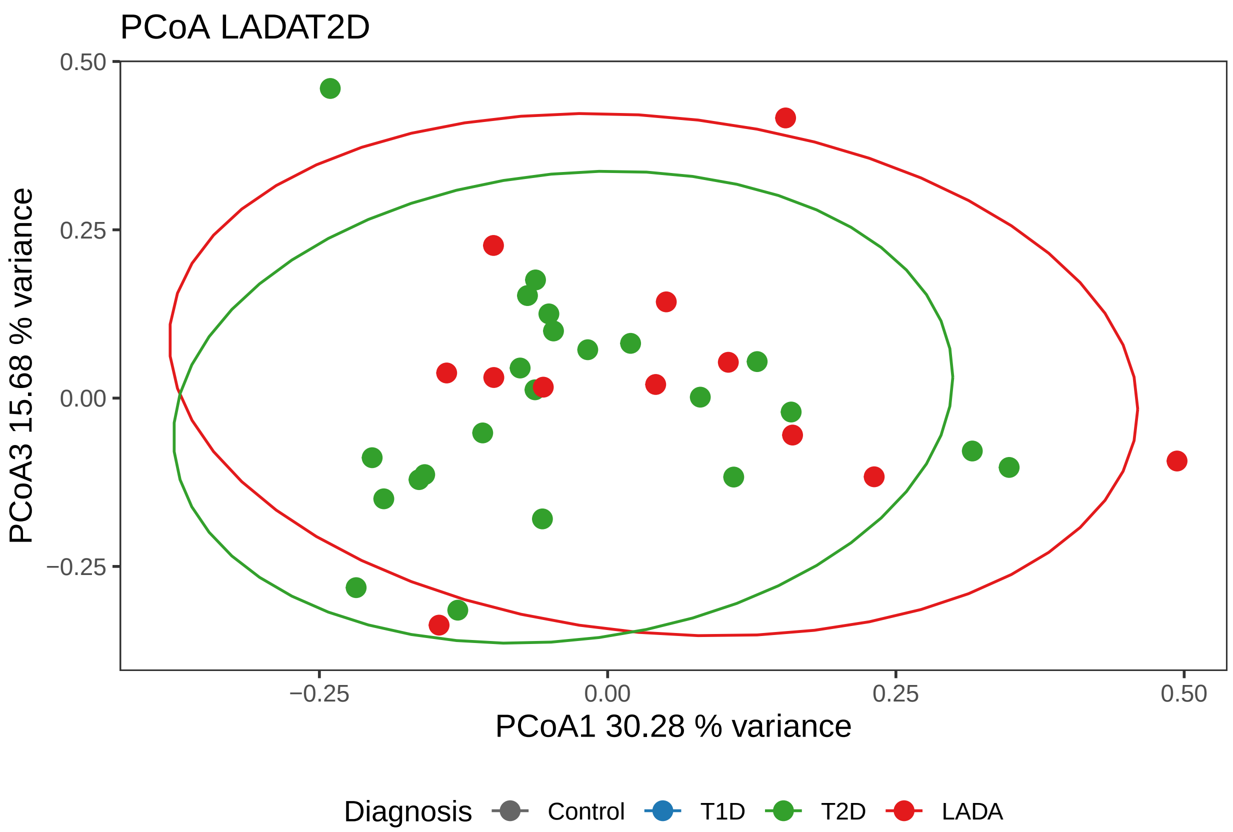

PCoA LADAT2D
PCoA3 15.68 % variance
0.50
0.25
0.00
−0.25
−0.25
0.00
0.25
0.50
PCoA1 30.28 % variance
Diagnosis
Control
T1D
T2D
LADA


```
## [1] "All"
```

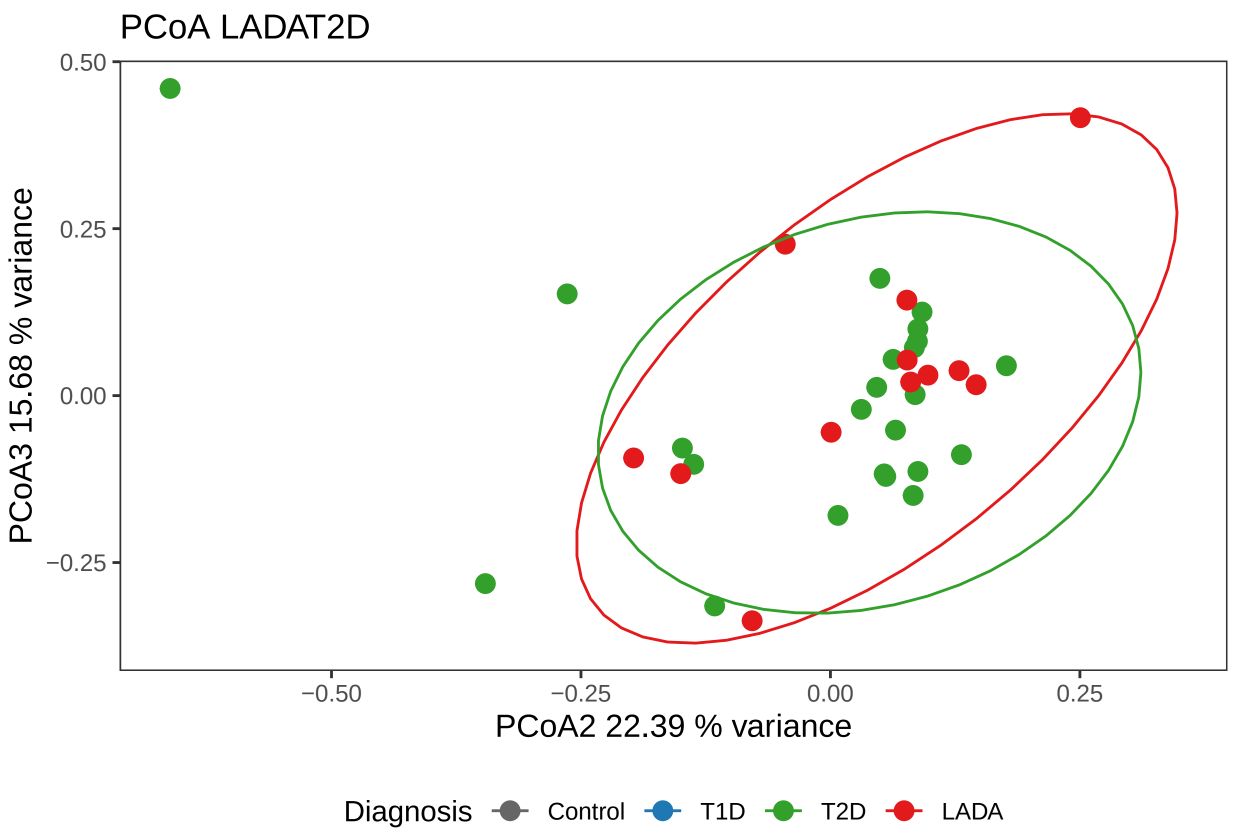
PCoA LADAT2D
0.50
0.25
0.00
−0.25
PCoA3 15.68 % variance
−0.50
−0.25
0.00
0.25
PCoA2 22.39 % variance
Diagnosis
Control
T1D
T2D
LADA

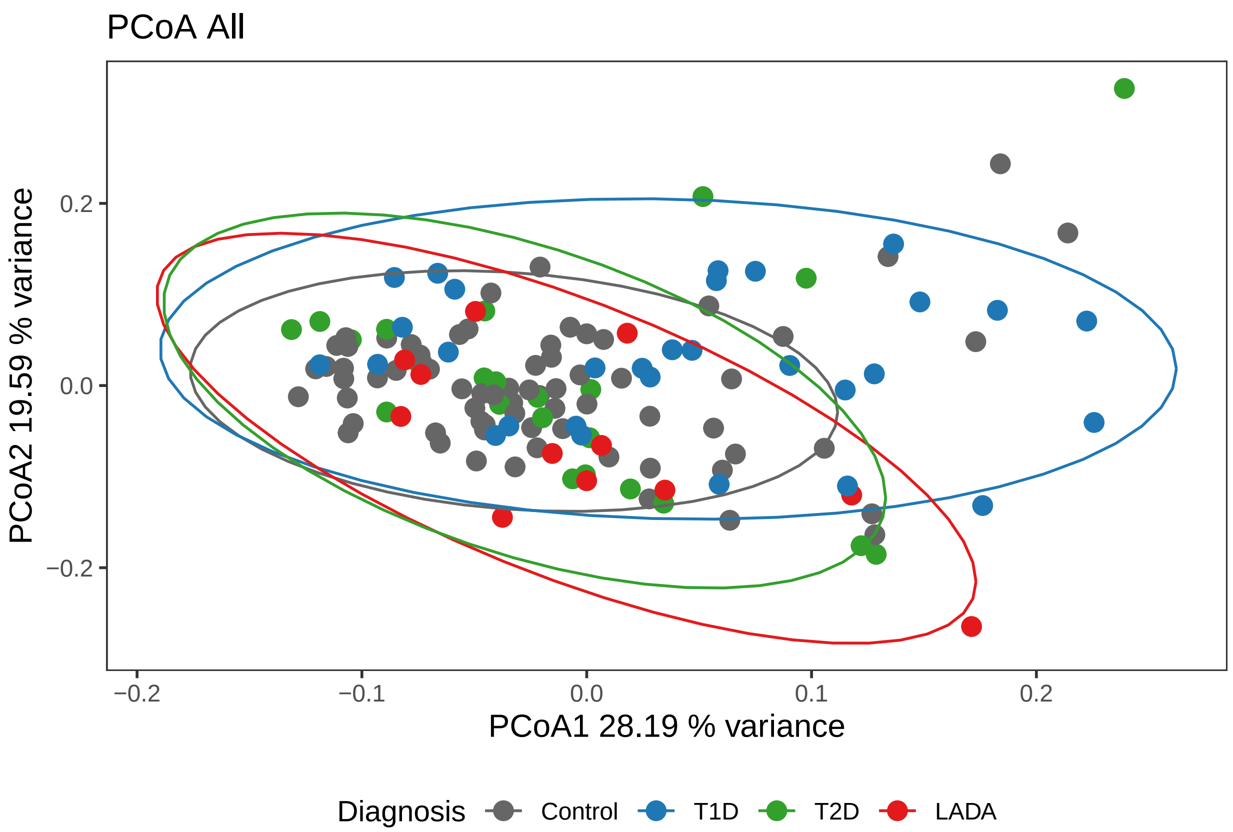
PCoA All
PCoA2 19.59 % variance
0.2
0.0
−0.2
−0.2
−0.1
0.0
0.1
0.2
PCoA1 28.19 % variance
Diagnosis
Control
T1D
T2D
LADA

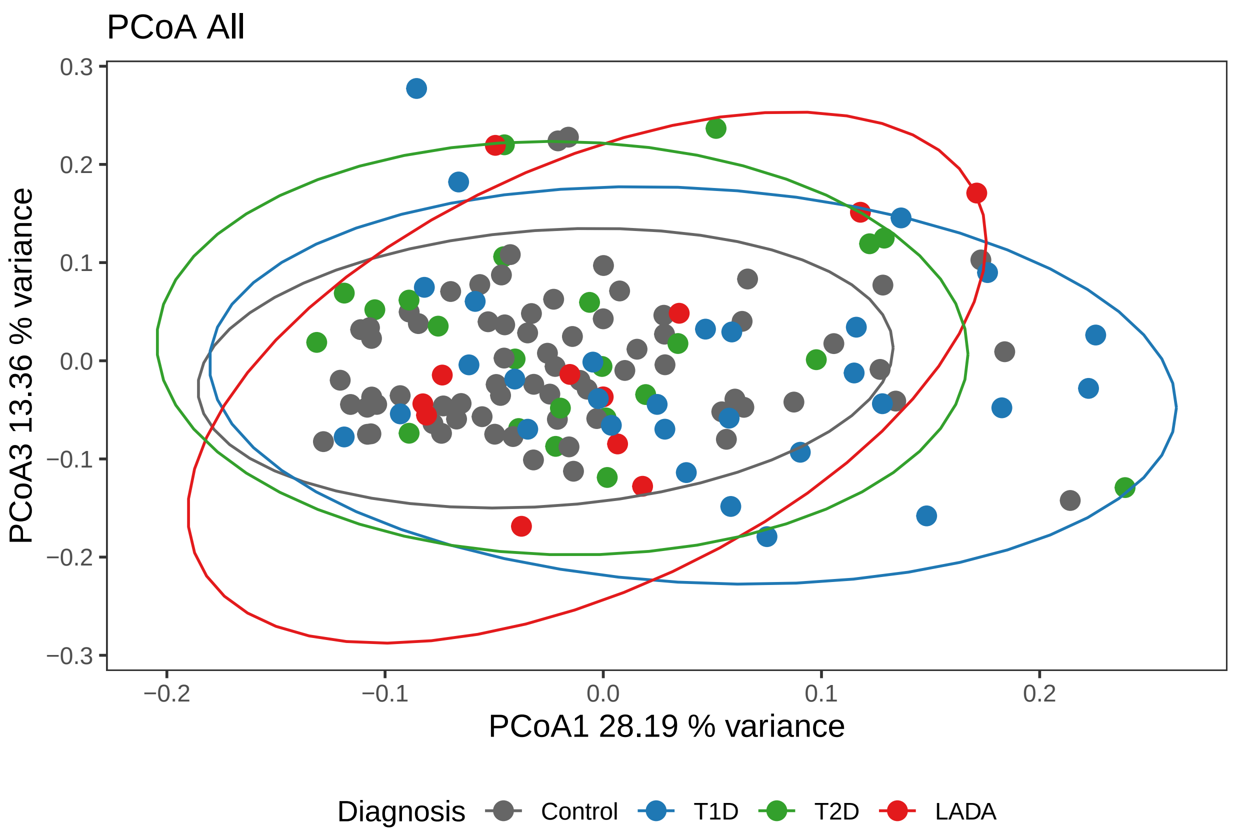
PCoA All
PCoA3 13.36 % variance
PCoA1 28.19 % variance
0.3
0.2
0.1
0.0
−0.1
−0.2
−0.3
−0.2
−0.1
0.0
0.1
0.2
Diagnosis
Control
T1D
T2D
LADA

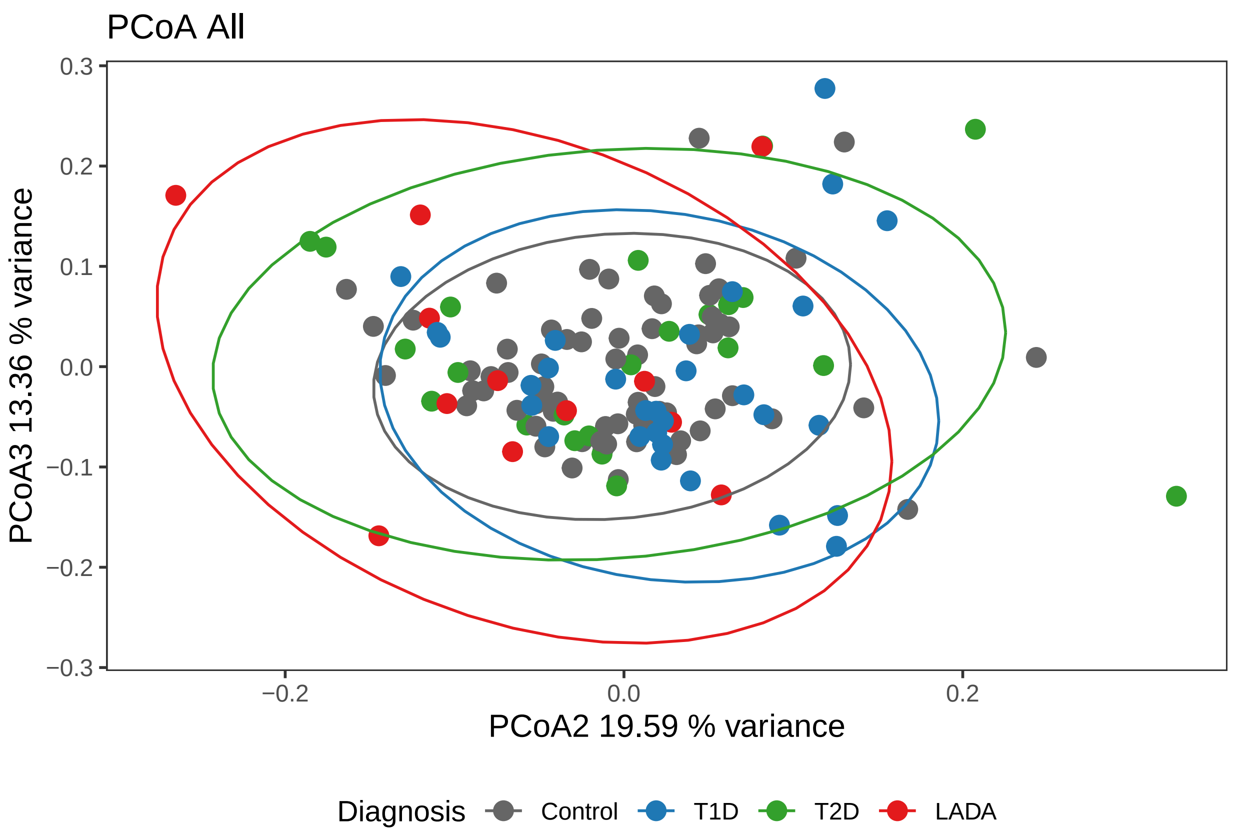
PCoA All
0.3
0.2
0.1
0.0
−0.1
−0.2
−0.3
PCoA3 13.36 % variance
−0.2
0.0
0.2
PCoA2 19.59 % variance
Diagnosis
Control
T1D
T2D
LADA

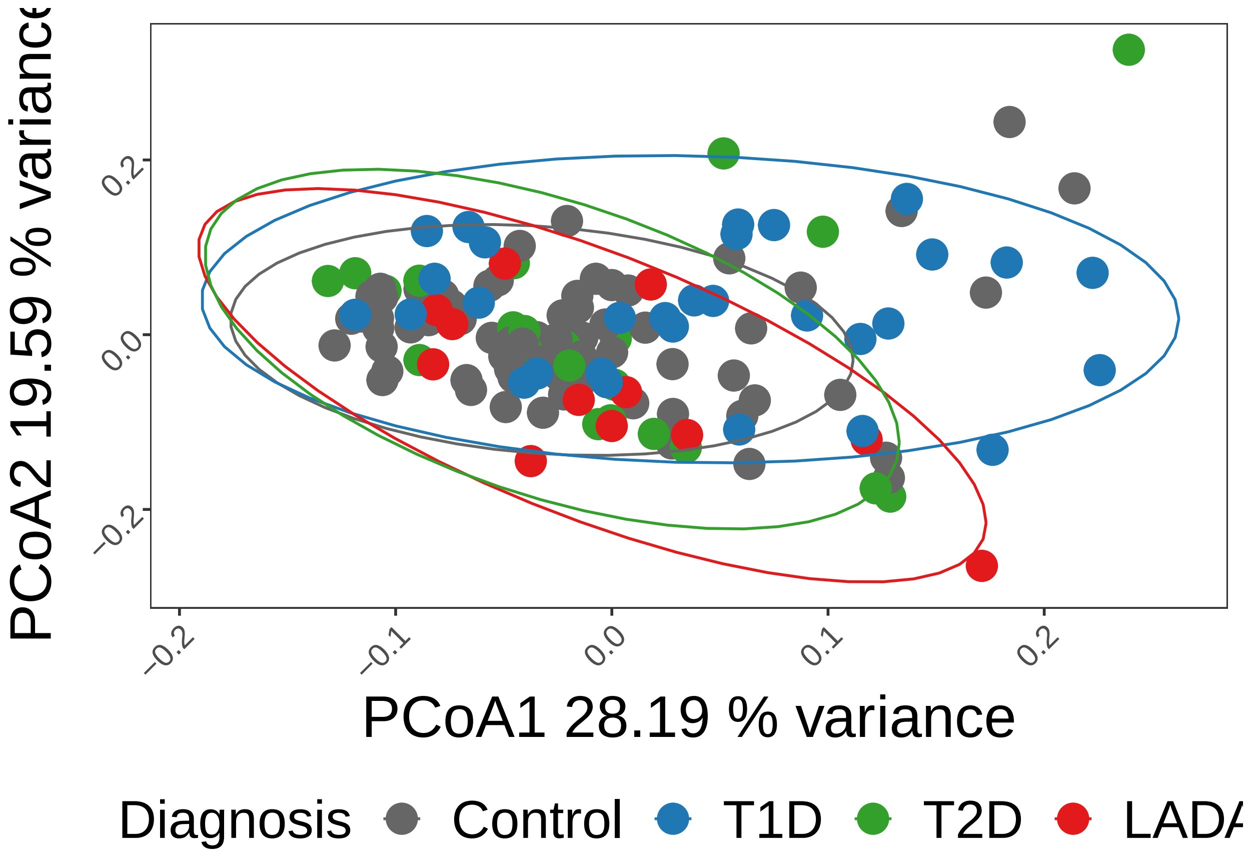

Create figure 1

Investigating grouping diagnosis. Which group does LADA resemble the most and which are different form each other.

```
#Have the plots stored in list
lay <- rbind(c(1,2,3,4),
             c(1,2,3,4),
             c(5,5,5,5),
             c(5,5,5,5))


pdf(paste("MicroLADA_Figure1_Func.pdf", sep=""), width=15, height=15)
grid.arrange(Fig1List$Alpharichness,
             Fig1List$AlphaPielou,
             Fig1List$AlphaShannon,
             Fig1List$DissiBrayHel,
             Fig1List$PCoAall, layout_matrix = lay)
dev.off()
```

```
## pdf
##   2
```

```r
pdf(paste("MicroLADA_Figure1RemMet_Func.pdf", sep=""), width=15, height=15)
grid.arrange(Fig1ListRemMet$Alpharichness,
             Fig1ListRemMet$AlphaPielou,
             Fig1ListRemMet$AlphaShannon,
             Fig1ListRemMet$DissiBrayHel,
             Fig1ListRemMet$PCoAall, layout_matrix = lay)
dev.off()
```

```
## pdf
##   2
```

PERMANOVA

Permutational Multivariate ANOVA based on dissimilarities (Bray-Curtis, Hellinger transformed data) using vegan::adonis
Added to figure 1 using inkscape.

```r
rm(list=setdiff(ls(), c("Metadata", "MetadataMed", "Feature", "TaxonomyDA", "Taxonomy")))

#Hellinger transformation
Taxonomy2 <- data.frame(t(decostand(t(Taxonomy), method="hellinger")))
#Maks TSS
Taxonomy2<-sweep(Taxonomy2, 2, colSums(Taxonomy2), FUN="/")

#Dissimilarity
distmatrix <- vegdist(t(Taxonomy2), method="bray")

#adonis can handle both continous and factor predictors
set.seed(1)
adonisObject<-adonis2(distmatrix ~ Diagnosis, Metadata, by="terms",
                      perm=999) #, perm=999 can increase to get exact p-values
adonisObject #If significant then difference between groups
```

```
## Permutation test for adonis under reduced model
## Terms added sequentially (first to last)
## Permutation: free
## Number of permutations: 999
##
## adonis2(formula = distmatrix ~ Diagnosis, data = Metadata, permutations = 999, by = "terms")
##            Df SumOfSqs      R2      F Pr(>F)
## Diagnosis   3  0.01249 0.03699 2.8934  0.001 ***
## Residual  226  0.32528 0.96301
## Total     229  0.33777 1.00000
## ---
## Signif. codes:  0 '***' 0.001 '**' 0.01 '*' 0.05 '.' 0.1 ' ' 1
```

```r
#With Metformin
set.seed(1)
adonisObject<-adonis2(distmatrix ~ Diagnosis + Metformin, Metadata, by="terms",
                      perm=999) #, perm=999 can increase to get exact p-values
adonisObject #If significant then difference between groups
```

```
## Permutation test for adonis under reduced model
## Terms added sequentially (first to last)
## Permutation: free
## Number of permutations: 999
##
## adonis2(formula = distmatrix ~ Diagnosis + Metformin, data = Metadata, permutations = 999, by = "ter
##            Df SumOfSqs      R2      F Pr(>F)
## Diagnosis   3  0.01249 0.03699 2.8965  0.001 ***
## Metformin   1  0.00178 0.00528 1.2404  0.275
## Residual  225  0.32350 0.95773
## Total     229  0.33777 1.00000
## ---
## Signif. codes:  0 '***' 0.001 '**' 0.01 '*' 0.05 '.' 0.1 ' ' 1
```

```
set.seed(1)
adonisObject<-adonis2(distmatrix ~ Diagnosis * Metformin, Metadata, by="terms",
                      perm=999) #, perm=999 can increase to get exact p-values
adonisObject #If significant then difference between groups
```

```
## Permutation test for adonis under reduced model
## Terms added sequentially (first to last)
## Permutation: free
## Number of permutations: 999
##
## adonis2(formula = distmatrix ~ Diagnosis * Metformin, data = Metadata, permutations = 999, by = "ter
##                      Df SumOfSqs      R2      F Pr(>F)
## Diagnosis             3  0.01249 0.03699 2.9005  0.001 ***
## Metformin             1  0.00178 0.00528 1.2422  0.275
## Diagnosis:Metformin   1  0.00189 0.00560 1.3172  0.235
## Residual            224  0.32160 0.95213
## Total               229  0.33777 1.00000
## ---
## Signif. codes:  0 '***' 0.001 '**' 0.01 '*' 0.05 '.' 0.1 ' ' 1
```

```
#With Metformin first in the model formula
set.seed(1)
adonisObject<-adonis2(distmatrix ~ Metformin + Diagnosis, Metadata, by="terms",
                      perm=999) #, perm=999 can increase to get exact p-values
adonisObject #If significant then difference between groups
```

```
## Permutation test for adonis under reduced model
## Terms added sequentially (first to last)
## Permutation: free
## Number of permutations: 999
##
## adonis2(formula = distmatrix ~ Metformin + Diagnosis, data = Metadata, permutations = 999, by = "ter
##            Df SumOfSqs      R2      F Pr(>F)
## Metformin   1  0.00551 0.01632 3.8343  0.004 **
## Diagnosis   3  0.00876 0.02595 2.0318  0.009 **
## Residual  225  0.32350 0.95773
## Total     229  0.33777 1.00000
## ---
## Signif. codes:  0 '***' 0.001 '**' 0.01 '*' 0.05 '.' 0.1 ' ' 1
```

```r
#Actual test reported in figure 1
#With by = margin does not matter the order
set.seed(1)
adonisObject<-adonis2(distmatrix ~ Metformin + Diagnosis, Metadata, by="margin",
                      perm=999) #, perm=999 can increase to get exact p-values
adonisObject #If significant then difference between groups
```

```
## Permutation test for adonis under reduced model
## Marginal effects of terms
## Permutation: free
## Number of permutations: 999
##
## adonis2(formula = distmatrix ~ Metformin + Diagnosis, data = Metadata, permutations = 999, by = "marg
##            Df SumOfSqs      R2      F Pr(>F)
## Metformin   1  0.00178 0.00528 1.2404  0.275
## Diagnosis   3  0.00876 0.02595 2.0318  0.009 **
## Residual  225  0.32350 0.95773
## Total     229  0.33777 1.00000
## ---
## Signif. codes:  0 '***' 0.001 '**' 0.01 '*' 0.05 '.' 0.1 ' ' 1
```

```r
#Include BMI
set.seed(1)
adonisObject<-adonis2(distmatrix ~ Metformin + Diagnosis + BMI, Metadata, by="margin",
                      perm=999) #, perm=999 can increase to get exact p-values
adonisObject #If significant then difference between groups
```

```
## Permutation test for adonis under reduced model
## Marginal effects of terms
## Permutation: free
## Number of permutations: 999
##
## adonis2(formula = distmatrix ~ Metformin + Diagnosis + BMI, data = Metadata, permutations = 999, by =
##            Df SumOfSqs      R2      F Pr(>F)
## Metformin   1  0.00178 0.00526 1.2334  0.275
## Diagnosis   3  0.00868 0.02568 2.0092  0.011 *
## BMI         1  0.00110 0.00325 0.7627  0.584
## Residual  224  0.32240 0.95448
## Total     229  0.33777 1.00000
## ---
## Signif. codes:  0 '***' 0.001 '**' 0.01 '*' 0.05 '.' 0.1 ' ' 1
```

```r
set.seed(1)
adonisObject<-adonis2(distmatrix ~ Metformin + Diagnosis + BMIord, Metadata, by="margin",
                      perm=999) #, perm=999 can increase to get exact p-values
adonisObject #If significant then difference between groups
```

```
## Permutation test for adonis under reduced model
## Marginal effects of terms
## Permutation: free
## Number of permutations: 999
##
```

```
## adonis2(formula = distmatrix ~ Metformin + Diagnosis + BMIord, data = Metadata, permutations = 999, b
##            Df SumOfSqs      R2      F Pr(>F)
## Metformin   1  0.00183 0.00541 1.2636  0.265
## Diagnosis   3  0.00850 0.02517 1.9598  0.016 *
## BMIord      5  0.00537 0.01589 0.7425  0.810
## Residual  220  0.31813 0.94184
## Total     229  0.33777 1.00000
## ---
## Signif. codes:  0 '***' 0.001 '**' 0.01 '*' 0.05 '.' 0.1 ' ' 1
```

```
set.seed(1)
adonisObject<-adonis2(distmatrix ~ Metformin + Diagnosis + BMIclass, Metadata, by="margin",
                      perm=999) #, perm=999 can increase to get exact p-values
adonisObject #If significant then difference between groups
```

```
## Permutation test for adonis under reduced model
## Marginal effects of terms
## Permutation: free
## Number of permutations: 999
##
## adonis2(formula = distmatrix ~ Metformin + Diagnosis + BMIclass, data = Metadata, permutations = 999
##            Df SumOfSqs      R2      F Pr(>F)
## Metformin   1  0.00178 0.00526 1.2321  0.284
## Diagnosis   3  0.00880 0.02604 2.0321  0.010 **
## BMIclass    2  0.00175 0.00518 0.6069  0.863
## Residual  223  0.32174 0.95255
## Total     229  0.33777 1.00000
## ---
## Signif. codes:  0 '***' 0.001 '**' 0.01 '*' 0.05 '.' 0.1 ' ' 1
```

```
#set.seed(1)
#adonisObject<-adonis2(distmatrix ~ Metformin + Diagnosis + BMIordclass, Metadata, by="margin",
#                      perm=999) #, perm=999 can increase to get exact p-values
#adonisObject #If significant then difference between groups same ass BMIclass

set.seed(1)
adonisObject<-adonis2(distmatrix ~ Metformin + Diagnosis + BMIq, Metadata, by="margin",
                      perm=999) #, perm=999 can increase to get exact p-values
adonisObject #If significant then difference between groups
```

```
## Permutation test for adonis under reduced model
## Marginal effects of terms
## Permutation: free
## Number of permutations: 999
##
## adonis2(formula = distmatrix ~ Metformin + Diagnosis + BMIq, data = Metadata, permutations = 999, by
##            Df SumOfSqs      R2      F Pr(>F)
## Metformin   1  0.00177 0.00525 1.2317  0.279
## Diagnosis   3  0.00868 0.02571 2.0090  0.011 *
## BMIq        1  0.00075 0.00223 0.5218  0.802
## Residual  224  0.32274 0.95551
## Total     229  0.33777 1.00000
## ---
## Signif. codes:  0 '***' 0.001 '**' 0.01 '*' 0.05 '.' 0.1 ' ' 1
```

```r
set.seed(1)
adonisObject<-adonis2(distmatrix ~ Metformin * Diagnosis * BMIq, Metadata, by="margin",
                      perm=999) #, perm=999 can increase to get exact p-values
adonisObject #If significant then difference between groups
```

```
## Permutation test for adonis under reduced model
## Marginal effects of terms
## Permutation: free
## Number of permutations: 999
##
## adonis2(formula = distmatrix ~ Metformin * Diagnosis * BMIq, data = Metadata, permutations = 999, by
##                              Df SumOfSqs      R2     F Pr(>F)
## Metformin:Diagnosis:BMIq   1  0.00122 0.00360 0.846  0.539
## Residual                  218  0.31357 0.92834
## Total                     229  0.33777 1.00000
```

```r
set.seed(1)
adonisObject<-adonis2(distmatrix ~ Diagnosis + BMIq, Metadata, by="margin",
                      perm=999) #, perm=999 can increase to get exact p-values
adonisObject #If significant then difference between groups
```

```
## Permutation test for adonis under reduced model
## Marginal effects of terms
## Permutation: free
## Number of permutations: 999
##
## adonis2(formula = distmatrix ~ Diagnosis + BMIq, data = Metadata, permutations = 999, by = "margin")
##            Df SumOfSqs      R2      F Pr(>F)
## Diagnosis   3  0.01185 0.03510 2.7398  0.001 ***
## BMIq        1  0.00076 0.00225 0.5274  0.799
## Residual  225  0.32452 0.96076
## Total     229  0.33777 1.00000
## ---
## Signif. codes:  0 '***' 0.001 '**' 0.01 '*' 0.05 '.' 0.1 ' ' 1
```

```r
## Evaluating the model assumptions
TestModel <- with(Metadata, betadisper(distmatrix, Diagnosis)) #Can not run
  #betadisper with multiple independant variables
#TestModel
#plot(TestModel)
plot(TestModel, label=FALSE)
```

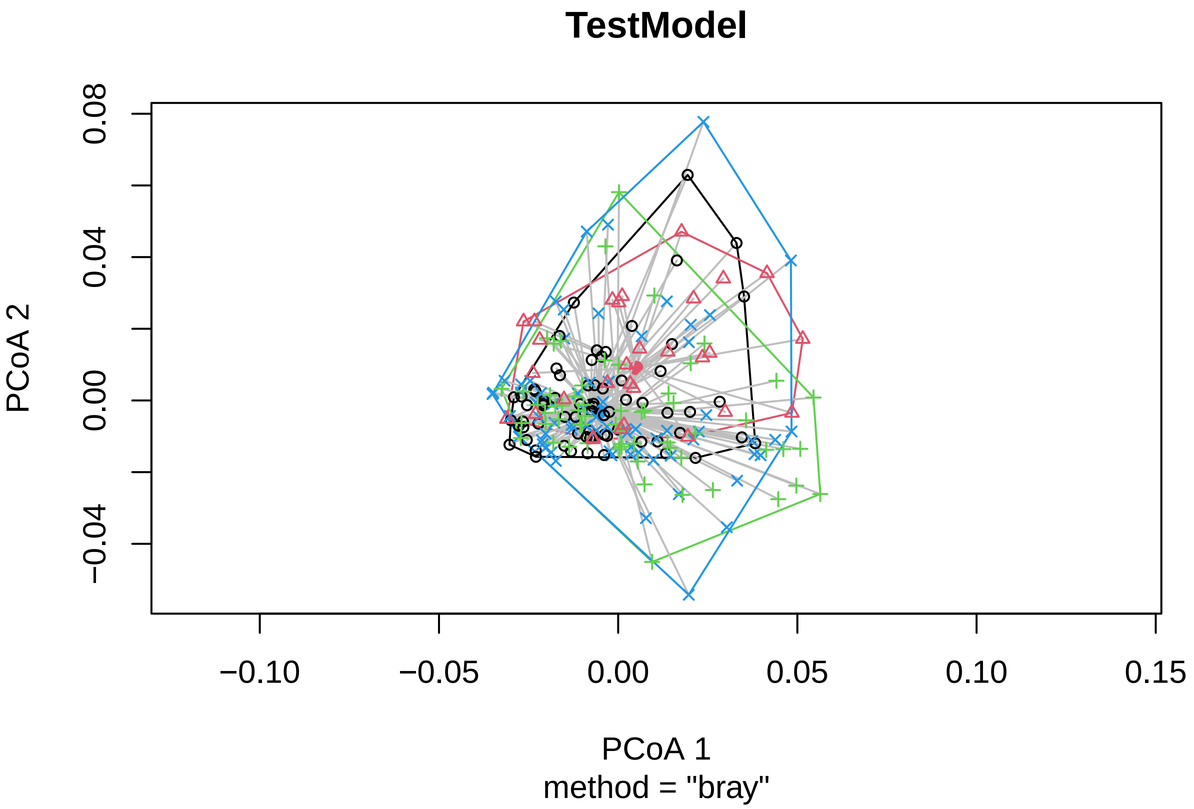


```
boxplot(TestModel)
```

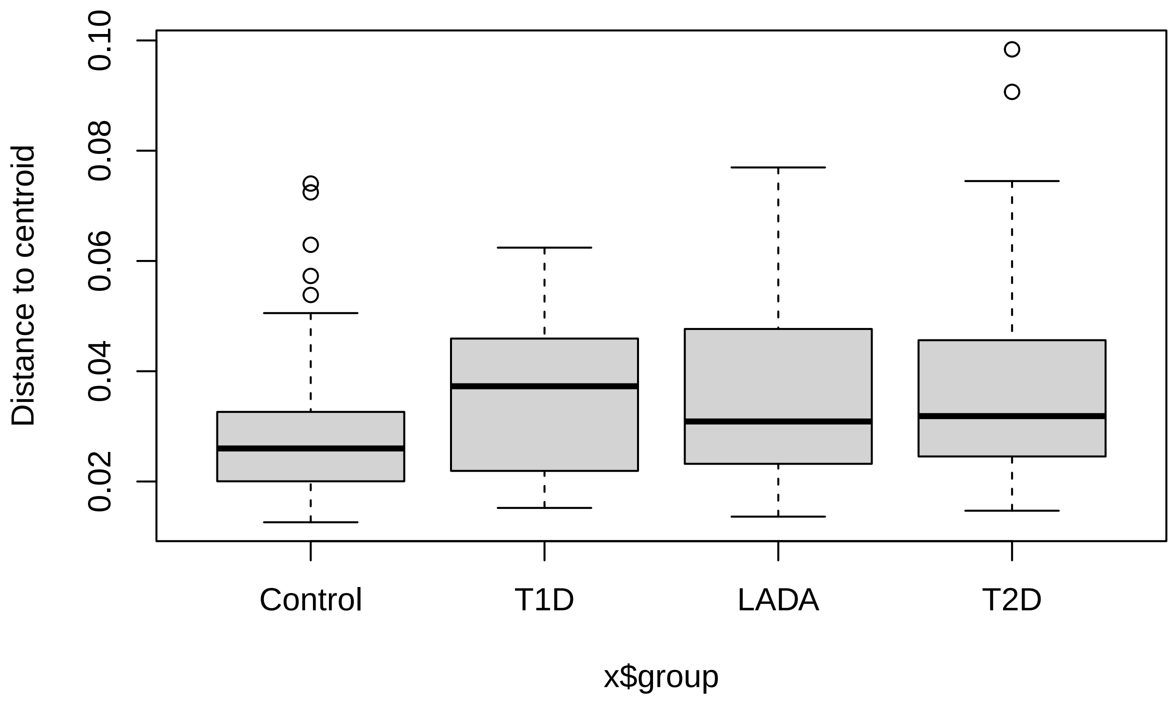


```
anova(TestModel) #p>0.05 -> Assumption met
```

```
## Analysis of Variance Table
##
## Response: Distances
##            Df   Sum Sq   Mean Sq F value   Pr(>F)
## Groups      3 0.002895 0.0009650  4.0445 0.007934 **
## Residuals 226 0.053923 0.0002386
## ---
## Signif. codes:  0 '***' 0.001 '**' 0.01 '*' 0.05 '.' 0.1 ' ' 1
```

```
#permutest(TestModel)

table(Metadata$Metformin, Metadata$Diagnosis)
```

```
##
##     Control T1D LADA T2D
##   0      70  30   12  23
##   1       0   0   48  47
```

PERMANOVA remove metformin

```r
rm(list=setdiff(ls(), c("Metadata", "MetadataMed", "Feature", "TaxonomyDA", "Taxonomy")))

##Removing treated with metformin
#Metadata$Metformin <- ifelse(is.na(Metadata$Metformin), "Unknown", Metadata$Metformin)
Metadata2<-filter(Metadata, !Metformin == 1)
##Applying subsetting to OTU tables
Taxonomy2<-dplyr::select(Taxonomy, one_of(as.character(Metadata2$MicrobiomeID)))
##Remove orgs that are not present after subsetting.
Taxonomy2 <- Taxonomy2[rowSums(Taxonomy2)>0,]


#Hellinger transformation
Taxonomy2 <- data.frame(t(decostand(t(Taxonomy2), method="hellinger")))
#Maks TSS
Taxonomy2<-sweep(Taxonomy2, 2, colSums(Taxonomy2), FUN="/")

#Dissimilarity
distmatrix <- vegdist(t(Taxonomy2), method="bray")

set.seed(1)
adonisObject<-adonis2(distmatrix ~ Diagnosis, Metadata2, by="margin",
                      perm=999) #, perm=999 can increase to get exact p-values
adonisObject #If significant then difference between groups
```

```
## Permutation test for adonis under NA model
## Marginal effects of terms
## Permutation: free
## Number of permutations: 999
##
## adonis2(formula = distmatrix ~ Diagnosis, data = Metadata2, permutations = 999, by = "margin")
##            Df SumOfSqs      R2      F Pr(>F)
## Diagnosis   3 0.009086 0.05015 2.3054  0.003 **
## Residual  131 0.172095 0.94985
## Total     134 0.181181 1.00000
## ---
## Signif. codes:  0 '***' 0.001 '**' 0.01 '*' 0.05 '.' 0.1 ' ' 1
```

```r
set.seed(1)
adonisObject<-adonis2(distmatrix ~ Diagnosis + BMIq, Metadata2, by="margin",
                      perm=999) #, perm=999 can increase to get exact p-values
adonisObject #If significant then difference between groups
```

```
## Permutation test for adonis under reduced model
## Marginal effects of terms
## Permutation: free
## Number of permutations: 999
##
## adonis2(formula = distmatrix ~ Diagnosis + BMIq, data = Metadata2, permutations = 999, by = "margin"
##            Df SumOfSqs      R2      F Pr(>F)
## Diagnosis   3 0.009181 0.05067 2.3198  0.002 **
## BMIq        1 0.000597 0.00330 0.4528  0.843
## Residual  130 0.171498 0.94656
```

```
## Total      134 0.181181 1.00000
## ---
## Signif. codes:  0 '***' 0.001 '**' 0.01 '*' 0.05 '.' 0.1 ' ' 1
```

```
## Evaluating the model assumptions
TestModel <- with(Metadata2, betadisper(distmatrix, Diagnosis)) #Can not run
  #betadisper with multiple independant variables
#TestModel
#plot(TestModel)
plot(TestModel, label=FALSE)
```

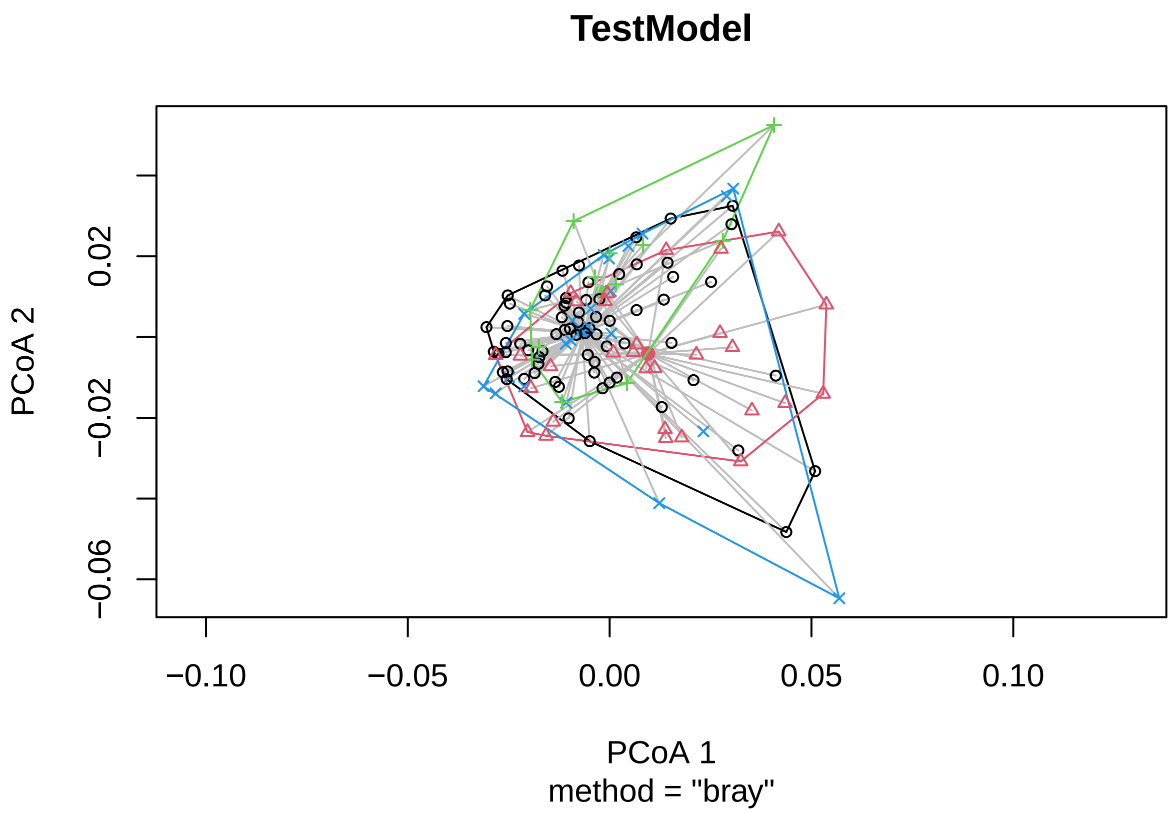


```
boxplot(TestModel)
```

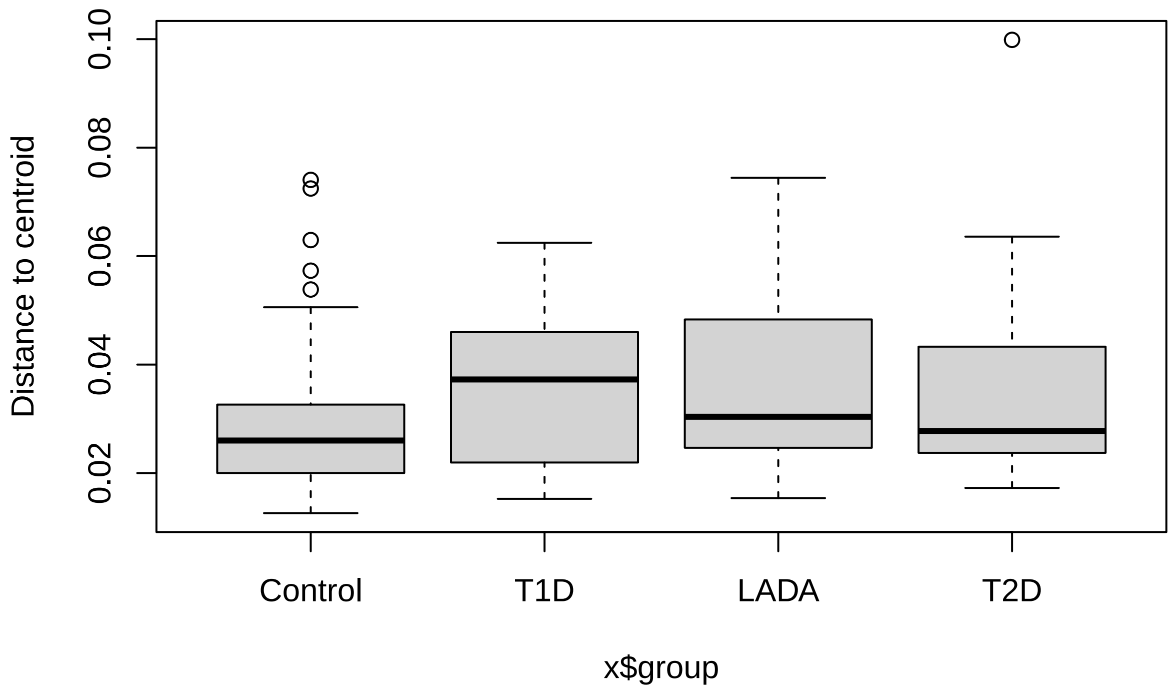


```
anova(TestModel) #p>0.05 -> Assumption met
```

```
## Analysis of Variance Table
##
## Response: Distances
##            Df   Sum Sq    Mean Sq F value  Pr(>F)
## Groups      3 0.001726 0.00057532  2.5539 0.05822 .
## Residuals 131 0.029510 0.00022527
## ---
## Signif. codes:  0 '***' 0.001 '**' 0.01 '*' 0.05 '.' 0.1 ' ' 1
```

```
#permutest(TestModel)

table(Metadata2$Metformin, Metadata2$Diagnosis)
```

```
##
##     Control T1D LADA T2D
##   0      70  30   12  23
```

PERMANOVA MED subset

```
rm(list=setdiff(ls(), c("Metadata", "MetadataMed", "Feature", "TaxonomyDA", "Taxonomy")))

##Metadatamed
##Applying subsetting to OTU tables
Taxonomy2<-dplyr::select(Taxonomy, one_of(as.character(MetadataMed$MicrobiomeID)))

#Hellinger transformation
Taxonomy2 <- data.frame(t(decostand(t(Taxonomy2), method="hellinger")))
#Maks TSS
Taxonomy2<-sweep(Taxonomy2, 2, colSums(Taxonomy2), FUN="/")

#Dissimilarity
distmatrix <- vegdist(t(Taxonomy2), method="bray")

#With by = margin does not matter the order
set.seed(1)
adonisObject<-adonis2(distmatrix ~ Diagnosis + BMIq, MetadataMed, by="margin",
                      perm=999) #, perm=999 can increase to get exact p-values
adonisObject #If significant then difference between groups
```

```
## Permutation test for adonis under reduced model
## Marginal effects of terms
## Permutation: free
## Number of permutations: 999
##
## adonis2(formula = distmatrix ~ Diagnosis + BMIq, data = MetadataMed, permutations = 999, by = "margi
##            Df SumOfSqs      R2      F Pr(>F)
## Diagnosis   2 0.008625 0.03274 2.6514  0.006 **
## BMIq        1 0.001292 0.00490 0.7942  0.583
## Residual  156 0.253748 0.96311
## Total     159 0.263468 1.00000
## ---
## Signif. codes:  0 '***' 0.001 '**' 0.01 '*' 0.05 '.' 0.1 ' ' 1
```

```
set.seed(1)
adonisObject<-adonis2(distmatrix ~ Metformin + Diagnosis, MetadataMed, by="margin",
                      perm=999) #, perm=999 can increase to get exact p-values
adonisObject #If significant then difference between groups
```

```
## Permutation test for adonis under reduced model
## Marginal effects of terms
## Permutation: free
## Number of permutations: 999
##
## adonis2(formula = distmatrix ~ Metformin + Diagnosis, data = MetadataMed, permutations = 999, by = "
##            Df SumOfSqs      R2      F Pr(>F)
## Metformin   1 0.001783 0.00677 1.0986   0.34
## Diagnosis   2 0.006084 0.02309 1.8737   0.04 *
## Residual  156 0.253256 0.96124
## Total     159 0.263468 1.00000
## ---
## Signif. codes:  0 '***' 0.001 '**' 0.01 '*' 0.05 '.' 0.1 ' ' 1
```

```
set.seed(1)
adonisObject<-adonis2(distmatrix ~ Diagnosis + Metformin + BMIq , MetadataMed, by="margin",
                      perm=999) #, perm=999 can increase to get exact p-values
adonisObject #If significant then difference between groups
```

```
## Permutation test for adonis under reduced model
## Marginal effects of terms
## Permutation: free
## Number of permutations: 999
##
## adonis2(formula = distmatrix ~ Diagnosis + Metformin + BMIq, data = MetadataMed, permutations = 999,
##            Df SumOfSqs      R2      F Pr(>F)
## Diagnosis   2 0.006481 0.02460 1.9937  0.029 *
## Metformin   1 0.001814 0.00688 1.1158  0.329
## BMIq        1 0.001322 0.00502 0.8133  0.566
## Residual  155 0.251934 0.95622
## Total     159 0.263468 1.00000
## ---
## Signif. codes:  0 '***' 0.001 '**' 0.01 '*' 0.05 '.' 0.1 ' ' 1
```

```
set.seed(1)
adonisObject<-adonis2(distmatrix ~ Diagnosis + Metformin + BMIq + med_insulin + med_statins + med_protor
                      perm=999) #, perm=999 can increase to get exact p-values
adonisObject #If significant then difference between groups
```

```
## Permutation test for adonis under reduced model
## Marginal effects of terms
## Permutation: free
## Number of permutations: 999
##
## adonis2(formula = distmatrix ~ Diagnosis + Metformin + BMIq + med_insulin + med_statins + med_protonp
##                           Df SumOfSqs      R2      F Pr(>F)
## Diagnosis                  2 0.006495 0.02465 1.9936  0.027 *
## Metformin                  1 0.001670 0.00634 1.0249  0.386
## BMIq                       1 0.001194 0.00453 0.7329  0.620
## med_insulin                1 0.001048 0.00398 0.6433  0.687
## med_statins                1 0.001730 0.00656 1.0617  0.359
## med_protonpump_inhibitor   1 0.001476 0.00560 0.9058  0.466
## Residual                 152 0.247620 0.93985
## Total                    159 0.263468 1.00000
## ---
## Signif. codes:  0 '***' 0.001 '**' 0.01 '*' 0.05 '.' 0.1 ' ' 1
```

```
## Evaluating the model assumptions
TestModel <- with(MetadataMed, betadisper(distmatrix, Diagnosis)) #Can not run
  #betadisper with multiple independant variables
#TestModel
#plot(TestModel)
plot(TestModel, label=FALSE)
```

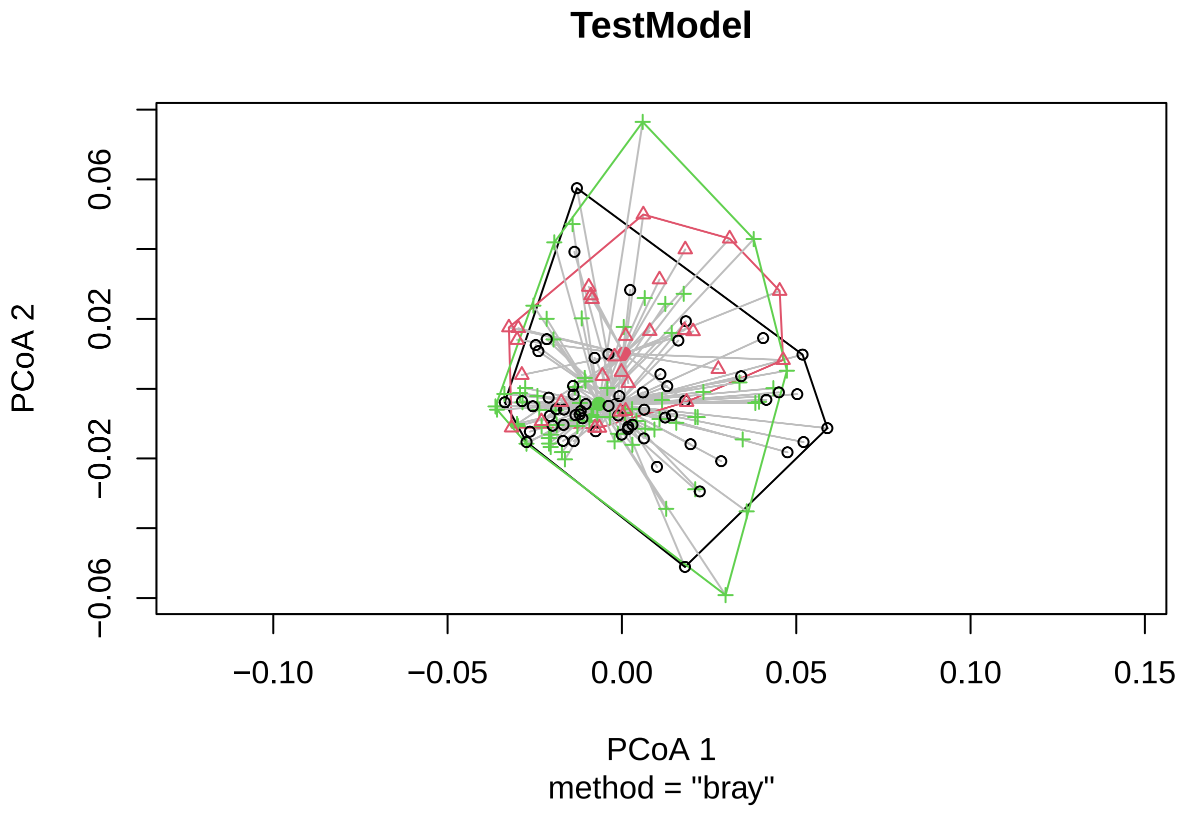


```
boxplot(TestModel)
```

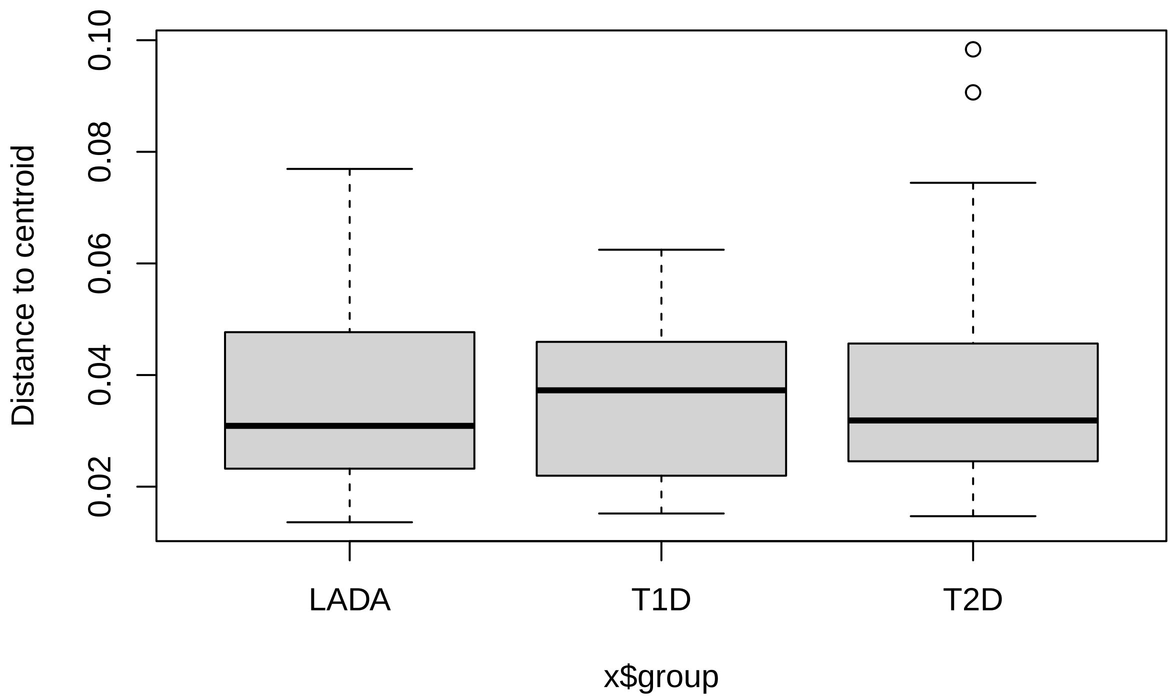


```
anova(TestModel) #p>0.05 -> Assumption met
```

```
## Analysis of Variance Table
##
## Response: Distances
##            Df   Sum Sq    Mean Sq F value Pr(>F)
## Groups      2 0.000058 2.8772e-05  0.1066  0.899
## Residuals 157 0.042392 2.7001e-04
```

```
#permutest(TestModel)
```

Pairwise PERMANOVA

Pairwise adonis, found this function from Pedro Martinez Arbizu on researchgate (https://www.researchgate.net/post/How_can_I_do_PerMANOVA_pairwise_contrasts_in_R) He also made implementation of adonis2 https://github.com/pmartinezarbizu/pairwiseAdonis

```
rm(list=setdiff(ls(), c("Metadata", "MetadataMed", "Feature", "TaxonomyDA", "Taxonomy")))

##Initial normalization/transformation the function runs method from adonis passed from
##vegdist
#Hellinger transformation
Taxonomy2 <- data.frame(t(decostand(t(Taxonomy), method="hellinger")))
#Maks TSS
```

```r
Taxonomy2<-sweep(Taxonomy2, 2, colSums(Taxonomy2), FUN="/")

##Testing function
#x <- t(Taxonomy2)
#factors <- as.character(Metadata$Diagnosis)
#sim.method <- 'bray'
#p.adjust.m <- 'bonferroni'
#elem<-1

pairwise.adonis <- function(x, factors, sim.method = 'bray', p.adjust.m ='bonferroni')
#x = the community table
#factors = a column or vector with all factors to be tested pairwise
#sim.method = similarity function, one of the functions available in vegdist(); default is
#'bray' for bray-curtis.
#p.adjust.m = the p.value correction method, one of the methods supported by p.adjust();
#default is 'bonferroni'
{
#library(vegan)
co = combn(unique(factors),2)
pairs = c()
F.Model =c()
R2 = c()
p.value = c()
for(elem in 1:ncol(co)){
ad = adonis(x[factors %in% c(co[1,elem],co[2,elem]),] ~
              factors[factors %in% c(co[1,elem],co[2,elem])] ,
            method =sim.method); #Extracting a matrix only containing samples of a pair
pairs = c(pairs,paste(co[1,elem],'vs',co[2,elem]));
F.Model =c(F.Model,ad$aov.tab[1,4]);
R2 = c(R2,ad$aov.tab[1,5]);
p.value = c(p.value,ad$aov.tab[1,6])
}
p.adjusted = p.adjust(p.value,method=p.adjust.m) #adjusting after all comparisons
pairw.res = data.frame(pairs,F.Model,R2,p.value,p.adjusted)
return(pairw.res)
}

pairwise.adonis(t(Taxonomy2), as.character(Metadata$Diagnosis))
```

```
##              pairs   F.Model          R2 p.value p.adjusted
## 1  T2D vs Control 1.7605106 0.012596624   0.090      0.540
## 2     T2D vs LADA 0.9577613 0.007426938   0.408      1.000
## 3      T2D vs T1D 3.3350811 0.032911417   0.007      0.042
## 4 Control vs LADA 3.8449904 0.029162962   0.004      0.024
## 5  Control vs T1D 4.7357403 0.046096327   0.002      0.012
## 6     LADA vs T1D 4.2447073 0.046015727   0.001      0.006
```

```r
pairw.res<-pairwise.adonis(t(Taxonomy2), as.character(Metadata$Diagnosis))


##Implementation of adonis2 can not get to work
##My implementation of adonis2 for my data when running with diagnosis
```

```r
##Testing function
x <- t(Taxonomy2)
factors <- as.character(Metadata$Diagnosis)
factors2 <- as.character(Metadata$Metformin)
sim.method <- 'bray'
p.adjust.m <- 'bonferroni'
#elem<-1
#elem<-5

#pairwise.adonis.multiplevar <- function(x, factors, factors2, sim.method = 'bray',
#p.adjust.m ='bonferroni')
#x = the community table
#factors = a column or vector with all factors to be tested pairwise
#sim.method = similarity function, one of the functions available in vegdist(); default is
#'bray' for bray-curtis
#p.adjust.m = the p.value correction method, one of the methods supported by p.adjust();
#default is 'bonferroni'
#{
#library(vegan)
co = combn(unique(factors),2)
pairs = c()
F.Model =c()
R2 = c()
p.value = c()
F.Model.2 =c()
R2.2 = c()
p.value.2 = c()
#for(elem in 1:ncol(co)){
for(elem in c(1:4,6)){
ad = adonis2(x[factors %in% c(co[1,elem],co[2,elem]),] ~
               factors[factors %in% c(co[1,elem],co[2,elem])] +
               factors2[factors %in% c(co[1,elem],co[2,elem])],
             method =sim.method, by="margin"); #Extracting a matrix only containing
                                               #samples of a pair
pairs = c(pairs,paste(co[1,elem],'vs',co[2,elem]));
F.Model =c(F.Model,ad$F[1]);
R2 = c(R2,ad$R2[1]);
p.value = c(p.value,ad$`Pr(>F)`[1])
F.Model.2 =c(F.Model.2,ad$F[2])
R2.2 = c(R2.2,ad$R2[2])
p.value.2 = c(p.value.2,ad$`Pr(>F)`[2])
}
p.adjusted = p.adjust(p.value,method=p.adjust.m)
p.adjusted.2 = p.adjust(p.value.2,method=p.adjust.m)
pairw.res.2 = data.frame(pairs, F.Model, R2, p.value, p.adjusted,
                         F.Model.2, R2.2, p.value.2, p.adjusted.2)
#return(pairw.res)
#}

#pairwise.adonis.multiplevar(t(Taxonomy2), factors = as.character(Metadata$Diagnosis),
#                             factors2 = as.character(Metadata$Metformin),
#                             sim.method = 'bray', p.adjust.m ='bonferroni')
```

```
pairw.res.2
```

```
##              pairs   F.Model          R2 p.value p.adjusted F.Model.2
## 1  T2D vs Control 0.5187954 0.003687712   0.820      1.000 1.9100461
## 2     T2D vs LADA 0.9121886 0.007069354   0.437      1.000 1.0759017
## 3      T2D vs T1D 1.7753031 0.017414704   0.101      0.505 1.5876860
## 4 Control vs LADA 1.8837435 0.014304937   0.078      0.390 0.8445350
## 5     LADA vs T1D 3.0276423 0.032935825   0.014      0.070 0.6954837
##          R2.2 p.value.2 p.adjusted.2
## 1 0.013577031     0.085        0.425
## 2 0.008338111     0.366        1.000
## 3 0.015574288     0.141        0.705
## 4 0.006413304     0.525        1.000
## 5 0.007565732     0.662        1.000
```

```
write.table(pairw.res, file="adonis_pairwisediagnosis_Func.txt",
            quote = F, row.names = F, sep="\t")
write.table(pairw.res.2, file="adonis2_pairwisediagnosiswvarmet_Func.txt",
            quote = F, row.names = F, sep="\t")
```

Pairwise PERMANOVA remove metformin

```
rm(list=setdiff(ls(), c("Metadata", "MetadataMed", "Feature", "TaxonomyDA", "Taxonomy")))

##Removing treated with metformin
#Metadata$Metformin <- ifelse(is.na(Metadata$Metformin), "Unknown", Metadata$Metformin)
Metadata2<-filter(Metadata, !Metformin == 1)
##Applying subsetting to OTU tables
Taxonomy2<-dplyr::select(Taxonomy, one_of(as.character(Metadata2$MicrobiomeID)))
##Remove orgs that are not present after subsetting.
Taxonomy2 <- Taxonomy2[rowSums(Taxonomy2)>0,]

##Initial normalization/transformation the function runs method from adonis passed from
##vegdist
#Hellinger transformation
Taxonomy2 <- data.frame(t(decostand(t(Taxonomy2), method="hellinger")))
#Maks TSS
Taxonomy2<-sweep(Taxonomy2, 2, colSums(Taxonomy2), FUN="/")

##Testing function
#x <- t(Taxonomy2)
#factors <- as.character(Metadata$Diagnosis)
#sim.method <- 'bray'
#p.adjust.m <- 'bonferroni'
#elem<-1

pairwise.adonis <- function(x, factors, sim.method = 'bray', p.adjust.m ='bonferroni')
#x = the community table
#factors = a column or vector with all factors to be tested pairwise
#sim.method = similarity function, one of the functions available in vegdist(); default is
#'bray' for bray-curtis.
```

```r
#p.adjust.m = the p.value correction method, one of the methods supported by p.adjust();
#default is 'bonferroni'
{
#library(vegan)
co = combn(unique(factors),2)
pairs = c()
F.Model =c()
R2 = c()
p.value = c()
for(elem in 1:ncol(co)){
ad = adonis(x[factors %in% c(co[1,elem],co[2,elem]),] ~
              factors[factors %in% c(co[1,elem],co[2,elem])] ,
            method =sim.method); #Extracting a matrix only containing samples of a pair
pairs = c(pairs,paste(co[1,elem],'vs',co[2,elem]));
F.Model =c(F.Model,ad$aov.tab[1,4]);
R2 = c(R2,ad$aov.tab[1,5]);
p.value = c(p.value,ad$aov.tab[1,6])
}
p.adjusted = p.adjust(p.value,method=p.adjust.m) #adjusting after all comparisons
pairw.res = data.frame(pairs,F.Model,R2,p.value,p.adjusted)
return(pairw.res)
}

pairwise.adonis(t(Taxonomy2), as.character(Metadata2$Diagnosis))
```

```
##              pairs   F.Model          R2 p.value p.adjusted
## 1  Control vs T2D 0.5888035 0.006428772   0.736      1.000
## 2 Control vs LADA 2.1326667 0.025966120   0.048      0.288
## 3  Control vs T1D 4.7357403 0.046096327   0.002      0.012
## 4     T2D vs LADA 1.0237678 0.030089784   0.399      1.000
## 5      T2D vs T1D 1.8191877 0.034441797   0.089      0.534
## 6     LADA vs T1D 2.9824999 0.069388702   0.013      0.078
```

```r
pairw.res<-pairwise.adonis(t(Taxonomy2), as.character(Metadata2$Diagnosis))


##Implementation of adonis2 can not get to work
##Made my implementation of adonis2 for my data when running with diagnosis and
  #in this case the other factor metformin so removed when removing samples with
  #patients treated with metformin


write.table(pairw.res, file="adonis_pairwisediagnosisremovedmetsamples.txt",
            quote = F, row.names = F, sep="\t")
```

Heatmap

```r
rm(list=setdiff(ls(), c("Metadata", "MetadataMed", "Feature", "TaxonomyDA", "Taxonomy")))


#Add a categorical indicator of sex in Metadata also used in rest of script
```

```r
Metadata$Sex<-as.factor(ifelse(grepl("1", Metadata$sex), "Male",
                                      ifelse(grepl("2", Metadata$sex), "Female",
                                             "hmmmmm")))

#The organisms clustering
OrgCluster<-"correlation"

#How many organisms to include in heatmap
Orgs<-25 #Write a number 20-50 seems appropiate for readability

HeatmapExplainers<-c("Sex", "Metformin", "Diagnosis")

#Make list of annotation colors
annotation_colorsNew = list(Diagnosis = c(Control = "#666666", T1D = "#1F78B4",
                                          LADA = "#E31A1C", T2D = "#33A02C"),
                            Metformin = c("0" = "white", "1" = "black"),
                            Sex = c(Male = "black", Female = "white"))


#Order genera, based on rowsums
TaxHeatmap <- Taxonomy[order(rowSums(Taxonomy), decreasing = T),]

#Impose a maximum number of plotted genera
TaxHeatmap <- TaxHeatmap[1:min(c(nrow(TaxHeatmap), Orgs)),]


##Then I standardized the orgs into zero mean and unit variance
TaxHeatmap <- data.frame(t(decostand(t(TaxHeatmap), method="standardize")))
#Can also use scale in pheatmap, but not exactly sure what scaling that is being performed


#Make dataframe with Metadata for heatmap annotation
colannodf <- data.frame(Metadata[, HeatmapExplainers], row.names = Metadata$MicrobiomeID)

#Calculate sample-distance matrix
#Note, that this is done on the full set, not just the shown. Makes sense eventhough not
#show in heatmap they can be in the clustering calculations, this also means samples can
#look more similar in the heatmap but not cluster as closely. Also calculates on the not
#log transformed data.
#filtering of the Counttable depending on rowSums.
Tax2 <- Taxonomy[rowSums(Taxonomy)>0,] #Removing all rows that only contains zeroes
#Tax2 <- Tax2[rowSums(Tax2)>(5*ncol(Tax2)),] #Removing all rows(Species)  that is below an
#average count of 5.
# replace 0 values with an estimate using simple multiplicative replacement
#Tax2 <- t(cmultRepl(t(Tax2), method="CZM", label=0))
#Maks TSS
Tax2<-sweep(Tax2, 2, colSums(Tax2), FUN="/")
#Calculate sample-distance matrix
#Note, that this is done on the full set, not just the shown. Makes sense eventhough not
#show in heatmap they can be in the clustering calculations, this also means samples can
#look more similar in the heatmap but not cluster as closely. Also calculates on the not
#log transformed data.
#distmatrix_Species <- vegdist(ilr(t(Tax2)), method="euclidean") #Previously
```

```r
distmatrix_Species <- vegdist(decostand(t(Tax2), method="hellinger"), method="bray")

#Draw the heatmap
pdf(paste("MicroLADA_Heatmap_Func", ".pdf", sep=""), width=15, height=5)
pheatmap(TaxHeatmap,
         #color = colorRampPalette(rev(brewer.pal(n = 7, name = "Blues")))(100),
         color = colorRampPalette(rev(brewer.pal(n = 7, name ="RdYlBu")))(100),
         margins=c(8,8),
         treeheight_row = 70,
         treeheight_col = 70,
         scale="none",
         clustering_distance_cols = distmatrix_Species,
         clustering_distance_rows = OrgCluster,
         annotation_col = colannodf,
         cutree_cols = 2,
         show_colnames = FALSE,
         #cellwidth=5,
         #cellheight=4,
         fontsize=10,
         annotation_colors = annotation_colorsNew[1:7],
         annotation_legend = TRUE)
dev.off()
```

```
## pdf
##   3
```

```r
#Does not output plot to knitr
```

CCA

```r
rm(list=setdiff(ls(), c("Metadata", "MetadataMed", "Feature", "TaxonomyDA", "Taxonomy", "Fig2List")))

#Create a list to hold the plot objects.
Fig2List <- list()

#Norm of Tax. Should be taken into account. The chi square distance that is the basis of
#cca makes raw counts appropriate.
#See https://sites.google.com/site/mb3gustame/reference/dissimilarity

#Filtering of the Counttable depending on rowSums. Prevents overplotting
Taxonomy2 <- Taxonomy[rowSums(Taxonomy)>(50*length(Taxonomy)),]
#Taxonomy2<-Taxonomy

##Total sum scaling (Use relative abundances)
Taxonomy2<-sweep(Taxonomy2, 2, colSums(Taxonomy2), FUN="/")


##Perform cca
ccaord <- cca(t(Taxonomy2) ~ Diagnosis + Condition(Metformin), Metadata)
ccaord
```

```
## Call: cca(formula = t(Taxonomy2) ~ Diagnosis + Condition(Metformin),
## data = Metadata)
##
##                 Inertia Proportion Rank
## Total          0.0256833  1.0000000
## Conditional    0.0003353  0.0130559    1
## Constrained    0.0006626  0.0257970    3
## Unconstrained 0.0246853  0.9611432   73
## Inertia is scaled Chi-square
##
## Eigenvalues for constrained axes:
##      CCA1      CCA2      CCA3
## 0.0004234 0.0001989 0.0000402
##
## Eigenvalues for unconstrained axes:
##      CA1      CA2      CA3      CA4      CA5      CA6      CA7      CA8
## 0.007451 0.005424 0.003383 0.003040 0.002349 0.000868 0.000473 0.000359
## (Showing 8 of 73 unconstrained eigenvalues)
```

```
#summary(ccaord)
plot(ccaord)
```

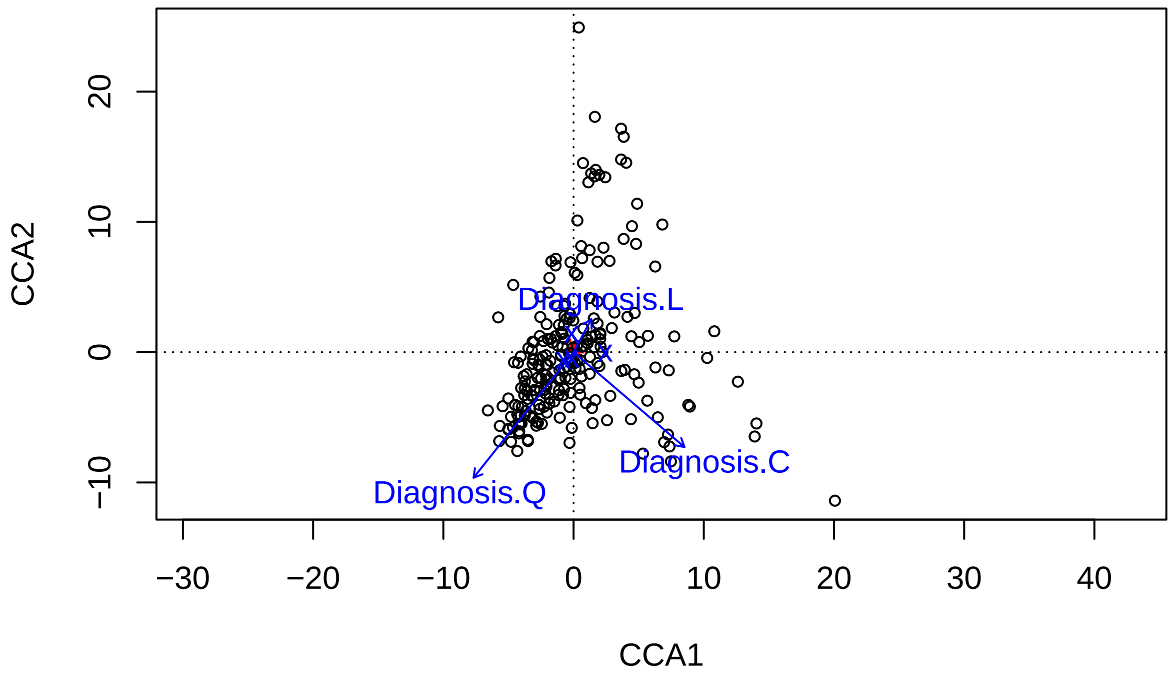


```
anova(ccaord) #Only Diagnosis
```

```
## Permutation test for cca under reduced model
```

```
## Permutation: free
## Number of permutations: 999
##
## Model: cca(formula = t(Taxonomy2) ~ Diagnosis + Condition(Metformin), data = Metadata)
##           Df ChiSquare     F Pr(>F)
## Model      3 0.0006626 2.013  0.011 *
## Residual 225 0.0246853
## ---
## Signif. codes:  0 '***' 0.001 '**' 0.01 '*' 0.05 '.' 0.1 ' ' 1
```

```
##Other model
#ccaord2 <- cca(t(Taxonomy2)~Diagnosis+Metformin, Metadata)
#ccaord2
#summary(ccaord2)
#plot(ccaord2)
#anova(ccaord2, by="margin", permutations=199)

##ggvegan implementation of visualization
#devtools::install_github("gavinsimpson/ggvegan")
#library(ggvegan)
#autoplot(ccaord)

#Custom modification refer to cca object description
#https://www.rdocumentation.org/packages/vegan/versions/2.4-2/topics/cca.object


##My own implementation of cca visualization
#Extract scores from cca object
scores <- vegan::scores(ccaord, display=c("sp", "wa", "cn", "bp"), choices=c(1,2,3))

#Extract site information and add to metadata
sites <- data.frame(scores$sites)
sites$MicrobiomeID <- rownames(sites)
Metadata2 <- merge(Metadata, sites, by="MicrobiomeID")

#Extract species information and add to feature information
species <- data.frame(scores$species)
species$Genus <- rownames(species)
Feature2 <- merge(Feature, species, by="Genus")


##Create column org grouping for colouring
Feature2$Level_1 <- ifelse(Feature2$Level_1=="Metabolism",
                           "Metabolism",
          ifelse(Feature2$Level_1=="Cellular Processes",
                 "Cellular Processes",
          ifelse(Feature2$Level_1=="Genetic Information Processing",
                 "Genetic Information Processing",
          ifelse(Feature2$Level_1=="Environmental Information Processing",
                 "Environmental Information Processing",
          ifelse(Feature2$Level_1=="Unclassified metabolism",
                 "Unclassified metabolism",
                 "Other")))))
#Order Level_1 for plotting
```

```
Feature2$Level_1 <- factor(Feature2$Level_1,
                           levels=c("Cellular Processes",
                                    "Environmental Information Processing",
                                    "Genetic Information Processing",
                                    "Metabolism",
                                    "Unclassified metabolism",
                                    "Other"))


#Extract information on inertia
inertia <- ccaord$CCA$tot.chi/ccaord$tot.chi
subheader <- paste("Inertia constrained by the explanatory variables",
                   round(inertia, digits=2))
##Extract information on eig
#eig1 <- (cca$CCA$eig[1]/(sum(cca$CCA$eig)+sum(cca$CA$eig)))*100
#eig_1 <- paste("CCA1", round(eig1, digits=2), "% of total variance")
#eig2 <- (cca$CCA$eig[2]/(sum(cca$CCA$eig)+sum(cca$CA$eig)))*100
#eig_2 <- paste("CCA2", round(eig2, digits=2), "% of total variance")

#Add a categorical indicator of group in Metadata
Metadata2$Metformin<-as.factor(ifelse(grepl("1", Metadata2$Metformin), "Yes",
                                      ifelse(grepl("0", Metadata2$Metformin), "No",
                                             "Unknown")))
#Order Treatment metformine for plotting
Metadata2$Metformin <- factor(Metadata2$Metformin, levels=c("Yes", "No", "Unknown"))

arrows<-data.frame(scores$centroids)
arrows$xstart<-0
arrows$ystart<-0
arrows$naming<-row.names(arrows)
arrows$naming<-str_remove(arrows$naming, "Diagnosis")

##Add text specific organisms to plot
#textgenus<-Feature2 %>% dplyr::select(one_of("Genus", "CCA1", "CCA2")) %>%
#  filter(Genus=="Faecalibacterium" |
#            Genus=="Actinomyces" |
#            Genus=="Butyricicoccus" |
#            Genus=="Lachnoclostridium")


p1<-ggplot() +
  geom_point(data=Metadata2[which(Metadata2$Diagnosis=='Control'), ],
             aes(x=CCA1, y=CCA2, shape=Metformin, group=Diagnosis),
             color="#666666", size=1.5, alpha=0.5) +
  geom_point(data=Metadata2[which(Metadata2$Diagnosis=='T1D'), ],
             aes(x=CCA1, y=CCA2, shape=Metformin, group=Diagnosis),
             color="#1F78B4", size=1.5, alpha=0.5) +
  geom_point(data=Metadata2[which(Metadata2$Diagnosis=='T2D'), ],
             aes(x=CCA1, y=CCA2, shape=Metformin, group=Diagnosis),
             color="#33A02C", size=1.5, alpha=0.5) +
  geom_point(data=Metadata2[which(Metadata2$Diagnosis=='LADA'), ],
             aes(x=CCA1, y=CCA2, shape=Metformin, group=Diagnosis),
             color="#E31A1C", size=1.5, alpha=0.5) +
```

```r
  scale_shape_manual(values=c(3, 16)) +
  geom_point(data=Feature2[which(Feature2$Level_1=="Cellular Processes"), ],
             aes(x=CCA1, y=CCA2, group=Genus), color="#A6CEE3", shape=18, size=0.7) +
  geom_point(data=Feature2[which(Feature2$Level_1=="Genetic Information Processing"), ],
             aes(x=CCA1, y=CCA2, group=Genus), color="#FB9A99", shape=18, size=0.7) +
  geom_point(data=Feature2[which(Feature2$Level_1=="Metabolism"), ],
             aes(x=CCA1, y=CCA2, group=Genus), color="#FDBF6F", shape=18, size=0.7) +
  geom_point(data=Feature2[which(Feature2$Level_1=="Environmental Information Processing"), ],
             aes(x=CCA1, y=CCA2, group=Genus),
             color="#B2DF8A", shape=18, size=0.7, alpha=0.75) +
  geom_point(data=Feature2[which(Feature2$Level_1=="Unclassified metabolism"), ],
             aes(x=CCA1, y=CCA2, group=Genus), color="#CAB2D6", shape=18, size=0.7) +
  geom_point(data=Feature2[which(Feature2$Level_1=="Other"), ],
             aes(x=CCA1, y=CCA2, group=Genus), color="#B3B3B3", shape=18, size=0.7) +
  geom_segment(data=arrows, aes(x=xstart, y=ystart, xend=CCA1, yend=CCA2),
               arrow=arrow(length = unit(0.01, "npc"))) +
  geom_text(data=arrows, aes(x = CCA1, y = CCA2, label = naming),
            hjust = 0, nudge_x = 0.05, size=6) +
  theme_bw() +
  theme(panel.grid.major = element_blank(),
        panel.grid.minor = element_blank(),
        axis.title=element_text(size=22),
        legend.position="none",
        legend.title=element_text(size=20),
        legend.text=element_text(size=20),
        axis.text.x = element_text(angle = 45, hjust = 1, size=12),
        axis.text.y = element_text(angle = 45, hjust = 1, size=12)) +
  scale_y_reverse()
pdf("MicroLADA_CCA_Func.pdf", width=9, height=6)
p1
dev.off()
```

```
## pdf
##   2
```

```r
#Save to list
Fig2List[[ "CCAalls" ]] <- p1

#Created plotly version only to be able to look into specific genera plotted
ggplotly(p1)
```

```r
ggplotly(p1+xlim(-1.5, 2.5)+ylim(-1.5,2))
```

```r
#Overlay plots with cowplot
#p2<-p1+xlim(-1.5, 2.5)+ylim(-1.5,2)
p2<-ggplot() +
#  geom_point(data=Metadata2[which(Metadata2$Diagnosis=='Control'), ],
#             aes(x=CCA1, y=CCA2, shape=Metformin, group=Diagnosis),
#             color="#666666", size=1.5, alpha=0.5) +
#  geom_point(data=Metadata2[which(Metadata2$Diagnosis=='T1D'), ],
#             aes(x=CCA1, y=CCA2, shape=Metformin, group=Diagnosis),
#             color="#1F78B4", size=1.5, alpha=0.5) +
```

```
#  geom_point(data=Metadata2[which(Metadata2$Diagnosis=='T2D'), ],
#             aes(x=CCA1, y=CCA2, shape=Metformin, group=Diagnosis),
#             color="#33A02C", size=1.5, alpha=0.5) +
#  geom_point(data=Metadata2[which(Metadata2$Diagnosis=='LADA'), ],
#             aes(x=CCA1, y=CCA2, shape=Metformin, group=Diagnosis),
#             color="#E31A1C", size=1.5, alpha=0.5) +
  scale_shape_manual(values=c(3, 16)) +
  geom_point(data=Feature2[which(Feature2$Level_1=="Cellular Processes"), ],
             aes(x=CCA1, y=CCA2, group=Genus), color="#A6CEE3", shape=18, size=3) +
  geom_point(data=Feature2[which(Feature2$Level_1=="Genetic Information Processing"), ],
             aes(x=CCA1, y=CCA2, group=Genus), color="#FB9A99", shape=18, size=3) +
  geom_point(data=Feature2[which(Feature2$Level_1=="Metabolism"), ],
             aes(x=CCA1, y=CCA2, group=Genus), color="#FDBF6F", shape=18, size=3) +
  geom_point(data=Feature2[which(Feature2$Level_1=="Environmental Information Processing"), ],
             aes(x=CCA1, y=CCA2, group=Genus),
             color="#B2DF8A", shape=18, size=3, alpha=0.75) +
  geom_point(data=Feature2[which(Feature2$Level_1=="Unclassified metabolism"), ],
             aes(x=CCA1, y=CCA2, group=Genus), color="#CAB2D6", shape=18, size=3) +
  geom_point(data=Feature2[which(Feature2$Level_1=="Other"), ],
             aes(x=CCA1, y=CCA2, group=Genus), color="#B3B3B3", shape=18, size=3) +
  geom_segment(data=arrows, aes(x=xstart, y=ystart, xend=CCA1, yend=CCA2),
               arrow=arrow(length = unit(0.01, "npc"))) +
  geom_text(data=arrows, aes(x = CCA1, y = CCA2, label = naming),
            hjust = 0, nudge_x = 0.05, size=10) +
#  geom_text(data=textgenus, aes(x = CCA1, y = CCA2, label = Genus),
#            hjust = 0, nudge_x = 0.05, size=8) +
  theme_bw() +
  theme(panel.grid.major = element_blank(),
        panel.grid.minor = element_blank(),
        axis.title=element_text(size=30),
        legend.position="none",
        legend.title=element_text(size=20),
        legend.text=element_text(size=20),
        axis.text.x = element_text(angle = 45, hjust = 1, size=22),
        axis.text.y = element_text(angle = 45, hjust = 1, size=22)) +
  scale_y_reverse() +
  xlim(-2.5, 2.5) +
  ylim(-1.5,2)


p3<-p1 + theme(axis.line=element_blank(),
               axis.text.x=element_blank(),
               axis.text.y=element_blank(),
               axis.ticks=element_blank(),
               axis.title.x=element_blank(),
               axis.title.y=element_blank(),
               legend.position="none",
               panel.background=element_blank(),
               #panel.border=element_blank(),
               panel.grid.major=element_blank(),
               panel.grid.minor=element_blank(),
               plot.background=element_blank())
```

```
ggdraw(p2) + draw_plot(p3, x = 0.30, y = -0.22, scale = 0.40)
```

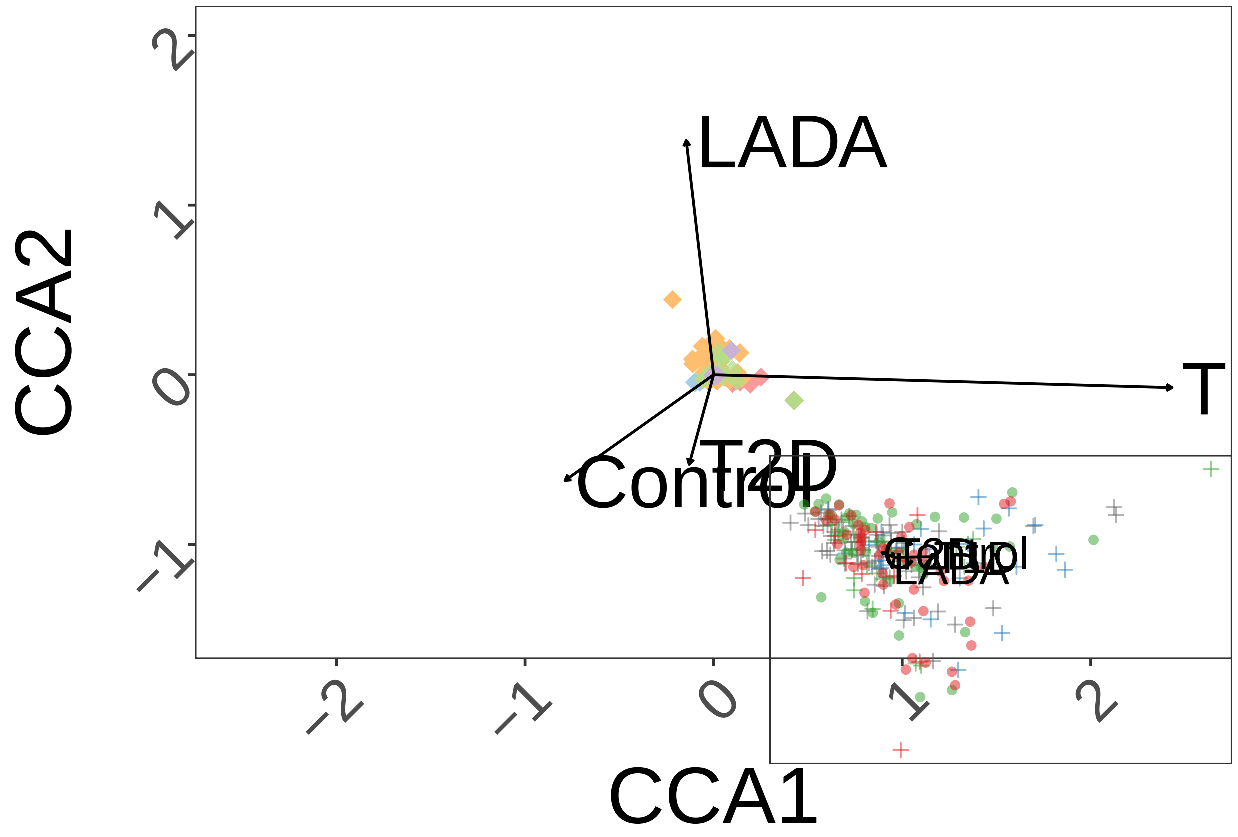


```
p4<-ggdraw(p2) + draw_plot(p3, x = 0.30, y = -0.18, scale = 0.40) #-22 below axis
#Save to list
Fig2List[[ "CCAoverlay" ]] <- p4
pdf("MicroLADA_CCAoverlay_Func.pdf", width=9, height=6)
p4
dev.off()
```

```
## pdf
##   2
```

```
##Extract legends
#Extract legends Level_1
Orgcol<-c("Cellular Processes" = "#A6CEE3",
          "Genetic Information Processing" = "#FB9A99",
          "Metabolism" = "#FDBF6F",
          "Environmental Information Processing" = "#B2DF8A",
          "Unclassified metabolism" = "#CAB2D6",
          "Other" = "#B3B3B3")
#Plot with only legend colors for Level_1
legend<-ggplot() +
  geom_point(data=Feature2, aes(x=CCA1, y=CCA2, group=Genus, color=Level_1),
             shape=18, size=6) +
```

```
  scale_color_manual(values=Orgcol) +
  theme_bw() +
  theme(legend.title=element_text(size=16),
        legend.text=element_text(size=14),
        legend.position="bottom")
#Store legends
legendplot<-get_legend(legend)
pdf("LegendCCALevel_1_bottom_Func.pdf", width=24, height=12)
grid.arrange(legendplot)
dev.off()
```

```
## pdf
##   2
```

```
#Save to list
Fig2List[[ "legendLevel_1bottom" ]] <- legendplot

#Plot with only legend colors for Level_1
legend<-ggplot() +
  geom_point(data=Feature2, aes(x=CCA1, y=CCA2, group=Genus, color=Level_1),
             shape=18, size=6) +
  scale_color_manual(values=Orgcol) +
  theme_bw() +
  theme(legend.title=element_text(size=16),
        legend.text=element_text(size=14),
        legend.position="right")
#Store legends
legendplot<-get_legend(legend)
pdf("LegendCCALevel_1_right_Func.pdf", width=24, height=12)
grid.arrange(legendplot)
dev.off()
```

```
## pdf
##   2
```

```
#Save to list
Fig2List[[ "legendLevel_1right" ]] <- legendplot

#Extract legends diagnosis
legend<-ggplot() +
  geom_point(data=Metadata2,
             aes(x=CCA1, y=CCA2, shape=Metformin, color=Diagnosis, group=Diagnosis),
             size=6, alpha=0.5) +
  scale_shape_manual(values=c(3, 16)) +
  scale_color_manual(values=c(Control = "#666666", T1D = "#1F78B4",
                              T2D = "#33A02C", LADA = "#E31A1C")) +
  theme_bw() +
  theme(legend.title=element_text(size=16),
        legend.text=element_text(size=14),
        legend.position="bottom")
#Store legends
legendplot<-get_legend(legend)
```

```
pdf("LegendCCAsamples_bottom_Func.pdf", width=24, height=12)
grid.arrange(legendplot)
dev.off()
```

```
## pdf
##   2
```

```
#Save to list
Fig2List[[ "legendsamplesbottom" ]] <- legendplot

#Extract legends diagnosis
legend<-ggplot() +
  geom_point(data=Metadata2,
             aes(x=CCA1, y=CCA2, shape=Metformin, color=Diagnosis, group=Diagnosis),
             size=6, alpha=0.5) +
  scale_shape_manual(values=c(3, 16)) +
  scale_color_manual(values=c(Control = "#666666", T1D = "#1F78B4",
                              T2D = "#33A02C", LADA = "#E31A1C")) +
  theme_bw() +
  theme(legend.title=element_text(size=16),
        legend.text=element_text(size=14),
        legend.position="right")

#Store legends
legendplot<-get_legend(legend)
pdf("LegendCCAsamples_right_Func.pdf", width=24, height=12)
grid.arrange(legendplot)
dev.off()
```

```
## pdf
##   2
```

```
#Save to list
Fig2List[[ "legendsamplesright" ]] <- legendplot
```

DESeq2 LRT

Analysis of deviance ANODEV
Size factors as column sums (Relative abundance), Total count standardized relative abundance as column sums

```
rm(list=setdiff(ls(), c("Metadata", "MetadataMed", "Feature", "TaxonomyDA", "Taxonomy", "Fig2List")))

#Can also be run with metformin as covariate, but decided not to due to treatment follow
#diagnosis.
#Better to compare with analysis run with individuals receiving metformin removed.

#Reassign names
Metadata2<-Metadata
Taxonomy2<-TaxonomyDA
```

```r
#The factor can not be ordered for DESeq to run
Metadata2$Diagnosis<-factor(Metadata2$Diagnosis, ordered=FALSE)


#Create design formula
#design <- formula(paste("~ ", "Diagnosis", "+",  "Metformin"))
design <- formula(paste("~ ", "Diagnosis", "+",  "BMIq"))
#design <- formula(paste("~ ", "Diagnosis"))
#Create DESeq2 object with matrix and metadata
dds <- DESeqDataSetFromMatrix(countData = Taxonomy2,
                              colData = Metadata2,
                              design = design)


##Assess and calculate size factors using the different methods
SF<-data.frame(Ratio=sizeFactors(estimateSizeFactors(dds, type="ratio")),
               Poscounts=sizeFactors(estimateSizeFactors(dds, type="poscounts")),
               Iterate=sizeFactors(estimateSizeFactors(dds, type="poscounts")),
               #"iterate" takes a lot of time changed to "poscounts" but kept due to the
               #following code
               Relative=colSums(Taxonomy2)/mean(colSums(Taxonomy2))
               )
SF<-add_rownames(SF, var="MicrobiomeID")
#Adding total abundances
# old path path<-paste("Q:/",
#             "Projects/",
#             "LADA/",
#             "LADA_Sandra_Evelina/",
#             "LADA_JKV/",
#             "LADA_R_AfterFlow_Analysis_FinalCounts/",
#             "LADA_FinalCounts/",
#             "2019-09-03_CountAnalysis.rds", sep="")

path <- paste("J:/",
              "CBMR/",
              "SUN-CBMR-Hansen-Group/",
              "Projects/",
              "LADA/",
              "LADA_Sandra_Evelina/",
              "LADA_JKV/",
              "LADA_R_AfterFlow_Analysis_FinalCounts/",
              "LADA_FinalCounts/",
              "2019-09-03_CountAnalysis.rds", sep="")


TotalCounts<-readRDS(path)
TotalCountsProcess <-
  data.frame(MicrobiomeID=TotalCounts$DF_Save_w$ID,
             CellNorm=TotalCounts$DF_Save_w$Cells_per_g_feces_FR_corrected_mean)
TotalCountsProcess$MicrobiomeID <- paste("L", TotalCountsProcess$MicrobiomeID,
                                         sep="")
SF2<-merge(SF, TotalCountsProcess, by="MicrobiomeID")
```

```r
#One value is NaN in sample 602059 assigns value as average
SF2$CellNorm[is.nan(SF2$CellNorm)]<-mean(na.omit(SF2$CellNorm))

SF2$CellNormStand<-SF2$CellNorm/mean(SF2$CellNorm) #Watch out for NaN values can assign
                                                    #average, but wants the error
SF3<-merge(Metadata2, SF2, by="MicrobiomeID")
#DESeq divides with sizeFactor (High cell count should have low sizeFactor providing
#larger counts in a sample)
#Still have to consider seq data is relative
SF3$NormRelCell<-SF3$Relative/SF3$CellNormStand
#Overview of normalization factors
aggregate(SF3[, 14:20], list(SF3$Diagnosis), mean) #Have to run heatmap chunk adding sex
```

```
##   Group.1    Ratio Poscounts  Iterate  Relative    CellNorm CellNormStand
## 1 Control 1.070161  1.059005 1.059005 0.9884311 20384275367     1.0420944
## 2     T1D 1.105743  1.096874 1.096874 1.0263342 18731782275     0.9576149
## 3    LADA 1.093748  1.089036 1.089036 0.9984496 18940404238     0.9682802
## 4     T2D 1.087719  1.081155 1.081155 1.0016117 19624620327     1.0032590
##   NormRelCell
## 1    1.016138
## 2    1.210062
## 3    1.092808
## 4    1.139338
```

```r
                                                    #to metadata
aggregate(SF3[, 14:20], list(SF3$Diagnosis), sd)
```

```
##   Group.1     Ratio Poscounts   Iterate  Relative   CellNorm CellNormStand
## 1 Control 0.4597056 0.4523032 0.4523032 0.4273249 6781846681     0.3467047
## 2     T1D 0.4010043 0.3962703 0.3962703 0.3829215 5712095609     0.2920164
## 3    LADA 0.4434083 0.4388107 0.4388107 0.4058688 5544046390     0.2834253
## 4     T2D 0.3344981 0.3296724 0.3296724 0.3105289 6146706989     0.3142348
##   NormRelCell
## 1   0.4752640
## 2   0.6768389
## 3   0.4609629
## 4   0.6643237
```

```r
pairs(SF3[, c(14:17,19,20)], col=SF3$Diagnosis)
```

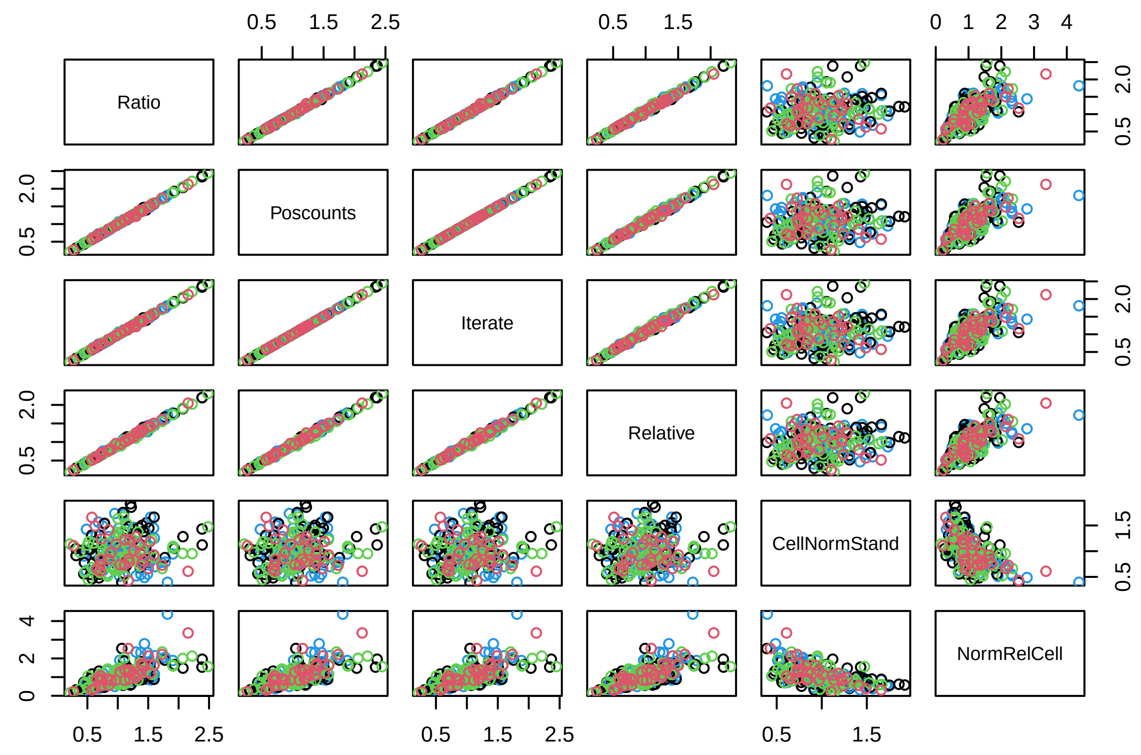


```
pairs(SF3[, c(15,17,19,20)], col=SF3$Diagnosis)
```

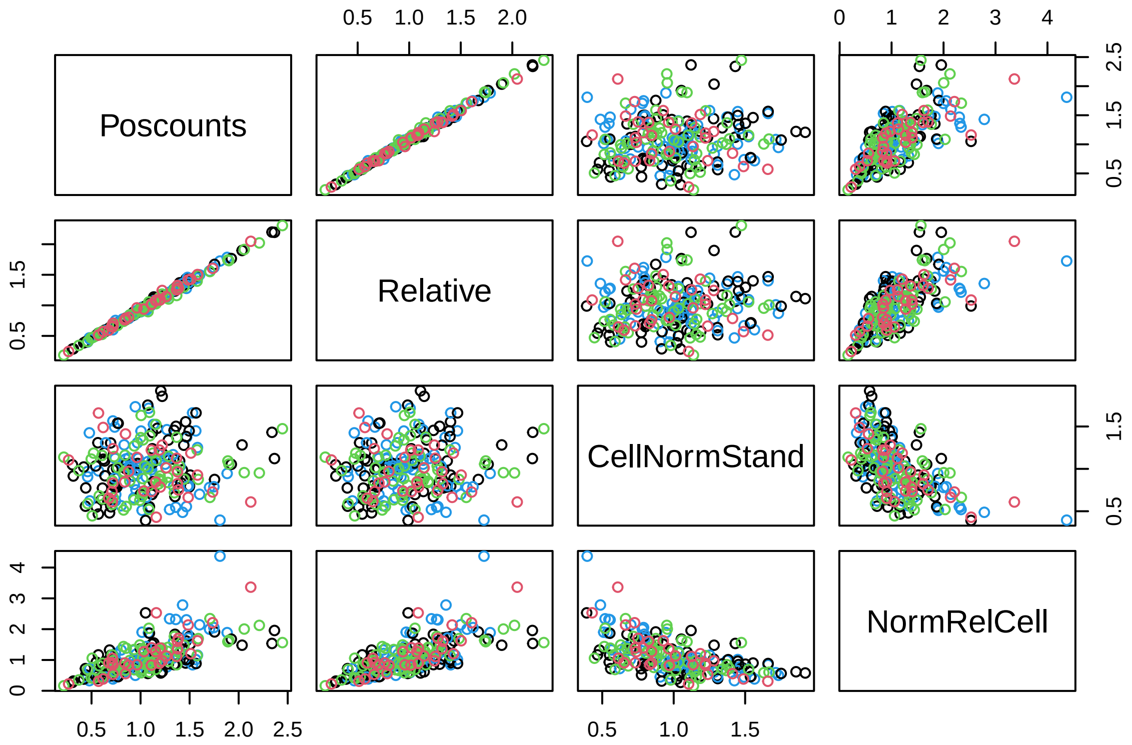


```
#I do want CellNormStand and NormRelCell to be negatively correlated due to DESeq divide
#with sizeFactor. It is also good that CellNormStand seems uncorrelated to the other
#normalization factors.


##Select size factors calculated above for normalization
dds@colData@listData$sizeFactor <- SF3[,20]
#hist(dds@colData@listData$sizeFactor)
#colnames(SF3[13])
normname<-colnames(SF3[20])

##See vignette for note on factor levels
#Filtering of the Counttable. Bare minimum is performed later. Also to reduce memory of
#the dds
dds2 <- dds[ rowSums(counts(dds))>50*length(Taxonomy2),] #Previously 0 instead of 1

#Estimate dispersions and fit the GLM
#dds2 <- DESeq(dds2, test="LRT", reduced = ~Metformin)
dds2 <- DESeq(dds2, test="LRT", reduced = ~1)

res<-results(dds2)
summary(res)
```

```
##
## out of 197 with nonzero total read count
```

```
## adjusted p-value < 0.1
## LFC > 0 (up)       : 11, 5.6%
## LFC < 0 (down)     : 4, 2%
## outliers [1]       : 0, 0%
## low counts [2]     : 0, 0%
## (mean count < 64)
## [1] see 'cooksCutoff' argument of ?results
## [2] see 'independentFiltering' argument of ?results
```

```
resultsNames(dds2)
```

```
## [1] "Intercept"                 "Diagnosis_T1D_vs_Control"
## [3] "Diagnosis_LADA_vs_Control" "Diagnosis_T2D_vs_Control"
## [5] "BMIq"
```

```
#MA plots from inbuild DESeq function
plotMA(dds2, alpha=0.1)
```

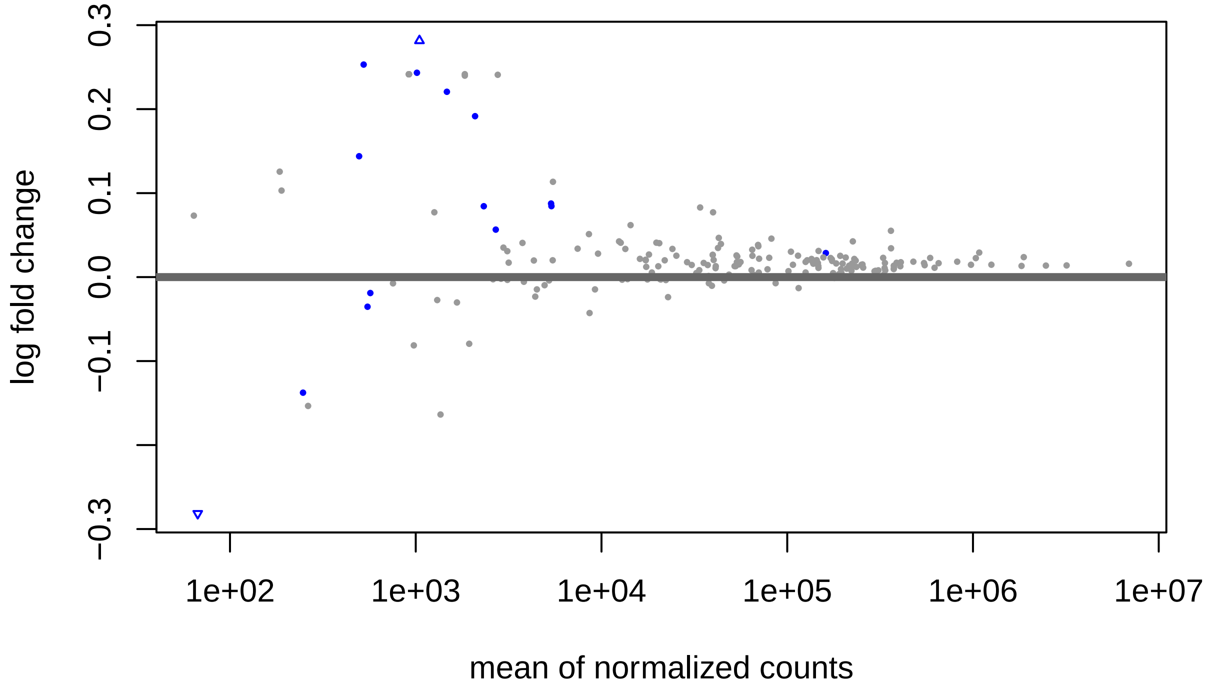


```
plotMA(res, ylim=c(-2,2))
```

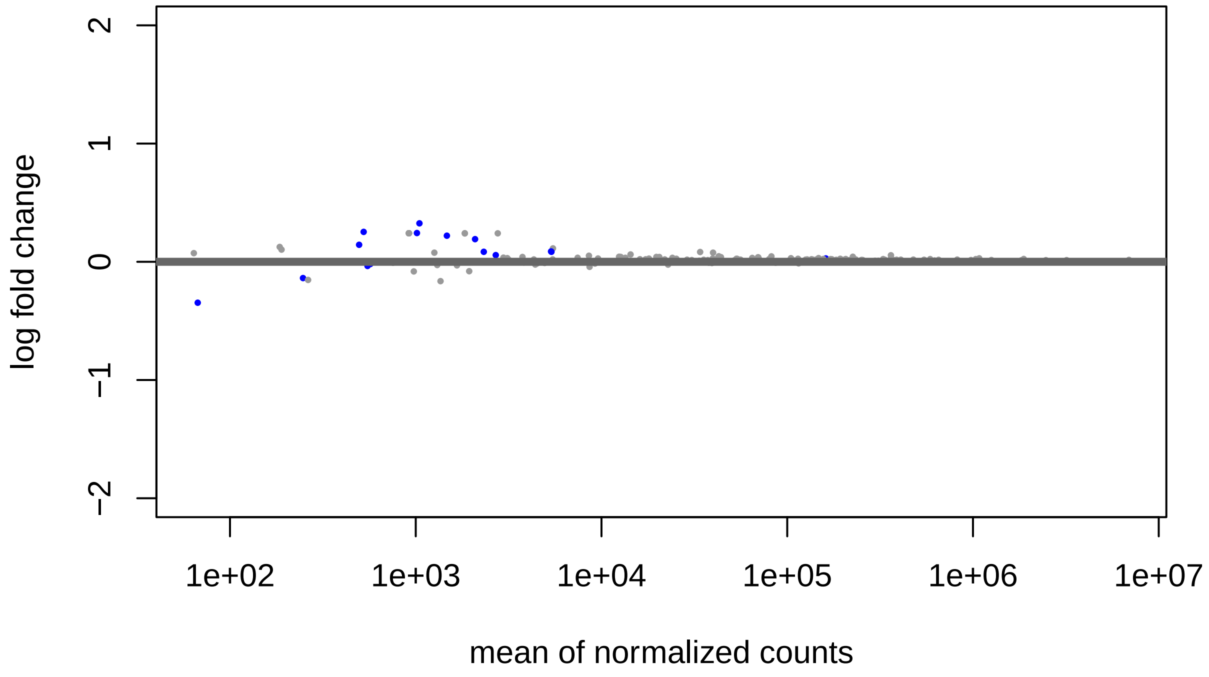


```
#Unshrunken/shrunken
#res<-results(dds2, addMLE=TRUE)
#resLFC <- DESeq2::lfcShrink(dds2, coef=2)
#resLFC
#DESeq2::plotMA(resLFC, ylim=c(-2,2))

#Make histogram
#pdf(paste("HistogramDESeq_Func", ".pdf", sep=""), height=6, width=12)
hist(res$pvalue[res$baseMean > 1], breaks = 0:20/20,
     col = "grey50", border = "white")
```

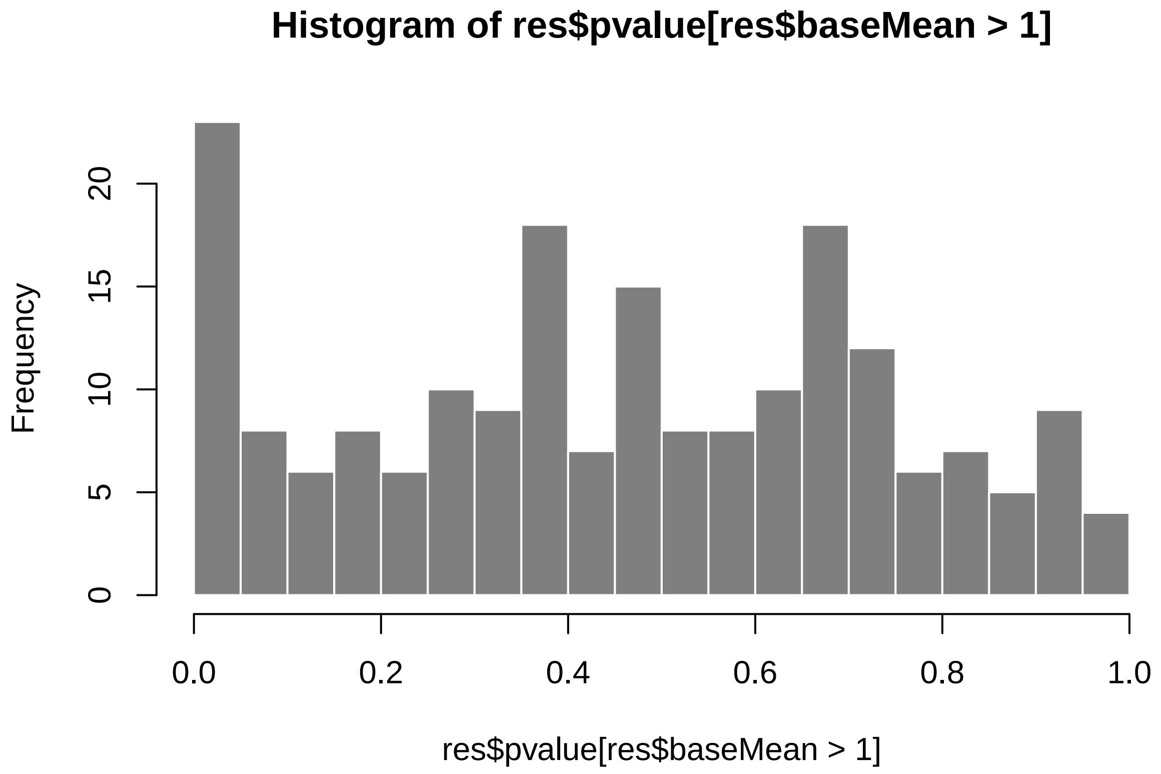


```
#dev.off()

#Show dispersion plot with shrinkage
#pdf(paste("DispersionplotDESeq_Func", ".pdf", sep=""), height=9, width=12)
plotDispEsts(dds2)
```

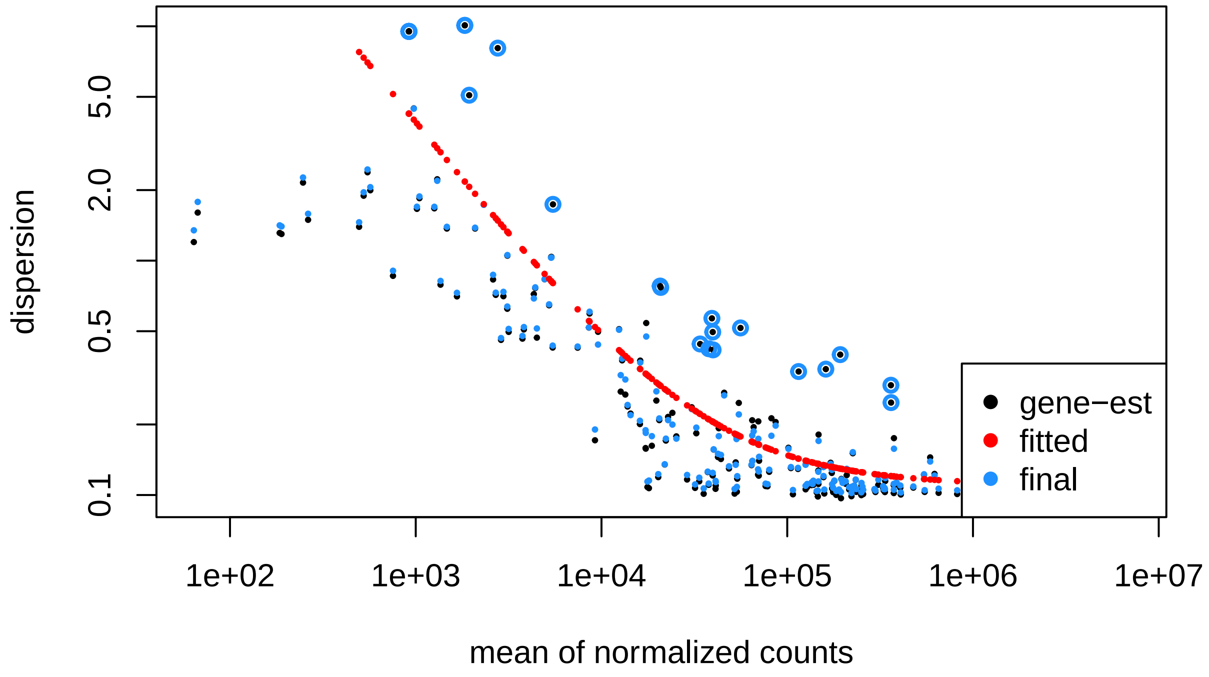


```
#dev.off()

##Plot PCA
#dds2_rlog<-rlogTransformation(dds2)
##pdf(paste("PCADESeq", "_Func.pdf", sep=""), height=9, width=9)
#plotPCA(dds2_rlog, intgroup=c("Diagnosis"))+coord_fixed()
##dev.off()

#Data structuring
resSig=subset(res, pvalue<0.05) #Could also have selected other value or padj
df <- data.frame(res)
dfSig <- data.frame(resSig)

#Save table.
write.table(dfSig, file="DESeq_SigLRT_Func.txt", sep="\t", dec=",", row.names = T)
print("Significant LRT")
```

```
## [1] "Significant LRT"
```

```
kable(dfSig)
```

	baseMean	log2FoldChange	lfcSE	stat	pvalue	padj
p53 signaling pathway [PATH:ko04115]	67.03765	-0.3462220	0.1234586	17.868335	0.0013094	0.0214960
Bacterial chemotaxis [PATH:ko02030]	161375.11235	0.0286387	0.0539908	15.062812	0.0045727	0.0643444
Flagellar assembly [PATH:ko02040]	192929.94040	0.0253150	0.0579786	13.237947	0.0101698	0.1252158
Biofilm formation - Escherichia coli [PATH:ko02026]	147465.48787	0.0311363	0.0379490	9.944097	0.0413800	0.3544284
Biofilm formation - Pseudomonas aeruginosa [PATH:ko02025]	82196.16111	0.0457934	0.0389151	12.165083	0.0161650	0.1676053
Two-component system [PATH:ko02020]	588209.87941	0.0228148	0.0342942	10.055614	0.0395012	0.3537154
Betalain biosynthesis [PATH:ko00965]	524.40214	0.2530536	0.1288235	58.867950	0.0000000	0.0000000
Glycosphingolipid biosynthesis - lacto and neolacto series [PATH:ko00601]	247.27768	-0.1376275	0.1385842	16.900184	0.0020212	0.0306288
alpha-Linolenic acid metabolism [PATH:ko00592]	1471.38976	0.2206515	0.1086613	73.388659	0.0000000	0.0000000
Arachidonic acid metabolism [PATH:ko00590]	3111.36013	0.0308902	0.0734511	12.640277	0.0131740	0.1441822
Ether lipid metabolism [PATH:ko00565]	495.64764	0.1439061	0.1112097	73.197018	0.0000000	0.0000000
Linoleic acid metabolism [PATH:ko00591]	1047.12814	0.3253528	0.1261657	84.732581	0.0000000	0.0000000
Retinol metabolism [PATH:ko00830]	4324.77225	0.0197545	0.0764130	12.799084	0.0123004	0.1425399
Biosynthesis of ansamycins [PATH:ko01051]	40162.46897	0.0204030	0.0364185	10.115696	0.0385232	0.3537154
Biosynthesis of siderophore group nonribosomal peptides [PATH:ko01053]	12436.02756	0.0424890	0.0655159	11.300248	0.0233890	0.2303819
Carotenoid biosynthesis [PATH:ko00906]	2324.42828	0.0843719	0.1210795	22.217312	0.0001814	0.0032489
Geraniol degradation [PATH:ko00281]	2086.54612	0.1916058	0.1081870	71.573730	0.0000000	0.0000000
Caprolactam degradation [PATH:ko00930]	2696.88468	0.0565364	0.0786146	14.456868	0.0059710	0.0784193
Drug metabolism - cytochrome P450 [PATH:ko00982]	5377.28344	0.0844695	0.0934196	42.585161	0.0000000	0.0000003
Ethylbenzene degradation [PATH:ko00642]	1015.07229	0.2433126	0.1199433	57.995719	0.0000000	0.0000000
Fluorobenzoate degradation [PATH:ko00364]	550.12354	-0.0352785	0.1440534	30.316102	0.0000042	0.0000831
Metabolism of xenobiotics by cytochrome P450 [PATH:ko00980]	5353.44294	0.0875807	0.0933537	42.270019	0.0000000	0.0000003
Toluene degradation [PATH:ko00623]	569.52117	-0.0189657	0.1320982	51.254154	0.0000000	0.0000000

DESeq2 LRT remove metformin

Analysis of deviance ANODEV
Size factors as column sums (Relative abundance), Total count standardized relative abundance as column sums

```r
rm(list=setdiff(ls(), c("Metadata", "MetadataMed", "Feature", "TaxonomyDA", "Taxonomy",
                        "Fig2List")))
#Reassign names
#Metadata2<-Metadata
Taxonomy2<-TaxonomyDA

##Removing treated with metformin
#Metadata$Metformin <- ifelse(is.na(Metadata$Metformin), "Unknown", Metadata$Metformin)
Metadata2<-filter(Metadata, !Metformin == 1)
##Applying subsetting to OTU tables
Taxonomy2<-dplyr::select(Taxonomy2, one_of(as.character(Metadata2$MicrobiomeID)))
```

```
##Remove orgs that are not present after subsetting.
Taxonomy2 <- Taxonomy2[rowSums(Taxonomy2)>0,]


#Can also be run with metformin as covariate, but decided not to due to treatment follow
#diagnosis.
#Better to compare with analysis run with individuals receiving metformin removed.
#As performed here


#The factor can not be ordered for DESeq to run
Metadata2$Diagnosis<-factor(Metadata2$Diagnosis, ordered=FALSE)


#Create design formula
#design <- formula(paste("~ ", "Diagnosis", "+",  "Metformin"))
design <- formula(paste("~ ", "Diagnosis", "+",  "BMIq"))
#design <- formula(paste("~ ", "Diagnosis"))
#Create DESeq2 object with matrix and metadata
dds <- DESeqDataSetFromMatrix(countData = Taxonomy2,
                              colData = Metadata2,
                              design = design)


##Assess and calculate size factors using the different methods
SF<-data.frame(Ratio=sizeFactors(estimateSizeFactors(dds, type="ratio")),
               Poscounts=sizeFactors(estimateSizeFactors(dds, type="poscounts")),
               Iterate=sizeFactors(estimateSizeFactors(dds, type="poscounts")),
               #"iterate" takes a lot of time changed to "poscounts" but kept due to the
               #following code
               Relative=colSums(Taxonomy2)/mean(colSums(Taxonomy2))
               )
SF<-add_rownames(SF, var="MicrobiomeID")
#Adding total abundances
# old path path<-paste("Q:/",
#             "Projects/",
#             "LADA/",
#             "LADA_Sandra_Evelina/",
#             "LADA_JKV/",
#             "LADA_R_AfterFlow_Analysis_FinalCounts/",
#             "LADA_FinalCounts/",
#             "2019-09-03_CountAnalysis.rds", sep="")

path <- paste("J:/",
              "CBMR/",
              "SUN-CBMR-Hansen-Group/",
              "Projects/",
              "LADA/",
              "LADA_Sandra_Evelina/",
              "LADA_JKV/",
              "LADA_R_AfterFlow_Analysis_FinalCounts/",
              "LADA_FinalCounts/",
              "2019-09-03_CountAnalysis.rds", sep="")
```

```r
TotalCounts<-readRDS(path)
TotalCountsProcess <-
  data.frame(MicrobiomeID=TotalCounts$DF_Save_w$ID,
             CellNorm=TotalCounts$DF_Save_w$Cells_per_g_feces_FR_corrected_mean)
TotalCountsProcess$MicrobiomeID <- paste("L", TotalCountsProcess$MicrobiomeID,
                                         sep="")
SF2<-merge(SF, TotalCountsProcess, by="MicrobiomeID")

#One value is NaN in sample 602059 assigns value as average
SF2$CellNorm[is.nan(SF2$CellNorm)]<-mean(na.omit(SF2$CellNorm))

SF2$CellNormStand<-SF2$CellNorm/mean(SF2$CellNorm) #Watch out for NaN values can assign
                                                   #average, but wants the error
SF3<-merge(Metadata2, SF2, by="MicrobiomeID")
#DESeq divides with sizeFactor (High cell count should have low sizeFactor providing
#larger counts in a sample)
#Still have to consider seq data is relative
SF3$NormRelCell<-SF3$Relative/SF3$CellNormStand
#Overview of normalization factors
aggregate(SF3[, 14:20], list(SF3$Diagnosis), mean) #Have to run heatmap chunk adding sex
```

```
##   Group.1    Ratio Poscounts  Iterate  Relative    CellNorm CellNormStand
## 1 Control 1.094542  1.087591 1.087591 1.0032044 20384275367     1.0722125
## 2     T1D 1.131654  1.127744 1.127744 1.0416740 18731782275     0.9852914
## 3    LADA 1.004481  0.998413 0.998413 0.9142228 15590817082     0.8200767
## 4     T2D 1.069550  1.065472 1.065472 0.9806434 16982538343     0.8932812
##   NormRelCell
## 1    1.002356
## 2    1.193649
## 3    1.193676
## 4    1.191757
```

```r
                                                   #to metadata
aggregate(SF3[, 14:20], list(SF3$Diagnosis), sd)
```

```
##   Group.1     Ratio Poscounts   Iterate  Relative   CellNorm CellNormStand
## 1 Control 0.4695326 0.4639309 0.4639309 0.4337118 6781846681     0.3567250
## 2     T1D 0.4106770 0.4065139 0.4065139 0.3886448 5712095609     0.3004561
## 3    LADA 0.3345323 0.3282978 0.3282978 0.3071066 4604942130     0.2422199
## 4     T2D 0.3912487 0.3857159 0.3857159 0.3582493 4888235680     0.2571211
##   NormRelCell
## 1   0.4688179
## 2   0.6676588
## 3   0.5263781
## 4   0.5509790
```

```r
pairs(SF3[, c(14:17,19,20)], col=SF3$Diagnosis)
```

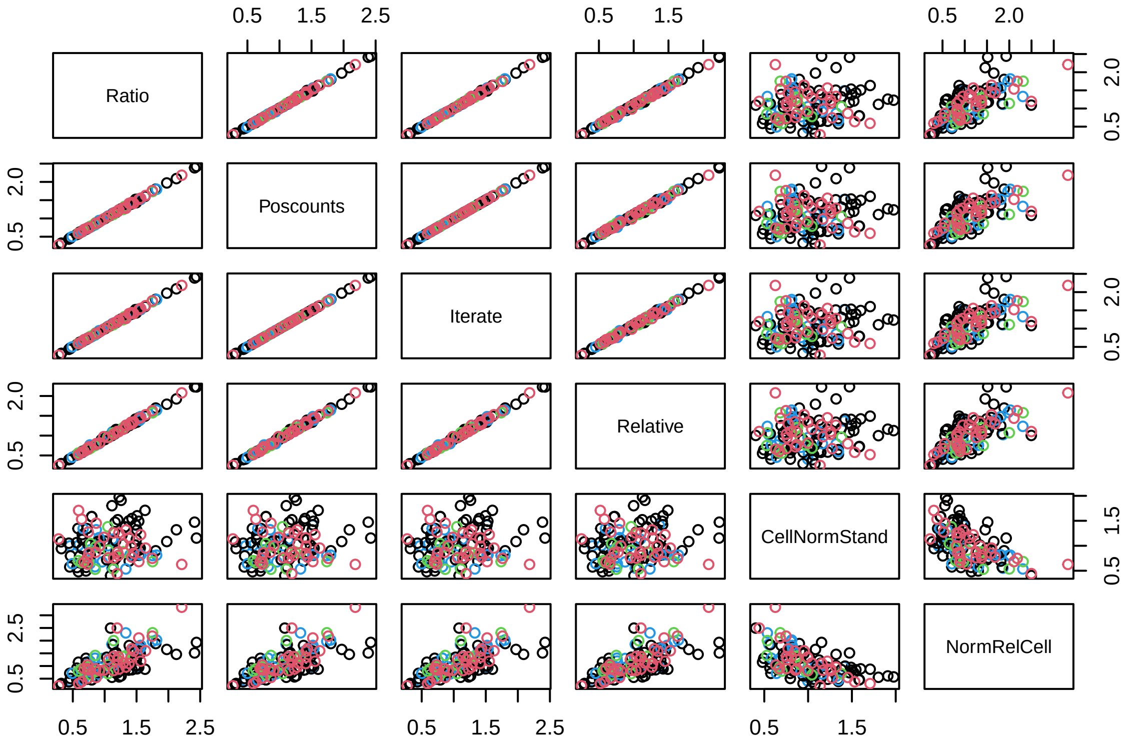


```
pairs(SF3[, c(15,17,19,20)], col=SF3$Diagnosis)
```

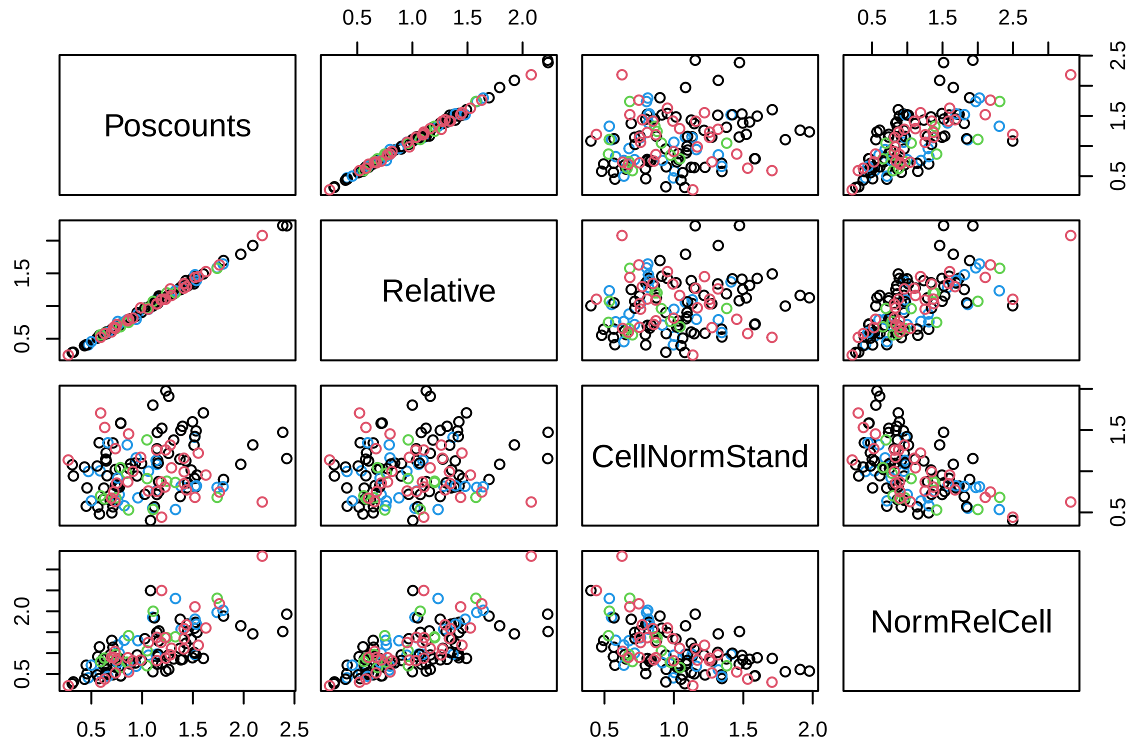


```
#I do want CellNormStand and NormRelCell to be negatively correlated due to DESeq divide
#with sizeFactor. It is also good that CellNormStand seems uncorrelated to the other
#normalization factors.


##Select size factors calculated above for normalization
dds@colData@listData$sizeFactor <- SF3[,20]
#hist(dds@colData@listData$sizeFactor)
#colnames(SF3[13])
normname<-colnames(SF3[20])

##See vignette for note on factor levels
#Filtering of the Counttable. Bare minimum is performed later. Also to reduce memory of
#the dds
dds2 <- dds[ rowSums(counts(dds))>50*length(Taxonomy2),] #Previously 0 instead of 1

#Estimate dispersions and fit the GLM
#dds2 <- DESeq(dds2, test="LRT", reduced = ~Metformin)
dds2 <- DESeq(dds2, test="LRT", reduced = ~1)

res<-results(dds2)
summary(res)
```

```
##
## out of 196 with nonzero total read count
```

```
## adjusted p-value < 0.1
## LFC > 0 (up)       : 79, 40%
## LFC < 0 (down)     : 1, 0.51%
## outliers [1]       : 0, 0%
## low counts [2]     : 42, 21%
## (mean count < 3775)
## [1] see 'cooksCutoff' argument of ?results
## [2] see 'independentFiltering' argument of ?results
```

```
resultsNames(dds2)
```

```
## [1] "Intercept"                 "Diagnosis_T1D_vs_Control"
## [3] "Diagnosis_LADA_vs_Control" "Diagnosis_T2D_vs_Control"
## [5] "BMIq"
```

```
#MA plots from inbuild DESeq function
plotMA(dds2, alpha=0.1)
```

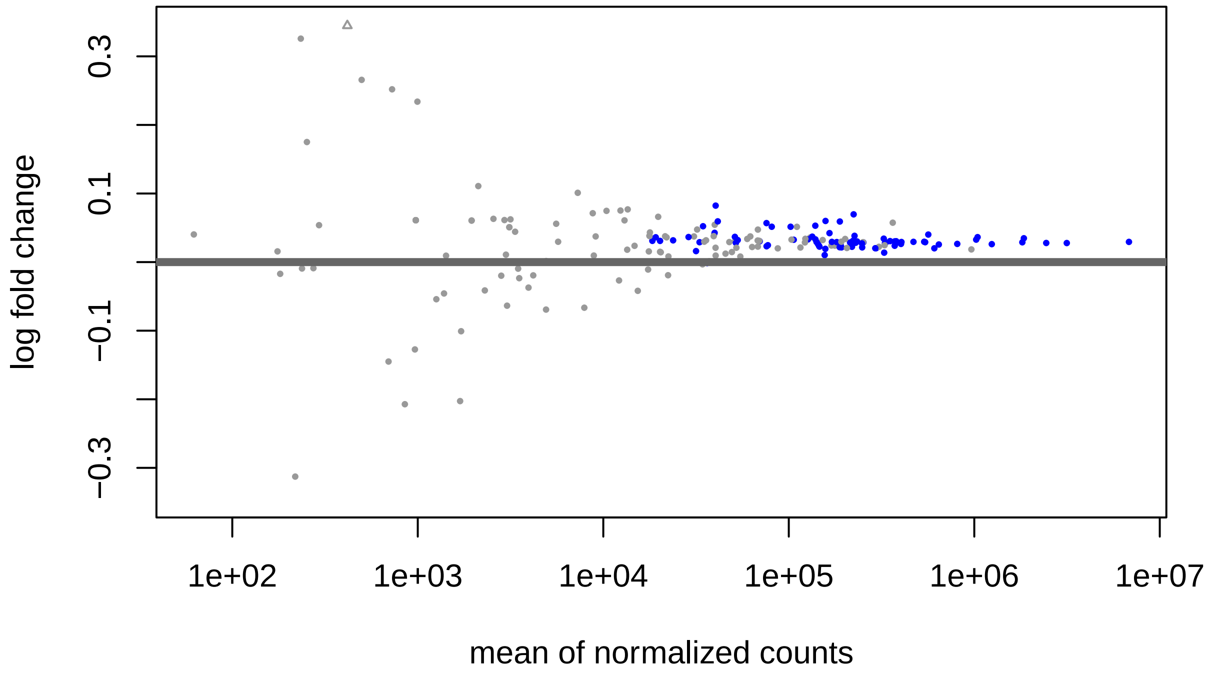


```
plotMA(res, ylim=c(-2,2))
```

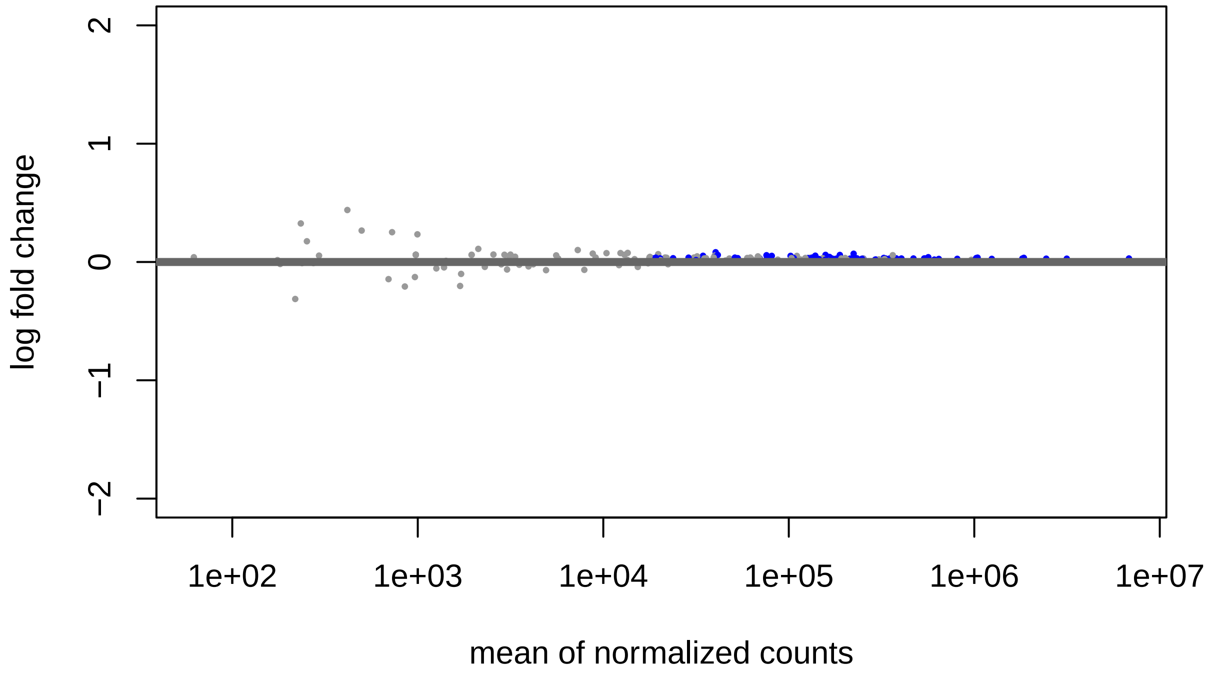


```
#Unshrunken/shrunken
#res<-results(dds2, addMLE=TRUE)
#resLFC <- DESeq2::lfcShrink(dds2, coef=2)
#resLFC
#DESeq2::plotMA(resLFC, ylim=c(-2,2))

#Make histogram
#pdf(paste("HistogramDESeq_Func", ".pdf", sep=""), height=6, width=12)
hist(res$pvalue[res$baseMean > 1], breaks = 0:20/20,
     col = "grey50", border = "white")
```

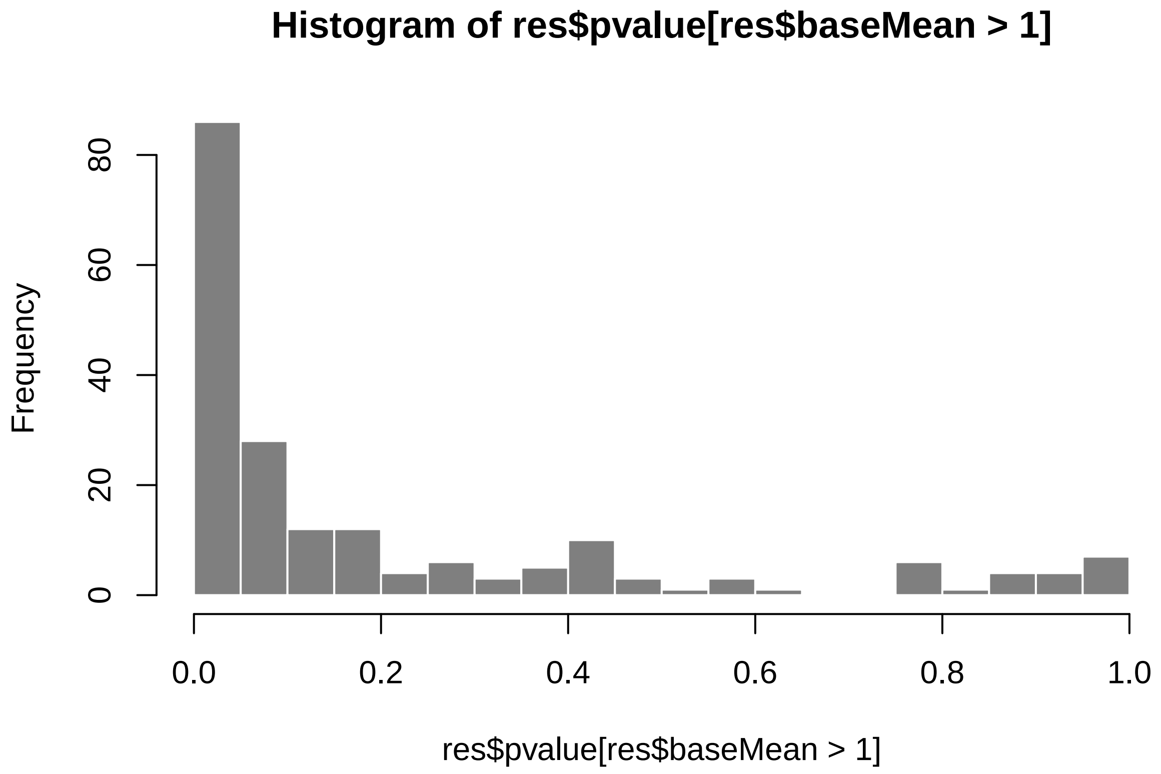


```
#dev.off()

#Show dispersion plot with shrinkage
#pdf(paste("DispersionplotDESeq_Func", ".pdf", sep=""), height=9, width=12)
plotDispEsts(dds2)
```

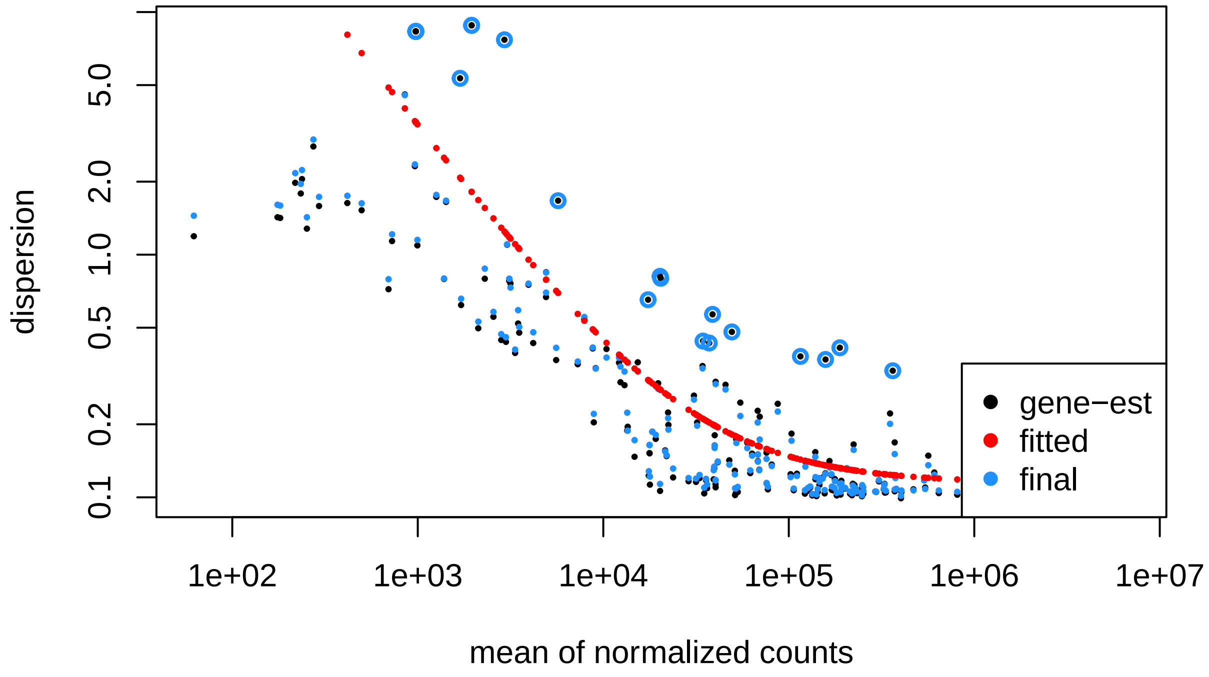


```
#dev.off()

##Plot PCA
#dds2_rlog<-rlogTransformation(dds2)
##pdf(paste("PCADESeq_Func", ".pdf", sep=""), height=9, width=9)
#plotPCA(dds2_rlog, intgroup=c("Diagnosis"))+coord_fixed()
##dev.off()

#Data structuring
resSig=subset(res, pvalue<0.05) #Could also have selected other value or padj
df <- data.frame(res)
dfSig <- data.frame(resSig)

#Save table.
write.table(dfSig, file="DESeq_SigLRTRemMet_Func.txt", sep="\t", dec=",", row.names = T)
print("Significant LRT")
```

```
## [1] "Significant LRT"
```

```
kable(dfSig)
```

	baseMean	log2FoldChange	lfcSE	stat	pvalue	padj
Cell cycle - Caulobacter [PATH:ko04112]	156155.0223	0.0102294	0.0390383	3.743665	0.0449742	0.0980163

	baseMean	log2FoldChange	lfcSE	stat	pvalue	padj
Necroptosis [PATH:ko04217]	75913.7098	0.0230857	0.0403843	12.761703	0.0125008	0.0716421
Bacterial chemotaxis [PATH:ko02030]	157791.6914	0.0599794	0.0725526	15.635683	0.0035491	0.0716421
Flagellar assembly [PATH:ko02040]	188508.8904	0.0591581	0.0767095	14.741204	0.0052692	0.0716421
Biofilm formation - Escherichia coli [PATH:ko02026]	139018.7817	0.0530369	0.0457020	14.803896	0.0051257	0.0716421
Biofilm formation - Pseudomonas aeruginosa [PATH:ko02025]	75850.0002	0.0568518	0.0452705	13.761869	0.0080952	0.0716421
Quorum sensing [PATH:ko02024]	609155.5539	0.0201238	0.0419884	10.571415	0.0318274	0.0980163
ABC transporters [PATH:ko02010]	1042044.9923	0.0363906	0.0404626	13.371063	0.0095981	0.0716421
Phosphotransferase system (PTS) [PATH:ko02060]	351515.7936	0.0306307	0.0534649	10.672218	0.0305056	0.0958748
AMPK signaling pathway [PATH:ko04152]	18387.7027	0.0309781	0.0515280	9.505000	0.0496446	0.0980163
Phosphatidylinositol signaling system [PATH:ko04070]	33035.9265	0.0289692	0.0419434	10.198823	0.0372085	0.0980163
Two-component system [PATH:ko02020]	565135.9901	0.0401506	0.0439340	15.208515	0.0042877	0.0716421
Protein export [PATH:ko03060]	219109.5944	0.0223135	0.0384590	11.315310	0.0232398	0.0872911
RNA degradation [PATH:ko03018]	192358.4294	0.0214721	0.0395286	9.569535	0.0483379	0.0980163
Sulfur relay system [PATH:ko04122]	102284.1173	0.0515231	0.0415281	14.386724	0.0061578	0.0716421
Base excision repair [PATH:ko03410]	143343.1025	0.0268067	0.0392458	10.069562	0.0392721	0.0980163
DNA replication [PATH:ko03030]	248750.5462	0.0214473	0.0399714	9.991782	0.0405663	0.0980163
Homologous recombination [PATH:ko03440]	326813.5597	0.0137626	0.0400694	11.112314	0.0253303	0.0911045
Mismatch repair [PATH:ko03430]	295711.6414	0.0202449	0.0387539	12.625659	0.0132574	0.0716421
Nucleotide excision repair [PATH:ko03420]	146391.9670	0.0225549	0.0406170	10.715397	0.0299558	0.0958748
RNA polymerase [PATH:ko03020]	69732.4972	0.0303577	0.0496302	11.102278	0.0254383	0.0911045
Aminoacyl-tRNA biosynthesis [PATH:ko00970]	1242724.7973	0.0261808	0.0393309	11.334319	0.0230529	0.0872911
Ribosome biogenesis in eukaryotes [PATH:ko03008]	34492.8040	0.0522575	0.0791854	12.447430	0.0143165	0.0716421
RNA transport [PATH:ko03013]	40338.5823	0.0823277	0.0645627	12.512931	0.0139180	0.0716421
Alanine, aspartate and glutamate metabolism [PATH:ko00250]	372509.6882	0.0302277	0.0391481	10.174194	0.0375934	0.0980163
Arginine biosynthesis [PATH:ko00220]	233417.0192	0.0300883	0.0388012	12.635580	0.0132008	0.0716421
Cysteine and methionine metabolism [PATH:ko00270]	402736.7400	0.0264769	0.0380029	11.660462	0.0200631	0.0792235
Glycine, serine and threonine metabolism [PATH:ko00260]	329925.6733	0.0274425	0.0390247	10.057557	0.0394692	0.0980163
Histidine metabolism [PATH:ko00340]	176722.9416	0.0254128	0.0394197	10.408960	0.0340744	0.0980163
Lysine biosynthesis [PATH:ko00300]	252348.0759	0.0287302	0.0388821	12.071911	0.0168245	0.0769422
Phenylalanine, tyrosine and tryptophan biosynthesis [PATH:ko00400]	333727.4578	0.0285433	0.0389202	13.420080	0.0093956	0.0716421
Valine, leucine and isoleucine biosynthesis [PATH:ko00290]	214043.7487	0.0285730	0.0387760	14.044909	0.0071531	0.0716421
Betalain biosynthesis [PATH:ko00965]	234.0752	0.3257942	0.1671929	18.420445	0.0010211	NA
Carbapenem biosynthesis [PATH:ko00332]	31621.9628	0.0160440	0.0412298	12.430464	0.0144215	0.0716421
Flavone and flavonol biosynthesis [PATH:ko00944]	1712.2201	- 0.1008116	0.0968405	9.893287	0.0422641	NA
Glucosinolate biosynthesis [PATH:ko00966]	20233.1183	0.0308950	0.0402006	13.874131	0.0077078	0.0716421
Monobactam biosynthesis [PATH:ko00261]	106341.5172	0.0325544	0.0393192	12.105928	0.0165807	0.0769422
Novobiocin biosynthesis [PATH:ko00401]	51850.3070	0.0279951	0.0393228	9.838846	0.0432316	0.0980163
Phenazine biosynthesis [PATH:ko00405]	28826.6477	0.0365387	0.0413389	14.155665	0.0068145	0.0716421
Streptomycin biosynthesis [PATH:ko00521]	139233.4459	0.0331009	0.0415286	10.127737	0.0383301	0.0980163
Amino sugar and nucleotide sugar metabolism [PATH:ko00520]	543686.3387	0.0290336	0.0392484	9.612601	0.0474843	0.0980163

	baseMean	log2FoldChange	lfcSE	stat	pvalue	padj
C5-Branched dibasic acid metabolism [PATH:ko00660]	126907.2600	0.0330652	0.0394487	12.572351	0.0135659	0.0716421
Fructose and mannose metabolism [PATH:ko00051]	372388.2684	0.0237951	0.0463177	10.294160	0.0357537	0.0980163
Galactose metabolism [PATH:ko00052]	375800.7897	0.0271139	0.0413407	9.974816	0.0408541	0.0980163
Glycolysis / Gluconeogenesis [PATH:ko00010]	470076.0858	0.0295308	0.0389930	11.669156	0.0199888	0.0792235
Pentose phosphate pathway [PATH:ko00030]	325138.2598	0.0341630	0.0392972	12.796910	0.0123120	0.0716421
Propanoate metabolism [PATH:ko00640]	229437.8083	0.0282093	0.0393520	9.679454	0.0461876	0.0980163
Starch and sucrose metabolism [PATH:ko00500]	536904.8335	0.0296438	0.0408857	12.445962	0.0143255	0.0716421
Carbon fixation in photosynthetic organisms [PATH:ko00710]	221545.5958	0.0308479	0.0398933	13.018823	0.0111842	0.0716421
Nitrogen metabolism [PATH:ko00910]	133882.2838	0.0370665	0.0383328	10.913645	0.0275519	0.0922388
Photosynthesis [PATH:ko00195]	157444.2964	0.0193221	0.0421436	15.760283	0.0033583	0.0716421
Sulfur metabolism [PATH:ko00920]	165767.9102	0.0422248	0.0434381	11.906316	0.0180617	0.0792235
Glycosphingolipid biosynthesis - lacto and neolacto series [PATH:ko00601]	218.4148	-0.3127370	0.1760430	23.790826	0.0000880	NA
Peptidoglycan biosynthesis [PATH:ko00550]	292604.4221	0.0202084	0.0387679	13.000352	0.0112741	0.0716421
alpha-Linolenic acid metabolism [PATH:ko00592]	726.8127	0.2519042	0.1315093	23.602559	0.0000959	NA
Ether lipid metabolism [PATH:ko00565]	252.5127	0.1750544	0.1427429	20.730118	0.0003582	NA
Glycerolipid metabolism [PATH:ko00561]	145328.6061	0.0240442	0.0413365	14.561904	0.0057017	0.0716421
Glycerophospholipid metabolism [PATH:ko00564]	226187.9392	0.0383040	0.0395252	13.768433	0.0080720	0.0716421
Linoleic acid metabolism [PATH:ko00591]	417.1435	0.4393820	0.1579400	28.572233	0.0000095	NA
Nicotinate and nicotinamide metabolism [PATH:ko00760]	170759.3562	0.0294414	0.0396938	9.938247	0.0414808	0.0980163
One carbon pool by folate [PATH:ko00670]	188159.1347	0.0216635	0.0402430	10.969075	0.0269138	0.0921049
Pantothenate and CoA biosynthesis [PATH:ko00770]	247957.9855	0.0272438	0.0381104	13.915977	0.0075681	0.0716421
Porphyrin and chlorophyll metabolism [PATH:ko00860]	223648.1833	0.0696239	0.0472561	9.527985	0.0491754	0.0980163
Thiamine metabolism [PATH:ko00730]	224290.1230	0.0316751	0.0385939	14.485060	0.0058975	0.0716421
Vitamin B6 metabolism [PATH:ko00750]	77161.2179	0.0245918	0.0396806	9.837047	0.0432639	0.0980163
beta-Alanine metabolism [PATH:ko00410]	51220.4380	0.0367816	0.0420666	9.987803	0.0406336	0.0980163
D-Alanine metabolism [PATH:ko00473]	36295.1835	-0.0014071	0.0401163	9.549670	0.0487366	0.0980163
D-Glutamine and D-glutamate metabolism [PATH:ko00471]	53072.2312	0.0321141	0.0396355	9.776386	0.0443677	0.0980163
Selenocompound metabolism [PATH:ko00450]	141097.1809	0.0293988	0.0382624	12.763053	0.0124935	0.0716421
Biosynthesis of ansamycins [PATH:ko01051]	39830.4805	0.0426722	0.0476885	15.956461	0.0030781	0.0716421
Geraniol degradation [PATH:ko00281]	995.3726	0.2339052	0.1280344	23.424733	0.0001041	NA
Polyketide sugar unit biosynthesis [PATH:ko00523]	80950.4860	0.0515218	0.0437570	9.661384	0.0465347	0.0980163
Terpenoid backbone biosynthesis [PATH:ko00900]	181462.8427	0.0291771	0.0385264	12.049460	0.0169872	0.0769422
Purine metabolism [PATH:ko00230]	809598.0460	0.0265205	0.0387243	10.329582	0.0352272	0.0980163
Pyrimidine metabolism [PATH:ko00240]	644062.4895	0.0256654	0.0389685	10.115657	0.0385239	0.0980163
Aminobenzoate degradation [PATH:ko00627]	19177.7585	0.0361688	0.0507485	9.796643	0.0439961	0.0980163
Benzoate degradation [PATH:ko00362]	41420.4099	0.0594041	0.0447097	9.650295	0.0467490	0.0980163
Chloroalkane and chloroalkene degradation [PATH:ko00625]	23793.3043	0.0317244	0.0432505	9.910533	0.0419620	0.0980163

	baseMean	log2FoldChange	lfcSE	stat	pvalue	padj
Ethylbenzene degradation [PATH:ko00642]	498.1493	0.2656974	0.1523683	18.656069	0.0009181	NA
2-Oxocarboxylic acid metabolism	404909.1603	0.0293035	0.0389662	13.573739	0.0087875	0.0716421
Biosynthesis of amino acids	1815934.8140	0.0288538	0.0384808	13.463480	0.0092198	0.0716421
Biosynthesis of antibiotics	2443269.7600	0.0278728	0.0383875	11.749795	0.0193122	0.0792235
Biosynthesis of secondary metabolites	3152810.7079	0.0277881	0.0380911	11.777829	0.0190822	0.0792235
Carbon metabolism	1023857.9733	0.0327533	0.0400323	9.774653	0.0443996	0.0980163
Metabolic pathways	6821207.1504	0.0294363	0.0382936	10.692735	0.0302432	0.0958748
Microbial metabolism in diverse environments	1850407.2352	0.0347478	0.0393555	11.025602	0.0262778	0.0919722

DESeq Wald

All pairwise Differential abundance analysis comparison (DESeq and visualized venn)

```
rm(list=setdiff(ls(), c("Metadata", "MetadataMed", "Feature", "TaxonomyDA", "Taxonomy", "Fig2List")))

#Reassign names
Metadata2<-Metadata
#Order Diagnosis
Metadata2$Diagnosis<-ordered(Metadata2$Diagnosis,
                            levels=c("Control", "T1D", "T2D", "LADA"))
#Included filtering
Taxonomy2<-TaxonomyDA
#Taxonomy2<-Taxonomy[length(Taxonomy)-rowSums(Taxonomy == 0) >= 30,]


#The factor can not be ordered for DESeq to run
Metadata2$Diagnosis<-factor(Metadata2$Diagnosis, ordered=FALSE)

#Create design formula
#design <- formula(paste("~ ", "Diagnosis"))
design <- formula(paste("~ ", "Diagnosis", "+",  "BMIq"))
#design <- formula(paste("~ ", "Diagnosis", "+ Metformin"))
#Create DESeq2 object with matrix and metadata
dds <- DESeqDataSetFromMatrix(countData = Taxonomy2,
                              colData = Metadata2,
                              design = design)


##Assess and calculate size factors using the different methods
SF<-data.frame(Ratio=sizeFactors(estimateSizeFactors(dds, type="ratio")),
               Poscounts=sizeFactors(estimateSizeFactors(dds, type="poscounts")),
               #"iterate" takes a lot of time changed to "poscounts" but kept due to the
               #following code
               Iterate=sizeFactors(estimateSizeFactors(dds, type="poscounts")),
               Relative=colSums(Taxonomy2)/mean(colSums(Taxonomy2))
               )
SF<-add_rownames(SF, var="MicrobiomeID")
#Adding total abundances
# old path path<-paste("Q:/",
#             "Projects/",
#             "LADA/",
```

```
#             "LADA_Sandra_Evelina/",
#             "LADA_JKV/",
#             "LADA_R_AfterFlow_Analysis_FinalCounts/",
#             "LADA_FinalCounts/",
#             "2019-09-03_CountAnalysis.rds", sep="")

path <- paste("J:/",
              "CBMR/",
              "SUN-CBMR-Hansen-Group/",
              "Projects/",
              "LADA/",
              "LADA_Sandra_Evelina/",
              "LADA_JKV/",
              "LADA_R_AfterFlow_Analysis_FinalCounts/",
              "LADA_FinalCounts/",
              "2019-09-03_CountAnalysis.rds", sep="")

TotalCounts<-readRDS(path)
TotalCountsProcess <-
  data.frame(MicrobiomeID=TotalCounts$DF_Save_w$ID,
             CellNorm=TotalCounts$DF_Save_w$Cells_per_g_feces_FR_corrected_mean)
TotalCountsProcess$MicrobiomeID <- paste("L", TotalCountsProcess$MicrobiomeID,
                                         sep="")
SF2<-merge(SF, TotalCountsProcess, by="MicrobiomeID")

#One value is NaN in sample 602059 assigns value as average
SF2$CellNorm[is.nan(SF2$CellNorm)]<-mean(na.omit(SF2$CellNorm))

SF2$CellNormStand<-SF2$CellNorm/mean(SF2$CellNorm) #Watch out for NaN values can assign
                                                   #average, but wants the error
SF3<-merge(Metadata2, SF2, by="MicrobiomeID")
#DESeq divides with sizeFactor (High cell count should have low sizeFactor providing
#larger counts in a sample)
#Still have to consider seq data is relative
SF3$NormRelCell<-SF3$Relative/SF3$CellNormStand
#Overview of normalization factors
aggregate(SF3[, 14:20], list(SF3$Diagnosis), mean) #Have to run heatmap chunk adding sex
```

Group.1 Ratio Poscounts Iterate Relative CellNorm CellNormStand 1 Control 1.070161 1.059005 1.059005 0.9884311 20384275367 1.0420944 2 T1D 1.105743 1.096874 1.096874 1.0263342 18731782275 0.9576149 3 T2D 1.087719 1.081155 1.081155 1.0016117 19624620327 1.0032590 4 LADA 1.093748 1.089036 1.089036 0.9984496 18940404238 0.9682802 NormRelCell 1 1.016138 2 1.210062 3 1.139338 4 1.092808

```
                                                   #to metadata
aggregate(SF3[, 14:20], list(SF3$Diagnosis), sd)
```

Group.1 Ratio Poscounts Iterate Relative CellNorm CellNormStand 1 Control 0.4597056 0.4523032 0.4523032 0.4273249 6781846681 0.3467047 2 T1D 0.4010043 0.3962703 0.3962703 0.3829215 5712095609 0.2920164 3 T2D 0.3344981 0.3296724 0.3296724 0.3105289 6146706989 0.3142348 4 LADA 0.4434083 0.4388107 0.4388107 0.4058688 5544046390 0.2834253 NormRelCell 1 0.4752640 2 0.6768389 3 0.6643237 4 0.4609629

```r
pairs(SF3[, c(14:17,19,20)], col=SF3$Diagnosis)
```

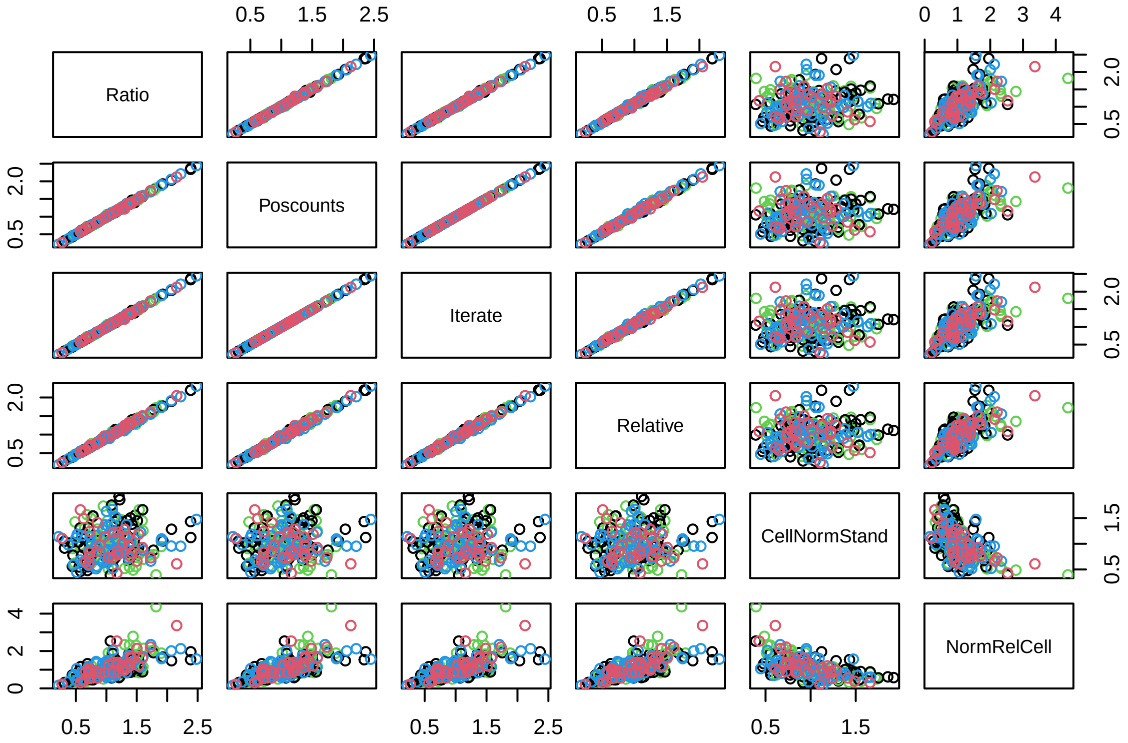


```r
pairs(SF3[, c(15,17,19,20)], col=SF3$Diagnosis)
```

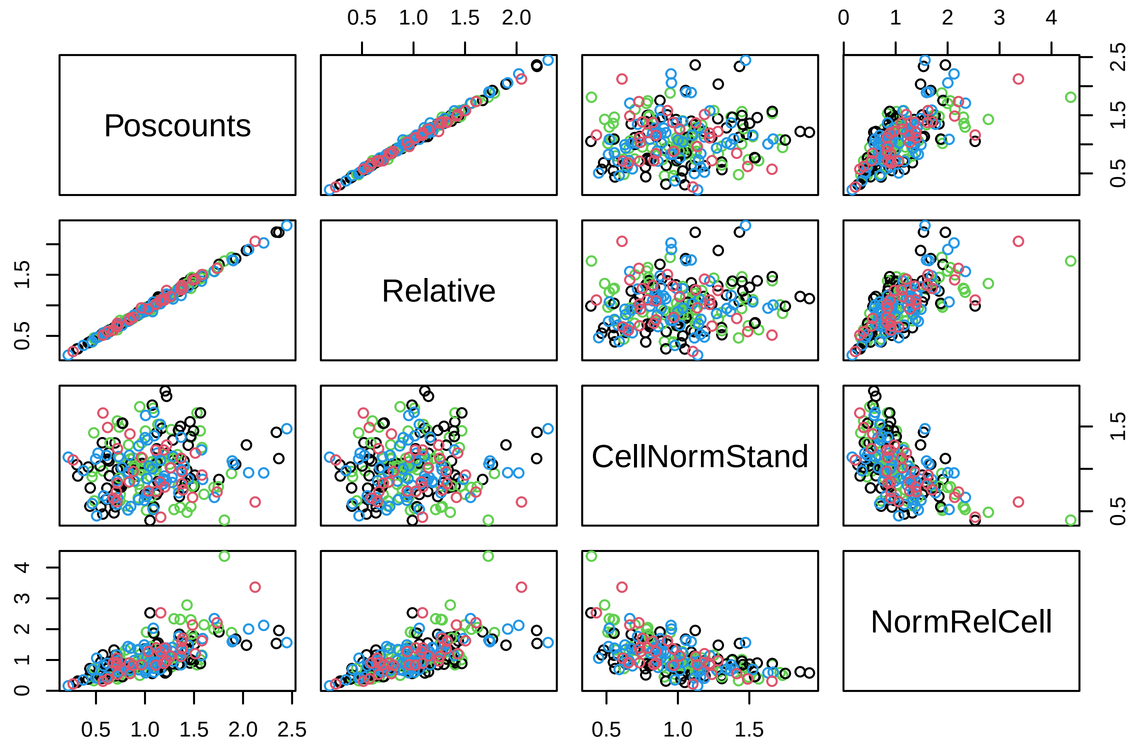


```
#I do want CellNormStand and NormRelCell to be negatively correlated due to DESeq divide
#with sizeFactor.
#It is also good that CellNormStand seems uncorrelated to the other normalization factors.

##Select size factors calculated above for normalization
dds@colData@listData$sizeFactor <- SF3[,20]

##See vignette for note on factor levels
#Filtering of the Counttable. Bare minimum is performed later. Also to reduce memory of

#the dds
dds <- dds[ rowSums(counts(dds))>(50*length(Taxonomy2)),] #Previously 0 instead of 1

#Estimate dispersions and fit the GLM
dds <- DESeq(dds)

res<-results(dds)
summary(res)
```

out of 197 with nonzero total read count adjusted p-value < 0.1 LFC > 0 (up) : 0, 0% LFC < 0 (down) : 0, 0% outliers [1] : 0, 0% low counts [2] : 0, 0% (mean count < 64) [1] see 'cooksCutoff' argument of ?results [2] see 'independentFiltering' argument of ?results

```
resultsNames(dds)
```

[1] "Intercept" "Diagnosis_T1D_vs_Control" [3] "Diagnosis_T2D_vs_Control" "Diagnosis_LADA_vs_Control"
[5] "BMIq"

```
#Threshold for FDR-adjusted p-values
padj_threshold <- 0.1

if (is.factor(colData(dds)[,"Diagnosis"]) ==  TRUE) {

#Find all two-wise combinations of levels in chosen test factor
test <- combn(levels(colData(dds)[,"Diagnosis"]), 2)
test[,3]<-c("LADA", "Control") #Want LADA first
test[,5]<-c("LADA", "T1D") #Want LADA first
test[,6]<-c("LADA", "T2D") #Want LADA first
foo = test[1,]
poo = test[2,]

#Do DESeq2 analysis on all combinations of levels and save as list
result <- vector("list",length(foo))
for (i in 1:length(foo) ) {
  for (j in 1:length(poo)) {
    result[i] = results(dds, contrast = c("Diagnosis" ,foo[i], poo[i]))
  }
}

#Produce output tables with significant taxa from each comparison
res_list <- vector("list",length(foo))
for (i in 1:length(result) ) {
  res_stat <- data.frame(result[i])
  res_stat <- res_stat[is.na(res_stat$pvalue) == FALSE,]
  stat_sig <- na.omit(res_stat[res_stat$padj < padj_threshold,])
  res_stat$gene <- rownames(res_stat)
  res_stat$g1 <- test[1,i]
  res_stat$g2 <- test[2,i]
  res_stat$compare <- paste(test[1,i], "vs", test[2,i])
  res_list[i] <- list(res_stat)
}

res_stat <- do.call('rbind', res_list)
rownames(res_stat) <- 1:nrow(res_stat)
res_stat <- res_stat[with(res_stat, order(padj, pvalue, abs(log2FoldChange))),]
stat_sig <- na.omit(res_stat[res_stat$padj < padj_threshold,])
}

if (is.numeric(colData(dds)[,"Diagnosis"]) ==  TRUE) {

res_stat <- data.frame((results(dds)))
res_stat$maxCooks <- apply(assays(dds)[["cooks"]], 1, max)
res_stat <- res_stat[is.na(res_stat$pvalue) == FALSE,]
res_stat <- res_stat[with(res_stat, order(padj, pvalue, abs(log2FoldChange))), ]
res_stat$gene <- rownames(res_stat)
max_cooks <- quantile(na.omit(res_stat$maxCooks), cooks_quantile_cutoff)
stat_sig <-
  na.omit(res_stat[res_stat$padj < padj_threshold & res_stat$maxCooks < max_cooks,])
}
```

```r
#All comparisons
write.table(res_stat, file="DESeqRes_Orgs_Func.txt", quote = F, row.names = F, sep="\t")

#Significant taxa in table
if (nrow(stat_sig) > 0) {
kable(stat_sig, row.names = F)
write.table(stat_sig, file="DESeqRes_SigOrgs_Func.txt", quote = F, row.names = F, sep="\t")
} else {
  print("No significant taxa were found.")
}
print("Significant Wald")
```

[1] "Significant Wald"

```r
kable(stat_sig)
```

	baseMean	log2FoldChange	lfcSE	stat	pvalue	padj	gene	g1	g2	compare
523	1047.12814	2.4393983	0.3547467	6.875945	0.0000000	0.0000001	Linoleic acid metabolism [PATH:ko00591]	LADA	Control	LADA vs Control
555	2086.54612	1.9324055	0.3041946	6.352530	0.0000000	0.0000000	Geraniol degradation [PATH:ko00281]	LADA	Control	LADA vs Control
515	1471.38976	1.9402023	0.3055370	6.350139	0.0000000	0.0000000	alpha-Linolenic acid metabolism [PATH:ko00592]	LADA	Control	LADA vs Control
321	495.64764	-2.0441804	0.3162538	-6.463734	0.0000000	0.0000000	Ether lipid metabolism [PATH:ko00565]	Control	T2D	Control vs T2D
326	1047.12814	-2.2027510	0.3587623	-6.139861	0.0000000	0.0000001	Linoleic acid metabolism [PATH:ko00591]	Control	T2D	Control vs T2D
318	1471.38976	-1.8970076	0.3089962	-6.139260	0.0000000	0.0000001	alpha-Linolenic acid metabolism [PATH:ko00592]	Control	T2D	Control vs T2D
358	2086.54612	-1.8493987	0.3076410	-6.011548	0.0000000	0.0000001	Geraniol degradation [PATH:ko00281]	Control	T2D	Control vs T2D
518	495.64764	1.8501672	0.3127254	5.916267	0.0000000	0.0000002	Ether lipid metabolism [PATH:ko00565]	LADA	Control	LADA vs Control
580	569.52112	2.1868309	0.3714235	5.887702	0.0000000	0.0000002	Toluene degradation [PATH:ko00623]	LADA	Control	LADA vs Control

	baseMean	log2FoldChange	lfcSE	stat	pvalue	padj	gene	g1	g2	compare
574	1015.07229	1.9352606	0.3372653	5.738072	0.0000000	0.0000003	Ethylbenzene degradation [PATH:ko00642]	LADA	Control	LADA vs Control
859	524.40214	2.6950453	0.4570651	5.896461	0.0000000	0.0000007	Betalain biosynthesis [PATH:ko00965]	LADA	T1D	LADA vs T1D
917	1047.12814	2.5390140	0.4473623	5.675521	0.0000000	0.0000014	Linoleic acid metabolism [PATH:ko00591]	LADA	T1D	LADA vs T1D
662	524.40214	-2.6143293	0.4598840	-5.684759	0.0000000	0.0000026	Betalain biosynthesis [PATH:ko00965]	T1D	T2D	T1D vs T2D
377	1015.07229	-1.8248998	0.3410848	-5.350282	0.0000001	0.0000033	Ethylbenzene degradation [PATH:ko00642]	Control	T2D	Control vs T2D
383	569.52117	-2.0013156	0.3756481	-5.327635	0.0000001	0.0000033	Toluene degradation [PATH:ko00623]	Control	T2D	Control vs T2D
720	1047.12814	-2.4023667	0.4501371	-5.336967	0.0000001	0.0000093	Linoleic acid metabolism [PATH:ko00591]	T1D	T2D	T1D vs T2D
572	5377.28344	1.3369416	0.2626654	5.089910	0.0000004	0.0000101	Drug metabolism - cytochrome P450 [PATH:ko00982]	LADA	Control	LADA vs Control
576	5353.44294	1.3268594	0.2624795	5.055098	0.0000004	0.0000106	Metabolism of xenobiotics by cytochrome P450 [PATH:ko00980]	LADA	Control	LADA vs Control
715	495.64764	-2.0572152	0.3968193	-5.184262	0.0000002	0.0000142	Ether lipid metabolism [PATH:ko00565]	T1D	T2D	T1D vs T2D
974	569.52117	2.2889539	0.4683860	4.886897	0.0000010	0.0000673	Toluene degradation [PATH:ko00623]	LADA	T1D	LADA vs T1D
465	524.40214	1.6799277	0.3621542	4.638737	0.0000035	0.0000767	Betalain biosynthesis [PATH:ko00965]	LADA	Control	LADA vs Control
912	495.64764	1.8632020	0.3943804	4.724372	0.0000023	0.0001137	Ether lipid metabolism [PATH:ko00565]	LADA	T1D	LADA vs T1D
949	2086.54612	1.7944439	0.3835254	4.678818	0.0000029	0.0001137	Geraniol degradation [PATH:ko00281]	LADA	T1D	LADA vs T1D
375	5377.28344	-1.1975369	0.2656421	-4.508084	0.0000065	0.0001841	Drug metabolism - cytochrome P450 [PATH:ko00982]	Control	T2D	Control vs T2D
379	5353.44294	-1.1869504	0.2654546	-4.471388	0.0000078	0.0001914	Metabolism of xenobiotics by cytochrome P450 [PATH:ko00980]	Control	T2D	Control vs T2D

	baseMean	log2FoldChange	lfcSE	stat	pvalue	padj	gene	g1	g2	compare
268	524.40214	-1.5992117	0.3662559	-4.366378	0.0000126	0.0002765	Betalain biosynthesis [PATH:ko00965]	Control	T2D	Control vs T2D
909	1471.38976	1.7044940	0.3852124	4.424810	0.0000097	0.0003169	alpha-Linolenic acid metabolism [PATH:ko00592]	LADA	T1D	LADA vs T1D
777	569.52117	-2.1034386	0.4713141	-4.462923	0.0000081	0.0003631	Toluene degradation [PATH:ko00623]	T1D	T2D	T1D vs T2D
752	2086.54612	-1.7114371	0.3859112	-4.434795	0.0000092	0.0003631	Geraniol degradation [PATH:ko00281]	T1D	T2D	T1D vs T2D
378	550.12354	-1.7218263	0.4096114	-4.203561	0.0000263	0.0005176	Fluorobenzoate degradation [PATH:ko00364]	Control	T2D	Control vs T2D
900	247.27768	2.1059149	0.4916842	4.283076	0.0000184	0.0005188	Glycosphingolipid biosynthesis - lacto and neolacto series [PATH:ko00601]	LADA	T1D	LADA vs T1D
712	1471.38976	-1.6612993	0.3876074	-4.286036	0.0000182	0.0005972	alpha-Linolenic acid metabolism [PATH:ko00592]	T1D	T2D	T1D vs T2D
772	550.12354	-2.1173231	0.5139542	-4.119673	0.0000379	0.0010678	Fluorobenzoate degradation [PATH:ko00364]	T1D	T2D	T1D vs T2D
554	2324.42828	1.3695622	0.3404379	4.022940	0.0000575	0.0011323	Carotenoid biosynthesis [PATH:ko00906]	LADA	Control	LADA vs Control
575	550.12354	1.5484008	0.4050238	3.823006	0.0001318	0.0023610	Fluorobenzoate degradation [PATH:ko00364]	LADA	Control	LADA vs Control
969	550.12354	1.9438976	0.5107743	3.805782	0.0001414	0.0034809	Fluorobenzoate degradation [PATH:ko00364]	LADA	T1D	LADA vs T1D
968	1015.07229	1.5983085	0.4252040	3.758921	0.0001706	0.0037353	Ethylbenzene degradation [PATH:ko00642]	LADA	T1D	LADA vs T1D
966	5377.28314	1.2173639	0.3311548	3.676118	0.0002368	0.0046652	Drug metabolism - cytochrome P450 [PATH:ko00982]	LADA	T1D	LADA vs T1D
970	5353.44294	1.2080161	0.3309230	3.650466	0.0002618	0.0046880	Metabolism of xenobiotics by cytochrome P450 [PATH:ko00980]	LADA	T1D	LADA vs T1D
703	247.27768	-1.7797907	0.4947889	-3.597071	0.0003218	0.0079248	Glycosphingolipid biosynthesis - lacto and neolacto series [PATH:ko00601]	T1D	T2D	T1D vs T2D
771	1015.07229	-1.4879477	0.4278472	-3.477755	0.0005056	0.0110677	Ethylbenzene degradation [PATH:ko00642]	T1D	T2D	T1D vs T2D

	baseMean	log2FoldChange	lfcSE	stat	pvalue	padj	gene	g1	g2	compare
537	4324.772[illegible]	0.7193709	0.2148483	3.348270	0.0008102	0.013349[illegible]	Retinol metabolism [PATH:ko00830]	LADA	Control	LADA vs Control
769	5377.28344	-1.0779593	0.3332169	-3.235008	0.0012164	0.023962[illegible]	Drug metabolism - cytochrome P450 [PATH:ko00982]	T1D	T2D	T1D vs T2D
773	5353.44294	-1.0681071	0.3329817	-3.207705	0.0013380	0.023962[illegible]	Metabolism of xenobiotics by cytochrome P450 [PATH:ko00980]	T1D	T2D	T1D vs T2D
9	161375.1[illegible]	0.6889689	0.1847832	3.728566	0.0001926	0.035944[illegible]	Bacterial chemotaxis [PATH:ko02030]	Control	T1D	Control vs T1D
10	192929.9[illegible]	0.7072483	0.1984293	3.564237	0.0003649	0.035944[illegible]	Flagellar assembly [PATH:ko02040]	Control	T1D	Control vs T1D
357	2324.42828	-1.0629809	0.3442985	-3.087381	0.0020193	0.036163[illegible]	Carotenoid biosynthesis [PATH:ko00906]	Control	T2D	Control vs T2D
604	82196.16111	-0.4007366	0.1388035	-2.887080	0.0038884	0.063833[illegible]	Biofilm formation - Pseudomonas aeruginosa [PATH:ko02025]	T1D	T2D	T1D vs T2D
600	161375.11235	-0.5426158	0.1925753	-2.817680	0.004837[illegible]	0.073302[illegible]	Bacterial chemotaxis [PATH:ko02030]	T1D	T2D	T1D vs T2D
842	33974.13943	-0.6102019	0.2165670	-2.817613	0.0048382	0.079427[illegible]	Ribosome biogenesis in eukaryotes [PATH:ko03008]	LADA	T1D	LADA vs T1D
586	1305.3013[illegible]	1.0439611	0.3829467	2.726120	0.0064082	0.090351[illegible]	Biosynthesis of terpenoids and steroids	LADA	Control	LADA vs Control
516	3111.3600[illegible]	0.5628725	0.2065227	2.725470	0.0064209	0.090351[illegible]	Arachidonic acid metabolism [PATH:ko00590]	LADA	Control	LADA vs Control
402	67.03765	0.9359374	0.3469992	2.697220	0.0069920	0.09182[illegible]	p53 signaling pathway [PATH:ko04115]	LADA	Control	LADA vs Control

```
kable(stat_sig[,c(5:7,10)])
```

	pvalue	padj	gene	compare
523	0.0000000	0.0000000	Linoleic acid metabolism [PATH:ko00591]	LADA vs Control
555	0.0000000	0.0000000	Geraniol degradation [PATH:ko00281]	LADA vs Control
515	0.0000000	0.0000000	alpha-Linolenic acid metabolism [PATH:ko00592]	LADA vs Control

	pvalue	padj	gene	compare
321	0.0000000	0.0000000	Ether lipid metabolism [PATH:ko00565]	Control vs T2D
326	0.0000000	0.0000001	Linoleic acid metabolism [PATH:ko00591]	Control vs T2D
318	0.0000000	0.0000001	alpha-Linolenic acid metabolism [PATH:ko00592]	Control vs T2D
358	0.0000000	0.0000001	Geraniol degradation [PATH:ko00281]	Control vs T2D
518	0.0000000	0.0000002	Ether lipid metabolism [PATH:ko00565]	LADA vs Control
580	0.0000000	0.0000002	Toluene degradation [PATH:ko00623]	LADA vs Control
574	0.0000000	0.0000003	Ethylbenzene degradation [PATH:ko00642]	LADA vs Control
859	0.0000000	0.0000007	Betalain biosynthesis [PATH:ko00965]	LADA vs T1D
917	0.0000000	0.0000014	Linoleic acid metabolism [PATH:ko00591]	LADA vs T1D
662	0.0000000	0.0000026	Betalain biosynthesis [PATH:ko00965]	T1D vs T2D
377	0.0000001	0.0000033	Ethylbenzene degradation [PATH:ko00642]	Control vs T2D
383	0.0000001	0.0000033	Toluene degradation [PATH:ko00623]	Control vs T2D
720	0.0000001	0.0000093	Linoleic acid metabolism [PATH:ko00591]	T1D vs T2D
572	0.0000004	0.0000101	Drug metabolism - cytochrome P450 [PATH:ko00982]	LADA vs Control
576	0.0000004	0.0000106	Metabolism of xenobiotics by cytochrome P450 [PATH:ko00980]	LADA vs Control
715	0.0000002	0.0000142	Ether lipid metabolism [PATH:ko00565]	T1D vs T2D
974	0.0000010	0.0000673	Toluene degradation [PATH:ko00623]	LADA vs T1D
465	0.0000035	0.0000767	Betalain biosynthesis [PATH:ko00965]	LADA vs Control
912	0.0000023	0.0001137	Ether lipid metabolism [PATH:ko00565]	LADA vs T1D
949	0.0000029	0.0001137	Geraniol degradation [PATH:ko00281]	LADA vs T1D
375	0.0000065	0.0001841	Drug metabolism - cytochrome P450 [PATH:ko00982]	Control vs T2D
379	0.0000078	0.0001914	Metabolism of xenobiotics by cytochrome P450 [PATH:ko00980]	Control vs T2D
268	0.0000126	0.0002765	Betalain biosynthesis [PATH:ko00965]	Control vs T2D
909	0.0000097	0.0003169	alpha-Linolenic acid metabolism [PATH:ko00592]	LADA vs T1D
777	0.0000081	0.0003631	Toluene degradation [PATH:ko00623]	T1D vs T2D
752	0.0000092	0.0003631	Geraniol degradation [PATH:ko00281]	T1D vs T2D
378	0.0000263	0.0005176	Fluorobenzoate degradation [PATH:ko00364]	Control vs T2D
900	0.0000184	0.0005188	Glycosphingolipid biosynthesis - lacto and neolacto series [PATH:ko00601]	LADA vs T1D
712	0.0000182	0.0005972	alpha-Linolenic acid metabolism [PATH:ko00592]	T1D vs T2D

	pvalue	padj	gene	compare
772	0.0000379	0.0010678	Fluorobenzoate degradation [PATH:ko00364]	T1D vs T2D
554	0.0000575	0.0011323	Carotenoid biosynthesis [PATH:ko00906]	LADA vs Control
575	0.0001318	0.0023610	Fluorobenzoate degradation [PATH:ko00364]	LADA vs Control
969	0.0001414	0.0034809	Fluorobenzoate degradation [PATH:ko00364]	LADA vs T1D
968	0.0001706	0.0037353	Ethylbenzene degradation [PATH:ko00642]	LADA vs T1D
966	0.0002368	0.0046652	Drug metabolism - cytochrome P450 [PATH:ko00982]	LADA vs T1D
970	0.0002618	0.0046880	Metabolism of xenobiotics by cytochrome P450 [PATH:ko00980]	LADA vs T1D
703	0.0003218	0.0079248	Glycosphingolipid biosynthesis - lacto and neolacto series [PATH:ko00601]	T1D vs T2D
771	0.0005056	0.0110677	Ethylbenzene degradation [PATH:ko00642]	T1D vs T2D
537	0.0008132	0.0133495	Retinol metabolism [PATH:ko00830]	LADA vs Control
769	0.0012164	0.0239621	Drug metabolism - cytochrome P450 [PATH:ko00982]	T1D vs T2D
773	0.0013380	0.0239621	Metabolism of xenobiotics by cytochrome P450 [PATH:ko00980]	T1D vs T2D
9	0.0001926	0.0359443	Bacterial chemotaxis [PATH:ko02030]	Control vs T1D
10	0.0003649	0.0359443	Flagellar assembly [PATH:ko02040]	Control vs T1D
357	0.0020193	0.0361635	Carotenoid biosynthesis [PATH:ko00906]	Control vs T2D
604	0.0038884	0.0638339	Biofilm formation - Pseudomonas aeruginosa [PATH:ko02025]	T1D vs T2D
600	0.0048372	0.0733021	Bacterial chemotaxis [PATH:ko02030]	T1D vs T2D
842	0.0048382	0.0794274	Ribosome biogenesis in eukaryotes [PATH:ko03008]	LADA vs T1D
586	0.0064082	0.0903513	Biosynthesis of terpenoids and steroids	LADA vs Control
516	0.0064209	0.0903513	Arachidonic acid metabolism [PATH:ko00590]	LADA vs Control
402	0.0069920	0.0918277	p53 signaling pathway [PATH:ko04115]	LADA vs Control

```
##Venn diagram comparing LADA to other groups
sig_LADA <- subset(stat_sig, g1=="LADA")
sig_LADA_T1D <- subset(sig_LADA, g2=="T1D") %>% dplyr::select(one_of("gene")) %>%
  pull(gene)
sig_LADA_T2D <- subset(sig_LADA, g2=="T2D") %>% dplyr::select(one_of("gene")) %>%
  pull(gene)
sig_LADA_Control <- subset(sig_LADA, g2=="Control") %>% dplyr::select(one_of("gene")) %>%
  pull(gene)

#Colors
myCol <- c( "#1F78B4", "#33A02C", "#666666")
#
temp <- venn.diagram(list(T1D = sig_LADA_T1D,
                          T2D = sig_LADA_T2D,
```

```
                  Control = sig_LADA_Control), filename = NULL,

              # Circles
              lwd = 2,
              lty = 'blank',
              fill = myCol,

              # Numbers
              cex = 3,
              fontface = "bold",
              fontfamily = "sans",

              # Set names
              cat.cex = 3,
              cat.fontface = "bold",
              cat.default.pos = "outer",
              cat.pos = c(0, 0, 0),
              cat.dist = c(0.085, 0.085, 0.024),
              cat.fontfamily = "sans",
              rotation = 1)

grid.arrange(gTree(children=temp))
```

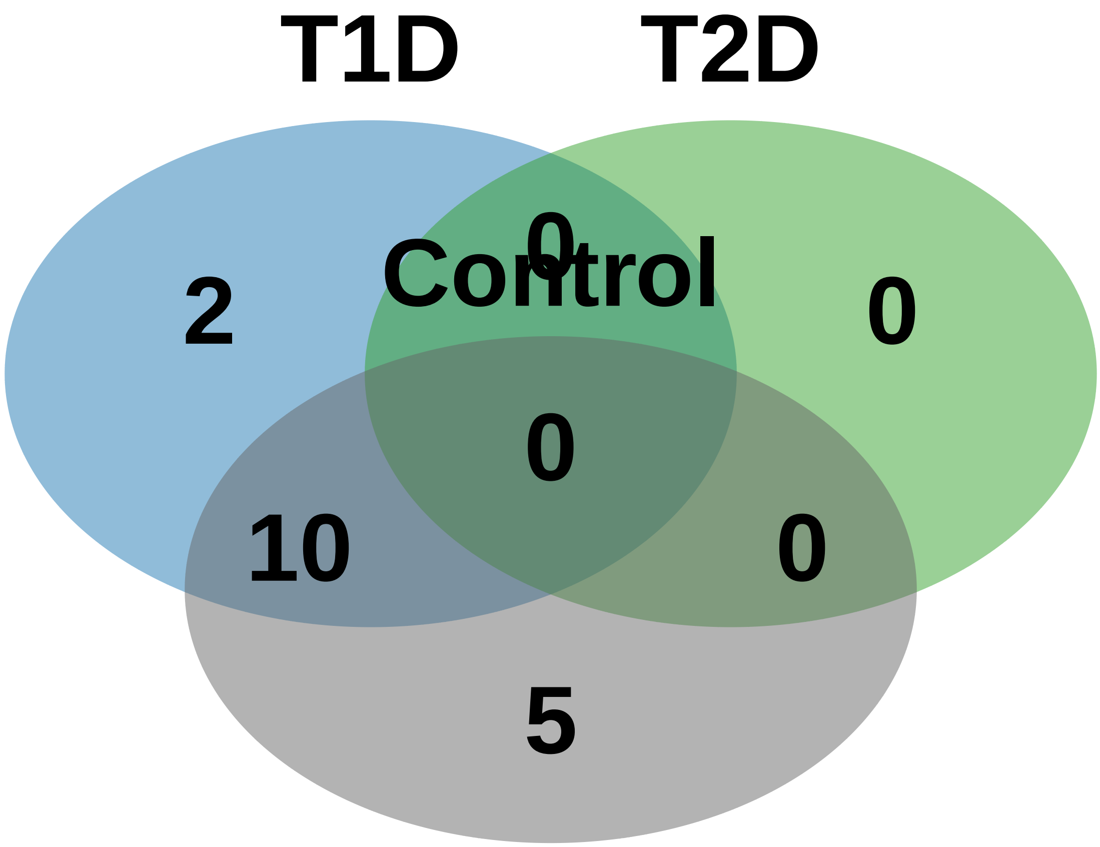

```r
Fig2List[[ "VennLADA" ]] <-
  gTree(children=temp, top="N differential abundant relative to LADA")

#Genera LADA that are significantly different from all other groups
intersect(intersect(sig_LADA_T1D,sig_LADA_T2D),sig_LADA_Control)
```

character(0)

```r
##Venn diagram comparing T1D to other groups
sig_T1D <- subset(stat_sig, g1=="T1D" | g2=="T1D")
sig_T1D_LADA <- subset(sig_T1D, g1=="LADA") %>% dplyr::select(one_of("gene")) %>%
  pull(gene)
sig_T1D_T2D <- subset(sig_T1D, g2=="T2D") %>% dplyr::select(one_of("gene")) %>%
  pull(gene)
sig_T1D_Control <- subset(sig_T1D, g1=="Control") %>% dplyr::select(one_of("gene")) %>%
  pull(gene)
#Colors
myCol <- c( "#E31A1C", "#33A02C", "#666666")
#
temp <- venn.diagram(list(LADA = sig_T1D_LADA,
                          T2D = sig_T1D_T2D,
                          Control = sig_T1D_Control), filename = NULL,

                     # Circles
                     lwd = 2,
                     lty = 'blank',
                     fill = myCol,

                     # Numbers
                     cex = 2,
                     fontface = "bold",
                     fontfamily = "sans",

                     # Set names
                     cat.cex = 2,
                     cat.fontface = "bold",
                     cat.default.pos = "outer",
                     cat.pos = c(0, 0, 0),
                     cat.dist = c(0.055, 0.055, 0.024),
                     cat.fontfamily = "sans",
                     rotation = 1)

grid.arrange(gTree(children=temp))
```

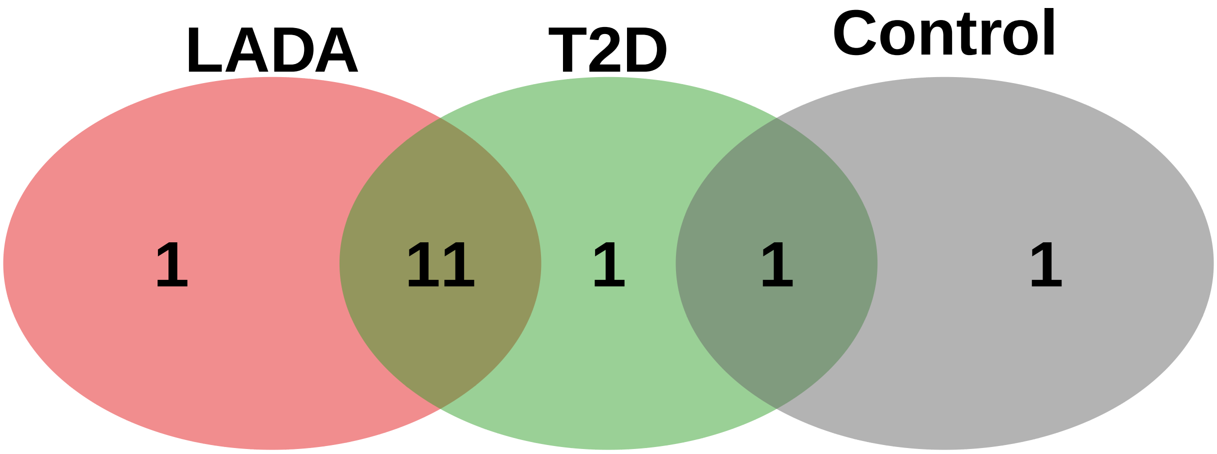


```
Fig2List[[ "VennT1D" ]] <-
  gTree(children=temp, top="N differential abundant relative to T1D")

#Genera T1D that are significantly different from all other groups
intersect(intersect(sig_T1D_LADA,sig_T1D_T2D),sig_T1D_Control)
```

character(0)

```
##Venn diagram comparing T2D to other groups
sig_T2D <- subset(stat_sig, g1=="T2D" | g2=="T2D")
sig_T2D_LADA <- subset(sig_T2D, g1=="LADA") %>% dplyr::select(one_of("gene")) %>%
  pull(gene)
sig_T2D_T1D <- subset(sig_T2D, g1=="T1D") %>% dplyr::select(one_of("gene")) %>%
  pull(gene)
sig_T2D_Control <- subset(sig_T2D, g1=="Control") %>% dplyr::select(one_of("gene")) %>%
  pull(gene)
#Colors
myCol <- c( "#E31A1C", "#1F78B4", "#666666")
#
temp <- venn.diagram(list(LADA = sig_T2D_LADA,
                          T1D = sig_T2D_T1D,
                          Control = sig_T2D_Control), filename = NULL,

                      # Circles
                      lwd = 2,
```

```
                      lty = 'blank',
                      fill = myCol,

                      # Numbers
                      cex = 2,
                      fontface = "bold",
                      fontfamily = "sans",

                      # Set names
                      cat.cex = 2,
                      cat.fontface = "bold",
                      cat.default.pos = "outer",
                      cat.pos = c(0, 0, 0),
                      cat.dist = c(0.055, 0.055, 0.024),
                      cat.fontfamily = "sans",
                      rotation = 1)

grid.arrange(gTree(children=temp))
```

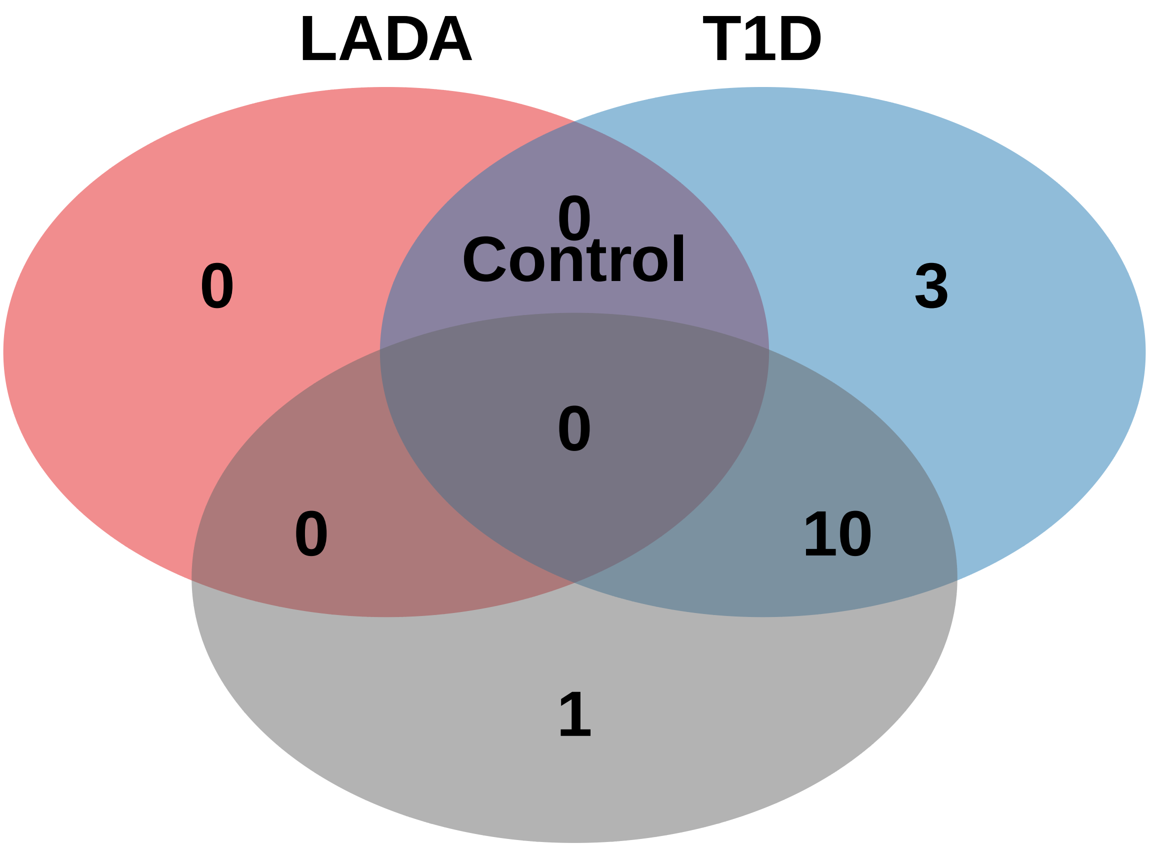


```
Fig2List[[ "VennT2D" ]] <-
  gTree(children=temp, top="N differential abundant relative to T2D")

#Genera T2D that are significantly different from all other groups
intersect(intersect(sig_T2D_LADA,sig_T2D_T1D),sig_T2D_Control)
```

character(0)

```
##Venn diagram comparing controls to other groups
sig_Control <- subset(stat_sig, g1=="Control" | g2=="Control")
sig_Control_LADA <- subset(sig_Control, g1=="LADA") %>% dplyr::select(one_of("gene")) %>%
  pull(gene)
sig_Control_T1D <- subset(sig_Control, g2=="T1D") %>% dplyr::select(one_of("gene")) %>%
  pull(gene)
sig_Control_T2D <- subset(sig_Control, g2=="T2D") %>% dplyr::select(one_of("gene")) %>%
  pull(gene)
#Colors
myCol <- c( "#E31A1C", "#1F78B4", "#33A02C")
#
temp <- venn.diagram(list(LADA = sig_Control_LADA,
                          T1D = sig_Control_T1D,
                          T2D = sig_Control_T2D), filename = NULL,

                     # Circles
                     lwd = 2,
                     lty = 'blank',
                     fill = myCol,

                     # Numbers
                     cex = 3,
                     fontface = "bold",
                     fontfamily = "sans",

                     # Set names
                     cat.cex = 3,
                     cat.fontface = "bold",
                     cat.default.pos = "outer",
                     cat.pos = c(0, 0, 0),
                     cat.dist = c(0.055, 0.055, 0.024),
                     cat.fontfamily = "sans",
                     rotation = 1)

grid.arrange(gTree(children=temp))
```

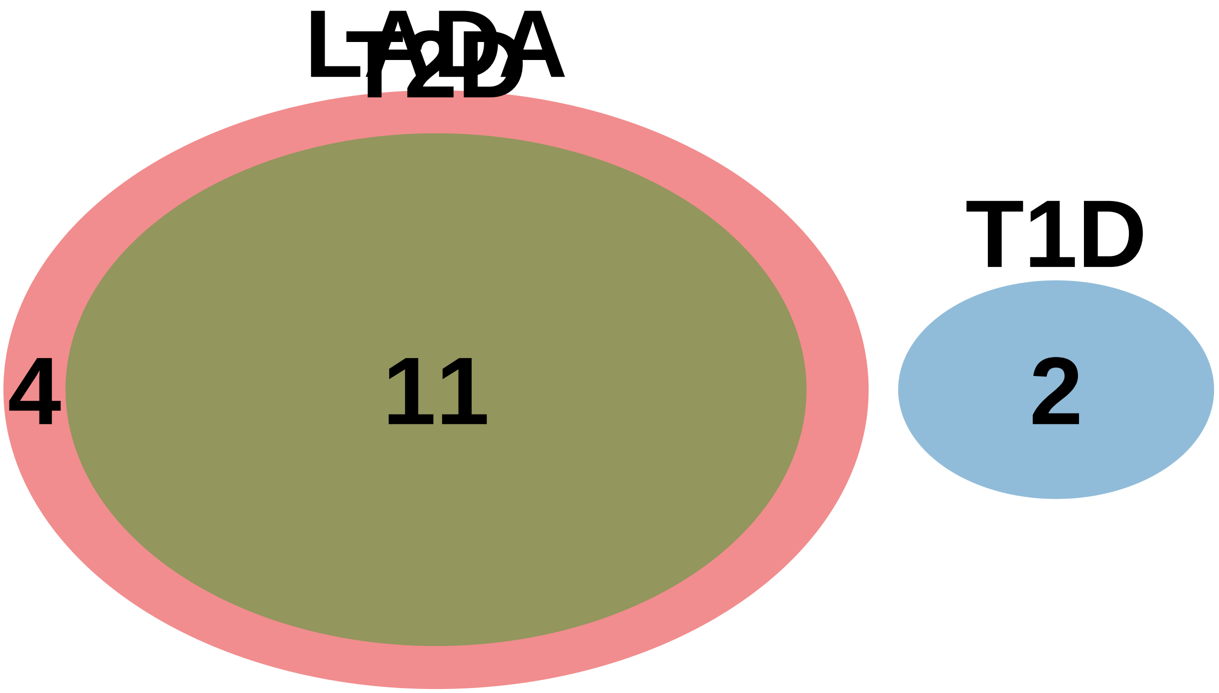


```
Fig2List[[ "VennControls" ]] <-
  gTree(children=temp, top="N differential abundant relative to controls")

#Genera Controls that are significantly different from all other groups
intersect(intersect(sig_T2D_LADA,sig_T2D_T1D),sig_T2D_Control)
```

character(0)

```
##Create boxplots / violin plots
#Normalize to get cells pr. gram feces
if (setequal(colnames(Taxonomy2), SF3$MicrobiomeID)==FALSE) {
  stop("Metadata and Taxonomy out of sync")
}

#Total sum scaling (Use relative abundances)
Taxonomy3<-sweep(Taxonomy2, 2, colSums(Taxonomy), FUN="/")
#Obtain values as cells pr. gram feces
Taxonomy3<-sweep(Taxonomy3, 2, SF3$CellNorm, FUN="*")
#Make to micro gram
Taxonomy3<-Taxonomy3/10^6


##Select organisms
SelOrgs<-unique(stat_sig$gene)
```

```r
tTaxSelect<-dplyr::select(as.data.frame(t(Taxonomy3)), one_of(c(SelOrgs)))
#Reducing the number because duplicates are removed
tTaxSelect<-add_rownames(tTaxSelect, "MicrobiomeID")
Plotting<-merge(SF3, tTaxSelect, by="MicrobiomeID")

##Might have issues with special characters, so this chunk might be needed
SelOrgs<-str_replace_all(SelOrgs, " ","_")
SelOrgs<-str_replace_all(SelOrgs, "-","_")
SelOrgs<-str_replace_all(SelOrgs, "/","_")
SelOrgs<-gsub("\\[|\\]", "", SelOrgs)
SelOrgs<-str_replace_all(SelOrgs, ":","_")
colnames(Plotting) <- str_replace_all(colnames(Plotting)," ","_")
colnames(Plotting) <- str_replace_all(colnames(Plotting),"-","_")
colnames(Plotting) <- str_replace_all(colnames(Plotting),"/","_")
colnames(Plotting) <- gsub("\\[|\\]", "", colnames(Plotting))
colnames(Plotting) <- str_replace_all(colnames(Plotting),":","_")

#Plot logs on y axis
Plotting2 <- bind_cols(Plotting[,1:16], log10(Plotting[,17:ncol(Plotting)]+1))
#See end of chunk for boxplots removed the violinplots, because of redundancy


##Create vulcano plots
#Cut offs Benjamini-Hochberg method to add to vulcano plot
#Don't know how to get exact so it is a cut-off corresponding to the BH method
BHAll<-aggregate(stat_sig[, 5:6], list(stat_sig$compare), max)
row.names(BHAll)<-BHAll$Group.1
BHLadaCon <- BHAll[which(rownames(BHAll)=="LADA vs Control"),
                   which(colnames(BHAll)=="pvalue")]
BHLadaT1D <- BHAll[which(rownames(BHAll)=="LADA vs T1D"),
                   which(colnames(BHAll)=="pvalue")]
BHLadaT2D <- BHAll[which(rownames(BHAll)=="LADA vs T2D"),
                   which(colnames(BHAll)=="pvalue")]


#Vulcano plot
res_stat$minuslog10<--log(res_stat$pvalue)
#
#range(res_stat$minuslog10)
#range(res_stat$log2FoldChange)

names(res_stat)[names(res_stat) == 'gene'] <- 'Genus'
Feature2<-merge(res_stat, Feature, by="Genus")

#Create column org grouping for colouring
Feature2$Level_1 <- ifelse(Feature2$Level_1=="Environmental Information Processing",
                           "Environmental Information Processing",
          ifelse(Feature2$Level_1=="Cellular Processes", "Cellular Processes",
          ifelse(Feature2$Level_1=="Metabolism", "Metabolism",
          ifelse(Feature2$Level_1=="Genetic Information Processing", "Genetic Information Processing",
          ifelse(Feature2$Level_1=="Unclassified metabolism", "Unclassified metabolism",
                 "Other")))))
#Order Level_1 for plotting
```

```
Feature2$Level_1 <- factor(Feature2$Level_1, levels=c("Cellular Processes", "Genetic Information Process
                                                      "Metabolism", "Environmental Information Processing",
                                                      "Unclassified metabolism", "Other"))


##Summary significant orgs
#aggregate(Plotting[, 17:ncol(Plotting)], list(Plotting$Diagnosis), mean)
#aggregate(Plotting[, 17:ncol(Plotting)], list(Plotting$Diagnosis), sd)
zeroes <- function(x){
  sum(x == 0)
}
#aggregate(Plotting[, 17:ncol(Plotting)], list(Plotting$Diagnosis), zeroes)
zerocounts <- aggregate(Plotting[, 17:ncol(Plotting)], list(Plotting$Diagnosis), zeroes)
#bind_cols(zerocounts[,1], zerocounts[,2:ncol(zerocounts)]/c(70,30,70,70)*100)


#Add prevalence to plotting
prevalence<-data.frame((240-apply(Taxonomy, 1, zeroes))/240*100)
colnames(prevalence) <- c("Prevalence")
prevalence<-add_rownames(prevalence, var = "Genus")
Feature2 <- merge(Feature2, prevalence, by="Genus")


#Define boundaries
#Always run first without these lines to get indication of very low and high
#log2FoldChange and pvalue
Featurein <- filter(Feature2, -3<log2FoldChange & log2FoldChange<3 & 10>minuslog10)
Featureout <- filter(Feature2, -3>log2FoldChange | 3<log2FoldChange | 10<minuslog10)
Featureout$log2FoldChange[Featureout$log2FoldChange > 3] <- 3
Featureout$log2FoldChange[Featureout$log2FoldChange < (-3)] <- -3
Featureout$minuslog10[Featureout$minuslog10 > 10] <- 10


#Add text to vulcano plots
p1<-ggplot() +
  geom_point(data=Featurein[which(Featurein$Level_1=="Cellular Processes" &
                                    Featurein$compare=="LADA vs T1D"), ],
             aes(x=log2FoldChange, y=minuslog10, group=Genus), color="#A6CEE3", shape=16,
             size=Featurein[which(Featurein$Level_1=="Cellular Processes" &
                                    Featurein$compare=="LADA vs T1D"), ]
             [,ncol(Featurein)]/40+0.5) +
  #did not add jitter to the first on purpose. Sizes are scaled from percent
  #to the range 0.5-3
  geom_point(data=Featureout[which(Featureout$Level_1=="Cellular Processes" &
                                     Featureout$compare=="LADA vs T1D"), ],
             aes(x=log2FoldChange, y=minuslog10, group=Genus), color="#A6CEE3", shape=18,
             size=Featureout[which(Featureout$Level_1=="Cellular Processes" &
                                     Featureout$compare=="LADA vs T1D"), ]
             [,ncol(Featureout)]/40+0.5) +
  geom_point(data=Featurein[which(Featurein$Level_1=="Genetic Information Processing" &
                                    Featurein$compare=="LADA vs T1D"), ],
             aes(x=log2FoldChange, y=minuslog10, group=Genus), color="#FB9A99", shape=16,
             size=Featurein[which(Featurein$Level_1=="Genetic Information Processing" &
```

```
                                    Featurein$compare=="LADA vs T1D"), ]
           [,ncol(Featurein)]/40+0.5) +
geom_jitter(data=Featureout[which(Featureout$Level_1=="Genetic Information Processing" &
                                    Featureout$compare=="LADA vs T1D"), ],
           aes(x=log2FoldChange, y=minuslog10, group=Genus), color="#FB9A99", shape=18,
           size=Featureout[which(Featureout$Level_1=="Genetic Information Processing" &
                                    Featureout$compare=="LADA vs T1D"), ]
           [,ncol(Featureout)]/40+0.5, width = 0.1, height = 0.1) +
geom_point(data=Featurein[which(Featurein$Level_1=="Metabolism" &
                                    Featurein$compare=="LADA vs T1D"), ],
           aes(x=log2FoldChange, y=minuslog10, group=Genus), color="#FDBF6F", shape=16,
           size=Featurein[which(Featurein$Level_1=="Metabolism" &
                                    Featurein$compare=="LADA vs T1D"), ]
           [,ncol(Featurein)]/40+0.5) +
geom_jitter(data=Featureout[which(Featureout$Level_1=="Metabolism" &
                                    Featureout$compare=="LADA vs T1D"), ],
           aes(x=log2FoldChange, y=minuslog10, group=Genus), color="#FDBF6F", shape=18,
           size=Featureout[which(Featureout$Level_1=="Metabolism" &
                                    Featureout$compare=="LADA vs T1D"), ]
           [,ncol(Featureout)]/40+0.5, width = 0.1, height = 0.1) +
geom_point(data=Featurein[which(Featurein$Level_1=="Environmental Information Processing" &
                                    Featurein$compare=="LADA vs T1D"), ],
           aes(x=log2FoldChange, y=minuslog10, group=Genus),
           color="#B2DF8A", shape=16, alpha=0.75,
           size=Featurein[which(Featurein$Level_1=="Environmental Information Processing" &
                                    Featurein$compare=="LADA vs T1D"), ]
           [,ncol(Featurein)]/40+0.5) +
geom_jitter(data=Featureout[which(Featureout$Level_1=="Environmental Information Processing" &
                                    Featureout$compare=="LADA vs T1D"), ],
           aes(x=log2FoldChange, y=minuslog10, group=Genus),
           color="#B2DF8A", shape=18,
           size=Featureout[which(Featureout$Level_1=="Environmental Information Processing" &
                                    Featureout$compare=="LADA vs T1D"), ]
           [,ncol(Featureout)]/40+0.5, width = 0.1, height = 0.1) +
geom_point(data=Featurein[which(Featurein$Level_1=="Unclassified metabolism" &
                                    Featurein$compare=="LADA vs T1D"), ],
           aes(x=log2FoldChange, y=minuslog10, group=Genus), color="#CAB2D6", shape=16,
           size=Featurein[which(Featurein$Level_1=="Unclassified metabolism" &
                                    Featurein$compare=="LADA vs T1D"), ]
           [,ncol(Featurein)]/40+0.5) +
geom_jitter(data=Featureout[which(Featureout$Level_1=="Unclassified metabolism" &
                                    Featureout$compare=="LADA vs T1D"), ],
           aes(x=log2FoldChange, y=minuslog10, group=Genus), color="#CAB2D6", shape=18,
           size=Featureout[which(Featureout$Level_1=="Unclassified metabolism" &
                                    Featureout$compare=="LADA vs T1D"), ]
           [,ncol(Featureout)]/40+0.5, width = 0.1, height = 0.1) +
geom_point(data=Featurein[which(Featurein$Level_1=="Other" &
                                    Featurein$compare=="LADA vs T1D"), ],
           aes(x=log2FoldChange, y=minuslog10, group=Genus), color="#B3B3B3", shape=16,
           size=Featurein[which(Featurein$Level_1=="Other" &
                                    Featurein$compare=="LADA vs T1D"), ]
           [,ncol(Featurein)]/40+0.5) +
geom_jitter(data=Featureout[which(Featureout$Level_1=="Other" &
```

```r
                                     Featureout$compare=="LADA vs T1D"), ],
             aes(x=log2FoldChange, y=minuslog10, group=Genus), color="#B3B3B3", shape=18,
             size=Featureout[which(Featureout$Level_1=="Other" &
                                    Featureout$compare=="LADA vs T1D"), ]
             [,ncol(Featureout)]/40+0.5, width = 0.1, height = 0.1) +
  geom_text(data=Featurein[which(Featurein$padj<padj_threshold &
                                  Featurein$compare=="LADA vs T1D"), ],
             aes(x=log2FoldChange, y=minuslog10, group=Genus, label=Genus),
            nudge_y = 0.2, size=2.5) +
  geom_text(data=Featureout[which(Featureout$padj<padj_threshold &
                                   Featureout$compare=="LADA vs T1D"), ],
             aes(x=log2FoldChange, y=minuslog10, group=Genus, label=Genus),
            nudge_x=0.6, nudge_y = -0.2, size=2.5) +
#  geom_text(data=Featureout[which(Featureout$padj<padj_threshold &
#                                    Featureout$compare=="LADA vs T1D" &
#                                    Featureout$Genus=="Rikenellaceae_RC9_gut_group"), ],
#              aes(x=log2FoldChange, y=minuslog10, group=Genus, label=Genus),
#             nudge_x=1.22, nudge_y = 0) +
  geom_hline(yintercept = -log(0.05), linetype="dashed") +
  geom_hline(yintercept = -log(BHLadaT1D), linetype="dashed", alpha=0.1) +
  geom_vline(xintercept = -1, linetype="dashed") +
  geom_vline(xintercept = 1, linetype="dashed") +
  theme_bw() +
  ggtitle("T1D") +
  xlab("Log2 fold change") +
  ylab("-log10(P)") +
  theme(panel.grid.major = element_blank(),
        panel.grid.minor = element_blank(),
        axis.title=element_text(size=22),
        legend.position="bottom",
        legend.title=element_text(size=20),
        legend.text=element_text(size=20),
        axis.text.x = element_text(angle = 45, hjust = 1, size=12),
        axis.text.y = element_text(angle = 45, hjust = 1, size=12),
        plot.title = element_text(size = 22, hjust=0.5))
#ggplotly(p1)
p1
```

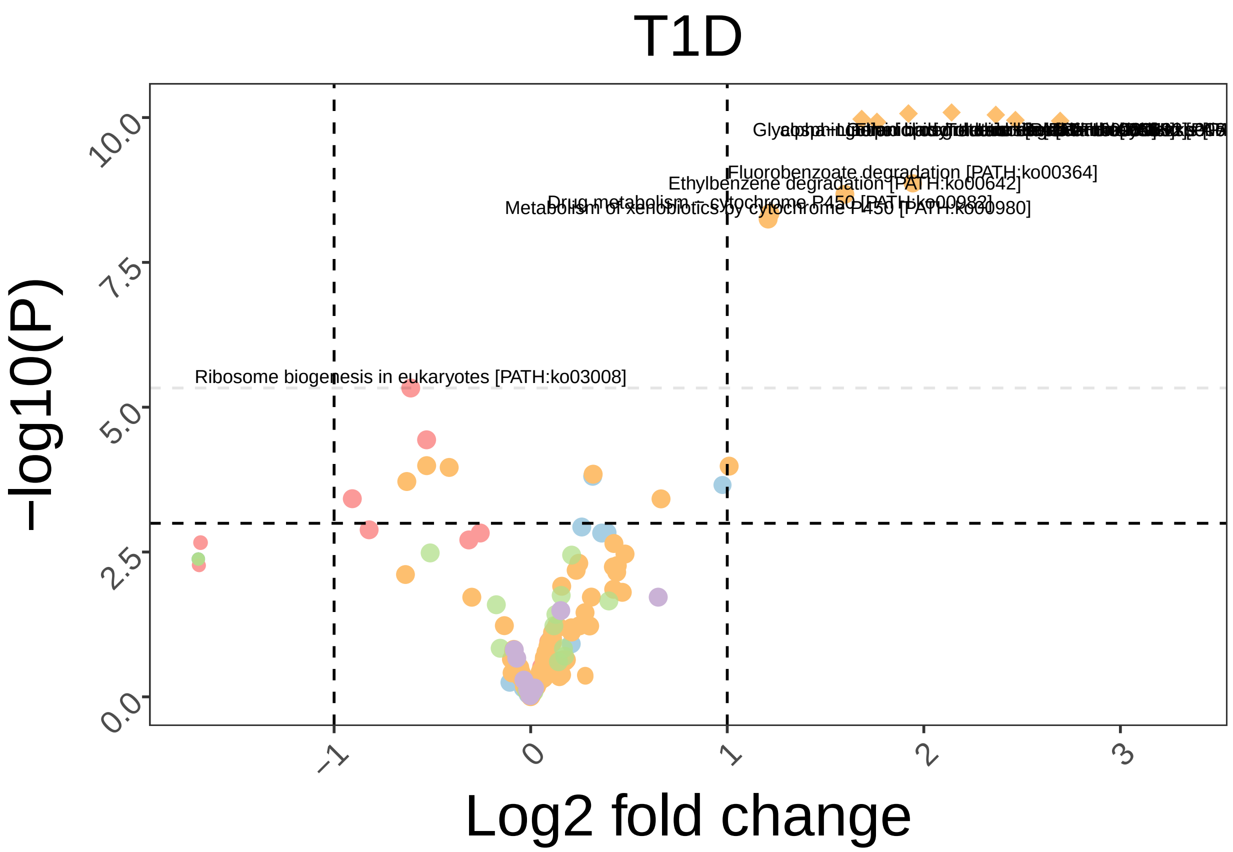


```
Fig2List[["vulcLadaT1D"]] <- p1


p2<-ggplot() +
  geom_point(data=Featurein[which(Featurein$Level_1=="Cellular Processes" &
                                    Featurein$compare=="LADA vs T2D"), ],
             aes(x=log2FoldChange, y=minuslog10, group=Genus), color="#A6CEE3", shape=16,
             size=Featurein[which(Featurein$Level_1=="Cellular Processes" &
                                    Featurein$compare=="LADA vs T2D"), ]
             [,ncol(Featurein)]/40+0.5) +
  #did not add jitter to the first on purpose. Sizes are scaled from percent
  #to the range 0.5-3
  geom_point(data=Featureout[which(Featureout$Level_1=="Cellular Processes" &
                                     Featureout$compare=="LADA vs T2D"), ],
             aes(x=log2FoldChange, y=minuslog10, group=Genus), color="#A6CEE3", shape=18,
             size=Featureout[which(Featureout$Level_1=="Cellular Processes" &
                                     Featureout$compare=="LADA vs T2D"), ]
             [,ncol(Featureout)]/40+0.5) +
  geom_point(data=Featurein[which(Featurein$Level_1=="Genetic Information Processing" &
                                    Featurein$compare=="LADA vs T2D"), ],
             aes(x=log2FoldChange, y=minuslog10, group=Genus), color="#FB9A99", shape=16,
             size=Featurein[which(Featurein$Level_1=="Genetic Information Processing" &
                                    Featurein$compare=="LADA vs T2D"), ]
             [,ncol(Featurein)]/40+0.5) +
  geom_jitter(data=Featureout[which(Featureout$Level_1=="Genetic Information Processing" &
                                      Featureout$compare=="LADA vs T2D"), ],
```

```
           aes(x=log2FoldChange, y=minuslog10, group=Genus), color="#FB9A99", shape=18,
           size=Featureout[which(Featureout$Level_1=="Genetic Information Processing" &
                                  Featureout$compare=="LADA vs T2D"), ]
           [,ncol(Featureout)]/40+0.5,
           width = 0.1, height = 0.1) +
  geom_point(data=Featurein[which(Featurein$Level_1=="Metabolism" &
                                  Featurein$compare=="LADA vs T2D"), ],
           aes(x=log2FoldChange, y=minuslog10, group=Genus), color="#FDBF6F", shape=16,
           size=Featurein[which(Featurein$Level_1=="Metabolism" &
                                  Featurein$compare=="LADA vs T2D"), ]
           [,ncol(Featurein)]/40+0.5) +
  geom_jitter(data=Featureout[which(Featureout$Level_1=="Metabolism" &
                                  Featureout$compare=="LADA vs T2D"), ],
           aes(x=log2FoldChange, y=minuslog10, group=Genus), color="#FDBF6F", shape=18,
           size=Featureout[which(Featureout$Level_1=="Metabolism" &
                                  Featureout$compare=="LADA vs T2D"), ]
           [,ncol(Featureout)]/40+0.5, width = 0.1, height = 0.1) +
  geom_point(data=Featurein[which(Featurein$Level_1=="Environmental Information Processing" &
                                  Featurein$compare=="LADA vs T2D"), ],
           aes(x=log2FoldChange, y=minuslog10, group=Genus),
           color="#B2DF8A", shape=16, alpha=0.75,
           size=Featurein[which(Featurein$Level_1=="Environmental Information Processing" &
                                  Featurein$compare=="LADA vs T2D"), ]
           [,ncol(Featurein)]/40+0.5) +
  geom_jitter(data=Featureout[which(Featureout$Level_1=="Environmental Information Processing" &
                                  Featureout$compare=="LADA vs T2D"), ],
           aes(x=log2FoldChange, y=minuslog10, group=Genus),
           color="#B2DF8A", shape=18,
           size=Featureout[which(Featureout$Level_1=="Environmental Information Processing" &
                                  Featureout$compare=="LADA vs T2D"), ]
           [,ncol(Featureout)]/40+0.5, width = 0.1, height = 0.1) +
  geom_point(data=Featurein[which(Featurein$Level_1=="Unclassified metabolism" &
                                  Featurein$compare=="LADA vs T2D"), ],
           aes(x=log2FoldChange, y=minuslog10, group=Genus), color="#CAB2D6", shape=16,
           size=Featurein[which(Featurein$Level_1=="Unclassified metabolism" &
                                  Featurein$compare=="LADA vs T2D"), ]
           [,ncol(Featurein)]/40+0.5) +
  geom_jitter(data=Featureout[which(Featureout$Level_1=="Unclassified metabolism" &
                                  Featureout$compare=="LADA vs T2D"), ],
           aes(x=log2FoldChange, y=minuslog10, group=Genus), color="#CAB2D6", shape=18,
           size=Featureout[which(Featureout$Level_1=="Unclassified metabolism" &
                                  Featureout$compare=="LADA vs T2D"), ]
           [,ncol(Featureout)]/40+0.5, width = 0.1, height = 0.1) +
  geom_point(data=Featurein[which(Featurein$Level_1=="Other" &
                                  Featurein$compare=="LADA vs T2D"), ],
           aes(x=log2FoldChange, y=minuslog10, group=Genus), color="#B3B3B3", shape=16,
           size=Featurein[which(Featurein$Level_1=="Other" &
                                  Featurein$compare=="LADA vs T2D"), ]
           [,ncol(Featurein)]/40+0.5) +
  geom_jitter(data=Featureout[which(Featureout$Level_1=="Other" &
                                  Featureout$compare=="LADA vs T2D"), ],
           aes(x=log2FoldChange, y=minuslog10, group=Genus), color="#B3B3B3", shape=18,
           size=Featureout[which(Featureout$Level_1=="Other" &
```

```
                                        Featureout$compare=="LADA vs T2D"), ]
             [,ncol(Featureout)]/40+0.5, width = 0.1, height = 0.1) +
  geom_text(data=Featurein[which(Featurein$padj<padj_threshold &
                                  Featurein$compare=="LADA vs T2D"), ],
             aes(x=log2FoldChange, y=minuslog10, group=Genus, label=Genus),
            nudge_y = 0.2, size=2.5) +
  geom_text(data=Featureout[which(Featureout$padj<padj_threshold &
                                   Featureout$compare=="LADA vs T2D"), ],
             aes(x=log2FoldChange, y=minuslog10, group=Genus, label=Genus),
            nudge_x=0.6, nudge_y = -0.2, size=2.5) +
#  geom_text(data=Featureout[which(Featureout$padj<padj_threshold &
#                                    Featureout$compare=="LADA vs T1D" &
#                                    Featureout$Genus=="Rikenellaceae_RC9_gut_group"), ],
#              aes(x=log2FoldChange, y=minuslog10, group=Genus, label=Genus),
#             nudge_x=1.22, nudge_y = 0) +
  geom_hline(yintercept = -log(0.05), linetype="dashed") +
  geom_hline(yintercept = -log(BHLadaT2D), linetype="dashed", alpha=0.1) +
  geom_vline(xintercept = -1, linetype="dashed") +
  geom_vline(xintercept = 1, linetype="dashed") +
  theme_bw() +
  ggtitle("T2D") +
  xlab("Log2 fold change") +
  ylab("-log10(P)") +
  theme(panel.grid.major = element_blank(),
        panel.grid.minor = element_blank(),
        axis.title=element_text(size=22),
        legend.position="bottom",
        legend.title=element_text(size=20),
        legend.text=element_text(size=20),
        axis.text.x = element_text(angle = 45, hjust = 1, size=12),
        axis.text.y = element_text(angle = 45, hjust = 1, size=12),
        plot.title = element_text(size = 22, hjust=0.5))
#ggplotly(p2)
p2
```

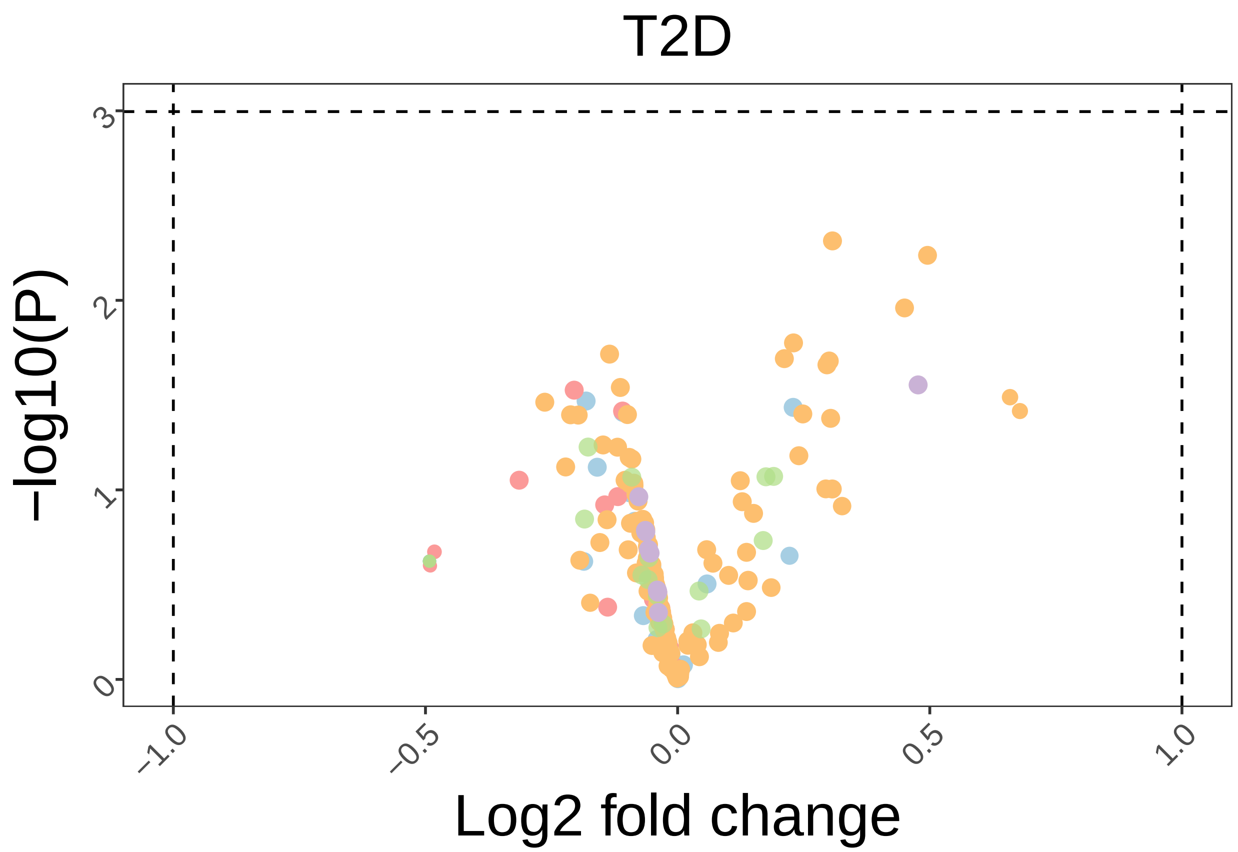


```
Fig2List[["vulcLadaT2D"]] <- p2


p3<-ggplot() +
  geom_point(data=Featurein[which(Featurein$Level_1=="Cellular Processes" &
                                   Featurein$compare=="LADA vs Control"), ],
             aes(x=log2FoldChange, y=minuslog10, group=Genus), color="#A6CEE3", shape=16,
             size=Featurein[which(Featurein$Level_1=="Cellular Processes" &
                                   Featurein$compare=="LADA vs Control"), ]
             [,ncol(Featurein)]/40+0.5) +
  #did not add jitter to the first on purpose. Sizes are scaled from percent to the range 0.5-3
  geom_point(data=Featureout[which(Featureout$Level_1=="Cellular Processes" &
                                    Featureout$compare=="LADA vs Control"), ],
             aes(x=log2FoldChange, y=minuslog10, group=Genus), color="#A6CEE3", shape=18,
             size=Featureout[which(Featureout$Level_1=="Cellular Processes" &
                                    Featureout$compare=="LADA vs Control"), ]
             [,ncol(Featureout)]/40+0.5) +
  geom_point(data=Featurein[which(Featurein$Level_1=="Genetic Information Processing" &
                                   Featurein$compare=="LADA vs Control"), ],
             aes(x=log2FoldChange, y=minuslog10, group=Genus), color="#FB9A99", shape=16,
             size=Featurein[which(Featurein$Level_1=="Genetic Information Processing" &
                                   Featurein$compare=="LADA vs Control"), ]
             [,ncol(Featurein)]/40+0.5) +
  geom_jitter(data=Featureout[which(Featureout$Level_1=="Genetic Information Processing" &
                                     Featureout$compare=="LADA vs Control"), ],
             aes(x=log2FoldChange, y=minuslog10, group=Genus), color="#FB9A99", shape=18,
```

```
          size=Featureout[which(Featureout$Level_1=="Genetic Information Processing" &
                                      Featureout$compare=="LADA vs Control"), ]
          [,ncol(Featureout)]/40+0.5, width = 0.1, height = 0.1) +
  geom_point(data=Featurein[which(Featurein$Level_1=="Metabolism" &
                                     Featurein$compare=="LADA vs Control"), ],
          aes(x=log2FoldChange, y=minuslog10, group=Genus), color="#FDBF6F", shape=16,
          size=Featurein[which(Featurein$Level_1=="Metabolism" &
                                     Featurein$compare=="LADA vs Control"), ]
          [,ncol(Featurein)]/40+0.5) +
  geom_jitter(data=Featureout[which(Featureout$Level_1=="Metabolism" &
                                       Featureout$compare=="LADA vs Control"), ],
          aes(x=log2FoldChange, y=minuslog10, group=Genus), color="#FDBF6F", shape=18,
          size=Featureout[which(Featureout$Level_1=="Metabolism" &
                                      Featureout$compare=="LADA vs Control"), ]
          [,ncol(Featureout)]/40+0.5, width = 0.1, height = 0.1) +
  geom_point(data=Featurein[which(Featurein$Level_1=="Environmental Information Processing" &
                                     Featurein$compare=="LADA vs Control"), ],
          aes(x=log2FoldChange, y=minuslog10, group=Genus),
          color="#B2DF8A", shape=16, alpha=0.75,
          size=Featurein[which(Featurein$Level_1=="Environmental Information Processing" &
                                     Featurein$compare=="LADA vs Control"), ]
          [,ncol(Featurein)]/40+0.5) +
  geom_jitter(data=Featureout[which(Featureout$Level_1=="Environmental Information Processing" &
                                       Featureout$compare=="LADA vs Control"), ],
          aes(x=log2FoldChange, y=minuslog10, group=Genus),
          color="#B2DF8A", shape=18,
          size=Featureout[which(Featureout$Level_1=="Environmental Information Processing" &
                                      Featureout$compare=="LADA vs Control"), ]
          [,ncol(Featureout)]/40+0.5, width = 0.1, height = 0.1) +
  geom_point(data=Featurein[which(Featurein$Level_1=="Unclassified metabolism" &
                                     Featurein$compare=="LADA vs Control"), ],
          aes(x=log2FoldChange, y=minuslog10, group=Genus), color="#CAB2D6", shape=16,
          size=Featurein[which(Featurein$Level_1=="Unclassified metabolism" &
                                     Featurein$compare=="LADA vs Control"), ]
          [,ncol(Featurein)]/40+0.5) +
  geom_jitter(data=Featureout[which(Featureout$Level_1=="Unclassified metabolism" &
                                       Featureout$compare=="LADA vs Control"), ],
          aes(x=log2FoldChange, y=minuslog10, group=Genus), color="#CAB2D6", shape=18,
          size=Featureout[which(Featureout$Level_1=="Unclassified metabolism" &
                                      Featureout$compare=="LADA vs Control"), ]
          [,ncol(Featureout)]/40+0.5, width = 0.1, height = 0.1) +
  geom_point(data=Featurein[which(Featurein$Level_1=="Other" &
                                     Featurein$compare=="LADA vs Control"), ],
          aes(x=log2FoldChange, y=minuslog10, group=Genus), color="#B3B3B3", shape=16,
          size=Featurein[which(Featurein$Level_1=="Other" &
                                     Featurein$compare=="LADA vs Control"), ]
          [,ncol(Featurein)]/40+0.5) +
  geom_jitter(data=Featureout[which(Featureout$Level_1=="Other" &
                                       Featureout$compare=="LADA vs Control"), ],
          aes(x=log2FoldChange, y=minuslog10, group=Genus), color="#B3B3B3", shape=18,
          size=Featureout[which(Featureout$Level_1=="Other" &
                                      Featureout$compare=="LADA vs Control"), ]
          [,ncol(Featureout)]/40+0.5, width = 0.1, height = 0.1) +
```

```
  geom_text(data=Featurein[which(Featurein$padj<padj_threshold &
                                 Featurein$compare=="LADA vs Control"), ],
            aes(x=log2FoldChange, y=minuslog10, group=Genus, label=Genus),
           nudge_y = 0.2, size=2.5) +
  geom_text(data=Featureout[which(Featureout$padj<padj_threshold &
                                  Featureout$compare=="LADA vs Control"), ],
            aes(x=log2FoldChange, y=minuslog10, group=Genus, label=Genus),
           nudge_x=0.6, nudge_y = -0.2, size=2.5) +
#  geom_text(data=Featureout[which(Featureout$padj<padj_threshold &
#                                   Featureout$compare=="LADA vs T1D" &
#                                   Featureout$Genus=="Rikenellaceae_RC9_gut_group"), ],
#             aes(x=log2FoldChange, y=minuslog10, group=Genus, label=Genus),
#            nudge_x=1.22, nudge_y = 0) +
  geom_hline(yintercept = -log(0.05), linetype="dashed") +
  geom_hline(yintercept = -log(BHLadaCon), linetype="dashed", alpha=0.1) +
  geom_vline(xintercept = -1, linetype="dashed") +
  geom_vline(xintercept = 1, linetype="dashed") +
  theme_bw() +
  ggtitle("Controls") +
  xlab("Log2 fold change") +
  ylab("-log10(P)") +
  theme(panel.grid.major = element_blank(),
        panel.grid.minor = element_blank(),
        axis.title=element_text(size=22),
        legend.position="bottom",
        legend.title=element_text(size=20),
        legend.text=element_text(size=20),
        axis.text.x = element_text(angle = 45, hjust = 1, size=12),
        axis.text.y = element_text(angle = 45, hjust = 1, size=12),
        plot.title = element_text(size = 22, hjust=0.5))
#ggplotly(p3)
p3
```

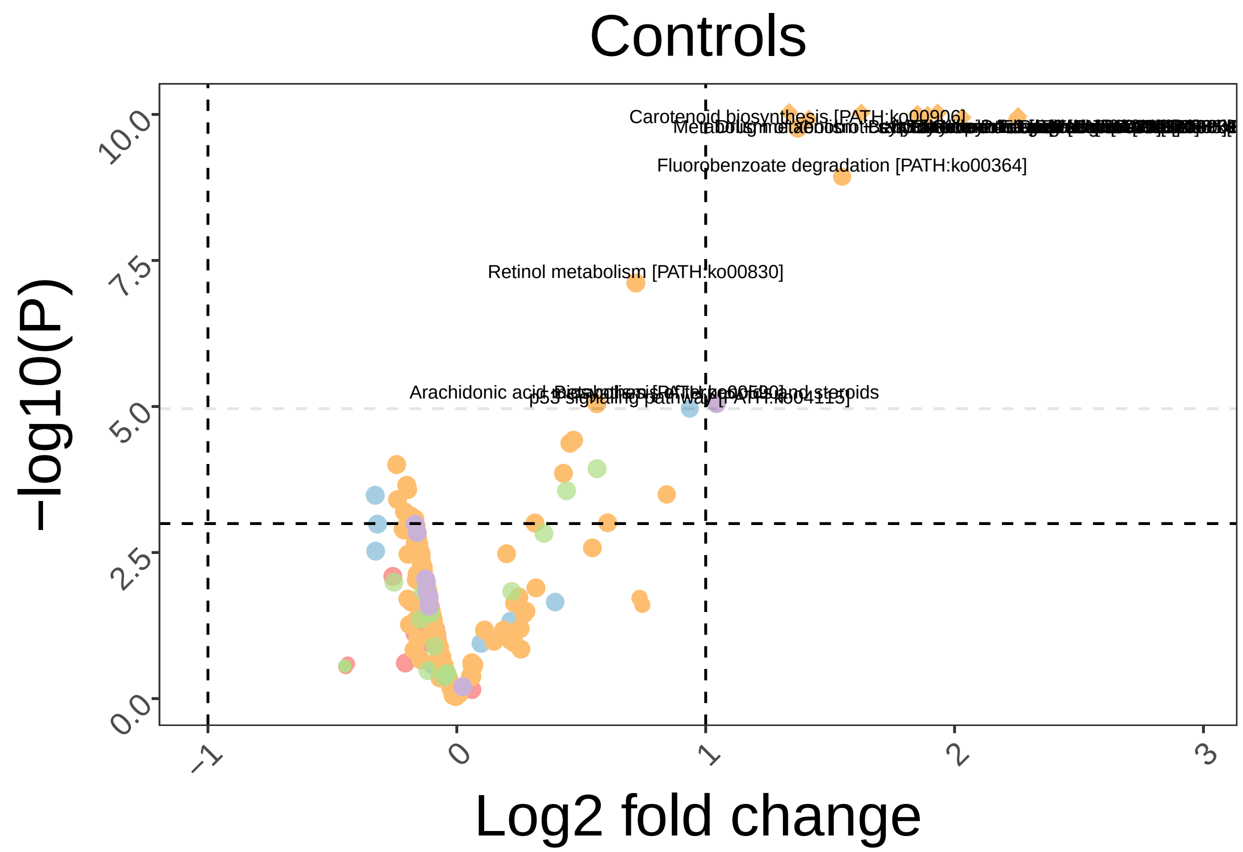


```
Fig2List[["vulcLadaControl"]] <- p3

lay <- rbind(c(1,2,3))
pdf(paste("MicroLADA_Vulcano_Func.pdf", sep=""), width=15, height=5)
grid.arrange(p1,p2,p3,layout_matrix = lay)
dev.off()
```

pdf 2

```
##Boxplot with stats intertwined
#Stats have to be in the end of the chunk don't ask me why
#Have to use the same naming in res_stat to output tables
res_stat$Genus<-str_replace_all(res_stat$Genus, " ","_")
res_stat$Genus<-str_replace_all(res_stat$Genus, "/","_")
res_stat$Genus<-gsub("\\[|\\]", "", res_stat$Genus)
res_stat$Genus<-str_replace_all(res_stat$Genus, ":","_")
res_stat$Genus<-str_replace_all(res_stat$Genus, "-","_")

#Order Diagnosis
Plotting2$Diagnosis<-ordered(Plotting2$Diagnosis,
                             levels=c("Control", "T1D", "LADA", "T2D"))

#SelOrgs<-c('Linoleic_acid_metabolism_PATH_ko00591')
for (i in SelOrgs) {
Boxplot <-
```

```
ggplot(Plotting2, aes_string(x="Diagnosis", y=i, group="Diagnosis")) +
    geom_boxplot() +
#    geom_boxplot(aes(fill=Diagnosis, trim=FALSE)) +
#    geom_jitter() +
    geom_jitter(aes(color=Diagnosis), size=2) +
    stat_summary(fun.data="mean_sdl",
                 mult=1, #mean plus minus a constant (mult=1) times the st.dev
                 geom="pointrange",
                 width=0.2 ) +
    #stat_summary(fun.y = mean, geom = "point") +
    #facet_grid(. ~ Metformin, scales="fixed") + #Scales can also be "free"
    ggtitle(i) +
    #xlab("Diagnosis") +
    ylab("Cells pr. g feces") +
    scale_fill_manual(values=c(Control = "#666666", T1D = "#1F78B4",
                               T2D = "#33A02C", LADA = "#E31A1C")) +
    scale_color_manual(values=c(Control = "#666666", T1D = "#1F78B4",
                               T2D = "#33A02C", LADA = "#E31A1C")) +
    theme_bw() +
    theme(legend.position="none",
          title =element_text(size=18, face='bold'),
          panel.grid.major = element_blank(),
          panel.grid.minor = element_blank(),
          axis.title=element_text(size=16),
          axis.title.x = element_blank(),
          axis.text.x = element_text(angle = 45, hjust = 1, size=14),
          axis.text.y = element_text(angle = 45, hjust = 1, size=12))
Fig2List[[i]] <- Boxplot
#print(kable(stat_sig[which(stat_sig$gene==i), c(5,6,7,10)]))
cat("\n")
tabling<-res_stat[which(res_stat$Genus==i), c(5,6,7,10)]
print(kable(tabling))
#print(tabling)
cat("\n")
print(Boxplot)
}
```

	pvalue	padj	Genus	compare
523	0.0000000	0.0000000	Linoleic_acid_metabolism_PATH_ko00591	LADA vs Control
326	0.0000000	0.0000001	Linoleic_acid_metabolism_PATH_ko00591	Control vs T2D
917	0.0000000	0.0000014	Linoleic_acid_metabolism_PATH_ko00591	LADA vs T1D
720	0.0000001	0.0000093	Linoleic_acid_metabolism_PATH_ko00591	T1D vs T2D
129	0.6440983	0.7508128	Linoleic_acid_metabolism_PATH_ko00591	Control vs T1D
1114	0.6989891	0.9515738	Linoleic_acid_metabolism_PATH_ko00591	LADA vs T2D

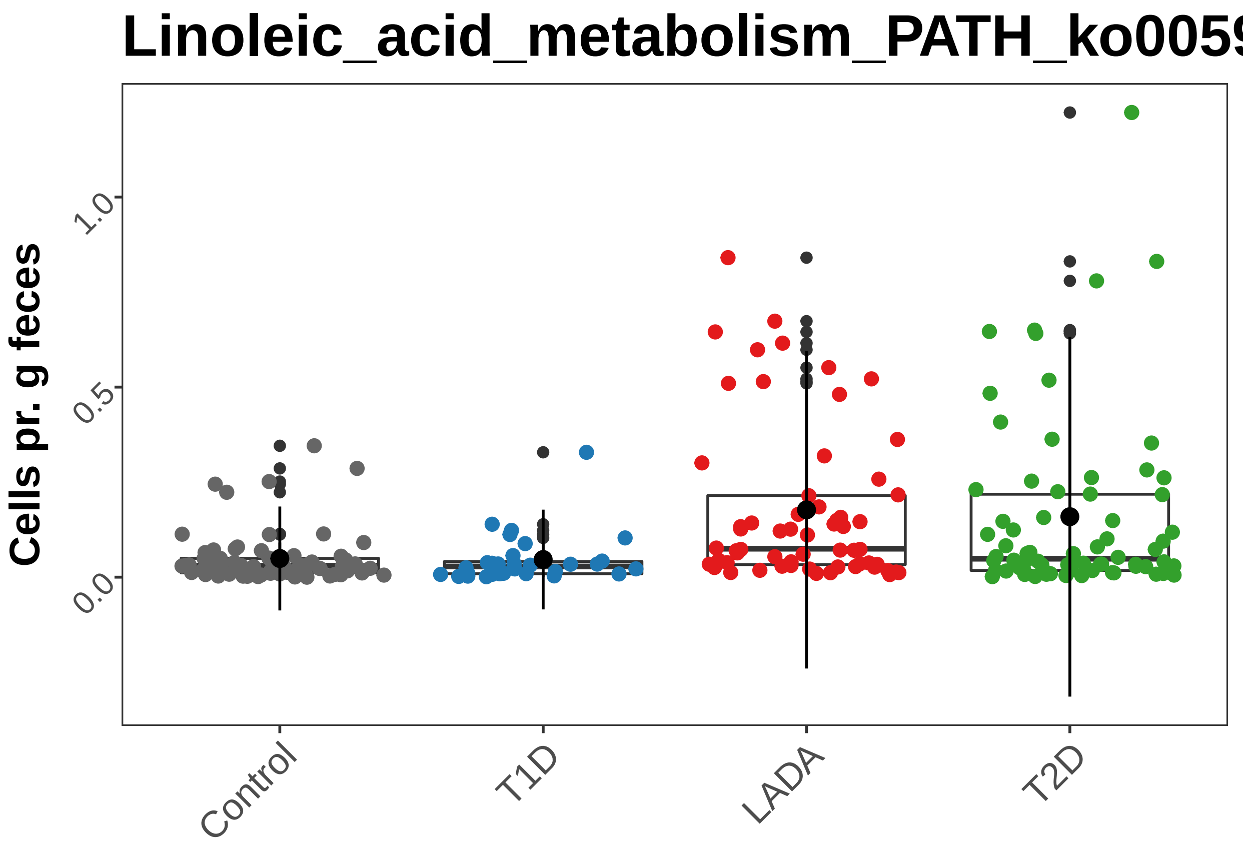

	pvalue	padj	Genus	compare
555	0.0000000	0.0000000	Geraniol_degradation_PATH_ko00281	LADA vs Control
358	0.0000000	0.0000001	Geraniol_degradation_PATH_ko00281	Control vs T2D
949	0.0000029	0.0001137	Geraniol_degradation_PATH_ko00281	LADA vs T1D
752	0.0000092	0.0003631	Geraniol_degradation_PATH_ko00281	T1D vs T2D
161	0.7095106	0.7991831	Geraniol_degradation_PATH_ko00281	Control vs T1D
1146	0.7841883	0.9521572	Geraniol_degradation_PATH_ko00281	LADA vs T2D

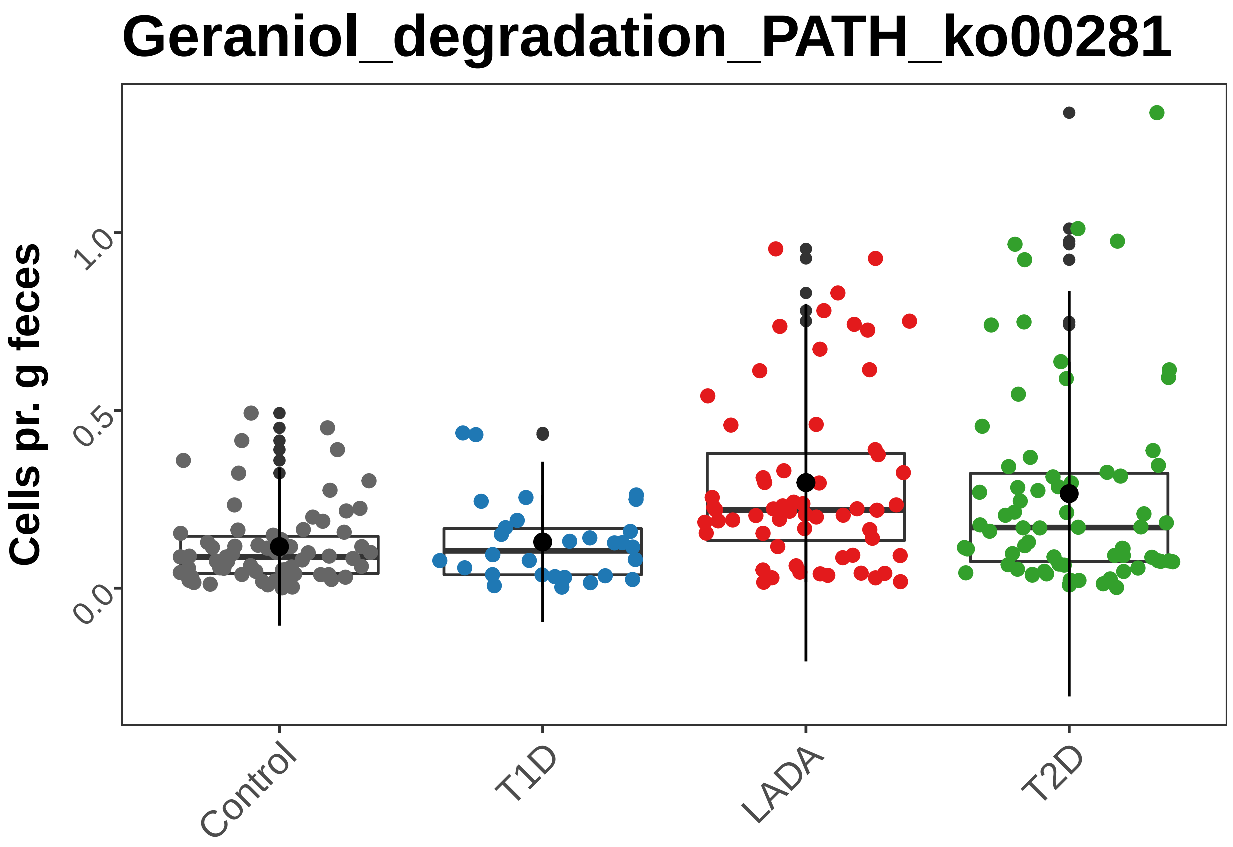

	pvalue	padj	Genus	compare
515	0.0000000	0.0000000	alpha_Linolenic_acid_metabolism_PATH_ko00592	LADA vs Control
318	0.0000000	0.0000001	alpha_Linolenic_acid_metabolism_PATH_ko00592	Control vs T2D
909	0.0000097	0.0003169	alpha_Linolenic_acid_metabolism_PATH_ko00592	LADA vs T1D
712	0.0000182	0.0005972	alpha_Linolenic_acid_metabolism_PATH_ko00592	T1D vs T2D
121	0.5263376	0.6912567	alpha_Linolenic_acid_metabolism_PATH_ko00592	Control vs T1D
1106	0.8871584	0.9763698	alpha_Linolenic_acid_metabolism_PATH_ko00592	LADA vs T2D

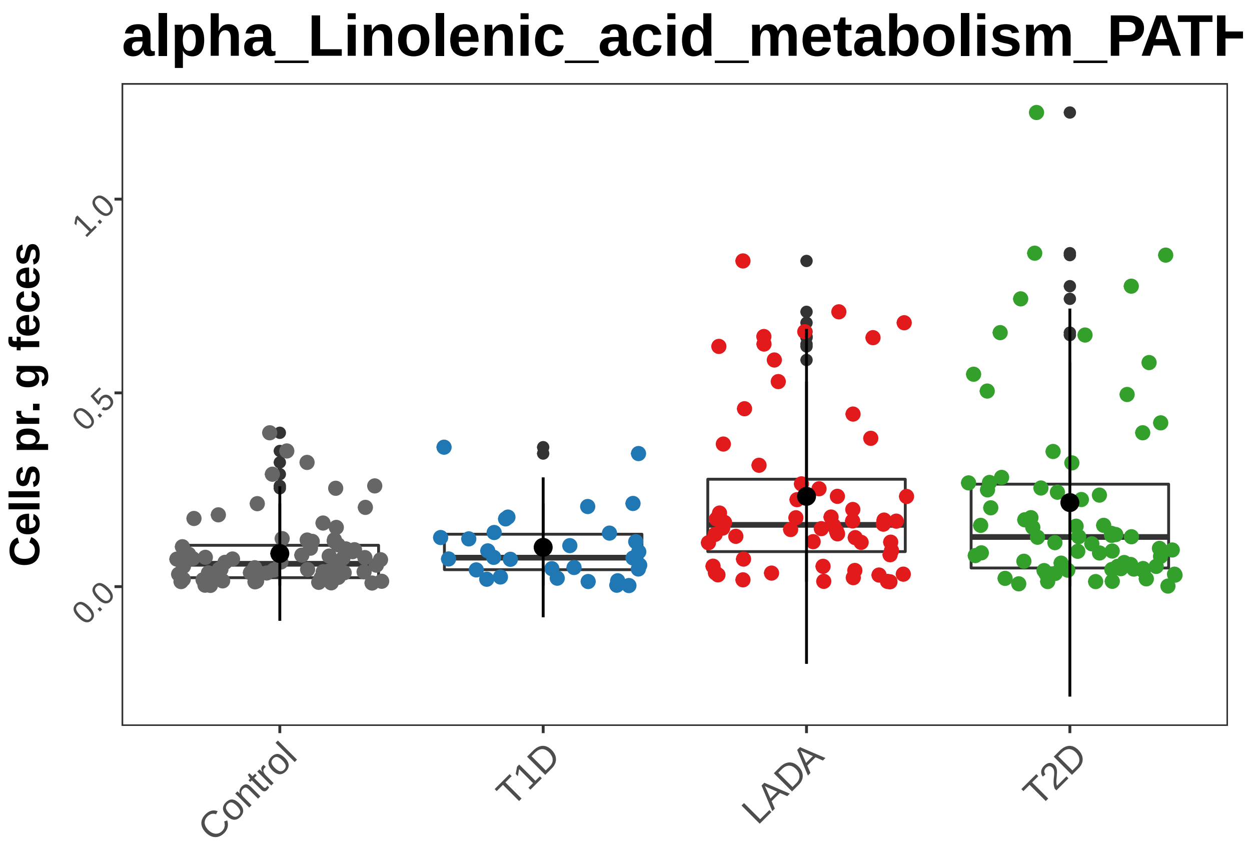

	pvalue	padj	Genus	compare
321	0.0000000	0.0000000	Ether__lipid__metabolism__PATH__ko00565	Control vs T2D
518	0.0000000	0.0000002	Ether__lipid__metabolism__PATH__ko00565	LADA vs Control
715	0.0000002	0.0000142	Ether__lipid__metabolism__PATH__ko00565	T1D vs T2D
912	0.0000023	0.0001137	Ether__lipid__metabolism__PATH__ko00565	LADA vs T1D
1109	0.5332483	0.9515738	Ether__lipid__metabolism__PATH__ko00565	LADA vs T2D
124	0.9727093	0.9826858	Ether__lipid__metabolism__PATH__ko00565	Control vs T1D

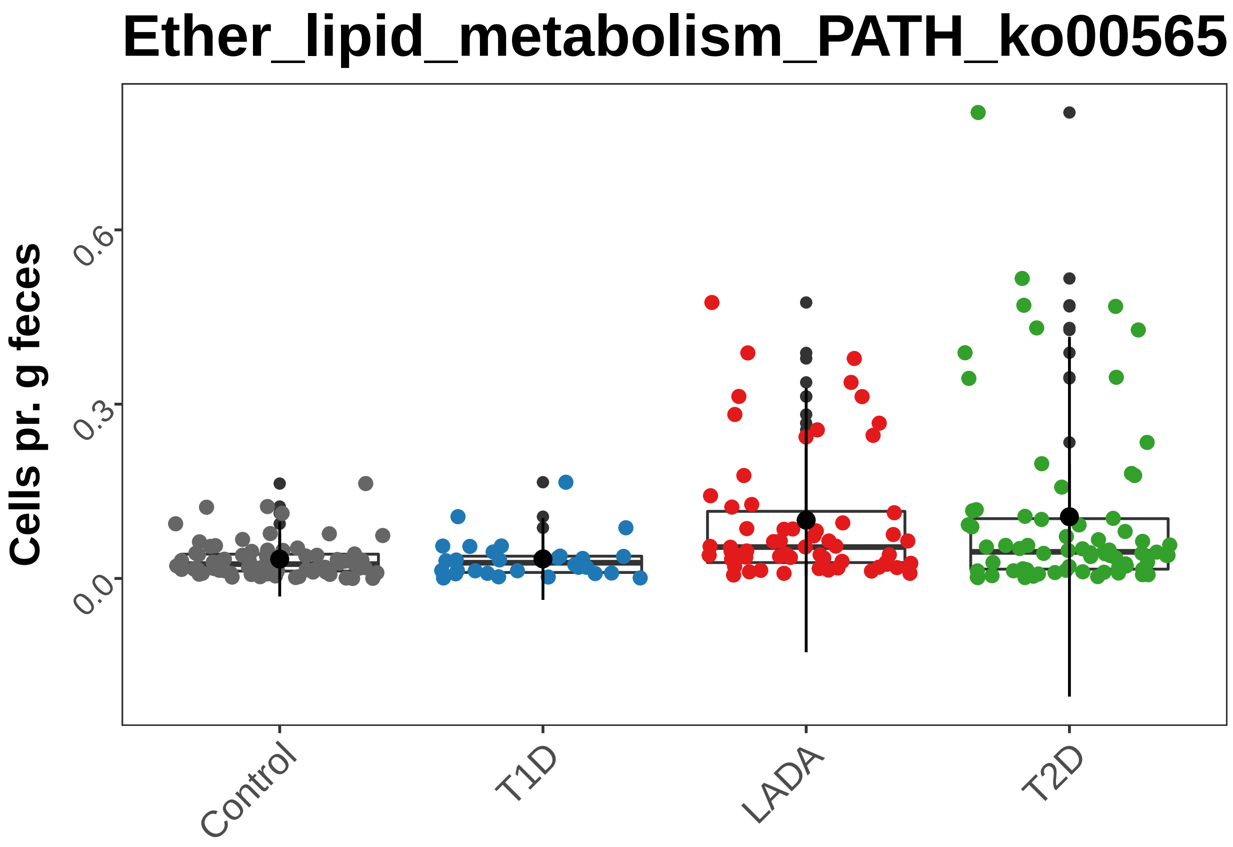

	pvalue	padj	Genus	compare
580	0.0000000	0.0000002	Toluene_degradation_PATH_ko00623	LADA vs Control
383	0.0000001	0.0000033	Toluene_degradation_PATH_ko00623	Control vs T2D
974	0.0000010	0.0000673	Toluene_degradation_PATH_ko00623	LADA vs T1D
777	0.0000081	0.0003631	Toluene_degradation_PATH_ko00623	T1D vs T2D
186	0.8214088	0.8853256	Toluene_degradation_PATH_ko00623	Control vs T1D
1171	0.6160638	0.9515738	Toluene_degradation_PATH_ko00623	LADA vs T2D

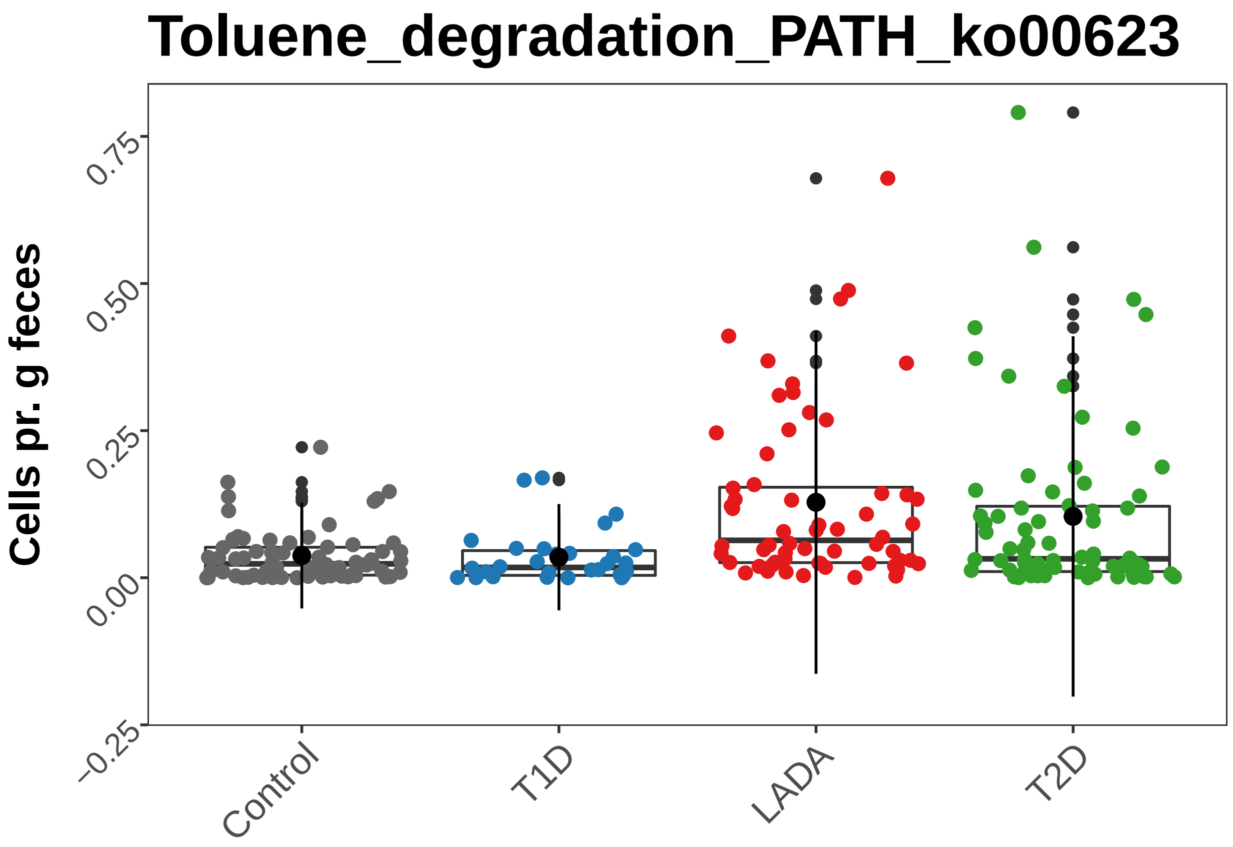

	pvalue	padj	Genus	compare
574	0.0000000	0.0000003	Ethylbenzene_degradation_PATH_ko00642	LADA vs Control
377	0.0000001	0.0000033	Ethylbenzene_degradation_PATH_ko00642	Control vs T2D
968	0.0001706	0.0037353	Ethylbenzene_degradation_PATH_ko00642	LADA vs T1D
771	0.0005056	0.0110677	Ethylbenzene_degradation_PATH_ko00642	T1D vs T2D
180	0.4119094	0.5880155	Ethylbenzene_degradation_PATH_ko00642	Control vs T1D
1165	0.7425645	0.9515738	Ethylbenzene_degradation_PATH_ko00642	LADA vs T2D

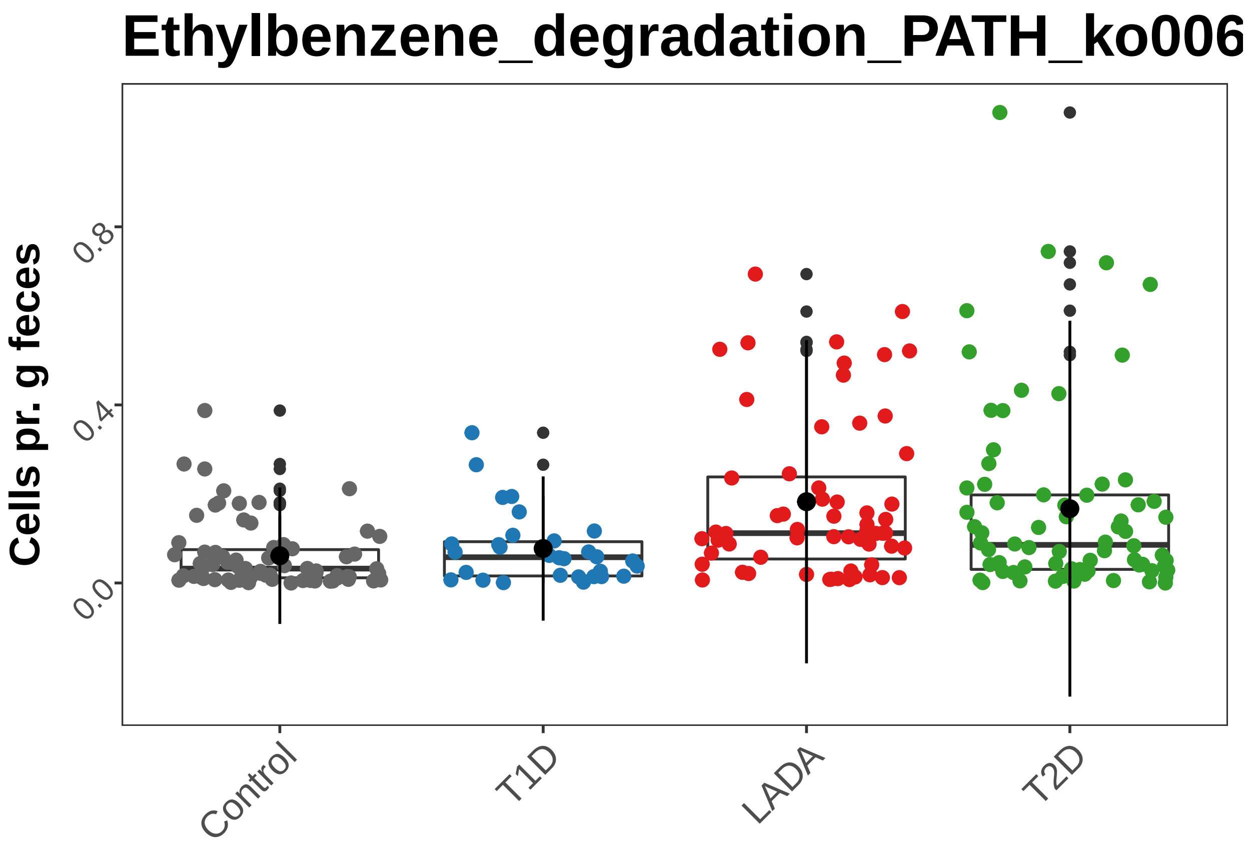

	pvalue	padj	Genus	compare
859	0.0000000	0.0000007	Betalain_biosynthesis_PATH_ko00965	LADA vs T1D
662	0.0000000	0.0000026	Betalain_biosynthesis_PATH_ko00965	T1D vs T2D
465	0.0000035	0.0000767	Betalain_biosynthesis_PATH_ko00965	LADA vs Control
268	0.0000126	0.0002765	Betalain_biosynthesis_PATH_ko00965	Control vs T2D
71	0.0214920	0.3170574	Betalain_biosynthesis_PATH_ko00965	Control vs T1D
1056	0.8229511	0.9521572	Betalain_biosynthesis_PATH_ko00965	LADA vs T2D

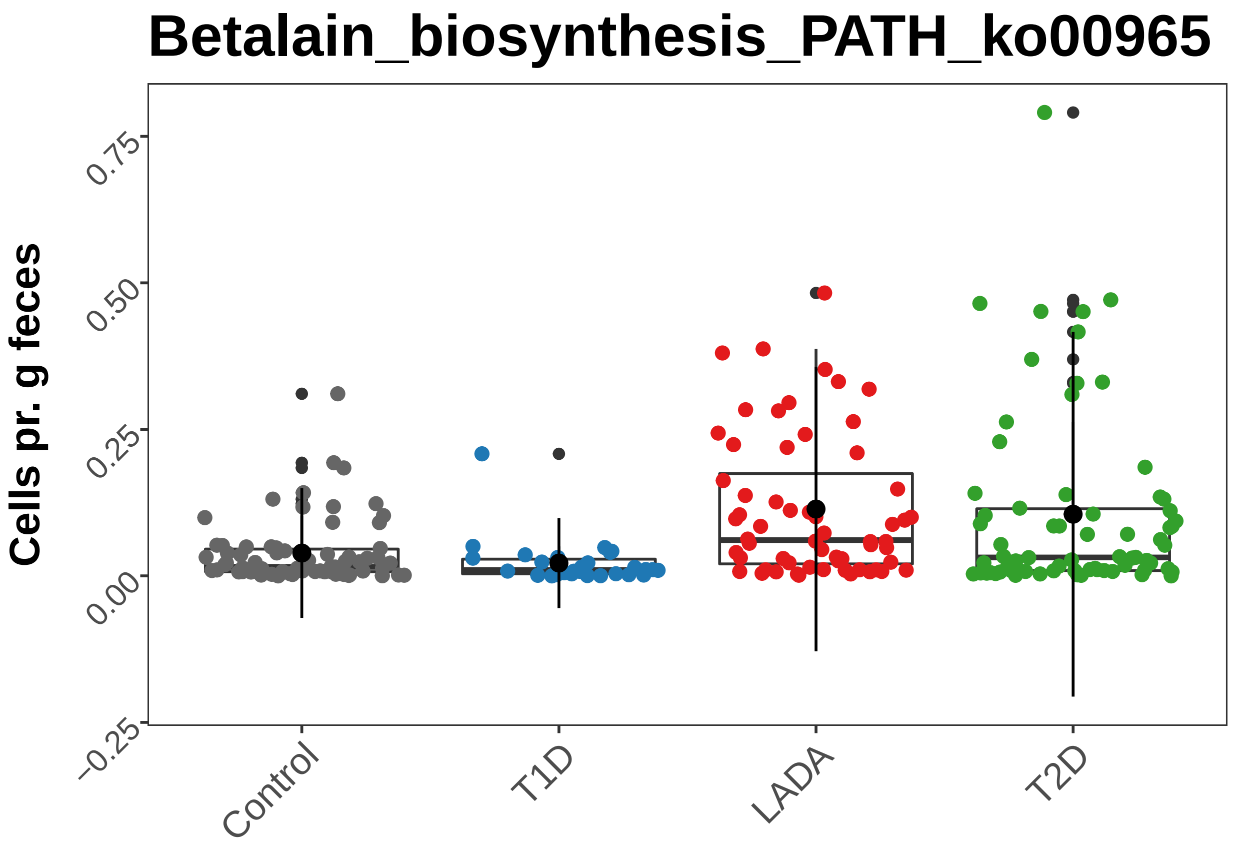

	pvalue	padj	Genus	compare
572	0.0000004	0.0000101	Drug_metabolism_____cytochrome_P450_PATH_ko00982	LADA vs Control
375	0.0000065	0.0001841	Drug_metabolism_____cytochrome_P450_PATH_ko00982	Control vs T2D
966	0.0002368	0.0046652	Drug_metabolism_____cytochrome_P450_PATH_ko00982	LADA vs T1D
769	0.0012164	0.0239621	Drug_metabolism_____cytochrome_P450_PATH_ko00982	T1D vs T2D
178	0.7084203	0.7991831	Drug_metabolism_____cytochrome_P450_PATH_ko00982	Control vs T1D
1163	0.5943236	0.9515738	Drug_metabolism_____cytochrome_P450_PATH_ko00982	LADA vs T2D

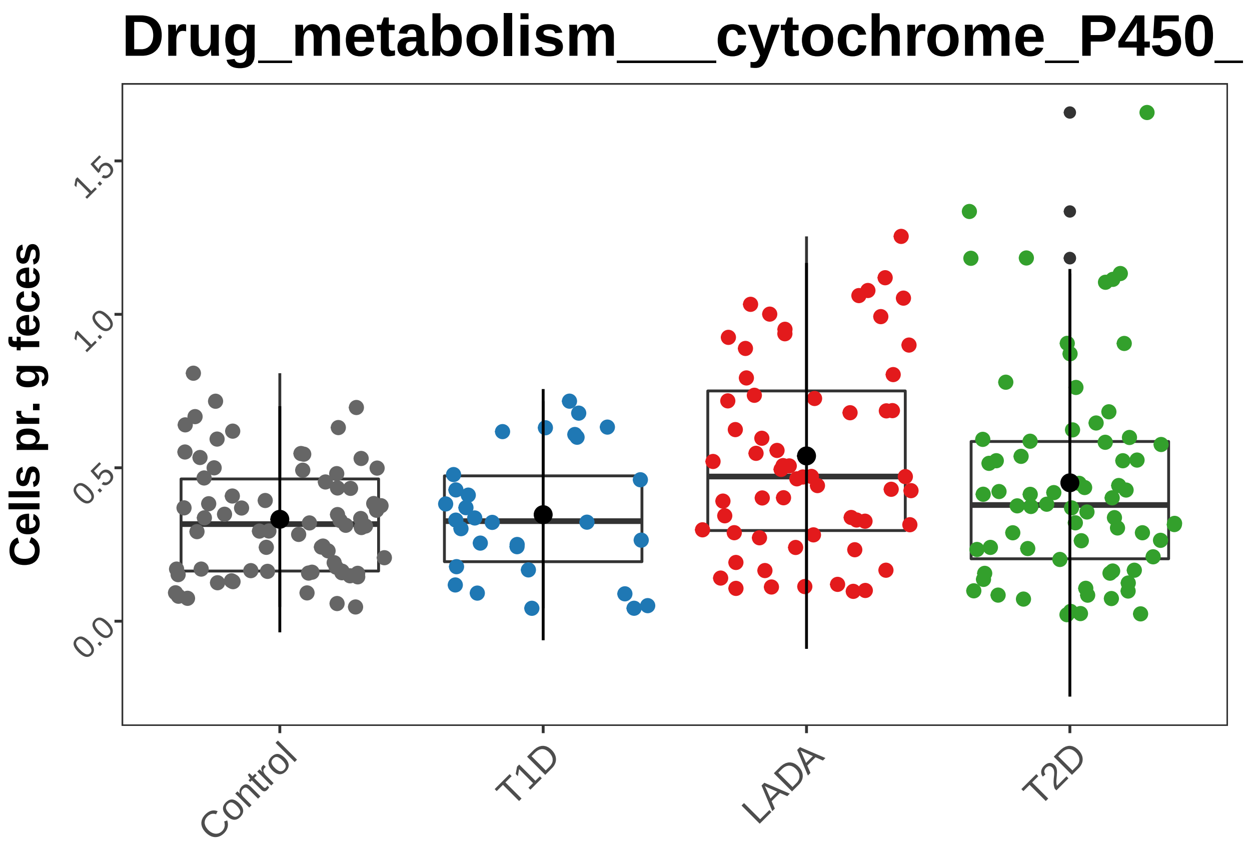

	pvalue	padj	Genus	compare
576	0.0000004	0.0000106	Metabolism_of_xenobiotics_by_cytochrome_P450_PATH_ko00980	LADA vs Control
379	0.0000078	0.0001914	Metabolism_of_xenobiotics_by_cytochrome_P450_PATH_ko00980	Control vs T2D
970	0.0002618	0.0046880	Metabolism_of_xenobiotics_by_cytochrome_P450_PATH_ko00980	LADA vs T1D
773	0.0013380	0.0239621	Metabolism_of_xenobiotics_by_cytochrome_P450_PATH_ko00980	T1D vs T2D
182	0.7099343	0.7991831	Metabolism_of_xenobiotics_by_cytochrome_P450_PATH_ko00980	Control vs T1D
1167	0.5927290	0.9515738	Metabolism_of_xenobiotics_by_cytochrome_P450_PATH_ko00980	LADA vs T2D

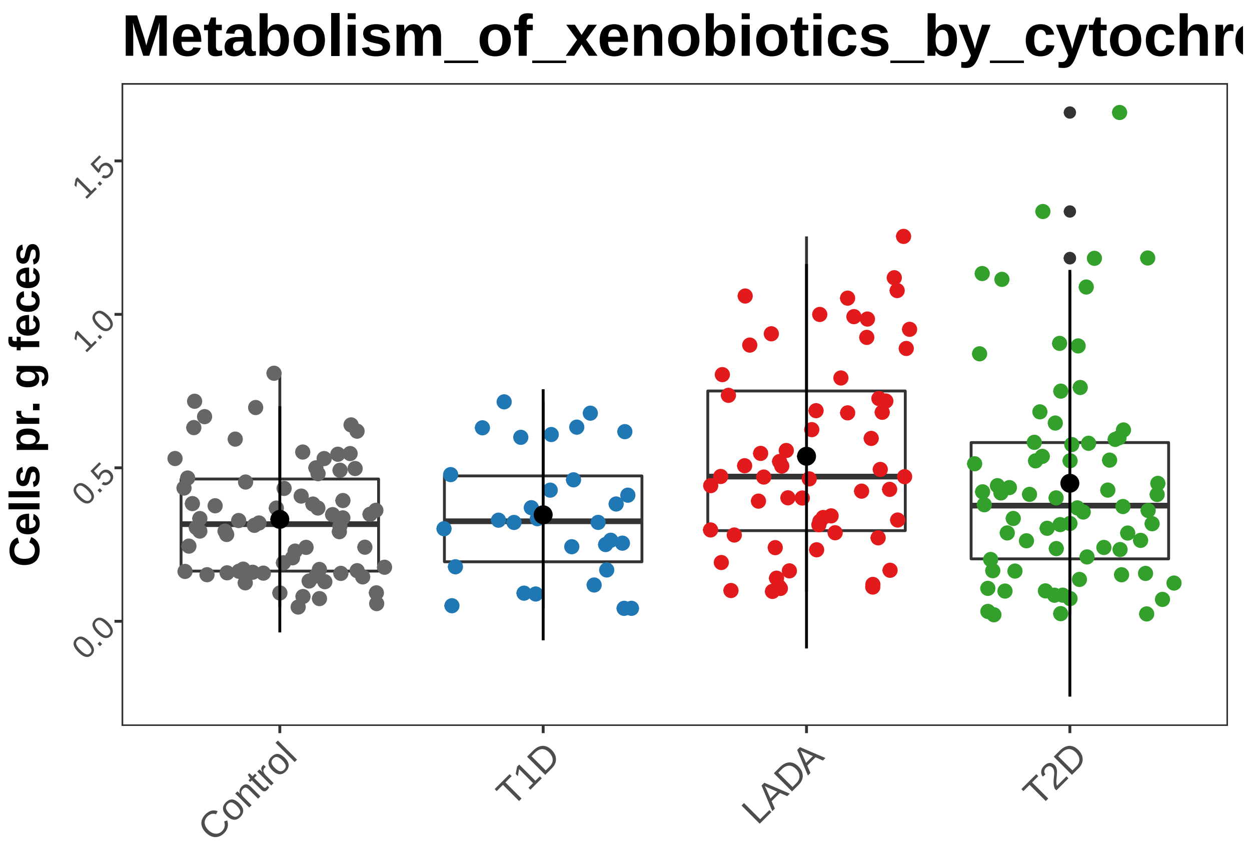

	pvalue	padj	Genus	compare
378	0.0000263	0.0005176	Fluorobenzoate_degradation_PATH_ko00364	Control vs T2D
772	0.0000379	0.0010678	Fluorobenzoate_degradation_PATH_ko00364	T1D vs T2D
575	0.0001318	0.0023610	Fluorobenzoate_degradation_PATH_ko00364	LADA vs Control
969	0.0001414	0.0034809	Fluorobenzoate_degradation_PATH_ko00364	LADA vs T1D
181	0.4226744	0.5990421	Fluorobenzoate_degradation_PATH_ko00364	Control vs T1D
1166	0.6673487	0.9515738	Fluorobenzoate_degradation_PATH_ko00364	LADA vs T2D

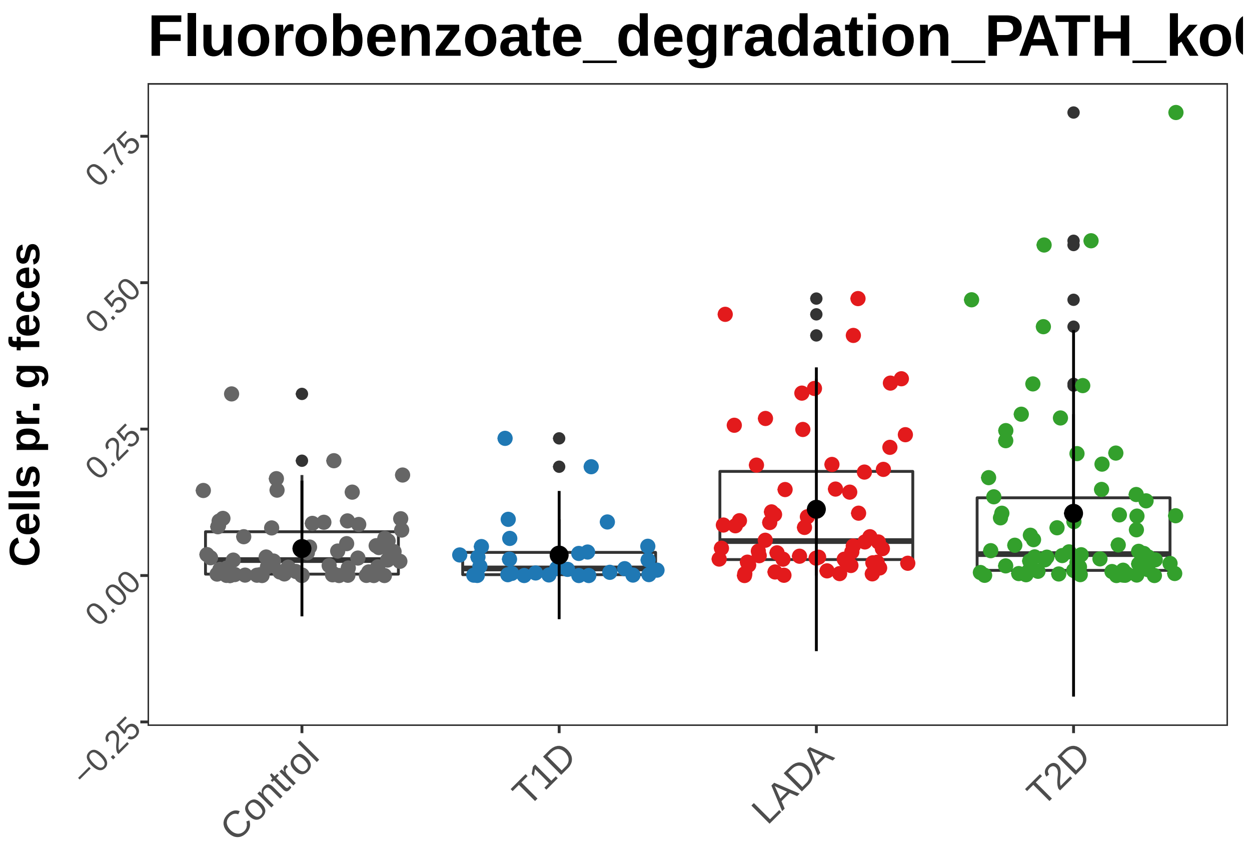

	pvalue	padj	Genus	compare
900	0.0000184	0.0005188	Glycosphingolipid_biosynthesis_____lacto_and_neolacto_series_PATH_ko00601	LADA vs T1D
703	0.0003218	0.0079248	Glycosphingolipid_biosynthesis_____lacto_and_neolacto_series_PATH_ko00601	T1D vs T2D
112	0.0078437	0.2321398	Glycosphingolipid_biosynthesis_____lacto_and_neolacto_series_PATH_ko00601	Control vs T1D
506	0.0303540	0.2426050	Glycosphingolipid_biosynthesis_____lacto_and_neolacto_series_PATH_ko00601	LADA vs Control
309	0.1891414	0.8082167	Glycosphingolipid_biosynthesis_____lacto_and_neolacto_series_PATH_ko00601	Control vs T2D
1097	0.4007451	0.9515738	Glycosphingolipid_biosynthesis_____lacto_and_neolacto_series_PATH_ko00601	LADA vs T2D

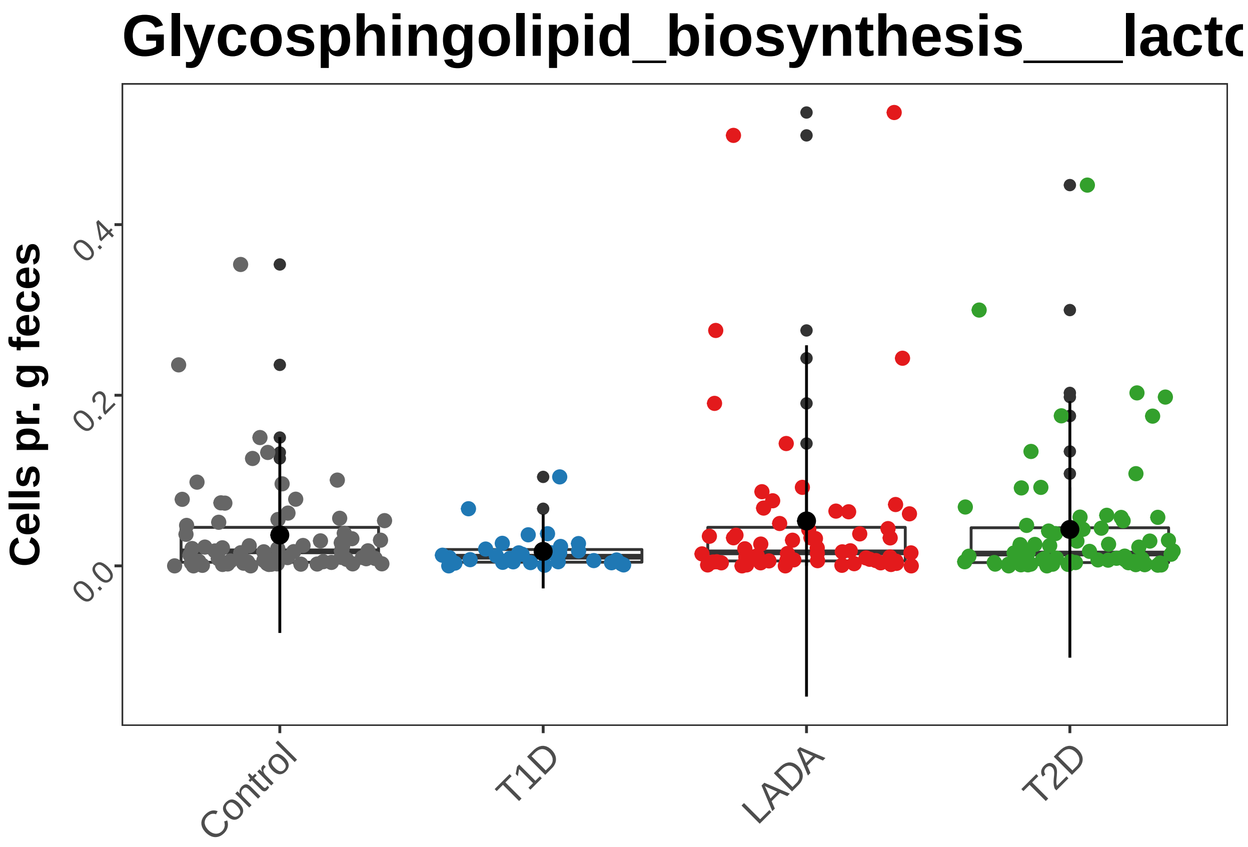

	pvalue	padj	Genus	compare
554	0.0000575	0.0011323	Carotenoid_biosynthesis_PATH_ko00906	LADA vs Control
357	0.0020193	0.0361635	Carotenoid_biosynthesis_PATH_ko00906	Control vs T2D
948	0.0186444	0.2346965	Carotenoid_biosynthesis_PATH_ko00906	LADA vs T1D
160	0.3852207	0.5663320	Carotenoid_biosynthesis_PATH_ko00906	Control vs T1D
751	0.1035017	0.6324066	Carotenoid_biosynthesis_PATH_ko00906	T1D vs T2D
1145	0.3661533	0.9515738	Carotenoid_biosynthesis_PATH_ko00906	LADA vs T2D

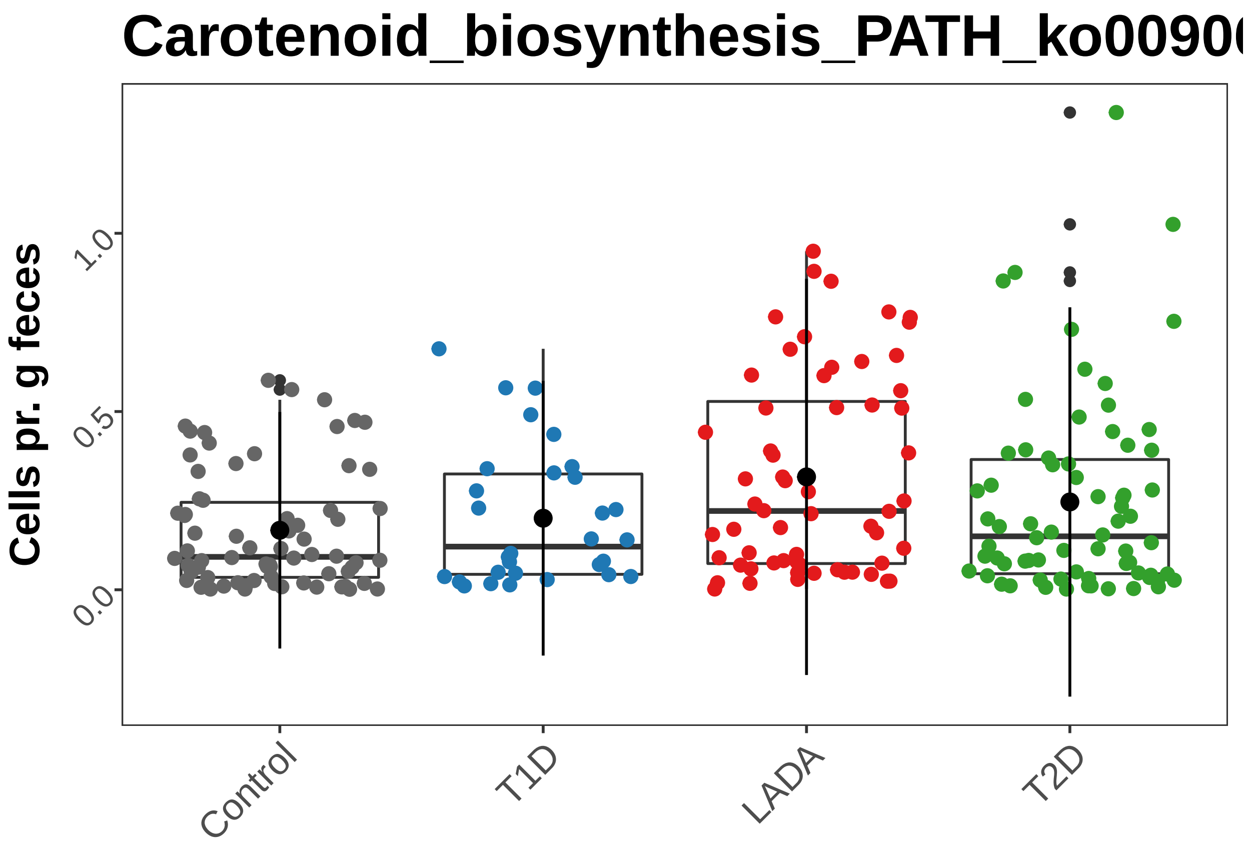

	pvalue	padj	Genus	compare
537	0.0008132	0.0133495	Retinol_metabolism_PATH_ko00830	LADA vs Control
340	0.0301080	0.3954189	Retinol_metabolism_PATH_ko00830	Control vs T2D
931	0.1038239	0.4751596	Retinol_metabolism_PATH_ko00830	LADA vs T1D
143	0.2864287	0.5095493	Retinol_metabolism_PATH_ko00830	Control vs T1D
1128	0.2464473	0.9515738	Retinol_metabolism_PATH_ko00830	LADA vs T2D
734	0.4801648	0.9751541	Retinol_metabolism_PATH_ko00830	T1D vs T2D

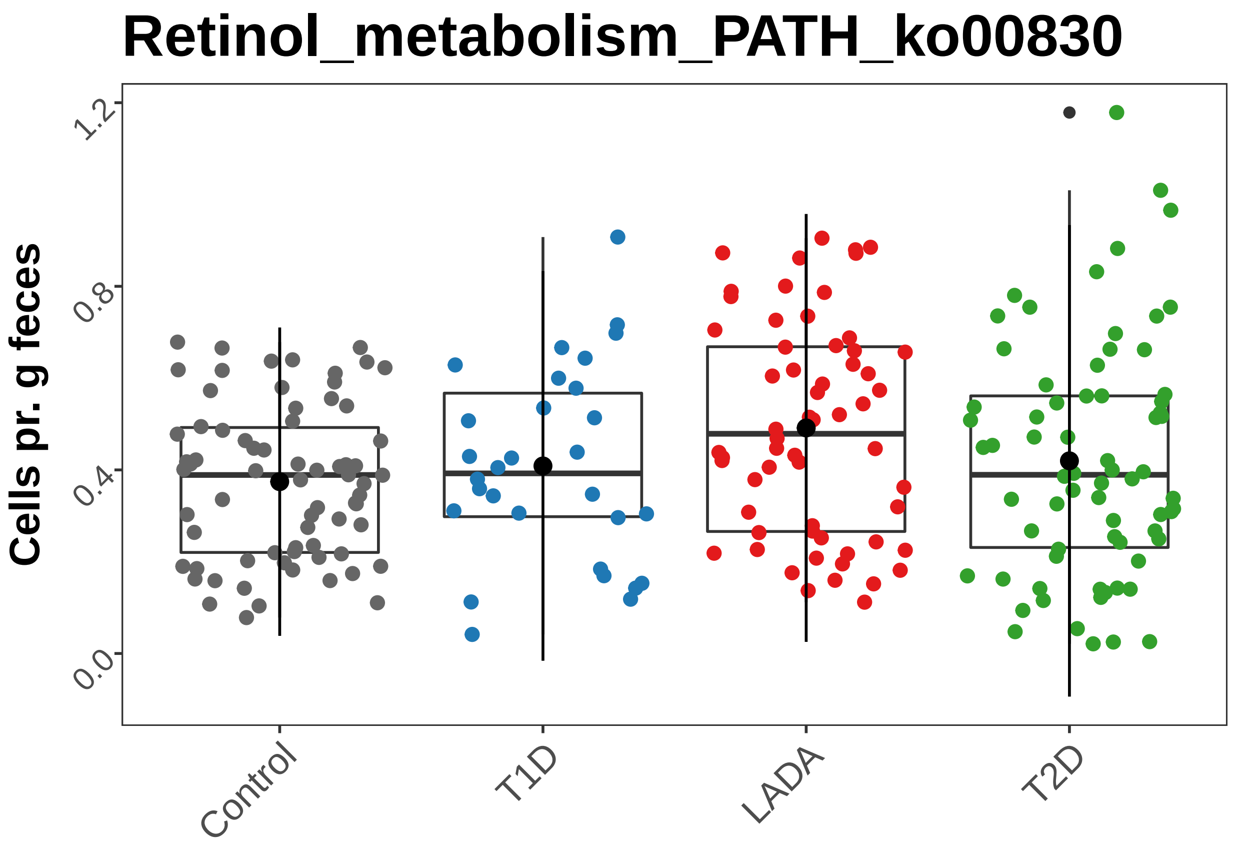

	pvalue	padj	Genus	compare
9	0.0001926	0.0359443	Bacterial_chemotaxis_PATH_ko02030	Control vs T1D
600	0.0048372	0.0733021	Bacterial_chemotaxis_PATH_ko02030	T1D vs T2D
403	0.0307874	0.2426050	Bacterial_chemotaxis_PATH_ko02030	LADA vs Control
797	0.0591860	0.4318389	Bacterial_chemotaxis_PATH_ko02030	LADA vs T1D
206	0.3404387	0.8103002	Bacterial_chemotaxis_PATH_ko02030	Control vs T2D
994	0.2302161	0.9515738	Bacterial_chemotaxis_PATH_ko02030	LADA vs T2D

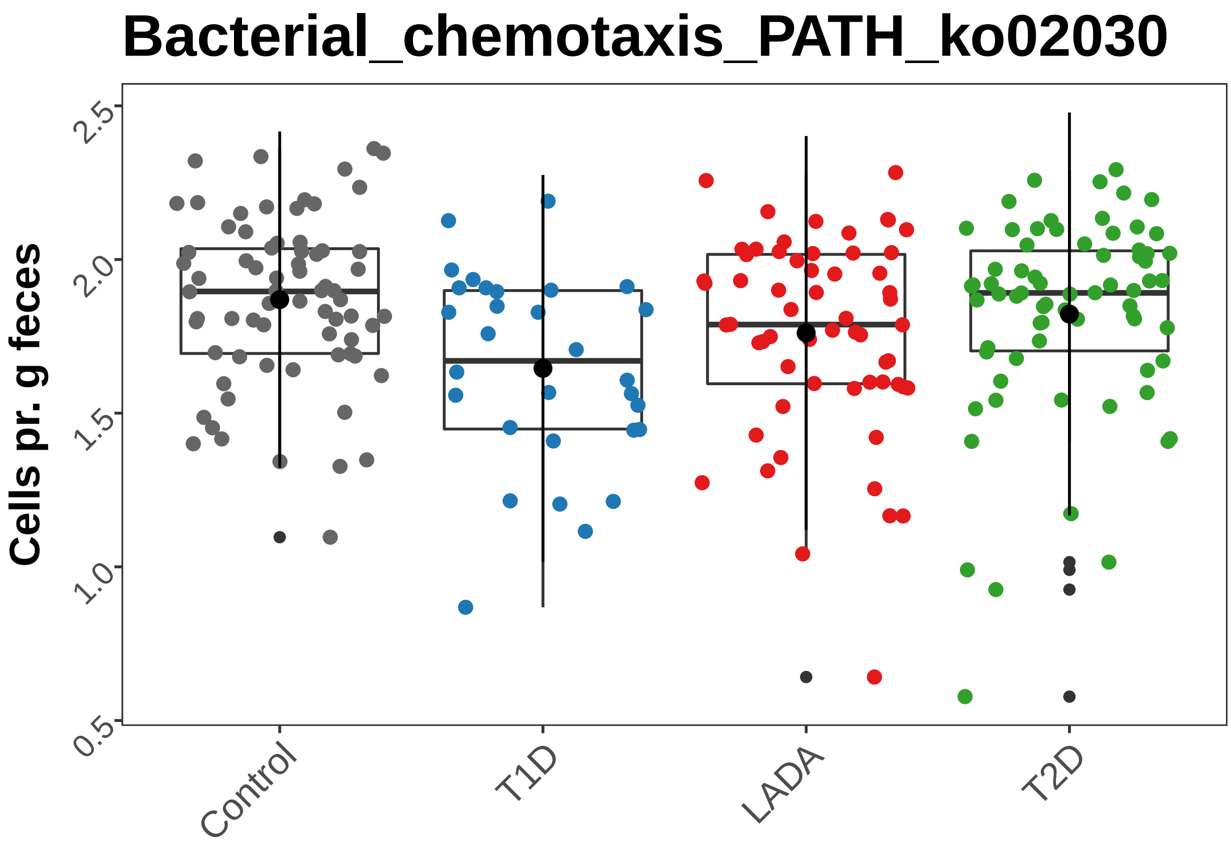

	pvalue	padj	Genus	compare
10	0.0003649	0.0359443	Flagellar_assembly_PATH_ko02040	Control vs T1D
601	0.0080684	0.1059644	Flagellar_assembly_PATH_ko02040	T1D vs T2D
404	0.0504922	0.2910370	Flagellar_assembly_PATH_ko02040	LADA vs Control
798	0.0587595	0.4318389	Flagellar_assembly_PATH_ko02040	LADA vs T1D
207	0.3336179	0.8103002	Flagellar_assembly_PATH_ko02040	Control vs T2D
995	0.3264398	0.9515738	Flagellar_assembly_PATH_ko02040	LADA vs T2D

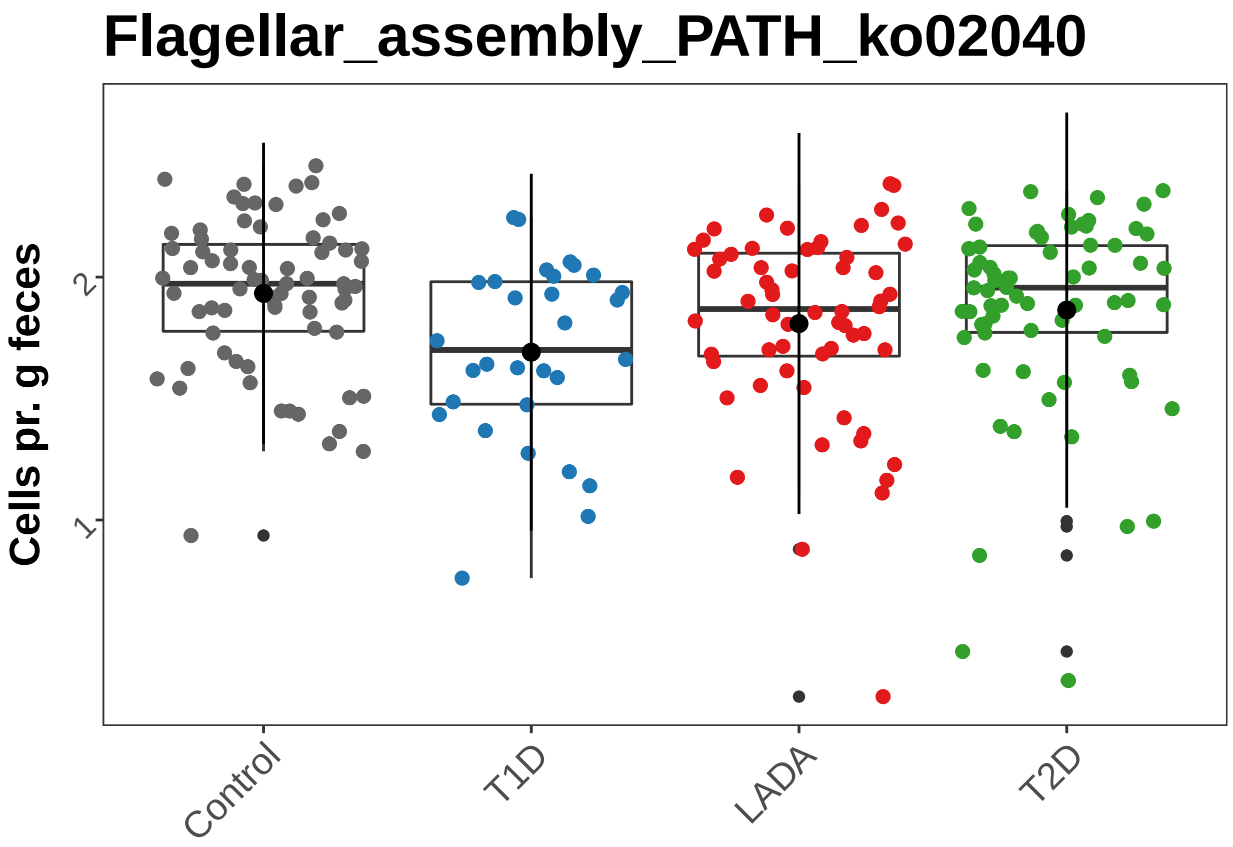

	pvalue	padj	Genus	compare
604	0.0038884	0.0638339	Biofilm_formation____Pseudomonas_aeruginosa_PATH_ko02025	T1D vs T2D
13	0.0082486	0.2321398	Biofilm_formation____Pseudomonas_aeruginosa_PATH_ko02025	Control vs T1D
801	0.0222073	0.2430461	Biofilm_formation____Pseudomonas_aeruginosa_PATH_ko02025	LADA vs T1D
407	0.7394639	0.7707639	Biofilm_formation____Pseudomonas_aeruginosa_PATH_ko02025	LADA vs Control
210	0.6585744	0.9438779	Biofilm_formation____Pseudomonas_aeruginosa_PATH_ko02025	Control vs T2D
998	0.4341410	0.9515738	Biofilm_formation____Pseudomonas_aeruginosa_PATH_ko02025	LADA vs T2D

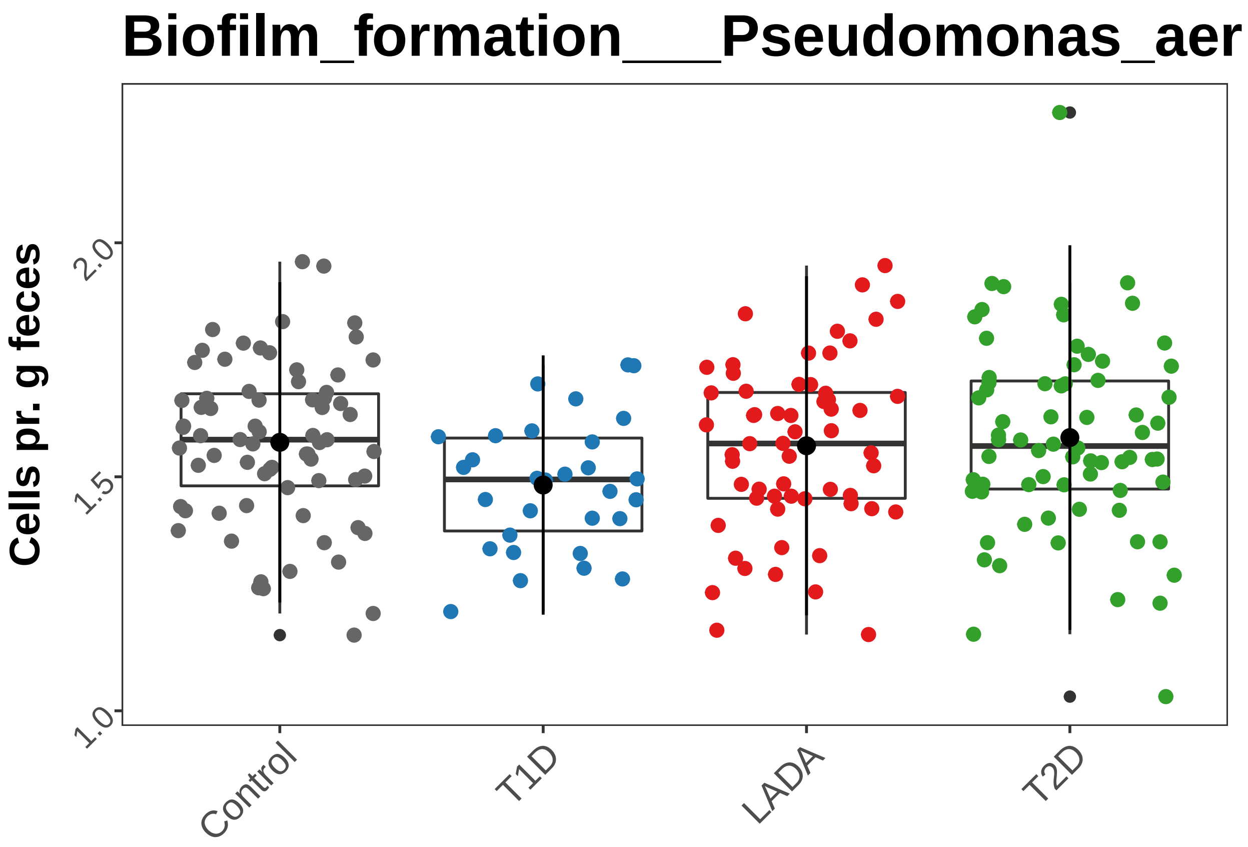

	pvalue	padj	Genus	compare
842	0.0048382	0.0794274	Ribosome_biogenesis_in_eukaryotes_PATH_ko03008	LADA vs T1D
645	0.0326435	0.3215380	Ribosome_biogenesis_in_eukaryotes_PATH_ko03008	T1D vs T2D
54	0.0345688	0.3474094	Ribosome_biogenesis_in_eukaryotes_PATH_ko03008	Control vs T1D
448	0.3271977	0.4786823	Ribosome_biogenesis_in_eukaryotes_PATH_ko03008	LADA vs Control
1039	0.3981702	0.9515738	Ribosome_biogenesis_in_eukaryotes_PATH_ko03008	LADA vs T2D
251	0.8916443	0.9946959	Ribosome_biogenesis_in_eukaryotes_PATH_ko03008	Control vs T2D

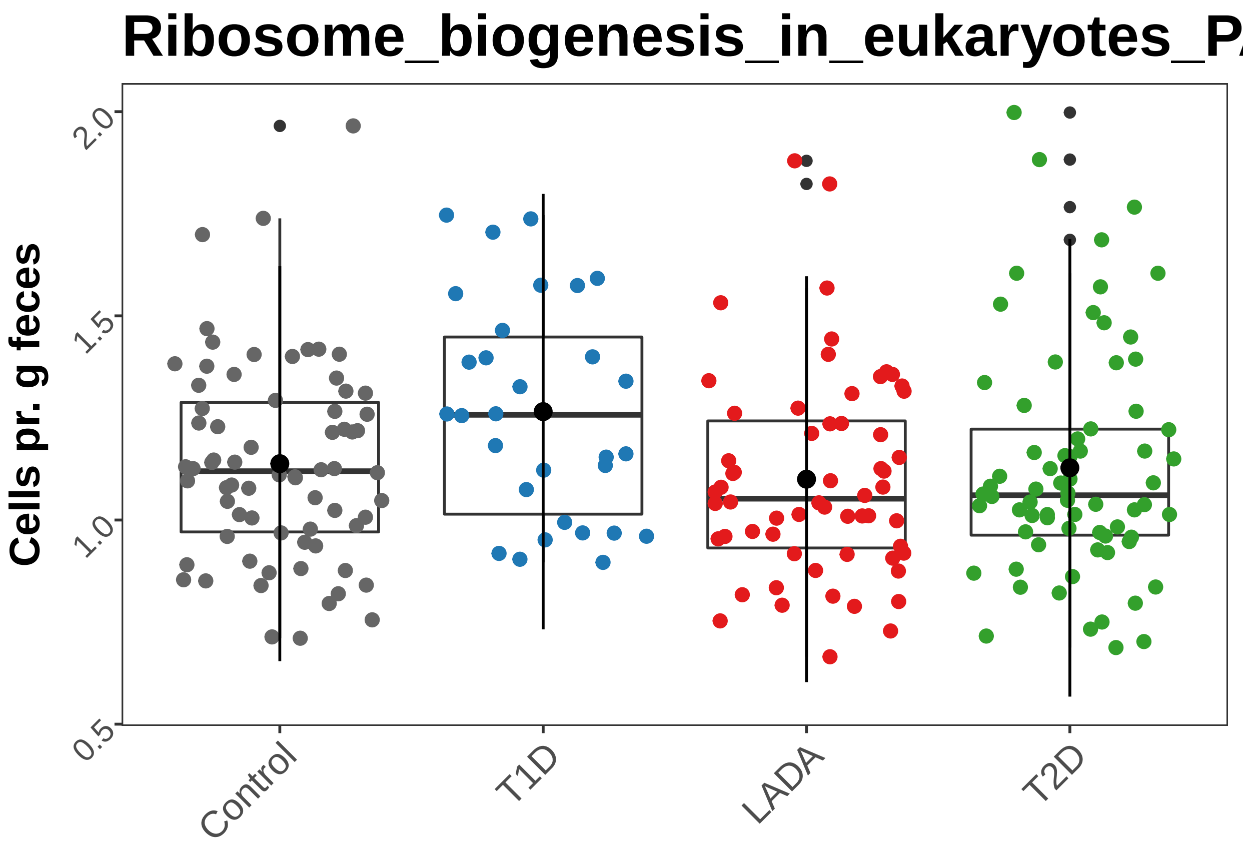

	pvalue	padj	Genus	compare
586	0.0064082	0.0903513	Biosynthesis_of_terpenoids_and_steroids	LADA vs Control
192	0.3967237	0.5746660	Biosynthesis_of_terpenoids_and_steroids	Control vs T1D
980	0.1789222	0.6535682	Biosynthesis_of_terpenoids_and_steroids	LADA vs T1D
389	0.1431341	0.8082167	Biosynthesis_of_terpenoids_and_steroids	Control vs T2D
1177	0.2114413	0.9515738	Biosynthesis_of_terpenoids_and_steroids	LADA vs T2D
783	0.7232565	0.9781873	Biosynthesis_of_terpenoids_and_steroids	T1D vs T2D

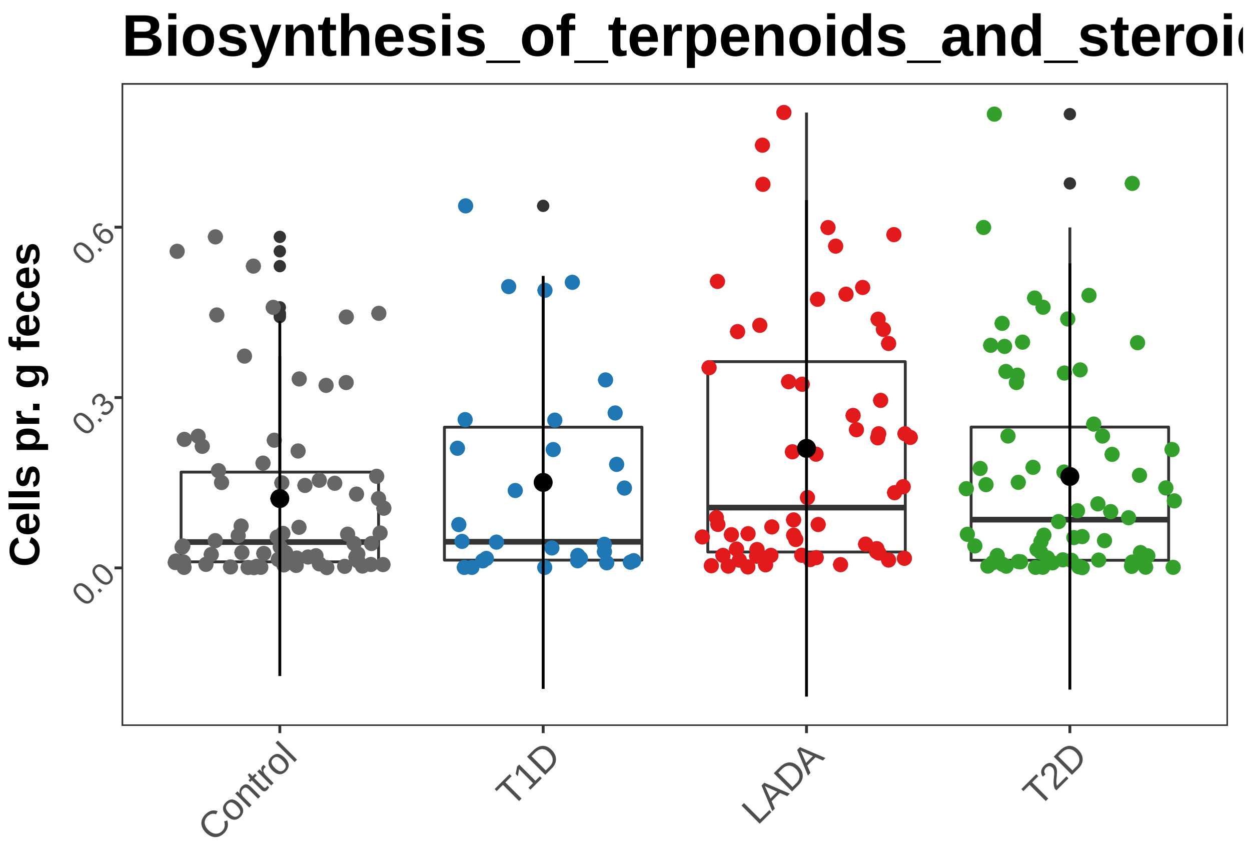

	pvalue	padj	Genus	compare
516	0.0064209	0.0903513	Arachidonic_acid_metabolism_PATH_ko00590	LADA vs Control
319	0.0074259	0.1219090	Arachidonic_acid_metabolism_PATH_ko00590	Control vs T2D
910	0.1061270	0.4751596	Arachidonic_acid_metabolism_PATH_ko00590	LADA vs T1D
713	0.1114670	0.6324066	Arachidonic_acid_metabolism_PATH_ko00590	T1D vs T2D
122	0.5717637	0.7139235	Arachidonic_acid_metabolism_PATH_ko00590	Control vs T1D
1107	0.9855521	0.9959905	Arachidonic_acid_metabolism_PATH_ko00590	LADA vs T2D

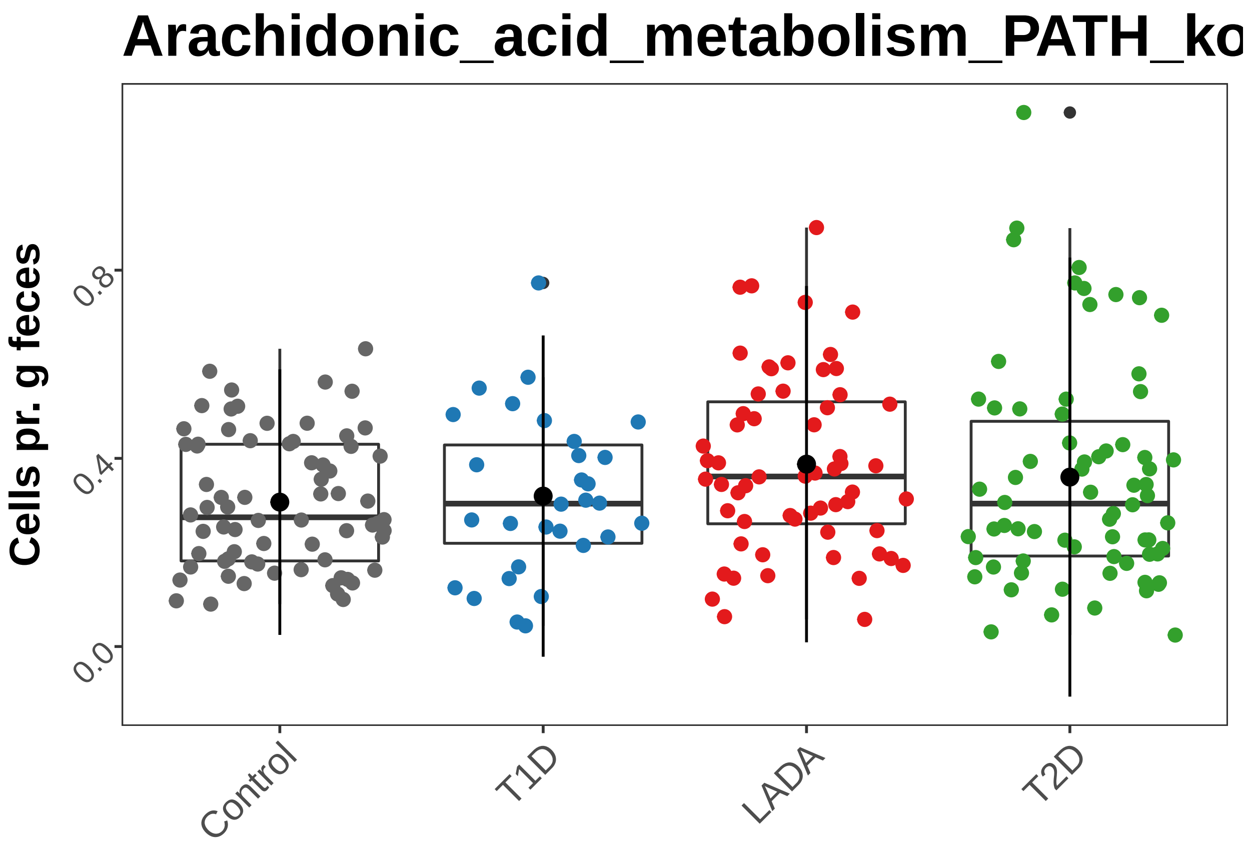

	pvalue	padj	Genus	compare
402	0.0069920	0.0918277	p53__signaling__pathway__PATH__ko04115	LADA vs Control
796	0.0258031	0.2541601	p53__signaling__pathway__PATH__ko04115	LADA vs T1D
205	0.0419884	0.5169818	p53__signaling__pathway__PATH__ko04115	Control vs T2D
599	0.0870940	0.6324066	p53__signaling__pathway__PATH__ko04115	T1D vs T2D
8	0.9248012	0.9488846	p53__signaling__pathway__PATH__ko04115	Control vs T1D
993	0.5207974	0.9515738	p53__signaling__pathway__PATH__ko04115	LADA vs T2D

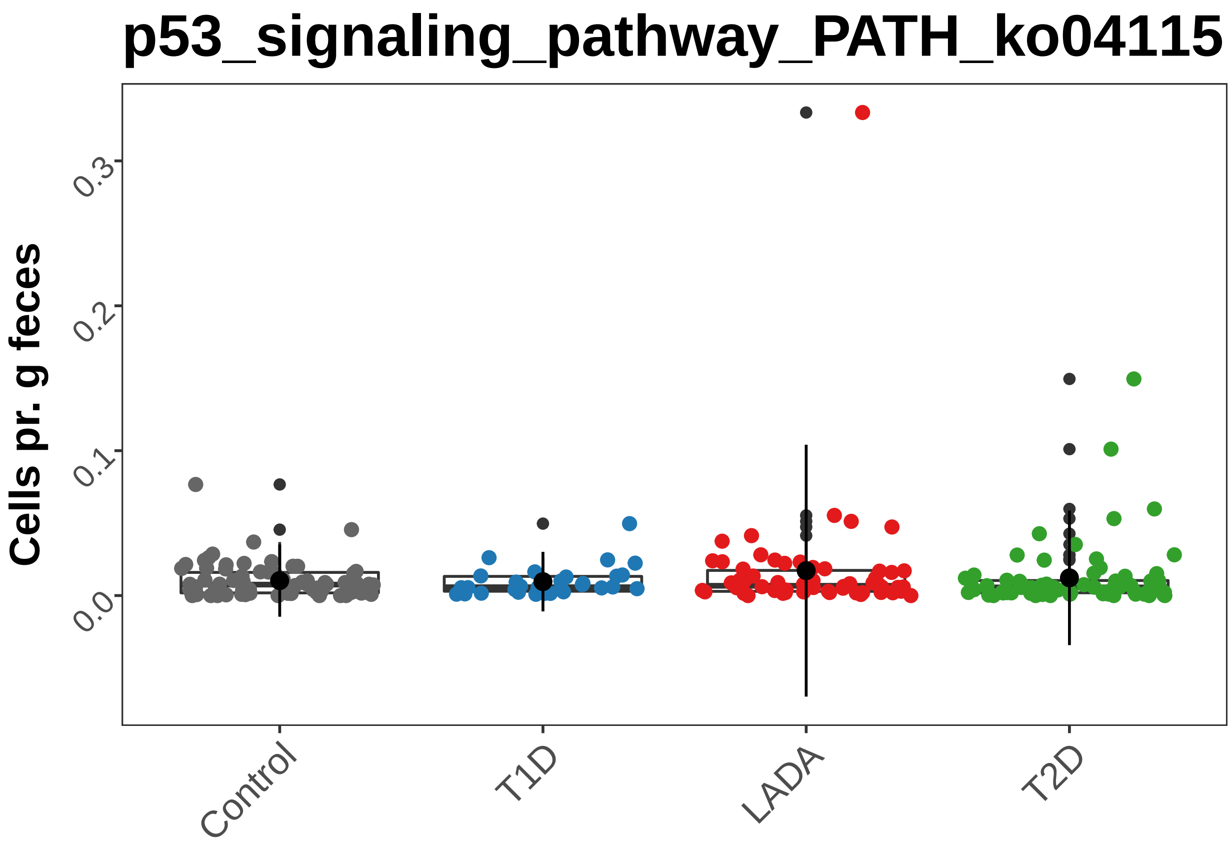


```
lay <- rbind(c(1,2,3))
pdf(paste("MicroLADA_FuncDA.pdf", sep=""), width=15, height=5)
grid.arrange(Fig2List$Geraniol_degradation_PATH_ko00281,
             Fig2List$Carotenoid_biosynthesis_PATH_ko00906,
             Fig2List$Retinol_metabolism_PATH_ko00830,layout_matrix = lay)
dev.off()
```

pdf 2

```
#ggplot(Plotting2, aes(x=BMI, y=Actinomyces)) +
#  geom_point()
```

DESeq Wald Remove metformin

All pairwise Differential abundance analysis comparison (DESeq and visualized venn)

```
rm(list=setdiff(ls(), c("Metadata", "MetadataMed", "Feature", "TaxonomyDA", "Taxonomy", "Fig2List")))
#Create a list to hold the plot objects.
Fig2ListRemMet <- list()

#Reassign names
#Metadata2<-Metadata
Taxonomy2<-TaxonomyDA
```

```
##Removing treated with metformin
#Metadata$Metformin <- ifelse(is.na(Metadata$Metformin), "Unknown", Metadata$Metformin)
Metadata2<-filter(Metadata, !Metformin == 1)
##Applying subsetting to OTU tables
Taxonomy2<-dplyr::select(Taxonomy2, one_of(as.character(Metadata2$MicrobiomeID)))
##Remove orgs that are not present after subsetting.
Taxonomy2 <- Taxonomy2[rowSums(Taxonomy2)>0,]



#Order Diagnosis
Metadata2$Diagnosis<-ordered(Metadata2$Diagnosis,
                             levels=c("Control", "T1D", "T2D", "LADA"))
#The factor can not be ordered for DESeq to run
Metadata2$Diagnosis<-factor(Metadata2$Diagnosis, ordered=FALSE)


#Create design formula
design <- formula(paste("~ ", "Diagnosis")) #To be consistent the analysis with metformin
  #removed is always without correcting for BMI
#design <- formula(paste("~ ", "Diagnosis", "+",  "BMIq"))
#design <- formula(paste("~ ", "Diagnosis", "+ Metformin"))
#Create DESeq2 object with matrix and metadata
dds <- DESeqDataSetFromMatrix(countData = Taxonomy2,
                              colData = Metadata2,
                              design = design)


##Assess and calculate size factors using the different methods
SF<-data.frame(Ratio=sizeFactors(estimateSizeFactors(dds, type="ratio")),
               Poscounts=sizeFactors(estimateSizeFactors(dds, type="poscounts")),
               #"iterate" takes a lot of time changed to "poscounts" but kept due to the
               #following code
               Iterate=sizeFactors(estimateSizeFactors(dds, type="poscounts")),
               Relative=colSums(Taxonomy2)/mean(colSums(Taxonomy2))
               )
SF<-add_rownames(SF, var="MicrobiomeID")
#Adding total abundances
# old path path<-paste("Q:/",
#             "Projects/",
#             "LADA/",
#             "LADA_Sandra_Evelina/",
#             "LADA_JKV/",
#             "LADA_R_AfterFlow_Analysis_FinalCounts/",
#             "LADA_FinalCounts/",
#             "2019-09-03_CountAnalysis.rds", sep="")

path <- paste("J:/",
              "CBMR/",
              "SUN-CBMR-Hansen-Group/",
              "Projects/",
              "LADA/",
              "LADA_Sandra_Evelina/",
```

```
              "LADA_JKV/",
              "LADA_R_AfterFlow_Analysis_FinalCounts/",
              "LADA_FinalCounts/",
              "2019-09-03_CountAnalysis.rds", sep="")

TotalCounts<-readRDS(path)
TotalCountsProcess <-
  data.frame(MicrobiomeID=TotalCounts$DF_Save_w$ID,
             CellNorm=TotalCounts$DF_Save_w$Cells_per_g_feces_FR_corrected_mean)
TotalCountsProcess$MicrobiomeID <- paste("L", TotalCountsProcess$MicrobiomeID,
                                         sep="")
SF2<-merge(SF, TotalCountsProcess, by="MicrobiomeID")

#One value is NaN in sample 602059 assigns value as average
SF2$CellNorm[is.nan(SF2$CellNorm)]<-mean(na.omit(SF2$CellNorm))

SF2$CellNormStand<-SF2$CellNorm/mean(SF2$CellNorm) #Watch out for NaN values can assign
                                                   #average, but wants the error
SF3<-merge(Metadata2, SF2, by="MicrobiomeID")
#DESeq divides with sizeFactor (High cell count should have low sizeFactor providing
#larger counts in a sample)
#Still have to consider seq data is relative
SF3$NormRelCell<-SF3$Relative/SF3$CellNormStand
#Overview of normalization factors
aggregate(SF3[, 14:20], list(SF3$Diagnosis), mean) #Have to run heatmap chunk adding sex
```

Group.1 Ratio Poscounts Iterate Relative CellNorm CellNormStand 1 Control 1.094542 1.087591 1.087591 1.0032044 20384275367 1.0722125 2 T1D 1.131654 1.127744 1.127744 1.0416740 18731782275 0.9852914 3 T2D 1.069550 1.065472 1.065472 0.9806434 16982538343 0.8932812 4 LADA 1.004481 0.998413 0.998413 0.9142228 15590817082 0.8200767 NormRelCell 1 1.002356 2 1.193649 3 1.191757 4 1.193676

```
                                                   #to metadata
aggregate(SF3[, 14:20], list(SF3$Diagnosis), sd)
```

Group.1 Ratio Poscounts Iterate Relative CellNorm CellNormStand 1 Control 0.4695326 0.4639309 0.4639309 0.4337118 6781846681 0.3567250 2 T1D 0.4106770 0.4065139 0.4065139 0.3886448 5712095609 0.3004561 3 T2D 0.3912487 0.3857159 0.3857159 0.3582493 4888235680 0.2571211 4 LADA 0.3345323 0.3282978 0.3282978 0.3071066 4604942130 0.2422199 NormRelCell 1 0.4688179 2 0.6676588 3 0.5509790 4 0.5263781

```
pairs(SF3[, c(14:17,19,20)], col=SF3$Diagnosis)
```

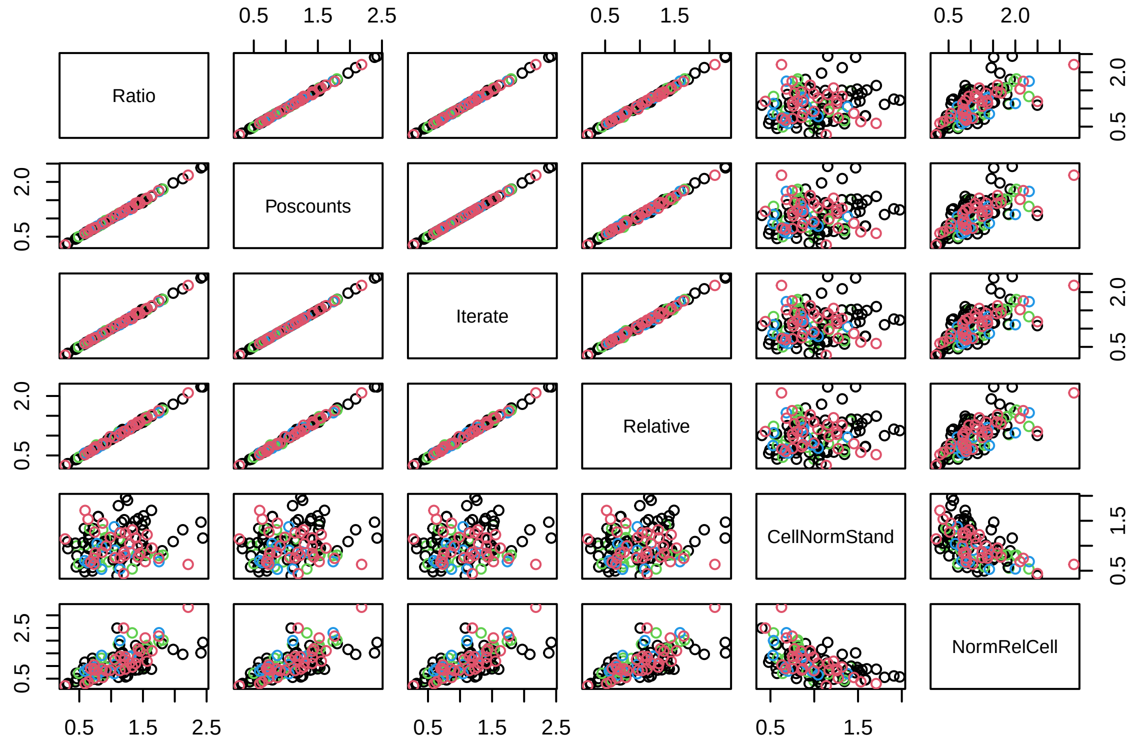


```
pairs(SF3[, c(15,17,19,20)], col=SF3$Diagnosis)
```

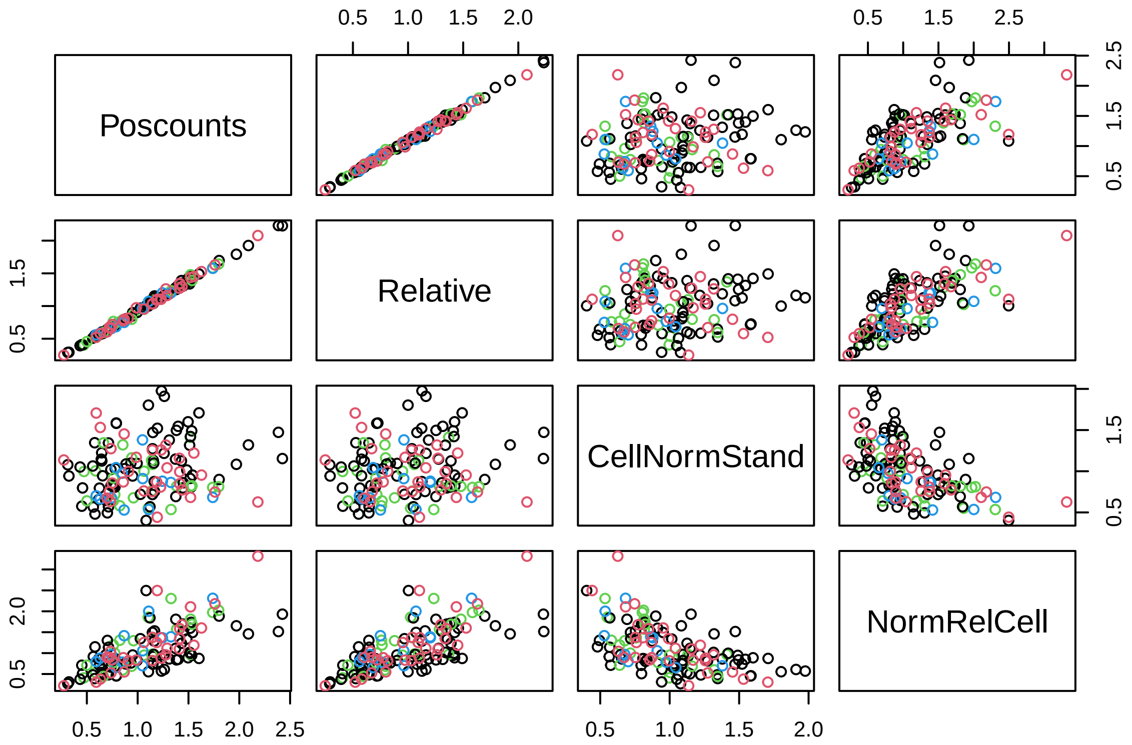


```
#I do want CellNormStand and NormRelCell to be negatively correlated due to DESeq divide
#with sizeFactor.
#It is also good that CellNormStand seems uncorrelated to the other normalization factors.

##Select size factors calculated above for normalization
dds@colData@listData$sizeFactor <- SF3[,20]

##See vignette for note on factor levels
#Filtering of the Counttable. Bare minimum is performed later. Also to reduce memory of

#the dds
dds <- dds[ rowSums(counts(dds))>(50*length(Taxonomy2)),] #Previously 0 instead of 1

#Estimate dispersions and fit the GLM
dds <- DESeq(dds)

res<-results(dds)
summary(res)
```

out of 196 with nonzero total read count adjusted p-value < 0.1 LFC > 0 (up) : 10, 5.1% LFC < 0 (down) : 109, 56% outliers [1] : 0, 0% low counts [2] : 0, 0% (mean count < 62) [1] see 'cooksCutoff' argument of ?results [2] see 'independentFiltering' argument of ?results

```
resultsNames(dds)
```

[1] "Intercept" "Diagnosis_T1D_vs_Control" [3] "Diagnosis_T2D_vs_Control" "Diagnosis_LADA_vs_Control"

```r
#Threshold for FDR-adjusted p-values
padj_threshold <- 0.1

if (is.factor(colData(dds)[,"Diagnosis"]) ==  TRUE) {

#Find all two-wise combinations of levels in chosen test factor
test <- combn(levels(colData(dds)[,"Diagnosis"]), 2)
test[,3]<-c("LADA", "Control") #Want LADA first
test[,5]<-c("LADA", "T1D") #Want LADA first
test[,6]<-c("LADA", "T2D") #Want LADA first
foo = test[1,]
poo = test[2,]

#Do DESeq2 analysis on all combinations of levels and save as list
result <- vector("list",length(foo))
for (i in 1:length(foo) ) {
  for (j in 1:length(poo)) {
    result[i] = results(dds, contrast = c("Diagnosis" ,foo[i], poo[i]))
  }
}

#Produce output tables with significant taxa from each comparison
res_list <- vector("list",length(foo))
for (i in 1:length(result) ) {
  res_stat <- data.frame(result[i])
  res_stat <- res_stat[is.na(res_stat$pvalue) == FALSE,]
  stat_sig <- na.omit(res_stat[res_stat$padj < padj_threshold,])
  res_stat$gene <- rownames(res_stat)
  res_stat$g1 <- test[1,i]
  res_stat$g2 <- test[2,i]
  res_stat$compare <- paste(test[1,i], "vs", test[2,i])
  res_list[i] <- list(res_stat)
}

res_stat <- do.call('rbind', res_list)
rownames(res_stat) <- 1:nrow(res_stat)
res_stat <- res_stat[with(res_stat, order(padj, pvalue, abs(log2FoldChange))),]
stat_sig <- na.omit(res_stat[res_stat$padj < padj_threshold,])
}

if (is.numeric(colData(dds)[,"Diagnosis"]) ==  TRUE) {

res_stat <- data.frame((results(dds)))
res_stat$maxCooks <- apply(assays(dds)[["cooks"]], 1, max)
res_stat <- res_stat[is.na(res_stat$pvalue) == FALSE,]
res_stat <- res_stat[with(res_stat, order(padj, pvalue, abs(log2FoldChange))), ]
res_stat$gene <- rownames(res_stat)
max_cooks <- quantile(na.omit(res_stat$maxCooks), cooks_quantile_cutoff)
stat_sig <-
  na.omit(res_stat[res_stat$padj < padj_threshold & res_stat$maxCooks < max_cooks,])
}

#All comparisons
```

```r
write.table(res_stat, file="DESeqRes_OrgsRemMet_Func.txt", quote = F, row.names = F, sep="\t")

#Significant taxa in table
if (nrow(stat_sig) > 0) {
kable(stat_sig, row.names = F)
write.table(stat_sig, file="DESeqRes_SigOrgsRemMet_Func.txt", quote = F, row.names = F, sep="\t")
} else {
  print("No significant taxa were found.")
}
print("Significant Wald")
```

[1] "Significant Wald"

```r
kable(stat_sig)
```

	baseMean	log2FoldChange	lfcSE	stat	pvalue	padj	gene	g1	g2	compare
895	218.4148	3.0165381	0.7294476	4.135370	0.0000354	0.0069458	Glycosphingolipid biosynthesis - lacto and neolacto series [PATH:ko00601]	LADA	T1D	LADA vs T1D
552	995.3726	1.8495682	0.4867835	3.799578	0.0001449	0.0162870	Geraniol degradation [PATH:ko00281]	LADA	Control	LADA vs Control
512	726.8127	1.8839190	0.5003060	3.765530	0.0001662	0.0162870	alpha-Linolenic acid metabolism [PATH:ko00592]	LADA	Control	LADA vs Control
571	498.1493	1.9506775	0.5788543	3.369890	0.0007520	0.0333090	Ethylbenzene degradation [PATH:ko00642]	LADA	Control	LADA vs Control
466	20233.1183	-0.4952563	0.1516660	-3.265441	0.0010929	0.0333090	Glucosinolate biosynthesis [PATH:ko00966]	LADA	Control	LADA vs Control
459	214043.7487	-0.4659223	0.1462785	-3.185173	0.0014467	0.0333090	Valine, leucine and isoleucine biosynthesis [PATH:ko00290]	LADA	Control	LADA vs Control
579	404909.1603	-0.4656151	0.1469976	-3.167502	0.0015375	0.0333090	2-Oxocarboxylic acid metabolism	LADA	Control	LADA vs Control
472	28826.6477	-0.4930175	0.1560719	-3.158913	0.0015836	0.0333090	Phenazine biosynthesis [PATH:ko00405]	LADA	Control	LADA vs Control
515	217.3927	1.6211457	0.5139137	3.154522	0.0016076	0.0333090	Ether lipid metabolism [PATH:ko00565]	LADA	Control	LADA vs Control

	baseMean	log2FoldChange	lfcSE	stat	pvalue	padj	gene	g1	g2	compare
536	224290.1230	-0.4588622	0.1456522	-3.150396	0.0016305	0.0333090	Thiamine metabolism [PATH:ko00730]	LADA	Control	LADA vs Control
456	333727.4578	-0.4561018	0.1468106	-3.106737	0.0018916	0.0333090	Phenylalanine, tyrosine and tryptophan biosynthesis [PATH:ko00400]	LADA	Control	LADA vs Control
532	247957.9855	-0.4433762	0.1437419	-3.084531	0.0020387	0.0333090	Pantothenate and CoA biosynthesis [PATH:ko00770]	LADA	Control	LADA vs Control
580	1815934.8143	-0.4477558	0.1451658	-3.084445	0.0020393	0.0333090	Biosynthesis of amino acids	LADA	Control	LADA vs Control
480	126907.2600	-0.4531856	0.1488881	-3.043801	0.0023361	0.0346055	C5-Branched dibasic acid metabolism [PATH:ko00660]	LADA	Control	LADA vs Control
497	157444.2964	-0.4779052	0.1587955	-3.009563	0.0026162	0.0346055	Photosynthesis [PATH:ko00195]	LADA	Control	LADA vs Control
449	233417.0192	-0.4330230	0.1463935	-2.957939	0.0030970	0.0346055	Arginine biosynthesis [PATH:ko00220]	LADA	Control	LADA vs Control
518	145328.6061	-0.4551536	0.1558211	-2.921000	0.0034891	0.0346055	Glycerolipid metabolism [PATH:ko00561]	LADA	Control	LADA vs Control
488	325138.2598	-0.4317065	0.1483497	-2.910059	0.0036106	0.0346055	Pentose phosphate pathway [PATH:ko00030]	LADA	Control	LADA vs Control
442	1242724.7973	-0.4305298	0.1483038	-2.903027	0.0036957	0.0346055	Aminoacyl-tRNA biosynthesis [PATH:ko00970]	LADA	Control	LADA vs Control
453	252348.0759	-0.4249807	0.1466721	-2.897489	0.0037606	0.0346055	Lysine biosynthesis [PATH:ko00300]	LADA	Control	LADA vs Control
581	2443269.7600	-0.4135049	0.1447959	-2.855779	0.0042931	0.0346055	Biosynthesis of antibiotics	LADA	Control	LADA vs Control
475	139233.4459	-0.4466294	0.1566944	-2.850322	0.0043675	0.0346055	Streptomycin biosynthesis [PATH:ko00521]	LADA	Control	LADA vs Control

	baseMean	log2FoldChange	lfcSE	stat	pvalue	padj	gene	g1	g2	compare
558	181462.8427	-0.4128674	0.1453436	-2.840630	0.0045024	0.0346055	Terpenoid backbone biosynthesis [PATH:ko00900]	LADA	Control	LADA vs Control
439	146391.9670	-0.4323084	0.1530901	-2.823883	0.0047406	0.0346055	Nucleotide excision repair [PATH:ko03420]	LADA	Control	LADA vs Control
546	141097.1809	-0.4071760	0.1443530	-2.820697	0.0047909	0.0346055	Selenocompound metabolism [PATH:ko00450]	LADA	Control	LADA vs Control
492	221545.5958	-0.4243774	0.1505185	-2.819436	0.0048108	0.0346065	Carbon fixation in photosynthetic organisms [PATH:ko00710]	LADA	Control	LADA vs Control
450	402736.7400	-0.4031390	0.1433237	-2.812788	0.0049104	0.0346065	Cysteine and methionine metabolism [PATH:ko00270]	LADA	Control	LADA vs Control
464	31621.9628	-0.4346740	0.1553161	-2.798641	0.0051308	0.0346065	Carbapenem biosynthesis [PATH:ko00332]	LADA	Control	LADA vs Control
582	3152810.7079	-0.4012594	0.1436862	-2.792610	0.0052285	0.0346055	Biosynthesis of secondary metabolites	LADA	Control	LADA vs Control
434	248750.5462	-0.4191483	0.1506456	-2.782346	0.0053967	0.0346055	DNA replication [PATH:ko03030]	LADA	Control	LADA vs Control
437	295711.6414	-0.4057619	0.1460507	-2.778227	0.0054656	0.0346055	Mismatch repair [PATH:ko03430]	LADA	Control	LADA vs Control
556	80950.4860	-0.4568832	0.1655288	-2.760143	0.0057776	0.0346055	Polyketide sugar unit biosynthesis [PATH:ko00523]	LADA	Control	LADA vs Control
588	1850407.2352	-0.4094011	0.1485772	-2.755477	0.0058607	0.0346055	Microbial metabolism in diverse environments	LADA	Control	LADA vs Control
399	75913.7098	-0.4183935	0.1522231	-2.748555	0.0059859	0.0346055	Necroptosis [PATH:ko04217]	LADA	Control	LADA vs Control
468	106341.5172	-0.4052658	0.1483929	-2.731032	0.0063106	0.0346055	Monobactam biosynthesis [PATH:ko00261]	LADA	Control	LADA vs Control

	baseMean	log2FoldChange	lfcSE	stat	pvalue	padj	gene	g1	g2	compare
510	292604.4221	-0.3959879	0.1460985	-2.710418	0.0067198	0.0346055	Peptidoglycan biosynthesis [PATH:ko00550]	LADA	Control	LADA vs Control
452	176722.9416	-0.4026854	0.1486319	-2.709280	0.0067429	0.0346055	Histidine metabolism [PATH:ko00340]	LADA	Control	LADA vs Control
560	809598.0466	-0.3928493	0.1460364	-2.690078	0.0071435	0.0346055	Purine metabolism [PATH:ko00230]	LADA	Control	LADA vs Control
451	329925.6733	-0.3953180	0.1471814	-2.685923	0.0072330	0.0346055	Glycine, serine and threonine metabolism [PATH:ko00260]	LADA	Control	LADA vs Control
429	219109.5944	-0.3886034	0.1449681	-2.680613	0.0073488	0.0346055	Protein export [PATH:ko03060]	LADA	Control	LADA vs Control
447	372509.6882	-0.3955061	0.1477005	-2.677757	0.0074107	0.0346055	Alanine, aspartate and glutamate metabolism [PATH:ko00250]	LADA	Control	LADA vs Control
587	6821207.1504	-0.3837743	0.1444684	-2.656459	0.0078966	0.0346055	Metabolic pathways	LADA	Control	LADA vs Control
561	644062.4895	-0.3886689	0.1469399	-2.645088	0.0081670	0.0346055	Pyrimidine metabolism [PATH:ko00240]	LADA	Control	LADA vs Control
503	218.4148	1.7624287	0.6668258	2.643010	0.0082172	0.0346055	Glycosphingolipid biosynthesis - lacto and neolacto series [PATH:ko00601]	LADA	Control	LADA vs Control
470	51850.3070	-0.3908660	0.1483092	-2.635480	0.0084018	0.0346055	Novobiocin biosynthesis [PATH:ko00401]	LADA	Control	LADA vs Control
444	964710.8102	-0.4001222	0.1519152	-2.633852	0.0084422	0.0346055	Ribosome [PATH:ko03010]	LADA	Control	LADA vs Control
543	53072.2312	-0.3931689	0.1495685	-2.628688	0.0085715	0.0346055	D-Glutamine and D-glutamate metabolism [PATH:ko00471]	LADA	Control	LADA vs Control
484	470076.0858	-0.3865503	0.1471068	-2.627685	0.0085968	0.0346055	Glycolysis / Gluconeogenesis [PATH:ko00010]	LADA	Control	LADA vs Control

	baseMean	log2FoldChange	lfcSE	stat	pvalue	padj	gene	g1	g2	compare
406	609155.5539	-0.4151082	0.1582191	-2.623630	0.0086998	0.0346055	Quorum sensing [PATH:ko02024]	LADA	Control	LADA vs Control
548	39830.4805	-0.4685157	0.1800728	-2.601813	0.0092733	0.0346055	Biosynthesis of ansamycins [PATH:ko01051]	LADA	Control	LADA vs Control
584	1023857.9733	-0.3928505	0.1510695	-2.600463	0.0093098	0.0346055	Carbon metabolism	LADA	Control	LADA vs Control
538	77161.2179	-0.3889528	0.1496063	-2.599843	0.0093267	0.0346055	Vitamin B6 metabolism [PATH:ko00750]	LADA	Control	LADA vs Control
395	156155.0223	-0.3820294	0.1470148	-2.598577	0.0093611	0.0346055	Cell cycle - Caulobacter [PATH:ko04112]	LADA	Control	LADA vs Control
441	69732.4972	-0.4853486	0.1871100	-2.593922	0.0094888	0.0346055	RNA polymerase [PATH:ko03020]	LADA	Control	LADA vs Control
431	192358.4294	-0.3858619	0.1489797	-2.590029	0.0095968	0.0346055	RNA degradation [PATH:ko03018]	LADA	Control	LADA vs Control
530	170759.3562	-0.3864684	0.1497340	-2.581032	0.0098505	0.0346055	Nicotinate and nicotinamide metabolism [PATH:ko00760]	LADA	Control	LADA vs Control
436	326813.5597	-0.3893499	0.1509256	-2.579746	0.0098873	0.0346055	Homologous recombination [PATH:ko03440]	LADA	Control	LADA vs Control
854	234.0752	2.4289121	0.6950592	3.494540	0.0004749	0.0358391	Betalain biosynthesis [PATH:ko00965]	LADA	T1D	LADA vs T1D
837	34492.8040	-1.1280988	0.3264315	-3.455852	0.0005486	0.0358391	Ribosome biogenesis in eukaryotes [PATH:ko03008]	LADA	T1D	LADA vs T1D
432	102284.1173	-0.4010417	0.1572148	-2.550916	0.0107440	0.0369484	Sulfur relay system [PATH:ko04122]	LADA	Control	LADA vs Control
486	52178.4796	-0.4679973	0.1839627	-2.543980	0.0109597	0.0369488	Inositol phosphate metabolism [PATH:ko00562]	LADA	Control	LADA vs Control

	baseMean	log2FoldChange	lfcSE	stat	pvalue	padj	gene	g1	g2	compare
483	375800.7897	-0.3957822	0.1558915	-2.538832	0.0111203	0.0369468	Galactose metabolism [PATH:ko00052]	LADA	Control	LADA vs Control
489	229437.8083	-0.3745192	0.1484285	-2.523229	0.0116283	0.0379856	Propanoate metabolism [PATH:ko00640]	LADA	Control	LADA vs Control
478	54800.4783	-0.5239446	0.2089664	-2.507315	0.0121652	0.0386411	Ascorbate and aldarate metabolism [PATH:ko00053]	LADA	Control	LADA vs Control
531	188159.1347	-0.3800339	0.1516718	-2.505634	0.0122232	0.0386401	One carbon pool by folate [PATH:ko00670]	LADA	Control	LADA vs Control
519	226187.9392	-0.3727765	0.1493114	-2.496638	0.0125377	0.0390061	Glycerophospholipid metabolism [PATH:ko00564]	LADA	Control	LADA vs Control
8	157791.6064	0.6905708	0.1888731	3.656309	0.0002559	0.0390382	Bacterial chemotaxis [PATH:ko02030]	Control	T1D	Control vs T1D
9	188508.8974	0.7082239	0.1999969	3.541175	0.0003983	0.0390382	Flagellar assembly [PATH:ko02040]	Control	T1D	Control vs T1D
461	39632.6542	-0.4103159	0.1647847	-2.490012	0.0127739	0.0391200	Acarbose and validamycin biosynthesis [PATH:ko00525]	LADA	Control	LADA vs Control
495	133882.2838	-0.3570076	0.1447994	-2.465532	0.0136810	0.0408927	Nitrogen metabolism [PATH:ko00910]	LADA	Control	LADA vs Control
482	372388.2684	-0.4299961	0.1745675	-2.463208	0.0137700	0.0408927	Fructose and mannose metabolism [PATH:ko00051]	LADA	Control	LADA vs Control
418	40343.0949	-0.3753999	0.1539671	-2.438183	0.0147613	0.0431823	HIF-1 signaling pathway [PATH:ko04066]	LADA	Control	LADA vs Control
462	234.0752	1.5430200	0.6355087	2.428008	0.0151820	0.0437599	Betalain biosynthesis [PATH:ko00965]	LADA	Control	LADA vs Control
423	33035.9265	-0.3821498	0.1581888	-2.415784	0.0157014	0.0442859	Phosphatidylinositol signaling system [PATH:ko04070]	LADA	Control	LADA vs Control

	baseMean	log2FoldChange	lfcSE	stat	pvalue	padj	gene	g1	g2	compare
412	1042044.9923	-0.3686654	0.1527751	-2.413125	0.0158104	0.0442859	ABC transporters [PATH:ko02010]	LADA	Control	LADA vs Control
433	143343.1025	-0.3559107	0.1480025	-2.404761	0.0161800	0.0446743	Base excision repair [PATH:ko03410]	LADA	Control	LADA vs Control
573	3114.1040	0.9598400	0.4012802	2.391940	0.0167594	0.0450038	Metabolism of xenobiotics by cytochrome P450 [PATH:ko00980]	LADA	Control	LADA vs Control
569	3116.3570	0.9594571	0.4011284	2.391895	0.0167606	0.0450038	Drug metabolism - cytochrome P450 [PATH:ko00982]	LADA	Control	LADA vs Control
491	536904.8335	-0.3622632	0.1542304	-2.348844	0.0188308	0.0493905	Starch and sucrose metabolism [PATH:ko00500]	LADA	Control	LADA vs Control
408	8775.0280	-0.6809803	0.2900864	-2.347509	0.0188994	0.0493905	Autophagy - yeast [PATH:ko04138]	LADA	Control	LADA vs Control
477	543686.3387	-0.3468003	0.1480588	-2.342315	0.0191645	0.0494243	Amino sugar and nucleotide sugar metabolism [PATH:ko00520]	LADA	Control	LADA vs Control
490	379607.9172	-0.3433669	0.1481374	-2.317894	0.0204551	0.0517856	Pyruvate metabolism [PATH:ko00620]	LADA	Control	LADA vs Control
476	35023.1733	-0.3449914	0.1490192	-2.315080	0.0206085	0.0517856	Tropane, piperidine and pyridine alkaloid biosynthesis [PATH:ko00960]	LADA	Control	LADA vs Control
550	21941.8889	-0.4000995	0.1738548	-2.301343	0.0213702	0.0530248	Biosynthesis of vancomycin group antibiotics [PATH:ko01055]	LADA	Control	LADA vs Control
533	223648.1833	-0.4086375	0.1792528	-2.279671	0.0226272	0.0549700	Porphyrin and chlorophyll metabolism [PATH:ko00860]	LADA	Control	LADA vs Control
516	177269.3156	-0.3498961	0.1535873	-2.278158	0.0227172	0.0549700	Fatty acid biosynthesis [PATH:ko00061]	LADA	Control	LADA vs Control
446	40338.5823	-0.5554690	0.2443756	-2.273013	0.0230254	0.0550363	RNA transport [PATH:ko03013]	LADA	Control	LADA vs Control

	baseMean	log2FoldChange	lfcSE	stat	pvalue	padj	gene	g1	g2	compare
586	192044.3923	-0.3431576	0.1520407	-2.257013	0.0240073	0.0566919	Fatty acid metabolism	LADA	Control	LADA vs Control
521	17743.1539	-0.4105272	0.1825900	-2.248355	0.0245535	0.0569158	Primary bile acid biosynthesis [PATH:ko00120]	LADA	Control	LADA vs Control
414	351515.7936	-0.4527736	0.2015615	-2.246330	0.0246829	0.0569158	Phosphotransferase system (PTS) [PATH:ko02060]	LADA	Control	LADA vs Control
445	34492.8040	-0.6681708	0.2985989	-2.237686	0.0252405	0.0574628	Ribosome biogenesis in eukaryotes [PATH:ko03008]	LADA	Control	LADA vs Control
539	51220.4380	-0.3536103	0.1588104	-2.226619	0.0259727	0.0574628	beta-Alanine metabolism [PATH:ko00410]	LADA	Control	LADA vs Control
479	201350.0983	-0.3309800	0.1489087	-2.222705	0.0262357	0.0574628	Butanoate metabolism [PATH:ko00650]	LADA	Control	LADA vs Control
485	251341.3035	-0.3321076	0.1494912	-2.221587	0.0263103	0.0574628	Glyoxylate and dicarboxylate metabolism [PATH:ko00630]	LADA	Control	LADA vs Control
522	17771.8975	-0.4042692	0.1823325	-2.217209	0.0266088	0.0574628	Secondary bile acid biosynthesis [PATH:ko00121]	LADA	Control	LADA vs Control
528	169835.3685	-0.3510131	0.1587020	-2.211776	0.0269822	0.0574628	Folate biosynthesis [PATH:ko00790]	LADA	Control	LADA vs Control
559	17603.1775	-0.3552200	0.1608332	-2.208623	0.0272008	0.0574628	Zeatin biosynthesis [PATH:ko00908]	LADA	Control	LADA vs Control
493	306154.1550	-0.3401062	0.1542894	-2.204340	0.0275005	0.0574628	Carbon fixation pathways in prokaryotes [PATH:ko00720]	LADA	Control	LADA vs Control
496	328532.7684	-0.3332191	0.1512218	-2.203512	0.0275587	0.0574628	Oxidative phosphorylation [PATH:ko00190]	LADA	Control	LADA vs Control
236	102284.1173	0.3485672	0.1209359	2.882247	0.0039485	0.062948[illegible]	Sulfur relay system [PATH:ko04122]	Control	T2D	Control vs T2D

	baseMean	log2FoldChange	lfcSE	stat	pvalue	padj	gene	g1	g2	compare
322	145328.60	0.3444981	0.119863	2.874080	0.0040520	0.0629443	Glycerolipid metabolism [PATH:ko00561]	Control	T2D	Control vs T2D
366	19177.758	0.4220452	0.147242	2.866328	0.0041506	0.0629443	Aminobenzoate degradation [PATH:ko00627]	Control	T2D	Control vs T2D
230	565135.99	0.3623313	0.127625	2.839014	0.0045253	0.0629443	Two-component system [PATH:ko02020]	Control	T2D	Control vs T2D
207	139018.78	0.3767423	0.132998	2.832686	0.0046159	0.0629443	Biofilm formation - Escherichia coli [PATH:ko02026]	Control	T2D	Control vs T2D
314	292604.40	0.3158246	0.112384	2.810210	0.0049509	0.0629443	Peptidoglycan biosynthesis [PATH:ko00550]	Control	T2D	Control vs T2D
323	226187.93	0.3207975	0.114856	2.793036	0.0052206	0.0629443	Glycerophospholipid metabolism [PATH:ko00564]	Control	T2D	Control vs T2D
301	157444.29	0.3380870	0.122151	2.767764	0.0056402	0.0629443	Photosynthesis [PATH:ko00195]	Control	T2D	Control vs T2D
218	351515.79	0.4290365	0.155049	2.767103	0.0056557	0.0629443	Phosphotransferase system (PTS) [PATH:ko02060]	Control	T2D	Control vs T2D
352	39830.480	0.3814512	0.138519	2.753779	0.0058901	0.0629443	Biosynthesis of ansamycins [PATH:ko01051]	Control	T2D	Control vs T2D
302	165767.91	0.3393162	0.126231	2.688039	0.0071873	0.0629443	Sulfur metabolism [PATH:ko00920]	Control	T2D	Control vs T2D
370	23793.304	0.3320518	0.125492	2.645942	0.0081464	0.0629443	Chloroalkane and chloroalkene degradation [PATH:ko00625]	Control	T2D	Control vs T2D
336	247957.98	0.2901592	0.110571	2.624170	0.0086860	0.0629443	Pantothenate and CoA biosynthesis [PATH:ko00770]	Control	T2D	Control vs T2D
288	470076.08	0.2939371	0.113160	2.597529	0.0093897	0.0629443	Glycolysis / Gluconeogenesis [PATH:ko00010]	Control	T2D	Control vs T2D
345	36295.183	0.3008855	0.116178	2.589864	0.0096014	0.0629443	D-Alanine metabolism [PATH:ko00473]	Control	T2D	Control vs T2D
340	224290.12	0.2899024	0.112041	2.587461	0.0096686	0.0629443	Thiamine metabolism [PATH:ko00730]	Control	T2D	Control vs T2D
203	75913.709	0.3021866	0.117095	2.580676	0.0098607	0.0629443	Necroptosis [PATH:ko04217]	Control	T2D	Control vs T2D
296	221545.59	0.2973814	0.115784	2.568400	0.0102169	0.0629443	Carbon fixation in photosynthetic organisms [PATH:ko00710]	Control	T2D	Control vs T2D

	baseMean	log2FoldChange	lfcSE	stat	pvalue	padj	gene	g1	g2	compare
276	28826.6407730726890.120055275593870.010485706294413						Phenazine biosynthesis [PATH:ko00405]	Control	T2D	Control vs T2D
241	295711.6412861342 0.112347285468590.010869706294413						Mismatch repair [PATH:ko03430]	Control	T2D	Control vs T2D
216	1042044.92286446 0.117520265412100.011046906294413						ABC transporters [PATH:ko02010]	Control	T2D	Control vs T2D
263	214043.7412784037 20.112523305242590.011594030629413						Valine, leucine and isoleucine biosynthesis [PATH:ko00290]	Control	T2D	Control vs T2D
286	372388.2063437745 50.134284215151560.011898006294413						Fructose and mannose metabolism [PATH:ko00051]	Control	T2D	Control vs T2D
295	536904.8332978421 0.118640215104670.012057106294413						Starch and sucrose metabolism [PATH:ko00500]	Control	T2D	Control vs T2D
343	51220.438303055463 0.122163365011240.012380006294413						beta-Alanine metabolism [PATH:ko00410]	Control	T2D	Control vs T2D
272	106341.50172853569 0.114149264998500.012424606294413						Monobactam biosynthesis [PATH:ko00261]	Control	T2D	Control vs T2D
233	219109.5924784597 0.111515214970580.012522906294413						Protein export [PATH:ko03060]	Control	T2D	Control vs T2D
260	333727.4557816193 0.112932234937000.012640906294413						Phenylalanine, tyrosine and tryptophan biosynthesis [PATH:ko00400]	Control	T2D	Control vs T2D
384	1815934.8124782626 0.111667214918930.012706406294413						Biosynthesis of amino acids	Control	T2D	Control vs T2D
208	75850.0003276914 0.131832748566 0.012930106294413						Biofilm formation - Pseudomonas aeruginosa [PATH:ko02025]	Control	T2D	Control vs T2D
350	141097.1802749259 0.111041294758760.013290006294413						Selenocompound metabolism [PATH:ko00450]	Control	T2D	Control vs T2D
237	143343.10275779836 0.113849244416780.014619206294413						Base excision repair [PATH:ko03410]	Control	T2D	Control vs T2D
383	404909.16027550577 0.113076224364610.014831806294413						2-Oxocarboxylic acid metabolism	Control	T2D	Control vs T2D
217	190235.9724734360 0.112253304358900.014855206294413						Bacterial secretion system [PATH:ko03070]	Control	T2D	Control vs T2D
362	181462.8427717666 0.111803394307430.015067906294413						Terpenoid backbone biosynthesis [PATH:ko00900]	Control	T2D	Control vs T2D

	baseMean	log2FoldChange	lfcSE	stat	pvalue	padj	gene	g1	g2	compare
378	14742.178	0.3479546	0.1434426	2.425759	0.0152764	0.0629443	Naphthalene degradation [PATH:ko00626]	Control	T2D	Control vs T2D
386	3152810.0276	0.2675682	0.1105292	2.420790	0.0154866	0.0629443	Biosynthesis of secondary metabolites	Control	T2D	Control vs T2D
292	325138.2592	0.2759617	0.1141163	2.418249	0.0155954	0.0629443	Pentose phosphate pathway [PATH:ko00030]	Control	T2D	Control vs T2D
268	31621.9622	0.2877683	0.1194728	2.408610	0.0160134	0.0629443	Carbapenem biosynthesis [PATH:ko00332]	Control	T2D	Control vs T2D
270	20233.1183	0.2801932	0.1166652	2.401678	0.0163201	0.0629443	Glucosinolate biosynthesis [PATH:ko00966]	Control	T2D	Control vs T2D
335	188159.1347	0.2800632	0.1166712	2.400435	0.0163756	0.0629443	One carbon pool by folate [PATH:ko00670]	Control	T2D	Control vs T2D
253	233417.0192	0.2700907	0.1126115	2.398429	0.0164656	0.0629443	Arginine biosynthesis [PATH:ko00220]	Control	T2D	Control vs T2D
240	326813.5592	0.2775516	0.1160979	2.390668	0.0168177	0.0629443	Homologous recombination [PATH:ko03440]	Control	T2D	Control vs T2D
257	252348.0759	0.2697013	0.1128258	2.390422	0.0168290	0.0629443	Lysine biosynthesis [PATH:ko00300]	Control	T2D	Control vs T2D
204	157791.6914	0.4944263	0.2080188	2.376830	0.0174609	0.0629443	Bacterial chemotaxis [PATH:ko02030]	Control	T2D	Control vs T2D
254	402736.7406	0.2617229	0.1102502	2.373900	0.0176003	0.0629443	Cysteine and methionine metabolism [PATH:ko00270]	Control	T2D	Control vs T2D
385	2443269.7203	0.2636503	0.1113826	2.367069	0.0179296	0.0629443	Biosynthesis of antibiotics	Control	T2D	Control vs T2D
205	188508.8954	0.5196077	0.2202727	2.358929	0.0183278	0.0630017	Flagellar assembly [PATH:ko02040]	Control	T2D	Control vs T2D
944	995.3726	1.6957014	0.5321833	3.186311	0.0014410	0.0632892	Geraniol degradation [PATH:ko00281]	LADA	T1D	LADA vs T1D
838	40338.5823	-0.8390318	0.2671543	-3.140627	0.0016859	0.0632892	RNA transport [PATH:ko03013]	LADA	T1D	LADA vs T1D
833	69732.4972	-0.6340390	0.2045511	-3.099661	0.0019374	0.0632892	RNA polymerase [PATH:ko03020]	LADA	T1D	LADA vs T1D
541	36295.1835	-0.3255017	0.1510288	-2.155230	0.0311438	0.0642547	D-Alanine metabolism [PATH:ko00473]	LADA	Control	LADA vs Control

	baseMean	log2FoldChange	lfcSE	stat	pvalue	padj	gene	g1	g2	compare
467	17841.4191	-0.3384042	0.1573568	-2.150554	0.0315104	0.0643358	Isoquinoline alkaloid biosynthesis [PATH:ko00950]	LADA	Control	LADA vs Control
267	32062.939	0.3590665	0.1539650	2.332130	0.0196908	0.0661577	Biosynthesis of various secondary metabolites - part 1 [PATH:ko00999]	Control	T2D	Control vs T2D
281	543686.333	0.2639215	0.1138926	2.317284	0.0204882	0.0661577	Amino sugar and nucleotide sugar metabolism [PATH:ko00520]	Control	T2D	Control vs T2D
284	126907.260	0.2653928	0.1145303	2.317228	0.0204910	0.0661577	C5-Branched dibasic acid metabolism [PATH:ko00660]	Control	T2D	Control vs T2D
391	6821207.125	0.2567892	0.1111307	2.310695	0.0208497	0.0661577	Metabolic pathways	Control	T2D	Control vs T2D
390	192044.392	0.2690991	0.1169557	2.300864	0.0213993	0.0666206	Fatty acid metabolism	Control	T2D	Control vs T2D
294	379607.917	0.2604543	0.1139531	2.285627	0.0222761	0.0676342	Pyruvate metabolism [PATH:ko00620]	Control	T2D	Control vs T2D
320	177269.315	0.2691740	0.1181454	2.278328	0.0227070	0.0676342	Fatty acid biosynthesis [PATH:ko00061]	Control	T2D	Control vs T2D
199	156155.022	0.2558325	0.1130895	2.262212	0.0236843	0.0676342	Cell cycle - Caulobacter [PATH:ko04112]	Control	T2D	Control vs T2D
299	133882.283	0.2507092	0.1113854	2.250827	0.0243965	0.0676342	Nitrogen metabolism [PATH:ko00910]	Control	T2D	Control vs T2D
278	47804.827	0.2868530	0.1278215	2.244169	0.0248215	0.0676342	Prodigiosin biosynthesis [PATH:ko00333]	Control	T2D	Control vs T2D
256	176722.941	0.2549246	0.1143334	2.229660	0.0257700	0.0676342	Histidine metabolism [PATH:ko00340]	Control	T2D	Control vs T2D
368	41420.409	0.2904633	0.1302931	2.229308	0.0257934	0.0676342	Benzoate degradation [PATH:ko00362]	Control	T2D	Control vs T2D
287	375800.789	0.2668424	0.1199178	2.225211	0.0260671	0.0676342	Galactose metabolism [PATH:ko00052]	Control	T2D	Control vs T2D
222	40343.094	0.2622117	0.1184375	2.213920	0.0268340	0.0676342	HIF-1 signaling pathway [PATH:ko04066]	Control	T2D	Control vs T2D
364	809598.046	0.2486653	0.1123369	2.213568	0.0268585	0.0676342	Purine metabolism [PATH:ko00230]	Control	T2D	Control vs T2D
246	1242724.073	0.2521506	0.1140810	2.210276	0.0270860	0.0676342	Aminoacyl-tRNA biosynthesis [PATH:ko00970]	Control	T2D	Control vs T2D

	baseMean	log2FoldChange	lfcSE	stat	pvalue	padj	gene	g1	g2	compare
374	122202.8072494803		0.1132165	2.203560	0.0275504	0.0676342	Drug metabolism - other enzymes [PATH:ko00983]	Control	T2D	Control vs T2D
365	644062.4892489122		0.1130319	2.202140	0.0276553	0.0676342	Pyrimidine metabolism [PATH:ko00240]	Control	T2D	Control vs T2D
389	39376.4932743875		0.1246324	2.201539	0.0276979	0.0676342	Degradation of aromatic compounds	Control	T2D	Control vs T2D
283	201350.0982511005		0.1145464	2.192129	0.0283702	0.0676342	Butanoate metabolism [PATH:ko00650]	Control	T2D	Control vs T2D
293	229437.8082488987		0.1141772	2.179938	0.0292621	0.0676342	Propanoate metabolism [PATH:ko00640]	Control	T2D	Control vs T2D
209	110632.9152648642		0.1215794	2.178529	0.0293667	0.0676342	Biofilm formation - Vibrio cholerae [PATH:ko05111]	Control	T2D	Control vs T2D
334	170759.3562506394		0.1151812	2.176041	0.0295520	0.0676342	Nicotinate and nicotinamide metabolism [PATH:ko00760]	Control	T2D	Control vs T2D
392	1850407.02485962		0.1142914	2.175109	0.0296219	0.0676342	Microbial metabolism in diverse environments	Control	T2D	Control vs T2D
235	192358.4092488111		0.1146010	2.171108	0.0299230	0.0676342	RNA degradation [PATH:ko03018]	Control	T2D	Control vs T2D
243	146391.9672544012		0.1177627	2.160287	0.0307505	0.0681056	Nucleotide excision repair [PATH:ko03420]	Control	T2D	Control vs T2D
331	122860.0732728459		0.1266616	2.154132	0.0312298	0.0681056	Biotin metabolism [PATH:ko00780]	Control	T2D	Control vs T2D
252	130403.3482482688		0.1153478	2.152349	0.0313699	0.0681056	Arginine and proline metabolism [PATH:ko00330]	Control	T2D	Control vs T2D
458	51343.4178	-0.3147050	0.1485792	-2.118095	0.0341670	0.0690384	Tyrosine metabolism [PATH:ko00350]	LADA	Control	LADA vs Control
342	77161.2172464532		0.1150829	2.141529	0.0322314	0.0690674	Vitamin B6 metabolism [PATH:ko00750]	Control	T2D	Control vs T2D
566	23793.3043	-0.3434241	0.1631396	-2.105094	0.0352832	0.0705663	Chloroalkane and chloroalkene degradation [PATH:ko00625]	LADA	Control	LADA vs Control
210	609155.5532581158		0.1217082	2.120775	0.0339407	0.0717977	Quorum sensing [PATH:ko02024]	Control	T2D	Control vs T2D

	baseMean	log2FoldChange	lfcSE	stat	pvalue	padj	gene	g1	g2	compare
274	51850.3072	0.2409535	0.1140853	2.112052	0.0346802	0.0724371	Novobiocin biosynthesis [PATH:ko00401]	Control	T2D	Control vs T2D
219	18387.7027	0.3145539	0.1494326	2.104988	0.0352923	0.0725327	AMPK signaling pathway [PATH:ko04152]	Control	T2D	Control vs T2D
251	372509.6882	0.2386007	0.1136171	2.100044	0.0357249	0.0725327	Alanine, aspartate and glutamate metabolism [PATH:ko00250]	Control	T2D	Control vs T2D
255	329925.6733	0.2373496	0.1132176	2.096402	0.0360466	0.0725327	Glycine, serine and threonine metabolism [PATH:ko00260]	Control	T2D	Control vs T2D
535	68161.9513	-0.3522197	0.1689119	-2.085227	0.0370487	0.0733489	Riboflavin metabolism [PATH:ko00740]	LADA	Control	LADA vs Control
474	47804.8275	-0.3449603	0.1661656	-2.076003	0.0378907	0.0735070	Prodigiosin biosynthesis [PATH:ko00333]	LADA	Control	LADA vs Control
498	165767.9102	-0.3404788	0.1640994	-2.074833	0.0380002	0.0735070	Sulfur metabolism [PATH:ko00920]	LADA	Control	LADA vs Control
547	40296.1565	-0.3195153	0.1543928	-2.069496	0.0384996	0.0735070	Taurine and hypotaurine metabolism [PATH:ko00430]	LADA	Control	LADA vs Control
585	39376.4934	-0.3350818	0.1620224	-2.068121	0.0386287	0.0735070	Degradation of aromatic compounds	LADA	Control	LADA vs Control
577	237.5929	1.3837092	0.6719626	2.059209	0.0394743	0.0743938	Toluene degradation [PATH:ko00623]	LADA	Control	LADA vs Control
413	190235.9724	-0.2994332	0.1459272	-2.051936	0.0401759	0.0749949	Bacterial secretion system [PATH:ko03070]	LADA	Control	LADA vs Control
455	69330.4338	-0.3310732	0.1618366	-2.045726	0.0407804	0.0754107	Phenylalanine metabolism [PATH:ko00360]	LADA	Control	LADA vs Control
347	53072.2312	0.2382419	0.1150537	2.070700	0.0383866	0.0763108	D-Glutamine and D-glutamate metabolism [PATH:ko00471]	Control	T2D	Control vs T2D
300	328532.7684	0.2388005	0.1163227	2.052861	0.0400861	0.0767378	Oxidative phosphorylation [PATH:ko00190]	Control	T2D	Control vs T2D

	baseMean	log2FoldChange	lfcSE	stat	pvalue	padj	gene	g1	g2	compare
318	35807.7547	0.2451670	0.1195092	2.051436	0.0402205	0.0767378	Biosynthesis of unsaturated fatty acids [PATH:ko01040]	Control	T2D	Control vs T2D
238	248750.5462	0.2375583	0.1158824	2.049995	0.0403609	0.0767378	DNA replication [PATH:ko03030]	Control	T2D	Control vs T2D
227	33035.9265	0.2493327	0.1216848	2.049000	0.0404618	0.0767378	Phosphatidylinositol signaling system [PATH:ko04070]	Control	T2D	Control vs T2D
262	51343.4178	0.2336064	0.1142932	2.043922	0.0409613	0.0768024	Tyrosine metabolism [PATH:ko00350]	Control	T2D	Control vs T2D
382	13015.2065	0.4053301	0.1990327	2.036500	0.0417002	0.0773094	Xylene degradation [PATH:ko00622]	Control	T2D	Control vs T2D
415	18387.7027	-0.3934727	0.1942596	-2.025500	0.0428161	0.0779543	AMPK signaling pathway [PATH:ko04152]	LADA	Control	LADA vs Control
514	35807.7547	-0.3144737	0.1553606	-2.024153	0.0429504	0.0779543	Biosynthesis of unsaturated fatty acids [PATH:ko01040]	LADA	Control	LADA vs Control
289	251341.3033	0.2323220	0.1149945	2.020288	0.0433505	0.0794814	Glyoxylate and dicarboxylate metabolism [PATH:ko00630]	Control	T2D	Control vs T2D
570	122202.8726	-0.2951924	0.1471796	-2.005661	0.0448924	0.0807239	Drug metabolism - other enzymes [PATH:ko00983]	LADA	Control	LADA vs Control
517	62032.3100	-0.3230009	0.1618218	-1.996028	0.0459308	0.0816046	Fatty acid degradation [PATH:ko00071]	LADA	Control	LADA vs Control
494	363521.1595	-0.5206225	0.2611697	-1.993426	0.0462149	0.0816046	Methane metabolism [PATH:ko00680]	LADA	Control	LADA vs Control
564	41420.4099	-0.3365861	0.1693785	-1.987183	0.0469021	0.0820786	Benzoate degradation [PATH:ko00362]	LADA	Control	LADA vs Control
904	726.8127	1.6271311	0.5469625	2.974812	0.0029307	0.0820872	alpha-Linolenic acid metabolism [PATH:ko00592]	LADA	T1D	LADA vs T1D
330	13542.6964	0.3006716	0.1510856	1.990075	0.0465827	0.0828378	Synthesis and degradation of ketone bodies [PATH:ko00072]	Control	T2D	Control vs T2D
258	39888.8702	0.2789747	0.1403554	1.987630	0.0468526	0.0828378	Lysine degradation [PATH:ko00310]	Control	T2D	Control vs T2D

	baseMean	log2FoldChange	lfcSE	stat	pvalue	padj	gene	g1	g2	compare
363	17603.1775	0.2452567	0.1237191	1.982362	0.0474387	0.0828378	Zeatin biosynthesis [PATH:ko00908]	Control	T2D	Control vs T2D
388	1023857.9733	0.2303367	0.1162085	1.982098	0.0474683	0.0828378	Carbon metabolism	Control	T2D	Control vs T2D
380	9097.0503	0.3989945	0.2015042	1.980080	0.0476945	0.0828378	Styrene degradation [PATH:ko00643]	Control	T2D	Control vs T2D
371	2818.5689	0.4684353	0.2371681	1.975110	0.0482554	0.0829389	Chlorocyclohexane and chlorobenzene degradation [PATH:ko00361]	Control	T2D	Control vs T2D
527	122860.0732	-0.3254984	0.1646582	-1.976813	0.0480627	0.0831633	Biotin metabolism [PATH:ko00780]	LADA	Control	LADA vs Control
426	565135.9901	-0.3275260	0.1659116	-1.974099	0.0483705	0.0831633	Two-component system [PATH:ko02020]	LADA	Control	LADA vs Control
576	9097.0503	-0.5149420	0.2619530	-1.965780	0.0493240	0.0840653	Styrene degradation [PATH:ko00643]	LADA	Control	LADA vs Control
410	63429.4212	-0.3382360	0.1732376	-1.952440	0.0508860	0.0859799	Peroxisome [PATH:ko04146]	LADA	Control	LADA vs Control
405	110632.9151	-0.3073605	0.1580512	-1.944690	0.0518124	0.0867968	Biofilm formation - Vibrio cholerae [PATH:ko05111]	LADA	Control	LADA vs Control
506	175.3137	1.0936551	0.5702890	1.917720	0.0551464	0.0910330	Mannose type O-glycan biosynthesis [PATH:ko00515]	LADA	Control	LADA vs Control
481	205734.1088	-0.3128637	0.1632264	-1.916747	0.0552700	0.0910330	Citrate cycle (TCA cycle) [PATH:ko00020]	LADA	Control	LADA vs Control
261	21549.3035	0.2628463	0.1361656	1.930340	0.0535643	0.0911146	Tryptophan metabolism [PATH:ko00380]	Control	T2D	Control vs T2D
337	223648.1836	0.2633404	0.1378881	1.909810	0.0561576	0.0945510	Porphyrin and chlorophyll metabolism [PATH:ko00860]	Control	T2D	Control vs T2D
248	964710.8102	0.2207059	0.1168591	1.888650	0.0589387	0.0981236	Ribosome [PATH:ko03010]	Control	T2D	Control vs T2D
280	35023.1733	0.2160469	0.1146313	1.884710	0.0594688	0.0981236	Tropane, piperidine and pyridine alkaloid biosynthesis [PATH:ko00960]	Control	T2D	Control vs T2D

	baseMean	log2FoldChange	lfcSE	stat	pvalue	padj	gene	g1	g2	compare
372	12380.6572	0.3814862	0.2040416	1.869640	0.0615308	0.0996512	Dioxin degradation [PATH:ko00621]	Control	T2D	Control vs T2D
279	139233.4452	0.2252978	0.1205352	1.869146	0.0616025	0.0996512	Streptomycin biosynthesis [PATH:ko00521]	Control	T2D	Control vs T2D

```
kable(stat_sig[,c(5:7,10)])
```

	pvalue	padj	gene	compare
895	0.0000354	0.0069458	Glycosphingolipid biosynthesis - lacto and neolacto series [PATH:ko00601]	LADA vs T1D
552	0.0001449	0.0162870	Geraniol degradation [PATH:ko00281]	LADA vs Control
512	0.0001662	0.0162870	alpha-Linolenic acid metabolism [PATH:ko00592]	LADA vs Control
571	0.0007520	0.0333090	Ethylbenzene degradation [PATH:ko00642]	LADA vs Control
466	0.0010929	0.0333090	Glucosinolate biosynthesis [PATH:ko00966]	LADA vs Control
459	0.0014467	0.0333090	Valine, leucine and isoleucine biosynthesis [PATH:ko00290]	LADA vs Control
579	0.0015375	0.0333090	2-Oxocarboxylic acid metabolism	LADA vs Control
472	0.0015836	0.0333090	Phenazine biosynthesis [PATH:ko00405]	LADA vs Control
515	0.0016076	0.0333090	Ether lipid metabolism [PATH:ko00565]	LADA vs Control
536	0.0016305	0.0333090	Thiamine metabolism [PATH:ko00730]	LADA vs Control
456	0.0018916	0.0333090	Phenylalanine, tyrosine and tryptophan biosynthesis [PATH:ko00400]	LADA vs Control
532	0.0020387	0.0333090	Pantothenate and CoA biosynthesis [PATH:ko00770]	LADA vs Control
580	0.0020393	0.0333090	Biosynthesis of amino acids	LADA vs Control
480	0.0023361	0.0346055	C5-Branched dibasic acid metabolism [PATH:ko00660]	LADA vs Control
497	0.0026162	0.0346055	Photosynthesis [PATH:ko00195]	LADA vs Control
449	0.0030970	0.0346055	Arginine biosynthesis [PATH:ko00220]	LADA vs Control
518	0.0034891	0.0346055	Glycerolipid metabolism [PATH:ko00561]	LADA vs Control
488	0.0036136	0.0346055	Pentose phosphate pathway [PATH:ko00030]	LADA vs Control
442	0.0036957	0.0346055	Aminoacyl-tRNA biosynthesis [PATH:ko00970]	LADA vs Control
453	0.0037616	0.0346055	Lysine biosynthesis [PATH:ko00300]	LADA vs Control

	pvalue	padj	gene	compare
581	0.0042931	0.0346055	Biosynthesis of antibiotics	LADA vs Control
475	0.0043675	0.0346055	Streptomycin biosynthesis [PATH:ko00521]	LADA vs Control
558	0.0045024	0.0346055	Terpenoid backbone biosynthesis [PATH:ko00900]	LADA vs Control
439	0.0047446	0.0346055	Nucleotide excision repair [PATH:ko03420]	LADA vs Control
546	0.0047919	0.0346055	Selenocompound metabolism [PATH:ko00450]	LADA vs Control
492	0.0048108	0.0346055	Carbon fixation in photosynthetic organisms [PATH:ko00710]	LADA vs Control
450	0.0049114	0.0346055	Cysteine and methionine metabolism [PATH:ko00270]	LADA vs Control
464	0.0051318	0.0346055	Carbapenem biosynthesis [PATH:ko00332]	LADA vs Control
582	0.0052285	0.0346055	Biosynthesis of secondary metabolites	LADA vs Control
434	0.0053967	0.0346055	DNA replication [PATH:ko03030]	LADA vs Control
437	0.0054656	0.0346055	Mismatch repair [PATH:ko03430]	LADA vs Control
556	0.0057776	0.0346055	Polyketide sugar unit biosynthesis [PATH:ko00523]	LADA vs Control
588	0.0058607	0.0346055	Microbial metabolism in diverse environments	LADA vs Control
399	0.0059859	0.0346055	Necroptosis [PATH:ko04217]	LADA vs Control
468	0.0063136	0.0346055	Monobactam biosynthesis [PATH:ko00261]	LADA vs Control
510	0.0067198	0.0346055	Peptidoglycan biosynthesis [PATH:ko00550]	LADA vs Control
452	0.0067429	0.0346055	Histidine metabolism [PATH:ko00340]	LADA vs Control
560	0.0071435	0.0346055	Purine metabolism [PATH:ko00230]	LADA vs Control
451	0.0072330	0.0346055	Glycine, serine and threonine metabolism [PATH:ko00260]	LADA vs Control
429	0.0073488	0.0346055	Protein export [PATH:ko03060]	LADA vs Control
447	0.0074117	0.0346055	Alanine, aspartate and glutamate metabolism [PATH:ko00250]	LADA vs Control
587	0.0078966	0.0346055	Metabolic pathways	LADA vs Control
561	0.0081670	0.0346055	Pyrimidine metabolism [PATH:ko00240]	LADA vs Control
503	0.0082172	0.0346055	Glycosphingolipid biosynthesis - lacto and neolacto series [PATH:ko00601]	LADA vs Control
470	0.0084018	0.0346055	Novobiocin biosynthesis [PATH:ko00401]	LADA vs Control
444	0.0084422	0.0346055	Ribosome [PATH:ko03010]	LADA vs Control

	pvalue	padj	gene	compare
543	0.0085715	0.0346055	D-Glutamine and D-glutamate metabolism [PATH:ko00471]	LADA vs Control
484	0.0085968	0.0346055	Glycolysis / Gluconeogenesis [PATH:ko00010]	LADA vs Control
406	0.0086998	0.0346055	Quorum sensing [PATH:ko02024]	LADA vs Control
548	0.0092733	0.0346055	Biosynthesis of ansamycins [PATH:ko01051]	LADA vs Control
584	0.0093098	0.0346055	Carbon metabolism	LADA vs Control
538	0.0093267	0.0346055	Vitamin B6 metabolism [PATH:ko00750]	LADA vs Control
395	0.0093611	0.0346055	Cell cycle - Caulobacter [PATH:ko04112]	LADA vs Control
441	0.0094888	0.0346055	RNA polymerase [PATH:ko03020]	LADA vs Control
431	0.0095968	0.0346055	RNA degradation [PATH:ko03018]	LADA vs Control
530	0.0098505	0.0346055	Nicotinate and nicotinamide metabolism [PATH:ko00760]	LADA vs Control
436	0.0098873	0.0346055	Homologous recombination [PATH:ko03440]	LADA vs Control
854	0.0004749	0.0358391	Betalain biosynthesis [PATH:ko00965]	LADA vs T1D
837	0.0005486	0.0358391	Ribosome biogenesis in eukaryotes [PATH:ko03008]	LADA vs T1D
432	0.0107440	0.0369444	Sulfur relay system [PATH:ko04122]	LADA vs Control
486	0.0109597	0.0369488	Inositol phosphate metabolism [PATH:ko00562]	LADA vs Control
483	0.0111223	0.0369488	Galactose metabolism [PATH:ko00052]	LADA vs Control
489	0.0116283	0.0379856	Propanoate metabolism [PATH:ko00640]	LADA vs Control
478	0.0121652	0.0386411	Ascorbate and aldarate metabolism [PATH:ko00053]	LADA vs Control
531	0.0122232	0.0386411	One carbon pool by folate [PATH:ko00670]	LADA vs Control
519	0.0125377	0.0390061	Glycerophospholipid metabolism [PATH:ko00564]	LADA vs Control
8	0.0002559	0.0390382	Bacterial chemotaxis [PATH:ko02030]	Control vs T1D
9	0.0003983	0.0390382	Flagellar assembly [PATH:ko02040]	Control vs T1D
461	0.0127739	0.0391200	Acarbose and validamycin biosynthesis [PATH:ko00525]	LADA vs Control
495	0.0136810	0.0408927	Nitrogen metabolism [PATH:ko00910]	LADA vs Control
482	0.0137700	0.0408927	Fructose and mannose metabolism [PATH:ko00051]	LADA vs Control
418	0.0147613	0.0431823	HIF-1 signaling pathway [PATH:ko04066]	LADA vs Control

	pvalue	padj	gene	compare
462	0.0151820	0.0437599	Betalain biosynthesis [PATH:ko00965]	LADA vs Control
423	0.0157014	0.0442859	Phosphatidylinositol signaling system [PATH:ko04070]	LADA vs Control
412	0.0158164	0.0442859	ABC transporters [PATH:ko02010]	LADA vs Control
433	0.0161830	0.0446743	Base excision repair [PATH:ko03410]	LADA vs Control
573	0.0167594	0.0450038	Metabolism of xenobiotics by cytochrome P450 [PATH:ko00980]	LADA vs Control
569	0.0167616	0.0450038	Drug metabolism - cytochrome P450 [PATH:ko00982]	LADA vs Control
491	0.0188318	0.0493905	Starch and sucrose metabolism [PATH:ko00500]	LADA vs Control
408	0.0188994	0.0493905	Autophagy - yeast [PATH:ko04138]	LADA vs Control
477	0.0191645	0.0494243	Amino sugar and nucleotide sugar metabolism [PATH:ko00520]	LADA vs Control
490	0.0204551	0.0517856	Pyruvate metabolism [PATH:ko00620]	LADA vs Control
476	0.0206085	0.0517856	Tropane, piperidine and pyridine alkaloid biosynthesis [PATH:ko00960]	LADA vs Control
550	0.0213722	0.0530248	Biosynthesis of vancomycin group antibiotics [PATH:ko01055]	LADA vs Control
533	0.0226272	0.0549700	Porphyrin and chlorophyll metabolism [PATH:ko00860]	LADA vs Control
516	0.0227172	0.0549700	Fatty acid biosynthesis [PATH:ko00061]	LADA vs Control
446	0.0230254	0.0550363	RNA transport [PATH:ko03013]	LADA vs Control
586	0.0240073	0.0566919	Fatty acid metabolism	LADA vs Control
521	0.0245535	0.0569158	Primary bile acid biosynthesis [PATH:ko00120]	LADA vs Control
414	0.0246829	0.0569158	Phosphotransferase system (PTS) [PATH:ko02060]	LADA vs Control
445	0.0252415	0.0574628	Ribosome biogenesis in eukaryotes [PATH:ko03008]	LADA vs Control
539	0.0259727	0.0574628	beta-Alanine metabolism [PATH:ko00410]	LADA vs Control
479	0.0262357	0.0574628	Butanoate metabolism [PATH:ko00650]	LADA vs Control
485	0.0263113	0.0574628	Glyoxylate and dicarboxylate metabolism [PATH:ko00630]	LADA vs Control
522	0.0266088	0.0574628	Secondary bile acid biosynthesis [PATH:ko00121]	LADA vs Control
528	0.0269822	0.0574628	Folate biosynthesis [PATH:ko00790]	LADA vs Control
559	0.0272008	0.0574628	Zeatin biosynthesis [PATH:ko00908]	LADA vs Control
493	0.0275005	0.0574628	Carbon fixation pathways in prokaryotes [PATH:ko00720]	LADA vs Control

	pvalue	padj	gene	compare
496	0.0275587	0.0574628	Oxidative phosphorylation [PATH:ko00190]	LADA vs Control
236	0.0039485	0.0629443	Sulfur relay system [PATH:ko04122]	Control vs T2D
322	0.0040520	0.0629443	Glycerolipid metabolism [PATH:ko00561]	Control vs T2D
366	0.0041526	0.0629443	Aminobenzoate degradation [PATH:ko00627]	Control vs T2D
230	0.0045253	0.0629443	Two-component system [PATH:ko02020]	Control vs T2D
207	0.0046159	0.0629443	Biofilm formation - Escherichia coli [PATH:ko02026]	Control vs T2D
314	0.0049509	0.0629443	Peptidoglycan biosynthesis [PATH:ko00550]	Control vs T2D
323	0.0052216	0.0629443	Glycerophospholipid metabolism [PATH:ko00564]	Control vs T2D
301	0.0056442	0.0629443	Photosynthesis [PATH:ko00195]	Control vs T2D
218	0.0056557	0.0629443	Phosphotransferase system (PTS) [PATH:ko02060]	Control vs T2D
352	0.0058911	0.0629443	Biosynthesis of ansamycins [PATH:ko01051]	Control vs T2D
302	0.0071873	0.0629443	Sulfur metabolism [PATH:ko00920]	Control vs T2D
370	0.0081464	0.0629443	Chloroalkane and chloroalkene degradation [PATH:ko00625]	Control vs T2D
336	0.0086860	0.0629443	Pantothenate and CoA biosynthesis [PATH:ko00770]	Control vs T2D
288	0.0093897	0.0629443	Glycolysis / Gluconeogenesis [PATH:ko00010]	Control vs T2D
345	0.0096014	0.0629443	D-Alanine metabolism [PATH:ko00473]	Control vs T2D
340	0.0096686	0.0629443	Thiamine metabolism [PATH:ko00730]	Control vs T2D
203	0.0098607	0.0629443	Necroptosis [PATH:ko04217]	Control vs T2D
296	0.0102169	0.0629443	Carbon fixation in photosynthetic organisms [PATH:ko00710]	Control vs T2D
276	0.0104857	0.0629443	Phenazine biosynthesis [PATH:ko00405]	Control vs T2D
241	0.0108697	0.0629443	Mismatch repair [PATH:ko03430]	Control vs T2D
216	0.0110469	0.0629443	ABC transporters [PATH:ko02010]	Control vs T2D
263	0.0115943	0.0629443	Valine, leucine and isoleucine biosynthesis [PATH:ko00290]	Control vs T2D
286	0.0118980	0.0629443	Fructose and mannose metabolism [PATH:ko00051]	Control vs T2D
295	0.0120571	0.0629443	Starch and sucrose metabolism [PATH:ko00500]	Control vs T2D
343	0.0123800	0.0629443	beta-Alanine metabolism [PATH:ko00410]	Control vs T2D

	pvalue	padj	gene	compare
272	0.0124246	0.0629443	Monobactam biosynthesis [PATH:ko00261]	Control vs T2D
233	0.0125229	0.0629443	Protein export [PATH:ko03060]	Control vs T2D
260	0.0126419	0.0629443	Phenylalanine, tyrosine and tryptophan biosynthesis [PATH:ko00400]	Control vs T2D
384	0.0127064	0.0629443	Biosynthesis of amino acids	Control vs T2D
208	0.0129311	0.0629443	Biofilm formation - Pseudomonas aeruginosa [PATH:ko02025]	Control vs T2D
350	0.0132910	0.0629443	Selenocompound metabolism [PATH:ko00450]	Control vs T2D
237	0.0146192	0.0629443	Base excision repair [PATH:ko03410]	Control vs T2D
383	0.0148318	0.0629443	2-Oxocarboxylic acid metabolism	Control vs T2D
217	0.0148552	0.0629443	Bacterial secretion system [PATH:ko03070]	Control vs T2D
362	0.0150679	0.0629443	Terpenoid backbone biosynthesis [PATH:ko00900]	Control vs T2D
378	0.0152764	0.0629443	Naphthalene degradation [PATH:ko00626]	Control vs T2D
386	0.0154866	0.0629443	Biosynthesis of secondary metabolites	Control vs T2D
292	0.0155954	0.0629443	Pentose phosphate pathway [PATH:ko00030]	Control vs T2D
268	0.0160134	0.0629443	Carbapenem biosynthesis [PATH:ko00332]	Control vs T2D
270	0.0163201	0.0629443	Glucosinolate biosynthesis [PATH:ko00966]	Control vs T2D
335	0.0163756	0.0629443	One carbon pool by folate [PATH:ko00670]	Control vs T2D
253	0.0164656	0.0629443	Arginine biosynthesis [PATH:ko00220]	Control vs T2D
240	0.0168177	0.0629443	Homologous recombination [PATH:ko03440]	Control vs T2D
257	0.0168290	0.0629443	Lysine biosynthesis [PATH:ko00300]	Control vs T2D
204	0.0174619	0.0629443	Bacterial chemotaxis [PATH:ko02030]	Control vs T2D
254	0.0176013	0.0629443	Cysteine and methionine metabolism [PATH:ko00270]	Control vs T2D
385	0.0179296	0.0629443	Biosynthesis of antibiotics	Control vs T2D
205	0.0183278	0.0630017	Flagellar assembly [PATH:ko02040]	Control vs T2D
944	0.0014410	0.0632892	Geraniol degradation [PATH:ko00281]	LADA vs T1D
838	0.0016859	0.0632892	RNA transport [PATH:ko03013]	LADA vs T1D
833	0.0019374	0.0632892	RNA polymerase [PATH:ko03020]	LADA vs T1D

	pvalue	padj	gene	compare
541	0.0311438	0.0642547	D-Alanine metabolism [PATH:ko00473]	LADA vs Control
467	0.0315114	0.0643358	Isoquinoline alkaloid biosynthesis [PATH:ko00950]	LADA vs Control
267	0.0196938	0.0661577	Biosynthesis of various secondary metabolites - part 1 [PATH:ko00999]	Control vs T2D
281	0.0204882	0.0661577	Amino sugar and nucleotide sugar metabolism [PATH:ko00520]	Control vs T2D
284	0.0204913	0.0661577	C5-Branched dibasic acid metabolism [PATH:ko00660]	Control vs T2D
391	0.0208497	0.0661577	Metabolic pathways	Control vs T2D
390	0.0213993	0.0666206	Fatty acid metabolism	Control vs T2D
294	0.0222761	0.0676342	Pyruvate metabolism [PATH:ko00620]	Control vs T2D
320	0.0227070	0.0676342	Fatty acid biosynthesis [PATH:ko00061]	Control vs T2D
199	0.0236843	0.0676342	Cell cycle - Caulobacter [PATH:ko04112]	Control vs T2D
299	0.0243965	0.0676342	Nitrogen metabolism [PATH:ko00910]	Control vs T2D
278	0.0248215	0.0676342	Prodigiosin biosynthesis [PATH:ko00333]	Control vs T2D
256	0.0257700	0.0676342	Histidine metabolism [PATH:ko00340]	Control vs T2D
368	0.0257934	0.0676342	Benzoate degradation [PATH:ko00362]	Control vs T2D
287	0.0260671	0.0676342	Galactose metabolism [PATH:ko00052]	Control vs T2D
222	0.0268340	0.0676342	HIF-1 signaling pathway [PATH:ko04066]	Control vs T2D
364	0.0268585	0.0676342	Purine metabolism [PATH:ko00230]	Control vs T2D
246	0.0270860	0.0676342	Aminoacyl-tRNA biosynthesis [PATH:ko00970]	Control vs T2D
374	0.0275547	0.0676342	Drug metabolism - other enzymes [PATH:ko00983]	Control vs T2D
365	0.0276553	0.0676342	Pyrimidine metabolism [PATH:ko00240]	Control vs T2D
389	0.0276979	0.0676342	Degradation of aromatic compounds	Control vs T2D
283	0.0283702	0.0676342	Butanoate metabolism [PATH:ko00650]	Control vs T2D
293	0.0292621	0.0676342	Propanoate metabolism [PATH:ko00640]	Control vs T2D
209	0.0293667	0.0676342	Biofilm formation - Vibrio cholerae [PATH:ko05111]	Control vs T2D
334	0.0295520	0.0676342	Nicotinate and nicotinamide metabolism [PATH:ko00760]	Control vs T2D
392	0.0296219	0.0676342	Microbial metabolism in diverse environments	Control vs T2D

	pvalue	padj	gene	compare
235	0.0299230	0.0676342	RNA degradation [PATH:ko03018]	Control vs T2D
243	0.0307505	0.0681056	Nucleotide excision repair [PATH:ko03420]	Control vs T2D
331	0.0312298	0.0681056	Biotin metabolism [PATH:ko00780]	Control vs T2D
252	0.0313699	0.0681056	Arginine and proline metabolism [PATH:ko00330]	Control vs T2D
458	0.0341670	0.0690384	Tyrosine metabolism [PATH:ko00350]	LADA vs Control
342	0.0322314	0.0690674	Vitamin B6 metabolism [PATH:ko00750]	Control vs T2D
566	0.0352832	0.0705663	Chloroalkane and chloroalkene degradation [PATH:ko00625]	LADA vs Control
210	0.0339407	0.0717977	Quorum sensing [PATH:ko02024]	Control vs T2D
274	0.0346820	0.0724371	Novobiocin biosynthesis [PATH:ko00401]	Control vs T2D
219	0.0352923	0.0725327	AMPK signaling pathway [PATH:ko04152]	Control vs T2D
251	0.0357249	0.0725327	Alanine, aspartate and glutamate metabolism [PATH:ko00250]	Control vs T2D
255	0.0360466	0.0725327	Glycine, serine and threonine metabolism [PATH:ko00260]	Control vs T2D
535	0.0370487	0.0733489	Riboflavin metabolism [PATH:ko00740]	LADA vs Control
474	0.0378937	0.0735070	Prodigiosin biosynthesis [PATH:ko00333]	LADA vs Control
498	0.0380020	0.0735070	Sulfur metabolism [PATH:ko00920]	LADA vs Control
547	0.0384996	0.0735070	Taurine and hypotaurine metabolism [PATH:ko00430]	LADA vs Control
585	0.0386287	0.0735070	Degradation of aromatic compounds	LADA vs Control
577	0.0394743	0.0743938	Toluene degradation [PATH:ko00623]	LADA vs Control
413	0.0401759	0.0749949	Bacterial secretion system [PATH:ko03070]	LADA vs Control
455	0.0407834	0.0754107	Phenylalanine metabolism [PATH:ko00360]	LADA vs Control
347	0.0383866	0.0763108	D-Glutamine and D-glutamate metabolism [PATH:ko00471]	Control vs T2D
300	0.0400861	0.0767378	Oxidative phosphorylation [PATH:ko00190]	Control vs T2D
318	0.0402245	0.0767378	Biosynthesis of unsaturated fatty acids [PATH:ko01040]	Control vs T2D
238	0.0403649	0.0767378	DNA replication [PATH:ko03030]	Control vs T2D
227	0.0404618	0.0767378	Phosphatidylinositol signaling system [PATH:ko04070]	Control vs T2D
262	0.0409613	0.0768024	Tyrosine metabolism [PATH:ko00350]	Control vs T2D

	pvalue	padj	gene	compare
382	0.0417002	0.0773094	Xylene degradation [PATH:ko00622]	Control vs T2D
415	0.0428161	0.0779543	AMPK signaling pathway [PATH:ko04152]	LADA vs Control
514	0.0429544	0.0779543	Biosynthesis of unsaturated fatty acids [PATH:ko01040]	LADA vs Control
289	0.0433535	0.0794814	Glyoxylate and dicarboxylate metabolism [PATH:ko00630]	Control vs T2D
570	0.0448924	0.0807239	Drug metabolism - other enzymes [PATH:ko00983]	LADA vs Control
517	0.0459308	0.0816046	Fatty acid degradation [PATH:ko00071]	LADA vs Control
494	0.0462149	0.0816046	Methane metabolism [PATH:ko00680]	LADA vs Control
564	0.0469021	0.0820786	Benzoate degradation [PATH:ko00362]	LADA vs Control
904	0.0029317	0.0820872	alpha-Linolenic acid metabolism [PATH:ko00592]	LADA vs T1D
330	0.0465827	0.0828378	Synthesis and degradation of ketone bodies [PATH:ko00072]	Control vs T2D
258	0.0468526	0.0828378	Lysine degradation [PATH:ko00310]	Control vs T2D
363	0.0474387	0.0828378	Zeatin biosynthesis [PATH:ko00908]	Control vs T2D
388	0.0474683	0.0828378	Carbon metabolism	Control vs T2D
380	0.0476945	0.0828378	Styrene degradation [PATH:ko00643]	Control vs T2D
371	0.0482554	0.0829389	Chlorocyclohexane and chlorobenzene degradation [PATH:ko00361]	Control vs T2D
527	0.0480627	0.0831633	Biotin metabolism [PATH:ko00780]	LADA vs Control
426	0.0483705	0.0831633	Two-component system [PATH:ko02020]	LADA vs Control
576	0.0493240	0.0840653	Styrene degradation [PATH:ko00643]	LADA vs Control
410	0.0508860	0.0859799	Peroxisome [PATH:ko04146]	LADA vs Control
405	0.0518124	0.0867968	Biofilm formation - Vibrio cholerae [PATH:ko05111]	LADA vs Control
506	0.0551464	0.0910330	Mannose type O-glycan biosynthesis [PATH:ko00515]	LADA vs Control
481	0.0552700	0.0910330	Citrate cycle (TCA cycle) [PATH:ko00020]	LADA vs Control
261	0.0535643	0.0911146	Tryptophan metabolism [PATH:ko00380]	Control vs T2D
337	0.0561576	0.0945510	Porphyrin and chlorophyll metabolism [PATH:ko00860]	Control vs T2D
248	0.0589387	0.0981236	Ribosome [PATH:ko03010]	Control vs T2D
280	0.0594688	0.0981236	Tropane, piperidine and pyridine alkaloid biosynthesis [PATH:ko00960]	Control vs T2D

	pvalue	padj	gene	compare
372	0.0615338	0.0996512	Dioxin degradation [PATH:ko00621]	Control vs T2D
279	0.0616025	0.0996512	Streptomycin biosynthesis [PATH:ko00521]	Control vs T2D

```r
##Venn diagram comparing LADA to other groups
sig_LADA <- subset(stat_sig, g1=="LADA")
sig_LADA_T1D <- subset(sig_LADA, g2=="T1D") %>% dplyr::select(one_of("gene")) %>%
  pull(gene)
sig_LADA_T2D <- subset(sig_LADA, g2=="T2D") %>% dplyr::select(one_of("gene")) %>%
  pull(gene)
sig_LADA_Control <- subset(sig_LADA, g2=="Control") %>% dplyr::select(one_of("gene")) %>%
  pull(gene)

#Colors
myCol <- c( "#1F78B4", "#33A02C", "#666666")
#
temp <- venn.diagram(list(T1D = sig_LADA_T1D,
                          T2D = sig_LADA_T2D,
                          Control = sig_LADA_Control), filename = NULL,

                     # Circles
                     lwd = 2,
                     lty = 'blank',
                     fill = myCol,

                     # Numbers
                     cex = 3,
                     fontface = "bold",
                     fontfamily = "sans",

                     # Set names
                     cat.cex = 3,
                     cat.fontface = "bold",
                     cat.default.pos = "outer",
                     cat.pos = c(0, 0, 0),
                     cat.dist = c(0.085, 0.085, 0.024),
                     cat.fontfamily = "sans",
                     rotation = 1)

grid.arrange(gTree(children=temp))
```

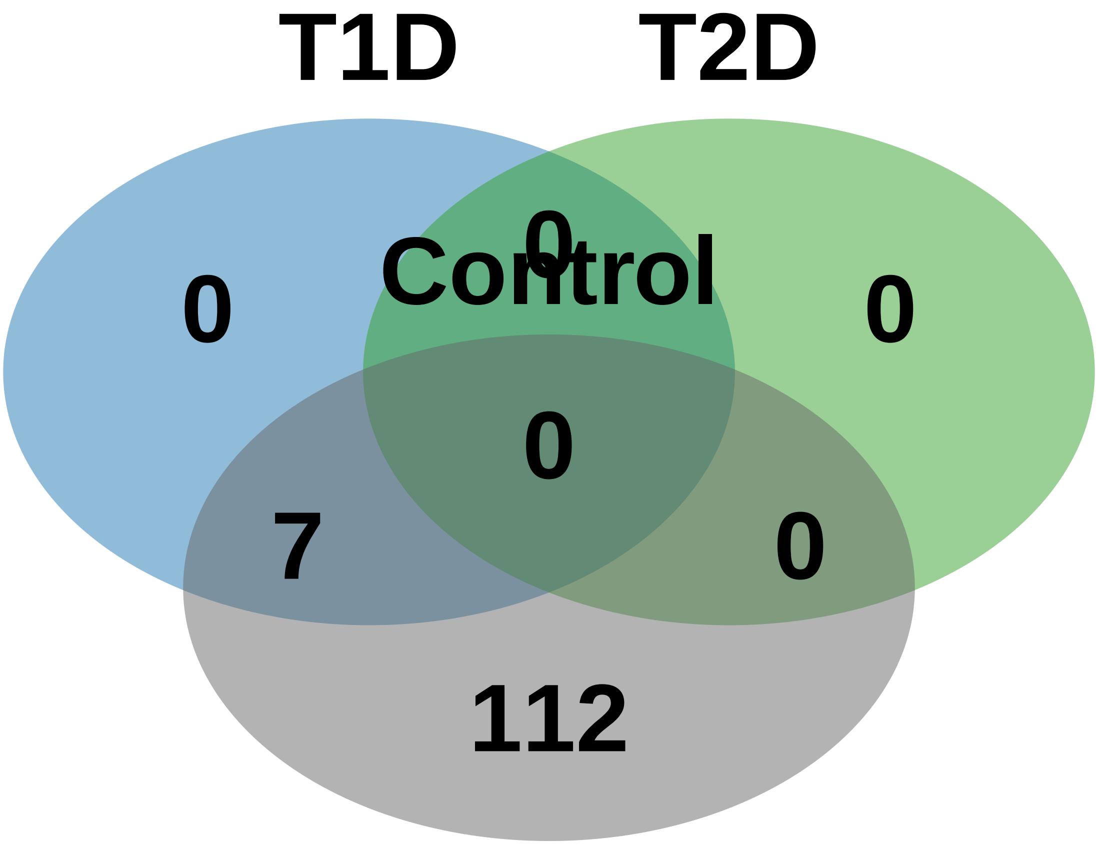


```
Fig2ListRemMet[[ "VennLADA" ]] <-
  gTree(children=temp, top="N differential abundant relative to LADA")

#Genera LADA that are significantly different from all other groups
intersect(intersect(sig_LADA_T1D,sig_LADA_T2D),sig_LADA_Control)
```

character(0)

```
##Venn diagram comparing T1D to other groups
sig_T1D <- subset(stat_sig, g1=="T1D" | g2=="T1D")
sig_T1D_LADA <- subset(sig_T1D, g1=="LADA") %>% dplyr::select(one_of("gene")) %>%
  pull(gene)
sig_T1D_T2D <- subset(sig_T1D, g2=="T2D") %>% dplyr::select(one_of("gene")) %>%
  pull(gene)
sig_T1D_Control <- subset(sig_T1D, g1=="Control") %>% dplyr::select(one_of("gene")) %>%
  pull(gene)
#Colors
myCol <- c( "#E31A1C", "#33A02C", "#666666")
#
temp <- venn.diagram(list(LADA = sig_T1D_LADA,
                          T2D = sig_T1D_T2D,
                          Control = sig_T1D_Control), filename = NULL,

                     # Circles
                     lwd = 2,
```

```
                        lty = 'blank',
                        fill = myCol,

                        # Numbers
                        cex = 2,
                        fontface = "bold",
                        fontfamily = "sans",

                        # Set names
                        cat.cex = 2,
                        cat.fontface = "bold",
                        cat.default.pos = "outer",
                        cat.pos = c(0, 0, 0),
                        cat.dist = c(0.055, 0.055, 0.024),
                        cat.fontfamily = "sans",
                        rotation = 1)

grid.arrange(gTree(children=temp))
```

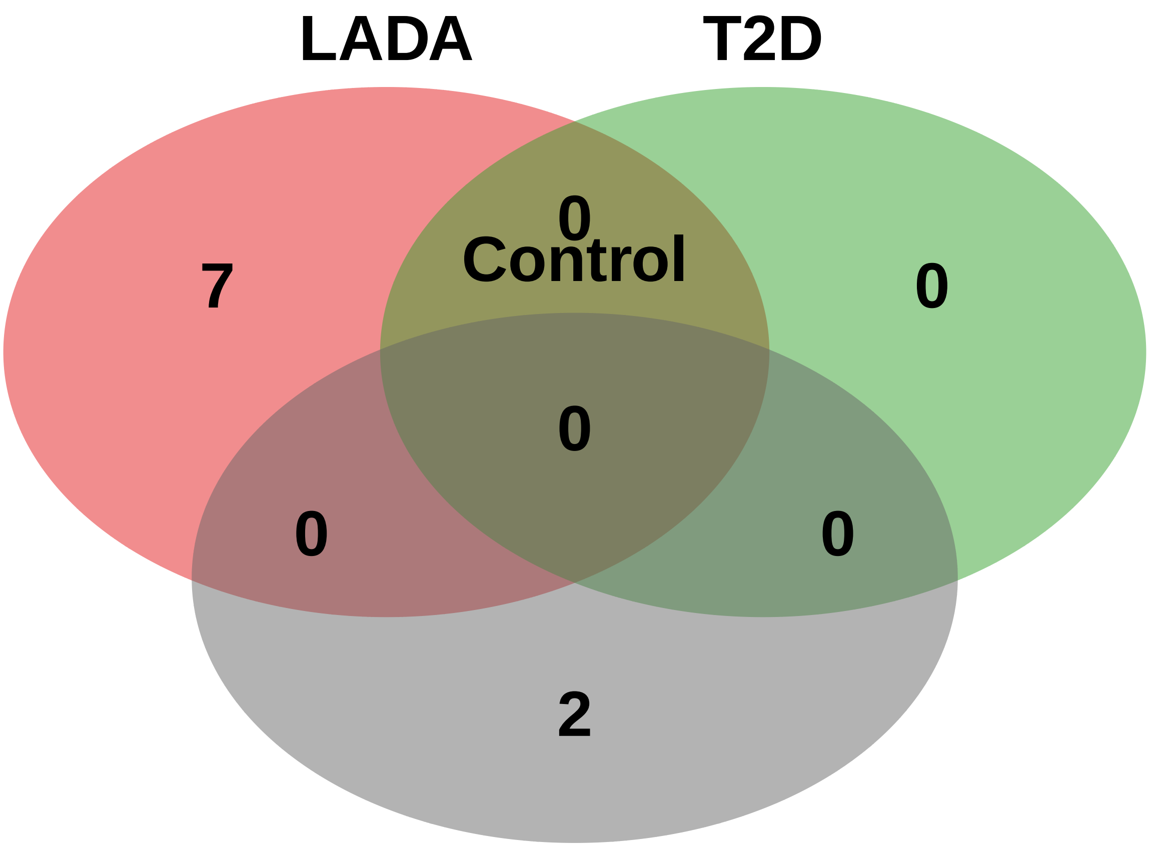


```
Fig2ListRemMet[[ "VennT1D" ]] <-
  gTree(children=temp, top="N differential abundant relative to T1D")

#Genera T1D that are significantly different from all other groups
intersect(intersect(sig_T1D_LADA,sig_T1D_T2D),sig_T1D_Control)
```

character(0)

```r
##Venn diagram comparing T2D to other groups
sig_T2D <- subset(stat_sig, g1=="T2D" | g2=="T2D")
sig_T2D_LADA <- subset(sig_T2D, g1=="LADA") %>% dplyr::select(one_of("gene")) %>%
  pull(gene)
sig_T2D_T1D <- subset(sig_T2D, g1=="T1D") %>% dplyr::select(one_of("gene")) %>%
  pull(gene)
sig_T2D_Control <- subset(sig_T2D, g1=="Control") %>% dplyr::select(one_of("gene")) %>%
  pull(gene)
#Colors
myCol <- c( "#E31A1C", "#1F78B4", "#666666")
#
temp <- venn.diagram(list(LADA = sig_T2D_LADA,
                          T1D = sig_T2D_T1D,
                          Control = sig_T2D_Control), filename = NULL,

                     # Circles
                     lwd = 2,
                     lty = 'blank',
                     fill = myCol,

                     # Numbers
                     cex = 2,
                     fontface = "bold",
                     fontfamily = "sans",

                     # Set names
                     cat.cex = 2,
                     cat.fontface = "bold",
                     cat.default.pos = "outer",
                     cat.pos = c(0, 0, 0),
                     cat.dist = c(0.055, 0.055, 0.024),
                     cat.fontfamily = "sans",
                     rotation = 1)

grid.arrange(gTree(children=temp))
```

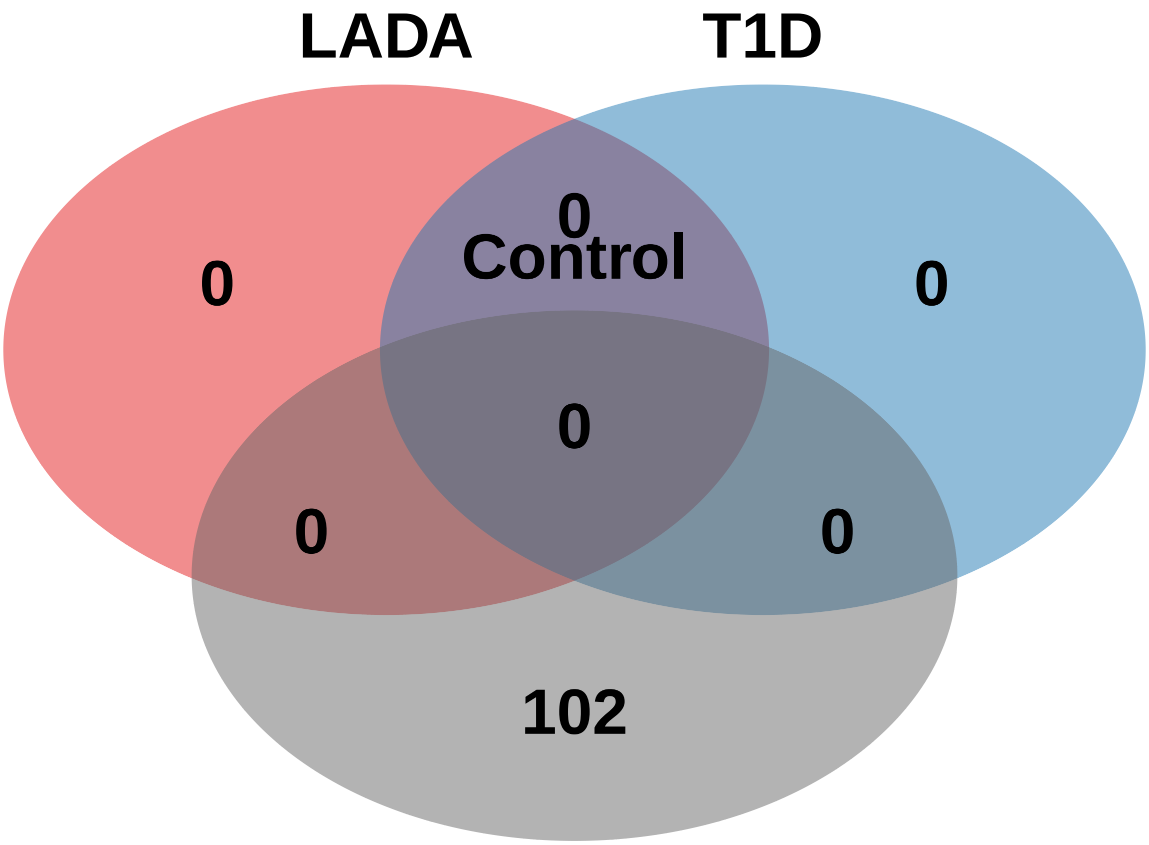


```
Fig2ListRemMet[[ "VennT2D" ]] <-
  gTree(children=temp, top="N differential abundant relative to T2D")

#Genera T2D that are significantly different from all other groups
intersect(intersect(sig_T2D_LADA,sig_T2D_T1D),sig_T2D_Control)
```

character(0)

```
##Venn diagram comparing controls to other groups
sig_Control <- subset(stat_sig, g1=="Control" | g2=="Control")
sig_Control_LADA <- subset(sig_Control, g1=="LADA") %>% dplyr::select(one_of("gene")) %>%
  pull(gene)
sig_Control_T1D <- subset(sig_Control, g2=="T1D") %>% dplyr::select(one_of("gene")) %>%
  pull(gene)
sig_Control_T2D <- subset(sig_Control, g2=="T2D") %>% dplyr::select(one_of("gene")) %>%
  pull(gene)
#Colors
myCol <- c( "#E31A1C", "#1F78B4", "#33A02C")
#
temp <- venn.diagram(list(LADA = sig_Control_LADA,
                          T1D = sig_Control_T1D,
                          T2D = sig_Control_T2D), filename = NULL,

                     # Circles
                     lwd = 2,
```

```
                     lty = 'blank',
                     fill = myCol,

                     # Numbers
                     cex = 3,
                     fontface = "bold",
                     fontfamily = "sans",

                     # Set names
                     cat.cex = 3,
                     cat.fontface = "bold",
                     cat.default.pos = "outer",
                     cat.pos = c(0, 0, 0),
                     cat.dist = c(0.055, 0.055, 0.024),
                     cat.fontfamily = "sans",
                     rotation = 1)

grid.arrange(gTree(children=temp))
```

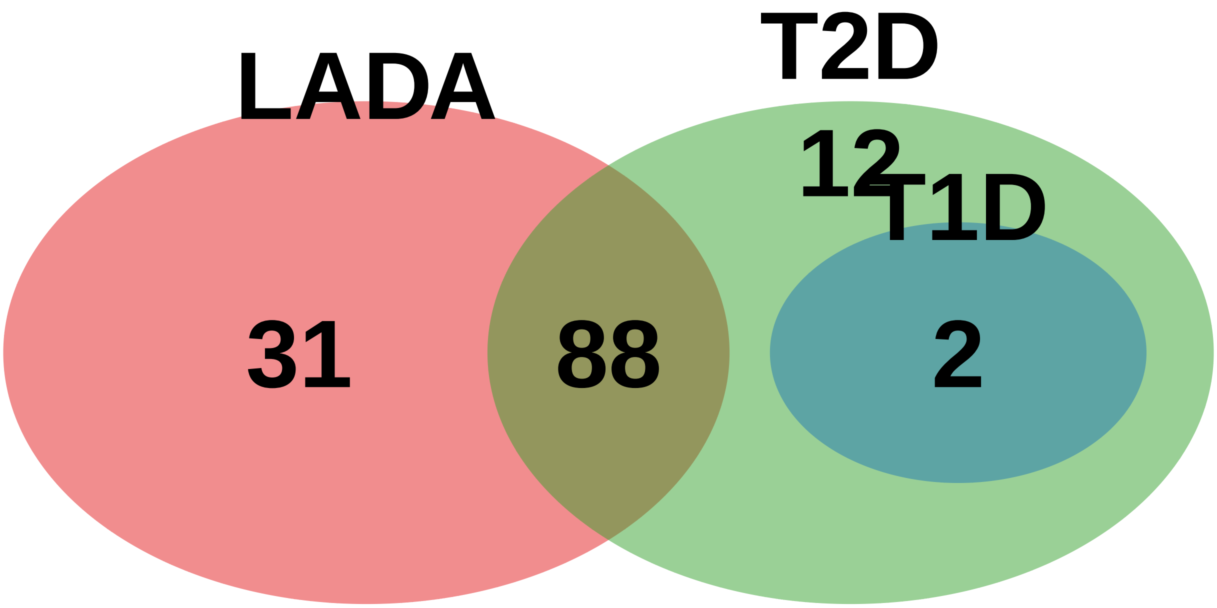


```
Fig2ListRemMet[[ "VennControls" ]] <-
  gTree(children=temp, top="N differential abundant relative to controls")

#Genera Controls that are significantly different from all other groups
intersect(intersect(sig_T2D_LADA,sig_T2D_T1D),sig_T2D_Control)
```

character(0)

```
##Create boxplots / violin plots
#Normalize to get cells pr. gram feces
if (setequal(colnames(Taxonomy2), SF3$MicrobiomeID)==FALSE) {
  stop("Metadata and Taxonomy out of sync")
}

#Total sum scaling (Use relative abundances)
Taxonomy3<-sweep(Taxonomy2, 2, colSums(Taxonomy), FUN="/")
#Obtain values as cells pr. gram feces
Taxonomy3<-sweep(Taxonomy3, 2, SF3$CellNorm, FUN="*")
#Make to micro gram
Taxonomy3<-Taxonomy3/10^6


##Select organisms
SelOrgs<-unique(stat_sig$gene)

tTaxSelect<-dplyr::select(as.data.frame(t(Taxonomy3)), one_of(c(SelOrgs)))
#Reducing the number because duplicates are removed
tTaxSelect<-add_rownames(tTaxSelect, "MicrobiomeID")
Plotting<-merge(SF3, tTaxSelect, by="MicrobiomeID")

##Might have issues with special characters, so this chunk might be needed
SelOrgs<-str_replace_all(SelOrgs, " ","_")
SelOrgs<-str_replace_all(SelOrgs, "-","_")
SelOrgs<-str_replace_all(SelOrgs, "/","_")
SelOrgs<-gsub("\\[|\\]", "", SelOrgs)
SelOrgs<-str_replace_all(SelOrgs, ":","_")
SelOrgs<-str_replace_all(SelOrgs, ",","_")
SelOrgs<-str_replace_all(SelOrgs, "2_","Two_")
SelOrgs<-str_replace_all(SelOrgs, "\\(","_")
SelOrgs<-str_replace_all(SelOrgs, "\\)","_")
colnames(Plotting) <- str_replace_all(colnames(Plotting)," ","_")
colnames(Plotting) <- str_replace_all(colnames(Plotting),"-","_")
colnames(Plotting) <- str_replace_all(colnames(Plotting),"/","_")
colnames(Plotting) <- gsub("\\[|\\]", "", colnames(Plotting))
colnames(Plotting) <- str_replace_all(colnames(Plotting),":","_")
colnames(Plotting) <- str_replace_all(colnames(Plotting),",","_")
colnames(Plotting) <- str_replace_all(colnames(Plotting),"2_","Two_")
colnames(Plotting) <- str_replace_all(colnames(Plotting),"\\(","_")
colnames(Plotting) <- str_replace_all(colnames(Plotting),"\\)","_")

#Plot logs on y axis
Plotting2 <- bind_cols(Plotting[,1:16], log10(Plotting[,17:ncol(Plotting)]+1))
#See end of chunk for boxplots removed the violinplots, because of redundancy


##Create vulcano plots
#Cut offs Benjamini-Hochberg method to add to vulcano plot
#Don't know how to get exact so it is a cut-off corresponding to the BH method
BHAll<-aggregate(stat_sig[, 5:6], list(stat_sig$compare), max)
row.names(BHAll)<-BHAll$Group.1
```

```
BHLadaCon <- BHAll[which(rownames(BHAll)=="LADA vs Control"),
                   which(colnames(BHAll)=="pvalue")]
BHLadaT1D <- BHAll[which(rownames(BHAll)=="LADA vs T1D"),
                   which(colnames(BHAll)=="pvalue")]
BHLadaT2D <- BHAll[which(rownames(BHAll)=="LADA vs T2D"),
                   which(colnames(BHAll)=="pvalue")]


#Vulcano plot
res_stat$minuslog10<--log(res_stat$pvalue)
#
#range(res_stat$minuslog10)
#range(res_stat$log2FoldChange)

names(res_stat)[names(res_stat) == 'gene'] <- 'Genus'
Feature2<-merge(res_stat, Feature, by="Genus")

#Create column org grouping for colouring
Feature2$Level_1 <- ifelse(Feature2$Level_1=="Environmental Information Processing",
                           "Environmental Information Processing",
          ifelse(Feature2$Level_1=="Cellular Processes", "Cellular Processes",
          ifelse(Feature2$Level_1=="Metabolism", "Metabolism",
          ifelse(Feature2$Level_1=="Genetic Information Processing", "Genetic Information Processing",
          ifelse(Feature2$Level_1=="Unclassified metabolism", "Unclassified metabolism",
                 "Other")))))
#Order Level_1 for plotting
Feature2$Level_1 <- factor(Feature2$Level_1, levels=c("Cellular Processes", "Genetic Information Process
                                                  "Metabolism", "Environmental Information Processing",
                                                  "Unclassified metabolism", "Other"))


##Summary significant orgs
#aggregate(Plotting[, 17:ncol(Plotting)], list(Plotting$Diagnosis), mean)
#aggregate(Plotting[, 17:ncol(Plotting)], list(Plotting$Diagnosis), sd)
zeroes <- function(x){
  sum(x == 0)
}
#aggregate(Plotting[, 17:ncol(Plotting)], list(Plotting$Diagnosis), zeroes)
zerocounts <- aggregate(Plotting[, 17:ncol(Plotting)], list(Plotting$Diagnosis), zeroes)
#bind_cols(zerocounts[,1], zerocounts[,2:ncol(zerocounts)]/c(70,30,70,70)*100)


#Add prevalence to plotting
prevalence<-data.frame((240-apply(Taxonomy, 1, zeroes))/240*100)
colnames(prevalence) <- c("Prevalence")
prevalence<-add_rownames(prevalence, var = "Genus")
Feature2 <- merge(Feature2, prevalence, by="Genus")


#Define boundaries
#Always run first without these lines to get indication of very low and high
#log2FoldChange and pvalue
Featurein <- filter(Feature2, -3<log2FoldChange & log2FoldChange<3 & 10>minuslog10)
```

```
Featureout <- filter(Feature2, -3>log2FoldChange | 3<log2FoldChange | 10<minuslog10)
Featureout$log2FoldChange[Featureout$log2FoldChange > 3] <- 3
Featureout$log2FoldChange[Featureout$log2FoldChange < (-3)] <- -3
Featureout$minuslog10[Featureout$minuslog10 > 10] <- 10


#Add text to vulcano plots
p1<-ggplot() +
  geom_point(data=Featurein[which(Featurein$Level_1=="Cellular Processes" &
                                    Featurein$compare=="LADA vs T1D"), ],
             aes(x=log2FoldChange, y=minuslog10, group=Genus), color="#A6CEE3", shape=16,
             size=Featurein[which(Featurein$Level_1=="Cellular Processes" &
                                    Featurein$compare=="LADA vs T1D"), ]
             [,ncol(Featurein)]/40+0.5) +
  #did not add jitter to the first on purpose. Sizes are scaled from percent
  #to the range 0.5-3
  geom_point(data=Featureout[which(Featureout$Level_1=="Cellular Processes" &
                                     Featureout$compare=="LADA vs T1D"), ],
             aes(x=log2FoldChange, y=minuslog10, group=Genus), color="#A6CEE3", shape=18,
             size=Featureout[which(Featureout$Level_1=="Cellular Processes" &
                                     Featureout$compare=="LADA vs T1D"), ]
             [,ncol(Featureout)]/40+0.5) +
  geom_point(data=Featurein[which(Featurein$Level_1=="Genetic Information Processing" &
                                    Featurein$compare=="LADA vs T1D"), ],
             aes(x=log2FoldChange, y=minuslog10, group=Genus), color="#FB9A99", shape=16,
             size=Featurein[which(Featurein$Level_1=="Genetic Information Processing" &
                                    Featurein$compare=="LADA vs T1D"), ]
             [,ncol(Featurein)]/40+0.5) +
  geom_jitter(data=Featureout[which(Featureout$Level_1=="Genetic Information Processing" &
                                      Featureout$compare=="LADA vs T1D"), ],
             aes(x=log2FoldChange, y=minuslog10, group=Genus), color="#FB9A99", shape=18,
             size=Featureout[which(Featureout$Level_1=="Genetic Information Processing" &
                                     Featureout$compare=="LADA vs T1D"), ]
             [,ncol(Featureout)]/40+0.5, width = 0.1, height = 0.1) +
  geom_point(data=Featurein[which(Featurein$Level_1=="Metabolism" &
                                    Featurein$compare=="LADA vs T1D"), ],
             aes(x=log2FoldChange, y=minuslog10, group=Genus), color="#FDBF6F", shape=16,
             size=Featurein[which(Featurein$Level_1=="Metabolism" &
                                    Featurein$compare=="LADA vs T1D"), ]
             [,ncol(Featurein)]/40+0.5) +
  geom_jitter(data=Featureout[which(Featureout$Level_1=="Metabolism" &
                                      Featureout$compare=="LADA vs T1D"), ],
             aes(x=log2FoldChange, y=minuslog10, group=Genus), color="#FDBF6F", shape=18,
             size=Featureout[which(Featureout$Level_1=="Metabolism" &
                                     Featureout$compare=="LADA vs T1D"), ]
             [,ncol(Featureout)]/40+0.5, width = 0.1, height = 0.1) +
  geom_point(data=Featurein[which(Featurein$Level_1=="Environmental Information Processing" &
                                    Featurein$compare=="LADA vs T1D"), ],
             aes(x=log2FoldChange, y=minuslog10, group=Genus),
             color="#B2DF8A", shape=16, alpha=0.75,
             size=Featurein[which(Featurein$Level_1=="Environmental Information Processing" &
                                    Featurein$compare=="LADA vs T1D"), ]
             [,ncol(Featurein)]/40+0.5) +
```

```
  geom_jitter(data=Featureout[which(Featureout$Level_1=="Environmental Information Processing" &
                                     Featureout$compare=="LADA vs T1D"), ],
              aes(x=log2FoldChange, y=minuslog10, group=Genus),
              color="#B2DF8A", shape=18,
              size=Featureout[which(Featureout$Level_1=="Environmental Information Processing" &
                                     Featureout$compare=="LADA vs T1D"), ]
              [,ncol(Featureout)]/40+0.5, width = 0.1, height = 0.1) +
  geom_point(data=Featurein[which(Featurein$Level_1=="Unclassified metabolism" &
                                   Featurein$compare=="LADA vs T1D"), ],
              aes(x=log2FoldChange, y=minuslog10, group=Genus), color="#CAB2D6", shape=16,
              size=Featurein[which(Featurein$Level_1=="Unclassified metabolism" &
                                   Featurein$compare=="LADA vs T1D"), ]
              [,ncol(Featurein)]/40+0.5) +
  geom_jitter(data=Featureout[which(Featureout$Level_1=="Unclassified metabolism" &
                                     Featureout$compare=="LADA vs T1D"), ],
              aes(x=log2FoldChange, y=minuslog10, group=Genus), color="#CAB2D6", shape=18,
              size=Featureout[which(Featureout$Level_1=="Unclassified metabolism" &
                                     Featureout$compare=="LADA vs T1D"), ]
              [,ncol(Featureout)]/40+0.5, width = 0.1, height = 0.1) +
  geom_point(data=Featurein[which(Featurein$Level_1=="Other" &
                                   Featurein$compare=="LADA vs T1D"), ],
              aes(x=log2FoldChange, y=minuslog10, group=Genus), color="#B3B3B3", shape=16,
              size=Featurein[which(Featurein$Level_1=="Other" &
                                   Featurein$compare=="LADA vs T1D"), ]
              [,ncol(Featurein)]/40+0.5) +
  geom_jitter(data=Featureout[which(Featureout$Level_1=="Other" &
                                     Featureout$compare=="LADA vs T1D"), ],
              aes(x=log2FoldChange, y=minuslog10, group=Genus), color="#B3B3B3", shape=18,
              size=Featureout[which(Featureout$Level_1=="Other" &
                                     Featureout$compare=="LADA vs T1D"), ]
              [,ncol(Featureout)]/40+0.5, width = 0.1, height = 0.1) +
  geom_text(data=Featurein[which(Featurein$padj<padj_threshold &
                                  Featurein$compare=="LADA vs T1D"), ],
              aes(x=log2FoldChange, y=minuslog10, group=Genus, label=Genus),
             nudge_y = 0.2, size=2.5) +
  geom_text(data=Featureout[which(Featureout$padj<padj_threshold &
                                   Featureout$compare=="LADA vs T1D"), ],
              aes(x=log2FoldChange, y=minuslog10, group=Genus, label=Genus),
             nudge_x=0.6, nudge_y = -0.2, size=2.5) +
#  geom_text(data=Featureout[which(Featureout$padj<padj_threshold &
#                                    Featureout$compare=="LADA vs T1D" &
#                                    Featureout$Genus=="Rikenellaceae_RC9_gut_group"), ],
#              aes(x=log2FoldChange, y=minuslog10, group=Genus, label=Genus),
#             nudge_x=1.22, nudge_y = 0) +
  geom_hline(yintercept = -log(0.05), linetype="dashed") +
  geom_hline(yintercept = -log(BHLadaT1D), linetype="dashed", alpha=0.1) +
  geom_vline(xintercept = -1, linetype="dashed") +
  geom_vline(xintercept = 1, linetype="dashed") +
  theme_bw() +
  ggtitle("T1D") +
  xlab("Log2 fold change") +
  ylab("-log10(P)") +
  theme(panel.grid.major = element_blank(),
```

```
        panel.grid.minor = element_blank(),
        axis.title=element_text(size=22),
        legend.position="bottom",
        legend.title=element_text(size=20),
        legend.text=element_text(size=20),
        axis.text.x = element_text(angle = 45, hjust = 1, size=12),
        axis.text.y = element_text(angle = 45, hjust = 1, size=12),
        plot.title = element_text(size = 22, hjust=0.5))
#ggplotly(p1)
p1
```

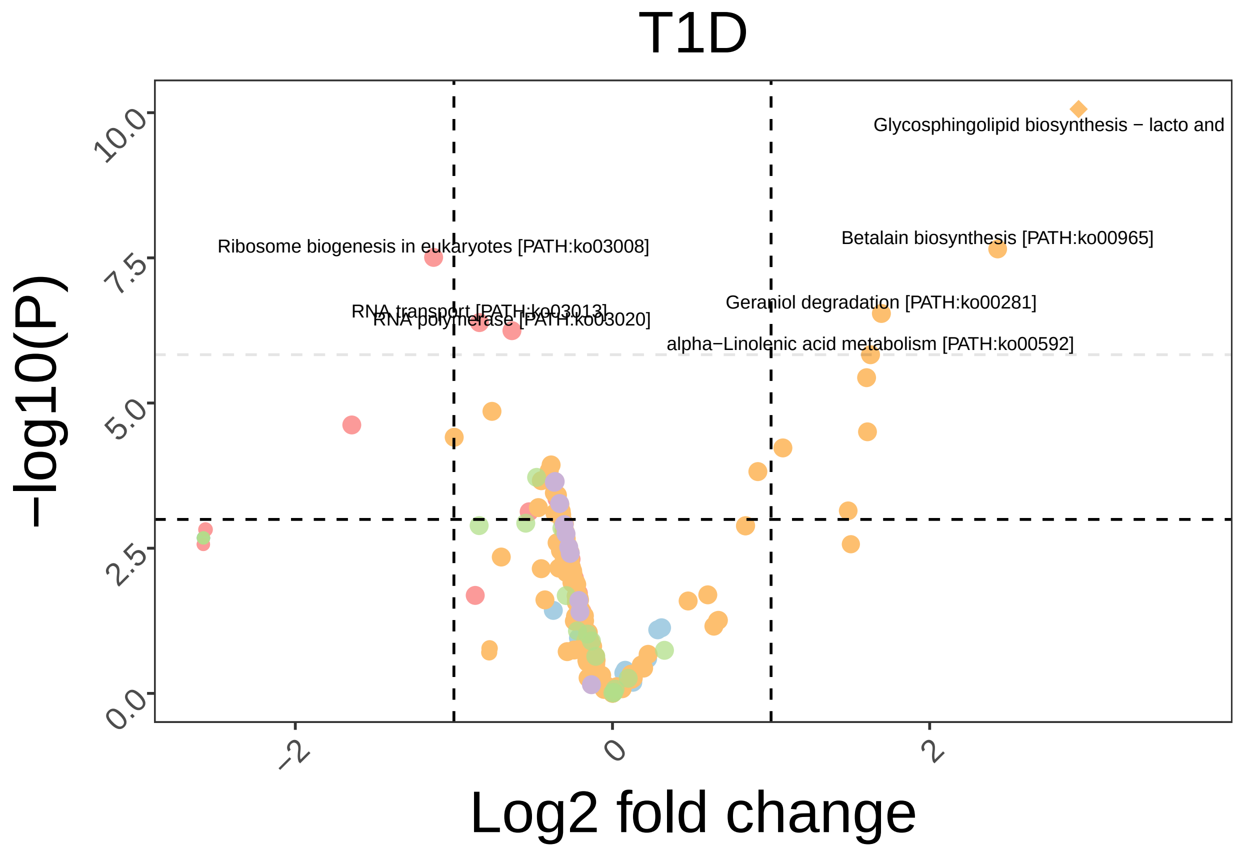


```
Fig2ListRemMet[["vulcLadaT1D"]] <- p1


p2<-ggplot() +
  geom_point(data=Featurein[which(Featurein$Level_1=="Cellular Processes" &
                                  Featurein$compare=="LADA vs T2D"), ],
             aes(x=log2FoldChange, y=minuslog10, group=Genus), color="#A6CEE3", shape=16,
             size=Featurein[which(Featurein$Level_1=="Cellular Processes" &
                                  Featurein$compare=="LADA vs T2D"), ]
             [,ncol(Featurein)]/40+0.5) +
  #did not add jitter to the first on purpose. Sizes are scaled from percent
  #to the range 0.5-3
  geom_point(data=Featureout[which(Featureout$Level_1=="Cellular Processes" &
                                   Featureout$compare=="LADA vs T2D"), ],
```

```
           aes(x=log2FoldChange, y=minuslog10, group=Genus), color="#A6CEE3", shape=18,
           size=Featureout[which(Featureout$Level_1=="Cellular Processes" &
                                  Featureout$compare=="LADA vs T2D"), ]
           [,ncol(Featureout)]/40+0.5) +
  geom_point(data=Featurein[which(Featurein$Level_1=="Genetic Information Processing" &
                                  Featurein$compare=="LADA vs T2D"), ],
           aes(x=log2FoldChange, y=minuslog10, group=Genus), color="#FB9A99", shape=16,
           size=Featurein[which(Featurein$Level_1=="Genetic Information Processing" &
                                  Featurein$compare=="LADA vs T2D"), ]
           [,ncol(Featurein)]/40+0.5) +
  geom_jitter(data=Featureout[which(Featureout$Level_1=="Genetic Information Processing" &
                                    Featureout$compare=="LADA vs T2D"), ],
           aes(x=log2FoldChange, y=minuslog10, group=Genus), color="#FB9A99", shape=18,
           size=Featureout[which(Featureout$Level_1=="Genetic Information Processing" &
                                  Featureout$compare=="LADA vs T2D"), ]
           [,ncol(Featureout)]/40+0.5,
           width = 0.1, height = 0.1) +
  geom_point(data=Featurein[which(Featurein$Level_1=="Metabolism" &
                                  Featurein$compare=="LADA vs T2D"), ],
           aes(x=log2FoldChange, y=minuslog10, group=Genus), color="#FDBF6F", shape=16,
           size=Featurein[which(Featurein$Level_1=="Metabolism" &
                                  Featurein$compare=="LADA vs T2D"), ]
           [,ncol(Featurein)]/40+0.5) +
  geom_jitter(data=Featureout[which(Featureout$Level_1=="Metabolism" &
                                    Featureout$compare=="LADA vs T2D"), ],
           aes(x=log2FoldChange, y=minuslog10, group=Genus), color="#FDBF6F", shape=18,
           size=Featureout[which(Featureout$Level_1=="Metabolism" &
                                  Featureout$compare=="LADA vs T2D"), ]
           [,ncol(Featureout)]/40+0.5, width = 0.1, height = 0.1) +
  geom_point(data=Featurein[which(Featurein$Level_1=="Environmental Information Processing" &
                                  Featurein$compare=="LADA vs T2D"), ],
           aes(x=log2FoldChange, y=minuslog10, group=Genus),
           color="#B2DF8A", shape=16, alpha=0.75,
           size=Featurein[which(Featurein$Level_1=="Environmental Information Processing" &
                                  Featurein$compare=="LADA vs T2D"), ]
           [,ncol(Featurein)]/40+0.5) +
  geom_jitter(data=Featureout[which(Featureout$Level_1=="Environmental Information Processing" &
                                    Featureout$compare=="LADA vs T2D"), ],
           aes(x=log2FoldChange, y=minuslog10, group=Genus),
           color="#B2DF8A", shape=18,
           size=Featureout[which(Featureout$Level_1=="Environmental Information Processing" &
                                  Featureout$compare=="LADA vs T2D"), ]
           [,ncol(Featureout)]/40+0.5, width = 0.1, height = 0.1) +
  geom_point(data=Featurein[which(Featurein$Level_1=="Unclassified metabolism" &
                                  Featurein$compare=="LADA vs T2D"), ],
           aes(x=log2FoldChange, y=minuslog10, group=Genus), color="#CAB2D6", shape=16,
           size=Featurein[which(Featurein$Level_1=="Unclassified metabolism" &
                                  Featurein$compare=="LADA vs T2D"), ]
           [,ncol(Featurein)]/40+0.5) +
  geom_jitter(data=Featureout[which(Featureout$Level_1=="Unclassified metabolism" &
                                    Featureout$compare=="LADA vs T2D"), ],
           aes(x=log2FoldChange, y=minuslog10, group=Genus), color="#CAB2D6", shape=18,
           size=Featureout[which(Featureout$Level_1=="Unclassified metabolism" &
```

```r
                                  Featureout$compare=="LADA vs T2D"), ]
            [,ncol(Featureout)]/40+0.5, width = 0.1, height = 0.1) +
  geom_point(data=Featurein[which(Featurein$Level_1=="Other" &
                                 Featurein$compare=="LADA vs T2D"), ],
            aes(x=log2FoldChange, y=minuslog10, group=Genus), color="#B3B3B3", shape=16,
            size=Featurein[which(Featurein$Level_1=="Other" &
                                 Featurein$compare=="LADA vs T2D"), ]
            [,ncol(Featurein)]/40+0.5) +
  geom_jitter(data=Featureout[which(Featureout$Level_1=="Other" &
                                   Featureout$compare=="LADA vs T2D"), ],
            aes(x=log2FoldChange, y=minuslog10, group=Genus), color="#B3B3B3", shape=18,
            size=Featureout[which(Featureout$Level_1=="Other" &
                                  Featureout$compare=="LADA vs T2D"), ]
            [,ncol(Featureout)]/40+0.5, width = 0.1, height = 0.1) +
  geom_text(data=Featurein[which(Featurein$padj<padj_threshold &
                                Featurein$compare=="LADA vs T2D"), ],
            aes(x=log2FoldChange, y=minuslog10, group=Genus, label=Genus),
           nudge_y = 0.2, size=2.5) +
  geom_text(data=Featureout[which(Featureout$padj<padj_threshold &
                                 Featureout$compare=="LADA vs T2D"), ],
            aes(x=log2FoldChange, y=minuslog10, group=Genus, label=Genus),
           nudge_x=0.6, nudge_y = -0.2, size=2.5) +
#  geom_text(data=Featureout[which(Featureout$padj<padj_threshold &
#                                  Featureout$compare=="LADA vs T1D" &
#                                  Featureout$Genus=="Rikenellaceae_RC9_gut_group"), ],
#             aes(x=log2FoldChange, y=minuslog10, group=Genus, label=Genus),
#            nudge_x=1.22, nudge_y = 0) +
  geom_hline(yintercept = -log(0.05), linetype="dashed") +
  geom_hline(yintercept = -log(BHLadaT2D), linetype="dashed", alpha=0.1) +
  geom_vline(xintercept = -1, linetype="dashed") +
  geom_vline(xintercept = 1, linetype="dashed") +
  theme_bw() +
  ggtitle("T2D") +
  xlab("Log2 fold change") +
  ylab("-log10(P)") +
  theme(panel.grid.major = element_blank(),
        panel.grid.minor = element_blank(),
        axis.title=element_text(size=22),
        legend.position="bottom",
        legend.title=element_text(size=20),
        legend.text=element_text(size=20),
        axis.text.x = element_text(angle = 45, hjust = 1, size=12),
        axis.text.y = element_text(angle = 45, hjust = 1, size=12),
        plot.title = element_text(size = 22, hjust=0.5))
#ggplotly(p2)
p2
```

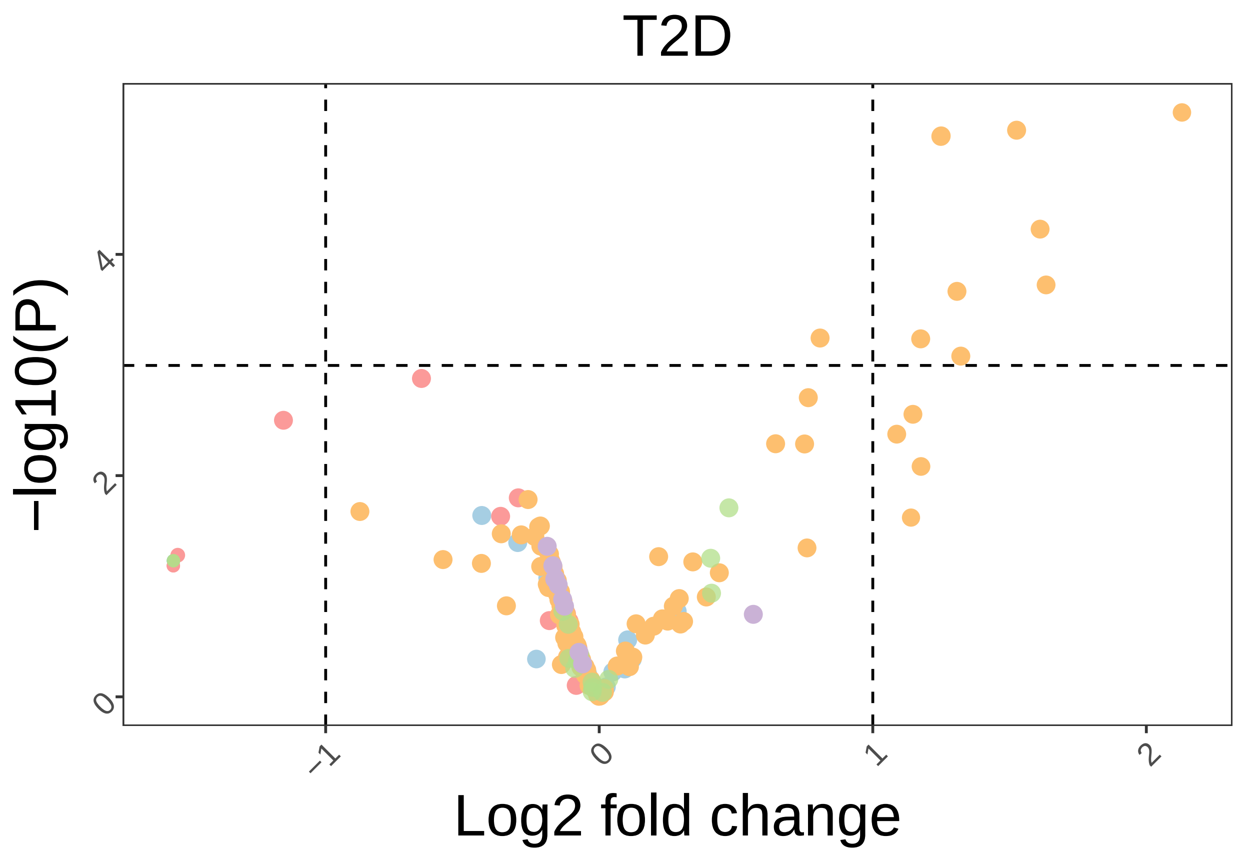


```
Fig2ListRemMet[["vulcLadaT2D"]] <- p2


p3<-ggplot() +
  geom_point(data=Featurein[which(Featurein$Level_1=="Cellular Processes" &
                                  Featurein$compare=="LADA vs Control"), ],
             aes(x=log2FoldChange, y=minuslog10, group=Genus), color="#A6CEE3", shape=16,
             size=Featurein[which(Featurein$Level_1=="Cellular Processes" &
                                  Featurein$compare=="LADA vs Control"), ]
             [,ncol(Featurein)]/40+0.5) +
  #did not add jitter to the first on purpose. Sizes are scaled from percent to the range 0.5-3
  geom_point(data=Featureout[which(Featureout$Level_1=="Cellular Processes" &
                                   Featureout$compare=="LADA vs Control"), ],
             aes(x=log2FoldChange, y=minuslog10, group=Genus), color="#A6CEE3", shape=18,
             size=Featureout[which(Featureout$Level_1=="Cellular Processes" &
                                   Featureout$compare=="LADA vs Control"), ]
             [,ncol(Featureout)]/40+0.5) +
  geom_point(data=Featurein[which(Featurein$Level_1=="Genetic Information Processing" &
                                  Featurein$compare=="LADA vs Control"), ],
             aes(x=log2FoldChange, y=minuslog10, group=Genus), color="#FB9A99", shape=16,
             size=Featurein[which(Featurein$Level_1=="Genetic Information Processing" &
                                  Featurein$compare=="LADA vs Control"), ]
             [,ncol(Featurein)]/40+0.5) +
  geom_jitter(data=Featureout[which(Featureout$Level_1=="Genetic Information Processing" &
                                    Featureout$compare=="LADA vs Control"), ],
             aes(x=log2FoldChange, y=minuslog10, group=Genus), color="#FB9A99", shape=18,
```

```
            size=Featureout[which(Featureout$Level_1=="Genetic Information Processing" &
                                        Featureout$compare=="LADA vs Control"), ]
            [,ncol(Featureout)]/40+0.5, width = 0.1, height = 0.1) +
geom_point(data=Featurein[which(Featurein$Level_1=="Metabolism" &
                                       Featurein$compare=="LADA vs Control"), ],
            aes(x=log2FoldChange, y=minuslog10, group=Genus), color="#FDBF6F", shape=16,
            size=Featurein[which(Featurein$Level_1=="Metabolism" &
                                       Featurein$compare=="LADA vs Control"), ]
            [,ncol(Featurein)]/40+0.5) +
geom_jitter(data=Featureout[which(Featureout$Level_1=="Metabolism" &
                                         Featureout$compare=="LADA vs Control"), ],
            aes(x=log2FoldChange, y=minuslog10, group=Genus), color="#FDBF6F", shape=18,
            size=Featureout[which(Featureout$Level_1=="Metabolism" &
                                        Featureout$compare=="LADA vs Control"), ]
            [,ncol(Featureout)]/40+0.5, width = 0.1, height = 0.1) +
geom_point(data=Featurein[which(Featurein$Level_1=="Environmental Information Processing" &
                                       Featurein$compare=="LADA vs Control"), ],
            aes(x=log2FoldChange, y=minuslog10, group=Genus),
            color="#B2DF8A", shape=16, alpha=0.75,
            size=Featurein[which(Featurein$Level_1=="Environmental Information Processing" &
                                       Featurein$compare=="LADA vs Control"), ]
            [,ncol(Featurein)]/40+0.5) +
geom_jitter(data=Featureout[which(Featureout$Level_1=="Environmental Information Processing" &
                                         Featureout$compare=="LADA vs Control"), ],
            aes(x=log2FoldChange, y=minuslog10, group=Genus),
            color="#B2DF8A", shape=18,
            size=Featureout[which(Featureout$Level_1=="Environmental Information Processing" &
                                        Featureout$compare=="LADA vs Control"), ]
            [,ncol(Featureout)]/40+0.5, width = 0.1, height = 0.1) +
geom_point(data=Featurein[which(Featurein$Level_1=="Unclassified metabolism" &
                                       Featurein$compare=="LADA vs Control"), ],
            aes(x=log2FoldChange, y=minuslog10, group=Genus), color="#CAB2D6", shape=16,
            size=Featurein[which(Featurein$Level_1=="Unclassified metabolism" &
                                       Featurein$compare=="LADA vs Control"), ]
            [,ncol(Featurein)]/40+0.5) +
geom_jitter(data=Featureout[which(Featureout$Level_1=="Unclassified metabolism" &
                                         Featureout$compare=="LADA vs Control"), ],
            aes(x=log2FoldChange, y=minuslog10, group=Genus), color="#CAB2D6", shape=18,
            size=Featureout[which(Featureout$Level_1=="Unclassified metabolism" &
                                        Featureout$compare=="LADA vs Control"), ]
            [,ncol(Featureout)]/40+0.5, width = 0.1, height = 0.1) +
geom_point(data=Featurein[which(Featurein$Level_1=="Other" &
                                       Featurein$compare=="LADA vs Control"), ],
            aes(x=log2FoldChange, y=minuslog10, group=Genus), color="#B3B3B3", shape=16,
            size=Featurein[which(Featurein$Level_1=="Other" &
                                       Featurein$compare=="LADA vs Control"), ]
            [,ncol(Featurein)]/40+0.5) +
geom_jitter(data=Featureout[which(Featureout$Level_1=="Other" &
                                         Featureout$compare=="LADA vs Control"), ],
            aes(x=log2FoldChange, y=minuslog10, group=Genus), color="#B3B3B3", shape=18,
            size=Featureout[which(Featureout$Level_1=="Other" &
                                        Featureout$compare=="LADA vs Control"), ]
            [,ncol(Featureout)]/40+0.5, width = 0.1, height = 0.1) +
```

```
  geom_text(data=Featurein[which(Featurein$padj<padj_threshold &
                                  Featurein$compare=="LADA vs Control"), ],
            aes(x=log2FoldChange, y=minuslog10, group=Genus, label=Genus),
           nudge_y = 0.2, size=2.5) +
  geom_text(data=Featureout[which(Featureout$padj<padj_threshold &
                                   Featureout$compare=="LADA vs Control"), ],
            aes(x=log2FoldChange, y=minuslog10, group=Genus, label=Genus),
           nudge_x=0.6, nudge_y = -0.2, size=2.5) +
#  geom_text(data=Featureout[which(Featureout$padj<padj_threshold &
#                                   Featureout$compare=="LADA vs T1D" &
#                                   Featureout$Genus=="Rikenellaceae_RC9_gut_group"), ],
#             aes(x=log2FoldChange, y=minuslog10, group=Genus, label=Genus),
#            nudge_x=1.22, nudge_y = 0) +
  geom_hline(yintercept = -log(0.05), linetype="dashed") +
  geom_hline(yintercept = -log(BHLadaCon), linetype="dashed", alpha=0.1) +
  geom_vline(xintercept = -1, linetype="dashed") +
  geom_vline(xintercept = 1, linetype="dashed") +
  theme_bw() +
  ggtitle("Controls") +
  xlab("Log2 fold change") +
  ylab("-log10(P)") +
  theme(panel.grid.major = element_blank(),
        panel.grid.minor = element_blank(),
        axis.title=element_text(size=22),
        legend.position="bottom",
        legend.title=element_text(size=20),
        legend.text=element_text(size=20),
        axis.text.x = element_text(angle = 45, hjust = 1, size=12),
        axis.text.y = element_text(angle = 45, hjust = 1, size=12),
        plot.title = element_text(size = 22, hjust=0.5))
#ggplotly(p3)
p3
```

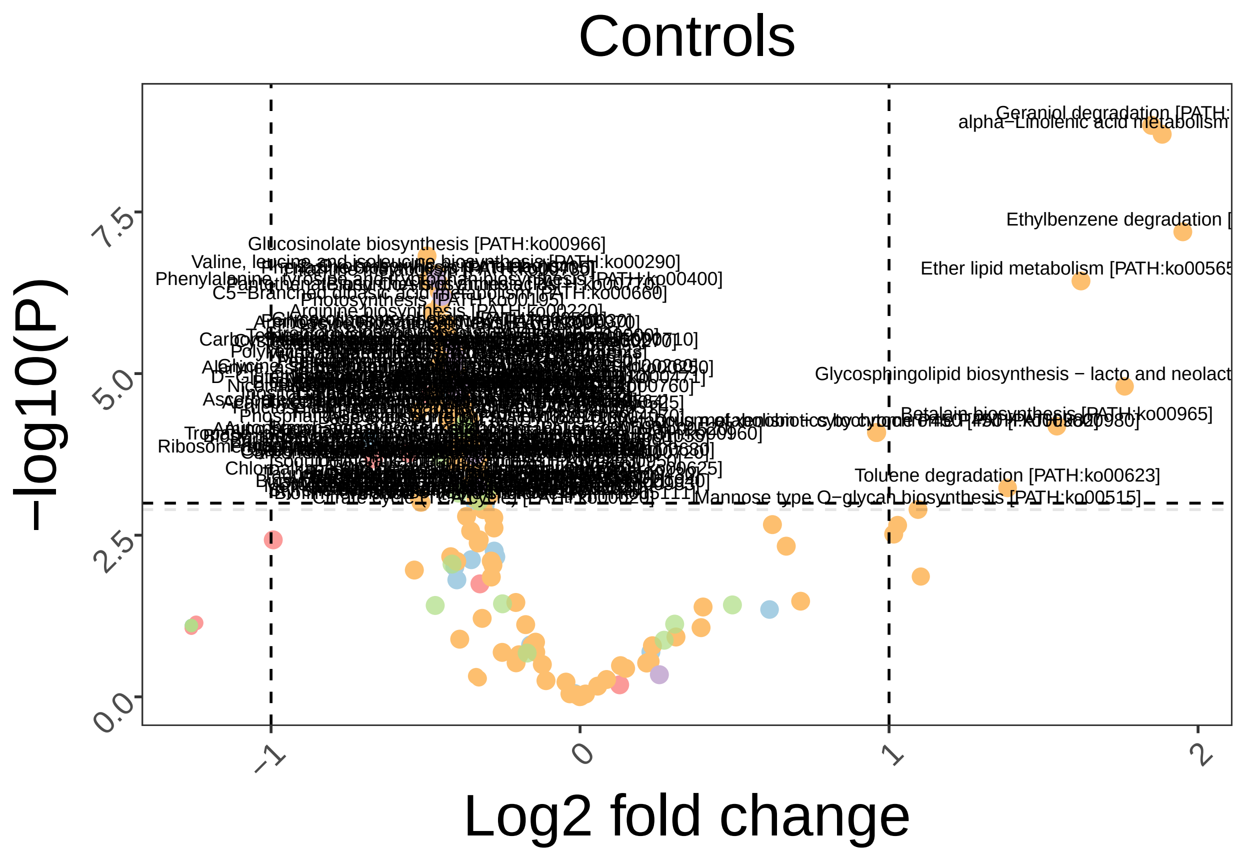


```
Fig2ListRemMet[["vulcLadaControl"]] <- p3

lay <- rbind(c(1,2,3))
pdf(paste("MicroLADA_VulcanoRemMet_Func.pdf", sep=""), width=15, height=5)
grid.arrange(p1,p2,p3,layout_matrix = lay)
dev.off()
```

pdf 2

```
##Boxplot with stats intertwined
#Stats have to be in the end of the chunk don't ask me why
#Have to use the same naming in res_stat to output tables
res_stat$Genus<-str_replace_all(res_stat$Genus, " ","_")
res_stat$Genus<-str_replace_all(res_stat$Genus, "/","_")
res_stat$Genus<-gsub("\\[|\\]", "", res_stat$Genus)
res_stat$Genus<-str_replace_all(res_stat$Genus, ":","_")
res_stat$Genus<-str_replace_all(res_stat$Genus, "-","_")
res_stat$Genus<-str_replace_all(res_stat$Genus, ",","_")
res_stat$Genus<-str_replace_all(res_stat$Genus, "2_","Two_")
res_stat$Genus<-str_replace_all(res_stat$Genus, "\\(","_")
res_stat$Genus<-str_replace_all(res_stat$Genus, "\\)","_")

#Order Diagnosis
Plotting2$Diagnosis<-ordered(Plotting2$Diagnosis,
                             levels=c("Control", "T1D", "LADA", "T2D"))
```

```r
for (i in SelOrgs) {
Boxplot <-
ggplot(Plotting2, aes_string(x="Diagnosis", y=i, group="Diagnosis")) +
    geom_boxplot() +
#    geom_boxplot(aes(fill=Diagnosis, trim=FALSE)) +
#    geom_jitter() +
    geom_jitter(aes(color=Diagnosis), size=2) +
    stat_summary(fun.data="mean_sdl",
                 mult=1, #mean plus minus a constant (mult=1) times the st.dev
                 geom="pointrange",
                 width=0.2 ) +
    #stat_summary(fun.y = mean, geom = "point") +
    #facet_grid(. ~ Metformin, scales="fixed") + #Scales can also be "free"
    ggtitle(i) +
    #xlab("Diagnosis") +
    ylab("Cells pr. g feces") +
    scale_fill_manual(values=c(Control = "#666666", T1D = "#1F78B4",
                               T2D = "#33A02C", LADA = "#E31A1C")) +
    scale_color_manual(values=c(Control = "#666666", T1D = "#1F78B4",
                               T2D = "#33A02C", LADA = "#E31A1C")) +
    theme_bw() +
    theme(legend.position="none",
          title =element_text(size=18, face='bold'),
          panel.grid.major = element_blank(),
          panel.grid.minor = element_blank(),
          axis.title=element_text(size=16),
          axis.title.x = element_blank(),
          axis.text.x = element_text(angle = 45, hjust = 1, size=14),
          axis.text.y = element_text(angle = 45, hjust = 1, size=12))
Fig2ListRemMet[[i]] <- Boxplot
#print(kable(stat_sig[which(stat_sig$gene==i), c(5,6,7,10)]))
cat("\n")
tabling<-res_stat[which(res_stat$Genus==i), c(5,6,7,10)]
print(kable(tabling))
#print(tabling)
cat("\n")
print(Boxplot)
}
```

	pvalue	padj	Genus	compare
895	0.0000354	0.0069458	Glycosphingolipid_biosynthesis____lacto_and_neolacto_series_PATH_ko00601	LADA vs T1D
503	0.0082172	0.0346055	Glycosphingolipid_biosynthesis____lacto_and_neolacto_series_PATH_ko00601	LADA vs Control
111	0.0072064	0.2017796	Glycosphingolipid_biosynthesis____lacto_and_neolacto_series_PATH_ko00601	Control vs T1D
1091	0.0050767	0.3088442	Glycosphingolipid_biosynthesis____lacto_and_neolacto_series_PATH_ko00601	LADA vs T2D
699	0.1346867	0.6495082	Glycosphingolipid_biosynthesis____lacto_and_neolacto_series_PATH_ko00601	T1D vs T2D
307	0.4738221	NA	Glycosphingolipid_biosynthesis____lacto_and_neolacto_series_PATH_ko00601	Control vs T2D

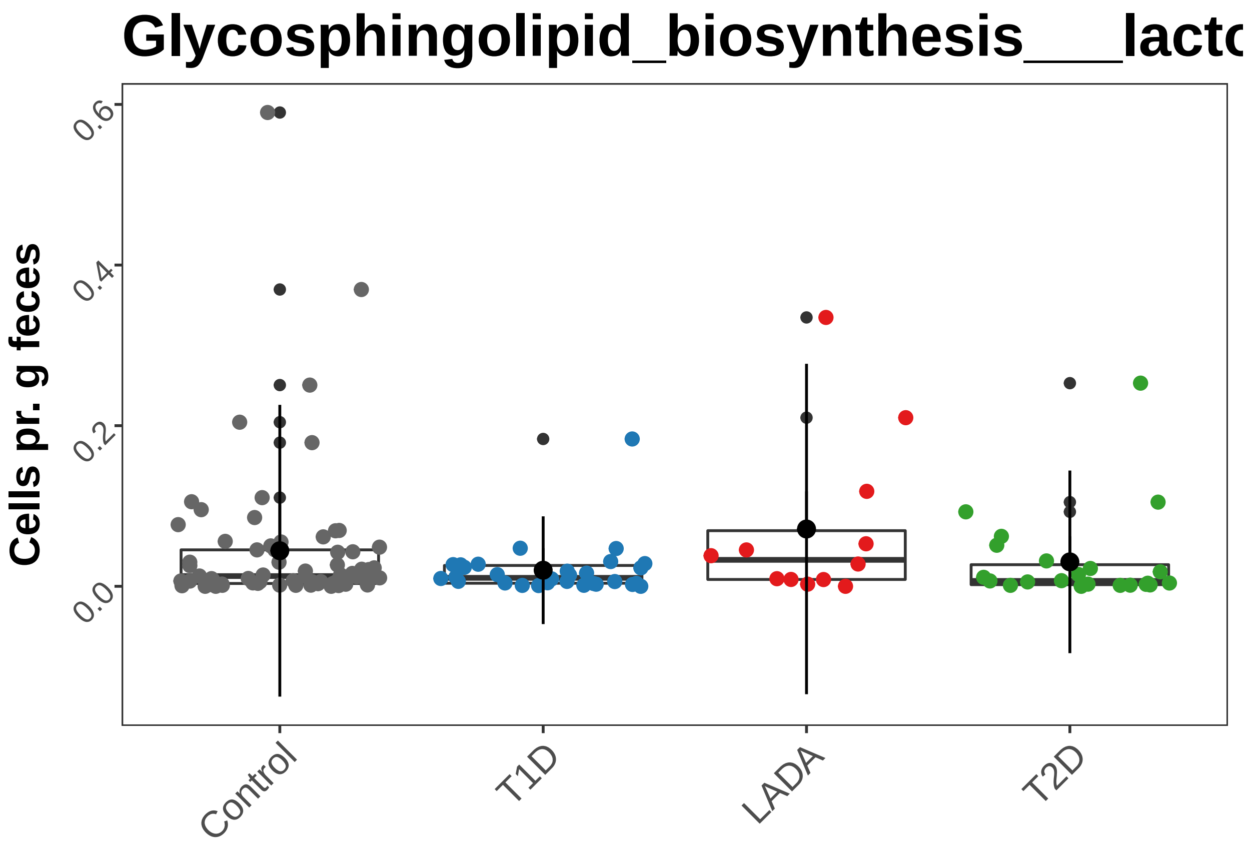

	pvalue	padj	Genus	compare
552	0.0001449	0.0162870	Geraniol_degradation_PATH_ko00281	LADA vs Control
944	0.0014410	0.0632892	Geraniol_degradation_PATH_ko00281	LADA vs T1D
1140	0.0059609	0.3088442	Geraniol_degradation_PATH_ko00281	LADA vs T2D
160	0.6509860	0.7594837	Geraniol_degradation_PATH_ko00281	Control vs T1D
748	0.6939565	0.7862166	Geraniol_degradation_PATH_ko00281	T1D vs T2D
356	0.3872905	NA	Geraniol_degradation_PATH_ko00281	Control vs T2D

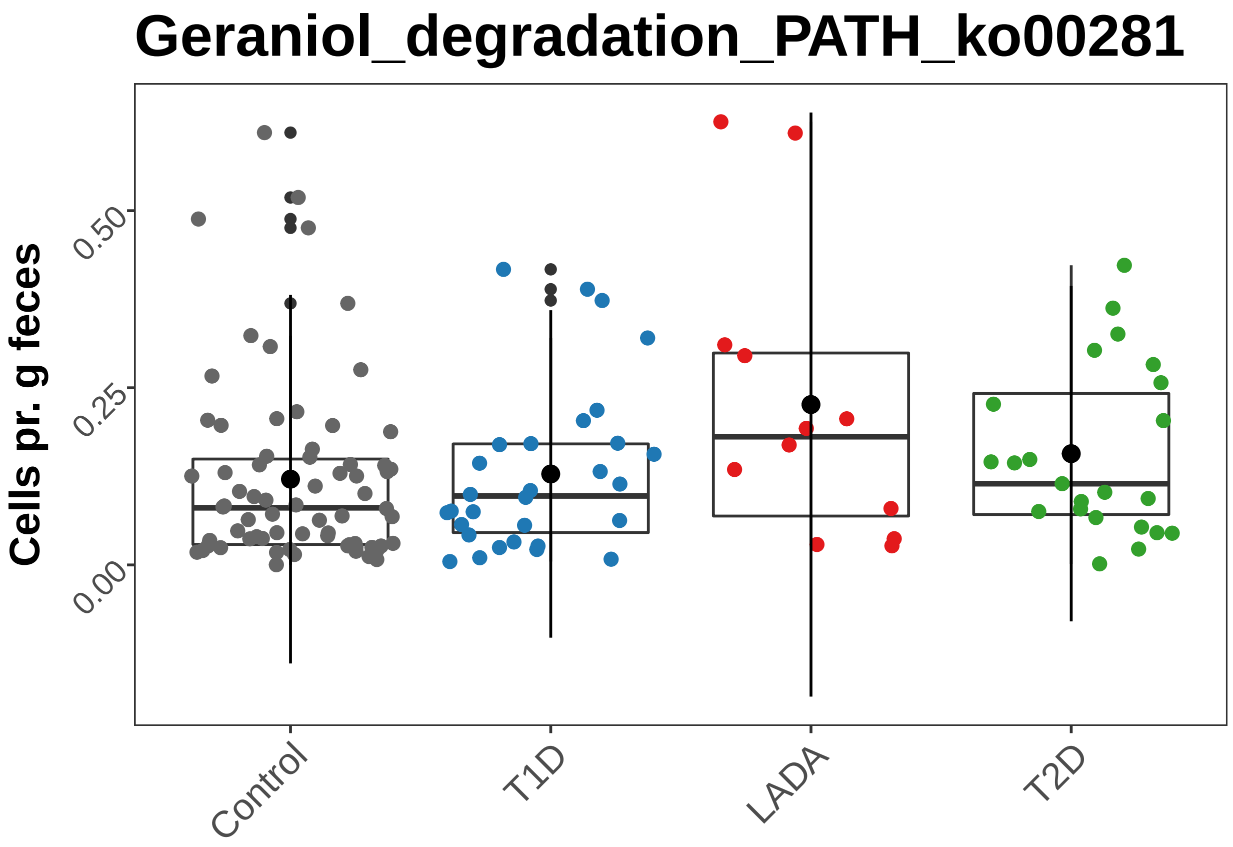

	pvalue	padj	Genus	compare
512	0.0001662	0.0162870	alpha__Linolenic__acid__metabolism__PATH__ko00592LADA vs Control	
904	0.0029317	0.0820872	alpha__Linolenic__acid__metabolism__PATH__ko00592LADA vs T1D	
120	0.4626422	0.6399468	alpha__Linolenic__acid__metabolism__PATH__ko00592Control vs T1D	
708	0.3086442	0.6495082	alpha__Linolenic__acid__metabolism__PATH__ko00592T1D vs T2D	
1100	0.0393109	0.8415375	alpha__Linolenic__acid__metabolism__PATH__ko00592LADA vs T2D	
316	0.0656192	NA	alpha__Linolenic__acid__metabolism__PATH__ko00592Control vs T2D	

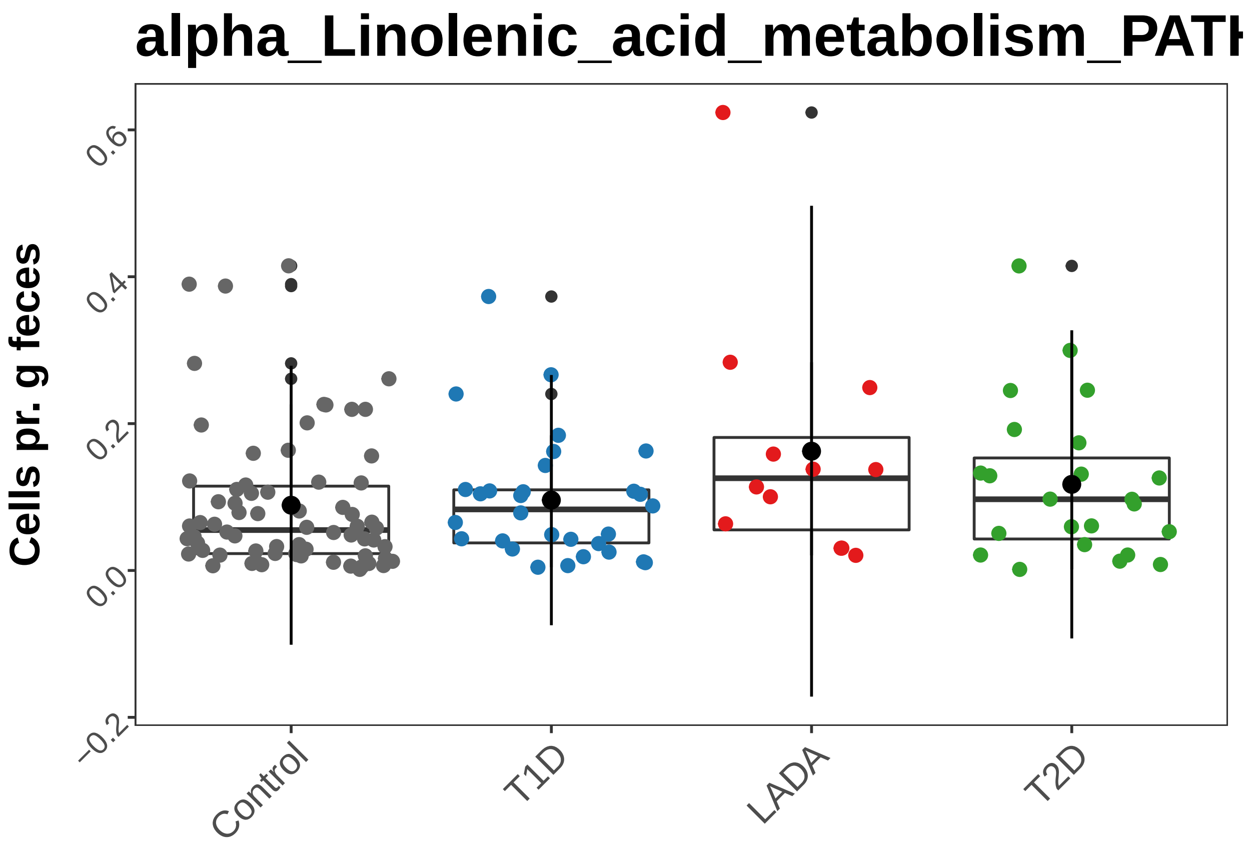

	pvalue	padj	Genus	compare
571	0.0007520	0.0333090	Ethylbenzene_degradation_PATH_ko00642	LADA vs Control
963	0.0110563	0.1970026	Ethylbenzene_degradation_PATH_ko00642	LADA vs T1D
1159	0.0145780	0.5714587	Ethylbenzene_degradation_PATH_ko00642	LADA vs T2D
179	0.3968882	0.5762229	Ethylbenzene_degradation_PATH_ko00642	Control vs T1D
767	0.9943129	0.9943129	Ethylbenzene_degradation_PATH_ko00642	T1D vs T2D
375	0.4466309	NA	Ethylbenzene_degradation_PATH_ko00642	Control vs T2D

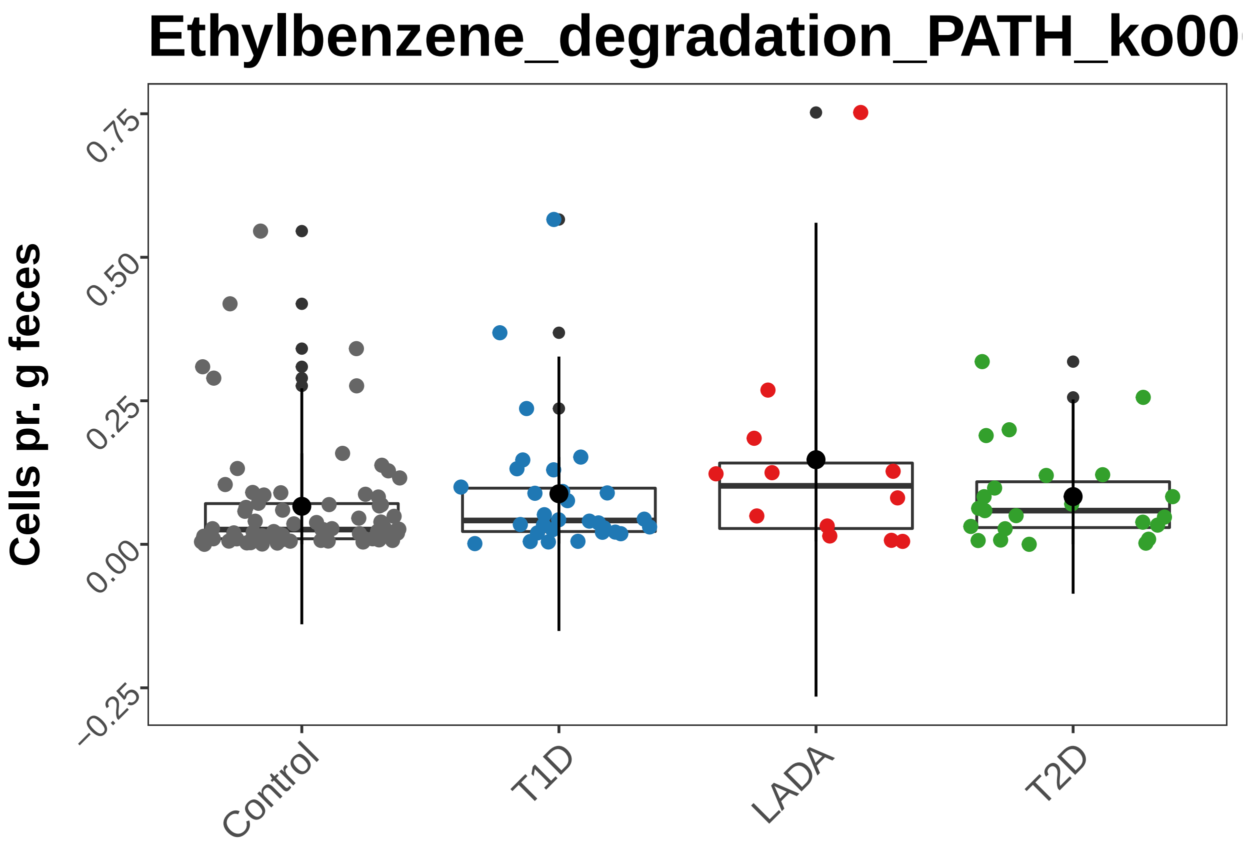

	pvalue	padj	Genus	compare
466	0.0010929	0.0333090	Glucosinolate_biosynthesis_PATH_ko00966	LADA vs Control
270	0.0163201	0.0629443	Glucosinolate_biosynthesis_PATH_ko00966	Control vs T2D
858	0.0195618	0.2312928	Glucosinolate_biosynthesis_PATH_ko00966	LADA vs T1D
74	0.3071963	0.5208704	Glucosinolate_biosynthesis_PATH_ko00966	Control vs T1D
662	0.2009961	0.6495082	Glucosinolate_biosynthesis_PATH_ko00966	T1D vs T2D
1054	0.2134509	0.8415375	Glucosinolate_biosynthesis_PATH_ko00966	LADA vs T2D

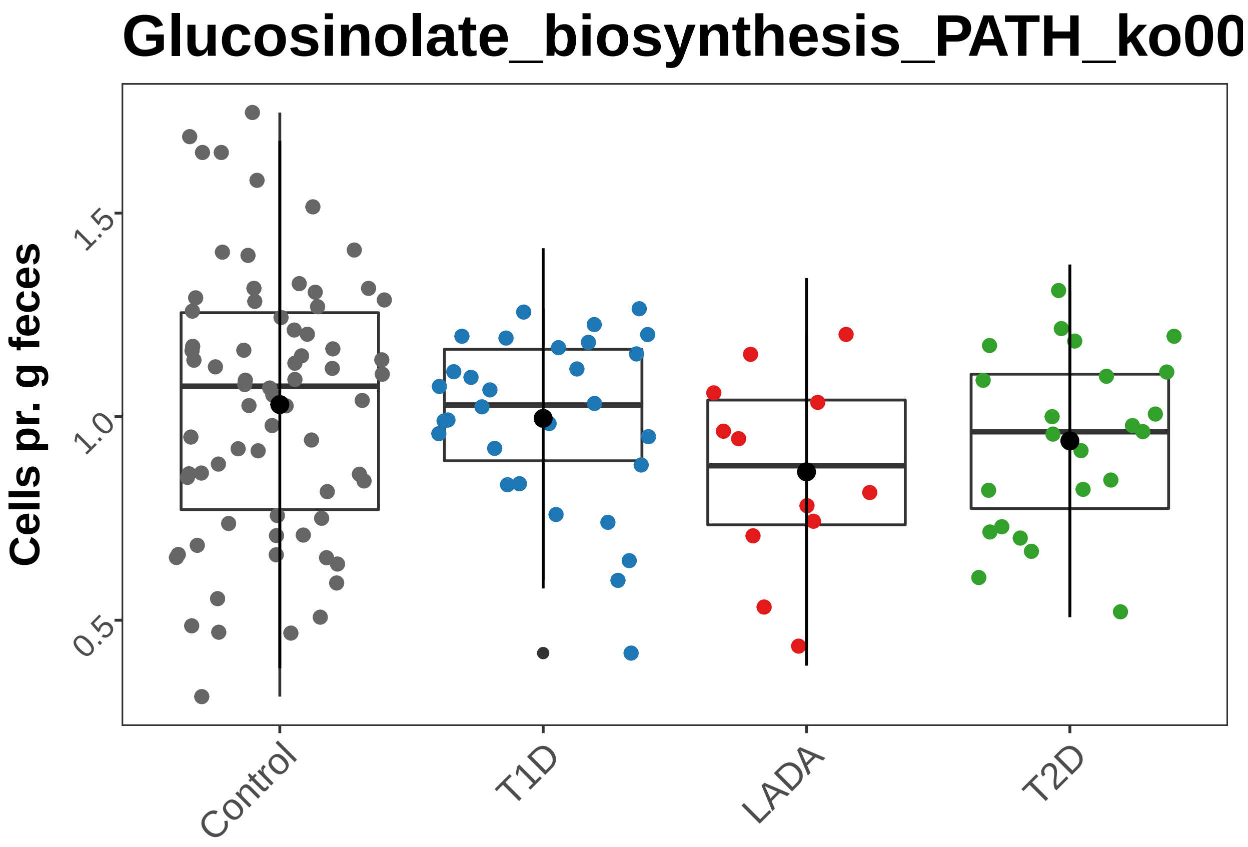

	pvalue	padj	Genus	compare
459	0.0014467	0.0333090	Valine___leucine_and_isoleucine_biosynthesis_PATH_ko00290	LADA vs Control
263	0.0115943	0.0629443	Valine___leucine_and_isoleucine_biosynthesis_PATH_ko00290	Control vs T2D
851	0.0433602	0.2312928	Valine___leucine_and_isoleucine_biosynthesis_PATH_ko00290	LADA vs T1D
67	0.1620155	0.4612224	Valine___leucine_and_isoleucine_biosynthesis_PATH_ko00290	Control vs T1D
655	0.2765856	0.6495082	Valine___leucine_and_isoleucine_biosynthesis_PATH_ko00290	T1D vs T2D
1047	0.2752953	0.8415375	Valine___leucine_and_isoleucine_biosynthesis_PATH_ko00290	LADA vs T2D

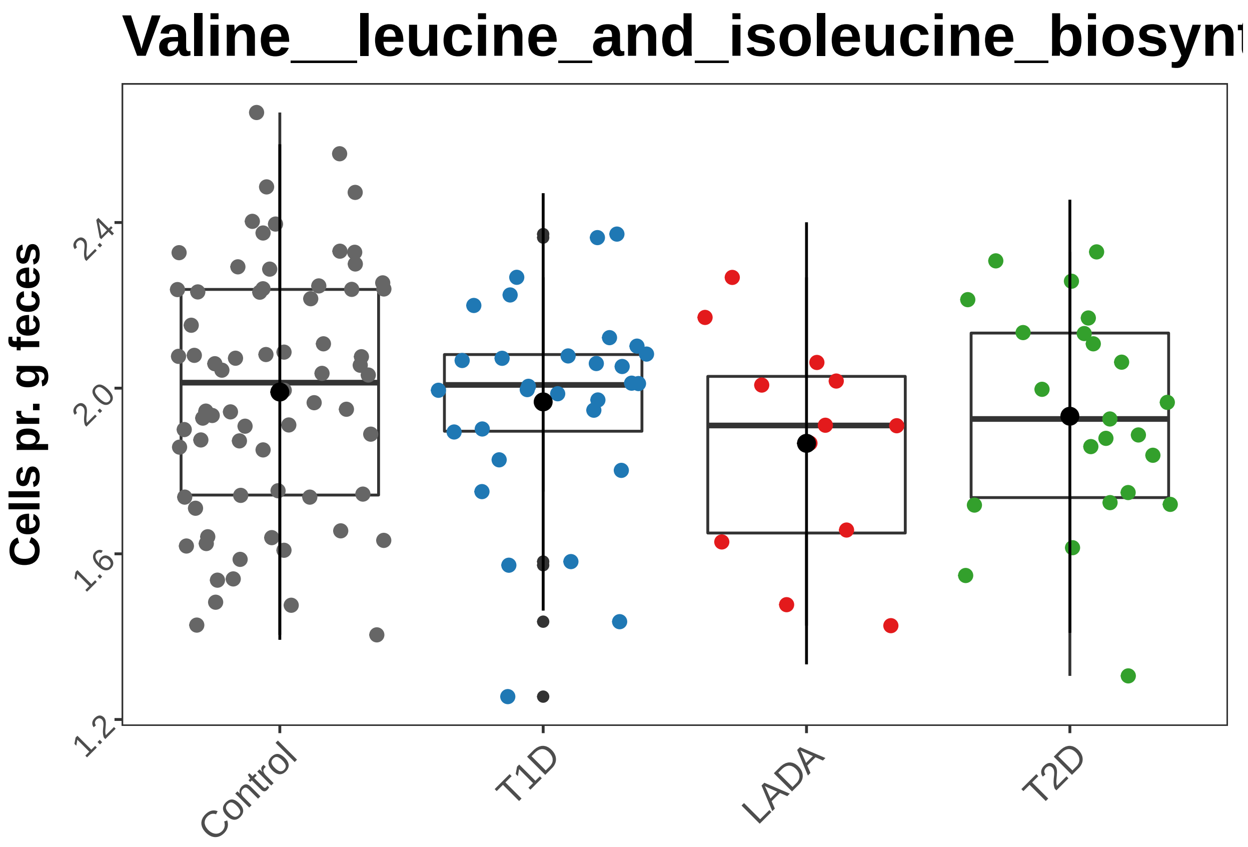

	pvalue	padj	Genus	compare
579	0.0015375	0.0333090	Two__Oxocarboxylic_acid__metabolism	LADA vs Control
383	0.0148318	0.0629443	Two__Oxocarboxylic_acid__metabolism	Control vs T2D
971	0.0380121	0.2312928	Two__Oxocarboxylic_acid__metabolism	LADA vs T1D
187	0.1978450	0.4786856	Two__Oxocarboxylic_acid__metabolism	Control vs T1D
775	0.2717792	0.6495082	Two__Oxocarboxylic_acid__metabolism	T1D vs T2D
1167	0.2565010	0.8415375	Two__Oxocarboxylic_acid__metabolism	LADA vs T2D

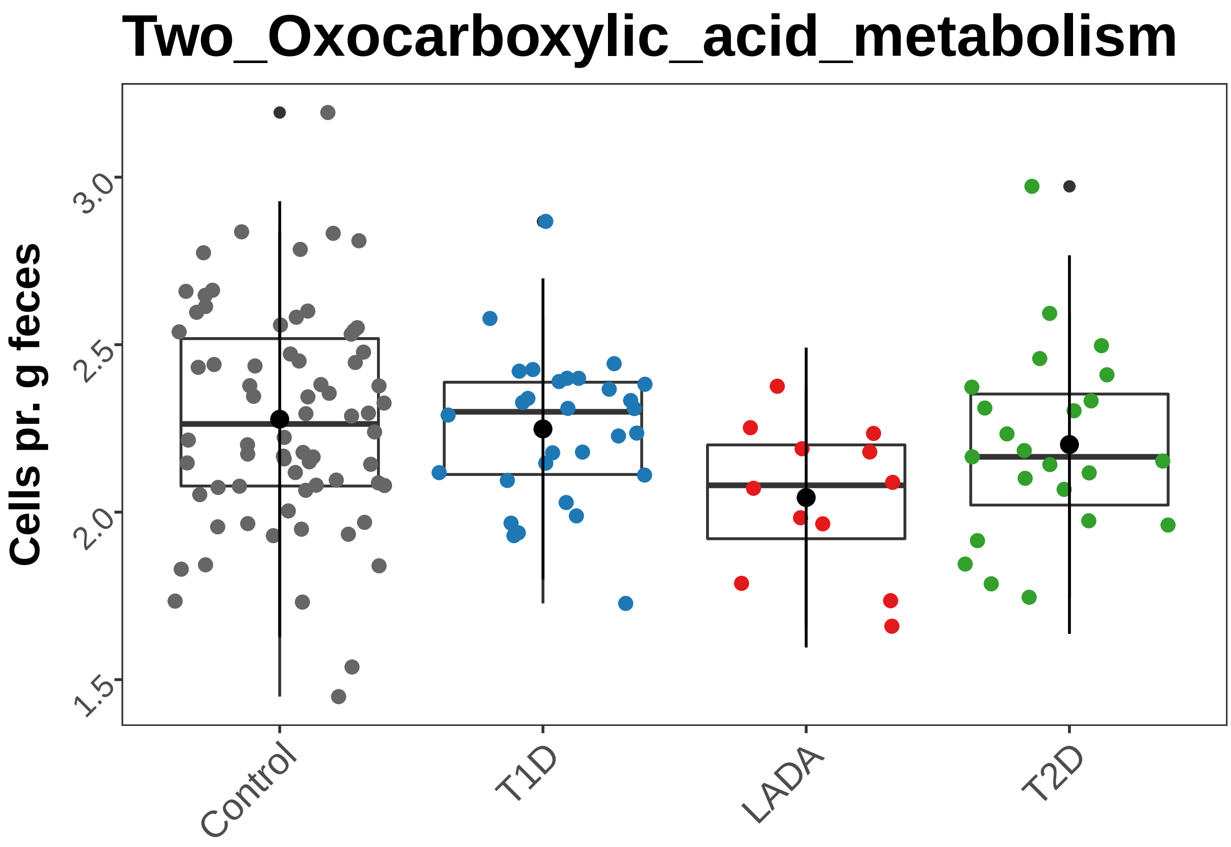

	pvalue	padj	Genus	compare
472	0.0015836	0.0333090	Phenazine_biosynthesis_PATH_ko00405	LADA vs Control
276	0.0104857	0.0629443	Phenazine_biosynthesis_PATH_ko00405	Control vs T2D
864	0.0316984	0.2312928	Phenazine_biosynthesis_PATH_ko00405	LADA vs T1D
80	0.2458719	0.4839499	Phenazine_biosynthesis_PATH_ko00405	Control vs T1D
668	0.1916229	0.6495082	Phenazine_biosynthesis_PATH_ko00405	T1D vs T2D
1060	0.2963872	0.8415375	Phenazine_biosynthesis_PATH_ko00405	LADA vs T2D

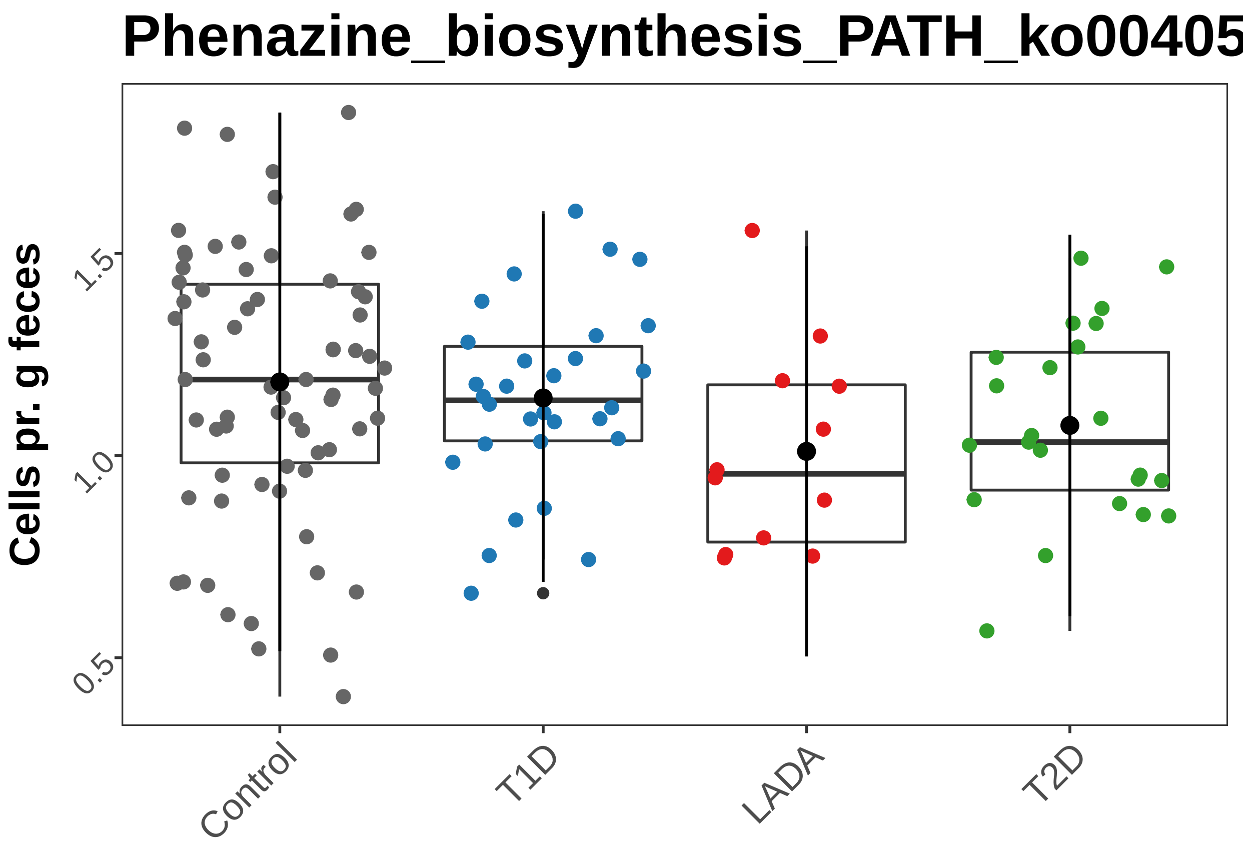

	pvalue	padj	Genus	compare
515	0.0016076	0.0333090	Ether__lipid__metabolism__PATH__ko00565	LADA vs Control
907	0.0043533	0.1066569	Ether__lipid__metabolism__PATH__ko00565	LADA vs T1D
711	0.5186839	0.6862683	Ether__lipid__metabolism__PATH__ko00565	T1D vs T2D
1103	0.0255849	0.7163761	Ether__lipid__metabolism__PATH__ko00565	LADA vs T2D
123	0.9582080	0.9731024	Ether__lipid__metabolism__PATH__ko00565	Control vs T1D
319	0.4284327	NA	Ether__lipid__metabolism__PATH__ko00565	Control vs T2D

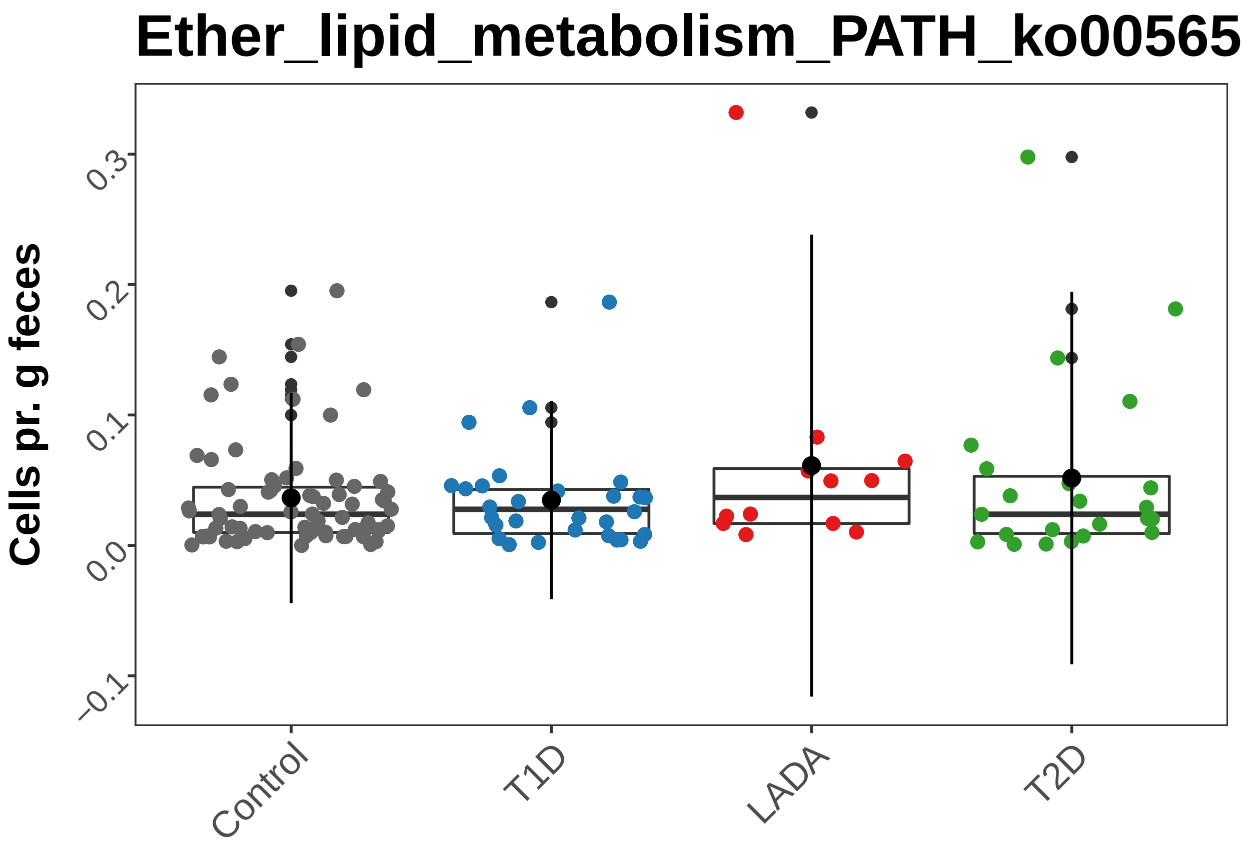

	pvalue	padj	Genus	compare
536	0.0016305	0.0333090	Thiamine_metabolism_PATH_ko00730	LADA vs Control
340	0.0096686	0.0629443	Thiamine_metabolism_PATH_ko00730	Control vs T2D
928	0.0642766	0.2312928	Thiamine_metabolism_PATH_ko00730	LADA vs T1D
144	0.1064037	0.4612224	Thiamine_metabolism_PATH_ko00730	Control vs T1D
732	0.3307697	0.6495082	Thiamine_metabolism_PATH_ko00730	T1D vs T2D
1124	0.3087824	0.8415375	Thiamine_metabolism_PATH_ko00730	LADA vs T2D

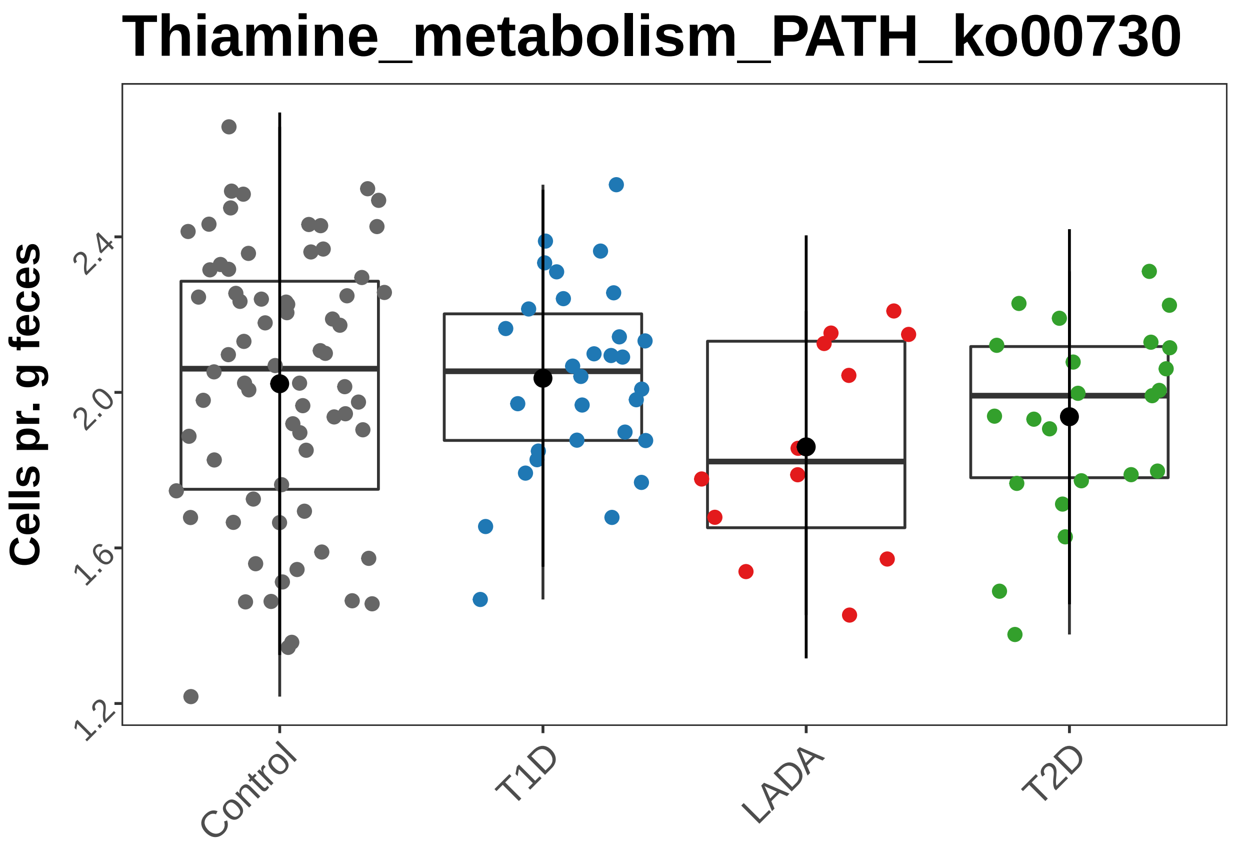

	pvalue	padj	Genus	compare
456	0.0018916	0.0333090	Phenylalanine___tyrosine_and_tryptophan_biosynthesis_PATH_ko00400	LADA vs Control
260	0.0126419	0.0629443	Phenylalanine___tyrosine_and_tryptophan_biosynthesis_PATH_ko00400	Control vs T2D
848	0.0387265	0.2312928	Phenylalanine___tyrosine_and_tryptophan_biosynthesis_PATH_ko00400	LADA vs T1D
64	0.2252529	0.4786856	Phenylalanine___tyrosine_and_tryptophan_biosynthesis_PATH_ko00400	Control vs T1D
652	0.2271650	0.6495082	Phenylalanine___tyrosine_and_tryptophan_biosynthesis_PATH_ko00400	T1D vs T2D
1044	0.2970618	0.8415375	Phenylalanine___tyrosine_and_tryptophan_biosynthesis_PATH_ko00400	LADA vs T2D

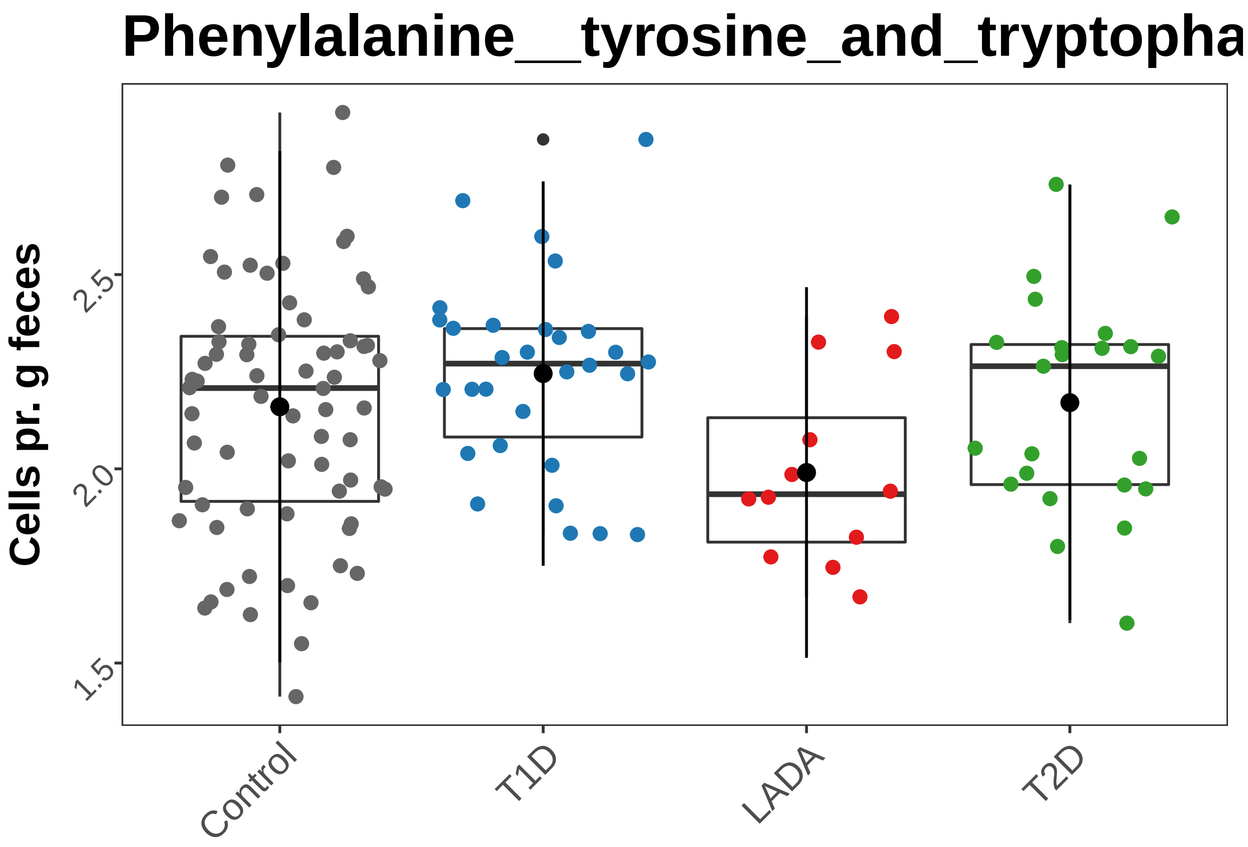

	pvalue	padj	Genus	compare
532	0.0020387	0.0333090	Pantothenate_and_CoA_biosynthesis_PATH_ko00770	LADA vs Control
336	0.0086860	0.0629443	Pantothenate_and_CoA_biosynthesis_PATH_ko00770	Control vs T2D
924	0.0537796	0.2312928	Pantothenate_and_CoA_biosynthesis_PATH_ko00770	LADA vs T1D
140	0.1622246	0.4612224	Pantothenate_and_CoA_biosynthesis_PATH_ko00770	Control vs T1D
728	0.2399097	0.6495082	Pantothenate_and_CoA_biosynthesis_PATH_ko00770	T1D vs T2D
1120	0.3496774	0.8415375	Pantothenate_and_CoA_biosynthesis_PATH_ko00770	LADA vs T2D

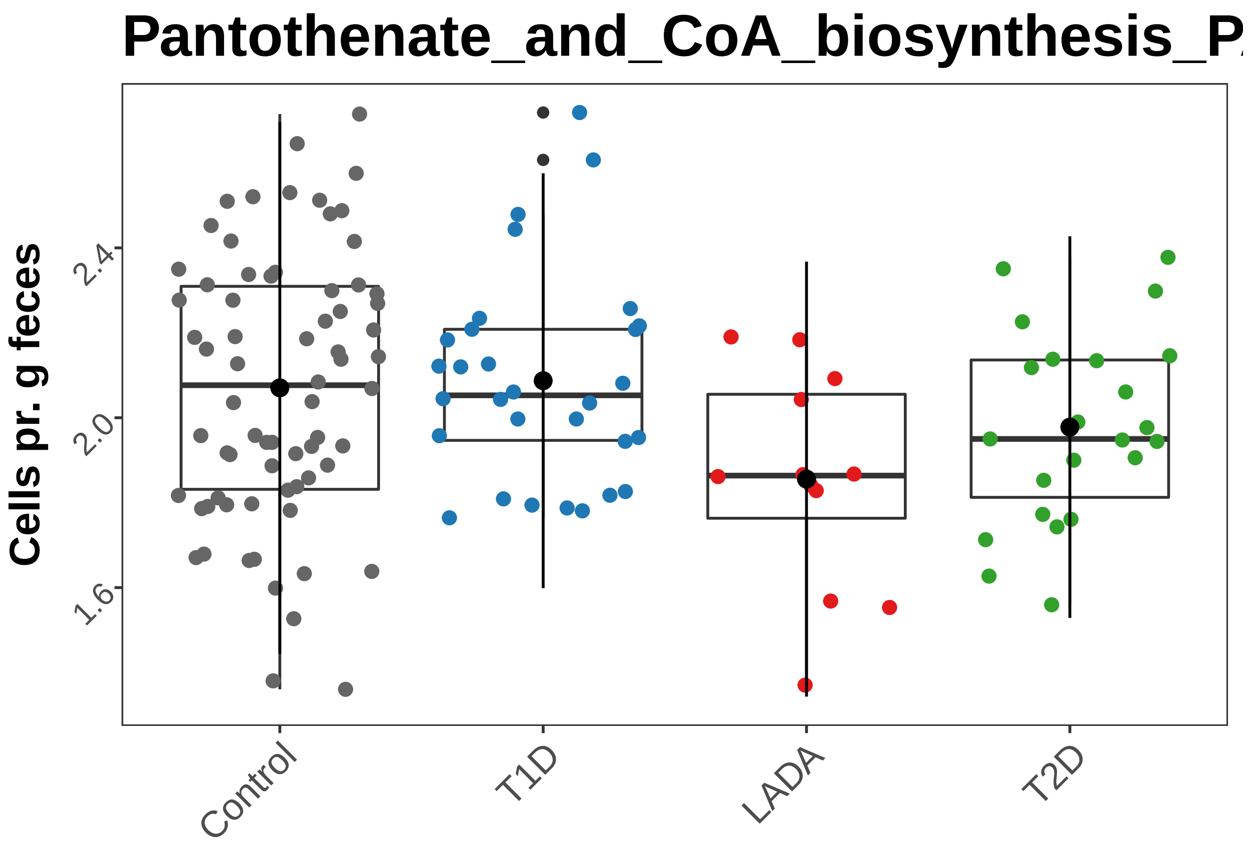

	pvalue	padj	Genus	compare
580	0.0020393	0.0333090	Biosynthesis_of_amino_acids	LADA vs Control
384	0.0127064	0.0629443	Biosynthesis_of_amino_acids	Control vs T2D
972	0.0546752	0.2312928	Biosynthesis_of_amino_acids	LADA vs T1D
188	0.1589208	0.4612224	Biosynthesis_of_amino_acids	Control vs T1D
776	0.2929017	0.6495082	Biosynthesis_of_amino_acids	T1D vs T2D
1168	0.3056394	0.8415375	Biosynthesis_of_amino_acids	LADA vs T2D

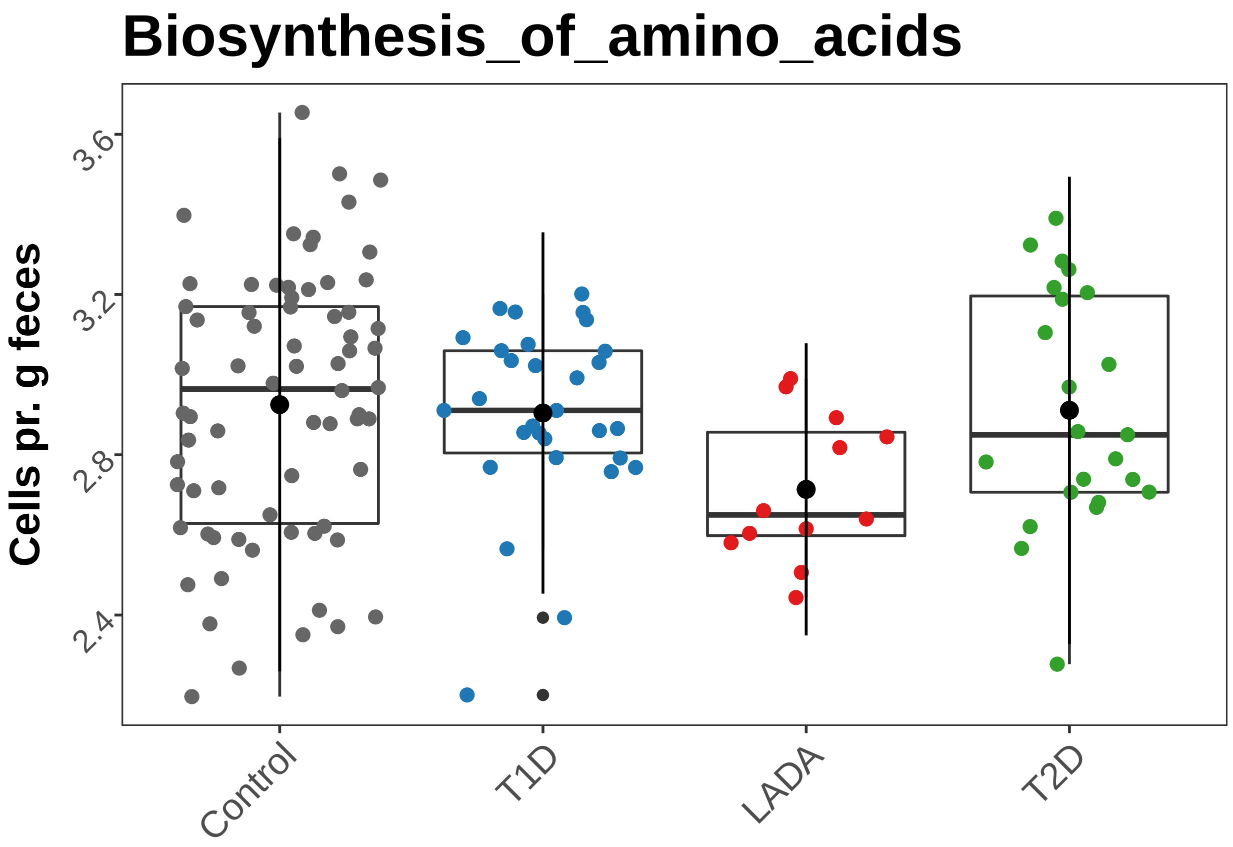

	pvalue	padj	Genus	compare
480	0.0023361	0.0346055	C5_Branched_dibasic_acid_metabolism_PATH_ko00660	LADA vs Control
284	0.0204913	0.0661577	C5_Branched_dibasic_acid_metabolism_PATH_ko00660	Control vs T2D
872	0.0328969	0.2312928	C5_Branched_dibasic_acid_metabolism_PATH_ko00660	LADA vs T1D
88	0.3082702	0.5208704	C5_Branched_dibasic_acid_metabolism_PATH_ko00660	Control vs T1D
676	0.2273298	0.6495082	C5_Branched_dibasic_acid_metabolism_PATH_ko00660	T1D vs T2D
1068	0.2684490	0.8415375	C5_Branched_dibasic_acid_metabolism_PATH_ko00660	LADA vs T2D

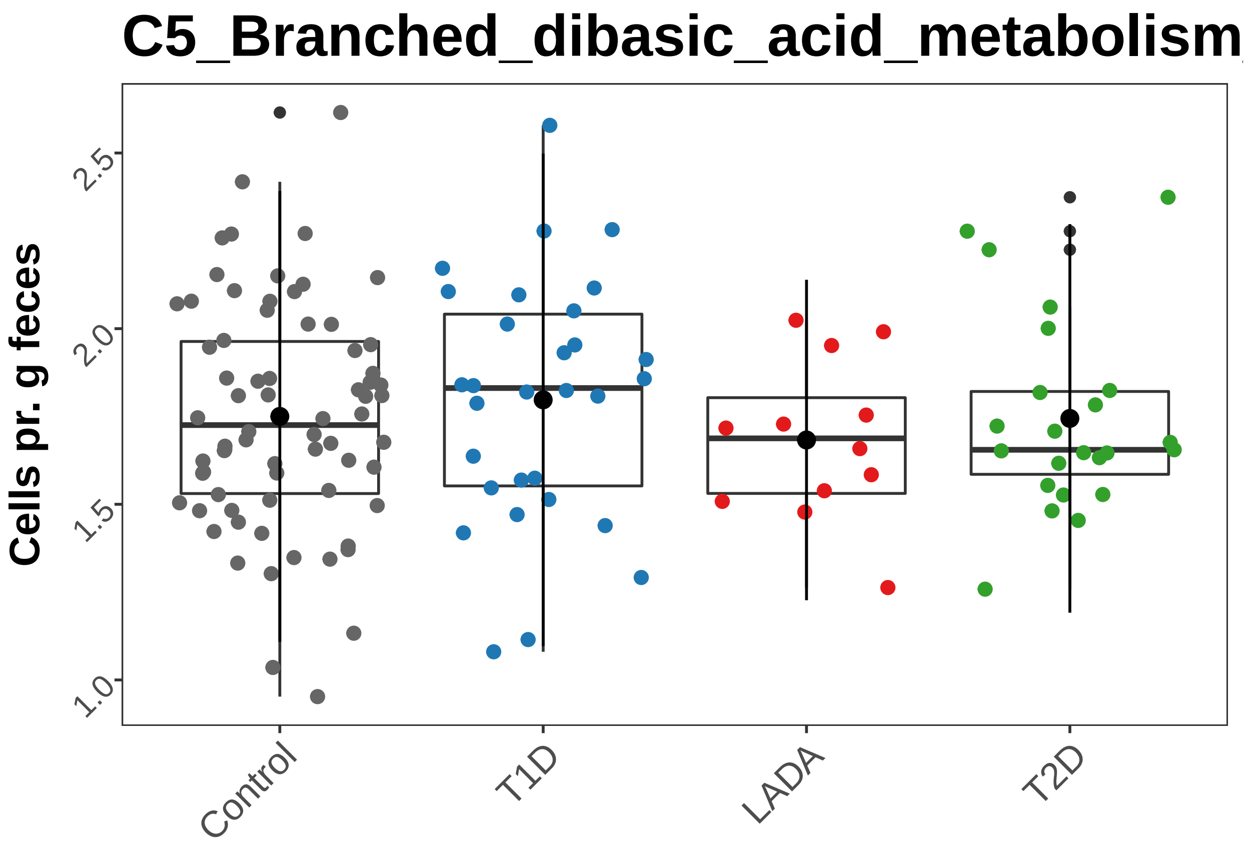

	pvalue	padj	Genus	compare
497	0.0026162	0.0346055	Photosynthesis_PATH_ko00195	LADA vs Control
301	0.0056442	0.0629443	Photosynthesis_PATH_ko00195	Control vs T2D
105	0.0143556	0.2904843	Photosynthesis_PATH_ko00195	Control vs T1D
889	0.2345111	0.3940555	Photosynthesis_PATH_ko00195	LADA vs T1D
693	0.6365576	0.7539875	Photosynthesis_PATH_ko00195	T1D vs T2D
1085	0.4398030	0.8415375	Photosynthesis_PATH_ko00195	LADA vs T2D

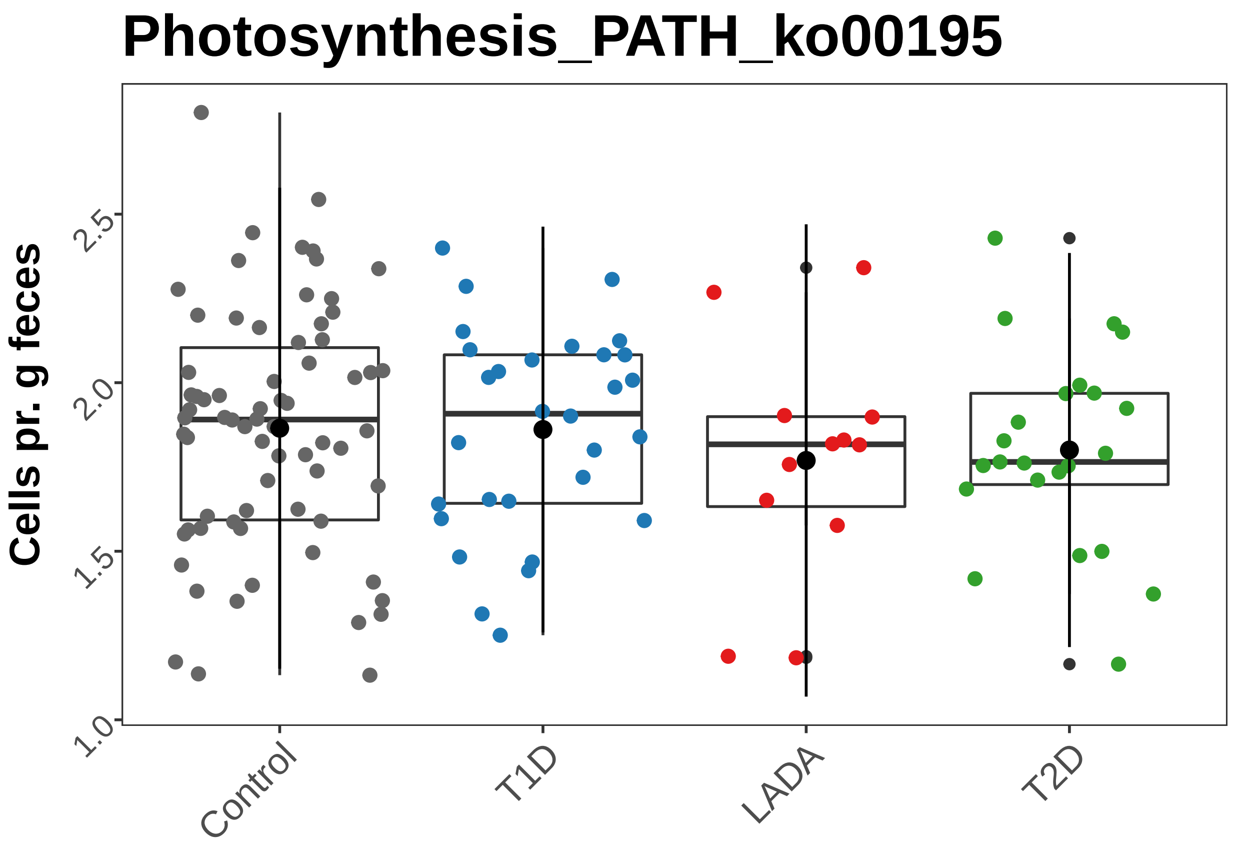

	pvalue	padj	Genus	compare
449	0.0030970	0.0346055	Arginine_biosynthesis_PATH_ko00220	LADA vs Control
253	0.0164656	0.0629443	Arginine_biosynthesis_PATH_ko00220	Control vs T2D
841	0.0783535	0.2321553	Arginine_biosynthesis_PATH_ko00220	LADA vs T1D
57	0.1389304	0.4612224	Arginine_biosynthesis_PATH_ko00220	Control vs T1D
645	0.3603161	0.6495082	Arginine_biosynthesis_PATH_ko00220	T1D vs T2D
1037	0.3288157	0.8415375	Arginine_biosynthesis_PATH_ko00220	LADA vs T2D

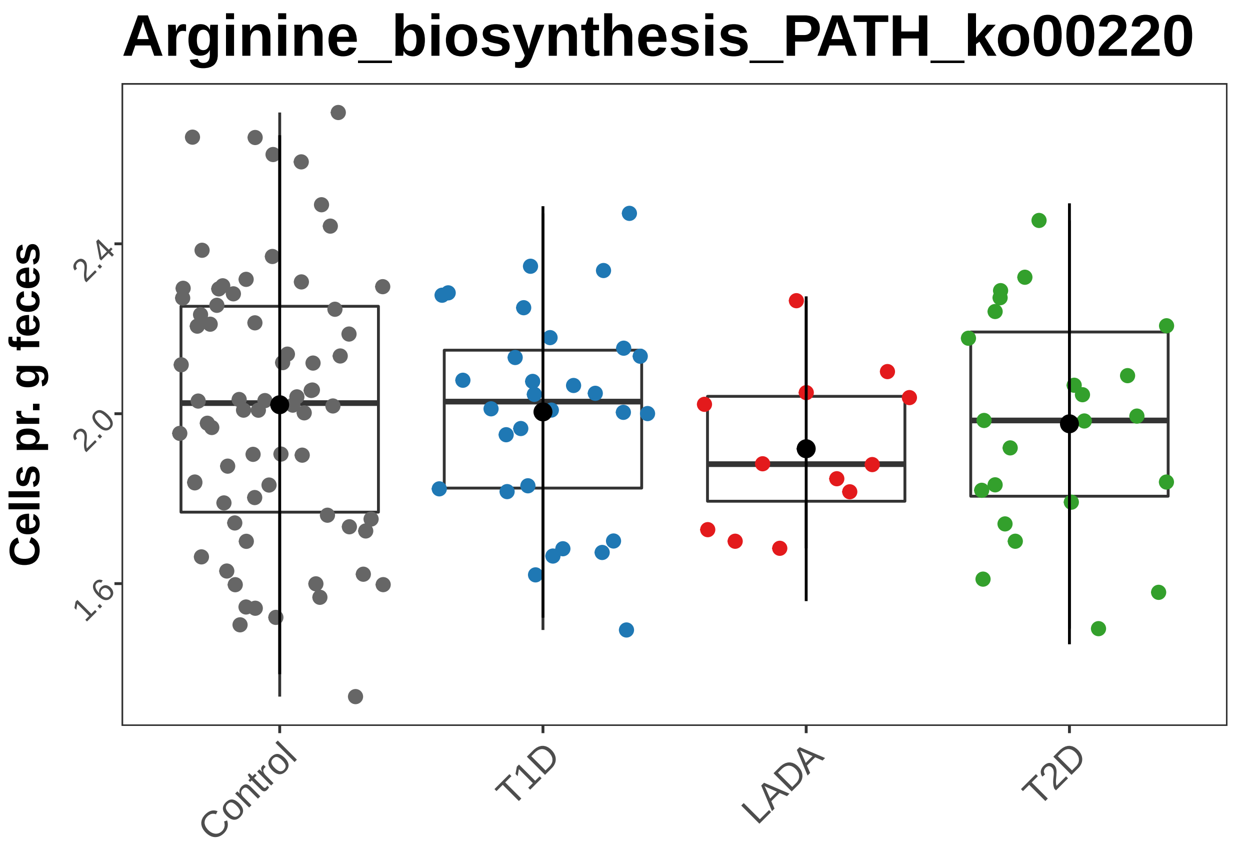

	pvalue	padj	Genus	compare
518	0.0034891	0.0346055	Glycerolipid__metabolism__PATH__ko00561	LADA vs Control
322	0.0040520	0.0629443	Glycerolipid__metabolism__PATH__ko00561	Control vs T2D
910	0.1367180	0.2954567	Glycerolipid__metabolism__PATH__ko00561	LADA vs T1D
126	0.0638894	0.3992571	Glycerolipid__metabolism__PATH__ko00561	Control vs T1D
714	0.3014038	0.6495082	Glycerolipid__metabolism__PATH__ko00561	T1D vs T2D
1106	0.5332410	0.8428648	Glycerolipid__metabolism__PATH__ko00561	LADA vs T2D

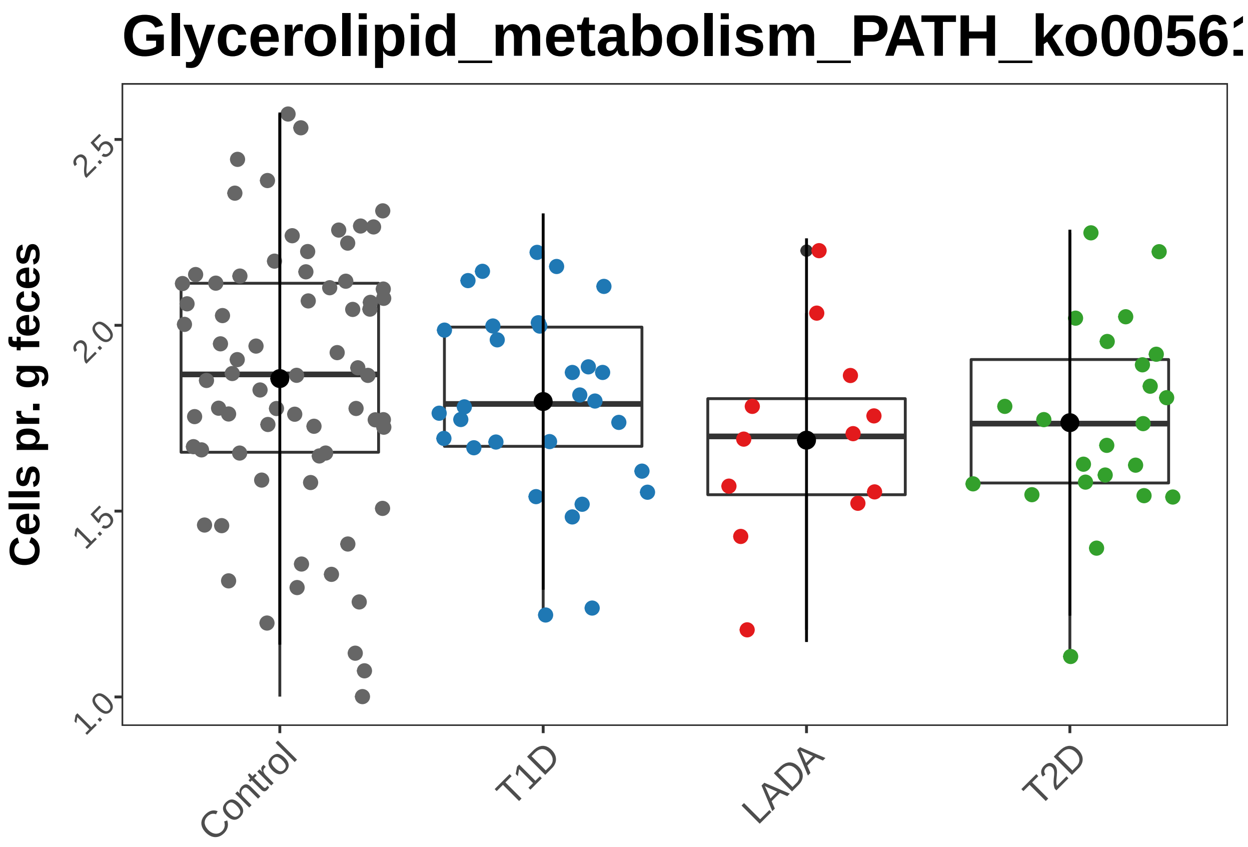

	pvalue	padj	Genus	compare
488	0.0036136	0.0346055	Pentose_phosphate_pathway_PATH_ko00030	LADA vs Control
292	0.0155954	0.0629443	Pentose_phosphate_pathway_PATH_ko00030	Control vs T2D
880	0.1003267	0.2458003	Pentose_phosphate_pathway_PATH_ko00030	LADA vs T1D
96	0.1108369	0.4612224	Pentose_phosphate_pathway_PATH_ko00030	Control vs T1D
684	0.3999706	0.6495082	Pentose_phosphate_pathway_PATH_ko00030	T1D vs T2D
1076	0.3569896	0.8415375	Pentose_phosphate_pathway_PATH_ko00030	LADA vs T2D

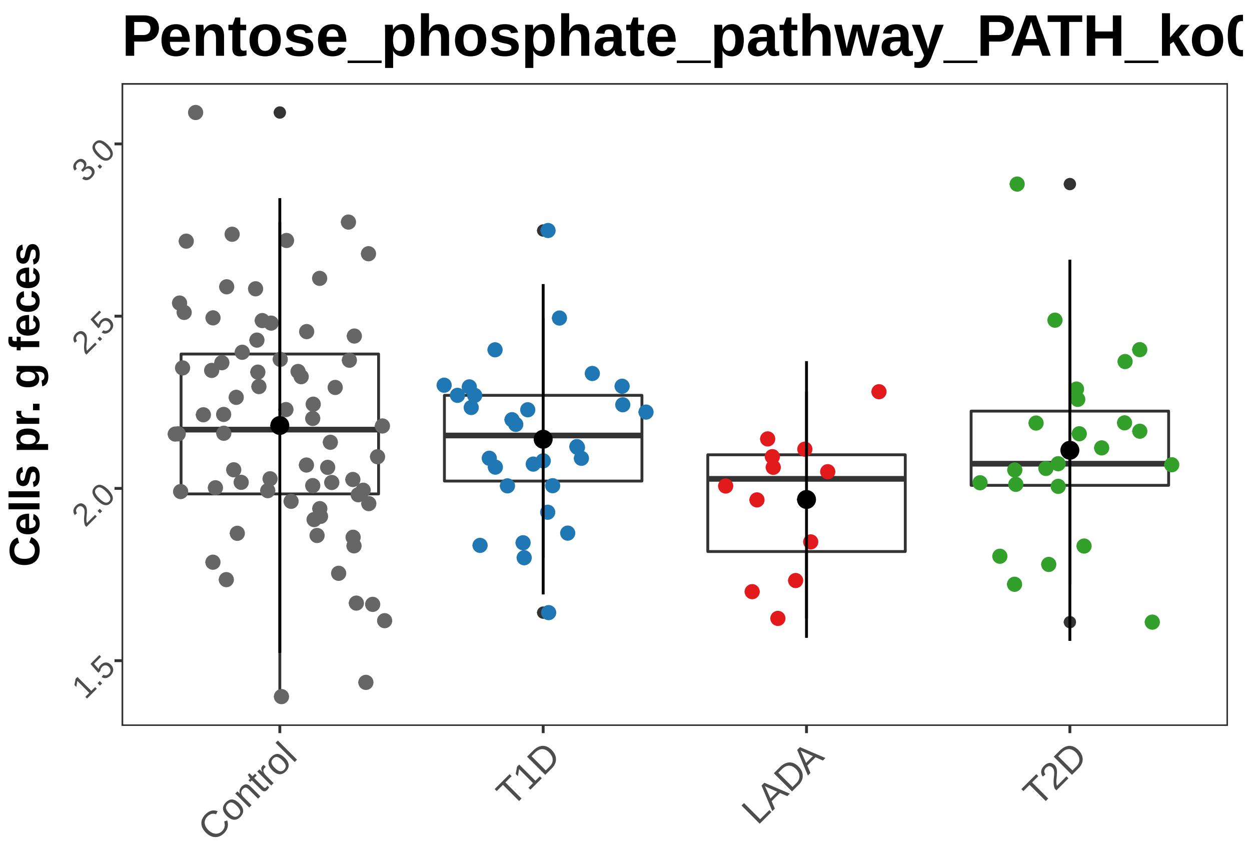

	pvalue	padj	Genus	compare
442	0.0036957	0.0346055	Aminoacyl__tRNA__biosynthesis__PATH__ko00970	LADA vs Control
246	0.0270860	0.0676342	Aminoacyl__tRNA__biosynthesis__PATH__ko00970	Control vs T2D
834	0.0601947	0.2312928	Aminoacyl__tRNA__biosynthesis__PATH__ko00970	LADA vs T1D
50	0.2244304	0.4786856	Aminoacyl__tRNA__biosynthesis__PATH__ko00970	Control vs T1D
638	0.3369466	0.6495082	Aminoacyl__tRNA__biosynthesis__PATH__ko00970	T1D vs T2D
1030	0.2912837	0.8415375	Aminoacyl__tRNA__biosynthesis__PATH__ko00970	LADA vs T2D

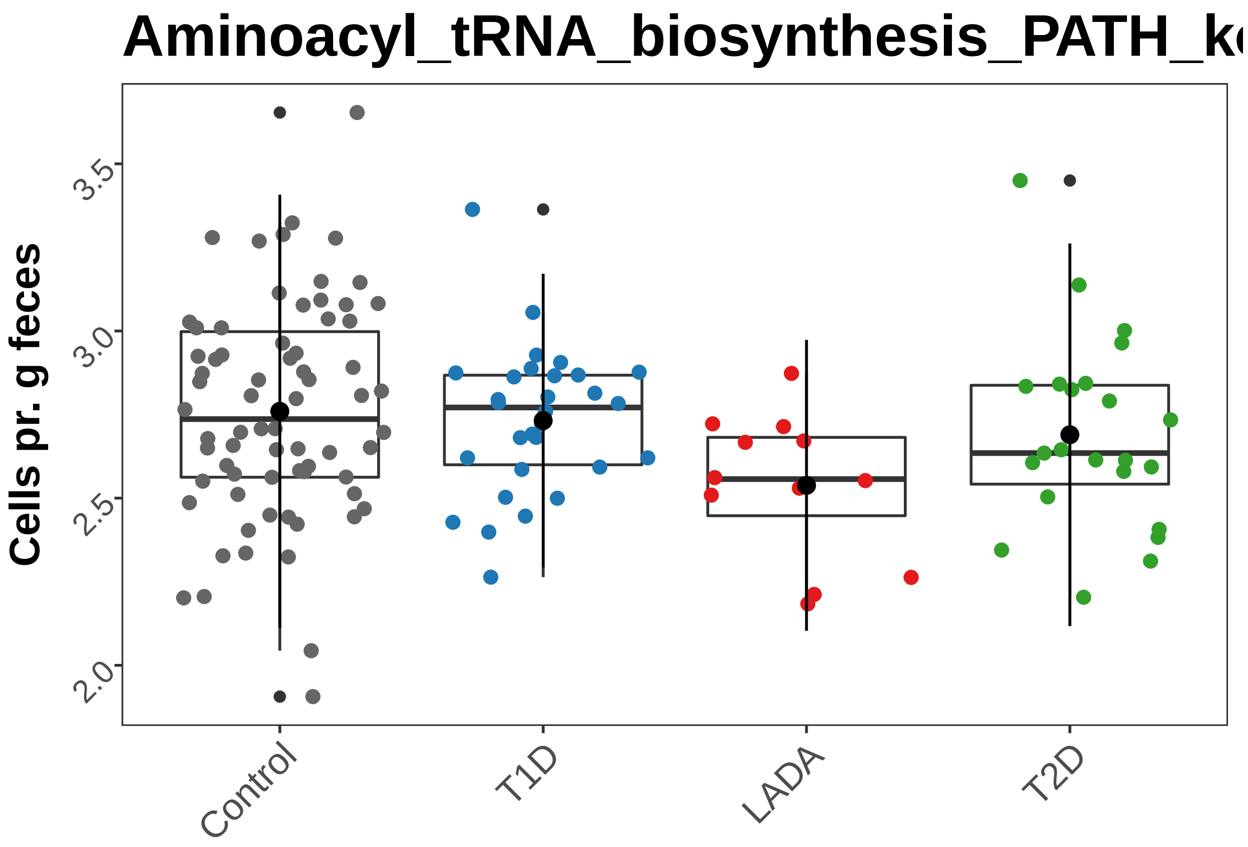

	pvalue	padj	Genus	compare
453	0.0037616	0.0346055	Lysine_biosynthesis_PATH_ko00300	LADA vs Control
257	0.0168290	0.0629443	Lysine_biosynthesis_PATH_ko00300	Control vs T2D
845	0.0639477	0.2312928	Lysine_biosynthesis_PATH_ko00300	LADA vs T1D
61	0.2117082	0.4786856	Lysine_biosynthesis_PATH_ko00300	Control vs T1D
649	0.2758781	0.6495082	Lysine_biosynthesis_PATH_ko00300	T1D vs T2D
1041	0.3529573	0.8415375	Lysine_biosynthesis_PATH_ko00300	LADA vs T2D

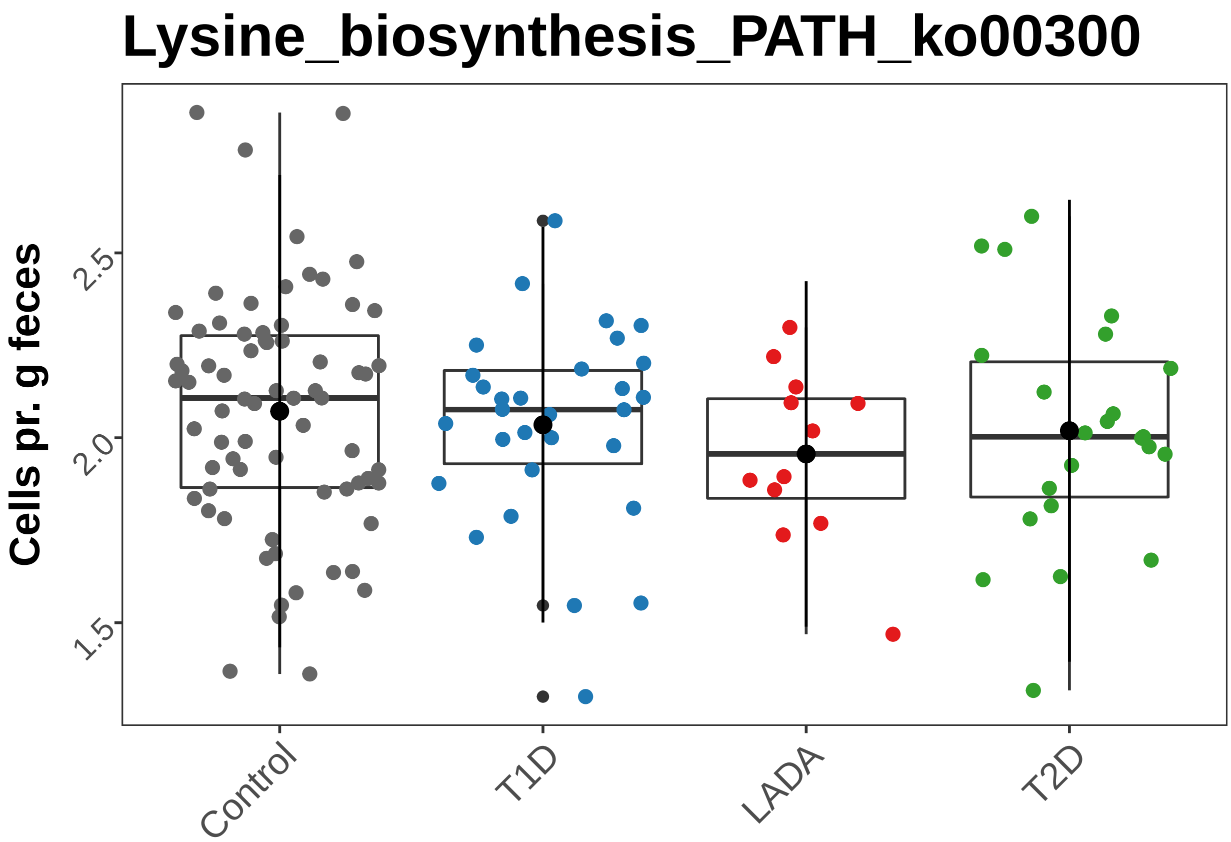

	pvalue	padj	Genus	compare
581	0.0042931	0.0346055	Biosynthesis_of_antibiotics	LADA vs Control
385	0.0179296	0.0629443	Biosynthesis_of_antibiotics	Control vs T2D
973	0.0640528	0.2312928	Biosynthesis_of_antibiotics	LADA vs T1D
189	0.2339251	0.4839499	Biosynthesis_of_antibiotics	Control vs T1D
777	0.2646391	0.6495082	Biosynthesis_of_antibiotics	T1D vs T2D
1169	0.3638604	0.8415375	Biosynthesis_of_antibiotics	LADA vs T2D

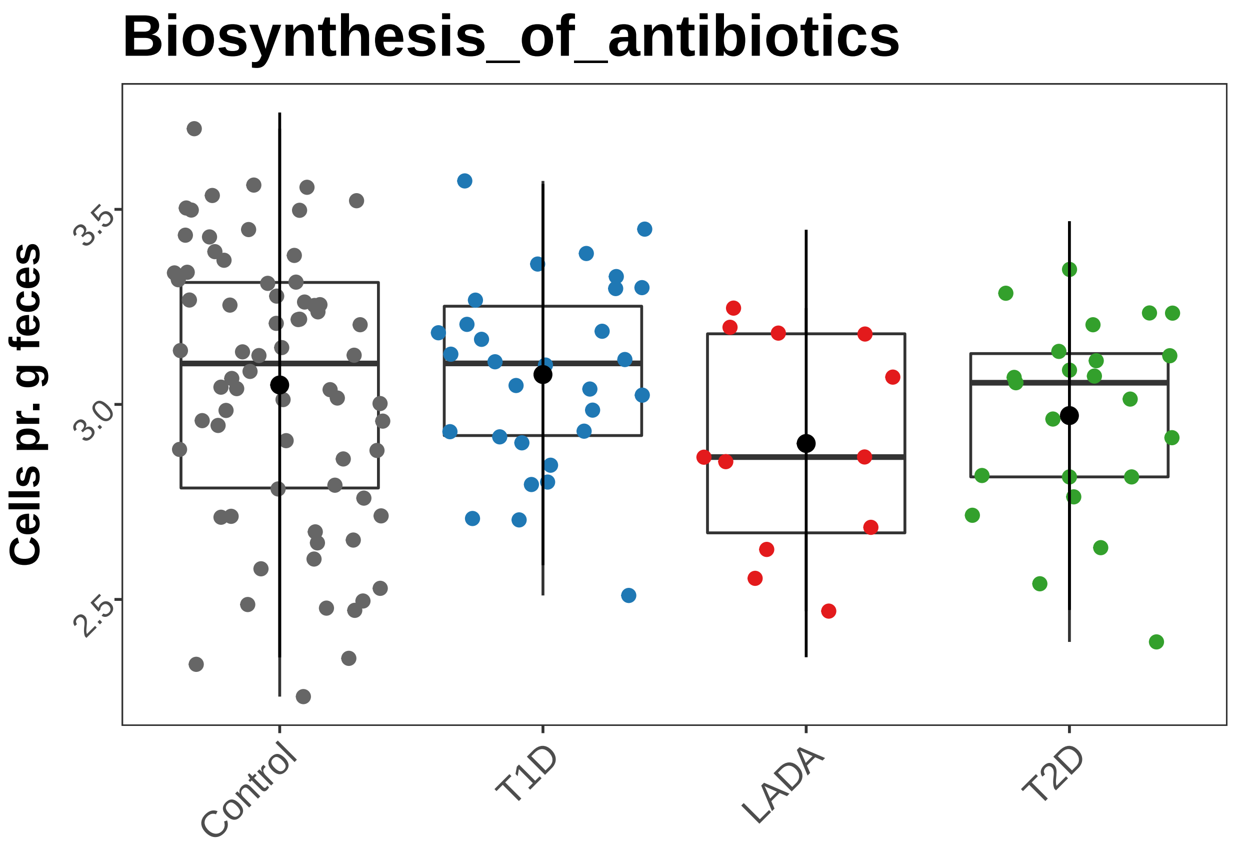

	pvalue	padj	Genus	compare
475	0.0043675	0.0346055	Streptomycin_biosynthesis_PATH_ko00521	LADA vs Control
279	0.0616025	0.0996512	Streptomycin_biosynthesis_PATH_ko00521	Control vs T2D
867	0.0442686	0.2312928	Streptomycin_biosynthesis_PATH_ko00521	LADA vs T1D
83	0.3510858	0.5414362	Streptomycin_biosynthesis_PATH_ko00521	Control vs T1D
671	0.3752407	0.6495082	Streptomycin_biosynthesis_PATH_ko00521	T1D vs T2D
1063	0.2152327	0.8415375	Streptomycin_biosynthesis_PATH_ko00521	LADA vs T2D

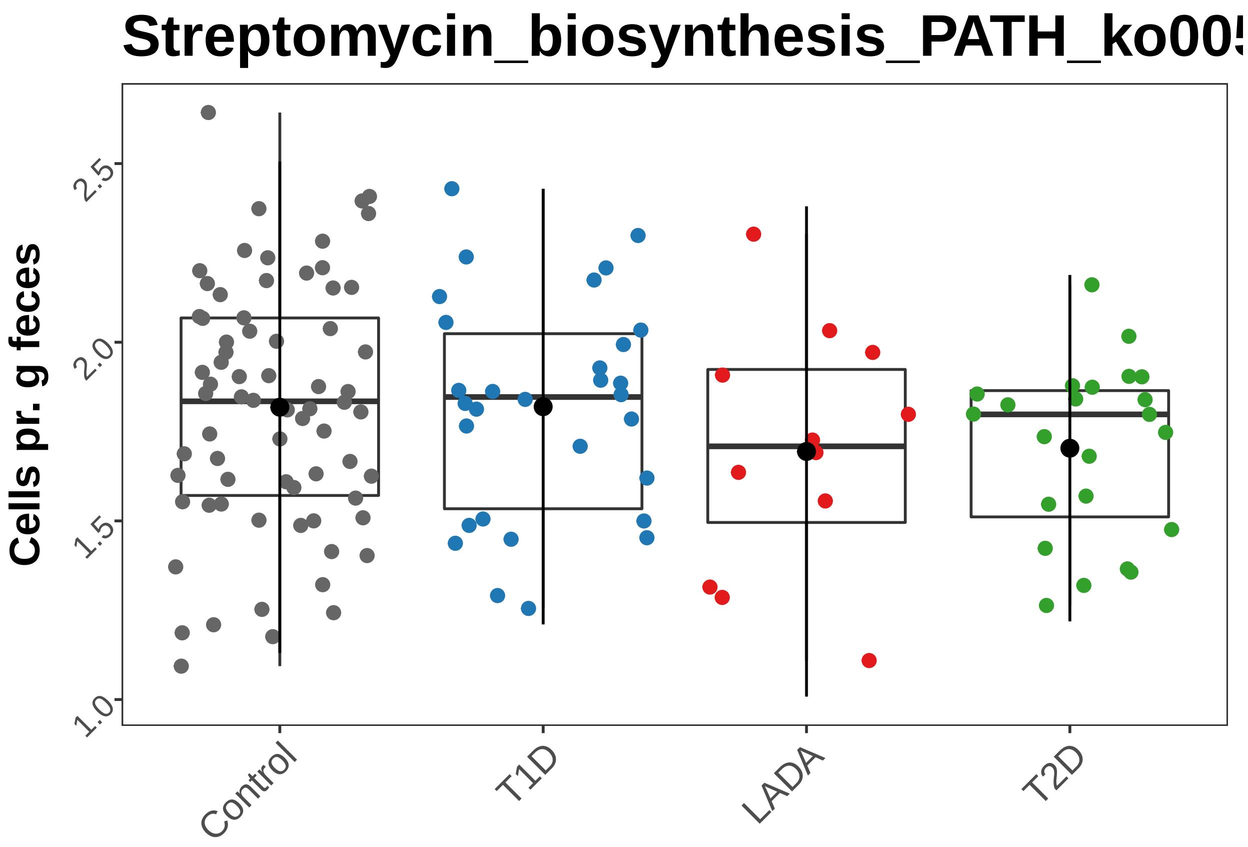

	pvalue	padj	Genus	compare
558	0.0045024	0.0346055	Terpenoid__backbone__biosynthesis__PATH__ko00900	LADA vs Control
362	0.0150679	0.0629443	Terpenoid__backbone__biosynthesis__PATH__ko00900	Control vs T2D
950	0.0797460	0.2321553	Terpenoid__backbone__biosynthesis__PATH__ko00900	LADA vs T1D
166	0.1853051	0.4762288	Terpenoid__backbone__biosynthesis__PATH__ko00900	Control vs T1D
754	0.2868863	0.6495082	Terpenoid__backbone__biosynthesis__PATH__ko00900	T1D vs T2D
1146	0.3943428	0.8415375	Terpenoid__backbone__biosynthesis__PATH__ko00900	LADA vs T2D

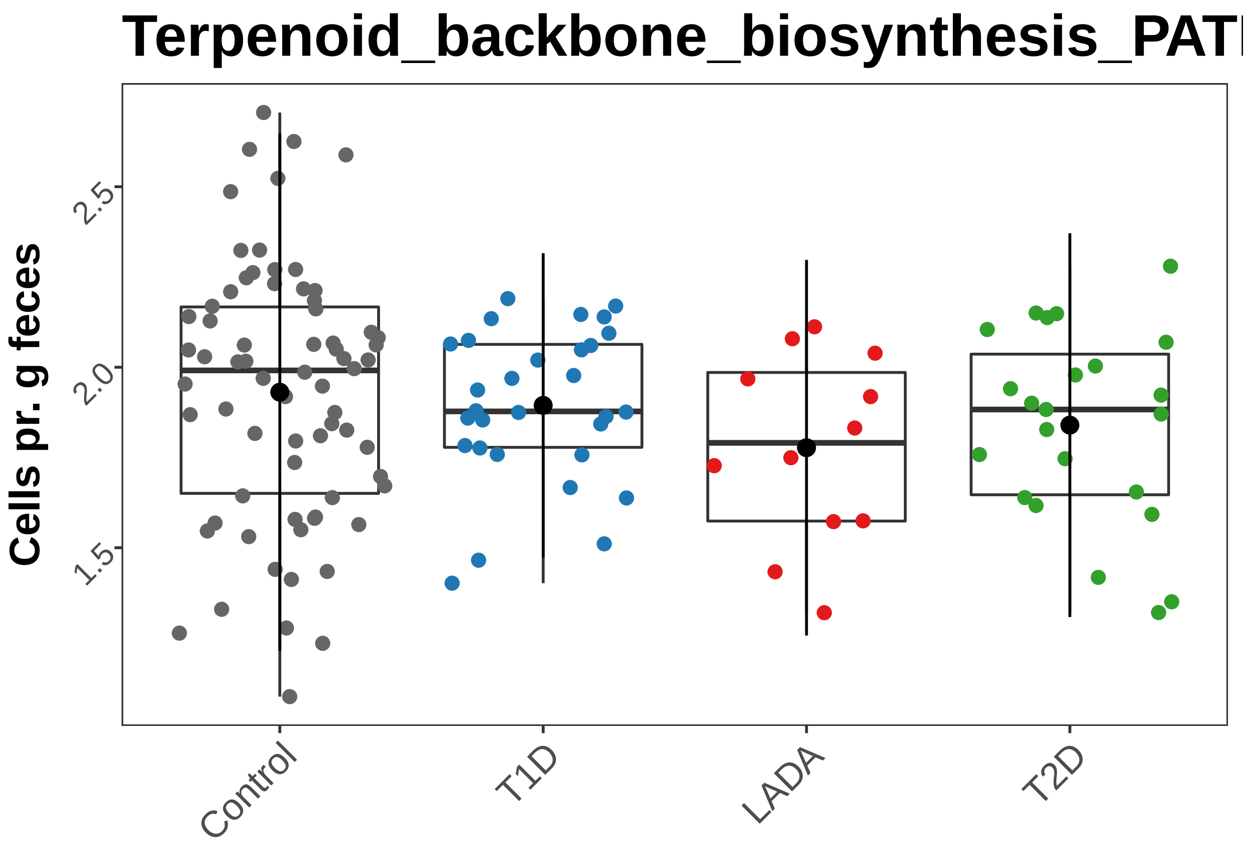

	pvalue	padj	Genus	compare
439	0.0047446	0.0346055	Nucleotide_excision_repair_PATH_ko03420	LADA vs Control
243	0.0307505	0.0681056	Nucleotide_excision_repair_PATH_ko03420	Control vs T2D
831	0.0785437	0.2321553	Nucleotide_excision_repair_PATH_ko03420	LADA vs T1D
47	0.1971887	0.4786856	Nucleotide_excision_repair_PATH_ko03420	Control vs T1D
635	0.3908968	0.6495082	Nucleotide_excision_repair_PATH_ko03420	T1D vs T2D
1027	0.3079132	0.8415375	Nucleotide_excision_repair_PATH_ko03420	LADA vs T2D

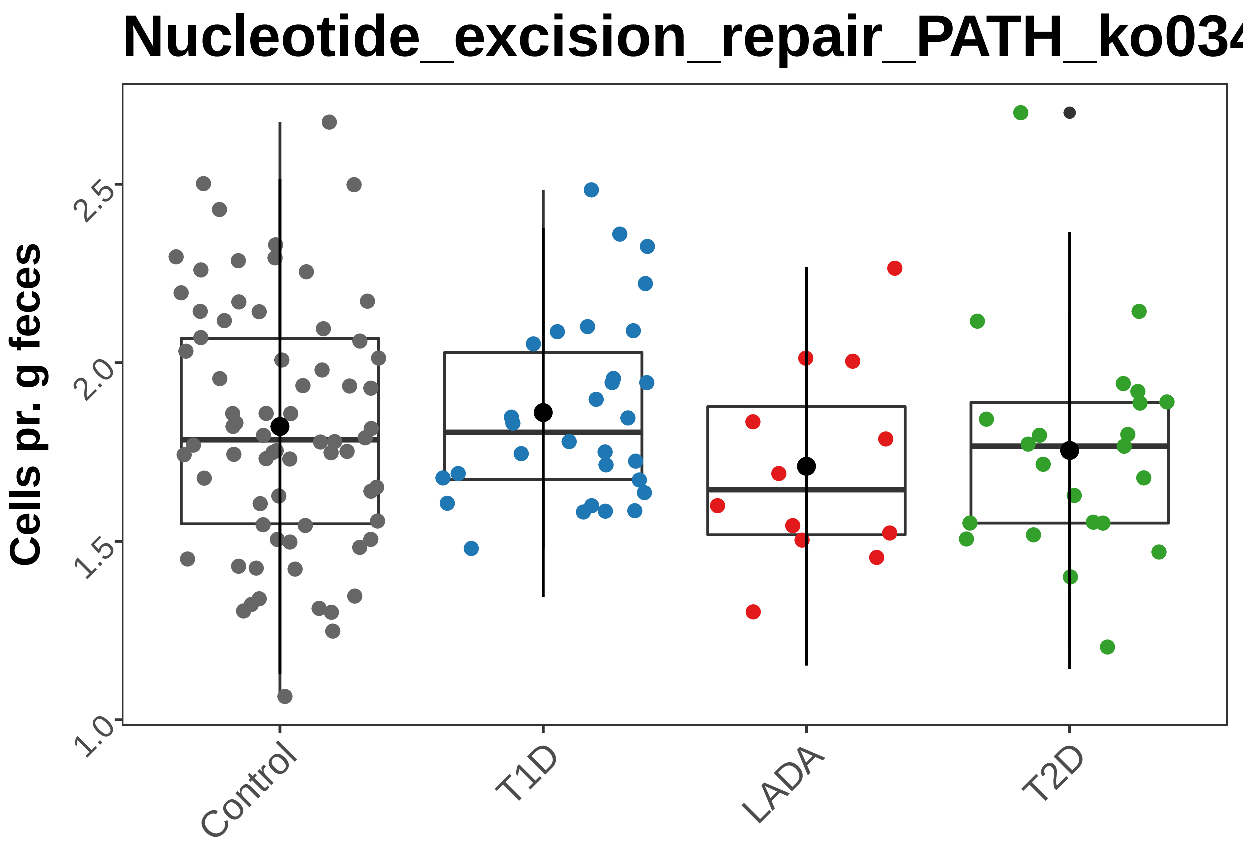

	pvalue	padj	Genus	compare
546	0.0047919	0.0346055	Selenocompound_metabolism_PATH_ko00450	LADA vs Control
350	0.0132910	0.0629443	Selenocompound_metabolism_PATH_ko00450	Control vs T2D
938	0.1533177	0.3130237	Selenocompound_metabolism_PATH_ko00450	LADA vs T1D
154	0.0712959	0.3992571	Selenocompound_metabolism_PATH_ko00450	Control vs T1D
742	0.4672432	0.6743371	Selenocompound_metabolism_PATH_ko00450	T1D vs T2D
1134	0.4215018	0.8415375	Selenocompound_metabolism_PATH_ko00450	LADA vs T2D

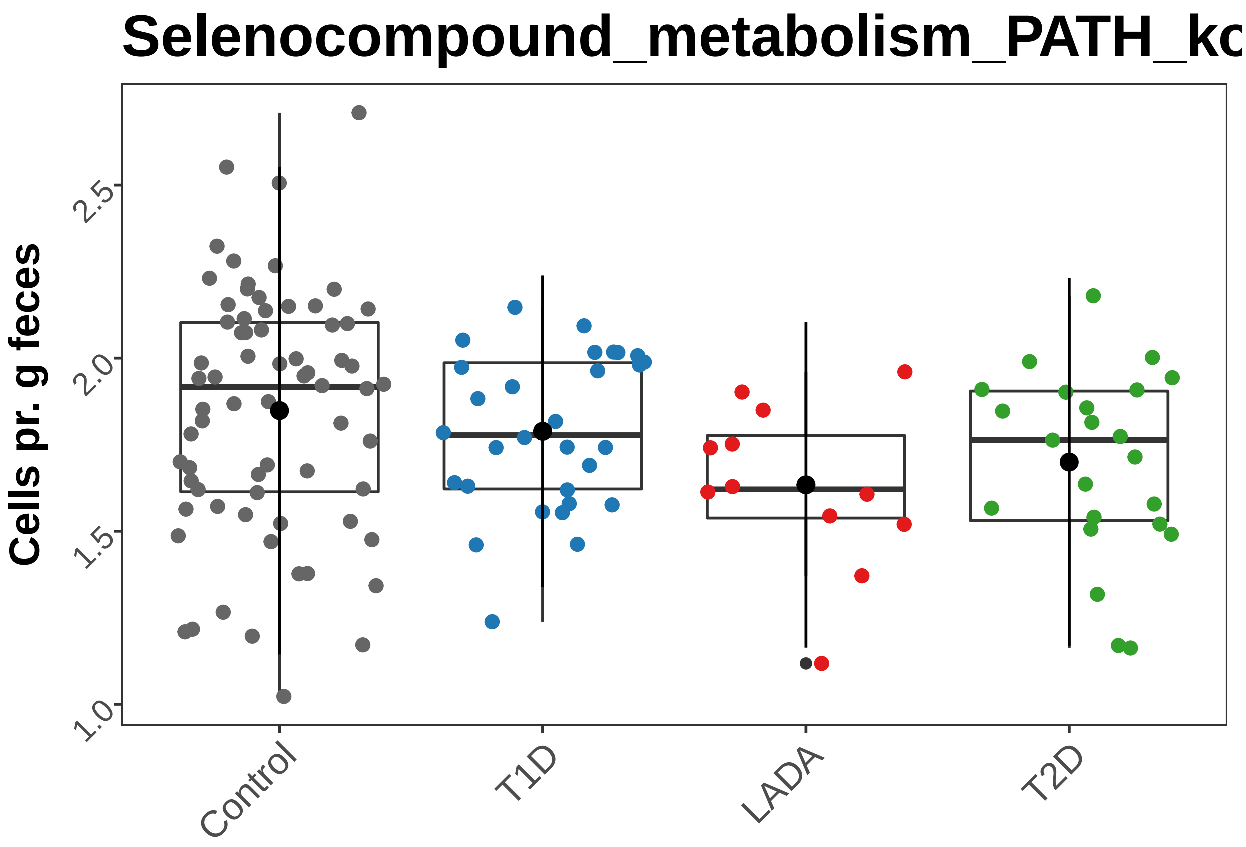

	pvalue	padj	Genus	compare
492	0.0048108	0.0346055	Carbon_fixation_in_photosynthetic_organisms_PATH_ko00710	LADA vs Control
296	0.0102169	0.0629443	Carbon_fixation_in_photosynthetic_organisms_PATH_ko00710	Control vs T2D
884	0.1447090	0.3082930	Carbon_fixation_in_photosynthetic_organisms_PATH_ko00710	LADA vs T1D
100	0.0794411	0.4208232	Carbon_fixation_in_photosynthetic_organisms_PATH_ko00710	Control vs T1D
688	0.3973843	0.6495082	Carbon_fixation_in_photosynthetic_organisms_PATH_ko00710	T1D vs T2D
1080	0.4591388	0.8415375	Carbon_fixation_in_photosynthetic_organisms_PATH_ko00710	LADA vs T2D

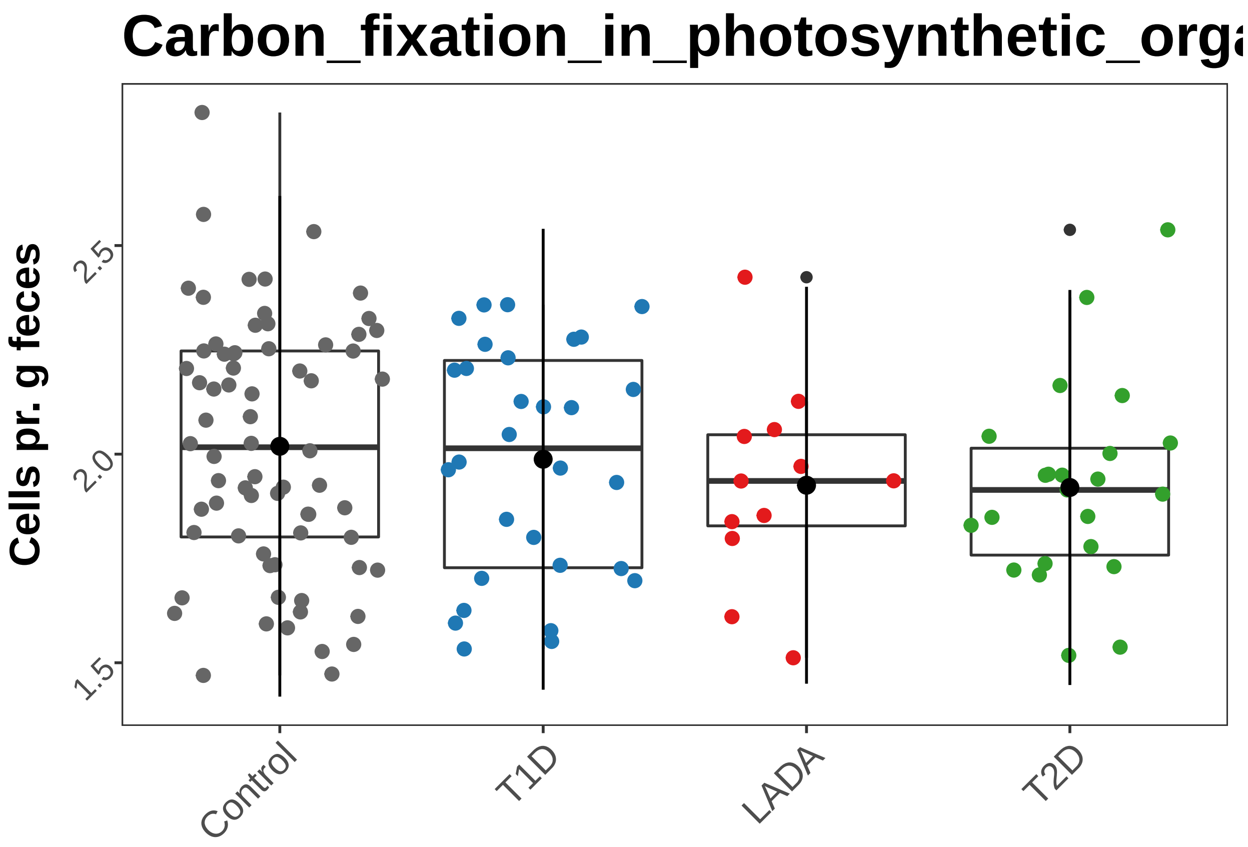

	pvalue	padj	Genus	compare
450	0.0049114	0.0346055	Cysteine_and_methionine_metabolism_PATH_ko00270	LADA vs Control
254	0.0176013	0.0629443	Cysteine_and_methionine_metabolism_PATH_ko00270	Control vs T2D
842	0.0886413	0.2443633	Cysteine_and_methionine_metabolism_PATH_ko00270	LADA vs T1D
58	0.1731049	0.4762288	Cysteine_and_methionine_metabolism_PATH_ko00270	Control vs T1D
646	0.3241323	0.6495082	Cysteine_and_methionine_metabolism_PATH_ko00270	T1D vs T2D
1038	0.3866531	0.8415375	Cysteine_and_methionine_metabolism_PATH_ko00270	LADA vs T2D

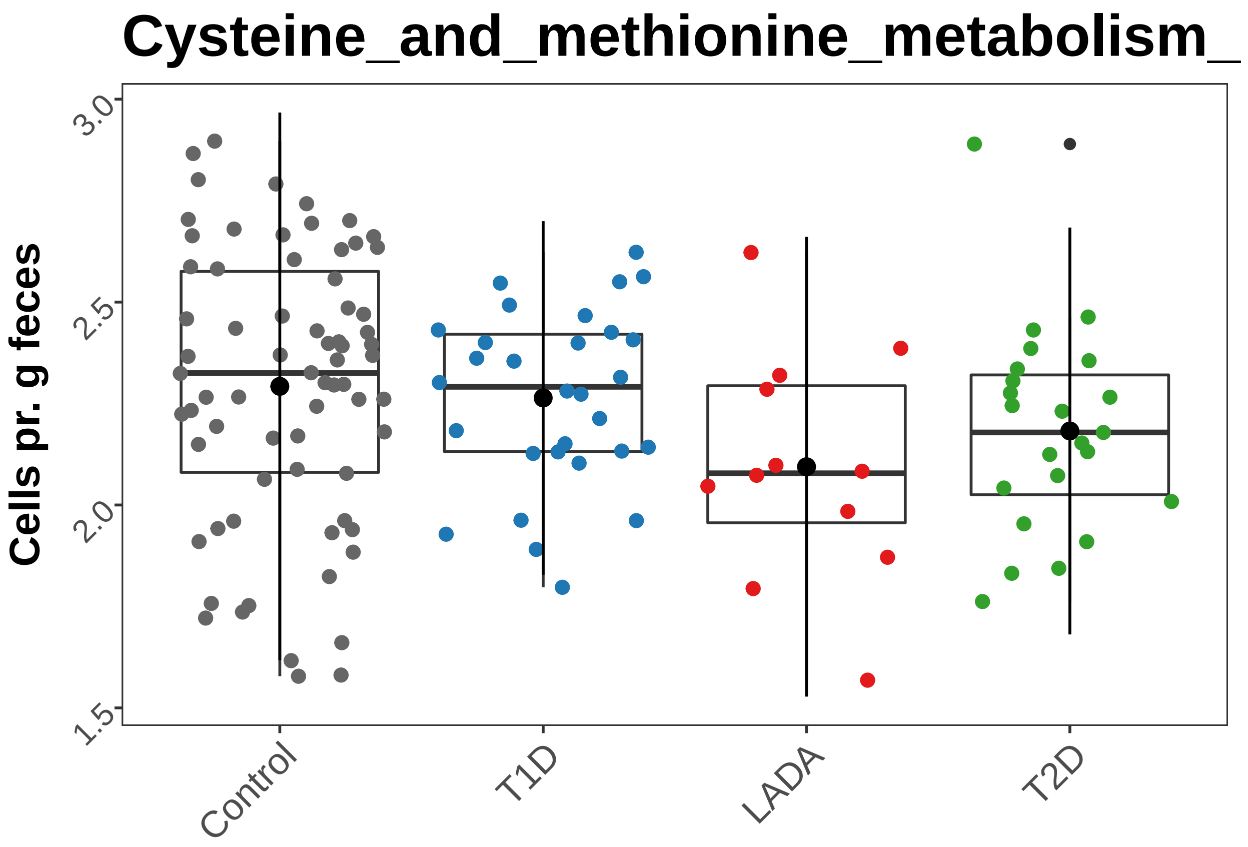

	pvalue	padj	Genus	compare
464	0.0051318	0.0346055	Carbapenem_biosynthesis_PATH_ko00332	LADA vs Control
268	0.0160134	0.0629443	Carbapenem_biosynthesis_PATH_ko00332	Control vs T2D
856	0.2079393	0.3628392	Carbapenem_biosynthesis_PATH_ko00332	LADA vs T1D
72	0.0417515	0.3989340	Carbapenem_biosynthesis_PATH_ko00332	Control vs T1D
660	0.6272200	0.7496044	Carbapenem_biosynthesis_PATH_ko00332	T1D vs T2D
1052	0.4066123	0.8415375	Carbapenem_biosynthesis_PATH_ko00332	LADA vs T2D

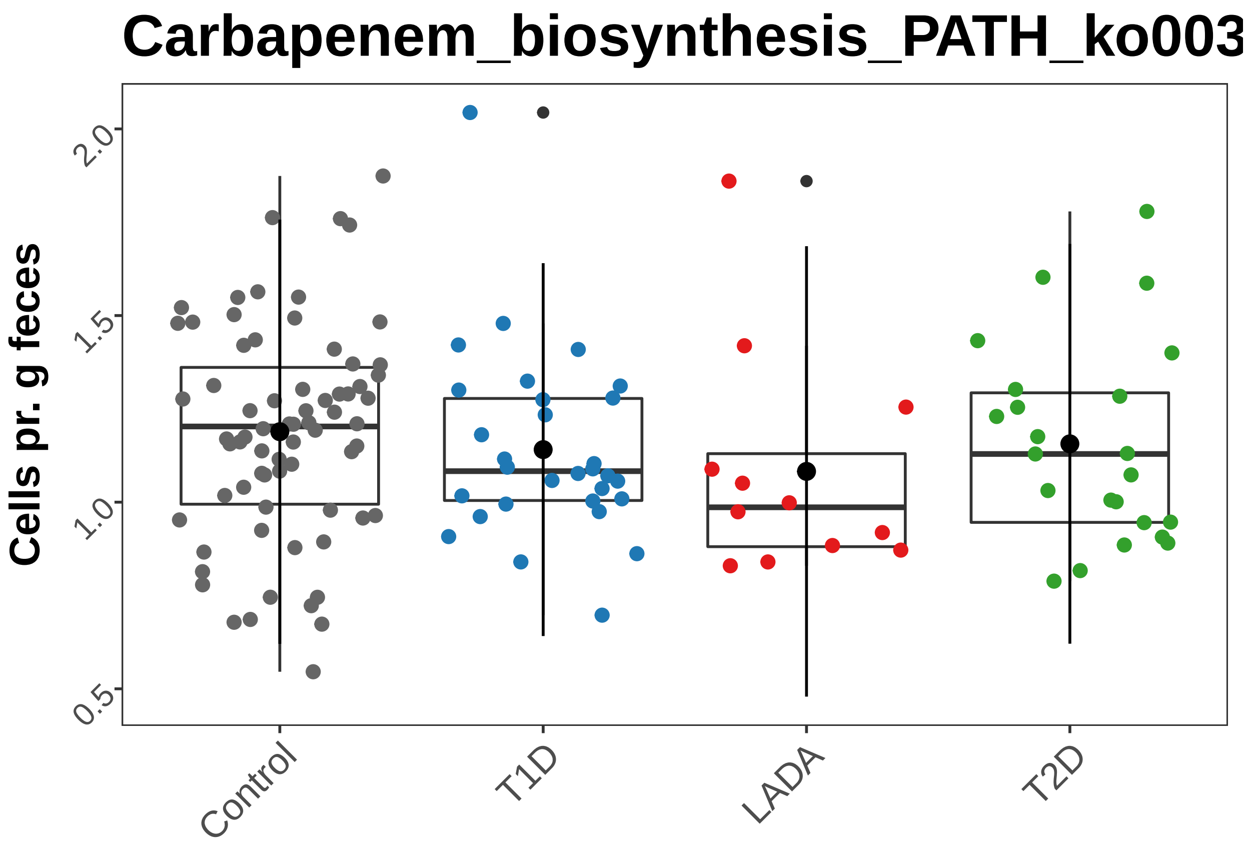

	pvalue	padj	Genus	compare
582	0.0052285	0.0346055	Biosynthesis_of_secondary_metabolites	LADA vs Control
386	0.0154866	0.0629443	Biosynthesis_of_secondary_metabolites	Control vs T2D
974	0.0897661	0.2443633	Biosynthesis_of_secondary_metabolites	LADA vs T1D
190	0.1793504	0.4762288	Biosynthesis_of_secondary_metabolites	Control vs T1D
778	0.2973876	0.6495082	Biosynthesis_of_secondary_metabolites	T1D vs T2D
1170	0.4143008	0.8415375	Biosynthesis_of_secondary_metabolites	LADA vs T2D

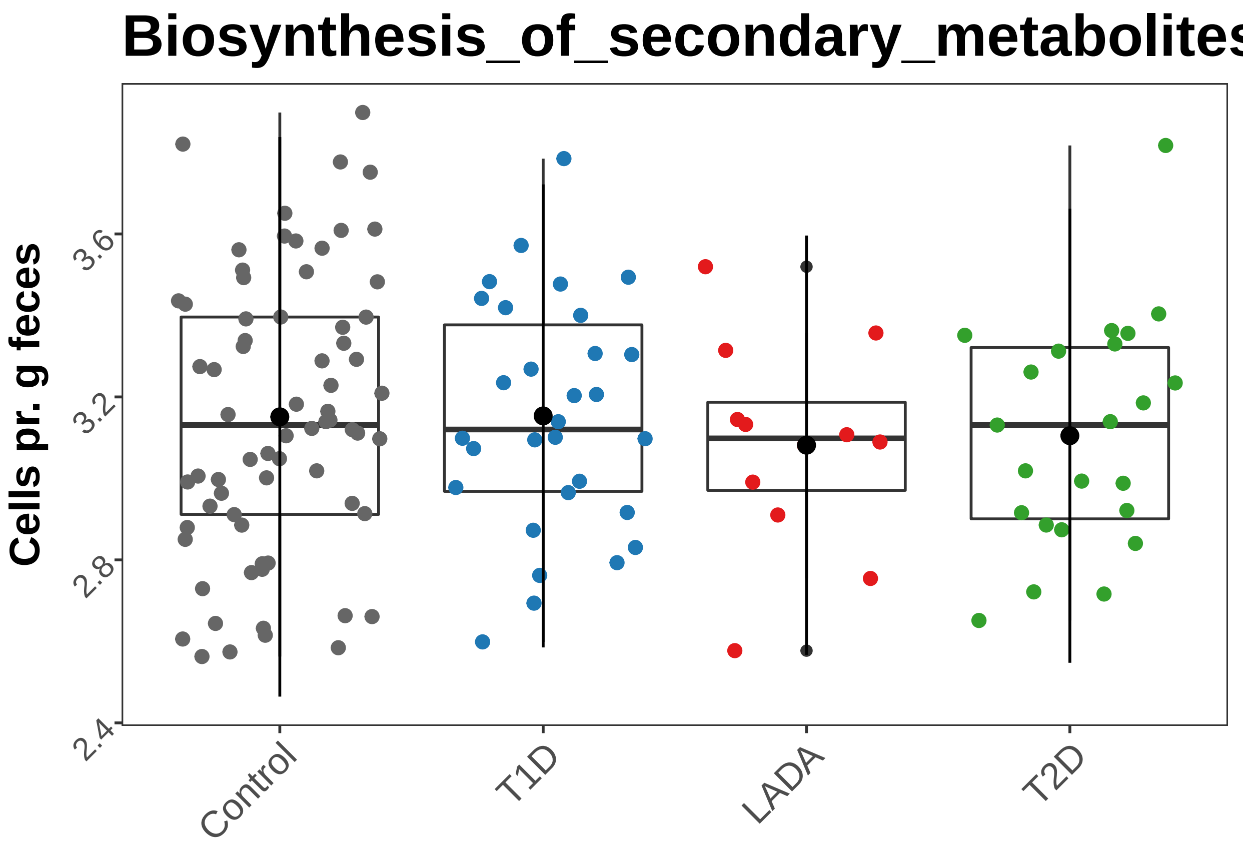

	pvalue	padj	Genus	compare
434	0.0053967	0.0346055	DNA_replication_PATH_ko03030	LADA vs Control
238	0.0403649	0.0767378	DNA_replication_PATH_ko03030	Control vs T2D
826	0.0474250	0.2312928	DNA_replication_PATH_ko03030	LADA vs T1D
42	0.3785154	0.5578121	DNA_replication_PATH_ko03030	Control vs T1D
630	0.2782070	0.6495082	DNA_replication_PATH_ko03030	T1D vs T2D
1022	0.2902359	0.8415375	DNA_replication_PATH_ko03030	LADA vs T2D

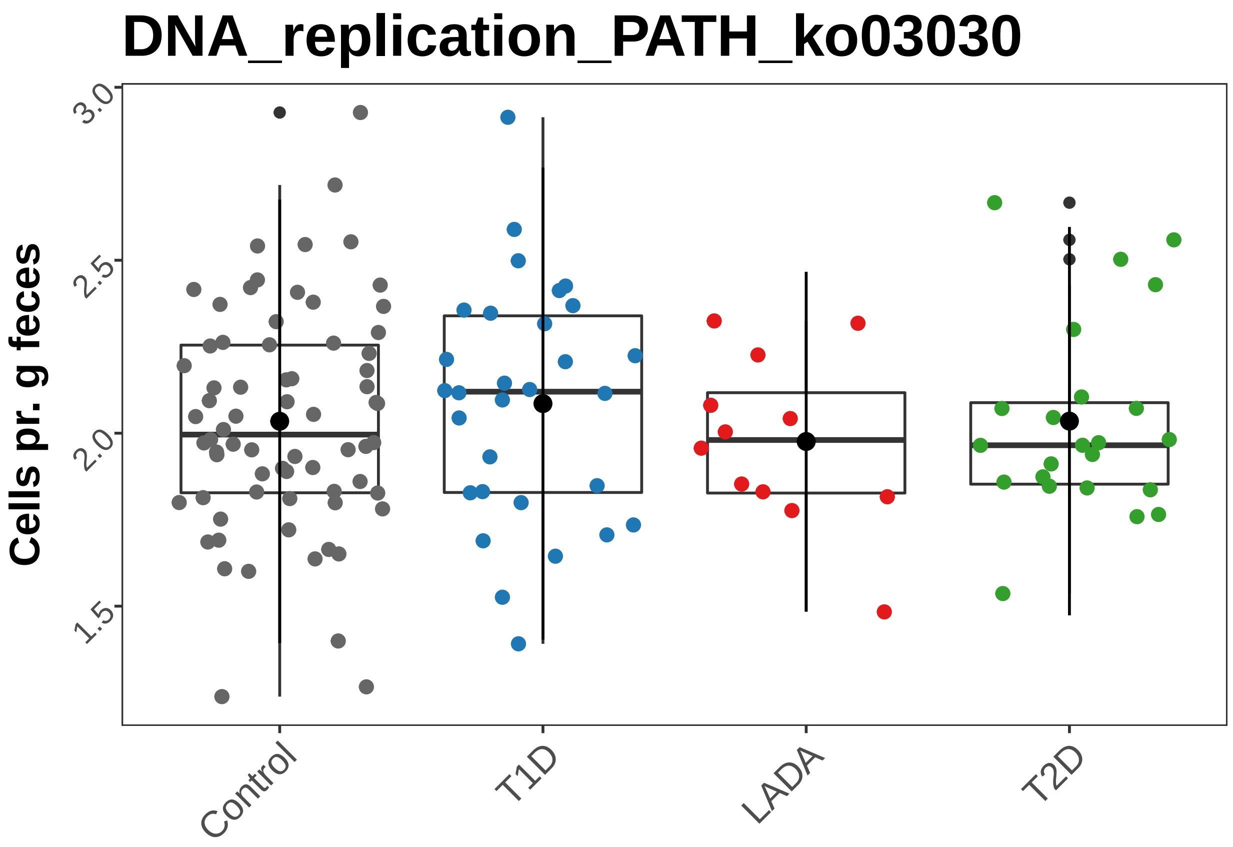

	pvalue	padj	Genus	compare
437	0.0054656	0.0346055	Mismatch_repair_PATH_ko03430	LADA vs Control
241	0.0108697	0.0629443	Mismatch_repair_PATH_ko03430	Control vs T2D
829	0.1780928	0.3554025	Mismatch_repair_PATH_ko03430	LADA vs T1D
45	0.0614876	0.3992571	Mismatch_repair_PATH_ko03430	Control vs T1D
633	0.4615725	0.6743371	Mismatch_repair_PATH_ko03430	T1D vs T2D
1025	0.4723578	0.8415375	Mismatch_repair_PATH_ko03430	LADA vs T2D

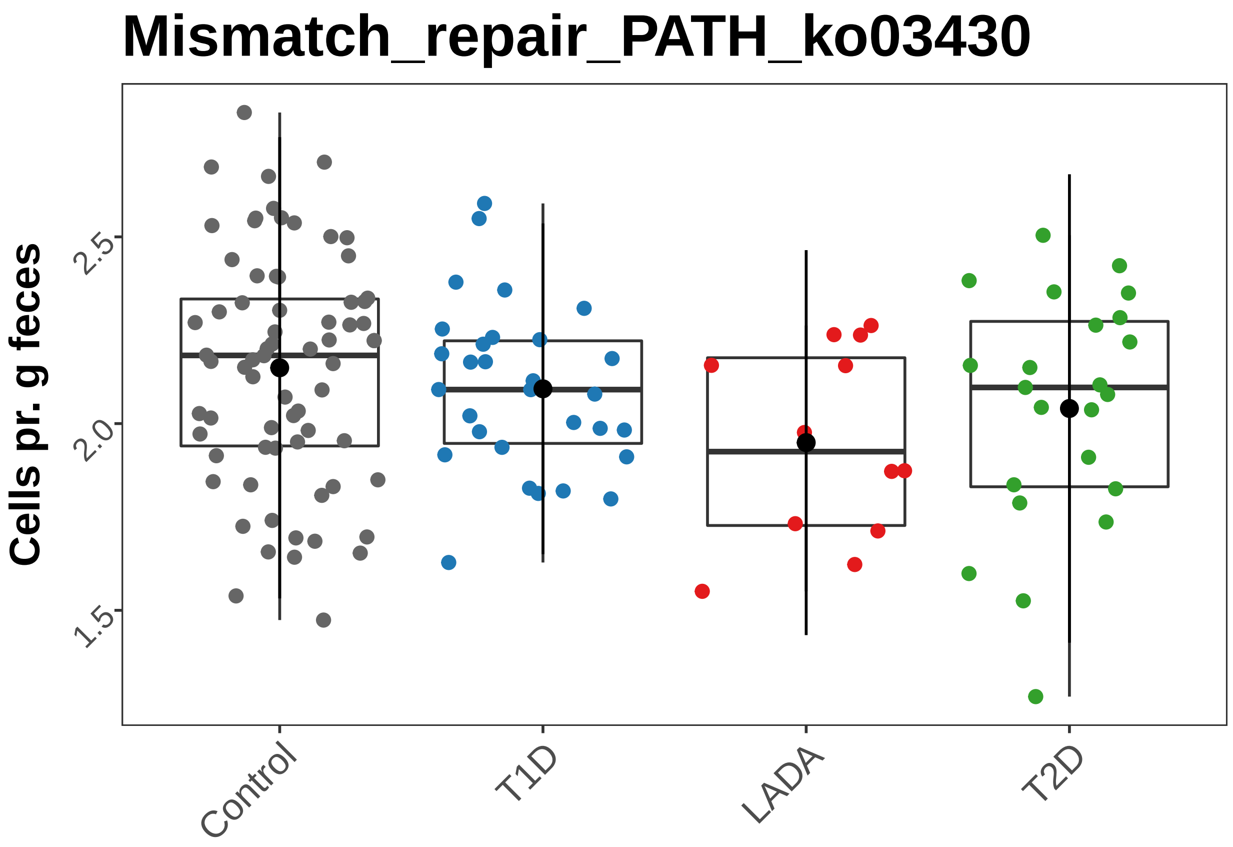

	pvalue	padj	Genus	compare
556	0.0057776	0.0346055	Polyketide_sugar_unit_biosynthesis_PATH_ko00523	LADA vs Control
360	0.1222032	0.1708773	Polyketide_sugar_unit_biosynthesis_PATH_ko00523	Control vs T2D
948	0.0447616	0.2312928	Polyketide_sugar_unit_biosynthesis_PATH_ko00523	LADA vs T1D
164	0.4175665	0.5940928	Polyketide_sugar_unit_biosynthesis_PATH_ko00523	Control vs T1D
752	0.4826515	0.6743371	Polyketide_sugar_unit_biosynthesis_PATH_ko00523	T1D vs T2D
1144	0.1680312	0.8415375	Polyketide_sugar_unit_biosynthesis_PATH_ko00523	LADA vs T2D

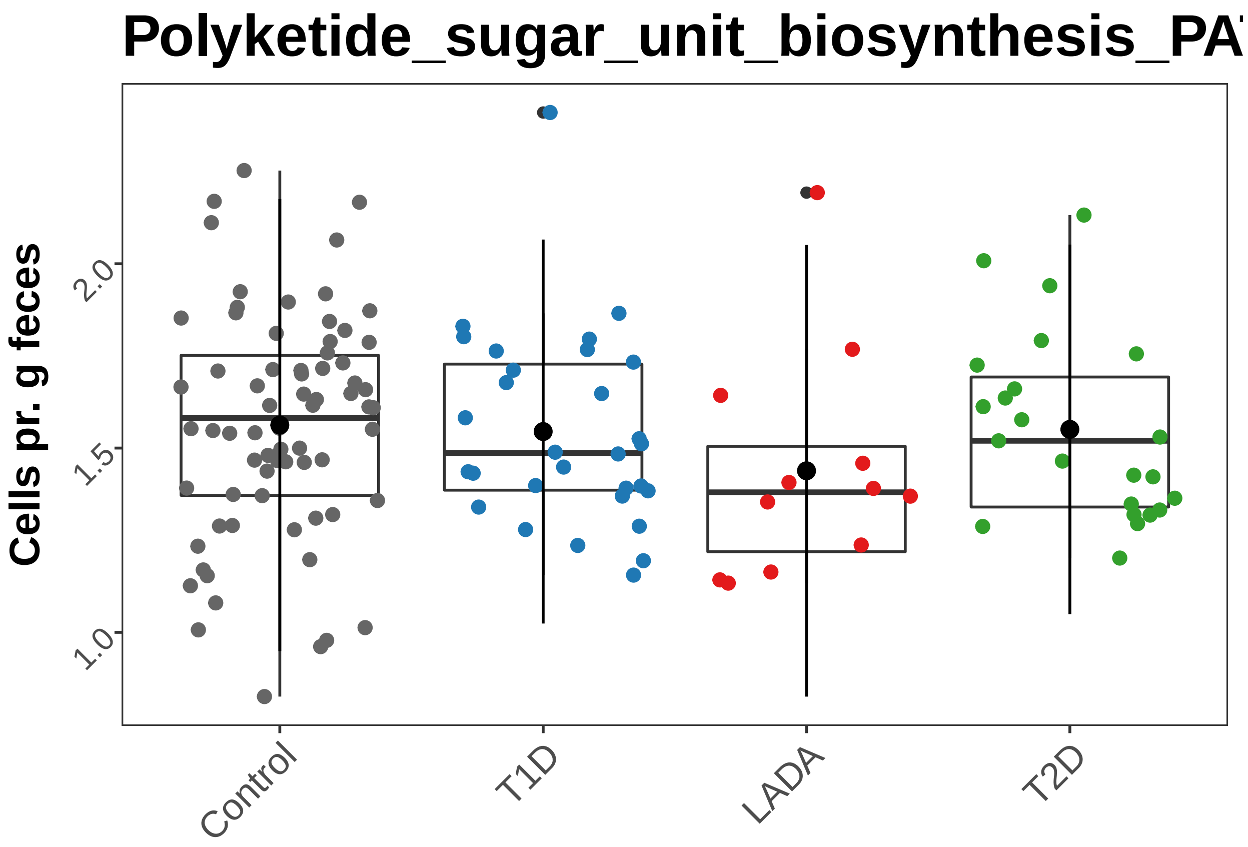

	pvalue	padj	Genus	compare
588	0.0058607	0.0346055	Microbial_metabolism_in_diverse_environments	LADA vs Control
392	0.0296219	0.0676342	Microbial_metabolism_in_diverse_environments	Control vs T2D
980	0.0260302	0.2312928	Microbial_metabolism_in_diverse_environments	LADA vs T1D
784	0.1277677	0.6495082	Microbial_metabolism_in_diverse_environments	T1D vs T2D
196	0.6445281	0.7564521	Microbial_metabolism_in_diverse_environments	Control vs T1D
1176	0.3423220	0.8415375	Microbial_metabolism_in_diverse_environments	LADA vs T2D

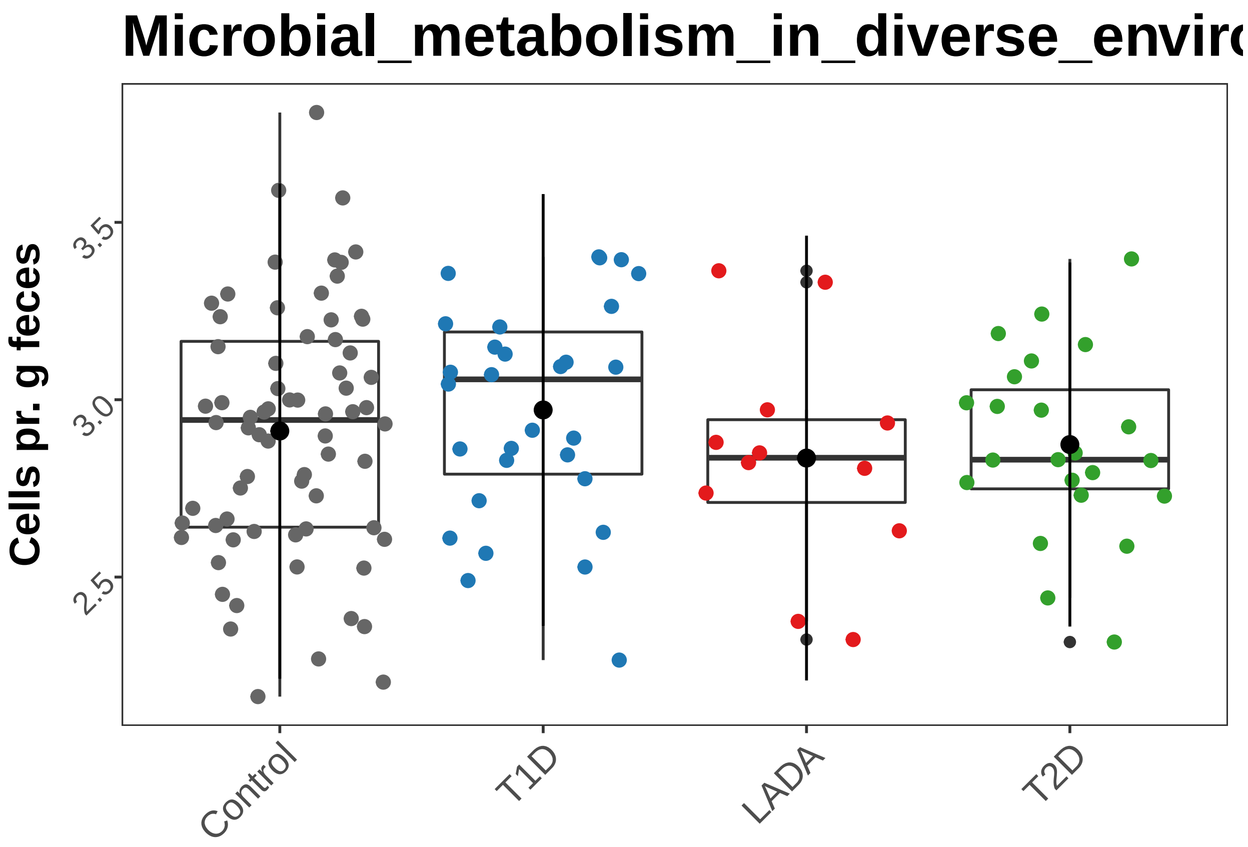

	pvalue	padj	Genus	compare
399	0.0059859	0.0346055	Necroptosis_PATH_ko04217	LADA vs Control
203	0.0098607	0.0629443	Necroptosis_PATH_ko04217	Control vs T2D
791	0.1961349	0.3597318	Necroptosis_PATH_ko04217	LADA vs T1D
7	0.0558735	0.3992571	Necroptosis_PATH_ko04217	Control vs T1D
595	0.4638783	0.6743371	Necroptosis_PATH_ko04217	T1D vs T2D
987	0.5029915	0.8415375	Necroptosis_PATH_ko04217	LADA vs T2D

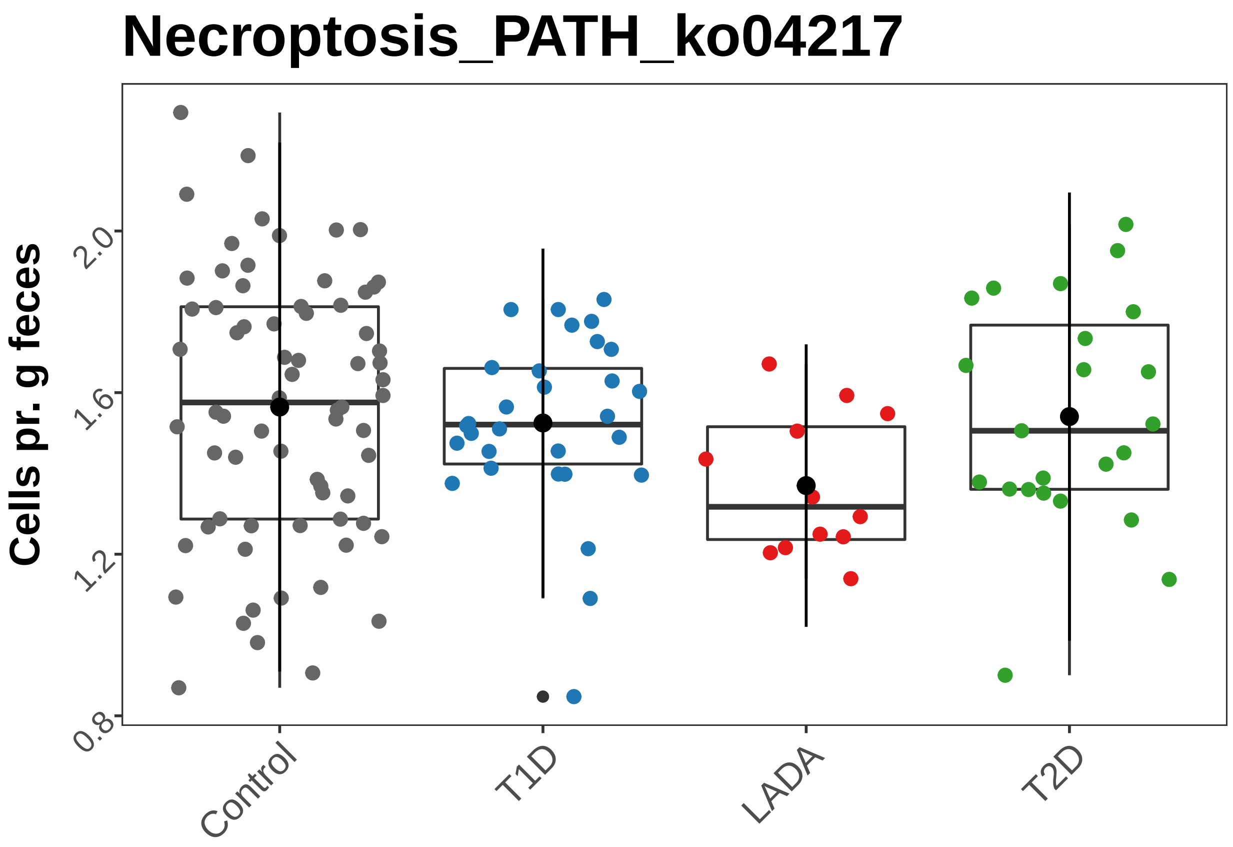

	pvalue	padj	Genus	compare
468	0.0063136	0.0346055	Monobactam_biosynthesis_PATH_ko00261	LADA vs Control
272	0.0124246	0.0629443	Monobactam_biosynthesis_PATH_ko00261	Control vs T2D
860	0.1212508	0.2744316	Monobactam_biosynthesis_PATH_ko00261	LADA vs T1D
76	0.1375919	0.4612224	Monobactam_biosynthesis_PATH_ko00261	Control vs T1D
664	0.3179150	0.6495082	Monobactam_biosynthesis_PATH_ko00261	T1D vs T2D
1056	0.4783453	0.8415375	Monobactam_biosynthesis_PATH_ko00261	LADA vs T2D

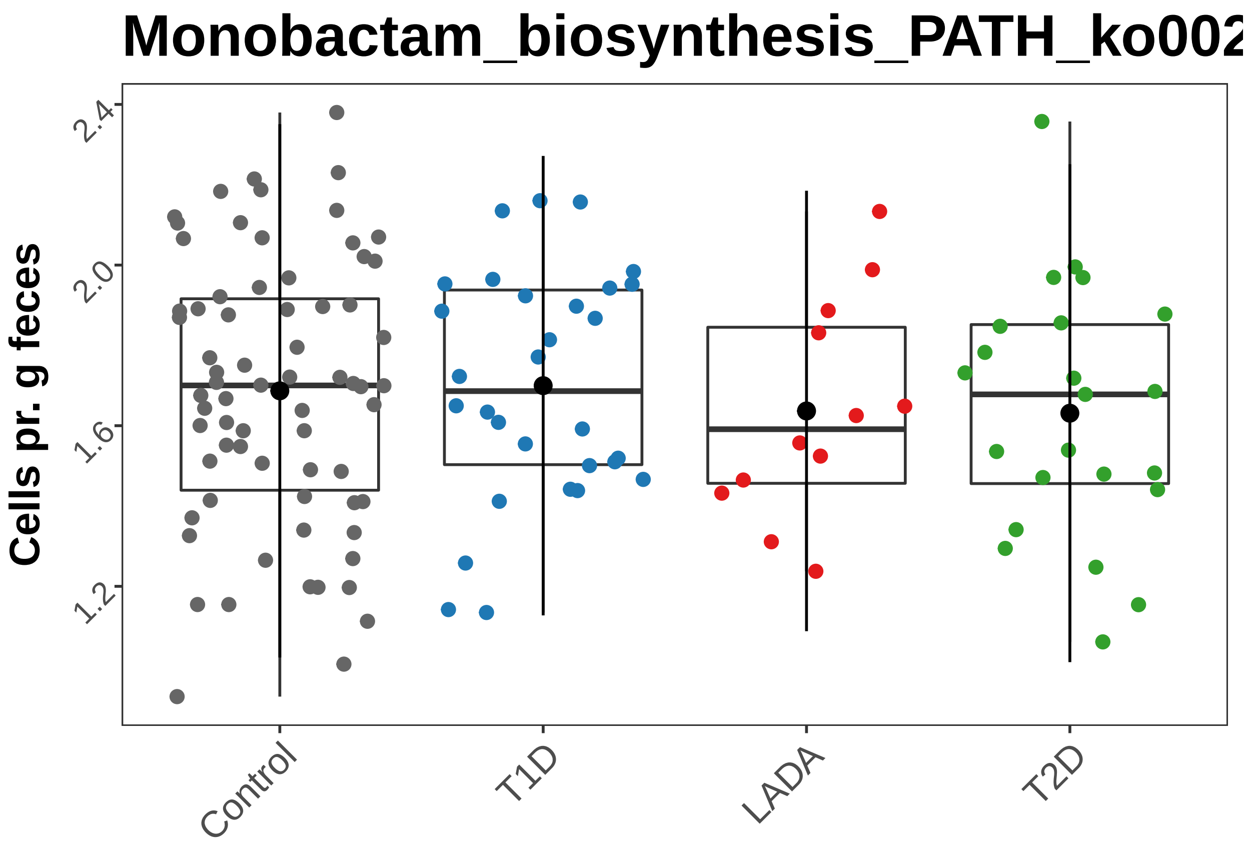

	pvalue	padj	Genus	compare
510	0.0067198	0.0346055	Peptidoglycan__biosynthesis__PATH__ko00550	LADA vs Control
314	0.0049509	0.0629443	Peptidoglycan__biosynthesis__PATH__ko00550	Control vs T2D
902	0.1480007	0.3094575	Peptidoglycan__biosynthesis__PATH__ko00550	LADA vs T1D
118	0.1060094	0.4612224	Peptidoglycan__biosynthesis__PATH__ko00550	Control vs T1D
706	0.2443036	0.6495082	Peptidoglycan__biosynthesis__PATH__ko00550	T1D vs T2D
1098	0.6302230	0.8841442	Peptidoglycan__biosynthesis__PATH__ko00550	LADA vs T2D

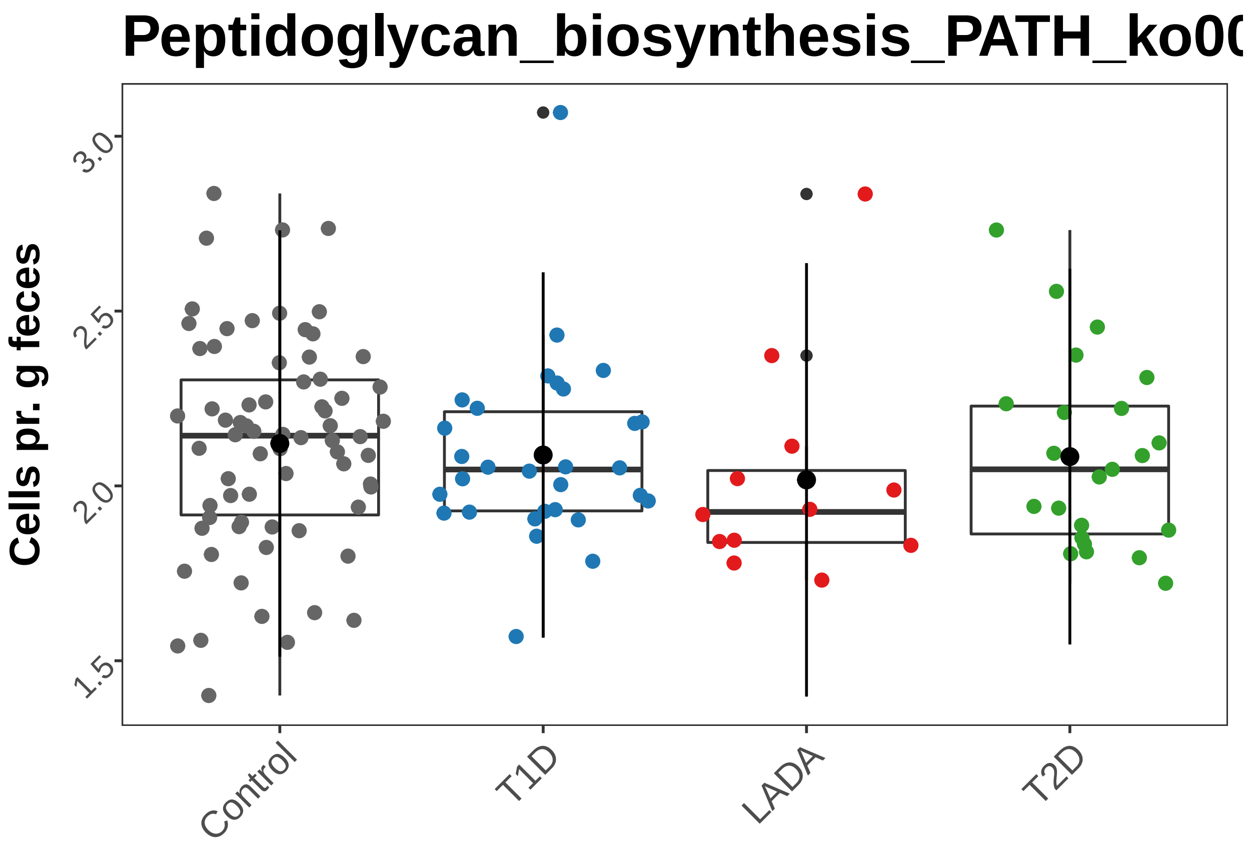

	pvalue	padj	Genus	compare
452	0.0067429	0.0346055	Histidine_metabolism_PATH_ko00340	LADA vs Control
256	0.0257700	0.0676342	Histidine_metabolism_PATH_ko00340	Control vs T2D
844	0.0600206	0.2312928	Histidine_metabolism_PATH_ko00340	LADA vs T1D
60	0.3495677	0.5414362	Histidine_metabolism_PATH_ko00340	Control vs T1D
648	0.2312996	0.6495082	Histidine_metabolism_PATH_ko00340	T1D vs T2D
1040	0.3830784	0.8415375	Histidine_metabolism_PATH_ko00340	LADA vs T2D

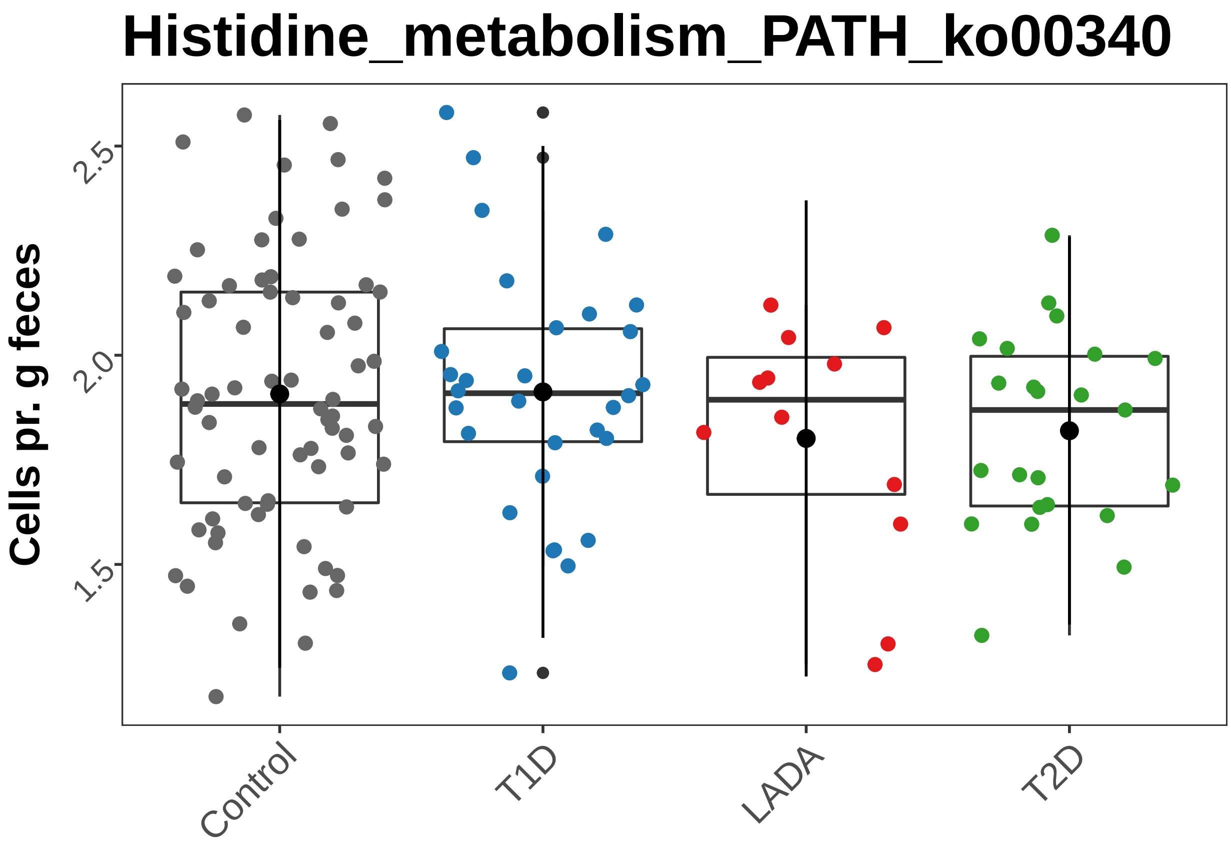

	pvalue	padj	Genus	compare
560	0.0071435	0.0346055	Purine_metabolism_PATH_ko00230	LADA vs Control
364	0.0268585	0.0676342	Purine_metabolism_PATH_ko00230	Control vs T2D
952	0.0621940	0.2312928	Purine_metabolism_PATH_ko00230	LADA vs T1D
168	0.3510477	0.5414362	Purine_metabolism_PATH_ko00230	Control vs T1D
756	0.2358902	0.6495082	Purine_metabolism_PATH_ko00230	T1D vs T2D
1148	0.3863526	0.8415375	Purine_metabolism_PATH_ko00230	LADA vs T2D

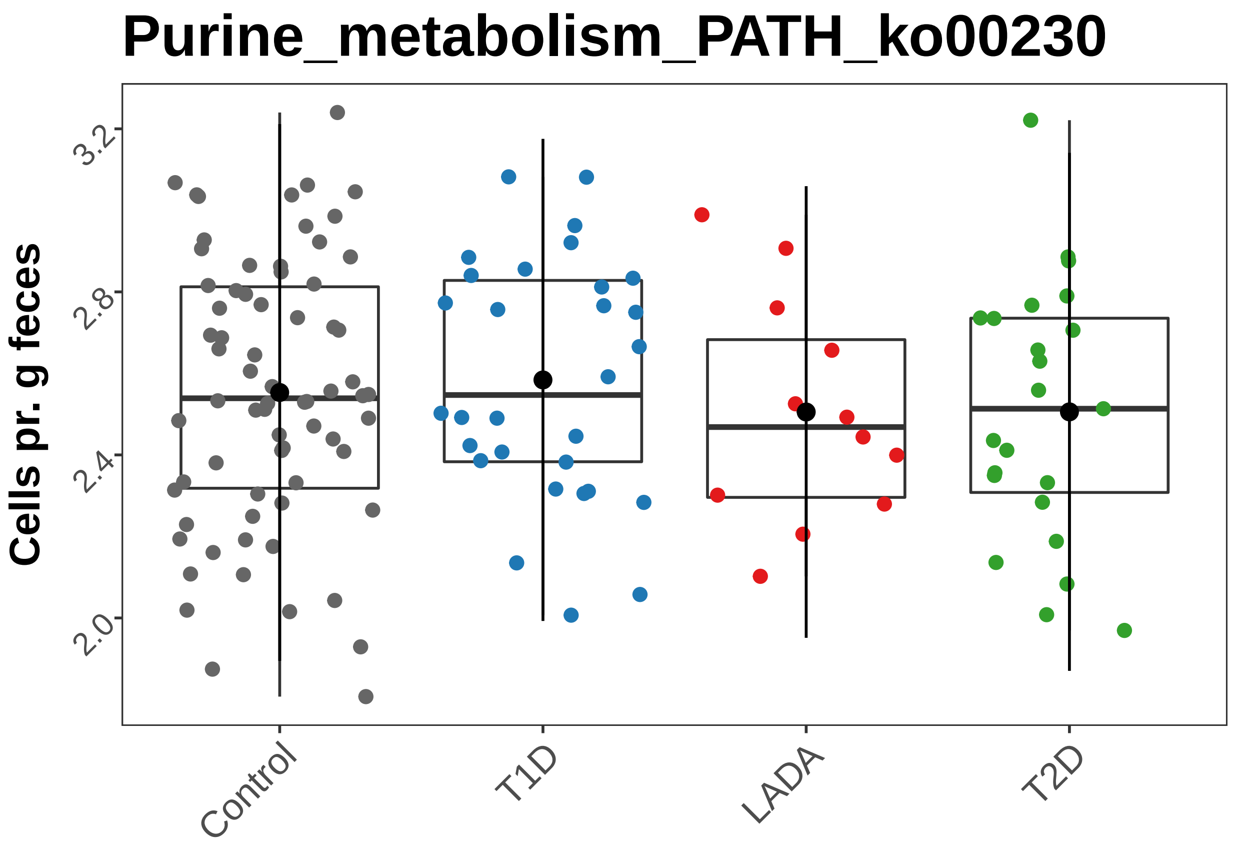

	pvalue	padj	Genus	compare
451	0.0072330	0.0346055	Glycine___serine_and_threonine_metabolism_PATH_ko00260	LADA vs Control
255	0.0360466	0.0725327	Glycine___serine_and_threonine_metabolism_PATH_ko00260	Control vs T2D
843	0.0919352	0.2458003	Glycine___serine_and_threonine_metabolism_PATH_ko00260	LADA vs T1D
59	0.2271314	0.4786856	Glycine___serine_and_threonine_metabolism_PATH_ko00260	Control vs T1D
647	0.3859337	0.6495082	Glycine___serine_and_threonine_metabolism_PATH_ko00260	T1D vs T2D
1039	0.3463547	0.8415375	Glycine___serine_and_threonine_metabolism_PATH_ko00260	LADA vs T2D

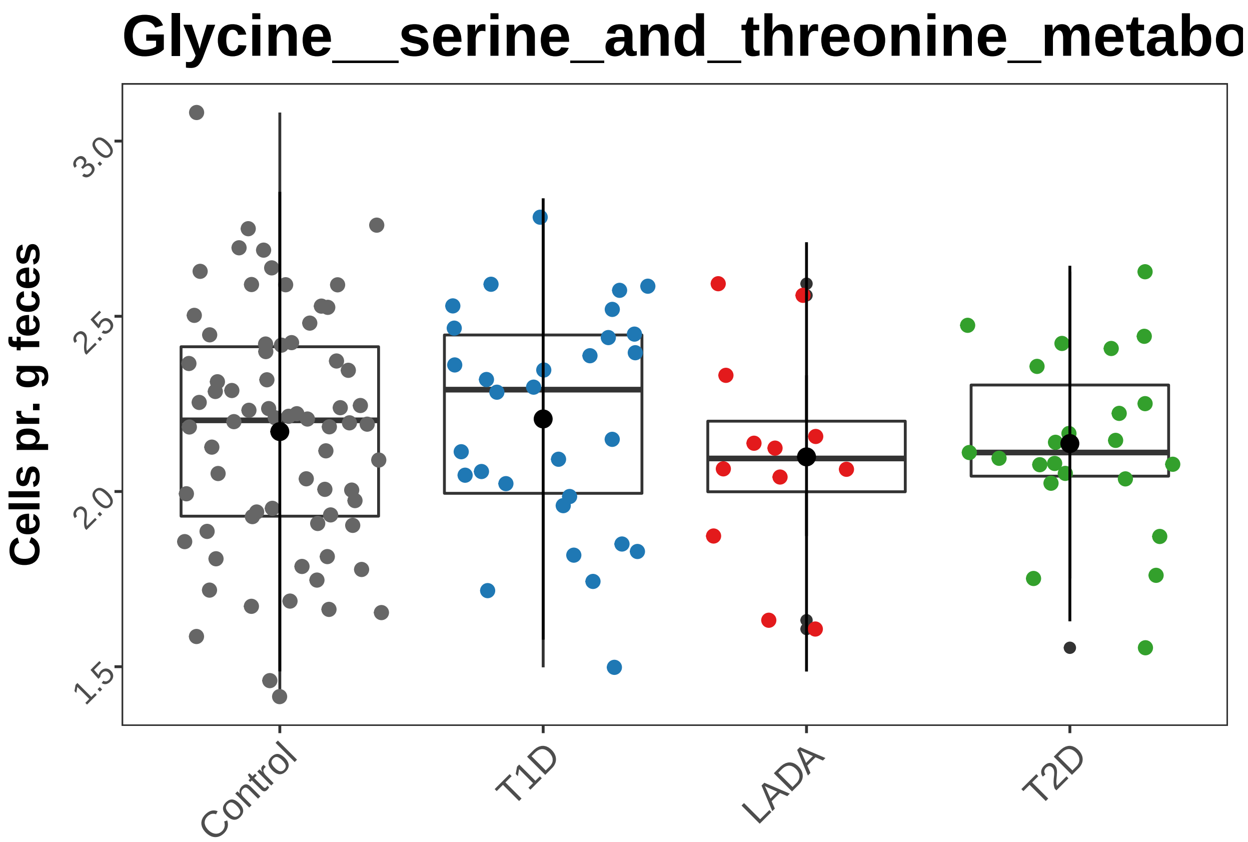

	pvalue	padj	Genus	compare
429	0.0073488	0.0346055	Protein_export_PATH_ko03060	LADA vs Control
233	0.0125229	0.0629443	Protein_export_PATH_ko03060	Control vs T2D
821	0.0990909	0.2458003	Protein_export_PATH_ko03060	LADA vs T1D
37	0.2089255	0.4786856	Protein_export_PATH_ko03060	Control vs T1D
625	0.2395623	0.6495082	Protein_export_PATH_ko03060	T1D vs T2D
1017	0.5050186	0.8415375	Protein_export_PATH_ko03060	LADA vs T2D

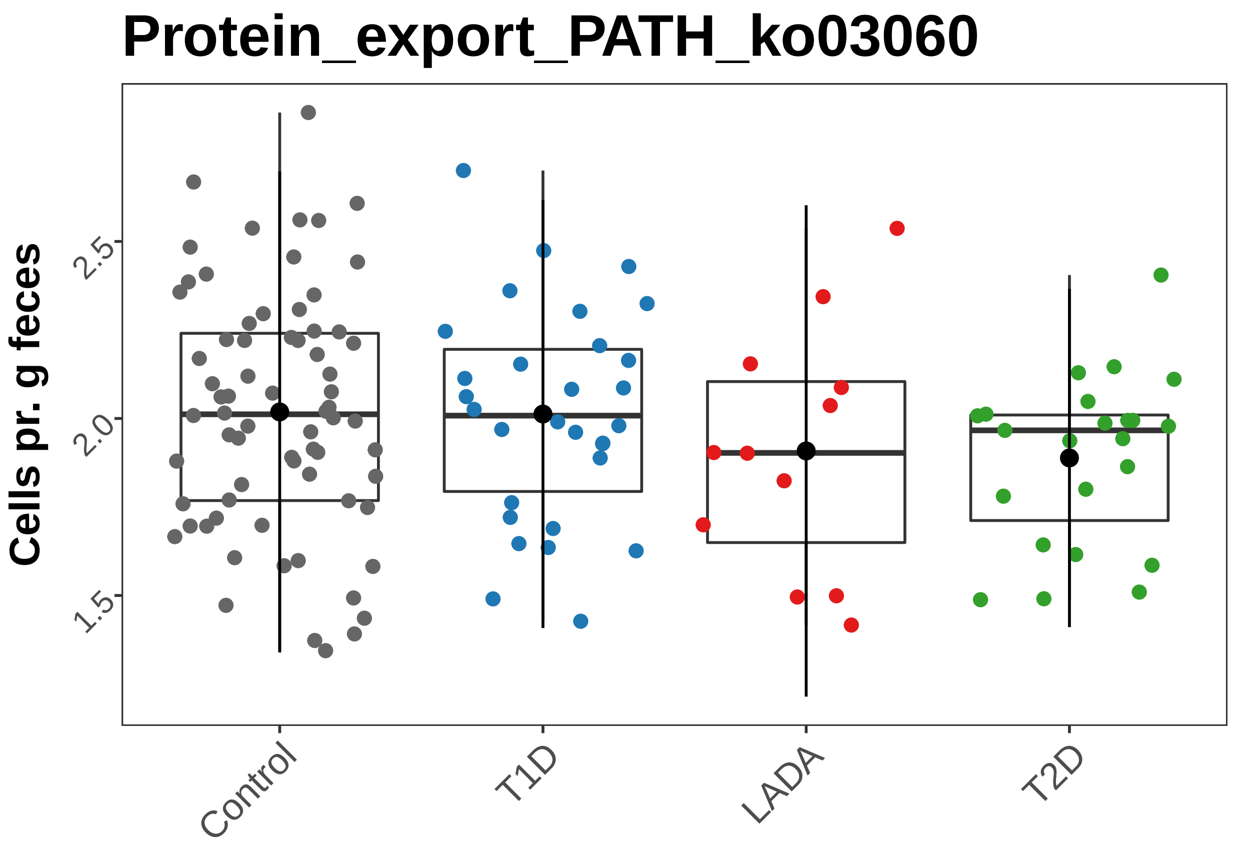

	pvalue	padj	Genus	compare
447	0.0074117	0.0346055	Alanine___aspartate_and_glutamate_metabolism_PATH_ko00250	LADA vs Control
251	0.0357249	0.0725327	Alanine___aspartate_and_glutamate_metabolism_PATH_ko00250	Control vs T2D
839	0.0982838	0.2458003	Alanine___aspartate_and_glutamate_metabolism_PATH_ko00250	LADA vs T1D
55	0.2126734	0.4786856	Alanine___aspartate_and_glutamate_metabolism_PATH_ko00250	Control vs T1D
643	0.4009719	0.6495082	Alanine___aspartate_and_glutamate_metabolism_PATH_ko00250	T1D vs T2D
1035	0.3513056	0.8415375	Alanine___aspartate_and_glutamate_metabolism_PATH_ko00250	LADA vs T2D

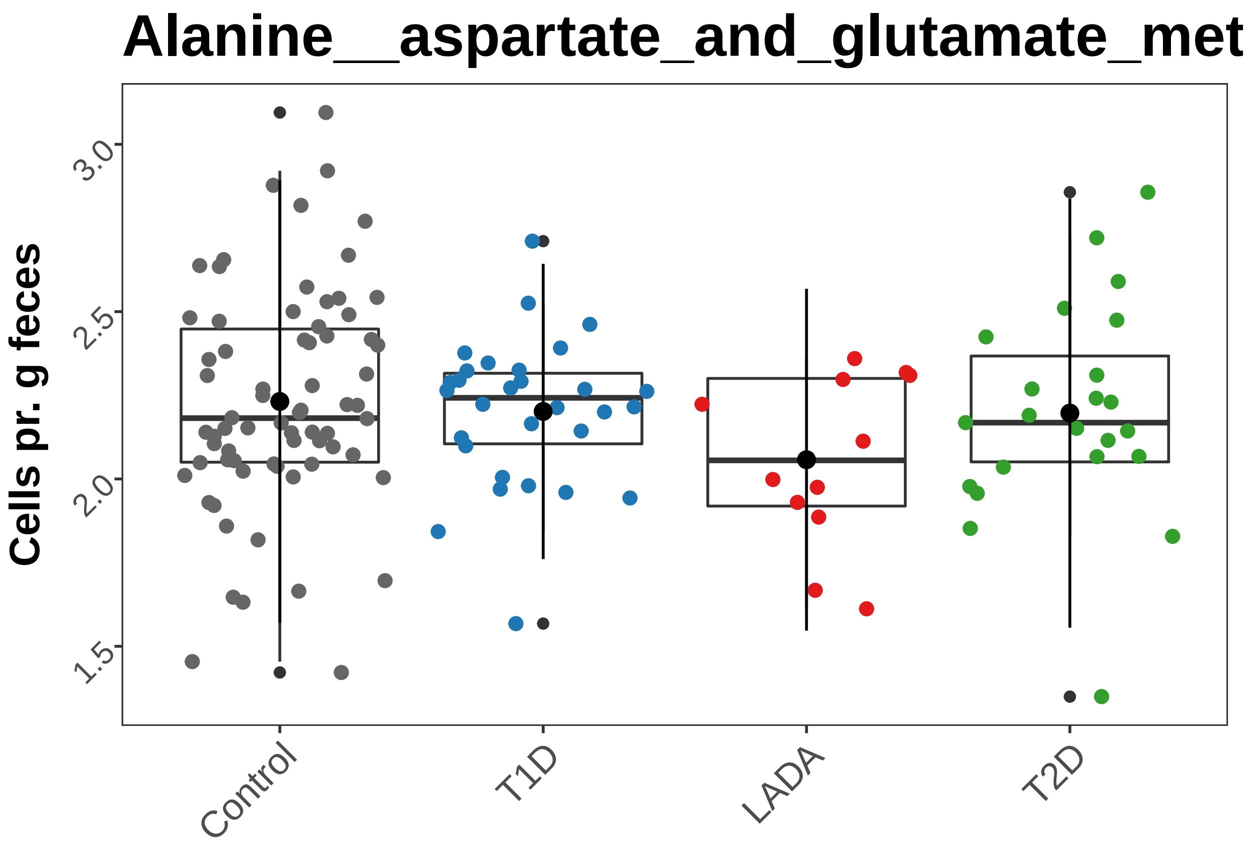

	pvalue	padj	Genus	compare
587	0.0078966	0.0346055	Metabolic_pathways	LADA vs Control
391	0.0208497	0.0661577	Metabolic_pathways	Control vs T2D
979	0.0805437	0.2321553	Metabolic_pathways	LADA vs T1D
195	0.2854587	0.5040976	Metabolic_pathways	Control vs T1D
783	0.2449106	0.6495082	Metabolic_pathways	T1D vs T2D
1175	0.4405875	0.8415375	Metabolic_pathways	LADA vs T2D

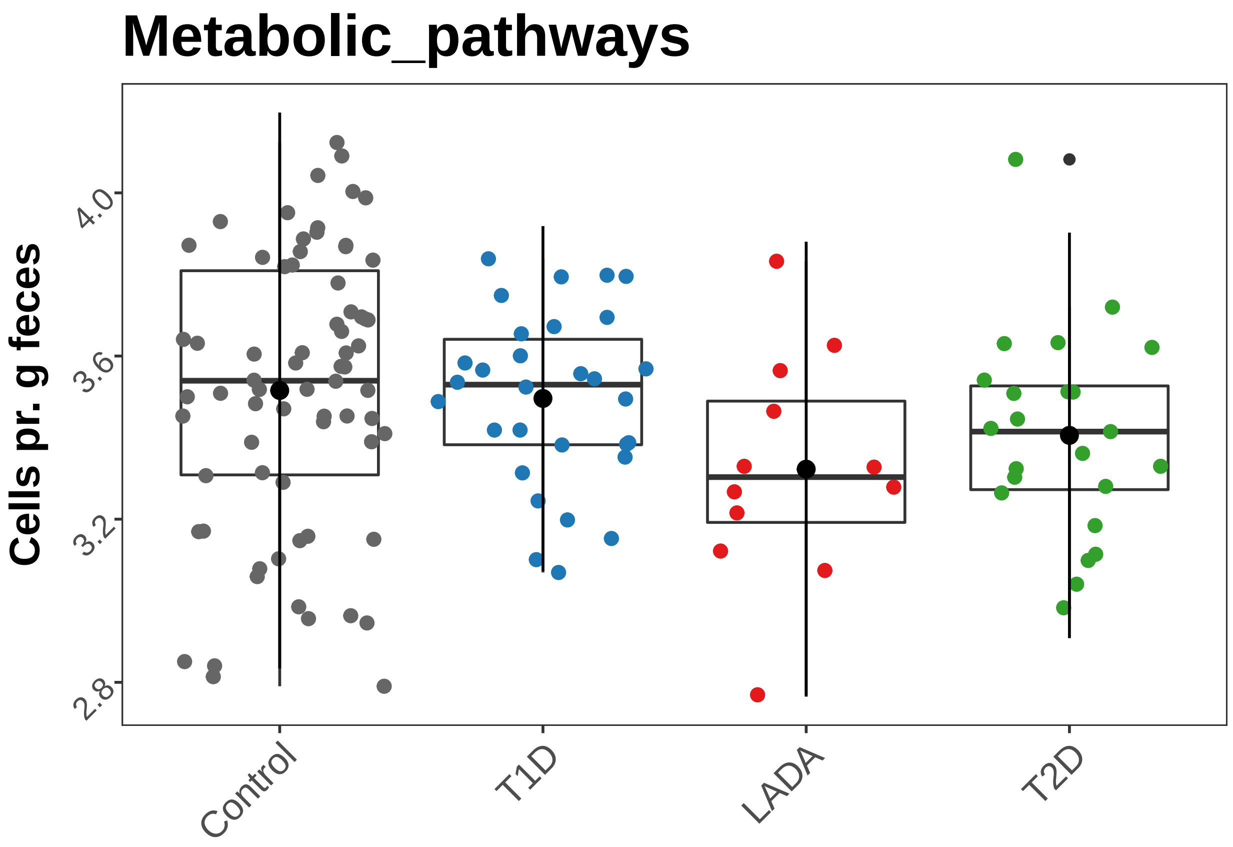

	pvalue	padj	Genus	compare
561	0.0081670	0.0346055	Pyrimidine_metabolism_PATH_ko00240	LADA vs Control
365	0.0276553	0.0676342	Pyrimidine_metabolism_PATH_ko00240	Control vs T2D
953	0.0461198	0.2312928	Pyrimidine_metabolism_PATH_ko00240	LADA vs T1D
757	0.1658723	0.6495082	Pyrimidine_metabolism_PATH_ko00240	T1D vs T2D
169	0.5056515	0.6651524	Pyrimidine_metabolism_PATH_ko00240	Control vs T1D
1149	0.4040054	0.8415375	Pyrimidine_metabolism_PATH_ko00240	LADA vs T2D

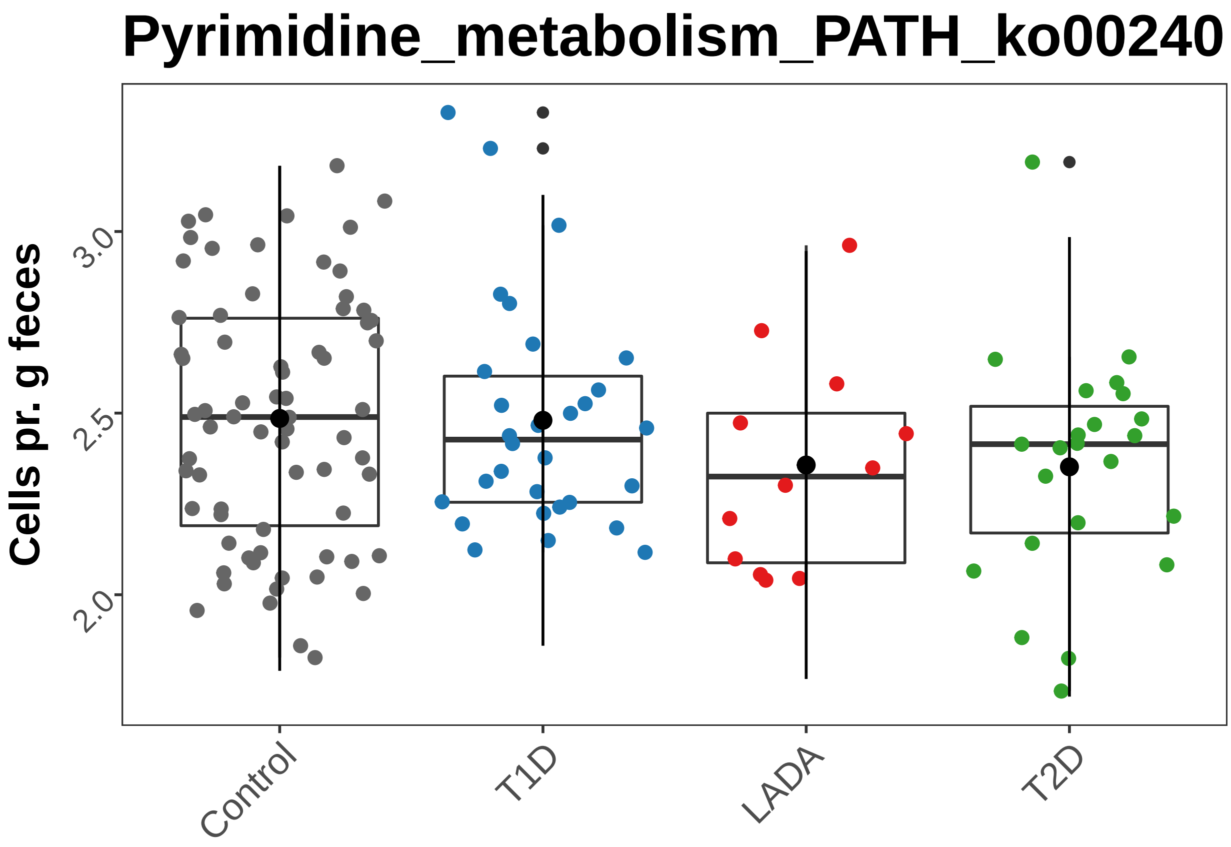

	pvalue	padj	Genus	compare
470	0.0084018	0.0346055	Novobiocin_biosynthesis_PATH_ko00401	LADA vs Control
274	0.0346820	0.0724371	Novobiocin_biosynthesis_PATH_ko00401	Control vs T2D
862	0.0877216	0.2443633	Novobiocin_biosynthesis_PATH_ko00401	LADA vs T1D
78	0.2710234	0.4873448	Novobiocin_biosynthesis_PATH_ko00401	Control vs T1D
666	0.3345997	0.6495082	Novobiocin_biosynthesis_PATH_ko00401	T1D vs T2D
1058	0.3751514	0.8415375	Novobiocin_biosynthesis_PATH_ko00401	LADA vs T2D

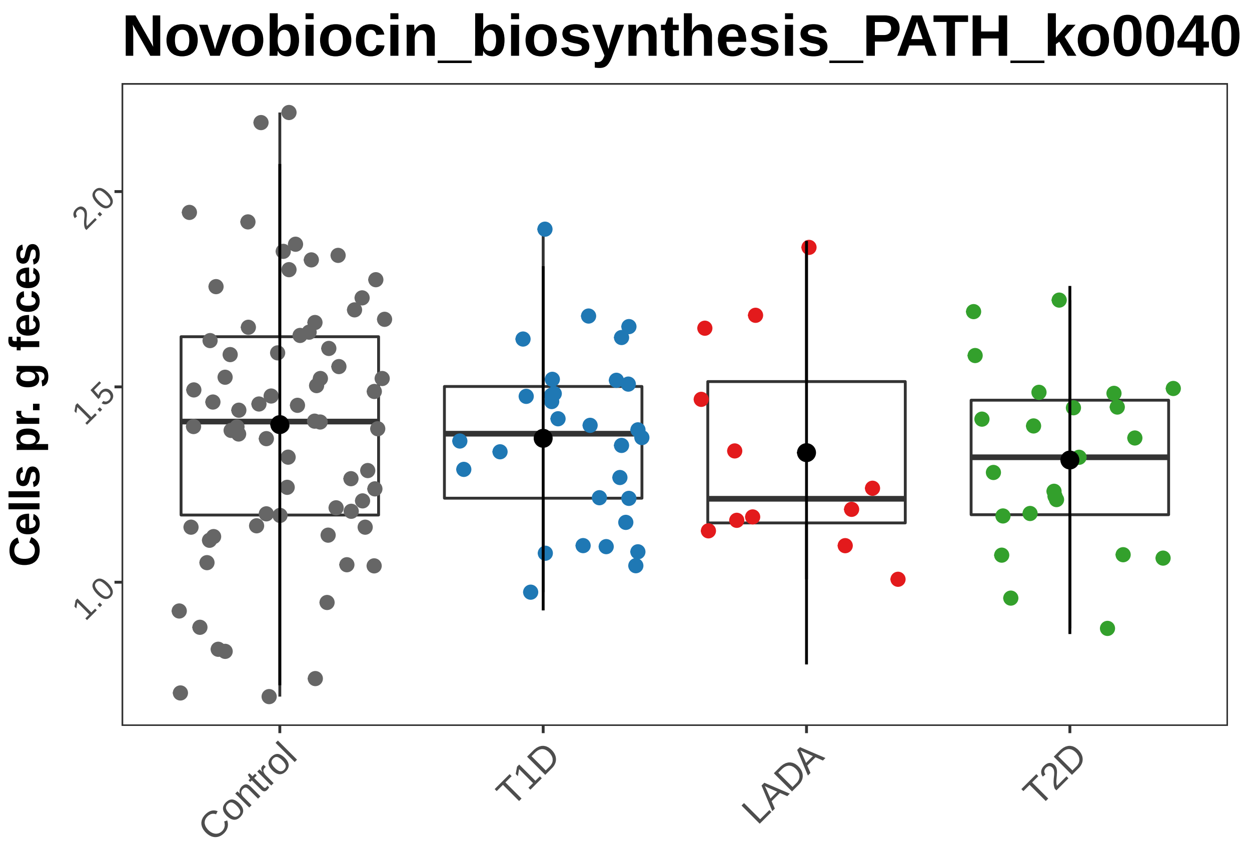

	pvalue	padj	Genus	compare
444	0.0084422	0.0346055	Ribosome_PATH_ko03010	LADA vs Control
248	0.0589387	0.0981236	Ribosome_PATH_ko03010	Control vs T2D
836	0.0350523	0.2312928	Ribosome_PATH_ko03010	LADA vs T1D
640	0.2054318	0.6495082	Ribosome_PATH_ko03010	T1D vs T2D
52	0.6369647	0.7537556	Ribosome_PATH_ko03010	Control vs T1D
1032	0.3001035	0.8415375	Ribosome_PATH_ko03010	LADA vs T2D

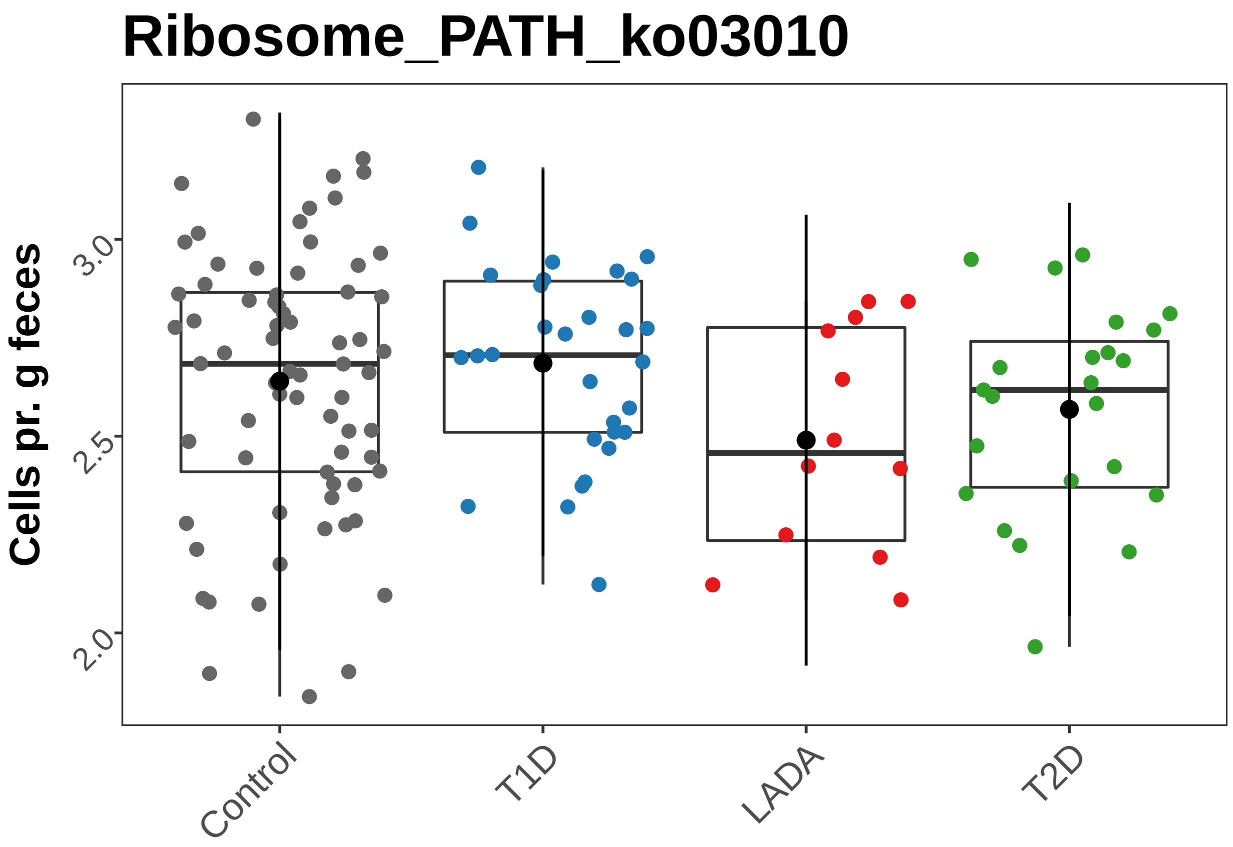

	pvalue	padj	Genus	compare
543	0.0085715	0.0346055	D_Glutamine_and_D_glutamate_metabolism_PATH_ko00471	LADA vs Control
347	0.0383866	0.0763108	D_Glutamine_and_D_glutamate_metabolism_PATH_ko00471	Control vs T2D
935	0.0689628	0.2312928	D_Glutamine_and_D_glutamate_metabolism_PATH_ko00471	LADA vs T1D
151	0.3591158	0.5414362	D_Glutamine_and_D_glutamate_metabolism_PATH_ko00471	Control vs T1D
739	0.2829842	0.6495082	D_Glutamine_and_D_glutamate_metabolism_PATH_ko00471	T1D vs T2D
1131	0.3634483	0.8415375	D_Glutamine_and_D_glutamate_metabolism_PATH_ko00471	LADA vs T2D

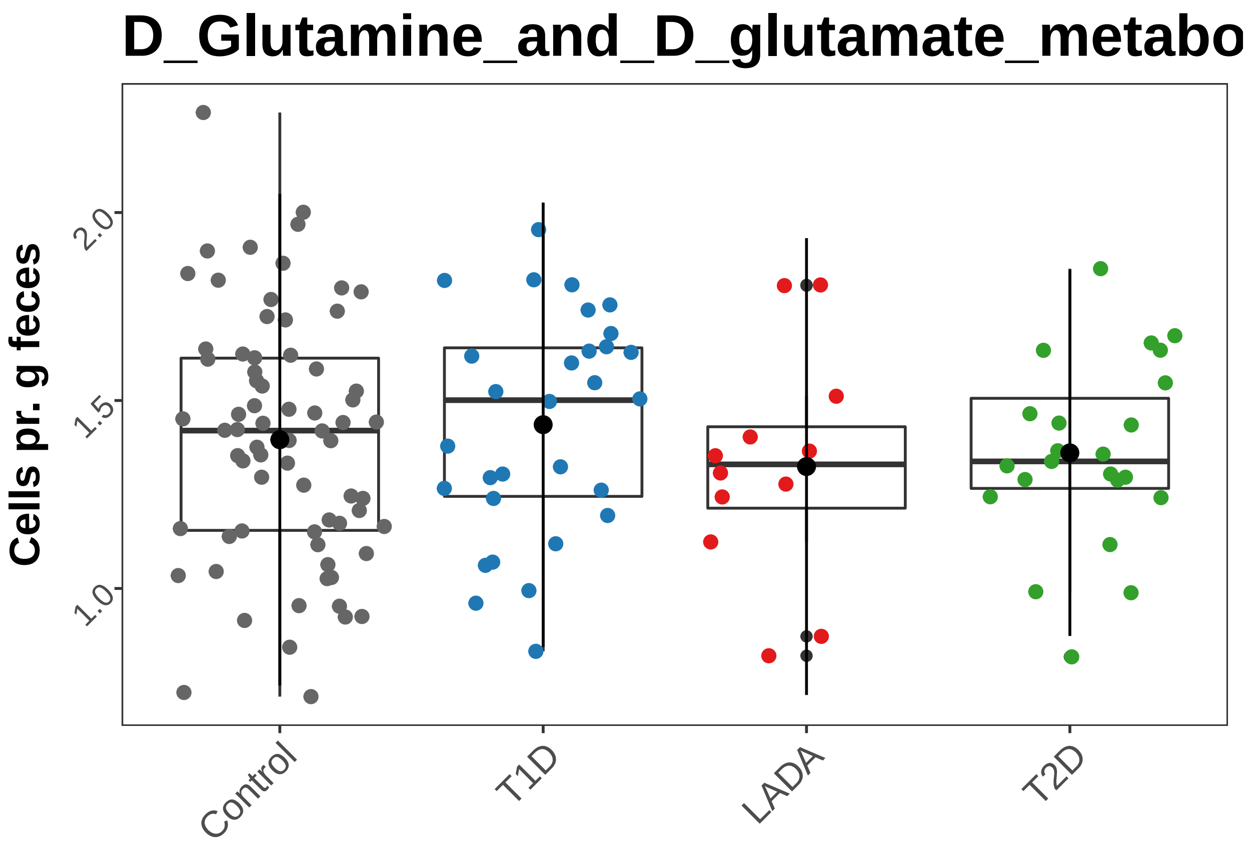

	pvalue	padj	Genus	compare
484	0.0085968	0.0346055	Glycolysis_____Gluconeogenesis_PATH_ko00010	LADA vs Control
288	0.0093897	0.0629443	Glycolysis_____Gluconeogenesis_PATH_ko00010	Control vs T2D
876	0.0696239	0.2312928	Glycolysis_____Gluconeogenesis_PATH_ko00010	LADA vs T1D
92	0.3563381	0.5414362	Glycolysis_____Gluconeogenesis_PATH_ko00010	Control vs T1D
680	0.1269311	0.6495082	Glycolysis_____Gluconeogenesis_PATH_ko00010	T1D vs T2D
1072	0.5806969	0.8682081	Glycolysis_____Gluconeogenesis_PATH_ko00010	LADA vs T2D

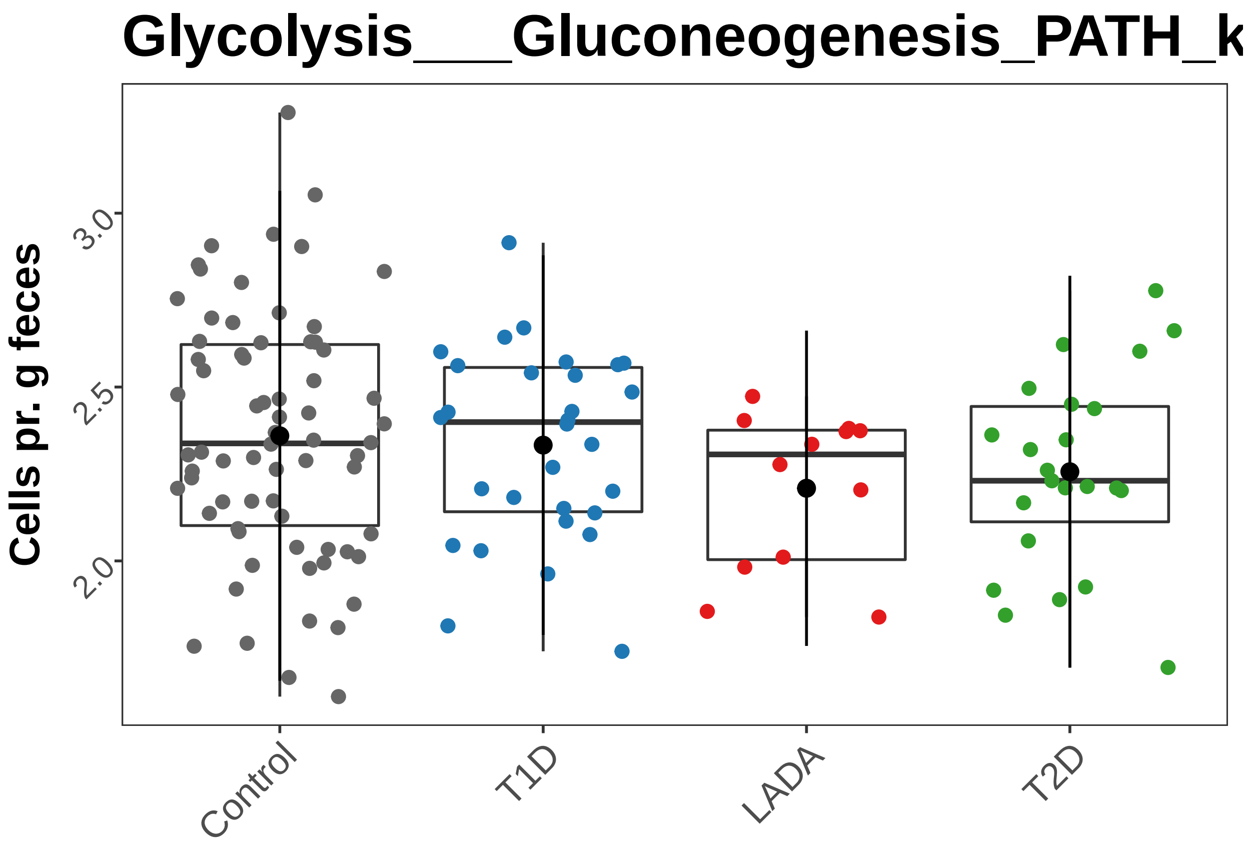

	pvalue	padj	Genus	compare
406	0.0086998	0.0346055	Quorum__sensing__PATH__ko02024	LADA vs Control
210	0.0339407	0.0717977	Quorum__sensing__PATH__ko02024	Control vs T2D
798	0.2318049	0.3940555	Quorum__sensing__PATH__ko02024	LADA vs T1D
14	0.0594483	0.3992571	Quorum__sensing__PATH__ko02024	Control vs T1D
602	0.7225598	0.8046688	Quorum__sensing__PATH__ko02024	T1D vs T2D
994	0.3839847	0.8415375	Quorum__sensing__PATH__ko02024	LADA vs T2D

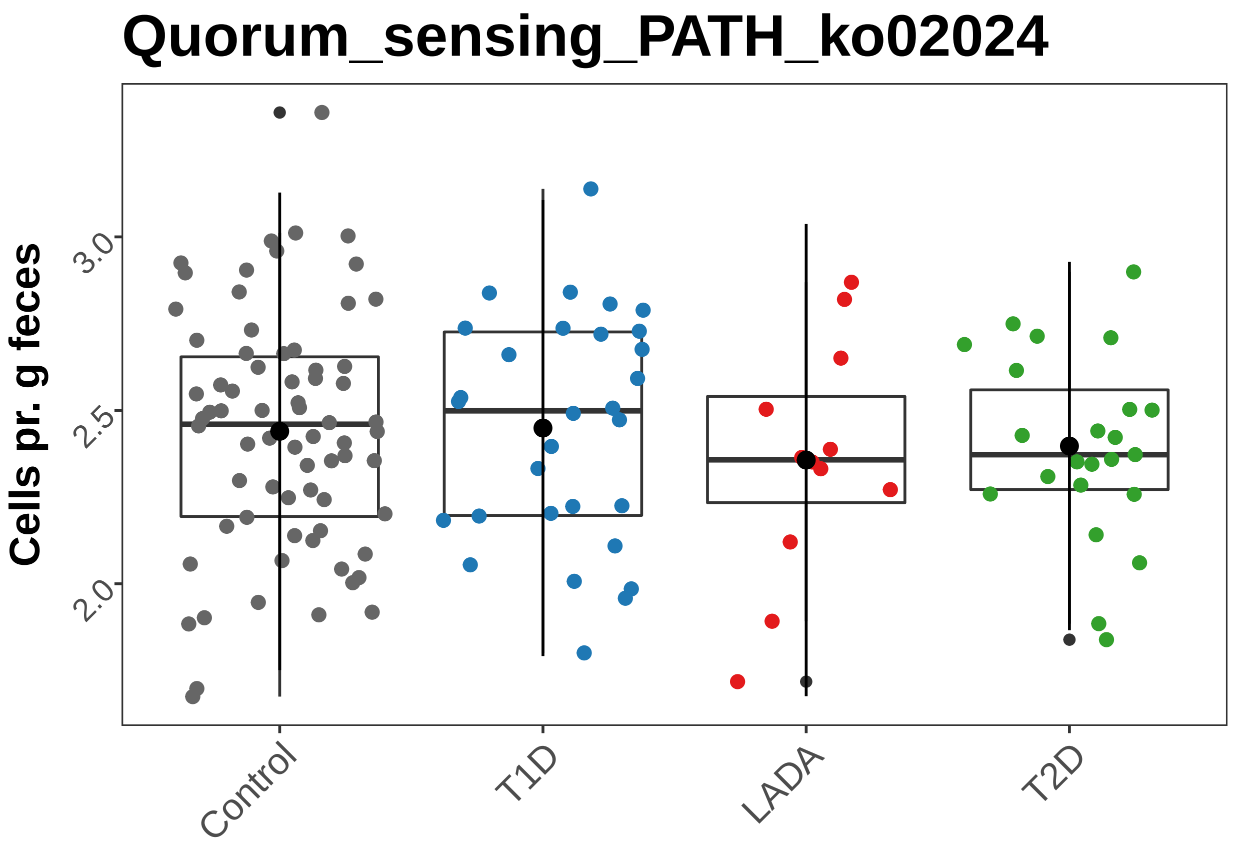

	pvalue	padj	Genus	compare
548	0.0092733	0.0346055	Biosynthesis_of_ansamycins_PATH_ko01051	LADA vs Control
352	0.0058911	0.0629443	Biosynthesis_of_ansamycins_PATH_ko01051	Control vs T2D
156	0.0051576	0.1684814	Biosynthesis_of_ansamycins_PATH_ko01051	Control vs T1D
940	0.5531848	0.6765319	Biosynthesis_of_ansamycins_PATH_ko01051	LADA vs T1D
744	0.8526337	0.8876857	Biosynthesis_of_ansamycins_PATH_ko01051	T1D vs T2D
1136	0.6714134	0.8952178	Biosynthesis_of_ansamycins_PATH_ko01051	LADA vs T2D

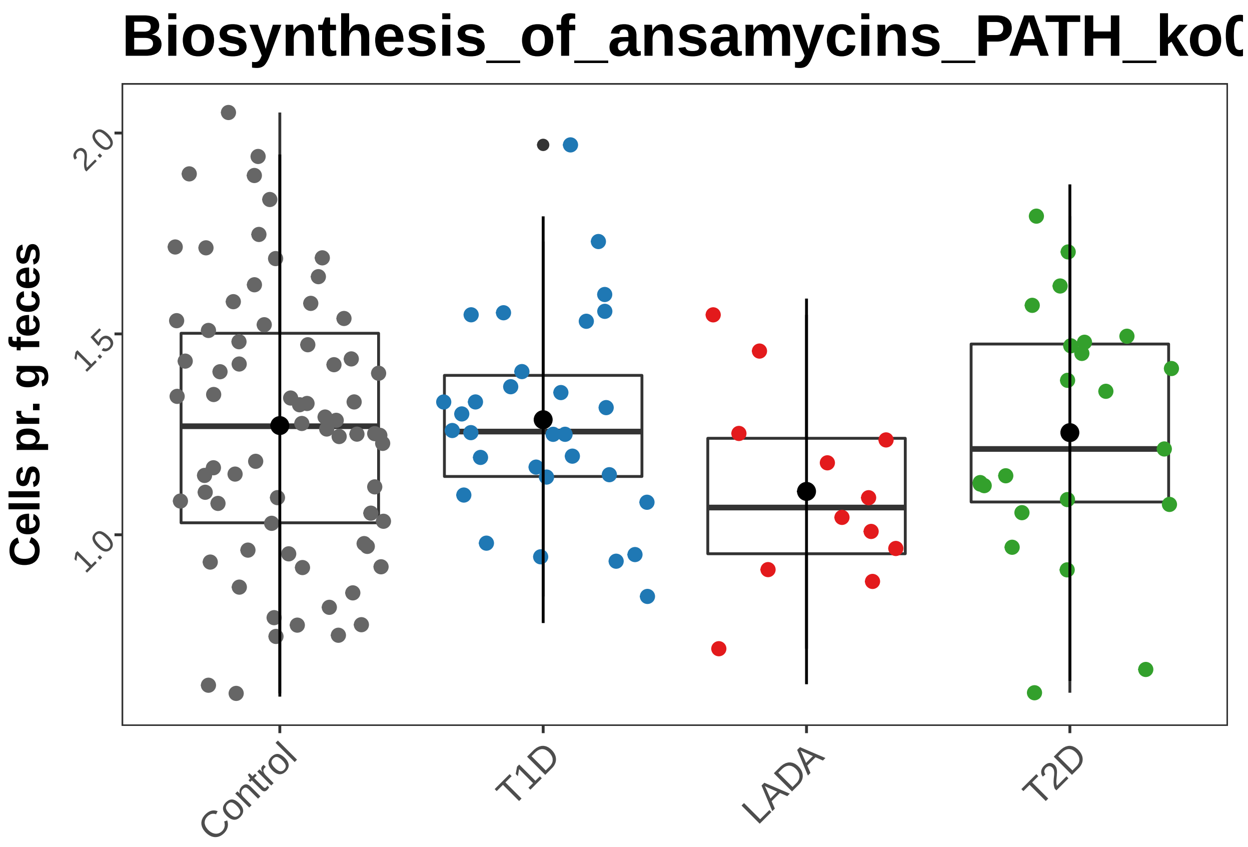

	pvalue	padj	Genus	compare
584	0.0093098	0.0346055	Carbon_metabolism	LADA vs Control
388	0.0474683	0.0828378	Carbon_metabolism	Control vs T2D
976	0.0263571	0.2312928	Carbon_metabolism	LADA vs T1D
780	0.1274183	0.6495082	Carbon_metabolism	T1D vs T2D
1172	0.3452479	0.8415375	Carbon_metabolism	LADA vs T2D
192	0.8048908	0.8764366	Carbon_metabolism	Control vs T1D

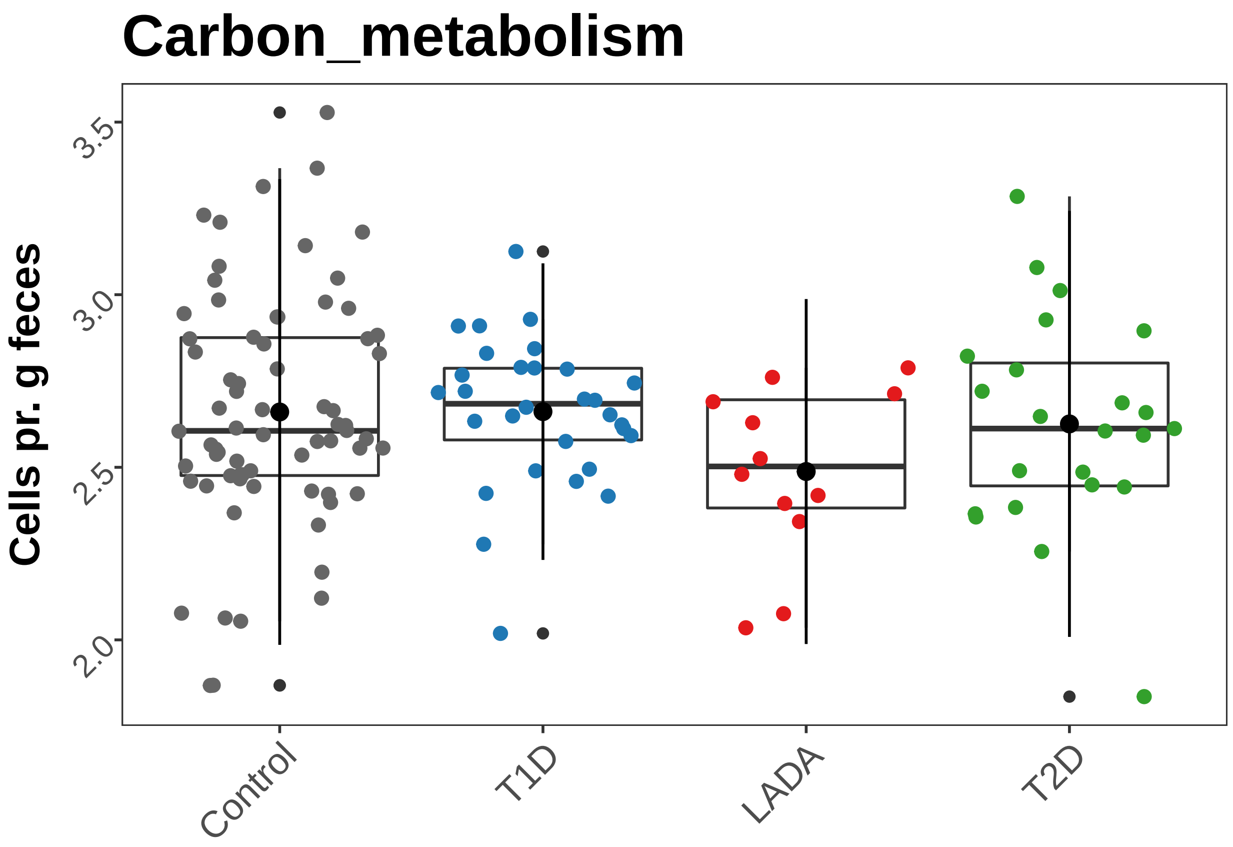

	pvalue	padj	Genus	compare
538	0.0093267	0.0346055	Vitamin_B6_metabolism_PATH_ko00750	LADA vs Control
342	0.0322314	0.0690674	Vitamin_B6_metabolism_PATH_ko00750	Control vs T2D
930	0.1277440	0.2813238	Vitamin_B6_metabolism_PATH_ko00750	LADA vs T1D
146	0.1807526	0.4762288	Vitamin_B6_metabolism_PATH_ko00750	Control vs T1D
734	0.4218191	0.6614124	Vitamin_B6_metabolism_PATH_ko00750	T1D vs T2D
1126	0.4033226	0.8415375	Vitamin_B6_metabolism_PATH_ko00750	LADA vs T2D

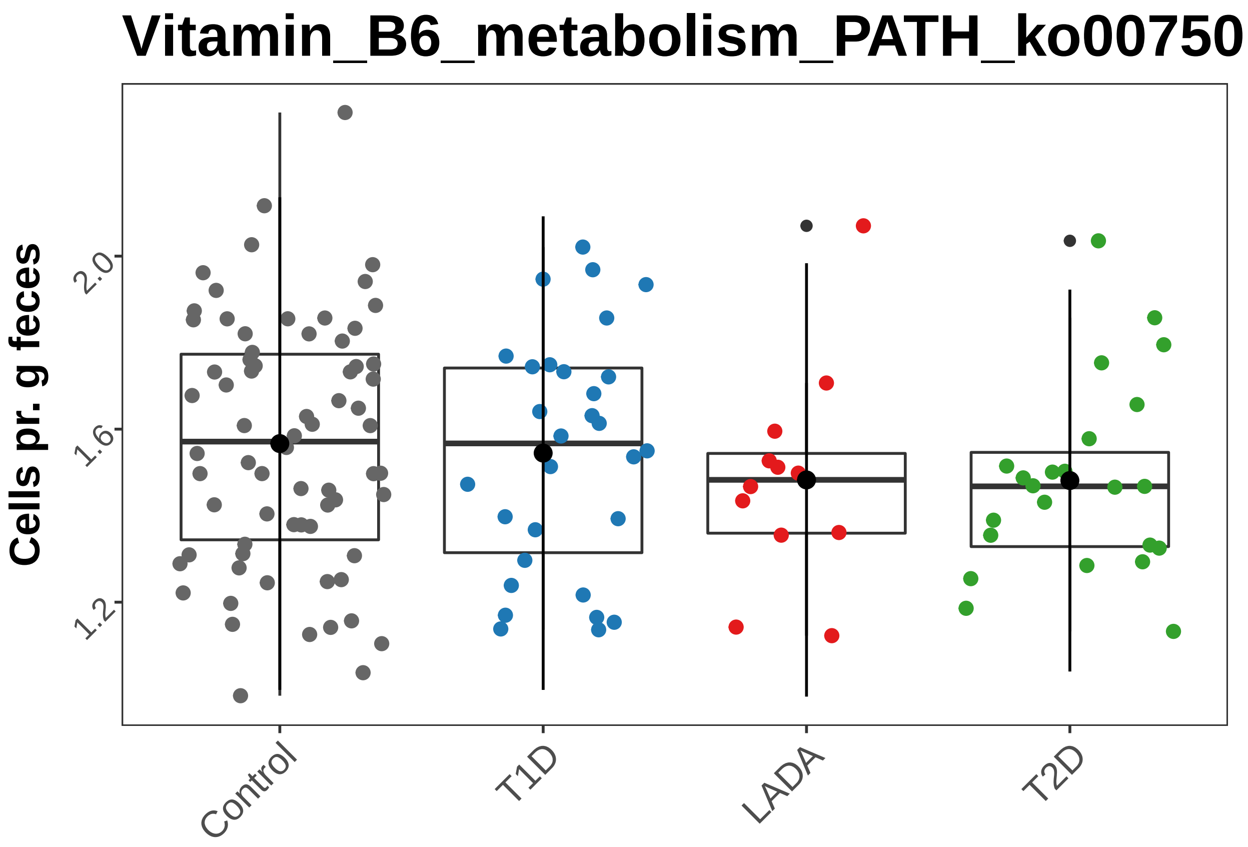

	pvalue	padj	Genus	compare
395	0.0093611	0.0346055	Cell_cycle____Caulobacter_PATH_ko04112	LADA vs Control
199	0.0236843	0.0676342	Cell_cycle____Caulobacter_PATH_ko04112	Control vs T2D
787	0.0969302	0.2458003	Cell_cycle____Caulobacter_PATH_ko04112	LADA vs T1D
3	0.2616893	0.4839499	Cell_cycle____Caulobacter_PATH_ko04112	Control vs T1D
591	0.2810224	0.6495082	Cell_cycle____Caulobacter_PATH_ko04112	T1D vs T2D
983	0.4513661	0.8415375	Cell_cycle____Caulobacter_PATH_ko04112	LADA vs T2D

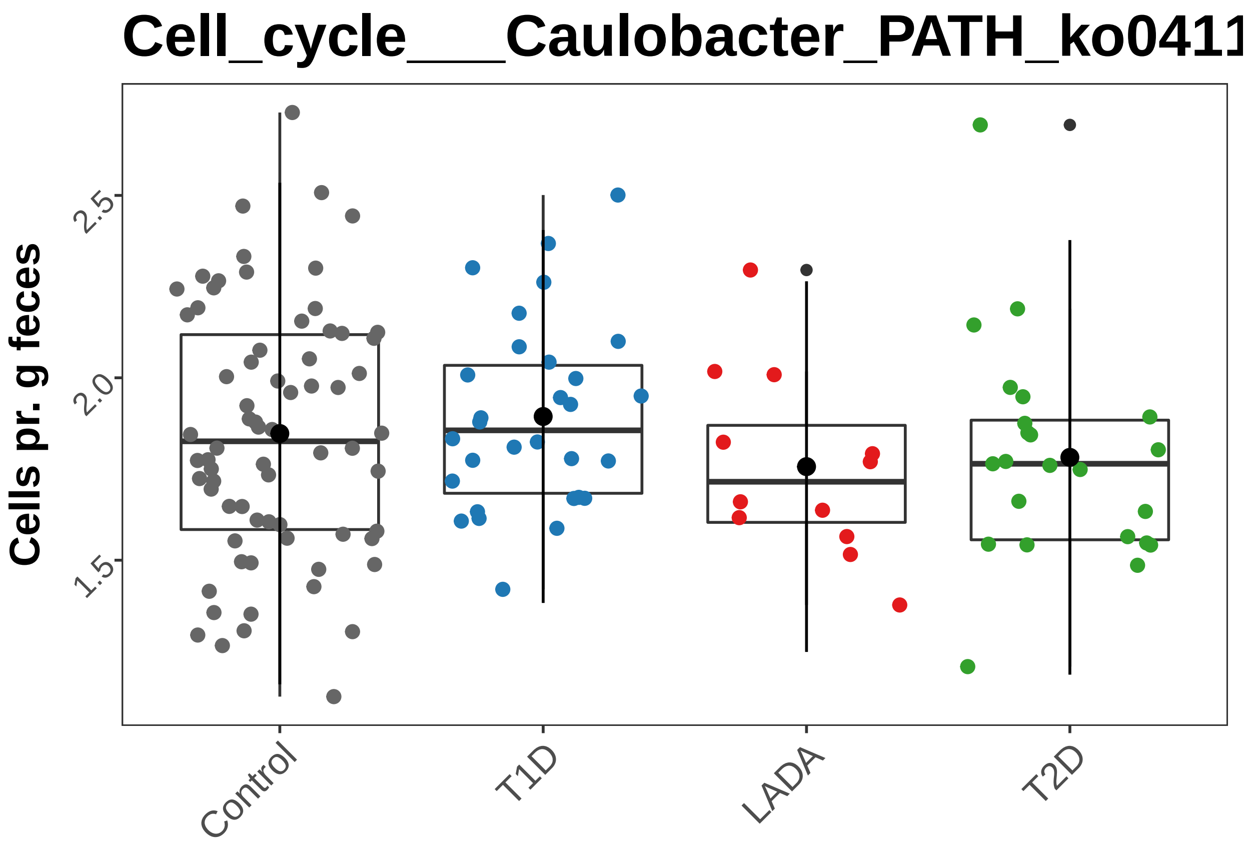

	pvalue	padj	Genus	compare
441	0.0094888	0.0346055	RNA_polymerase_PATH_ko03020	LADA vs Control
833	0.0019374	0.0632892	RNA_polymerase_PATH_ko03020	LADA vs T1D
245	0.1877779	0.2478669	RNA_polymerase_PATH_ko03020	Control vs T2D
49	0.2552029	0.4839499	RNA_polymerase_PATH_ko03020	Control vs T1D
637	0.0415381	0.6495082	RNA_polymerase_PATH_ko03020	T1D vs T2D
1029	0.1654793	0.8415375	RNA_polymerase_PATH_ko03020	LADA vs T2D

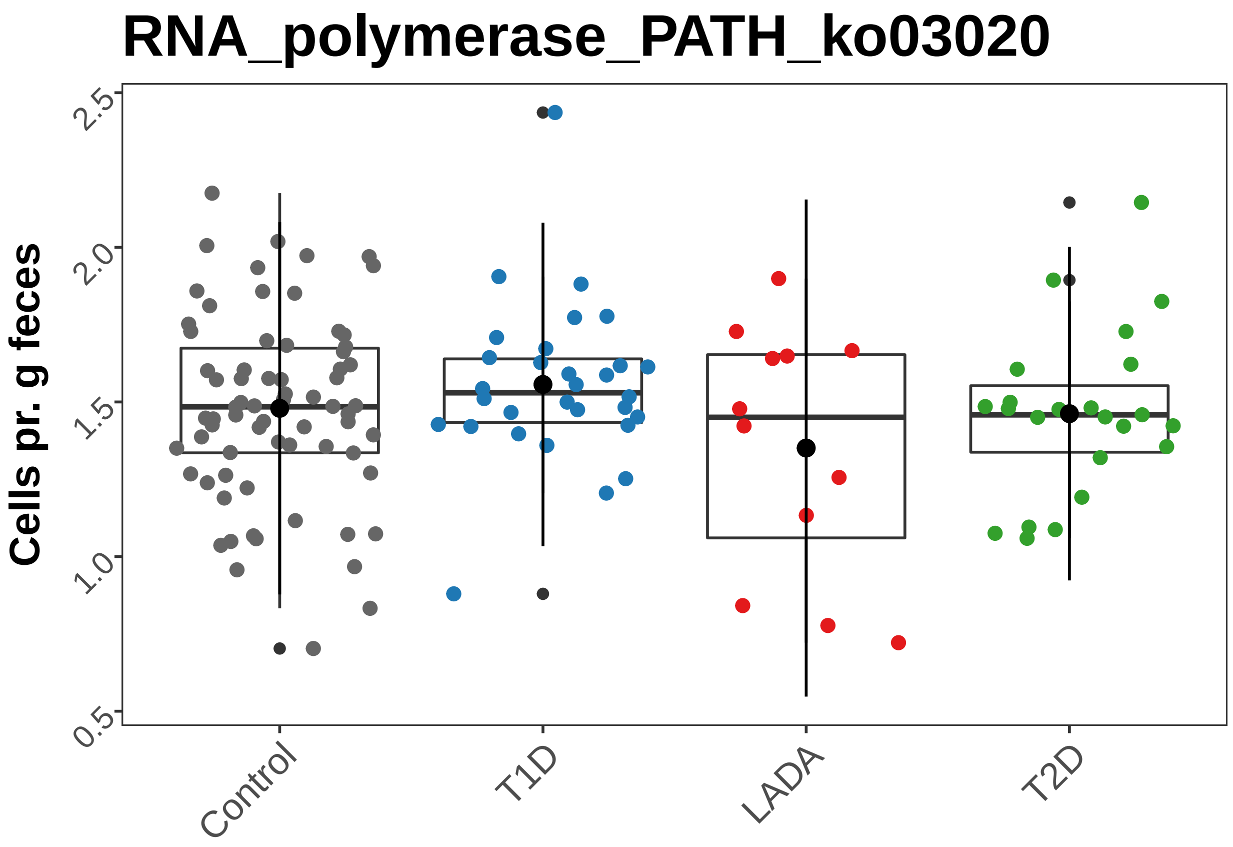

	pvalue	padj	Genus	compare
431	0.0095968	0.0346055	RNA_degradation_PATH_ko03018	LADA vs Control
235	0.0299230	0.0676342	RNA_degradation_PATH_ko03018	Control vs T2D
823	0.0747378	0.2321553	RNA_degradation_PATH_ko03018	LADA vs T1D
39	0.3581075	0.5414362	RNA_degradation_PATH_ko03018	Control vs T1D
627	0.2463768	0.6495082	RNA_degradation_PATH_ko03018	T1D vs T2D
1019	0.4195931	0.8415375	RNA_degradation_PATH_ko03018	LADA vs T2D

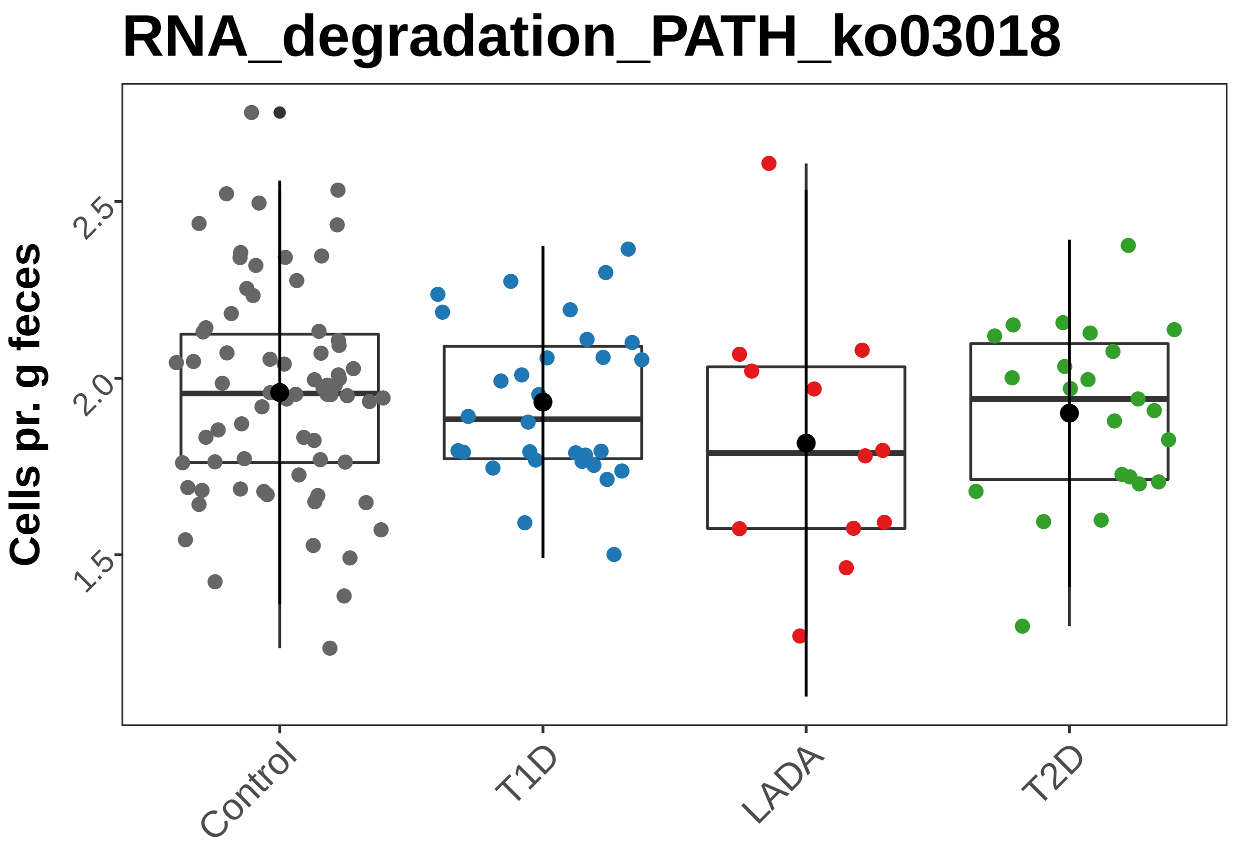

	pvalue	padj	Genus	compare
530	0.0098505	0.0346055	Nicotinate_and_nicotinamide_metabolism_PATH_ko00760	LADA vs Control
334	0.0295520	0.0676342	Nicotinate_and_nicotinamide_metabolism_PATH_ko00760	Control vs T2D
922	0.1137191	0.2685416	Nicotinate_and_nicotinamide_metabolism_PATH_ko00760	LADA vs T1D
138	0.2225672	0.4786856	Nicotinate_and_nicotinamide_metabolism_PATH_ko00760	Control vs T1D
726	0.3540970	0.6495082	Nicotinate_and_nicotinamide_metabolism_PATH_ko00760	T1D vs T2D
1118	0.4260888	0.8415375	Nicotinate_and_nicotinamide_metabolism_PATH_ko00760	LADA vs T2D

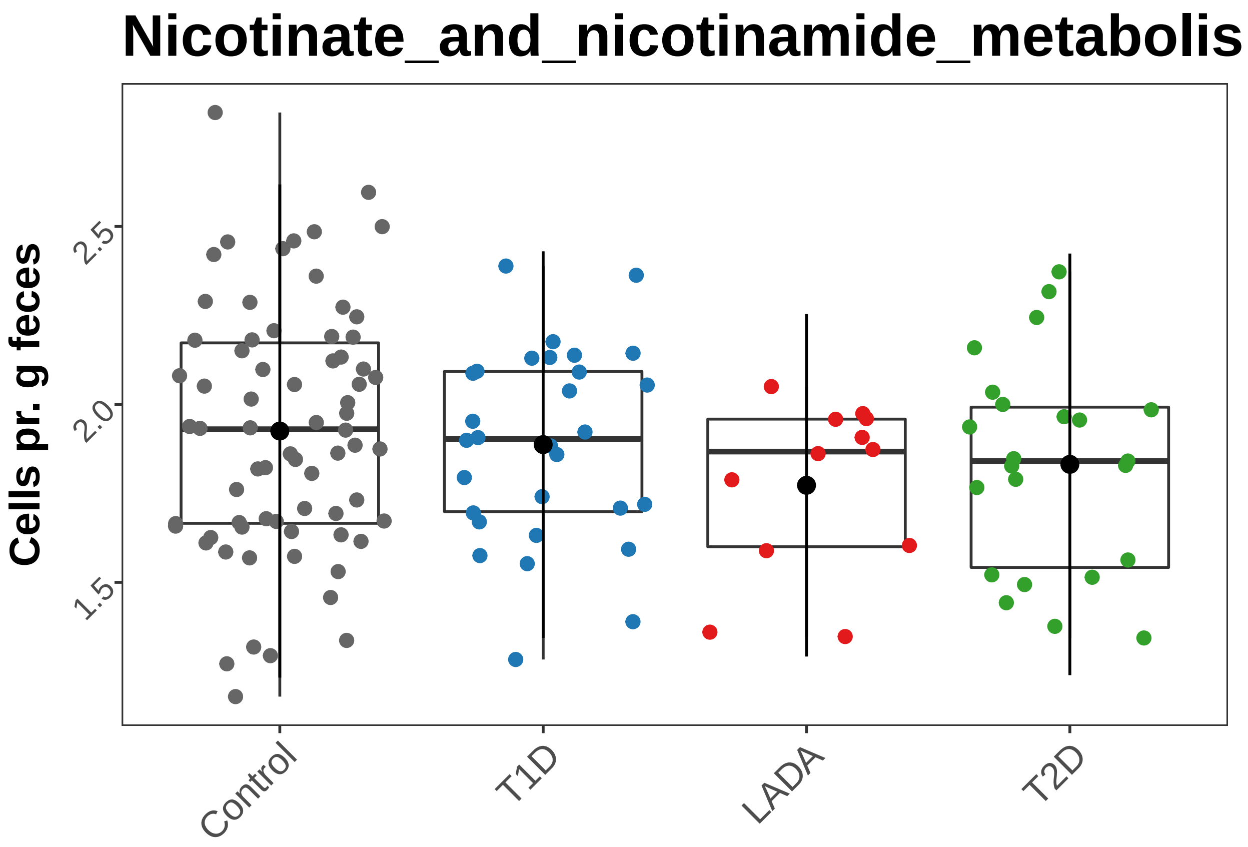

	pvalue	padj	Genus	compare
436	0.0098873	0.0346055	Homologous_recombination_PATH_ko03440	LADA vs Control
240	0.0168177	0.0629443	Homologous_recombination_PATH_ko03440	Control vs T2D
828	0.2444332	0.3950690	Homologous_recombination_PATH_ko03440	LADA vs T1D
44	0.0612454	0.3992571	Homologous_recombination_PATH_ko03440	Control vs T1D
632	0.5488814	0.7077681	Homologous_recombination_PATH_ko03440	T1D vs T2D
1024	0.5157439	0.8415375	Homologous_recombination_PATH_ko03440	LADA vs T2D

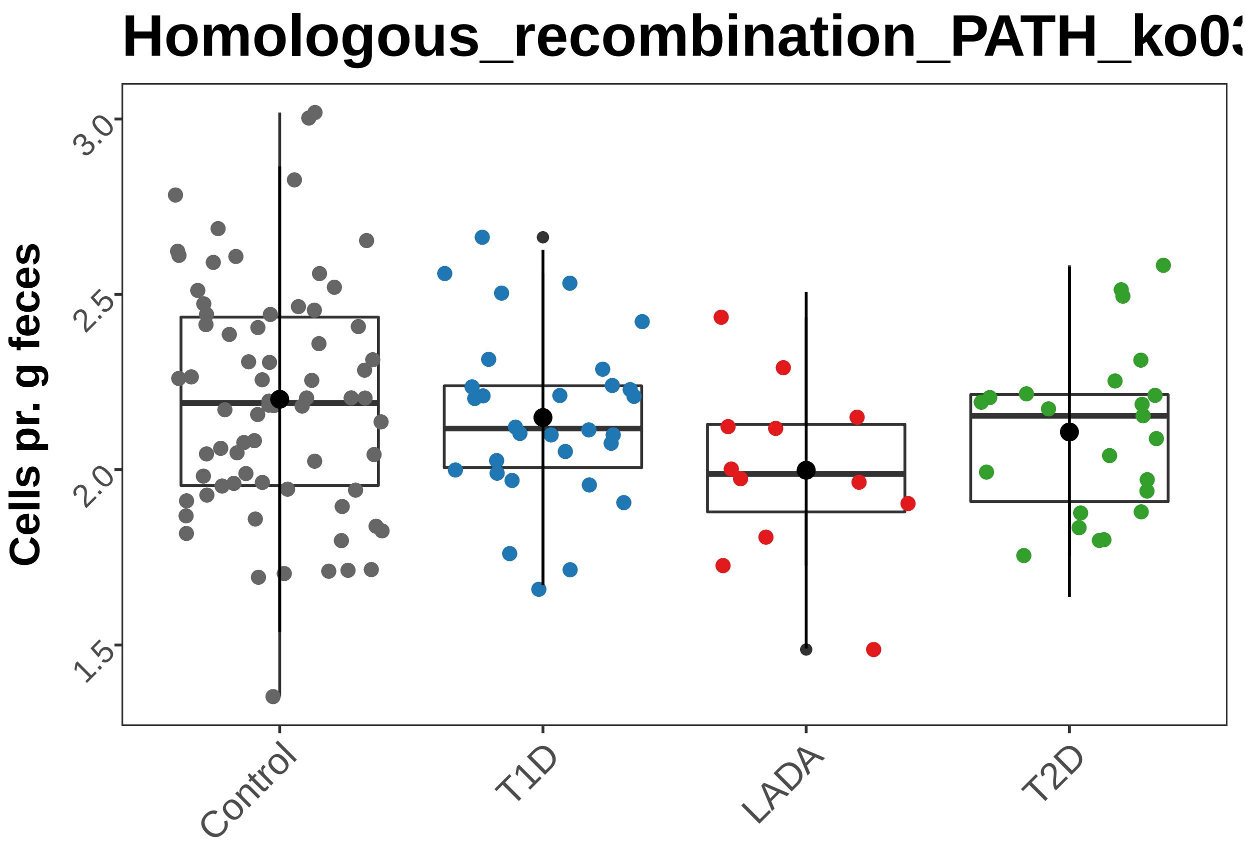

	pvalue	padj	Genus	compare
854	0.0004749	0.0358391	Betalain_biosynthesis_PATH_ko00965	LADA vs T1D
462	0.0151820	0.0437599	Betalain_biosynthesis_PATH_ko00965	LADA vs Control
70	0.0463003	0.3992571	Betalain_biosynthesis_PATH_ko00965	Control vs T1D
658	0.1588008	0.6495082	Betalain_biosynthesis_PATH_ko00965	T1D vs T2D
1050	0.0241387	0.7163761	Betalain_biosynthesis_PATH_ko00965	LADA vs T2D
266	0.8531983	NA	Betalain_biosynthesis_PATH_ko00965	Control vs T2D

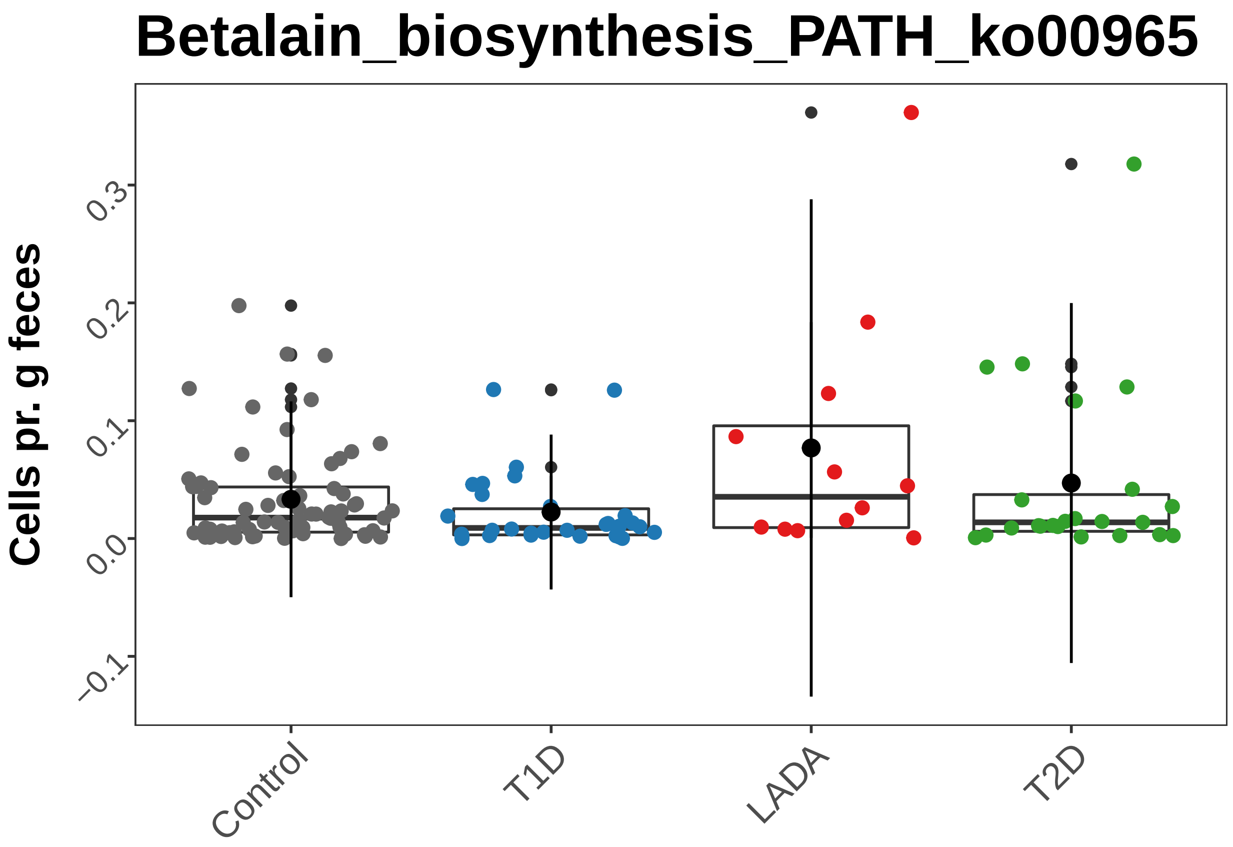

	pvalue	padj	Genus	compare
837	0.0005486	0.0358391	Ribosome_biogenesis_in_eukaryotes_PATH_ko03008	LADA vs T1D
445	0.0252415	0.0574628	Ribosome_biogenesis_in_eukaryotes_PATH_ko03008	LADA vs Control
53	0.0274263	0.3732360	Ribosome_biogenesis_in_eukaryotes_PATH_ko03008	Control vs T1D
641	0.0709048	0.6495082	Ribosome_biogenesis_in_eukaryotes_PATH_ko03008	T1D vs T2D
1033	0.0562470	0.8415375	Ribosome_biogenesis_in_eukaryotes_PATH_ko03008	LADA vs T2D
249	0.9360017	0.9474864	Ribosome_biogenesis_in_eukaryotes_PATH_ko03008	Control vs T2D

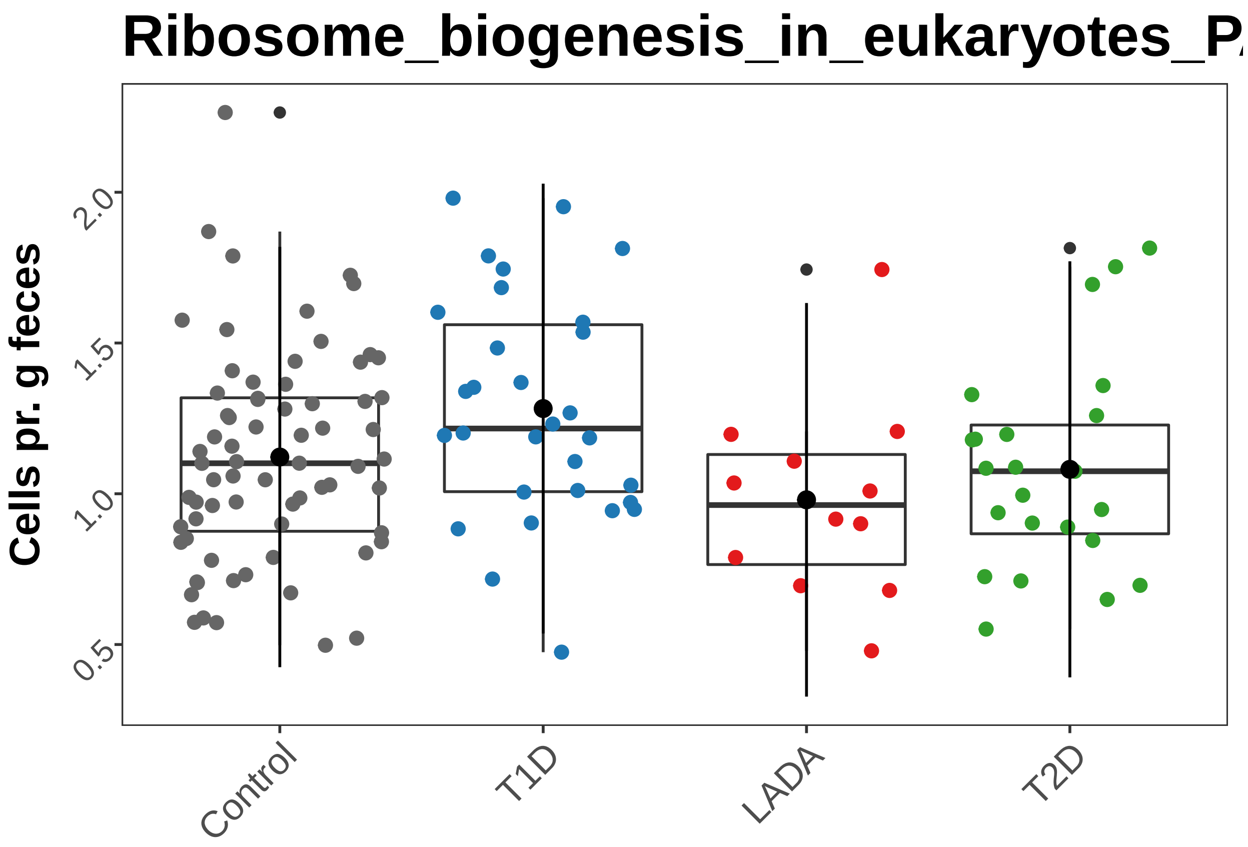

	pvalue	padj	Genus	compare
432	0.0107440	0.0369444	Sulfur_relay_system_PATH_ko04122	LADA vs Control
236	0.0039485	0.0629443	Sulfur_relay_system_PATH_ko04122	Control vs T2D
824	0.2403419	0.3950690	Sulfur_relay_system_PATH_ko04122	LADA vs T1D
40	0.0695942	0.3992571	Sulfur_relay_system_PATH_ko04122	Control vs T1D
628	0.2842821	0.6495082	Sulfur_relay_system_PATH_ko04122	T1D vs T2D
1020	0.7696384	0.9035121	Sulfur_relay_system_PATH_ko04122	LADA vs T2D

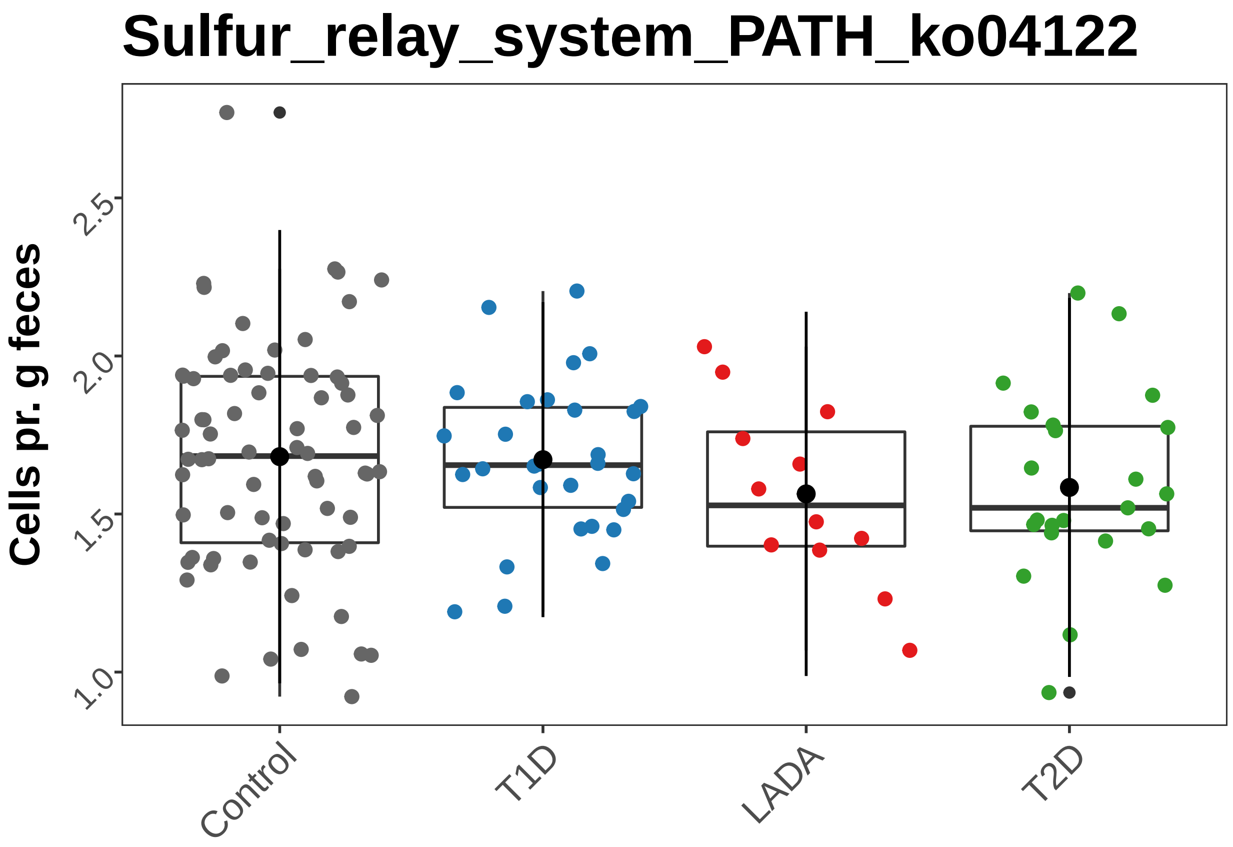

	pvalue	padj	Genus	compare
486	0.0109597	0.0369488	Inositol_phosphate_metabolism_PATH_ko00562	LADA vs Control
290	0.0725466	0.1129262	Inositol_phosphate_metabolism_PATH_ko00562	Control vs T2D
878	0.0256663	0.2312928	Inositol_phosphate_metabolism_PATH_ko00562	LADA vs T1D
682	0.1501277	0.6495082	Inositol_phosphate_metabolism_PATH_ko00562	T1D vs T2D
1074	0.3076733	0.8415375	Inositol_phosphate_metabolism_PATH_ko00562	LADA vs T2D
94	0.8807551	0.9133756	Inositol_phosphate_metabolism_PATH_ko00562	Control vs T1D

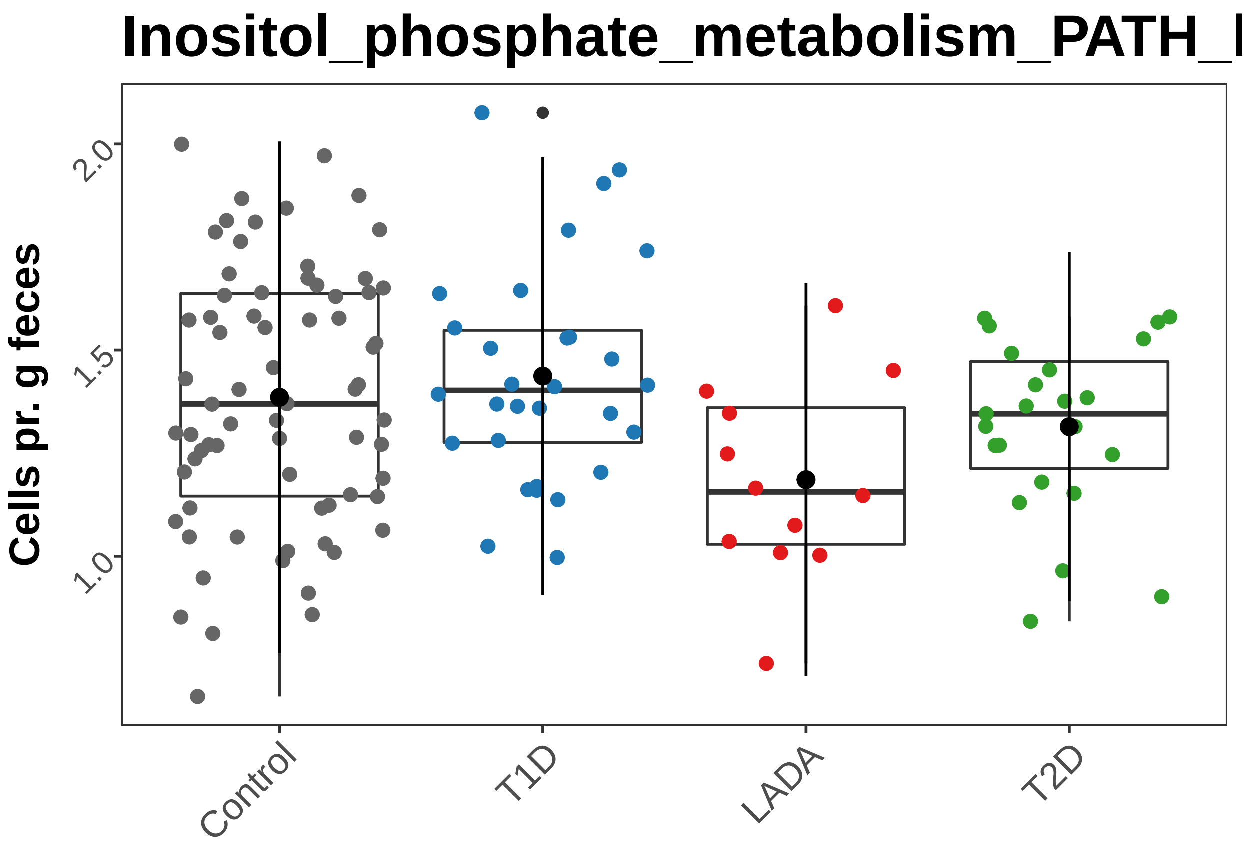

	pvalue	padj	Genus	compare
483	0.0111223	0.0369488	Galactose_metabolism_PATH_ko00052	LADA vs Control
287	0.0260671	0.0676342	Galactose_metabolism_PATH_ko00052	Control vs T2D
875	0.1527901	0.3130237	Galactose_metabolism_PATH_ko00052	LADA vs T1D
91	0.1623691	0.4612224	Galactose_metabolism_PATH_ko00052	Control vs T1D
679	0.4067555	0.6531957	Galactose_metabolism_PATH_ko00052	T1D vs T2D
1071	0.4680274	0.8415375	Galactose_metabolism_PATH_ko00052	LADA vs T2D

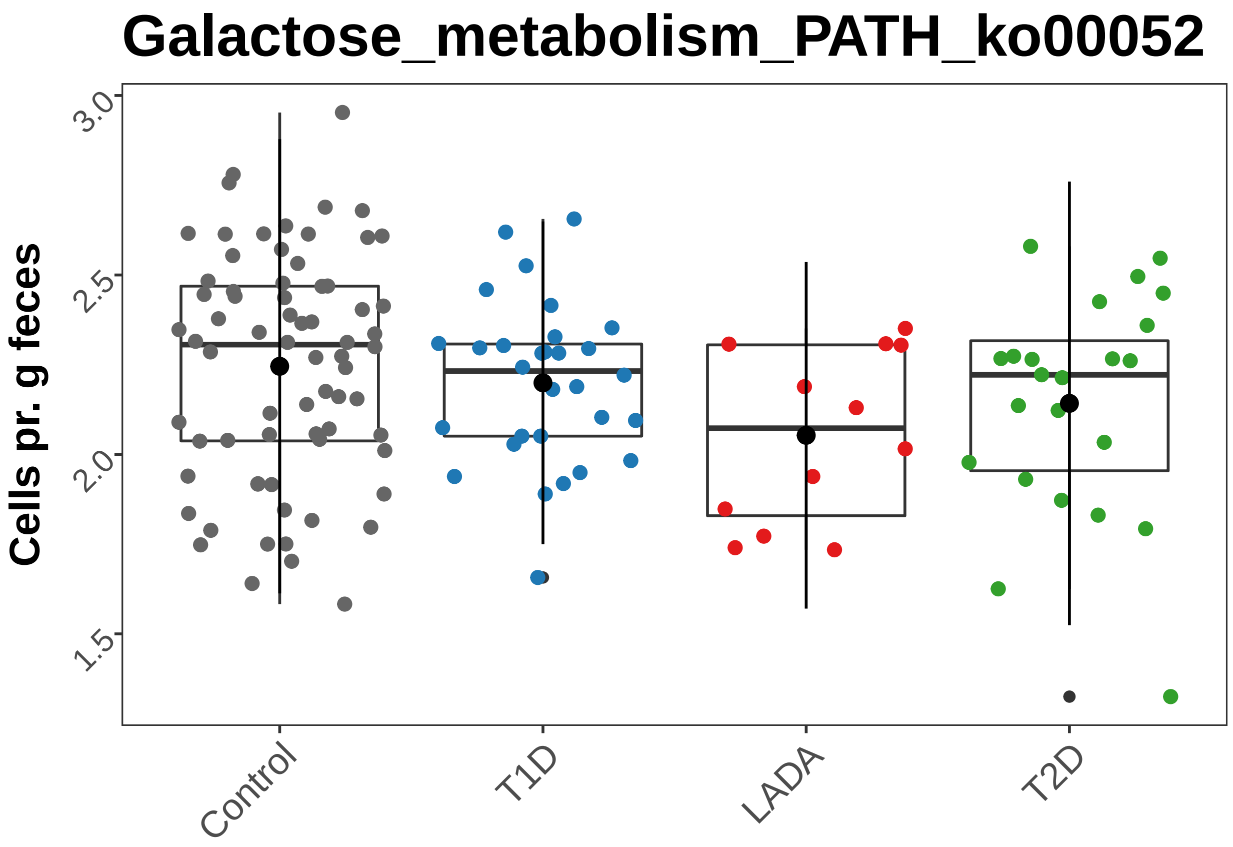

	pvalue	padj	Genus	compare
489	0.0116283	0.0379856	Propanoate_metabolism_PATH_ko00640	LADA vs Control
293	0.0292621	0.0676342	Propanoate_metabolism_PATH_ko00640	Control vs T2D
881	0.0500475	0.2312928	Propanoate_metabolism_PATH_ko00640	LADA vs T1D
685	0.1440432	0.6495082	Propanoate_metabolism_PATH_ko00640	T1D vs T2D
97	0.5853946	0.7171084	Propanoate_metabolism_PATH_ko00640	Control vs T1D
1077	0.4577488	0.8415375	Propanoate_metabolism_PATH_ko00640	LADA vs T2D

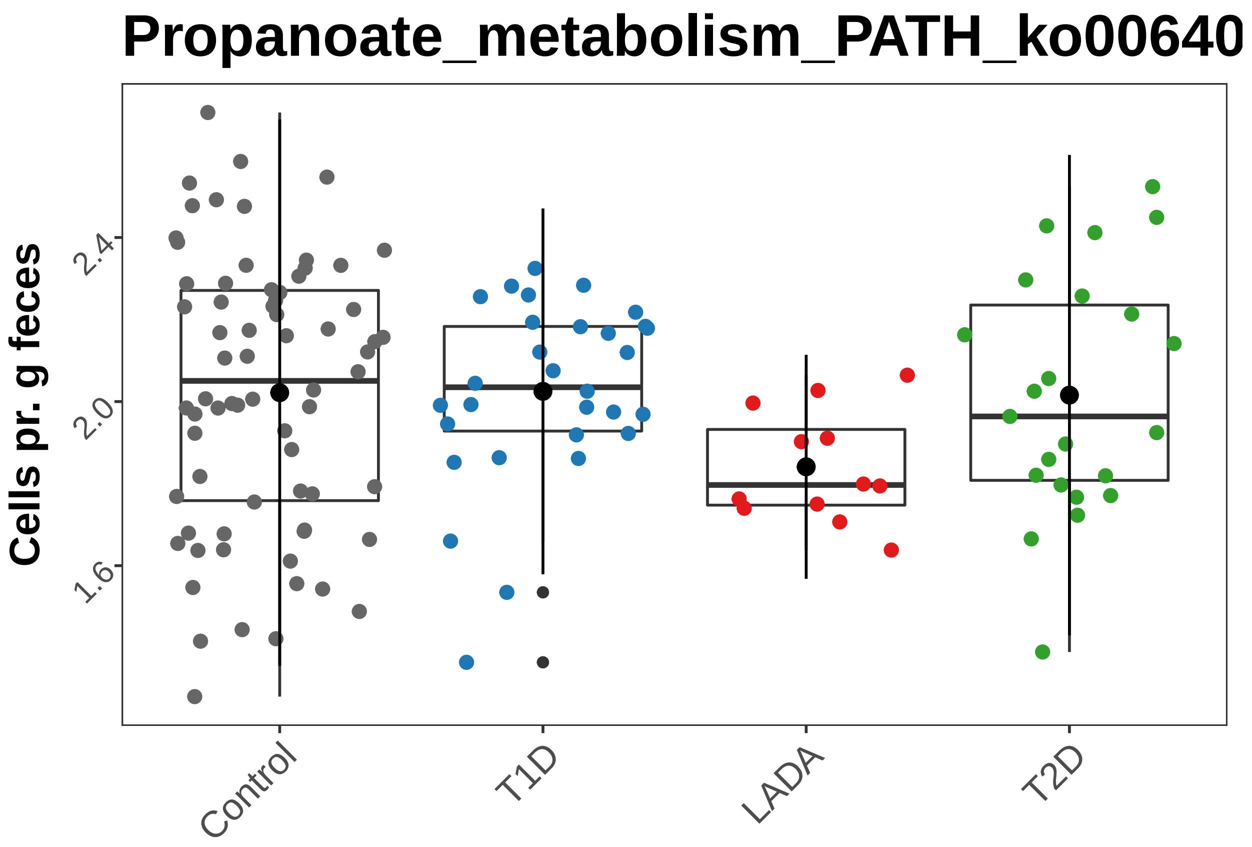

	pvalue	padj	Genus	compare
478	0.0121652	0.0386411	Ascorbate_and_aldarate_metabolism_PATH_ko00053	LADA vs Control
282	0.1373404	0.1888431	Ascorbate_and_aldarate_metabolism_PATH_ko00053	Control vs T2D
870	0.0407827	0.2312928	Ascorbate_and_aldarate_metabolism_PATH_ko00053	LADA vs T1D
674	0.3255773	0.6495082	Ascorbate_and_aldarate_metabolism_PATH_ko00053	T1D vs T2D
86	0.6981221	0.7863905	Ascorbate_and_aldarate_metabolism_PATH_ko00053	Control vs T1D
1066	0.2312647	0.8415375	Ascorbate_and_aldarate_metabolism_PATH_ko00053	LADA vs T2D

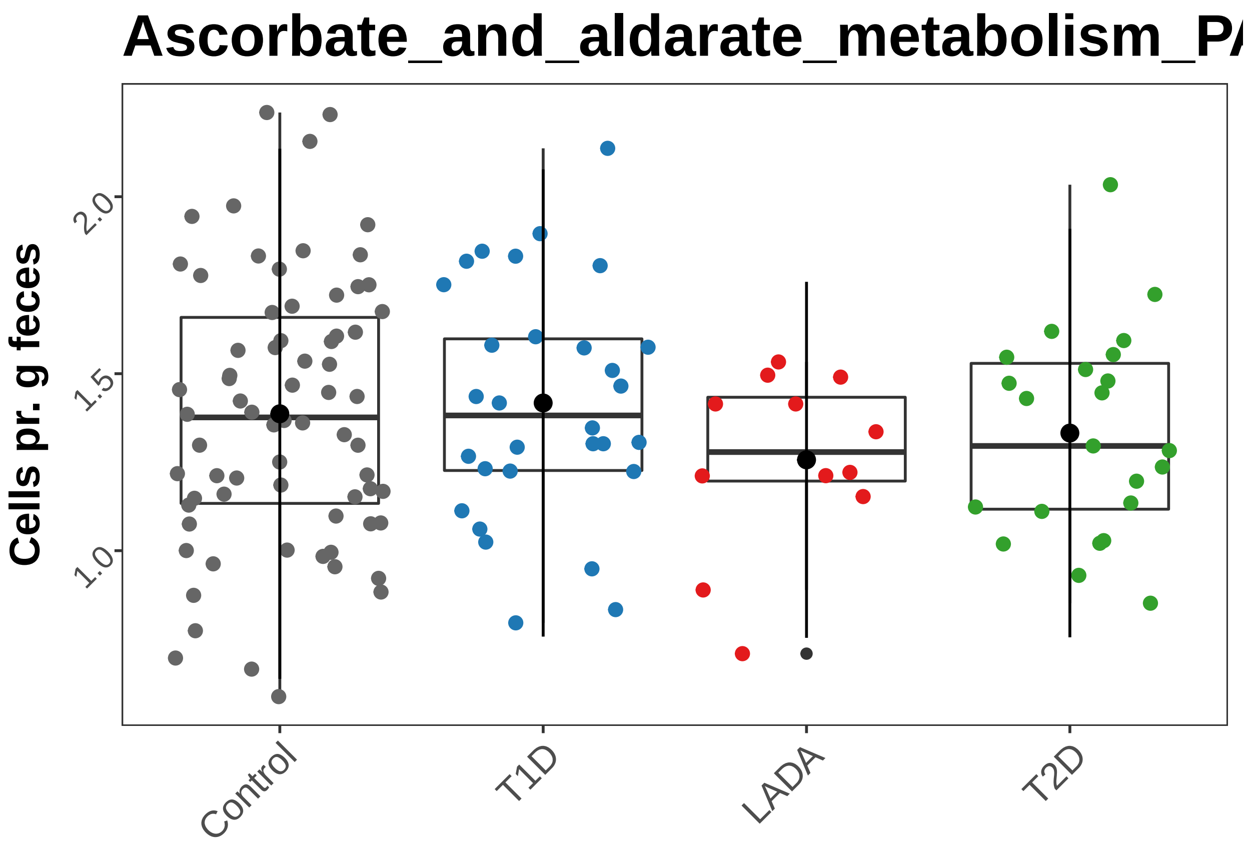

	pvalue	padj	Genus	compare
531	0.0122232	0.0386411	One_carbon_pool_by_folate_PATH_ko00670	LADA vs Control
335	0.0163756	0.0629443	One_carbon_pool_by_folate_PATH_ko00670	Control vs T2D
139	0.0672435	0.3992571	One_carbon_pool_by_folate_PATH_ko00670	Control vs T1D
923	0.2615182	0.4051834	One_carbon_pool_by_folate_PATH_ko00670	LADA vs T1D
727	0.5217040	0.6862683	One_carbon_pool_by_folate_PATH_ko00670	T1D vs T2D
1119	0.5630593	0.8682081	One_carbon_pool_by_folate_PATH_ko00670	LADA vs T2D

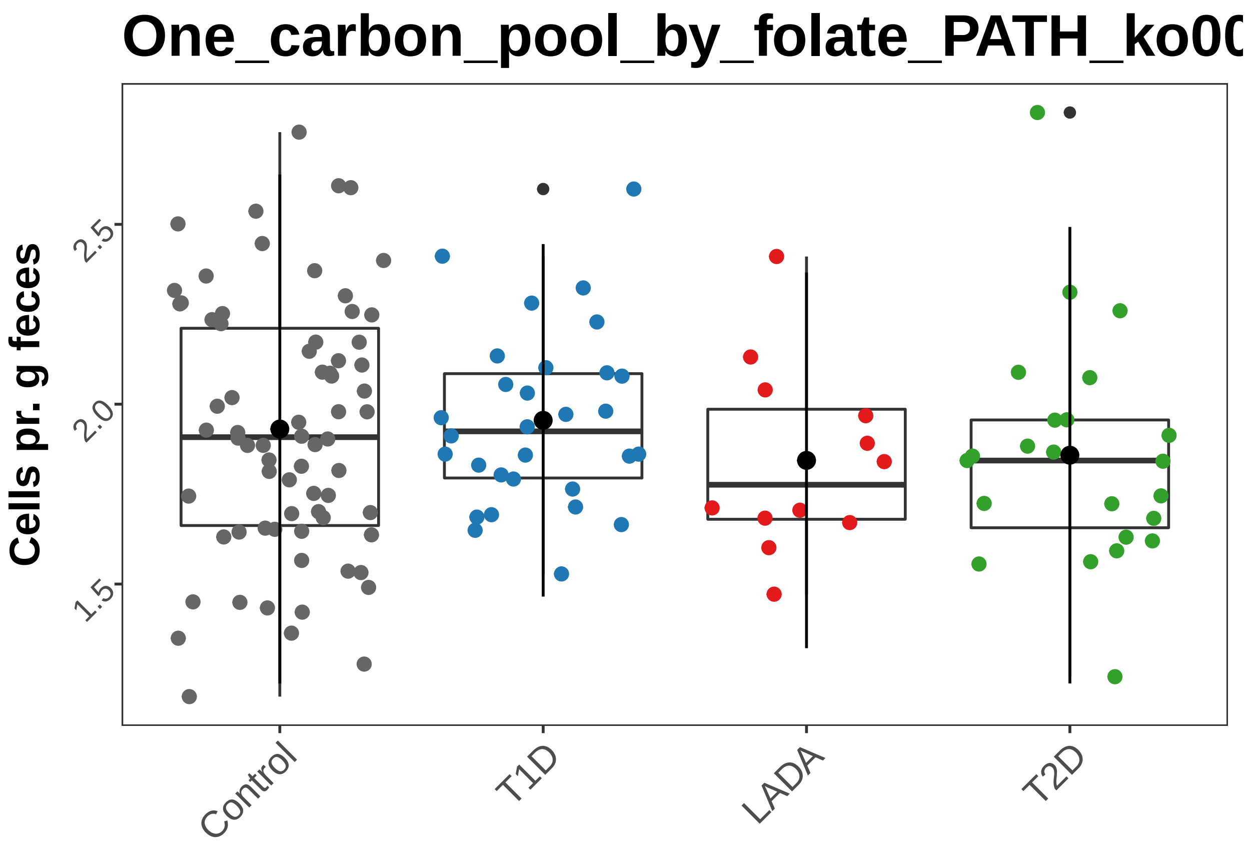

	pvalue	padj	Genus	compare
519	0.0125377	0.0390061	Glycerophospholipid_metabolism_PATH_ko00564	LADA vs Control
323	0.0052216	0.0629443	Glycerophospholipid_metabolism_PATH_ko00564	Control vs T2D
127	0.0346349	0.3989340	Glycerophospholipid_metabolism_PATH_ko00564	Control vs T1D
911	0.3502724	0.4939093	Glycerophospholipid_metabolism_PATH_ko00564	LADA vs T1D
715	0.4480294	0.6743371	Glycerophospholipid_metabolism_PATH_ko00564	T1D vs T2D
1107	0.7600330	0.9035121	Glycerophospholipid_metabolism_PATH_ko00564	LADA vs T2D

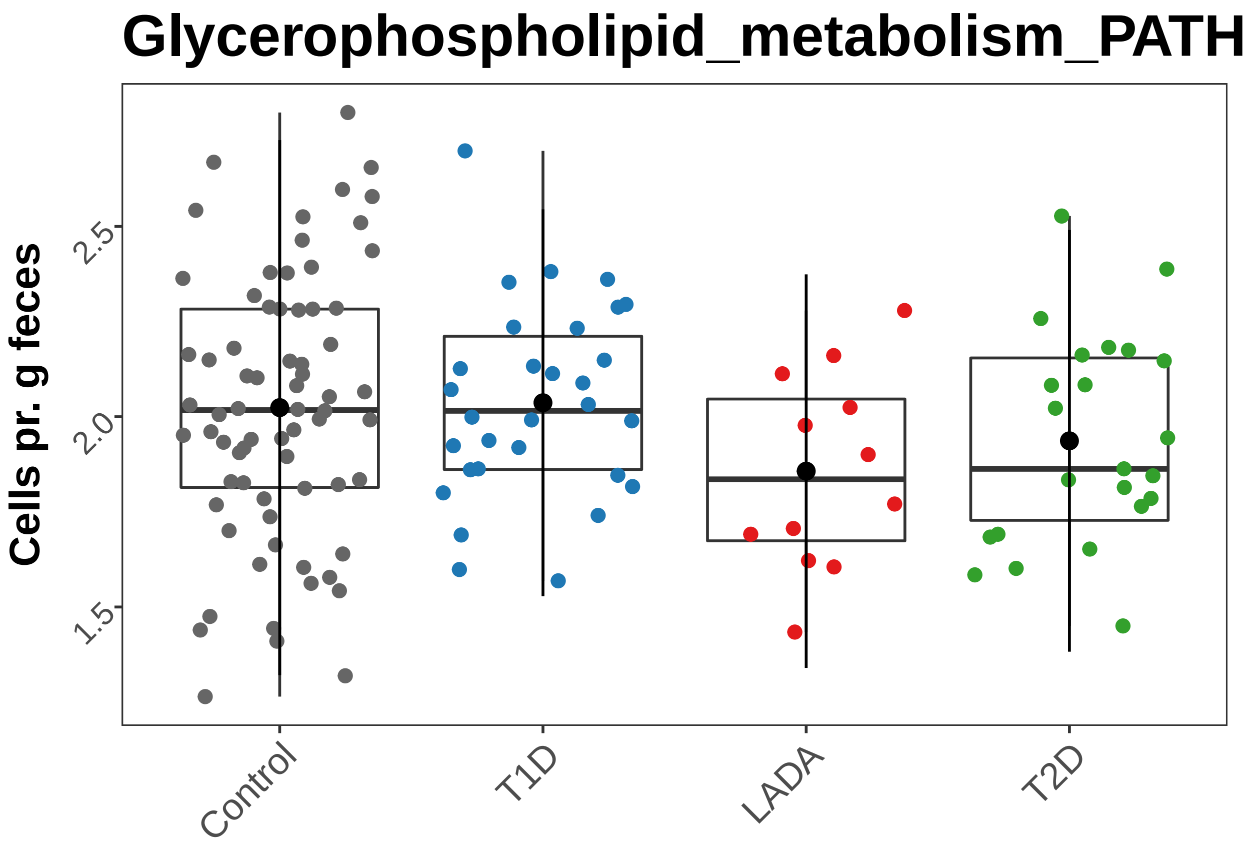

	pvalue	padj	Genus	compare
8	0.0002559	0.0390382	Bacterial_chemotaxis_PATH_ko02030	Control vs T1D
204	0.0174619	0.0629443	Bacterial_chemotaxis_PATH_ko02030	Control vs T2D
400	0.1339488	0.1888774	Bacterial_chemotaxis_PATH_ko02030	LADA vs Control
792	0.3345360	0.4786062	Bacterial_chemotaxis_PATH_ko02030	LADA vs T1D
596	0.4135351	0.6536523	Bacterial_chemotaxis_PATH_ko02030	T1D vs T2D
988	0.7724100	0.9035121	Bacterial_chemotaxis_PATH_ko02030	LADA vs T2D

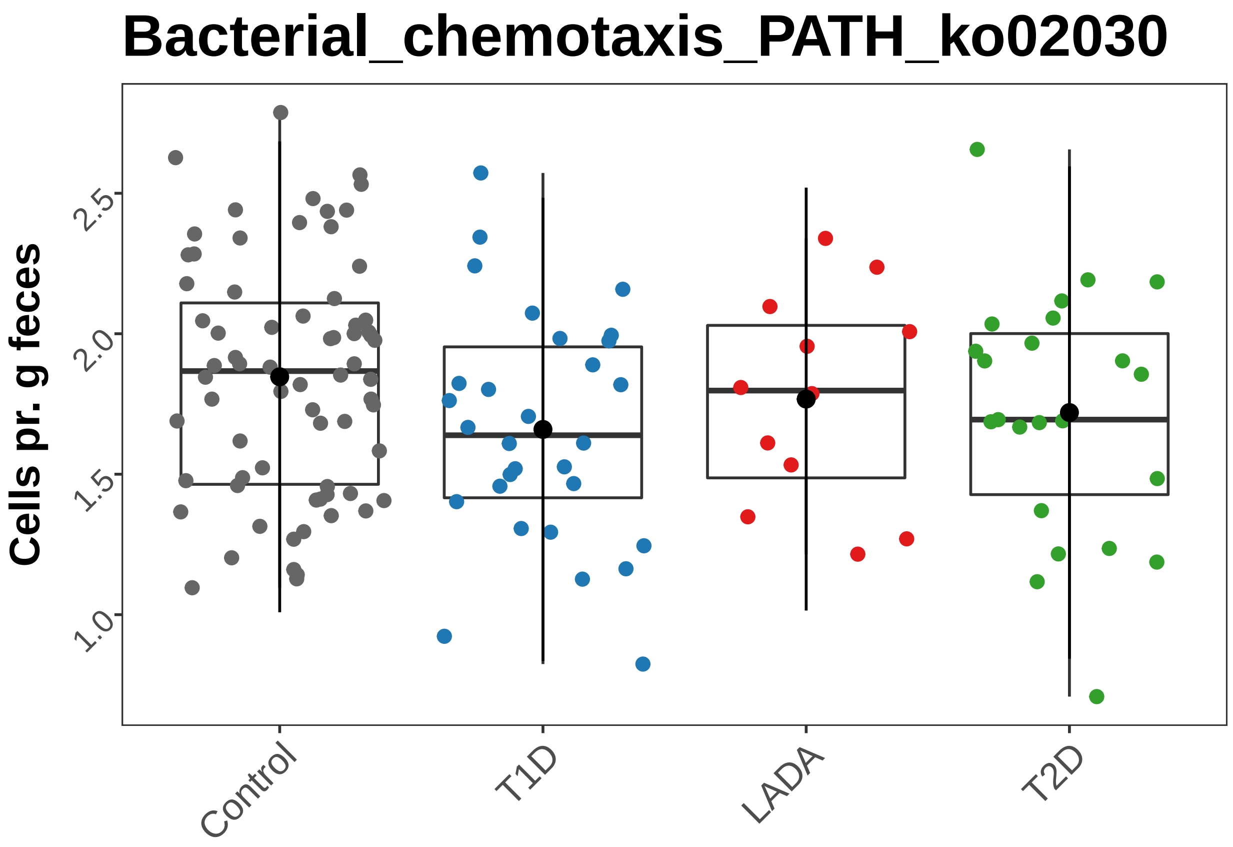

	pvalue	padj	Genus	compare
9	0.0003983	0.0390382	Flagellar_assembly_PATH_ko02040	Control vs T1D
205	0.0183278	0.0630017	Flagellar_assembly_PATH_ko02040	Control vs T2D
401	0.1637355	0.2244207	Flagellar_assembly_PATH_ko02040	LADA vs Control
793	0.3229047	0.4653626	Flagellar_assembly_PATH_ko02040	LADA vs T1D
597	0.4577468	0.6743371	Flagellar_assembly_PATH_ko02040	T1D vs T2D
989	0.7112165	0.9028767	Flagellar_assembly_PATH_ko02040	LADA vs T2D

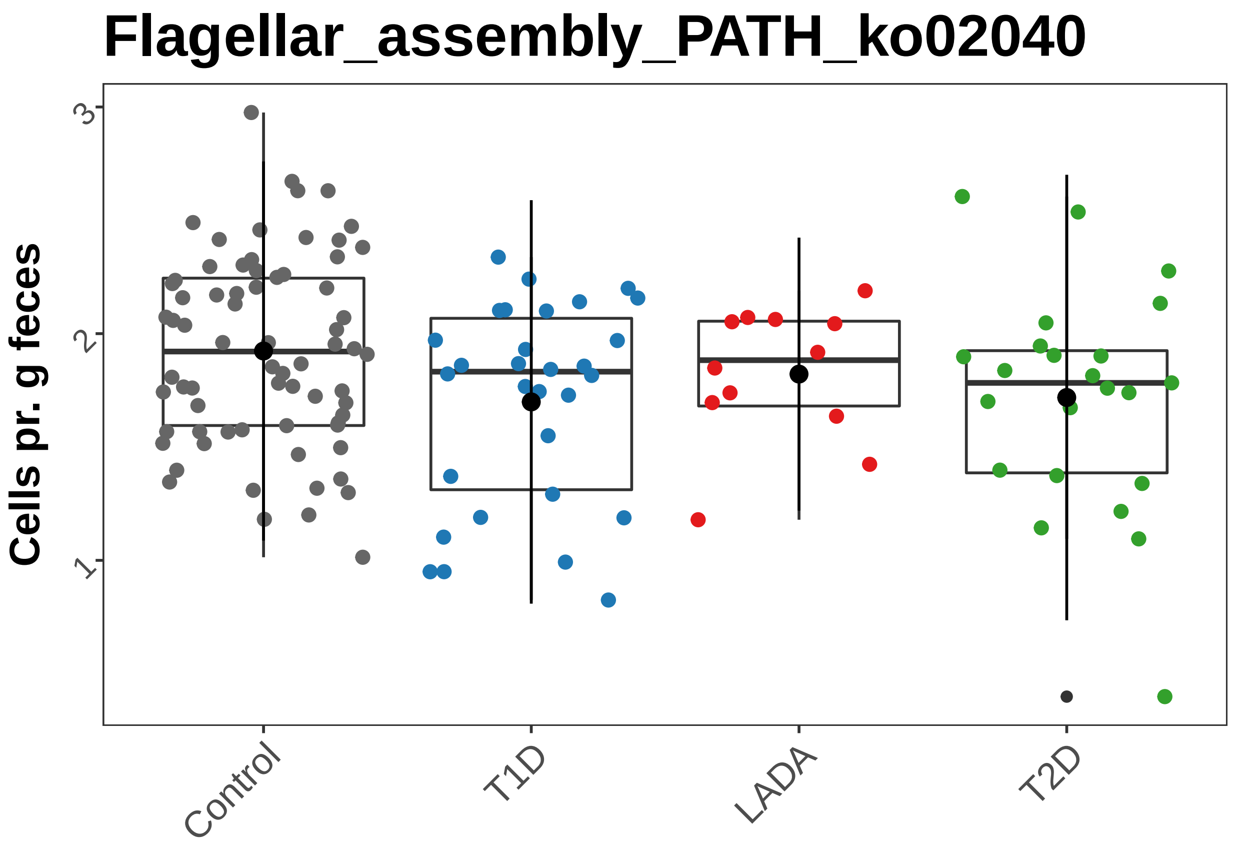

	pvalue	padj	Genus	compare
461	0.0127739	0.0391200	Acarbose_and_validamycin_biosynthesis_PATH_ko00525	LADA vs Control
265	0.1201192	0.1708584	Acarbose_and_validamycin_biosynthesis_PATH_ko00525	Control vs T2D
853	0.1098309	0.2625227	Acarbose_and_validamycin_biosynthesis_PATH_ko00525	LADA vs T1D
69	0.2880557	0.5040976	Acarbose_and_validamycin_biosynthesis_PATH_ko00525	Control vs T1D
657	0.6091039	0.7461523	Acarbose_and_validamycin_biosynthesis_PATH_ko00525	T1D vs T2D
1049	0.2560853	0.8415375	Acarbose_and_validamycin_biosynthesis_PATH_ko00525	LADA vs T2D

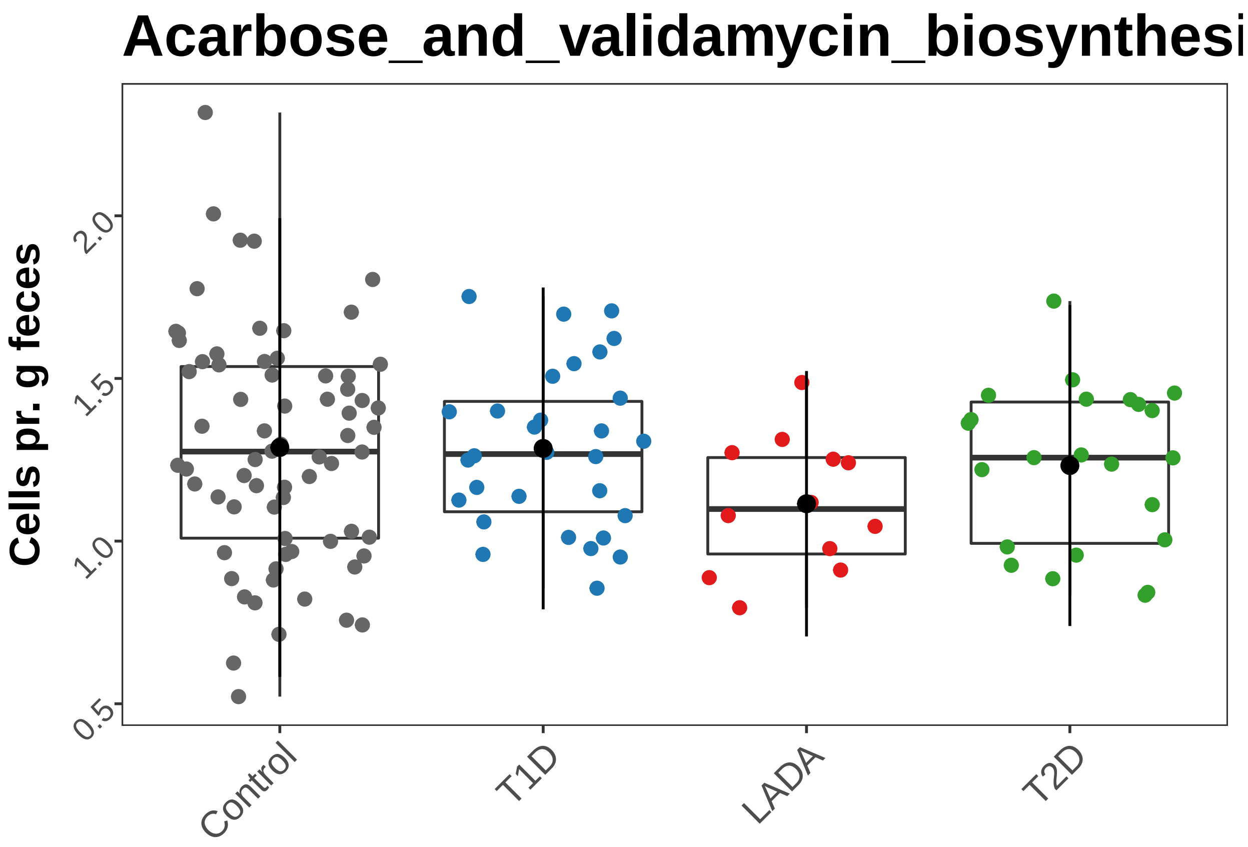

	pvalue	padj	Genus	compare
495	0.0136810	0.0408927	Nitrogen_metabolism_PATH_ko00910	LADA vs Control
299	0.0243965	0.0676342	Nitrogen_metabolism_PATH_ko00910	Control vs T2D
887	0.2621789	0.4051834	Nitrogen_metabolism_PATH_ko00910	LADA vs T1D
103	0.0758886	0.4131714	Nitrogen_metabolism_PATH_ko00910	Control vs T1D
691	0.5793903	0.7326484	Nitrogen_metabolism_PATH_ko00910	T1D vs T2D
1083	0.5195161	0.8415375	Nitrogen_metabolism_PATH_ko00910	LADA vs T2D

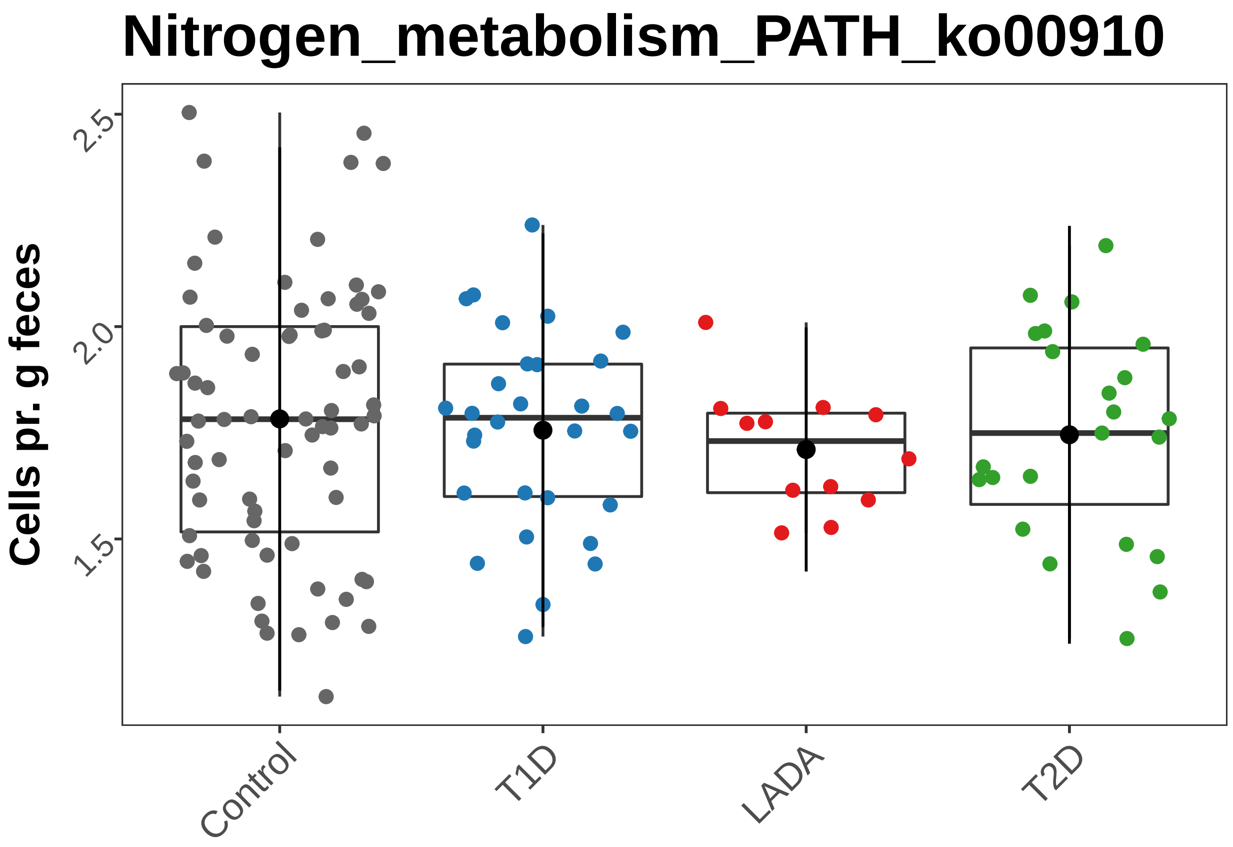

	pvalue	padj	Genus	compare
482	0.0137700	0.0408927	Fructose_and_mannose_metabolism_PATH_ko00051	LADA vs Control
286	0.0118980	0.0629443	Fructose_and_mannose_metabolism_PATH_ko00051	Control vs T2D
874	0.0856896	0.2434080	Fructose_and_mannose_metabolism_PATH_ko00051	LADA vs T1D
90	0.4027257	0.5803987	Fructose_and_mannose_metabolism_PATH_ko00051	Control vs T1D
678	0.1279380	0.6495082	Fructose_and_mannose_metabolism_PATH_ko00051	T1D vs T2D
1070	0.6428962	0.8841442	Fructose_and_mannose_metabolism_PATH_ko00051	LADA vs T2D

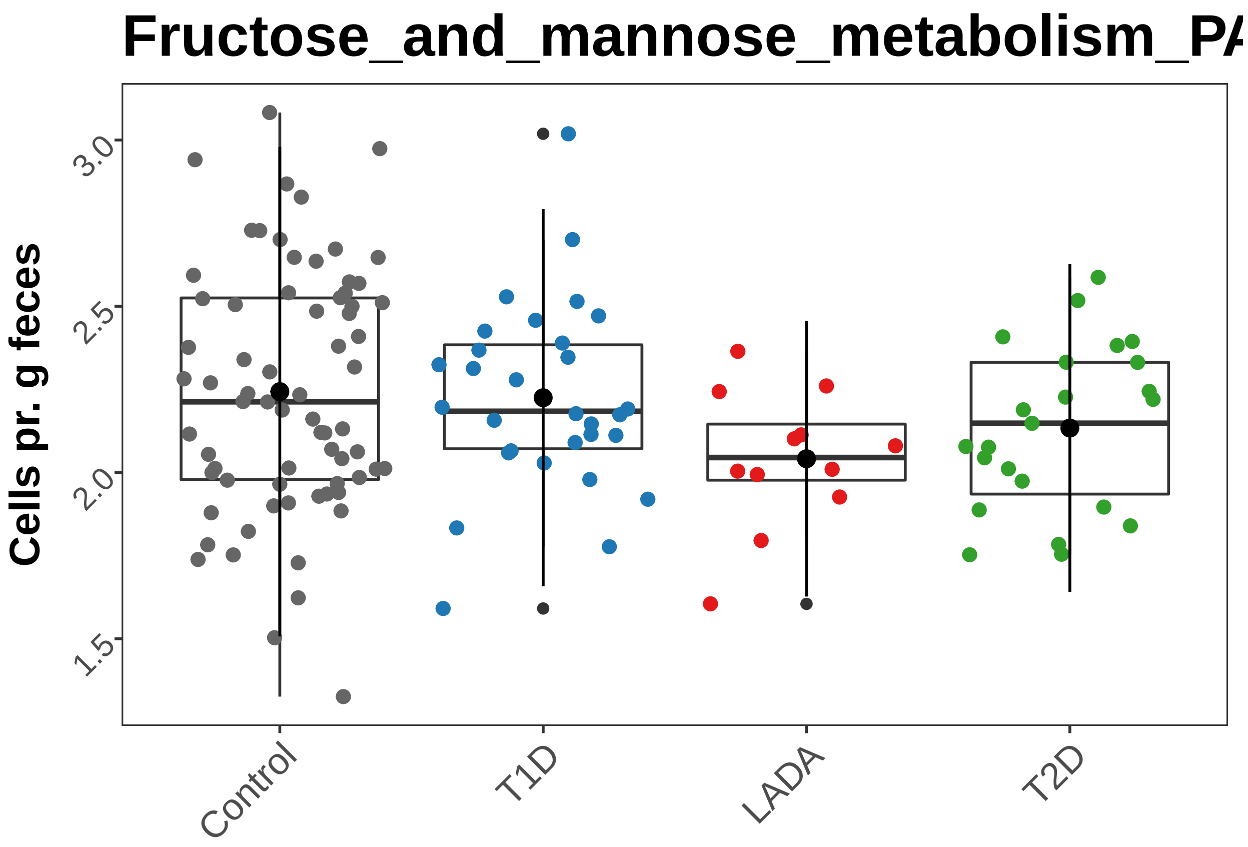

	pvalue	padj	Genus	compare
418	0.0147613	0.0431823	HIF_1_signaling_pathway_PATH_ko04066	LADA vs Control
222	0.0268340	0.0676342	HIF_1_signaling_pathway_PATH_ko04066	Control vs T2D
810	0.0581556	0.2312928	HIF_1_signaling_pathway_PATH_ko04066	LADA vs T1D
614	0.1320422	0.6495082	HIF_1_signaling_pathway_PATH_ko04066	T1D vs T2D
26	0.5991991	0.7249570	HIF_1_signaling_pathway_PATH_ko04066	Control vs T1D
1006	0.5189261	0.8415375	HIF_1_signaling_pathway_PATH_ko04066	LADA vs T2D

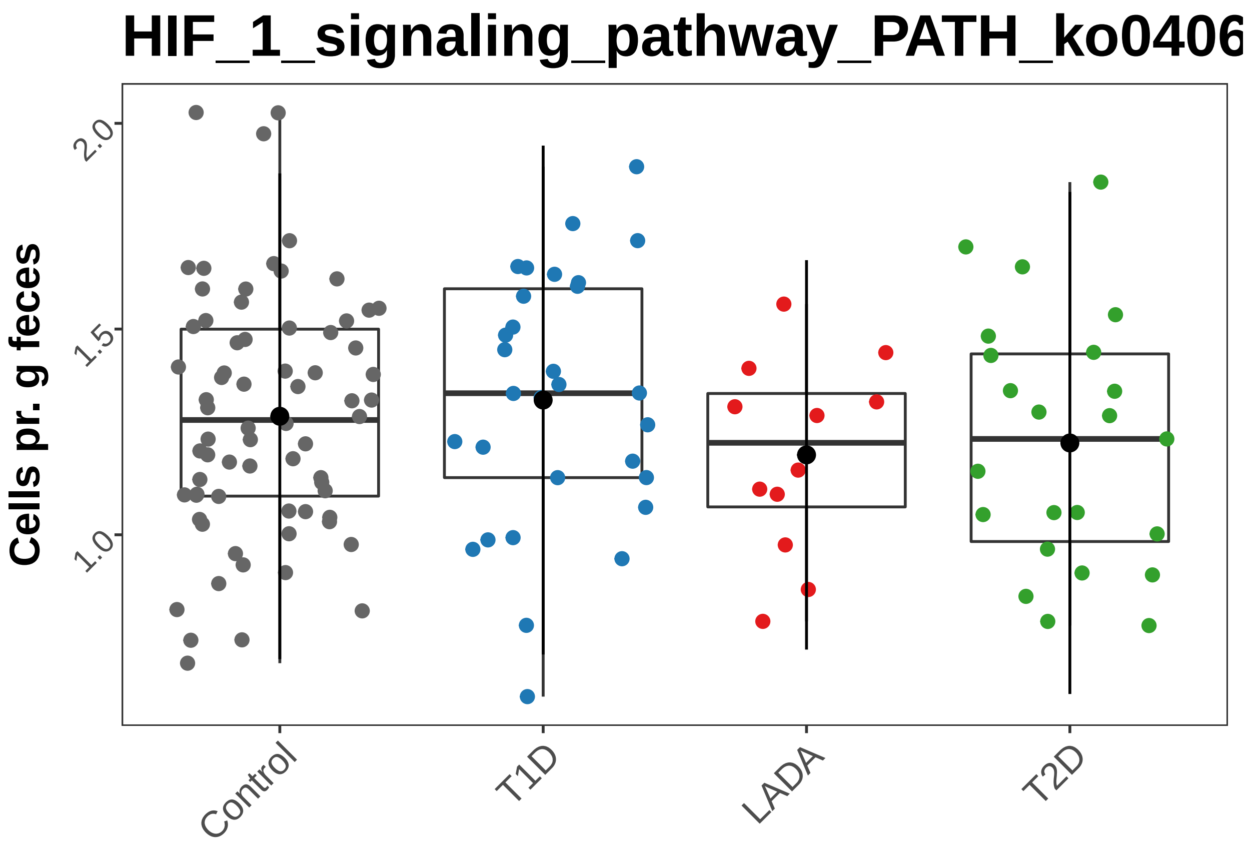

	pvalue	padj	Genus	compare
423	0.0157014	0.0442859	Phosphatidylinositol_signaling_system_PATH_ko04070	LADA vs Control
227	0.0404618	0.0767378	Phosphatidylinositol_signaling_system_PATH_ko04070	Control vs T2D
31	0.0427429	0.3989340	Phosphatidylinositol_signaling_system_PATH_ko04070	Control vs T1D
815	0.3600446	0.5040625	Phosphatidylinositol_signaling_system_PATH_ko04070	LADA vs T1D
1011	0.4613299	0.8415375	Phosphatidylinositol_signaling_system_PATH_ko04070	LADA vs T2D
619	0.8559827	0.8876857	Phosphatidylinositol_signaling_system_PATH_ko04070	T1D vs T2D

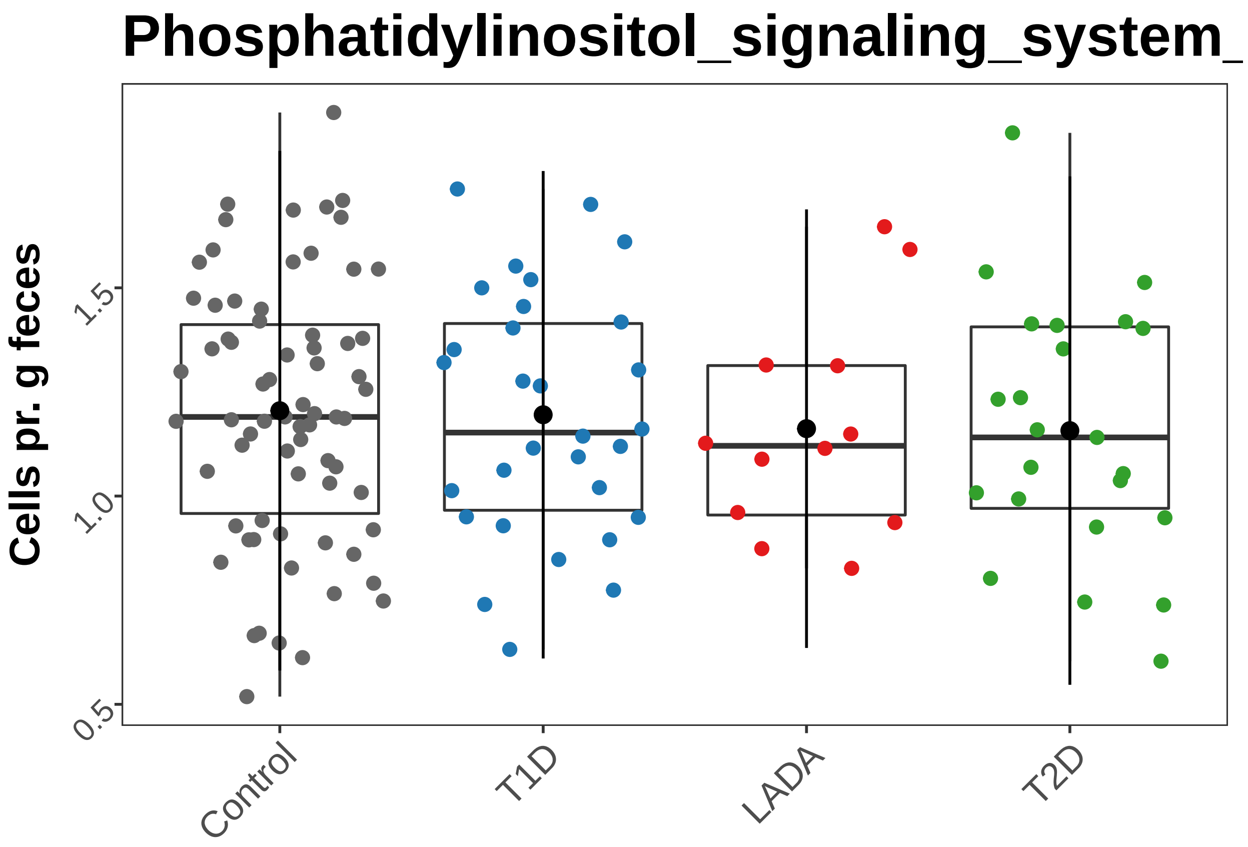

	pvalue	padj	Genus	compare
412	0.0158164	0.0442859	ABC_transporters_PATH_ko02010	LADA vs Control
216	0.0110469	0.0629443	ABC_transporters_PATH_ko02010	Control vs T2D
20	0.0136110	0.2904843	ABC_transporters_PATH_ko02010	Control vs T1D
804	0.5280291	0.6550234	ABC_transporters_PATH_ko02010	LADA vs T1D
608	0.7940983	0.8695154	ABC_transporters_PATH_ko02010	T1D vs T2D
1000	0.6875910	0.9028767	ABC_transporters_PATH_ko02010	LADA vs T2D

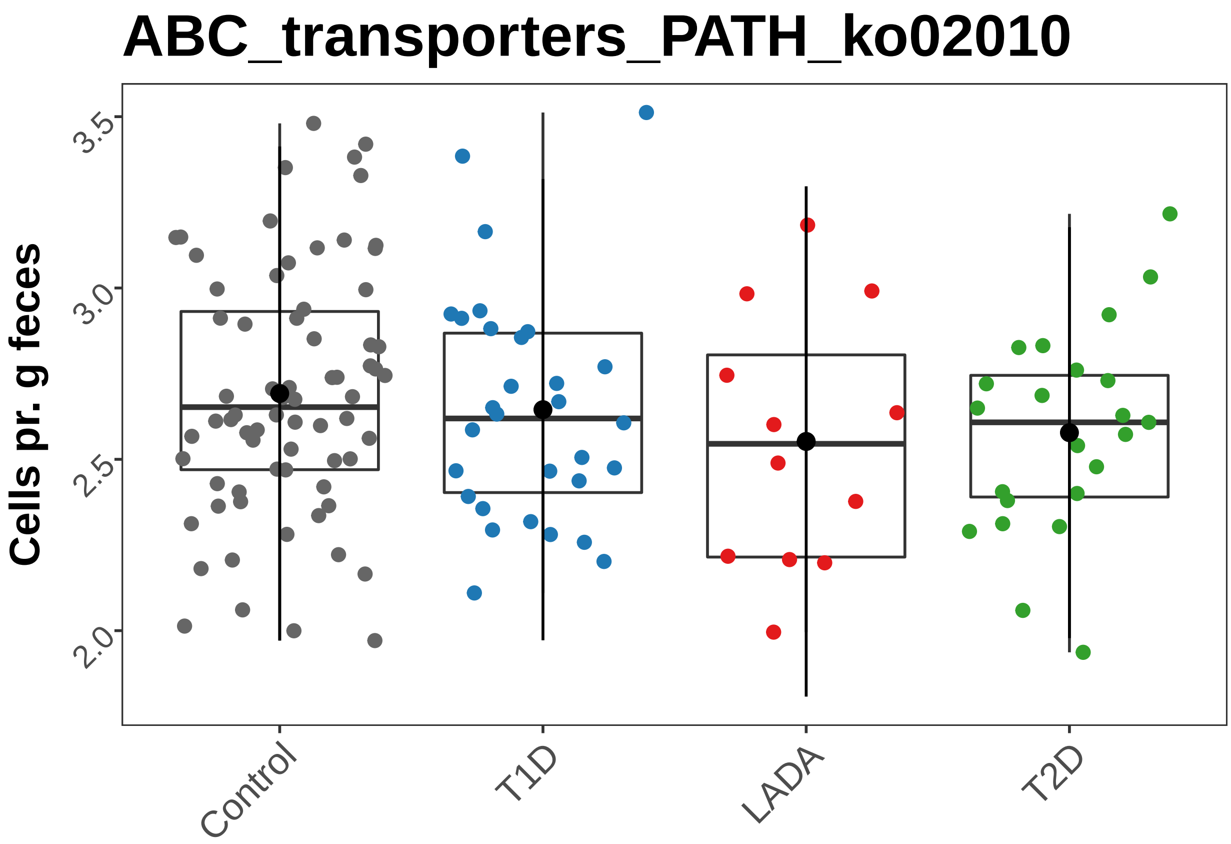

	pvalue	padj	Genus	compare
433	0.0161830	0.0446743	Base_excision_repair_PATH_ko03410	LADA vs Control
237	0.0146192	0.0629443	Base_excision_repair_PATH_ko03410	Control vs T2D
825	0.1371763	0.2954567	Base_excision_repair_PATH_ko03410	LADA vs T1D
41	0.2641971	0.4839499	Base_excision_repair_PATH_ko03410	Control vs T1D
629	0.2156093	0.6495082	Base_excision_repair_PATH_ko03410	T1D vs T2D
1021	0.6441080	0.8841442	Base_excision_repair_PATH_ko03410	LADA vs T2D

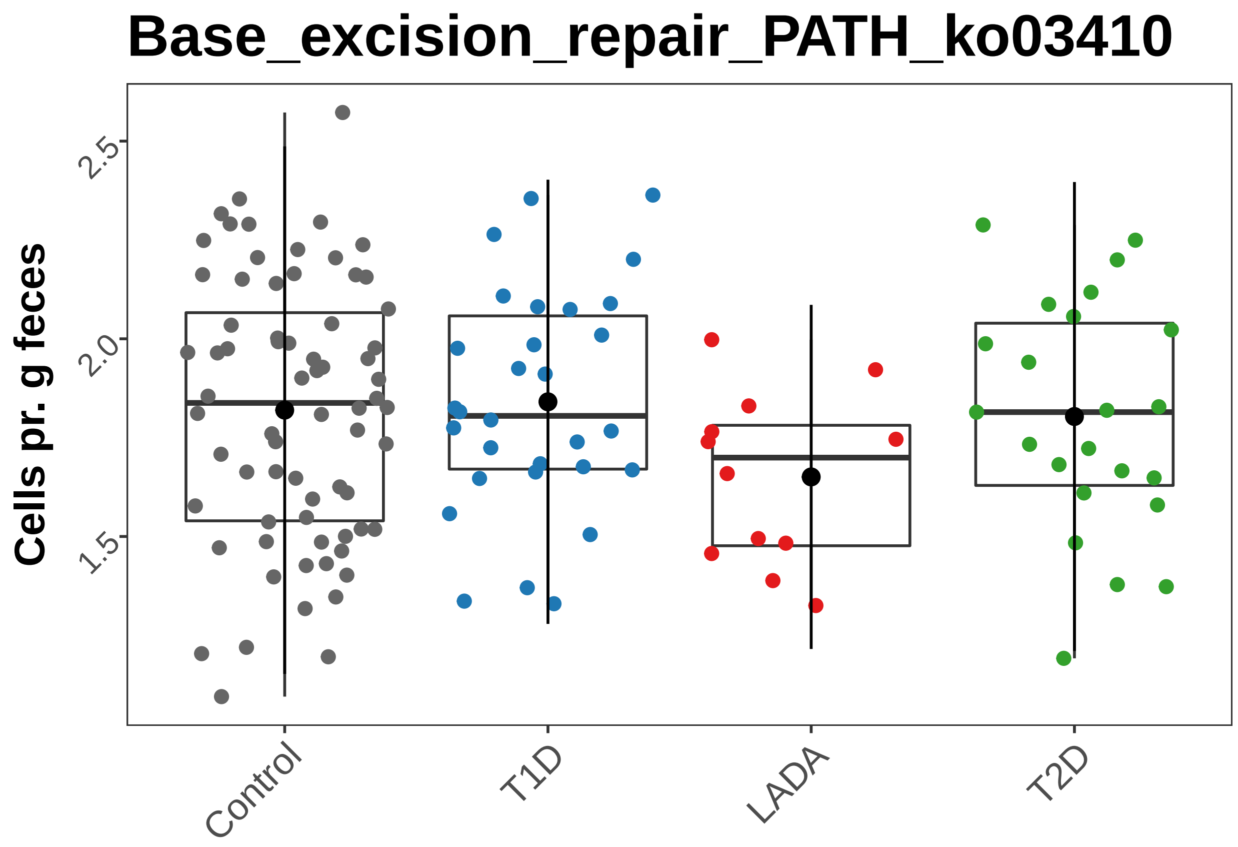

	pvalue	padj	Genus	compare
573	0.0167594	0.0450038	Metabolism_of_xenobiotics_by_cytochrome_P450_PATH_ko00980	LADA vs Control
965	0.0556750	0.2312928	Metabolism_of_xenobiotics_by_cytochrome_P450_PATH_ko00980	LADA vs T1D
1161	0.0062758	0.3088442	Metabolism_of_xenobiotics_by_cytochrome_P450_PATH_ko00980	LADA vs T2D
377	0.3472540	0.4257036	Metabolism_of_xenobiotics_by_cytochrome_P450_PATH_ko00980	Control vs T2D
769	0.2488134	0.6495082	Metabolism_of_xenobiotics_by_cytochrome_P450_PATH_ko00980	T1D vs T2D
181	0.6676012	0.7652037	Metabolism_of_xenobiotics_by_cytochrome_P450_PATH_ko00980	Control vs T1D

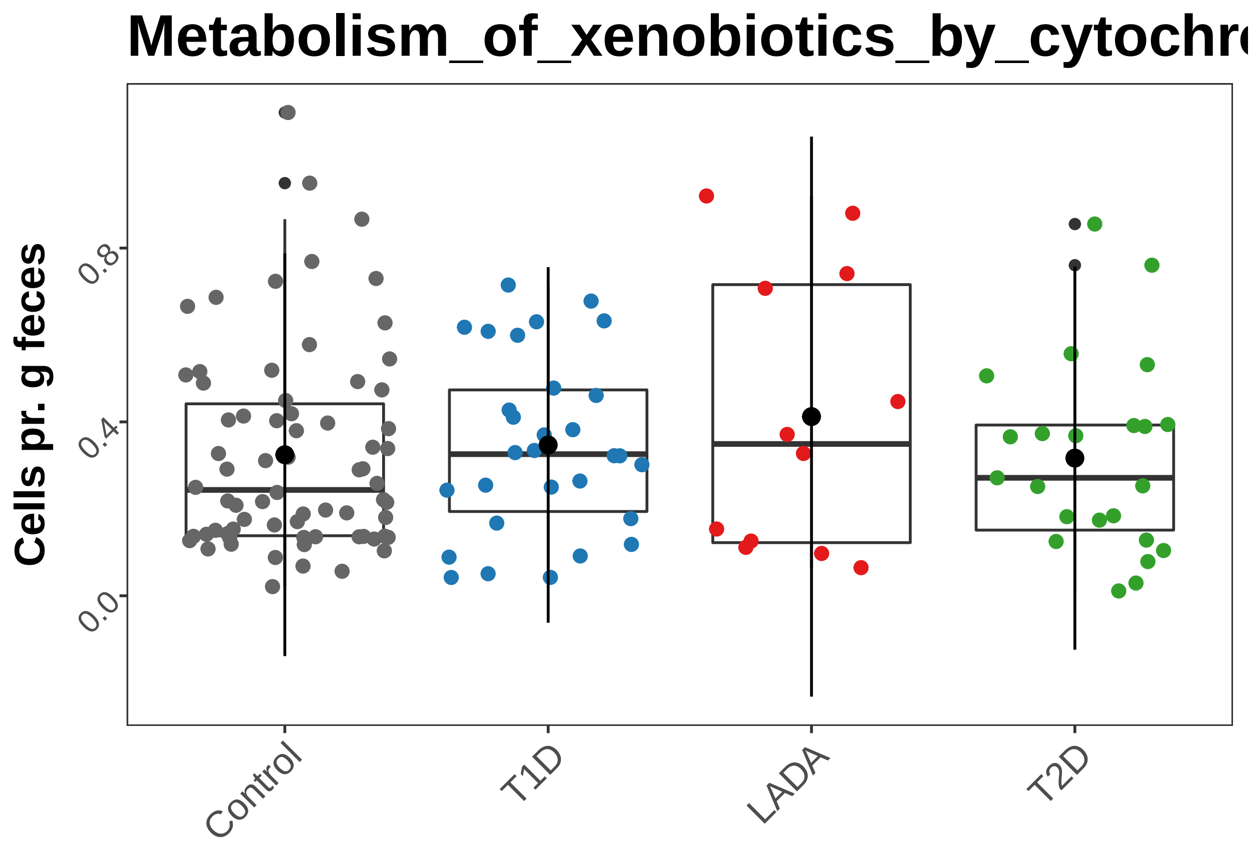

	pvalue	padj	Genus	compare
569	0.0167616	0.0450038	Drug_metabolism____cytochrome_P450_PATH_ko00982	LADA vs Control
961	0.0558959	0.2312928	Drug_metabolism____cytochrome_P450_PATH_ko00982	LADA vs T1D
1157	0.0063029	0.3088442	Drug_metabolism____cytochrome_P450_PATH_ko00982	LADA vs T2D
373	0.3483030	0.4257036	Drug_metabolism____cytochrome_P450_PATH_ko00982	Control vs T2D
765	0.2486905	0.6495082	Drug_metabolism____cytochrome_P450_PATH_ko00982	T1D vs T2D
177	0.6656882	0.7652037	Drug_metabolism____cytochrome_P450_PATH_ko00982	Control vs T1D

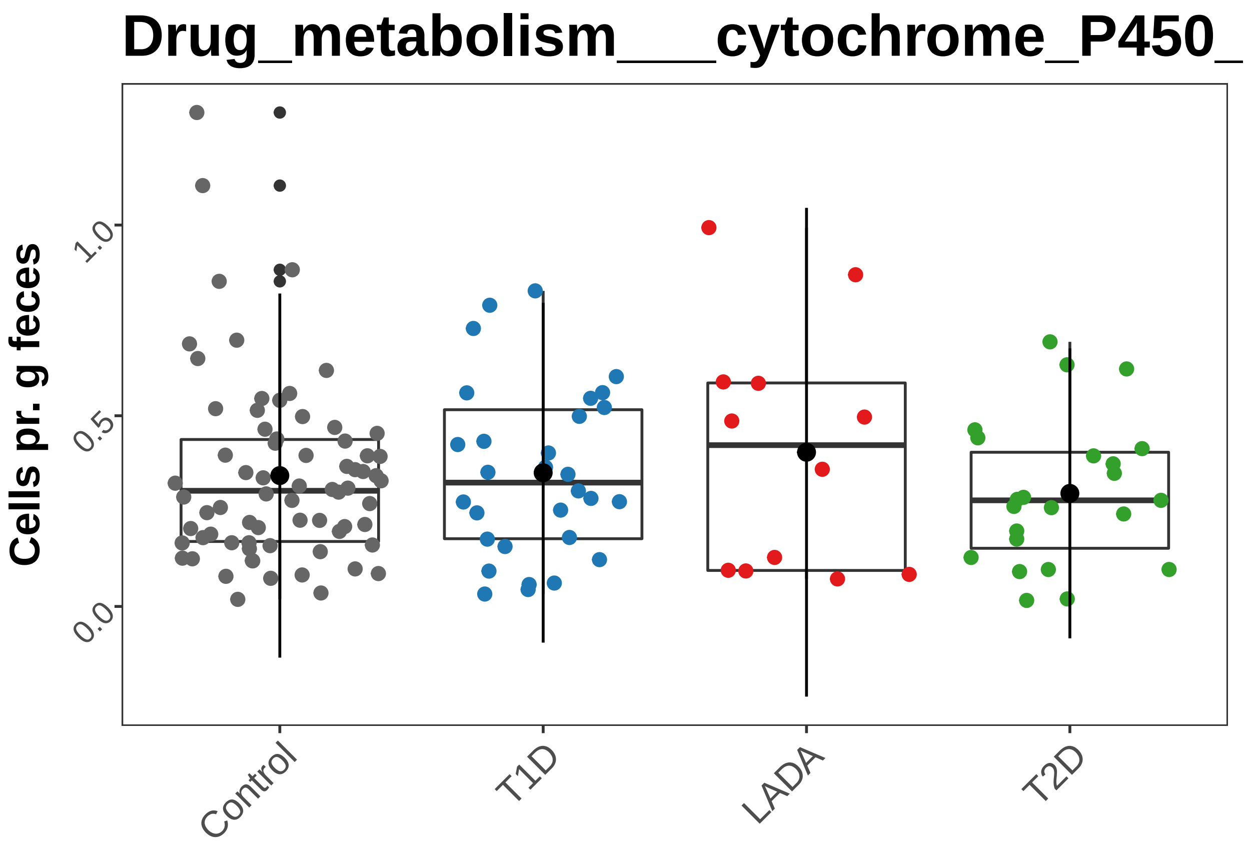

	pvalue	padj	Genus	compare
491	0.0188318	0.0493905	Starch_and_sucrose_metabolism_PATH_ko00500	LADA vs Control
295	0.0120571	0.0629443	Starch_and_sucrose_metabolism_PATH_ko00500	Control vs T2D
99	0.0177848	0.2904843	Starch_and_sucrose_metabolism_PATH_ko00500	Control vs T1D
883	0.5258331	0.6550234	Starch_and_sucrose_metabolism_PATH_ko00500	LADA vs T1D
687	0.7558444	0.8322782	Starch_and_sucrose_metabolism_PATH_ko00500	T1D vs T2D
1079	0.7140096	0.9028767	Starch_and_sucrose_metabolism_PATH_ko00500	LADA vs T2D

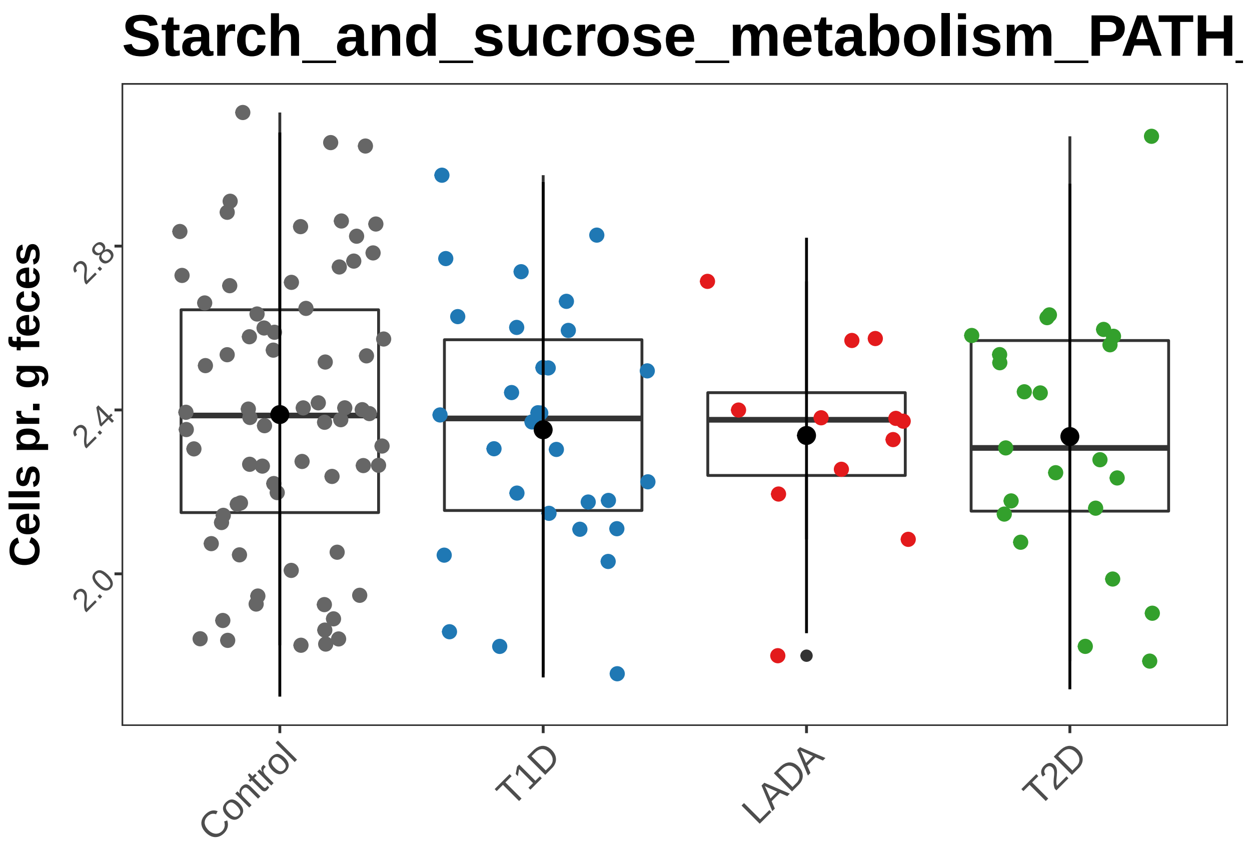

	pvalue	padj	Genus	compare
408	0.0188994	0.0493905	Autophagy_____yeast__PATH__ko04138	LADA vs Control
212	0.2593734	0.3292047	Autophagy_____yeast__PATH__ko04138	Control vs T2D
800	0.2394439	0.3950690	Autophagy_____yeast__PATH__ko04138	LADA vs T1D
16	0.1285572	0.4612224	Autophagy_____yeast__PATH__ko04138	Control vs T1D
996	0.1941252	0.8415375	Autophagy_____yeast__PATH__ko04138	LADA vs T2D
604	0.8269710	0.8857176	Autophagy_____yeast__PATH__ko04138	T1D vs T2D

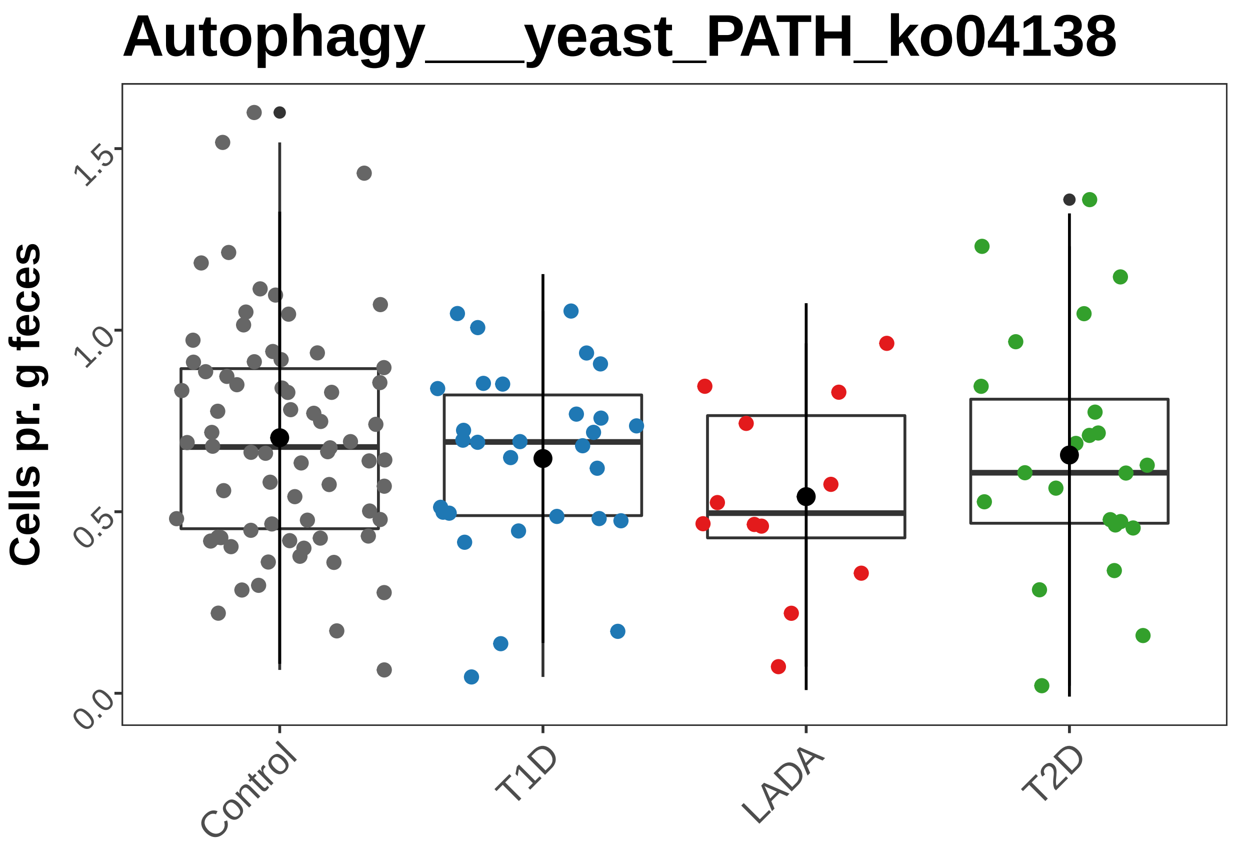

	pvalue	padj	Genus	compare
477	0.0191645	0.0494243	Amino_sugar_and_nucleotide_sugar_metabolism_PATH_ko00520	LADA vs Control
281	0.0204882	0.0661577	Amino_sugar_and_nucleotide_sugar_metabolism_PATH_ko00520	Control vs T2D
869	0.1941789	0.3597318	Amino_sugar_and_nucleotide_sugar_metabolism_PATH_ko00520	LADA vs T1D
85	0.1863352	0.4762288	Amino_sugar_and_nucleotide_sugar_metabolism_PATH_ko00520	Control vs T1D
673	0.3325322	0.6495082	Amino_sugar_and_nucleotide_sugar_metabolism_PATH_ko00520	T1D vs T2D
1065	0.6233342	0.8841442	Amino_sugar_and_nucleotide_sugar_metabolism_PATH_ko00520	LADA vs T2D

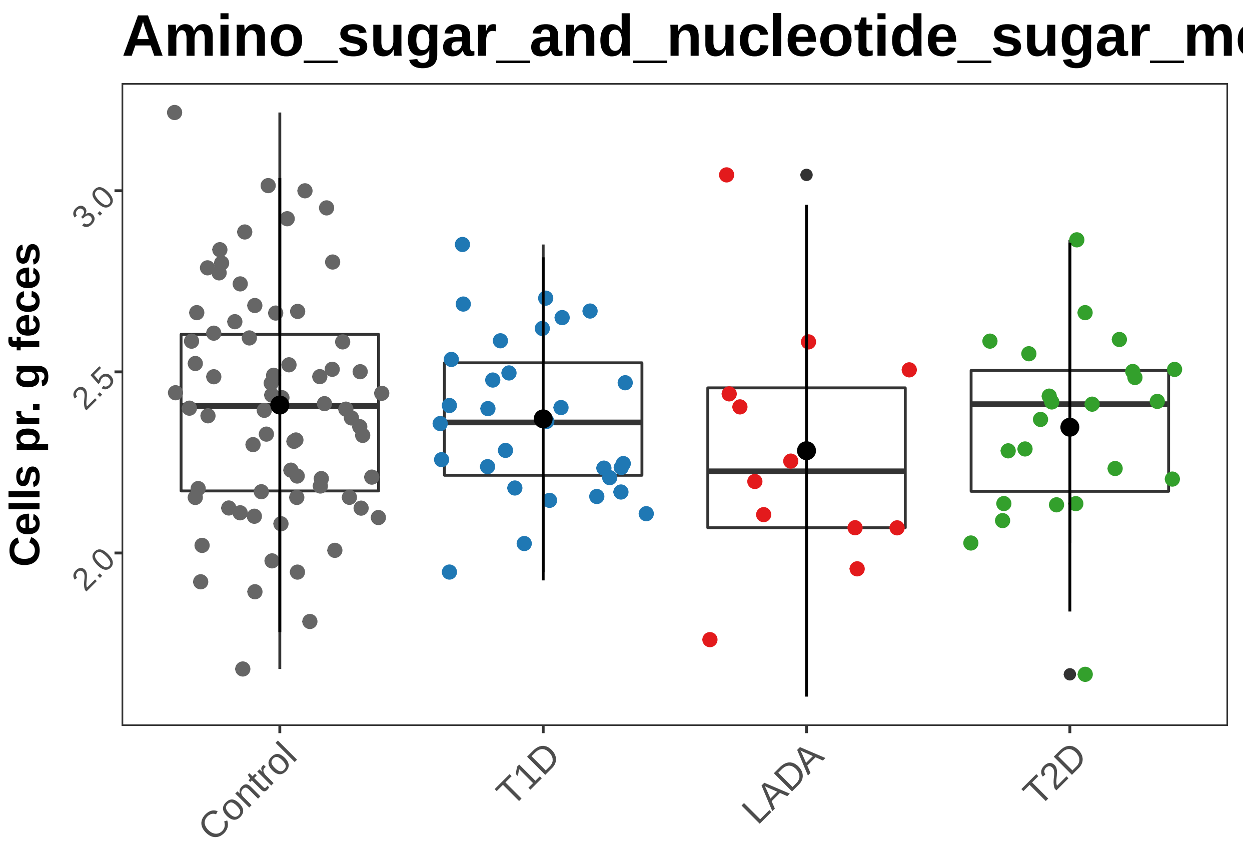

	pvalue	padj	Genus	compare
490	0.0204551	0.0517856	Pyruvate_metabolism_PATH_ko00620	LADA vs Control
294	0.0222761	0.0676342	Pyruvate_metabolism_PATH_ko00620	Control vs T2D
882	0.0687580	0.2312928	Pyruvate_metabolism_PATH_ko00620	LADA vs T1D
686	0.1069516	0.6495082	Pyruvate_metabolism_PATH_ko00620	T1D vs T2D
98	0.6383849	0.7537556	Pyruvate_metabolism_PATH_ko00620	Control vs T1D
1078	0.6233769	0.8841442	Pyruvate_metabolism_PATH_ko00620	LADA vs T2D

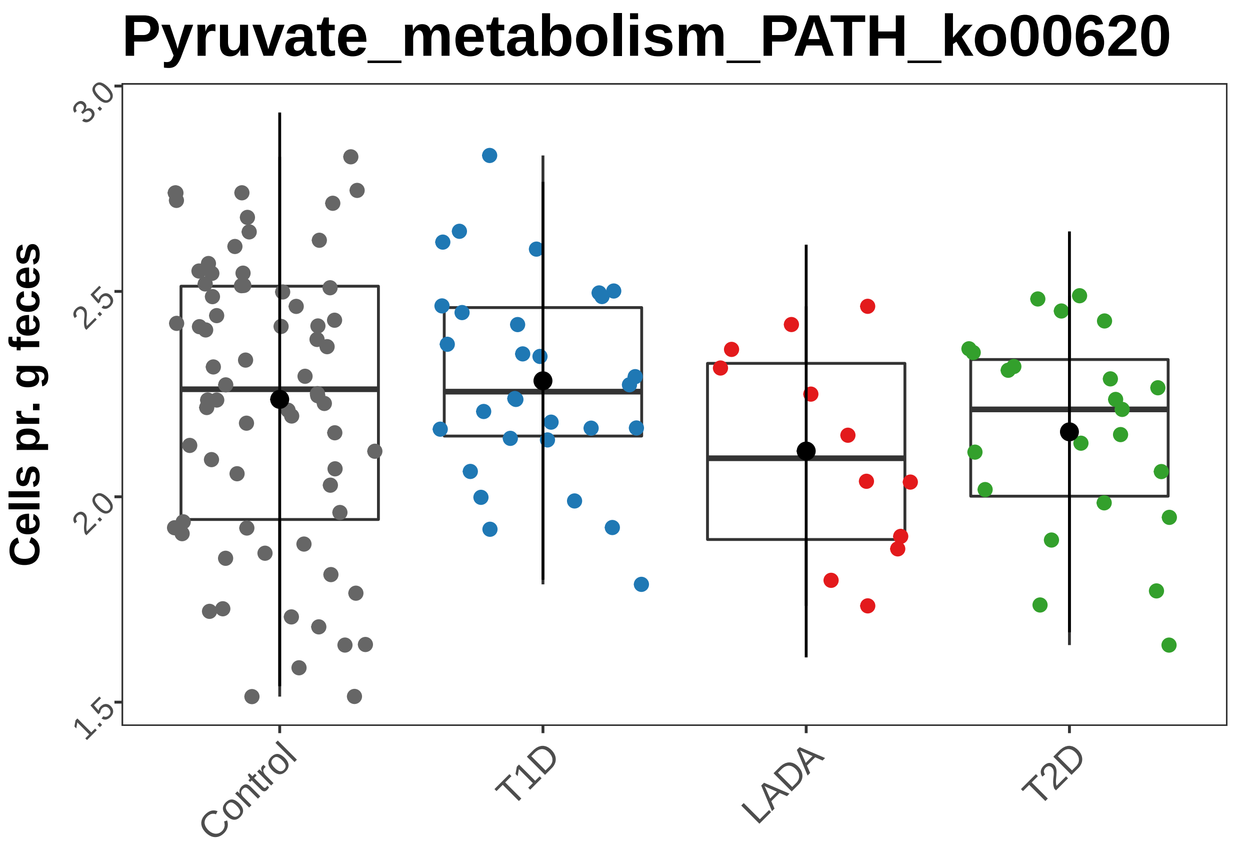

	pvalue	padj	Genus	compare
476	0.0206085	0.0517856	Tropane___piperidine_and_pyridine_alkaloid_biosynthesis_PATH_ko00960	LADA_vs_Control
280	0.0594688	0.0981236	Tropane___piperidine_and_pyridine_alkaloid_biosynthesis_PATH_ko00960	Control_vs_T2D
868	0.1806223	0.3554025	Tropane___piperidine_and_pyridine_alkaloid_biosynthesis_PATH_ko00960	LADA_vs_T1D
84	0.2228203	0.4786856	Tropane___piperidine_and_pyridine_alkaloid_biosynthesis_PATH_ko00960	Control_vs_T1D
672	0.4999617	0.6852622	Tropane___piperidine_and_pyridine_alkaloid_biosynthesis_PATH_ko00960	T1D_vs_T2D
1064	0.4477410	0.8415375	Tropane___piperidine_and_pyridine_alkaloid_biosynthesis_PATH_ko00960	LADA_vs_T2D

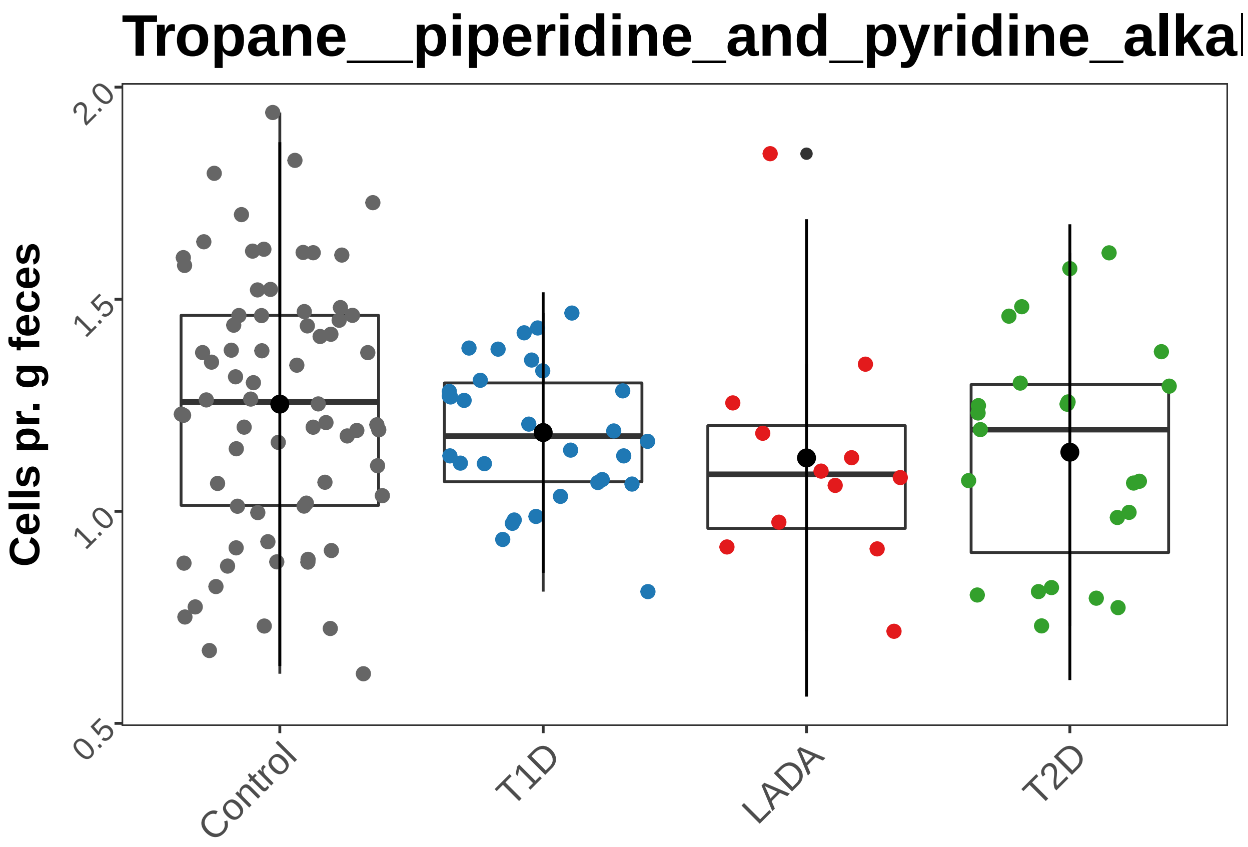

	pvalue	padj	Genus	compare
550	0.0213722	0.0530248	Biosynthesis_of_vancomycin_group_antibiotics_PATH_ko01055	LADA vs Control
942	0.1247153	0.2777750	Biosynthesis_of_vancomycin_group_antibiotics_PATH_ko01055	LADA vs T1D
354	0.2183312	0.2859099	Biosynthesis_of_vancomycin_group_antibiotics_PATH_ko01055	Control vs T2D
158	0.3724298	0.5530018	Biosynthesis_of_vancomycin_group_antibiotics_PATH_ko01055	Control vs T1D
746	0.7149550	0.8007496	Biosynthesis_of_vancomycin_group_antibiotics_PATH_ko01055	T1D vs T2D
1138	0.2346910	0.8415375	Biosynthesis_of_vancomycin_group_antibiotics_PATH_ko01055	LADA vs T2D

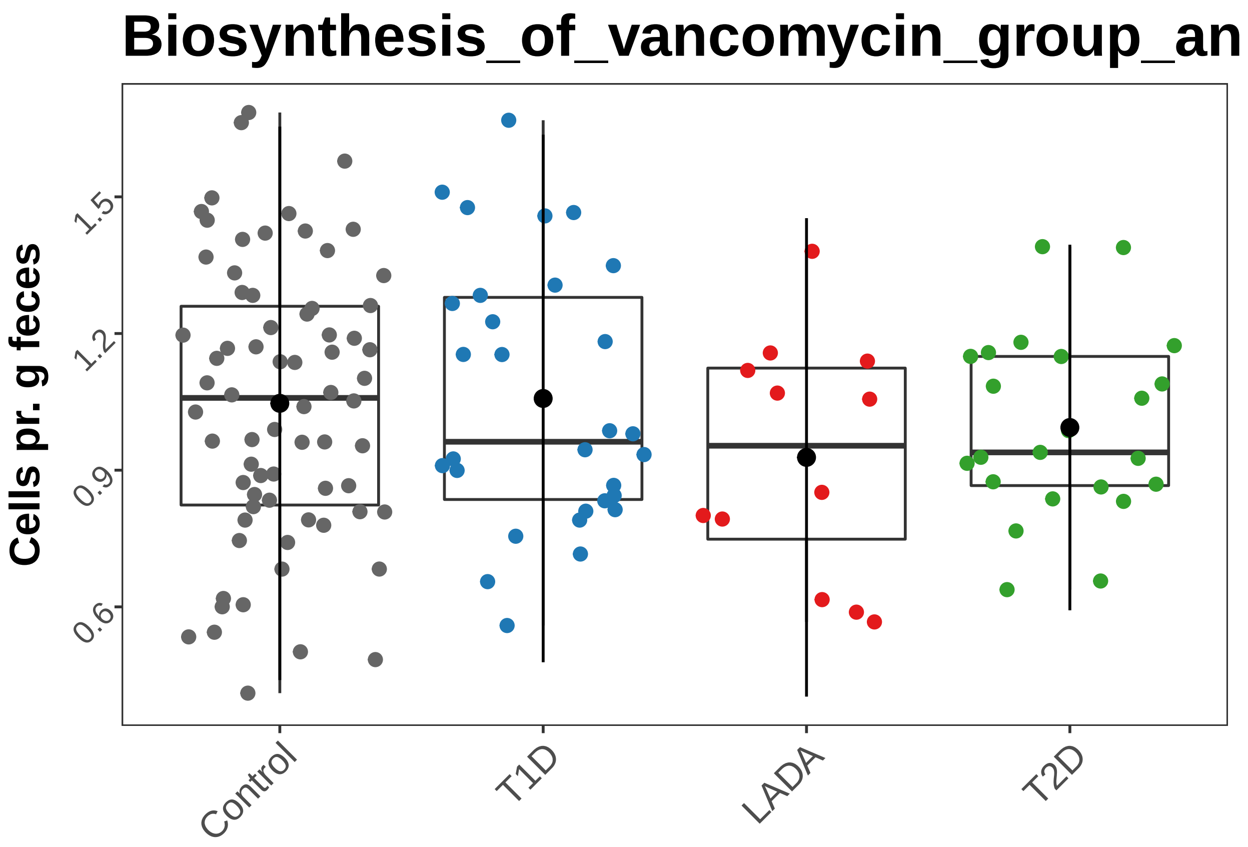

	pvalue	padj	Genus	compare
533	0.0226272	0.0549700	Porphyrin_and_chlorophyll_metabolism_PATH_ko00860	LADA vs Control
337	0.0561576	0.0945510	Porphyrin_and_chlorophyll_metabolism_PATH_ko00860	Control vs T2D
925	0.0747015	0.2321553	Porphyrin_and_chlorophyll_metabolism_PATH_ko00860	LADA vs T1D
729	0.1995785	0.6495082	Porphyrin_and_chlorophyll_metabolism_PATH_ko00860	T1D vs T2D
141	0.6353121	0.7537556	Porphyrin_and_chlorophyll_metabolism_PATH_ko00860	Control vs T1D
1121	0.4769734	0.8415375	Porphyrin_and_chlorophyll_metabolism_PATH_ko00860	LADA vs T2D

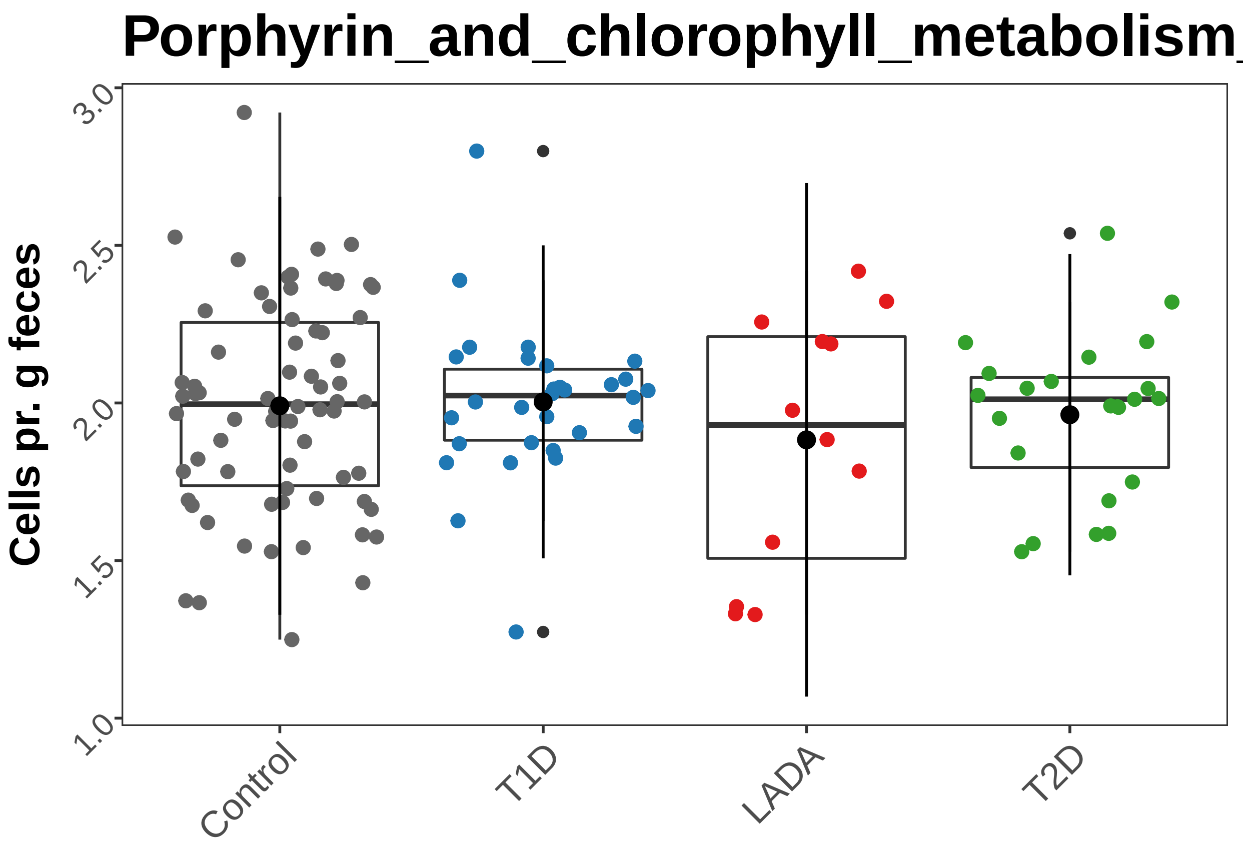

	pvalue	padj	Genus	compare
516	0.0227172	0.0549700	Fatty__acid__biosynthesis__PATH__ko00061	LADA vs Control
320	0.0227070	0.0676342	Fatty__acid__biosynthesis__PATH__ko00061	Control vs T2D
908	0.1848778	0.3554025	Fatty__acid__biosynthesis__PATH__ko00061	LADA vs T1D
124	0.2354304	0.4839499	Fatty__acid__biosynthesis__PATH__ko00061	Control vs T1D
712	0.2976214	0.6495082	Fatty__acid__biosynthesis__PATH__ko00061	T1D vs T2D
1104	0.6447043	0.8841442	Fatty__acid__biosynthesis__PATH__ko00061	LADA vs T2D

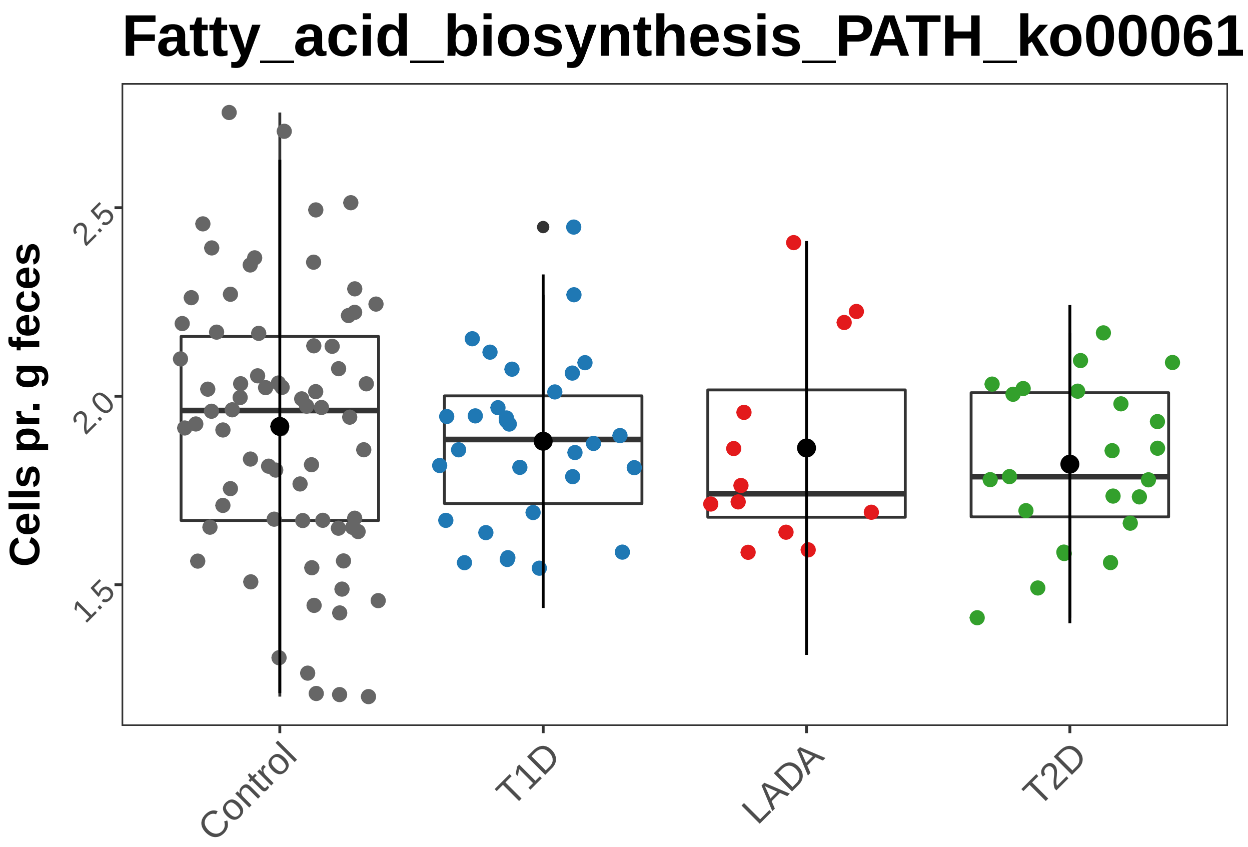

	pvalue	padj	Genus	compare
446	0.0230254	0.0550363	RNA_transport_PATH_ko03013	LADA vs Control
838	0.0016859	0.0632892	RNA_transport_PATH_ko03013	LADA vs T1D
250	0.2995526	0.3744407	RNA_transport_PATH_ko03013	Control vs T2D
54	0.0966328	0.4612224	RNA_transport_PATH_ko03013	Control vs T1D
642	0.0272615	0.6495082	RNA_transport_PATH_ko03013	T1D vs T2D
1034	0.1956129	0.8415375	RNA_transport_PATH_ko03013	LADA vs T2D

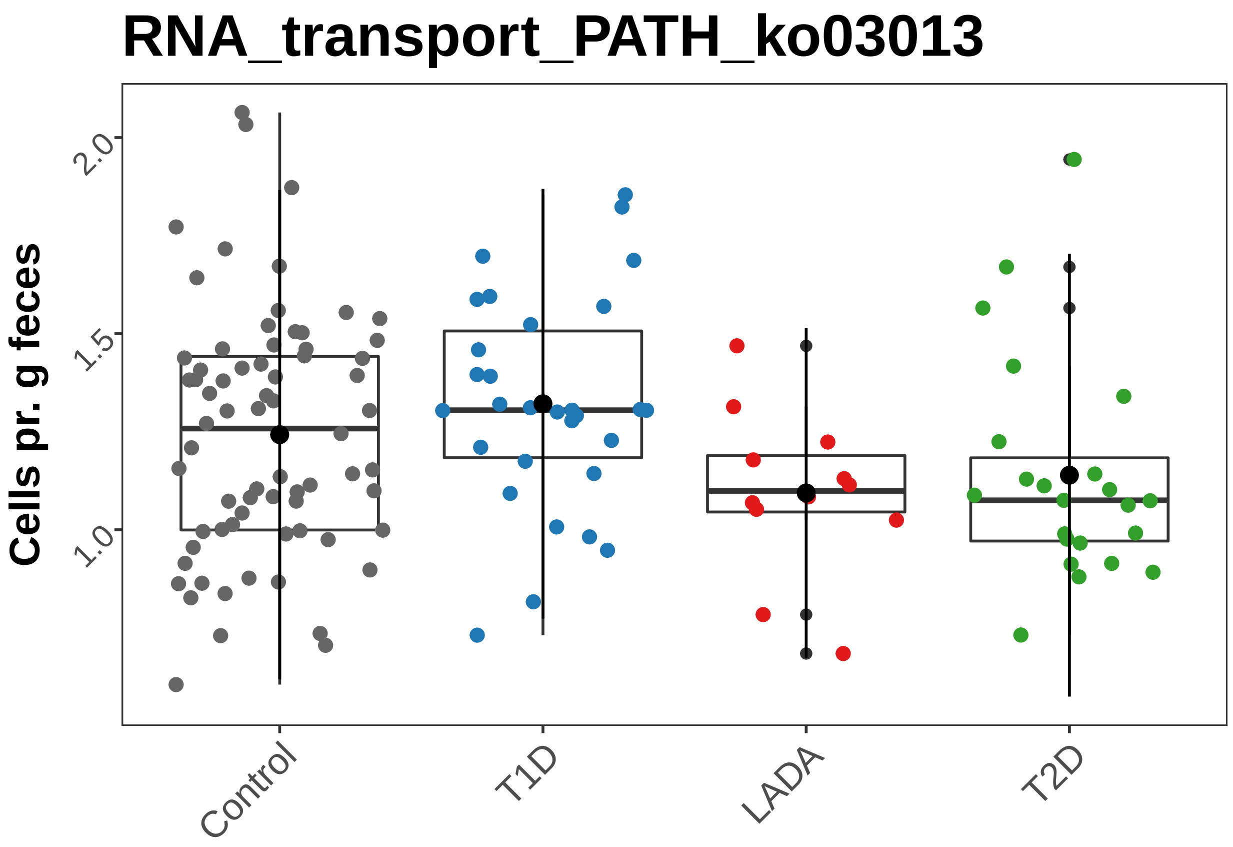

	pvalue	padj	Genus	compare
586	0.0240073	0.0566919	Fatty_acid_metabolism	LADA vs Control
390	0.0213993	0.0666206	Fatty_acid_metabolism	Control vs T2D
978	0.2019922	0.3597318	Fatty_acid_metabolism	LADA vs T1D
194	0.2170335	0.4786856	Fatty_acid_metabolism	Control vs T1D
782	0.3061560	0.6495082	Fatty_acid_metabolism	T1D vs T2D
1174	0.6691103	0.8952178	Fatty_acid_metabolism	LADA vs T2D

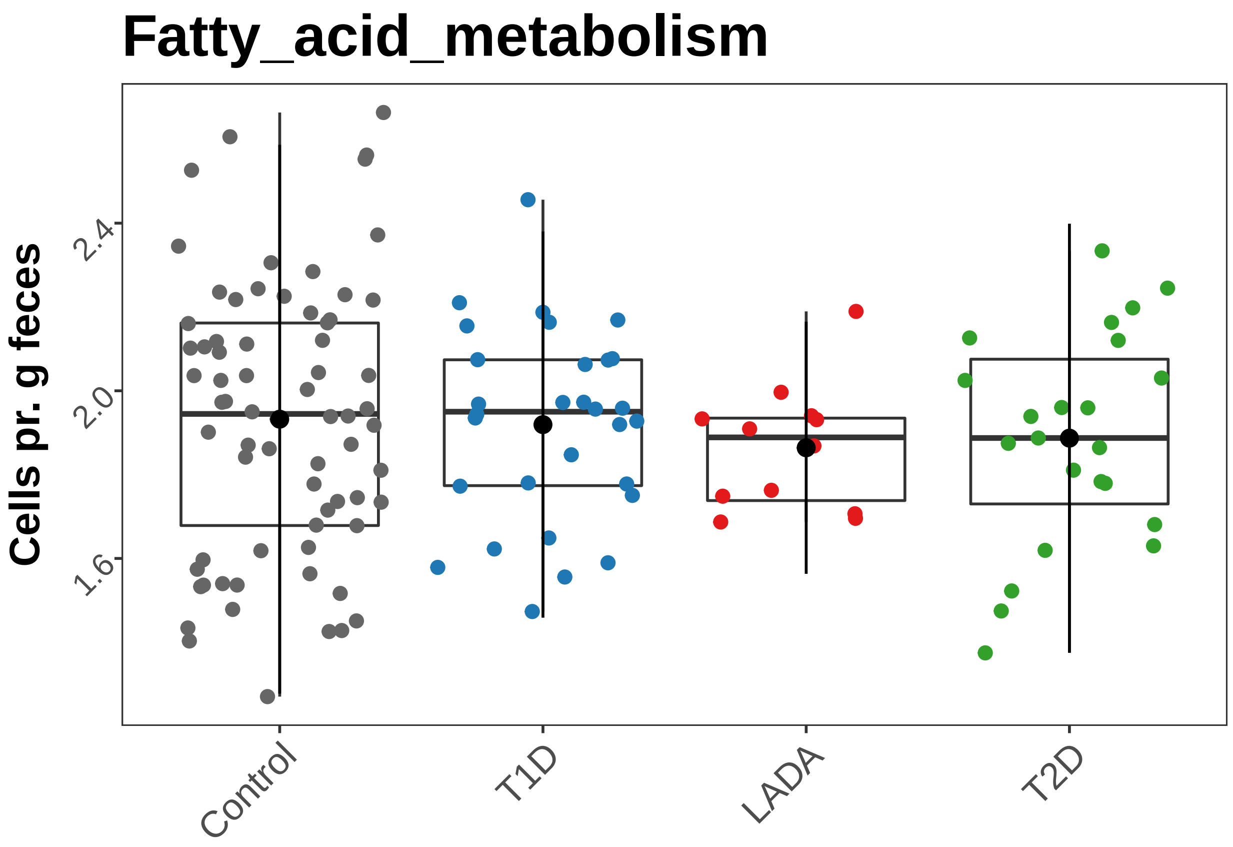

	pvalue	padj	Genus	compare
521	0.0245535	0.0569158	Primary__bile__acid__biosynthesis_PATH__ko00120	LADA vs Control
325	0.1162234	0.1682181	Primary__bile__acid__biosynthesis_PATH__ko00120	Control vs T2D
129	0.0568772	0.3992571	Primary__bile__acid__biosynthesis_PATH__ko00120	Control vs T1D
913	0.4008845	0.5483058	Primary__bile__acid__biosynthesis_PATH__ko00120	LADA vs T1D
1109	0.3615058	0.8415375	Primary__bile__acid__biosynthesis_PATH__ko00120	LADA vs T2D
717	0.8909005	0.9047487	Primary__bile__acid__biosynthesis_PATH__ko00120	T1D vs T2D

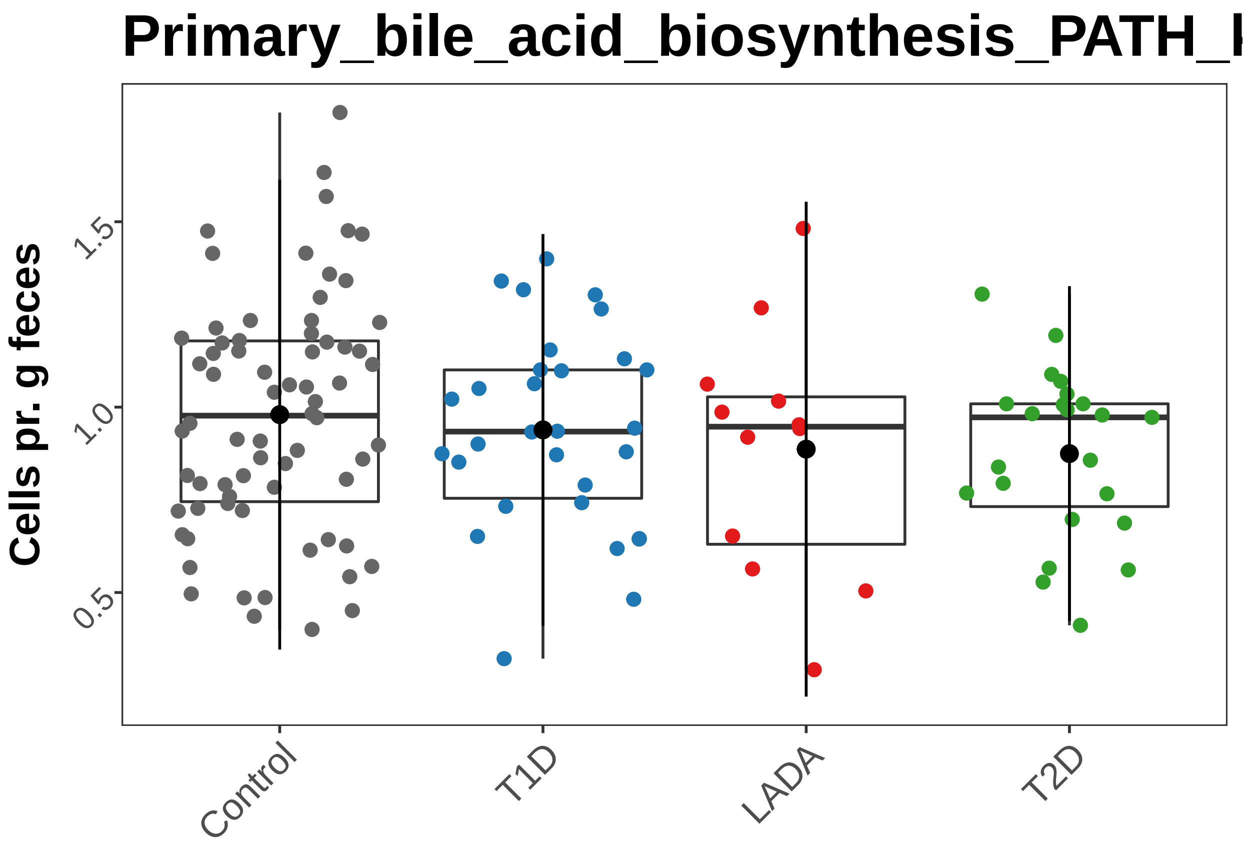

	pvalue	padj	Genus	compare
414	0.0246829	0.0569158	Phosphotransferase_system___PTS___PATH_ko02060	LADA vs Control
218	0.0056557	0.0629443	Phosphotransferase_system___PTS___PATH_ko02060	Control vs T2D
806	0.1854911	0.3554025	Phosphotransferase_system___PTS___PATH_ko02060	LADA vs T1D
22	0.2527021	0.4839499	Phosphotransferase_system___PTS___PATH_ko02060	Control vs T1D
610	0.1338708	0.6495082	Phosphotransferase_system___PTS___PATH_ko02060	T1D vs T2D
1002	0.9177048	0.9828969	Phosphotransferase_system___PTS___PATH_ko02060	LADA vs T2D

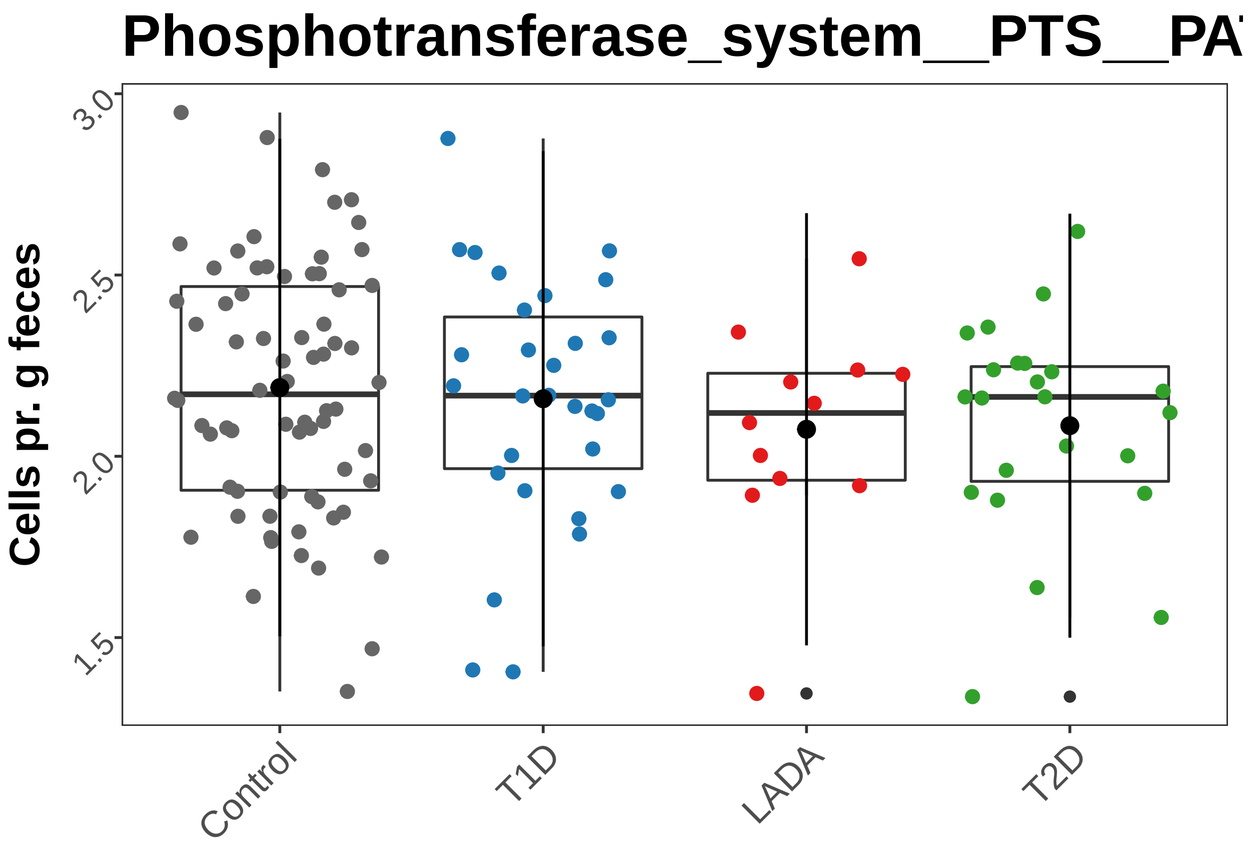

	pvalue	padj	Genus	compare
539	0.0259727	0.0574628	beta__Alanine__metabolism__PATH__ko00410	LADA vs Control
343	0.0123800	0.0629443	beta__Alanine__metabolism__PATH__ko00410	Control vs T2D
931	0.1867676	0.3554025	beta__Alanine__metabolism__PATH__ko00410	LADA vs T1D
147	0.2620385	0.4839499	beta__Alanine__metabolism__PATH__ko00410	Control vs T1D
735	0.1984936	0.6495082	beta__Alanine__metabolism__PATH__ko00410	T1D vs T2D
1127	0.7905944	0.9061784	beta__Alanine__metabolism__PATH__ko00410	LADA vs T2D

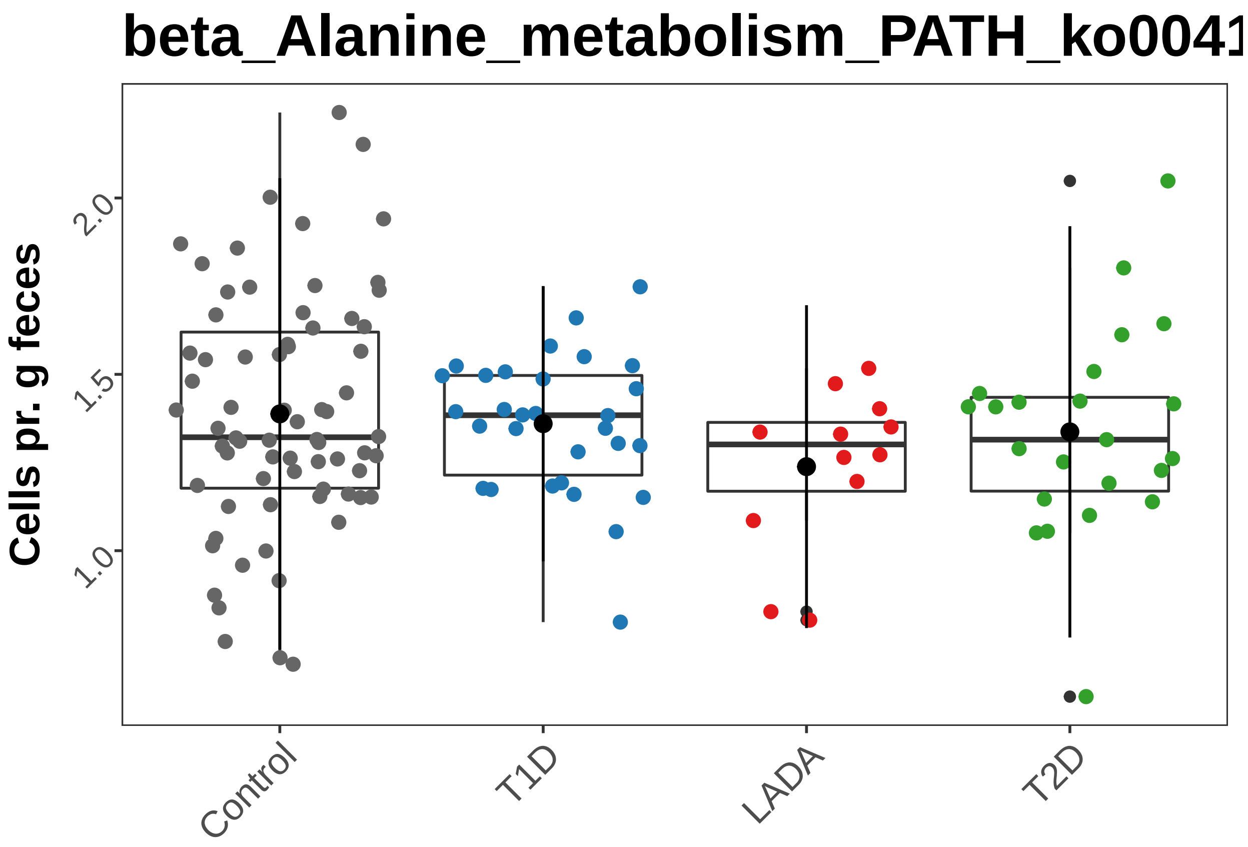

	pvalue	padj	Genus	compare
479	0.0262357	0.0574628	Butanoate_metabolism_PATH_ko00650	LADA vs Control
283	0.0283702	0.0676342	Butanoate_metabolism_PATH_ko00650	Control vs T2D
871	0.0970624	0.2458003	Butanoate_metabolism_PATH_ko00650	LADA vs T1D
675	0.1498178	0.6495082	Butanoate_metabolism_PATH_ko00650	T1D vs T2D
87	0.5583668	0.6970694	Butanoate_metabolism_PATH_ko00650	Control vs T1D
1067	0.6378868	0.8841442	Butanoate_metabolism_PATH_ko00650	LADA vs T2D

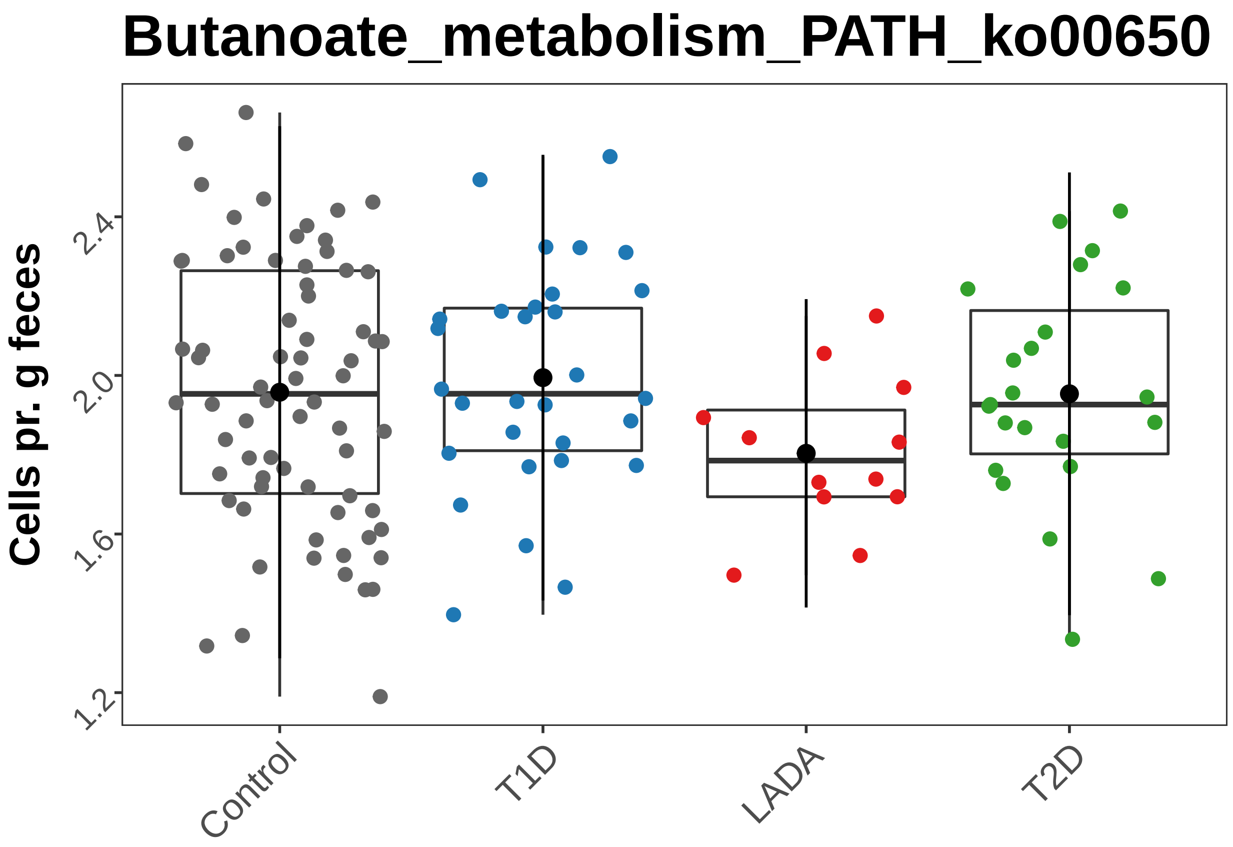

	pvalue	padj	Genus	compare
485	0.0263113	0.0574628	Glyoxylate_and_dicarboxylate_metabolism_PATH_ko00630	LADA vs Control
289	0.0433535	0.0794814	Glyoxylate_and_dicarboxylate_metabolism_PATH_ko00630	Control vs T2D
877	0.2666769	0.4051834	Glyoxylate_and_dicarboxylate_metabolism_PATH_ko00630	LADA vs T1D
93	0.1492374	0.4612224	Glyoxylate_and_dicarboxylate_metabolism_PATH_ko00630	Control vs T1D
681	0.5376224	0.6978411	Glyoxylate_and_dicarboxylate_metabolism_PATH_ko00630	T1D vs T2D
1073	0.5581089	0.8681693	Glyoxylate_and_dicarboxylate_metabolism_PATH_ko00630	LADA vs T2D

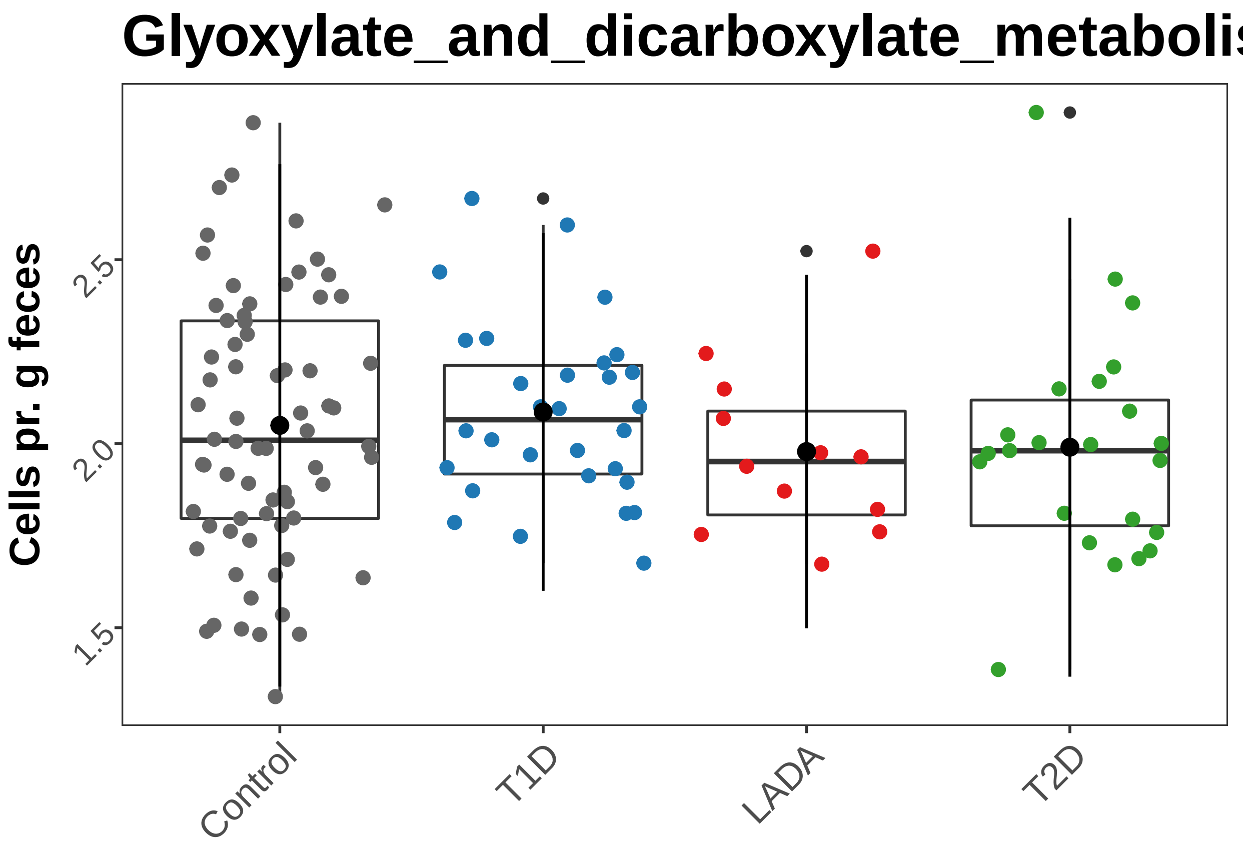

	pvalue	padj	Genus	compare
522	0.0266088	0.0574628	Secondary__bile__acid__biosynthesis__PATH__ko00121	LADA vs Control
326	0.1186247	0.1702007	Secondary__bile__acid__biosynthesis__PATH__ko00121	Control vs T2D
130	0.0575187	0.3992571	Secondary__bile__acid__biosynthesis__PATH__ko00121	Control vs T1D
914	0.4152528	0.5574627	Secondary__bile__acid__biosynthesis__PATH__ko00121	LADA vs T1D
1110	0.3723443	0.8415375	Secondary__bile__acid__biosynthesis__PATH__ko00121	LADA vs T2D
718	0.8869271	0.9047487	Secondary__bile__acid__biosynthesis__PATH__ko00121	T1D vs T2D

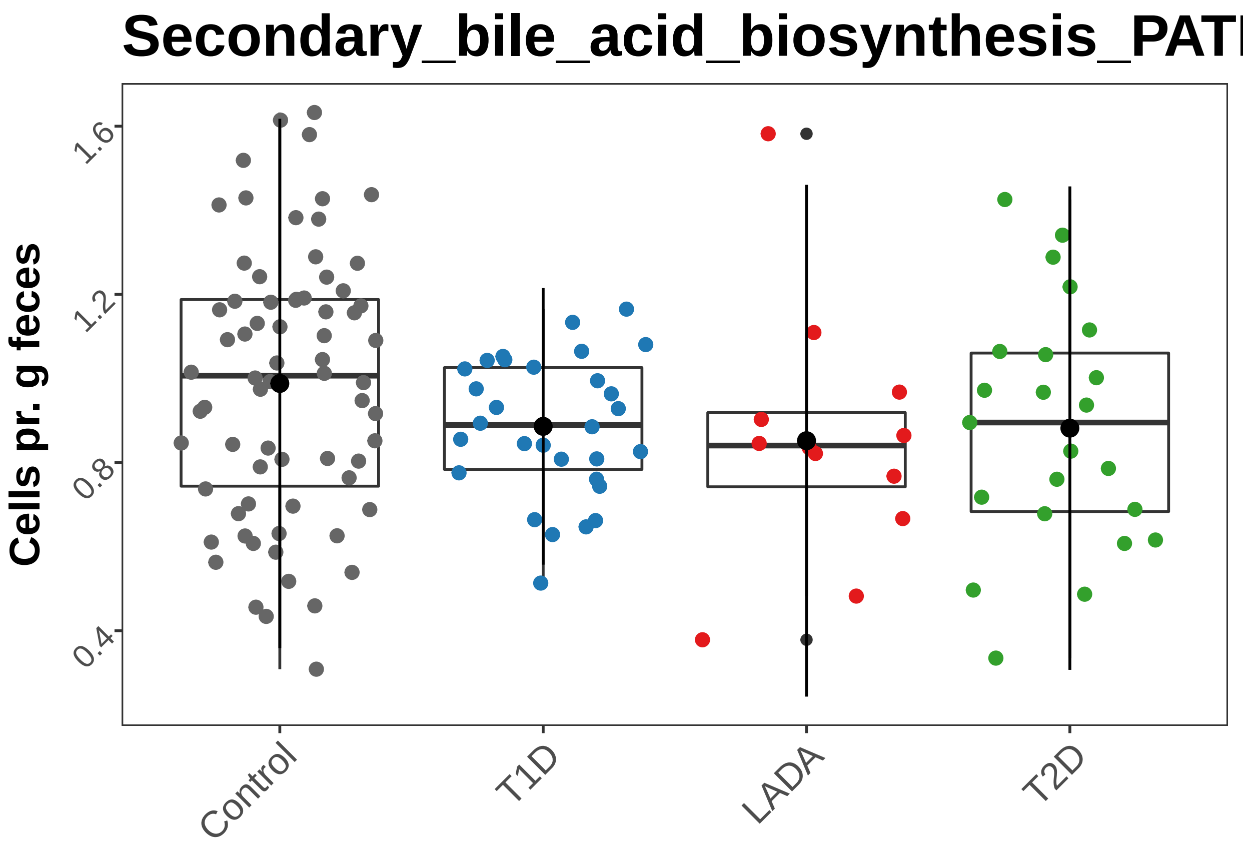

	pvalue	padj	Genus	compare
528	0.0269822	0.0574628	Folate_biosynthesis_PATH_ko00790	LADA vs Control
332	0.0950507	0.1425761	Folate_biosynthesis_PATH_ko00790	Control vs T2D
920	0.0215652	0.2312928	Folate_biosynthesis_PATH_ko00790	LADA vs T1D
724	0.0740582	0.6495082	Folate_biosynthesis_PATH_ko00790	T1D vs T2D
136	0.6671496	0.7652037	Folate_biosynthesis_PATH_ko00790	Control vs T1D
1116	0.4156975	0.8415375	Folate_biosynthesis_PATH_ko00790	LADA vs T2D

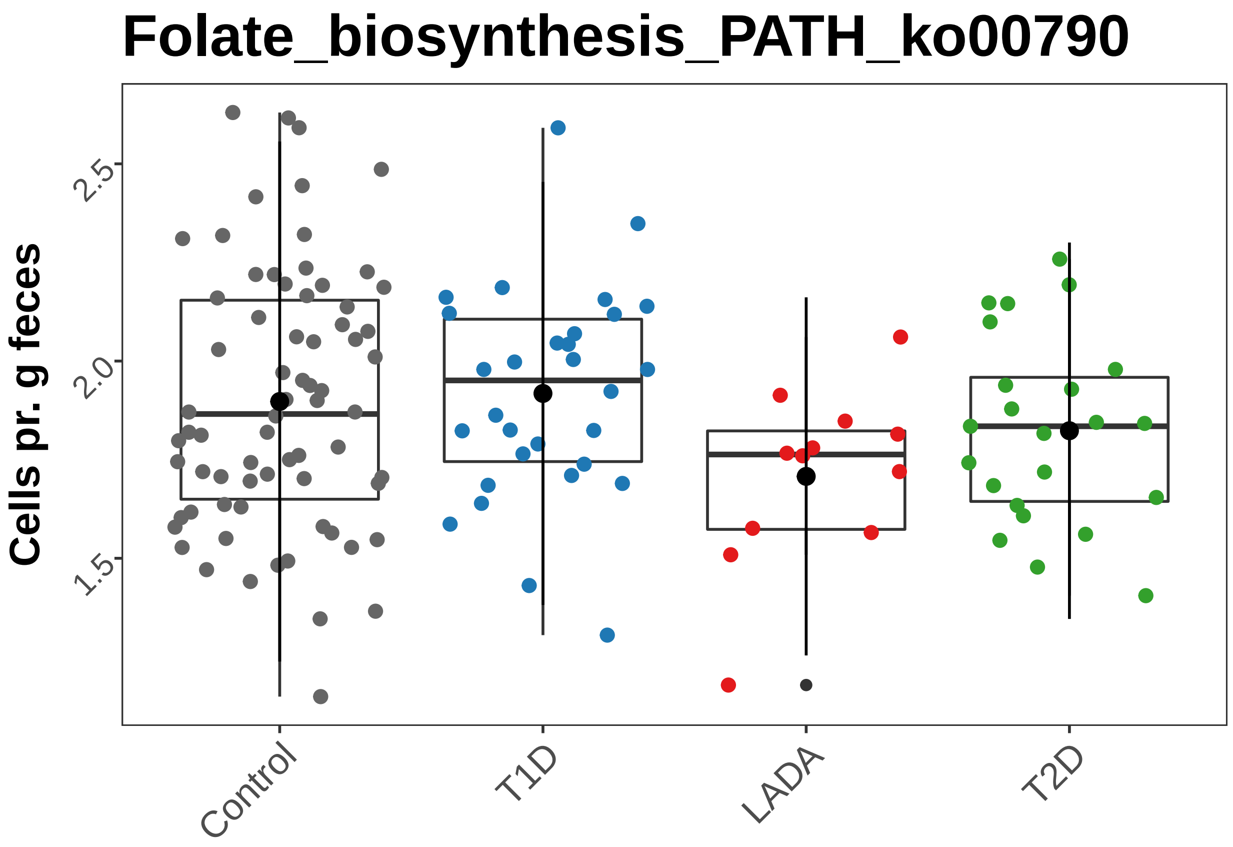

	pvalue	padj	Genus	compare
559	0.0272008	0.0574628	Zeatin_biosynthesis_PATH_ko00908	LADA vs Control
363	0.0474387	0.0828378	Zeatin_biosynthesis_PATH_ko00908	Control vs T2D
951	0.2980140	0.4359011	Zeatin_biosynthesis_PATH_ko00908	LADA vs T1D
167	0.1252058	0.4612224	Zeatin_biosynthesis_PATH_ko00908	Control vs T1D
755	0.6087806	0.7461523	Zeatin_biosynthesis_PATH_ko00908	T1D vs T2D
1147	0.5485906	0.8601900	Zeatin_biosynthesis_PATH_ko00908	LADA vs T2D

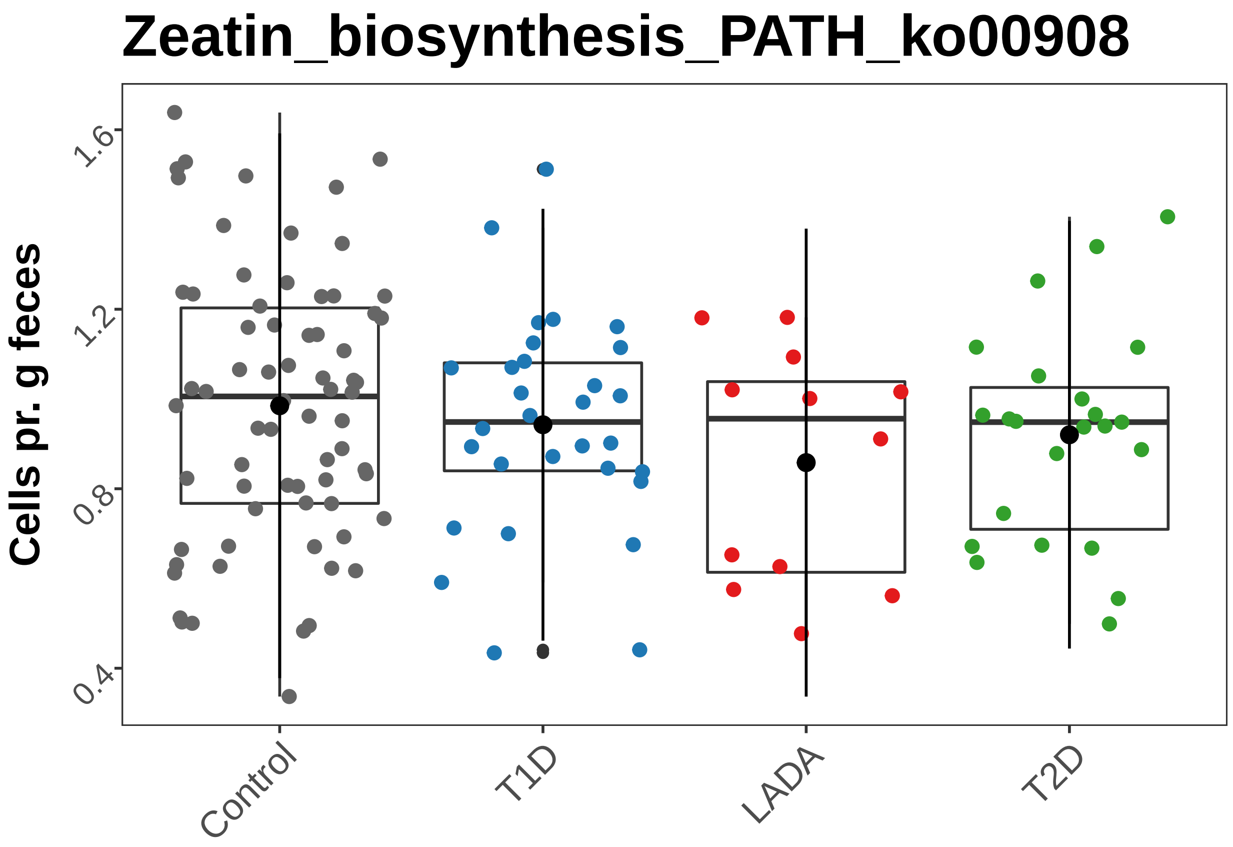

	pvalue	padj	Genus	compare
493	0.0275005	0.0574628	Carbon_fixation_pathways_in_prokaryotes_PATH_ko00720	LADA vs Control
297	0.0818376	0.1238826	Carbon_fixation_pathways_in_prokaryotes_PATH_ko00720	Control vs T2D
885	0.0427364	0.2312928	Carbon_fixation_pathways_in_prokaryotes_PATH_ko00720	LADA vs T1D
689	0.1282056	0.6495082	Carbon_fixation_pathways_in_prokaryotes_PATH_ko00720	T1D vs T2D
1081	0.4474880	0.8415375	Carbon_fixation_pathways_in_prokaryotes_PATH_ko00720	LADA vs T2D
101	0.9876412	0.9876412	Carbon_fixation_pathways_in_prokaryotes_PATH_ko00720	Control vs T1D

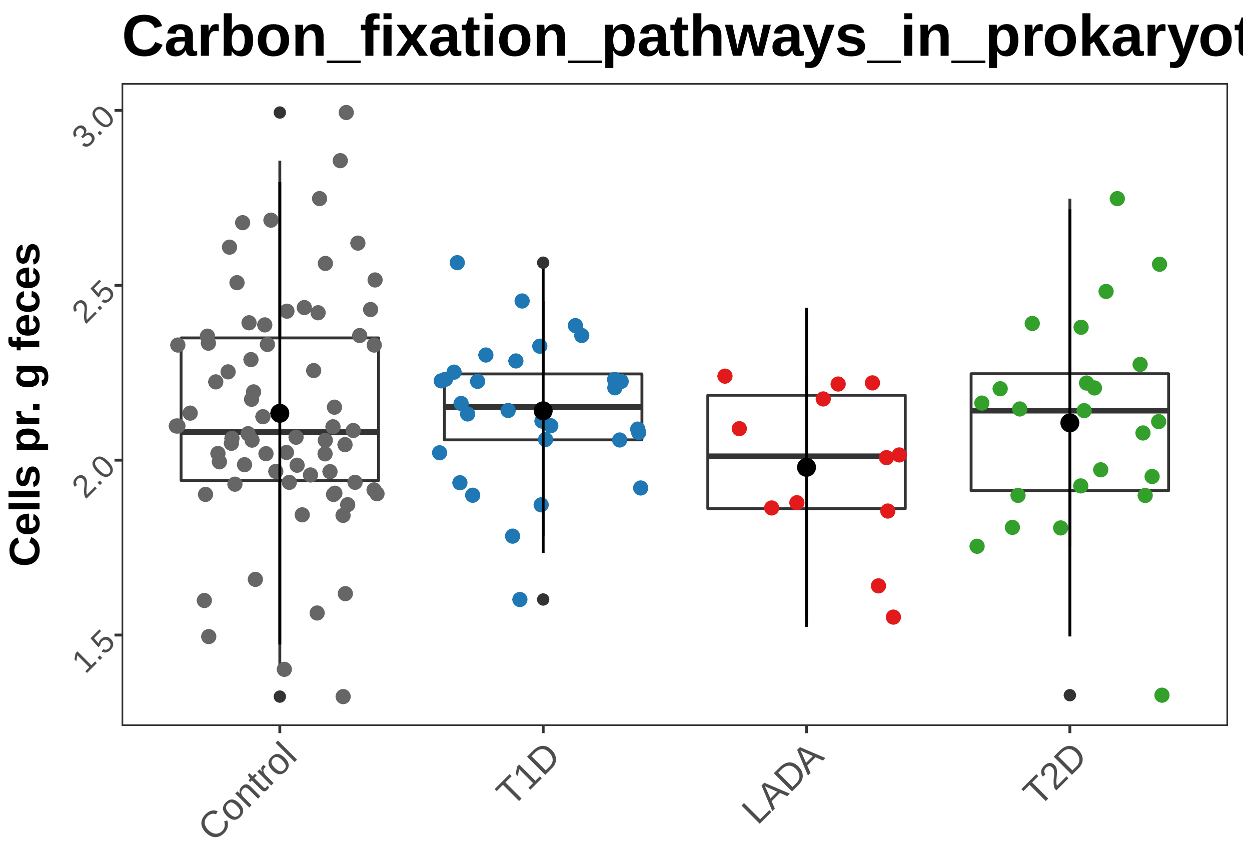

	pvalue	padj	Genus	compare
496	0.0275587	0.0574628	Oxidative_phosphorylation_PATH_ko00190	LADA vs Control
300	0.0400861	0.0767378	Oxidative_phosphorylation_PATH_ko00190	Control vs T2D
888	0.1044541	0.2527532	Oxidative_phosphorylation_PATH_ko00190	LADA vs T1D
692	0.1945877	0.6495082	Oxidative_phosphorylation_PATH_ko00190	T1D vs T2D
104	0.5395052	0.6911308	Oxidative_phosphorylation_PATH_ko00190	Control vs T1D
1084	0.5838217	0.8682081	Oxidative_phosphorylation_PATH_ko00190	LADA vs T2D

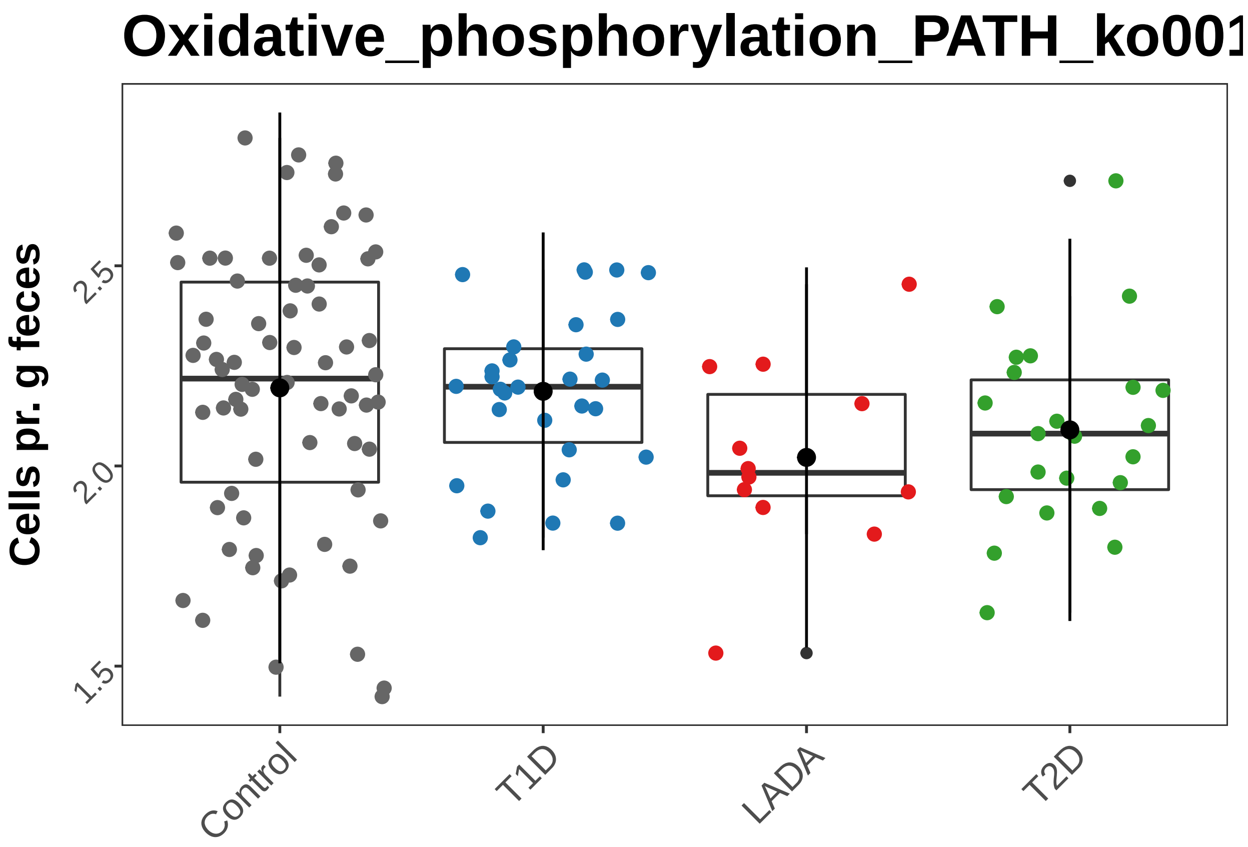

	pvalue	padj	Genus	compare
366	0.0041526	0.0629443	Aminobenzoate_degradation_PATH_ko00627	Control vs T2D
562	0.0881554	0.1350951	Aminobenzoate_degradation_PATH_ko00627	LADA vs Control
954	0.2649059	0.4051834	Aminobenzoate_degradation_PATH_ko00627	LADA vs T1D
170	0.4861562	0.6482083	Aminobenzoate_degradation_PATH_ko00627	Control vs T1D
758	0.0527060	0.6495082	Aminobenzoate_degradation_PATH_ko00627	T1D vs T2D
1150	0.6610626	0.8952178	Aminobenzoate_degradation_PATH_ko00627	LADA vs T2D

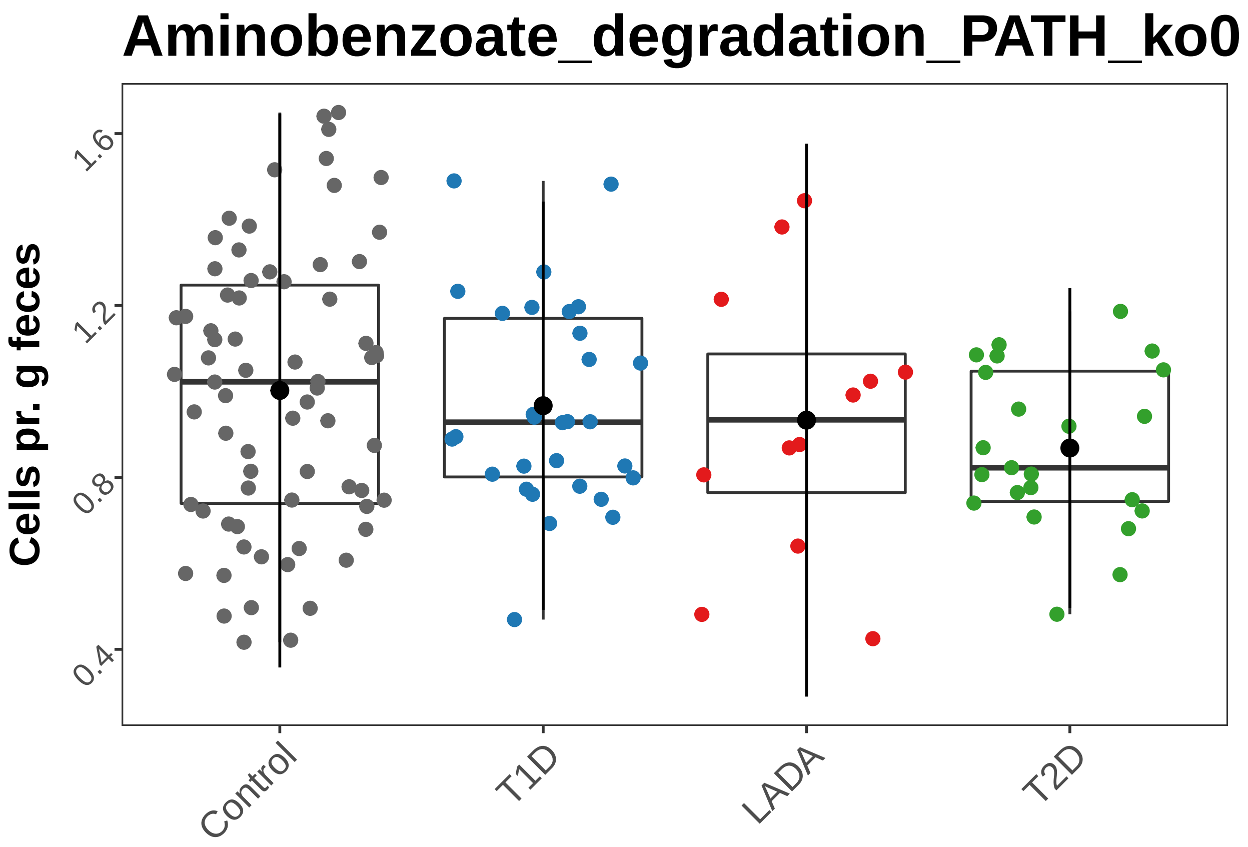

	pvalue	padj	Genus	compare
230	0.0045253	0.0629443	Two__component__system__PATH__ko02020	Control vs T2D
426	0.0483705	0.0831633	Two__component__system__PATH__ko02020	LADA vs Control
34	0.0030105	0.1382121	Two__component__system__PATH__ko02020	Control vs T1D
622	0.8996383	0.9089129	Two__component__system__PATH__ko02020	T1D vs T2D
1014	0.8539672	0.9483589	Two__component__system__PATH__ko02020	LADA vs T2D
818	0.9286360	0.9691189	Two__component__system__PATH__ko02020	LADA vs T1D

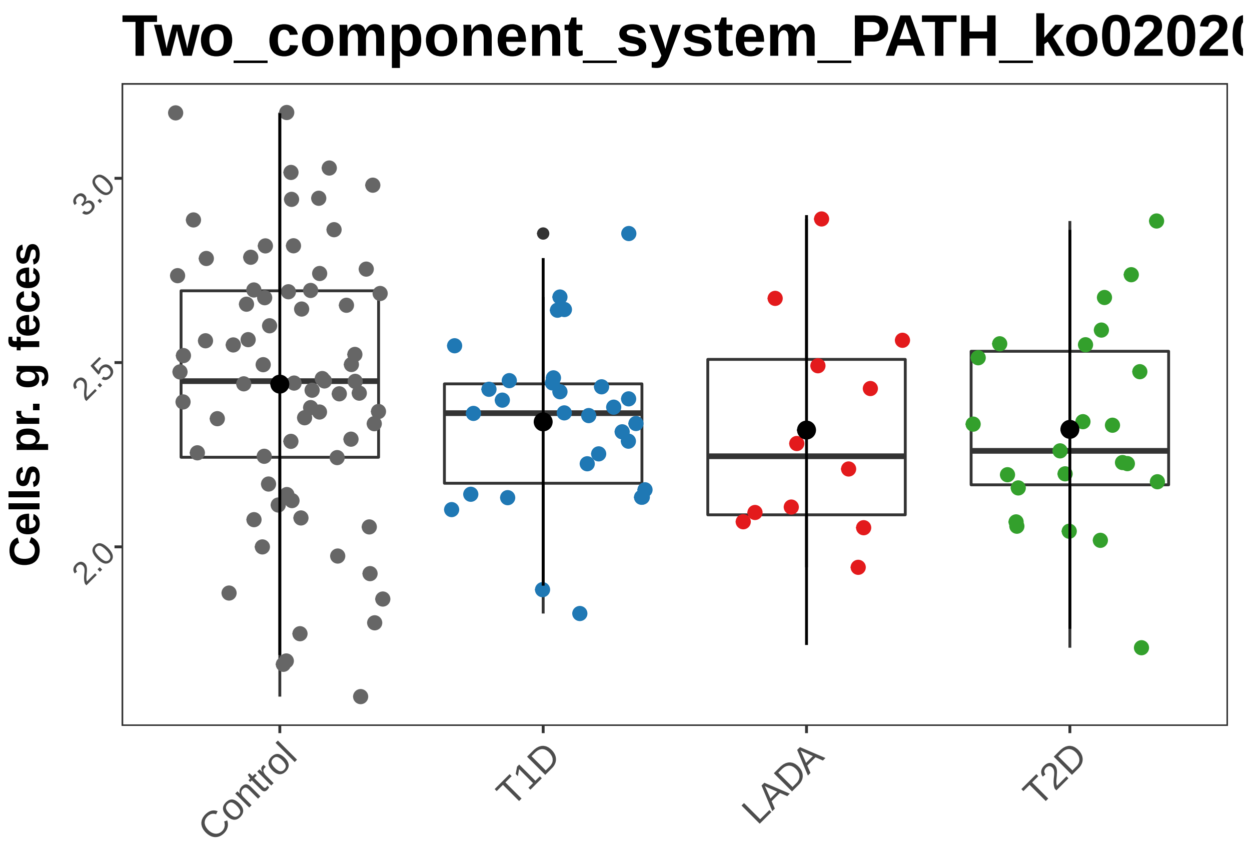

	pvalue	padj	Genus	compare
207	0.0046159	0.0629443	Biofilm_formation____Escherichia_coli_PATH_ko02026	Control vs T2D
11	0.0034702	0.1382121	Biofilm_formation____Escherichia_coli_PATH_ko02026	Control vs T1D
403	0.1146559	0.1689666	Biofilm_formation____Escherichia_coli_PATH_ko02026	LADA vs Control
795	0.6714485	0.7833566	Biofilm_formation____Escherichia_coli_PATH_ko02026	LADA vs T1D
991	0.5977305	0.8808660	Biofilm_formation____Escherichia_coli_PATH_ko02026	LADA vs T2D
599	0.8766262	0.9043091	Biofilm_formation____Escherichia_coli_PATH_ko02026	T1D vs T2D

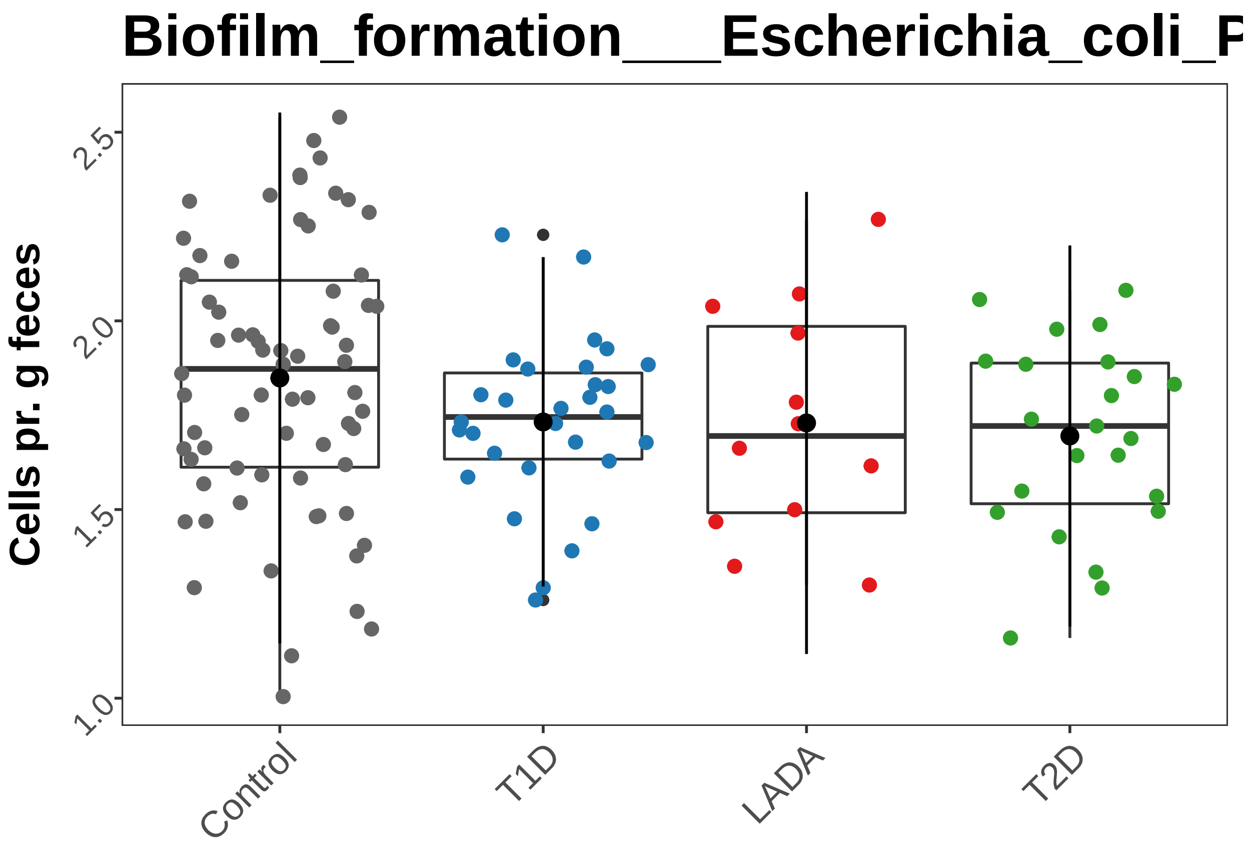

	pvalue	padj	Genus	compare
302	0.0071873	0.0629443	Sulfur__metabolism__PATH__ko00920	Control vs T2D
498	0.0380020	0.0735070	Sulfur__metabolism__PATH__ko00920	LADA vs Control
106	0.0384875	0.3989340	Sulfur__metabolism__PATH__ko00920	Control vs T1D
694	0.4829973	0.6743371	Sulfur__metabolism__PATH__ko00920	T1D vs T2D
890	0.5648333	0.6828644	Sulfur__metabolism__PATH__ko00920	LADA vs T1D
1086	0.9950402	0.9986549	Sulfur__metabolism__PATH__ko00920	LADA vs T2D

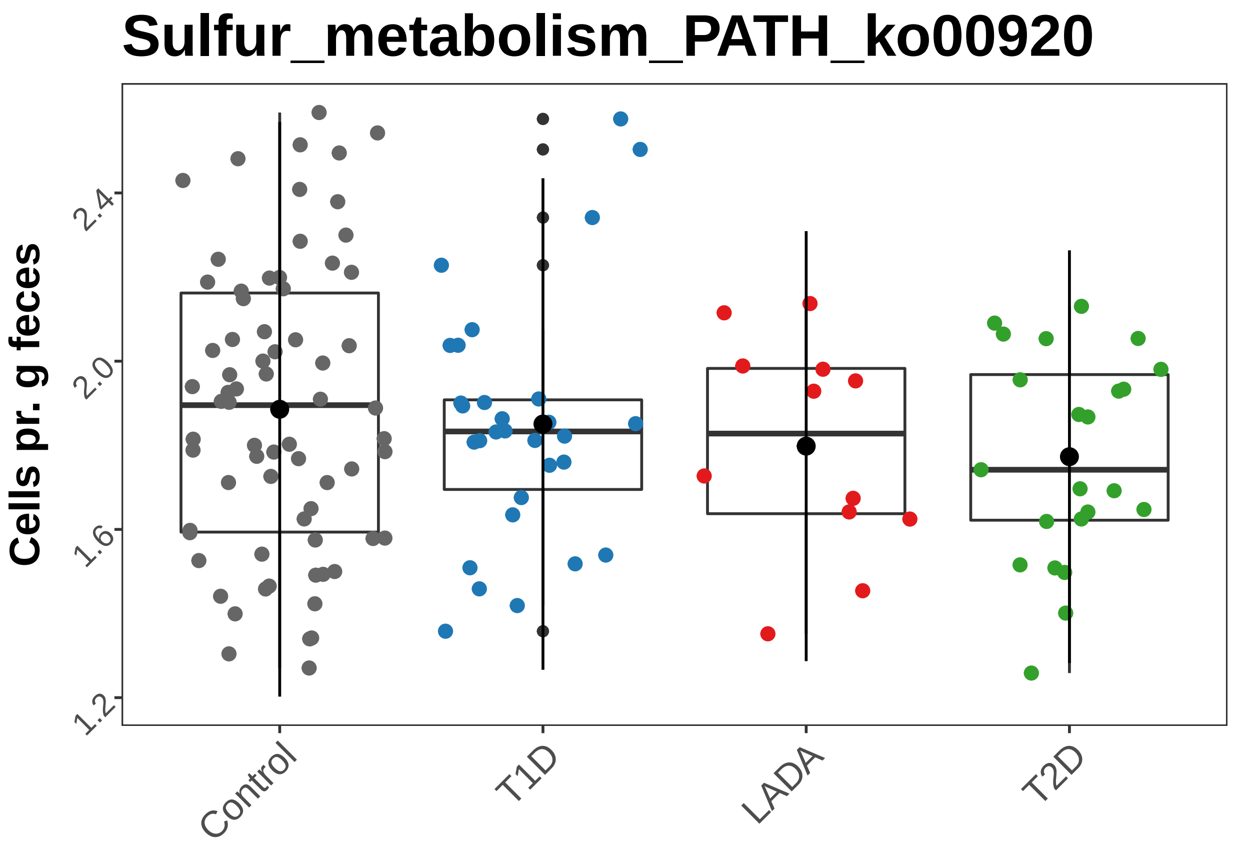

	pvalue	padj	Genus	compare
370	0.0081464	0.0629443	Chloroalkane_and_chloroalkene_degradation_PATH_ko00625	Control vs T2D
566	0.0352832	0.0705663	Chloroalkane_and_chloroalkene_degradation_PATH_ko00625	LADA vs Control
958	0.2030764	0.3597318	Chloroalkane_and_chloroalkene_degradation_PATH_ko00625	LADA vs T1D
174	0.3069114	0.5208704	Chloroalkane_and_chloroalkene_degradation_PATH_ko00625	Control vs T1D
762	0.1362050	0.6495082	Chloroalkane_and_chloroalkene_degradation_PATH_ko00625	T1D vs T2D
1154	0.9512310	0.9986549	Chloroalkane_and_chloroalkene_degradation_PATH_ko00625	LADA vs T2D

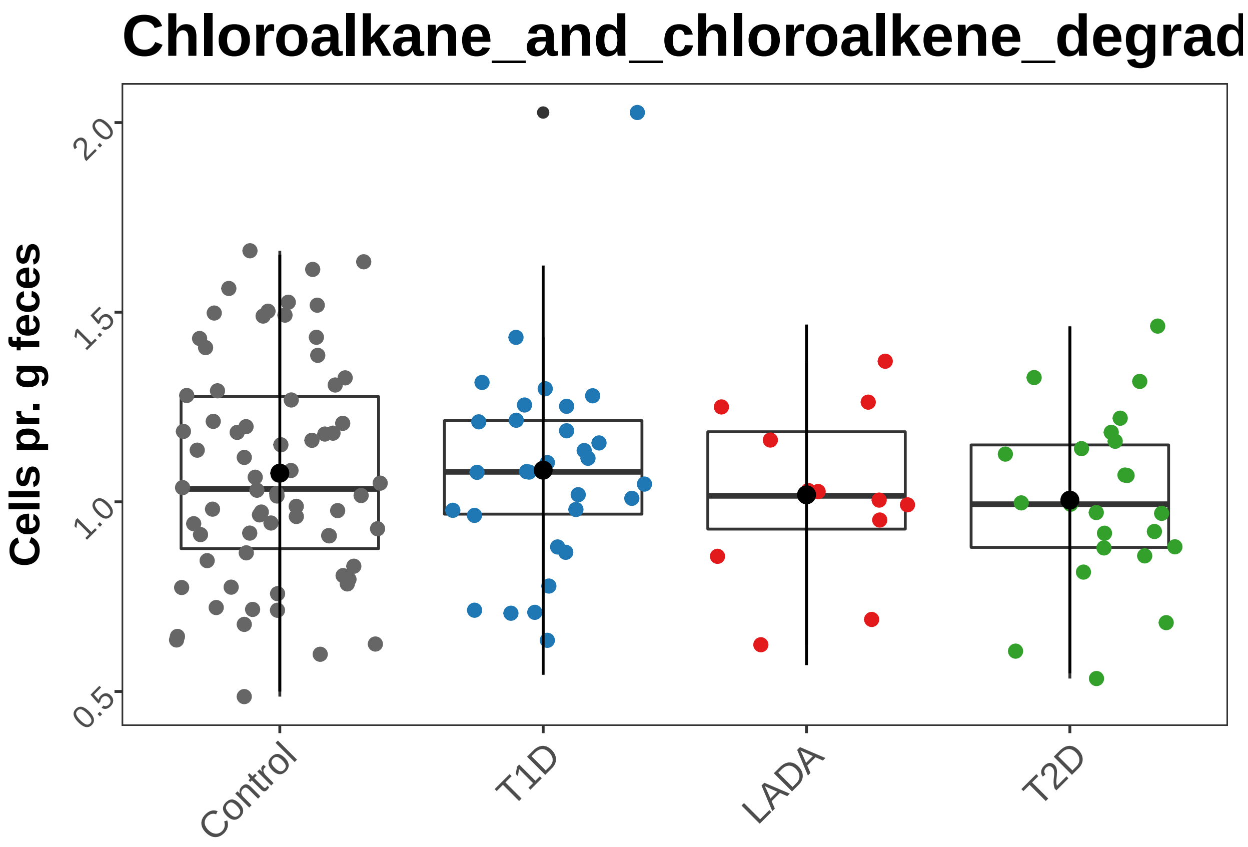

	pvalue	padj	Genus	compare
345	0.0096014	0.0629443	D_Alanine_metabolism_PATH_ko00473	Control vs T2D
541	0.0311438	0.0642547	D_Alanine_metabolism_PATH_ko00473	LADA vs Control
933	0.2866482	0.4244349	D_Alanine_metabolism_PATH_ko00473	LADA vs T1D
149	0.1561826	0.4612224	D_Alanine_metabolism_PATH_ko00473	Control vs T1D
737	0.2587296	0.6495082	D_Alanine_metabolism_PATH_ko00473	T1D vs T2D
1129	0.8862870	0.9704595	D_Alanine_metabolism_PATH_ko00473	LADA vs T2D

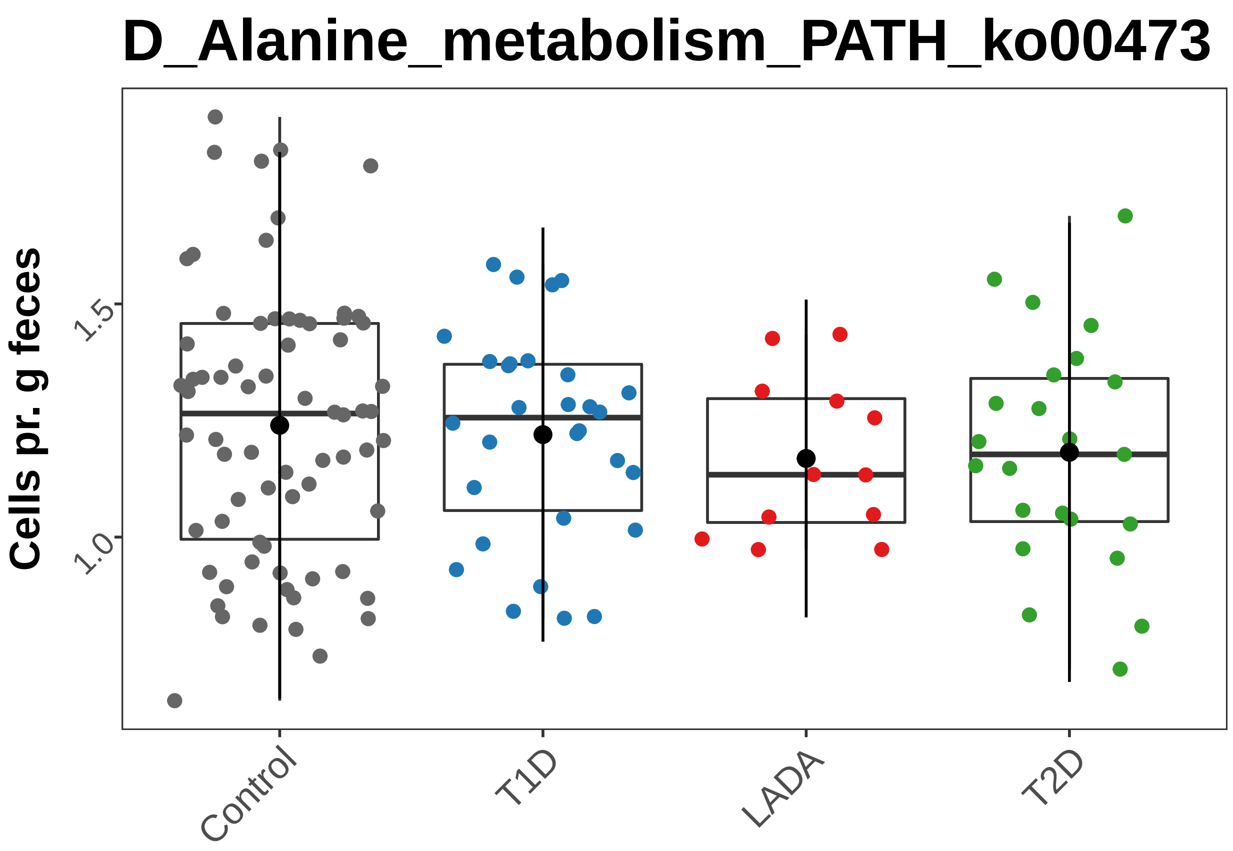

	pvalue	padj	Genus	compare
208	0.0129311	0.0629443	Biofilm_formation____Pseudomonas_aeruginosa_PATH_ko02025	Control vs T2D
12	0.0035258	0.1382121	Biofilm_formation____Pseudomonas_aeruginosa_PATH_ko02025	Control vs T1D
404	0.1049697	0.1570539	Biofilm_formation____Pseudomonas_aeruginosa_PATH_ko02025	LADA vs Control
796	0.7031251	0.8154587	Biofilm_formation____Pseudomonas_aeruginosa_PATH_ko02025	LADA vs T1D
600	0.8872420	0.9047487	Biofilm_formation____Pseudomonas_aeruginosa_PATH_ko02025	T1D vs T2D
992	0.7985782	0.9100077	Biofilm_formation____Pseudomonas_aeruginosa_PATH_ko02025	LADA vs T2D

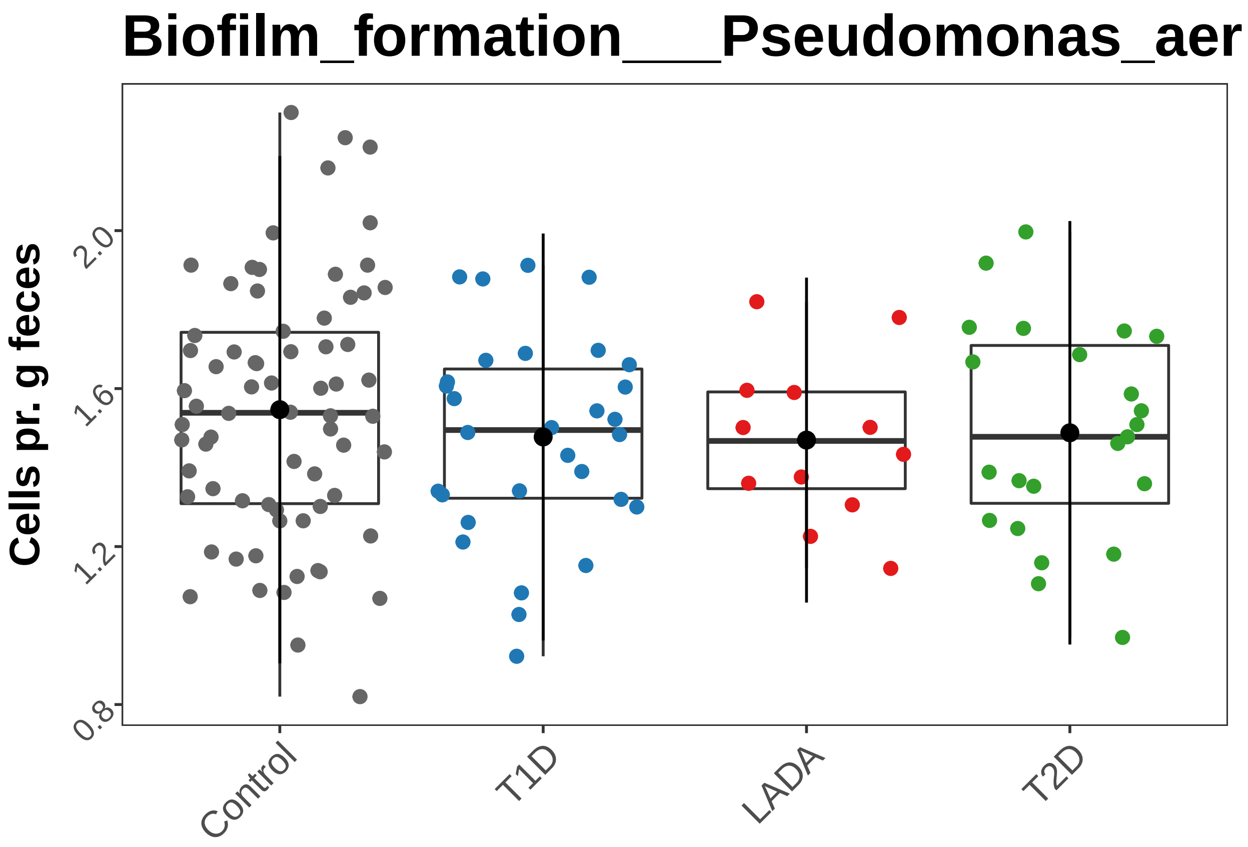

	pvalue	padj	Genus	compare
217	0.0148552	0.0629443	Bacterial_secretion_system_PATH_ko03070	Control vs T2D
413	0.0401759	0.0749949	Bacterial_secretion_system_PATH_ko03070	LADA vs Control
21	0.1017852	0.4612224	Bacterial_secretion_system_PATH_ko03070	Control vs T1D
805	0.4056344	0.5483058	Bacterial_secretion_system_PATH_ko03070	LADA vs T1D
609	0.4099136	0.6531957	Bacterial_secretion_system_PATH_ko03070	T1D vs T2D
1001	0.8757910	0.9643542	Bacterial_secretion_system_PATH_ko03070	LADA vs T2D

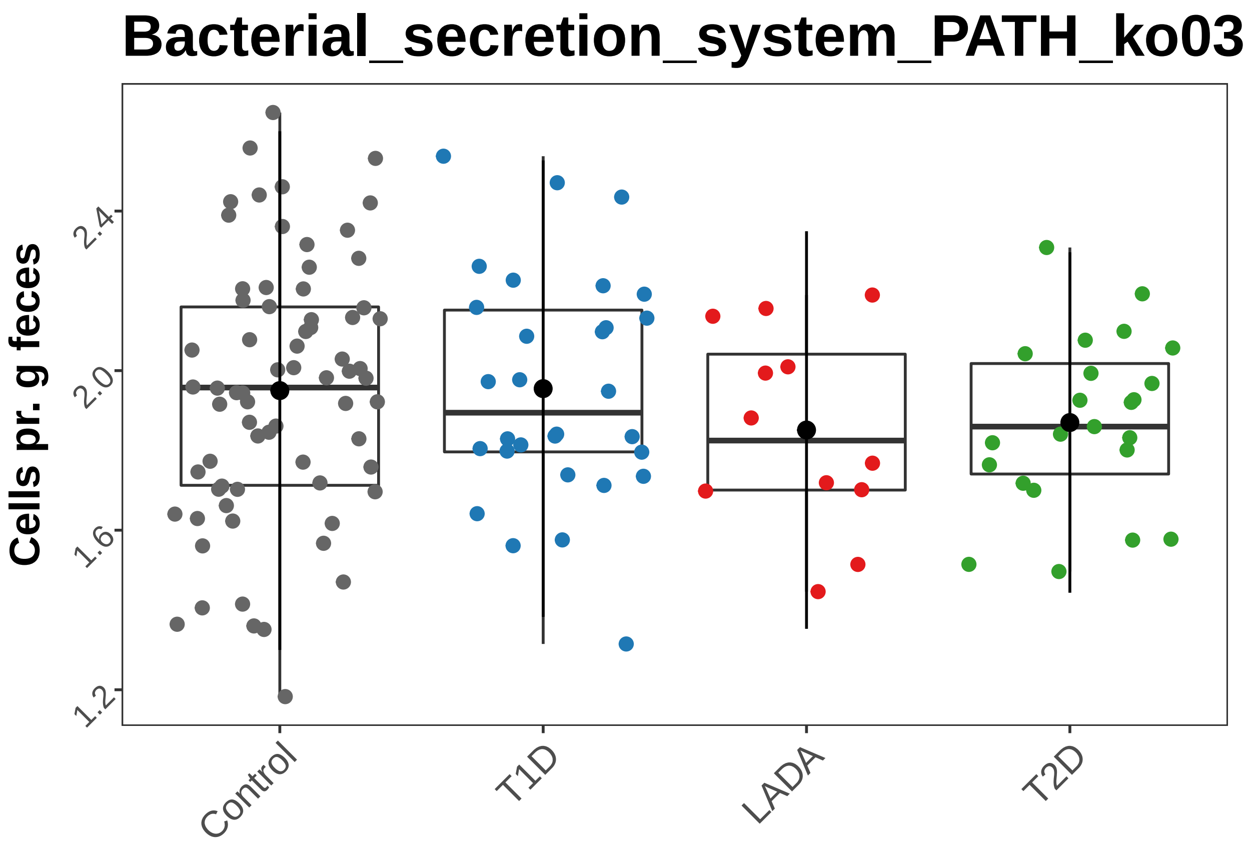

	pvalue	padj	Genus	compare
378	0.0152764	0.0629443	Naphthalene_degradation_PATH_ko00626	Control vs T2D
574	0.1302975	0.1850602	Naphthalene_degradation_PATH_ko00626	LADA vs Control
182	0.1750750	0.4762288	Naphthalene_degradation_PATH_ko00626	Control vs T1D
770	0.3002635	0.6495082	Naphthalene_degradation_PATH_ko00626	T1D vs T2D
966	0.6047801	0.7184054	Naphthalene_degradation_PATH_ko00626	LADA vs T1D
1162	0.7567110	0.9035121	Naphthalene_degradation_PATH_ko00626	LADA vs T2D

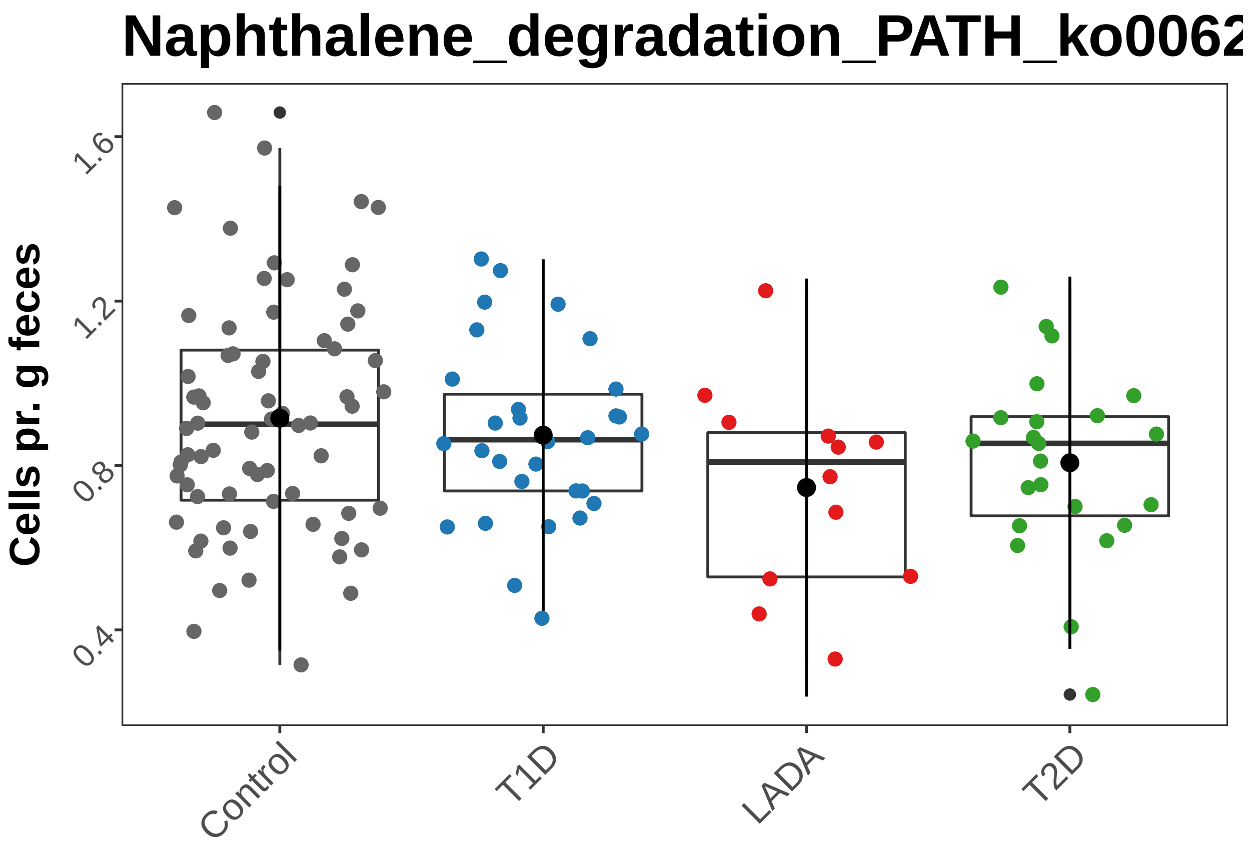

	pvalue	padj	Genus	compare
467	0.0315114	0.0643358	Isoquinoline_alkaloid_biosynthesis_PATH_ko00950	LADA vs Control
271	0.1240067	0.1719420	Isoquinoline_alkaloid_biosynthesis_PATH_ko00950	Control vs T2D
859	0.2479259	0.3950690	Isoquinoline_alkaloid_biosynthesis_PATH_ko00950	LADA vs T1D
75	0.2038661	0.4786856	Isoquinoline_alkaloid_biosynthesis_PATH_ko00950	Control vs T1D
663	0.7388067	0.8181137	Isoquinoline_alkaloid_biosynthesis_PATH_ko00950	T1D vs T2D
1055	0.3960348	0.8415375	Isoquinoline_alkaloid_biosynthesis_PATH_ko00950	LADA vs T2D

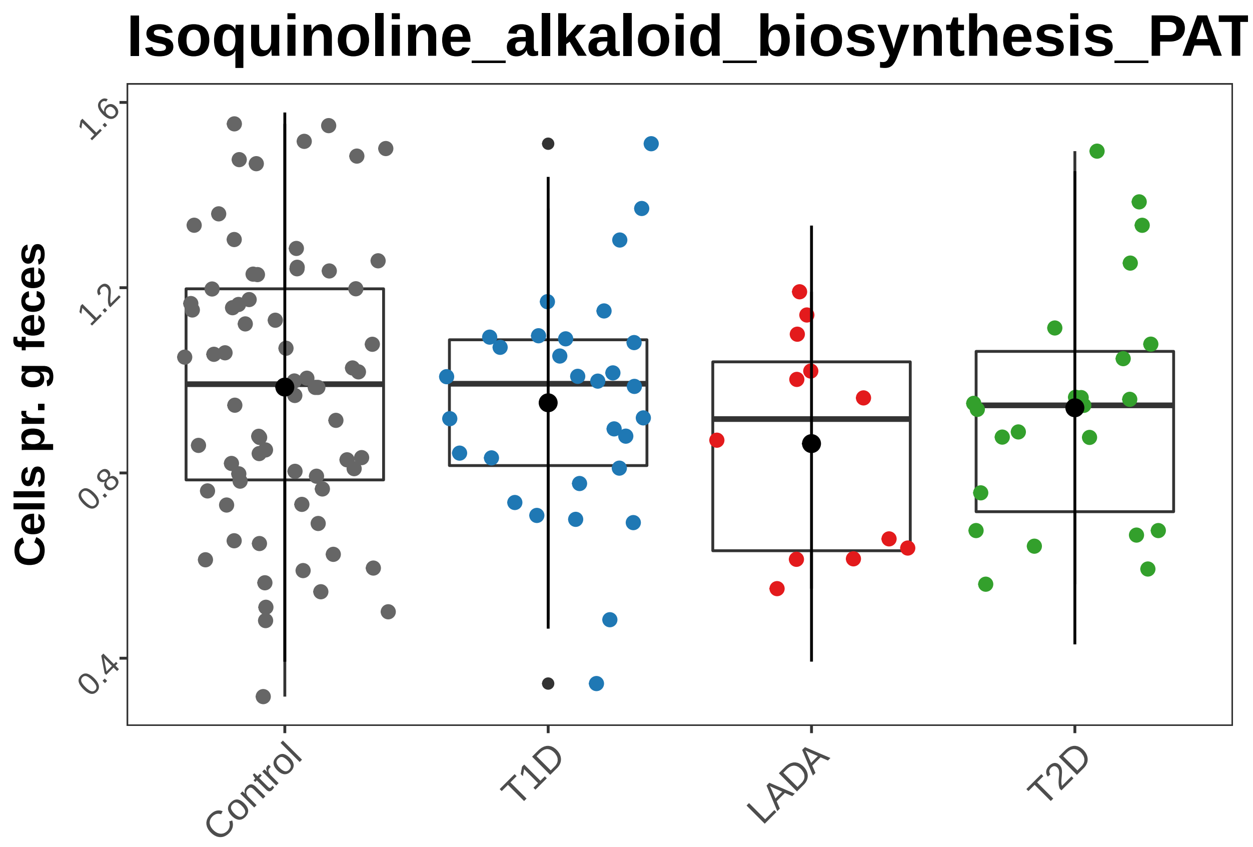

	pvalue	padj	Genus	compare
267	0.0196938	0.0661577	Biosynthesis_of_various_secondary_metabolites_____part_1_PATH_ko00999	Control vs T2D
463	0.0768995	0.1205785	Biosynthesis_of_various_secondary_metabolites_____part_1_PATH_ko00999	LADA vs Control
71	0.1072874	0.4612224	Biosynthesis_of_various_secondary_metabolites_____part_1_PATH_ko00999	Control vs T1D
659	0.4506432	0.6743371	Biosynthesis_of_various_secondary_metabolites_____part_1_PATH_ko00999	T1D vs T2D
855	0.5557227	0.6765319	Biosynthesis_of_various_secondary_metabolites_____part_1_PATH_ko00999	LADA vs T1D
1051	0.9824926	0.9986549	Biosynthesis_of_various_secondary_metabolites_____part_1_PATH_ko00999	LADA vs T2D

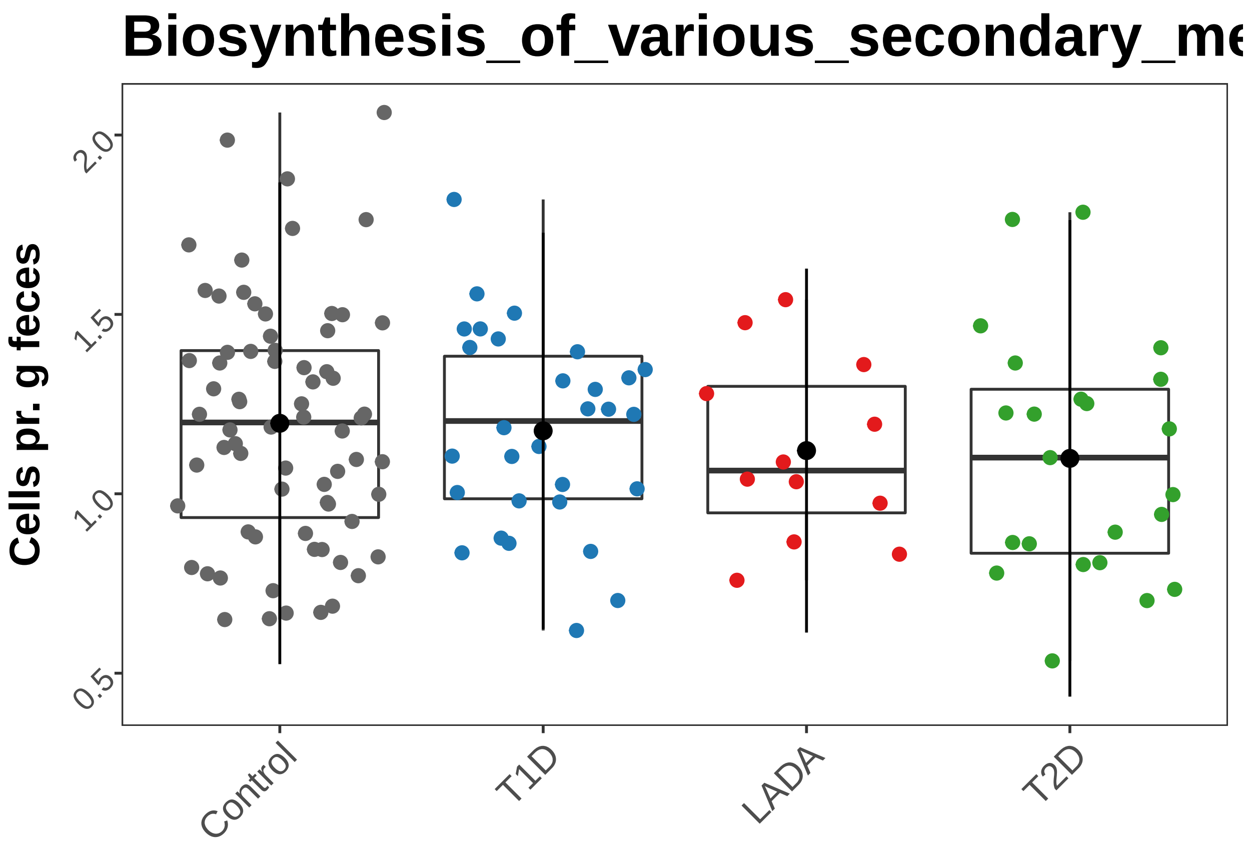

	pvalue	padj	Genus	compare
278	0.0248215	0.0676342	Prodigiosin_biosynthesis_PATH_ko00333	Control vs T2D
474	0.0378937	0.0735070	Prodigiosin_biosynthesis_PATH_ko00333	LADA vs Control
866	0.2091879	0.3628392	Prodigiosin_biosynthesis_PATH_ko00333	LADA vs T1D
82	0.3140599	0.5216587	Prodigiosin_biosynthesis_PATH_ko00333	Control vs T1D
670	0.2487250	0.6495082	Prodigiosin_biosynthesis_PATH_ko00333	T1D vs T2D
1062	0.7589838	0.9035121	Prodigiosin_biosynthesis_PATH_ko00333	LADA vs T2D

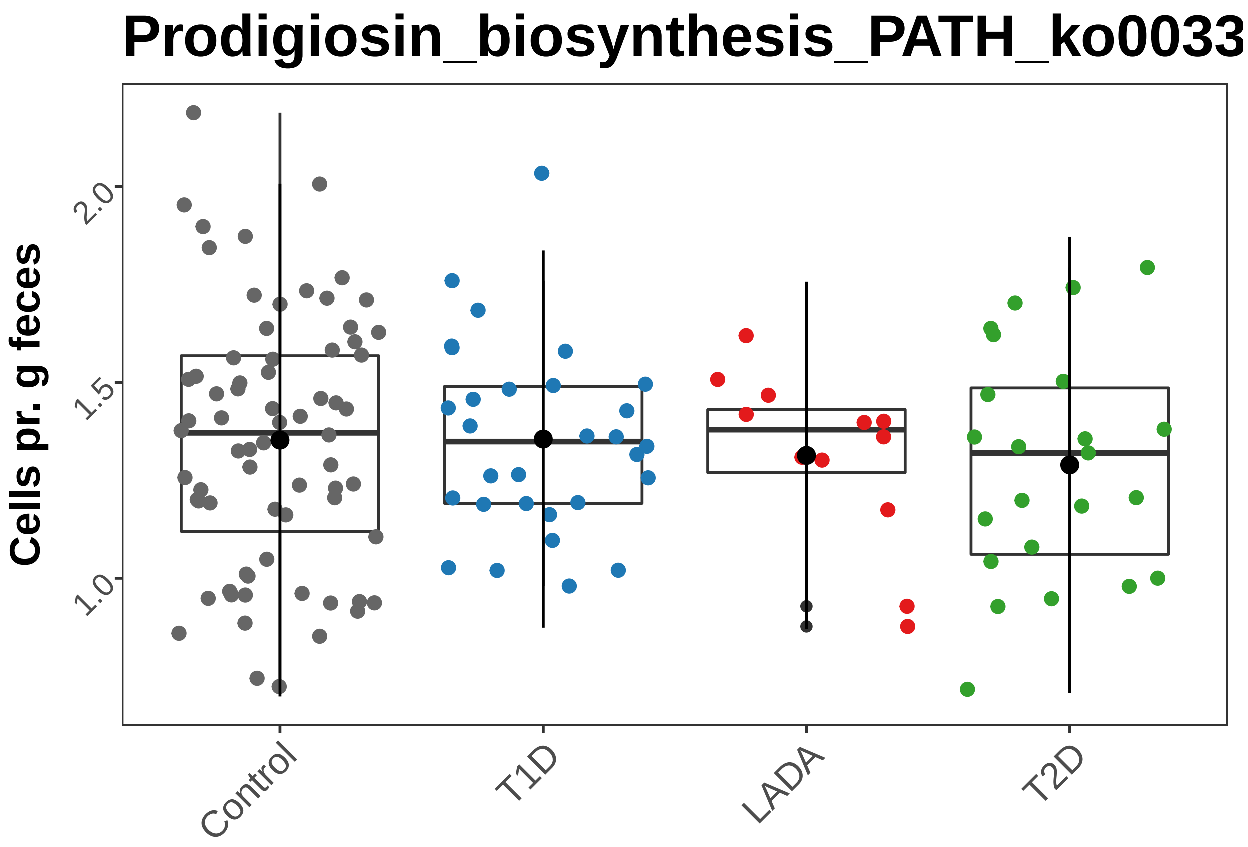

	pvalue	padj	Genus	compare
368	0.0257934	0.0676342	Benzoate_degradation_PATH_ko00362	Control vs T2D
564	0.0469021	0.0820786	Benzoate_degradation_PATH_ko00362	LADA vs Control
172	0.1356570	0.4612224	Benzoate_degradation_PATH_ko00362	Control vs T1D
956	0.3873540	0.5346576	Benzoate_degradation_PATH_ko00362	LADA vs T1D
760	0.4482381	0.6743371	Benzoate_degradation_PATH_ko00362	T1D vs T2D
1152	0.8111711	0.9163617	Benzoate_degradation_PATH_ko00362	LADA vs T2D

Benzoate_degradation_PATH_ko00362

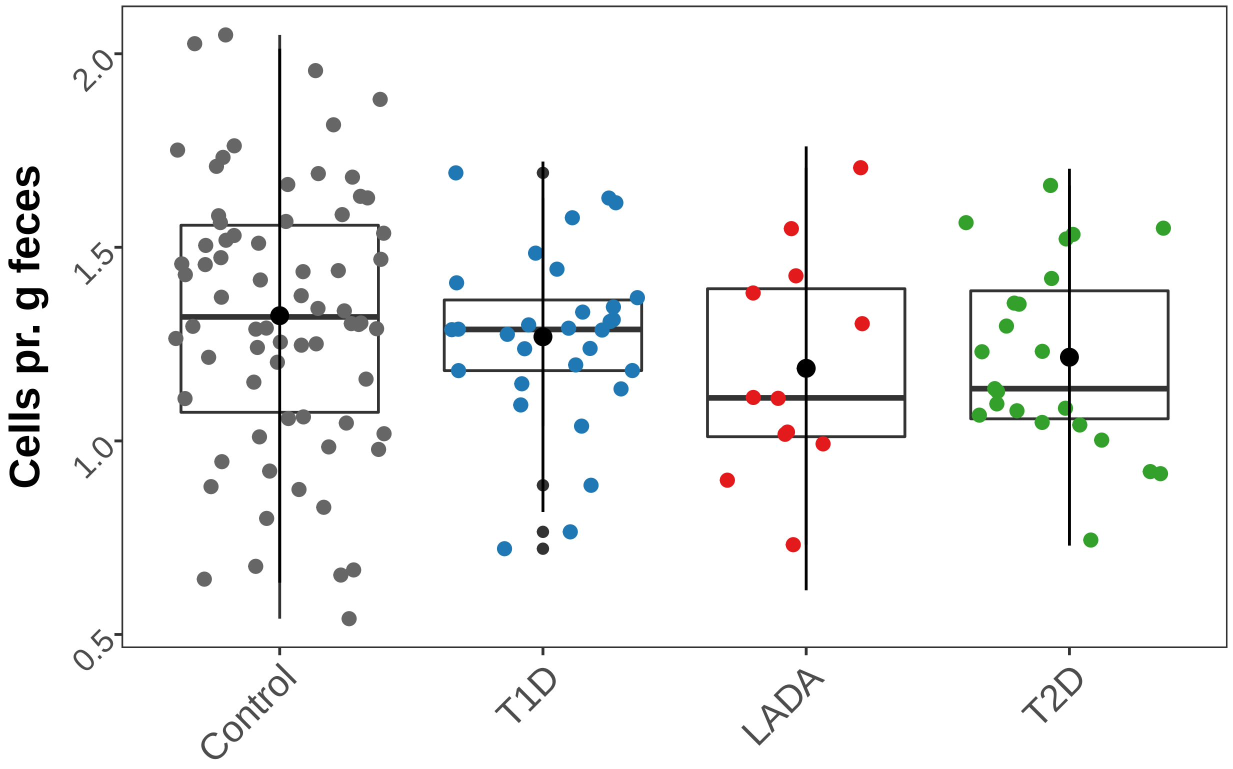

	pvalue	padj	Genus	compare
374	0.0275547	0.0676342	Drug_metabolism___other_enzymes_PATH_ko00983	Control vs T2D
570	0.0448924	0.0807239	Drug_metabolism___other_enzymes_PATH_ko00983	LADA vs Control
962	0.2662998	0.4051834	Drug_metabolism___other_enzymes_PATH_ko00983	LADA vs T1D
178	0.2577608	0.4839499	Drug_metabolism___other_enzymes_PATH_ko00983	Control vs T1D
766	0.3077980	0.6495082	Drug_metabolism___other_enzymes_PATH_ko00983	T1D vs T2D
1158	0.7852353	0.9053301	Drug_metabolism___other_enzymes_PATH_ko00983	LADA vs T2D

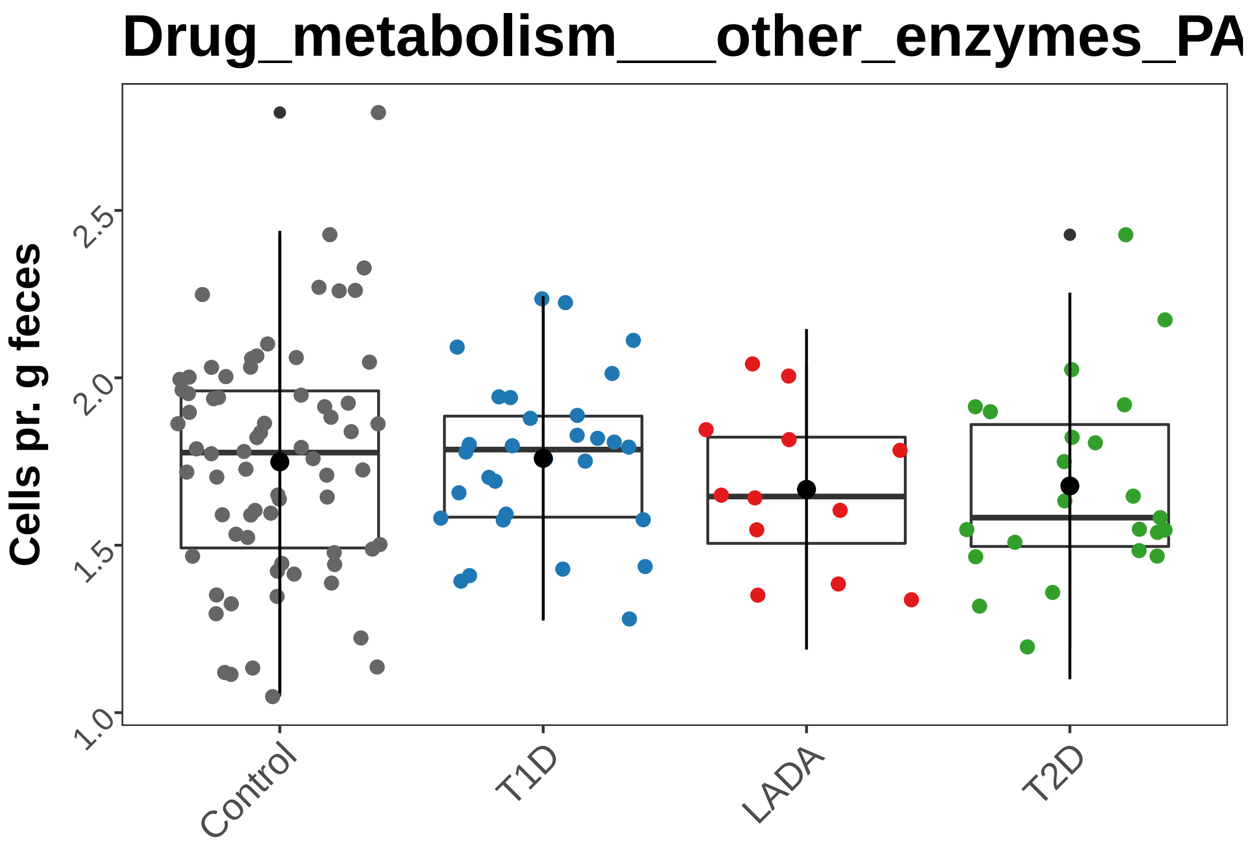

	pvalue	padj	Genus	compare
389	0.0276979	0.0676342	Degradation_of_aromatic_compounds	Control vs T2D
585	0.0386287	0.0735070	Degradation_of_aromatic_compounds	LADA vs Control
977	0.2460279	0.3950690	Degradation_of_aromatic_compounds	LADA vs T1D
193	0.2520755	0.4839499	Degradation_of_aromatic_compounds	Control vs T1D
781	0.3137589	0.6495082	Degradation_of_aromatic_compounds	T1D vs T2D
1173	0.7424051	0.9035121	Degradation_of_aromatic_compounds	LADA vs T2D

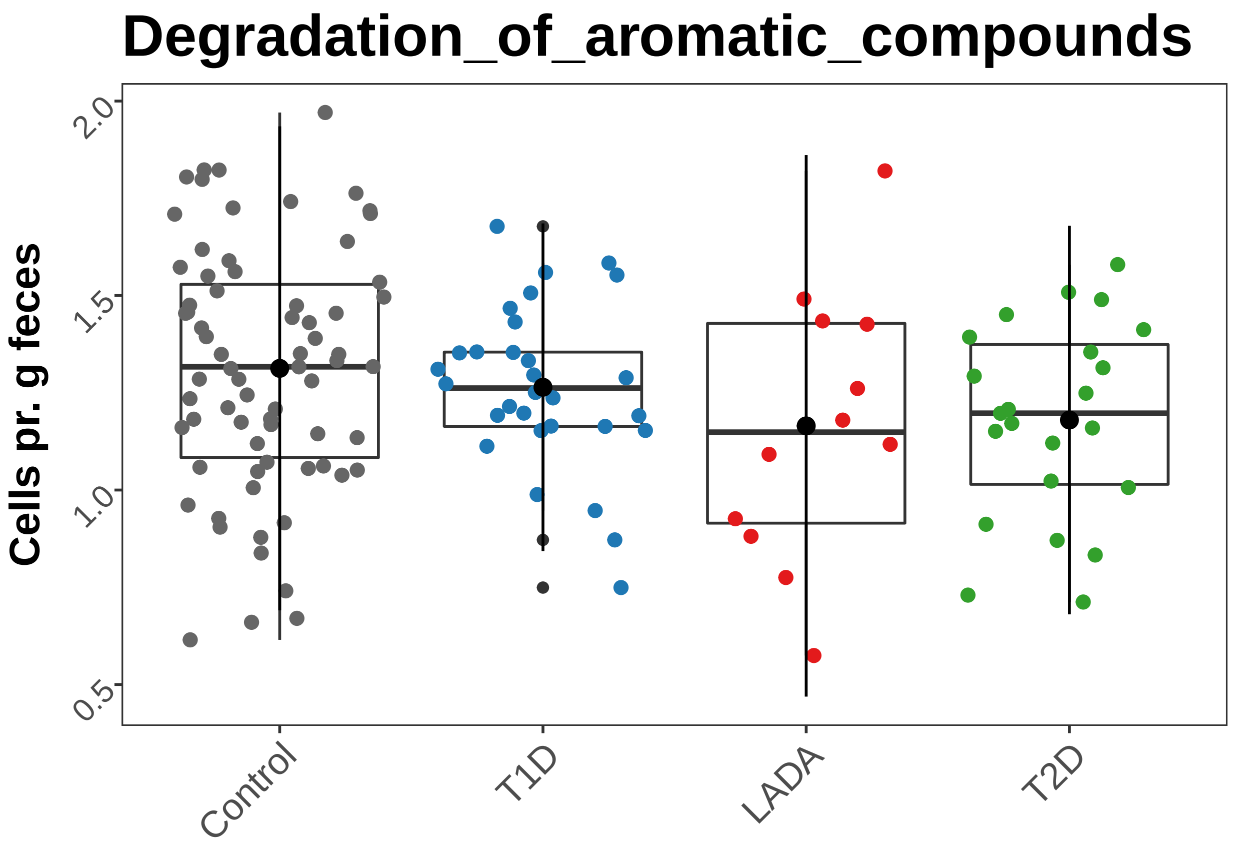

	pvalue	padj	Genus	compare
209	0.0293667	0.0676342	Biofilm_formation____Vibrio_cholerae_PATH_ko05111	Control vs T2D
405	0.0518124	0.0867968	Biofilm_formation____Vibrio_cholerae_PATH_ko05111	LADA vs Control
13	0.1151252	0.4612224	Biofilm_formation____Vibrio_cholerae_PATH_ko05111	Control vs T1D
797	0.4399535	0.5826411	Biofilm_formation____Vibrio_cholerae_PATH_ko05111	LADA vs T1D
601	0.5165646	0.6862683	Biofilm_formation____Vibrio_cholerae_PATH_ko05111	T1D vs T2D
993	0.8135048	0.9163617	Biofilm_formation____Vibrio_cholerae_PATH_ko05111	LADA vs T2D

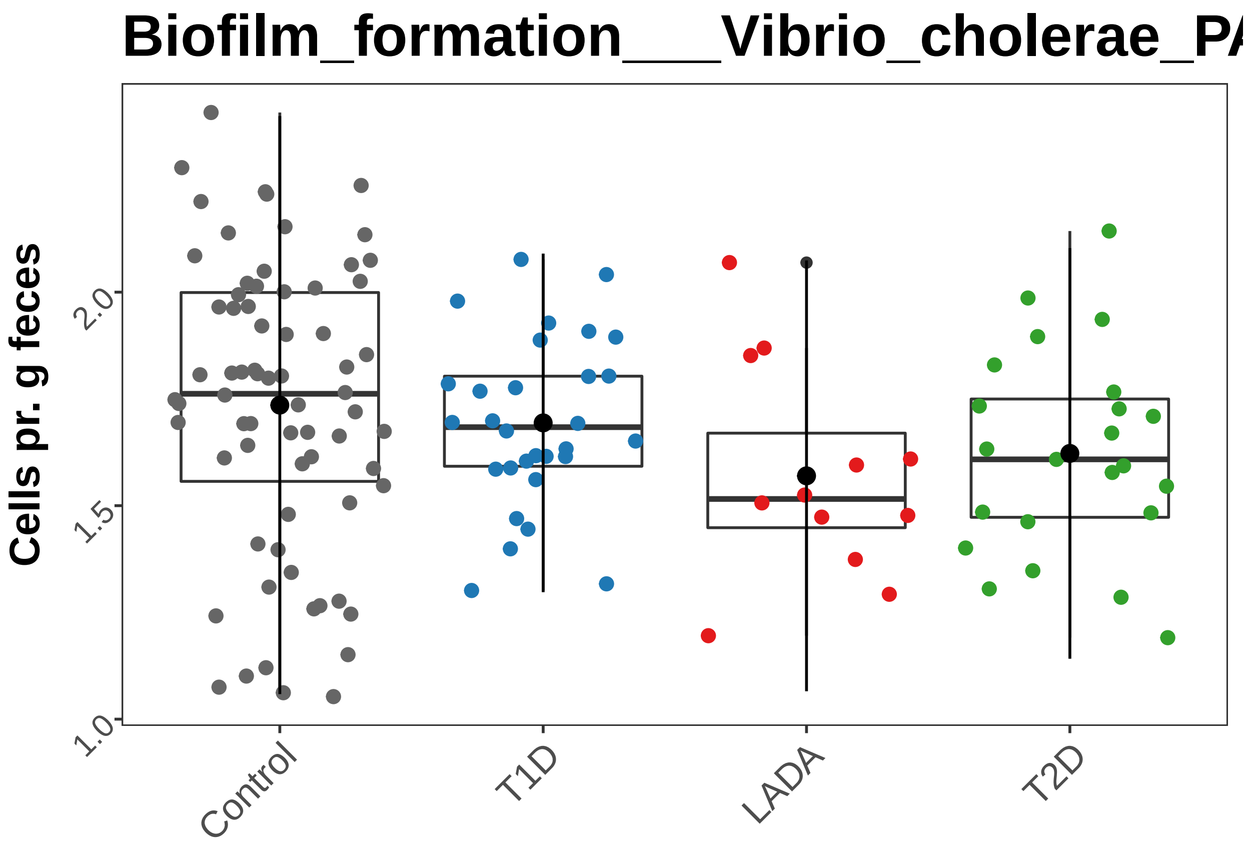

	pvalue	padj	Genus	compare
331	0.0312298	0.0681056	Biotin_metabolism_PATH_ko00780	Control vs T2D
527	0.0480627	0.0831633	Biotin_metabolism_PATH_ko00780	LADA vs Control
919	0.2456691	0.3950690	Biotin_metabolism_PATH_ko00780	LADA vs T1D
135	0.3109569	0.5209193	Biotin_metabolism_PATH_ko00780	Control vs T1D
723	0.2844967	0.6495082	Biotin_metabolism_PATH_ko00780	T1D vs T2D
1115	0.7790487	0.9035121	Biotin_metabolism_PATH_ko00780	LADA vs T2D

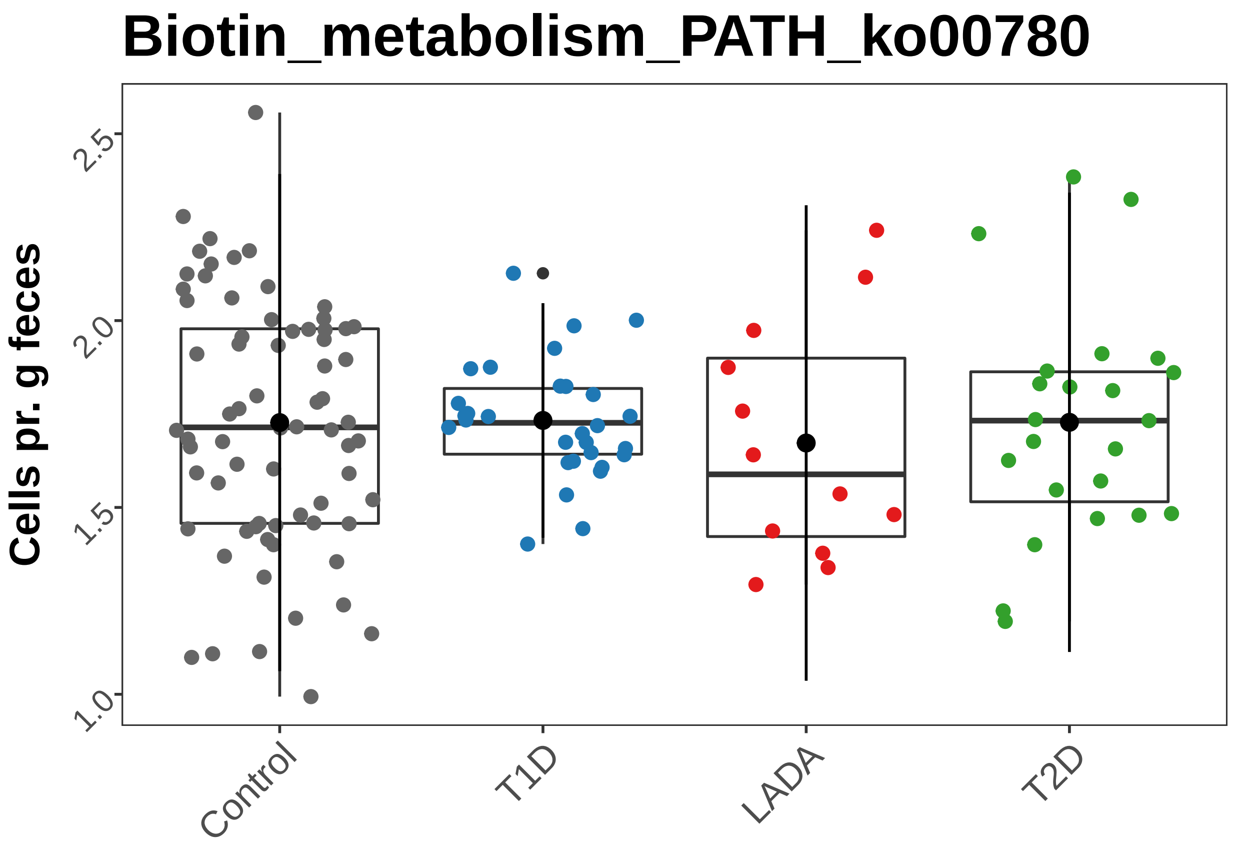

	pvalue	padj	Genus	compare
252	0.0313699	0.0681056	Arginine_and_proline_metabolism_PATH_ko00330	Control vs T2D
448	0.0626192	0.1014328	Arginine_and_proline_metabolism_PATH_ko00330	LADA vs Control
56	0.1412318	0.4612224	Arginine_and_proline_metabolism_PATH_ko00330	Control vs T1D
840	0.4453473	0.5858260	Arginine_and_proline_metabolism_PATH_ko00330	LADA vs T1D
644	0.4788779	0.6743371	Arginine_and_proline_metabolism_PATH_ko00330	T1D vs T2D
1036	0.8564261	0.9483589	Arginine_and_proline_metabolism_PATH_ko00330	LADA vs T2D

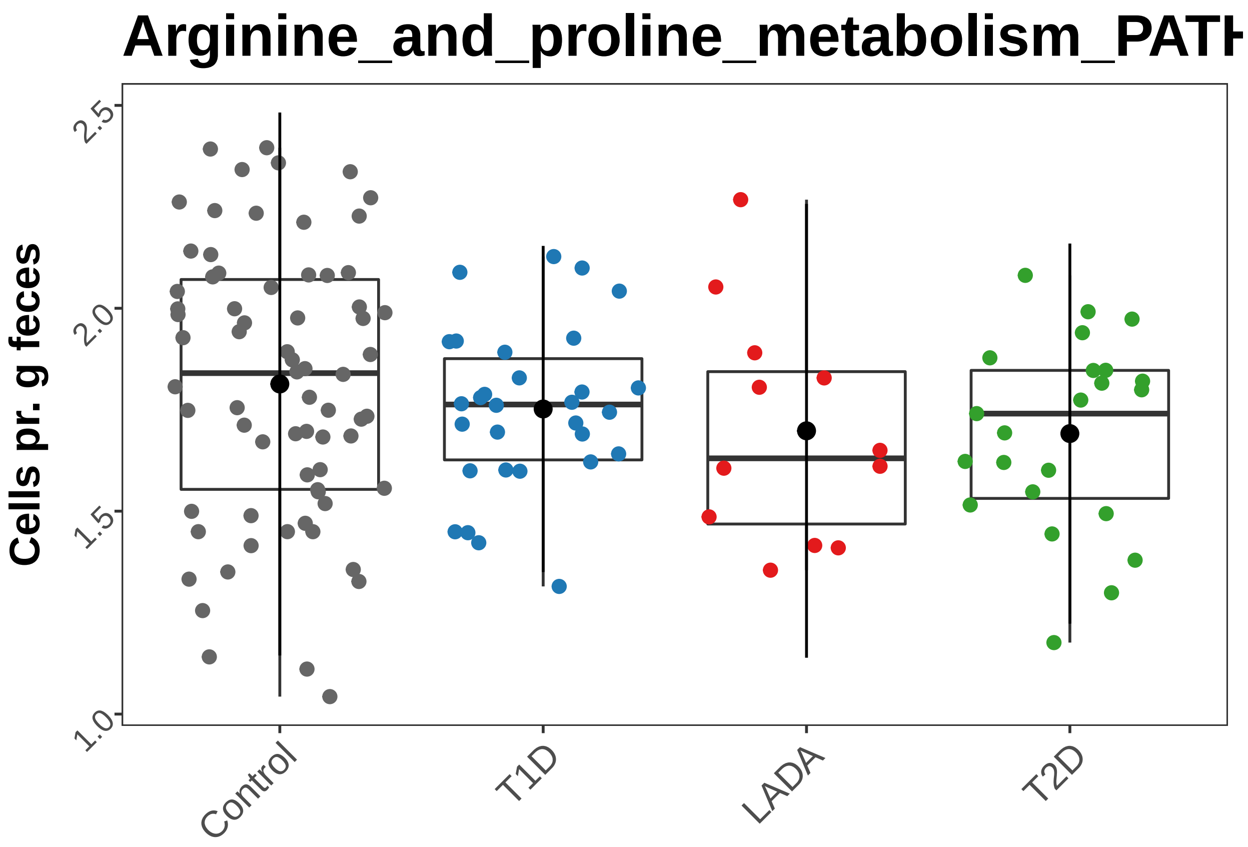

	pvalue	padj	Genus	compare
458	0.0341670	0.0690384	Tyrosine_metabolism_PATH_ko00350	LADA vs Control
262	0.0409613	0.0768024	Tyrosine_metabolism_PATH_ko00350	Control vs T2D
850	0.2004295	0.3597318	Tyrosine_metabolism_PATH_ko00350	LADA vs T1D
66	0.3036615	0.5208704	Tyrosine_metabolism_PATH_ko00350	Control vs T1D
654	0.3357612	0.6495082	Tyrosine_metabolism_PATH_ko00350	T1D vs T2D
1046	0.6320113	0.8841442	Tyrosine_metabolism_PATH_ko00350	LADA vs T2D

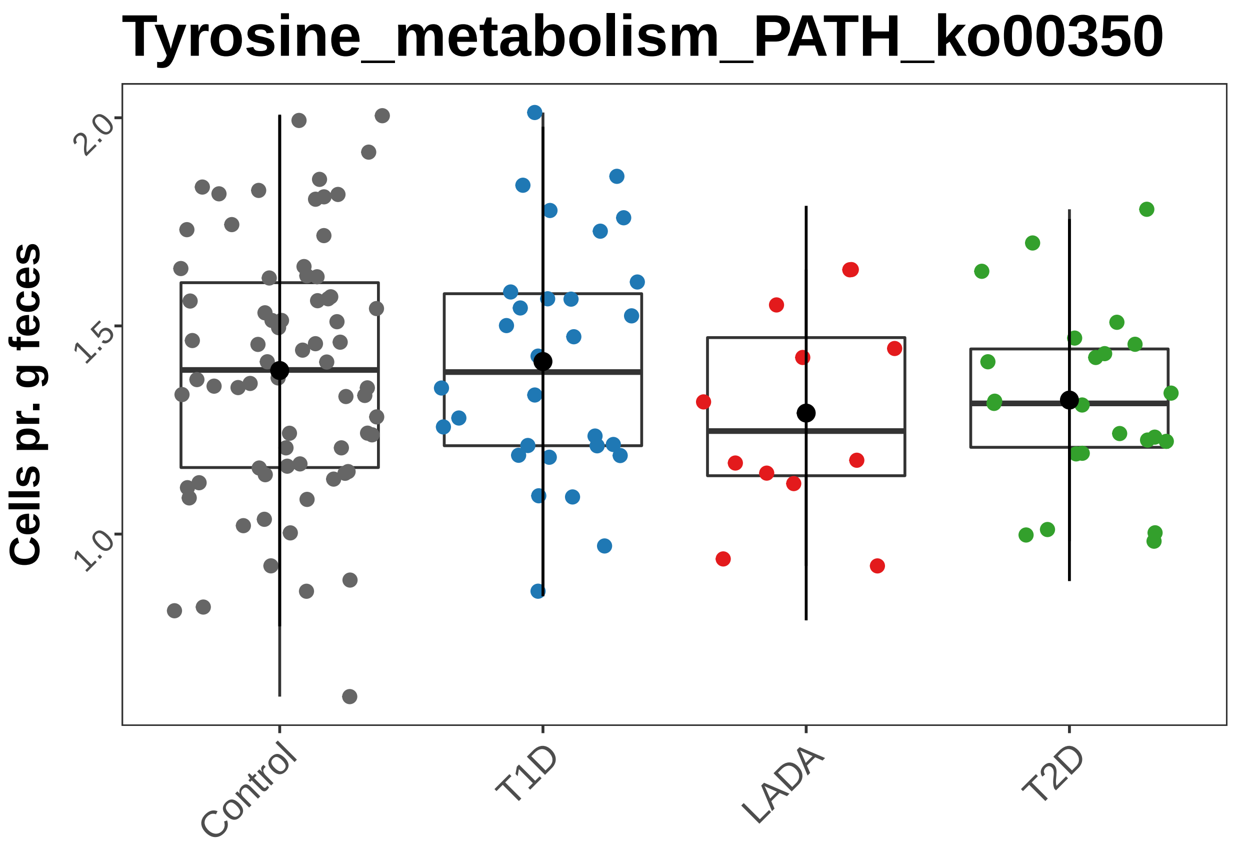

	pvalue	padj	Genus	compare
219	0.0352923	0.0725327	AMPK_signaling_pathway_PATH_ko04152	Control vs T2D
415	0.0428161	0.0779543	AMPK_signaling_pathway_PATH_ko04152	LADA vs Control
807	0.0242413	0.2312928	AMPK_signaling_pathway_PATH_ko04152	LADA vs T1D
611	0.0203944	0.6495082	AMPK_signaling_pathway_PATH_ko04152	T1D vs T2D
23	0.5307493	0.6889196	AMPK_signaling_pathway_PATH_ko04152	Control vs T1D
1003	0.7215122	0.9035121	AMPK_signaling_pathway_PATH_ko04152	LADA vs T2D

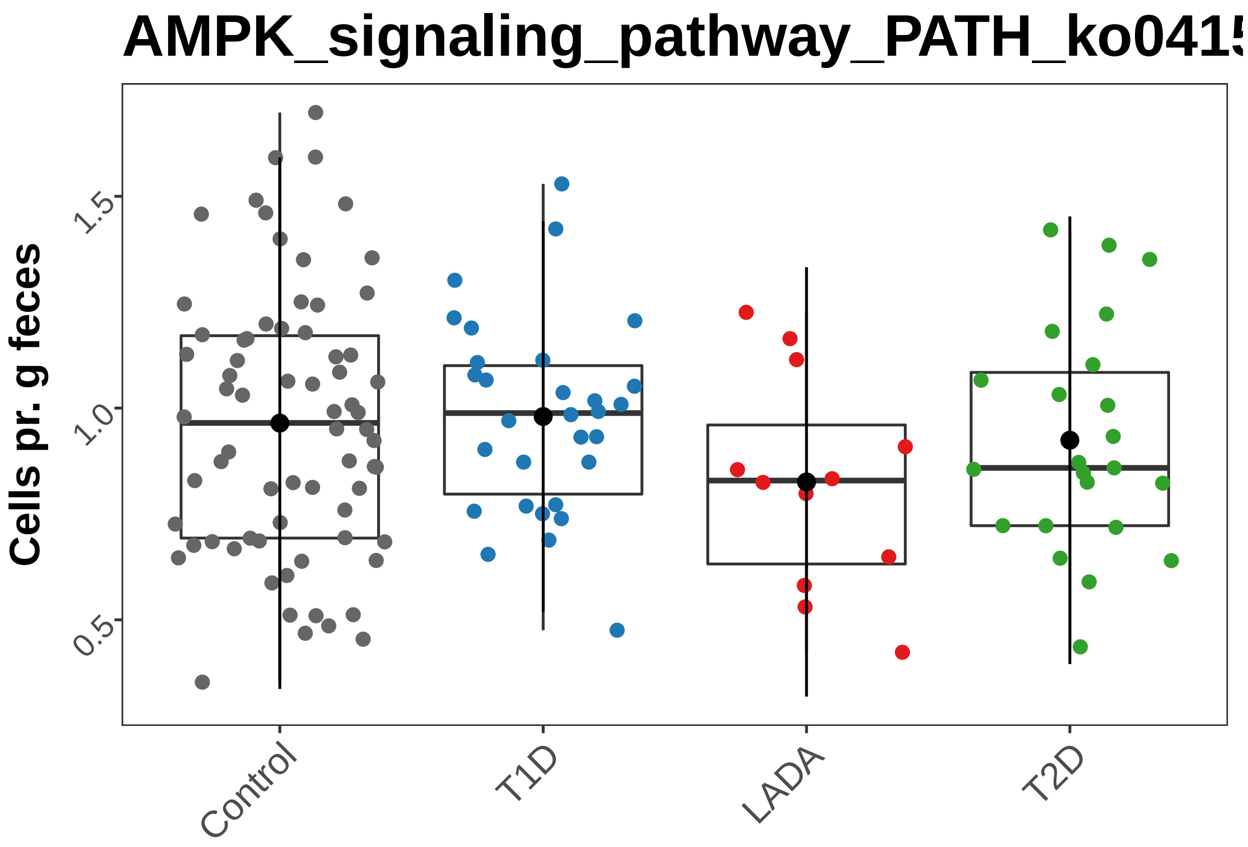

	pvalue	padj	Genus	compare
535	0.0370487	0.0733489	Riboflavin_metabolism_PATH_ko00740	LADA vs Control
339	0.0766963	0.1182699	Riboflavin_metabolism_PATH_ko00740	Control vs T2D
927	0.0996344	0.2458003	Riboflavin_metabolism_PATH_ko00740	LADA vs T1D
731	0.2248701	0.6495082	Riboflavin_metabolism_PATH_ko00740	T1D vs T2D
143	0.6831189	0.7784378	Riboflavin_metabolism_PATH_ko00740	Control vs T1D
1123	0.5255508	0.8415375	Riboflavin_metabolism_PATH_ko00740	LADA vs T2D

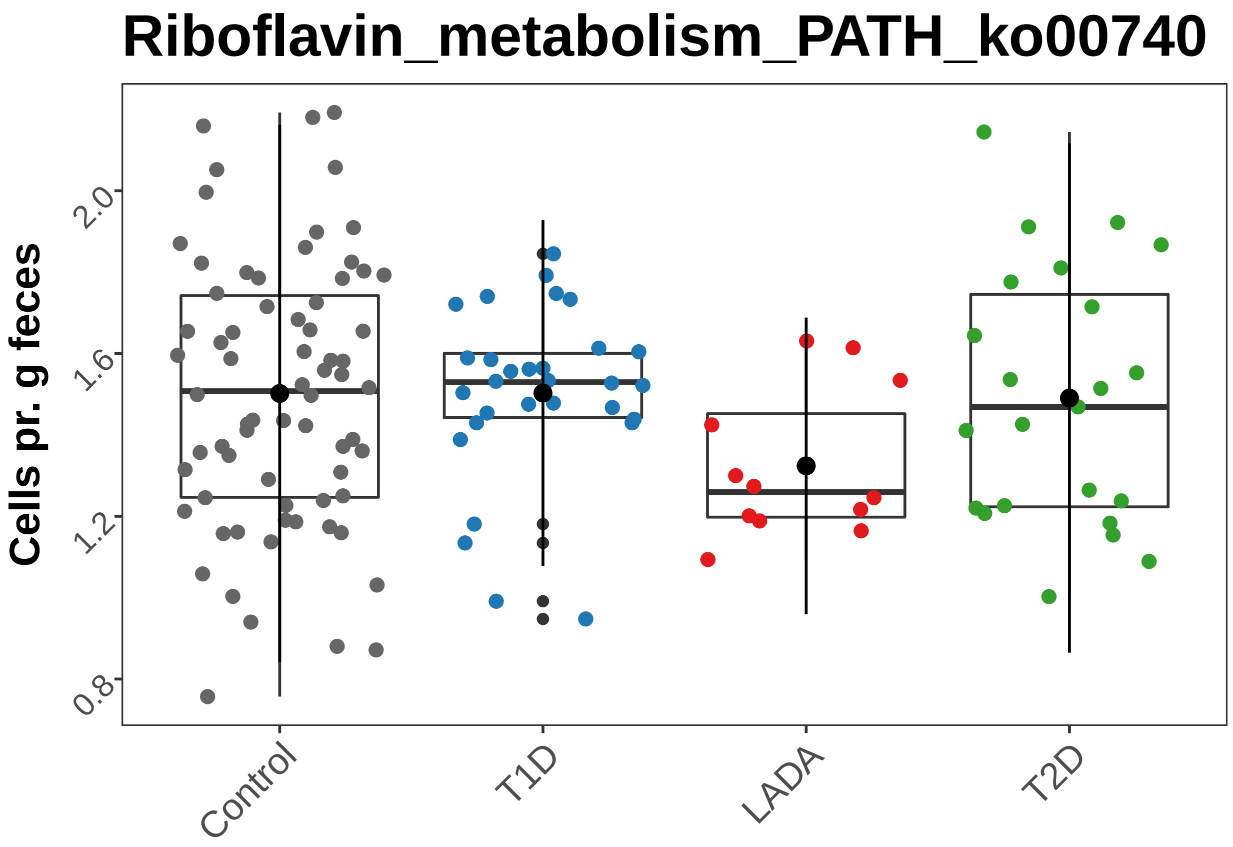

	pvalue	padj	Genus	compare
547	0.0384996	0.0735070	Taurine_and_hypotaurine_metabolism_PATH_ko00430	LADA vs Control
351	0.0811697	0.1238826	Taurine_and_hypotaurine_metabolism_PATH_ko00430	Control vs T2D
939	0.2352270	0.3940555	Taurine_and_hypotaurine_metabolism_PATH_ko00430	LADA vs T1D
155	0.2691132	0.4873448	Taurine_and_hypotaurine_metabolism_PATH_ko00430	Control vs T1D
743	0.5207430	0.6862683	Taurine_and_hypotaurine_metabolism_PATH_ko00430	T1D vs T2D
1135	0.5230035	0.8415375	Taurine_and_hypotaurine_metabolism_PATH_ko00430	LADA vs T2D

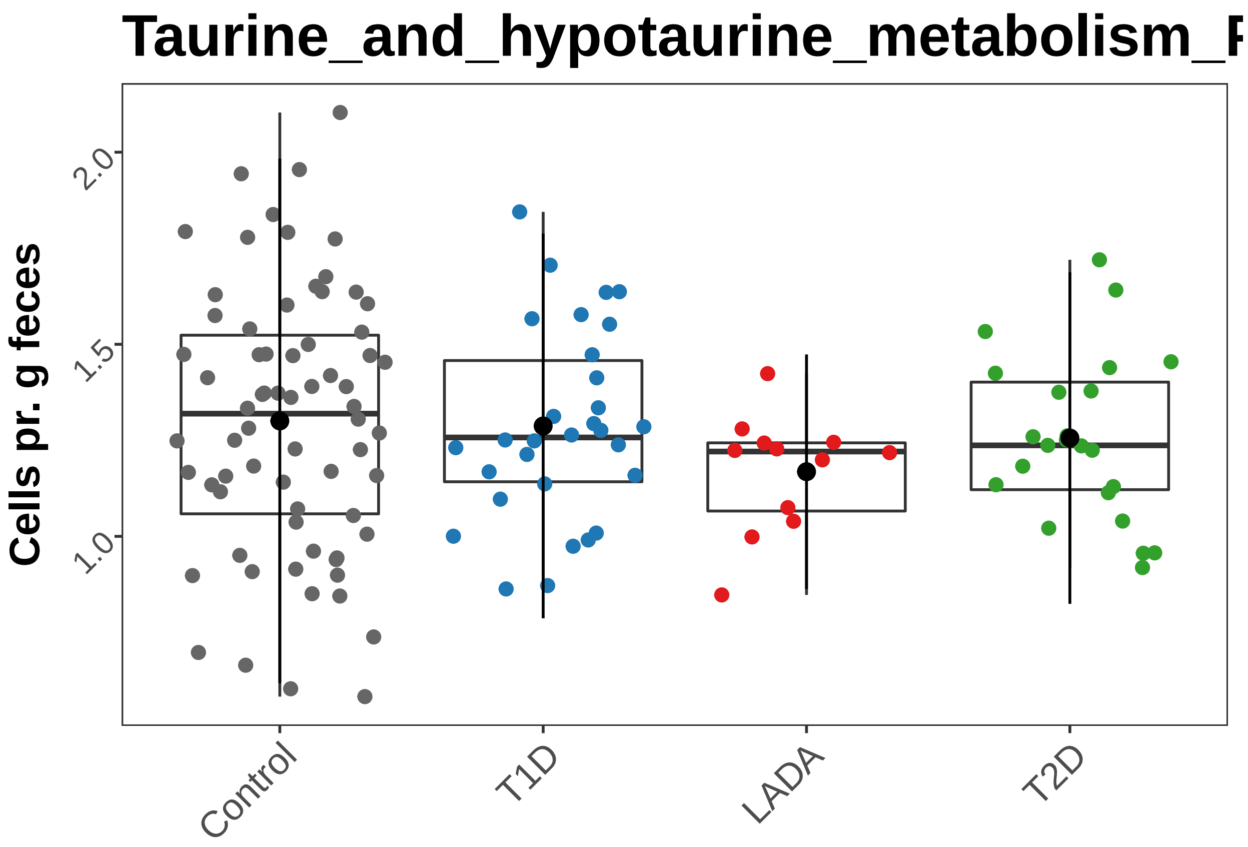

	pvalue	padj	Genus	compare
577	0.0394743	0.0743938	Toluene_degradation_PATH_ko00623	LADA vs Control
969	0.0430751	0.2312928	Toluene_degradation_PATH_ko00623	LADA vs T1D
773	0.6031845	0.7461523	Toluene_degradation_PATH_ko00623	T1D vs T2D
1165	0.1246054	0.8415375	Toluene_degradation_PATH_ko00623	LADA vs T2D
185	0.8271268	0.8835907	Toluene_degradation_PATH_ko00623	Control vs T1D
381	0.6883169	NA	Toluene_degradation_PATH_ko00623	Control vs T2D

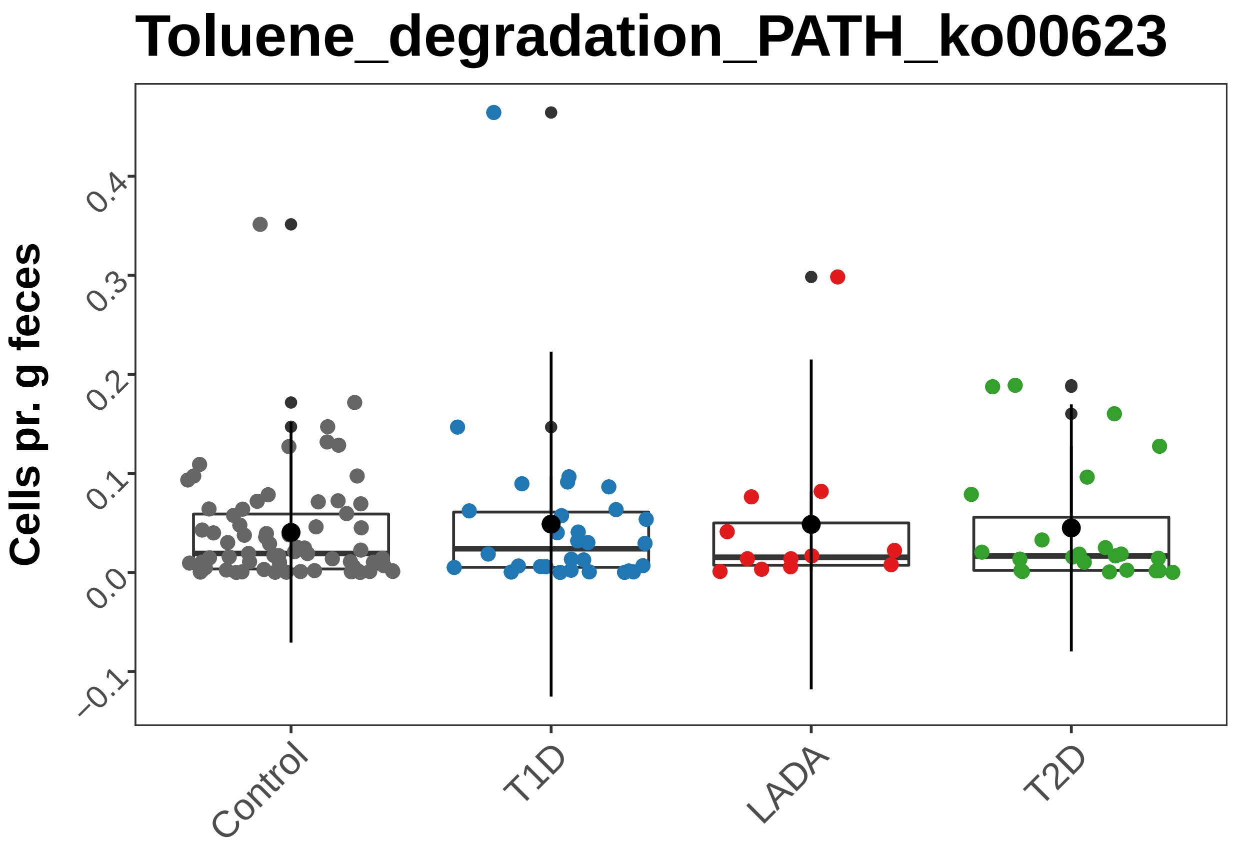

	pvalue	padj	Genus	compare
455	0.0407834	0.0754107	Phenylalanine_metabolism_PATH_ko00360	LADA vs Control
259	0.0653762	0.1037218	Phenylalanine_metabolism_PATH_ko00360	Control vs T2D
847	0.1484133	0.3094575	Phenylalanine_metabolism_PATH_ko00360	LADA vs T1D
651	0.2833699	0.6495082	Phenylalanine_metabolism_PATH_ko00360	T1D vs T2D
63	0.5047713	0.6651524	Phenylalanine_metabolism_PATH_ko00360	Control vs T1D
1043	0.5814776	0.8682081	Phenylalanine_metabolism_PATH_ko00360	LADA vs T2D

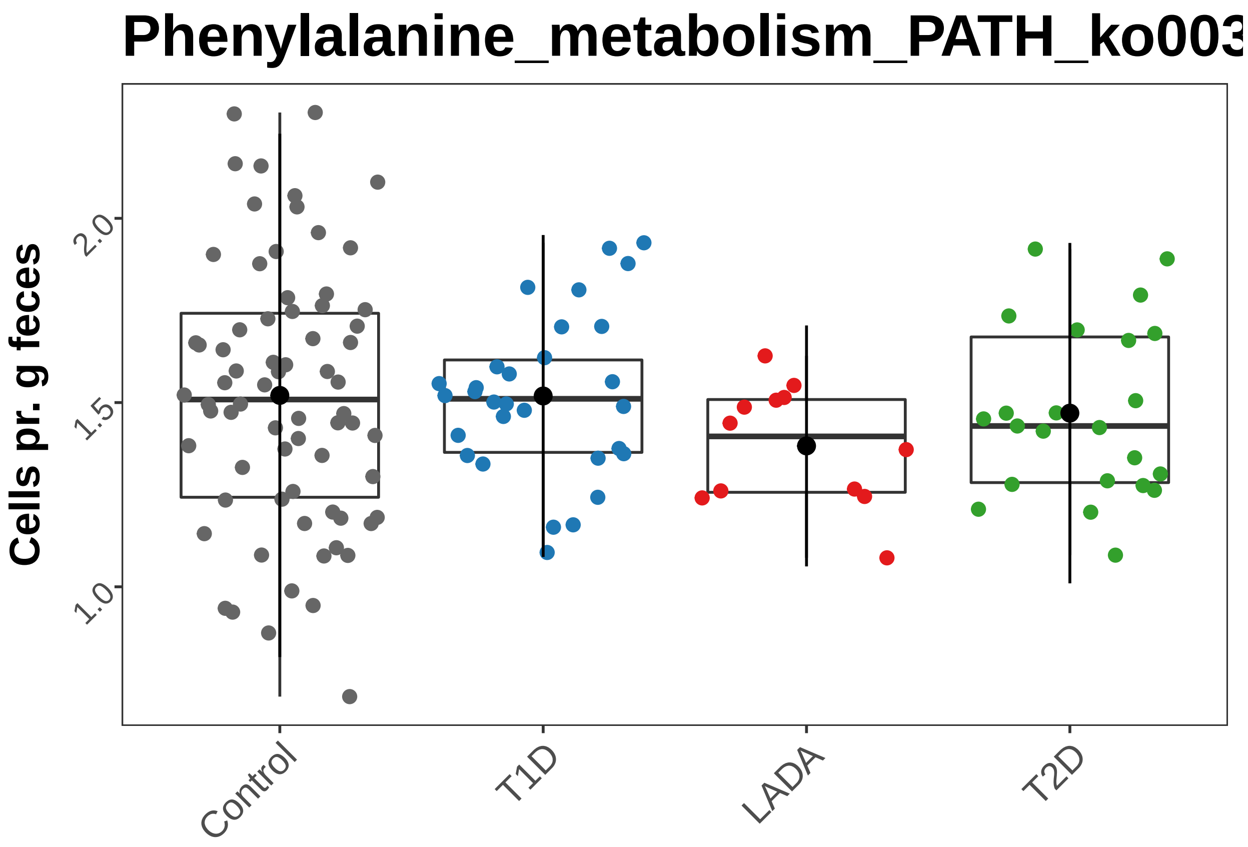

	pvalue	padj	Genus	compare
318	0.0402245	0.0767378	Biosynthesis_of_unsaturated_fatty_acids_PATH_ko01040	Control vs T2D
514	0.0429544	0.0779543	Biosynthesis_of_unsaturated_fatty_acids_PATH_ko01040	LADA vs Control
906	0.2037257	0.3597318	Biosynthesis_of_unsaturated_fatty_acids_PATH_ko01040	LADA vs T1D
122	0.3635102	0.5438778	Biosynthesis_of_unsaturated_fatty_acids_PATH_ko01040	Control vs T1D
710	0.2875507	0.6495082	Biosynthesis_of_unsaturated_fatty_acids_PATH_ko01040	T1D vs T2D
1102	0.6955014	0.9028767	Biosynthesis_of_unsaturated_fatty_acids_PATH_ko01040	LADA vs T2D

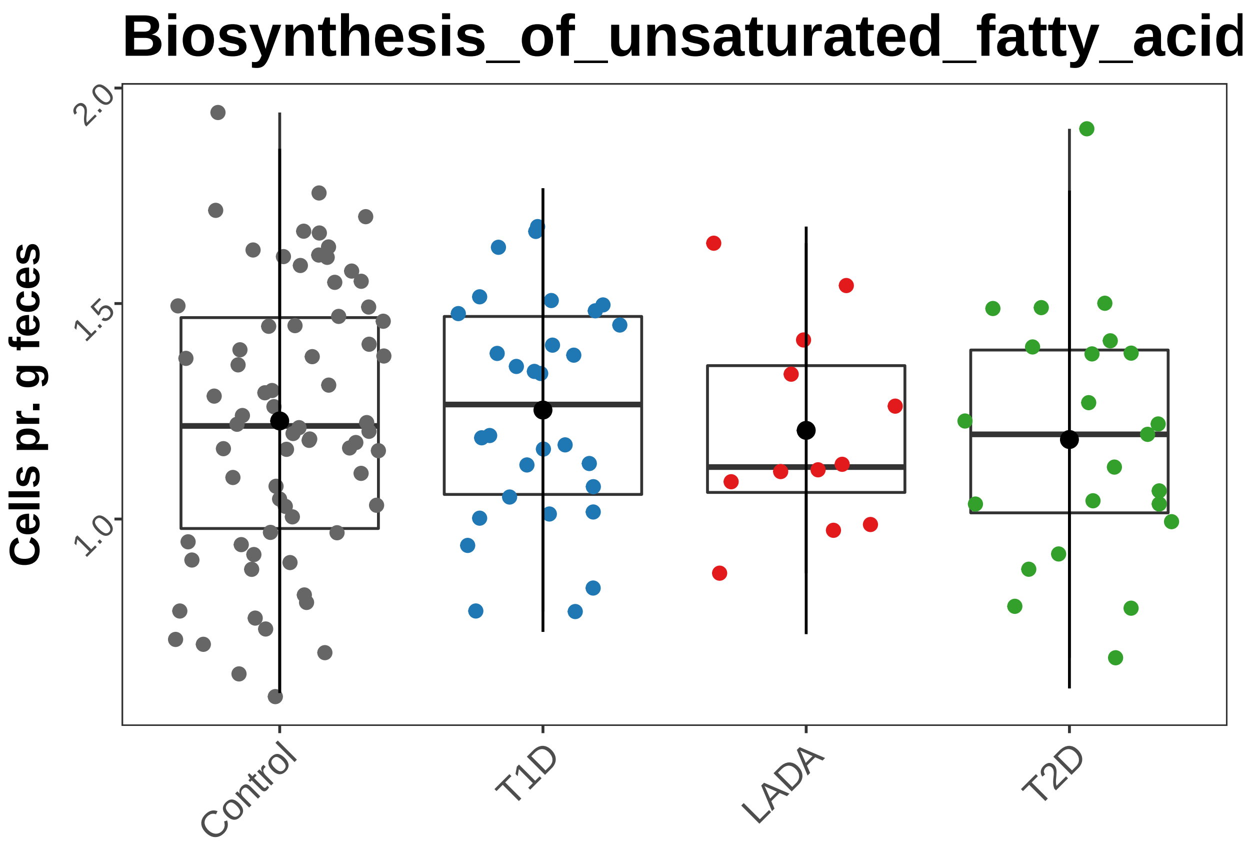

	pvalue	padj	Genus	compare
382	0.0417002	0.0773094	Xylene_degradation_PATH_ko00622	Control vs T2D
578	0.1229678	0.1772183	Xylene_degradation_PATH_ko00622	LADA vs Control
186	0.1887068	0.4762288	Xylene_degradation_PATH_ko00622	Control vs T1D
774	0.4647125	0.6743371	Xylene_degradation_PATH_ko00622	T1D vs T2D
970	0.5678924	0.6828644	Xylene_degradation_PATH_ko00622	LADA vs T1D
1166	0.9831066	0.9986549	Xylene_degradation_PATH_ko00622	LADA vs T2D

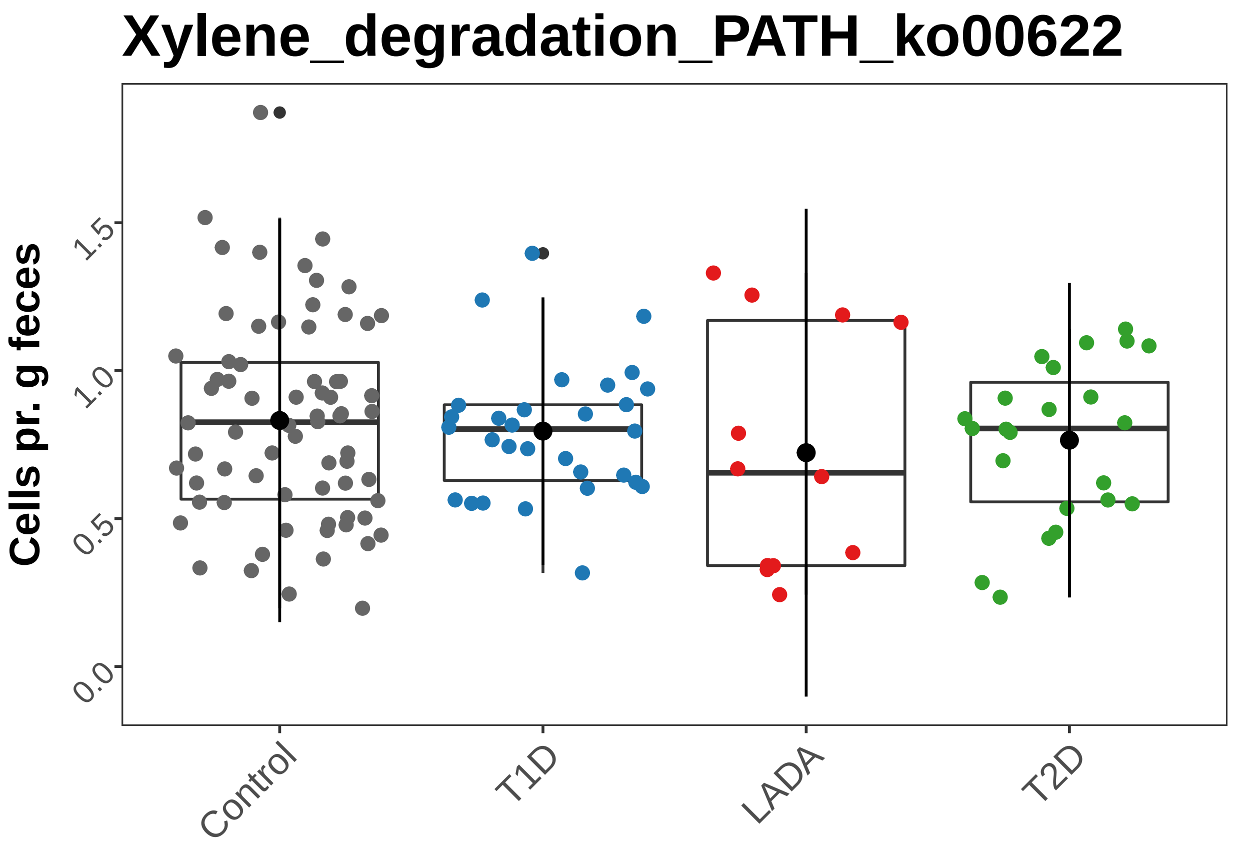

	pvalue	padj	Genus	compare
517	0.0459308	0.0816046	Fatty_acid_degradation_PATH_ko00071	LADA vs Control
321	0.1042285	0.1535510	Fatty_acid_degradation_PATH_ko00071	Control vs T2D
909	0.2328950	0.3940555	Fatty_acid_degradation_PATH_ko00071	LADA vs T1D
125	0.3218636	0.5217679	Fatty_acid_degradation_PATH_ko00071	Control vs T1D
713	0.5293999	0.6917492	Fatty_acid_degradation_PATH_ko00071	T1D vs T2D
1105	0.5126303	0.8415375	Fatty_acid_degradation_PATH_ko00071	LADA vs T2D

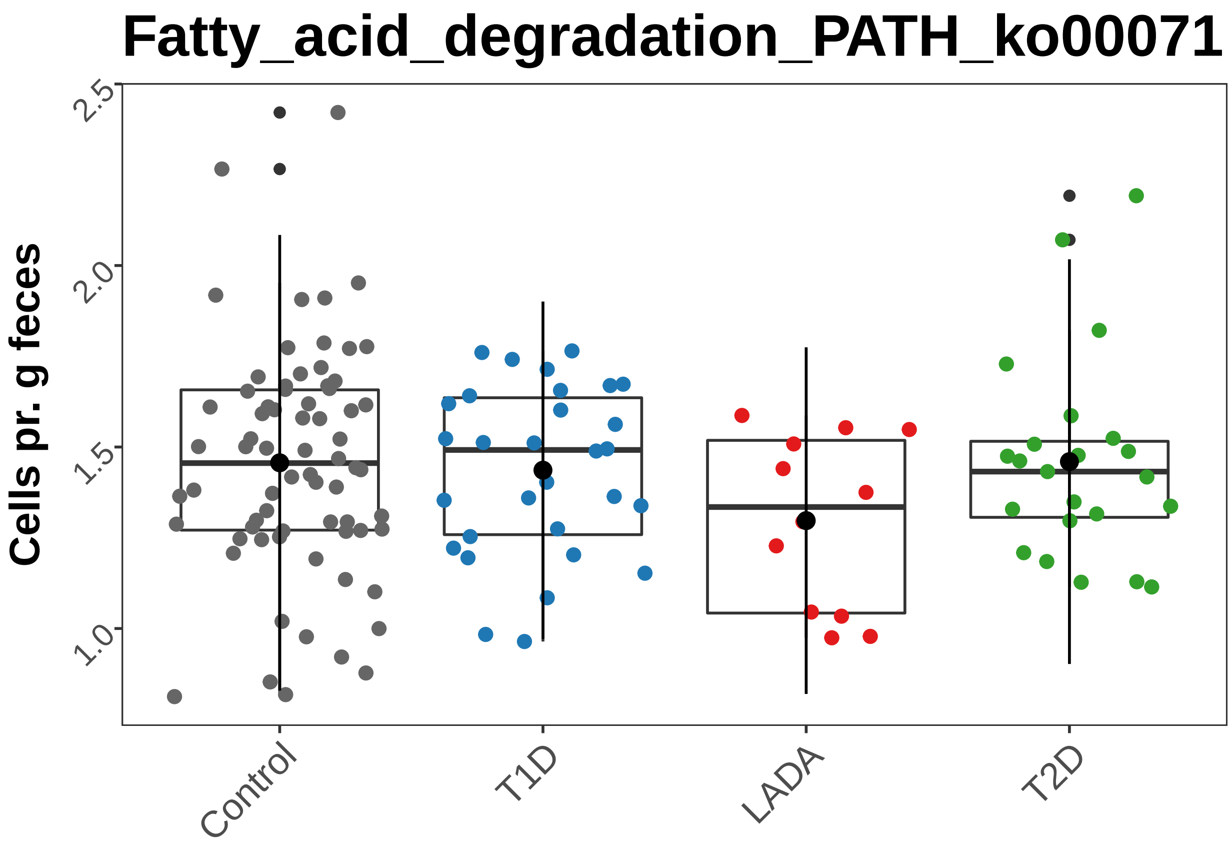

	pvalue	padj	Genus	compare
494	0.0462149	0.0816046	Methane_metabolism_PATH_ko00680	LADA vs Control
886	0.0077757	0.1693375	Methane_metabolism_PATH_ko00680	LADA vs T1D
102	0.1895196	0.4762288	Methane_metabolism_PATH_ko00680	Control vs T1D
298	0.4183861	0.4966454	Methane_metabolism_PATH_ko00680	Control vs T2D
690	0.0827800	0.6495082	Methane_metabolism_PATH_ko00680	T1D vs T2D
1082	0.2290402	0.8415375	Methane_metabolism_PATH_ko00680	LADA vs T2D

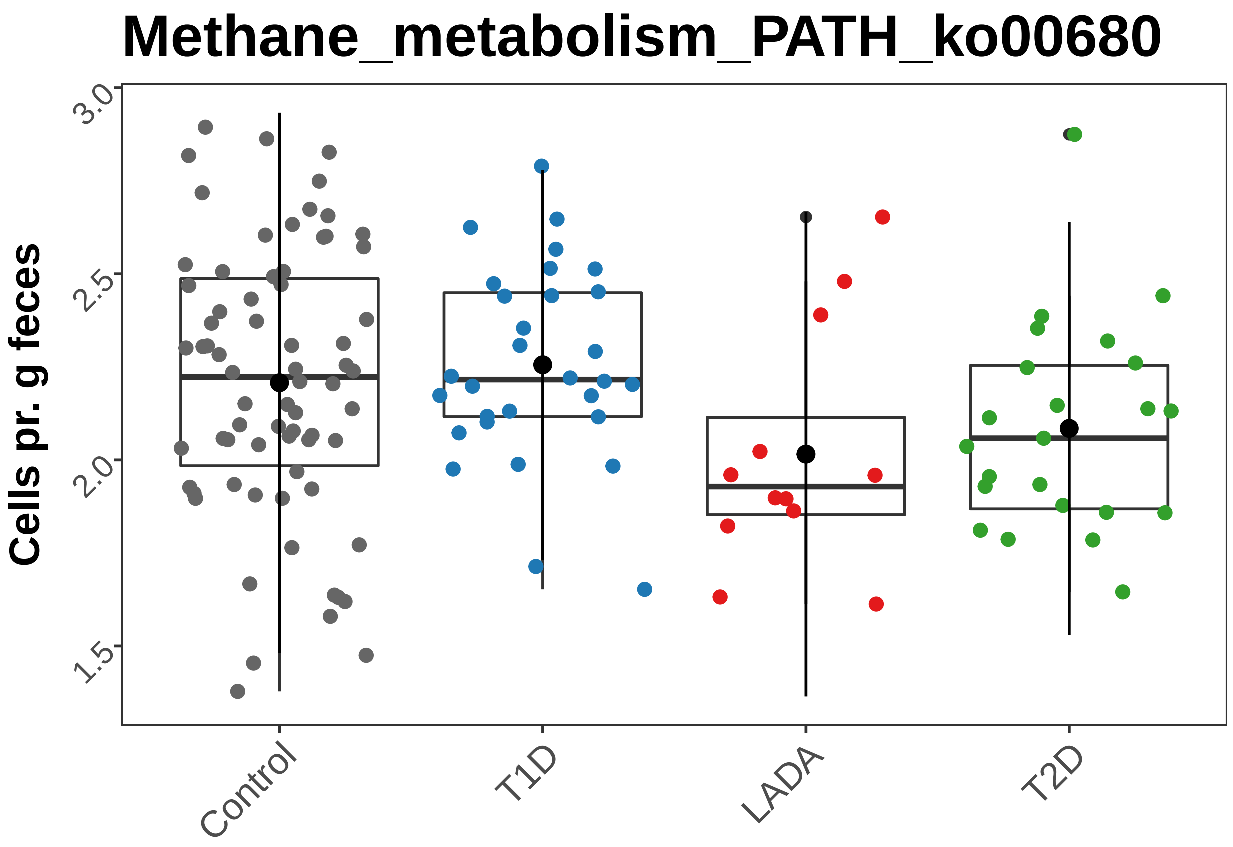

	pvalue	padj	Genus	compare
330	0.0465827	0.0828378	Synthesis_and_degradation_of_ketone_bodies_PATH_ko00072	Control vs T2D
526	0.0613909	0.1002718	Synthesis_and_degradation_of_ketone_bodies_PATH_ko00072	LADA vs Control
918	0.1155838	0.2692112	Synthesis_and_degradation_of_ketone_bodies_PATH_ko00072	LADA vs T1D
722	0.1196542	0.6495082	Synthesis_and_degradation_of_ketone_bodies_PATH_ko00072	T1D vs T2D
134	0.8294933	0.8835907	Synthesis_and_degradation_of_ketone_bodies_PATH_ko00072	Control vs T1D
1114	0.7655910	0.9035121	Synthesis_and_degradation_of_ketone_bodies_PATH_ko00072	LADA vs T2D

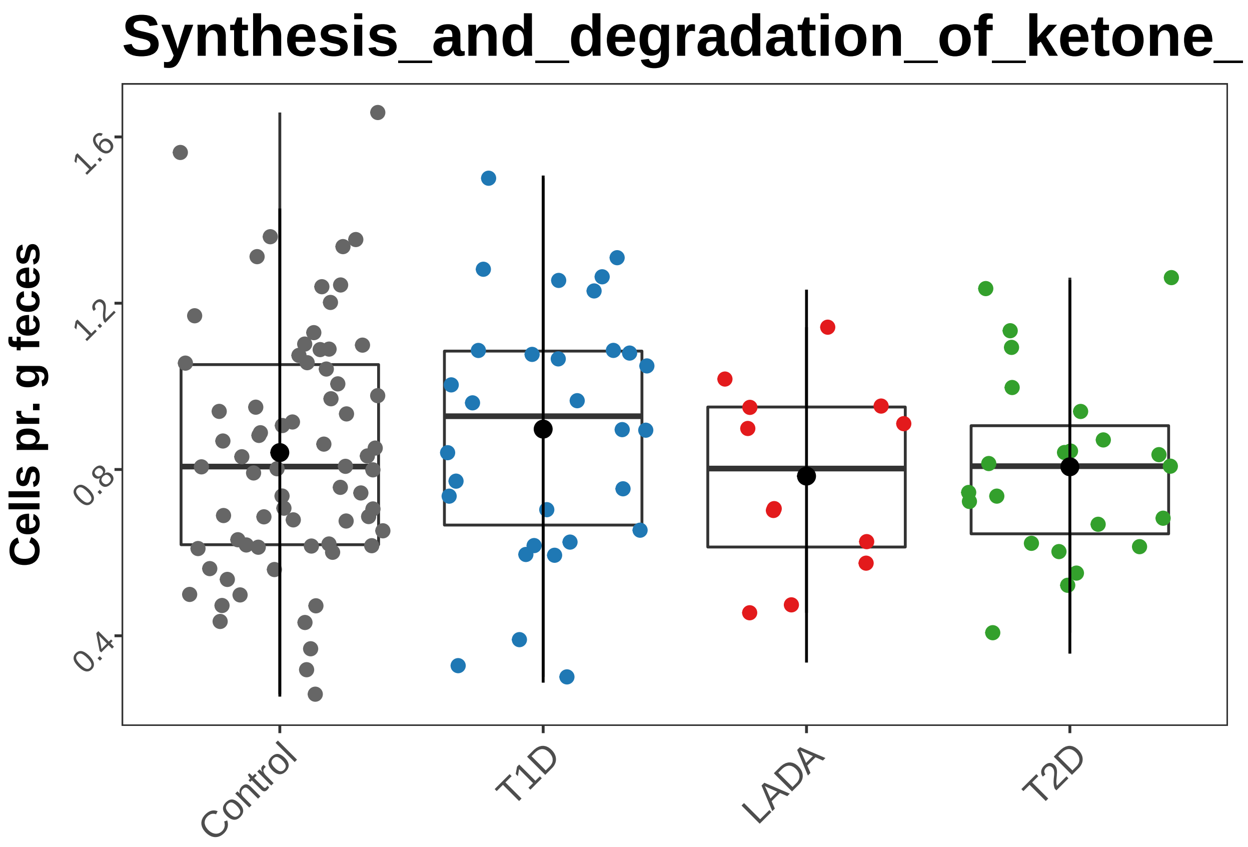

	pvalue	padj	Genus	compare
258	0.0468526	0.0828378	Lysine_degradation_PATH_ko00310	Control vs T2D
454	0.4294406	0.5010140	Lysine_degradation_PATH_ko00310	LADA vs Control
650	0.2093092	0.6495082	Lysine_degradation_PATH_ko00310	T1D vs T2D
62	0.5520914	0.6970694	Lysine_degradation_PATH_ko00310	Control vs T1D
846	0.7316920	0.8289689	Lysine_degradation_PATH_ko00310	LADA vs T1D
1042	0.5168330	0.8415375	Lysine_degradation_PATH_ko00310	LADA vs T2D

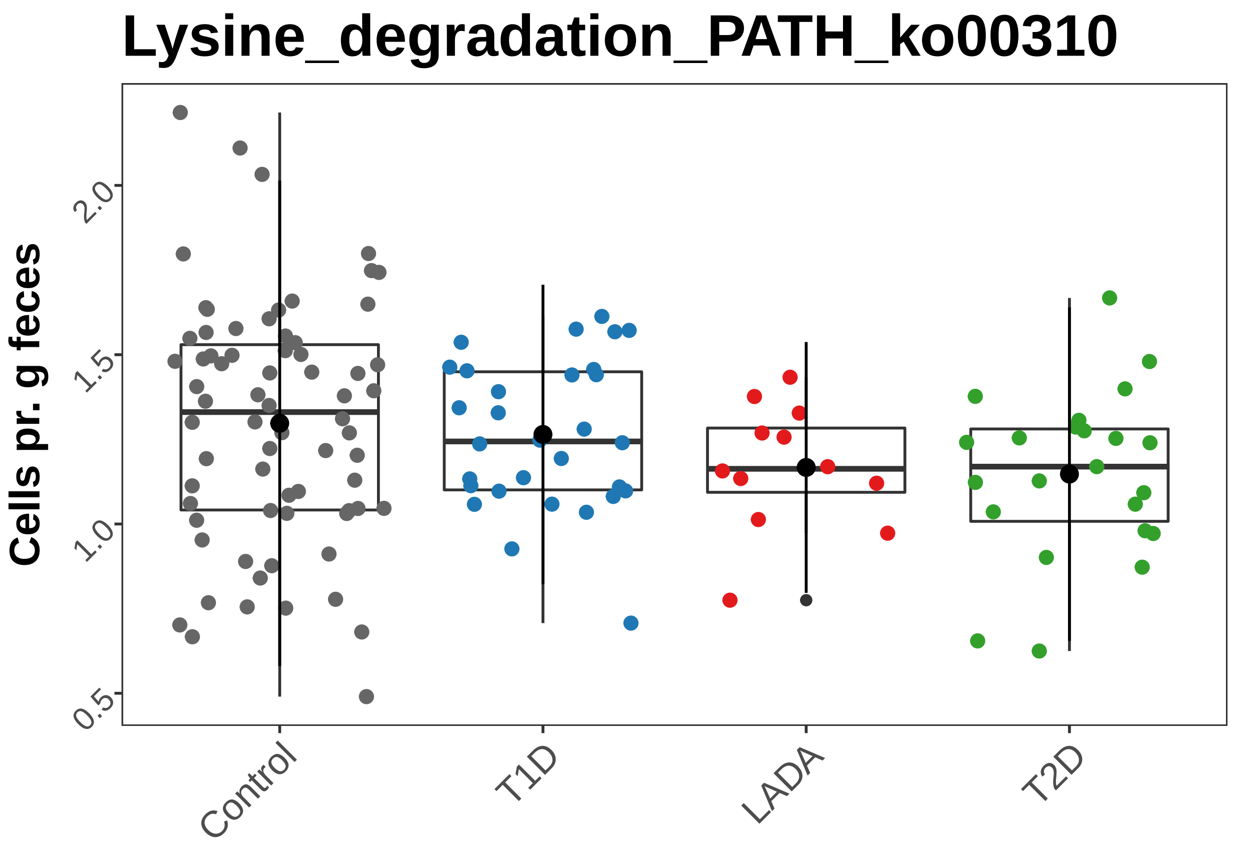

	pvalue	padj	Genus	compare
380	0.0476945	0.0828378	Styrene_degradation_PATH_ko00643	Control vs T2D
576	0.0493240	0.0840653	Styrene_degradation_PATH_ko00643	LADA vs Control
968	0.1167498	0.2692112	Styrene_degradation_PATH_ko00643	LADA vs T1D
772	0.1515305	0.6495082	Styrene_degradation_PATH_ko00643	T1D vs T2D
184	0.7192996	0.8056155	Styrene_degradation_PATH_ko00643	Control vs T1D
1164	0.6977563	0.9028767	Styrene_degradation_PATH_ko00643	LADA vs T2D

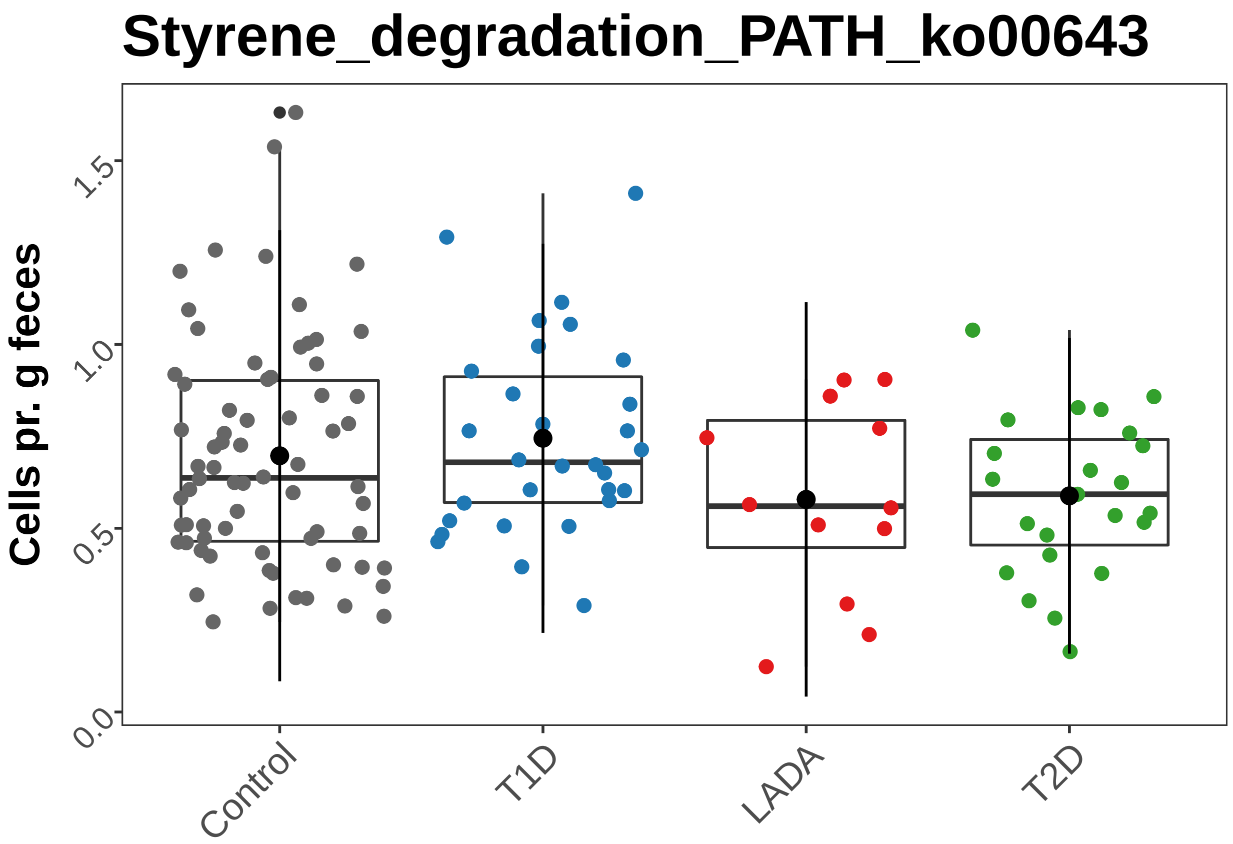

	pvalue	padj	Genus	compare
371	0.0482554	0.0829389	Chlorocyclohexane_and_chlorobenzene_degradation_PATH_ko00361	Control vs T2D
567	0.5216851	0.5809675	Chlorocyclohexane_and_chlorobenzene_degradation_PATH_ko00361	LADA vs Control
959	0.4736233	0.6100058	Chlorocyclohexane_and_chlorobenzene_degradation_PATH_ko00361	LADA vs T1D
763	0.0609790	0.6495082	Chlorocyclohexane_and_chlorobenzene_degradation_PATH_ko00361	T1D vs T2D
1155	0.4407326	0.8415375	Chlorocyclohexane_and_chlorobenzene_degradation_PATH_ko00361	LADA vs T2D
175	0.8381750	0.8851806	Chlorocyclohexane_and_chlorobenzene_degradation_PATH_ko00361	Control vs T1D

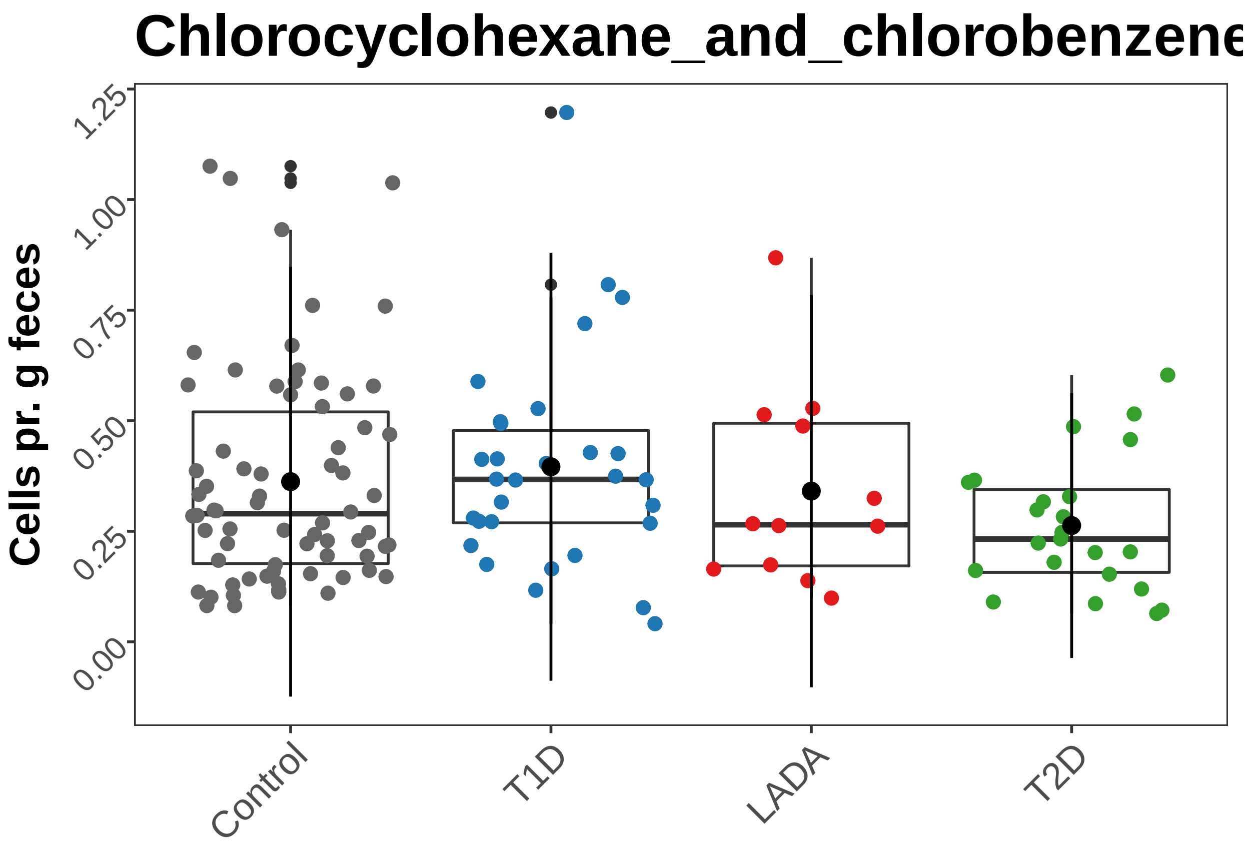

	pvalue	padj	Genus	compare
410	0.0508860	0.0859799	Peroxisome__PATH__ko04146	LADA vs Control
214	0.2592456	0.3292047	Peroxisome__PATH__ko04146	Control vs T2D
802	0.2503840	0.3957683	Peroxisome__PATH__ko04146	LADA vs T1D
18	0.3190838	0.5217679	Peroxisome__PATH__ko04146	Control vs T1D
998	0.3412941	0.8415375	Peroxisome__PATH__ko04146	LADA vs T2D
606	0.8462895	0.8870200	Peroxisome__PATH__ko04146	T1D vs T2D

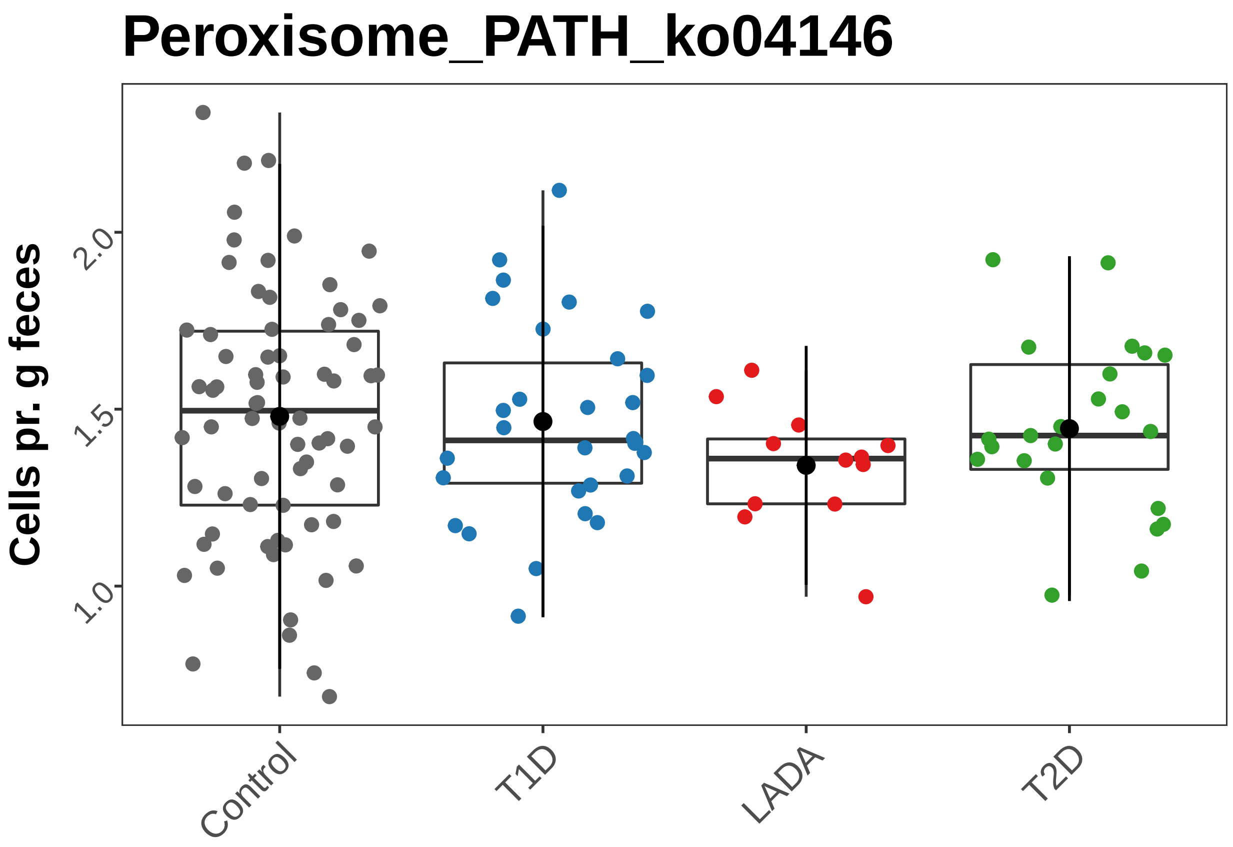

	pvalue	padj	Genus	compare
506	0.0551464	0.0910330	Mannose_type_O_glycan_biosynthesis_PATH_ko00515	LADA vs Control
898	0.2838597	0.4244349	Mannose_type_O_glycan_biosynthesis_PATH_ko00515	LADA vs T1D
114	0.2858122	0.5040976	Mannose_type_O_glycan_biosynthesis_PATH_ko00515	Control vs T1D
702	0.3445426	0.6495082	Mannose_type_O_glycan_biosynthesis_PATH_ko00515	T1D vs T2D
1094	0.0777549	0.8415375	Mannose_type_O_glycan_biosynthesis_PATH_ko00515	LADA vs T2D
310	0.9037870	NA	Mannose_type_O_glycan_biosynthesis_PATH_ko00515	Control vs T2D

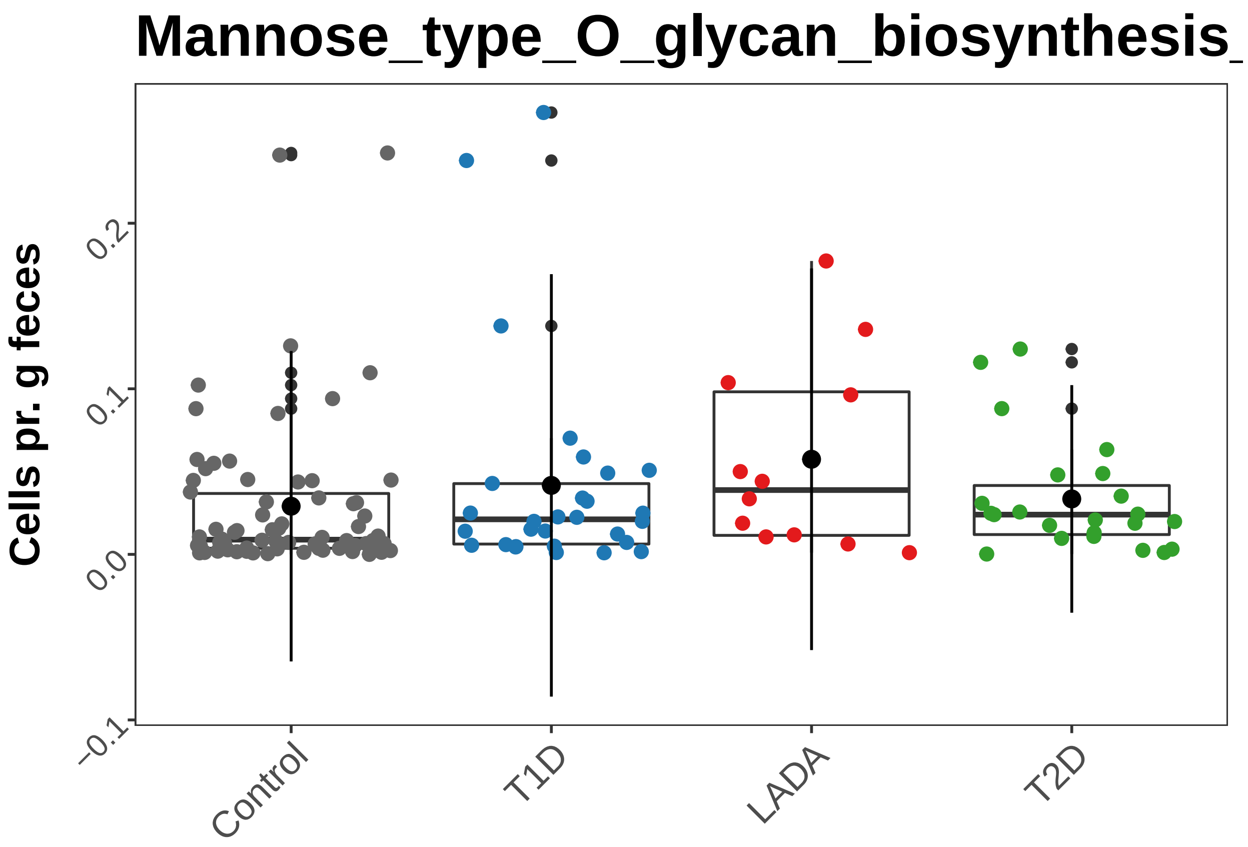

	pvalue	padj	Genus	compare
481	0.0552700	0.0910330	Citrate_cycle___TCA_cycle___PATH_ko00020	LADA vs Control
285	0.1448769	0.1975594	Citrate_cycle___TCA_cycle___PATH_ko00020	Control vs T2D
873	0.0450772	0.2312928	Citrate_cycle___TCA_cycle___PATH_ko00020	LADA vs T1D
677	0.1156899	0.6495082	Citrate_cycle___TCA_cycle___PATH_ko00020	T1D vs T2D
89	0.6948470	0.7863905	Citrate_cycle___TCA_cycle___PATH_ko00020	Control vs T1D
1069	0.4853153	0.8415375	Citrate_cycle___TCA_cycle___PATH_ko00020	LADA vs T2D

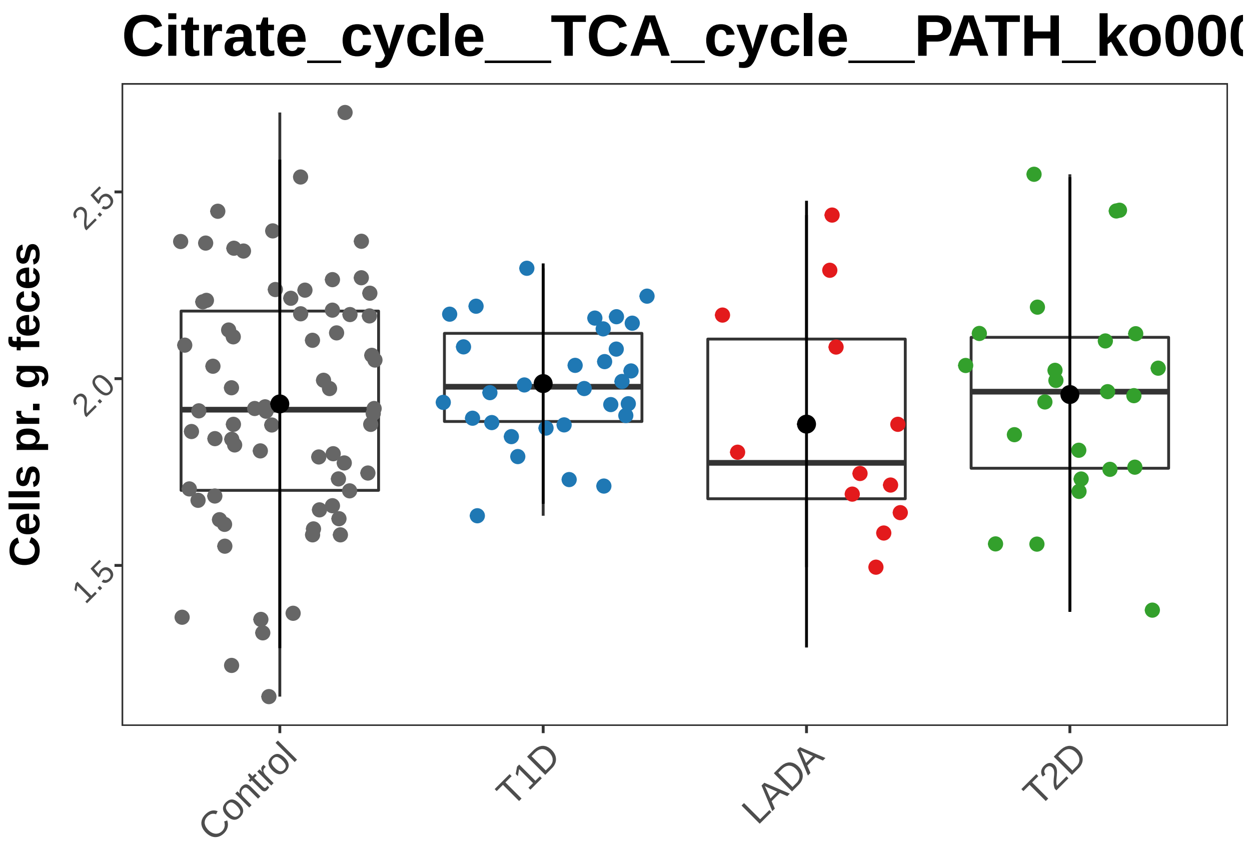

	pvalue	padj	Genus	compare
261	0.0535643	0.0911146	Tryptophan__metabolism__PATH__ko00380	Control vs T2D
653	0.1374214	0.6495082	Tryptophan__metabolism__PATH__ko00380	T1D vs T2D
457	0.7965545	0.8304504	Tryptophan__metabolism__PATH__ko00380	LADA vs Control
1045	0.2816343	0.8415375	Tryptophan__metabolism__PATH__ko00380	LADA vs T2D
65	0.8107696	0.8779605	Tryptophan__metabolism__PATH__ko00380	Control vs T1D
849	0.9339681	0.9691189	Tryptophan__metabolism__PATH__ko00380	LADA vs T1D

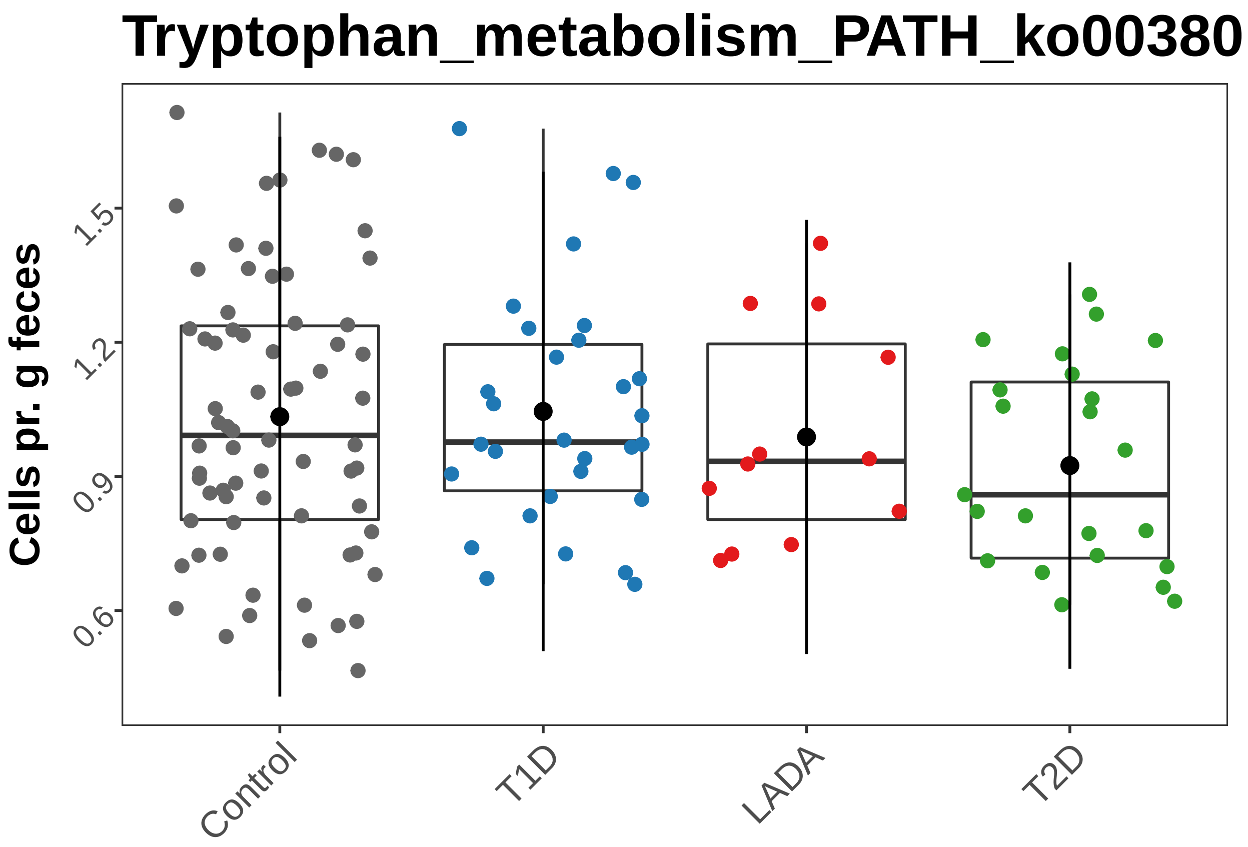

	pvalue	padj	Genus	compare
372	0.0615338	0.0996512	Dioxin_degradation_PATH_ko00621	Control vs T2D
568	0.1144990	0.1689666	Dioxin_degradation_PATH_ko00621	LADA vs Control
176	0.1593391	0.4612224	Dioxin_degradation_PATH_ko00621	Control vs T1D
960	0.5860194	0.7003646	Dioxin_degradation_PATH_ko00621	LADA vs T1D
764	0.6077607	0.7461523	Dioxin_degradation_PATH_ko00621	T1D vs T2D
1156	0.9021828	0.9769493	Dioxin_degradation_PATH_ko00621	LADA vs T2D

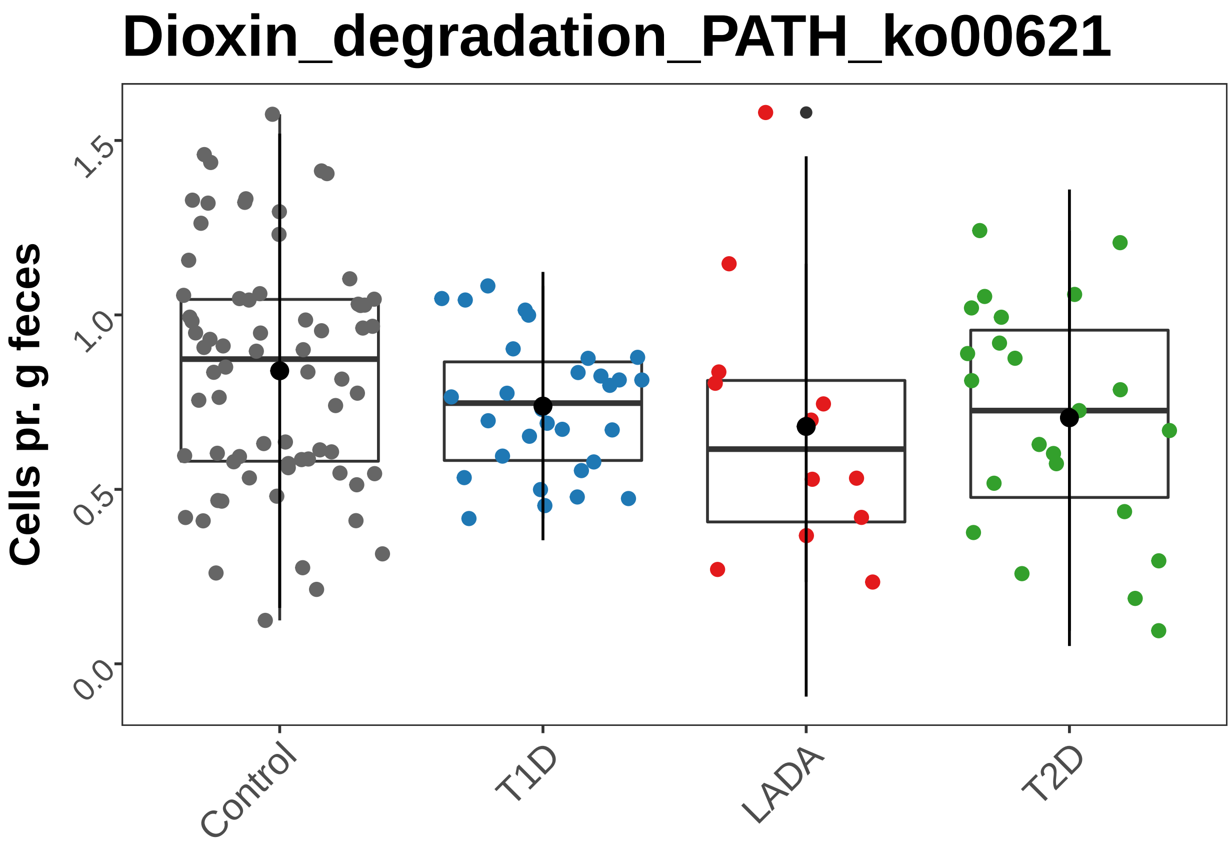


```
lay <- rbind(c(1,2,3))
pdf(paste("MicroLADA_GeneraDARemMet_Func.pdf", sep=""), width=15, height=5)
grid.arrange(Fig2List$alpha_Linolenic_acid_metabolism_PATH_ko00592,
             Fig2List$Geraniol_degradation_PATH_ko00281,
             Fig2List$Bacterial_chemotaxis_PATH_ko02030,layout_matrix = lay)
dev.off()
```

pdf 2

```
#ggplot(Plotting2, aes(x=BMI, y=Actinomyces)) +
#   geom_point()
```

Create figure 2

Investigating grouping diagnosis. Which group does LADA resemble the most and which are different from each other.

```
#Have the plots stored in list
lay <- rbind(c(1,2,3),
             c(4,5,6))


pdf(paste("MicroLADA_Figure2_Func.pdf", sep=""), width=15, height=10)
grid.arrange(Fig2List$vulcLadaT1D,
```

```
             Fig2List$vulcLadaT2D,
             Fig2List$vulcLadaControl,
             Fig2List$Geraniol_degradation_PATH_ko00281,
             Fig2List$Carotenoid_biosynthesis_PATH_ko00906,
             Fig2List$Retinol_metabolism_PATH_ko00830, layout_matrix = lay)
dev.off()
```

```
## pdf
##   2
```

```
pdf(paste("MicroLADA_Figure2RemMet_Func.pdf", sep=""), width=15, height=10)
grid.arrange(Fig2ListRemMet$vulcLadaT1D,
             Fig2ListRemMet$vulcLadaT2D,
             Fig2ListRemMet$vulcLadaControl,
             Fig2ListRemMet$Geraniol_degradation_PATH_ko00281,
             Fig2ListRemMet$alpha_Linolenic_acid_metabolism_PATH_ko00592,
             Fig2ListRemMet$Bacterial_chemotaxis_PATH_ko02030, layout_matrix = lay)
dev.off()
```

```
## pdf
##   2
```

DESeq heatmap

Figure 3
Many models to be included so all validation plots are removed. Includes both LRT and Wald
Structured to be compatible with creating af heatmap. Includes a dataframe of log2-fold changes a dataframe of padj and afterwards creating a dataframe containing symbols for significance levels. Density plot based on the log2-fold changes.

```
rm(list=setdiff(ls(), c("Metadata", "MetadataMed", "Feature", "TaxonomyDA", "Taxonomy")))

#Reassign names
Metadata2<-Metadata
Taxonomy2<-TaxonomyDA

#The factor can not be ordered for DESeq to run
Metadata2$Diagnosis<-factor(Metadata2$Diagnosis, ordered=FALSE)
##Other
#Metadata2$BMIq<-factor(Metadata2$BMIq)


##LRT none
#Create design formula
design <- formula(paste("~ ", "Diagnosis"))
#Create DESeq2 object with matrix and metadata
dds <- DESeqDataSetFromMatrix(countData = Taxonomy2,
                              colData = Metadata2,
                              design = design)



##Assess and calculate size factors using the different methods
```

```r
SF<-data.frame(Ratio=sizeFactors(estimateSizeFactors(dds, type="ratio")),
               Poscounts=sizeFactors(estimateSizeFactors(dds, type="poscounts")),
               Iterate=sizeFactors(estimateSizeFactors(dds, type="poscounts")),
               #"iterate" takes a lot of time changed to "poscounts" but kept due to the
               #following code
               Relative=colSums(Taxonomy2)/mean(colSums(Taxonomy2))
               )
SF<-add_rownames(SF, var="MicrobiomeID")
#Adding total abundances
path <- paste("J:/",
              "CBMR/",
              "SUN-CBMR-Hansen-Group/",
              "Projects/",
              "LADA/",
              "LADA_Sandra_Evelina/",
              "LADA_JKV/",
              "LADA_R_AfterFlow_Analysis_FinalCounts/",
              "LADA_FinalCounts/",
              "2019-09-03_CountAnalysis.rds", sep="")
TotalCounts<-readRDS(path)
TotalCountsProcess <-
  data.frame(MicrobiomeID=TotalCounts$DF_Save_w$ID,
             CellNorm=TotalCounts$DF_Save_w$Cells_per_g_feces_FR_corrected_mean)
TotalCountsProcess$MicrobiomeID <- paste("L", TotalCountsProcess$MicrobiomeID,
                                         sep="")
SF2<-merge(SF, TotalCountsProcess, by="MicrobiomeID")

#One value is NaN in sample 602059 assigns value as average
SF2$CellNorm[is.nan(SF2$CellNorm)]<-mean(na.omit(SF2$CellNorm))
SF2$CellNormStand<-SF2$CellNorm/mean(SF2$CellNorm) #Watch out for NaN values can assign
                                                   #average, but wants the error
SF3<-merge(Metadata2, SF2, by="MicrobiomeID")
#DESeq divides with sizeFactor (High cell count should have low sizeFactor providing
  #larger counts in a sample)
#Still have to consider seq data is relative
SF3$NormRelCell<-SF3$Relative/SF3$CellNormStand

##Select size factors calculated above for normalization
dds@colData@listData$sizeFactor <- SF3[,20]
#hist(dds@colData@listData$sizeFactor)
#colnames(SF3[13])
normname<-colnames(SF3[20])

##See vignette for note on factor levels
#Filtering of the Counttable. Bare minimum is performed later. Also to reduce memory of
#the dds
dds2 <- dds[ rowSums(counts(dds))>50*length(Taxonomy2),] #Previously 0 instead of 1

#Estimate dispersions and fit the GLM
dds2 <- DESeq(dds2, test="LRT", reduced = ~1)

res<-results(dds2)
summary(res)
```

```
##
## out of 197 with nonzero total read count
## adjusted p-value < 0.1
## LFC > 0 (up)       : 15, 7.6%
## LFC < 0 (down)     : 2, 1%
## outliers [1]       : 0, 0%
## low counts [2]     : 0, 0%
## (mean count < 57)
## [1] see 'cooksCutoff' argument of ?results
## [2] see 'independentFiltering' argument of ?results
```

```
resultsNames(dds2)
```

```
## [1] "Intercept"                  "Diagnosis_T1D_vs_Control"
## [3] "Diagnosis_LADA_vs_Control" "Diagnosis_T2D_vs_Control"
```

```
#Data structuring
df <- data.frame(res)
df <- tibble::rownames_to_column(df, "Genera")
#After this it is a select and change naming, then merge
df_log2 <- df %>%  select(one_of("Genera", "log2FoldChange")) %>%
  dplyr::rename("LRT none"="log2FoldChange")

df_padj <- df %>% select(one_of("Genera", "padj")) %>%
  dplyr::rename("LRT none"="padj")









##Wald none
#Create design formula
design <- formula(paste("~ ", "Diagnosis"))
#Create DESeq2 object with matrix and metadata
dds <- DESeqDataSetFromMatrix(countData = Taxonomy2,
                              colData = Metadata2,
                              design = design)

##Select size factors calculated above for normalization
dds@colData@listData$sizeFactor <- SF3[,20]

##See vignette for note on factor levels
#Filtering of the Counttable. Bare minimum is performed later. Also to reduce memory of

#the dds
dds <- dds[ rowSums(counts(dds))>(50*length(Taxonomy2)),] #Previously 0 instead of 1

#Estimate dispersions and fit the GLM
dds <- DESeq(dds)
```

```r
res<-results(dds)
summary(res)
```

```
##
## out of 197 with nonzero total read count
## adjusted p-value < 0.1
## LFC > 0 (up)       : 14, 7.1%
## LFC < 0 (down)     : 0, 0%
## outliers [1]       : 0, 0%
## low counts [2]     : 0, 0%
## (mean count < 57)
## [1] see 'cooksCutoff' argument of ?results
## [2] see 'independentFiltering' argument of ?results
```

```r
resultsNames(dds)
```

```
## [1] "Intercept"                 "Diagnosis_T1D_vs_Control"
## [3] "Diagnosis_LADA_vs_Control" "Diagnosis_T2D_vs_Control"
```

```r
#Threshold for FDR-adjusted p-values
padj_threshold <- 0.1

if (is.factor(colData(dds)[,"Diagnosis"]) ==  TRUE) {

#Find all two-wise combinations of levels in chosen test factor
test <- combn(levels(colData(dds)[,"Diagnosis"]), 2)
test[,4]<-c("LADA", "T1D") #Want LADA first
foo = test[1,]
poo = test[2,]

#Do DESeq2 analysis on all combinations of levels and save as list
result <- vector("list",length(foo))
for (i in 1:length(foo) ) {
  for (j in 1:length(poo)) {
    result[i] = results(dds, contrast = c("Diagnosis" ,foo[i], poo[i]))
  }
}

#Produce output tables with significant taxa from each comparison
res_list <- vector("list",length(foo))
for (i in 1:length(result) ) {
  res_stat <- data.frame(result[i])
  res_stat <- res_stat[is.na(res_stat$pvalue) == FALSE,]
  stat_sig <- na.omit(res_stat[res_stat$padj < padj_threshold,])
  res_stat$gene <- rownames(res_stat)
  res_stat$g1 <- test[1,i]
  res_stat$g2 <- test[2,i]
  res_stat$compare <- paste(test[1,i], "vs", test[2,i])
  res_list[i] <- list(res_stat)
}

res_stat <- do.call('cbind', res_list)
```

```r
#rownames(res_stat) <- 1:nrow(res_stat)
#res_stat <- res_stat[with(res_stat, order(padj, pvalue, abs(log2FoldChange))),]
#stat_sig <- na.omit(res_stat[res_stat$padj < padj_threshold,])
}

((res_stat[,7]==res_stat[,17])==(res_stat[,27]==res_stat[,37]))==
  ((res_stat[,47]==res_stat[,57]))
```

```
##   [1] TRUE TRUE TRUE TRUE TRUE TRUE TRUE TRUE TRUE TRUE TRUE TRUE TRUE TRUE TRUE
##  [16] TRUE TRUE TRUE TRUE TRUE TRUE TRUE TRUE TRUE TRUE TRUE TRUE TRUE TRUE TRUE
##  [31] TRUE TRUE TRUE TRUE TRUE TRUE TRUE TRUE TRUE TRUE TRUE TRUE TRUE TRUE TRUE
##  [46] TRUE TRUE TRUE TRUE TRUE TRUE TRUE TRUE TRUE TRUE TRUE TRUE TRUE TRUE TRUE
##  [61] TRUE TRUE TRUE TRUE TRUE TRUE TRUE TRUE TRUE TRUE TRUE TRUE TRUE TRUE TRUE
##  [76] TRUE TRUE TRUE TRUE TRUE TRUE TRUE TRUE TRUE TRUE TRUE TRUE TRUE TRUE TRUE
##  [91] TRUE TRUE TRUE TRUE TRUE TRUE TRUE TRUE TRUE TRUE TRUE TRUE TRUE TRUE TRUE
## [106] TRUE TRUE TRUE TRUE TRUE TRUE TRUE TRUE TRUE TRUE TRUE TRUE TRUE TRUE TRUE
## [121] TRUE TRUE TRUE TRUE TRUE TRUE TRUE TRUE TRUE TRUE TRUE TRUE TRUE TRUE TRUE
## [136] TRUE TRUE TRUE TRUE TRUE TRUE TRUE TRUE TRUE TRUE TRUE TRUE TRUE TRUE TRUE
## [151] TRUE TRUE TRUE TRUE TRUE TRUE TRUE TRUE TRUE TRUE TRUE TRUE TRUE TRUE TRUE
## [166] TRUE TRUE TRUE TRUE TRUE TRUE TRUE TRUE TRUE TRUE TRUE TRUE TRUE TRUE TRUE
## [181] TRUE TRUE TRUE TRUE TRUE TRUE TRUE TRUE TRUE TRUE TRUE TRUE TRUE TRUE TRUE
## [196] TRUE TRUE
```

```r
#testordernone<-res_stat[2,c(10,20,30,40,50,60)]
df_log2_merge <- res_stat[,c(7,12,2,22,32,52,42)]
colnames(df_log2_merge)<- c("Genera", "No adj - Control vs. LADA", "No adj - Control vs. T1D",
                            "No adj - Control vs. T2D", "No adj - LADA vs. T1D",
                            "No adj - LADA vs. T2D", "No adj - T1D vs. T2D")
df_log2 <- merge(df_log2, df_log2_merge, by="Genera")

df_padj_merge <- res_stat[,c(7,16,6,26,36,56,46)]
colnames(df_padj_merge)<- c("Genera", "No adj - Control vs. LADA", "No adj - Control vs. T1D",
                            "No adj - Control vs. T2D", "No adj - LADA vs. T1D",
                            "No adj - LADA vs. T2D", "No adj - T1D vs. T2D")
df_padj <- merge(df_padj, df_padj_merge, by="Genera")







##LRT BMIq
#Create design formula
design <- formula(paste("~ ", "Diagnosis", "+", "BMIq"))
#Create DESeq2 object with matrix and metadata
dds <- DESeqDataSetFromMatrix(countData = Taxonomy2,
                              colData = Metadata2,
                              design = design)



##Assess and calculate size factors using the different methods
SF<-data.frame(Ratio=sizeFactors(estimateSizeFactors(dds, type="ratio")),
```

```
              Poscounts=sizeFactors(estimateSizeFactors(dds, type="poscounts")),
              Iterate=sizeFactors(estimateSizeFactors(dds, type="poscounts")),
              #"iterate" takes a lot of time changed to "poscounts" but kept due to the
              #following code
              Relative=colSums(Taxonomy2)/mean(colSums(Taxonomy2))
              )
SF<-add_rownames(SF, var="MicrobiomeID")
#Adding total abundances
TotalCounts<-readRDS(path)
TotalCountsProcess <-
  data.frame(MicrobiomeID=TotalCounts$DF_Save_w$ID,
             CellNorm=TotalCounts$DF_Save_w$Cells_per_g_feces_FR_corrected_mean)
TotalCountsProcess$MicrobiomeID <- paste("L", TotalCountsProcess$MicrobiomeID,
                                         sep="")
SF2<-merge(SF, TotalCountsProcess, by="MicrobiomeID")

#One value is NaN in sample 602059 assigns value as average
SF2$CellNorm[is.nan(SF2$CellNorm)]<-mean(na.omit(SF2$CellNorm))
SF2$CellNormStand<-SF2$CellNorm/mean(SF2$CellNorm) #Watch out for NaN values can assign
                                                   #average, but wants the error
SF3<-merge(Metadata2, SF2, by="MicrobiomeID")
#DESeq divides with sizeFactor (High cell count should have low sizeFactor providing
  #larger counts in a sample)
#Still have to consider seq data is relative
SF3$NormRelCell<-SF3$Relative/SF3$CellNormStand

##Select size factors calculated above for normalization
dds@colData@listData$sizeFactor <- SF3[,20]
#hist(dds@colData@listData$sizeFactor)
#colnames(SF3[13])
normname<-colnames(SF3[20])

##See vignette for note on factor levels
#Filtering of the Counttable. Bare minimum is performed later. Also to reduce memory of
#the dds
dds2 <- dds[ rowSums(counts(dds))>50*length(Taxonomy2),] #Previously 0 instead of 1

#Estimate dispersions and fit the GLM
dds2 <- DESeq(dds2, test="LRT", reduced = ~BMIq)

res<-results(dds2)
summary(res)
```

```
##
## out of 197 with nonzero total read count
## adjusted p-value < 0.1
## LFC > 0 (up)       : 11, 5.6%
## LFC < 0 (down)     : 3, 1.5%
## outliers [1]       : 0, 0%
## low counts [2]     : 0, 0%
## (mean count < 64)
## [1] see 'cooksCutoff' argument of ?results
## [2] see 'independentFiltering' argument of ?results
```

```
resultsNames(dds2)
```

```
## [1] "Intercept"                 "Diagnosis_T1D_vs_Control"
## [3] "Diagnosis_LADA_vs_Control" "Diagnosis_T2D_vs_Control"
## [5] "BMIq"
```

```
#Data structuring
df <- data.frame(res)
df <- tibble::rownames_to_column(df, "Genera")
#After this it is a select and change naming, then merge
df_log2_merge <- df %>%  select(one_of("Genera", "log2FoldChange")) %>%
  dplyr::rename("LRT BMI"="log2FoldChange")
df_log2 <- merge(df_log2, df_log2_merge, by="Genera")

df_padj_merge <- df %>% select(one_of("Genera", "padj")) %>%
  dplyr::rename("LRT BMI"="padj")
df_padj <- merge(df_padj, df_padj_merge, by="Genera")







##Wald BMI
#Create design formula
design <- formula(paste("~ ", "Diagnosis", "+", "BMIq"))
#Create DESeq2 object with matrix and metadata
dds <- DESeqDataSetFromMatrix(countData = Taxonomy2,
                              colData = Metadata2,
                              design = design)

##Select size factors calculated above for normalization
dds@colData@listData$sizeFactor <- SF3[,20]

##See vignette for note on factor levels
#Filtering of the Counttable. Bare minimum is performed later. Also to reduce memory of

#the dds
dds <- dds[ rowSums(counts(dds))>(50*length(Taxonomy2)),] #Previously 0 instead of 1

#Estimate dispersions and fit the GLM
dds <- DESeq(dds)

res<-results(dds)
summary(res)
```

```
##
## out of 197 with nonzero total read count
## adjusted p-value < 0.1
## LFC > 0 (up)       : 0, 0%
## LFC < 0 (down)     : 0, 0%
## outliers [1]       : 0, 0%
## low counts [2]     : 0, 0%
```

```
## (mean count < 64)
## [1] see 'cooksCutoff' argument of ?results
## [2] see 'independentFiltering' argument of ?results
```

```r
resultsNames(dds)
```

```
## [1] "Intercept"                  "Diagnosis_T1D_vs_Control"
## [3] "Diagnosis_LADA_vs_Control" "Diagnosis_T2D_vs_Control"
## [5] "BMIq"
```

```r
#Threshold for FDR-adjusted p-values
padj_threshold <- 0.1

if (is.factor(colData(dds)[,"Diagnosis"]) ==  TRUE) {

#Find all two-wise combinations of levels in chosen test factor
test <- combn(levels(colData(dds)[,"Diagnosis"]), 2)
test[,4]<-c("LADA", "T1D") #Want LADA first
foo = test[1,]
poo = test[2,]

#Do DESeq2 analysis on all combinations of levels and save as list
result <- vector("list",length(foo))
for (i in 1:length(foo) ) {
  for (j in 1:length(poo)) {
    result[i] = results(dds, contrast = c("Diagnosis" ,foo[i], poo[i]))
  }
}

#Produce output tables with significant taxa from each comparison
res_list <- vector("list",length(foo))
for (i in 1:length(result) ) {
  res_stat <- data.frame(result[i])
  res_stat <- res_stat[is.na(res_stat$pvalue) == FALSE,]
  stat_sig <- na.omit(res_stat[res_stat$padj < padj_threshold,])
  res_stat$gene <- rownames(res_stat)
  res_stat$g1 <- test[1,i]
  res_stat$g2 <- test[2,i]
  res_stat$compare <- paste(test[1,i], "vs", test[2,i])
  res_list[i] <- list(res_stat)
}

res_stat <- do.call('cbind', res_list)
#rownames(res_stat) <- 1:nrow(res_stat)
#res_stat <- res_stat[with(res_stat, order(padj, pvalue, abs(log2FoldChange))),]
#stat_sig <- na.omit(res_stat[res_stat$padj < padj_threshold,])
}

((res_stat[,7]==res_stat[,17])==(res_stat[,27]==res_stat[,37]))==
  ((res_stat[,47]==res_stat[,57]))
```

```
##   [1] TRUE TRUE TRUE TRUE TRUE TRUE TRUE TRUE TRUE TRUE TRUE TRUE TRUE TRUE TRUE
##  [16] TRUE TRUE TRUE TRUE TRUE TRUE TRUE TRUE TRUE TRUE TRUE TRUE TRUE TRUE TRUE
```

```
##  [31] TRUE TRUE TRUE TRUE TRUE TRUE TRUE TRUE TRUE TRUE TRUE TRUE TRUE TRUE TRUE
##  [46] TRUE TRUE TRUE TRUE TRUE TRUE TRUE TRUE TRUE TRUE TRUE TRUE TRUE TRUE TRUE
##  [61] TRUE TRUE TRUE TRUE TRUE TRUE TRUE TRUE TRUE TRUE TRUE TRUE TRUE TRUE TRUE
##  [76] TRUE TRUE TRUE TRUE TRUE TRUE TRUE TRUE TRUE TRUE TRUE TRUE TRUE TRUE TRUE
##  [91] TRUE TRUE TRUE TRUE TRUE TRUE TRUE TRUE TRUE TRUE TRUE TRUE TRUE TRUE TRUE
## [106] TRUE TRUE TRUE TRUE TRUE TRUE TRUE TRUE TRUE TRUE TRUE TRUE TRUE TRUE TRUE
## [121] TRUE TRUE TRUE TRUE TRUE TRUE TRUE TRUE TRUE TRUE TRUE TRUE TRUE TRUE TRUE
## [136] TRUE TRUE TRUE TRUE TRUE TRUE TRUE TRUE TRUE TRUE TRUE TRUE TRUE TRUE TRUE
## [151] TRUE TRUE TRUE TRUE TRUE TRUE TRUE TRUE TRUE TRUE TRUE TRUE TRUE TRUE TRUE
## [166] TRUE TRUE TRUE TRUE TRUE TRUE TRUE TRUE TRUE TRUE TRUE TRUE TRUE TRUE TRUE
## [181] TRUE TRUE TRUE TRUE TRUE TRUE TRUE TRUE TRUE TRUE TRUE TRUE TRUE TRUE TRUE
## [196] TRUE TRUE
```

```
#testorderBMI<-res_stat[2,c(10,20,30,40,50,60)]
#testordernone==testorderBMI
df_log2_merge <- res_stat[,c(7,12,2,22,32,52,42)]
colnames(df_log2_merge)<- c("Genera", "BMI - Control vs. LADA", "BMI - Control vs. T1D",
                            "BMI - Control vs. T2D", "BMI - LADA vs. T1D",
                            "BMI - LADA vs. T2D", "BMI - T1D vs. T2D")
df_log2 <- merge(df_log2, df_log2_merge, by="Genera")

df_padj_merge <- res_stat[,c(7,16,6,26,36,56,46)]
colnames(df_padj_merge)<- c("Genera", "BMI - Control vs. LADA", "BMI - Control vs. T1D",
                            "BMI - Control vs. T2D", "BMI - LADA vs. T1D",
                            "BMI - LADA vs. T2D", "BMI - T1D vs. T2D")
df_padj <- merge(df_padj, df_padj_merge, by="Genera")








##LRT metformin
#Instead of adding to formula removes them from datasets since it is only present in some
  #groups
##Removing treated with metformin
#Metadata$Metformin <- ifelse(is.na(Metadata$Metformin), "Unknown", Metadata$Metformin)
Metadata2<-filter(Metadata, !Metformin == 1)
table(Metadata2$Diagnosis)
```

```
##
## Control     T1D    LADA     T2D
##      70      30      12      23
```

```
##Applying subsetting to OTU tables
Taxonomy2<-dplyr::select(Taxonomy, one_of(as.character(Metadata2$MicrobiomeID)))
##Reassign names
#Metadata2<-Metadata
Taxonomy2<-Taxonomy2[which(row.names(Taxonomy) %in% row.names(TaxonomyDA)), ]

#The factor can not be ordered for DESeq to run
Metadata2$Diagnosis<-factor(Metadata2$Diagnosis, ordered=FALSE)
```

```r
#Create design formula
design <- formula(paste("~ ", "Diagnosis"))
#Create DESeq2 object with matrix and metadata
dds <- DESeqDataSetFromMatrix(countData = Taxonomy2,
                              colData = Metadata2,
                              design = design)


##Assess and calculate size factors using the different methods
SF<-data.frame(Ratio=sizeFactors(estimateSizeFactors(dds, type="ratio")),
               Poscounts=sizeFactors(estimateSizeFactors(dds, type="poscounts")),
               Iterate=sizeFactors(estimateSizeFactors(dds, type="poscounts")),
               #"iterate" takes a lot of time changed to "poscounts" but kept due to the
               #following code
               Relative=colSums(Taxonomy2)/mean(colSums(Taxonomy2))
               )
SF<-add_rownames(SF, var="MicrobiomeID")
#Adding total abundances
TotalCounts<-readRDS(path)
TotalCountsProcess <-
  data.frame(MicrobiomeID=TotalCounts$DF_Save_w$ID,
             CellNorm=TotalCounts$DF_Save_w$Cells_per_g_feces_FR_corrected_mean)
TotalCountsProcess$MicrobiomeID <- paste("L", TotalCountsProcess$MicrobiomeID,
                                          sep="")
SF2<-merge(SF, TotalCountsProcess, by="MicrobiomeID")

#One value is NaN in sample 602059 assigns value as average
SF2$CellNorm[is.nan(SF2$CellNorm)]<-mean(na.omit(SF2$CellNorm))
SF2$CellNormStand<-SF2$CellNorm/mean(SF2$CellNorm) #Watch out for NaN values can assign
                                                   #average, but wants the error
SF3<-merge(Metadata2, SF2, by="MicrobiomeID")
#DESeq divides with sizeFactor (High cell count should have low sizeFactor providing
  #larger counts in a sample)
#Still have to consider seq data is relative
SF3$NormRelCell<-SF3$Relative/SF3$CellNormStand

##Select size factors calculated above for normalization
dds@colData@listData$sizeFactor <- SF3[,20]
#hist(dds@colData@listData$sizeFactor)
#colnames(SF3[13])
normname<-colnames(SF3[20])

##See vignette for note on factor levels
#Filtering of the Counttable. Bare minimum is performed later. Also to reduce memory of
#the dds
dds2 <- dds[ rowSums(counts(dds))>40*length(Taxonomy2),] #Previously 0 instead of 1
  #Changed to 40 instead of 50 after subsetting
    #Otherwise Actinomyces and Family.Erysi... are removed

#Estimate dispersions and fit the GLM
dds2 <- DESeq(dds2, test="LRT", reduced = ~1)

res<-results(dds2)
```

```
summary(res)
```

```
##
## out of 197 with nonzero total read count
## adjusted p-value < 0.1
## LFC > 0 (up)       : 0, 0%
## LFC < 0 (down)     : 107, 54%
## outliers [1]       : 0, 0%
## low counts [2]     : 46, 23%
## (mean count < 4917)
## [1] see 'cooksCutoff' argument of ?results
## [2] see 'independentFiltering' argument of ?results
```

```
resultsNames(dds2)
```

```
## [1] "Intercept"                 "Diagnosis_T1D_vs_Control"
## [3] "Diagnosis_LADA_vs_Control" "Diagnosis_T2D_vs_Control"
```

```
#Data structuring
df <- data.frame(res)
df <- tibble::rownames_to_column(df, "Genera")
#After this it is a select and change naming, then merge
df_log2_merge <- df %>%  select(one_of("Genera", "log2FoldChange")) %>%
  dplyr::rename("LRT Metformin"="log2FoldChange")
df_log2 <- merge(df_log2, df_log2_merge, by="Genera")

df_padj_merge <- df %>% select(one_of("Genera", "padj")) %>%
  dplyr::rename("LRT Metformin"="padj")
df_padj <- merge(df_padj, df_padj_merge, by="Genera")

#setdiff(df$Genera, df_log2$Genera)







##Wald Metformin
#Create design formula
design <- formula(paste("~ ", "Diagnosis"))
#Create DESeq2 object with matrix and metadata
dds <- DESeqDataSetFromMatrix(countData = Taxonomy2,
                              colData = Metadata2,
                              design = design)
##Select size factors calculated above for normalization
dds@colData@listData$sizeFactor <- SF3[,20]

##See vignette for note on factor levels
#Filtering of the Counttable. Bare minimum is performed later. Also to reduce memory of

#the dds
dds <- dds[ rowSums(counts(dds))>(40*length(Taxonomy2)),] #Previously 0 instead of 1
```

```r
  #Changed to 40 instead of 50 after subsetting
    #Otherwise Actinomyces and Family.Erysi... are removed

#Estimate dispersions and fit the GLM
dds <- DESeq(dds)

res<-results(dds)
summary(res)
```

```
##
## out of 197 with nonzero total read count
## adjusted p-value < 0.1
## LFC > 0 (up)       : 0, 0%
## LFC < 0 (down)     : 103, 52%
## outliers [1]       : 0, 0%
## low counts [2]     : 42, 21%
## (mean count < 3513)
## [1] see 'cooksCutoff' argument of ?results
## [2] see 'independentFiltering' argument of ?results
```

```r
resultsNames(dds)
```

```
## [1] "Intercept"                 "Diagnosis_T1D_vs_Control"
## [3] "Diagnosis_LADA_vs_Control" "Diagnosis_T2D_vs_Control"
```

```r
#Threshold for FDR-adjusted p-values
padj_threshold <- 0.1

if (is.factor(colData(dds)[,"Diagnosis"]) ==  TRUE) {

#Find all two-wise combinations of levels in chosen test factor
test <- combn(levels(colData(dds)[,"Diagnosis"]), 2)
test[,4]<-c("LADA", "T1D") #Want LADA first
foo = test[1,]
poo = test[2,]

#Do DESeq2 analysis on all combinations of levels and save as list
result <- vector("list",length(foo))
for (i in 1:length(foo) ) {
  for (j in 1:length(poo)) {
    result[i] = results(dds, contrast = c("Diagnosis" ,foo[i], poo[i]))
  }
}

#Produce output tables with significant taxa from each comparison
res_list <- vector("list",length(foo))
for (i in 1:length(result) ) {
  res_stat <- data.frame(result[i])
  res_stat <- res_stat[is.na(res_stat$pvalue) == FALSE,]
  stat_sig <- na.omit(res_stat[res_stat$padj < padj_threshold,])
  res_stat$gene <- rownames(res_stat)
  res_stat$g1 <- test[1,i]
```

```
  res_stat$g2 <- test[2,i]
  res_stat$compare <- paste(test[1,i], "vs", test[2,i])
  res_list[i] <- list(res_stat)
}

res_stat <- do.call('cbind', res_list)
#rownames(res_stat) <- 1:nrow(res_stat)
#res_stat <- res_stat[with(res_stat, order(padj, pvalue, abs(log2FoldChange))),]
#stat_sig <- na.omit(res_stat[res_stat$padj < padj_threshold,])
}

((res_stat[,7]==res_stat[,17])==(res_stat[,27]==res_stat[,37]))==
  ((res_stat[,47]==res_stat[,57]))
```

```
##   [1] TRUE TRUE TRUE TRUE TRUE TRUE TRUE TRUE TRUE TRUE TRUE TRUE TRUE TRUE TRUE
##  [16] TRUE TRUE TRUE TRUE TRUE TRUE TRUE TRUE TRUE TRUE TRUE TRUE TRUE TRUE TRUE
##  [31] TRUE TRUE TRUE TRUE TRUE TRUE TRUE TRUE TRUE TRUE TRUE TRUE TRUE TRUE TRUE
##  [46] TRUE TRUE TRUE TRUE TRUE TRUE TRUE TRUE TRUE TRUE TRUE TRUE TRUE TRUE TRUE
##  [61] TRUE TRUE TRUE TRUE TRUE TRUE TRUE TRUE TRUE TRUE TRUE TRUE TRUE TRUE TRUE
##  [76] TRUE TRUE TRUE TRUE TRUE TRUE TRUE TRUE TRUE TRUE TRUE TRUE TRUE TRUE TRUE
##  [91] TRUE TRUE TRUE TRUE TRUE TRUE TRUE TRUE TRUE TRUE TRUE TRUE TRUE TRUE TRUE
## [106] TRUE TRUE TRUE TRUE TRUE TRUE TRUE TRUE TRUE TRUE TRUE TRUE TRUE TRUE TRUE
## [121] TRUE TRUE TRUE TRUE TRUE TRUE TRUE TRUE TRUE TRUE TRUE TRUE TRUE TRUE TRUE
## [136] TRUE TRUE TRUE TRUE TRUE TRUE TRUE TRUE TRUE TRUE TRUE TRUE TRUE TRUE TRUE
## [151] TRUE TRUE TRUE TRUE TRUE TRUE TRUE TRUE TRUE TRUE TRUE TRUE TRUE TRUE TRUE
## [166] TRUE TRUE TRUE TRUE TRUE TRUE TRUE TRUE TRUE TRUE TRUE TRUE TRUE TRUE TRUE
## [181] TRUE TRUE TRUE TRUE TRUE TRUE TRUE TRUE TRUE TRUE TRUE TRUE TRUE TRUE TRUE
## [196] TRUE TRUE
```

```
#testorderMetformin<-res_stat[2,c(10,20,30,40,50,60)]
#testordernone==testorderMetformin
df_log2_merge <- res_stat[,c(7,12,2,22,32,52,42)]
colnames(df_log2_merge)<- c("Genera", "Metformin - Control vs. LADA", "Metformin - Control vs. T1D",
                            "Metformin - Control vs. T2D", "Metformin - LADA vs. T1D",
                            "Metformin - LADA vs. T2D", "Metformin - T1D vs. T2D")
df_log2 <- merge(df_log2, df_log2_merge, by="Genera")

df_padj_merge <- res_stat[,c(7,16,6,26,36,56,46)]
colnames(df_padj_merge)<- c("Genera", "Metformin - Control vs. LADA", "Metformin - Control vs. T1D",
                            "Metformin - Control vs. T2D", "Metformin - LADA vs. T1D",
                            "Metformin - LADA vs. T2D", "Metformin - T1D vs. T2D")
df_padj <- merge(df_padj, df_padj_merge, by="Genera")













#Subsetting taxonomy when running with metadatamed
```

```r
Metadata2 <- MetadataMed
#Metadata2$BMIq<-factor(Metadata2$BMIq)
Metadata2$med_insulin<-factor(Metadata2$med_insulin)
Metadata2$med_statins<-factor(Metadata2$med_statins)
Metadata2$med_protonpump_inhibitor<-factor(Metadata2$med_protonpump_inhibitor)
##Applying subsetting to OTU tables
Taxonomy2<-dplyr::select(Taxonomy, one_of(as.character(Metadata2$MicrobiomeID)))

##Wald insulin
#Create design formula
design <- formula(paste("~ ", "Diagnosis", "+", "med_insulin"))
#Create DESeq2 object with matrix and metadata
dds <- DESeqDataSetFromMatrix(countData = Taxonomy2,
                              colData = Metadata2,
                              design = design)
##Assess and calculate size factors using the different methods
SF<-data.frame(Ratio=sizeFactors(estimateSizeFactors(dds, type="ratio")),
               Poscounts=sizeFactors(estimateSizeFactors(dds, type="poscounts")),
               Iterate=sizeFactors(estimateSizeFactors(dds, type="poscounts")),
               #"iterate" takes a lot of time changed to "poscounts" but kept due to the
               #following code
               Relative=colSums(Taxonomy2)/mean(colSums(Taxonomy2))
               )
SF<-add_rownames(SF, var="MicrobiomeID")
#Adding total abundances
TotalCounts<-readRDS(path)
TotalCountsProcess <-
  data.frame(MicrobiomeID=TotalCounts$DF_Save_w$ID,
             CellNorm=TotalCounts$DF_Save_w$Cells_per_g_feces_FR_corrected_mean)
TotalCountsProcess$MicrobiomeID <- paste("L", TotalCountsProcess$MicrobiomeID,
                                         sep="")
SF2<-merge(SF, TotalCountsProcess, by="MicrobiomeID")

#One value is NaN in sample 602059 assigns value as average
SF2$CellNorm[is.nan(SF2$CellNorm)]<-mean(na.omit(SF2$CellNorm))
SF2$CellNormStand<-SF2$CellNorm/mean(SF2$CellNorm) #Watch out for NaN values can assign
                                                   #average, but wants the error
SF3<-merge(Metadata2, SF2, by="MicrobiomeID")
#DESeq divides with sizeFactor (High cell count should have low sizeFactor providing
  #larger counts in a sample)
#Still have to consider seq data is relative
SF3$NormRelCell<-SF3$Relative/SF3$CellNormStand
##Select size factors calculated above for normalization
dds@colData@listData$sizeFactor <- SF3[,20]

##See vignette for note on factor levels
#Filtering of the Counttable. Bare minimum is performed later. Also to reduce memory of

#the dds
dds <- dds[ rowSums(counts(dds))>(50*length(Taxonomy2)),] #Previously 0 instead of 1

#Estimate dispersions and fit the GLM
dds <- DESeq(dds)
```

```r
res<-results(dds)
summary(res)
```

```
##
## out of 197 with nonzero total read count
## adjusted p-value < 0.1
## LFC > 0 (up)       : 0, 0%
## LFC < 0 (down)     : 0, 0%
## outliers [1]       : 0, 0%
## low counts [2]     : 0, 0%
## (mean count < 60)
## [1] see 'cooksCutoff' argument of ?results
## [2] see 'independentFiltering' argument of ?results
```

```r
resultsNames(dds)
```

```
## [1] "Intercept"              "Diagnosis_T1D_vs_LADA" "Diagnosis_T2D_vs_LADA"
## [4] "med_insulin_1_vs_0"
```

```r
#Threshold for FDR-adjusted p-values
padj_threshold <- 0.1

if (is.factor(colData(dds)[,"Diagnosis"]) ==  TRUE) {

#Find all two-wise combinations of levels in chosen test factor
test <- combn(levels(colData(dds)[,"Diagnosis"]), 2)
foo = test[1,]
poo = test[2,]

#Do DESeq2 analysis on all combinations of levels and save as list
result <- vector("list",length(foo))
for (i in 1:length(foo) ) {
  for (j in 1:length(poo)) {
    result[i] = results(dds, contrast = c("Diagnosis" ,foo[i], poo[i]))
  }
}

#Produce output tables with significant taxa from each comparison
res_list <- vector("list",length(foo))
for (i in 1:length(result) ) {
  res_stat <- data.frame(result[i])
  res_stat <- res_stat[is.na(res_stat$pvalue) == FALSE,]
  stat_sig <- na.omit(res_stat[res_stat$padj < padj_threshold,])
  res_stat$gene <- rownames(res_stat)
  res_stat$g1 <- test[1,i]
  res_stat$g2 <- test[2,i]
  res_stat$compare <- paste(test[1,i], "vs", test[2,i])
  res_list[i] <- list(res_stat)
}

res_stat <- do.call('cbind', res_list)
#rownames(res_stat) <- 1:nrow(res_stat)
```

```
#res_stat <- res_stat[with(res_stat, order(padj, pvalue, abs(log2FoldChange))),]
#stat_sig <- na.omit(res_stat[res_stat$padj < padj_threshold,])
}

((res_stat[,7]==res_stat[,17])==(res_stat[,27]==res_stat[,7]))
```

```
##   [1] TRUE TRUE TRUE TRUE TRUE TRUE TRUE TRUE TRUE TRUE TRUE TRUE TRUE TRUE TRUE
##  [16] TRUE TRUE TRUE TRUE TRUE TRUE TRUE TRUE TRUE TRUE TRUE TRUE TRUE TRUE TRUE
##  [31] TRUE TRUE TRUE TRUE TRUE TRUE TRUE TRUE TRUE TRUE TRUE TRUE TRUE TRUE TRUE
##  [46] TRUE TRUE TRUE TRUE TRUE TRUE TRUE TRUE TRUE TRUE TRUE TRUE TRUE TRUE TRUE
##  [61] TRUE TRUE TRUE TRUE TRUE TRUE TRUE TRUE TRUE TRUE TRUE TRUE TRUE TRUE TRUE
##  [76] TRUE TRUE TRUE TRUE TRUE TRUE TRUE TRUE TRUE TRUE TRUE TRUE TRUE TRUE TRUE
##  [91] TRUE TRUE TRUE TRUE TRUE TRUE TRUE TRUE TRUE TRUE TRUE TRUE TRUE TRUE TRUE
## [106] TRUE TRUE TRUE TRUE TRUE TRUE TRUE TRUE TRUE TRUE TRUE TRUE TRUE TRUE TRUE
## [121] TRUE TRUE TRUE TRUE TRUE TRUE TRUE TRUE TRUE TRUE TRUE TRUE TRUE TRUE TRUE
## [136] TRUE TRUE TRUE TRUE TRUE TRUE TRUE TRUE TRUE TRUE TRUE TRUE TRUE TRUE TRUE
## [151] TRUE TRUE TRUE TRUE TRUE TRUE TRUE TRUE TRUE TRUE TRUE TRUE TRUE TRUE TRUE
## [166] TRUE TRUE TRUE TRUE TRUE TRUE TRUE TRUE TRUE TRUE TRUE TRUE TRUE TRUE TRUE
## [181] TRUE TRUE TRUE TRUE TRUE TRUE TRUE TRUE TRUE TRUE TRUE TRUE TRUE TRUE TRUE
## [196] TRUE TRUE
```

```
#res_stat[2,c(10,20,30)]
#testordernone==testorderBMI
df_log2_merge <- res_stat[,c(7,2,12,22)]
colnames(df_log2_merge)<- c("Genera", "Insulin - LADA vs. T1D",
                            "Insulin - LADA vs. T2D", "Insulin - T1D vs. T2D")
df_log2 <- merge(df_log2, df_log2_merge, by="Genera")

df_padj_merge <- res_stat[,c(7,6,16,26)]
colnames(df_padj_merge)<- c("Genera", "Insulin - LADA vs. T1D",
                            "Insulin - LADA vs. T2D", "Insulin - T1D vs. T2D")
df_padj <- merge(df_padj, df_padj_merge, by="Genera")






##Wald statins
#Create design formula
design <- formula(paste("~ ", "Diagnosis", "+", "med_statins"))
#Create DESeq2 object with matrix and metadata
dds <- DESeqDataSetFromMatrix(countData = Taxonomy2,
                              colData = Metadata2,
                              design = design)

##Select size factors calculated above for normalization
dds@colData@listData$sizeFactor <- SF3[,20]

##See vignette for note on factor levels
#Filtering of the Counttable. Bare minimum is performed later. Also to reduce memory of

#the dds
```

```
dds <- dds[ rowSums(counts(dds))>(50*length(Taxonomy2)),] #Previously 0 instead of 1

#Estimate dispersions and fit the GLM
dds <- DESeq(dds)

res<-results(dds)
summary(res)
```

```
##
## out of 197 with nonzero total read count
## adjusted p-value < 0.1
## LFC > 0 (up)       : 0, 0%
## LFC < 0 (down)     : 0, 0%
## outliers [1]       : 0, 0%
## low counts [2]     : 0, 0%
## (mean count < 60)
## [1] see 'cooksCutoff' argument of ?results
## [2] see 'independentFiltering' argument of ?results
```

```
resultsNames(dds)
```

```
## [1] "Intercept"             "Diagnosis_T1D_vs_LADA" "Diagnosis_T2D_vs_LADA"
## [4] "med_statins_1_vs_0"
```

```
#Threshold for FDR-adjusted p-values
padj_threshold <- 0.1

if (is.factor(colData(dds)[,"Diagnosis"]) ==  TRUE) {

#Find all two-wise combinations of levels in chosen test factor
test <- combn(levels(colData(dds)[,"Diagnosis"]), 2)
foo = test[1,]
poo = test[2,]

#Do DESeq2 analysis on all combinations of levels and save as list
result <- vector("list",length(foo))
for (i in 1:length(foo) ) {
  for (j in 1:length(poo)) {
    result[i] = results(dds, contrast = c("Diagnosis" ,foo[i], poo[i]))
  }
}

#Produce output tables with significant taxa from each comparison
res_list <- vector("list",length(foo))
for (i in 1:length(result) ) {
  res_stat <- data.frame(result[i])
  res_stat <- res_stat[is.na(res_stat$pvalue) == FALSE,]
  stat_sig <- na.omit(res_stat[res_stat$padj < padj_threshold,])
  res_stat$gene <- rownames(res_stat)
  res_stat$g1 <- test[1,i]
  res_stat$g2 <- test[2,i]
  res_stat$compare <- paste(test[1,i], "vs", test[2,i])
```

```
  res_list[i] <- list(res_stat)
}

res_stat <- do.call('cbind', res_list)
#rownames(res_stat) <- 1:nrow(res_stat)
#res_stat <- res_stat[with(res_stat, order(padj, pvalue, abs(log2FoldChange))),]
#stat_sig <- na.omit(res_stat[res_stat$padj < padj_threshold,])
}

((res_stat[,7]==res_stat[,17])==(res_stat[,27]==res_stat[,7]))
```

```
##   [1] TRUE TRUE TRUE TRUE TRUE TRUE TRUE TRUE TRUE TRUE TRUE TRUE TRUE TRUE TRUE
##  [16] TRUE TRUE TRUE TRUE TRUE TRUE TRUE TRUE TRUE TRUE TRUE TRUE TRUE TRUE TRUE
##  [31] TRUE TRUE TRUE TRUE TRUE TRUE TRUE TRUE TRUE TRUE TRUE TRUE TRUE TRUE TRUE
##  [46] TRUE TRUE TRUE TRUE TRUE TRUE TRUE TRUE TRUE TRUE TRUE TRUE TRUE TRUE TRUE
##  [61] TRUE TRUE TRUE TRUE TRUE TRUE TRUE TRUE TRUE TRUE TRUE TRUE TRUE TRUE TRUE
##  [76] TRUE TRUE TRUE TRUE TRUE TRUE TRUE TRUE TRUE TRUE TRUE TRUE TRUE TRUE TRUE
##  [91] TRUE TRUE TRUE TRUE TRUE TRUE TRUE TRUE TRUE TRUE TRUE TRUE TRUE TRUE TRUE
## [106] TRUE TRUE TRUE TRUE TRUE TRUE TRUE TRUE TRUE TRUE TRUE TRUE TRUE TRUE TRUE
## [121] TRUE TRUE TRUE TRUE TRUE TRUE TRUE TRUE TRUE TRUE TRUE TRUE TRUE TRUE TRUE
## [136] TRUE TRUE TRUE TRUE TRUE TRUE TRUE TRUE TRUE TRUE TRUE TRUE TRUE TRUE TRUE
## [151] TRUE TRUE TRUE TRUE TRUE TRUE TRUE TRUE TRUE TRUE TRUE TRUE TRUE TRUE TRUE
## [166] TRUE TRUE TRUE TRUE TRUE TRUE TRUE TRUE TRUE TRUE TRUE TRUE TRUE TRUE TRUE
## [181] TRUE TRUE TRUE TRUE TRUE TRUE TRUE TRUE TRUE TRUE TRUE TRUE TRUE TRUE TRUE
## [196] TRUE TRUE
```

```
#res_stat[2,c(10,20,30)]
#testordernone==testorderBMI
df_log2_merge <- res_stat[,c(7,2,12,22)]
colnames(df_log2_merge)<- c("Genera", "Statins - LADA vs. T1D",
                            "Statins - LADA vs. T2D", "Statins - T1D vs. T2D")
df_log2 <- merge(df_log2, df_log2_merge, by="Genera")

df_padj_merge <- res_stat[,c(7,6,16,26)]
colnames(df_padj_merge)<- c("Genera", "Statins - LADA vs. T1D",
                            "Statins - LADA vs. T2D", "Statins - T1D vs. T2D")
df_padj <- merge(df_padj, df_padj_merge, by="Genera")









##Wald proton pump inhibitors
#Create design formula
design <- formula(paste("~ ", "Diagnosis", "+", "med_protonpump_inhibitor"))
#Create DESeq2 object with matrix and metadata
dds <- DESeqDataSetFromMatrix(countData = Taxonomy2,
                              colData = Metadata2,
                              design = design)
```

```r
##Select size factors calculated above for normalization
dds@colData@listData$sizeFactor <- SF3[,20]

##See vignette for note on factor levels
#Filtering of the Counttable. Bare minimum is performed later. Also to reduce memory of

#the dds
dds <- dds[ rowSums(counts(dds))>(50*length(Taxonomy2)),] #Previously 0 instead of 1

#Estimate dispersions and fit the GLM
dds <- DESeq(dds)

res<-results(dds)
summary(res)
```

```
##
## out of 197 with nonzero total read count
## adjusted p-value < 0.1
## LFC > 0 (up)       : 0, 0%
## LFC < 0 (down)     : 2, 1%
## outliers [1]       : 0, 0%
## low counts [2]     : 0, 0%
## (mean count < 73)
## [1] see 'cooksCutoff' argument of ?results
## [2] see 'independentFiltering' argument of ?results
```

```r
resultsNames(dds)
```

```
## [1] "Intercept"                    "Diagnosis_T1D_vs_LADA"
## [3] "Diagnosis_T2D_vs_LADA"        "med_protonpump_inhibitor_1_vs_0"
```

```r
#Threshold for FDR-adjusted p-values
padj_threshold <- 0.1

if (is.factor(colData(dds)[,"Diagnosis"]) ==  TRUE) {

#Find all two-wise combinations of levels in chosen test factor
test <- combn(levels(colData(dds)[,"Diagnosis"]), 2)
foo = test[1,]
poo = test[2,]

#Do DESeq2 analysis on all combinations of levels and save as list
result <- vector("list",length(foo))
for (i in 1:length(foo) ) {
  for (j in 1:length(poo)) {
    result[i] = results(dds, contrast = c("Diagnosis" ,foo[i], poo[i]))
  }
}

#Produce output tables with significant taxa from each comparison
res_list <- vector("list",length(foo))
for (i in 1:length(result) ) {
```

```r
  res_stat <- data.frame(result[i])
  res_stat <- res_stat[is.na(res_stat$pvalue) == FALSE,]
  stat_sig <- na.omit(res_stat[res_stat$padj < padj_threshold,])
  res_stat$gene <- rownames(res_stat)
  res_stat$g1 <- test[1,i]
  res_stat$g2 <- test[2,i]
  res_stat$compare <- paste(test[1,i], "vs", test[2,i])
  res_list[i] <- list(res_stat)
}

res_stat <- do.call('cbind', res_list)
#rownames(res_stat) <- 1:nrow(res_stat)
#res_stat <- res_stat[with(res_stat, order(padj, pvalue, abs(log2FoldChange))),]
#stat_sig <- na.omit(res_stat[res_stat$padj < padj_threshold,])
}

((res_stat[,7]==res_stat[,17])==(res_stat[,27]==res_stat[,7]))
```

```
##   [1] TRUE TRUE TRUE TRUE TRUE TRUE TRUE TRUE TRUE TRUE TRUE TRUE TRUE TRUE TRUE
##  [16] TRUE TRUE TRUE TRUE TRUE TRUE TRUE TRUE TRUE TRUE TRUE TRUE TRUE TRUE TRUE
##  [31] TRUE TRUE TRUE TRUE TRUE TRUE TRUE TRUE TRUE TRUE TRUE TRUE TRUE TRUE TRUE
##  [46] TRUE TRUE TRUE TRUE TRUE TRUE TRUE TRUE TRUE TRUE TRUE TRUE TRUE TRUE TRUE
##  [61] TRUE TRUE TRUE TRUE TRUE TRUE TRUE TRUE TRUE TRUE TRUE TRUE TRUE TRUE TRUE
##  [76] TRUE TRUE TRUE TRUE TRUE TRUE TRUE TRUE TRUE TRUE TRUE TRUE TRUE TRUE TRUE
##  [91] TRUE TRUE TRUE TRUE TRUE TRUE TRUE TRUE TRUE TRUE TRUE TRUE TRUE TRUE TRUE
## [106] TRUE TRUE TRUE TRUE TRUE TRUE TRUE TRUE TRUE TRUE TRUE TRUE TRUE TRUE TRUE
## [121] TRUE TRUE TRUE TRUE TRUE TRUE TRUE TRUE TRUE TRUE TRUE TRUE TRUE TRUE TRUE
## [136] TRUE TRUE TRUE TRUE TRUE TRUE TRUE TRUE TRUE TRUE TRUE TRUE TRUE TRUE TRUE
## [151] TRUE TRUE TRUE TRUE TRUE TRUE TRUE TRUE TRUE TRUE TRUE TRUE TRUE TRUE TRUE
## [166] TRUE TRUE TRUE TRUE TRUE TRUE TRUE TRUE TRUE TRUE TRUE TRUE TRUE TRUE TRUE
## [181] TRUE TRUE TRUE TRUE TRUE TRUE TRUE TRUE TRUE TRUE TRUE TRUE TRUE TRUE TRUE
## [196] TRUE TRUE
```

```r
#res_stat[2,c(10,20,30)]
#testordernone==testorderBMI
df_log2_merge <- res_stat[,c(7,2,12,22)]
colnames(df_log2_merge)<- c("Genera", "PPI - LADA vs. T1D",
                            "PPI - LADA vs. T2D", "PPI - T1D vs. T2D")
df_log2 <- merge(df_log2, df_log2_merge, by="Genera")

df_padj_merge <- res_stat[,c(7,6,16,26)]
colnames(df_padj_merge)<- c("Genera", "PPI - LADA vs. T1D",
                            "PPI - LADA vs. T2D", "PPI - T1D vs. T2D")
df_padj <- merge(df_padj, df_padj_merge, by="Genera")








##Wald all
#Create design formula
```

```r
design <- formula(paste("~ ", "Diagnosis",
                        "+", "BMIq",
                        #"+", "Metformin",
                        "+", "med_insulin",
                        "+", "med_statins",
                        "+", "med_protonpump_inhibitor"))
#Create DESeq2 object with matrix and metadata
dds <- DESeqDataSetFromMatrix(countData = Taxonomy2,
                              colData = Metadata2,
                              design = design)

##Select size factors calculated above for normalization
dds@colData@listData$sizeFactor <- SF3[,20]

##See vignette for note on factor levels
#Filtering of the Counttable. Bare minimum is performed later. Also to reduce memory of

#the dds
dds <- dds[ rowSums(counts(dds))>(50*length(Taxonomy2)),] #Previously 0 instead of 1

#Estimate dispersions and fit the GLM
dds <- DESeq(dds)

res<-results(dds)
summary(res)
```

```
##
## out of 197 with nonzero total read count
## adjusted p-value < 0.1
## LFC > 0 (up)       : 0, 0%
## LFC < 0 (down)     : 2, 1%
## outliers [1]       : 0, 0%
## low counts [2]     : 0, 0%
## (mean count < 73)
## [1] see 'cooksCutoff' argument of ?results
## [2] see 'independentFiltering' argument of ?results
```

```r
resultsNames(dds)
```

```
## [1] "Intercept"                       "Diagnosis_T1D_vs_LADA"
## [3] "Diagnosis_T2D_vs_LADA"           "BMIq"
## [5] "med_insulin_1_vs_0"              "med_statins_1_vs_0"
## [7] "med_protonpump_inhibitor_1_vs_0"
```

```r
#Threshold for FDR-adjusted p-values
padj_threshold <- 0.1

if (is.factor(colData(dds)[,"Diagnosis"]) ==  TRUE) {

#Find all two-wise combinations of levels in chosen test factor
test <- combn(levels(colData(dds)[,"Diagnosis"]), 2)
foo = test[1,]
```

```r
poo = test[2,]

#Do DESeq2 analysis on all combinations of levels and save as list
result <- vector("list",length(foo))
for (i in 1:length(foo) ) {
  for (j in 1:length(poo)) {
    result[i] = results(dds, contrast = c("Diagnosis" ,foo[i], poo[i]))
  }
}

#Produce output tables with significant taxa from each comparison
res_list <- vector("list",length(foo))
for (i in 1:length(result) ) {
  res_stat <- data.frame(result[i])
  res_stat <- res_stat[is.na(res_stat$pvalue) == FALSE,]
  stat_sig <- na.omit(res_stat[res_stat$padj < padj_threshold,])
  res_stat$gene <- rownames(res_stat)
  res_stat$g1 <- test[1,i]
  res_stat$g2 <- test[2,i]
  res_stat$compare <- paste(test[1,i], "vs", test[2,i])
  res_list[i] <- list(res_stat)
}

res_stat <- do.call('cbind', res_list)
#rownames(res_stat) <- 1:nrow(res_stat)
#res_stat <- res_stat[with(res_stat, order(padj, pvalue, abs(log2FoldChange))),]
#stat_sig <- na.omit(res_stat[res_stat$padj < padj_threshold,])
}

((res_stat[,7]==res_stat[,17])==(res_stat[,27]==res_stat[,7]))
```

```
##   [1] TRUE TRUE TRUE TRUE TRUE TRUE TRUE TRUE TRUE TRUE TRUE TRUE TRUE TRUE TRUE
##  [16] TRUE TRUE TRUE TRUE TRUE TRUE TRUE TRUE TRUE TRUE TRUE TRUE TRUE TRUE TRUE
##  [31] TRUE TRUE TRUE TRUE TRUE TRUE TRUE TRUE TRUE TRUE TRUE TRUE TRUE TRUE TRUE
##  [46] TRUE TRUE TRUE TRUE TRUE TRUE TRUE TRUE TRUE TRUE TRUE TRUE TRUE TRUE TRUE
##  [61] TRUE TRUE TRUE TRUE TRUE TRUE TRUE TRUE TRUE TRUE TRUE TRUE TRUE TRUE TRUE
##  [76] TRUE TRUE TRUE TRUE TRUE TRUE TRUE TRUE TRUE TRUE TRUE TRUE TRUE TRUE TRUE
##  [91] TRUE TRUE TRUE TRUE TRUE TRUE TRUE TRUE TRUE TRUE TRUE TRUE TRUE TRUE TRUE
## [106] TRUE TRUE TRUE TRUE TRUE TRUE TRUE TRUE TRUE TRUE TRUE TRUE TRUE TRUE TRUE
## [121] TRUE TRUE TRUE TRUE TRUE TRUE TRUE TRUE TRUE TRUE TRUE TRUE TRUE TRUE TRUE
## [136] TRUE TRUE TRUE TRUE TRUE TRUE TRUE TRUE TRUE TRUE TRUE TRUE TRUE TRUE TRUE
## [151] TRUE TRUE TRUE TRUE TRUE TRUE TRUE TRUE TRUE TRUE TRUE TRUE TRUE TRUE TRUE
## [166] TRUE TRUE TRUE TRUE TRUE TRUE TRUE TRUE TRUE TRUE TRUE TRUE TRUE TRUE TRUE
## [181] TRUE TRUE TRUE TRUE TRUE TRUE TRUE TRUE TRUE TRUE TRUE TRUE TRUE TRUE TRUE
## [196] TRUE TRUE
```

```r
#res_stat[2,c(10,20,30)]
#testordernone==testorderBMI
df_log2_merge <- res_stat[,c(7,2,12,22)]
colnames(df_log2_merge)<- c("Genera", "All - LADA vs. T1D",
                            "All - LADA vs. T2D", "All - T1D vs. T2D")
df_log2 <- merge(df_log2, df_log2_merge, by="Genera")
```

```r
df_padj_merge <- res_stat[,c(7,6,16,26)]
colnames(df_padj_merge)<- c("Genera", "All - LADA vs. T1D",
                            "All - LADA vs. T2D", "All - T1D vs. T2D")
df_padj <- merge(df_padj, df_padj_merge, by="Genera")






#GeneraList1 <-df_padj[as.numeric(cut_number(df_padj$`LRT none`,4))==1, 1]
#GeneraList2 <-df_padj[as.numeric(cut_number(df_padj$`LRT none`,4))==2, 1]
#GeneraList3 <-df_padj[as.numeric(cut_number(df_padj$`LRT none`,4))==3, 1]
#GeneraList4 <-df_padj[as.numeric(cut_number(df_padj$`LRT none`,4))==4, 1]

#Filter or merge according to list of genera wanting to include.
#GeneraList<-c("Actinomyces", "Roseburia", "Bifidobacterium", "Odoribacter",
#              "Lactobacillus", "Faecalibacterium")
#Based on lowest padj from LRT none. #Below median of LRT none
GeneraList <- df_padj[as.numeric(cut_number(df_padj$`LRT none`,4))==1, 1]
df_log2_plot<-subset(df_log2, Genera %in% GeneraList)
df_padj_plot<-subset(df_padj, Genera %in% GeneraList)


#Change genera to rownames
row.names(df_log2_plot) <- df_log2_plot$Genera
df_log2_plot <- select(df_log2_plot, -one_of("Genera"))

row.names(df_padj_plot) <- df_padj_plot$Genera
df_padj_plot <- select(df_padj_plot, -one_of("Genera"))


#Make symbol according to significance level
df_padj_plotsym<-df_padj_plot #Gets a df with same dimensions
#overrides so can just go from lowest and up
for (i in 1:nrow(df_padj_plotsym)) {
  for (j in 1:ncol(df_padj_plotsym)) {
    ifelse(df_padj_plot[i,j]<0.001, df_padj_plotsym[i,j]<-"***",
           ifelse(df_padj_plot[i,j]<0.01, df_padj_plotsym[i,j]<-"**",
                  ifelse(df_padj_plot[i,j]<0.05, df_padj_plotsym[i,j]<-"*",
    ifelse(df_padj_plot[i,j]<0.1, df_padj_plotsym[i,j]<-"\U00B7",
           ifelse(df_padj_plot[i,j]<2, df_padj_plotsym[i,j]<-"", "hmmm"))))) #2 values range 0-1
      #knows it is less
  }
}


max(abs(df_log2_plot))
```

```
## [1] 3.016542
```

```r
range <- max(abs(df_log2_plot))

#Create heatmap
```

```r
range(df_log2_plot)
```

```
## [1] -2.776998  3.016542
```

```r
pheatmap(df_log2_plot,
         display_numbers = df_padj_plotsym,
         gaps_col = c(7, 14, 21, 24, 27, 30),
         cluster_rows=FALSE,
         show_rownames=TRUE,
         cluster_cols=FALSE,
         show_colnames=TRUE,
         fontsize_number=20,
         legend=TRUE,
         breaks = seq(-range, range, length.out = 30), #Colors centered at 0
         color=colorRampPalette(c("blue", "white", "red"))(30))
```

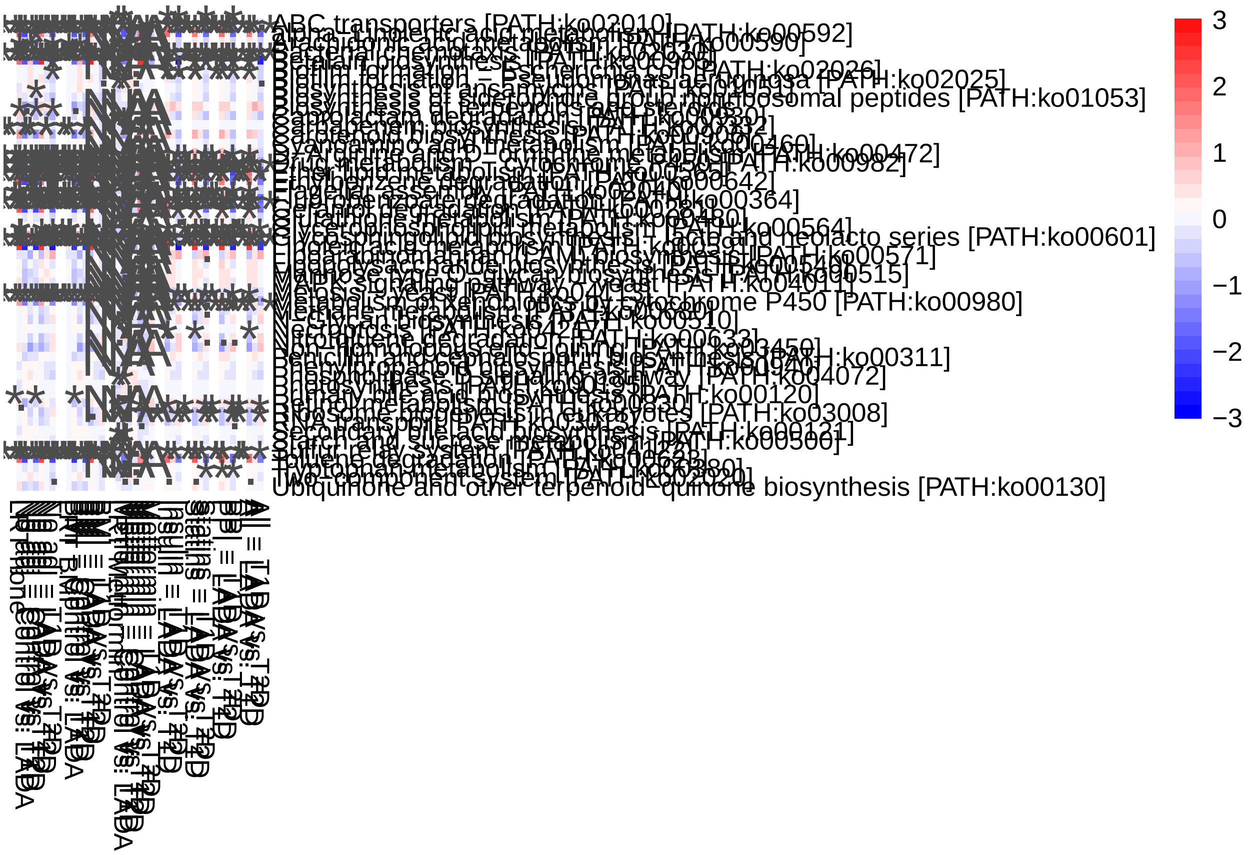


```r
#Draw the heatmap
dev.off()
```

```
## pdf
##   3
```

```
pdf(paste("MicroLADA_Figure3DESeqHeatmap1_Func", ".pdf", sep=""), width=10, height=7)
pheatmap(df_log2_plot,
         display_numbers = df_padj_plotsym,
         gaps_col = c(7, 14, 21, 24, 27, 30),
         cluster_rows=FALSE,
         show_rownames=TRUE,
         cluster_cols=FALSE,
         show_colnames=TRUE,
         fontsize_number=10,
         legend=TRUE,
         breaks = seq(-range, range, length.out = 30),
         color=colorRampPalette(c("blue", "white", "red"))(30))

dev.off()
```

```
## pdf
##   3
```

```
#Make density plot
#In the end a solution with new_scale_fill and defining two background datasets
df_density<-data.frame(values=as.vector(as.matrix(df_log2_plot)))
xvalues <- c(-7, 7)
coloring<-c(colorRampPalette(c("red", "white", "red"))(30))
coloring2<-c(colorRampPalette(c("blue", "white", "blue"))(30))
background <- data.frame(lower = seq( xvalues[1], xvalues[2]-sum(abs(xvalues))/30,
                                      sum(abs(xvalues))/30 ),
                         upper = seq( xvalues[1]+sum(abs(xvalues))/30, xvalues[2],
                                      sum(abs(xvalues))/30 ),
                         col = coloring,
                         col2 = coloring2)

background1<-background[1:15,]
background2<-background[16:30,]

ggplot() +
    geom_rect(data = background1 ,
              mapping = aes(xmin = lower ,
                            xmax = upper ,
                            ymin = 0 ,
                            ymax = 0.7 ,
                            fill = col ) ,
              alpha = .9 ) +
    scale_fill_manual(values = alpha(background1$col2, 0.9))+
    new_scale_fill()+
    geom_rect(data = background2 ,
              mapping = aes(xmin = lower ,
                            xmax = upper ,
                            ymin = 0 ,
                            ymax = 0.7 ,
                            fill = col2 ) ,
              alpha = .9 ) +
    scale_fill_manual(values = alpha(background1$col, 0.9)) +
    theme_classic()+
```

```
    geom_density(data = df_density, aes(x=values), size=2) +
    theme_classic() +
    xlab("Log2-fold change") +
    ylab("Density") +
    theme(legend.position = "none")

pdf(paste("MicroLADA_Figure3DESeqHeatmap1density_Func", ".pdf", sep=""), width=6, height=4)
ggplot() +
    geom_rect(data = background1 ,
              mapping = aes(xmin = lower ,
                            xmax = upper ,
                            ymin = 0 ,
                            ymax = 2 ,
                            fill = col ) ,
              alpha = .9 ) +
    scale_fill_manual(values = alpha(background1$col2, 0.9))+
    new_scale_fill()+
    geom_rect(data = background2 ,
              mapping = aes(xmin = lower ,
                            xmax = upper ,
                            ymin = 0 ,
                            ymax = 2 ,
                            fill = col2 ) ,
              alpha = .9 ) +
    scale_fill_manual(values = alpha(background1$col, 0.9)) +
    theme_classic()+
    geom_density(data = df_density, aes(x=values), size=2) +
    theme_classic() +
    xlab("Log2-fold change") +
    ylab("Density") +
    theme(legend.position = "none")
dev.off()
```

```
## pdf
##   3
```

```
##Here the next functions
#Filter or merge according to list of genera wanting to include.
#GeneraList<-c("Actinomyces", "Roseburia", "Bifidobacterium", "Odoribacter",
#               "Lactobacillus", "Faecalibacterium")
#Based on lowest padj from LRT none. #Below median of LRT none
GeneraList <- df_padj[as.numeric(cut_number(df_padj$`LRT none`,4))==2, 1]
df_log2_plot<-subset(df_log2, Genera %in% GeneraList)
df_padj_plot<-subset(df_padj, Genera %in% GeneraList)


#Change genera to rownames
row.names(df_log2_plot) <- df_log2_plot$Genera
df_log2_plot <- select(df_log2_plot, -one_of("Genera"))

row.names(df_padj_plot) <- df_padj_plot$Genera
df_padj_plot <- select(df_padj_plot, -one_of("Genera"))
```

```
#Make symbol according to significance level
df_padj_plotsym<-df_padj_plot #Gets a df with same dimensions
#overrides so can just go from lowest and up
for (i in 1:nrow(df_padj_plotsym)) {
  for (j in 1:ncol(df_padj_plotsym)) {
    ifelse(df_padj_plot[i,j]<0.001, df_padj_plotsym[i,j]<-"***",
           ifelse(df_padj_plot[i,j]<0.01, df_padj_plotsym[i,j]<-"**",
                  ifelse(df_padj_plot[i,j]<0.05, df_padj_plotsym[i,j]<-"*",
    ifelse(df_padj_plot[i,j]<0.1, df_padj_plotsym[i,j]<-"\U00B7",
           ifelse(df_padj_plot[i,j]<2, df_padj_plotsym[i,j]<-"", "hmmm"))))) #2 values range 0-1
      #knows it is less
  }
}


#max(abs(df_log2_plot))
#range <- max(abs(df_log2_plot)) #Would rather use the previous to make the heatmaps
  #directly comparable

#Create heatmap
range(df_log2_plot)
```

```
## [1] -2.579323  1.264146
```

```
pheatmap(df_log2_plot,
         display_numbers = df_padj_plotsym,
         gaps_col = c(7, 14, 21, 24, 27, 30),
         cluster_rows=FALSE,
         show_rownames=TRUE,
         cluster_cols=FALSE,
         show_colnames=TRUE,
         fontsize_number=20,
         legend=TRUE,
         breaks = seq(-range, range, length.out = 30), #Colors centered at 0
         color=colorRampPalette(c("blue", "white", "red"))(30))


#Draw the heatmap
dev.off()
```

```
## null device
##           1
```

```
pdf(paste("MicroLADA_Figure3DESeqHeatmap2_Func", ".pdf", sep=""), width=10, height=7)
pheatmap(df_log2_plot,
         display_numbers = df_padj_plotsym,
         gaps_col = c(7, 14, 21, 24, 27, 30),
         cluster_rows=FALSE,
         show_rownames=TRUE,
         cluster_cols=FALSE,
         show_colnames=TRUE,
         fontsize_number=10,
```

```
        legend=TRUE,
        breaks = seq(-range, range, length.out = 30),
        color=colorRampPalette(c("blue", "white", "red"))(30))

dev.off()
```

```
## null device
##           1
```

```
#Make density plot
#In the end a solution with new_scale_fill and defining two background datasets
df_density<-data.frame(values=as.vector(as.matrix(df_log2_plot)))
xvalues <- c(-7, 7)
coloring<-c(colorRampPalette(c("red", "white", "red"))(30))
coloring2<-c(colorRampPalette(c("blue", "white", "blue"))(30))
background <- data.frame(lower = seq( xvalues[1], xvalues[2]-sum(abs(xvalues))/30,
                                      sum(abs(xvalues))/30 ),
                         upper = seq( xvalues[1]+sum(abs(xvalues))/30, xvalues[2],
                                      sum(abs(xvalues))/30 ),
                         col = coloring,
                         col2 = coloring2)

background1<-background[1:15,]
background2<-background[16:30,]

ggplot() +
    geom_rect(data = background1 ,
              mapping = aes(xmin = lower ,
                            xmax = upper ,
                            ymin = 0 ,
                            ymax = 3 ,
                            fill = col ) ,
              alpha = .9 ) +
    scale_fill_manual(values = alpha(background1$col2, 0.9))+
    new_scale_fill()+
    geom_rect(data = background2 ,
              mapping = aes(xmin = lower ,
                            xmax = upper ,
                            ymin = 0 ,
                            ymax = 3 ,
                            fill = col2 ) ,
              alpha = .9 ) +
    scale_fill_manual(values = alpha(background1$col, 0.9)) +
    theme_classic()+
    geom_density(data = df_density, aes(x=values), size=2) +
    theme_classic() +
    xlab("Log2-fold change") +
    ylab("Density") +
    theme(legend.position = "none")


pdf(paste("MicroLADA_Figure3DESeqHeatmap2density_Func", ".pdf", sep=""), width=6, height=4)
ggplot() +
```

```
    geom_rect(data = background1 ,
              mapping = aes(xmin = lower ,
                            xmax = upper ,
                            ymin = 0 ,
                            ymax = 3 ,
                            fill = col ) ,
              alpha = .9 ) +
    scale_fill_manual(values = alpha(background1$col2, 0.9))+
    new_scale_fill()+
    geom_rect(data = background2 ,
              mapping = aes(xmin = lower ,
                            xmax = upper ,
                            ymin = 0 ,
                            ymax = 3 ,
                            fill = col2 ) ,
              alpha = .9 ) +
    scale_fill_manual(values = alpha(background1$col, 0.9)) +
    theme_classic()+
    geom_density(data = df_density, aes(x=values), size=2) +
    theme_classic() +
    xlab("Log2-fold change") +
    ylab("Density") +
    theme(legend.position = "none")
dev.off()
```

```
## pdf
##   2
```

```
##Here the next functions
#Filter or merge according to list of genera wanting to include.
#GeneraList<-c("Actinomyces", "Roseburia", "Bifidobacterium", "Odoribacter",
#               "Lactobacillus", "Faecalibacterium")
#Based on lowest padj from LRT none. #Below median of LRT none
GeneraList <- df_padj[as.numeric(cut_number(df_padj$`LRT none`,4))==3, 1]
df_log2_plot<-subset(df_log2, Genera %in% GeneraList)
df_padj_plot<-subset(df_padj, Genera %in% GeneraList)


#Change genera to rownames
row.names(df_log2_plot) <- df_log2_plot$Genera
df_log2_plot <- select(df_log2_plot, -one_of("Genera"))

row.names(df_padj_plot) <- df_padj_plot$Genera
df_padj_plot <- select(df_padj_plot, -one_of("Genera"))


#Make symbol according to significance level
df_padj_plotsym<-df_padj_plot #Gets a df with same dimensions
#overrides so can just go from lowest and up
for (i in 1:nrow(df_padj_plotsym)) {
  for (j in 1:ncol(df_padj_plotsym)) {
    ifelse(df_padj_plot[i,j]<0.001, df_padj_plotsym[i,j]<-"***",
           ifelse(df_padj_plot[i,j]<0.01, df_padj_plotsym[i,j]<-"**",
```

```
                  ifelse(df_padj_plot[i,j]<0.05, df_padj_plotsym[i,j]<-"*",
    ifelse(df_padj_plot[i,j]<0.1, df_padj_plotsym[i,j]<-"\U00B7",
           ifelse(df_padj_plot[i,j]<2, df_padj_plotsym[i,j]<-"", "hmmm"))))) #2 values range 0-1
      #knows it is less
  }
}


#max(abs(df_log2_plot))
#range <- max(abs(df_log2_plot)) #Would rather use the previous to make the heatmaps
  #directly comparable

#Create heatmap
range(df_log2_plot)
```

```
## [1] -2.579961  1.547577
```

```
pheatmap(df_log2_plot,
         display_numbers = df_padj_plotsym,
         gaps_col = c(7, 14, 21, 24, 27, 30),
         cluster_rows=FALSE,
         show_rownames=TRUE,
         cluster_cols=FALSE,
         show_colnames=TRUE,
         fontsize_number=20,
         legend=TRUE,
         breaks = seq(-range, range, length.out = 30), #Colors centered at 0
         color=colorRampPalette(c("blue", "white", "red"))(30))


#Draw the heatmap
dev.off()
```

```
## null device
##           1
```

```
pdf(paste("MicroLADA_Figure3DESeqHeatmap3_Func", ".pdf", sep=""), width=10, height=7)
pheatmap(df_log2_plot,
         display_numbers = df_padj_plotsym,
         gaps_col = c(7, 14, 21, 24, 27, 30),
         cluster_rows=FALSE,
         show_rownames=TRUE,
         cluster_cols=FALSE,
         show_colnames=TRUE,
         fontsize_number=10,
         legend=TRUE,
         breaks = seq(-range, range, length.out = 30),
         color=colorRampPalette(c("blue", "white", "red"))(30))

dev.off()
```

```
## null device
##           1
```

```r
#Make density plot
#In the end a solution with new_scale_fill and defining two background datasets
df_density<-data.frame(values=as.vector(as.matrix(df_log2_plot)))
xvalues <- c(-7, 7)
coloring<-c(colorRampPalette(c("red", "white", "red"))(30))
coloring2<-c(colorRampPalette(c("blue", "white", "blue"))(30))
background <- data.frame(lower = seq( xvalues[1], xvalues[2]-sum(abs(xvalues))/30,
                                      sum(abs(xvalues))/30 ),
                         upper = seq( xvalues[1]+sum(abs(xvalues))/30, xvalues[2],
                                      sum(abs(xvalues))/30 ),
                         col = coloring,
                         col2 = coloring2)

background1<-background[1:15,]
background2<-background[16:30,]

ggplot() +
    geom_rect(data = background1 ,
              mapping = aes(xmin = lower ,
                            xmax = upper ,
                            ymin = 0 ,
                            ymax = 4 ,
                            fill = col ) ,
              alpha = .9 ) +
    scale_fill_manual(values = alpha(background1$col2, 0.9))+
    new_scale_fill()+
    geom_rect(data = background2 ,
              mapping = aes(xmin = lower ,
                            xmax = upper ,
                            ymin = 0 ,
                            ymax = 4 ,
                            fill = col2 ) ,
              alpha = .9 ) +
    scale_fill_manual(values = alpha(background1$col, 0.9)) +
    theme_classic()+
    geom_density(data = df_density, aes(x=values), size=2) +
    theme_classic() +
    xlab("Log2-fold change") +
    ylab("Density") +
    theme(legend.position = "none")


pdf(paste("MicroLADA_Figure3DESeqHeatmap3density_Func", ".pdf", sep=""), width=6, height=4)
ggplot() +
    geom_rect(data = background1 ,
              mapping = aes(xmin = lower ,
                            xmax = upper ,
                            ymin = 0 ,
                            ymax = 4 ,
                            fill = col ) ,
              alpha = .9 ) +
    scale_fill_manual(values = alpha(background1$col2, 0.9))+
    new_scale_fill()+
```

```
    geom_rect(data = background2 ,
              mapping = aes(xmin = lower ,
                            xmax = upper ,
                            ymin = 0 ,
                            ymax = 4 ,
                            fill = col2 ) ,
              alpha = .9 ) +
    scale_fill_manual(values = alpha(background1$col, 0.9)) +
    theme_classic()+
    geom_density(data = df_density, aes(x=values), size=2) +
    theme_classic() +
    xlab("Log2-fold change") +
    ylab("Density") +
    theme(legend.position = "none")
dev.off()
```

```
## pdf
##   2
```

```
##Here the next functions
#Filter or merge according to list of genera wanting to include.
#GeneraList<-c("Actinomyces", "Roseburia", "Bifidobacterium", "Odoribacter",
#               "Lactobacillus", "Faecalibacterium")
#Based on lowest padj from LRT none. #Below median of LRT none
GeneraList <- df_padj[as.numeric(cut_number(df_padj$`LRT none`,4))==4, 1]
df_log2_plot<-subset(df_log2, Genera %in% GeneraList)
df_padj_plot<-subset(df_padj, Genera %in% GeneraList)


#Change genera to rownames
row.names(df_log2_plot) <- df_log2_plot$Genera
df_log2_plot <- select(df_log2_plot, -one_of("Genera"))

row.names(df_padj_plot) <- df_padj_plot$Genera
df_padj_plot <- select(df_padj_plot, -one_of("Genera"))


#Make symbol according to significance level
df_padj_plotsym<-df_padj_plot #Gets a df with same dimensions
#overrides so can just go from lowest and up
for (i in 1:nrow(df_padj_plotsym)) {
  for (j in 1:ncol(df_padj_plotsym)) {
    ifelse(df_padj_plot[i,j]<0.001, df_padj_plotsym[i,j]<-"***",
           ifelse(df_padj_plot[i,j]<0.01, df_padj_plotsym[i,j]<-"**",
                  ifelse(df_padj_plot[i,j]<0.05, df_padj_plotsym[i,j]<-"*",
    ifelse(df_padj_plot[i,j]<0.1, df_padj_plotsym[i,j]<-"\U00B7",
           ifelse(df_padj_plot[i,j]<2, df_padj_plotsym[i,j]<-"", "hmmm"))))) #2 values range 0-1
      #knows it is less
  }
}


#max(abs(df_log2_plot))
```

```r
#range <- max(abs(df_log2_plot)) #Would rather use the previous to make the heatmaps
  #directly comparable

#Create heatmap
range(df_log2_plot)
```

```
## [1] -0.9359374  0.9758666
```

```r
pheatmap(df_log2_plot,
         display_numbers = df_padj_plotsym,
         gaps_col = c(7, 14, 21, 24, 27, 30),
         cluster_rows=FALSE,
         show_rownames=TRUE,
         cluster_cols=FALSE,
         show_colnames=TRUE,
         fontsize_number=20,
         legend=TRUE,
         breaks = seq(-range, range, length.out = 30), #Colors centered at 0
         color=colorRampPalette(c("blue", "white", "red"))(30))


#Draw the heatmap
dev.off()
```

```
## null device
##           1
```

```r
pdf(paste("MicroLADA_Figure3DESeqHeatmap4_Func", ".pdf", sep=""), width=10, height=7)
pheatmap(df_log2_plot,
         display_numbers = df_padj_plotsym,
         gaps_col = c(7, 14, 21, 24, 27, 30),
         cluster_rows=FALSE,
         show_rownames=TRUE,
         cluster_cols=FALSE,
         show_colnames=TRUE,
         fontsize_number=10,
         legend=TRUE,
         breaks = seq(-range, range, length.out = 30),
         color=colorRampPalette(c("blue", "white", "red"))(30))

dev.off()
```

```
## null device
##           1
```

```r
#Make density plot
#In the end a solution with new_scale_fill and defining two background datasets
df_density<-data.frame(values=as.vector(as.matrix(df_log2_plot)))
xvalues <- c(-7, 7)
coloring<-c(colorRampPalette(c("red", "white", "red"))(30))
coloring2<-c(colorRampPalette(c("blue", "white", "blue"))(30))
```

```
background <- data.frame(lower = seq( xvalues[1], xvalues[2]-sum(abs(xvalues))/30,
                                      sum(abs(xvalues))/30 ),
                         upper = seq( xvalues[1]+sum(abs(xvalues))/30, xvalues[2],
                                      sum(abs(xvalues))/30 ),
                         col = coloring,
                         col2 = coloring2)

background1<-background[1:15,]
background2<-background[16:30,]

ggplot() +
    geom_rect(data = background1 ,
              mapping = aes(xmin = lower ,
                            xmax = upper ,
                            ymin = 0 ,
                            ymax = 5 ,
                            fill = col ) ,
              alpha = .9 ) +
    scale_fill_manual(values = alpha(background1$col2, 0.9))+
    new_scale_fill()+
    geom_rect(data = background2 ,
              mapping = aes(xmin = lower ,
                            xmax = upper ,
                            ymin = 0 ,
                            ymax = 5 ,
                            fill = col2 ) ,
              alpha = .9 ) +
    scale_fill_manual(values = alpha(background1$col, 0.9)) +
    theme_classic()+
    geom_density(data = df_density, aes(x=values), size=2) +
    theme_classic() +
    xlab("Log2-fold change") +
    ylab("Density") +
    theme(legend.position = "none")


pdf(paste("MicroLADA_Figure3DESeqHeatmap4density_Func", ".pdf", sep=""), width=6, height=4)
ggplot() +
    geom_rect(data = background1 ,
              mapping = aes(xmin = lower ,
                            xmax = upper ,
                            ymin = 0 ,
                            ymax = 5 ,
                            fill = col ) ,
              alpha = .9 ) +
    scale_fill_manual(values = alpha(background1$col2, 0.9))+
    new_scale_fill()+
    geom_rect(data = background2 ,
              mapping = aes(xmin = lower ,
                            xmax = upper ,
                            ymin = 0 ,
                            ymax = 5 ,
                            fill = col2 ) ,
```

```
              alpha = .9 ) +
    scale_fill_manual(values = alpha(background1$col, 0.9)) +
    theme_classic()+
    geom_density(data = df_density, aes(x=values), size=2) +
    theme_classic() +
    xlab("Log2-fold change") +
    ylab("Density") +
    theme(legend.position = "none")
dev.off()
```

```
## pdf
##   2
```

```
#Make table log2fold changes and p values
df_log2 <- data.frame(df_log2[,-1], row.names = df_log2[,1])
df_padj <- data.frame(df_padj[,-1], row.names = df_padj[,1])

if((sum(colnames(df_log2)!=colnames(df_padj))==0)==FALSE) {stop("Columns does not match")}
if((sum(rownames(df_log2)!=rownames(df_padj))==0)==FALSE) {stop("Rows does not match")}

df_paste <- df_log2 #just to get dimensions, row- and columnnames

for (i in 1:nrow(df_log2)) {
  for (j in 1:ncol(df_log2)) {
    df_paste[i,j]<-paste(signif(df_log2[i,j], digits=4), " (",
                         signif(df_padj[i,j], digits=4) , ")")
  }
}
write.table(df_paste, file="DESeqHeatmapl2fpval_SupTab_Func.txt", sep="\t", dec=",", row.names = T)
```

Additional

Session information

```
sessionInfo()
```

```
## R version 4.1.2 (2021-11-01)
## Platform: x86_64-w64-mingw32/x64 (64-bit)
## Running under: Windows 10 x64 (build 19044)
##
## Matrix products: default
##
## locale:
## [1] LC_COLLATE=Danish_Denmark.1252  LC_CTYPE=Danish_Denmark.1252
## [3] LC_MONETARY=Danish_Denmark.1252 LC_NUMERIC=C
## [5] LC_TIME=Danish_Denmark.1252
##
## attached base packages:
##  [1] stats4    grid      tcltk     stats     graphics  grDevices utils
##  [8] datasets  methods   base
```

```
##
## other attached packages:
##  [1] Maaslin2_1.8.0              DESeq2_1.34.0
##  [3] SummarizedExperiment_1.24.0 Biobase_2.54.0
##  [5] MatrixGenerics_1.6.0        matrixStats_0.61.0
##  [7] GenomicRanges_1.46.1        GenomeInfoDb_1.30.0
##  [9] IRanges_2.28.0              S4Vectors_0.32.3
## [11] BiocGenerics_0.40.0         SIAMCAT_1.14.0
## [13] phyloseq_1.38.0             mlr_2.19.0
## [15] ParamHelpers_1.14           ALDEx2_1.26.0
## [17] BiocParallel_1.28.3         mixOmics_6.18.1
## [19] ggnewscale_0.4.5            hablar_0.3.0
## [21] VennDiagram_1.7.1           futile.logger_1.4.3
## [23] cowplot_1.1.1               seqinr_4.2-8
## [25] stringr_1.4.0               RColorBrewer_1.1-2
## [27] pheatmap_1.0.12             BiodiversityR_2.14-1
## [29] curl_4.3.2                  htmlTable_2.4.0
## [31] plotly_4.10.0               nlme_3.1-153
## [33] exactRankTests_0.8-34       mime_0.12
## [35] digest_0.6.28               tidyr_1.1.4
## [37] reshape2_1.4.4              compositions_2.0-2
## [39] zCompositions_1.3.4         truncnorm_1.0-8
## [41] NADA_1.6-1.1                survival_3.2-13
## [43] MASS_7.3-54                 gridExtra_2.3
## [45] vegan_2.5-7                 lattice_0.20-45
## [47] permute_0.9-5               ggplot2_3.3.5
## [49] knitr_1.37                  dplyr_1.0.7
## [51] tibble_3.1.6
##
## loaded via a namespace (and not attached):
##   [1] bit64_4.0.5            DelayedArray_0.20.0    data.table_1.14.2
##   [4] rpart_4.1-15           KEGGREST_1.34.0        RCurl_1.98-1.5
##   [7] generics_0.1.1         lambda.r_1.2.4         RSQLite_2.2.9
##  [10] proxy_0.4-26           bit_4.0.4              bayesm_3.1-4
##  [13] PRROC_1.3.1            relimp_1.0-5           xfun_0.29
##  [16] hms_1.1.1              LiblineaR_2.10-12      evaluate_0.14
##  [19] DEoptimR_1.0-10        fansi_0.5.0            progress_1.2.2
##  [22] readxl_1.3.1           igraph_1.2.10          DBI_1.1.2
##  [25] geneplotter_1.72.0     htmlwidgets_1.5.4      tensorA_0.36.2
##  [28] rARPACK_0.11-0         purrr_0.3.4            ellipsis_0.3.2
##  [31] crosstalk_1.2.0        corrplot_0.92          RSpectra_0.16-0
##  [34] backports_1.4.1        insight_0.14.5         survey_4.1-1
##  [37] annotate_1.72.0        gridBase_0.4-7         vctrs_0.3.8
##  [40] abind_1.4-5            cachem_1.0.6           withr_2.4.3
##  [43] RcmdrMisc_2.7-2        robustbase_0.93-9      checkmate_2.0.0
##  [46] prettyunits_1.1.1      getopt_1.20.3          cluster_2.1.2
##  [49] ape_5.6                lazyeval_0.2.2         crayon_1.4.2
##  [52] ellipse_0.4.2          genefilter_1.76.0      labeling_0.4.2
##  [55] glmnet_4.1-3           pkgconfig_2.0.3        Rcmdr_2.7-2
##  [58] nnet_7.3-16            rlang_0.4.12           lifecycle_1.0.1
##  [61] sandwich_3.0-1         cellranger_1.1.0       Matrix_1.4-0
##  [64] carData_3.0-5          lpsymphony_1.22.0      Rhdf5lib_1.16.0
##  [67] boot_1.3-28            zoo_1.8-9              base64enc_0.1-3
##  [70] png_0.1-7              viridisLite_0.4.0      bitops_1.0-7
```

```
##  [73] rhdf5filters_1.6.0     pROC_1.18.0            Biostrings_2.62.0
##  [76] blob_1.2.2             shape_1.4.6            jpeg_0.1-9
##  [79] scales_1.1.1           memoise_2.0.1          magrittr_2.0.1
##  [82] plyr_1.8.6             zlibbioc_1.40.0        compiler_4.1.2
##  [85] lme4_1.1-27.1          ade4_1.7-18            XVector_0.34.0
##  [88] formatR_1.11           Formula_1.2-4          mgcv_1.8-38
##  [91] tidyselect_1.1.1       stringi_1.7.6          tcltk2_1.2-11
##  [94] forcats_0.5.1          highr_0.9              mitools_2.4
##  [97] yaml_2.2.1             locfit_1.5-9.4         latticeExtra_0.6-29
## [100] ggrepel_0.9.1          fastmatch_1.1-3        tools_4.1.2
## [103] parallel_4.1.2         rstudioapi_0.13        foreach_1.5.1
## [106] foreign_0.8-81         optparse_1.7.1         farver_2.1.0
## [109] RcppZiggurat_0.1.6     nortest_1.0-4          Rcpp_1.0.7
## [112] car_3.0-12             infotheo_1.2.0         httr_1.4.2
## [115] AnnotationDbi_1.56.2   effects_4.2-0          colorspace_2.0-2
## [118] XML_3.99-0.8           splines_4.1.2          multtest_2.50.0
## [121] xtable_1.8-4           jsonlite_1.7.2         nloptr_1.2.2.3
## [124] futile.options_1.0.1   BBmisc_1.11            corpcor_1.6.10
## [127] Rfast_2.0.4            R6_2.5.1               Hmisc_4.6-0
## [130] pillar_1.6.4           htmltools_0.5.2        glue_1.5.0
## [133] fastmap_1.1.0          minqa_1.2.4            class_7.3-19
## [136] beanplot_1.2           codetools_0.2-18       pcaPP_1.9-74
## [139] mvtnorm_1.1-3          utf8_1.2.2             biglm_0.9-2.1
## [142] parallelMap_1.5.1      rmarkdown_2.11         biomformat_1.22.0
## [145] munsell_0.5.0          e1071_1.7-9            rhdf5_2.38.0
## [148] GenomeInfoDbData_1.2.7 iterators_1.0.13       haven_2.4.3
## [151] gtable_0.3.0
```

This document was processed on:

```
Sys.Date()
```

```
## [1] "2022-02-24"
```
